# Machine learning-driven prediction of immune checkpoint inhibitor responses against cholangiocarcinoma: a bile biopsy perspective

**DOI:** 10.3389/fimmu.2025.1614683

**Published:** 2025-06-16

**Authors:** Dengyong Zhang, Xinrui Li, Zhonglin Wang, Jingyuan Fan, Yuhang Yang, Sophia Han, Wanliang Sun, Dongdong Wang, Shuo Zhou, Zhong Liu, Shihao Chen, Yan Yang, Yan Zhu, Zheng Lu

**Affiliations:** ^1^ Department of General Surgery, The First Affiliated Hospital of Bengbu Medical University, Bengbu, Anhui, China; ^2^ Biology Department, Bates College, Lewiston, ME, United States; ^3^ School of Public Policy & Management, Tsinghua University, Beijing, China; ^4^ The Biology Department, Carnegie Vanguard High School, Houston, TX, United States; ^5^ The Biology Department, Awty International School, Houston, TX, United States; ^6^ Institute of Epigenetics and Epigenomics and University of Animal Science and Technology, Yangzhou University, Yangzhou, China; ^7^ Department of Oncology, The First Affiliated Hospital of Bengbu Medical University, Bengbu, Anhui, China

**Keywords:** cholangiocarcinoma, immune checkpoint inhibitors, bile metabolomics, machine learning, programmed cell death protein 1 (PD-1) therapy, immune hot-cold index, metabolites, biomarker prediction

## Abstract

**Background:**

The treatment of cholangiocarcinoma (CCA) continues to face numerous clinical challenges, including the prediction of sensitivity to immunotherapy and the development of preoperative diagnostic models.

**Methods:**

In this study, we aimed to address these challenges by collecting bile samples from CCA patients for metabolomic and microbiomic analyses. We also performed immunofluorescence (IF) staining on tissue formalin-fixed, paraffin-embedded (FFPE) blocks to assess the expression of relevant biomarkers. Additionally, we followed up with patients to analyze prognostic indicators based on their survival times. Using advanced machine learning techniques, specifically LASSO regression, we constructed a predictive model to determine the effectiveness of programmed cell death protein 1 (PD-1) inhibitors in treating CCA. The model integrates bile metabolomic data with an Immune Hot-Cold Index (IHC Index) derived from IF results, providing a comprehensive metric of the patient’s immune environment.

**Results:**

Our findings revealed significant differences in metabolomic profiles between CCA patients and those with non-malignant liver diseases, as well as between patients with different genetic mutations. The IHC Index successfully differentiated between immune “hot” and “cold” states, correlating strongly with patient responses to immunotherapy. Furthermore, in one CCA patient, the model’s predictions were validated, demonstrating high accuracy and clinical relevance.

**Conclusion:**

Our predictive model offers a robust tool for assessing the sensitivity of CCA patients to PD-1 inhibitors, potentially guiding personalized treatment strategies. Additionally, the integration of bile metabolomics with IF data provides a promising approach for developing preoperative diagnostic models, enhancing early detection and treatment planning for CCA.

## 1 Introduction

Cholangiocarcinoma (CCA) is a heterogeneous malignant tumor originating from bile duct, with an incidence rate second only to that of hepatocellular carcinoma (HCC) among primary liver tumors. CCA accounts for approximately 1% of all cancers in the population and about 10-15% of all primary liver cancers (1–3). Most patients are asymptomatic in the early stage and lack of effective diagnostic biomarkers, making early clinical diagnosis challenging. Consequently, only about one-third of patients are candidates for surgical resection at the time of diagnosis​ (4). Current treatment options for inoperable patients are limited, with gemcitabine combined with platinum chemotherapy being the most commonly used regimen. However, the clinical efficacy of this treatment is extremely poor (5, 6).

As a novel class of immunotherapeutic agents, immune check point inhibitors targeting programmed cell death protein 1 (PD-1) have shown clinical efficacy in treating various tumors (7, 8), but their role in CCA remains in the exploratory stages, with ongoing clinical trials. In the Phase I of clinical treatment of advanced CCA, the median overall survival time was reported to be 15 ± 4 months, with 11 of 30 patients achieving objective remission (9). The ongoing TOPAZ-1 project used durvalumab combined with platinum and gemcitabine to treat advanced CCA in Phase III clinical trial, demonstrating partial clinical efficacy (10, 11). However, not all patients benefit from this treatment regimen. At present, there is no reliable index to predict whether patients are effective in PD-1 treatment, which is crucial for guiding patients in clinical treatment. Therefore, developing predictive tools to for this purpose is essential. Clinically, patients with CCA are often accompanied by obstructive jaundice. To mitigate liver function damage, percutaneous transhepatic cholangial drainage (PTCD) puncture and drainage of bile are needed to improve liver function, which also serves as a reserve for subsequent drug treatments. This procedure facilitates the collection of bile samples in a clinical setting.

Existing research predominantly focused on the relationship between specific metabolites in bile and CCA. Hashim AbdAlla et al. (12) focused on phosphatidylcholine (PtC) and bile acids, comparing their levels in bile from CCA patients and those with benign biliary diseases. Similarly, Sharif et al. (13) analyzed glycine-conjugated bile acids, primary bile acids, and PtC in bile from CCA patients. Albiin et al. (14) studied the levels of PtC, bile acids, lipid, and cholesterol in bile, highlighting significant differences between CCA patients and those with benign biliary conditions. Volinsky et al. (15) explored oxidized phosphatidylcholines in cellular signaling and their role in various diseases, including CCA. Won-Suk Song et al. (16) identified glycocholic acid (GCA) and taurochenodeoxycholic acid (TCDCA) as specific metabolic biomarkers for CCA. Gomez et al. (17) developed and validated an LC-MS/MS method for quantifying various bile acids, highlighting the significance of free and conjugated bile acids in disease mechanisms.

Although these studies have provided significant insights, they are limited by their focus on specific metabolites. Concentrating solely on individual metabolites can overlook broader metabolic interactions and comprehensive biochemical alterations associated with CCA. This narrow scope may miss potential biomarkers and therapeutic targets that could be identified through a more holistic analysis. Metabolomics, the comprehensive study of metabolites in a biological system, reveals that cancer’s metabolic reprogramming affects numerous pathways and involves a wide array of metabolites. Therefore, analyzing bile as a whole can offer a more complete picture of the disease and its interactions, potentially leading to more effective predictive models and therapeutic strategies (18).

To address these limitations, our research aims to take a comprehensive approach by analyzing bile as a whole and exploring its association with CCA. By examining the entire spectrum of bile metabolites, we hope to develop a more robust predictive model for PD-1 inhibitor response in CCA patients. In this study, we used bile samples from patients with CCA for metabolomics and microbiology detection, sequenced exons of corresponding cancer tissue samples of patients, and detected the expression of immune-related indicators in tissues by immunofluorescence (IF). We then built a prediction model for the therapeutic effect of PD-1 on CCA through machine learning and verified the model in some patients treated with PD-1.

## 2 Methods and materials

### 2.1 Human subjects

Bile was collected from patients between January 2019 and July 2023 at The First Affiliated Hospital of Bengbu Medical University. A total of 66 CCA patients who had not received neoadjuvant therapy were selected for this study. Among them, 62 patients underwent surgical procedures for tumor collection. Additionally, bile from 38 patients with non-malignant liver diseases, such as gallbladder stones or liver hemangioma, was collected and used as a negative control. The collection and preservation of bile samples followed the same protocols described in our previous publication (19). Written informed consent was obtained from each patient, and the study was approved by the Ethics Committee of Bengbu Medical University (No. 2021230 and No. 2019035).

### 2.2 Metabolomics

Completed by Huada Gene Company (Shenzhen, China) in the same way as the previous published articles (19).

### 2.3 Microbiology (16S)

Completed by Huada Gene Company (Shenzhen, China). The microbial community DNA was extracted using MagPure Stool DNA KF kit B(Magen, China) following the manufacturer’s instructions. DNA was quantified with a Qubit Fluorometer by using Qubit dsDNA BR Assay kit (Invitrogen, USA) and the quality was checked by running aliquot on 1% agarose gel. Variable regions V4 of bacterial 16S rRNA gene was amplified with degenerate PCR primers, 515F (5-GTGCCAGCMGCCGCGGTAA-3’) and 806R (5’-GGACTACHVGGGTWTCTAAT-3’). Both forward and reverse primers were tagged with Illumina adapter, pad and linker sequences. Then do PCR reaction. The Libraries were qualified by the Agilent Technologies 2100 bioanalyzer. The validated libraries were used for sequencing on MGISEQ-2000 platform (BGI, Shenzhen, China) following the standard pipelines of Illumina, and generating 2 × 250 bp paired-end reads.

### 2.4 Whole-exome sequencing

Completed by LC Bio Tech (Hangzhou, China). We sequenced exons of 61 formalin fixed paraffin embedded (FFPE) blocks in these patients with CCA. The total DNA was extracted using QIAGEN DNeasy Blood & Tissue Kit (69506, QIAGEN) or QIAamp DNA FFPE Tissue (56404, QIAGEN). Then the DNA which was fragmented by using Covaris M220 Focused-ultrasonicator were subjected to sequencing library construction. Exome capture was performed using the Human Exome 2.0 Plus (Twist Bioscience) following the vendor’s recommended protocol. The final libraries were sequenced for paired-end 150 bp using the Illumina NovaSeq 6000 Sequencing System (Illumina) at LC-Bio Technology Co., Ltd (Hangzhou, China).

### 2.5 Immunofluorescence

The routine steps for IF are detailed in the literature. Briefly, the process involves dewaxing FFPE blocks to water, antigen repair, blocking endogenous peroxidase with 3% hydrogen peroxide, serum blocking, and sequentially adding primary and secondary antibodies. In this study, we detected 12 protein indexes in 62 cases of CCA using a four-label fluorescence staining method. The first antibody was stained and photographed, followed by antigen repair and blocking steps, repeated until the fourth antibody was obtained. We tested the following 12 indexes on the same FFPE block. The details of the primary and secondary antibodies used for multiplex immunofluorescence are summarized in (**Table 1**).

**Table 1:** Primary and secondary antibodies used for multiplex immunofluorescence staining of FFPE blocks.

Primary antibody	Cat no.	Company	Species	Ratio	Secondary antibody
OX40	AB264466	Abcam	Rab	1:4000	CY3(red)
ICOS	A00291-3	BOSTER Biological Technology	Rab	1:400	488(green)
CD20	60271-1-AP	Proteintech Group	Mou	1:400	CY5(pink)
CD86	AB239075	Abcam	Rab	1:200	594 (yellow)
LAG3	AB209236	Abcam	Rab	1:1000	CY3(red)
TIM3	AB241332	Abcam	Rab	1:1000	488(green)
PD-1	GB12338	Servicebio	Mou	1:1000	CY5(pink)
VISTA	24849-1-AP	Proteintech Group	Rab	1:200	594(yellow)
CD3	GB13014	Servicebio	Rab	1:200	CY3(red)
CD206	AB64693	Abcam	Rab	1:500	488(green)
CD8	GB12068	Servicebio	Mou	1:500	CY5(pink)
CD4	AB133616	Abcam	Rab	1:500	594(yellow)

This table lists the 12 immune-related protein markers analyzed in 62 cholangiocarcinoma (CCA) tissue samples. For each marker, the primary antibody name, catalog number, source company, host species, dilution ratio, and the corresponding fluorophore-conjugated secondary antibody are provided.

### 2.6 Calculation and assessment of the Immune Hot-Cold Index

We utilized IHC results to indicate the overall level of a patient’s immune environment, characterized by a specific metric. This involves treating OX40, ICOS, CD20, CD86, LAG3, TIM3, PD-1, VISTA, CD3, CD206, CD8, and CD4 as twelve dimensions of vectors, thereby creating a patient-specific IHC vector space. Within this space, we calculated the Euclidean distance between the 12-dimensional vectors and a zero vector, which serves as a measure of immune response strength—denoted as the IHC index. The Euclidean distance formula used is:


$$IHC\;index=d{\left(p,q\right)}^{2}=\sqrt{\left(\sum_{i=1}^{N}{\left(p_{i}-q_{i}\right)}^{2}\right)}$$

where *p* and *q* represent the two vectors (data points).

We adopted the same computational approach as in previous studies, compared the clustering results, and assessed the scoring method based on recall, F1 score, precision, and accuracy. Additionally, t-SNE was employed for visualization.

### 2.7 Model construction

After testing various machine learning algorithms, we selected the Least Absolute Shrinkage and Selection Operator (LASSO) regression for our dataset. Our focus was on predicting a continuous variable, the immune hot-cold index, using biliary metabolomic sequencing data. LASSO regression’s advantage lies in its ability to assume coefficient sparsity, which reduces overfitting by setting the coefficients of less significant variables to zero. This method enhances the model’s interpretability, allowing us to identify promising targets among numerous metabolic products.

To prevent overfitting, we initially used Spearman correlation to reduce the number of metabolic products used as inputs to the model. Following this, we employed a five-fold cross-validation to establish the predictive model using LASSO regression. This approach ensured robust model performance by testing it on multiple subsets of the data. Finally, we tested statistical hypotheses to determine the optimal parameters for the model, ensuring its accuracy and reliability.

### 2.8 Statistical analysis

Statistical test was finished by t test in excel (version 16.58) and p value < 0.05 was determined as significance.

## 3 Results

### 3.1 Patient characteristics

A total of 66 CCA patients (intrahepatic CCA n= 17; extrahepatic CCA n= 49) that did not receive neoadjuvant therapy were selected for this study. 62 CCA samples (intrahepatic CCA n= 15; extrahepatic CCA n= 47) underwent surgery to collect tumors. Among 66 CCA patients, 39% were female and with a median age of 66 years (range, 40–83; **Supplementary Table 1**). Forty percent of patients were alive at the latest follow-up. Ninety seven percent of patients were diagnosed at advanced stages (T2 or higher). Bile from 38 patient with non-malignant liver diseases, such as gallbladder stone or liver hemangioma, were collected as negative control: 45% were female and with a median age of 59 years (range, 25–90; **Supplementary Table 1**).

### 3.2 The significant difference in metabolites instead of microbes between CCA and control

**Figure 1:**
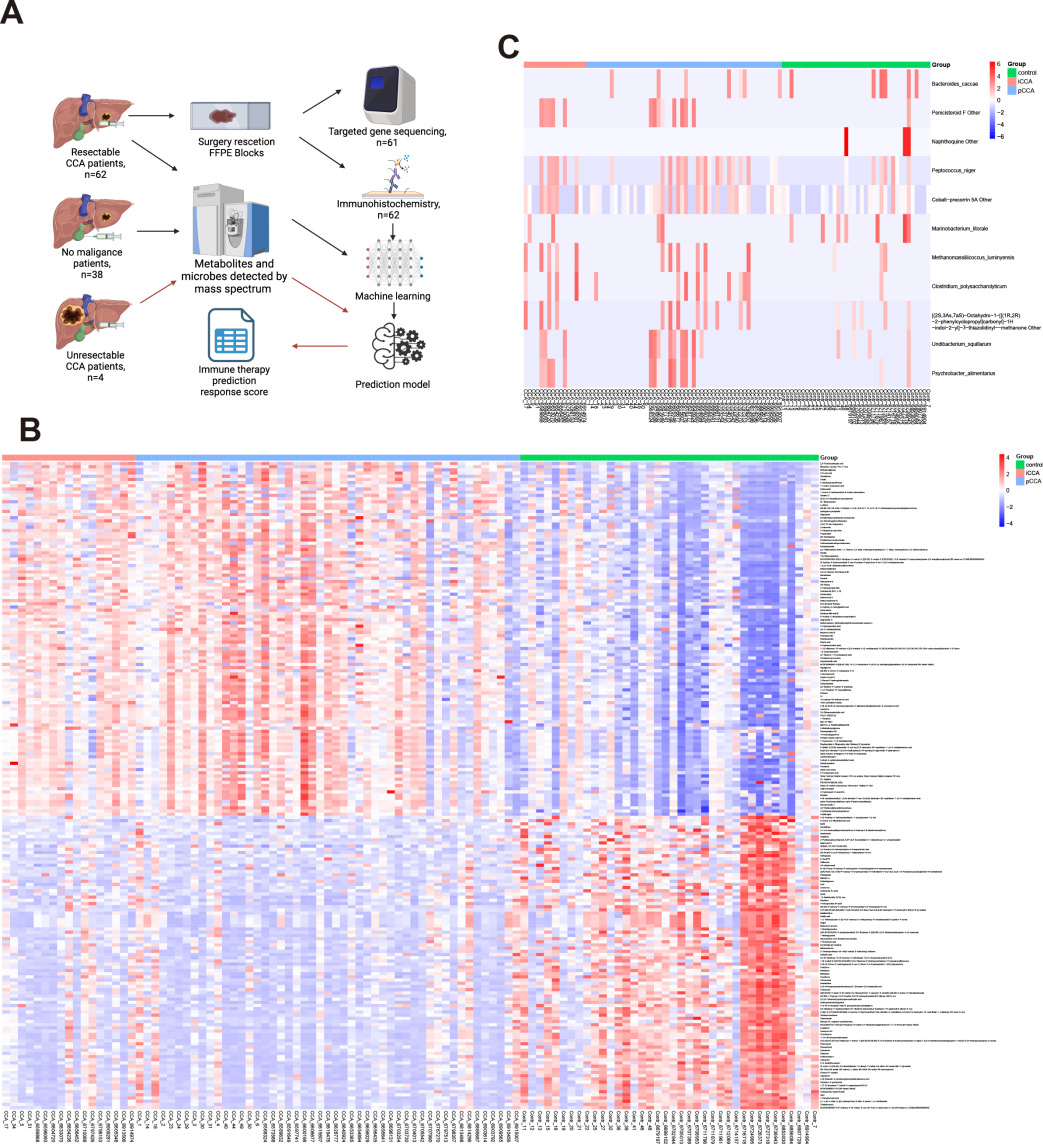
Overview of study design and comparison of metabolomic and microbial profiles between CCA and control groups. **(A)** Schematic of patient selection, sample collection, and processing workflow. **(B)** Heatmap showing the top 100 up- and down-regulated metabolites in bile samples between CCA and control groups. **(C)** Heatmap showing microbial taxa significantly altered between CCA and control groups based on 16S rRNA sequencing.

We identified 8783 substances in the metabolism-related database (1132 were down-regulated and 1459 were up-regulated by the criteria: p-value<0.05). We also identified 1316 substances in the microbe-related database (12 microbes shows significant changes by the criteria: p-value<0.05). The heatmap of top 100 up/down-regulated metabolisms and microbes shows in **Figures 1A–C**. Due to the limited changes in microbes, we will focus on metabolites in following research.

### 3.3 The significant difference in metabolites between patients harboring mutated and wild-type genes

**Figure 2:**
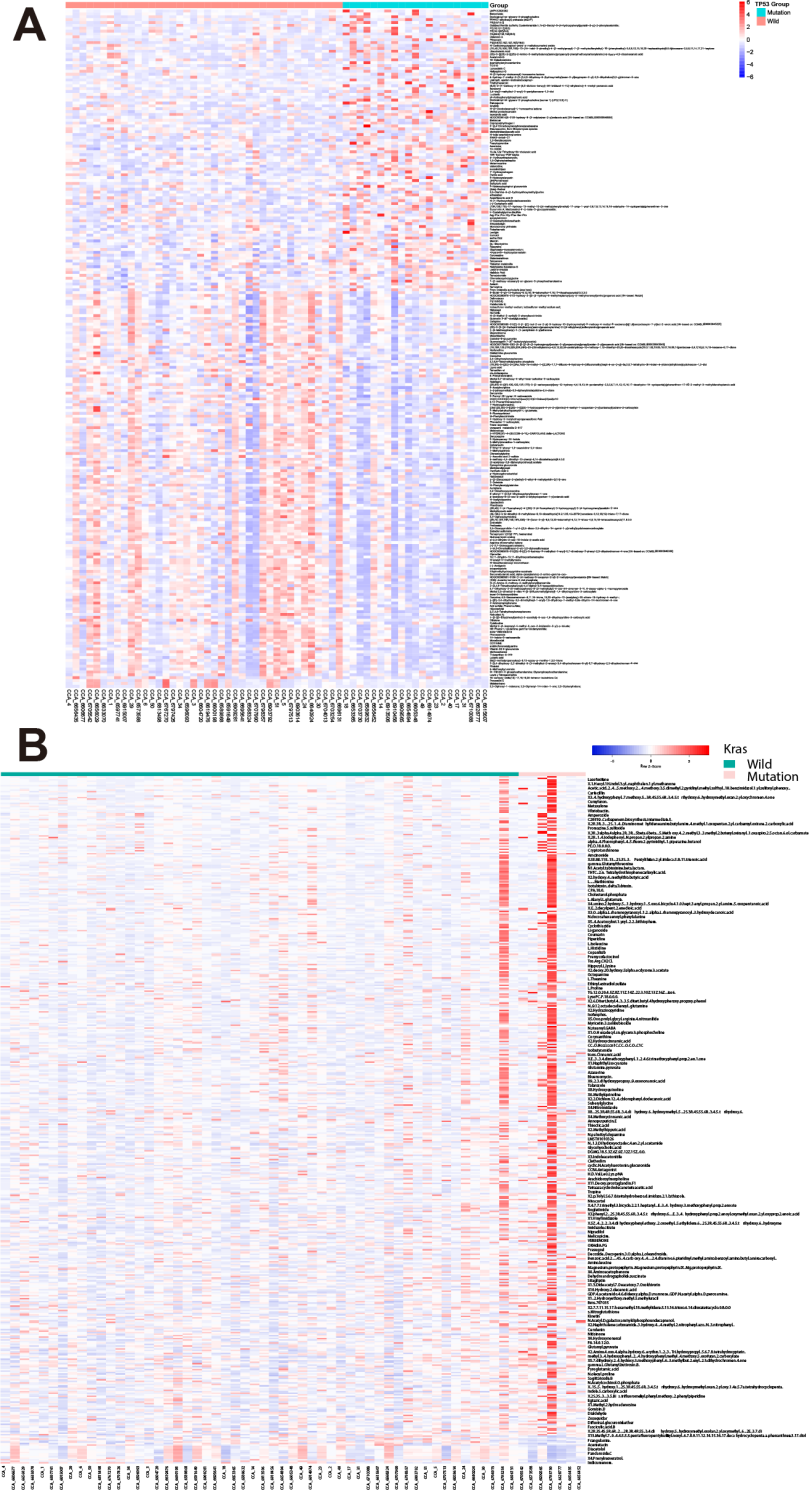
Differential bile metabolite profiles in CCA patients with TP53 and K-Ras mutations. **(A)** Volcano plot showing significantly altered metabolites in patients harboring TP53 mutations versus wild-type. **(B)** Volcano plot showing significantly altered metabolites in patients harboring K-Ras mutations versus wild-type.

We examined metabolite profiles in patients harboring specific mutations. Among 62 CCA patients, 21 of them harbor TP53 mutations (**Supplementary Table 1**). Compared with wild-type patients, 236 metabolites show significant changes by the criteria: p-value<0.05 (98 up-regulated and 138 down-regulated, **Figure 2A**). Besides, in seven patients harboring K-Ras mutations, 446 metabolites up-regulated while only 18 metabolites down-regulated (**Figure 2B**). These results suggest that mutations in oncogenes and tumor suppressor genes show different impact on the profile of metabolites in bile.

### 3.4 The expression of immune cell and immune checkpoint markers

**Figure 3:**
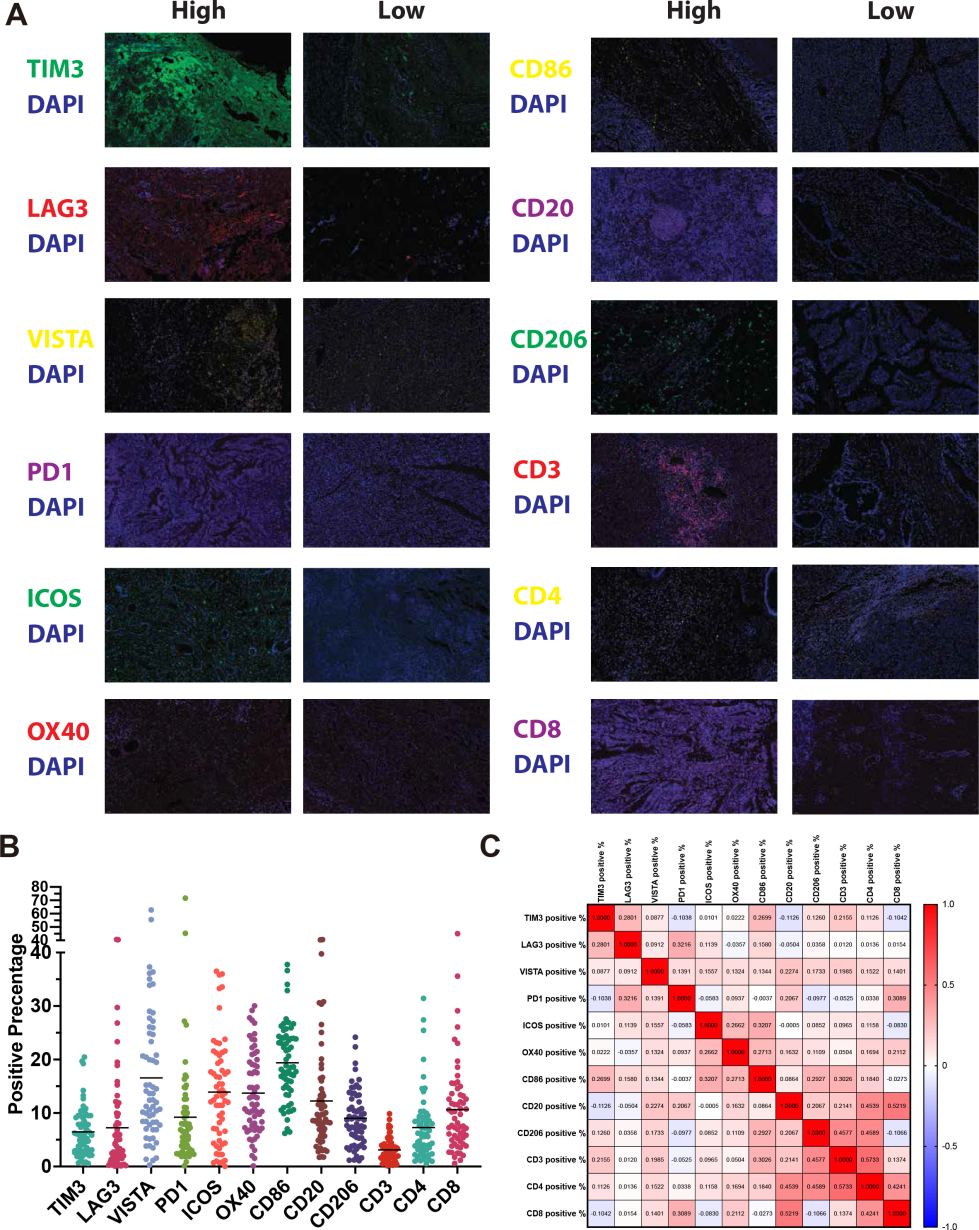
Immune profiling of CCA tissues using immunofluorescence. **(A)** Representative photomicrographs of CCA tissue samples showing high and low expression of immune markers. **(B)** Dot plot summarizing the percentage of positive staining for each of the 12 immune markers across all patients. **(C)** Correlation matrix showing Spearman correlations between expression levels of immune cell and immune checkpoint markers.

All 62 samples underwent singleplex IHC for 12 immune cell and immune checkpoint markers. Representative examples of staining are shown in **Figure 3A** and the positive rates of each marker are shown in **Figure 3B**. To further explore the relationships between these markers, we examined their correlations (**Figure 3C**). As expected, high correlations in CD4:CD8 (ρ = 0.42) and CD20:CD8 (ρ = 0.52) are observed. Notably, the correlation between CD3 and CD8 is rather low (ρ = 0.14). While few other strong correlations were observed, including CD206:CD3 (ρ = 0.46), CD206:CD4 (ρ = 0.46), CD20:CD4 (ρ = 0.45), and CD3:CD4 (ρ = 0.57).

### 3.5 Validation and universality of the IHC Index

In the following step, we calculated the IHC Index in each sample based on the expression of immune cell and immune checkpoint markers.

**Figure 4:**
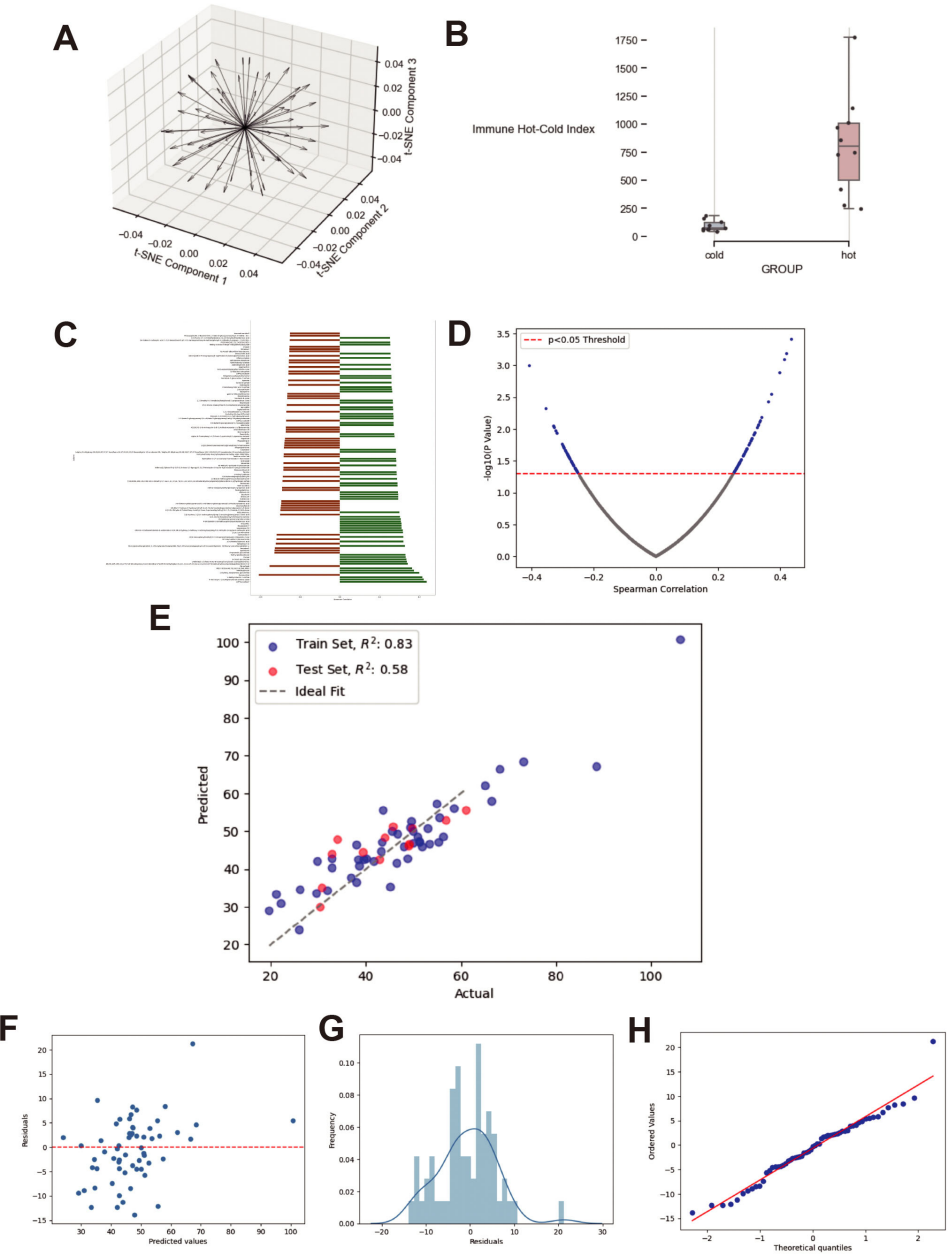
Construction and validation of the Immune Hot-Cold (IHC) index and predictive model. **(A)** Three-dimensional projection of IHC vectors from patient samples using t-SNE dimensionality reduction. **(B)** Validation of the IHC index using an external iCCA cohort (PMID: 34510503), demonstrating separation of immune “hot” and “cold” groups. **(C)** Spearman correlation between bile metabolites and the IHC index. **(D)** Scatter plot of selected metabolites significantly associated with IHC index (p < 0.05). **(E)** Predicted vs actual values of the IHC index using LASSO regression in training and test sets. **(F)** Residual plot showing homoscedasticity. **(G)** Histogram of residuals indicating normality assumption. **(H)** Q-Q plot confirming the normal distribution of residuals.

First, we illustrated the three-dimensional projection of all patients’ immune vectors in the IHC vector space after dimensionality reduction using t-SNE (**Figure 4A**), which provides a continuous scalar value representing an individual’s overall level of immune hotness or coldness. To validate the reliability of this method, we subsequently applied this methodology to retrospectively assess a previous study of 96 patients with intrahepatic cholangiocarcinoma (iCCA) (https://pubmed.ncbi.nlm.nih.gov/34510503/), achieving convincing results (**Figure 4B**). This retrospective validation suggests that our approach may be applicable to multi-target IHC data; however, additional datasets and prospective studies are needed to confirm its broader applicability. The study identified two groups—immune “hot” and immune “cold”—and provided IHC data targeting similar markers as our current work. Additionally, it presented a code set of 773 immune genes associated with immune exhaustion, affirming the reliability of IHC-based groupings. **Figure 4B** shows the calculation of the IHC index using our method on this dataset, where using a single threshold accurately distinguished between the original hot and cold groups with an accuracy of 1, proving our defined IHC index’s ability to continuously describe and universally address similar issues, solving previous challenges associated with the lack of a continuous scale.

### 3.6 Establishment of the IHC index predictive model

Initially, based on Spearman correlation (setting significance at p<0.05), we identified 163 metabolic products strongly correlated with the IHC index to minimize overfitting in model predictions. **Figures 4C, D** display the correlation between these selected metabolites and their predictive values.

Using the LASSO regression method, we developed a predictive model; **Figure 4E** illustrates its performance on the test and training sets, showcasing R² values of 0.83 and 0.58, respectively. The grey dashed line represents a perfect prediction, and while the model’s performance slightly diminishes at higher score ranges, it does not deviate significantly from the ideal fit line, demonstrating that our model captures the data characteristics well and exhibits good predictive performance.

**Figures 4F–H** respectively display the model’s residuals values, residuals distribution, and Q-Q plot. The residuals values are scattered equally on both sides of a horizontal red line, follow a normal distribution, and ordered residuals align closely with the red line, thus fulfilling the model assumptions of normality, linearity, equality of variance, and independence.

**Figure 5:**
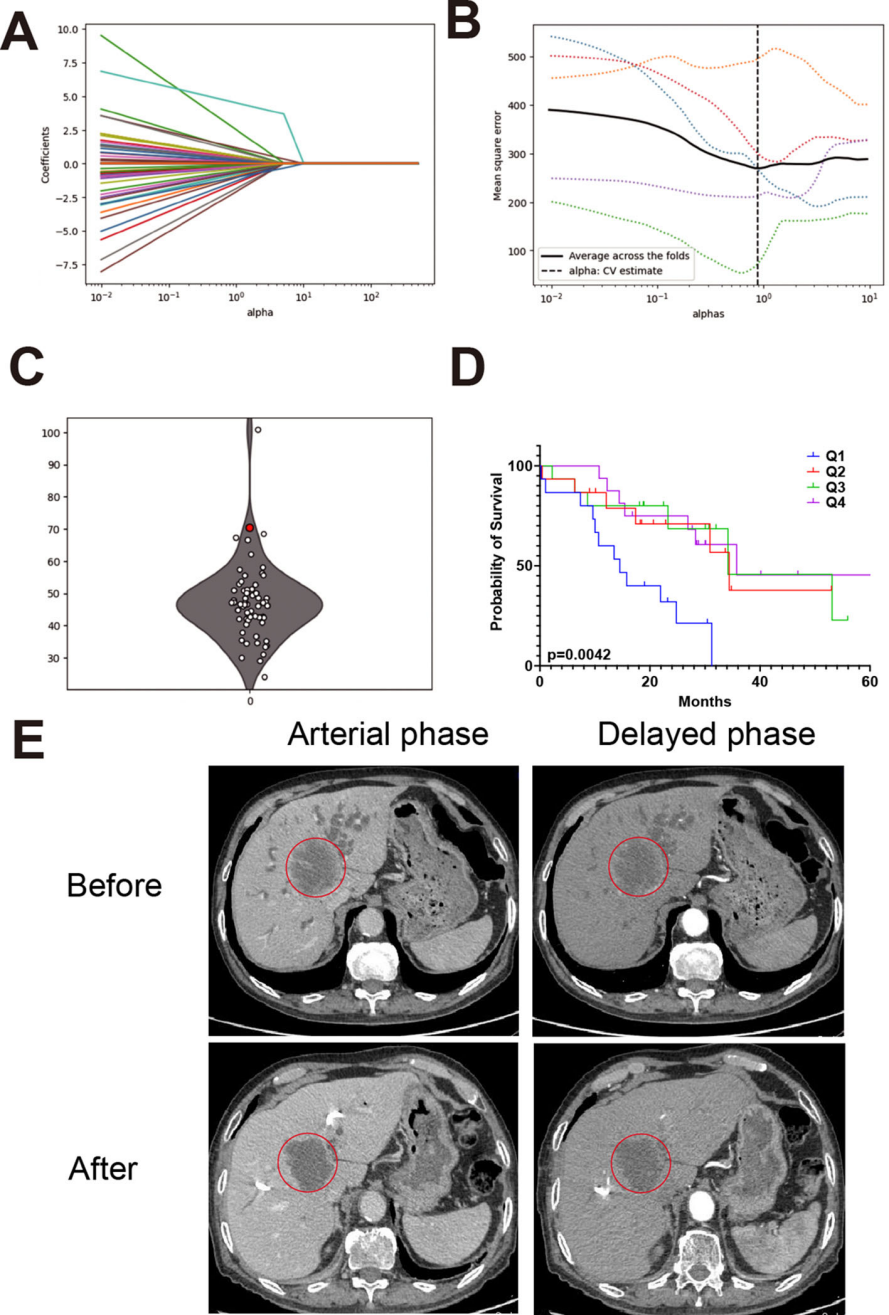
Model performance and case validation in clinical settings. **(A)** Cross-validation curve for LASSO regression showing the relationship between lambda values and model error. **(B)** Optimal alpha value selected for the final model based on lowest cross-validation error. **(C)** Predicted IHC index values for newly recruited patients using the trained model. **(D)** Boxplot categorizing patients into IHC index quartiles with a red dot highlighting an unresectable patient enrolled in immunotherapy. **(E)** CT images from a representative patient before and after PD-1 inhibitor treatment, showing a reduction in tumor size and successful surgical resection.

**Figures 5A, B** show the LASSO model’s selection of alphas and the optimal alpha used in this model. **Figure 5C** presents predictions of the IHC index for newly recruited patients. **Figure 5D** displays these predictions as a boxplot, which falls into four quartiles, highlighting the predictive model’s effectiveness and consistency. Based on these four quartiles, we calculated the survival curve of CCA patients. As shown in **Figure 5D**, Q1 group with the lowest scores shows significant lower survival rate compared with other three groups. **Figure 5E** presents CT images from a representative patient before and after PD-1 inhibitor treatment, demonstrating a notable reduction in tumor size that enabled successful surgical resection.

### 3.7 The clinical validation of this model

Among four unresectable patients, the predicted IHC index values were 81.6, 23.1, 28.5, and 75.8, respectively. The patient with the highest IHC index (81.6) underwent PD-1 inhibitor treatment and subsequently demonstrated a favorable clinical response.

At initial presentation (March 22, 2022), the patient had a CA19–9 level exceeding 1200 IU/mL and was diagnosed with unresectable intrahepatic cholangiocarcinoma. After four cycles of PD-1 inhibitor therapy, the CA19–9 level dropped to 118.07 IU/mL (June 18, 2022). A preoperative measurement on July 25, 2022, showed a sustained decline at 299.68 IU/mL. CT imaging revealed a 35% reduction in tumor size, leading to surgical eligibility. Surgery was performed on July 29, 2022, with frozen section confirming negative margins. Final pathology (August 3, 2022) reported a resected 5.5×4.0×3.0 cm hilar cholangiocarcinoma with necrosis and no lymph node involvement (0/5).

This case illustrates the potential clinical utility of the IHC index in identifying immunologically active tumors that are more likely to respond to PD-1 inhibitors and become surgically resectable.

## 4 Discussion

Intrahepatic cholangiocarcinoma is the second most common liver tumor, characterized by high malignancy, resistance to chemotherapy, and poor prognosis. Anti-PD-1 immunotherapy (Nivolumab, durvalumab, etc.) has shown certain effects in treating intrahepatic cholangiocarcinoma (20). The Phase II of the KEYNOTE-158 trial evaluated the efficacy of PD-1 inhibitory monoclonal antibody pembrolizumab in a cohort of 104 patients with advanced biliary cancer who received standard chemotherapy. The results showed an objective remission rate of 5.8% (21). Consequently, PD-1 inhibitors have been increasingly used in clinical practice for treating cholangiocarcinoma. However, there is a current challenge in evaluating the sensitivity of patients to PD-1 inhibitors and selecting the appropriate PD-1 drugs. There are few related studies at present.

We present, for the first time, a numerical standard for assessing the overall immune environment’s heat level based on multi-target IF. Traditional metrics for measuring immune heat have significant limitations, often focusing on PD-L1 or just 1–3 IF targets. They typically rely on the percentage of stained cells for stratification, using a 0–2 criterion to describe the overall level of immune heat, such as 1,2. These immune scores do not provide a continuous quantitative evaluation. Our newly created index offers a promising framework to this issue, based on a multi-dimensional vector length constructed from the percentage of cells stained for multiple IF targets, straightforwardly reflecting the definition of immune heat intensity. It is crucial to note that while some target expression levels are considered to have positive or negative associations with immune responses, there is evidence that they are also involved in regulatory processes and immune responses in other, deeper ways. Our focus is on the overall immune response strength, hence, considering the positive or negative impact of individual targets on immune response strength is limited. Our metric preserves the intensity information of individual targets to the greatest extent.

Our study also examined the genetic aspects of CCA, identifying significant differences in metabolomic profiles between patients with specific mutations, such as TP53 and K-Ras. Research indicates that these mutations are commonly found in iCCA and significantly influence the disease’s development and progression. For instance, mutations in TP53 and K-Ras can lead to distinct metabolic alterations, which are known to impact the bile metabolomic profiles, adding complexity to the understanding of CCA. The presence of these mutations is associated with changes in metabolic pathways that are critical for tumor growth and response to therapy (22). This highlights the necessity for personalized therapeutic approaches, considering the unique genetic and metabolic landscape of each patient.

To translate the IHC index into clinically actionable insights, we aimed to identify a low-cost, clinically oriented bridge through biliary metabolites that could provide convenient, rapid, and accurate predictions for cholangiocarcinoma patients’ responses to immune checkpoint inhibitors.

During model development, we initially tested fully connected neural networks, ResNet, and XGBoost regressors. However, these models exhibited notable overfitting and poor generalization in our relatively small-sample, high-dimensional dataset, even after applying pruning and other regularization strategies. LASSO regression was therefore selected for its superior stability and interpretability in this context. Before using LASSO regression, we also compared its performance with these alternative methods, but LASSO consistently yielded more reliable results, particularly in terms of avoiding overfitting and maintaining model clarity. Therefore, LASSO was ultimately chosen to ensure the majority of parameters are zero-valued.

Furthermore, our research illustrates the broader implications of integrating multi-omic data in clinical diagnostics. The combination of metabolomics with IF data may improve the predictive performance of our model and provide additional insights into the tumor microenvironment, though further validation is required. This integrative approach has the potential to be extended to other cancer types and may inform the development of future diagnostic and prognostic tools.

We also attempted to construct a preoperative diagnostic model of cholangiocarcinoma based on bile metabolomics, supplemented by microbiome analysis. However, the microbiome results revealed only 12 taxa with statistically significant differences between CCA and control groups, and no strong overall microbial signal was observed. Given the known antimicrobial properties of bile and its typically low microbial biomass, the diversity and functional relevance of bile microbiota may be inherently limited compared to other compartments like the gut. These biological constraints, combined with the limited statistical differences detected, led us to deprioritize the microbiomic data and focus our model development on metabolomics. Although preliminary attempts at diagnostic modeling using bile metabolomics alone were not yet satisfactory, we plan to expand our sample size and consider additional molecular layers, such as virology or proteomics, in future work to enhance diagnostic potential.

Despite the promising results, our study has limitations that need to be addressed in future research. The relatively small sample size and the single-center nature of the study may limit the generalizability of our findings. To mitigate the sample size limitation, we used LASSO regression, which performs well in small-sample, high-dimensional contexts by penalizing less informative features. Spearman correlation-based preselection and five-fold cross-validation further helped control for overfitting. In addition, we retrospectively validated the IHC Index using a previously published iCCA dataset (PMID: 34510503), achieving accurate classification of immune ‘hot’ and ‘cold’ profiles. Although complete external validation of the bile metabolomics model is limited by data availability, these findings support the model’s robustness and potential generalizability. Future studies should aim to include larger, multi-center cohorts to validate and refine our predictive model further. Additionally, while our model focuses on bile metabolomics and immune biomarkers, incorporating other omics data, such as proteomics and transcriptomics, could provide a more comprehensive understanding of CCA and its response to immunotherapy.

Moreover, the dynamic nature of the immune response and its interaction with metabolic pathways suggest that longitudinal studies are necessary to capture these changes over time. Such studies could provide insights into the temporal aspects of treatment response and resistance, further enhancing the clinical utility of our predictive model (23).

Our findings offer an early observation that may contribute to understanding the relationship between metabolomic and immune profiles. However, the mechanistic links between individual bile metabolites and specific immune signaling pathways remain to be fully elucidated. While prior studies have identified compounds such as glycocholic acid (GCA) and taurochenodeoxycholic acid (TCDCA) as biomarkers for CCA, their direct roles in modulating T-cell responses or immune checkpoints are not well established. Future studies involving pathway-based metabolomic analysis and experimental validation will be needed to explore how specific bile metabolites contribute to immune regulation in the CCA microenvironment. **Figure 4B** demonstrates that our defined IHC index reliably captures the variables measured in comparable studies. This accuracy is further validated by the immune exhaustion gene code set provided in the referenced study. We anticipate that future research will continue to utilize and refine this standard, thereby enhancing its application and validity in diverse contexts.

## 5 Conclusions

In summary, our study presents a novel, integrative approach to predicting PD-1 inhibitor responses in CCA patients. By combining bile metabolomics with immune biomarkers, we have developed a preliminary predictive model that shows potential for informing personalized treatment strategies. Our findings underscore the importance of a holistic approach in cancer research, paving the way for more effective and tailored therapeutic interventions. Future research should continue to build on these findings, expanding the scope of biomarker discovery and refining predictive models to enhance the clinical management of CCA.

## Data Availability

The original contributions presented in the study are included in the article/**Supplementary Material**. Further inquiries can be directed to the corresponding author.
