# Supplementary material for: Machine learning-driven prediction of immune checkpoint inhibitor responses against cholangiocarcinoma: a bile biopsy perspective
Source: Front Immunol. 2025 Jun 16;16:1614683. doi: 10.3389/fimmu.2025.1614683 (PMC12206865; doi:10.3389/fimmu.2025.1614683)
Supplement: Supplementary file 1 [file DataSheet1.pdf]

Name	Metabolite	Ion Mode	RT	Molecular
cancer Type				
**ARID1A**				
**ATM**				
**BAP1**				
**BRAF**				
**BRCA1**				
**BRCA2**				
**CDKN2A**				
**EGFR**				
**ELF3**				
**ERBB2**				
**FBXW7**				
**FGFR2**				
**IDH1**				
**NF1**				
**NF2**				
**SMAD4**				
**STK11**				
**TP53**				
**ARID2**				
**KRAS**				
blood CA199（ng/ml)				
blood CEA（ng/ml)				
blood albumin（g/L)				
blood TBIL（μmol/L)				
blood ALT（U/L)				
blood AST（U/L)				
blood cholesterol（mmol/L)				
blood triglyceride（mmol/L)				
blood total bile acid（μmol/L)				
Sex				
age				
group				
TNM stage				
survival(0 for alive, 1 for death)				
survive time（month)				
**DAPI nuclear number**				
**TIM3 positive cell number**				
**LAG3 positive cell number**				
**VISTA positive cell number**				
**PD1 positive cell number**				
**TIM3 positive %**				
**LAG3 positive %**				
**VISTA positive %**				
**PD1 positive %**				
**ICOS positive %**				
**OX40 positive %**				
**CD86 positive %**				
**CD20 positive %**				
**CD206 positive %**				
**CD3 positive %**				
**CD4 positive %**				
**CD8 positive %**				
Burkholderia_contaminans				
Other				
Pseudomonas_aeruginosa				

**Toluene**	8.479_92.0	POS	8.479	92.0627
Janthinobacterium_lividum				
**3,4,5-Trimethoxycinnamic acid**	5.475_238.	POS	5.475	238.0843
**4-Allylphenol sulfate**	5.595_214.	NEG	5.595	214.03
**4-vinylphenol sulfate**	4.697_200.	NEG	4.697	200.0143
**Panomifene**	7.877_427.	POS	7.877	427.1746
**Crenolanib**	7.615_443.	POS	7.615	443.2342
**3-Methylglutaric acid**	3.541_128.	POS	3.541	128.0475
**24S-hydroxycholesterol 3-sulfate, 24-D-glucuronic**	7.636_658.	POS	7.636	658.336
**3-Carboxy-4-methyl-5-propyl-2-furanpropionic a**	7.347_240.	POS	7.347	240.0998
**phenylglucoside**	3.363_256.	NEG	3.363	256.0945
**zaprinast**	4.101_271.	POS	4.101	271.1056
**N-Allylsecoboldine**	7.635_367.	POS	7.635	367.1782
**Asparagine ethyl ester**	3.142_160.	POS	3.142	160.0851
**2,15,16-trihydroxy palmitic acid**	7.98_304.2	POS	7.98	304.2246
**Benzo[g]quinoline**	4.241_179.	POS	4.241	179.0738
**PG(18:3(9Z,12Z,15Z)/18:3(9Z,12Z,15Z))**	7.643_766.	POS	7.643	766.479
**Luminespib**	7.624_465.	POS	7.624	465.2262
**2,4-Dihydroxybenzoic acid**	3.48_154.0	NEG	3.48	154.0267
Lactobacillus_plantarum				
**Dialdehyde**	7.951_368.	POS	7.951	368.1737
**4-{[(5,5,8,8-Tetramethyl-5,6,7,8-tetrahydronaphtha**	7.639_351.	POS	7.639	351.1832
**CDP-DG(16:0/22:3(10Z,13Z,16Z))**	7.625_103	POS	7.625	1031.562
Rothia_mucilaginosa				
**5-aminopentanoate**	2.667_99.0	POS	2.667	99.0685
**Bevirimat**	7.623_584.	POS	7.623	584.4089
**Norbuprenorphine**	7.613_413.	POS	7.613	413.2565
**Ilaprazole**	0.745_366.	POS	0.745	366.1137
**N-(4-Benzyloxybenzyl)acetohydroxamic acid**	7.296_271.	POS	7.296	271.1207
**Methyl gallate**	4.362_184.	POS	4.362	184.0374
**beta-Rhodomycin; Rhodomycin B;**	7.643_543.	POS	7.643	543.2116
**10-Hydroxydecanoic acid**	7.139_188.	NEG	7.139	188.1413
**[(2R,3R,4R,5R)-4-acetyloxy-5-(2,4-dioxopyrimidin-**	7.654_481.	POS	7.654	481.1674
**Leptin F**	8.273_352.	POS	8.273	352.1885
**N-Methyl-2-pyrrolidone**	3.001_99.0	POS	3.001	99.0685
**3-(3-Oxobutanoyloxy)butanoic acid**	3.537_188.	POS	3.537	188.0687
**Methanesulfonic acid; Methanesulfonate;**	0.651_95.9	NEG	0.651	95.98816
**12-[Methyl-(4-nitro-2,1,3-benzoxadiazol-7-yl)ami**	8.52_476.2	NEG	8.52	476.2986
**Benzenepropanoic acid, beta-((2-carboxyethyl)thio**	8.271_442.	POS	8.271	442.2177
**O-Sulfotyrosine**	1.981_261.	POS	1.981	261.0308
**2',3'-Didehydro-2',3'-dideoxyguanosine**	2.677_249.	POS	2.677	249.0862
**3-(3,4,5-Trimethoxyphenyl)propanoic acid**	5.77_240.0	NEG	5.77	240.0997
**23R,acetoxy-3beta,15alpha,24R,25-tetrahydroxy-cy**	8.259_678.	POS	8.259	678.3984
**Posatirelin**	8.424_352.	POS	8.424	352.2101
**(+)-castanospermine**	2.711_189.	NEG	2.711	189.1002
**Losartan N2-glucuronide**	8.295_598.	POS	8.295	598.1958
**N3,N4-Dimethyl-L-arginine**	0.734_202.	POS	0.734	202.1432
Mogibacterium_pumilum				
**From Dolabella auricularia (sea hare)**	7.985_784.	POS	7.985	784.4908
**Tert-butyl N-[2-(prop-2-enamido)ethyl]carbamate**	0.753_214.	POS	0.753	214.1319
**Genistein 4'-O-glucuronide**	7.781_446.	POS	7.781	446.0851
**Ingenol mebutate**	7.748_430.	POS	7.748	430.2356
**Zizybeoside II;**	7.309_594.	POS	7.309	594.2151
**Astacin**	7.989_592.	POS	7.989	592.3552
**Dihydroergotamine**	7.883_583.	POS	7.883	583.282
**NOTA**	3.507_303.	POS	3.507	303.143
Eubacterium_sulci				
**Pentamorphone**	7.919_368.	POS	7.919	368.2098

**Cholesterol phosphate**	9.719_466.POS	9.719	466.32
**1-Hydroxycyclohexyl phenyl ketone**	7.858_204.POS	7.858	204.1154
**Emodin**	7.494_270.POS	7.494	270.0527
**Resiquimod**	7.642_314.POS	7.642	314.1729
**Phenethylamine glucuronide**	6.129_297.POS	6.129	297.1209
**D-(+)-Fucose**	0.731_164.NEG	0.731	164.0684
**1,5-Anhydrosorbitol**	0.731_210.NEG	0.731	210.0739
**3',4'-Anhydrovinblastine;**	8.305_792.POS	8.305	792.4089
**Descyclopropyl Abacavir**	3.383_246.POS	3.383	246.1221
Acinetobacter_johnsonii			
**Eribulin**	8.279_729.POS	8.279	729.4112
**DEHYDROCHOLIC ACID**	7.717_402.POS	7.717	402.2405
**Acetosyringone**	4.681_218.POS	4.681	218.0556
**Lexibulin**	7.297_434.POS	7.297	434.2442
**beta-Alanine, N-(N-(1-carboxy-2-phenylethyl)-L-**	7.464_384.POS	7.464	384.1678
**N-Benzo[1,3]dioxol-5-yl-2-[5-(2,6-dioxo-1,3-dipr**	6.032_496.POS	6.032	496.1715
**Xyloketal**	8.385_456.POS	8.385	456.361
**4-Hydroxy-2-(3-methylbutanoyl)-5-(3-methylbut-**	7.72_362.2POS	7.72	362.2093
**(1Ar,3S,4aS,7S,8aS)-3-[(1S,2R,3R)-3-[(2R,5S)-5,6-d**	10.208_44NEG	10.208	448.3553
**Dihydroferulic acid**	2.978_196.NEG	2.978	196.0736
**Dimethyl pimelimidate**	5.237_186.POS	5.237	186.1371
**Ferroxamine; Ferrioxamine; Desferal-iron(III);**	7.983_613.POS	7.983	613.2653
Aeribacillus_pallidus			
Neisseria_subflava			
**PI(22:4(7Z,10Z,13Z,16Z)/19:0)**	7.671_928.POS	7.671	928.6035
**6alpha-Hydroxycastasterone**	10.206_46NEG	10.206	466.366
**N-(Dimethylamino)methylene-9-((2-hydroxy-1-(h**	7.291_310.POS	7.291	310.139
**PI(22:6(4Z,7Z,10Z,13Z,16Z,19Z)/22:4(7Z,10Z,13Z,16**	7.97_958.5POS	7.97	958.5591
**PHODA-PS**	7.335_707.POS	7.335	707.4006
**13(S)-HOTrE**	7.832_294.POS	7.832	294.2193
**(1R,2S,4R,5S,6S,11R,12R,14S,16R)-5,6,11-trihydrox**	7.846_562.POS	7.846	562.3489
**Kinetin**	3.207_237.POS	3.207	237.0617
**N-Desthienylethyl-rotigotine**	7.407_205.POS	7.407	205.1468
**Disperse Blue 1; 1,4,5,8-Tetraamino-9,10-anthrace**	4.155_268.POS	4.155	268.0962
**LMST01090007**	7.99_1030.POS	7.99	1030.494
**100-1;**	7.987_566.POS	7.987	566.2141
**Epigalanthamin**	7.893_287.POS	7.893	287.1519
**Perazine sulfoxide**	6.619_355.NEG	6.619	355.1714
**3-(2-(N-(1-Carboxy-3-phenylpropyl)amino)propio**	4.101_377.POS	4.101	377.1586
**PI(17:1(9Z)/22:6(4Z,7Z,10Z,13Z,16Z,19Z))**	7.934_894.POS	7.934	894.5249
**Epoxomicin**	7.967_554.POS	7.967	554.3666
Other			
**Burttinonedehydrate**	7.674_420.POS	7.674	420.1916
Leptotrichia_buccalis			
**2-Hydroxy-22-methyltetracosanoic acid**	10.591_39NEG	10.591	398.3762
**Prostaglandin H1**	8.302_336.POS	8.302	336.2273
**Allantoate; Allantoic acid;**	0.642_176.POS	0.642	176.054
**20-Hydroxyleukotriene E4; 20-OH-LTE4; 20-OH-Le**	7.286_455.POS	7.286	455.2341
**Leucine butyl ester**	5.23_187.1POS	5.23	187.1573
**Sarcostin;**	7.744_382.POS	7.744	382.2356
**L-Ascorbic acid 2-sulfate**	0.634_255.NEG	0.634	255.9887
**Pirbuterol**	1.217_240.POS	1.217	240.1476
**PA(12:0/15:0)**	6.898_578.NEG	6.898	578.3935
**Rosterolone**	9.133_346.POS	9.133	346.2872
**NCGC00381130-01!methyl (2E,8E)-9-[3a-hydroxy-**	7.787_431.POS	7.787	431.2671
**[2-[4-hydroxy-2,5-bis(hydroxymethyl)-3-(2-methy**	7.274_706.POS	7.274	706.3789
**Oxypurinol**	1.142_152.NEG	1.142	152.0335
**1,8-Diazabicyclo [5.4.0]undec-7-ene**	4.091_152.POS	4.091	152.1314

1-[(2R,3R,4S,5R)-3,4-Dihydroxy-5-(hydroxymethyl	0.746_244.	POS	0.746	244.0695
2-(2-Benzoylanilino)-3-[4-[2-[methyl(2-pyridinyl)a	7.87_495.2	POS	7.87	495.215
2',4',6'-Trihydroxyacetophenone	3.161_168.	POS	3.161	168.0425
4-(N-Maleimido)phenyltrimethylammonium iodide	7.346_358.	POS	7.346	358.1757
Neisseria_macacae				
Methyl 6-methyl-3-(2-methylpropyl)-4-(3-nitroph	6.59_386.1	POS	6.59	386.1305
Other				
Alloprevotella_tannerae				
Isomatteucinol	7.289_314.	POS	7.289	314.1153
Formononetin 7-O-glucuronide	6.175_444.	POS	6.175	444.1063
Promestrienum	7.843_328.	POS	7.843	328.2402
(2R,4S)-Pyrrolidine-2,4-dicarboxylic acid	1.018_159.	POS	1.018	159.0534
Pseudouridine	1.013_244.	NEG	1.013	244.0693
Lachnoanaerobaculum_umeaense				
Pentanamide	3.706_101.	POS	3.706	101.0842
Luteolin 7-sulfate-3'-rutinoside	4.975_674.	NEG	4.975	674.1165
1-(2-methoxy-heneicosanyl)-sn-glycero-3-phosph	9.146_539.	POS	9.146	539.3926
Acinetobacter_baumannii				
Tolmetin glucuronide	7.697_433.	POS	7.697	433.1363
6-hydroxytetradeca-7,8-dien-10,12-diynoic acid	7.626_232.	POS	7.626	232.1097
Tris(2-ethylhexyl) phosphate	8.88_434.3	POS	8.88	434.3516
2,6R-Dimethyl-2E-octen-1,8-dioic acid	5.625_200.	POS	5.625	200.1051
Isoferulic acid	4.503_194.	POS	4.503	194.0582
Vanillic acid	5.274_168.	POS	5.274	168.0425
Genistein 7-sulfate	5.109_350.	NEG	5.109	350.0098
Sarmentoside B	7.807_664.	POS	7.807	664.3115
1-(5,10-dioxo-2,3,5a,6,7,8-hexahydro-1H-dipyrrol	3.811_351.	POS	3.811	351.1182
Ophthalmic acid	2.335_289.	POS	2.335	289.1273
Parthenium	5.715_270.	POS	5.715	270.1232
(3-{(1r)-3-(3,4-Dimethoxyphenyl)-1-[({(2s)-1-[(2s)	8.419_705.	POS	8.419	705.3161
NCGC00381146-01!2-[5-[2-[2-[5-(2-oxopropyl)ox	7.492_380.	POS	7.492	380.2197
Bis(2-propylheptyl) phthalate	10.034_44	NEG	10.034	446.3397
Nocardioides_daedukensis				
Kocuria_gwangalliensis				
Indole-3-propionic acid	5.86_189.0	POS	5.86	189.0792
Phenylpyruvic acid	3.458_164.	NEG	3.458	164.0475
H-Arg(NO2)-Obzl	7.542_309.	NEG	7.542	309.1448
<U+03A5>-Glutamylcysteine	3.333_250.	POS	3.333	250.0584
Drospirenone	7.3_384.22	POS	7.3	384.23
7-Methoxy tropisetron	7.042_314.	POS	7.042	314.1628
NCGC00385552-01![3-hydroxy-3-methyl-1-(7-oxo	7.309_408.	POS	7.309	408.1175
15-HETE-VA	7.275_455.	POS	7.275	455.3039
Picumast	7.603_440.	POS	7.603	440.1869
2-(5-Chloro-2-fluorophenyl)-N-(pyridin-4-yl)pteri	7.315_352.	POS	7.315	352.064
Anaeroglobus_geminatus				
1-(cis-13-Docosenoyl)-rac-glycerol	10.212_41	NEG	10.212	412.3555
N-(2'-(4-benzenesulfonamide)-ethyl) arachidonoy	9.769_486.	NEG	9.769	486.2899
Methyl bixin/ (Bixin dimethyl ester)	8.428_408.	POS	8.428	408.2301
LTB4 ethanol amide	7.876_379.	POS	7.876	379.2724
9-Methoxy-2,2-dimethyl-2,6-dihydro-pyrano[3,2-	7.298_257.	POS	7.298	257.1052
Cefquinome	7.302_528.	POS	7.302	528.1275
Capnocytophaga_sputigena				
Cicloprolol	7.257_323.	POS	7.257	323.2092
Mucronine A;	7.869_506.	POS	7.869	506.2877
Aripiprazole;	7.956_447.	POS	7.956	447.1484
Brivanib	8.437_370.	POS	8.437	370.1455
Rhodojaponin IV;	7.236_454.	POS	7.236	454.2574
Myxalamid D;	8.333_373.	POS	8.333	373.262

**Paroxetine**	5.162_329.	POS	5.162	329.1431
**N-(6-Chloro-9h-pyrido[3,4-b]indol-8-yl)-3-pyridi**	7.81_322.0	POS	7.81	322.0626
Leuconostoc_citreum				
**Diospolysaponin A**	7.952_786.	POS	7.952	786.4045
Geobacillus_stearothermophilus				
**2-Deoxyglucose**	0.75_186.0	POS	0.75	186.0507
**Avocadene 2-acetate**	9.423_310.	POS	9.423	310.2508
**6-[[3-Fluoro-5-(4-methoxyoxan-4-yl)phenoxy]me**	8.443_397.	POS	8.443	397.1676
**5-Androstenetriol**	8.925_306.	POS	8.925	306.2195
**Pyridin-2-ylmethyl N-[(2S)-1-[[(2S,4S,5S)-4-hydro**	7.297_659.	POS	7.297	659.275
**Sativic acid**	7.489_348.	POS	7.489	348.2508
**2-[[2-[(Dimethylamino)methyl]phenyl]thio]-5-fluo**	6.6_276.11	POS	6.6	276.1109
Other				
Vulcaniibacterium_thermophilum				
**6-Thioxanthylic acid**	5.975_380.	NEG	5.975	380.0204
**20-Cooh Itb4**	7.821_380.	POS	7.821	380.2199
**Hetastarch**	7.294_736.	POS	7.294	736.301
**Quadazocine**	7.604_383.	POS	7.604	383.2825
**Diffusoside D**	7.279_462.	POS	7.279	462.1741
Other				
**Alisol A**	9.309_490.	NEG	9.309	490.3657
**8-Anilino-1-naphthalene sulfonic acid; 1-Anilino-8**	7.016_299.	NEG	7.016	299.0615
Bacillus_licheniformis				
**PG(P-20:0/18:3(9Z,12Z,15Z))**	8.308_784.	POS	8.308	784.5644
**6-[[5-(hydroxymethyl)-2,5,8a-trimethyl-1,4,4a,6,7,**	7.94_400.2	POS	7.94	400.2227
**Gitoxigenin Diacetate**	7.67_474.2	POS	7.67	474.2596
**Imatinib**	6.224_493.	POS	6.224	493.2584
Other				
Aquabacterium_parvum				
**(5Z,7E)-(3S)-26,26,26-Trifluoro-27-nor-9,10-seco-**	7.339_440.	POS	7.339	440.2888
**methyl palmitoleate**	9.319_268.	POS	9.319	268.2403
**Ornithine**	0.846_132.	NEG	0.846	132.09
**PI(20:4(5Z,8Z,11Z,14Z)/22:1(11Z))**	7.939_940.	POS	7.939	940.6017
Other				
**(20R)-24-Hydroxygeminivitamin D3**	10.223_51	NEG	10.223	518.3967
**2-amino-5-(amino-dimethylamino-methylidene)a**	0.966_202.	NEG	0.966	202.143
**1,3(2H,4H)-Isoquinolinedione, 2-(2-(4-(2-methoxy**	7.741_407.	POS	7.741	407.2214
Pedobacter_kyungheensis				
Streptococcus_salivarius				
Other				
Megasphaera_micronuciformis				
Oribacterium_parvum				
Other				
**Olaquindox**	8.372_263.	POS	8.372	263.0905
**Prolinedithiocarbamate**	7.753_147.	POS	7.753	147.0179
**Lipoic acid**	0.648_206.	NEG	0.648	206.0425
**Delgocitinib**	6.573_310.	POS	6.573	310.1529
**Pimelic acid**	5.553_142.	POS	5.553	142.0632
**Visnagin**	6.352_230.	POS	6.352	230.058
**Rimsulfuron;**	4.109_431.	POS	4.109	431.0571
**LysoPC(P-16:0/0:0)**	9.699_479.	POS	9.699	479.3381
**Pyrrolidin-1-yl N,N-bis(sulfanyl)carbamate**	1.211_194.	POS	1.211	194.0188
**9,10-dichloro-octadecanoic acid**	10.196_35	NEG	10.196	352.1937
**Ieodomycin A**	6.753_242.	POS	6.753	242.1518
**15-Keto prostaglandin F1a**	8.356_336.	POS	8.356	336.2274
**3,12-dihydroxy palmitic acid**	8.193_288.	NEG	8.193	288.23
**13,14-Dihydro-15-keto prostaglandin F2a**	7.853_354.	POS	7.853	354.2407
**1-(Tert-Butyl)-3-(2-((4-(diethylamino)butyl)amino**	10.099_52	NEG	10.099	523.3268

**PG(22:0/22:6(4Z,7Z,10Z,13Z,16Z,19Z))**	7.942_878.	POS	7.942	878.6017
**Escitalopram**	9.569_324.	NEG	9.569	324.1624
**Ipurolic acid**	7.973_260.	POS	7.973	260.1991
**Glucobrassicanapin; 4-Pentenyl glucosinolate;**	4.087_387.	POS	4.087	387.0673
**Amafolone**	8.318_305.	POS	8.318	305.2354
**(2S)-2-Amino-3-[(2S)-2-aminopropanoyl]oxyprop**	4.135_176.	POS	4.135	176.0798
**Grayanotoxin I**	7.609_412.	POS	7.609	412.2464
**5-[2-(furan-3-yl)ethyl]-8-hydroxy-5,6,8a-trimethy**	7.786_354.	POS	7.786	354.1809
**Dichlorophenyl-bis-triazolylpropanol**	7.366_338.	POS	7.366	338.0458
**(+/-)12(13)-DiHOME**	9.156_296.	POS	9.156	296.2351
**manoalide**	7.715_416.	POS	7.715	416.2562
**2,2'-Bipyridine**	7.779_156.	POS	7.779	156.0689
**dolichyl-4-D-xylosyl phosphate**	7.606_572.	POS	7.606	572.3464
**Tebipenem**	8.412_383.	POS	8.412	383.0957
**Olivetoric acid**	5.574_494.	POS	5.574	494.1907
**Nilvadipine**	5.692_385.	POS	5.692	385.1273
**(2S,3R,4R)-2-Amino-3,4-dihydroxy-2-(hydroxyme**	8.434_401.	POS	8.434	401.278
Elizabethkingia_anophelis				
Fibrobacter_succinogenes				
**Medicarpin 3-O-glucoside-6'-malonate;**	3.753_518.	NEG	3.753	518.1418
**1,7,8-trihydroxy-3-methyl-2,3,4,7-tetrahydro-1H-**	7.65_310.1	POS	7.65	310.1205
**Starch, acetate**	7.827_734.	POS	7.827	734.285
**Microlenin;**	7.673_494.	POS	7.673	494.2313
**PG(22:1(11Z)/22:6(4Z,7Z,10Z,13Z,16Z,19Z))**	8.312_876.	POS	8.312	876.5856
**L-(+)-Tartaric acid**	0.628_150.	NEG	0.628	150.0165
**Betulonic acid**	7.311_454.	POS	7.311	454.345
**(6RS)-22-hydroxy-23,24,25,26,27-pentanorvitamin**	5.705_394.	POS	5.705	394.2187
**2-Methacryloyloxyethyl phenyl phosphate; Phenyl-**	7.293_286.	POS	7.293	286.0606
**3',5'-Dideoxythymidine**	2.707_210.	POS	2.707	210.1007
**(23S)-23,25-dihdroxy-24-oxovitamine D3 23-(beta**	7.764_606.	POS	7.764	606.3428
**Lipoxin D4**	4.135_512.	POS	4.135	512.2559
**Cortisol lactate**	7.734_450.	POS	7.734	450.2273
**Ganetespib**	7.811_364.	POS	7.811	364.1521
**4,4'-Bis(2-hydroxyethylethylamino)-2,2'-dimethyla**	7.74_384.2	POS	7.74	384.2513
Chryseobacterium_taklimakanense				
**LMPK12113225**	5.679_456.	POS	5.679	456.1763
**beta-D-Gentiobiosyl crocetin;**	6.595_652.	POS	6.595	652.2745
**(3,6,9-trimethylidene-2-oxo-3a,4,5,6a,7,8,9a,9b-oc**	7.743_305.	POS	7.743	305.1642
**Elaeokanine C;**	7.942_211.	POS	7.942	211.1575
**Tulipinolide**	6.069_312.	POS	6.069	312.1338
**LMPK12010025**	7.936_727.	POS	7.936	727.2096
**(2S)-2-[(1S)-1-carboxyundecyl]-5-oxotetrahydrofu**	7.21_328.1	NEG	7.21	328.1886
**6-Methylthioinosine**	2.77_298.0	POS	2.77	298.0744
**Indole-3-lactic acid**	3.154_205.	NEG	3.154	205.0738
**PKDdiA-PG**	7.92_708.3	POS	7.92	708.3842
**2-Propenoic acid, 3-(3,4-dihydroxyphenyl)-, 2-phe**	7.681_284.	POS	7.681	284.1048
Other				
**Isopropyl 4-hydroxybenzoate**	7.347_180.	POS	7.347	180.0788
**Pyrimethamine**	2.658_248.	POS	2.658	248.0833
**19-Hydroxyprostaglandin E2**	7.851_368.	POS	7.851	368.2199
**GM4 Ganglioside**	7.29_736.4	POS	7.29	736.4699
**Saracatinib**	6.282_541.	POS	6.282	541.2071
**3-Methyl-2-phenyl-N-[(1S)-1-phenylpropyl]quino**	5.609_380.	POS	5.609	380.1878
**1,1,1-Trichloroheptane**	0.876_202.	POS	0.876	202.0092
**Thymol blue**	6.19_466.1	POS	6.19	466.1815
**3-[18-(2-Carboxyethyl)-8,13-diethyl-3,7,12,17-tet**	5.136_568.	POS	5.136	568.3075
**Genistein 4',7-O-diglucuronide**	6.598_622.	POS	6.598	622.1169
**Alanyltyrosine**	4.332_252.	POS	4.332	252.1111

**Nepsilon,nepsilon,nepsilon-trimethyllysine**	0.648_188.	POS	0.648	188.1527
**2,3-Dinor-8-iso prostaglandin F1alpha; 2,3-Dinor-8**	7.773_328.	POS	7.773	328.2247
**Esmolol**	7.839_295.	POS	7.839	295.1782
**Gentamicin C**	7.233_449.	POS	7.233	449.2864
**N-(Carboxymethyl)-N-(2-((carboxymethyl)amino)e**	4.086_234.	POS	4.086	234.0853
**Norepinephrine**	2.858_169.	POS	2.858	169.0741
Exiguobacterium_mexicanum				
**Itaconate**	0.641_130.	NEG	0.641	130.0267
**PA(O-16:0/0:0)**	10.061_39	POS	10.061	396.2642
**Irtemazole**	7.341_288.	POS	7.341	288.1377
Granulicatella_elegans				
**(S)-4-(2-(4-Amino-1,2,5-oxadiazol-3-yl)-1-ethyl-7**	4.089_425.	POS	4.089	425.2161
**Guanine**	2.816_151.	POS	2.816	151.0496
**(R)-6-(4-((4-Ethylpiperazin-1-yl)methyl)phenyl)-N**	9.786_440.	NEG	9.786	440.2709
**(2S,3S,4S,5R,6R)-6-(3-benzoyloxy-2-hydroxypropo**	4.011_394.	POS	4.011	394.0877
**Filipin II**	7.827_638.	POS	7.827	638.4033
**Finerenone**	5.618_378.	POS	5.618	378.1706
**Vitexin 7-O-sulfate**	5.652_512.	NEG	5.652	512.0622
**2,3-Dihydroxypropyl dodecanoate**	7.697_274.	POS	7.697	274.2145
**5-Carboxytetramethylrhodamine succinimidyl ester**	7.833_527.	POS	7.833	527.1696
**4-Hydroxychlorothalonil**	6.457_245.	NEG	6.457	245.9153
**MG(0:0/24:1(15Z)/0:0)**	10.211_44	NEG	10.211	440.3867
**6-Methyl-3E,5-heptadien-2-one**	5.789_124.	POS	5.789	124.089
**Valproic acid**	6.413_144.	NEG	6.413	144.1151
**Prorocentrolide;**	9.126_979.	POS	9.126	979.6064
**2-Hydroxy-5-(trifluoromethoxy)benzoic Acid**	1.215_222.	POS	1.215	222.0135
**Celecoxib**	7.916_381.	POS	7.916	381.0758
**3-Hydroxytetradecanedioic acid**	7.805_274.	POS	7.805	274.1777
**Chlorethylclonidine**	7.322_334.	POS	7.322	334.0535
**Caproic acid**	9.443_97.9	POS	9.443	97.97704
**Adipate**	4.864_146.	POS	4.864	146.0581
Lactococcus_lactis				
**DL-a-Aminocaprylic acid**	4.661_159.	POS	4.661	159.1261
**Palmatoside G;**	7.288_492.	POS	7.288	492.1973
**MG(20:4(8Z,11Z,14Z,17Z)/0:0/0:0)**	8.331_378.	POS	8.331	378.277
**Ruscogenin**	9.691_430.	POS	9.691	430.3086
**4-Dodecylbenzenesulfonic Acid**	10.015_32	NEG	10.015	326.1915
**Neopikromycin;**	7.846_525.	POS	7.846	525.3311
**Contignasterol**	8.343_508.	POS	8.343	508.3407
**3-keto Fusidic acid**	8.784_530.	POS	8.784	530.3229
**5-hydroxy-2,2-dimethyl-10-(2-methylbut-3-en-2-**	6.103_334.	POS	6.103	334.1164
**9,10-Dihydroxystearate;**	8.779_316.	POS	8.779	316.2612
**Visoltricin**	6.19_234.1	POS	6.19	234.137
**Neocuproine**	4.314_208.	POS	4.314	208.1003
Arthrobacter_soli				
Arcticibacter_pallidicorallinus				
**3-(4-(2-Dimethylamino-1-methylethoxy)phenyl)-1**	6.615_354.	NEG	6.615	354.1679
**Cetirizine**	7.289_388.	NEG	7.289	388.1558
**9-[2-(Phosphanylmethoxy)propyl]purin-6-amine**	2.573_239.	POS	2.573	239.0947
**3-Hydroxysebacic acid**	3.662_218.	NEG	3.662	218.1153
Other				
**Antibiotic K 252a**	4.138_467.	POS	4.138	467.1474
**Tranylcypromine**	8.287_133.	POS	8.287	133.0894
**4-Oxo-9-cis-retinoyl-beta-glucuronide**	7.719_490.	POS	7.719	490.2206
**2-Hydroxydodecyl methacrylate**	8.896_270.	POS	8.896	270.2195
**Rotigotine**	7.039_315.	POS	7.039	315.1662
**5-Formyl-2H-pyran-6-carboxamide**	3.106_153.	POS	3.106	153.0428
**PA(14:1(9Z)/15:0)**	8.33_604.4	POS	8.33	604.4093

**Thiobenzyl benzyloxycarbonyl-L-lysinate**	4.153_386.	POS	4.153	386.1682
**(+/-)9(10)-EpOME**	10.132_59.	NEG	10.132	592.4707
**ISOROTENONE**	7.748_416.	POS	7.748	416.1224
**N-Acetyl-L-alanine**	1.16_131.0	POS	1.16	131.0585
**Amino leucine**	4.458_146.	POS	4.458	146.1057
**4-[3-(4-Chlorophenyl)-1,2,4-oxadiazol-5-yl]butan**	5.615_266.	POS	5.615	266.0463
**Epoxypheophorbide a;**	8.399_608.	POS	8.399	608.2631
**2,3-Dinor-TXB1**	7.915_342.	POS	7.915	342.2406
Elusimicrobium_minutum				
**N-(5-(((5-(1,1-Dimethylethyl)-2-oxazolyl)methyl)t**	4.136_380.	POS	4.136	380.1327
**[(2R,5S)-5-(Octadecylcarbamoyloxymethyl)oxolan-**	8.436_662.	POS	8.436	662.4077
Prevotella_pallens				
**Cinncassiol B;**	7.839_400.	POS	7.839	400.2097
**Skullcapflavone II**	8.376_374.	POS	8.376	374.1
TM7_phylum				
**1-(O-alpha-D-glucopyranosyl)-(1,3R,25R)-hexacos**	9.449_576.	POS	9.449	576.4607
**fructose-6-phosphate lactate**	6.154_362.	POS	6.154	362.0241
Butyrivibrio_hungatei				
**methyl 9-(alpha-D-galactosyloxy)nonanoate**	5.504_350.	POS	5.504	350.1933
**Docosatrienoic acid**	10.608_33	NEG	10.608	334.2871
**N-Methacryloyl-L-glutamic acid**	3.444_215.	POS	3.444	215.0796
**PS(O-16:0/22:2(13Z,16Z))**	10.628_80	POS	10.628	801.5888
**zinniol**	4.463_288.	POS	4.463	288.1322
**N-(3-oxo-decanoyl)-homoserine lactone**	7.26_269.1	POS	7.26	269.1626
**N-Acetyldemethylphosphinothricin tripeptide;**	4.099_351.	POS	4.099	351.1193
**2R-hydroxy-hexacosanoic acid**	10.865_41.	NEG	10.865	412.3919
Obesumbacterium_proteus				
Aquamicrobium_aerolatum				
Geothrix_fermentans				
Taibaiella_koreensis				
Faucicola_mancuniensis				
Peredibacter_starrii				
Buchnera_aphidicola				
Bacillus_marisflavi				
Marinilactibacillus_piezotolerans				
Vogesella_perlucida				
Phenylobacterium_haematophilum				
Peptoniphilus_gorbachii				
Beijerinckia_indica				
**2-Hydroxydecanedioic acid**	4.868_218.	POS	4.868	218.1154
**Dicyclomine**	7.772_309.	POS	7.772	309.2665
**(1R)-5-[2-[(1S,7As)-1-[(1S)-1-(3-hydroxy-3-meth**	9.703_418.	NEG	9.703	418.3084
**Homocholic acid**	8.336_422.	POS	8.336	422.3034
**2,7,7,11,15,17-hexamethyl-18-methylidene-5,13,1**	6.238_480.	POS	6.238	480.2111
**Thiamine acetic acid; Thiamin acetic acid;**	8.281_279.	POS	8.281	279.0928
**6-Hydroxyindolelactate;**	5.379_221.	POS	5.379	221.069
**Motesanib**	7.92_373.1	POS	7.92	373.1887
**Cenicriviroc**	7.752_696.	POS	7.752	696.373
Other				
Limnobacter_thiooxidans				
**Benzo[a]pyrene-7,8-dihydrodiol-9,10-oxide; 7,8-D**	4.118_302.	POS	4.118	302.0937
**NCGC00385536-01!(2E,4E)-N-(1,3-dihydroxy-3-m**	4.75_241.1	POS	4.75	241.1679
**Roxatidine acetate**	7.743_348.	POS	7.743	348.2045
**Alinamin**	6.362_356.	POS	6.362	356.1346
**PS(O-16:0/22:4(7Z,10Z,13Z,16Z))**	10.452_79	POS	10.452	797.5572
**Apigenin**	8.339_270.	POS	8.339	270.0529
**Quercetin 3-methyl ether 5-glucoside-3'-sulfate**	3.785_558.	NEG	3.785	558.0685
**(S)-Boldine**	3.476_349.	POS	3.476	349.1272

**Aryl sulfate; Phenol sulfate;**	2.845_173.	NEG	2.845	173.9987
**Morellin;**	7.235_544.	POS	7.235	544.2481
**Clopidogrelum**	7.812_321.	POS	7.812	321.0586
**lasalocid**	8.336_590.	POS	8.336	590.382
**agnuside**	7.714_466.	POS	7.714	466.1453
**Iprofenin**	6.977_308.	NEG	6.977	308.137
**N-(2-Fluoro-4-(methylsulfonyl)phenyl)-6-(4-(3-is**	4.144_505.	POS	4.144	505.156
**Methylpyrogallol sulfate 3**	3.003_220.	NEG	3.003	220.004
**Nicotinuric acid**	2.407_180.	NEG	2.407	180.0536
**Neopterin**	6.799_253.	NEG	6.799	253.0803
**Dioctyl sebacate**	10.352_42	NEG	10.352	426.3711
**OKHdiA-PA**	7.235_590.	POS	7.235	590.2856
**Benzenesulfonamide**	2.907_157.	POS	2.907	157.02
**Nep-IN-2**	1.372_341.	NEG	1.372	341.111
**Cloricromen**	7.334_395.	POS	7.334	395.148
**Dienestrol**	7.711_266.	POS	7.711	266.1307
**Solacetal C**	7.859_478.	POS	7.859	478.2935
**Lusitanicoside**	7.247_464.	POS	7.247	464.166
**Ioxitalamic acid**	8.771_643.	POS	8.771	643.779
**(+)-Brusatol**	7.93_520.1	POS	7.93	520.1965
**28-Glucosylarjunolate 3-[rhamnosyl-(1->3)-glucur**	9.129_972.	POS	9.129	972.4939
**Capryloylglycine**	5.667_201.	NEG	5.667	201.1365
Thiomonas_delicata				
Chelatococcus_caeni				
Thermomonas_hydrothermalis				
Colwellia_maris				
Sporolituus_thermophilus				
Thiothrix_flexilis				
Acidisoma_sibiricum				
Sphingobacterium_thermophilum				
Brevibacillus_thermoruber				
Laceyella_putida				
Other				
Winogradskyella_echinorum				
Oleispira_antarctica				
Other				
Other				
Sulfitobacter_undariae				
Acidithiobacillus_albertensis				
Other				
Celeribacter_neptunius				
**4-Hydroxyquinazoline**	4.129_146.	POS	4.129	146.0482
**Milameline**	3.998_154.	POS	3.998	154.1109
**(E)-5-hydroxy-N-[3-[5-[3-[[(E)-5-hydroxy-3-meth**	8.582_469.	POS	8.582	469.289
**(2S)-2-Amino-N-[(1S)-7,7-dimethyl-1-[[4-(2-meth**	8.856_554.	POS	8.856	554.259
**Pefurazoate;**	6.137_345.	POS	6.137	345.1684
**Diphacinone**	5.657_340.	POS	5.657	340.1093
Chryseobacterium_anthropi				
**Ansamitocin P-3**	7.985_634.	POS	7.985	634.2635
**Docosa-4,7,10,13,16,19-hexaenoic acid ethyl ester**	10.265_35	NEG	10.265	356.2717
**trans,octacis-Decaprenylphospho-beta-D-erythro-**	8.238_908.	POS	8.238	908.6338
**Heteratisine;**	4.218_391.	POS	4.218	391.2356
**Oseltamivir**	7.677_312.	POS	7.677	312.2048
**9Z,12E-Octadecadienoic acid**	10.016_28	POS	10.016	280.2402
**Perchloric acid**	0.713_99.9	NEG	0.713	99.95634
**m-Hydroxybenzoylecgonine**	5.659_305.	POS	5.659	305.1257
Other				
**Dexamethasone isonicotinate**	7.681_497.	POS	7.681	497.2219

**Propapyriogenin A2;**	8.328_484.POS	8.328	484.3196
**E-3179**	8.269_420.POS	8.269	420.1466
**Fenethylline**	4.09_341.1POS	4.09	341.1837
**[3-(2-Aminopropyl)-6-methylidenecyclohexa-1,3-**	5.472_195.POS	5.472	195.1263
**Methocarbamol**	4.089_241.POS	4.089	241.0951
**Tanshinone IIA**	4.09_294.1POS	4.09	294.1214
**Canrenone**	7.287_340.POS	7.287	340.2039
**Sibenadet**	7.705_464.POS	7.705	464.1448
Tepidimonas_fonticaldi			
Moraxella_osloensis			
**Everolimus**	7.761_957.POS	7.761	957.5803
**arthrobactin**	8.359_498.POS	8.359	498.2325
**2-Cyclohexylidenecyclohexanone**	7.189_178.POS	7.189	178.136
**4-amino-2-hydroxy-5-[[1-hydroxy-1-(5-oxo-6-bi**	4.127_343.POS	4.127	343.1738
**18-hydroxy-9S,10R-dihydroxy-stearic acid**	7.366_332.POS	7.366	332.2561
**N-linolenoyl glutamic acid**	7.607_407.POS	7.607	407.2671
**3-((5-(2,3-Dichlorophenyl)-1H-tetrazol-1-yl)meth**	4.192_305.POS	4.192	305.0235
**Labetalol**	5.394_328.POS	5.394	328.1798
**1-(O-alpha-D-galactopyranosyl)-(1,3R,27S,29R)-tr**	9.654_648.POS	9.654	648.5181
Sphingobacterium_daejeonense			
**Uridine**	1.155_244.NEG	1.155	244.0693
Aeromonas_salmonicida			
**2-{2-[3-(2-Carbamimidoylsulfanyl-ethyl)-phenyl]-**	2.606_282.POS	2.606	282.0964
**Forsythoside B**	8.392_756.POS	8.392	756.2495
**Nonanoic acid**	7.896_314.POS	7.896	314.2455
**Acridine**	7.443_179.POS	7.443	179.0736
**Pirenzepine**	7.735_351.POS	7.735	351.1696
**Thalassotalic acid C**	7.355_319.POS	7.355	319.1785
**Thromboxane B2**	3.758_392.POS	3.758	392.2158
**6,7-dihydroxy stearic acid**	8.725_316.NEG	8.725	316.2613
**Prulaurasin**	3.868_312.POS	3.868	312.132
Schlegelella_aquatica			
Chryseobacterium_lactis			
**O-Demethyl apixaban**	4.148_445.POS	4.148	445.1751
**8,8a-Diepiswainsonine**	0.74_173.1POS	0.74	173.1053
Prevotella_salivae			
Prevotella_maculosa			
Slackia_exigua			
Prevotella_multiformis			
Parascardovia_denticolens			
Propionibacterium_acidifaciens			
Treponema_socranskii			
Streptococcus_constellatus			
Prevotella_veroralis			
Selenomonas_infelix			
Pseudomonas_peli			
Oribacterium_asaccharolyticum			
Selenomonas_sputigena			
Sphingobacterium_composti			
Mogibacterium_timidum			
Actinomyces_cardiffensis			
Tessaracoccus_lapidicaptus			
Treponema_maltophilum			
Shuttleworthia_satelles			
Other			
Chryseobacterium_geocarposphaerae			
Stomatobaculum_longum			
Tannerella_forsythia			

Mobiluncus_curtisii				
**Tabimorelin**	8.374_528.	POS	8.374	528.3122
**2-O-[6-O-Octanoyl-alpha-D-glucopyranosyl-(1->**	7.998_556.	POS	7.998	556.2375
**Primidolol;**	3.139_333.	POS	3.139	333.1689
**3-Desmethylcolchicine**	2.438_385.	NEG	2.438	385.1533
Corynebacterium_deserti				
**D-Saccharic acid**	0.63_210.C	NEG	0.63	210.0376
**C8-sterol isomerase inhibitor Naegleria fowleri Am**	7.788_706.	POS	7.788	706.3577
**NETILMICIN SULFATE**	4.083_595.	POS	4.083	595.249
**Hippuryl-L-lysine**	7.238_307.	POS	7.238	307.1531
**2-[[2-(4-Hydroxyphenyl)ethylamino]methyl]-3,4-d**	4.047_295.	POS	4.047	295.1579
**Clocapramine**	8.419_480.	POS	8.419	480.2664
**Auricularine;**	10.002_49	POS	10.002	494.3401
**2-(Ethoxymethyl)-1h-Imidazo[4,5-C]quinolin-4-Ar**	4.797_242.	POS	4.797	242.1156
**Indolelactic acid**	4.902_205.	POS	4.902	205.0741
**Licochalcone A**	4.662_338.	POS	4.662	338.1477
**16-Glutaryloxy-1alpha,25-dihydroxyvitamin D3**	8.338_546.	POS	8.338	546.3566
**Indazole**	2.201_118.	POS	2.201	118.0533
**1-Methyl-3-(2-oxopropylidene)indol-2-one**	5.704_201.	POS	5.704	201.0792
**Verimol A**	7.976_298.	POS	7.976	298.1205
**Red chlorophyll catabolite;**	8.136_626.	POS	8.136	626.2742
**Robustaol A;**	7.237_474.	POS	7.237	474.1909
**Androsterone sulfate**	7.443_370.	NEG	7.443	370.1816
**Furagin**	5.619_264.	POS	5.619	264.0508
Actinomyces_odontolyticus				
**Sucrose, 1-laurate**	4.986_524.	POS	4.986	524.2817
**LysoPI(16:0/0:0)**	9.657_572.	NEG	9.657	572.297
**Zygadenine;**	7.191_493.	POS	7.191	493.3042
**Palomid 529**	7.708_406.	POS	7.708	406.1416
Other				
**beta-Casomorphin-7**	7.646_692.	POS	7.646	692.3528
**1'-Hydroxybufuralol**	7.495_277.	POS	7.495	277.1678
**(r)-malate**	0.641_134.	NEG	0.641	134.0216
**Pteroic acid**	3.393_312.	POS	3.393	312.0959
**Norethindrone**	7.835_298.	POS	7.835	298.1928
**6,8-dihydroxy-2,2,4,4-tetramethyl-7-(3-methylbut**	7.158_460.	POS	7.158	460.2463
**2-(beta-D-Mannopyranosyl)-L-tryptophan**	2.471_366.	POS	2.471	366.1425
**NCGC00347754-02!(5-benzoyloxy-4,6-dihydroxy-**	7.306_380.	POS	7.306	380.1286
**Methyl isoeugenol**	6.457_178.	NEG	6.457	178.0994
**Piperine**	7.696_285.	POS	7.696	285.1364
**PS(O-20:0/20:5(5Z,8Z,11Z,14Z,17Z))**	10.498_82	POS	10.498	823.5734
**(S)-Homostachydrine**	4.439_157.	POS	4.439	157.1105
**Vitamin A**	7.872_286.	POS	7.872	286.2295
Massilia_suwonensis				
Other				
**Mecadox**	5.367_262.	NEG	5.367	262.069
Actinomyces_graevenitzii				
**Hydroxycelecoxib**	6.189_397.	POS	6.189	397.0706
**7-Hexadecynoic acid**	9.244_252.	POS	9.244	252.209
Sphingomonas_glacialis				
Other				
**Diprafenone**	7.778_369.	POS	7.778	369.2295
**Tinidazole**	5.621_247.	POS	5.621	247.0633
**N-arachidonoyl D-serine**	7.272_391.	POS	7.272	391.2724
**Cefcapene**	5.681_453.	POS	5.681	453.0763
Other				
Brevundimonas_poindexterae				
Other				

Sphingobacterium_nematocida				
Prevotella_bryantii				
Sulfuricella_denitrificans				
Arenibacter_troitsensis				
Snodgrassella_alvi				
**delta10-13-PhytoF**	6.566_344.	POS	6.566	344.2198
**Asebotoxin**	7.727_412.	POS	7.727	412.2462
**4-keto pentadecanoic acid**	7.696_256.	POS	7.696	256.2041
**4-(Dodecylamino)Phenol**	9.453_277.	POS	9.453	277.2406
**Rehmaionoside B;**	4.102_390.	POS	4.102	390.2243
**[(3R,4S,8S,8aS)-3-hydroxy-6,8a-dimethyl-8-[(Z)-2-**	5.84_478.2	POS	5.84	478.2334
**Cefaclor**	7.364_367.	POS	7.364	367.0378
**L-Theanine**	0.842_174.	POS	0.842	174.112
**o6-Benzyl-8-oxoguanine**	3.903_257.	POS	3.903	257.09
Other				
**1-O-(8R-hydroxy-8-methyl-3Z,9-decadienoyl)-be**	6.893_360.	POS	6.893	360.1785
**Benzo(1,2-b:4,3-b')dipyrrole-2-carboxylic acid, 3,6,**	8.372_291.	POS	8.372	291.0853
**Trandolaprilat**	5.853_402.	POS	5.853	402.2155
**Oleate**	9.674_299.	POS	9.674	299.2824
Other				
Other				
**DI(Hydroxyethyl)ether**	5.62_106.C	POS	5.62	106.0631
**Ketocaine**	6.188_291.	POS	6.188	291.2197
**1-Acetylproline**	3.158_157.	POS	3.158	157.0741
**Nvp-aew541**	7.82_439.2	POS	7.82	439.2393
Other				
**N-Acetylcarnosine**	0.747_268.	POS	0.747	268.1172
**Mannitol**	0.697_182.	NEG	0.697	182.0791
**3'-N-Debenzoyltaxol; 13-O-3-Phenylisoserinoyl ba**	7.952_749.	POS	7.952	749.3085
**Aminopentol**	8.426_405.	POS	8.426	405.3455
**N-Acetyl-L-tyrosine**	3.464_223.	POS	3.464	223.0849
Tepidiphilus_succinatimandens				
**Heptadecanoyl carnitine**	8.909_413.	POS	8.909	413.3506
**Methylmercury; Methylmercury ion; CH3Hg+;**	0.689_216.	POS	0.689	216.9942
**Hexa-D-arginine**	8.398_953.	POS	8.398	953.6341
**Doxorubicinolone**	4.234_416.	POS	4.234	416.1104
**N2-Malonyl-D-tryptophan;**	5.536_290.	POS	5.536	290.0902
**8-Prenyllepidissipyrone**	5.592_490.	POS	5.592	490.1997
**3'-Azido-2',3'-dideoxyuridine 5'-(1,4-dihydro-1-m**	4.104_374.	POS	4.104	374.1333
Stenotrophomonas_nitritireducens				
**6-Hydroxyketamine**	4.08_253.C	POS	4.08	253.087
**PG(14:0/17:1(9Z))**	7.751_706.	POS	7.751	706.4786
**Clonixin**	5.621_262.	POS	5.621	262.0503
**5-Methylangelicin;**	7.804_200.	POS	7.804	200.0475
**3-carboxy-4-methyl-5-pentyl-2-furanpropanoic a**	8.001_268.	POS	8.001	268.1313
**Dimethyl sulfone**	0.941_94.C	POS	0.941	94.00899
Other				
**Butenylcarnitine**	4.302_229.	NEG	4.302	229.1314
**Retinal**	7.982_284.	POS	7.982	284.2139
**5-Methyltetrahydrofolic acid**	7.79_459.1	POS	7.79	459.1829
Fluviicola_hefeinensis				
Vibrio_rumoiensis				
Rubinisphaera_brasiliensis				
Acidovorax_caeni				
Catabacter_hongkongensis				
Arcobacter_butzleri				
Other				
Lactobacillus_dextrinicus				

Asticcacaulis_excentricus				
Rheinheimera_tangshanensis				
Arcobacter_ellisii				
Other				
Phenylobacterium_falsum				
Thermobacillus_xylanilyticus				
Brevundimonas_aurantiaca				
Other				
Microcystis_aeruginosa				
Other				
Skermanella_stibiiresistens				
**1-((2-Methyl-5-methylene-5-oxotetrahydrofuran-**	2.988_275.	POS	2.988	275.1007
**CerP(d18:1/14:0)**	10.429_58	POS	10.429	589.4478
**11Z-Hexadecen-7,9-diynoic acid**	9.12_246.1	POS	9.12	246.1621
**2-quinolinecarboxylic acid**	3.828_173.	POS	3.828	173.0479
**PS(18:1(9Z)/0:0)**	9.765_523.	NEG	9.765	523.2906
**NCGC00380635-01![(2R,3R,4S,5S,6R)-3-acetyloxy-**	7.784_630.	POS	7.784	630.3615
Corynebacterium_accolens				
**Alpidem**	6.389_403.	POS	6.389	403.12
**1-Methyladenosine**	1.031_281.	POS	1.031	281.1124
**Porphobilinogen**	4.83_226.0	NEG	4.83	226.097
**S-Nitrosohomocysteine**	1.075_164.	POS	1.075	164.0259
**Luteolin 7-glucuronide-4'-rhamnoside**	5.827_608.	POS	5.827	608.1383
**PE-Cer(d14:2(4E,6E)/21:0)**	10.564_67	POS	10.564	672.5202
**Bis(3-methoxysalicylidene)ethylenediamine**	7.757_328.	NEG	7.757	328.1423
**4-Trimethylammoniobutanoic acid**	3.405_145.	POS	3.405	145.1105
**Phytolaccoside B; Esculentoside B;**	7.193_664.	POS	7.193	664.3832
**12a-HYDROXY-5-DEOXYDEHYDROMUNDUSERON**	5.74_342.1	POS	5.74	342.1104
**Trimethylamine N-oxide**	0.73_75.06	POS	0.73	75.06853
**bhas#20**	6.389_362.	POS	6.389	362.2303
**(1S,4aS,7aS)-7-[[(E)-3-phenylprop-2-enoyl]oxyme**	4.083_521.	POS	4.083	521.1907
**Epristeride**	8.4_399.27	POS	8.4	399.2773
**methyl (4aR,6S,7R,7aS)-4a,7-dihydroxy-7-methyl-(**	7.32_552.1	POS	7.32	552.1829
**Mycinamicin III;**	7.385_681.	POS	7.385	681.4079
**(+)-Dihydrokavain**	2.962_254.	POS	2.962	254.0904
**9,12,13-TriHOME**	7.688_330.	NEG	7.688	330.2406
**Pregna-5,16,20-triene-3beta,20-diol diacetate;**	7.965_398.	POS	7.965	398.2456
Olsenella_profusa				
**ascr#36**	9.446_458.	NEG	9.446	458.3608
**Norfenefrine**	3.834_153.	POS	3.834	153.0792
Haemophilus_parainfluenzae				
**1,8-Diazacyclotetradecane-2,9-dione;**	3.372_226.	POS	3.372	226.1683
**Cyclopropanecarboxamide, 1,2,2,3,3-pentamethyl-**	4.977_155.	POS	4.977	155.1313
**Apomine**	8.096_562.	NEG	8.096	562.3183
**Creatine**	0.731_131.	NEG	0.731	131.0696
**Creatinine**	0.737_113.	NEG	0.737	113.059
**Q-VD-OPh**	3.726_513.	POS	3.726	513.1721
**4-methyl-5-carboxyethyl-2-furanpentanoic acid**	7.494_254.	POS	7.494	254.1152
**Sotagliflozin**	7.793_424.	POS	7.793	424.1127
**Myxochelin C;**	6.214_539.	POS	6.214	539.1912
**2,3-Dihydroxypropyl octanoate**	7.219_218.	POS	7.219	218.1519
**Leukotriene E3**	7.892_441.	POS	7.892	441.2556
**3-[2-Methoxy-4-[3-methoxy-4-(2-phenyl-1H-tetr**	7.236_627.	POS	7.236	627.2368
**Irbesartan**	7.226_428.	POS	7.226	428.2322
**TTPA**	7.289_568.	POS	7.289	568.2895
**(11S)-11-Benzyl-6-chloro-1,2,10,11,12,13,14,15,16,**	7.769_508.	POS	7.769	508.2365
**Methyl 2-(3-(((3-(6-amino-2-butoxy-8-oxo-7,8-di**	6.815_569.	POS	6.815	569.3327
**L-Histidine**	0.713_155.	NEG	0.713	155.0696

**2-Isopropylthioxanthone**	4.807_232.	POS	4.807	232.0947
**[6-Hydroxy-8-methyl-3-[3,4,5-trihydroxy-6-(hydr**	4.225_480.	POS	4.225	480.1641
Pseudoxanthomonas_taiwanensis				
Sphaerobacter_thermophilus				
**Grazoprevir**	7.744_766.	POS	7.744	766.3359
**Pracinostat**	6.568_358.	POS	6.568	358.2355
**Harmine**	3.713_212.	POS	3.713	212.0949
**N-(3-oxo-heptanoyl)-homoserine lactone**	3.304_227.	POS	3.304	227.1159
Other				
**Crinamidine;**	6.363_317.	POS	6.363	317.1261
**17beta-Hydroxy-4-mercaptoandrost-4-en-3-one 4**	5.616_418.	POS	5.616	418.2182
Other				
**Fluoromisonidazole**	5.237_189.	POS	5.237	189.0554
**2-(3-(2-Aminoethyl)-1h-indol-5-yloxy)acetyl-l-tyr**	6.2_453.20	POS	6.2	453.2
**Cafenstrole;**	3.073_350.	POS	3.073	350.1413
**5-sulfooxymethylfurfural**	1.785_205.	NEG	1.785	205.9884
**(5E,8E,11E)-13-[(2S,3S)-3-Pentylthiiran-2-yl]tridec**	9.735_336.	POS	9.735	336.2121
**(7R,8R)-7,8-dihydroxy-3,5,7-trimethyl-8H-isochro**	3.772_222.	POS	3.772	222.0893
**[(2E)-2-hexa-2,4-diynylidene-1,6-dioxaspiro[4.4]n**	6.166_313.	POS	6.166	313.1314
**N-lactoyl-Tyrosine**	6.336_253.	POS	6.336	253.0951
**Equol 4'-O-glucuronide**	7.704_418.	POS	7.704	418.1263
**(11R,16S)-misoprostol**	9.537_382.	POS	9.537	382.272
**CerP(d18:1/12:0)**	10.154_56	POS	10.154	561.4161
**Valsartan**	7.739_435.	POS	7.739	435.2272
Singulisphaera_rosea				
**Eurycomanol**	4.26_410.1	POS	4.26	410.1595
**Taurochenodeoxycholate-3-sulfate**	7.171_579.	NEG	7.171	579.2542
**Benzylpenicilloyl G**	7.683_465.	POS	7.683	465.1953
**4-[1-(1,3-benzodioxol-5-yl)propan-2-yl]-4,5-dime**	7.705_356.	POS	7.705	356.1622
**N-butyl arachidonoyl amine**	10.079_35	POS	10.079	359.3185
**Alatrofloxacin**	5.078_558.	POS	5.078	558.1822
**Isobutyrylmallotochromene;**	6.653_470.	POS	6.653	470.1919
**25-Hydroxy-atrotosterone A**	7.843_510.	POS	7.843	510.3204
**9,10-Epoxystearic acid**	9.384_298.	POS	9.384	298.2507
**Tebufenpyrad**	5.935_333.	POS	5.935	333.1609
**Mevalonate-PP**	0.621_308.	NEG	0.621	308.0048
**N-ACETYLPROLINE**	3.009_157.	POS	3.009	157.0741
**O(6)-Methyl-2'-deoxyguanosine**	0.737_281.	POS	0.737	281.1123
**Epanolol**	6.996_369.	POS	6.996	369.1687
**2,6-Ditert-butyl-4-[(3,5-ditert-butyl-4-hydroxyph**	7.86_488.2	POS	7.86	488.2763
**Nalbuphine**	7.739_357.	POS	7.739	357.1939
**Chletric acid**	8.328_518.	POS	8.328	518.3614
Other				
Skermanella_aerolata				
**a-Linolenoyl ethanolamide**	9.526_321.	POS	9.526	321.2667
**Kaempferol 3-rhamno-glucoside**	6.177_594.	POS	6.177	594.1593
**7-[(1R,2R,3R,5S)-3,5-Dihydroxy-2-[(3S)-3-hydroxy**	7.854_415.	POS	7.854	415.2723
**Aranidipine**	6.936_388.	POS	6.936	388.1272
**3-Furoic acid**	0.642_112.	NEG	0.642	112.0161
Other				
**2-[[9-(1-Methylethyl)-6-[(phenylmethyl)amino]-9**	7.783_354.	POS	7.783	354.2169
Stenotrophomonas_rhizophila				
**Ursocholanic Acid**	10.807_36	NEG	10.807	360.3029
**Ecadotril**	7.704_385.	POS	7.704	385.135
**9R,10S,18-trihydroxy-stearic acid**	6.949_332.	NEG	6.949	332.2563
Methylobacterium_goesingense				
**Genistin**	6.634_432.	POS	6.634	432.1056
**Desmethyl rizatriptan**	5.219_255.	POS	5.219	255.1486

**Allopurinol**	1.041_136.	POS	1.041	136.0387
**3-Hydroxydodecanedioic acid**	5.978_246.	POS	5.978	246.1468
**N-(4-((5-(3-(2-Aminoethyl)-1H-indol-5-yl)-1,2,4-**	5.896_411.	NEG	5.896	411.1354
**6-Thioinosine-5'-monophosphate**	6.462_364.	NEG	6.462	364.0253
Lachnoanaerobaculum_orale				
**VIBEGRON**	7.437_444.	POS	7.437	444.2153
**Cer(t18:1(6OH)/25:0(2OH))**	11.061_69	NEG	11.061	695.6458
**PA(21:0/20:5(5Z,8Z,11Z,14Z,17Z))**	8.371_764.	POS	8.371	764.537
**Betulin**	9.764_480.	POS	9.764	480.3357
**PA(22:6(4Z,7Z,10Z,13Z,16Z,19Z)/20:5(5Z,8Z,11Z,14Z**	5.698_766.	POS	5.698	766.4559
**PS(12:0/18:2(9Z,12Z))**	9.736_703.	POS	9.736	703.4401
**1-Methylindole**	3.286_131.	POS	3.286	131.0736
**2,4-Dodecadienoic acid, 11-methoxy-3,7,11-trimet**	8.784_310.	POS	8.784	310.2508
**3-Ethylbenzothiazoline-6-sulfonic acid**	3.638_245.	POS	3.638	245.0182
**Droxidopa**	2.399_213.	POS	2.399	213.064
**11(Z)-Eicosenoic acid**	10.836_31	NEG	10.836	310.2871
**(4,7,7-trimethyl-3-bicyclo[2.2.1]heptanyl) (E)-3-(4-**	4.542_368.	POS	4.542	368.1373
**9-Tetradecenoic acid**	8.583_226.	POS	8.583	226.1935
**Dipivefrin**	6.158_351.	POS	6.158	351.2043
**Glucose chloride**	4.216_214.	POS	4.216	214.0245
**Ioversol**	2.075_806.	POS	2.075	806.8645
**Irigenin**	7.771_360.	POS	7.771	360.0849
**12-Ketodeoxycholic acid**	7.846_406.	POS	7.846	406.2718
**Cefadroxil**	7.385_363.	POS	7.385	363.0873
**Adipic dihydrazide**	4.679_174.	POS	4.679	174.1119
**NCGC00384737-01!1-(3,4-dimethoxyphenyl)-2-(2-**	7.257_356.	POS	7.257	356.1621
**Triethylene glycol monohexyl ether**	5.547_234.	POS	5.547	234.1833
**7-[2-(furan-3-yl)ethyl]-5-hydroxy-7,8-dimethyl-5,**	4.754_352.	POS	4.754	352.1635
**Val-Cit**	0.745_274.	POS	0.745	274.1641
Other				
**Hycanthone**	8.255_356.	POS	8.255	356.1553
**Rolitetracycline**	7.106_527.	POS	7.106	527.2276
**3`-Aminoacetophenone**	5.652_135.	POS	5.652	135.0686
**Aplysiatoxin, 17-debromo-**	6.529_592.	POS	6.529	592.327
**Indolylmethylthiohydroximate; (E)-2-(1H-Indol-3-**	4.101_206.	POS	4.101	206.0516
**Saponarin**	6.52_594.1	POS	6.52	594.1589
**Integerrimine**	6.06_335.1	POS	6.06	335.173
**a-Hydroxyhippuric acid**	3.471_195.	POS	3.471	195.0534
**Lividic acid**	5.584_522.	POS	5.584	522.1857
Clostridium_polysaccharolyticum				
**Amisulpride**	7.016_369.	POS	7.016	369.1739
**Bolton-Hunter reagent**	5.07_263.0	POS	5.07	263.0794
**(1R,2R,4S,7S,8R,9R,10S,11R,12S,13S,14R,17R,18R,19**	4.637_490.	POS	4.637	490.221
**Fenuron**	4.123_164.	POS	4.123	164.0952
**6-Methylnicotinamide**	0.718_136.	POS	0.718	136.064
**LMPK12120008**	3.007_308.	POS	3.007	308.1406
**N,N'-Diacetylchitobiose 6'-phosphate;**	3.925_504.	NEG	3.925	504.138
**Aloeresin B**	5.677_394.	POS	5.677	394.1264
Other				
**Metapro**	9.817_424.	NEG	9.817	424.2591
**Nipradilol**	4.54_326.1	POS	4.54	326.1475
**PS(20:1(11Z)/0:0)**	7.778_551.	POS	7.778	551.3238
**Citrate**	0.638_192.	NEG	0.638	192.0272
**Xanthohumol(Random Configuration)**	7.257_354.	POS	7.257	354.1466
**8-[(2S,3R,4R,5S,6R)-3,4-dihydroxy-6-(hydroxymetl**	5.188_594.	POS	5.188	594.1589
**Prostaglandin E2-1-glyceryl ester**	7.76_443.2	POS	7.76	443.2967
**3,4-dimethyl-5-carboxybutyl-2-furanpentanoic aci**	6.186_296.	POS	6.186	296.1624
**LysoPI(18:0/0:0)**	10.037_60	NEG	10.037	600.3278

**Deacetoxy-7-Oxogedunin**	6.67_460.1	POS	6.67	460.1848
**Dihomomethionine; L-Dihomomethionine;**	3.066_177.	POS	3.066	177.0825
**Val-glu-pro-ile-pro-tyr**	6.069_716.	POS	6.069	716.3747
**Carboxycelecoxib**	7.187_411.	POS	7.187	411.05
Prevotella_nanceiensis				
Other				
**(2S,3R,4S,5R)-2-[(2R,3R,4S,5S,6R)-4,5-dihydroxy-6**	4.152_416.	POS	4.152	416.1699
**2-(4-Morpholinyl)benzothiazole**	6.615_220.	POS	6.615	220.0671
**Am-LPE(0:0/18:2(9Z,12Z))**	7.066_639.	POS	7.066	639.3371
**N-Acetyl-leu-leu-tyr-amide; NAc-LLY-amide;**	7.925_448.	NEG	7.925	448.2673
**5-[(4S,8aS)-4-acetyloxy-2,5,5,8a-tetramethyl-3-ox**	7.317_416.	POS	7.317	416.1947
**2-Methyl-4'-(methylthio)-2-morpholinopropiophe**	8.413_279.	POS	8.413	279.1293
Patulibacter_minatonensis				
**Artoindonesianin B**	6.163_468.	POS	6.163	468.1762
**Forchlorfenuron**	4.121_247.	POS	4.121	247.0516
**2-[(4-Benzamidophenyl)sulfonylamino]acetic acid**	7.241_334.	NEG	7.241	334.0623
**N-[2-(5-Hydroxy-1H-indol-3-YL)ethyl]-3-(4-hydr**	4.14_352.1	POS	4.14	352.1409
**Cloxacillin**	6.155_435.	POS	6.155	435.0659
Prevotella_shahii				
**Hesperetin 3',7-O-diglucuronide**	3.224_654.	NEG	3.224	654.1445
**Cervonoyl ethanolamide**	7.015_372.	POS	7.015	372.2664
**(-)TAN-67;**	7.481_344.	POS	7.481	344.1872
**3,6-dibenzyl-9-[(2,3-diamino-1,2-dihydroindol-3-**	7.502_529.	POS	7.502	529.28
**Eriocarpin;**	8.304_562.	POS	8.304	562.2434
**Phenol**	2.874_94.0	NEG	2.874	94.0418
**9H-Pyrido[3,4-b]indole-3-carboxylic acid**	6.169_212.	POS	6.169	212.0587
Turicibacter_sanguinis				
**arabinofuranosylguanine**	2.78_284.1	POS	2.78	284.1008
**Galactoarabinan**	7.842_500.	POS	7.842	500.2116
**ent-9-F1t-PhytoP**	7.181_328.	NEG	7.181	328.2252
**N,N-Dimethylsphingosine**	10.071_30	POS	10.071	309.3031
**Pyriminobac-methyl, (Z)-**	3.049_361.	POS	3.049	361.1274
**L-Serine-phosphoethanolamine; Serine phosphoet**	4.15_228.0	POS	4.15	228.0513
Other				
**(5S,8S)-8-benzoyl-2-[(Z,1S,2S)-1,2-dihydroxyhex-**	5.585_448.	POS	5.585	448.1844
**1-Phenyl-1-cyclohexene**	7.43_158.1	POS	7.43	158.1097
**Ethyl butylacetylaminopropionate**	5.07_215.1	POS	5.07	215.1525
**3-Genistein-8-C-glucoside**	5.628_432.	NEG	5.628	432.1058
**Puquitinib**	4.629_317.	POS	4.629	317.1374
**Tomentolide A**	7.344_402.	POS	7.344	402.1459
**1-hydroxyhexanoylglycine**	2.383_189.	POS	2.383	189.1004
**2-Aminoadenosine;**	4.797_282.	POS	4.797	282.1076
Bacillus_circulans				
**Myxothiazol A;**	4.061_487.	POS	4.061	487.1958
**(1S,8R,16S,17R)-11,12-dimethoxy-5,15-diazahexac**	7.44_369.2	POS	7.44	369.2053
**Cymarin**	8.034_548.	NEG	8.034	548.3013
**Cholest-5-EN-3-one**	10.015_38	POS	10.015	384.3391
Treponema_medium				
Actinomyces_dentalis				
**4'-(Imidazol-1-yl)acetophenone**	4.115_186.	POS	4.115	186.0774
**LysoPC(22:2(13Z,16Z)/0:0)**	10.004_57	POS	10.004	575.3955
**(2S,3S)-Nemonapride**	5.84_387.1	POS	5.84	387.1716
**Elsibucol**	9.446_602.	NEG	9.446	602.3457
**[(2S,3R,4S,5S,6R)-3,4,5-trihydroxy-6-(hydroxymeth**	4.56_346.1	NEG	4.56	346.1628
Lactobacillus_paracasei				
**Dibenzoylmethane**	8.311_224.	POS	8.311	224.0839
**PS(12:0/15:0)**	9.701_665.	POS	9.701	665.4274
**N-Acetylaspartic acid**	0.637_175.	NEG	0.637	175.0482

**N(6),O(2)-Dimethyladenosine**	3.27_295.1	POS	3.27	295.1283
**O-Palmitoylcarnitine**	8.434_404.	POS	8.434	404.2926
**Levulinic acid**	1.206_116.	NEG	1.206	116.0473
**Phytyl 2-O-methyl-dimethylarsinoylribose**	5.134_546.	POS	5.134	546.3256
**Sabiporide**	4.986_408.	POS	4.986	408.1534
**Devd-amc**	7.327_633.	NEG	7.327	633.229
Rhizobium_soli				
**Vanoxerine**	4.282_450.	POS	4.282	450.2478
**Sulindac sulfone**	4.052_372.	POS	4.052	372.082
**Penitrem E**	9.149_599.	POS	9.149	599.3245
**3'-Chloroacetophenone**	7.813_154.	POS	7.813	154.0185
**beraprost**	7.744_398.	POS	7.744	398.2093
**Moroxydine**	0.75_171.1	POS	0.75	171.1122
Providencia_sneebia				
**Cytochalasin B**	6.156_479.	POS	6.156	479.2662
**Ancistrocladine**	7.599_407.	POS	7.599	407.2096
**N-(2-(3-(Piperazin-1-ylmethyl)imidazo[2,1-b]thiaz**	6.637_469.	POS	6.637	469.1702
**VD 2716**	6.055_482.	POS	6.055	482.2855
**Zotiraciclib**	7.13_372.1	NEG	7.13	372.1967
**N2,N2-Dimethylguanosine**	3.063_311.	POS	3.063	311.1229
**alpha-Caryophyllene**	8.953_204.	POS	8.953	204.1881
**PS(18:1(9Z)/22:2(13Z,16Z))**	10.489_84	POS	10.489	841.5834
**Methylprednisolone aceponate**	7.96_472.2	POS	7.96	472.2466
**Elarofiban**	7.598_416.	POS	7.598	416.2414
**(3Z,6E)-2,2,6-trimethyl-12-oxabicyclo[8.2.1]trideca**	4.635_263.	POS	4.635	263.1522
**Voreloxin**	4.888_401.	NEG	4.888	401.1144
**Sakuranetin**	7.278_286.	POS	7.278	286.084
**PS(20:2(11Z,14Z)/0:0)**	7.773_549.	POS	7.773	549.3077
Other				
**Vasicinone**	3.747_202.	POS	3.747	202.0721
**Chenodeoxycholylphenylalanine**	8.355_539.	POS	8.355	539.3617
**LysoPC(16:1(9Z)/0:0)**	10.215_49	NEG	10.215	493.3166
**Benzylpenicilloyl**	7.709_350.	POS	7.709	350.13
**Methyl 4-(2-(2-hydroxy-2-(3-chlorophenyl)ethylai**	6.187_377.	POS	6.187	377.1406
**3-Butyl-4-methylphenol**	8.429_164.	POS	8.429	164.1204
**Equol**	6.199_242.	POS	6.199	242.0943
**Implitapide**	8.698_531.	POS	8.698	531.2875
**4,4'-Thiobis(6-tert-butyl-m-cresol)**	7.145_358.	POS	7.145	358.1963
**(R)-2-(4-(Tert-butoxycarbonyl)morpholin-3-yl)ace**	0.739_245.	POS	0.739	245.1263
**methyl (4R,8aS)-1-hydroxy-2-(hydroxymethyl)-5,5**	7.447_436.	POS	7.447	436.2462
**BURIMAMIDE**	2.352_212.	POS	2.352	212.1101
**2-[3,4-dihydroxy-2,2-dimethyl-8-(3-methylbut-2-**	5.811_478.	POS	5.811	478.1593
**Colocynthenin E**	9.359_564.	POS	9.359	564.3278
**Chloroneb;**	5.412_205.	POS	5.412	205.9896
**3-Hydroxyhippuric acid**	3.336_195.	NEG	3.336	195.0531
**(2S,3S,4S,5R)-3,4,5-Trihydroxy-6-(5-methyl-2-pro**	7.228_326.	POS	7.228	326.1366
**6-epi-19,20-epoxycytochalasin P**	12.978_54	POS	12.978	541.2663
**3-METHYL-2-OXOVALERATE**	2.861_130.	NEG	2.861	130.0631
**Nobiletin**	7.473_402.	POS	7.473	402.1316
**Carbaryl**	4.236_201.	POS	4.236	201.0793
**Noopept**	6.111_318.	POS	6.111	318.1577
**PE-Cer(d15:2(4E,6E)/22:0)**	10.935_70	POS	10.935	700.5518
**Decylubiquinol; 6-Decylubiquinol; 2-Decyl-5,6-dir**	8.562_324.	NEG	8.562	324.23
**(S)-3-Sulfolactate; (2S)-3-Sulfolactate;**	0.632_169.	NEG	0.632	169.9886
Methyloversatilis_universalis				
**L-arginine**	0.709_174.	POS	0.709	174.1119
**ascr#33**	9.406_442.	POS	9.406	442.3274
**Arctigenic acid**	5.074_390.	POS	5.074	390.1693

**PE(18:2(9Z,12Z)/17:2(9Z,12Z))**	10.612_72!	POS	10.612	725.4998
Dialister_invisus				
**Benzyl alcohol**	4.659_108.	POS	4.659	108.0577
**Noroxymorphone**	6.154_287.	POS	6.154	287.1162
Dysgonomonas_gadei				
**Dinoseb; 2,4-Dinitro-6-(1-methylpropyl)phenol;**	2.687_240.	POS	2.687	240.0749
**(4-Aminophenyl)phosphonic acid**	8.281_173.	POS	8.281	173.0244
Brevibacillus_choshinensis				
Mucilaginibacter_oryzae				
Other				
Other				
Acinetobacter_variabilis				
Hydrogenobacter_subterraneus				
Caulobacter_profundus				
Vibrio_owensii				
Acidovorax_delafieldii				
**PS(O-16:0/22:0)**	10.321_80!	NEG	10.321	805.6223
**Cirsimaritin**	1.655_336.	POS	1.655	336.0627
**Tyramine**	3.365_137.	POS	3.365	137.0843
**Ambroxol**	5.213_375.	POS	5.213	375.9787
**Hydrocortisone phosphate**	7.751_442.	POS	7.751	442.1742
**Endoxifen O-glucuronide**	8.997_551.	POS	8.997	551.2539
**24,24-difluoro-1alpha,25-dihydroxy-26,27-dimeth**	10.004_48(	POS	10.004	480.3434
**Cimicifugoside**	8.924_674.	POS	8.924	674.3639
**3-O-(alpha-L-rhamnopyranosyl-(1-2)-alpha-L-rha**	4.817_480.	POS	4.817	480.2552
**2,5-Furandicarboxylic acid**	1.167_156.	POS	1.167	156.0061
**Heptanoic acid**	4.644_128.	POS	4.644	128.0839
Pseudomonas_psychrotolerans				
**Sulfoacetic acid**	0.633_139.	NEG	0.633	139.9781
Bacillus_korlensis				
**Dopaxanthin quinone;**	6.313_388.	POS	6.313	388.0909
**Morphine-3-glucuronide**	1.567_461.	NEG	1.567	461.1684
**bhos#20**	5.279_362.	NEG	5.279	362.2305
**NCGC00179845-03!5-chloro-3-[(E)-3,4-dihydroxy-**	7.182_368.	POS	7.182	368.1407
**2-Hydroxyflavanone**	6.986_240.	POS	6.986	240.0787
**1-(2-methoxy-tetradecanyl)-sn-glycero-3-phosph**	8.209_441.	POS	8.209	441.2875
**Antibiotic M83**	7.911_426.	POS	7.911	426.1525
**Pentoxyresorufin**	7.921_283.	POS	7.921	283.1209
**hydroquinidine**	7.308_364.	POS	7.308	364.1557
**Rotenone**	6.582_411.	POS	6.582	411.1693
Macrococcus_lamae				
Clostridium_scindens				
**p-Chloroacetoacetanilide; 4'-Chloroacetoacetanilid**	7.811_211.	POS	7.811	211.0401
**6-Amino-4-(4-phenoxyphenylethylamino)quinazol**	7.947_356.	POS	7.947	356.1623
**Isoscutellarein 4'-methyl ether 8-(2''-sulfatoglucur**	4.279_556.	NEG	4.279	556.0532
**Vistusertib**	8.558_462.	POS	8.558	462.2386
**baicalin**	5.971_446.	NEG	5.971	446.085
**Dox-ga3**	6.125_882.	POS	6.125	882.2313
**(2S)-2-Cyclopentyl-2-[4-[(2,4-dimethylpyrido[2,3-**	6.745_531.	NEG	6.745	531.286
**Domperidone**	5.624_425.	POS	5.624	425.1628
**Desidustat**	5.341_332.	POS	5.341	332.1007
**NCGC00169702-02!(5R)-5-hydroxy-1-(4-hydroxy-**	7.735_276.	POS	7.735	276.1724
**Z-Arg-Arg-NHMec; Benzyloxycarbonylarginyl-argi**	7.987_621.	NEG	7.987	621.3024
**NCGC00381425-01!8-hydroxy-8-(3-octyloxiran-2-**	7.984_355.	POS	7.984	355.2719
**N-Acetyl-Asp-Glu-Val-Asp-CHO**	6.372_530.	POS	6.372	530.1875
**Methyl bisnorbiotinyl ketone**	4.125_214.	POS	4.125	214.0779
**2E,4E,6E,8E,10E,12E,14E,16E,18E,20E,22E-tetracosa**	6.71_330.1	POS	6.71	330.1976
**L-Glutamic acid**	0.649_147.	NEG	0.649	147.0533

**(2R,6R)-6-[(3R,10S,12S,13R,17R)-3-(2-carboxyacet**	7.268_610.	POS	7.268	610.3267
**Patuletin 3-(6''-(E)-feruloylglucoside)**	6.175_670.	POS	6.175	670.1545
**Pyrenocin A putative**	6.108_208.	POS	6.108	208.0738
**Phenylbutazone**	7.961_308.	POS	7.961	308.1521
**9,10-Phenanthrenequinone**	6.986_208.	POS	6.986	208.0526
**2-Hydroxyiminostilbene**	6.493_209.	POS	6.493	209.0842
**N-Acetylsphinganine**	9.386_343.	POS	9.386	343.3085
**BENPERIDOL**	7.372_381.	POS	7.372	381.1849
**Taurocholic acid 3-sulfate**	6.771_595.	NEG	6.771	595.2489
**Betaxolol**	7.281_307.	POS	7.281	307.2147
**(2E,4E)-N-[2-(4-hydroxyphenyl)ethyl]dodeca-2,4-**	6.182_315.	POS	6.182	315.2197
**Glutathionylaminopropylcadaverine;**	7.758_448.	POS	7.758	448.2454
**Tenax GC**	4.295_274.	POS	4.295	274.1351
**NCGC00380959-01![(2R,3S,4S,5R,6S)-3,4,5-trihydro**	9.198_513.	POS	9.198	513.2767
**17-Ethynyl-16-fluoroestradiol**	4.173_314.	POS	4.173	314.167
**(+/-)9,10-dihydroxy-12Z-octadecenoic acid**	8.397_314.	NEG	8.397	314.2456
Deinococcus_caeni				
Campylobacter_jejuni				
**8-Hydroxyloxapine**	4.069_343.	POS	4.069	343.1091
**5-Hydroxydiclofenac**	7.265_311.	POS	7.265	311.0114
**2,3,4,6-Tetra-O-acetyl-beta-D-glucopyranosyl isot**	5.234_389.	POS	5.234	389.0799
Sphingomonas_faeni				
**Farnesiferol A**	6.655_404.	POS	6.655	404.1946
**(2R,3S,4S,5R,6R)-2-[[(2R,3R,4R,5S)-3,4-dihydroxy-5**	5.114_448.	POS	5.114	448.2327
**10,16-Dihydroxyhexadecanoic acid; Hexadecanoic a**	7.84_288.2	POS	7.84	288.2299
**N-[(2S)-1-[[(2S)-1-Amino-1-oxopropan-2-yl]amin**	7.369_456.	POS	7.369	456.2365
**Halopemide**	7.833_416.	POS	7.833	416.1407
**3,5-dihydroxydecanoic acid**	4.932_204.	NEG	4.932	204.1362
**Rizatriptan**	5.211_269.	POS	5.211	269.1627
**2-Hydroxydodecanoic acid**	7.84_216.1	POS	7.84	216.1727
**8-Hydroxy-2-desoxyguanosine**	0.769_283.	POS	0.769	283.0915
**PILOCARPINE NITRATE**	6.466_288.	POS	6.466	288.1439
**(1S,3S)-(R)-Cyano(3-phenoxyphenyl)methyl 3-(2,2**	5.154_415.	NEG	5.154	415.0761
**Halofantrine**	5.68_499.1	POS	5.68	499.1633
Other				
Corynebacterium_marinum				
Balneola_vulgaris				
Deinococcus_citri				
Tessaracoccus_flavescens				
Luteimonas_lutimaris				
Erysipelothrix_tonsillarum				
Luteimonas_marina				
Other				
Other				
Anaerococcus_tetradius				
Psychrobacter_adeliensis				
Selenomonas_ruminantium				
Other				
Gulosibacter_chungangensis				
Tissierella_creatinophila				
Other				
Aequorivita_viscosa				
**2,4-Dimethoxyquinazoline**	4.521_190.	POS	4.521	190.0745
**(2S)-5-(Diaminomethylideneamino)-2-[[2-(2,2-dip**	7.916_412.	POS	7.916	412.2097
**Alloxydim;**	4.813_323.	NEG	4.813	323.1733
**Aclacinomycin Y**	6.487_809.	POS	6.487	809.3266
**Dabigatran**	7.352_471.	POS	7.352	471.2006
**Gelsemine**	4.288_322.	POS	4.288	322.1636

**Methanone, ((2R,5R)-5-(((5-fluoro-2-pyridinyl)oxy**	7.225_424.	POS	7.225	424.1732
**Ptaquiloside**	6.881_398.	POS	6.881	398.193
Peptostreptococcus_stomatis				
Other				
**24alpha-Methoxydihydrocucurbitacin D**	8.31_548.3	POS	8.31	548.337
**D-Psicose**	0.988_180.	NEG	0.988	180.0634
**Glycerophosphoserine**	0.645_259.	NEG	0.645	259.0455
**Pyochelin**	7.811_324.	POS	7.811	324.0587
**1-Hydroxypyrene;**	1.844_218.	POS	1.844	218.0728
**Bergaptol sulfate**	3.348_281.	POS	3.348	281.9829
**Apigenin 7-xyloside**	8.24_402.0	POS	8.24	402.0954
**Didemnin C**	6.59_1014.	POS	6.59	1014.59
Other				
**1-Palmitoyl-2-(5-keto-6-octenedioyl)-sn-glycero-**	7.745_663.	POS	7.745	663.3718
**3-Palmitoylcatechin**	8.807_528.	POS	8.807	528.3072
**3-O-alpha-L-rhamnopyranosyl-3-hydroxydecanoi**	6.471_334.	POS	6.471	334.1988
**THC**	10.087_31	NEG	10.087	314.228
**Butyl 3-O-beta-D-glucopyranosyl-butanoate**	4.098_322.	POS	4.098	322.1643
**(2R)-2-[(2R,5S)-5-[(2S)-2-hydroxybutyl]oxolan-2-**	6.658_216.	POS	6.658	216.1363
**N-((1,2,3,5,6,7-Hexahydro-s-indacen-4-yl)carbam**	6.273_404.	POS	6.273	404.1406
**Caprylic acid**	6.498_144.	NEG	6.498	144.1151
**(2S,3R)-3-(4-Hydroxyphenyl)-2-(4-{[(2S)-2-pyrroli**	6.156_463.	POS	6.156	463.1799
**Tubacin**	7.514_721.	POS	7.514	721.284
**Tandutinib**	7.977_562.	POS	7.977	562.3291
**PC(14:0/14:1(9Z))**	10.567_67	POS	10.567	675.4833
**13-(2-Methylcrotonoyl)oxylupanine; 13-(2-Methyl**	8.453_346.	POS	8.453	346.2259
**PE(13:0/14:1(9Z))**	9.867_619.	POS	9.867	619.4235
**14-hydroxy-12-tetradecenoic acid**	8.918_242.	POS	8.918	242.1883
**4-Guanidinobutyric acid**	0.879_145.	POS	0.879	145.0853
**3-Methyl-2(3H)-benzothiazolone**	4.939_165.	POS	4.939	165.0251
**1-(2-Chloro-4-(6,7-dimethoxyquinazolin-4-yloxy)**	6.62_416.1	POS	6.62	416.1251
**NCGC00384869-01!4,4,8,10,14-pentamethyl-17-(4**	7.803_468.	POS	7.803	468.3241
**Pipecolate**	0.614_129.	POS	0.614	129.0792
**7-Methylxanthine**	0.65_166.0	NEG	0.65	166.0478
**LMST04010325**	7.794_382.	POS	7.794	382.2145
**NCGC00380083-01!(E)-3-[2,4-dihydroxy-5-(2-met**	7.652_338.	POS	7.652	338.1518
**1(3H)-Isobenzofuranone, 3-butylidene-**	7.773_188.	POS	7.773	188.084
**desmethylenylnocardamine**	6.21_624.2	POS	6.21	624.2906
**Litchioside C**	2.967_406.	POS	2.967	406.2214
**Losulazine**	4.056_558.	POS	4.056	558.136
**9-(6-Methylsulfanylpurin-9-yl)-3H-purine-6-thior**	3.611_316.	NEG	3.611	316.03
**(E,E)-2,4-Decadienoic isobutylamide**	9.543_223.	POS	9.543	223.1938
**PC(6:2(3E,5E)/14:2(11E,13E))**	7.435_557.	POS	7.435	557.3107
**3-[(2S,3R,4S,5R,6R)-3,5-dihydroxy-6-(hydroxymetl**	5.628_594.	NEG	5.628	594.1585
**Prochlorperazine Sulfoxide**	4.385_389.	POS	4.385	389.1323
**2-(4-(((2-Amino-4-oxo-3,4,7,8-tetrahydropteridin**	6.204_443.	POS	6.204	443.155
**Cucurbitacin J**	8.245_532.	NEG	8.245	532.3062
**Glycohyocholic acid**	7.688_465.	POS	7.688	465.3092
**Sodium Tetradecyl Sulfate**	7.56_316.1	POS	7.56	316.1675
**Momelotinib**	6.82_414.1	POS	6.82	414.1792
**Cer(d14:1(4E)/20:1(11Z)(2OH))**	10.485_55	POS	10.485	551.4924
**Lappaol B;**	7.391_550.	POS	7.391	550.2219
Other				
**PA(P-16:0/12:0)**	9.153_576.	POS	9.153	576.4142
**Indole-3-acetic-acid-O-glucuronide**	3.538_351.	NEG	3.538	351.0955
**6-(Cyclopentylideneamino)-1H-pyrimidin-2-one**	3.082_177.	POS	3.082	177.0905
**m-Toluamide**	4.449_135.	POS	4.449	135.0686
**Pregnene**	10.155_28	NEG	10.155	286.2659

**TRIOXIFENE**	6.46_453.2	POS	6.46	453.2298
**Dihydrocaffeic acid 3-O-glucuronide**	3.618_358.	NEG	3.618	358.0901
**Acetyl-L-carnitine**	2.395_203.	POS	2.395	203.116
**Dimethylsphingosine**	8.153_355.	POS	8.153	355.2724
**17-Aminogeldanamycin**	7.294_545.	POS	7.294	545.2742
**Methylenediphosphonic acid**	5.578_175.	NEG	5.578	175.9635
**Lacosamide**	7.062_250.	POS	7.062	250.1318
**1-Cyclopropyl-3-(3-(5-(morpholinomethyl)-1H-be**	4.116_381.	POS	4.116	381.1899
**Taurodeoxycholic acid**	8.892_499.	POS	8.892	499.2973
**5,6-Dimethylbenzimidazole**	4.22_146.C	POS	4.22	146.0846
Methylobacterium_gossipiicola				
Acinetobacter_harbinensis				
**Disofenin**	6.144_350.	POS	6.144	350.184
**Taurine**	0.877_146.	POS	0.877	146.9968
Lactobacillus_kefiranofaciens				
**(3-(Tert-Butoxyimino-methyl)-4-chloro-phenyl)-tl**	2.693_328.	NEG	2.693	328.1019
**10-alpha-methoxy-9,10-dihydrolysergol**	3.678_286.	POS	3.678	286.1682
**Ac-Pro-Gly-Pro-OH**	2.546_311.	POS	2.546	311.1482
**N,N'-METHYLENEBISACRYLAMIDE**	0.708_154.	POS	0.708	154.0744
**N-Carbobenzoxyglycylproline; Z-Gly-Pro; Z-Gly-Pr**	6.404_306.	POS	6.404	306.1214
**2,4-Bis(methylthio)butyric acid**	0.639_180.	NEG	0.639	180.0271
**Pinoxaden-TP NOA 407854**	7.189_316.	POS	7.189	316.1785
**Azelaic acid**	2.944_188.	NEG	2.944	188.1049
**Hypoxanthine**	1.059_136.	NEG	1.059	136.0386
**Ranitidine**	3.749_314.	POS	3.749	314.1423
**11-Oxo-androsterone glucuronide**	7.414_480.	POS	7.414	480.2362
**(2S)-3-Hydroxy-1-[(2S)-pyrrolidine-2-carbonyl]py**	3.498_228.	POS	3.498	228.1112
**Tyrosylalanine**	3.543_252.	POS	3.543	252.111
**Dibenzothiophene-5-oxide;**	0.752_200.	NEG	0.752	200.0297
**Suberylglycine**	4.778_231.	POS	4.778	231.1108
**ascr#9**	5.1_248.12	POS	5.1	248.126
**Saterinone**	7.022_474.	NEG	7.022	474.2285
**2-Propyl-2,4-pentadienoic acid**	5_140.083!	POS	5	140.0839
**Pinokalant**	4.229_712.	POS	4.229	712.3395
Porphyromonas_catoniae				
**Florfenicol amine**	0.642_247.	NEG	0.642	247.069
**Dynasore**	7.442_322.	POS	7.442	322.0952
**Fucose**	0.663_146.	NEG	0.663	146.058
**4,5-Dihydro-drospirenone-3-sulfate**	6.883_434.	POS	6.883	434.2144
**N-(4-Cyanophenyl)-2-[4-(2,6-dioxo-1,3-dipropyl-**	6.167_486.	POS	6.167	486.2006
**4-Phenyl-1,2,4-triazoline-3,5-dione**	0.735_175.	NEG	0.735	175.0376
**Theonellasterol B**	7.412_422.	POS	7.412	422.3548
**Phytoporphyrin**	10.481_53	POS	10.481	534.2641
**Chol-11-Enic Acid**	5.836_372.	POS	5.836	372.2665
**Taurochenodeoxycholic acid**	7.241_499.	NEG	7.241	499.2956
**Senegenin**	7.261_536.	POS	7.261	536.2925
**Plumbagin**	4.653_188.	NEG	4.653	188.0474
**Nostocyclopeptide A2;**	8.367_790.	POS	8.367	790.405
Pantoea_stewartii				
Paracoccus_chinensis				
**1,3,2-Dioxathiolane 2,2-dioxide**	0.651_123.	NEG	0.651	123.9832
**4-Phenylpiperidine-4-carboxylic acid**	5.542_205.	POS	5.542	205.1107
**Cilazaprilat; (1S,9S)-9-((S)-1-Carboxy-3-phenylpro**	6.144_389.	POS	6.144	389.1949
**Selachyl alcohol**	10.317_34	NEG	10.317	342.3135
**PE(15:0/20:5(5Z,8Z,11Z,14Z,17Z))**	10.535_72	POS	10.535	723.4841
**Estromustine**	5.632_437.	POS	5.632	437.1505
**Hydroxy Tyrosol -Acetate**	5.134_225.	POS	5.134	225.1003
**voxtalisib**	6.402_270.	POS	6.402	270.1232

**4a-Hydroxytetrahydrobiopterin; 4a-Hydroxy-5,6,4,**	2.211_257.	POS	2.211	257.1125
**3,3-Diphenylpyrrolidine**	4.213_223.	POS	4.213	223.1371
**2-[2-[(1R,2R)-1-(5-Tert-butyl-2-methoxyphenyl)-:**	9.981_414.	NEG	9.981	414.2534
Ruminococcus_callidus				
**(8alpha,10alpha,13alpha,17beta)-17-[(4-Hydroxypl**	7.878_420.	POS	7.878	420.229
**Rotenonone**	5.078_406.	POS	5.078	406.1053
**Efaproxiral**	3.332_341.	POS	3.332	341.1639
**2-Methylbutyroylcarnitine**	3.618_245.	POS	3.618	245.1627
**Hydromorphone-3-glucoside**	6.169_447.	POS	6.169	447.19
**Aramite;**	1.051_334.	NEG	1.051	334.1011
**ZD-7155**	7.737_438.	POS	7.737	438.219
**Islatravir**	2.995_293.	POS	2.995	293.0936
**Hydroxymethyl cimetidine**	0.741_268.	POS	0.741	268.1106
**2,6-Diaminopurine 2',3'-dideoxyriboside**	7.453_250.	POS	7.453	250.1181
**Lidofenin**	3.981_294.	POS	3.981	294.1215
**taurolithocholic acid**	9.281_465.	POS	9.281	465.2917
**(2R,3S,4S,5R,6S)-2-(hydroxymethyl)-6-[4-hydroxy-**	7.437_340.	POS	7.437	340.1538
**Lactandrate**	6.174_562.	POS	6.174	562.2715
**Calcitriol**	9.865_416.	NEG	9.865	416.3293
**Apadenoson**	7.821_486.	POS	7.821	486.2228
**PC(14:1(9Z)/16:1(9Z))**	10.653_70	POS	10.653	701.4995
**Lithocholyltaurine**	9.307_483.	POS	9.307	483.3023
**7-Chloroemodin;**	3.295_304.	POS	3.295	304.0144
**Taurodeoxycholic acid 3-glucuronide**	7.174_675.	NEG	7.174	675.3313
**[(3S,8R,9S,10R,13S,14S)-16-Hydroxy-10,13-dimeth**	7.591_384.	POS	7.591	384.1604
**3,6,7-Trihydroxycholan-24-oic acid**	9.121_408.	POS	9.121	408.2877
Thermoanaerobacter_brockii				
**Methyl 16-hydroxyhexadecanoate**	9.941_286.	POS	9.941	286.2507
**Daidzin 4'-O-glucuronide**	6.322_592.	POS	6.322	592.1434
**alpha-carboxyethylhydroxychroman glutamine**	5.217_406.	POS	5.217	406.2104
**Metharbital**	3.426_198.	NEG	3.426	198.1004
**Validoxylamine A 7'-phosphate;**	6.218_415.	POS	6.218	415.1236
**2-[(3,17-Dihydroxy-13-methyl-8,9,11,12,14,15,16,1**	6.124_359.	POS	6.124	359.173
**2-deoxy-20-hydroxy-5alpha-ecdysone 3-acetate**	8.167_506.	NEG	8.167	506.3241
**Geranylbenzoquinone;**	9.78_244.1	POS	9.78	244.1466
Morganella_morganii				
**6-Bromo-5E,9Z,13Z,24Z-octacosatetraenoic acid**	6.175_480.	POS	6.175	480.2602
**3-[(E)-3,4-dihydroxy-3,5-dimethylhept-1-enyl]-7,8**	4.447_390.	POS	4.447	390.1426
**4-Androsten-3beta,17beta-diol disulfate**	5.491_450.	NEG	5.491	450.1381
**NCGC00381317-01!(E)-1-[2-(hydroxymethyl)pyrro**	7.794_263.	POS	7.794	263.1885
**Garcinol**	7.6_602.36	POS	7.6	602.3613
**Minosaminomycin;**	6.426_618.	POS	6.426	618.3325
**(2S,3S)-3-[[3,5-Bis(trifluoromethyl)phenyl]methoxy**	5.671_403.	POS	5.671	403.1379
**Retusin**	6.173_358.	POS	6.173	358.1049
**Tetradecanedioic acid**	7.176_258.	NEG	7.176	258.1831
**Isorhamnetin 3-galactoside**	4.9_478.11	NEG	4.9	478.111
Pseudochrobactrum_asaccharolyticum				
**Cocaine-alcohol**	5.627_319.	POS	5.627	319.1424
**Bis(2-propoxyethyl) 2,6-dimethyl-4-(3-nitropheny**	6.506_490.	POS	6.506	490.2333
**(E)-5-(1-(2-Hydroxyethyl)-3-(pyridin-4-yl)-1H-py**	6.153_334.	POS	6.153	334.1437
**17alpha-(N-acetyl-D-glucosaminyl)estradiol 3-glu**	6.989_651.	NEG	6.989	651.2918
**3,5-Dimethoxybenzoic acid**	5.466_182.	POS	5.466	182.0582
Helicobacter_macacae				
**2,3-Dimercapto-1-propanol tributyrate**	3.457_334.	POS	3.457	334.1278
**4,4-Difluoropregn-5-ene-3,20-dione;**	7.708_350.	POS	7.708	350.2067
**Spongipregnoloside D**	7.884_800.	NEG	7.884	800.422
**Sulfatroxazole**	7.768_267.	POS	7.768	267.0676
**Thymol-beta-D-glucoside**	7.18_312.1	POS	7.18	312.1572

**Wogonin**	6.787_284.	POS	6.787	284.0679
**Cefonicid**	3.779_542.	NEG	3.779	542.0357
**N-(2-Benzyl-3-((2-amino-4-methylpentyl)dithio)-**	6.118_474.	POS	6.118	474.2007
**Ketotrexate**	4.239_473.	POS	4.239	473.2022
**(S)-N-[3-(3,4-Methylenedioxyphenyl)-2-(mercapto**	5.95_354.C	POS	5.95	354.0893
**Coralyne Chloride**	5.548_437.	POS	5.548	437.0785
**Ceftizoxime**	3.794_383.	POS	3.794	383.035
**geniposide**	5.789_405.	POS	5.789	405.1617
**Tert-butyl (2-{7-[(2S)-3-(4-cyanophenoxy)-2-hydr**	7.319_446.	POS	7.319	446.2518
**geniposidic acid**	3.734_374.	POS	3.734	374.1227
**Dimethyl suberimidate**	3.754_200.	POS	3.754	200.1527
**3,4-Dimethoxycinnamic acid**	4.266_208.	POS	4.266	208.0714
**N-Acetylhistamine**	3.651_153.	POS	3.651	153.0903
Rheinheimera_arenilitoris				
**L-Olivosyl-oleandolide**	7.888_516.	NEG	7.888	516.2945
**Hexadecadienylcarnitine**	9.791_381.	POS	9.791	381.3243
**1-Vinylimidazole**	0.728_94.C	NEG	0.728	94.05308
**L-Cystine**	0.658_240.	NEG	0.658	240.0237
**N3-Cyclopropyl-7-[(4-propan-2-ylphenyl)methyl]**	6.815_371.	POS	6.815	371.2127
**Tetranor-12R-HETE**	9.117_248.	POS	9.117	248.1778
Duganella_phyllosphaerae				
Oceanobacillus_oncorhynchi				
Proteocatella_sphenisci				
Thermotoga_petrophila				
Loktanella_rosea				
Muricauda_zhangzhouensis				
Nitrososphaera_viennensis				
Other				
Other				
Other				
Roseburia_hominis				
Galbitalea_soli				
Other				
Thermodesulfobacterium_commune				
Other				
**Phaeantharine;**	8.313_632.	POS	8.313	632.2858
**(2S,3S,4S,5R,6S)-3,4,5-trihydroxy-6-[5-hydroxy-2-**	6.039_476.	POS	6.039	476.0959
**PE(12:0/12:0)**	7.589_579.	POS	7.589	579.3884
**Undecylenic acid**	9.508_184.	POS	9.508	184.1466
Ohtaekwangia_koreensis				
**Nimustine**	4.654_272.	POS	4.654	272.0797
**Trans-aconitate**	0.645_174.	NEG	0.645	174.0165
**Acutumine;**	4.419_397.	POS	4.419	397.1274
**(S)-Nicotine**	1.057_162.	POS	1.057	162.1159
**Incadronic acid**	2.738_287.	POS	2.738	287.0687
**PA(O-16:0/18:4(6Z,9Z,12Z,15Z))**	9.869_654.	POS	9.869	654.461
**4-Piperidinol, 1-hydroxy-2,2,6,6-tetramethyl-**	4.978_173.	POS	4.978	173.1419
**2-Benzothiazolesulfonic acid**	3.971_214.	NEG	3.971	214.971
**Galactaric acid**	0.867_232.	POS	0.867	232.0196
**Capecitabine**	5.389_359.	POS	5.389	359.1482
**N-(4-Coumaroyl)-L-homoserine lactone;**	5.148_247.	POS	5.148	247.0845
**Xanthine**	1.749_152.	POS	1.749	152.0337
**TRIETHYL PHOSPHATE**	5.642_182.	POS	5.642	182.0711
**Rutilantinone**	7.361_466.	POS	7.361	466.0671
**14-(acetyloxy)-1,6-dihydroxy-8-(hydroxymethyl)-4**	7.645_490.	POS	7.645	490.2572
Other				
**Clomipramine N-oxide**	7.527_330.	POS	7.527	330.1496
**N-D-Glucosylarylamine;**	4.946_255.	POS	4.946	255.1108

**Cyclamic acid**	3.464_179.	NEG	3.464	179.0618
**3',4'-Dihydrodiol**	5.728_286.	POS	5.728	286.0955
**Hydroxyvernolide;**	4.971_378.	POS	4.971	378.1314
**(2-((3,5-Di-t-butyl-4-hydroxyphenyl)thio)-1-meth**	10.068_36	POS	10.068	368.2014
**Arterolane**	10.196_39	NEG	10.196	392.2694
**SM(d18:1/18:1(11Z))**	11.413_72	POS	11.413	728.5834
**Mandelate**	3.175_152.	NEG	3.175	152.0475
**Pyrano(2,3-c)pyrazol-6(2H)-one, 3,4-dimethyl-2-(**	4.057_260.	POS	4.057	260.0619
**Toosendanin**	7.33_574.2	POS	7.33	574.2417
**(3R-(3alpha,4alpha(2R*,3R*),5beta,6beta))-5-Meth**	4.171_325.	POS	4.171	325.1887
**Irehine;**	10.002_34	POS	10.002	345.3029
**Metyrapone**	6.501_226.	POS	6.501	226.1107
**6,7-Dimethyl-8-(D-ribityl)lumazine;**	5.059_326.	POS	5.059	326.1242
**Chaulmoogric Acid**	9.382_280.	POS	9.382	280.2402
**Fusicoccin H;**	7.255_482.	POS	7.255	482.2886
**14S-Methyl-8E-hexadecenal**	8.779_252.	POS	8.779	252.2454
**2-O-Glutaroyl-1-O-palmitoyl-sn-glycero-3-phosp**	9.52_609.3	POS	9.52	609.3639
**N-Acetylserotonin**	5.742_218.	POS	5.742	218.1091
**[(1S,3aR,5R,5aR,8aR,9S,9aR)-1,5,8a-trimethyl-2,8-d**	5.792_390.	POS	5.792	390.1427
**Curcumin**	3.457_385.	POS	3.457	385.1533
**1,1,1-Trifluorohenicosa-6,9,12,15-tetraene**	7.84_342.2	POS	7.84	342.2519
**Clothiapine**	4.426_343.	POS	4.426	343.0904
**5-S-Cysteinyldopamine**	0.761_272.	POS	0.761	272.0832
**PE(22:6(4Z,7Z,10Z,13Z,16Z,19Z)/15:0)**	10.516_74	POS	10.516	749.4992
**Antcin B**	8.269_468.	POS	8.269	468.2857
**Metabolite B**	8.827_632.	NEG	8.827	632.3942
**Stelleracin E**	4.76_626.3	POS	4.76	626.3062
**3-[[(2R)-2-Acetamido-2-carboxyethyl]disulfanyl]-**	1.026_282.	POS	1.026	282.0344
**1-(2,5-Dimethoxy-4-iodophenyl)-1-methylethylan**	6.053_321.	POS	6.053	321.0227
**N,N-Diethyl-P-phenylenediamine**	3.132_164.	POS	3.132	164.1314
**Scyphostatin A**	7.795_511.	POS	7.795	511.3305
**Pent-2-enoic acid**	2.598_100.	POS	2.598	100.0526
**Valnemulin**	9.759_564.	POS	9.759	564.36
**Azelnidipine**	7.804_582.	POS	7.804	582.2484
**Budralazine**	5.502_240.	POS	5.502	240.1363
**candidone**	5.885_374.	POS	5.885	374.1478
**Bergaptol**	4.301_202.	POS	4.301	202.0269
**[(2S,3As,7aS)-Octahydro-1-[[(1R,2R)-2-phenylcycl**	4.141_384.	POS	4.141	384.1889
Other				
Other				
**Algesterone acetophenide**	7.464_448.	POS	7.464	448.2609
**3,4-Methylenedioxybenzoic acid**	10.446_16	NEG	10.446	166.0267
**LMST01031089**	10.118_43	POS	10.118	432.3605
**2-[[10-(2-Hydroxyethoxy)anthracen-9-yl]methylar**	3.994_355.	POS	3.994	355.1787
**Floionic acid**	6.963_346.	NEG	6.963	346.2357
**SANTALOL**	9.383_220.	POS	9.383	220.1828
**Meprobamate**	2.851_218.	POS	2.851	218.1268
**Acetamide, N-(4-((6-(4-(trifluoromethyl)phenyl)-4**	5.882_430.	POS	5.882	430.069
**Monoethylglycinexylidide**	3.785_206.	POS	3.785	206.1421
**(1S,6R,10S,13S,14R,15R)-1,6-dihydroxy-8-(hydroxy**	7.84_548.2	POS	7.84	548.2989
**Hexamethylpropylene amine**	6.43_141.1	POS	6.43	141.152
Streptococcus_oralis				
**1alpha,25-dihydroxy-22-oxavitamin D3 3-hemiglu**	8.354_532.	POS	8.354	532.3412
**Telocinobufagin**	10_402.24	POS	10	402.2404
**7-benzyl-11,14,20-trimethyl-16-(2-methylpropyl)-**	8.925_669.	POS	8.925	669.4094
**Triaminophenyl phosphate**	4.482_371.	NEG	4.482	371.104
**9-(1,3-Dioxolan-2-yl)purine-2,6-diamine**	5.63_222.0	POS	5.63	222.087
**Tuberonic acid glucoside**	4.24_388.1	POS	4.24	388.1748

**Coumeroic acid;**	6.534_358.	POS	6.534	358.0799
**taurohyodeoxycholic acid**	6.997_463.	POS	6.997	463.2761
Ruminococcus_torques				
**Isobutyl benzoate**	7.832_178.	POS	7.832	178.0996
**O-Demethyl apixaban sulfate**	6.179_525.	POS	6.179	525.1344
**2-hydroxy-4-(methylthio)butyric acid**	1.049_132.	POS	1.049	132.0247
Olsenella_uli				
**Mycalamide B;**	9.611_517.	POS	9.611	517.2882
**10-Deoxymethymycin; YC-17;**	8.29_453.3	NEG	8.29	453.3091
**C75 trans**	7.037_254.	POS	7.037	254.1517
**fluticasone 17beta-carboxylic acid**	4.161_396.	POS	4.161	396.175
Methylobacterium_jeotgali				
**L-Olivosyl-oleandolide;**	9.672_516.	POS	9.672	516.2959
**PS(O-16:0/18:3(9Z,12Z,15Z))**	10.207_74:	POS	10.207	743.5107
**neoandrographolide**	9.61_480.2	POS	9.61	480.2746
**Ilexolide A;**	8.874_586.	POS	8.874	586.3866
**1,4-Butanediol diglycidyl ether**	8.443_202.	POS	8.443	202.1209
**Impromidine**	6.301_321.	POS	6.301	321.1725
**Laprafylline**	9.667_500.	POS	9.667	500.2905
Bacteroides_faecichinchillae				
**2alpha-(3-Hydroxypropyl)-1alpha,25-dihydroxy-1!**	9.759_462.	POS	9.759	462.3713
**Ajugol**	4.245_348.	POS	4.245	348.1434
**Galaxolide**	8.442_258.	POS	8.442	258.1985
Lactobacillus_salivarius				
**1-(6-Hydroxy-2-azabicyclo[2.2.1]heptane-3-carbo**	3.223_235.	POS	3.223	235.1323
**Promethazine**	6.174_284.	POS	6.174	284.1347
**(E)-5-(4-methoxy-5-methyl-6-oxopyran-2-yl)-3-r**	6.365_266.	POS	6.365	266.1151
**Ribociclib**	5.194_434.	POS	5.194	434.2532
**Nafenopin**	7.221_310.	POS	7.221	310.1567
**Thioxanthene**	2.525_198.	POS	2.525	198.0508
**2-Chlorohexadecanoic Acid**	9.802_290.	NEG	9.802	290.2011
**Baohuoside VI**	7.857_822.	POS	7.857	822.296
**Lisinopril, epsilon-biotinamidocaproyl-**	8.28_744.3	POS	8.28	744.3854
**Isoxaben**	6.816_332.	NEG	6.816	332.1737
**PE(22:1(13Z)/0:0)**	9.777_535.	POS	9.777	535.3649
**L-Homoserine**	4.426_101.	POS	4.426	101.0479
**10-Nitrolinoleic acid**	7.74_325.2	POS	7.74	325.2252
**fluvoxamino acid**	6.16_318.1	POS	6.16	318.118
**dasc#1**	8.854_534.	POS	8.854	534.3025
**TG(18:2(9Z,12Z)/20:0/20:4(5Z,8Z,11Z,14Z))[iso6]**	0.68_934.7	POS	0.68	934.7981
**PI(22:2(13Z,16Z)/22:6(4Z,7Z,10Z,13Z,16Z,19Z))**	7.365_962.	NEG	7.365	962.5913
**Metomidate**	5.946_230.	NEG	5.946	230.1054
**Ajmaline**	4.168_326.	POS	4.168	326.1939
**Retinyl beta-glucuronide**	9.697_462.	POS	9.697	462.2638
**NCGC00170014-03!(E)-N-(4-acetamidobutyl)-3-(4**	5.385_306.	POS	5.385	306.1578
**NCGC00169972-02!3,6-dihydroxy-8-methoxy-3-m**	6.347_374.	POS	6.347	374.0749
**Benzonitrile; Phenyl cyanide; Cyanobenzene;**	5.672_103.	POS	5.672	103.0423
**3,5-dihydroxy-4-[3,4,5-trihydroxy-6-[[4-(2-hydro>**	4.061_498.	POS	4.061	498.1754
**kavain**	7.332_230.	POS	7.332	230.0944
**Luteolin 7-(6'''-acetylsophoroside)**	5.616_652.	POS	5.616	652.1612
**Atalaphylline**	6.397_417.	POS	6.397	417.1358
**beta-Mercaptoethanol**	0.768_78.C	POS	0.768	78.01407
**LMPK12010064**	4.22_887.2	POS	4.22	887.264
**Cadiamine;**	5.644_282.	POS	5.644	282.1942
Gemella_sanguinis				
**Indene**	7.971_116.	POS	7.971	116.0626
Intestinibacter_bartlettii				
**Olamufloxacin**	6.959_386.	NEG	6.959	386.1764

**Testosterone sulfate**	6.955_368.	NEG	6.955	368.1659
**Avermectin A1a aglycone;**	7.503_598.	POS	7.503	598.3491
**PG(14:0/20:4(5Z,8Z,11Z,14Z))**	7.176_742.	POS	7.176	742.4804
**19R-hydroxy-PGF1alpha**	7.851_372.	POS	7.851	372.2509
**Benzo[b]naphtho[2,1-d]thiophene; 1,2-Benzo-9-th**	0.694_234.	POS	0.694	234.0496
**Isolupalbigenin**	7.267_406.	POS	7.267	406.1756
**Jervine**	9.727_425.	POS	9.727	425.2931
**EICOSAPENTAENOATE**	7.277_324.	POS	7.277	324.2087
**(R)-Piperazine-2-carboxamide;**	1.007_129.	POS	1.007	129.0904
Acidovorax_defluvii				
Other				
Other				
Lactobacillus_intestinalis				
**Prulifloxacin**	6.773_461.	POS	6.773	461.1039
**Bergaptin**	7.734_338.	POS	7.734	338.1519
**Cholylsarcosine**	8.147_479.	POS	8.147	479.325
Sphingobacterium_siyangense				
Phascolarctobacterium_succinatutens				
**3-Hydroxybutyrylcarnitine**	4.731_247.	POS	4.731	247.1421
**Benfuracarb**	7.176_410.	POS	7.176	410.1879
**2,6-Dimethoxy-4-methylphenol**	4.719_168.	POS	4.719	168.0789
**Aminocarb**	4.7_208.12	POS	4.7	208.1213
**N-acetyltyramine**	3.708_179.	POS	3.708	179.0949
**Agar**	4.455_336.	POS	4.455	336.1421
**Radicinin**	4.519_236.	POS	4.519	236.0687
**Paritaprevir**	7.92_765.2	POS	7.92	765.2921
Pseudomonas_alcaligenes				
**quinone sulfate**	2.411_189.	NEG	2.411	189.9936
**Tryptophan 2-C-mannoside**	2.034_366.	NEG	2.034	366.1429
**8-Hydroxy-3,4,5-trimethyl-6-oxo-4,6-dihydro-3H-**	5.627_250.	POS	5.627	250.0841
**FLESTOLOL**	7.658_327.	POS	7.658	327.1582
**Minabeolide-8**	8.803_470.	POS	8.803	470.3036
**1H-Indole-2-carboxamide, 5-chloro-N-((1S,2R)-3-**	5.538_399.	NEG	5.538	399.1352
**6-Methyl-5,6-dihydro-4H-1,3-thiazin-2-amine**	2.953_130.	POS	2.953	130.0566
**3'-Amino-3'-deoxythimidine**	1.852_241.	POS	1.852	241.1062
**Glycine, N-[3-(4-hydroxy-3-methoxyphenyl)-1-ox**	3.361_251.	NEG	3.361	251.0792
**Z-Leu-Leu-Norvalinal**	7.499_461.	POS	7.499	461.2891
**Emivirine**	5.6_302.16	NEG	5.6	302.163
**[Ala9-Ala10]-moroidin**	3.708_564.	POS	3.708	564.2906
**1-Benzyl-4-(chloromethyl)benzene**	5.989_216.	POS	5.989	216.0706
**Macromomycin B**	3.864_249.	NEG	3.864	249.0635
Dialister_succinatiphilus				
**2,6-Diamino-9-(2-hydroxyethoxymethyl)purine**	5.08_224.1	POS	5.08	224.1025
**Hexapeptide analogue**	6.171_702.	POS	6.171	702.2902
**Cilnidipine**	7.537_492.	POS	7.537	492.1889
**L-Alanyl-gamma-D-glutamyl-meso-2,6-diaminohe**	2.687_390.	POS	2.687	390.1753
**DG(12:0/12:0/0:0)[iso2]**	9.994_456.	POS	9.994	456.3821
Other				
**4,6-Dimethyl-1-nitro-4-(trifluoromethyl)cyclohexe**	3.616_223.	POS	3.616	223.0825
Akkermansia_muciniphila				
**LMST01060017**	9.67_516.3	POS	9.67	516.3819
Mycoplasma_hyorhinis				
Other				
**N-Acetylserotonin glucuronide**	5.344_394.	POS	5.344	394.1374
**Dipyridamole**	7.924_504.	POS	7.924	504.3182
**N-Acetyl-b-D-galactosamine**	0.74_221.0	POS	0.74	221.0903
**3-Dibenzofuran-3-yl-2-(phosphonomethylamino)**	5.286_349.	NEG	5.286	349.0719
**Procarbazine**	3.619_221.	POS	3.619	221.1531

**Maltol**	0.67_144.0	NEG	0.67	144.0423
**OHHdiA-PE**	10.001_63!	NEG	10.001	635.3424
**N2-gamma-Glutamylglutamine**	0.653_275.	NEG	0.653	275.1115
**Validamycin B;**	6.307_513.	POS	6.307	513.2044
Endobacter_medicaginis				
**METAZACHLOR**	2.762_277.	POS	2.762	277.0984
**Homocysteine**	7.176_135.	POS	7.176	135.0357
**Calmagite**	5.236_358.	POS	5.236	358.0615
**Etretinate**	9.172_354.	POS	9.172	354.2195
**Glutamic acid glutamate**	0.583_290.	NEG	0.583	290.0747
**NNAL-N-glucuronide**	9.879_386.	NEG	9.879	386.1548
**Cer(d18:1/16:0)**	11.518_53	POS	11.518	537.5128
**N-Carbamoyl glucuronide lorcaserin**	5.064_409.	NEG	5.064	409.1671
**4-[7-acetyloxy-5-chloro-3-[(1E,3E)-3,5-dimethylh**	2.98_475.1	POS	2.98	475.1741
**DL-Acetylcarnitine**	5.164_225.	POS	5.164	225.0978
Anaerobiospirillum_succiniciproducens				
**Oxysophocarpine**	5.281_262.	NEG	5.281	262.1679
**Prostaglandin F2a 1-11-lactone**	8.847_318.	NEG	8.847	318.2194
**17-trimethylarsenylheptadecan-1-ol**	7.113_375.	POS	7.113	375.2615
**Fludrocortisone acetate**	2.981_422.	POS	2.981	422.2085
**karakin**	2.682_483.	POS	2.682	483.0983
**Benthiavalicarb isopropyl;**	3.831_381.	POS	3.831	381.1531
**Octinoxate**	7.172_290.	POS	7.172	290.1881
**Dihydroxytryptamine**	2.791_192.	POS	2.791	192.09
**Mandelonitrile benzoate**	3.727_237.	POS	3.727	237.0784
**Pancopride**	5.228_349.	POS	5.228	349.156
**4-Methylburimamide;**	3.518_226.	POS	3.518	226.1257
**Cyclohexene**	9.024_82.0	POS	9.024	82.0783
**Cyclohexanecarboxamide, N-(2-((ethylsulfonyl)ami**	4.193_379.	POS	4.193	379.1179
**Adrenic acid**	10.329_33	NEG	10.329	332.2716
Parvimonas_micra				
Enterococcus_cecorum				
Solobacterium_moorei				
**(+/-)9-HpODE**	8.213_312.	NEG	8.213	312.23
**Isolariciresinol sulfate**	5.317_440.	NEG	5.317	440.1144
**Exophilin A2**	9.521_618.	POS	9.521	618.3955
**bhos#22**	6.909_376.	POS	6.909	376.2461
Other				
**1-(2-Methylbenzo[d]oxazol-6-yl)-3-(1,5-naphthyr**	5.848_319.	POS	5.848	319.1054
**oroxindin**	5.782_460.	NEG	5.782	460.1006
**8-(5-Chloro-2-(4-methylpiperazin-1-yl)isonicotina**	4.02_559.1	POS	4.02	559.1899
**Dimerum acid**	5.768_484.	POS	5.768	484.254
**(1S,2S,6R,15S,16R)-5-(Cyclopropylmethyl)-16-(2-h**	9.671_467.	POS	9.671	467.3019
**Hydroxyethylflurazepam**	4.271_403.	POS	4.271	403.1477
**L-Arogenate; L-Arogenic acid; Pretyrosine;**	4.349_227.	POS	4.349	227.0796
**Rhizochalinin D**	9.723_646.	POS	9.723	646.5153
**8-Methylxanthen-9-one-4-acetic acid**	6.989_268.	POS	6.989	268.0736
**NCGC00180802-02! [IIN-based: Match]**	4.711_488.	POS	4.711	488.2051
**Soquinolol**	6.782_306.	POS	6.782	306.1941
Other				
**alpha-hydroxysalmeterol**	5.64_431.2	POS	5.64	431.2674
Mesorhizobium_sangaii				
Hyphomicrobium_facile				
Methylobacterium_rhodinum				
**Propranolol glucuronide**	6.528_435.	POS	6.528	435.1893
**1-Methyl-1,2-dihydropyridine-3-carboxamide**	3.984_138.	POS	3.984	138.0796
**Eseroline Fumarate**	6.022_334.	POS	6.022	334.1526
**Chloromethiuron;**	3.315_228.	POS	3.315	228.0486

**Cer(d18:1/18:1(9Z))**	11.596_56:	POS	11.596	563.5283
**PS(22:6(4Z,7Z,10Z,13Z,16Z,19Z)/19:0)**	10.15_849.	NEG	10.15	849.556
**Terbutaline**	6.046_225.	POS	6.046	225.1369
**[(1R,2R,3aR,5R,6E,10R,11S,13R,13aS)-1,2,3a,10,11-p**	7.36_650.2	POS	7.36	650.2589
**(1R,2R)-3-oxo-2-pentyl-cyclopentanebutanoic aci**	9.973_240.	POS	9.973	240.1727
**3'-DEOXY-3'-FLUOROTHYMIDINE**	5.1_244.08	POS	5.1	244.0848
**Galactosylpyridinoline**	6.369_590.	POS	6.369	590.2432
**PE(P-20:0/0:0)**	9.85_493.3	POS	9.85	493.3541
**(S)-3-(6-Methoxypyridin-3-yl)-3-(2-oxo-3-(3-(5,6**	6.429_439.	POS	6.429	439.2241
**(-)-Slaframine**	3.651_198.	POS	3.651	198.1371
**LysoPC(P-18:0/0:0)**	9.809_507.	POS	9.809	507.3695
**11beta,17beta-Dihydroxy-9alpha-fluoro-17alpha-**	9.263_338.	POS	9.263	338.2245
**2beta-acetoxy-14-deoxy-15beta,16beta-epoxymel**	6.119_512.	POS	6.119	512.2032
**Trocimine**	7.174_307.	POS	7.174	307.1783
**4'-Hydroxyfenoprofen glucuronide**	6.288_434.	POS	6.288	434.1213
**4-amino-5-(5-hydroxy-2',6-dimethyl-5'-prop-1-e**	5.613_419.	POS	5.613	419.1693
**Chlorpenthixol**	4.818_400.	POS	4.818	400.1361
Alistipes_onderdonkii				
Other				
**KOBUSONE**	9.172_222.	POS	9.172	222.1622
**Neolinustatin;**	5.833_423.	POS	5.833	423.1746
Alloprevotella_rava				
Other				
**3-carboxy-4-methyl-5-(4-hydroxypentyl)-2-furan**	5.465_284.	NEG	5.465	284.1258
**2E,4E,6E,8E,10E,12E,14E,16E,18E,20E-docosadecaen**	6.152_304.	POS	6.152	304.1827
**Hydroxydehydro Nifedipine Carboxylate**	5.258_346.	POS	5.258	346.0799
**(10R,13S,17S)-17-Hydroxy-13-methyl-10-[(4-meth**	7.606_414.	POS	7.606	414.2555
**PI(20:5(5Z,8Z,11Z,14Z,17Z)/0:0)**	7.901_618.	POS	7.901	618.2803
**NCGC00380601-01!N-[1-(4-methoxy-6-oxopyran-**	5.801_222.	POS	5.801	222.0894
**Bromacil;**	4.253_260.	NEG	4.253	260.017
**Dihydrocaffeic acid 3-sulfate**	4.24_262.0	NEG	4.24	262.0144
**LIPID_MAPS_STRUCTURE_DATABASE**	7.36_594.2	POS	7.36	594.2697
Other				
**4-Nitro-1-naphthylamine**	4.264_188.	POS	4.264	188.059
**N-Arachidonoyl dopamine**	10.176_43:	NEG	10.176	439.3062
**Tetranor-PGDM**	2.53_310.1	POS	2.53	310.1454
**PS(18:0/18:1(9Z))**	11.208_78:	NEG	11.208	789.5559
**2-Hydroxybenzothiazole**	5.687_151.	POS	5.687	151.0094
**Hydroxymethylbilane;**	7.429_854.	POS	7.429	854.285
Lactobacillus_mucosae				
**Metameconine**	2.827_194.	POS	2.827	194.0582
**Palmitoyl sphingomyelin**	11.357_70:	POS	11.357	702.5675
**12(13)Ep-9-KODE**	7.022_310.	POS	7.022	310.2141
**1,2-Benzisothiazole**	4.096_135.	NEG	4.096	135.0143
Other				
**(2R,3R,4S,5S,6R)-2-(3-(4-Cyclopropylbenzyl)-4-flu**	5.699_427.	POS	5.699	427.1778
Senegalimassilia_anaerobia				
**15-isoLG hydroxylactam-ethanolamine**	4.93_409.2	POS	4.93	409.2458
**Perfluorooctanesulfonic acid**	8.304_499.	NEG	8.304	499.9372
**3-Hydroxyhexanoylcarnitine**	2.552_275.	POS	2.552	275.1733
**Canthaxanthin**	10.485_56	POS	10.485	564.3968
**PG(P-16:0/0:0)**	9.849_468.	NEG	9.849	468.285
Geobacillus_thermocatenulatus				
Petrimonas_sulfuriphila				
Aquihabitans_daechungensis				
Nocardioides_exalbidus				
Ruminococcus_albus				
**(E)-3-(3,4-dimethoxyphenyl)-1-(2,4,6-trimethoxyp**	3.478_380.	POS	3.478	380.122

**Loperamide**	4.524_476.	POS	4.524	476.2238
**Ferulic acid 4-O-sulfate**	4.784_274.	NEG	4.784	274.0145
**N-alpha-acetyl-l-asparagine**	0.73_174.C	POS	0.73	174.0643
**Altersolanol A;**	4.007_336.	POS	4.007	336.0845
**3-ethylphenyl Sulfate**	5.104_202.	NEG	5.104	202.03
**PHOOA-PE**	7.993_607.	POS	7.993	607.3455
**4-Amino-3-phenylbutanoic acid**	8.632_179.	POS	8.632	179.0949
**Quiflapon**	8.165_586.	POS	8.165	586.2069
**p-Hydroxyubenimex**	4.649_324.	POS	4.649	324.1682
**N-Acetyl-L-glutamate**	2.465_171.	POS	2.465	171.0534
**PS(O-20:0/20:4(5Z,8Z,11Z,14Z))**	9.998_825.	NEG	9.998	825.5906
**(9Z,12E)-15,16-dihydroxyoctadeca-9,12-dienoic ac**	8.429_312.	POS	8.429	312.23
**Petroselinic acid**	9.52_282.2	POS	9.52	282.2558
**5-Hydroperoxy-1H-indole**	5.462_149.	POS	5.462	149.0479
**Loteprednol**	8.387_394.	POS	8.387	394.1546
**3-(4-hydroxy-5-oxo-3-phenyl-2H-furan-2-yl)pro**	3.269_265.	POS	3.269	265.0962
**5'-(3',4'-Dihydroxyphenyl)-gamma-valerolactone s**	4.89_288.C	NEG	4.89	288.0303
**PE-Cer(d14:1(4E)/22:0)**	11.102_68	POS	11.102	688.5519
**lup-20(29)-en-3**	10.068_61	POS	10.068	618.4305
**Alpha-Hydroxydeoxycholic Acid**	9.329_430.	POS	9.329	430.2486
**PE(22:0/0:0)**	10.024_53	POS	10.024	537.3801
**L-Tyrosine methyl ester**	5.651_195.	POS	5.651	195.0898
**Serotonin O-sulfate**	2.686_256.	POS	2.686	256.052
**Xamoterol;**	5.087_339.	POS	5.087	339.1793
**methyl (Z)-6-(3,7-dimethyl-2-oxo-1,3-benzoxazol**	8.179_450.	NEG	8.179	450.181
**Valsartan metabolite**	3.94_266.C	NEG	3.94	266.0822
Propionibacterium_acnes				
Other				
**4',5'-Dimethylfluorescein**	5.135_360.	POS	5.135	360.0998
**TEMANOGREL**	4.963_436.	NEG	4.963	436.2097
**Corticosterone**	10.143_34	POS	10.143	346.2184
**2-METHYLPROPANAL**	7.498_144.	POS	7.498	144.1152
Pandoraea_pnomenusa				
Sphingomonas_polyaromaticivorans				
Alistipes_indistinctus				
Leptotrichia_goodfellowii				
Capnocytophaga_granulosa				
Caulobacter_henricii				
Lactobacillus_backii				
Other				
Clostridium_aldenense				
Hymenobacter_metalli				
Oceanobacillus_indicireducens				
Other				
Anaerococcus_vaginalis				
Gemmobacter_nectariphilus				
Aquamicrobium_lusatiense				
Lactobacillus_pontis				
**LMFA08020322**	8.602_293.	POS	8.602	293.2355
**Cryptotanshinone**	3.874_296.	POS	3.874	296.1469
**ROTENONIC ACID, METHYL ETHER**	6.919_432.	POS	6.919	432.155
**LysoPE(P-16:0/0:0)**	7.77_437.2	POS	7.77	437.2906
Other				
**ascr#22**	7.196_360.	POS	7.196	360.2508
**Curacin A;**	6.115_373.	POS	6.115	373.2426
**N-Desmethylrosuvastatin**	7.359_467.	NEG	7.359	467.154
**LMST01060029**	9.797_534.	POS	9.797	534.3924
**1-Indolizidinone; 1-Ketooctahydroindolizine;**	4.535_139.	POS	4.535	139.0999

**Glycitein**	7.68_284.C	NEG	7.68	284.0684
**2-Hydroxyestriol**	6.232_304.	POS	6.232	304.1687
**Kyotorphin; L-Tyrosyl-L-arginine;**	7.155_337.	POS	7.155	337.1736
**Type IV cyanolipid 18:2 ester**	9.749_359.	POS	9.749	359.2824
**12alpha-Fluoro-11beta,17beta-dihydroxyandrost-**	6.155_322.	POS	6.155	322.1934
**11-Deoxocucurbitacin I;**	7.533_500.	POS	7.533	500.3149
Peptococcus_niger				
**Khellin**	5.625_260.	POS	5.625	260.0684
Prevotella_intermedia				
Clostridium_saccharogumia				
Other				
Other				
**3-Chloro-4-(dichloromethyl)-5-hydroxy-2(5H)-fur**	0.702_215.	POS	0.702	215.9156
**Creatinine hippurate**	3.717_290.	POS	3.717	290.1002
**5beta-Cholanic acid**	10.315_36	POS	10.315	360.3029
**Tripelargonin**	9.733_512.	NEG	9.733	512.4075
**Calyxin J**	5.012_686.	NEG	5.012	686.25
**Saikogenin A**	7.851_472.	POS	7.851	472.3554
Anoxybacillus_flavithermus				
**2-(4-Amino-1-isopropyl-1H-pyrazolo[3,4-d]pyrim**	4.469_308.	NEG	4.469	308.1372
**(S)-N-((R)-1-(Benzo[d][1,3]dioxol-5-yl)butyl)-3,3-**	6.601_564.	POS	6.601	564.2939
**Biocytin**	4.066_372.	POS	4.066	372.1892
**Desmethylazelastine**	4.284_367.	POS	4.284	367.1449
**LysoPC(22:0/0:0)**	10.44_579.	POS	10.44	579.4271
**Lobaric acid**	5.503_478.	POS	5.503	478.1593
**9-Fluoro-11beta-hydroxy-16beta-methylandrosta-**	3.759_332.	POS	3.759	332.1778
**Luteolin 3',4'-diglucuronide**	3.748_636.	NEG	3.748	636.1344
**Hydromorphone-3-sulphate**	6.027_365.	NEG	6.027	365.0934
**Arginine chloromethyl ketone**	7.194_206.	POS	7.194	206.0945
**Tetraneurin E; Ambrosanolide;**	4.593_324.	NEG	4.593	324.1568
**Sabeluzole**	4.717_415.	POS	4.717	415.1744
**Glucocochlearin; 1-Methylpropyl glucosinolate;**	2.443_375.	POS	2.443	375.0671
**5,7-dihydroxy-4-(4-methoxyphenyl)-3,4-dihydrocl**	5.83_303.1	POS	5.83	303.1106
**N-Phenyl-p-phenylenediamine**	4.209_184.	POS	4.209	184.1004
**Eltoprazine**	4.504_220.	POS	4.504	220.1215
Novosphingobium_sediminicola				
**Decarbamoylgonyautoxin 1;**	2.664_368.	POS	2.664	368.0738
**PHHdiA-PG**	6.511_640.	POS	6.511	640.3237
**NCGC00380345-01!(2S,8R)-8-hydroxy-2-[(1S)-1-h**	8.871_264.	POS	8.871	264.1727
**PG(O-18:0/18:1(9Z))**	8.208_762.	POS	8.208	762.5754
**Moveltipril**	6.706_398.	NEG	6.706	398.1862
Alkanindiges_illinoisensis				
Blautia_wexlerae				
**THURFYL NICOTINATE**	5.854_207.	POS	5.854	207.0898
**6H,8H-3,4-Dihydropyrimido(4,5-c)(1,2)oxazin-7-or**	2.77_153.C	POS	2.77	153.0539
**Netilmicin**	4.815_475.	POS	4.815	475.2997
Alcaligenes_faecalis				
**Debromohymenialdisine**	0.746_245.	POS	0.746	245.0876
**Nikethamide**	3.421_178.	POS	3.421	178.1109
**PS(16:0/20:3(8Z,11Z,14Z))**	10_785.52	POS	10	785.5206
**PHODiA-PS**	7.443_667.	POS	7.443	667.3331
**Diclofenac acyl glucuronide**	7.453_471.	POS	7.453	471.0488
Finegoldia_magna				
**Proline betaine**	0.772_143.	POS	0.772	143.0948
**5,7-dihydroxy-2-(4-hydroxy-3-methoxyphenyl)-6-**	5.781_392.	POS	5.781	392.122
**Sertindole**	4.169_440.	POS	4.169	440.18
**Blood group A antigen type 2**	6.549_732.	POS	6.549	732.2834
**Bardoxolone methyl**	9.742_505.	POS	9.742	505.3178

**Furo(3,4-b)pyridine-3-carboxylic acid, 1,4,5,7-tetra**	3.335_365.	POS	3.335	365.1079
**OON-PA**	7.86_590.3	POS	7.86	590.3605
**Supinidine;**	3.467_139.	NEG	3.467	139.0998
Bifidobacterium_thermophilum				
Devosia_insulae				
**Biotin thiamine**	4.747_276.	POS	4.747	276.061
**Zearalenone**	7.457_318.	POS	7.457	318.1465
**16a-Hydroxydehydroepiandrosterone**	8.722_321.	POS	8.722	321.2303
**NCGC00168939-02!8-hydroxy-3-(4-oxopentyl)-3,4**	0.675_286.	POS	0.675	286.063
Other				
**Aniflorine;**	5.213_351.	POS	5.213	351.1583
**Daclatasvir**	7.581_738.	POS	7.581	738.3841
**Histidine glutamate**	6.061_300.	POS	6.061	300.1079
**Tromethamine; 2-Amino-2-(hydroxymethyl)-1,3-p**	0.708_121.	POS	0.708	121.0742
**1-O-Hexadecyl-sn-glycerol; 1-O-Hexadecylglycerc**	10.029_31	POS	10.029	316.2976
**Nitrendipine M (dehydro)**	6.178_358.	POS	6.178	358.1164
**tri-glyceride**	0.637_264.	NEG	0.637	264.0478
**MJN110**	6.693_483.	POS	6.693	483.0762
**(2S,8R)-8-hydroxy-2-[(1S)-1-hydroxyheptyl]-2,3,4**	6.813_282.	POS	6.813	282.1831
**2-beta-d-Ribofuranosyl thiazole-4-carboxamide**	2.677_276.	POS	2.677	276.0416
**PE(0:0/14:1(9Z))**	6.083_423.	POS	6.083	423.2372
**deferrioxamine E**	8.714_600.	NEG	8.714	600.3512
Lactobacillus_delbrueckii				
**Tetracenomycin B1;**	7.411_350.	POS	7.411	350.0791
**Siguazodan**	3.371_284.	POS	3.371	284.1374
Other				
**(1R,4E,6S,7S,17R)-4-Ethylidene-7-hydroxy-7-(hydr**	2.667_351.	POS	2.667	351.1675
**PC(18:3(9Z,12Z,15Z)/19:0)**	12.083_79	POS	12.083	797.5928
**6-Hydroxykaempferol 3,6-diglucoside 7-glucuroni**	3.718_802.	POS	3.718	802.1805
**Destomysin; Destomycin A;**	2.513_527.	NEG	2.513	527.2338
**Indolophenanthridine**	3.466_274.	POS	3.466	274.1469
**(+/-)5(6)-EET**	9.06_320.2	POS	9.06	320.2351
**1-O-Hexadecyl-sn-glycero-3-phosphocholine**	9.761_481.	POS	9.761	481.3536
**Isophthalamide**	2.48_164.0	POS	2.48	164.0587
**Azafenidin;**	1.911_337.	POS	1.911	337.04
**gamma-Glutamyl-2-aminobutyric acid**	0.783_232.	POS	0.783	232.106
**Isofraxidin**	5.353_239.	POS	5.353	239.0795
**Benzamide, 5-((1,5-bis(4-methoxyphenyl)-1H-pyra**	2.985_507.	POS	2.985	507.1571
**6,10a-dihydroxy-4-(hydroxymethyl)-4,7,11b-trime**	6.175_388.	POS	6.175	388.1634
**Daumone**	5.496_276.	NEG	5.496	276.1571
**Arachidyl carnitine**	9.258_455.	POS	9.258	455.3979
**Petromyzonol sulfate**	8.111_474.	NEG	8.111	474.2649
**5-O-[(3S)-1-Benzylpyrrolidin-3-yl] 3-O-methyl (4R**	8.118_491.	POS	8.118	491.2061
**Epoxy (4,5Alpha)-4,5-Dihydrosantonin**	3.068_284.	POS	3.068	284.101
**3-Deazaneplanocin**	5.634_262.	POS	5.634	262.107
**N-[4-[[3-[(1-Ethyl-3-fluoropiperidin-4-yl)amino]-**	7.365_604.	POS	7.365	604.2174
**3-(6-(Dimethylamino)-4-methylpyridin-3-yl)-2,5-c**	10.144_38	POS	10.144	380.2693
**Xanthenone-4-acetic acid**	7.524_254.	POS	7.524	254.0581
**Inproquone**	4.514_306.	NEG	4.514	306.1579
**S-Lactoylglutathione**	6.52_379.1	NEG	6.52	379.109
**(2s,4s,5r,6r)-5-Acetamido-4-hydroxy-2-(4-methyl-**	4.277_467.	POS	4.277	467.1438
**2-(2-Hydroxyethoxy)-6-(2H-tetrazol-5-yl)xanthen**	4.492_324.	POS	4.492	324.0844
**[(1S)-5-hydroxy-1-[(2S,3R,4S,5S,6R)-3,4,5-trihydro**	4.625_450.	NEG	4.625	450.1525
**Metronidazole benzoate**	3.741_275.	POS	3.741	275.0907
Other				
Crocinitomix_catalasitica				
Rickettsia_massiliae				
Other				

Bradyrhizobium_oligotrophicum				
Phormidium_sp				
**tetranor-PGFM**	6.577_312.	POS	6.577	312.1574
**taurochenodeoxycholic acid**	6.795_463.	POS	6.795	463.2785
**8-C-Methylvelloquercetin 3,5,3'-trimethyl ether**	8.787_424.	POS	8.787	424.154
**3-(3,4-Dihydroxyphenyl)-N-[2-(3,4-dihydroxypher**	6.181_315.	NEG	6.181	315.1107
**(Z)-3-[4-methoxy-2-[(2S,3R,4S,5S,6R)-3,4,5-trihyd**	5.13_356.1	POS	5.13	356.1105
**CC-401**	7.389_388.	POS	7.389	388.203
**Povpc**	9.562_593.	POS	9.562	593.3699
**Defibrotide**	6.827_444.	POS	6.827	444.1209
**2,5-Dimethoxyphenethylamine**	6.081_181.	POS	6.081	181.1105
**Tert-butyl 4-(1H-pyrazolo[3,4-d]pyrimidin-4-yl)pi**	6.129_304.	POS	6.129	304.1644
**LORZAFONE**	0.811_393.	POS	0.811	393.066
**Candletoxin A;**	6.122_608.	POS	6.122	608.296
**3-(2-Hydroxyethyl)histidine**	1.228_199.	POS	1.228	199.096
**Macimorelin**	7.501_474.	POS	7.501	474.2366
**Salicyluric acid**	2.882_195.	POS	2.882	195.0534
**Beclabuvir**	3.658_659.	POS	3.658	659.3118
**Metofluthrin;**	3.89_360.1	POS	3.89	360.1352
**Levofloxacin**	3.894_361.	POS	3.894	361.1431
**VASICINONE**	2.933_202.	POS	2.933	202.0742
**2-Naphthylaminomethyl-gamma-aminobutyric aci**	5.102_258.	NEG	5.102	258.1366
**Nicotinate D-ribonucleotide; beta-Nicotinate D-rib**	0.605_336.	NEG	0.605	336.0498
**Rogletimide**	4.228_218.	POS	4.228	218.1058
**Gossypetin 7-methyl ether 8-acetate**	0.625_374.	NEG	0.625	374.063
**octyl 3-methyl-butanoate**	9.241_214.	POS	9.241	214.1935
**Patamostat**	5.06_412.1	NEG	5.06	412.1192
Dysgonomonas_oryzarvi				
**7-Methoxy-2,3-dihydropyrrolo[2,1-b]quinazolin-9**	6.858_216.	POS	6.858	216.09
**Pyrrolnitrin;**	3.029_255.	POS	3.029	255.9812
**6-(4-carboxyphenoxy)-3,4,5-trihydroxyoxane-2-ca**	3.336_314.	POS	3.336	314.0638
**Alacepril**	6.631_406.	NEG	6.631	406.1562
Rubellimicrobium_roseum				
Other				
**Spirodiclofen;**	0.715_410.	NEG	0.715	410.1035
**N-[1'-(Aminocarbonyl)-2',2'-dimethylpropyl]-1-(c**	7.871_370.	POS	7.871	370.2356
**Gluconic acid**	0.659_196.	NEG	0.659	196.0581
**1-[(4R,5R)-3,3-Difluoro-4-hydroxy-5-(hydroxymet**	7.307_264.	POS	7.307	264.0549
**Statine**	1.855_175.	POS	1.855	175.1211
**3,5-dimethoxy-4-(sulfooxy)benzoic acid**	4.099_278.	NEG	4.099	278.0094
**N-Acetyl-L-phenylalanine**	4.992_207.	POS	4.992	207.0897
**k-252b**	4.306_453.	POS	4.306	453.1315
**LMST01010295**	9.745_378.	POS	9.745	378.2922
**N-Acetylserotonin sulfate**	3.014_298.	POS	3.014	298.0624
**(2S,3S,4S)-3,4-dihydroxy-6-methoxy-3-methyl-7-**	6.569_314.	POS	6.569	314.184
**DIHYDROROTENONE**	7.431_418.	POS	7.431	418.1376
**2-Methylhippuric acid**	3.255_193.	NEG	3.255	193.074
**1-Methylphenanthrene;**	3.076_192.	POS	3.076	192.0936
**PE(17:0/12:0)**	10.518_64	POS	10.518	649.4686
Other				
**Tribufos**	4.099_314.	POS	4.099	314.0974
**3-[(2-Benzyl-3-sulfanylpropanoyl)amino]propanoi**	6.054_267.	POS	6.054	267.0929
**Myxochelin B;**	6.885_403.	POS	6.885	403.1744
**((2R,3S)-3-Amino-2-hydroxy-2-(1H-imidazol-4-yl**	2.974_241.	POS	2.974	241.1427
**Skimmianine**	6.745_259.	POS	6.745	259.0845
**Amygdalin**	6.117_474.	POS	6.117	474.1826
Barnesiella_intestinihominis				
Staphylococcus_petrasii				

**Enterodiol sulfate**	0.725_382.	POS	0.725	382.1087
**N(2)-(4-Hydroxy-1-(1-methyl-1H-indol-3-yl)carb**	2.934_588.	NEG	2.934	588.2757
**(2S)-2-(Carboxymethylamino)-5-(diaminomethylid**	0.739_246.	POS	0.739	246.133
**Hydramethylnon; Amdro;**	5.305_494.	NEG	5.305	494.1899
**Benzene oxide**	2.618_94.0	POS	2.618	94.04186
**(1R)-1-Amino-2-sulfanylethanesulfinic acid**	0.703_140.	NEG	0.703	140.9919
**Pradimicinone I;**	4.477_549.	NEG	4.477	549.1259
**PE(0:0/24:1(15Z))**	7.796_563.	POS	7.796	563.3934
**Prednisolone**	6.516_360.	POS	6.516	360.1935
Sphingobium_scionense				
Dyella_japonica				
**17-Iodoheptadecanoic acid**	5.203_396.	POS	5.203	396.1531
**2-Methyl-4(3H)-quinazolinone**	5.331_160.	POS	5.331	160.0638
**1,2,3,4-Tetrahydro-2-methyl-b-carboline**	5.092_186.	POS	5.092	186.1161
**Phenylethanolamine**	5.799_137.	POS	5.799	137.0843
**Homogentisic acid**	1.747_168.	NEG	1.747	168.0423
**Isoorientin**	5.083_448.	POS	5.083	448.101
**7-(3-((3-Acetylphenoxy)methyl)-1,5-dimethyl-1H-**	3.714_587.	POS	3.714	587.2447
**(2R)-2-[[(2R)-2-[[(2S)-2-(Azepane-1-carbonylamir**	8.694_656.	NEG	8.694	656.3354
Brachybacterium_rhamnosum				
**Dansylamide**	4.362_250.	POS	4.362	250.0778
**(5Z)-4-[2-[2-(3,4-dihydroxyphenyl)ethoxy]-2-oxo**	4.846_526.	NEG	4.846	526.1684
**2H-1-Benzopyran-7-ol, 3-(4-methoxyphenyl)-4-((**	5.352_471.	POS	5.352	471.2402
**N-(((1-(2-(Diethylamino)ethyl)amino)-9-oxo-9H-t**	6.11_433.1	POS	6.11	433.1489
**Cotarnine Chloride**	6.606_289.	POS	6.606	289.095
**1,4a-dimethyl-9-oxo-7-propan-2-yl-3,4,10,10a-te**	7.196_331.	POS	7.196	331.2136
**myo-Inositol**	0.719_202.	POS	0.719	202.0456
**3,4,7-Trimethyl-2E,6-octadien-1-ol**	5.752_168.	NEG	5.752	168.1515
**1-(Methylnitrosoamino)-4-(3-pyridinyl)-1,4-butan**	3.019_225.	POS	3.019	225.1115
Other				
**Myristic acid**	9.749_228.	NEG	9.749	228.2089
**Purine**	0.659_120.	NEG	0.659	120.0423
**isoleucyl-thiazolidine**	5.739_202.	POS	5.739	202.1143
**Epicatechin**	2.985_290.	POS	2.985	290.076
**PE(18:4(6Z,9Z,12Z,15Z)/18:0)**	10.684_73	POS	10.684	739.5155
**9-(2-Phosphonylmethoxyethyl)-2,6-diaminopurine**	5.048_288.	POS	5.048	288.0739
**2-Vinylpyridine**	7.463_105.	POS	7.463	105.058
**ACTIPHENOL**	3.867_292.	POS	3.867	292.1422
**9-Deazaguanine**	2.606_150.	POS	2.606	150.0544
**2-amino-3-[[(6E,9E)-5-hydroxy-8-methoxy-5,9,13**	7.443_446.	POS	7.443	446.243
**Bromadiolone**	5.417_526.	NEG	5.417	526.0778
**3-(4-Iodophenyl)-2-mercapto-(Z)-2-propenoic aci**	6.692_305.	NEG	6.692	305.9211
**PE(22:2(13Z,16Z)/19:0)**	12.75_813.	POS	12.75	813.6252
Pediococcus_acidilactici				
**PS(O-16:0/22:6(4Z,7Z,10Z,13Z,16Z,19Z))**	10.185_79	POS	10.185	793.5257
Methanomassiliicoccus_luminyensis				
**Leucocholic Acid**	7.837_521.	POS	7.837	521.372
**Indole-5-carboxylic acid**	5.617_161.	POS	5.617	161.0478
**17-Deoxyestradiol**	6.849_256.	POS	6.849	256.1827
**1-(2-Hydroxyethoxy)methyl-5-methyluracil**	0.729_200.	NEG	0.729	200.0797
**manidipine**	8.37_610.2	POS	8.37	610.279
**1alpha-hydroxy-2beta-(5-hydroxypentoxy)vitamin**	10.26_502.	POS	10.26	502.4031
**1-Acetyl-2-phenylhydrazine**	3.581_150.	NEG	3.581	150.0794
Other				
**2-methoxy-5-methyl-3-(2-methylbut-3-en-2-yl)c**	7.442_274.	POS	7.442	274.1004
**Palmitoleic acid**	9.756_254.	POS	9.756	254.2247
**DN-isobutylamide**	3.322_436.	POS	3.322	436.2435
**4-decyl-3-hydroxy-5-oxooxolane-2,3-dicarboxylic**	6.574_330.	POS	6.574	330.1679

**1'-Acetoxychavicol acetate;**	7.44_234.C	POS	7.44	234.0903
**3-Oxoglycyrrhetinate;**	10.012_46	POS	10.012	468.3218
**Carboxybupranolol, 4-chloro-3-[3-(1,1-dimethylet**	6.32_301.1	POS	6.32	301.1096
**L-5-Hydroxytryptophan**	1.903_220.	POS	1.903	220.085
Prosthecobacter_fluviatilis				
**(2R,3S,4S,9R,12R,13R,14S,15R,16R,17S)-3,4,12,13,1**	4.353_420.	POS	4.353	420.1758
**2-Amino-2-deoxy-D-gluconic acid**	5.647_217.	POS	5.647	217.0563
**(5R,6S)-3-(Aminomethyl)-6-[(1R)-1-hydroxyethyl]**	3.089_244.	POS	3.089	244.052
**5-(4-Carboxybutylperoxy)pentanoic acid**	4.243_234.	POS	4.243	234.1104
**Acaranoic acid**	8.006_330.	POS	8.006	330.2404
**Roccanin**	5.686_510.	POS	5.686	510.2234
**Valyl-prolyl-glycyl-valyl-glycine**	5.629_427.	POS	5.629	427.2419
**Fluprostenol serinol amide**	12.709_55	POS	12.709	553.216
**Terpendole G;**	9.265_451.	POS	9.265	451.2703
**Dicloxacillin**	7.163_469.	POS	7.163	469.0266
**Cfp-aaf-pab**	6.436_588.	POS	6.436	588.2589
**Involucrin**	7.971_490.	POS	7.971	490.2572
**1-Naphthylamine**	3.462_143.	NEG	3.462	143.0736
**hydroxytyrosyl acetate**	4.062_239.	NEG	4.062	239.0792
Other				
**PG(18:3(6Z,9Z,12Z)/0:0)**	9.387_506.	POS	9.387	506.2652
**Bifonazole**	2.632_310.	POS	2.632	310.1455
**Lamtidine;**	6.882_344.	POS	6.882	344.2311
**4-(Methylnitrosamino)-1-(3-pyridyl)-1-butanol gl**	4.316_385.	NEG	4.316	385.1487
**Vitamin E Nicotinate**	10.085_53	POS	10.085	535.4011
**Goniothalenol**	5.865_249.	POS	5.865	249.1002
**desmethyladinazolam**	4.728_323.	POS	4.728	323.0937
**Quercetin 3,3'-dimethyl ether 7-rutinoside**	6.536_638.	POS	6.536	638.183
**PHYSOSTIGMINE SALICYLATE**	6.488_413.	POS	6.488	413.1951
**Boc-D-phenylalanine**	4.737_265.	POS	4.737	265.1315
Other				
Streptococcus_dysgalactiae				
**Mefenamic acid**	4.187_241.	POS	4.187	241.1112
**Usnic acid**	6.545_344.	POS	6.545	344.0892
**CE-108;**	7.42_739.3	POS	7.42	739.3792
**Bosentan**	0.741_551.	POS	0.741	551.1843
**Fluzinamide**	5.004_274.	POS	5.004	274.093
**4-Hydroxyclonidine**	4.541_245.	POS	4.541	245.0114
**N-(2-Hydroxyethyl)-morpholine N-oxide**	10.026_14	POS	10.026	147.0897
Corynebacterium_lubricantis				
Proteus_vulgaris				
**4,4'-Biphenyldithiol; 4,4'-Dimercaptobiphenyl; 4,4'**	0.627_218.	NEG	0.627	218.0232
**Azaserine**	0.66_190.C	POS	0.66	190.0697
**D-threo-Isocitric acid**	0.974_192.	POS	0.974	192.0273
**(3S,3'R,3'aS,6'S,6aS,6bS,7'aR,9R,11bR)-3-hydroxy-3**	6.568_447.	POS	6.568	447.2737
Prevotella_stercorea				
**2-Hydroxynaphthaldehydebenzoylhydrazone**	0.745_290.	POS	0.745	290.1048
**trans-3-Hexenoic acid**	4.54_114.C	POS	4.54	114.0683
**Bluensomycin;**	3.716_585.	POS	3.716	585.2471
**2,2-Dimethyl-3-[4-(acetyloxy)phenyl]-4-ethyl-2H-**	5.123_380.	POS	5.123	380.161
**Avocadyne 4-acetate**	8.238_326.	POS	8.238	326.2452
**1,2-Dilauroyl-sn-glycero-3-phosphocholine**	10.261_62	POS	10.261	621.4368
**(3S,4R,5R)-1,4,5,6-Tetrahydroxy-3-[(2S,3R,4S,5S,6R**	4.214_342.	POS	4.214	342.1151
**NCGC00384702-01!(2Z,6E,10E)-12-hydroxy-6,10-d**	7.645_342.	POS	7.645	342.2191
Bradyrhizobium_lupini				
**methyl (1S,4aR,7aR)-4a-hydroxy-7-(hydroxymethy**	4.343_404.	POS	4.343	404.1307
**1-[3-methoxy-4-[(2S,3R,4S,5S,6R)-3,4,5-trihydroxy**	5.757_496.	POS	5.757	496.1532
**Laurdan**	8.026_353.	POS	8.026	353.2718

**L-Anserine**	6.24_240.1	NEG	6.24	240.1262
**1-[3,4-Dihydroxy-5-(hydroxymethyl)oxolan-2-yl]-**	2.972_243.	POS	2.972	243.0745
**Quillaic acid 3-[xylosyl-(1->3)-[galactosyl-(1->2)]-**	6.957_956.	NEG	6.957	956.4641
**Isopropyl 9Z-octadecenoate**	10.108_32	POS	10.108	324.3028
**Malic acid**	0.93_156.0	POS	0.93	156.0037
Lactococcus_raffinolactis				
Jeotgalibaca_dankookensis				
**Indole-3-acrylic acid**	3.461_187.	NEG	3.461	187.0634
**NCGC00380142-01!(E)-5-(1,3-benzodioxol-5-yl)-N**	6.623_275.	POS	6.623	275.1522
**2-Hexynyl-5'-N-ethylcarboxamidoadenosine**	3.323_388.	POS	3.323	388.1846
**2-Amino-1,5-anhydro-2-deoxyglucitol;**	1.891_243.	POS	1.891	243.0507
**5-(Hydroxyphenyl)-gamma-valerolactone-O-sulph**	5.069_272.	POS	5.069	272.0347
**N-Formyl-Nle-Leu-Phe**	5.715_419.	POS	5.715	419.2421
**Angiotensin (5-7); Angiotensin-(5-7);**	3.771_365.	POS	3.771	365.2065
**Malonylglycine**	0.841_218.	POS	0.841	218.054
**Tricetin 7-glucoside-3'-[6''-(3-hydroxy-3-methylg**	4.167_770.	NEG	4.167	770.1941
**Propanamide, N-((1S,2S)-2-(3-cyanophenyl)-3-(4-**	4.059_489.	POS	4.059	489.2437
**PS(22:2(13Z,16Z)/22:6(4Z,7Z,10Z,13Z,16Z,19Z))**	9.834_887.	NEG	9.834	887.5694
**2-(Fluoromethyl)-3-(3,4-dihydroxyphenyl)alanine**	4.969_229.	POS	4.969	229.074
**Gomisin A**	6.587_416.	POS	6.587	416.1829
**2-Fluoro-5-hydroxy-L-phenylalanine**	2.937_199.	POS	2.937	199.0646
**Pipes**	0.73_302.0	NEG	0.73	302.0613
**Formetanate**	3.193_221.	POS	3.193	221.1166
**Alanycarb;**	4.677_399.	NEG	4.677	399.1279
**oscr#20**	6.61_346.2	POS	6.61	346.2352
**Emorfazone**	2.844_239.	POS	2.844	239.1271
**4-Phenylbutyric acid**	8.867_164.	POS	8.867	164.084
**Nafamostat**	6.822_347.	POS	6.822	347.1397
**18-bromo-9E,15E,17E-octadecatrien-5,7-diynoic a**	3.916_348.	NEG	3.916	348.0741
**2-p-Tolyl-5,6,7,8-tetrahydrobenzo[d]imidazo[2,1-**	0.733_268.	NEG	0.733	268.1033
**pre-putrebactin**	5.119_390.	POS	5.119	390.2132
**Sweroside**	3.883_358.	NEG	3.883	358.1264
**NCGC00385674-01!6,8-dihydroxy-3-(10-hydroxyu**	6.588_314.	POS	6.588	314.1878
**4-Hydroxy-(3',4'-dihydroxyphenyl)-valeric acid**	2.984_226.	NEG	2.984	226.0841
**2-Amino-4-oxo-4-alpha-hydroxy-6-(erythro-1',2'**	6.046_321.	POS	6.046	321.0922
**dhas#18**	6.316_364.	POS	6.316	364.2108
**Arhalofenate**	1.17_415.0	NEG	1.17	415.08
**1-(4-Aminophenyl)-7,8-dimethoxy-3-methyl-5H-**	6.407_325.	POS	6.407	325.1426
**Zolmitriptan N-Oxide**	4.106_303.	POS	4.106	303.1582
**3,7,11-Trimethyldodeca-1,6,10-triene**	9.245_206.	POS	9.245	206.2036
**Obtusifolin**	6.038_284.	POS	6.038	284.0686
**4-Oxoproline**	0.649_129.	NEG	0.649	129.0427
**3-Hydroxyanthranilic acid**	2.58_153.0	POS	2.58	153.0428
**N(4)-Oleylcytosine arabinoside**	9.153_493.	POS	9.153	493.3496
**4-Aminoanhydrochlortetracycline; 4-Aminoanhydr**	5.248_432.	NEG	5.248	432.0726
**DL-Stachydrine**	5.324_143.	POS	5.324	143.0948
Other				
**Corynanthine**	4.226_376.	POS	4.226	376.1748
**sialosyl-Tn saccharide**	6.502_512.	POS	6.502	512.1868
**Melatonin**	7.41_232.1	POS	7.41	232.1211
**2-methoxy-3,5-dimethyl-6-[(4Z)-4-[(E)-2-methyl-**	7.414_435.	POS	7.414	435.1086
**Idelalisib**	6.596_415.	POS	6.596	415.1553
**(8R,9R,10S,13S,14S)-13-Methyl-1,4,5,6,7,8,9,10,11,**	9.541_244.	POS	9.541	244.2191
Other				
**(4aR)-5-hydroxy-6-methoxy-1,1-dimethyl-7-prop**	6.608_368.	POS	6.608	368.1946
**Argopsin**	5.567_396.	NEG	5.567	396.0151
**NCGC00380570-01![(2R,3R,4R,5R,6S)-4,5-dihydrox**	6.146_568.	POS	6.146	568.1953
Streptococcus_infantarius				

**MONENSIN SODIUM (monensin A is shown)**	6.619_690.	POS	6.619	690.4308
**[7-(3,4-dihydroxyphenyl)-1-(4-hydroxyphenyl)hep**	4.783_380.	POS	4.783	380.1583
**Kaempferol 3-glucoside-7-xyloside**	6.534_580.	POS	6.534	580.1427
**Sulfometuron-methyl**	3.546_364.	NEG	3.546	364.083
**Laserpitin;**	9.006_450.	NEG	9.006	450.262
**2-Chlorobiphenyl**	0.724_188.	POS	0.724	188.0399
**Felbamate**	4.629_238.	POS	4.629	238.0954
**Saxagliptin**	4.246_315.	POS	4.246	315.1946
Catonella_morbi				
**L-Kynurenine**	2.401_208.	POS	2.401	208.085
**PI(21:0/22:6(4Z,7Z,10Z,13Z,16Z,19Z))**	7.676_952.	POS	7.676	952.6002
Prevotella_scopos				
Sulfitobacter_noctilucicola				
**Tiadinil;**	2.875_267.	POS	2.875	267.0235
**Dimethyltriazenoimidazole carboxamide**	3.81_182.C	POS	3.81	182.0921
**Atrimustine**	4.247_719.	POS	4.247	719.2767
**1alpha,25-dihydroxy-11beta-phenylvitamin D3 / 1**	9.811_492.	POS	9.811	492.3584
**Glutamine pyruvate**	0.702_232.	NEG	0.702	232.0702
**N-Benzylformamide**	5.15_135.C	POS	5.15	135.0686
**Pyrraline**	3.506_254.	POS	3.506	254.1268
**Taltrimide**	7.443_296.	POS	7.443	296.0825
**Proglumide**	6.773_334.	POS	6.773	334.189
**Hexazinone**	4.201_252.	POS	4.201	252.1574
Succiniclasticum_ruminis				
Other				
**CGA62826 (2-[2,6-dimethylphenyl)-methoxyacetyl**	3.784_265.	POS	3.784	265.1313
**2-Ethylbut-2-enedioic acid**	4.392_144.	NEG	4.392	144.0424
**Melevodopa**	5.568_211.	POS	5.568	211.0847
**Lincomycin**	7.386_406.	POS	7.386	406.2135
Other				
Bacillus_amyloliquefaciens				
Other				
Alistipes_shahii				
Negativicoccus_succinicivorans				
Other				
Porphyromonas_bennonis				
**3,5-Dihydroxy-3',4'-dimethoxy-6,7-methylenediox**	6.69_534.1	POS	6.69	534.1014
**ascr#6.1**	4.382_248.	NEG	4.382	248.1621
**3-Amino-3-methyl-N-[(3R)-2-oxo-1-[[4-[2-(2H-t**	7.257_509.	POS	7.257	509.2534
**Transfectam**	0.688_806.	POS	0.688	806.8093
**Aminopyrrolnitrin;**	0.704_226.	NEG	0.704	226.0065
**Fusidic Acid**	7.894_554.	POS	7.894	554.3028
**Dipteryxin**	5.12_314.C	POS	5.12	314.0786
**Fenozan 50F**	4.725_370.	POS	4.725	370.1378
**N(beta)-Epoxysuccinamoyl-DAP; N(beta)-Epoxysu**	1.905_217.	POS	1.905	217.0701
**NCGC00180705-02!(E)-5-hydroxy-1,7-diphenylhe**	9.822_280.	POS	9.822	280.1463
Fibrobacter_intestinalis				
**6-((2-((4-(2,4-Dichlorophenyl)-5-(4-methyl-1H-in**	6.614_464.	POS	6.614	464.1054
**17-Hydroxypregnenolone sulfate**	9.722_412.	POS	9.722	412.1912
**2-(Furan-2-yl)-7-(3-(4-methoxyphenyl)propyl)-7H**	5.754_389.	POS	5.754	389.1587
**Pyridoxamine**	0.663_168.	POS	0.663	168.0877
**10-Deoxymethymycin**	7.756_453.	POS	7.756	453.3099
**Minocycline**	6.817_457.	POS	6.817	457.1851
**3-Alizarinsulfonic acid**	4.738_319.	POS	4.738	319.9984
**5-Thia-1-azabicyclo[4.2.0]oct-2-ene-2-carboxylic**	3.916_424.	NEG	3.916	424.0691
**methyl (4aR)-5,6-dihydroxy-1,1-dimethyl-7-propa**	8.93_363.2	POS	8.93	363.2409
**N-(3-Oxooctanoyl)-L-homoserine lactone**	5.244_259.	POS	5.244	259.142
**1,2-Dioctanoylglycerol**	8.458_344.	POS	8.458	344.2562

**Niranthin**	6.172_432.	POS	6.172	432.2148
**PS(P-18:0/22:6(4Z,7Z,10Z,13Z,16Z,19Z))**	10.414_81!	POS	10.414	819.5419
Weissella_confusa				
**Nonate**	9.365_188.	POS	9.365	188.1051
**Jasmonic acid**	8.442_210.	POS	8.442	210.1259
**Galunisertib**	6.633_369.	POS	6.633	369.1607
**Dihydroisomorphine-3-glucuronide**	6.138_463.	POS	6.138	463.1844
**Naptalam; N-1-Naphthylphthalamic acid;**	5.035_291.	POS	5.035	291.0908
**Actein**	9.941_676.	NEG	9.941	676.3847
**2-Methyl-3-[4-(pyridin-3-ylmethyl)phenyl]prop-2**	3.002_253.	POS	3.002	253.1104
**PS(O-20:0/0:0)**	8.917_539.	NEG	8.917	539.3603
Bacillus_coagulans				
**Naringenin**	6.206_272.	POS	6.206	272.0684
**Dmean**	3.022_291.	POS	3.022	291.1385
**3-(2-Methylperoxyethyl)-1H-indole**	4.291_191.	POS	4.291	191.0944
Dorea_longicatena				
**Glucoscilliroside**	8.328_766.	POS	8.328	766.3413
**3-[3-(Sulfooxy)phenyl]propanoic acid**	3.627_246.	NEG	3.627	246.0195
**15-oxo-18Z-tetracosenoic acid**	10.587_38(	NEG	10.587	380.3293
**Amisulpride N-oxide**	5.17_385.1	POS	5.17	385.1638
**Roxadustat**	7.397_352.	POS	7.397	352.1076
**NCGC00385245-01!(2E,4E,14E)-15-(1,3-benzodiox**	6.041_433.	POS	6.041	433.2577
**Lidocaine**	4.097_234.	POS	4.097	234.1731
**[(2R,3R,4S,5S,6R)-3-acetyloxy-2-(acetyloxymethyl)**	7.565_332.	NEG	7.565	332.1838
**5-hydroxy-7-(hydroxymethyl)-2-methyl-2-(5-oxo**	6.055_309.	POS	6.055	309.1214
**N-[[2-(4-Amino-1,2,5-oxadiazol-3-yl)-1-ethylimid**	3.298_342.	POS	3.298	342.19
**Bruceantin;**	8.656_548.	POS	8.656	548.2284
**Azaribine**	3.947_371.	POS	3.947	371.0969
**(2R,4S)-1-(Tert-butoxycarbonyl)-4-phenylpyrrolidi**	3.768_291.	POS	3.768	291.1468
**a Methyl farnesoate**	9.813_250.	POS	9.813	250.1934
Other				
Other				
**Suplatast;**	2.824_328.	POS	2.824	328.1568
**Tocainide**	5.808_192.	NEG	5.808	192.1263
**N-acetyl Leukotriene E4**	7.574_481.	POS	7.574	481.2503
**Indole-3-acetic acid**	5.241_175.	POS	5.241	175.0636
**CH-223191**	4.23_333.1	POS	4.23	333.1574
**1alpha,25,28-trihydroxyvitamin D2 / 1alpha,25,28-**	9.813_444.	POS	9.813	444.3236
**(E)-3-[6-[(2S,3R,4S,5S,6R)-3,4,5-trihydroxy-6-(hyd**	3.583_392.	POS	3.583	392.072
**Isoleucylproline**	4.14_228.1	POS	4.14	228.1477
**3-Amino-1-(3-(cyclohexylmethoxy)phenyl)propan**	6.972_263.	POS	6.972	263.1884
**Sarcoehrendin I**	7.758_452.	POS	7.758	452.2778
**4E-Decenyl acetate**	9.384_198.	POS	9.384	198.1622
**Lomerizine**	5.117_468.	POS	5.117	468.2225
Anaerostipes_hadrus				
**lenticin**	3.396_246.	POS	3.396	246.1368
**(E)-4-[(1R,2S,3S,4R,8aS)-2,3,4-trihydroxy-2,5,5,8a-**	7.912_310.	POS	7.912	310.2143
**Etodolac acyl glucuronide**	5.499_463.	NEG	5.499	463.1843
**Minaxolone**	9.732_405.	POS	9.732	405.3244
**Columbamine;**	4.273_338.	POS	4.273	338.138
**Spermine**	0.652_202.	POS	0.652	202.216
**8-Methoxykynurenate**	4.515_219.	POS	4.515	219.0534
**NCGC00384865-01!5-hydroxy-7-[4-hydroxy-2-me**	2.71_436.1	POS	2.71	436.1882
**2,3-Diaminosalicylic acid**	3.031_168.	POS	3.031	168.0537
**{4-[2-Acetylamino-2-(1-biphenyl-4-ylmethyl-2-o**	4.414_637.	POS	4.414	637.2212
**Semiplenamide G**	9.099_411.	NEG	9.099	411.335
**Phenylalanocholic Acid**	8.549_555.	NEG	8.549	555.3566
**2,5-Dioxopyrrolidin-1-yl 4-((2,5-dioxo-2,5-dihydr**	5.648_334.	POS	5.648	334.1164

**LPIM2(19:0/0:0)**	7.931_938.	NEG	7.931	938.4473
**5-[(Diaminomethylidene)amino]-2-(sulfanylmethyl**	4.235_221.	POS	4.235	221.0666
**(2S,3R,5R,10R,13R,14S,17S)-2,3,14-trihydroxy-10,1:**	8.737_494.	POS	8.737	494.325
**9-(Tetrahydrofuran-2-yl)-9h-purin-6-amine**	2.977_205.	POS	2.977	205.0955
**Paracetamol sulfate**	3.946_231.	POS	3.946	231.0204
**2-Nitro-alpha-((1-propenyloxy)methyl)imidazole-:**	1.701_227.	POS	1.701	227.0907
**4-Acetylaminohippuric acid**	3.287_236.	POS	3.287	236.0796
**Anhydrochlortetracycline; Anhydro-7-chlorotetracy**	3.938_460.	NEG	3.938	460.104
**Batrachotoxinin A 20-alpha-benzoate**	6.132_521.	POS	6.132	521.2757
**4-Methylthiobenzamide-S-oxide**	5.155_167.	POS	5.155	167.0407
**4-[[4-Amino-6-(2,4,6-trimethylanilino)-1,3,5-triazi**	6.039_345.	POS	6.039	345.1687
**3-Hydroxybenzo(a)pyrene**	6.006_268.	POS	6.006	268.0882
**TIAZOFURIN**	5.596_260.	POS	5.596	260.0467
**Cinnamyl alcohol**	3.772_134.	NEG	3.772	134.0732
**2',3-Dimethyl-4-aminobiphenyl**	4.805_197.	POS	4.805	197.1207
**Deferitrin**	4.156_253.	POS	4.156	253.041
**N-(2-Hydroxy-3,3,3-trifluoropropyl)-2-(2-nitro-1-**	0.637_282.	NEG	0.637	282.0584
**NCGC00170025-02!N-(1-hydroxy-3-phenylpropan**	4.861_255.	POS	4.861	255.1268
**Lifibrol**	6.519_342.	POS	6.519	342.1829
**10-Deacetylbaccatin III**	7.602_544.	POS	7.602	544.2289
**5-Hydroxybenzofuran-2(3H)-one**	2.507_150.	NEG	2.507	150.0318
**8-hydroxy-5,8a-dimethyl-3-methylidene-3a,4,4a,8**	4.046_280.	POS	4.046	280.1315
**(3beta,5alpha,6beta,24R)-Stigmastane-3,5,6-triol**	11.135_44	POS	11.135	448.3896
**digitalin**	7.567_712.	POS	7.567	712.3672
**Petromyzonol**	9.796_394.	POS	9.796	394.3083
**2-[4-hydroxy-2-[(2S,3R,4S,5S,6R)-3,4,5-trihydroxy**	4.639_382.	POS	4.639	382.1745
**(2S,3S)-1-[[2-Methoxy-5-(trifluoromethoxy)pheny**	3.662_380.	NEG	3.662	380.1698
**Decadienyl-l-carnitine**	8.676_297.	POS	8.676	297.2304
**8-Br-cGMP; 8-Bromocyclic GMP;**	0.703_422.	NEG	0.703	422.9589
**Morpholine, 4-(3-(4-(1,1-dimethylpropyl)phenyl)-:**	9.666_317.	POS	9.666	317.2718
Streptomyces_hygroscopicus				
**LMST01031157**	9.792_460.	POS	9.792	460.3169
**Felodipine**	4.396_383.	NEG	4.396	383.0677
**Thiamine pyrophosphate**	4.948_425.	POS	4.948	425.0464
**4-methoxy-9-(2-methylbut-3-en-2-yl)furo[3,2-g]**	6.061_301.	POS	6.061	301.1317
Psychrobacter_fulvigenes				
**2,3-Dimethylfuran**	4.166_96.0	POS	4.166	96.05769
**Cer(t18:0/36:0(36OH))**	0.746_851.	NEG	0.746	851.8298
**Uric acid**	1.01_168.0	POS	1.01	168.0285
**1-Ethoxy-N-(3-morpholin-4-yloxadiazol-3-ium-5**	0.61_242.1	POS	0.61	242.1016
**Glycohyodeoxycholic acid**	7.928_447.	POS	7.928	447.2982
**Reserpine**	9.861_608.	POS	9.861	608.2632
**(2R,4R)-4-Aminopyrrolidine-2,4-dicarboxylic acid**	1.18_174.0	POS	1.18	174.0643
**Pyrido(3,4-d)pyridazine-1,4-dione, 8-amino-5-chl**	2.954_288.	POS	2.954	288.0422
**1-Amino-3-hydroxymethyl-5-methyl-adamantane**	9.541_195.	POS	9.541	195.1626
**Lorlatinib**	5.596_406.	POS	5.596	406.1561
**Quebrachitol**	0.931_232.	POS	0.931	232.035
**BENZOYLARGININE-2-NAPHTHYLAMIDE**	7.472_403.	POS	7.472	403.2028
**Terazosin**	6.413_387.	POS	6.413	387.1893
**Decoquinate**	6.278_417.	POS	6.278	417.2513
**Amobarbital**	4.437_226.	POS	4.437	226.132
**Diloxanide**	6.121_327.	POS	6.121	327.0062
**N-Succinimidyl 6-Maleimidohexanoate**	2.892_308.	POS	2.892	308.1007
**R-138727**	4.664_349.	POS	4.664	349.116
**Lumichrome**	3.733_242.	POS	3.733	242.0806
**N-Acetyl-DL-serine**	0.731_169.	POS	0.731	169.0352
**Prosafrinine**	7.475_283.	POS	7.475	283.2509
**Thiethylperazine**	6.022_399.	POS	6.022	399.1794

**cis-Linoleic acid**	9.957_266.	POS	9.957	266.2246
**Glisoxepide**	3.709_449.	POS	3.709	449.1721
**Sulfacarbamide**	4.442_215.	POS	4.442	215.0367
**Carbenicillin**	5.631_378.	POS	5.631	378.0878
Atopobium_rimae				
**N-lactoyl-Tryptophan**	3.738_276.	NEG	3.738	276.1108
**Ambamustine**	7.871_628.	POS	7.871	628.2055
**Gomisin D**	7.762_530.	POS	7.762	530.2175
**Calicheamicinone;**	0.734_423.	NEG	0.734	423.0281
**Melinamide**	9.948_383.	POS	9.948	383.3188
Other				
Coprococcus_catus				
**Physodic acid**	5.796_492.	POS	5.796	492.175
Kandleria_vitulina				
**norerythromycin**	6.595_705.	POS	6.595	705.4298
Hydrogenophilus_thermoluteolus				
**Kresoxim-methyl acid**	6.195_299.	POS	6.195	299.1155
**N,N-Bis(2-chloroethyl)-N',N''-diphenylphosphoric**	4.434_371.	POS	4.434	371.0724
**NCGC00380810-01! [IIN-based on: CCMSLIB00000**	6.868_541.	POS	6.868	541.207
Eubacterium_eligens				
**Ruberythric acid**	6.611_534.	POS	6.611	534.1379
**Temiverine**	9.51_385.2	NEG	9.51	385.2616
**Caracurine V;**	7.798_584.	POS	7.798	584.3135
**Tetraisopropylpyrophosphoric acid amide**	3.292_345.	POS	3.292	345.1476
**gamma-Glutamylfelinylglycine**	6.114_393.	POS	6.114	393.1587
Other				
**(1R,3S,5E)-5-[(2E)-2-[(1R,3As,7aR)-1-[(2R,3E,5E)-7**	8.935_440.	POS	8.935	440.3274
**Bis(7-methyloctyl) Cyclohexane-1,2-dicarboxylate**	9.757_424.	POS	9.757	424.3552
**2,4-dihydroxy-5-methoxy-1'',6'',8''-trimethyldispir**	2.651_418.	POS	2.651	418.1972
**5,7-dihydroxy-2-(4-methoxyphenyl)-6-(3-methylb**	3.246_371.	POS	3.246	371.174
**1-(4,5-Dimethylthiazol-2-yl)-3,5-diphenylformaza**	5.65_335.1	POS	5.65	335.1197
**3,4-Methylenedioxy-N-ethylamphetamine**	2.922_207.	POS	2.922	207.1262
**1,4-Dihydroxy-3,3-dimethylbutan-2-one**	3.38_132.0	POS	3.38	132.0789
**Inosine**	1.71_268.0	NEG	1.71	268.0805
Thermus_thermophilus				
**Casimiroedine;**	6.374_417.	POS	6.374	417.1903
**Diacetylpyxinol**	8.07_582.3	POS	8.07	582.3886
**Tazobactam**	3.238_300.	POS	3.238	300.0526
**6-methoxy-7-(3-methylbut-2-enoxy)chromen-2-c**	5.361_277.	POS	5.361	277.1314
**PYRAZOPHOS**	4.194_373.	POS	4.194	373.0877
**5,7,9,11,13-tetradecapentaenoic acid**	9.701_218.	POS	9.701	218.1309
**Besifloxacin**	5.848_393.	POS	5.848	393.1252
**(2R,3R)-3-[[(2S)-1-[4-(Diaminomethylideneamino)**	4.156_357.	POS	4.156	357.1999
**1-(N(Sup alpha)-Dansyl-L-arginyl)-4-ethylpiperidi**	6.052_502.	POS	6.052	502.272
**(2S)-2-(4-Chloroanilino)propanoic acid**	3.717_199.	POS	3.717	199.0405
**methyl 15-hydroperoxy-9Z,12Z,16E-octadecatrien**	6.741_324.	POS	6.741	324.23
**Sapacitabine**	9.167_490.	NEG	9.167	490.3141
**1b,3a,12a-Trihydroxy-5b-cholanoic acid**	7.905_408.	NEG	7.905	408.2875
**Oxprenolol**	6.512_265.	POS	6.512	265.1681
**4-Hydroxyphenylpyruvic acid**	3.429_180.	NEG	3.429	180.0424
Other				
Clostridium_bifermentans				
**12-beta-acetoxy-3beta,15alpha,16alpha,24alpha-t**	8.338_634.	POS	8.338	634.3365
**PC(17:0/22:6(4Z,7Z,10Z,13Z,16Z,19Z))**	11.643_81	POS	11.643	819.5785
Rothia_aeria				
**Cligosiban**	2.294_419.	POS	2.294	419.1174
**8-Hydroxydesmethylclomipramine**	6.404_316.	POS	6.404	316.1328
**7-Ethyl-10-[4-(1-piperidino)-1-piperidino]carbony**	8.299_586.	POS	8.299	586.2798

**PA(14:0/12:0)**	8.317_564.	POS	8.317	564.3771
**Peiminine**	10.084_42!	POS	10.084	429.3244
**methoxymorphinan**	5.877_257.	POS	5.877	257.1778
**15-Oxo-beta-bilirubin; beta-Staphylobilin;**	8.428_598.	POS	8.428	598.2434
**CD 1790**	6.624_473.	POS	6.624	473.2169
**2-Amino-6-methylpyrimidine-4-thiol**	4.473_141.	POS	4.473	141.0364
**LysoPC(14:1(9Z)/0:0)**	6.365_465.	POS	6.365	465.2843
Bacteroides_coprocola				
**PS(18:0/22:4(7Z,10Z,13Z,16Z))**	10.275_83!	POS	10.275	839.5682
**3-Pyroglutamylthiazolidine-4-carboxylic acid**	4.245_244.	NEG	4.245	244.0516
**Zalypsis**	6.227_709.	POS	6.227	709.258
**D-(+)-Maltose**	0.754_364.	POS	0.754	364.0981
**Carglumic acid**	0.655_190.	NEG	0.655	190.0591
**Dihydrojasmonic acid**	8.854_212.	POS	8.854	212.1414
**N-OCTANOYL-L-HOMOSERINE LACTONE**	4.244_227.	POS	4.244	227.152
**[4-acetyloxy-2,5-dihydroxy-6-(hydroxymethyl)oxa**	3.808_406.	POS	3.808	406.0647
**Wuhanic acid**	10.17_396.	NEG	10.17	396.3239
**1,2-Epoxy-3-(p-nitrophenoxy)propane**	3.637_195.	POS	3.637	195.0536
**NCGC00180266-02!3,8-dihydroxy-6-methyl-9-oxc**	4.64_268.C	POS	4.64	268.0372
**Y-27632 Dihydrochloride**	6.388_247.	POS	6.388	247.1685
**13,16,19-Docosatrienoic acid**	10.23_334.	POS	10.23	334.2871
**((4-(3-(4-Fluoro-alpha-hydroxybenzyl)-4-hydroxy**	6.4_425.12	POS	6.4	425.1255
**Cyclonite**	4.269_222.	POS	4.269	222.0352
**Diffractaic Acid**	4.533_374.	POS	4.533	374.1366
**N-Acetyl-leucyl-leucine;**	4.216_286.	POS	4.216	286.189
**5-oxo-7-octenoic acid**	5.305_156.	POS	5.305	156.0788
**10S,11R-epoxy-punaglandin 3**	12.937_51:	POS	12.937	512.1793
**methyl 2-[(2,3-dihydroxybenzoyl)amino]-3-hydrox**	4.633_255.	POS	4.633	255.0743
**Tropane**	0.75_125.1	POS	0.75	125.1206
**N-benzyl-2-(N-(furan-2-ylmethyl)acetamido)-2-(4**	6.838_383.	POS	6.838	383.1943
**PG(13:0/22:1(11Z))**	7.35_762.5	NEG	7.35	762.5438
**(E)-2-Hydroxy-N-desmethyldoxepin**	3.511_281.	POS	3.511	281.1422
**4-(4-(4-Fluorophenyl)-1-(piperidin-4-yl)-1H-imid**	6.537_353.	POS	6.537	353.1659
**Diphenyl phthalate**	3.026_318.	POS	3.026	318.0889
**1,5-Dihydro-7-(1-piperidinyl)-imidazo(2,1-b)quina**	6.5_270.14	POS	6.5	270.1467
**Anthramycin methyl ether**	3.277_329.	POS	3.277	329.1374
**3-(4-hydroxyphenyl)-7-methoxy-5-[(3R,4S,5S,6R)-**	3.781_484.	POS	3.781	484.0762
**7-(Trifluoromethoxy)indolin-2-one**	5.029_217.	POS	5.029	217.0353
**1,2,3,4-Tetrachloronaphthalene**	0.686_263.	NEG	0.686	263.9073
**[(2R,3S,4S,5R,6R)-6-[(2S,3S,4S,5R)-3,4-dihydroxy-2**	3.725_570.	POS	3.725	570.1569
**Benoxathian**	5.816_361.	POS	5.816	361.1352
**Metoprine**	0.813_268.	POS	0.813	268.0296
**26,26,26,27,27,27-hexafluoro-1alpha,25-dihydroxy**	6.343_518.	POS	6.343	518.2272
**Carduusyne B**	5.128_418.	POS	5.128	418.1487
**Atevirdine**	10.066_37!	POS	10.066	379.202
**Anastrozole**	6.245_293.	POS	6.245	293.1627
**PS(15:1(9Z)/22:2(13Z,16Z))**	10.239_79!	NEG	10.239	799.5394
**Daf-FM DA**	6.053_496.	POS	6.053	496.1094
**11-Deoxytetrodotoxin;**	0.751_303.	POS	0.751	303.1067
**PS(12:0/13:0)**	6.127_637.	POS	6.127	637.3965
**5-Hydroxy-6-methoxyindole glucuronide**	5.659_339.	POS	5.659	339.0953
**Fluvoxamine**	6.114_318.	POS	6.114	318.1547
**Bufotenin**	4.197_204.	POS	4.197	204.1266
**26,26,26-trifluoro-25-hydroxyvitamin D3**	7.764_454.	POS	7.764	454.3044
**4-[[(1R)-2-[[(2R)-2-(2-Adamantyloxycarbonylamin**	7.949_614.	POS	7.949	614.31
Other				
**Cycloastragenol**	10.013_49(	POS	10.013	490.3638
**1,4-Dihydroxynaphthalene**	5.804_160.	POS	5.804	160.0526

**methoxycatecholamine**	1.767_155.	POS	1.767	155.0584
**p-coumaroyl-homoserine lactone**	3.91_247.C	NEG	3.91	247.0843
**As-PL(20:0/16:0)**	7.511_101	POS	7.511	1014.577
**Fumiquinazoline F;**	7.082_358.	POS	7.082	358.1438
**1R-cis-3,3,5-Trimethylcyclohexyl ester5-oxo-L-pro**	7.308_253.	POS	7.308	253.1678
**Dapta**	5.666_856.	POS	5.666	856.3921
**Macrolactin P**	5.948_578.	POS	5.948	578.31
**Acetylcholine**	0.738_145.	POS	0.738	145.1105
**zaltoprofen**	0.744_298.	POS	0.744	298.0659
**4-(Cytidine 5'-diphospho)-2-C-methyl-D-erythrit**	3.588_521.	POS	3.588	521.0823
**1-Palmitoylphosphatidylcholine**	10.103_49	NEG	10.103	495.3322
**Thiamine**	7.187_265.	NEG	7.187	265.1135
**Lamivudine**	2.056_229.	POS	2.056	229.0521
Other				
**3,7-Epoxycaryophyllan-6-One**	9.787_254.	POS	9.787	254.1883
**Methylendiglyoxal**	1.155_128.	POS	1.155	128.0112
**1-Nitrobenzantrone**	4.034_275.	POS	4.034	275.0574
**Hypophyllanthin**	6.605_430.	POS	6.605	430.1988
**4-Anilino-4-oxobutanoic acid**	6.692_193.	POS	6.692	193.0741
**3-(4-Carboxybenzylidene)camphor**	6.251_284.	POS	6.251	284.1414
**N-Acetyl-D-galactosaminyldiphosphoundecapren**	8.018_100	POS	8.018	1003.568
**Phenyl valerate**	8.859_178.	POS	8.859	178.0996
**Imidazo(5,1-F)(1,2,4)triazine, 4-(3-fluoro-1-azetid**	6.062_431.	POS	6.062	431.1496
**2-[[(1R)-2-[Bis(carboxymethyl)amino]cyclohexyl]-[**	6.057_594.	POS	6.057	594.202
**SM(d16:1/24:1(15Z))**	12.895_78	POS	12.895	784.6457
**Monoisobutyl phthalic acid**	6.315_222.	POS	6.315	222.0894
**Tomaymycin-(DH)2-PPL hemiaminal**	5.502_151.	POS	5.502	151.0635
**trans-4,5-Dihydroxy-4,5-dihydropyrene; trans-4,5-**	0.754_236.	POS	0.754	236.0833
**4-Aminohippuric acid**	2.403_194.	NEG	2.403	194.0691
**DL-5-Methoxytryptophan**	5.418_234.	NEG	5.418	234.1003
Other				
**2-Octenoylcarnitine**	5.296_285.	POS	5.296	285.1954
**r-limonene**	6.658_184.	POS	6.658	184.1101
**tubercidin**	0.743_266.	POS	0.743	266.1017
**5-Methoxyindoleacetic acid**	3.894_205.	POS	3.894	205.0742
**Herbacetin 8-(3''-acetylxyloside)**	6.288_476.	POS	6.288	476.096
**(Z)-7-[(1R,2R,3R)-3-Hydroxy-2-[(E,3R)-3-hydroxy-**	4.513_465.	POS	4.513	465.1815
Nocardioides_hwasunensis				
**Tilisolol**	7.963_304.	POS	7.963	304.1784
**PE(14:0/0:0)**	9.162_425.	POS	9.162	425.2544
**OOV-PA**	7.283_534.	POS	7.283	534.2982
**Methionine ethyl ester**	2.94_177.C	POS	2.94	177.0826
**4-[2-[2-[3-Acetamido-2,5-dihydroxy-6-(hydroxym**	5.913_492.	POS	5.913	492.2048
**(3R,4S)-1-(4-Fluorophenyl)-4-[(3S)-3-(4-fluoroph**	6.595_409.	NEG	6.595	409.1486
**Dmap-ethyl-paf**	9.305_572.	POS	9.305	572.3928
Other				
**L-Alanyl-gamma-D-glutamyl-L-lysine; L-Ala-gam**	3.29_346.1	POS	3.29	346.1851
**Acetyl-N-formyl-5-methoxykynurenamine**	4.402_264.	POS	4.402	264.1119
**norzolmitripan**	4.345_259.	POS	4.345	259.1332
**Isomollupentin 7-O-glucoside**	6.211_564.	NEG	6.211	564.1485
**Naphthalene-1,2,4-trione**	4.447_174.	POS	4.447	174.0321
**2-C-Methyl-D-erythritol 2,4-cyclodiphosphate; 3-I**	0.841_277.	POS	0.841	277.9951
**[D-Met2, Pro5]-enkephalinamide**	6.441_612.	POS	6.441	612.2725
**Pyridine N-oxide glucuronide**	4.933_272.	POS	4.933	272.0773
**Fe-enterobactin; Fe-enterochlin;**	6.517_719.	POS	6.517	719.3447
**Mitoxantrone dicarboxylic acid**	4.297_472.	POS	4.297	472.1598
Other				
**Valactamide E putative**	10.591_42	POS	10.591	424.3319

**3-(3-Hydroxy-4-methoxynaphthalen-1-yl)oxyprop**	6.048_264.	POS	6.048	264.0999
**Phenylacetylglycine**	6.111_193.	POS	6.111	193.074
**4-(4,5-Diphenyl-1H-imidazol-2-yl)benzoyl Chlorid**	3.713_358.	POS	3.713	358.0865
**Thymol**	5.489_150.	POS	5.489	150.1047
**Deshydroxyethoxy Ticagrelor**	7.293_478.	NEG	7.293	478.1587
**10-Methyl-9-(10-methyl-1H-acridin-9-yl)-1H-acri**	3.256_388.	POS	3.256	388.1959
**Valeant**	4.922_374.	NEG	4.922	374.0751
**Imazapyr**	2.7_261.11	POS	2.7	261.1113
**Deacetoxycephalosporin C; DAOC;**	6.523_357.	POS	6.523	357.0986
**Sotalol**	5.37_272.1	POS	5.37	272.1195
Schwartzia_succinivorans				
Microvirga_vignae				
Tessaracoccus_oleiagri				
Paenibacillus_naphthalenovorans				
Terrabacter_lapilli				
Other				
Other				
Selenomonas_lacticifex				
Gaiella_occulta				
**11(Z),14(Z)-Eicosadienoic acid**	10.444_30	NEG	10.444	308.2715
**[(1S,3R,3aS,4S,8aR)-1-acetyloxy-3-hydroxy-6,8a-d**	6.413_416.	POS	6.413	416.2196
**(2S)-3-[3-[(4-Carbamimidoylbenzoyl)amino]propa**	6.484_489.	POS	6.484	489.1688
**gamma-Glutamyldopa**	4.401_326.	POS	4.401	326.1111
**N-Acetyl-9-aminominocycline, (4R)-**	5.616_397.	POS	5.616	397.2115
**alpha-carboxyethylhydroxychroman sulfate**	4.837_358.	NEG	4.837	358.1089
**20-HETE ethanolamide**	9.719_363.	POS	9.719	363.2774
**conocarpan**	4.16_288.1	POS	4.16	288.111
**1,4-Diazabicyclo[2.2.2]octane**	3.108_112.	POS	3.108	112.1001
**omega5(Z)-etherolenic acid**	7.878_292.	POS	7.878	292.2039
Bacillus_thermoamylovorans				
**3a-Methyl-2,3,4,5,5a,10,10a,10b-octahydro-1H-cy**	7.691_274.	POS	7.691	274.1569
**2-(4-(3-Ethynylanilino)-6-(2-methoxyethoxy)quin**	4.688_379.	POS	4.688	379.1529
**Dihydroferulic acid 4-O-glucuronide**	0.734_372.	NEG	0.734	372.1056
**6-Hydroxy-3,4-dihydro-2(1H)-quinolinone**	4.981_163.	POS	4.981	163.0635
**(1R,4R,5S)-1-[(1S)-1-Hydroxy-2-methylpropyl]-4-**	5.451_241.	POS	5.451	241.1315
**Anastatin B**	0.87_378.0	NEG	0.87	378.0739
**trans-Petroselinic acid**	10.187_28	NEG	10.187	282.2559
**(E)-1-(3,4-dihydroxy-2,6-dimethoxyphenyl)-3-phe**	5.936_317.	POS	5.936	317.1261
**13H-Dibenzo[a,g]fluorene;**	0.741_266.	POS	0.741	266.1104
**Decoside; Decogenin 3-O-alpha-L-oleandroside;**	7.605_546.	NEG	7.605	546.2855
**Bms-275183**	7.163_845.	POS	7.163	845.3834
**6,18-bis(1-hydroxy-3-methylbut-2-en-1-yl)-11,22**	6.537_552.	POS	6.537	552.2012
**9-[[3-methyl-3-[(4-methyl-5-oxo-2H-furan-2-yl)**	5.488_382.	POS	5.488	382.1053
**8-Hydroxymianserin**	7.856_280.	NEG	7.856	280.1589
**Gamma-linolenic acid**	8.43_278.2	POS	8.43	278.2245
**Acebutolol**	6.515_336.	POS	6.515	336.2048
**Valpromide**	2.957_143.	POS	2.957	143.1312
**Dihydrocapsaicin**	6.376_307.	POS	6.376	307.2148
**SM(d18:1/14:0)**	10.876_67	POS	10.876	674.5359
**14-Fluoro-6-thiaheptadecanoic acid**	6.214_306.	POS	6.214	306.2041
**NCGC00380375-01!6-[(4E,6E)-2,3-dihydroxy-4,6-d**	6.106_416.	POS	6.106	416.2199
**PG(22:4(7Z,10Z,13Z,16Z)/0:0)**	7.705_560.	POS	7.705	560.3135
Pseudoflavonifractor_capillosus				
**Dehydroeburicoic acid**	10.699_46	POS	10.699	468.3583
**trans-beta-damascenone**	7.464_190.	POS	7.464	190.136
**1-(2-methoxy-eicosanyl)-sn-glycero-3-phosphoet**	9.115_525.	POS	9.115	525.3772
**PGE2alpha dimethyl amine**	9.73_367.3	POS	9.73	367.3086
**cis-Entacapone**	5.022_305.	NEG	5.022	305.1011

**AMP-Deoxynojirimycin**	6.502_397.	POS	6.502	397.2825
**2-Propenoic acid, 3-(3,4-dihydroxyphenyl)-, pheny**	5.672_256.	NEG	5.672	256.0733
**Pentolame**	9.621_357.	POS	9.621	357.2667
**7-hydroxy-2-[4-[(2S,3R,4S,5S,6R)-3,4,5-trihydroxy**	7.444_418.	POS	7.444	418.1264
**Capsi-amide; N-(13-Methyltetradecyl)acetamide; C**	9.788_269.	POS	9.788	269.2719
**L-Iditol, 1,2,5,6-tetradeoxy-2,5-bis(((2S)-3-methyl-**	7.477_766.	POS	7.477	766.3931
Other				
Clostridium_disporicum				
**Bentazone**	4.35_240.0	POS	4.35	240.0572
**Pymetrozine**	3.392_217.	POS	3.392	217.0954
**Austrobuxusin L**	7.208_566.	NEG	7.208	566.3096
**Carbamic acid, (5-amino-1,2-dihydro-3-(4-methyl**	5.646_325.	POS	5.646	325.1528
**Ulipristal acetate**	9.192_475.	POS	9.192	475.27
**(2S)-6-Hydroxy-2,7,8-trimethyl-2-[(4R,8R)-4,8,12-**	8.922_458.	POS	8.922	458.3399
**13,16-Docosadienoic acid**	10.253_33	POS	10.253	336.3028
**Bentazepam**	0.816_296.	POS	0.816	296.0985
**Campesteryl glucoside**	9.786_562.	POS	9.786	562.4241
**2,3-Dinor prostaglandin E1**	6.703_326.	NEG	6.703	326.2093
**2-Keto-glutaramic acid**	1.12_145.0	POS	1.12	145.02
**4-acetamidobutanoate**	1.428_145.	POS	1.428	145.0741
**2,8-Dihydroxyquinoline-beta-D-glucuronide**	4.252_337.	POS	4.252	337.0793
**Neobavaisoflavone**	5.389_360.	POS	5.389	360.0775
**1-[4-[2-(7-methoxy-1,3-benzodioxol-5-yl)ethyl]p**	6.08_391.1	POS	6.08	391.1988
**5-hydroxyindoleacetate**	3.489_191.	POS	3.489	191.0586
**OKDdiA-PG**	9.519_734.	POS	9.519	734.4031
**[(2E,6Z,10Z)-7,11-bis(acetyloxymethyl)-3,15-dimet**	7.557_448.	POS	7.557	448.2829
**dimethyl 2,4-bis(4-hydroxyphenyl)cyclobutane-1,3**	6.074_373.	POS	6.074	373.1519
**O-(1->4)-alpha-L-Dihydrostreptosyl-streptidine 6**	4.3_488.16	POS	4.3	488.1652
**PG(O-16:0/12:0)**	10.605_65	POS	10.605	652.4686
**(3S,4S)-5-[(3S,4S)-4,10-dihydroxy-7,9-dimethoxy-**	6.548_616.	POS	6.548	616.1715
**Acetamide, N-(((5S)-3-(3-fluoro-4-(6-(1-methyl-1**	6.682_411.	POS	6.682	411.1456
**1-Pyrroline-5-carboxylic acid**	1.337_113.	POS	1.337	113.0479
**Pamicogrel**	0.588_448.	NEG	0.588	448.1444
**Diprotin A**	4.489_341.	POS	4.489	341.2304
**Amino (2S)-2,6-diaminohexanoate**	0.752_161.	POS	0.752	161.1166
**Melithiazole E; Cystothiazole A;**	5.322_422.	POS	5.322	422.1324
**Methylhippuric acid**	6.431_193.	POS	6.431	193.0741
**Filgotinib**	4.284_425.	POS	4.284	425.1512
**ent-17-Hydroxy-16beta-kauran-19-al**	10.015_30	POS	10.015	304.2402
**Nafagrel**	6.539_256.	POS	6.539	256.1211
**trans-cinnamoyl-beta-D-glucoside**	2.837_327.	POS	2.837	327.1317
**Derrusnin**	5.991_373.	POS	5.991	373.1161
**4,15,26-trihydroxy-1-oxa-4,9,15,20,26,31-hexazac**	5.679_640.	POS	5.679	640.2853
**Cobalt-precorrin 3;**	6.573_935.	POS	6.573	935.2402
**Thioxanthen-9-one**	0.749_212.	NEG	0.749	212.0296
**2-Chlorooctadecanoic acid**	10.187_31	NEG	10.187	318.2325
**TRIPHENYLPHOSPHINE**	6.305_262.	POS	6.305	262.0918
**Fluorescein dilaurate**	9.335_696.	POS	9.335	696.406
**3'-Hydroxyhexobarbital**	3.636_252.	POS	3.636	252.111
**Cerulenin**	7.595_223.	POS	7.595	223.1211
**Oxydisulfoton;**	6.443_290.	POS	6.443	290.0239
**Naloxegol**	6.418_651.	POS	6.418	651.363
**9Z,13-Tetradecadien-11-ynal**	9.777_204.	POS	9.777	204.1518
**PA(14:1(9Z)/0:0)**	3.293_380.	POS	3.293	380.1951
**Icofungipen**	3.625_141.	POS	3.625	141.0792
**N-Lactoylleucine**	4.316_203.	POS	4.316	203.116
**Nadolol**	6.356_309.	POS	6.356	309.1939
**Epinephrine glucuronide**	5.42_359.1	POS	5.42	359.1202

10-Deoxymethynolide;	7.618_296.	POS	7.618	296.1986
Indole-3-acetamide	4.413_174.	NEG	4.413	174.0794
Octadecadienylcarnitine	10.089_40	POS	10.089	409.3556
Isopilocarpinic acid	5.672_226.	NEG	5.672	226.1317
5beta-Cyprinolsulfate	8.346_532.	NEG	8.346	532.3064
12-Epileukotriene B4	8.745_336.	NEG	8.745	336.2301
TUG-891	2.668_364.	POS	2.668	364.1481
Pyridoxine	1.129_169.	POS	1.129	169.0741
Anaerotruncus_colihominis				
Veillonella_rogosae				
LMPK12010029	6.759_475.	POS	6.759	475.1253
Levosimendan	4.483_280.	POS	4.483	280.1059
Desacetylcefotaxime	4.435_413.	POS	4.435	413.0465
Indan-1-ol	5.752_134.	POS	5.752	134.0733
Other				
Ethyl octadeca-9,12,15-trienoate	8.963_306.	POS	8.963	306.2558
Tiocarbazil; Thiocarbazil;	6.395_279.	POS	6.395	279.1666
[(2R,3S,4R,5R)-5-(4-Amino-2-oxopyrimidin-1-yl)-	6.541_614.	POS	6.541	614.1459
3-Hydroxyphysodic acid	5.604_486.	POS	5.604	486.1878
Estramustine	6.506_439.	NEG	6.506	439.1665
Sutterella_stercoricanis				
Dioncophylline C	3.988_363.	POS	3.988	363.1831
9-Oxo-ODE	8.434_294.	POS	8.434	294.2193
Linustatin;	3.681_409.	POS	3.681	409.1597
N-(3-Phenylpropyl)-3,4-dihydroxybenzylidene cya	5.977_439.	POS	5.977	439.127
Meiothermus_ruber				
Methyl 3-benzoyloxy-8-(3-hydroxypropyl)-8-azab	5.645_347.	POS	5.645	347.1727
dTDP-4-amino-2,4-dideoxy-beta-L-xylose;	0.599_517.	NEG	0.599	517.0845
Losartan	8.438_422.	NEG	8.438	422.1628
(3R,4R)-4-Amino-1-[[4-[(3-methoxyphenyl)amino]	6.047_368.	POS	6.047	368.1949
Methylobacterium_komagatae				
Fluphenazine sulfoxide	4.187_453.	POS	4.187	453.1683
HC Blue No.1; 2,2'-[[4-(Methylamino)-3-nitrophen	3.315_255.	POS	3.315	255.1222
4-oxo-4-(3-oxodecan-2-ylamino)butanoic acid	5.764_271.	POS	5.764	271.1785
riboflavin thiamine	4.727_408.	POS	4.727	408.1088
Atenolol-desisopropyl	3.673_224.	POS	3.673	224.1163
Epigoitrin	5.739_129.	POS	5.739	129.025
[(2R,3R,4S,5S,6R)-3-acetyloxy-2-(acetyloxymethyl)	5.638_693.	POS	5.638	693.4294
8-Aminohexylamino cAMP	4.959_443.	POS	4.959	443.1691
ginnalin A	7.724_468.	NEG	7.724	468.0893
4-Amino-N,N-dimethylbenzenesulfonamide	2.33_200.0	POS	2.33	200.0623
1alpha,25-dihydroxy-26,27-dimethyl-22-thia-20-e	8.143_462.	POS	8.143	462.3184
4-((6-Methoxyquinolin-8-yl)amino)pentanoic acid	4.904_274.	NEG	4.904	274.1315
N-palmitoyl leucine	9.067_369.	POS	9.067	369.3242
6-Hydroxy-5-methoxy-1h-indole-2-carboxylic aci	4.802_207.	POS	4.802	207.0533
Tenofovir disoproxil	4.464_519.	POS	4.464	519.1746
p-N,N-(Dimethylamino)phenyldiazonium fluorobo	2.953_234.	POS	2.953	234.9906
2-Oxindole	4.788_133.	POS	4.788	133.0529
Tangeritin	7.857_372.	POS	7.857	372.1204
Ticagrelor	9.53_522.1	POS	9.53	522.1843
Flemichin A	6.118_488.	POS	6.118	488.2175
L-Methionine sulfoximine	5.781_202.	POS	5.781	202.0398
alpha-Methylene-gamma-butyrolactone	3.052_98.0	POS	3.052	98.03699
1-Dehydro-[6]-gingerdione;	7.82_290.1	POS	7.82	290.1517
Dapdiamide A; 3-{[(2E)-4-Amino-4-oxobut-2-eno	4.133_300.	POS	4.133	300.1431
rosavin	6.448_428.	POS	6.448	428.1684
skimmin	3.081_341.	POS	3.081	341.111
Timosaponin A-III;	9.332_740.	POS	9.332	740.4328

**N-stearoyl valine**	9.35_383.3	POS	9.35	383.34
**NCGC00380970-01!3-hydroxy-2-[[3-(3-hydroxy-6**	6.414_400.	POS	6.414	400.2464
**Edoxaban**	6.338_547.	POS	6.338	547.1746
**7-Hydroxycannabidiol**	8.318_330.	POS	8.318	330.2198
**Lotrafiban**	8.338_428.	POS	8.338	428.241
**Dihydrodaidzein 7-O-glucuronide**	6.437_432.	POS	6.437	432.1055
Lactobacillus_ultunensis				
**Benzoxazine-6-carboxylic acid**	4.074_177.	POS	4.074	177.0431
Other				
Other				
Rothia_amarae				
Other				
Sphingobacterium_psychroaquaticum				
Deinococcus_hopiensis				
Other				
Streptococcus_mutans				
**Z-Devd-fmk**	6.949_668.	POS	6.949	668.2699
**Stigmatellin Y;**	9.366_484.	POS	9.366	484.2831
**5-(4-Methylphenyl)-7-tert-butyl-4,5,6,7-tetrahydr**	6.824_285.	POS	6.824	285.1964
**Geranylgeranyl pyrophosphate**	3.721_450.	NEG	3.721	450.2002
**L-Valine, N-(2-hydroxy-3-butenyl)-**	4.759_187.	POS	4.759	187.1211
**Phoslactomycin B;**	6.922_513.	POS	6.922	513.2484
**[(1R,5S,6S)-5-acetyloxy-3-(hydroxymethyl)-2-oxo-**	4.352_362.	POS	4.352	362.1114
**4-[5-(3,4-dimethoxyphenyl)-3,4-dimethyloxolan-2**	4.285_345.	POS	4.285	345.1941
**N,N-Dimethyl-5-({2-Methyl-6-[(5-Methylpyrazin-**	6.814_432.	POS	6.814	432.1534
**Cellulose hydroxyethylate**	5.627_806.	POS	5.627	806.4513
**2-Nonenal**	5.987_140.	POS	5.987	140.1203
**3-oxo-3-[[(2R,3S,4S,5R,6S)-3,4,5-trihydroxy-6-(4-I**	6.048_414.	POS	6.048	414.1537
**Ergosta-4,6,8(14),22-tetraen-3-one**	10.858_39	POS	10.858	392.3081
Roseburia_faecis				
**12S-hydroxy-16-heptadecynoic acid**	9.734_282.	POS	9.734	282.2195
**(10S)-Juvenile hormone III acid diol;**	8.413_270.	POS	8.413	270.1832
**PE(O-18:0/0:0)**	10.658_46	NEG	10.658	467.3375
**(E)-2-(4,8-dimethylnona-3,7-dien-1-yl)-5-hydroxy**	9.415_354.	NEG	9.415	354.2196
**Morpholine**	2.093_87.0	POS	2.093	87.06855
**2-(2-Aminoethoxymethyl)-4-(2-chlorophenyl)-6-r**	6.677_408.	POS	6.677	408.1449
**Doranidazole**	0.646_247.	NEG	0.646	247.0803
**Bilobalide**	4.319_326.	POS	4.319	326.1002
**(4-((5,6-Diphenylpyrazin-2-yl)(isopropyl)amino)bu**	5.906_419.	POS	5.906	419.2207
**Cyclopamine**	9.862_411.	POS	9.862	411.3139
**Zaragozic acid A**	7.669_690.	POS	7.669	690.2897
Treponema_bryantii				
**NCGC00179744-02!(2S)-2-[(1S,2R)-1,2-dihydroxyp**	5.482_252.	POS	5.482	252.0974
**N-Acetyl-DL-homocysteine**	0.745_177.	POS	0.745	177.0463
**Lisinopril**	6.824_405.	POS	6.824	405.2262
**6-Sulfo-beta-D-quinovose;**	3.315_244.	POS	3.315	244.026
**(2S,3R,4S,5S,6R)-2-[3-Hydroxy-5-[2-(3-hydroxy-4**	5.644_420.	POS	5.644	420.14
Fusicatenibacter_saccharivorans				
**LMGP06030059**	7.486_822.	POS	7.486	822.5604
**Lamiide;**	0.735_422.	NEG	0.735	422.1424
**N-Nitrosomethylethylamine (NMEA)**	1.313_88.0	POS	1.313	88.06378
**Benzoyleneurea**	3.911_162.	POS	3.911	162.0431
Other				
**2-[[3-[[2-(Dimethylamino)phenyl]methyl]-2-pyridi**	11.941_42	POS	11.941	429.2878
**Tert-Butyl (4-(3-((7-(hydroxyamino)-7-oxoheptyl)**	6.403_446.	POS	6.403	446.2161
**3',4',5-Trihydroxy-3-methoxy-6,7-methylenedioxy**	5.7_520.08	NEG	5.7	520.0847
**urea citrate**	4.265_246.	POS	4.265	246.0132
**4-[2-[(1R,4aS,5R,6R,8aS)-6-hydroxy-5-(hydroxyme**	6.324_372.	POS	6.324	372.19

**Monepantel**	7.52_473.0 POS	7.52	473.0648
**RETENE**	6.448_234. POS	6.448	234.1418
**3,9-Dimethoxypterocarpan**	6.274_301. POS	6.274	301.1307
**beta-Sitosterol**	8.875_436. POS	8.875	436.3667
**methyl 3-(4-hydroxyphenyl)-2-[(4-hydroxyphenyl)**	6.169_387. POS	6.169	387.1318
**(-)-Arctigenin**	5.711_394. POS	5.711	394.1376
**Secobarbital**	5.598_238. NEG	5.598	238.1316
**6-Hydroxykaempferol 3,6-dimethyl ether 7-glucos**	4.792_490. NEG	4.792	490.1473
**Benzaldehyde**	3.198_106. POS	3.198	106.0421
**Fenclorac**	2.959_286. POS	2.959	286.0539
**Estradiol sulfamate**	6.272_351. POS	6.272	351.1511
**Thromboxane**	10.112_29 NEG	10.112	296.3079
**4-Octylphenol**	10.03_206. POS	10.03	206.1673
**Gliclazide**	6.177_323. POS	6.177	323.1301
**Zopiclone N-oxide**	7.149_404. POS	7.149	404.0994
Other			
**4,5-Dihydroorotic acid**	5.589_338. NEG	5.589	338.046
**Isovitexin 2''-O-arabinoside**	6.744_564. POS	6.744	564.1479
**PS(P-18:0/0:0)**	10.095_50 NEG	10.095	509.3116
**Ibuprofen glucuronide**	7.362_382. NEG	7.362	382.163
**N-[2-(3,4-Dihydroxyphenyl)ethyl]icosa-5,8,11,14-t**	9.78_439.3 POS	9.78	439.3066
**(Z)-2,6-dimethyl-7-(4-methyl-5-oxooxolan-2-yl)-**	8.559_463. POS	8.559	463.2419
**3-Indoleacetonitrile**	3.292_156. POS	3.292	156.0689
**Terbacil;**	5.137_216. POS	5.137	216.0676
**PA(17:0/22:0)**	8.418_746. POS	8.418	746.5813
**trans-Clovamide**	4.493_359. POS	4.493	359.1005
**L-Lysine**	0.617_146. POS	0.617	146.1057
**Oxyphenbutazone**	7.145_324. POS	7.145	324.1472
**5-OxoETE**	7.24_318.2 POS	7.24	318.219
**All-trans 4-Keto Retinoic Acid**	8.698_314. POS	8.698	314.188
**Urea, N,N'-dichloro-N,N'-bis(2,4,6-trichlorophenyl**	0.707_483. POS	0.707	483.782
**Propiconazole**	3.739_341. NEG	3.739	341.0683
**6-Chloro-2,3,4,9-tetrahydro-1H-carbazole-1-carb**	7.49_248.0 POS	7.49	248.0711
**6-formyl-10-(hydroxymethyl)-5-methoxy-3-methy**	3.784_409. POS	3.784	409.2097
**Palbociclib**	5.13_447.2 POS	5.13	447.2367
**3,4-dimethyl-5-carboxyethyl-2-furanpentanoic aci**	7.362_268. NEG	7.362	268.1308
**3-Des(dimethylaminoethyl)-3-acetic acid zolmitrip**	6.205_274. POS	6.205	274.0954
**Linoleamide**	9.542_279. POS	9.542	279.256
**(Z)-4-(2-Hydroxy-5-sulfonatophenyl)-2-oxo-3-bu**	3.672_271. NEG	3.672	271.999
**1-Hydroxytrimazosin**	7.125_451. POS	7.125	451.2084
**2-Methoxyphenylmetyrapone**	4.745_255. POS	4.745	255.1267
**PA(O-18:0/21:0)**	9.988_732. POS	9.988	732.6006
**Nervonic acid**	12.313_36 NEG	12.313	366.3499
**ascr#6.2**	5.521_248. POS	5.521	248.1624
**hyocholic acid**	6.952_157. POS	6.952	157.1469
**Drimianin C**	7.16_562.2 POS	7.16	562.2776
**PKOHA-PE**	5.948_591. POS	5.948	591.3145
**Cefapime**	6.515_462. POS	6.515	462.1167
Ezakiella_peruensis			
Varibaculum_cambriense			
Propionicimonas_paludicola			
**Lupinine;**	9.542_169. POS	9.542	169.1468
**1-Oxo-2,3-dihydro-1H-indene-4-carboximidamid**	3.297_174. POS	3.297	174.0794
**(2R,3S,4S,5R,6R)-6-[1,7-bis(4-hydroxyphenyl)hepta**	4.207_305. POS	4.207	305.6457
**Carbosulfan**	4.367_380. POS	4.367	380.2136
**angoletin**	3.175_317. POS	3.175	317.1624
**Imazamox**	2.964_305. POS	2.964	305.1375
**Di-deacetyl adrogolide**	6.27_315.1 NEG	6.27	315.13

**Loxapine**	4.055_327.	POS	4.055	327.114
**2-(3-Hydroxy-1H-indol-2-yl)acetic acid**	4.418_191.	POS	4.418	191.0586
**methyl (1S,12S,13E)-13-ethylidene-18-(hydroxyme**	6.361_399.	POS	6.361	399.2157
**Oxazolo[5,4-B]pyridine**	1.052_120.	POS	1.052	120.0325
**Corylifol A**	6.637_390.	POS	6.637	390.1824
**5'-Deoxy-5-fluorocytidine**	4.499_245.	POS	4.499	245.0801
**Cer(d16:2(4E,6E)/20:1(11Z))**	11.192_56	POS	11.192	561.5125
**Mono(2-ethylhexyl) phthalate (MEHP)**	7.873_278.	NEG	7.873	278.1516
**1-Hexadecylthio-1-deoxy-2-hexadecylphosphono**	10.082_78	POS	10.082	786.562
**Cosmosiin**	3.737_432.	NEG	3.737	432.1059
Comamonas_jiangduensis				
**2,3-Epoxyphylloquinone**	9.759_466.	POS	9.759	466.3449
**a-Lactose**	0.744_388.	NEG	0.744	388.1216
**Dhesn**	7.447_549.	POS	7.447	549.295
**(2S,3S)-(-)-3-Phenylglycidol**	2.853_150.	POS	2.853	150.0683
**N-Butyl-N-(4-hydroxybutyl)nitrosamine**	0.635_174.	POS	0.635	174.1371
**Glucoputranjivin; 1-Methylethyl glucosinolate;**	2.745_361.	POS	2.745	361.0517
Corynebacterium_doosanense				
**Methyl cinnamate**	7.488_162.	POS	7.488	162.0682
**PS(18:0/20:2(11Z,14Z))**	10.411_81	POS	10.411	815.5683
**Sulfotep**	0.849_322.	POS	0.849	322.0214
**GlcADG(18:3((9Z,12Z,15Z)/16:0)**	5.924_766.	NEG	5.924	766.5221
**3-(2-Ethyl-5-hydroxyhexyl)phthalic acid**	6.572_294.	POS	6.572	294.1469
**Deserpidine**	5.833_578.	POS	5.833	578.2657
**4-Methyl-N-[4-[(4-nitrophenyl)azaniumyl]phenyl]**	5.285_387.	NEG	5.285	387.1351
**NCGC00380826-01![1-(3,4-dihydroxy-5-methoxyp**	4.464_421.	POS	4.464	421.2101
Atopostipes_suicloacalis				
**4-Ethyl-2-propyl-1-(2'-(1H-tetrazol-5-yl)biphenyl**	4.971_416.	NEG	4.971	416.1949
**2-hydroxy-4-methoxy-3,5-bis(3-methylbut-2-eny**	7.479_412.	POS	7.479	412.2032
**Hhcfu**	6.177_273.	POS	6.177	273.1113
**2,4,6-Tri-tert-butylphenol**	9.539_262.	POS	9.539	262.2297
**hexamethylolmelamine pentamethyl ether (putativ**	5.926_376.	POS	5.926	376.2053
**Tiotropium**	5.11_392.0	POS	5.11	392.0994
**Laudanosine**	4.642_357.	POS	4.642	357.1935
**Alfaprostolum**	8.769_406.	POS	8.769	406.2719
**(S)-2-Hydroxypropyl-CoM; (2S)-2-Hydroxypropyl-**	2.952_200.	POS	2.952	200.0174
**Benzoxazine**	4.971_133.	POS	4.971	133.0529
**5-(3',4',5'-Trihydroxyphenyl)-gamma-valerolacton**	4.733_304.	POS	4.733	304.026
**Biotin**	2.75_266.0	POS	2.75	266.0702
**Glutarylglycine**	0.652_189.	NEG	0.652	189.0639
**(1R,2E,8S,10R,11S)-10,11-dihydroxy-6-(methoxym**	5.633_368.	POS	5.633	368.1469
Other				
**2,2',5,5'-Tetrachlorobenzidine;**	0.683_319.	NEG	0.683	319.9448
**Epitestosterone**	7.773_288.	POS	7.773	288.2088
Other				
**methyl 8-[3,5-epidioxy-2-(3-hydroperoxy-1-pente**	7.573_396.	POS	7.573	396.2513
**Pyrazolo[1,5-a]pyrimidine**	4.271_119.	NEG	4.271	119.0484
**(E,E,E)-Geranylgeranyl alcohol**	9.785_290.	POS	9.785	290.2606
**16beta-hydroxy-melianthugenin**	6.527_472.	POS	6.527	472.2106
**6-Gingerol**	7.343_276.	POS	7.343	276.1726
**Isolobinine;**	4.26_287.1	POS	4.26	287.1882
**Ikarisoside E**	5.095_498.	POS	5.095	498.151
**Cardiopetalidine;**	6.287_363.	POS	6.287	363.2408
**Candoxatrilat**	7.456_399.	POS	7.456	399.2258
**Docosahexaenoylethanolamine**	8.023_371.	POS	8.023	371.2822
**Spinacetin 3-O-sulfate**	5.747_426.	NEG	5.747	426.0257
**9-methoxy-4,4-dimethyl-13-phenyl-6,14-dioxatet**	5.423_358.	POS	5.423	358.1165
**Baohuoside I**	7.042_514.	POS	7.042	514.1844

**N-Methoxy-3-(3,5-di-tert-butyl-4-hydroxybenzyli**	7.323_331.	POS	7.323	331.2138
**Leurubicin**	6.625_656.	POS	6.625	656.2549
**1-[(1R,2R,3S,4S)-3-Hydroxy-4,7,7-trimethyl-2-bicy**	4.145_366.	POS	4.145	366.1612
**Xemilofiban**	6.386_358.	POS	6.386	358.1624
**2-((2'-(5-Ethyl-3,4-diphenyl-1H-pyrazol-1-yl)-[1,1**	0.743_474.	POS	0.743	474.1963
**Rose bengal; Tetrachlorotetraiodofluorescein disod**	7.382_101	NEG	7.382	1017.641
**Nolomirole**	6.438_333.	NEG	6.438	333.194
**2-Hydroxy-1,4-naphthoquinone**	3.919_174.	NEG	3.919	174.0317
Intestinimonas_butyriciproducens				
**Elubrixin**	7.141_462.	POS	7.141	462.0332
**COTININE**	2.931_176.	NEG	2.931	176.095
**3-[(4-Amino-6-chloro-1,3,5-triazin-2-yl)amino]be**	0.749_301.	POS	0.749	301.0031
**LMGP06030016**	7.428_814.	POS	7.428	814.5017
**10H-Pyrido(3,2-b)(1,4)benzothiazine, 10-(2-morph**	6.689_313.	POS	6.689	313.1244
**(+)-Eseroline**	5.153_218.	POS	5.153	218.1421
**True blue**	5.614_344.	POS	5.614	344.1258
**N-Succinyl-ala-ala-pro-phe-p-nitroanilide**	9.731_624.	POS	9.731	624.2524
**Norrhizocarpic acid**	7.127_455.	POS	7.127	455.1366
Pelomonas_aquatica				
**Rhamnocitrin**	6.845_300.	POS	6.845	300.0632
**NCGC00380239-01!16-hydroxy-4,4,8,10,14-pentan**	9.21_472.3	POS	9.21	472.3557
**1,1'-Spirobi[1H-indene]-5,5',6,6'-tetrol, 2,2',3,3'-te**	6.535_340.	POS	6.535	340.1668
**CPA(18:1(9Z))**	8.282_418.	POS	8.282	418.2502
**Tert-butyl formate**	8.266_102.	POS	8.266	102.0682
**n-octyl-beta-D-thioglucopyranoside**	8.89_308.1	POS	8.89	308.1664
Lactobacillus_panis				
**Celastrol**	8.829_468.	POS	8.829	468.288
Other				
Actinomadura_sputi				
**Formononetin**	7.151_268.	NEG	7.151	268.0733
**Hydroxyphenyllactic acid**	1.237_182.	NEG	1.237	182.058
**2-(((3,5-Dichlorophenyl)carbamoyl)oxy)-2-methyl-**	3.287_303.	POS	3.287	303.0068
Staphylococcus_equorum				
Methylobacterium_variabile				
**2',4-Dihydroxychalcone**	6.531_240.	POS	6.531	240.0787
**2-(Furan-2-yl)-7-phenethyl-7H-pyrazolo[4,3-e][1,**	6.419_345.	POS	6.419	345.1342
**alpha-(4-Fluorophenyl)-4-(5-fluoro-2-pyrimidinyl)**	4.169_348.	POS	4.169	348.1759
**Mdpbp**	6.324_261.	POS	6.324	261.1364
**AB-MECA**	4.692_399.	NEG	4.692	399.1643
**CI-1044**	6.471_397.	POS	6.471	397.1557
**1-(3,4-Dihydroxyphenyl)-1-decene-3,5-dione;**	6.247_276.	POS	6.247	276.1362
**Histidinyl-Lysine**	4.746_283.	POS	4.746	283.1645
**1-Nitrohydroxyphenyl-N-benzoylalanine**	4.888_330.	POS	4.888	330.085
**Lipoamide**	6.095_205.	POS	6.095	205.0598
**Losoxantrone**	6.707_425.	NEG	6.707	425.205
**Scutellarein 6,7,4'-trimethyl ether 5-(6'''-acetylgluc**	5.712_692.	POS	5.712	692.233
**1H-Indene-2-carboxylic acid, 1-(1,3-benzodioxol-!**	6.301_520.	POS	6.301	520.172
**1-Phenyl-2-benzylaminopropane**	9.02_225.1	POS	9.02	225.1519
**PG(15:1(9Z)/22:4(7Z,10Z,13Z,16Z))**	4.932_782.	POS	4.932	782.5134
**PA(15:1(9Z)/0:0)**	6.501_394.	POS	6.501	394.2102
**Lanceotoxin B;**	6.311_604.	POS	6.311	604.2907
**1-(O-alpha-D-galactopyranosyl)-3-keto-(1,27R,29**	10.514_64	POS	10.514	646.5052
**N-palmitoyl taurine**	9.628_363.	NEG	9.628	363.2443
**epsilon-(Hexanoyl)lysine**	4.452_244.	POS	4.452	244.1787
**Alpha-Ketooctanoic acid**	3.288_176.	POS	3.288	176.1052
**Medicagenic acid 3-O-b-D-glucuronide**	6.543_678.	POS	6.543	678.3582
**Urocanate**	0.744_138.	POS	0.744	138.0431
**Leupeptin**	8.448_426.	NEG	8.448	426.2965

**Bisnorbiotin**	0.735_216.	NEG	0.735	216.0569
**5-[5-hydroxy-3-(hydroxymethyl)pentyl]-8a-(hydro**	8.153_371.	POS	8.153	371.2672
**N6-Monobutyryl-cAMP**	4.159_399.	POS	4.159	399.0931
**Polyporenic acid C**	9.71_482.3	POS	9.71	482.3402
Other				
**N-(3-(Dimethylamino)propyl)acrylamide**	2.996_156.	POS	2.996	156.1265
**17-dimethylarsinoyl-9Z-heptadecenoic acid**	2.483_388.	POS	2.483	388.196
Faecalicoccus_acidiformans				
**ZOPOLRESTAT**	4.429_419.	POS	4.429	419.0544
**N-Acetylornithine**	0.739_174.	NEG	0.739	174.1005
**Lumefantrine**	5.105_527.	NEG	5.105	527.1567
**Phloroglucinol**	5.921_126.	NEG	5.921	126.0317
**Brominated Phosphatidylcholine mz 2+ C24H47BrN**	4.65_589.2	POS	4.65	589.2184
**4-Hydroxyquinoline**	4.205_145.	POS	4.205	145.0529
**3-bromohexadecanoic acid**	6.062_334.	POS	6.062	334.1491
**Clofop-isobutyl**	6.532_348.	POS	6.532	348.1144
**Pyraclonil;**	3.614_314.	POS	3.614	314.1048
**2_Methyl_4_pentenoic acid**	3.546_114.	POS	3.546	114.0682
**Xeniasterol-a**	10.206_49:	NEG	10.206	492.3813
**Scandinone**	6.232_418.	POS	6.232	418.1777
**Oxypyrithiamine; 1-(4-Hydroxy-2-methylpyrimid-!**	4.93_260.1	POS	4.93	260.14
**CAPERATIC ACID**	2.945_424.	POS	2.945	424.2433
**Eplivanserin**	6.136_328.	POS	6.136	328.1584
Helicobacter_fennelliae				
**Benzonitrile, 3-amino-4-((2-((dimethylamino)meth**	0.748_283.	POS	0.748	283.1142
**NCGC00380990-01!(2E,4E)-12-hydroxy-13-(hydrox**	5.585_394.	POS	5.585	394.1739
**(1R)-4-[18-[(1R,4R)-4-Hydroxy-2,6,6-trimethylcycl**	10.656_56	POS	10.656	568.4284
**Petroraspailyne B2**	9.695_268.	POS	9.695	268.2038
**7H-Benzimidazo(2,1-a)benz(de)isoquinolin-7-one**	2.264_270.	POS	2.264	270.0787
**(+)-2,7-Dideoxypancratistatin;**	5.709_293.	POS	5.709	293.0899
**Sorafenib N-oxide**	6.762_480.	POS	6.762	480.0799
**Palmitoyl Serinol**	9.722_329.	POS	9.722	329.293
**NVE_274.1802_16.6**	3.64_273.1	POS	3.64	273.1728
**Ser-Tyr**	3.872_268.	POS	3.872	268.1059
**2-[(3alpha,7alpha,12alpha-Trihydroxy-24-oxochola**	8.257_515.	POS	8.257	515.2926
**1D-1-Guanidino-1-deoxy-3-dehydro-scyllo-inosit**	0.663_219.	NEG	0.663	219.0854
**4-hydroxy-7-methyl-2-[2-[3,4,5-trihydroxy-6-(hy**	6.282_438.	NEG	6.282	438.1523
**7,8-Dihydroneopterin**	4.463_255.	POS	4.463	255.0932
**Artemetin 5-glucosyl-(1->4)-rhamnoside**	5.794_696.	POS	5.794	696.2253
**ascr#13**	6.496_288.	POS	6.496	288.1572
**6-Methoxy-2-naphthylacetic acid**	6.524_711.	POS	6.524	711.2318
**Clopenthixol decanoate**	6.544_554.	POS	6.544	554.2743
**2-Hydroxyterephthalic acid**	3.405_182.	NEG	3.405	182.0216
**Omeprazole**	5.622_345.	POS	5.622	345.1146
**Tensyuic acid C**	5.11_272.1	POS	5.11	272.1259
**20R-Camptothecin**	0.758_348.	POS	0.758	348.1104
**Epiyangambin**	7.474_446.	POS	7.474	446.1944
**3,5-dihydroxy-2-(4-hydroxyphenyl)-7-[3,4,5-trihy**	4.293_450.	POS	4.293	450.1167
**Formylmethionyl-leucyl-phenylalanine methyl este**	5.822_451.	POS	5.822	451.2143
**3-Thiacytidine**	0.736_260.	NEG	0.736	260.0464
**Altanserin**	5.512_411.	POS	5.512	411.1433
Other				
**ethyl 2-(2-acetyl-3,5-dihydroxyphenyl)acetate**	3.481_238.	NEG	3.481	238.084
**APC**	6.307_618.	POS	6.307	618.2685
**PGE2-dihydroxypropanylamine**	7.242_425.	POS	7.242	425.2783
**(2R,3S,4S,5R,6S)-2-[(3,4,5-trihydroxyoxan-2-yl)oxy**	3.353_495.	POS	3.353	495.197
**C17 Sphinganine**	7.49_287.2	POS	7.49	287.2823
**NCGC00385815-01!(2S)-8-[(E)-3-hydroxy-3-meth**	6.158_390.	POS	6.158	390.1427

**3,10a-Dihydrobenzo[a]pyrene**	3.781_254.	POS	3.781	254.1094
**Iloprost**	6.006_478.	POS	6.006	478.2717
**Phenol, 2-(2H-benzotriazol-2-yl)-4,6-bis(1,1-dime**	10.638_35	POS	10.638	351.231
**N7-Methylguanosine 5'-diphosphate; N7-Methyl-(**	2.994_458.	POS	2.994	458.05
**PUROMYCIN HYDROCHLORIDE**	4.015_581.	POS	4.015	581.1339
**N-palmitoyl dopamine**	8.023_389.	POS	8.023	389.293
**Mycinamicin VI;**	7.931_667.	POS	7.931	667.3938
**alpha-Acetyllysine**	0.735_188.	NEG	0.735	188.1161
**Isoforskolin**	7.45_410.2	POS	7.45	410.2304
**1-(2-(4-(3-Phenyl-2H-1-benzopyran-2-yl)phenox**	7.454_411.	POS	7.454	411.2191
**Mopidamol**	3.006_421.	POS	3.006	421.2427
**hydroxymatairesinol**	5.458_374.	POS	5.458	374.1366
**Munjistin**	6.306_284.	NEG	6.306	284.0319
**2-(2-Aminopropanylamino)-4-carbamoylbutyric a**	0.706_217.	NEG	0.706	217.106
**Cissoic acid**	5.637_430.	POS	5.637	430.1851
**phenyl [1-(N-succinylamino)pentyl]phosphonate**	4.048_343.	POS	4.048	343.1201
**alpha-Hydroxymetoprolol**	7.259_283.	POS	7.259	283.1782
**2,4(1H,3H)-Pyrimidinedione, 5-fluoro-1-(tetrahydr**	5.815_200.	POS	5.815	200.0589
**Quercetagetin 7-(6''-isobutyrylglucoside)**	6.507_550.	POS	6.507	550.1331
**Oxaziclomefone;**	6.337_375.	POS	6.337	375.078
**kainic acid**	4.945_213.	POS	4.945	213.1003
**Cinitapride**	6.523_402.	POS	6.523	402.225
**1,3-Diphenylpropane;**	8.009_196.	POS	8.009	196.1254
**N-Acetyl-L-carnosine**	7.979_268.	POS	7.979	268.1212
**Amperozide**	4.424_401.	POS	4.424	401.2259
**6alpha-Fluoro-17-hydroxypregn-4-ene-3,20-dion**	7.733_390.	POS	7.733	390.2188
**EI-1625-2**	6.8_496.22	POS	6.8	496.2214
**Lobetyolin**	6.52_396.1	POS	6.52	396.1784
Other				
**4alpha-Formylergosta-7,24(24(1))-dien-3beta-ol;**	8.867_426.	POS	8.867	426.3492
**PG(20:1(11Z)/0:0)**	9.888_538.	POS	9.888	538.328
**Haloperidol glucuronide**	6.351_551.	POS	6.351	551.1695
**Quercetin 3-(6''-acetylglucoside)**	6.322_506.	POS	6.322	506.1061
**2-(hydroxymethyl)-6-[5-[3-(hydroxymethyl)-5-(3-**	4.707_539.	POS	4.707	539.2392
**4-(7-Hydroxy-6,7-dihydro-5H-cyclopenta[d]pyrim**	6.037_252.	POS	6.037	252.1017
**Trilobolide;**	6.119_522.	POS	6.119	522.247
**7-Methylxanthosine;**	5.314_299.	NEG	5.314	299.1005
**Synaptolepis factor K1;**	10.458_61	POS	10.458	614.3793
**I-Urobilinogen; Mesobilirubinogen; Urobilinogen;**	7.879_592.	POS	7.879	592.3279
**LMGP06020021**	10.263_79	POS	10.263	796.5447
**Diflucortolone valerate**	9.669_478.	POS	9.669	478.2539
**Esculentic acid (Diplazium)**	9.656_488.	POS	9.656	488.3511
**(2-Butylbenzofuran-3-yl)(4-hydroxy-3,5-diiodoph**	9.696_545.	POS	9.696	545.9197
**Prasugrel**	5.147_373.	NEG	5.147	373.1163
**LMSL05000004**	7.434_620.	POS	7.434	620.4142
**(+/-)5(6)-DiHET**	7.691_355.	POS	7.691	355.2723
**Butyrylcarnitine**	6.611_231.	POS	6.611	231.1469
**Anandamide (20:1, n-9)**	9.708_353.	POS	9.708	353.3293
**Sodium tauroursodeoxycholate**	11.507_52	POS	11.507	521.2795
Lactobacillus_taiwanensis				
**(2R,4S)-4-Carbamimidamido-3-acetamido-2-((1R,**	4.832_472.	POS	4.832	472.2525
**1-Pyrrolidinecarboxaldehyde, 3-(1,3-benzodioxol-**	6.389_245.	POS	6.389	245.0689
**1-[2,3-Dimethyl-2-(2-methylbut-3-en-2-yl)furan-**	6.863_326.	POS	6.863	326.2092
**18-Hydroxyoctadecadienoic acid**	8.422_296.	POS	8.422	296.235
**Indicine**	3.2_299.17	POS	3.2	299.1734
**gamma-Glutamylleucine**	0.735_260.	NEG	0.735	260.1368
**Dodecanamide**	8.728_199.	POS	8.728	199.1939
**3-Hydroxyvaleric acid**	0.789_118.	NEG	0.789	118.063

**N-(2-((6-Chloro-3-(3-(N,N-dimethylsulfamoyl)phe**	3.794_450.	POS	3.794	450.1246
Bifidobacterium_pseudolongum				
**[(2R)-2-[(E,2S,4R)-4,6-dimethyloct-6-en-2-yl]-6-o**	8.317_452.	POS	8.317	452.2777
**Evasterioside B**	6.736_662.	POS	6.736	662.3319
**AK-toxin I;**	7.566_413.	NEG	7.566	413.1854
**Bromadiolone;**	5.589_526.	NEG	5.589	526.0773
**(2R,3S,4S,5R,6R)-2-(hydroxymethyl)-6-[[(2R,3S,4S,!**	4.413_491.	POS	4.413	491.2022
**Azepine**	5.678_93.0	POS	5.678	93.05797
**(-)-alpha-Tocopherol**	9.852_430.	POS	9.852	430.3813
**Apogossypol**	8.67_462.2	POS	8.67	462.2034
**(3S)-4-[[(1S)-1-Carboxy-2-phenylethyl]amino]-3-[**	4.29_463.2	POS	4.29	463.2422
**NCGC00380585-01!(E)-3-[1-[(E)-but-2-en-2-yl]-9**	4.737_462.	POS	4.737	462.1295
**3,4-Dihydro-6-[[4-[[[3-(methylsulfonyl)phenyl]me**	6.381_491.	POS	6.381	491.1254
Bacteroides_coprophilus				
**Oleamide**	8.515_281.	POS	8.515	281.2717
**10,11-Dihydro-10,11-dihydroxycarbamazepine**	6.181_270.	POS	6.181	270.1004
**Ambocin**	5.762_564.	NEG	5.762	564.1484
**Upadacitinib**	4.61_380.1	POS	4.61	380.1583
**Saccharin**	3.798_182.	POS	3.798	182.9986
**Validamine 7-phosphate;**	0.715_257.	NEG	0.715	257.0664
**Betamethasone phosphate**	6.447_472.	POS	6.447	472.1677
**Phosmet**	2.954_316.	POS	2.954	316.9959
**3,5,7-Trimethyl-2E,4E,6E,8E-decatetraene**	9.82_176.1	POS	9.82	176.1568
**indoprofen**	7.072_281.	POS	7.072	281.105
**Tridemorph; 2,6-Dimethyl-4-tridecylmorpholine;**	10.112_29	POS	10.112	297.3031
**Docosatetraenylethanolamide**	9.999_375.	POS	9.999	375.3133
**Petroformynic acid C**	9.524_698.	NEG	9.524	698.5259
**V-Pyrro/NO**	0.969_157.	NEG	0.969	157.0852
**[(3S,8R,9S,10S,13S,14S)-10,13-Dimethyl-17-oxo-1,**	6.447_370.	POS	6.447	370.1824
**DGMG(18:5(3Z,6Z,9Z,12Z,15Z)/0:0)**	5.117_672.	POS	5.117	672.3332
**(2R,3S,4S,5R,6S)-2-(hydroxymethyl)-6-(4-hydroxy-**	5.764_346.	POS	5.764	346.1628
**Zosuquidar**	3.297_527.	POS	3.297	527.2389
**Ethinyl estradiol sulfate**	4.205_376.	POS	4.205	376.1344
**LMPK12113302**	4.078_698.	NEG	4.078	698.2061
**4-[(5R,8S,10R,13R,17R)-3,6-Dihydroxy-10,13-dime**	9.773_392.	POS	9.773	392.2927
**Flunarizine**	3.482_404.	NEG	3.482	404.2047
**4-Hydroxyphenylacetylglutamic acid; p-Hydroxyph**	3.58_281.0	POS	3.58	281.09
Pseudomonas_fulva				
**DG(18:1(9Z)/18:3(9Z,12Z,15Z)/0:0)**	9.734_616.	POS	9.734	616.5043
**PS(15:0/20:5(5Z,8Z,11Z,14Z,17Z))**	6.537_767.	POS	6.537	767.4713
**Propionylcarnitine**	1.216_217.	POS	1.216	217.1317
**Glycerophosphoglycerol;**	0.685_246.	NEG	0.685	246.0504
**N-{4-[(3,5-Difluorophenyl)sulfonyl]benzyl}imidazo**	6.611_427.	POS	6.611	427.0813
**4-[[2-[(E)-1-hydroxybut-2-enyl]-5-oxooxolan-3-y**	3.286_308.	POS	3.286	308.1371
**Pentanamide, N-(cyanomethyl)-4-methyl-2-(((1S)-**	4.812_481.	POS	4.812	481.1637
**Iodosulfuron-methyl-sodium; Iodosulfuron-methy**	6.58_529.2	POS	6.58	529.2432
**PS(O-16:0/17:1(9Z))**	10.592_73	POS	10.592	733.5284
**Furametpyr;**	6.318_333.	POS	6.318	333.123
**Darusentan**	6.021_410.	POS	6.021	410.1479
**Phomalone**	4.587_254.	NEG	4.587	254.1154
**2-Oxoclopidogrel**	6.322_337.	POS	6.322	337.0538
**Ethyl (2S,3S)-3-[[(2S)-1-[[(2S)-1-hydroxypent-4-yı**	5.136_354.	NEG	5.136	354.1791
**Saxitoxin;**	7.368_299.	POS	7.368	299.1349
**Samidorphan**	6.53_370.1	POS	6.53	370.1888
**Dalpanol O-glucoside**	6.103_574.	POS	6.103	574.2022
**1,2-Dehydroreticuline; 1,2-Dehydroreticulinium; 3,**	6.134_328.	POS	6.134	328.1551
Veillonella_dispar				
**Tutin**	7.452_294.	POS	7.452	294.1103

**N-palmitoyl glycine**	9.701_313.	NEG	9.701	313.2617
**2,3,5,6-Tetramethylpyrazine phosphate**	4.713_228.	POS	4.713	228.0311
**4-Acetamidobenzenesulfonamide**	5.583_214.	POS	5.583	214.0414
**PKOHA-PG**	5.994_622.	POS	5.994	622.3144
**Pyraclostrobin**	3.639_387.	NEG	3.639	387.099
**N-Propionylprocainamide**	4.014_291.	POS	4.014	291.1945
**PS(18:4(6Z,9Z,12Z,15Z)/0:0)**	6.519_517.	POS	6.519	517.243
**N4-Acetylcytidine**	2.771_285.	POS	2.771	285.096
**Sedanolide**	9.623_176.	POS	9.623	176.1204
**Ethyl 3-hydroxypropanoate**	3.129_118.	POS	3.129	118.0631
**S-Nitrosocaptopril**	3.004_246.	POS	3.004	246.0676
Myroides_injenensis				
Sanguibacter_inulinus				
Cellulomonas_pakistanensis				
Rhizobium_subbaraonis				
Psychrobacter_cibarius				
Neorhizobium_galegae				
Rhizobium_yantingense				
Sanguibacter_antarcticus				
Brevundimonas_terrae				
**Cycloheximide**	4.142_281.	POS	4.142	281.1626
Coprococcus_comes				
**methyl 8-[2-(2-formyl-vinyl)-3-hydroxy-5-oxo-cy**	8.396_310.	POS	8.396	310.1786
Butyricicoccus_pullicaecorum				
Other				
**(5S)-5-hydroxy-1,7-diphenylheptan-3-one**	9.821_282.	POS	9.821	282.1619
**Uplandicine; 7-Acetyl-9-echimidinylretronecine;**	6.087_357.	POS	6.087	357.1786
Leucobacter_aridicollis				
**Garomefrine**	0.641_248.	NEG	0.641	248.0642
**Tricosanal**	12.562_33	POS	12.562	338.3548
**LMPK12113326**	6.518_470.	POS	6.518	470.158
**2-Amino-4-methylsulfanylbutanal**	2.775_133.	NEG	2.775	133.0562
**Genistein 4'-glucuronide-7-sulfate**	3.274_526.	NEG	3.274	526.0414
**18-fluoro-9Z,12Z-octadecadienoic acid**	6.82_298.2	POS	6.82	298.2321
**Indole-3-butyric acid**	3.6_203.09	NEG	3.6	203.0946
**1-(2-Azabicyclo[2.2.2]octan-2-yl)-2-[2-(3,5-dimetl**	7.168_562.	POS	7.168	562.3654
**Cimiaserin B**	9.603_488.	POS	9.603	488.3486
**3-((Aminooxy)methyl)phenol**	3.433_139.	POS	3.433	139.0636
Collinsella_stercoris				
**Cholesteryl hemisuccinate**	9.795_486.	POS	9.795	486.3715
**Thienamycin**	1.024_272.	NEG	1.024	272.0828
**N-acetylneuraminate**	0.666_309.	NEG	0.666	309.1058
**Phenanthrene**	4.095_178.	NEG	4.095	178.0791
**Narcotine hemiacetal;**	6.181_415.	POS	6.181	415.1636
**Melibiose**	0.716_342.	NEG	0.716	342.1163
**Proglumetacin**	6.29_843.3	POS	6.29	843.3973
**5-(4-Acetoxybut-1-ynyl)-2,2'-bithiophene;**	0.609_276.	NEG	0.609	276.0286
**2,4-bis(3-methylbut-2-enyl)-5-(2-phenylethyl)ben**	7.198_372.	POS	7.198	372.2048
**Verapamil metabolite D-617**	5.308_290.	NEG	5.308	290.1993
**Cyclothiazide**	0.743_389.	NEG	0.743	389.0286
**9-hydroxy-5Z-nonenoic acid**	4.554_172.	NEG	4.554	172.11
**2,3-Dihydro-6-(3-(2-hydroxymethyl)phenyl-2-pro**	7.572_282.	POS	7.572	282.1255
**Medroxyprogesterone**	9.373_344.	NEG	9.373	344.2352
**C17 Sphingosine-1-phosphate**	6.231_365.	POS	6.231	365.2314
**14-hydroxy-14-(hydroxymethyl)-5,9-dimethyltetra**	7.63_374.1	POS	7.63	374.1842
**Liquiritin apioside**	5.174_550.	NEG	5.174	550.1677
**Deacetylasperulosidic acid**	3.948_390.	POS	3.948	390.115
**Endostatin**	4.788_178.	POS	4.788	178.0744

**10-Hydroxy-2-decenoic acid**	6.741_186.	POS	6.741	186.1258
**(S)-5-(4-Hydroxy-4-methylisoxazolidine-2-carbon**	5.183_515.	NEG	5.183	515.1458
**Pentacyclo(5.4.0.02,6.03,10.05,9)undecane**	8.473_146.	POS	8.473	146.1097
**carviolin**	7.602_300.	POS	7.602	300.0632
**Promethazine sulfoxide**	4.589_300.	POS	4.589	300.1295
**triglyceride palmitate**	12.959_51	POS	12.959	518.2714
**NCGC00347750-02!(E,2S,3R,4R,5S)-4-acetyloxy-2-**	7.711_395.	POS	7.711	395.2672
**8-(2,4-Dichlorophenyl)-N-(1,3-dimethoxypropan-**	4.926_409.	POS	4.926	409.1073
**Kurilensoside F**	9.344_630.	POS	9.344	630.3982
**6beta-Hydroxydexbudesonide**	6.828_446.	POS	6.828	446.23
**(8S,9R,10S,13S,14S)-10,13-Dimethyl-2,3,4,5,6,7,8,9,**	7.62_288.2	POS	7.62	288.2089
**Estreptoquinasa**	5.795_197.	POS	5.795	197.1419
**15beta-Hydroxydesogestrel**	6.543_326.	POS	6.543	326.2246
**Bottromycin A2 Acid**	9.661_808.	POS	9.661	808.4251
**7-[(2-Benzyl-3-sulfanylpropanoyl)amino]heptanoi**	9.677_323.	POS	9.677	323.1546
**Mesoridazine**	6.524_386.	POS	6.524	386.1477
**Linoleic acid**	8.721_278.	POS	8.721	278.2246
**Sphinganine**	8.81_301.2	POS	8.81	301.2981
**5'-Hydroxytenoxicam**	0.787_353.	NEG	0.787	353.0133
**Bemcentinib**	7.507_506.	POS	7.507	506.2884
**Fura-2 acetoxymethyl**	4.296_713.	POS	4.296	713.1728
Spongiispira_norvegica				
Other				
**senkyunolide I**	5.709_224.	POS	5.709	224.1051
**p-Cresylsulfate**	4.076_188.	NEG	4.076	188.0143
**Phenacemide**	5.067_178.	NEG	5.067	178.0743
**Benzamide, 4-chloro-N-(2-(4-morpholinyl)ethyl)-,**	0.738_284.	POS	0.738	284.0941
Brachyspira_aalborgi				
Abiotrophia_defectiva				
**TRIETHYLENE GLYCOL**	2.396_150.	POS	2.396	150.0894
**3-Nitro-3,3a-dihydrobenzo[b]phenalen-7-one**	2.295_277.	POS	2.295	277.073
**Daidzein diglucuronide**	6.447_606.	POS	6.447	606.1229
**(alphaS,betaS)-alpha-Ethyl-alpha-(4-methoxypher**	3.685_333.	POS	3.685	333.1722
**N,N-Diethyl-2-(2-(4-methoxyphenyl)-5,7-dimethy**	7.585_366.	POS	7.585	366.2043
**Genistein**	6.429_270.	NEG	6.429	270.0525
**2-(Glutathione-S-yl)-hydroquinone;**	6.439_415.	POS	6.439	415.1034
**Deoxythioguanosine**	3.017_283.	POS	3.017	283.0726
**Cer(t18:1(6OH)/22:0(2OH))**	11.58_653.	NEG	11.58	653.5974
**Cannabisin B;**	6.85_596.2	POS	6.85	596.2172
**Pantothenic acid**	3.048_219.	POS	3.048	219.1108
**Carfentrazone-ethyl**	4.539_411.	POS	4.539	411.0376
**Latanoprost**	7.592_432.	POS	7.592	432.2883
**3-Methylglutarylcarnitine**	2.671_289.	POS	2.671	289.1524
**LMPK12010141**	4.093_727.	NEG	4.093	727.1848
Escherichia				
**Croconazole;**	0.903_310.	NEG	0.903	310.0864
**1-dimethylarsinoyl-(3Z,6Z,9Z,12Z,15Z,18Z)-docosa**	6.793_418.	POS	6.793	418.2203
**2-Oxo-10-methylthiodecanoic acid; 10-Methylthio**	6.402_232.	POS	6.402	232.1133
**Navarixin**	5.171_397.	POS	5.171	397.1639
**1-Hydroxyvitamin D5**	10.704_42	POS	10.704	428.3656
**2-(2-hydroxy-6-methylhept-5-en-2-yl)-7-(3-metl**	7.724_376.	POS	7.724	376.2247
**S-Adenosylmethioninamine**	6.174_355.	POS	6.174	355.1566
**Dexamethasone palmitate**	9.917_630.	POS	9.917	630.4277
**Difucol Hexamethyl Ether**	4.078_334.	POS	4.078	334.143
**Potassium dibasic phosphate;**	0.715_173.	POS	0.715	173.8889
**Sitagliptin**	6.562_407.	POS	6.562	407.115
**StemRegenin 1**	6.088_429.	POS	6.088	429.1637
**PS(P-20:0/16:0)**	10.507_77	POS	10.507	775.5746

**Thioapfelsaure**	2.35_134.0	POS	2.35	134.0037
**Thionazin;**	0.747_248.	POS	0.747	248.0385
**2,4,6-Octatriyn-1-ol**	1.177_118.	POS	1.177	118.0421
**Trifluperidol**	5.931_409.	POS	5.931	409.167
**Flecainide**	10.043_41	POS	10.043	414.1368
**alpha-Tocopheryloxyacetic acid**	9.803_488.	POS	9.803	488.387
**Vicenistatin;**	5.615_500.	POS	5.615	500.3622
**Simeconazole;**	6.795_293.	POS	6.795	293.1374
**5-Hydroxyisourate;**	1.008_184.	POS	1.008	184.0235
**Cer(t18:0/16:0)**	9.795_555.	POS	9.795	555.5232
**Busulfan**	0.84_246.0	POS	0.84	246.0227
**Paraxanthine**	3.624_180.	POS	3.624	180.0651
**Pentaerythritol mononitrate**	5.378_181.	POS	5.378	181.058
**3-Butyl-1-(hydroxymethyl)-1-(4-methylphenyl)sul**	6.834_300.	POS	6.834	300.1134
**Sophoracoumestan B**	0.658_326.	POS	0.658	326.0442
Other				
**10-Acetoxy-10,11-dihydro-5h-dibenz[b,f]azepine-**	7.522_296.	POS	7.522	296.116
**thiadiazinone**	1.55_320.0	POS	1.55	320.079
**Hydroiodic acid; Hydrogen iodide;**	0.696_127.	NEG	0.696	127.9124
**5-(4-Methoxybenzylidene)-1,3-thiazolidine-2,4-di**	1.807_235.	POS	1.807	235.0301
**Chlorphentermine**	0.802_183.	POS	0.802	183.0808
Brevundimonas_nasdae				
**Erythrocentaurin**	5.105_176.	POS	5.105	176.0476
**Diethyltoluamide**	6.891_191.	POS	6.891	191.1314
**1alpha,5alpha-Dimercaptoandrostane-3alpha,17be**	6.348_356.	POS	6.348	356.183
**2-(2-Methoxyphenyl)-1-(1-pentylindol-3-yl)ethan**	6.283_335.	POS	6.283	335.1883
**N-Acetylmuramate; N-Acetylmuramic acid; N-Acet**	0.789_293.	POS	0.789	293.111
**1,2,3,10-Tetramethoxy-7-(methylamino)-6,7-dihyc**	4.301_371.	POS	4.301	371.1732
Prevotella_multisaccharivorax				
**S-(4-Methylthiobutylthiohydroximoyl)-L-cysteine;**	4.862_252.	POS	4.862	252.061
**3-Methylhistamine**	0.599_125.	POS	0.599	125.0955
**chaetoglobosins**	6.105_566.	POS	6.105	566.2161
**3-Hydroxysuberic acid**	3.744_190.	POS	3.744	190.0843
**10-(Phosphonooxy)decyl methacrylate**	4.285_322.	POS	4.285	322.1531
Centipeda_periodontii				
**3-Methoxy-N-p-tolylquinoxalin-2-carboxamide**	3.726_293.	POS	3.726	293.1172
**(2S,3R,5S,9R)-2,3-dihydroxy-14-(hydroxymethyl)-5**	7.24_530.2	NEG	7.24	530.2739
**(2-Chlorophenyl)diphenylmethane**	0.821_278.	POS	0.821	278.0862
**Almitrine**	6.455_477.	POS	6.455	477.2472
**3-(2-Chlorophenyl)-N-(1-(3-methoxyphenyl)ethyl**	5.527_303.	POS	5.527	303.1402
**altenusin**	5.768_307.	POS	5.768	307.1056
**Acronycidine; Furo(2,3-b)quinoline, 4,5,7,8-tetrame**	5.384_289.	POS	5.384	289.095
**Garenoxacin**	5.791_426.	POS	5.791	426.1405
**12-Hydroxynevirapine glucuronide**	3.595_458.	NEG	3.595	458.1424
**(2S)-2-(2-Benzamidoethylamino)-5-(diaminometh**	2.476_321.	POS	2.476	321.18
**4-[2-(3,4-Dihydro-6,7-dimethoxy-4-methyl-3-oxo**	6.307_345.	POS	6.307	345.1069
**d-Glycero-d-galacto-heptose**	0.688_210.	NEG	0.688	210.0738
**Sulforaphane-cysteine**	4.609_298.	POS	4.609	298.0479
**NCGC00380830-01!methyl 2-[(2-hydroxy-3-methy**	2.837_231.	POS	2.837	231.1471
**D-Cloprostenol**	6.519_424.	POS	6.519	424.1634
**Lerisetron**	8.502_292.	NEG	8.502	292.1674
**Jadomycin A; Jadomycin B aglycon;**	2.972_419.	POS	2.972	419.1361
**(5R,6S)-6-[(1R)-1-Hydroxyethyl]-7-oxo-3-pyridin-**	5.006_292.	NEG	5.006	292.0517
**shogaol**	6.829_314.	POS	6.829	314.1292
**7-Chlorokynurenic acid**	0.699_223.	NEG	0.699	223.0043
**PA(14:0/0:0)**	5.85_382.2	NEG	5.85	382.2105
**(E)-1-(2,4-dihydroxyphenyl)-3-[4-(3-methylbut-2-**	6.192_346.	POS	6.192	346.1165
**Maritimetin 6-O-(3'',4'',6''-tri-O-acetylglucoside)**	6.832_574.	POS	6.832	574.1323

**Irisolidone 7-O-glucuronide**	6.864_490.	POS	6.864	490.1112
**5-oxo-6E,8Z-tetradecadienoic acid**	9.34_238.1	POS	9.34	238.157
**2-Methoxyresorcinol**	4.389_140.	POS	4.389	140.0476
**2-(1H-Indazol-3-yl)acetic acid**	4.45_176.0	NEG	4.45	176.0586
**(4E,10E,12E)-nonadec-4,10,12-trien-6,8-diyn-2-on**	6.474_268.	POS	6.474	268.1827
**6-Fluoro-1-methyl-1,4-dihydro-7-(1-piperazinyl)-**	6.384_305.	NEG	6.384	305.1164
**Hydrocinnamic acid**	9.022_150.	POS	9.022	150.0683
**Kynurenic acid**	3.055_189.	NEG	3.055	189.0427
**Naringenin 7-O-beta-D-glucoside 6''-acetate**	6.56_476.1	POS	6.56	476.1319
**N-Formyl-Met-Leu-Phe**	6.718_437.	NEG	6.718	437.1985
**4,8-Methano-8aH-bisbenzofuro[3,2-e:2',3'-g]isoq**	6.459_415.	POS	6.459	415.1775
**tephrosin**	6.418_432.	POS	6.418	432.1172
**Glutamine hydroxamate**	0.665_162.	NEG	0.665	162.0642
**Piroxantrone**	6.855_411.	POS	6.855	411.1907
**LMPK12140637**	3.343_362.	POS	3.343	362.1005
Other				
**(2E,4E)-1-(Pyrrolidin-1-yl)deca-2,4-dien-1-one**	6.244_221.	POS	6.244	221.1782
**Calix[6]arene**	6.359_636.	POS	6.359	636.2538
**Canagliflozin**	6.43_444.1	POS	6.43	444.1426
**PS(O-18:0/22:6(4Z,7Z,10Z,13Z,16Z,19Z))**	10.359_82	POS	10.359	821.5573
**LysoPC(20:1(11Z)/0:0)**	9.926_549.	POS	9.926	549.3805
**(2S,3S,4R,5R)-5-(6-Aminopurin-9-yl)-N-cycloprop**	4.708_320.	POS	4.708	320.1233
**alpha-D-Galactosyl-(1,1')-sn-glycerol 3-phosphate**	0.641_334.	NEG	0.641	334.0664
**8-epi-11-Hydroxyiridodial glucoside pentaacetate;**	5.652_556.	POS	5.652	556.2171
**Lidocaine N-oxide**	4.557_250.	POS	4.557	250.1682
Cytophaga_aurantiaca				
Other				
Thermomonas_carbonis				
Other				
Vampirovibrio_chlorellavorus				
Trichococcus_collinsii				
Propionibacterium_granulosum				
Tepidimonas_ignava				
Other				
Paenibacillus_timonensis				
Roseomonas_aerophila				
Other				
Modestobacter_versicolor				
Algoriphagus_terrigena				
Chryseobacterium_gambrini				
Cnuella_takakiae				
Sphingopyxis_bauzanensis				
Other				
Other				
Other				
Corynebacterium_lactis				
Hymenobacter_ocellatus				
Xanthobacter_flavus				
Phenylobacterium_muchangponense				
Other				
Burkholderia_multivorans				
Acinetobacter_soli				
Other				
Other				
Anaerococcus_octavius				
Cloacibacillus_porcorum				
Mucilaginibacter_defluvii				
Dyadobacter_hamtensis				

Aquisphaera_giovannonii				
Devosia_crocina				
Ottowia_thiooxydans				
Paenibacillus_rigui				
Cohnella_rhizosphaerae				
Sphingomonas_oryziterrae				
Dietzia_papillomatosis				
Corynebacterium_glucuronolyticum				
Gemmobacter_tilapiae				
Lactobacillus_iners				
Blastococcus_aggregatus				
Pseudomonas_zhaodongensis				
Tatumella_punctata				
Jeotgalicoccus_psychrophilus				
Lactobacillus_paralimentarius				
Rheinheimera_aquatica				
Pseudolabrys_taiwanensis				
Other				
Other				
Methylobacterium_iners				
Planococcus_salinarum				
Luteococcus_sanguinis				
Kaistia_hirudinis				
Eggerthia_catenaformis				
Bdellovibrio_bacteriovorus				
Nocardioides_kribbensis				
Gemmobacter_aquaticus				
**Hydroxy-lacosamide**	4.162_282.	POS	4.162	282.1215
**isorhapontin**	4.702_442.	POS	4.702	442.1235
**Aleuretic Acid**	10.065_30	POS	10.065	304.2255
**N-jasmonoyl-L-dopa**	6.101_389.	POS	6.101	389.1833
**2-(2,4-Dimethylphenyl)indan-1,3-dione**	3.318_250.	NEG	3.318	250.0986
**Pinolenic acid**	7.861_278.	POS	7.861	278.2246
**beta-Apopolygamatin**	2.721_366.	POS	2.721	366.1097
**Chloramphenicol glucuronide**	6.388_498.	NEG	6.388	498.0463
**Prizidilol**	6.768_331.	POS	6.768	331.1993
**(3S,8As)-7-hydroxy-3-(hydroxymethyl)-2,3,6,7,8,8a**	1.102_200.	POS	1.102	200.0802
**Triptophenolide**	5.604_334.	POS	5.604	334.1528
**2-Amino-4-(amino-methyl-oxo-lambda6-sulfanyli**	7.523_180.	POS	7.523	180.0575
**Guanosine**	1.828_283.	NEG	1.828	283.0914
Psychrobacter_alimentarius				
**Glucaron**	3.006_258.	POS	3.006	258.0371
**(2R,5S)-2-(6-Aminopurin-9-yl)-5-(methylsulfanylm**	0.749_297.	POS	0.749	297.0898
**N-(3-(3-Hydroxy-4-methoxyphenyl)propyl)-L-alpl**	6.052_444.	POS	6.052	444.1897
**Benodanil;**	0.802_322.	POS	0.802	322.9816
**Acrovestone;**	6.876_554.	POS	6.876	554.2892
**NCGC00380990-01!(2E,4E)-12-hydroxy-13-(hydro**	7.47_338.2	POS	7.47	338.2096
**[(2S,3R,4S,5S,6R)-3,4,5-trihydroxy-6-(hydroxymeth**	6.473_506.	POS	6.473	506.2173
Brevibacterium_pityocampae				
**PG(17:2(9Z,12Z)/0:0)**	6.166_494.	POS	6.166	494.2668
**PE(0:0/20:4;O)**	6.546_517.	POS	6.546	517.2793
**2-Amino-3-(2-fluoro-3,4-dihydroxyphenyl)propan**	4.682_215.	POS	4.682	215.0594
**9-Thiocyanato-androst-4-ene-3,11,17-trione;**	7.371_357.	POS	7.371	357.1402
**3-methylquercetin**	7.473_316.	POS	7.473	316.0584
**N-(2,5-Dimethoxybenzyl)-N-(5-fluoro-2-phenoxy**	6.044_395.	POS	6.044	395.1518
**NCGC00384737-01!1-(3,4-dimethoxyphenyl)-2-(2-**	4.688_378.	POS	4.688	378.1462
**N-Acetyldopamine**	3.271_195.	POS	3.271	195.0897
**Cilazapril**	7.4_417.22	POS	7.4	417.2282

**Suberoyl-L-carnitine**	2.412_317.	NEG	2.412	317.1838
**Pleconaril**	2.884_381.	POS	2.884	381.1284
Pseudoxanthomonas_japonensis				
**8-acetamido-2-methyl-7-oxononanoic acid**	3.446_243.	POS	3.446	243.1472
**NCGC00381075-01!3-(2,4-dihydroxypentyl)-6,8-di**	4.507_330.	POS	4.507	330.1074
**2-[(1S,2S,4aR,8aS)-1-hydroxy-4a-methyl-8-methy**	9.18_250.1	POS	9.18	250.157
**PS(18:3(6Z,9Z,12Z)/0:0)**	6.659_519.	POS	6.659	519.2589
**Moguisteine**	5.552_339.	POS	5.552	339.114
**Israpafant**	7.142_488.	POS	7.142	488.1803
**<i>para</i>-hydroxyatorvastatin**	7.887_574.	POS	7.887	574.2482
**MGMG(18:4;O/0:0)**	8.347_528.	POS	8.347	528.2948
**N-tetradecanoyl-homoserine lactone**	9.345_311.	NEG	9.345	311.246
**Foropafant**	10.005_46	NEG	10.005	464.2986
**Adtepp**	4.54_599.1	POS	4.54	599.1552
**Tiliacorine;**	7.201_576.	NEG	7.201	576.2609
**1H-Benzotriazole**	4.218_119.	POS	4.218	119.0485
**Aurantio-obtusin beta-D-glucoside;**	6.107_492.	POS	6.107	492.1271
**Atorvastatin lactone**	6.844_540.	POS	6.844	540.2425
**(S)-N-((S)-1-Amino-3,3-dimethyl-1-oxobutan-2-y**	6.838_499.	POS	6.838	499.2818
**NCGC00180673-03!1,12,23-trihydroxy-1,6,12,17,23**	7.214_600.	POS	7.214	600.3462
**20-hydroxyecdysone 22-phosphate**	6.215_560.	POS	6.215	560.2772
Clostridium_difficile				
**PA(22:4(7Z,10Z,13Z,16Z)/0:0)**	6.783_486.	POS	6.783	486.2766
**Aphidicolin**	4.48_360.2	POS	4.48	360.2261
**Fusarenon-X**	6.216_354.	POS	6.216	354.1291
**PHHdiA-PE**	9.909_609.	NEG	9.909	609.3253
**1-Methylxanthine**	2.991_166.	POS	2.991	166.0495
**Picloram**	0.746_239.	NEG	0.746	239.9258
**Apocholic acid**	8.673_407.	POS	8.673	407.3037
**High Temperature Requirement A2 Inhibitor I**	6.548_495.	POS	6.548	495.0876
**Dodecenoylcarnitine**	8.4_341.25	POS	8.4	341.2566
**Cepharanthine**	7.486_606.	POS	7.486	606.27
**(S)-3-{4-[2-(5-Methyl-2-phenyl-oxazol-4-yl)-etho**	5.633_408.	POS	5.633	408.2037
**Linezolid**	3.928_337.	NEG	3.928	337.1454
**Oxibendazole**	3.447_249.	POS	3.447	249.1114
**Egtazic acid**	4.505_380.	POS	4.505	380.1439
**Tetradecadienyl-l-carnitine**	9.236_353.	POS	9.236	353.2929
**1',2'-Dihydro-1',2'-dihydroxy-4-ketotoruelene/ (4-**	9.836_582.	POS	9.836	582.4078
**Phenylethylmalonamide**	4.98_206.1	NEG	4.98	206.1055
**Lamellosterol A**	9.252_516.	POS	9.252	516.3129
**2-decyl-3-hydroxypentanedioic acid**	6.967_288.	POS	6.967	288.1936
**(Z)-p-Methoxycinnamic acid**	7.348_178.	POS	7.348	178.0633
**repirinast**	7.569_355.	POS	7.569	355.1426
Other				
**Alclometasone**	5.617_408.	POS	5.617	408.1713
**5-Chlorodivaricatinic acid**	3.292_244.	POS	3.292	244.0518
**Zanamivir**	6.087_332.	POS	6.087	332.1334
**delta-Valerolactone**	0.725_100.	NEG	0.725	100.0525
**3-Acetylnerbowdine;**	6.086_361.	POS	6.086	361.1521
**cholesteryl beta-D-xyloside**	9.814_518.	POS	9.814	518.3979
**NCGC00179941-03!3-(3,4-dimethoxyphenyl)-1-(2-**	7.46_328.1	POS	7.46	328.1308
**CPA(18:0)**	10.011_42	NEG	10.011	420.264
**[(6E,10Z)-10-(acetyloxymethyl)-6-formyl-3-methy**	5.639_421.	POS	5.639	421.2098
**(2R,3R,4S,5R)-5-Fluoro-2,3,4-trihydroxy-6-oxohex**	5.318_262.	POS	5.318	262.0266
**N-(6-Amino-2-methyl-pyridin-3-ylmethyl)-2-(6-n**	5.715_455.	NEG	5.715	455.1614
**3-Chlorotyrosine**	2.447_215.	POS	2.447	215.035
**CerP(d18:1/2:0)**	6.239_421.	POS	6.239	421.2575
**Eseridine;**	5.321_291.	POS	5.321	291.1583

[4-[3,4,5-trihydroxy-6-(hydroxymethyl)oxan-2-yl](	5.923_511.	POS	5.923	511.2057
Vitamin D2 3-glucuronide	10.179_57.	POS	10.179	572.3717
Fluticasone propimonate	6.489_556.	POS	6.489	556.2124
2,4,4-Trimethyl-3-[3,7,12,16-tetramethyl-18-(2,6,6	10.059_55(	POS	10.059	550.4185
Squafosacin C	10.688_60	POS	10.688	608.5018
5,6-Dihydroxyuracil	0.757_144.	POS	0.757	144.0166
(2s,6as,12as)-6a-hydroxy-8,9-dimethoxy-2-(prop-	6.983_427.	POS	6.983	427.1633
14-HDoHE	9.615_356.	NEG	9.615	356.2353
(Z)-7-[(1R,4S,5R,6R)-5-[(E,3S)-3-Hydroxyoct-1-en	6.961_348.	POS	6.961	348.241
Glycerolphosphate	3.251_183.	POS	3.251	183.977
Ethanethioic acid, S-(2-(((2R)-2-(acetylamino)-3-m	3.367_264.	POS	3.367	264.0602
7-Hydroxymethyl-12-methylbenz[a]anthracene sul	4.125_352.	NEG	4.125	352.0763
Carboxy-ibuprofen	7.854_236.	POS	7.854	236.105
Demethylnobiletin	6.95_388.1	POS	6.95	388.1159
Hexobarbital	2.768_236.	POS	2.768	236.1162
1-Tert-Butyl 4-ethyl 3-oxopiperidine-1,4-dicarbox	6.295_271.	POS	6.295	271.1418
PS(14:0/12:0)	9.643_651.	POS	9.643	651.4115
Shewanella_xiamenensis				
6-Fluoropyridoxol	5.823_187.	POS	5.823	187.0636
p-Synephrine	5.463_167.	POS	5.463	167.0949
Dihydroferulic acid 4-O-sulfate	4.739_276.	POS	4.739	276.0313
11alpha-(fluoromethyl)-1alpha,25-dihydroxyvitam	10.661_44	POS	10.661	448.3344
1,4-Pentadien-3-one	4.267_82.C	POS	4.267	82.04189
(2S,3As,6aS)-1-[(2R)-2-carboxy-2-[[(2S)-1-ethoxy-	3.26_446.2	POS	3.26	446.2054
1-Nonadecanoyl-glycero-3-phosphoethanolamine	3.251_495.	POS	3.251	495.3331
2-(1-hydroxyethyl)-4-(2-hydroxypropyl)-2H-furan	3.287_203.	POS	3.287	203.1162
2-Hydroxy-1,4-benzoxazin-3-one glucuronide	3.25_341.C	POS	3.25	341.0745
Chlorotrifluoroethylene	3.294_115.	POS	3.294	115.9638
Dedimethylchlorpromazine	3.426_290.	POS	3.426	290.0653
Aldicarb	8.51_190.C	POS	8.51	190.0784
Aurin	4.76_290.C	POS	4.76	290.0933
Iophendylate	5.346_416.	POS	5.346	416.1195
N'-[(6-Oxo-5-prop-2-enyl-1-cyclohexa-2,4-dieny	7.017_392.	POS	7.017	392.2199
1,4-Bisdesoxycyadox	5.327_239.	POS	5.327	239.0807
Glaucine	4.584_355.	POS	4.584	355.1783
2-Methoxy-N-[3-[4-[3-methyl-4-[(6-methyl-3-py	8.903_469.	POS	8.903	469.2111
2,4-Dihydroxybenzophenone	6.483_214.	POS	6.483	214.0631
Camptothecin	1.092_348.	POS	1.092	348.1105
N-(3-oxo-pentanoyl)-homoserine lactone	2.971_199.	NEG	2.971	199.0845
Promazine 5-sulfoxide	1.048_300.	NEG	1.048	300.1295
Gabapentin Related Compound E	1.797_186.	NEG	1.797	186.0892
9-hydroxy-10,12-octadecadienoic acid	5.343_334.	POS	5.343	334.1906
Trihydroxycoprostanoic acid	9.771_464.	POS	9.771	464.3481
Succinyladenosine	3.144_383.	POS	3.144	383.1077
(+/-)-(E)-Ethyl-2-[(E)-hydroxyimino]-5-nitro-3-he	0.805_215.	POS	0.805	215.091
Doronine; 4,8-Secosenecionan-8,11,16-trione, 15,2	5.985_459.	POS	5.985	459.1644
Deoxyartemsinin	7.873_266.	POS	7.873	266.1518
Butyl acrylate	1.003_128.	NEG	1.003	128.0838
Viridin	7.443_352.	POS	7.443	352.0946
LMPK12140625	4.458_354.	POS	4.458	354.0743
N-Ethylnorcotinine	3.98_190.1	POS	3.98	190.1107
6-Methylflavone	4.906_236.	POS	4.906	236.0798
Leucokinin I;	6.122_891.	POS	6.122	891.3873
Procaine	6.551_236.	POS	6.551	236.1526
8-O-Acetyl shanzhiside methyl ester	7.878_448.	POS	7.878	448.1578
6-Acetylmorphine	7.24_327.1	POS	7.24	327.147
N-[(4-Hydroxy-3-methoxyphenyl)methyl]-8-meth	6.233_305.	POS	6.233	305.1989
[(2R,3R,4R,5R,6S)-4,5-dihydroxy-6-methyl-2-[[(2R,	7.332_586.	POS	7.332	586.2068

**PE(22:6(4Z,7Z,10Z,13Z,16Z,19Z)/17:1(9Z))**	10.623_77!	POS	10.623	775.5151
**GDP-4-acetamido-4,6-dideoxy-alpha-D-mannose;**	0.649_630.	POS	0.649	630.1071
**3-[4-Hydroxy-3-[3-(4-hydroxyphenyl)prop-2-eno**	5.386_326.	POS	5.386	326.0787
**Indapamide**	6.001_365.	POS	6.001	365.0601
**Tetracosahexaenoylethanolamide**	6.099_399.	POS	6.099	399.3136
**(2S,3S)-N-(2-Methoxy-5-(trifluoromethoxy)benzyl**	4.375_380.	POS	4.375	380.1693
**Betamethasone 17-benzoate**	6.835_496.	POS	6.835	496.2257
**Naveglitazar**	4.378_422.	POS	4.378	422.1724
**Roseoflavin; 8-Demethyl-8-(dimethylamino)ribofla**	6.549_405.	POS	6.549	405.1642
**3-Succinoylpyridine**	5.776_179.	POS	5.776	179.0585
**Methylparaben**	4.718_152.	POS	4.718	152.0475
**Salsolinol**	6.696_179.	POS	6.696	179.0948
**(1R,3aR,3bS,4aR,6S,7S,7aR,8R,8aR,9aR)-1,4a,8-tris(**	6.573_562.	POS	6.573	562.2775
**Calanolide A**	6.467_370.	POS	6.467	370.1774
**(-)-Erythromycin**	6.451_733.	POS	6.451	733.4608
**Methyl-2-alpha-L-fucopyranosyl-beta-D-galactos**	3.266_340.	POS	3.266	340.1379
**4-[[(2S)-2-(3-Chlorophenyl)-2-hydroxyethyl]amin**	6.928_479.	POS	6.928	479.1705
**NCGC00385666-01!(1R,2S)-7-hydroxy-1-(4-hydro**	6.833_684.	POS	6.833	684.2701
**2-Hydroxy-6-oxo-6-(4'-chlorophenyl)-hexa-2,4-d**	3.145_252.	POS	3.145	252.0198
**Benzyl edta**	4.828_382.	POS	4.828	382.1393
**onopordopicrin**	6.548_348.	POS	6.548	348.1585
**25-O-Deacetylrifabutin**	7.557_804.	POS	7.557	804.4347
**9-Fluoro-16alpha-methylpregn-4-ene-3,11,20-tric**	5.132_360.	POS	5.132	360.2088
**2beta-hydroxymelianthusigenin**	6.787_516.	POS	6.787	516.2363
**6-Hydroxymelatonin**	5.507_248.	NEG	5.507	248.1159
**Cefozopran**	7.547_515.	NEG	7.547	515.0797
**Dimaprit**	3.266_161.	POS	3.266	161.0991
**Aflatoxin M1;**	3.266_328.	POS	3.266	328.0586
**{3,5-Dichloro-4-[4-Hydroxy-3-(Propan-2-Yl)phen**	5.481_354.	NEG	5.481	354.0411
**Norendoxifen**	4.534_359.	POS	4.534	359.1882
**PG(22:6(4Z,7Z,10Z,13Z,16Z,19Z)/0:0)**	7.226_556.	POS	7.226	556.2819
**PG(O-18:0/0:0)**	9.433_498.	POS	9.433	498.3328
**(2S)-2-Amino-3-[(2S,3R)-2-amino-3-hydroxybuta**	3.29_206.0	POS	3.29	206.0906
Roseomonas_gilardii				
**Stepronin**	0.676_273.	NEG	0.676	273.0142
**Propantheline**	6.46_368.2	POS	6.46	368.2209
**[[(3R,4R,5S,6R)-3-Acetamido-4,5-dihydroxy-6-(hy**	3.257_355.	POS	3.257	355.1377
Blautia_luti				
**6-Chloro-9-((4-methoxy-3,5-dimethylpyridin-2-yl**	2.098_318.	POS	2.098	318.0982
**2,2,2-Trifluoroethyl**	0.814_164.	POS	0.814	164.0065
**(2R,3S,5R)-5-(4,5-Dihydroimidazo[2,1-f]purin-3-yl**	0.964_277.	POS	0.964	277.1162
**N-Monodesmethyl-rizatriptan**	6.132_285.	POS	6.132	285.1576
**Medroxyprogesteroneacetate**	8.992_386.	POS	8.992	386.2457
**1'-hydroxytriazolam**	1.008_358.	POS	1.008	358.0386
**Talbutal**	5.351_252.	NEG	5.351	252.1473
**S9947; 2'-(Benzyloxycarbonylaminomethyl)biphen**	6.437_465.	POS	6.437	465.2048
**(R)-Ruxolitinib**	5.777_306.	POS	5.777	306.1579
**Azeliragon**	7.216_531.	NEG	7.216	531.2662
**2-(hydroxymethyl)-6-(6-hydroxy-6-methyl-3-pro**	5.979_354.	POS	5.979	354.1652
**2-(9H-Fluoren-1-yl)acetamide**	7.443_223.	POS	7.443	223.0997
**Acetylshikonin**	6.318_330.	POS	6.318	330.1103
**cis-Resveratrol 3-O-glucuronide**	6.593_404.	POS	6.593	404.1105
**4-(2,6-Dioxo-1-propyl-3,7-dihydropurin-8-yl)ben**	0.809_350.	NEG	0.809	350.0678
**Mabuterol**	4.399_310.	POS	4.399	310.1065
Acinetobacter_bohemicus				
Flavobacterium_granuli				
Effusibacillus_lacus				
Hymenobacter_gelipurpurascens				

Eggerthella_lenta				
**METHYL 4-FORMYLBENZOATE**	4.545_164.	POS	4.545	164.0476
**Desmethylofloxacin**	6.669_347.	POS	6.669	347.1282
**7-Oxotetradecanoic acid**	8.442_242.	NEG	8.442	242.1881
**Methylone**	6.31_207.C	POS	6.31	207.0897
**fuzhuanin B**	5.24_266.C	POS	5.24	266.0789
**5alpha-Androstane**	10.022_26	NEG	10.022	260.2498
**Daurisoline**	3.666_610.	POS	3.666	610.3071
**9-(4-Fluoro-3-(hydroxymethyl)butyl)guanine**	6.489_255.	POS	6.489	255.1129
**Corrole**	6.397_298.	NEG	6.397	298.1205
**(2S,3S,4S,8R,9S,13R,14R,15S,16R)-3,4,8,14,15-penta**	6.364_406.	POS	6.364	406.1376
**Obscuraminol F**	9.621_255.	POS	9.621	255.2563
**Mycophenolic acid**	6.347_358.	POS	6.347	358.0802
**Benzamil**	5.664_319.	POS	5.664	319.0954
**[(6Z,10Z)-6-(acetyloxymethyl)-10-(hydroperoxyme**	6.253_460.	POS	6.253	460.1487
Amaricoccus_kaplicensis				
**Glutaric acid**	2.954_132.	POS	2.954	132.0424
**Glutaric anhydride**	2.947_114.	POS	2.947	114.0318
**4-[1,1-Dioxo-3-(2,3,4-trihydroxyphenyl)-2,1lambd**	3.348_418.	POS	3.348	418.0356
**Arginyl-glycyl-aspartic acid**	6.097_346.	POS	6.097	346.1608
**Dioxidine**	1.113_222.	POS	1.113	222.0644
**Grape seed extract**	6.295_590.	POS	6.295	590.1786
**Methyl alpha-D-galactopyranoside**	0.738_216.	POS	0.738	216.0611
**PS(O-18:0/18:3(6Z,9Z,12Z))**	10.395_77	POS	10.395	771.5408
**NCGC00347722-02!(2Z)-2-[(E)-6-(hydroxymethyl)-**	9.3_418.26	POS	9.3	418.2696
**1,5-Dihydroxy-4,8-bis[2-(methylamino)ethylamino**	5.933_384.	POS	5.933	384.1787
**n-methacryloyl-l-histidine methyl ester**	3.502_237.	POS	3.502	237.1115
**Phorone**	8.442_138.	POS	8.442	138.1045
**19-Norandrosterone**	8.437_276.	POS	8.437	276.2089
Other				
**4E,6E-undecenal**	10.065_16	POS	10.065	166.1358
**Pindolol**	6.245_248.	NEG	6.245	248.1529
**Sipoglitazar**	8.789_463.	POS	8.789	463.1571
Sporobacter_termitidis				
**4-Methylphenol**	4.075_108.	NEG	4.075	108.0576
**5-O-Methyleriodictyol 7-glucosyl-(1->4)-galactos**	4.922_626.	POS	4.922	626.1829
**Cer(d18:2(4E,8Z)/19:0(2OH[R]))**	9.762_593.	POS	9.762	593.539
**Chembl4513510**	7.397_330.	POS	7.397	330.1943
**O-geranylconiferyl alcohol**	6.316_316.	POS	6.316	316.2034
**Piplartine**	5.773_334.	POS	5.773	334.1528
**PC(O-18:1(9Z)/20:4(8Z,11Z,14Z,17Z))**	11.911_79	POS	11.911	793.5985
**19-Chloroproansamitocin;**	7.831_477.	POS	7.831	477.1904
**Chloro-{methyl-[1-(2-methyl-5,6-dihydro-[1,4]oxa**	6.097_369.	POS	6.097	369.0803
Treponema_brennaborense				
**Meclizine**	6.013_390.	POS	6.013	390.1864
**Ikarisoside D**	6.621_542.	POS	6.621	542.1778
Clavibacter_michiganensis				
**(2S,3R,5R,10R,13R,14S,17S)-2,3,14-trihydroxy-10,1**	9.151_480.	NEG	9.151	480.3086
**Diltiazem**	5.611_414.	NEG	5.611	414.1613
**3-acetyl deoxynivalenol**	5.089_338.	POS	5.089	338.1378
**3-[(2,6-dihydroxy-3,4-dimethylphenyl)methyl]-2,4**	6.06_337.1	POS	6.06	337.1526
**100-2;**	6.015_600.	POS	6.015	600.2226
**Benazepril**	6.411_424.	POS	6.411	424.1995
Undibacterium_squillarum				
**Estra-1,3,5(10)-triene-3,17-diol, 16-iodo-, (16alpha**	3.771_398.	POS	3.771	398.0731
**Bromocriptine**	7.101_653.	POS	7.101	653.2225
**ANILINE-2-SULFONATE**	3.024_173.	POS	3.024	173.0149
**3 Pound feminine-Methoxy Puerarin**	6.295_446.	POS	6.295	446.1211

Other				
**Cearoin**	3.282_266.	POS	3.282	266.056
**6,7-Dichloro-L-tryptophan;**	0.68_272.0	NEG	0.68	272.0109
**3-Piperidinecarboxylic acid, 1-(4,4-bis(3-methyl-2-**	5.1_375.13	POS	5.1	375.1316
**3,4-Dihydroxymandelic acid**	8.857_148.	POS	8.857	148.0162
**Tyrosol**	4.78_138.0	NEG	4.78	138.0682
**PS(13:0/22:0)**	11.595_77	NEG	11.595	777.5556
**Dimethylphosphate**	7.543_126.	POS	7.543	126.0084
**beta,beta-Dimethylacrylshikonin**	6.863_370.	POS	6.863	370.1414
**5-Cholesten-3beta-25-diol-3-sulfate**	9.282_482.	NEG	9.282	482.3065
**Ovalitenin B**	5.753_332.	POS	5.753	332.1008
**thiobutyric acid**	2.591_104.	POS	2.591	104.0298
**(2R,3S,4S,5R,6R)-2-[[(2R,3R,4R)-3,4-dihydroxy-5-(I**	3.262_477.	POS	3.262	477.2223
**Propiverine N-oxide**	3.263_383.	POS	3.263	383.2109
**Curculigoside**	8.004_466.	POS	8.004	466.1454
**(E)-4,4'-(Hex-3-ene-3,4-diyl)bis(4,1-phenylene) bi**	5.567_428.	NEG	5.567	428.0777
**Ergosterol Acetate**	5.363_488.	POS	5.363	488.3618
**N-linoleoyl dopamine**	10.184_41	NEG	10.184	415.3088
**Shikimate-3-phosphate**	3.172_254.	POS	3.172	254.0203
Paracoccus_sphaerophysae				
**Tetramethylrosamine;**	6.462_391.	POS	6.462	391.1575
**DGMG(18:4(6Z,9Z,12Z,15Z)/0:0)**	6.776_674.	POS	6.776	674.3524
**Benzyl viologen;**	9.75_338.1	POS	9.75	338.1769
**1-(2-methoxy-13-methyl-tetradecanyl)-sn-glycerc**	6.779_455.	POS	6.779	455.3011
**1,3-Dithiane-2-propanamine, N-(2-(3,4-dimethoxy**	7.767_481.	POS	7.767	481.2129
**3-(Cyclohexylamino)-2-hydroxy-1-propanesulfoni**	6.401_237.	NEG	6.401	237.1034
**Idanpramine;**	6.904_423.	POS	6.904	423.2159
**Betamethasone 17,21-dipropionate**	6.169_504.	POS	6.169	504.251
**PS(18:2(9Z,12Z)/18:0)**	10.268_78	POS	10.268	787.5361
**3-Cyclobutene-1,2-dione, 3-((3-(3-(4-(5-methoxy**	6.473_460.	POS	6.473	460.2224
**18-acetoxy-PGF2alpha-11-acetate**	5.033_454.	NEG	5.033	454.2565
**2-Cyano-3-oxo-N-[4-(trifluoromethyl)phenyl]hept**	3.463_308.	POS	3.463	308.0758
**(+)-Norgestrel**	8.441_312.	POS	8.441	312.2087
Other				
**Cucurbitacin IIa**	6.792_562.	POS	6.792	562.3522
Other				
**Verruculogen; TR 1; TR 1 toxin;**	7.22_511.2	POS	7.22	511.2325
**NCGC00347365-02!5-hydroxy-2,2-dimethyl-10-(2**	8.573_312.	POS	8.573	312.1362
**GHB-Gluc**	0.721_280.	NEG	0.721	280.0781
**N-Acetylcochinol-O-phosphate**	6.037_437.	POS	6.037	437.1258
**trans-10-Heptadecenoic acid**	10.097_26	NEG	10.097	268.24
**Imidazolone A**	4.621_318.	POS	4.621	318.1537
Ruminococcus_faecis				
**Triethylene glycol bis(3-tert-butyl-4-hydroxy-5-m**	10.081_58	NEG	10.081	586.351
**gamma-Glutamyltyramine; gamma-L-Glutamyltyra**	3.466_266.	POS	3.466	266.1266
**4-Nitrophenyl diphenylphosphinate**	2.666_339.	POS	2.666	339.065
Faecalibacterium_prausnitzii				
**(E)-2-(hydroxymethyl)-3-(3-oxo-5-propan-2-yl-4,**	7.776_280.	POS	7.776	280.131
**Diethyl 2-methyl-3-oxosuccinate; Diethyl oxalprop**	2.447_202.	NEG	2.447	202.0841
**Ginkgolide C; BN 52022;**	4.715_440.	POS	4.715	440.1324
Methylobacterium_populi				
**Metaflumizone**	5.48_506.1	POS	5.48	506.1178
**(17E,19E,21E,23E,25E)-4,6,8,10,12,14,16,27-Octahyc**	8.69_654.3	NEG	8.69	654.3992
**Aleprylic acid**	9.027_196.	POS	9.027	196.1466
**4-Amino-3-hydroxyphenylalanine**	2.337_196.	POS	2.337	196.0851
**2'-Deoxyinosine 5'-monophosphate**	3.51_332.0	NEG	3.51	332.0567
**methyl 2-benzamido-3-phenylpropanoate**	6.381_300.	POS	6.381	300.1474
**3a-(3,4-dimethoxyphenyl)-1-methyl-2,3,4,5-tetrah**	6.698_304.	POS	6.698	304.1786

(2S)-2-[(1S,2R)-1,2-dihydroxypentyl]-4-methoxy-2	0.743_247.	POS	0.743	247.142
Glutamylleucylarginine	2.66_416.2	POS	2.66	416.2379
Chrysin	6.768_254.	POS	6.768	254.0576
Dodecanedioylcarnitine	6.257_373.	POS	6.257	373.2461
AM-toxin I;	6.421_445.	POS	6.421	445.221
(6E)-2,6,10-trimethyldodeca-6,11-diene-2,3,10-tri	9.266_274.	POS	9.266	274.2145
3-(1-Carboxyvinyloxy)anthranilate; 2-Amino-3-[(1	3.852_223.	POS	3.852	223.0482
Glycerophospho-N-palmitoyl ethanolamine	9.564_453.	NEG	9.564	453.2857
2-[2-[(Z)-pent-2-enyl]-3-[3,4,5-trihydroxy-6-(hyd	2.592_396.	POS	2.592	396.1752
LMGP04110003	6.296_776.	POS	6.296	776.5027
Thymidine	2.665_242.	POS	2.665	242.0903
2-Amino-6-(2-hydroxypropanoyl)-2,3-dihydro-1H	6.483_237.	POS	6.483	237.0859
Febarbamate	4.871_405.	POS	4.871	405.1897
Spirapril	3.29_466.1	POS	3.29	466.1594
Methyltrienolone	9.461_284.	NEG	9.461	284.1775
tetradec-5-ynoic acid	8.713_224.	NEG	8.713	224.1776
Venustone	7.885_566.	POS	7.885	566.3471
QUINGESTANOL ACETATE	8.14_408.2	POS	8.14	408.2661
6-Deoxy-6-sulfo-D-glucono-1,5-lactone;	5.412_242.	POS	5.412	242.0089
3-(3,5-Di-tert-butyl-4-hydroxyphenyl)propionic ac	7.997_278.	NEG	7.997	278.188
4-(1,3-Benzothiazol-2-yl)-2-methylaniline	0.735_240.	NEG	0.735	240.072
Coumarin 10	6.833_257.	POS	6.833	257.1415
Vincosamide	7.681_498.	POS	7.681	498.2006
Prodolic acid	8.693_273.	POS	8.693	273.1366
Streptococcus_anginosus				
Homovanillic acid 4-glucuronide	4.464_358.	POS	4.464	358.0901
Melicopicine;	4.768_329.	NEG	4.768	329.1264
Oleoyl ethanolamide	9.739_325.	POS	9.739	325.2981
2-[1-[(2S)-2-[[4-[(E)-N'-Hydroxycarbamimidoyl]be	5.078_392.	POS	5.078	392.1695
Zuclopenthixol	5.113_400.	NEG	5.113	400.1372
Pentaerythritol tetranitrate	0.698_316.	POS	0.698	316.0153
Nephromopsic acid	8.132_326.	POS	8.132	326.2456
2-Hydroxy-3-carboxy-6-oxo-7-methylocta-2,4-di	2.456_228.	NEG	2.456	228.0633
Corotoxigenin-3-O-alpha-L-rhamnopyranoside;	8.143_534.	POS	8.143	534.2853
Norfluoxetine glucuronide	3.324_471.	POS	3.324	471.1506
malotilate	4.739_288.	POS	4.739	288.0485
Prevotella_copri				
Oleoyl sarcosine	9.726_353.	POS	9.726	353.2929
Nefazodone	6.028_469.	POS	6.028	469.2249
1,7-bis(3,4-dihydroxyphenyl)-6-hydroxyheptan-3-	4.6_351.09	POS	4.6	351.0956
DG(12:0/17:2(9Z,12Z)/0:0)[iso2]	10.206_52	POS	10.206	522.4294
As-PL(16:0/0:0)	3.634_720.	POS	3.634	720.2816
Armillarisin A	7.456_234.	POS	7.456	234.0529
3-{[5-(Azetidin-1-ylcarbonyl)pyrazin-2-yl]oxy}-5-{	6.667_478.	NEG	6.667	478.1949
PHODiA-PG	8.777_654.	POS	8.777	654.3376
GLUTARYLCARNITINE	7.873_550.	POS	7.873	550.2764
Aerucyclamide A	6.62_534.2	POS	6.62	534.2107
Neu5NAcalpha2->6lactose	6.639_633.	POS	6.639	633.2102
Methotrimeprazine sulfoxide	6.095_344.	POS	6.095	344.1553
Methyl 6-(4-isopropyl-4-methyl-5-oxo-2-imidazo	7.682_288.	POS	7.682	288.1475
bhas#36	10.017_46	NEG	10.017	460.3399
Melezitose	7.573_521.	POS	7.573	521.1949
Dodecafluoropentane	0.821_287.	POS	0.821	287.9806
2-Propenamide, 2-cyano-3-(4-hydroxy-3,5-bis(1-i	6.111_272.	POS	6.111	272.1524
11	9.852_306.	POS	9.852	306.2195
DG(12:0/20:5(5Z,8Z,11Z,14Z,17Z)/0:0)[iso2]	9.78_558.4	POS	9.78	558.4288
Cobalt-precorrin 5A;	9.832_963.	POS	9.832	963.2672
Other				

**Melampodinin;**	4.925_522.	POS	4.925	522.1725
**3-(2,4-dihydroxyphenyl)-7-hydroxy-6,8-bis(3-met**	4.609_406.	POS	4.609	406.1774
**HC-toxin;**	3.951_436.	POS	3.951	436.2319
**3-Methoxy prostaglandin F1a**	9.251_368.	POS	9.251	368.2564
**rhodioloside**	6.46_300.1	POS	6.46	300.1208
**Cephaloglycin**	6.595_405.	POS	6.595	405.0996
**Atractylenolide II**	8.817_232.	POS	8.817	232.1466
**Arnicolide D**	4.831_354.	POS	4.831	354.1427
**Glucosaminate**	2.94_195.0	POS	2.94	195.0754
**(1-acetyloxy-3-hydroxy-6,8a-dimethyl-7-oxo-3-p**	6.432_430.	POS	6.432	430.2003
**2-Piperidinone**	1.813_99.0	NEG	1.813	99.0684
**Thalsimine;**	7.603_636.	POS	7.603	636.283
**LMST01031059**	9.854_492.	POS	9.854	492.3822
**Foretinib**	7.802_632.	POS	7.802	632.2477
**Deuteroporphyrin**	9.793_510.	POS	9.793	510.2262
**1,2-Cyclohexanedicarboxylic acid, 4-methyl-**	5.437_186.	POS	5.437	186.0895
**LMPK12113361**	4.377_420.	POS	4.377	420.105
**methyl (4S,5Z,6S)-5-(2-acetyloxyethylidene)-4-[2-**	12.945_58:	POS	12.945	582.194
**(2R,3S,4S,5R,6R)-2-[[(2R,3S,4S,5R)-3,4-dihydroxy-5**	3.801_466.	POS	3.801	466.1239
**Monomethyl phthalate**	2.706_180.	NEG	2.706	180.0423
**2,6-dihydroxyanthraquinone**	0.944_257.	POS	0.944	257.0682
**1-[6-[(3-Acetyl-2,4,6-trihydroxy-5-methylphenyl)r**	5.936_516.	POS	5.936	516.1796
**5a-Tetrahydrocortisol**	7.375_383.	POS	7.375	383.2669
**Vinblastine sulfate;**	0.739_909.	NEG	0.739	909.0567
**Oxypeucedanin hydrate**	6.78_304.0	POS	6.78	304.0946
**Adinazolam**	3.3_351.12	POS	3.3	351.1257
**D-Glucosamine-6-phosphate**	0.844_259.	POS	0.844	259.0459
**Enterolactone 3'-sulfate**	0.97_378.0	POS	0.97	378.0767
**Levobunolol**	6.929_291.	POS	6.929	291.1834
**destruxin A**	9.303_594.	POS	9.303	594.3751
**PE(12:0/13:0)**	7.948_593.	NEG	7.948	593.4044
**beta-Alanyl-L-histidine**	0.722_226.	NEG	0.722	226.1065
**N-(1,3-Dihydroxyoctadec-4-en-2-yl)acetamide**	9.512_341.	POS	9.512	341.293
**Linoleoyl ethanolamide**	9.509_323.	POS	9.509	323.2824
**13-(3,4-dimethyl-5-propylfuran-2-yl)-tridecanoic**	9.808_350.	POS	9.808	350.2819
**Eleutheroside A; Sitogluside; beta-Sitosterol D-gluc**	9.811_576.	POS	9.811	576.4395
**Anhydro-5alpha-cyprinol**	9.707_434.	POS	9.707	434.3399
**LMPK12140594**	6.772_724.	POS	6.772	724.2375
**Fenspiride**	3.922_260.	POS	3.922	260.1525
**Coformycin**	0.698_284.	NEG	0.698	284.111
**OHHdiA-PS**	7.646_679.	POS	7.646	679.3307
**Lilaline;**	6.131_383.	POS	6.131	383.1007
**5,5-Dimethylhydantoin**	4.456_128.	NEG	4.456	128.0586
**(2R,3S,4S,5R,6R)-2-(hydroxymethyl)-6-[4-(4-hydrc**	7.177_374.	POS	7.177	374.2303
Other				
**8-((4-Chlorophenyl)thio)cyclic-3',5'-GMP**	0.692_487.	NEG	0.692	487.0126
Acinetobacter_guillouiae				
Synechococcus_sp				
**Quinoline-3-carboxamide**	5.815_172.	POS	5.815	172.0639
**2-nonylquinolin-4(1H)-one**	5.687_271.	POS	5.687	271.1935
**(E)-5-(1,2,4a,5-tetramethyl-7-oxo-3,4,8,8a-tetrahy**	9.757_335.	POS	9.757	335.246
**DG(18:3(6Z,9Z,12Z)/18:3(9Z,12Z,15Z)/0:0)[iso2]**	11.794_61:	POS	11.794	612.4729
**Tyrphostin A1**	6.162_184.	POS	6.162	184.0639
**PS(22:0/0:0)**	10.178_58:	NEG	10.178	581.3695
Capnocytophaga_leadbetteri				
**Decaline; Decalin;**	6.588_437.	POS	6.588	437.2212
**(R)-1-(5-Tert-Butyl-2,3-dihydro-1H-inden-1-yl)-3**	4.883_348.	NEG	4.883	348.1939
**Elastase Inhibitor III**	6.766_502.	POS	6.766	502.2178

1H,3H-Pyrimidine-4-one-2-thione, 1-methyl-	2.763_142.	NEG	2.763	142.0202
14-O-(alpha-D-mannopyranosyl)-7S,14S-dihydrox	12.574_52:	POS	12.574	522.283
Ropinirole	5.849_260.	NEG	5.849	260.1886
(2S)-4-(2-Aminophenyl)-2-formamido-4-oxobuta	4.996_236.	NEG	4.996	236.0796
Bornaprolol	8.08_303.2	POS	8.08	303.2189
3-oxo-tetracosanoic acid	11.005_38:	NEG	11.005	382.3449
MEK1-2-inhibitor	2.768_335.	POS	2.768	335.0703
Dazmegrel	6.754_283.	POS	6.754	283.132
Patuletin 3-(4''-acetylrhamnoside)-7-(2''',4'''-diace	4.706_750.	NEG	4.706	750.2025
2-[(4S,5S,5aS,9aS)-4-methoxy-6,6,9a-trimethyl-5-|	7.228_529.	NEG	7.228	529.2702
Ilaprazole sulfone	4.873_382.	NEG	4.873	382.1085
Spirorenone	3.305_364.	POS	3.305	364.2035
N-Nitrosodibutylamine	0.647_158.	POS	0.647	158.1421
Remifentanil Acid	5.227_362.	POS	5.227	362.1838
Rhodamine B hydrazide	7.92_456.2	NEG	7.92	456.2542
Glycine	0.696_75.0	NEG	0.696	75.03196
DG(13:0/18:3(9Z,12Z,15Z)/0:0)[iso2]	10.174_54	POS	10.174	548.4452
(1R,9R,13R)-1,13-Dimethyl-10-[[(2R)-oxolan-2-yl]	4.315_301.	POS	4.315	301.2041
GLP-1 antagonist	9.074_463.	POS	9.074	463.284
(S)-N-Benzyl-2-((S)-2-(2-hydroxyacetyl)pyrrolidin	7.466_359.	POS	7.466	359.1843
Sultopride	7.27_354.1	NEG	7.27	354.1614
Alopecurone G	2.657_422.	POS	2.657	422.2083
2-(1,2,3,4-Tetrahydroxybutyl)thiazolidine-4-carbo:	2.74_253.0	POS	2.74	253.0623
Estrane	7.937_246.	POS	7.937	246.2349
Barbamide;	4.452_460.	NEG	4.452	460.0528
N-(3-oxoheptanoyl)-L-homoserine lactone	3.394_227.	POS	3.394	227.1159
icas#1	6.255_419.	POS	6.255	419.1945
Naringenin 7-[3-acetyl-6-p-coumaroylglucoside]	6.468_622.	POS	6.468	622.1688
Caohuoside D	7.609_562.	POS	7.609	562.2067
(4,8,8-trimethyldecahydro-1,4-methanoazulen-9-y	8.13_222.1	POS	8.13	222.1983
DL-Mandelic acid	1.684_152.	NEG	1.684	152.0474
CCR3 Antagonist	5.658_486.	POS	5.658	486.2487
5,7-Dihydroxy-2-(4-methoxyphenyl)-8-(3-methylb	6.971_516.	POS	6.971	516.2013
L-Galacturonic acid calcium salt;	0.819_426.	POS	0.819	426.0324
Acinospesigenin A	9.261_530.	POS	9.261	530.3595
S-Oxide	2.738_153.	NEG	2.738	153.0249
Fetidine;	5.96_682.3	POS	5.96	682.323
4-[(2R,5R)-5-(6-Aminopurin-9-yl)-3,4-dihydroxyo	0.658_322.	NEG	0.658	322.1375
Heptanal	5.111_114.	POS	5.111	114.1046
19-Epischolaricine	6.863_356.	NEG	6.863	356.1748
purpactin A	8.2_431.19	POS	8.2	431.1948
N-(R)-[2-(Hydroxyaminocarbonyl)methyl]-4-meth	4.418_415.	POS	4.418	415.2782
Androsterone	9.442_307.	POS	9.442	307.2511
Clomipramine	9.681_314.	POS	9.681	314.1543
Compound WIN VIII; 5-(5-(2,6-Dichloro-4-(4,5-dih	2.688_472.	POS	2.688	472.1185
Millettocalyxin C	2.669_322.	POS	2.669	322.0836
3-(2,4-Dimethoxybenzylidene)anabaseine	7.853_308.	POS	7.853	308.1522
5-Methylphenazine-1-carboxylate;	6.488_239.	POS	6.488	239.082
Recoflavone	3.347_386.	POS	3.347	386.0983
Myo-inositol glutamate	0.628_323.	NEG	0.628	323.0851
4-O,6-O-Benzylidene-alpha-D-glucopyranose	4.643_268.	POS	4.643	268.0948
2-(3,4,5-Trimethoxybenzoyl)-4,4-diethyl-3,5-isoxa	5.84_351.1	POS	5.84	351.1317
2-(3,4-dihydroxyphenyl)-5-hydroxy-3,7-bis[[(2S,3I	6.849_594.	POS	6.849	594.1589
DIMBOA-Glc	5.822_373.	POS	5.822	373.0993
15(S)-HpETE	8.483_336.	NEG	8.483	336.23
TETRAHYDROURIDINE	0.656_248.	NEG	0.656	248.1007
5,8-tetradecadienal	8.131_208.	POS	8.131	208.1827
Magnolol	3.086_266.	POS	3.086	266.1268

**Pyroglutamic acid**	1.172_129.	POS	1.172	129.0428
Klebsiella_pneumoniae				
**Fipexide**	6.61_388.1	NEG	6.61	388.1189
**Fluprednisolone**	5.789_378.	POS	5.789	378.1825
**(2E)-3-[3-(sulfooxy)phenyl]prop-2-enoic acid**	3.62_244.C	NEG	3.62	244.0039
**Phenyl-Alanine**	9.858_165.	POS	9.858	165.0792
**Dehydroandrographolide succinate**	5.893_532.	POS	5.893	532.2332
**Phorbolol myristate acetate**	10.02_618.	POS	10.02	618.4134
**Niddamycin;**	6.022_769.	POS	6.022	769.4219
**1-dimethylarsinoyl-octadecane**	5.683_374.	POS	5.683	374.2543
**3-(2-(Benzoxazol-2-yl)ethyl)-5-ethyl-6-methylpyr**	7.32_282.1	NEG	7.32	282.1367
**Difluprednate**	3.755_508.	POS	3.755	508.2285
Other				
**Leu-Gly-Pro;**	3.254_285.	POS	3.254	285.1689
**N-Nitrosoanatabine**	3.626_189.	POS	3.626	189.0905
**N-arachidonoyl dihydroxypropylamine**	9.966_377.	POS	9.966	377.2929
**Dimyristoylphosphatidylcholine, DL-**	10.884_67	POS	10.884	677.4992
**Isovitexin 2''-O-xyloside**	6.091_564.	POS	6.091	564.1481
**LMPK12020083**	5.947_452.	POS	5.947	452.111
**Leteprinim**	2.319_327.	POS	2.319	327.0954
**Lestaurtinib**	6.83_439.1	POS	6.83	439.1548
**Aspacoside E**	6.433_652.	POS	6.433	652.3473
**NCGC00169183-02!5-hydroxy-2-(3-hydroxy-4,5-d**	6.581_374.	POS	6.581	374.0998
**N-(3-oxo-dodecanoyl)-homoserine thiolactone**	6.296_313.	POS	6.296	313.1718
Other				
**N-docosahexaenoyl phenylalanine**	9.867_475.	POS	9.867	475.309
**Mascaroside I**	7.366_508.	POS	7.366	508.2313
**Ligustroflavone**	6.828_724.	POS	6.828	724.2212
**Chaetoglobosin A**	12.936_52	POS	12.936	528.2598
**Xestoaminol A**	9.291_227.	POS	9.291	227.2251
**icas#7**	4.43_417.1	POS	4.43	417.1791
**3,5,7-Trihydroxy-2-[3-hydroxy-4-[(2S,5S)-3,4,5-tri**	4.45_464.C	POS	4.45	464.0959
**Blood group B type 1 tetrasaccharide**	7.145_691.	POS	7.145	691.2516
**DOPA sulfate**	3.438_277.	NEG	3.438	277.0255
**Advantame**	6.43_458.2	POS	6.43	458.2063
**5-(Aziridin-1-yl)-3-(hydroxymethyl)-2-(3-hydroxy**	7.038_288.	POS	7.038	288.111
**NCGC00380664-01!4-[(E)-3-hydroxy-8,10-dimethy**	9.257_333.	POS	9.257	333.2667
**Duvelisib**	5.535_416.	NEG	5.535	416.114
Brooklawnia_cerclae				
**2-Aminobicyclo[3.1.0]hexane-2,6-dicarboxylic acid**	4.299_185.	POS	4.299	185.0691
**Neodulin**	5.652_308.	POS	5.652	308.0694
**(1R,3R,4S,6S,8R,9R,10S,11S)-8-hydroxy-3,8-dimeth**	3.298_465.	POS	3.298	465.2015
**Istamycin AO;**	5.311_337.	POS	5.311	337.186
**Haplophytine;**	6.536_652.	POS	6.536	652.2891
**1-(Naphthalen-2-yl)-2-(pyrrolidin-1-yl)pentan-1-**	6.807_281.	POS	6.807	281.1777
**Lobariolcarboxylic acid (Lobarin)**	8.59_474.1	NEG	8.59	474.1902
**Fluorescin**	2.687_334.	POS	2.687	334.0836
**20,21,21-Trifluoro-3-methoxy-19-nor-17alpha-pr**	6.676_366.	POS	6.676	366.1789
**N-(4-Amino-5-methoxy-2-methylphenyl)benzami**	5.268_256.	NEG	5.268	256.121
**Ergothioneine**	4.446_229.	POS	4.446	229.0886
**Dihydrocitrinone**	6.103_266.	POS	6.103	266.0803
**PG(20:4(5Z,8Z,11Z,14Z)/0:0)**	7.297_532.	NEG	7.297	532.2793
**5-Fluoromuconolactone;**	1.052_160.	POS	1.052	160.0172
**Mahanimbine; (+)-3,11-Dihydro-3,5-dimethyl-3-(**	6.849_331.	POS	6.849	331.1921
**D-a-Hydroxyglutaric acid**	0.648_148.	NEG	0.648	148.0371
**tetra(methoxymethyl)melamine (putative)**	6.834_302.	POS	6.834	302.1655
**N-oleoyl proline**	9.764_379.	POS	9.764	379.3086
**2-amino-5-[2-[[(3S)-2,3-dihydroxy-2-[(1S)-1-hyd**	4.471_436.	POS	4.471	436.1261

**Serotonin sulfate**	0.913_274.	POS	0.913	274.0625
**LMST01031185**	8.158_458.	POS	8.158	458.3035
**Bis(4'-chlorophenyl)methane; DDM;**	0.721_236.	NEG	0.721	236.0156
**trihydroxystilbene**	6.35_228.0	POS	6.35	228.0787
**Tecostanine;**	9.542_183.	POS	9.542	183.1625
**Benzyloxycarbonyl-Val-Ala-Asp(OMe)-fluorometh**	7.717_467.	POS	7.717	467.2059
**Decanoic acid**	8.41_172.1	NEG	8.41	172.1464
**Docosahexaenoic acid**	9.924_328.	NEG	9.924	328.2402
**(2E)-6-{13,17-dihydroxy-7,7,12,16-tetramethyl-6-[**	8.82_642.3	POS	8.82	642.3732
**Phenazo**	6.652_484.	NEG	6.652	484.1152
**Donepezil metabolite M4**	6.733_289.	POS	6.733	289.1678
**Menoctone**	7.567_368.	POS	7.567	368.2352
**cefpodoxime proxetil**	6.622_557.	POS	6.622	557.1251
**Glaziovine**	7.877_297.	POS	7.877	297.1363
**Sulpiride**	6.859_341.	POS	6.859	341.1403
**Estriol succinate**	5.943_488.	POS	5.943	488.2051
**3-Hydroxyethylchlorophyllide a; 3-Devinyl-3-(1-hy**	6.006_632.	POS	6.006	632.2481
Catenibacterium_mitsuokai				
**1H-Indole-2-carboxamide**	4.105_160.	POS	4.105	160.0639
**2,5-Dioxopyrrolidin-1-yl quinolin-6-ylcarbamate**	3.915_285.	NEG	3.915	285.0748
**Remikiren**	5.188_630.	POS	5.188	630.3463
**3-(2,6-dihydroxyphenyl)-4-hydroxy-6-methyl-3H-**	4.986_289.	POS	4.986	289.095
**3-(Carboxycarbonylamino)-L-alanine; N3-Oxalyl-L**	0.634_176.	NEG	0.634	176.0434
**4-({5-[5-hydroxy-3-({[(2Z)-2-methylbut-2-enoyl]c**	8.272_522.	POS	8.272	522.3203
**3-phenyl-2-[(2S,3R,4S,5S,6R)-3,4,5-trihydroxy-6-[[**	0.627_472.	POS	0.627	472.1373
**Vofopitant**	6.462_432.	POS	6.462	432.1899
**1,3-Dipropyl-8-p-sulfophenylxanthine**	6.552_392.	POS	6.552	392.1138
**1,2-Benzisoxazole**	4.317_119.	NEG	4.317	119.0371
**1-Benzylpiperazine**	0.767_176.	POS	0.767	176.1316
**parthenolide**	8.372_248.	POS	8.372	248.1414
**PC(P-18:1(9Z)/16:0)**	12.396_74:	POS	12.396	743.5838
**4-Aminoquinoline**	5.818_144.	POS	5.818	144.0691
**PS(16:1(9Z)/22:1(11Z))**	11.509_81!	NEG	11.509	815.5707
**[(1R,5S,6S)-3-(hydroxymethyl)-5-[(Z)-2-methylbut**	4.725_402.	POS	4.725	402.1651
**4-[3-[(2E)-3,7-dimethylocta-2,6-dienyl]-2,6-dihyd**	7.504_455.	POS	7.504	455.1954
**2-Benzyl-4-chlorophenol**	5.245_218.	NEG	5.245	218.0496
**Camonagrel**	4.449_272.	POS	4.449	272.1161
**Cyheptamide**	4.835_237.	POS	4.835	237.1162
**N-Palmitoyl taurine**	7.152_363.	POS	7.152	363.2406
Coprococcus_eutactus				
**NCGC00169348-03!(3Z)-3-[[5-(2-methylbut-3-en-**	5.859_626.	POS	5.859	626.3312
**L-Proline, 1-(1-L-leucyl-L-prolyl)-**	3.196_325.	POS	3.196	325.1999
**3-hydroxy-1a,5-bis(hydroxymethyl)-5,6b-dimethyl**	8.908_254.	POS	8.908	254.1519
**Phenethyl isocyanate**	6.624_147.	POS	6.624	147.0685
**3-O-(alpha-L-arabinopyranosyl-(1->6)-beta-D-gl**	8.381_454.	POS	8.381	454.206
**[(7R,8R,10R,13S)-17-(furan-3-yl)-4,4,8,10,13-penta**	4.718_450.	POS	4.718	450.2402
**11-deoxy-PGF1b**	8.907_340.	POS	8.907	340.2613
**2'-N-Hydroxyrifampicin; 2'-N-Hydroxy-4-oxorifam**	6.742_838.	NEG	6.742	838.4031
**(6RS)-22-oxo-23,24,25,26,27-pentanorvitamin D3 (**	4.416_392.	POS	4.416	392.2025
**(2R,3R,4R)-Hexane-1,2,3,4,6-pentol**	0.726_166.	NEG	0.726	166.0843
Comamonas_thiooxydans				
**Theobromine**	2.976_180.	POS	2.976	180.065
**Diethyl sulfone**	2.555_122.	POS	2.555	122.0402
**Papyriferic acid;**	8.864_604.	POS	8.864	604.3983
**Chamanetin**	5.632_362.	POS	5.632	362.1155
**Fluorothymidine**	5.655_260.	POS	5.655	260.0798
Corynebacterium_matruchotii				
**N-Acetyl-1-aspartylglutamic acid**	1.345_304.	POS	1.345	304.0908

**Marmin**	7.861_332.	POS	7.861	332.1623
**Stoloniferone F**	9.152_462.	NEG	9.152	462.3346
**aloenin**	6.767_427.	POS	6.767	427.149
**Quazepam**	3.795_386.	POS	3.795	386.0283
**4-Aminoacetophenone**	4.289_135.	NEG	4.289	135.0685
**Aminoethylcysteine ketimine decarboxylated dimer**	6.116_228.	POS	6.116	228.0401
**Monatepil**	6.739_475.	POS	6.739	475.2112
**Marimastat**	3.254_331.	POS	3.254	331.2105
**9-(2,3-dihydroxy-3-methylbutyl)-4-methoxyfuro[3**	3.707_356.	POS	3.707	356.0677
**Mevalonic acid**	0.949_148.	NEG	0.949	148.0737
**Thyrotropin-releasing factor**	6.754_362.	POS	6.754	362.1686
**Cytisine**	3.689_190.	POS	3.689	190.1108
**Octopamine**	1.207_135.	POS	1.207	135.0686
**N(4)-Methylcytosine**	0.757_125.	POS	0.757	125.0591
**PC(16:1(9Z)/22:2(13Z,16Z))**	12.472_81	POS	12.472	811.6091
**(2R,3S,4S,5S,6R)-2-[[7-hydroxy-4-[(Z)-5-hydroxy-**	6.662_484.	NEG	6.662	484.3033
**homoharringtonine**	6.8_545.26	NEG	6.8	545.2651
**Norcholic acid**	7.814_394.	POS	7.814	394.2719
**Desacetycefapirin**	4.245_381.	POS	4.245	381.0449
**Astragaloside A**	9.328_784.	POS	9.328	784.4585
**6-Methylprednisone**	6.926_372.	POS	6.926	372.1938
**Elgodipine**	3.027_524.	POS	3.027	524.2313
**Alanine lactate**	0.672_177.	NEG	0.672	177.0638
**Sulfamethoxazole**	5.318_291.	POS	5.318	291.0067
**Estramustine phosphate**	4.275_519.	POS	4.275	519.132
**pongaflavone**	5.37_356.1	POS	5.37	356.1007
**4-(N-Maleimido)benzyltrimethylammonium iodide**	3.284_372.	POS	3.284	372.2004
**Nomegestrol acetate**	5.221_370.	POS	5.221	370.2126
Sphingomonas_oligophenolica				
**5-AMINOLEVULINATE**	1.163_113.	POS	1.163	113.0479
**PG(17:0/22:6(4Z,7Z,10Z,13Z,16Z,19Z))**	8.646_808.	POS	8.646	808.5277
**Zoledronate**	0.728_271.	POS	0.728	271.9961
**2-Naphthoic acid**	5.625_172.	POS	5.625	172.0526
**NCGC00178646-02!2,4-dihydroxyheptadecyl aceta**	9.728_312.	POS	9.728	312.2664
**Moclobemide**	5.189_268.	NEG	5.189	268.0979
**PE-GlcDG(P-16:0/16:1(9Z))**	10.748_83	NEG	10.748	835.5595
**Glaucarubinone;**	7.495_494.	POS	7.495	494.2159
**Apigenin 7-glucuronide**	4.199_446.	NEG	4.199	446.085
**ibuprofen**	4.858_206.	POS	4.858	206.1309
**1-[2,4,6-trihydroxy-3-[7-hydroxy-2-(4-hydroxyph**	10.823_54	POS	10.823	548.2798
**alpha-(Chloromethyl)-2-hydroxymethyl-5-nitroimi**	3.738_235.	NEG	3.738	235.0358
**3'-Demethylstaurosporine; 3'-Demethoxy-3'-hydro**	8.654_452.	POS	8.654	452.1826
**4-hydroxy ketorolac**	6.803_271.	POS	6.803	271.0844
**Guanosine monophosphate**	2.646_363.	POS	2.646	363.0576
**N-[(5-{2-[(6R)-2-Amino-4-oxo-3,4,5,6,7,8-hexahy**	1.704_449.	NEG	1.704	449.1354
**Clofazimine glucuronide**	5.448_664.	NEG	5.448	664.1471
**p-Hydroxyphenobarbital**	4.112_248.	POS	4.112	248.0798
**2-N,6-N-Bis(2,3-dihydroxybenzoyl)-L-lysine;**	6.646_418.	NEG	6.646	418.1379
**(1S,4aS,7R,7aS)-7-hydroxy-7-[[(E)-3-(4-hydroxyph**	6.072_553.	POS	6.072	553.1788
**Chlorocarbonic acid**	0.649_79.9	NEG	0.649	79.9663
**(-)-Ephedrine**	3.306_165.	POS	3.306	165.1156
**NCGC00179905-02!(E)-3-(4-methoxyphenyl)-1-[2,**	5.125_418.	POS	5.125	418.174
**triacsin c**	2.56_207.1	POS	2.56	207.1375
**hesperetin 3'-O-sulfate**	5.251_382.	NEG	5.251	382.0361
**1-Palmitoylglycerophosphocholine; 1-Palmitoyl-sn**	8.543_496.	NEG	8.543	496.3398
**9-Fluoro-11beta,17beta-dihydroxy-16alpha-methy**	1.607_334.	POS	1.607	334.1932
**5-Ethyl-5-phenyl-1,3-oxazolidine-2,4-dione**	5.827_205.	POS	5.827	205.074
**3-Ethylphenylsulfate**	0.747_306.	POS	0.747	306.0926

**11-Methoxy-vinorine;**	3.263_364.	POS	3.263	364.1791
**Dihydroartemisinin (DHA)**	7.509_284.	POS	7.509	284.1624
**Valclavam;**	3.282_329.	POS	3.282	329.1586
**Formononetin 7-sulfate**	5.98_348.C	NEG	5.98	348.0305
**Codeine-6-glucuronide**	6.466_475.	POS	6.466	475.1847
**Delamanid**	5.358_534.	NEG	5.358	534.1727
**1-(2-methoxy-18Z-pentacosenyl)-sn-glycero-3-pl**	9.876_637.	POS	9.876	637.4343
**4-Hydroxypropofol**	10.136_19	POS	10.136	194.131
**Endralazine**	6.215_269.	POS	6.215	269.1266
**Piperanine**	7.262_304.	POS	7.262	304.1789
**1-Methyl-L-histidine**	0.698_169.	POS	0.698	169.0854
**Bioepiderm**	2.978_244.	POS	2.978	244.0883
**Luotonin F**	3.734_301.	POS	3.734	301.0883
**PS(O-20:0/14:1(9Z))**	10.775_74	POS	10.775	747.5409
**2-(N-Morpholino)-ethanesulfonic acid**	2.501_195.	POS	2.501	195.0569
**Benzonitrile, 3-fluoro-5-(2-(2-(fluoromethyl)-4-th**	3.163_260.	POS	3.163	260.0222
**10-Propargyl-5,8-dideazafolic acid**	6.538_477.	POS	6.538	477.1642
**1-Hydroxy-2,2,5,5-tetramethylpyrrolidine-3-carbo**	6.693_201.	POS	6.693	201.1366
**Clopidogrel acyl glucuronide**	6.955_483.	POS	6.955	483.0758
**Thromboxane A2**	7.12_352.2	POS	7.12	352.225
**Darexaban glucuronide**	5.01_650.2	NEG	5.01	650.2595
**2-(Dimethylamino)ethyl methacrylate**	3.837_157.	POS	3.837	157.1104
**Imidazole citrate**	0.675_254.	NEG	0.675	254.0166
**Emricasan**	6.698_569.	POS	6.698	569.179
**Hydroquinone**	4.88_110.C	POS	4.88	110.037
Oligosphaera_ethanolica				
Syntrophothermus_lipocalidus				
**Epigallocatechin gallate**	6.087_458.	POS	6.087	458.0854
**DL-Tryptophan**	3.295_204.	POS	3.295	204.0883
**Kaempferol 3-rutinoside-7-glucuronide**	4.813_770.	POS	4.813	770.1901
**Luteolin 7,3'-dimethyl ether 5-glucoside**	5.149_474.	NEG	5.149	474.1527
Romboutsia_sedimentorum				
**PS(18:0/22:5(7Z,10Z,13Z,16Z,19Z))**	10.211_83	POS	10.211	837.5515
**N-Isobutyryl-L-cysteine**	2.603_191.	POS	2.603	191.0619
**Vanylglycol**	3.774_166.	POS	3.774	166.0632
**Tetraphenylarsonium;**	5.559_383.	POS	5.559	383.0791
**2-(1,3-Dimethyl-2,6-dioxo-1,2,3,6-tetrahydro-7H-**	6.041_355.	POS	6.041	355.1629
**PA(P-20:0/0:0)**	8.676_450.	POS	8.676	450.3098
**Trioctyl trimellitate**	9.82_546.3	POS	9.82	546.3919
**L-Prolinamide, 5-oxo-L-prolyl-L-norvalyl-**	4.817_324.	NEG	4.817	324.1796
**2,2,11,13,15-pentachloro-docosane-1,14-disulfate**	6.432_672.	POS	6.432	672.0716
**Alpha-Alectoronic acid**	9.077_542.	POS	9.077	542.2176
**N-(3-Methyl-2-pyridyl)-3-(4-chlorophenyl)succini**	0.614_300.	POS	0.614	300.0657
**Cefditoren**	2.912_506.	POS	2.912	506.0518
**Telatinib**	6.08_409.C	POS	6.08	409.0943
Prevotella_brevis				
**2-((5-(Diethylamino)-[1,2,4]triazolo[1,5-a]pyrimidi**	9.723_334.	POS	9.723	334.2483
**Firuglipel**	5.077_467.	POS	5.077	467.1841
**HT-2 Toxin**	6.006_424.	POS	6.006	424.2112
**Azeloprazole**	6.56_429.1	POS	6.56	429.1703
**(2R,4aR,7S,8S,8aR)-8-[(Z)-5-hydroxy-3-methylpen**	9.779_324.	POS	9.779	324.2665
**N-stearoyl GABA**	10.071_36	NEG	10.071	369.3243
**Hexadecanedioic acid**	8.193_286.	NEG	8.193	286.2143
**15S-HpEDE**	9.68_340.2	NEG	9.68	340.2614
**L,L-Cyclo(leucylprolyl)**	3.22_210.1	POS	3.22	210.1369
**4-hydroxy-5-[4-hydroxy-3-(sulfooxy)phenyl]penta**	3.481_306.	NEG	3.481	306.0409
**1,3,7-Trimethyl-8-(3-chlorostyryl)xanthine**	3.056_330.	POS	3.056	330.0887
**Methitural;**	1.049_288.	POS	1.049	288.0959

**2-Hydroxypent-3-enenitrile**	3.737_97.0	POS	3.737	97.05304
**Triglochinin; 2-Hexenedioic acid,4-(cyano(beta-D-**	6.371_359.	POS	6.371	359.0835
**5,10-Methenyltetrahydrofolate;**	2.712_456.	POS	2.712	456.1648
**LMPK12050358**	4.663_650.	POS	4.663	650.2196
**8Z,11Z,14Z-Eicosatrienoic acid**	10.176_30	NEG	10.176	306.2558
**Parvaquone**	5.56_256.1	POS	5.56	256.1102
Collinsella_tanakaei				
**NCGC00168955-03![3-hydroxy-1-oxo-1-(2,3,4,5,6-**	12.613_50	POS	12.613	500.3007
**Fluvastatin**	5.287_411.	NEG	5.287	411.1829
**Sorafenib beta-D-Glucuronide**	12.919_64	POS	12.919	640.1206
**(2-acetyloxy-3,6-diphenylcyclohexyl) acetate**	6.371_374.	POS	6.371	374.1477
**Lolicine B;**	6.104_617.	POS	6.104	617.3692
**N-Formylmaleamic acid;**	3.215_143.	POS	3.215	143.0222
**rac-5,6-Epoxy-retinoyl-beta-D-glucuronide**	8.952_492.	POS	8.952	492.234
**Tomaymycin-DH-PPL hemiaminal**	4.154_304.	POS	4.154	304.1422
**Nocardicin C;**	3.763_486.	NEG	3.763	486.1735
**Marinobufagenin**	6.821_400.	POS	6.821	400.2249
**Divinylprotochlorophyllide; 2,4-Divinylprotochloro**	3.311_610.	NEG	3.311	610.2059
Aneurinibacillus_danicus				
Truepera_radiovictrix				
**5'-Benzoylphosphoadenosine;**	4.472_451.	POS	4.472	451.0906
**PC(18:0/22:6(4Z,7Z,10Z,13Z,16Z,19Z))**	11.943_83	POS	11.943	833.5938
**2,3,6-Trihydroxypyridine;**	1.064_127.	POS	1.064	127.0271
**Norfloxacin**	6.552_319.	POS	6.552	319.1327
**Sec-o-Glucosylhamaudol**	7.087_438.	POS	7.087	438.1526
**N-linoleoyl taurine**	9.45_387.2	NEG	9.45	387.2445
**Ramixotidine**	0.925_335.	NEG	0.925	335.1309
Collinsella_aerofaciens				
**Thr-Leu**	3.33_232.1	POS	3.33	232.1425
**N-Hexanoylsphingosine**	10.249_39	POS	10.249	397.3556
**N-formylkynurenine**	1.838_236.	POS	1.838	236.0798
Porphyromonas_endodontalis				
**6,7-bis(hydroxymethyl)-4,9a-dimethyl-2,7,8,9-tetr**	7.237_250.	POS	7.237	250.1569
**(2R,7S,13R,14R,16S,19R,20S)-19-(furan-3-yl)-9,9,1**	7.685_528.	POS	7.685	528.2016
**4-Hydroxycinnamyl alcohol 4-D-glucoside; p-Cour**	3.057_312.	POS	3.057	312.1208
**Cer(d16:1/23:0)**	10.559_60	POS	10.559	607.5912
**Methyl 2-(4-isopropyl-4-methyl-5-oxo-2-imidazo**	5.276_288.	NEG	5.276	288.1473
**Iganidipine**	6.573_526.	POS	6.573	526.2796
**L-CARNITINE**	0.729_161.	POS	0.729	161.1054
**Imazethapyr**	4.519_289.	POS	4.519	289.1428
**Linolipin C**	6.745_950.	NEG	6.745	950.5574
**Bryotoxin A;**	4.967_618.	POS	4.967	618.2652
**Chlorophyll b;**	9.843_906.	POS	9.843	906.5132
**(2S)-2-(3,8-Dimethylazulen-5-yl)propanoic acid**	5.275_228.	POS	5.275	228.1149
**N-Succinyl-L-glutamate; (2S)-2-(3-Carboxypropan**	5.927_247.	POS	5.927	247.0701
**Tefluthrin;**	0.81_418.0	NEG	0.81	418.0551
**Methylglyoxal-bis-guanylhydrazone**	4.302_170.	POS	4.302	170.0921
**Norgarmultinone B**	10.235_54	POS	10.235	544.392
**N-[3,3-Dimethyl-1-(methylamino)-1-oxobutan-2-**	4.046_395.	POS	4.046	395.2055
**PI(20:1(11Z)/22:6(4Z,7Z,10Z,13Z,16Z,19Z))**	7.937_936.	NEG	7.937	936.5757
**Urolithin B**	6.231_212.	POS	6.231	212.0476
**Benzoylpaeoniflorin**	6.35_584.1	POS	6.35	584.1904
**3-Hydroxycolensoic acid**	4.356_458.	NEG	4.356	458.1915
**PS(20:4(5Z,8Z,11Z,14Z)/18:0)**	10.162_81	POS	10.162	811.5361
**2-((3-Aminopropyl)amino)ethanethiol**	3.259_134.	POS	3.259	134.0882
**LysoPC(28:0/0:0)**	0.756_663.	POS	0.756	663.5213
**Aconifine; Aconitane-3,8,10,13,14,15-hexol, 20-eth**	6.632_661.	POS	6.632	661.3117
**2-Aminobenzoic acid**	4.627_137.	POS	4.627	137.0479

**Rolafagrel**	6.493_240.	POS	6.493	240.0899
**Zofenoprilat**	3.422_325.	POS	3.422	325.08
**Dimethylellagic acid glucuronide**	3.767_506.	NEG	3.767	506.0694
**SQDG(16:0/14:0)**	6.986_780.	NEG	6.986	780.502
Flavobacterium_ceti				
**Botrydial;**	7.481_310.	NEG	7.481	310.1779
**Lauryl sulfate**	8.859_266.	NEG	8.859	266.155
**Golotimod**	3.786_333.	POS	3.786	333.1324
**Delimotecan**	6.548_620.	POS	6.548	620.2591
Methanobrevibacter_boviskoreani				
**Fluoranthene**	0.749_202.	POS	0.749	202.0777
**2-Chloro-N-(2,6-diethylphenyl)acetamide;**	4.744_225.	POS	4.744	225.0922
**Chlordiazepoxide**	5.1_299.08	POS	5.1	299.0826
**4-(2-Aminoethyl)cyclohexa-3,5-diene-1,2-diol**	5.856_155.	NEG	5.856	155.0948
Flavonifractor_plautii				
**(5E)-4-[(E)-3-(4-hydroxyphenyl)prop-2-enoyl]oxy-**	6.072_509.	POS	6.072	509.19
**MADECASSIC ACID**	9.821_252.	POS	9.821	252.1725
**latrunculin B**	5.05_395.1	POS	5.05	395.1766
**Aceclofenac**	6.828_353.	POS	6.828	353.0222
**Belinostat**	0.745_318.	NEG	0.745	318.0683
**Didemnin A**	7.153_942.	POS	7.153	942.5635
**4-Methoxycinnamic acid**	4.539_178.	POS	4.539	178.0631
**alpha-carboxyethylhydroxychroman taurine**	6.754_385.	POS	6.754	385.1559
**N8-Acetylspermidine**	0.704_187.	POS	0.704	187.1688
**N-(p-Toluenesulfonyl)-L-phenylalanine**	5.095_319.	POS	5.095	319.0878
**methyl chlorogenate**	3.659_368.	NEG	3.659	368.1109
**1,3,7-Trimethyl-8-(1,3,7-trimethyl-2,6-dioxo-8H-p**	4.663_388.	POS	4.663	388.1609
**(2S,3R,4R,5R,6S)-2-[(2R,3R,4S,5R,6R)-2-[2-(3,4-dih**	10.282_47	POS	10.282	479.199
**PG(14:1(9Z)/22:6(4Z,7Z,10Z,13Z,16Z,19Z))**	6.069_764.	POS	6.069	764.4663
**Cyclosomatostatin**	0.751_779.	POS	0.751	779.438
**8-Prenylnaringenin**	6.03_340.1	POS	6.03	340.1308
**Benzoate**	4.047_139.	POS	4.047	139.0635
**Bacmecillinam**	6.092_441.	POS	6.092	441.1935
**Spenolimycin;**	7.812_346.	NEG	7.812	346.1728
Clostridium_colinum				
**Lanthionine ketimine**	2.366_189.	NEG	2.366	189.0097
**7-(1,5-dimethyl-3-(phenoxymethyl)-1H-pyrazol-4**	12.695_54	POS	12.695	545.2312
**1-(2-methoxy-tetradecanyl)-sn-glycero-3-phosph**	7.376_485.	POS	7.376	485.2763
**PS(O-18:0/17:2(9Z,12Z))**	10.726_75	POS	10.726	759.5432
**methyl 3-[3,4-dihydroxy-5-(3-methylbut-2-enyl)p**	12.695_53	POS	12.695	539.2509
**Wanepimedoside A**	8.192_678.	POS	8.192	678.2531
**4-Bromophenol;**	0.711_171.	NEG	0.711	171.9516
**3-Methoxytyrosine**	2.024_211.	POS	2.024	211.0847
**3,8-Divinyl-2,7,12,18-tetramethyl-21H,22H,23H,24**	4.892_564.	POS	4.892	564.276
**2-Dehydro-D-xylonate;**	0.641_164.	NEG	0.641	164.0322
**NCGC00381175-01!3-hydroxy-2-[[3-[3-(3-hydroxy**	9.369_559.	POS	9.369	559.3722
**N-docosahexaenoyl GABA**	10.194_41	NEG	10.194	413.2932
**PG(22:6(4Z,7Z,10Z,13Z,16Z,19Z)/15:1(9Z))**	7.91_778.4	POS	7.91	778.4805
**Kaempferol 3-[2''',3''',4'''-triacetyl-alpha-L-arabino**	6.574_706.	POS	6.574	706.1721
Nesterenkonia_sandarakina				
**PE(0:0/14:0)**	9.481_425.	NEG	9.481	425.2545
**Cyclo(Arg-Gly-Asp-D-Phe-Val)**	3.995_574.	NEG	3.995	574.2839
**Famphur; Famophos;**	3.127_325.	POS	3.127	325.0224
**2-O-(6-O-isocaleryl-beta-D-glucopyranosyl) atrac**	3_566.307	POS	3	566.3071
**Sphingofungin B**	6.64_389.2	POS	6.64	389.2776
**4-Chloro-[2-(4-Pyridinyl)-1-butenyl]phenol; 4-[1-**	3.299_243.	POS	3.299	243.0805
**(3E,12E)-3,12-dimethyl-8-methylidene-6,18-dioxa**	6.082_350.	POS	6.082	350.1478
**3-hydroxydodecanoyl carnitine**	6.441_359.	POS	6.441	359.2669

**Fonofos; Dyphonate;**	3.249_246.	POS	3.249	246.0311
**Aloxistatin**	8.943_342.	POS	8.943	342.2169
**Brassicanal A;**	5.056_191.	NEG	5.056	191.0405
**LMST01130037**	6.749_620.	POS	6.749	620.2845
**(-)-alpha-Narcotine**	6.453_413.	POS	6.453	413.1474
**Pegdinetanib**	7.447_694.	POS	7.447	694.2845
**NCGC00380967-01! [IIN-based: Match]**	7.547_354.	POS	7.547	354.1101
**Nandrolone**	8.201_274.	POS	8.201	274.1929
**D-Galactose 6-sulfate;**	0.651_260.	NEG	0.651	260.0199
Other				
**Quilseconazole**	6.062_513.	POS	6.062	513.1047
**6alpha-Fluoropregn-4-ene-3,11,20-trione; 6alpha-**	3.211_346.	POS	3.211	346.1933
**4-acetyloxy-8-(3-oxo-2-pent-2-enylcyclopenten-**	6.783_388.	POS	6.783	388.1633
**Fotemustina**	6.425_315.	POS	6.425	315.0756
**3-(4-((3-Phenoxybenzyl)amino)phenyl)propanoic a**	5.066_347.	POS	5.066	347.1536
**Plevitrexed**	7.834_532.	POS	7.834	532.2003
**Tetraazacyclododecanetetraacetic acid**	3.292_404.	POS	3.292	404.1906
**1-Methyl-4-nitroimidazole**	2.166_127.	NEG	2.166	127.0382
**1-[(4-Aminophenyl)methyl]-3-(3-nitrophenyl)imid**	6.397_361.	NEG	6.397	361.1163
**Flavonol 3-O-[alpha-L-rhamnosyl-(1->6)-beta-D-**	4.209_546.	POS	4.209	546.1734
**[9b-hydroxy-6-(hydroxymethyl)-6,9a-dimethyl-3-**	5.709_410.	POS	5.709	410.169
**[(2R,3S,4S,5R,6S)-3,4,5-trihydroxy-6-(4-hydroxyph**	5.351_434.	NEG	5.351	434.1214
**Eugenosedin-A**	6.468_416.	NEG	6.468	416.187
**7-[(3S,4S)-3-(Benzenesulfonamido)-2-bicyclo[2.2.1**	1.042_377.	NEG	1.042	377.1673
**Diglyme**	3.535_134.	POS	3.535	134.0944
**Vardenafil**	7.552_488.	POS	7.552	488.2182
**3'-Hydroxy-e,e-caroten-3-one**	10.109_56	POS	10.109	566.4135
**PGF2alpha-dihydroxypropanylamine**	7.752_427.	POS	7.752	427.2932
**Mopidralazine**	6.569_273.	POS	6.569	273.158
**Fucoxanthinol 3-myristate**	10.298_82	POS	10.298	826.6114
**Miraxanthin-III;**	6.732_330.	POS	6.732	330.1214
**Hydrocodone**	5.946_299.	POS	5.946	299.1519
**Penicillamine disulfide**	3.252_296.	POS	3.252	296.0865
**1-Methyl-2-nitro-3-((tetrahydrofuran-3-yl)methyl**	1.68_202.1	POS	1.68	202.1069
Bacteroides_cellulosilyticus				
**gamma-L-Glutamyl-D-alanine; L-gamma-Glutamy**	0.655_218.	NEG	0.655	218.0902
**Hippuryl-glycyl-glycine**	2.611_293.	POS	2.611	293.1012
**Glechomafuran**	5.077_265.	POS	5.077	265.168
**Glucosamine 6-sulfate**	3.152_241.	POS	3.152	241.0264
**(9-hydroxy-5,8-dimethyl-1-methylidene-2,6-dioxo**	4.481_380.	POS	4.481	380.1217
**Adenosine dialdehyde**	2.657_265.	POS	2.657	265.0812
**Myo-inositol,1,3,4,5-tetrakis(dihydrogen phosphat**	0.703_499.	POS	0.703	499.9281
Bacteroides_caccae				
**1alpha,25-dihydroxy-26,26,26,27,27,27-hexafluoro**	4.558_506.	NEG	4.558	506.226
**Clavamycin B;**	0.66_362.1	NEG	0.66	362.1437
**Thiostatin**	3.776_191.	POS	3.776	191.0982
**Evasterioside C**	6.319_648.	POS	6.319	648.3155
**(2S)-1,2-Dimethyl-3,4-dioxoazetidine-2-carboxylic**	0.647_157.	NEG	0.647	157.0376
**Rescinnamine**	10.027_63	POS	10.027	634.2768
**Azalanstat**	6.127_429.	POS	6.127	429.1257
**p38 MAP Kinase Inhibitor**	4.429_365.	NEG	4.429	365.0745
**2-Aminobenzylstatine**	4.775_280.	NEG	4.775	280.1786
**12-Oxo phytodienoic acid**	8.883_310.	POS	8.883	310.2144
**Glycyl-prolyl-glutamic acid**	0.737_301.	POS	0.737	301.1273
**PC(O-18:0/20:4(8Z,11Z,14Z,17Z))**	12.638_79	POS	12.638	795.6142
**2-((2-(Dimethylamino)ethyl)thio)-3-phenylquinoli**	3.767_308.	POS	3.767	308.1347
**7-Amino-4-hydroxy-2-naphthalenesulfonic acid**	4.844_239.	POS	4.844	239.0252
**Biotin sulfoxide**	2.544_260.	POS	2.544	260.0832

**Trolox**	5.957_250.	NEG	5.957	250.1204
**Hypoglycin A**	3.069_141.	POS	3.069	141.0793
**Istamycin KL1;**	5.106_336.	POS	5.106	336.2026
**LMPK12140616**	4.632_612.	POS	4.632	612.1689
**Taccalonolide B**	8.352_660.	POS	8.352	660.2793
**3-hydroxydecanoyl carnitine**	6.348_331.	POS	6.348	331.2355
**Tetroxoprim**	4.905_334.	POS	4.905	334.1643
**Ampicillin**	4.382_349.	POS	4.382	349.1094
**3-(4-Hydroxyphenyl)-3,5,6,8-tetrahydro-2H-chron**	6.303_258.	POS	6.303	258.0891
**Pregnenediol**	10.469_31	POS	10.469	318.2559
**Fumaryl diketopiperazine**	3.332_452.	POS	3.332	452.1909
**PG(22:2(13Z,16Z)/0:0)**	8.149_564.	POS	8.149	564.3442
**Heteroartonin A**	4.445_452.	NEG	4.445	452.1824
**1H-Indole-5-carboxamide**	5.832_160.	NEG	5.832	160.0638
**Methazolamide**	0.801_236.	POS	0.801	236.0034
**PC(O-16:1(9Z)/18:0)**	12.554_74	POS	12.554	745.5991
**(-)-Jolkinol B**	4.299_464.	POS	4.299	464.2555
**Benzenesulfonic acid**	1.761_158.	NEG	1.761	158.0038
**Tetrahydrofuran**	11.476_72.	POS	11.476	72.05761
**tetrahydrothiazine**	0.805_103.	POS	0.805	103.0458
**Firsocostat**	3.246_569.	POS	3.246	569.1841
**8-(3-(1-((3-Fluorophenyl)methyl)-4-piperidinyl)-1**	3.352_420.	POS	3.352	420.2219
**2'-O-Methylguanosine**	3.593_297.	POS	3.593	297.1034
**alpha-guaiaconic acid**	6.11_357.1	POS	6.11	357.1573
**[(9R,10R)-10-acetyloxy-8,8-dimethyl-2-oxo-9,10-**	6.341_405.	POS	6.341	405.1788
Other				
**Griseofulvin**	3.75_369.C	POS	3.75	369.0994
**Isradipine**	6.613_371.	POS	6.613	371.148
**Acarbosa**	6.548_645.	POS	6.548	645.2475
**3,3',5-Trihydroxy-4'-methoxy-6,7-methylenedioxy**	6.958_520.	POS	6.958	520.0859
**Grepafloxacin**	4.504_359.	POS	4.504	359.1644
**2,3,4,5-Tetrahydrodipicolinate; Delta1-Piperidine-2**	3.492_171.	POS	3.492	171.0534
**2-Anisic acid**	4.436_134.	POS	4.436	134.037
**Coumarin**	1.186_146.	POS	1.186	146.037
**TG(16:0/18:1(9Z)/20:4(5Z,8Z,11Z,14Z))**	11.52_880.	POS	11.52	880.7498
**PG(P-20:0/0:0)**	7.035_524.	NEG	7.035	524.3471
**3-hydroxyoctanoyl carnitine**	4.961_303.	POS	4.961	303.2043
**1,2-Bis(2-aminophenoxy)ethane N,N,N',N'-tetraac**	5.254_548.	POS	5.254	548.1655
**Lenalidomide**	4.013_259.	POS	4.013	259.0957
**Spermidine**	0.635_145.	POS	0.635	145.1581
**PC(20:1(11Z)/20:3(8Z,11Z,14Z))**	12.753_83	POS	12.753	837.6242
**Coproporphyrin III tetramethyl ester**	5.322_710.	POS	5.322	710.3337
**Roxindole**	5.131_346.	POS	5.131	346.2049
**Ziritaxestat**	4.427_588.	POS	4.427	588.2408
**Octadeca-8,10,12-trienoic acid**	9.777_278.	NEG	9.777	278.2244
**N5-Phenyl-L-glutamine; gamma-Glutamylanilide;**	2.746_222.	POS	2.746	222.1006
**Draflazine**	5.607_603.	NEG	5.607	603.1965
**N-stearoyl dopamine**	10.039_41	NEG	10.039	419.3399
**Talactoferrin Alfa**	6.641_368.	NEG	6.641	368.1404
**Mitomycin B;**	3.099_335.	POS	3.099	335.1117
**Quercetagetin 4'-methyl ether 7-(6-(E)-caffeylgluc**	6.488_656.	POS	6.488	656.1376
**NALOXONE HYDROCHLORIDE**	4.384_380.	POS	4.384	380.1518
**Stavudine**	4.55_224.C	POS	4.55	224.0799
**Methyl 6,7-dimethoxy-4-ethyl-beta-carboline-3-c**	6.525_314.	POS	6.525	314.1263
**Flaxseeds extract**	5.615_362.	NEG	5.615	362.1729
**(3E,6Z)-3-Benzylidene-6-[[4-[2-(dimethylamino)et**	4.514_377.	POS	4.514	377.1749
**S-Adenosylmethionine folate**	5.912_839.	POS	5.912	839.2788
**TG(18:1(9Z)/16:0/18:3(9Z,12Z,15Z))**	10.641_85	POS	10.641	854.7342

**Butyrophenone**	6.801_148.	POS	6.801	148.089
**Clavamycin C;**	0.749_362.	POS	0.749	362.1435
**Amidinophenylpyruvic acid**	4.137_206.	POS	4.137	206.0694
**1H-Indole-2,3-dione**	4.091_147.	POS	4.091	147.0322
**N-(3-hydroxy-pentanoyl)-homoserine lactone**	3.048_201.	NEG	3.048	201.1003
**Nocamycin E;**	7.246_489.	POS	7.246	489.2004
**Sampatrilat**	7.462_584.	POS	7.462	584.2544
**7-benzyl-11,14-dimethyl-16-(2-methylpropyl)-10,**	10.758_65!	NEG	10.758	655.3944
**Moroidin**	4.309_493.	POS	4.309	493.2533
**Isovaleric acid**	0.68_102.C	NEG	0.68	102.0681
**Methyl prednisolonate**	7.507_388.	POS	7.507	388.1887
**Phenazine-2,3-diamine**	5.439_210.	POS	5.439	210.0896
**Netoglitazone**	4.586_381.	POS	4.586	381.0824
**Moroidin-[QLAVWRGH]**	6.901_472.	POS	6.901	472.2312
Other				
**Bisazobiphenyl**	4.787_206.	POS	4.787	206.0584
**NCGC00179830-03!2-[5-[2-[2-[5-(2-hydroxypropy**	7.046_368.	POS	7.046	368.22
**2-[(4s)-6-(4-Chlorophenyl)-8-Methoxy-1-Methyl-**	6.555_423.	POS	6.555	423.1465
**Biapenem**	6.951_350.	NEG	6.951	350.1033
**Arotinolol**	3.298_371.	POS	3.298	371.0783
**Desmethylranitidine**	2.918_300.	POS	2.918	300.1253
**Cinnarizine**	9.383_368.	POS	9.383	368.2237
**2-Amino-7-methoxy-3H-phenoxazin-3-one**	3.713_242.	POS	3.713	242.0689
Clostridium_lactatifermentans				
**6-Deoxocastasterone**	10.079_45(	POS	10.079	450.3706
**8-Epideoxyloganic acid;**	4.223_360.	POS	4.223	360.142
**Cholesterol glucuronide**	9.812_562.	POS	9.812	562.386
**Chlorethoxyfos;**	0.692_333.	NEG	0.692	333.892
**Sanggenon B;**	4.801_570.	POS	4.801	570.1889
**2-Hydroxy-3-morpholinopropanesulfonic Acid**	6.492_225.	POS	6.492	225.0665
**Phenmedipham**	6.01_300.1	POS	6.01	300.1108
**PG(22:4(7Z,10Z,13Z,16Z)/22:6(4Z,7Z,10Z,13Z,16Z,1!**	8.666_870.	POS	8.666	870.5428
**Tyramine glucuronide**	1.005_313.	POS	1.005	313.1161
**Undecanedioic acid**	5.834_216.	POS	5.834	216.1363
**Nirurin**	6.963_664.	POS	6.963	664.2385
**3-Butene-1,2-diol**	8.084_88.C	POS	8.084	88.05241
**Gemifloxacin**	5.609_389.	NEG	5.609	389.1508
**Caracemide**	0.666_189.	NEG	0.666	189.0751
**2,4,18-trihydroxy-15-(3-hydroxy-3-methylbutyl)-7**	2.755_412.	POS	2.755	412.1166
**Ethyl icosa-6,9,12,15-tetraenoate**	10.063_33:	POS	10.063	332.2715
**2-(3,5-dihydroxy-1,4-dioxo-1,4-dihydronaphthale**	4.717_354.	POS	4.717	354.0743
**N-[2-(3,5-Dioxopiperazino)propyl]-N-(2-amino-2-**	0.744_286.	POS	0.744	286.1276
**Fenothiocarb sulfoxide;**	4.979_269.	POS	4.979	269.1084
**Isoindole**	5.95_117.C	POS	5.95	117.0579
**(R)-2-Amino-3-benzylthio-N-(4-nitrophenyl)propi**	6.217_331.	POS	6.217	331.099
**14,15-DiHETrE**	8.969_338.	NEG	8.969	338.2459
**(20R)-24-Hydroxy-19-norgeminivitamin D3**	9.443_506.	POS	9.443	506.3989
**(22E,24E)-1alpha,25-dihydroxy-26,27-diethyl-22,2:**	9.743_482.	POS	9.743	482.3739
**Demethylphosphinothricin; (2S)-2-Amino-4-(hydro**	4.063_167.	NEG	4.063	167.0347
**Isoliquiritigenin**	5.109_256.	POS	5.109	256.0734
**Moupinamide**	6.663_351.	POS	6.663	351.0888
**Batelapine**	3.247_296.	POS	3.247	296.1739
**5-Hydroxy-2,4-dimethylthiophen-3-one**	3.699_144.	POS	3.699	144.0247
**Triangulyne E**	9.673_474.	POS	9.673	474.3114
**SERATRODAST**	9.259_354.	POS	9.259	354.1831
**Ochrephilone**	7.462_404.	POS	7.462	404.1583
**(3Z)-3-[[1,6-dimethyl-2-[(1E,3E)-penta-1,3-dienyl]**	3.263_437.	POS	3.263	437.1956
**Lenperone**	3.254_371.	POS	3.254	371.1709

2-Imidazolidinone, 4-hydroxy-1-(5-nitro-2-thiazol	4.089_230.	POS	4.089	230.0105
Tolbutamide	2.672_270.	POS	2.672	270.1037
Narcotoline;	5.161_399.	POS	5.161	399.1321
cis-Resveratrol 3-sulfate	3.255_308.	POS	3.255	308.0356
Echinasteroside C 15-O-sulfate	7.813_692.	NEG	7.813	692.3458
Nigericin	9.784_724.	POS	9.784	724.4736
Triethyl orthoformate	4.391_148.	POS	4.391	148.1102
7-methyl-1-[(2S,3R,4S,5S,6R)-3,4,5-trihydroxy-6-(I	7.009_402.	POS	7.009	402.0948
ajugalactone	5.173_516.	POS	5.173	516.2718
M8-Nelfinavir	7.181_583.	POS	7.181	583.3059
LMPK12010438	5.319_501.	POS	5.319	501.1044
[(2R,3S,4S,5S)-4-hydroxy-2,5-bis(hydroxymethyl)-!	4.312_455.	POS	4.312	455.1337
3-(Hydroxymethyl)-5,5-diphenylimidazolidine-2,4-	6.497_282.	POS	6.497	282.1004
Isoflavan	7.56_210.1	POS	7.56	210.1049
Methyl 6,6-bis[4-methoxy-3-(methoxycarbonyl)-5	6.709_484.	POS	6.709	484.2075
seglitide	7.615_808.	NEG	7.615	808.4273
2,3-Dihydroxypropyl 4-phenylbutanoate	6.087_238.	POS	6.087	238.1206
4-tert-Amylphenol	9.998_164.	NEG	9.998	164.1183
PI(14:1(9Z)/18:4(6Z,9Z,12Z,15Z))	9.79_800.4	POS	9.79	800.4515
9-Anthraldehyde	1.029_206.	NEG	1.029	206.0724
2,2,2-Trifluoroacetophenone	3.845_174.	POS	3.845	174.0294
Bardoxolone	8.985_491.	POS	8.985	491.3019
4-hydroxyalprazolam	5.833_324.	POS	5.833	324.0778
Hydantoin-5-propionate; Hydantoin-propionate;	0.703_172.	NEG	0.703	172.0485
delta-(L-2-Aminoadipyl)-L-cysteinyl-D-valine; N-[	1.679_363.	POS	1.679	363.1465
PG(17:1(9Z)/22:4(7Z,10Z,13Z,16Z))	9.776_810.	POS	9.776	810.5434
(2S)-2-(Benzenesulfonamido)-N-[(1S)-1-hydroxy-	4.694_484.	NEG	4.694	484.2056
Pitrazepin;	6.684_317.	POS	6.684	317.1656
1-{2-[(3-Ethylphenyl)amino]-2-oxoethyl}-6-oxo-1	5.272_300.	NEG	5.272	300.111
(2S)-3-(4-hydroxyphenyl)-2-[[(Z)-3-(4-hydroxyph	7.15_344.1	POS	7.15	344.1371
Norketamine	4.345_223.	POS	4.345	223.0765
PE(O-18:1(9Z)/0:0)	10.016_46	POS	10.016	465.3222
(4S,5Z,6S)-4-(2-methoxy-2-oxoethyl)-5-[2-[(E)-3-	3.278_202.	NEG	3.278	202.1205
2-[(1E,3E,5E)-3,7-Dimethylnona-1,3,5,8-tetraenyl]-	8.852_270.	POS	8.852	270.2348
Nelfinavir	7.391_567.	NEG	7.391	567.3122
Salbutamol 4-O-sulfate	3.198_319.	NEG	3.198	319.1089
Sn-glycerol 3-phosphate	0.991_172.	POS	0.991	172.0139
(R)-N-(Quinuclidin-3-yl)furo(2,3-C)pyridine-5-carl	4.899_271.	NEG	4.899	271.1318
Albafuran A; 4-((2E)-3,7-Dimethyl-2,6-octadienyl)-	2.946_378.	POS	2.946	378.1823
Disinomenine; Bisinomenine;	6.354_656.	POS	6.354	656.3069
Cer(d18:1/23:0)	11.961_63	POS	11.961	635.6219
NCGC00380344-01!(2E,4E)-N-[2-(4-hydroxypheny	8.042_315.	POS	8.042	315.2188
Methyl 4,8-decadienoate	10.191_18	POS	10.191	182.1309
Bctc	6.563_372.	POS	6.563	372.1718
Diethylamino 2,5-dihydroxybenzenesulfonate	3.303_261.	POS	3.303	261.0671
syringin	6.908_389.	POS	6.908	389.1667
CDP-ethanolamine; Cytidine diphosphate ethanola	0.895_446.	NEG	0.895	446.0611
Brainesteroside B	9.353_608.	POS	9.353	608.3536
(1R,3R)-5-[(2E)-2-[(3As,7aS)-7a-methyl-1-[(Z,2R)-	6.479_508.	POS	6.479	508.2426
Oleoyl glycine	9.385_339.	POS	9.385	339.2772
Blautia_producta				
NCGC00347636-02![2-[2-(2-methylpropanoyloxy)ı	6.876_402.	POS	6.876	402.1677
C20911; Tabtoxin biosynthesis intermediate 1;	0.626_275.	NEG	0.626	275.0638
Difenacoum	6.011_444.	POS	6.011	444.1719
Obtusaquinone	6.051_254.	POS	6.051	254.0948
PKODA-PE	5.924_661.	POS	5.924	661.3978
Lumirubin	8.195_584.	POS	8.195	584.2645
D-gamma-Glutamyl-D-glutamic acid	0.632_276.	NEG	0.632	276.0955

**3-[[(2S)-2,4-Dihydroxy-3,3-dimethylbutanoyl]amir**	3.034_241.	POS	3.034	241.0927
**Bambuterol**	4.411_367.	POS	4.411	367.2104
**1,2-Dihydroxy-5-(methylthio)pent-1-en-3-one; 1,**	3.656_162.	POS	3.656	162.0353
**Fluazifop-butyl**	1.68_383.1	POS	1.68	383.1329
**Fesoterodine**	7.861_411.	POS	7.861	411.2775
**3-(2-Butynyl)-5-methyl-2-(piperazin-1-yl)-3,5-dil**	4.414_286.	POS	4.414	286.1532
**4-Aminobenzoic acid**	4.3_119.03	POS	4.3	119.0373
**Asp-Asp-Asp-Asp-Lys**	5.926_606.	POS	5.926	606.2117
**Geranyl phosphate;**	5.844_234.	POS	5.844	234.1018
**3-Hydroxy-3-methylglutaric acid**	2.436_184.	POS	2.436	184.035
**L-(+)-Citrulline**	1.205_197.	POS	1.205	197.0803
**1,6-Dimethoxypyrene;**	3.079_262.	POS	3.079	262.0987
**2-Amino-5-formylamino-6-(5-phospho-D-ribosyl**	12.957_38	POS	12.957	381.0693
**LMPR0106140011**	9.822_512.	POS	9.822	512.3876
**Rhodamine 110**	1.016_330.	NEG	1.016	330.0997
**Butyl-(2,5-dimethyl-7-(2,4,6-trimethylphenyl)-7H-**	9.792_364.	POS	9.792	364.2613
**2-Methyl-4-(pyridin-3-ylethynyl)thiazole**	0.708_200.	NEG	0.708	200.0409
**3-Indoxyl sulphate**	3.022_213.	NEG	3.022	213.0095
**2-Hydroxyfelbamate**	3.25_254.0	POS	3.25	254.0902
**Pheophorbide a**	10.1_592.2	POS	10.1	592.2692
**Bergenin**	4.739_350.	POS	4.739	350.0613
**N-stearoyl glutamine**	9.6_412.33	POS	9.6	412.33
**8-Quinolinol, 5,7-dichloro-2-((dimethylamino)met**	0.713_270.	POS	0.713	270.0327
**2-(3-hydroxy-5-methoxyphenoxy)-6-(hydroxymet**	3.34_324.0	POS	3.34	324.0812
**(R*,S*)-4-[1-Ethyl-2-(4-fluorophenyl)butyl]phenol**	6.007_272.	POS	6.007	272.1576
**Chenodeoxycholic acid disulfate**	5.715_552.	POS	5.715	552.209
**sn-Glycero-3-phosphoethanolamine; Glycerophos**	6.431_215.	POS	6.431	215.0559
**Protoasukamycin;**	7.436_514.	NEG	7.436	514.2443
**2-(2,6-dihydroxy-4-methoxycarbonylbenzoyl)-3-h**	4.621_349.	POS	4.621	349.0799
Other				
**Asp f I**	7.033_502.	POS	7.033	502.1858
**Prothioconazole;**	4.925_343.	NEG	4.925	343.0314
**Dipfluzine**	3.641_416.	POS	3.641	416.2273
**11,11,11,12,12-Pentafluoro-9Z-dodecenyl acetate**	6.862_316.	POS	6.862	316.1447
**Cucurbitacin A**	5.691_574.	POS	5.691	574.3119
**Pipericine**	10.144_33	POS	10.144	335.3187
**Lapaquistat acetate**	7.574_644.	POS	7.574	644.2488
**Methanandamide**	9.858_361.	POS	9.858	361.298
**S-6-Hydroxywarfarin**	6.529_324.	POS	6.529	324.0996
Paenibacillus_thermoaerophilus				
Other				
**Mertiatide**	3.312_263.	POS	3.312	263.0577
**7-Oxocholesterol**	8.845_400.	POS	8.845	400.3342
Pseudoscardovia_radai				
Blastomonas_natatoria				
Other				
Other				
Eubacterium_nodatum				
Anaerovibrio_lipolyticus				
Sharpea_azabuensis				
Other				
Denitrobacterium_detoxificans				
Bacteroides_finegoldii				
**4H-1,2,4-Triazole, 3-(trans-1-(4-chlorophenyl)-3-**	6.732_331.	POS	6.732	331.1246
**Z-Tyr-Leu-NHOH; Benzyloxycarbonyl-Tyr-Leu-NH**	4.729_443.	POS	4.729	443.2059
**5-Amino-4-(2-hydroxy-3,4-dimethoxyphenyl)-6-(**	6.71_506.1	POS	6.71	506.1429
**Cadazolid**	7.48_585.1	POS	7.48	585.191
**Lactobionic acid**	0.721_358.	NEG	0.721	358.111

**4'-Iododoxorubicinol**	6.539_655.	POS	6.539	655.0926
**Anhydrotetracycline;**	0.639_426.	POS	0.639	426.1447
**3b-Hydroxy-5-cholenoic acid**	10.083_37	POS	10.083	374.2822
**3-Amino-4-(3-hexylphenylamino)-4-oxobutylphos**	4.64_342.1	POS	4.64	342.1703
**2-((2-((Dimethylamino)methyl)phenyl)thio)-5-iodo**	5.275_384.	NEG	5.275	384.0153
**Apramycin**	10.804_53	POS	10.804	539.2807
**Bucladesine**	3.543_469.	POS	3.543	469.1348
**Fasciculic acid B**	9.787_636.	POS	9.787	636.4217
**Glycerol lactate pyruvate**	0.731_234.	NEG	0.731	234.0739
**Rhizocarpic Acid**	3.88_469.1	POS	3.88	469.1522
**1-Hydroxyphenazine;**	6.483_196.	POS	6.483	196.0638
**Remogliflozin A**	4.377_450.	POS	4.377	450.237
**2-Quinoxalinecarboxylic acid**	5.511_174.	NEG	5.511	174.043
Acidovorax_wautersii				
**ibho#16**	4.762_477.	POS	4.762	477.2368
**Milbemycin A4 oxime**	12.924_55	POS	12.924	555.3233
**1-Iodononane**	0.709_254.	POS	0.709	254.0522
**Valeroidine;**	7.883_241.	POS	7.883	241.168
**Dodecanoic acid**	9.234_182.	POS	9.234	182.1673
**16-Hydroxyhexadecanoic acid**	9.414_272.	NEG	9.414	272.2349
**5-[(8aS)-2,5,5,8a-tetramethyl-3-oxo-4a,6,7,8-tetra**	9.811_337.	POS	9.811	337.2616
**Omapatrilat**	7.46_408.1	POS	7.46	408.1176
**Papilioerythrinone**	5.338_580.	POS	5.338	580.3937
**Pentanoic acid, 5-(dipentylamino)-5-oxo-4-((3-qu**	7.699_441.	POS	7.699	441.2627
Other				
Flavobacterium_ummariense				
Chryseobacterium_aahli				
**L-Proline**	0.744_115.	POS	0.744	115.0635
**2-Ethyl-6-hydroxyhexanoic acid**	4.43_160.1	POS	4.43	160.1102
**(S)-AL 8810**	6.072_384.	POS	6.072	384.2123
**Neoannonin**	10.187_56	POS	10.187	564.4393
**Spiroxamine**	8.672_297.	POS	8.672	297.2668
**1-(2-Chloroethyl)-1-nitrosourea**	0.741_151.	POS	0.741	151.015
**Wedelolactone**	5.095_314.	NEG	5.095	314.0426
**Mofarotene**	10.169_43	POS	10.169	433.2984
**2-Methylindole**	5.079_131.	POS	5.079	131.0737
**2,6-dihydroxy-7-methoxy-1,1,4a-trimethyl-3,4,10,**	12.168_30	POS	12.168	304.1675
**7-[(2E,5E)-7-hydroxy-3,7-dimethylocta-2,5-dieno**	6.493_352.	POS	6.493	352.1091
**4-Amino-5-[[2-[[1-chloro-6-(diaminomethylidene**	5.343_392.	POS	5.343	392.1583
**Geraniol**	3.268_192.	POS	3.268	192.0926
**ent-16-B1-PhytoP**	7.495_308.	POS	7.495	308.1988
**TRIPHENYL PHOSPHATE**	8.273_326.	POS	8.273	326.0707
**2-[[(2R,3S)-2-(3,4-dihydroxyphenyl)-3,5-dihydroxy**	5.746_422.	NEG	5.746	422.1212
Odoribacter_laneus				
**Naloxone-3-glucuronide**	4.541_503.	POS	4.541	503.1799
**Hydroxymatairesinol glucuronide 1**	5.827_550.	POS	5.827	550.1702
**As-PL(14:0/16:0)**	6.812_930.	POS	6.812	930.4841
**2,3-Dihydro-6-hydroxy-2-(4-hydroxyphenyl)-5,7-**	2.494_316.	POS	2.494	316.0953
**N-(2,3-Dihydroxybenzoyl)-L-serine; 2,3-Dihydroxy**	1.849_241.	POS	1.849	241.0588
**E,e-Carotene-3,3'-dione**	10.1_564.3	POS	10.1	564.397
**(3E,7E,11E,15E)-5,9,13,17,18-pentahydroxy-4,6,8,1**	8.323_541.	POS	8.323	541.3248
**Diethyl succinate**	5.554_174.	POS	5.554	174.0895
**Methylguanidine**	0.719_73.0	POS	0.719	73.0641
**LysoPC(P-18:1(9Z)/0:0)**	9.757_505.	POS	9.757	505.3539
**Condelphine;**	7.194_449.	POS	7.194	449.278
**eurysterol B sulfonic acid**	12.929_51	POS	12.929	512.2815
Lautropia_mirabilis				
Corynebacterium_durum				

**Temsavir**	6.59_473.1	POS	6.59	473.1801
**Ethyl (E)-3-diethoxyphosphanylsulfinyloxybut-2-e**	6.826_298.	POS	6.826	298.0655
**2-O-Acetylpseudolycorine;**	6.396_331.	POS	6.396	331.1418
**Etoxazole**	5.076_359.	POS	5.076	359.1705
**Dobutamine**	5.077_301.	POS	5.077	301.1674
**N-(1-Adamantyl)urea**	3.268_194.	POS	3.268	194.1421
**Prohydrojasmon;**	8.946_254.	POS	8.946	254.1883
**Ecgonine-methyl-ester (EME)**	5.641_199.	POS	5.641	199.1211
**Dihydrogedunic Acid, Methyl Ester**	4.473_476.	POS	4.473	476.2416
**Docosanoic acid**	12.184_34	NEG	12.184	340.3342
**jaeschkeanadiol**	9.228_238.	POS	9.228	238.1934
**Glycerylmonooleate**	8.171_370.	NEG	8.171	370.272
**Cephaeline**	12.6_466.2	POS	12.6	466.285
**Diethylenetriaminetetraacetic acid**	0.658_335.	NEG	0.658	335.1328
**Sudan Red 7B**	7.773_379.	POS	7.773	379.1814
**reparixin**	4.896_283.	NEG	4.896	283.1241
**Pristanic acid**	10.777_29	POS	10.777	298.2871
**LID_265.1546_9.2**	4.072_264.	POS	4.072	264.1475
**Tyr-D-thr-gly-phe-leu-thr**	5.084_700.	POS	5.084	700.3405
**Coumarin 6**	4.382_350.	POS	4.382	350.1078
**ZOLAZEPAM**	9.231_286.	POS	9.231	286.1231
**gmelinol**	3.601_419.	POS	3.601	419.194
**Cantharidin**	5.553_196.	POS	5.553	196.0714
**Imepitoin**	0.759_279.	POS	0.759	279.0779
**Cyclopentobarbital**	5.96_234.1	POS	5.96	234.1006
**CAPREOMYCIN SULFATE**	7.566_750.	POS	7.566	750.3167
**Gly-Pro-Gly-Arg-Ala-Phe**	7.306_603.	POS	7.306	603.3149
Pedobacter_steynii				
**Fenamiphos**	1.499_303.	POS	1.499	303.1065
**PIM1(17:0/16:0)**	7.435_986.	NEG	7.435	986.594
**TG(14:1(9Z)/15:1(9Z)/20:5(5Z,8Z,11Z,14Z,17Z))[iso6**	8.657_806.	POS	8.657	806.6389
**4-Aminophenylphosphorylcholine**	2.493_274.	POS	2.493	274.1095
**Cer(d18:1/24:0)**	12.302_64	POS	12.302	649.6375
**6alpha,16beta-diacetoxy-hopan-22-ol**	10.43_544.	POS	10.43	544.4117
**N-(5-((4-(1H-Indol-3-yl)pyrimidin-2-yl)amino)-2-**	7.491_485.	POS	7.491	485.2527
**15-HETE-T**	7.967_427.	POS	7.967	427.2394
**Flavanone**	8.68_224.0	POS	8.68	224.0839
**[6,10a-dihydroxy-4-(hydroxymethyl)-4,7,11b-trim**	6.718_408.	NEG	6.718	408.2148
**Messagenin;**	10.8_444.3	POS	10.8	444.3611
**Ulimorelin**	9.796_538.	POS	9.796	538.2949
**4,4'-Dichlorodiphenyldichloroethane**	0.786_317.	NEG	0.786	317.9552
**Vanillin**	4.549_152.	POS	4.549	152.0475
**Cholesterol nitrite**	5.936_415.	POS	5.936	415.3448
**Cinacalcet**	7.631_357.	NEG	7.631	357.169
**1,7-bis(4-hydroxyphenyl)heptane-3,5-diol**	5.121_354.	POS	5.121	354.1248
**desferrioxamine D1**	5.496_602.	POS	5.496	602.3645
Other				
**Phenazine-1-carboxylate;**	6.491_224.	POS	6.491	224.0586
**1,2-(8R,9R-epoxy-octadec-13Z,15Z-dien-4,6-diyn**	5.063_614.	POS	5.063	614.3272
**Phthalic anhydride**	6.097_148.	POS	6.097	148.0162
**methyl 2-(4a,6,10a-trihydroxy-1-methyl-5,10-diox**	4.9_367.12	POS	4.9	367.1269
**1beta-Methylcarbapenem**	4.417_123.	POS	4.417	123.0686
**2-(4-(Methylamino)phenyl)benzo[d]thiazol-6-ol**	0.742_256.	POS	0.742	256.0672
**8H-Purin-8-one, 7-(2-butynyl)-7,9-dihydro-9-(6-**	3.334_379.	POS	3.334	379.1742
**6-Ethyl-4-methyl-3E,5E,7E-decatriene**	10.19_178.	POS	10.19	178.1723
**Isorhamnetin 3-glucuronide-7-sulfate**	3.783_572.	NEG	3.783	572.0475
**Purifolin**	6.271_520.	POS	6.271	520.1224
**19-methyl-5E,9E-eicosadienoic acid**	10.512_32	POS	10.512	322.2871

**2-[3-(sulfooxy)phenyl]acetic acid**	4.613_232.	POS	4.613	232.0037
**alpha-(4-Pyridyl-1-oxide)-N-tert-butylnitrone**	3.31_194.1	POS	3.31	194.1058
Empedobacter_falsenii				
**5Z,9Z,17Z-hexacosatrienoic acid**	10.155_39(	POS	10.155	390.3497
Parasutterella_excrementihominis				
**Pyridine, 4-(5-((1R)-1-(5-(3-chlorophenyl)-3-isoxa**	5.42_381.C	NEG	5.42	381.0981
**Urushiol II**	9.727_335.	POS	9.727	335.2823
**[1,3-dihydroxy-1-(7-methoxy-2-oxochromen-6-yl**	3.34_414.1	POS	3.34	414.1062
**Adouetine Z;**	5.063_699.	POS	5.063	699.344
**N-acetyl-O-methyltyrosine**	4.925_237.	POS	4.925	237.1002
**N-Deschlorobenzoyl indomethacin**	4.925_219.	POS	4.925	219.0897
**Sulfanilic acid**	3.6_173.01	POS	3.6	173.0148
**Pomaglumetad methionil**	0.646_366.	NEG	0.646	366.0561
Bacteroides_clarus				
Other				
**Olivetolcarboxylic acid**	3.626_224.	NEG	3.626	224.1048
**5,6,7-Trimethoxycoumarin**	6.036_253.	POS	6.036	253.0951
**8'-(Pyridin-2-ylmethoxy)spiro[2,4-dioxatricyclo[7.3**	6.746_407.	POS	6.746	407.1138
**5-Methyltetrahydropteroyltri-L-glutamate;**	5.143_717.	NEG	5.143	717.27
**Octadec-9-enal**	10.112_26(	POS	10.112	266.261
**Sebacil**	9.79_168.1	POS	9.79	168.1153
**[12-hydroxy-6-(hydroxymethyl)-10-methyl-2-(4-r**	9.282_364.	POS	9.282	364.2612
Yersinia_enterocolitica				
Sphingomonas_melonis				
**4-Hydroxy-6-methyl-3-(1H-pyrazol-3-yl)-2H-pyra**	3.47_192.C	NEG	3.47	192.0535
**(8R,9S,10R,13S,14S,17S)-2,2,17-Trihydroxy-10,13-c**	7.811_320.	POS	7.811	320.1984
**PE(17:1(9Z)/17:0)**	12.11_717.	POS	12.11	717.5308
**12-Hydroxyheptadeca-5,8,10-trienoic acid**	9.744_280.	POS	9.744	280.2039
**Doxorubicin-semiquinone**	6.046_547.	POS	6.046	547.2061
**[(1S,6S,7R)-6-acetyloxy-1-(3-methylbutanoyloxy)s**	5.836_462.	POS	5.836	462.1641
**1,4-Divinylbenzene**	8.054_130.	POS	8.054	130.0784
**Lithocholate 3-O-glucuronide**	8.21_552.3	NEG	8.21	552.3291
**N-[4-({3-[(3,5-Dimethoxyphenyl)amino]quinoxalin**	12.934_59(	POS	12.934	599.1855
**N,N-di-n-propyldopamine**	3.635_237.	POS	3.635	237.1731
**Aziridyl benzoquinone**	4.441_338.	NEG	4.441	338.1479
**11-bromo-dodecanoic acid**	0.817_278.	POS	0.817	278.0879
**PS(17:2(9Z,12Z)/22:6(4Z,7Z,10Z,13Z,16Z,19Z))**	8.795_817.	POS	8.795	817.492
**LacCer(d18:1/16:0)**	11.083_86	POS	11.083	861.6176
**Cystone A**	8.194_442.	POS	8.194	442.2727
**Cer(d18:2/15:0)**	10.881_52	POS	10.881	521.4816
**Pepsinostreptin; N-Isobutyrylpepstatin; Isobutyryl-**	9.722_671.	NEG	9.722	671.4443
**Gemcabene**	6.763_302.	POS	6.763	302.209
**3-Methyl-cis,cis-hexadienedioate; 3-Methyl-cis,cis**	4.035_156.	POS	4.035	156.0425
**Dicarbamoyl (2S)-2-aminopentanedioate**	2.415_233.	NEG	2.415	233.0646
**2-Descarboxy-betanidin;**	2.292_345.	POS	2.292	345.1103
**Hydroxytyrosol 3'-sulfate**	2.467_234.	NEG	2.467	234.0197
**2-[(2,6-Dichloro-4-methoxyphenyl)amino]phenyla**	8.121_325.	NEG	8.121	325.0275
**Triethanolamine; Trolamine;**	11.177_14(	POS	11.177	149.1054
**Ranolazine**	7.144_427.	POS	7.144	427.2471
**1-[[(2S,3R,11bR)-3-ethyl-9,10-dimethoxy-2,3,4,6,7**	9.585_481.	POS	9.585	481.2951
**Propamidine**	10.015_31	POS	10.015	312.1597
**Ethyl 3-O-beta-D-glucopyranosyl-butanoate**	1.665_294.	POS	1.665	294.1329
**Kainic Acid**	2.523_213.	POS	2.523	213.1004
**3-(5-phenylthiophen-2-yl)prop-2-ynyl Acetate**	1.355_256.	POS	1.355	256.056
**Fibraurecdyside A**	8.862_656.	NEG	8.862	656.3789
**Ustilagic acid**	5.529_784.	POS	5.529	784.407
**Taurolithocholic acid 3-glucuronide**	7.46_659.3	NEG	7.46	659.3347
**Cloethocarb**	2.487_259.	POS	2.487	259.0624

**PE(O-16:0/22:5(4Z,7Z,10Z,13Z,16Z))**	12.534_75	POS	12.534	751.5514
**Gabexate**	2.646_321.	POS	2.646	321.1689
**4-[(E)-2-[(1R,4aS,5R,6R,8aR)-6-hydroxy-5-(hydrox**	6.843_332.	POS	6.843	332.1986
**Benzamide, N-[2-[4-(cyclopropylcarbonyl)-3-meth**	7.12_559.2	NEG	7.12	559.2817
**2-Aminopropiophenone**	5.595_149.	NEG	5.595	149.0842
**Mrz 2266 BS**	7.514_325.	POS	7.514	325.204
**3,N(4)-Ethenodeoxycytidine**	3.319_251.	POS	3.319	251.0908
**XANTHONE**	6.726_196.	POS	6.726	196.0527
**8-Hydroxyguanosine**	4.01_299.0	POS	4.01	299.088
**PE(P-16:0/22:6(4Z,7Z,10Z,12E,16Z,19Z)(14OH))**	11.39_763.	POS	11.39	763.5144
**HMBOA-Glc**	1.558_357.	POS	1.558	357.106
**11-Deoxycortisol-3-carboxymethyloxime**	6.546_419.	POS	6.546	419.2308
**6-Methylquinoline**	3.292_143.	POS	3.292	143.0737
**lysine phosphoester**	3.3_226.07	POS	3.3	226.0718
**Didesmethyl doxepin**	3.769_251.	NEG	3.769	251.1316
**7a-Hydroxytestosterone**	9.232_304.	POS	9.232	304.21
**(2R,3R,4S,5R,6R)-2-[[7-[(2R,3R,4R,5S)-3,4-dihydro**	6.213_604.	POS	6.213	604.3258
**6-Chloro-5-(4-(1-hydroxycyclobutyl)phenyl)-1H-i**	4.397_341.	POS	4.397	341.0833
**Tuliposide B**	0.651_294.	POS	0.651	294.0957
**Tetrahydrocoptisine**	1.153_323.	POS	1.153	323.1151
**Firocoxib**	6.794_336.	POS	6.794	336.104
**N2-Methylguanosine**	2.682_297.	NEG	2.682	297.1072
**Hexanoylglycine**	4.115_173.	NEG	4.115	173.1053
**Hexahydroindan**	9.383_124.	POS	9.383	124.1254
**3-O-(6-O-alpha-D-Xylosylphospho-alpha-D-man**	3.26_554.1	POS	3.26	554.1237
**Certonardosterol M**	9.383_482.	NEG	9.383	482.3607
**1-[5-(Thiophen-2-ylmethoxy)-1H-indol-3-yl]prop**	0.737_286.	NEG	0.737	286.1137
**(2S,3S,4S,5R,6R)-6-[[(3S,6aR,6bS,8aS,14bR)-8a-car**	9.873_632.	NEG	9.873	632.3939
**Diflunisal glucuronide ether**	2.086_426.	POS	2.086	426.0769
**N-(eicosanoyl)-homoserine lactone**	10.02_395.	POS	10.02	395.3399
**5-Hydroxy-2-[2-methyl-3-(trifluoromethyl)anilino**	0.821_312.	POS	0.821	312.0723
**PC(22:5(7Z,10Z,13Z,16Z,19Z)/18:0)**	12.18_835.	POS	12.18	835.6084
**Daturmeteside A**	8.994_478.	NEG	8.994	478.3294
**N-(5-Fluoro-2-piperidin-1-ylphenyl)pyridine-4-ca**	4.824_315.	POS	4.824	315.1218
**Bruceantinol;**	7.195_606.	POS	7.195	606.2334
**Imazalil nitrate;**	2.29_360.1	POS	2.29	360.1912
**Teneligliptin**	8.052_426.	NEG	8.052	426.2191
**1-[2,4-dihydroxy-6-[(2S,3R,4S,5S,6R)-3,4,5-trihydr**	4.306_472.	POS	4.306	472.1322
**3-O-alpha-L-rhamnopyranosyl-3-hydroxy-5Z-doc**	9.522_530.	POS	9.522	530.3442
**3-Nitrobenzaldoxime**	1.676_166.	POS	1.676	166.0381
**Malonylcarnitine**	3.281_247.	POS	3.281	247.1058
Comamonas_terrigena				
**N-(2(S)-(Acetylthiomethyl)-3-(2-methylphenyl)-1-**	3.689_411.	POS	3.689	411.1543
**2alpha,3alpha-(Difluoromethylene)-5alpha-andros**	8.478_366.	POS	8.478	366.2382
**Acedapsone**	4.38_332.0	POS	4.38	332.0828
**Semiplenamide B**	9.906_407.	POS	9.906	407.34
**3'-Deoxydihydrostreptomycin;**	7.561_567.	POS	7.561	567.2865
Coprobacter_fastidiosus				
**PE(15:1(9Z)/0:0)**	6.438_437.	POS	6.438	437.253
**Apiin**	5.949_564.	POS	5.949	564.1479
**2-Amino-1-phenylpropan-2-ol**	4.138_151.	POS	4.138	151.0998
**(-)-11-hydroxy-9,10-dihydrojasmonic acid 11-beta**	5.279_390.	NEG	5.279	390.189
**Pelargonidin 3-O-(6-O-malonyl-beta-D-glucoside**	2.645_519.	POS	2.645	519.1125
**Flugestone**	10.36_364.	POS	10.36	364.2065
**(S)-malyl alpha-D-glucosaminide**	0.646_295.	NEG	0.646	295.09
**Cimimanol B**	9.458_636.	NEG	9.458	636.3889
**BEKANAMYCIN SULFATE**	5.057_603.	POS	5.057	603.2039
Corynebacterium_tuberculostearicum				

3-Nitrobenzaldehyde	2.577_151.	POS	2.577	151.0272
PKODiA-PS	8.18_665.3	POS	8.18	665.3148
Esmeraldic acid;	4.428_518.	POS	4.428	518.1577
Cefradine	7.133_349.	POS	7.133	349.1094
Reichsteins Substance S	9.124_346.	POS	9.124	346.2144
TWS119	4.753_318.	POS	4.753	318.1102
2-Methylthiazolidine-4-carboxylic acid	1.06_147.C	POS	1.06	147.0356
Zatebradine	4.662_456.	POS	4.662	456.2627
8-iso-15-keto Prostaglandin F2a	8.434_334.	POS	8.434	334.212
Pristimerin	6.963_486.	POS	6.963	486.2768
DL-Methionine-4-antipyrineamide	5.186_334.	POS	5.186	334.1463
NCGC00169031-03![6-(2-formamido-3-methylbut	5.531_400.	POS	5.531	400.1863
Pratosartan	8.194_426.	POS	8.194	426.2189
1alpha-hydroxy-24-(dimethoxyphosphoryl)-25,26,	7.595_466.	NEG	7.595	466.2855
Azadirachtin	6.248_720.	POS	6.248	720.2646
Prevotella_histicola				
4-Hydroxy-5-[3-[(1-methylcyclohexyl)methoxy]ph	6.313_319.	POS	6.313	319.1247
Sphingofungin A	4.433_431.	POS	4.433	431.2996
11-O-Demethyl-17-O-deacetylvindoline;	7.897_400.	NEG	7.897	400.2007
delta9-Tetrahydrocannabinol hemisuccinate	7.822_414.	POS	7.822	414.2407
Cer(d18:1/22:0)	10.787_62	POS	10.787	621.6061
Piperic Acid	4.045_235.	POS	4.045	235.0846
Isophthalamide, N,N'-bis(2,3-dihydroxypropyl)-5-(	3.383_790.	NEG	3.383	790.8731
Physostigmine	3.206_275.	POS	3.206	275.1633
Fenbuconazole;	6.008_336.	POS	6.008	336.1145
Sirtinol	4.556_394.	POS	4.556	394.1678
9-hydroxy-1,4a-dimethyl-7-propan-2-yl-2,3,4,9,1(	6.989_333.	POS	6.989	333.2294
Iodoantipyrine	0.637_313.	NEG	0.637	313.9918
3,4-Dihydro-7-methoxy-2-methylene-3-oxo-2H-1	4.353_235.	POS	4.353	235.0484
1-(2-Hydroxyethyl-2-hydroxymethyl)-5-nitro-imic	2.41_187.C	NEG	2.41	187.0592
S-Adenosyl-4-methylthio-2-oxobutanoate; S-Ade	2.95_398.1	NEG	2.95	398.1146
Ciglitazone	5.886_333.	POS	5.886	333.1402
Arthrobactin	8.245_454.	POS	8.245	454.2697
LMPK12090048	4.681_356.	POS	4.681	356.0534
(S)-Isocorydine	4.047_341.	POS	4.047	341.1624
Xanthohumol	3.386_354.	NEG	3.386	354.1428
Glutathione thiol	2.779_339.	POS	2.779	339.0556
Glycolic acid	3.296_76.C	POS	3.296	76.01617
8-Isoprostaglandin F1	6.891_373.	POS	6.891	373.2827
Pangamic Acid Sodium	5.912_320.	POS	5.912	320.121
Latanoprost acid	6.722_390.	POS	6.722	390.2403
N-desmethyl loperamide	8.452_462.	POS	8.452	462.2069
Dimethyl succinate	4.087_146.	POS	4.087	146.0581
Urea, N'-(2,4-difluorophenyl)-N-((4-(2,2-dimethyl	4.436_430.	POS	4.436	430.2779
Other				
Abbott-195773	6.004_765.	POS	6.004	765.4191
7-Chloro-N-[3-(2-nitroimidazol-1-yl)propyl]quino	1.674_331.	POS	1.674	331.0837
s-Nitroglutathione	2.493_352.	NEG	2.493	352.069
(2S,3R,4S,5S,6R)-2-[[(1S,4aR,7aS)-7-(hydroxymeth	4.906_347.	POS	4.906	347.1563
(6R)-vitamin D2 6,19-sulfur dioxide adduct / (6R)-e	8.378_460.	NEG	8.378	460.3034
Desglymidodrine	4.899_197.	POS	4.899	197.1055
Indanidine	4.904_215.	POS	4.904	215.1161
(S)-Tembetarine;	10.02_344.	POS	10.02	344.1859
Apstatin	7.756_459.	POS	7.756	459.2494
Ectoine	13.066_14	POS	13.066	142.0744
pyrimidin-4-ol	1.074_96.C	NEG	1.074	96.03235
Butylcarbamoyl-(4-methylphenyl)sulfonylcarbamic	3.922_314.	POS	3.922	314.0939
Efonidipine	5.756_631.	POS	5.756	631.2423

Other				
**1-(2-methylpropylidene)-4-(propan-2-yl)-1H,2H,3**	7.581_328.	POS	7.581	328.1885
**Sulfate**	0.638_97.9	NEG	0.638	97.96737
**(3a,5b,7a,12a)-24-[(carboxymethyl)amino]-1,12-di**	7.888_641.	POS	7.888	641.3415
**2-Hydroxy-imipramine glucuronide**	7.284_472.	POS	7.284	472.2213
**Imicyafos;**	7.507_304.	POS	7.507	304.111
**4-Methoxycinnamaldehyde**	5.752_162.	POS	5.752	162.0683
**TRELAGLIPTIN**	7.124_357.	POS	7.124	357.1606
**5-(3',4'-Dihydroxyphenyl)-gamma-valerolactone**	6.466_208.	POS	6.466	208.0736
**PC(18:1(9Z)/P-18:0)**	12.623_77	POS	12.623	771.614
**Picolinoylglycine**	3.492_180.	POS	3.492	180.0538
**N-Arachidonoylglycine**	9.525_361.	NEG	9.525	361.2618
**4-(8-prop-1-en-2-yl-3,4,8,9-tetrahydro-2H-furo[2**	4.61_341.1	POS	4.61	341.1626
**7-Ethoxycoumarin**	4.988_190.	POS	4.988	190.0632
**S-Methyl N,N-Diethylthiocarbamate**	5.089_147.	POS	5.089	147.072
**Terconazole**	6.706_531.	POS	6.706	531.1798
**Miglustat**	3.258_219.	POS	3.258	219.1474
**Hydroxyalbendazole; Methyl [5-(3-hydroxypropyls**	3.844_281.	POS	3.844	281.0835
Clostridium_hiranonis				
**Dinitrosopentamethylenetetramine;**	3.341_186.	POS	3.341	186.0871
**Tame**	5.039_342.	POS	5.039	342.1364
**Hypusine**	0.579_233.	POS	0.579	233.1741
**trans-Crocetin (beta-D-glucosyl) (beta-D-gentibio**	5.637_814.	POS	5.637	814.3277
**4-(8-Amino-1,2,3,4-tetrahydro-2-methyl-4-isoqui**	6.617_254.	NEG	6.617	254.1418
Other				
**1-(2,4,6-Trimethylphenoxy)propan-2-amine**	3.618_193.	POS	3.618	193.147
**Blood group B trisaccharide**	6.847_488.	POS	6.847	488.1731
**eudesmin**	5.564_408.	POS	5.564	408.153
**Dihomo-gamma-linolenoyl dopamine**	9.801_441.	POS	9.801	441.3245
**5-[(Z)-14-(3,5-dihydroxyphenyl)tetradec-10-enyl]**	8.679_412.	POS	8.679	412.2616
**N1-Methyl-2-pyridone-5-carboxamide**	1.859_152.	POS	1.859	152.0588
**2-Amino-2'-fluoro-5-nitrobenzophenone**	5.689_260.	POS	5.689	260.0602
**4-O-methylolivetolcarboxylic acid**	5.502_238.	POS	5.502	238.1207
**D-Erythritol 1-phosphate;**	5.04_202.0	POS	5.04	202.0245
**5-(Acetylamino)-N,N'-bis(2,3-dihydroxypropyl)-2,4**	2.517_746.	POS	2.517	746.8442
**Penicilloic acid**	0.716_352.	NEG	0.716	352.1084
**2,2-Dichloro-12-(4-chlorophenyl)dodecanoic acid**	0.684_378.	NEG	0.684	378.0928
**1-(3-(4-Phenylbenzyl)-4-hydroxychroman-7-yl)cy**	4.307_428.	POS	4.307	428.1985
**Oxoguanine**	4.383_165.	POS	4.383	165.0285
**DL-Metanephrine**	4.427_197.	POS	4.427	197.1032
**(2S)-1-[2-[[(2S)-Pyrrolidine-2-carbonyl]amino]ace**	2.244_269.	POS	2.244	269.1376
**Talniflumate**	3.772_414.	POS	3.772	414.0844
**Peimisine**	8.266_427.	POS	8.266	427.3085
**15-Deoxy-d12,14 -Prostaglandin J2**	3.766_338.	NEG	3.766	338.1843
**Ustiloxin; Ustiloxin A;**	7.965_673.	POS	7.965	673.2637
**Nonoxynol-9**	9.301_616.	POS	9.301	616.4185
**13-dimethylarsinoyl-tridecanoic acid**	1.05_334.1	NEG	1.05	334.1503
**3,7-Dimethyl-2,6-octadienal**	10.084_15	POS	10.084	152.1203
**Lasofoxifene**	3.638_413.	POS	3.638	413.2355
Ilumatobacter_fluminis				
Other				
Other				
**(1S,4Ar,5R,8S,8aS)-2,5-dimethyl-8-propan-2-yl-4,4**	6.963_238.	POS	6.963	238.1935
**1-Benzylimidazole**	3.298_158.	POS	3.298	158.0846
**Avocadyne 1-acetate**	8.72_348.2	POS	8.72	348.2276
**Pantheric Acid A**	10.232_35	POS	10.232	352.2977
**LMPK12113227**	6.43_426.1	POS	6.43	426.131
**4-Methylumbelliferyl glucuronide**	5.044_352.	POS	5.044	352.0766

**Diisopentyl thiomalate**	1.686_290.	POS	1.686	290.1566
**NCGC00347365-02!5-hydroxy-2,2-dimethyl-10-(2**	3.328_334.	POS	3.328	334.1198
**4-Toluic acid**	4.467_136.	NEG	4.467	136.0525
Eubacterium_infirmum				
**1,7-bis(4-hydroxyphenyl)-5-[(2R,3R,4S,5S,6R)-3,4,5**	4.73_498.1	POS	4.73	498.1858
**serine thiol**	5.006_137.	POS	5.006	137.0149
**(+-)-Carbovir**	3.01_247.1	POS	3.01	247.1079
**17alpha-Chloroethynylestradiol; 21-Chloro-19-nor**	6.9_330.13	POS	6.9	330.1399
**Succinic acid, sulfo-, 1,4-dioctyl ester**	9.302_422.	NEG	9.302	422.234
**Indoleacrylic acid**	3.297_187.	POS	3.297	187.0636
**Lobeline Hydrochloride**	4.719_359.	POS	4.719	359.1845
**LMST04010294**	7.721_424.	NEG	7.721	424.2822
**Cortisol**	6.533_362.	POS	6.533	362.2091
**4-Nitrophenyl-3-ketovalidamine; p-Nitrophenyl-3**	5.518_296.	POS	5.518	296.1008
**Pyrenophorol**	4.093_329.	POS	4.093	329.1838
**Chlorambucil-tertiary butyl ester**	5.948_359.	POS	5.948	359.1403
Other				
**PC(18:3(9Z,12Z,15Z)/P-18:1(11Z))**	11.698_76	POS	11.698	765.5666
**N-Phenylsuccinimide**	4.954_175.	POS	4.954	175.0635
**Ethylmethylhydroxypyridine succinate**	6.422_251.	POS	6.422	251.0793
**1-[2-Amino-6-(3,5-dimethoxyphenyl)pyrido[2,3-d**	5.965_396.	POS	5.965	396.1895
**5-[(Z)-12-(3,5-dihydroxyphenyl)dodec-8-enyl]ben**	6.656_406.	POS	6.656	406.2104
**Muzolimine**	4.37_271.0	POS	4.37	271.0269
**Arachidoyl Ethanolamide**	8.639_355.	POS	8.639	355.345
**(Z)-2-((R)-1-((S)-1-(2-Chlorophenyl)-2-methoxy-2**	12.28_503.	POS	12.28	503.1147
**Cefoperazone A**	4.625_663.	POS	4.625	663.1533
**Setipiprant**	0.712_402.	NEG	0.712	402.1372
**N-(4-Fluorophenyl)-4,5-dimethyl-6-[(1S)-1-methy**	10.143_36	POS	10.143	362.1908
**4-amino-MX**	3.822_267.	POS	3.822	267.122
**Decylubiquinone**	7.819_322.	POS	7.819	322.2131
**beta-Tocopherol**	10.793_41	POS	10.793	416.3658
**Glycine anhydride**	6.314_114.	POS	6.314	114.0431
**Safflomin C**	5.943_614.	POS	5.943	614.1637
**3h-Adrenaline**	5.564_185.	NEG	5.564	185.1052
**Cycloxydim-TP BH 517-TSO E/Z-isomer**	7.75_341.1	POS	7.75	341.166
Olsenella_umbonata				
Brevundimonas_halotolerans				
**Flavin mononucleotide (FMN)**	4.198_456.	NEG	4.198	456.1089
**5,6-dihydropenicillic acid**	1.014_172.	NEG	1.014	172.0737
**Toluene-4-sulfonate; Tosylate;**	3.234_172.	NEG	3.234	172.0196
**benfluorex**	3.231_351.	POS	3.231	351.143
Clostridium_chartatabidum				
**Homovanillyl alcohol glucuronide**	3.65_344.1	POS	3.65	344.1123
**16alpha-Hydroxy DHEA 3-sulfate**	6.11_384.1	NEG	6.11	384.1607
**Cocapropylene**	6.841_331.	POS	6.841	331.1779
**PS(O-18:0/18:4(6Z,9Z,12Z,15Z))**	10.656_76	POS	10.656	769.5253
**Cyclandelate**	8.188_276.	POS	8.188	276.1722
**Moxaverine**	5.964_307.	POS	5.964	307.1571
**Mdz-glucuronide**	5.953_517.	POS	5.953	517.1058
Treponema_porcinum				
**Trityl candesartan cilexetil**	4.797_852.	POS	4.797	852.3632
**5,6-Dihydrouridine**	3.175_246.	POS	3.175	246.0852
**Ethylenediaminetetraaceticacid**	3.141_292.	POS	3.141	292.0901
**Benzylamine**	3.491_107.	POS	3.491	107.0737
**Sativanine B;**	10.471_51	POS	10.471	518.2918
**1-(2-methoxy-heneicosanyl)-sn-glycero-3-phosph**	10.456_58	NEG	10.456	583.3852
**15,17-dihydroxy-9-methyl-4,10-dioxatricyclo[11.4**	4.428_306.	NEG	4.428	306.1096
**Pulcherriminic acid; 2,5-Dihydroxy-3,6-bis(2-meth**	5.123_256.	POS	5.123	256.1423

**Celgosivir**	1.509_259.	POS	1.509	259.1421
**(2S,3R,4R,5R,6R)-2-methyl-6-[[(2R,3S,4S,5R,6R)-3,4**	8.645_502.	POS	8.645	502.237
**Carbuterol**	8.861_267.	NEG	8.861	267.1583
**4,4-Difluoro-17beta-hydroxyandrost-5-en-3-one**	7.501_380.	POS	7.501	380.2171
**Xylitol**	0.723_174.	POS	0.723	174.0507
**Ribitol**	0.737_152.	POS	0.737	152.0687
**Pentadecylamine**	7.542_227.	POS	7.542	227.2614
**N-Acetyl-S-(2-cyanoethyl)-L-cysteine**	4.473_216.	POS	4.473	216.0572
**Fidexaban**	3.162_526.	POS	3.162	526.1767
**trifluoroacetyl-l-lysyl-l-alaninanilide**	4.474_388.	POS	4.474	388.1736
**Lophirone J**	4.245_400.	POS	4.245	400.1308
**(3E,4R)-3-[2-[(1R,4aS,5R,6R,8aS)-6-hydroxy-5-(hy**	6.908_388.	POS	6.908	388.1634
**Secnidazole**	0.95_185.C	POS	0.95	185.0803
Other				
**Anandamide (20:2, n-6)**	9.441_351.	POS	9.441	351.3137
**Lurtotecan**	8.588_518.	NEG	8.588	518.2162
**Peregrinol diphosphate; (13E)-9-Hydroxy-8alpha-l**	5.85_468.2	POS	5.85	468.2044
**Isohomovanillic acid**	4.053_182.	POS	4.053	182.0581
**Promegestone**	9.861_326.	NEG	9.861	326.2246
**2,3-Dinor-8-epi-prostaglandin F2a**	7.26_343.2	POS	7.26	343.2357
**1H-Imidazole-1-ethanol, 2-nitro-alpha-((2,2,2-trifl**	3.408_337.	POS	3.408	337.0481
**Piceid 4'-sulfate**	5.063_470.	POS	5.063	470.088
**Glycylphenylalanylleucylglycine**	6.752_392.	POS	6.752	392.2061
**7,8-Dimethoxy-1-(4-nitrophenyl)-3,5-dihydro-2,3**	3.052_341.	POS	3.052	341.1017
**Arachidonic acid**	9.823_304.	POS	9.823	304.2401
**N-[4-Methoxy-5-[[4-(1-methylindol-3-yl)pyrimidi**	7.367_485.	NEG	7.367	485.2526
**NCGC00386067-01!2-[2-(19-acetamido-16,18-dih**	9.163_609.	POS	9.163	609.3512
**N-arachidonoyl alanine**	8.203_375.	POS	8.203	375.2773
Gemmatimonas_aurantiaca				
**N-Acetyl-L-leucine**	4.618_173.	POS	4.618	173.1054
**6**	9.516_390.	NEG	9.516	390.2173
**Cyclo(-D-Tyr-Arg-Gly-Asp-Cys(carboxymethyl)-OI**	5.093_668.	POS	5.093	668.2252
**NA**	0.713_89.C	NEG	0.713	89.04766
**Portulacaxanthin I;**	2.638_324.	POS	2.638	324.0956
**NCGC00347728-02!3-(5-hydroxy-2,2,7,8-tetrametl**	1.712_432.	POS	1.712	432.1498
**DG(13:0/22:6(4Z,7Z,10Z,13Z,16Z,19Z)/0:0)[iso2]**	10.069_59	POS	10.069	598.4581
**Val-Gly-Val-Ala-Pro-Gly**	3.819_498.	POS	3.819	498.2808
**Lefradafibanum**	7.151_439.	POS	7.151	439.1743
**Flavone**	0.665_222.	NEG	0.665	222.0674
**JACAGLABROSIDE B**	4.346_615.	POS	4.346	615.1933
**Calpeptin**	9.619_362.	POS	9.619	362.2221
**Quercetin 7,3',4'-trimethyl ether 3-sulfate**	4.835_424.	NEG	4.835	424.0466
**1,3-Dimethyluric acid**	2.924_196.	POS	2.924	196.0599
**Midazolam**	2.627_325.	POS	2.627	325.0798
**Pemptoporphyrin**	8.427_536.	POS	8.427	536.2429
**Tafenoquine**	7.292_463.	NEG	7.292	463.2104
Other				
**Indole-3-acetaldehyde**	5.079_159.	POS	5.079	159.0687
**N-succinyl-chitosan**	6.283_583.	POS	6.283	583.2207
**Vernomenin;**	6.713_276.	POS	6.713	276.0997
**gamma-Glutamylisoleucine**	3.101_260.	POS	3.101	260.1372
**3-(N-Maleimidopropionyl)biocytin**	6.703_523.	POS	6.703	523.2109
**3-Methoxycinnamic acid**	5.648_160.	POS	5.648	160.0527
**Dasabuvir**	6.854_493.	POS	6.854	493.169
**S-Carboxymethyl-L-cysteine**	0.732_197.	NEG	0.732	197.0359
Blautia_glucerasea				
**Proscillaridin**	9.717_530.	NEG	9.717	530.2874
**(4-Chlorophenyl) (1R)-6-chloro-1-(4-methoxyphe**	1.083_466.	POS	1.083	466.0831

**di-trans,poly-cis-Hexaprenyl diphosphate; ditrans,**	5.049_586.	POS	5.049	586.3203
**Indole-3-pyruvic acid**	1.197_203.	POS	1.197	203.0561
**Ibrutinib metabolite M37**	5.288_474.	NEG	5.288	474.2016
**Phenylacetic acid**	5.643_136.	POS	5.643	136.0527
Arthrobacter_ramosus				
**5-Amino-4-chloro-2-(2,3-dihydroxyphenyl)-3(2H)**	4.196_253.	POS	4.196	253.0264
**Refametinib**	3.322_572.	NEG	3.322	572.0113
**Lentialexin**	2.948_120.	NEG	2.948	120.0575
**Phenyllactic acid**	2.948_166.	NEG	2.948	166.0631
**Methoxyacetaldehyde**	2.279_74.0	POS	2.279	74.03692
**PRI_247.1079_16.2**	4.387_246.	POS	4.387	246.1006
**PS(12:0/14:1(9Z))**	9.475_649.	POS	9.475	649.3959
**Propranolol**	3.681_259.	POS	3.681	259.1572
**LysoPC(14:0/0:0)**	10.136_46	NEG	10.136	467.3012
**Metalaxyl-M-TP CGA108906**	3.049_295.	NEG	3.049	295.1055
**methyl 2-(9-hydroxy-1-methyl-5,10-dioxo-3,4-dil**	5.978_333.	POS	5.978	333.1231
**Mosesin 4;**	8.836_640.	NEG	8.836	640.42
**Anhydroicaritin 3-rhamnosyl-(1->2)-rhamnoside**	7.992_660.	POS	7.992	660.2434
**PG(20:2(11Z,14Z)/0:0)**	10.03_536.	POS	10.03	536.3121
**Fenarimol;**	6.376_330.	POS	6.376	330.0314
Arsenicicoccus_bolidensis				
Chryseobacterium_carnipullorum				
Acholeplasma_axanthum				
Adhaeribacter_aerophilus				
**N-Methyl-L-proline**	5.255_129.	POS	5.255	129.0792
**3,3`-Biphenol**	4.966_186.	NEG	4.966	186.0681
**Propoxur**	5.232_209.	NEG	5.232	209.1052
**FAHFA(18:2/15-O-18:2)**	10.082_55	POS	10.082	558.465
**Dimethisterone**	9.997_340.	NEG	9.997	340.2401
**Linkckoside F**	8.821_624.	NEG	8.821	624.389
**12-Epi-Salvinorin A**	6.388_432.	POS	6.388	432.1789
**4-Keto-4'-hydroxyalloxanthin**	9.596_594.	POS	9.596	594.3708
**Tatridin B**	7.774_264.	POS	7.774	264.136
**8-[2-(3,4-dihydroxy-2,5-dimethoxyoxolan-3-yl)etl**	5.33_430.2	POS	5.33	430.2582
**Lanopepden**	4.929_479.	POS	4.929	479.2647
**N-[5-[2-(3-Acetylanilino)-1,3-thiazol-4-yl]-4-metl**	5.162_434.	NEG	5.162	434.0878
Allobaculum_stercoricanis				
**8-Hydroxyalanylclavam;**	0.634_216.	NEG	0.634	216.0744
**Furofuran**	3.505_108.	POS	3.505	108.0214
**Amoxicilloyl**	4.007_381.	NEG	4.007	381.1363
**1H-Purine-2,6-dione, 3,7-dihydro-1,3-dimethyl-7-**	3.994_327.	POS	3.994	327.1681
**Cicletanine**	6.332_261.	POS	6.332	261.0555
**Benzo[d][2,1]benzothiazepine**	1.253_211.	POS	1.253	211.0458
**Dodecylamine**	10.186_18	POS	10.186	185.2146
Clostridium_ghonii				
**Trimetrexate**	5.999_369.	POS	5.999	369.1787
**LMST04010134**	9.857_388.	POS	9.857	388.2613
**NCGC00381416-01![3-methyl-1-[3-methyl-1-oxo-**	6.248_527.	POS	6.248	527.2925
**cefetamet**	4.393_397.	POS	4.393	397.0515
Other				
Ruminococcus_gnavus				
**[3-[2-Aminoethoxy(hydroxy)phosphoryl]oxy-2-hy**	8.776_453.	POS	8.776	453.2858
**16-a-Hydroxypregnenolone**	8.947_332.	POS	8.947	332.235
**Glasdegib**	6.363_374.	POS	6.363	374.1839
**Gcdcs**	7.771_529.	POS	7.771	529.2719
**1-[1-Butyl-2-oxo-4-[3-(pyridin-3-ylmethoxy)pher**	9.488_603.	NEG	9.488	603.3234
**Glycocholic acid 3-sulfate**	6.596_545.	POS	6.596	545.2666
**PS(18:4(6Z,9Z,12Z,15Z)/18:4(6Z,9Z,12Z,15Z))**	7.966_775.	POS	7.966	775.4402

**Arbekacin**	8.503_552.	NEG	8.503	552.3116
**(Z)-7-[(1S,4R,5R,6R)-5-[(E,3S)-4-(4-Fluorophenoxy**	3.685_404.	POS	3.685	404.1979
**Parbendazole**	4.327_247.	NEG	4.327	247.1321
**PS(P-20:0/20:2(11Z,14Z))**	12.177_82	POS	12.177	827.6043
**Ceftobiprole medocaril**	3.249_690.	POS	3.249	690.1196
**Docosanamide**	10.667_33	POS	10.667	339.35
**Cer(d18:0/22:0)**	0.716_623.	POS	0.716	623.6229
**Cer(d18:2/22:0)**	12.852_61	POS	12.852	619.5904
**2_Hydroxy_3_methylbutyric acid**	3.082_140.	POS	3.082	140.0452
**5,7-Dihydroxy-2-(4-hydroxyphenyl)-3-[(2S,5S,6R)-**	6.225_448.	POS	6.225	448.1009
**LMPK12120188**	5.816_238.	POS	5.816	238.0993
**Lovastatin putative derivative C26H38O6 observed**	8.248_446.	POS	8.248	446.2666
Ruminococcus_bromii				
Pseudomonas_bauzanensis				
**2-Hydroxy-1,4-benzoxazin-3-one sulfate**	3.045_244.	NEG	3.045	244.9991
Lactobacillus_apodemi				
**LMPK12113020**	6.913_344.	POS	6.913	344.0531
Eubacterium_pyruvativorans				
Sphingobium_limneticum				
**PA(19:0/0:0)**	10.181_45	NEG	10.181	452.2901
**Lithocholic acid glycine conjugate**	5.964_433.	POS	5.964	433.3192
**eribaxaban**	1.009_484.	POS	1.009	484.1301
**3,4-dihydroxyphenyl glycol**	3.973_152.	POS	3.973	152.0474
**2-deoxy-20-hydroxyecdysone 22-phosphate**	6.794_544.	POS	6.794	544.2828
**Tetranor-(+)-S-145**	4.338_323.	POS	4.338	323.119
**Glycochenodeoxycholic acid**	8.622_449.	POS	8.622	449.3141
**N,O-Didesmethyltramadol**	6.124_235.	POS	6.124	235.1582
**5-valerolactone**	5.223_100.	POS	5.223	100.0526
**cis-corossolone**	10.019_57	POS	10.019	578.4553
**(2R,3S,4S,5R,6S)-2-[[(2S,3R,4R)-3,4-dihydroxy-4-(h**	5.703_478.	NEG	5.703	478.1692
**(p-SCN-Bn)-dota**	7.127_551.	POS	7.127	551.2041
**Cetilistat**	9.811_401.	POS	9.811	401.2928
**4-methoxy-9-(3-methylbut-2-enyl)furo[3,2-g]chro**	6.55_301.1	POS	6.55	301.1312
**7-ethenyl-1,4a,7-trimethyl-6-oxo-2,3,4,8,8a,9,10,1**	8.219_316.	POS	8.219	316.2037
**Sinefungin**	5.428_381.	POS	5.428	381.1808
**3-Methyloxindole**	4.306_147.	POS	4.306	147.0684
**LMST01010336**	9.443_476.	POS	9.443	476.3507
**2-Ethylsulfanyl-1H-benzoimidazole**	5.067_178.	POS	5.067	178.0567
**RO4929097**	4.662_469.	POS	4.662	469.1409
**1-O-(2R-hydroxy-pentadecyl)-sn-glycerol**	9.493_318.	POS	9.493	318.2769
**Senecionine**	8.326_335.	POS	8.326	335.1766
**Bicyclomycin benzoate**	6.476_406.	POS	6.476	406.1376
**Nocardicin D;**	4.432_485.	POS	4.432	485.1439
**Norepinephrine sulfate**	2.815_249.	POS	2.815	249.0307
**(8R,9R,10R,13S,14S)-10,13-Dimethyl-4,5,6,7,8,9,11,**	9.071_290.	POS	9.071	290.2246
**7-Methyl-2'-deoxyguanosine-3'-monophosphate**	6.345_363.	POS	6.345	363.0938
**Prolyl-phenylalanyl-arginine-4-nitroanilide**	12.681_53	POS	12.681	538.2651
**20-acetoxy-clavulone I**	4.371_504.	POS	4.371	504.2338
**N7-(2-Carbamoyl-2-hydroxyethyl)guanine**	5.029_238.	POS	5.029	238.0818
**Ciproxifan**	5.615_270.	NEG	5.615	270.1366
**Methaniazide**	2.465_231.	POS	2.465	231.0312
**19-phenyl-15E-nonadecenoic acid**	9.771_372.	POS	9.771	372.3028
**13,14-Dihydro-15-keto Prostaglandin J2**	9.044_316.	NEG	9.044	316.2038
**Glycoperine;**	4.575_391.	POS	4.575	391.1267
**N(epsilon)-(carboxyethyl)lysine**	1.283_218.	NEG	1.283	218.1265
**Prostaglandin J2**	9.169_351.	POS	9.169	351.2409
**NCGC00381425-01!8-hydroxy-8-(3-octyloxiran-2-**	8.903_278.	POS	8.903	278.2246
**Amcinonide**	3.204_502.	POS	3.204	502.2391

**Uncarine c**	3.257_390.	POS	3.257	390.1538
**Isopongaflavone**	3.325_334.	NEG	3.325	334.1197
**Etonogestrel**	7.571_324.	POS	7.571	324.209
**TG(12:0/20:4(5Z,8Z,11Z,14Z)/22:3(10Z,13Z,16Z))[isc**	12.424_87(	POS	12.424	876.7184
**N-[(3s)-2-Oxotetrahydrofuran-3-Yl]butanamide**	0.746_171.	POS	0.746	171.0898
**6,6'-Methylenebis(2,2-dimethyl-4-methanesulfoni**	4.419_518.	NEG	4.419	518.155
**Prenylcysteine**	4.271_189.	POS	4.271	189.0823
Megasphaera_elsdenii				
**RCTR1**	6.46_665.2	POS	6.46	665.2987
Other				
**S-(6-Purinyl)cysteine**	4.666_239.	POS	4.666	239.0472
**methyl (2E)-3-[18-(acetyloxy)-7-(furan-3-yl)-17-h**	7.993_544.	POS	7.993	544.2331
**2-Methyl-5-nitroimidazole**	3.731_127.	POS	3.731	127.0383
**1-Naphthyl isocyanate**	3.292_169.	POS	3.292	169.0529
**Penitrem C;**	7.434_601.	POS	7.434	601.2984
**26-Hydroxybrassinolide;**	8.194_496.	POS	8.194	496.3407
**Captopril-cysteine disulfide**	4.431_336.	POS	4.431	336.0814
**PKODA-PS**	7.127_705.	POS	7.127	705.3849
**2-Cyclohexen-1-one, 3-(15-hydroxypentadecyl)-2,**	10.797_36	POS	10.797	364.3341
**Cholesterol carbonate**	9.036_430.	POS	9.036	430.3449
**N-(3-Benzooxazol-2-yl-4-hydroxy-phenyl)-2-p-tc**	5.461_374.	POS	5.461	374.126
**iodamide**	8.391_627.	POS	8.391	627.7834
**H-D-Val-Leu-Lys-pNA**	4.394_478.	POS	4.394	478.2892
Ruminobacter_amylophilus				
**Ligusticide**	4.627_212.	POS	4.627	212.0816
**(S)-3-(4-Methoxyphenyl)-N-((S)-1-((R)-2-methylc**	7.865_580.	POS	7.865	580.29
**17a-Hydroxyprogesterone**	4.242_330.	POS	4.242	330.2193
**3-(5-(4-(1H-Imidazol-1-yl)phenyl)-1-(4-carbamoy**	5.094_414.	POS	5.094	414.1694
**dTDP-beta-L-rhodinose;**	3.821_516.	NEG	3.821	516.0934
Olsenella_scatoligenes				
**CCR4 Antagonist**	8.574_467.	NEG	8.574	467.3246
**Taurodeoxycholic Acid**	8.699_481.	POS	8.699	481.2866
**9-Ethylguanine**	0.737_179.	POS	0.737	179.0809
**Meprednisone**	4.764_372.	POS	4.764	372.1896
**Acetoacetate**	1.087_102.	POS	1.087	102.0319
Other				
**2,5,5,8a-tetramethyl-4a,5,6,7,8,8a-hexahydro-4H-c**	6.779_349.	POS	6.779	349.2249
**Embelin**	7.385_294.	POS	7.385	294.1835
**N6-Acetyl-L-lysine**	0.754_188.	POS	0.754	188.1164
**galactose lactate**	0.766_268.	POS	0.766	268.0807
**2-Hydroxyphenylacetic acid glucuronide**	6.783_322.	NEG	6.783	322.1428
**D-(+)-raffinose**	0.742_526.	POS	0.742	526.1518
**Securinine**	6.075_234.	POS	6.075	234.1368
**Lewis y Tetrasaccharide**	8.016_675.	POS	8.016	675.2618
**Docosapentaenoic acid (22n-6)**	9.962_330.	POS	9.962	330.2558
**Pozanicline**	3.23_192.1	POS	3.23	192.1265
Other				
**Angiotensin III**	6.766_930.	POS	6.766	930.5033
**Englitazone**	2.595_353.	POS	2.595	353.1074
**6-Thioguanosine**	3.203_299.	POS	3.203	299.0688
**Astemizole**	3.337_458.	POS	3.337	458.2492
**(S)-1-Hydroxy-N-methylcanadine;**	5.721_370.	NEG	5.721	370.1661
**PA(22:1(11Z)/17:0)**	8.206_744.	POS	8.206	744.5656
**Pimecrolimus**	7.53_809.4	POS	7.53	809.4467
**epsilon-(gamma-Glutamyl)lysine**	2.622_275.	POS	2.622	275.1479
**2-Amino-1,3,4-octadecanetriol**	7.694_317.	POS	7.694	317.293
Methanobrevibacter_millerae				
**Gracilosulfate D**	8.864_526.	POS	8.864	526.2974

**Talarozole**	3.669_377.	POS	3.669	377.1687
**Shikokianin B**	6.274_508.	POS	6.274	508.2327
**Tubulin Polymerization Inhibitor**	3.83_328.1	POS	3.83	328.1092
**Tigecycline**	3.242_585.	POS	3.242	585.28
**DMS-Cl**	4.158_397.	NEG	4.158	397.0401
**Idarubicinol**	4.654_499.	POS	4.654	499.1822
Other				
**PE(22:6(4Z,7Z,10Z,13Z,16Z,19Z)/18:4(6Z,9Z,12Z,15Z**	10.024_78:	POS	10.024	783.4803
**[3,4,5-trihydroxy-6-[[3,4,5-trihydroxy-6-(hydroxym**	12.921_50!	POS	12.921	509.2454
**5-[4-Phenyl-5-(trifluoromethyl)-2-thienyl]-3-[3-(t**	5.6_440.04	NEG	5.6	440.0414
**Triletide**	4.914_505.	POS	4.914	505.232
**3-hydroxy-2-[[1-[2-[(4-hydroxybenzoyl)amino]-3-**	7.408_507.	POS	7.408	507.1428
**3-{4-[(6,7-Dimethoxyquinolin-4-yl)oxy]-3-fluorop**	3.032_491.	POS	3.032	491.1337
**3-Hydroxynorvaline**	0.714_133.	NEG	0.714	133.074
**3-Sulfobenzoic acid**	2.814_201.	NEG	2.814	201.9936
Herbaspirillum_huttiense				
**1,N6-Ethenoadenosine**	3.817_291.	NEG	3.817	291.0967
**1,6-dihydroxy-8-(hydroxymethyl)-4,12,12,15-tetra**	7.562_516.	POS	7.562	516.2712
**Orotidine 5'-monophosphate**	5.17_368.C	NEG	5.17	368.0204
**NCGC00169827-03!(2E,4E)-12-hydroxy-13-(hydro:**	7.124_306.	POS	7.124	306.1832
**(3R)-4,4-dimethyl-3-[(2S,3R,4S,5S,6R)-3,4,5-trihyd**	4.861_292.	NEG	4.861	292.117
**Robenacoxib**	3.275_327.	POS	3.275	327.0885
**5-Amino-6-(5'-phosphoribosylamino)uracil; 5-Ami**	6.676_354.	NEG	6.676	354.0574
**Dibenzthion; Sulbentine;**	6.941_314.	POS	6.941	314.0902
**2-Propanol, 1-((1-methylethyl)amino)-3-(2,4,6-trip**	9.818_335.	POS	9.818	335.2823
**Tripropyl phosphate**	9.362_224.	POS	9.362	224.118
**(10S)-Juvenile hormone III diol phosphate;**	6.642_364.	POS	6.642	364.1633
**Diethyl 2,4,6-trimethyl-3,4-dihydropyridine-3,5-di**	7.036_267.	POS	7.036	267.147
Pseudomonas_chlororaphis				
**4-(2-Nitroethyl)phenyl 6-O-**	5.166_478.	POS	5.166	478.179
**1-Adamantanecarbonyl-RF-NH2**	6.636_482.	POS	6.636	482.3
**LMFA09000010**	8.209_249.	POS	8.209	249.2456
**Sarcoehrendin B**	9.22_552.2	POS	9.22	552.2933
**Procyanidin B2**	5.343_578.	POS	5.343	578.1428
**SQMG(16:0/0:0)**	7.04_556.2	NEG	7.04	556.2938
**Ezutromid**	0.747_337.	POS	0.747	337.0763
**thielavin B**	5.902_588.	POS	5.902	588.1955
**2,2'-Anhydrocytidine**	0.997_225.	POS	0.997	225.0752
**3-[[3-Methoxy-5-(1H-pyrrol-2-yl)pyrrol-2-ylidene**	3.633_321.	POS	3.633	321.1853
**PS(O-18:0/15:0)**	10.787_73!	POS	10.787	735.5439
**NCGC00386062-01!(3S,6R,12S)-6-benzyl-3-butan-**	7.341_594.	POS	7.341	594.3425
Oribacterium_sinus				
**(S,S)-2-{1-Carboxy-2-[3-(3,5-dichloro-benzyl)-3H**	6.635_427.	POS	6.635	427.1051
**(Z)-7-[(1R,4S,5R,6S)-5-[(E,3R)-3-Hydroxyoct-1-en**	7.335_350.	POS	7.335	350.2456
**PA(19:1(9Z)/0:0)**	9.893_450.	NEG	9.893	450.2747
**Hydromorphone 3-beta-O-glucuronide**	6.497_461.	POS	6.497	461.1689
**(2E,4E)-5-[2-Methyl-2-(1,1,4,4-tetramethyl-1,2,3,4**	7.981_352.	POS	7.981	352.2401
**Tamoxifen**	3.99_371.2	POS	3.99	371.2243
**Isorhamnetin 3-sulfate**	0.673_394.	NEG	0.673	394.0005
**methyl (2R)-2-[(1S,2R,4S,9R,10R,14S,15S,17S)-17-(**	8.306_538.	POS	8.306	538.2472
**3-(5-hydroxy-2,2,7,8-tetramethyl-6-oxo-7,8-dihyc**	5.577_410.	POS	5.577	410.1689
**N-[(2R,3R,4R,5R)-3,5,6-Trihydroxy-1-oxo-4-[(2R,3**	6.28_383.1	NEG	6.28	383.1425
Other				
**PS(O-20:0/14:0)**	10.456_74!	POS	10.456	749.5588
**4-Nitro-3-(trifluoromethyl)aniline**	0.728_206.	NEG	0.728	206.0303
**Isopropyl 2-[[[(1R)-2-(6-aminopurin-9-yl)-1-meth**	3.669_519.	POS	3.669	519.211
Cupriavidus_pauculus				
**Rhetsinine**	7.025_319.	POS	7.025	319.1328

**Carbendazim; Mecarzole;**	6.429_191.POS	6.429	191.0691
**N3'-Acetylneomycin;**	7.944_656.POS	7.944	656.3209
**Paeonoside;**	5.954_328.POS	5.954	328.1174
**6-hydroxy-9,12,13-trimethyl-14-(2-methylpropyl)·**	5.892_439.POS	5.892	439.211
**2,3-Dihydroxy-9,10,11-trimethoxy-5,8,13,13a-tetra**	5.13_357.1POS	5.13	357.1573
**(2R,4'aR,5'S,6'R,6'aS,10'aR,10'bR)-6'-(acetyloxy)-4'**	7.068_516.POS	7.068	516.2114
**N-(5-(5-(2,4-Dioxo-1,3,8-triazaspiro[4.5]decan-8-**	2.849_612.NEG	2.849	612.1865
**Digalacturonate; Digalacturonic acid; alpha-D-Gala**	0.667_370.NEG	0.667	370.0746
**Biorobin**	5.476_594.NEG	5.476	594.1586
**PE(13:0/12:0)**	7.572_593.POS	7.572	593.405
**Hymenoxon**	8.208_282.POS	8.208	282.1467
Aerococcus_viridans			
**4-hydroxy-5-[2-[(E)-3-hydroxyprop-1-enyl]-4-me**	7.611_314.POS	7.611	314.1266
**3-Hydroxy-1-(4-hydroxyphenyl)-1-propanone**	5.345_166.POS	5.345	166.0632
**N,N-Dimethyldodecylamine N-oxide**	7.659_229.POS	7.659	229.2407
Cloacibacterium_rupense			
**5a-Dihydrotestosterone**	8.908_290.POS	8.908	290.2246
**2-Ethyl-2-phenylmalonamide**	5.63_206.1POS	5.63	206.1057
**Macamide**	10.058_37.POS	10.058	371.3188
**Daprodustat**	5.502_393.POS	5.502	393.19
**AM-1220**	9.384_382.POS	9.384	382.203
**2,3-Diphenyl-1-indanone; 2,3-Diphenyl-1H-inden·**	5.26_282.1POS	5.26	282.104
**Metaxalone**	4.485_221.NEG	4.485	221.1052
**Curvulalide**	5.494_214.NEG	5.494	214.0841
**9,10-DHOME**	10.089_31.POS	10.089	314.2456
**N-Desmethyl-o-O-sulfate rosiglitazone**	4.125_439.NEG	4.125	439.0508
**4,6'-Epoxyorotiniflavan-4-ol**	2.326_436.POS	2.326	436.1876
**Benzoylecgonine**	3.493_289.POS	3.493	289.1349
**[(2R,5R)-5-(6-Aminopurin-9-yl)-4-hydroxy-2-(hyc**	2.473_475.POS	2.473	475.1485
**(S)-3-(8-(Dimethylaminomethyl)-6,7,8,9-tetrahydr**	7.619_452.NEG	7.619	452.2232
**6-Thioinosine**	1.1_284.05POS	1.1	284.0581
**5-[(Z)-5-hydroxy-3-methylpent-3-enyl]-1,4a-dim**	7.01_376.1POS	7.01	376.1999
**Cotinine glucuronide**	5.344_352.POS	5.344	352.1269
**Myxalamid A;**	9.049_415.POS	9.049	415.3087
**(4-{4-[2-(gamma-L-Glutamylamino)ethyl]phenoxy**	6.398_375.POS	6.398	375.1789
**8-Hydroxycarteolol**	4.907_308.NEG	4.907	308.1735
**Carmofur**	5.767_257.POS	5.767	257.1164
**Mitragynine**	4.02_398.2POS	4.02	398.2277
**Bis(2-methylpropanoyloxy)-9,10-epoxy-p-mentha**	3.405_358.POS	3.405	358.1165
**Vibriobactin;**	2.799_705.POS	2.799	705.2639
**Metronidazole**	2.832_171.POS	2.832	171.0646
**Flunitrazepam**	3.777_313.POS	3.777	313.0847
**Nicotinamide**	1.041_122.POS	1.041	122.0482
**Juarezic Acid**	2.646_174.POS	2.646	174.0674
**Solasonine**	6.805_883.POS	6.805	883.4924
**1,4-Dioxin**	1.41_84.02POS	1.41	84.02127
**Scopoletin**	4.55_192.CPOS	4.55	192.0401
**Atrazine;**	6.42_215.CPOS	6.42	215.0947
**Grapiprant**	7.698_491.NEG	7.698	491.1973
**11-bromo-undecanoic acid**	0.713_264.POS	0.713	264.0737
**1,2,3,4,7,8,9-Heptachlorodibenzofuran**	0.693_405.NEG	0.693	405.7831
**Fluazifop**	3.164_327.NEG	3.164	327.0734
**Iobenguane**	5.342_274.NEG	5.342	274.9921
**Androsta-4,16-dien-3-one**	7.359_270.POS	7.359	270.1984
**Acevaltrate;**	3.198_480.POS	3.198	480.1974
**Mandipropamid;**	6.696_411.POS	6.696	411.1235
**1-[2-[3-[(2R,3R,4S,5S,6R)-6-[[(2R,3R,4R)-3,4-dihyd**	3.221_512.POS	3.221	512.1875
**Apatinib**	6.852_397.POS	6.852	397.1921

**Thapsigargin**	6.33_650.3	POS	6.33	650.3316
**Sobrepin**	10.188_17(	POS	10.188	170.1309
**4-Fluoro-1-[2-[(4-methyl-1-methylsulfonylpiperid**	1.668_346.	POS	1.668	346.1489
**Fulvinervin B**	5.139_386.	POS	5.139	386.151
**Carvedilol**	3.604_406.	POS	3.604	406.1878
Blautia_obeum				
**10-HDoHE**	8.83_344.2	NEG	8.83	344.2354
**(2Z)-4,6-dihydroxy-2-[(3-hydroxy-4-methoxypher**	8.233_317.	POS	8.233	317.0909
**Iohexol**	2.492_820.	POS	2.492	820.8801
**NA**	5.604_208.	POS	5.604	208.1102
**PE(18:2(9Z,12Z)/16:0)**	11.62_715.	POS	11.62	715.5153
**3-[(Z)-5-[6-hydroxy-5,5,8a-trimethyl-2-methylide**	6.935_570.	POS	6.935	570.3058
**Pegvaliase**	8.705_318.	POS	8.705	318.217
**Wander**	5.828_486.	POS	5.828	486.1573
**curvularin**	3.827_327.	POS	3.827	327.1684
**1,2-Cyclohexanediol**	3.4_116.08	POS	3.4	116.0839
**3'-AZIDO-2',3'-DIDEOXYURIDINE**	1.61_253.0	POS	1.61	253.0812
**Cletoquine**	3.221_307.	POS	3.221	307.1454
**Nirvanol**	4.583_204.	POS	4.583	204.0902
**Androsterone glucuronide**	7.268_466.	NEG	7.268	466.2566
**Gluconapin; 3-Butenylglucosinolate;**	3.215_373.	POS	3.215	373.0513
**methyl orsellinate**	3.202_199.	POS	3.202	199.0848
**[(8R,9S,13S,14S,17S)-17-Hydroxy-13-methyl-6,7,8,**	7.473_396.	POS	7.473	396.2665
**Tobramycin 1-N- or 3-N-carbamoyl**	4.769_510.	POS	4.769	510.2671
**Aflatoxin B1**	5.155_312.	POS	5.155	312.0633
**Isocaviunin 7-O-gentiobioside**	7.035_698.	POS	7.035	698.204
**MM 42842;**	5.215_410.	POS	5.215	410.1092
**2-[(3-Chlorophenyl)diazenyl]acetonitrile**	9.746_179.	POS	9.746	179.0255
**LysoPC(20:3(8Z,11Z,14Z)/0:0)**	10.014_54!	POS	10.014	545.3468
Selenomonas_bovis				
**Methionine sulfoxide**	1.045_165.	POS	1.045	165.0462
**NCGC00347564-02!(3S,6Z)-3-methyl-6-[[2-(2-met**	6.607_668.	POS	6.607	668.3063
**7-[[(2R)-2-Amino-2-carboxyethyl]thio]-2-[[[(1S)-2**	0.633_358.	POS	0.633	358.1573
**Kuwanone H;**	7.488_760.	POS	7.488	760.288
**Muricin-I**	10.75_606.	POS	10.75	606.4866
**(2S)-2-((1S)-1-Hydroxypentyl)-4-methoxy-2,3-dih**	5.906_214.	POS	5.906	214.1208
**dehydrocurvularin**	6.764_290.	POS	6.764	290.1151
**Victorin C;**	5.541_814.	POS	5.541	814.2087
**Glycine glutamate**	0.648_220.	NEG	0.648	220.0694
**Plicatic acid**	6.706_422.	POS	6.706	422.1212
**Peimine**	8.646_431.	POS	8.646	431.3399
**trans-3-Hydroxycotinine glucuronide**	5.169_368.	POS	5.169	368.1234
**L-Tryptophan**	3.016_204.	NEG	3.016	204.0899
**METAMITRON**	3.448_202.	POS	3.448	202.0846
**N-gamma-Glutamylcysteine ethyl ester**	4.81_278.0	POS	4.81	278.0926
**1-Stearoylglycerol**	10.102_35!	POS	10.102	358.3082
**5-hydroxy-7-[2-(2-hydroxy-5-oxo-2H-furan-3-yl)**	5.092_400.	POS	5.092	400.1303
**11-Deoxy prostaglandin F1**	8.468_357.	POS	8.468	357.2941
**16-dimethylarsinoyl-9Z-hexadecenoic acid**	6.552_374.	POS	6.552	374.1816
**N-desmethylimatinib**	5.878_479.	POS	5.878	479.2426
**4'-Chlorodiazepam**	0.789_318.	NEG	0.789	318.0327
**PC(18:2(9Z,12Z)/19:0)**	12.656_79!	POS	12.656	799.6092
**TG(14:1(9Z)/17:2(9Z,12Z)/18:3(6Z,9Z,12Z))[iso6]**	8.659_808.	POS	8.659	808.6547
**2-[(2E)-3,7-dimethylocta-2,6-dienyl]-3-methoxy-5**	5.272_402.	POS	5.272	402.1951
**Deoxycytidine**	3.331_227.	POS	3.331	227.0911
**Cobalt-factor III;**	3.782_933.	NEG	3.782	933.2207
**Bisnafide**	7.965_624.	NEG	7.965	624.1954
**Homosalate**	8.832_262.	POS	8.832	262.157

**Hellicoside;**	3.312_656.	NEG	3.312	656.1948
**5-Hydroxyemedastine**	7.56_318.2	POS	7.56	318.2044
**Cinnamoylglycine**	5.331_205.	POS	5.331	205.0741
**Niclosamide**	0.722_325.	NEG	0.722	325.9851
**L-Glutamic acid 5-benzyl ester**	6.144_237.	POS	6.144	237.1002
**3,4,5-trihydroxy-6-[5-hydroxy-2-(4-hydroxy-3-me**	5.176_506.	NEG	5.176	506.1057
**Moexipril**	4.762_498.	POS	4.762	498.2378
**1-Tert-butyl 4-ethyl 5-oxoazepane-1,4-dicarboxyl**	4.13_285.1	NEG	4.13	285.1575
**Ethyl 3-(4-Fluorophenyl)-3-Oxopropanoate**	3.944_210.	POS	3.944	210.0698
**aschantin**	6.283_417.	POS	6.283	417.1786
Caldicellulosiruptor_acetigenus				
**Progesterone**	7.957_314.	POS	7.957	314.2244
Marinobacterium_litorale				
**SM(d17:1/24:1(15Z))**	10.331_79	POS	10.331	798.6619
**Deoxydihydro-artemisinin**	6.074_268.	POS	6.074	268.1673
**Chenodeoxycholylleucine**	8.913_505.	POS	8.913	505.3774
**methyl (1S,4aS,6R,7S,7aS)-5,6-dihydroxy-7-(hydro**	3.823_422.	NEG	3.823	422.1439
**Tazarotene**	2.337_351.	NEG	2.337	351.1278
**Valdecoxib;**	6.423_314.	POS	6.423	314.0724
**(-)-Methallenestrilphenol; (-)-3-(6-Hydroxy-2-nap**	5.081_272.	POS	5.081	272.141
**Pimonidazole**	0.744_254.	POS	0.744	254.1379
**Lefamulin**	9.724_507.	POS	9.724	507.3024
**Irsogladine**	0.592_255.	POS	0.592	255.008
**Monoanhydroescholtzxanthin**	10.108_54	POS	10.108	548.403
**PI(22:2(13Z,16Z)/0:0)**	5.804_652.	POS	5.804	652.3591
**Misonidazole**	2.667_201.	POS	2.667	201.0752
**Phenolate oxygen**	6.714_202.	POS	6.714	202.0632
Other				
**3,7,21-trihydroxy-17,17-dimethyl-14-(3-methylbu**	2.79_436.1	POS	2.79	436.1516
**Melianthugenin**	5.811_456.	POS	5.811	456.2152
**Vinylcyclohexene**	8.662_108.	POS	8.662	108.0941
**PA(20:5(5Z,8Z,11Z,14Z,17Z)/0:0)**	7.12_456.2	POS	7.12	456.2289
**Carzelesin**	6.104_728.	POS	6.104	728.2547
**Fostriecin**	5.965_430.	POS	5.965	430.1394
**LMPK12010031**	4.655_579.	POS	4.655	579.1527
**Triethylhexanoin**	9.545_470.	POS	9.545	470.361
**Propylparaben sulfate**	5.036_259.	POS	5.036	259.0269
**Deoxysappanone B 7,3'-Dimethyl Ether**	6.302_314.	POS	6.302	314.1156
**3-[5-hydroxy-7-methoxy-2,3-dimethyl-6-(3-meth**	7.231_442.	POS	7.231	442.174
**2-[(Tetrahydrofurfuryl)oxy]ethanol**	3.994_146.	NEG	3.994	146.0944
**1H-Imidazole-1-ethanol, alpha-(1-aziridinylmethyl**	0.718_212.	NEG	0.718	212.0909
**DG(20:1(11Z)/14:0/0:0)**	12.961_59	POS	12.961	594.5236
**7-[3,4-dihydroxy-5-(hydroxymethyl)oxolan-2-yl]o**	6.152_428.	POS	6.152	428.1074
**Ethyl cinnamate; 3-Phenylpropenoic acid ethyl;**	4.125_176.	NEG	4.125	176.0836
**(3E,5S,7R,8R,11E,13S,15R,16R)-3,5,7,11,13,15-hexar**	8.933_515.	POS	8.933	515.3019
**Propranolol glycol**	5.963_218.	POS	5.963	218.0945
**Tenofovir exalidex**	10.324_56	NEG	10.324	569.3699
**Oxidized Latia luciferin;**	6.333_194.	POS	6.333	194.1673
**(1R,3As,4S,6aS)-1,4-di(benzo[d][1,3]dioxol-5-yl)he**	6.714_354.	POS	6.714	354.11
**3-Propoxy-1H-pyrazole-4,5-diol**	0.732_158.	POS	0.732	158.0694
**3',4',5',5,7-Pentamethoxyflavone**	4.415_372.	POS	4.415	372.1215
**Nisoldipine**	7.204_388.	POS	7.204	388.1633
**3beta-Hydroxypregn-5-en-20-one sulfate**	5.292_396.	POS	5.292	396.197
**1-Butylamine**	0.751_73.0	POS	0.751	73.08927
**5-(8,11,14-pentadecatrienyl)resorcinol**	6.835_336.	POS	6.835	336.2048
**Diphenhydramine N-glucuronide**	5.406_432.	POS	5.406	432.2006
Clostridium_nexile				
**Rolapitant**	4.787_500.	NEG	4.787	500.1888

**calycosin-7-O-beta-D-glucoside**	4.944_430.	POS	4.944	430.1252
**6-Formylpterin**	5.923_191.	POS	5.923	191.0441
Other				
**Gluconapoleiferin; 2-Hydroxy-4-pentenyl glucosin**	5.705_403.	POS	5.705	403.0591
**1,4-Bis((p-propylphenoxy)acetyl)piperazine**	6.776_438.	POS	6.776	438.2519
**1,5-Benzothiazepin-4(5H)-one, 2,3-dihydro-3-hyd**	4.662_358.	POS	4.662	358.1356
**(1R,3S,5Z)-5-[(2Z)-2-[(1R,3Ar,7aS)-1-[(2S)-1-[3-(2**	9.773_480.	POS	9.773	480.3246
**Terguride**	7.269_340.	POS	7.269	340.2248
**3-O-(alpha-L-arabinopyranosyl-(1->6)-beta-D-gl**	3.279_454.	NEG	3.279	454.2065
**Pseudoaconitine;**	6.712_689.	POS	6.712	689.3426
Corynebacterium_jeikeium				
Psychrobacter_sanguinis				
Corynebacterium_lipophiloflavum				
Pseudomonas_caeni				
**Hesperetin 3'-O-glucuronide**	7.44_478.1	POS	7.44	478.1118
**Propoxate**	6.111_258.	POS	6.111	258.137
**Terbuthylazine-TP CSCD692760 (LM3)**	1.03_198.C	NEG	1.03	198.0752
**Octadecyl fumarate**	10.101_36	POS	10.101	368.2926
**2-Propylthiazolo[4,5-c]quinolin-4-amine**	0.707_243.	NEG	0.707	243.083
**15-Deoxy-<U+0394>12,14-prostaglandin J2-2-gly**	8.311_408.	POS	8.311	408.2513
**Aragest**	8.314_386.	POS	8.314	386.2454
**Benzo(a)pyrene-3,6-quinone**	3.618_282.	POS	3.618	282.0674
**Methyl red**	4.716_269.	POS	4.716	269.1165
Helicobacter_equorum				
**Alverine**	9.717_281.	POS	9.717	281.2143
**13-OxoODE**	5.486_316.	POS	5.486	316.1999
**picein**	2.524_298.	POS	2.524	298.1049
**Androstane-3,17-diol 17-sulfate**	8.051_372.	NEG	8.051	372.1972
**Palmitoyl Ara-C**	9.905_481.	POS	9.905	481.3173
**Retrorsine**	6.405_351.	POS	6.405	351.1679
**7-Methylguanine**	1.04_165.C	POS	1.04	165.0653
**hellebrigenin-3-(14-hydroxymyristate)**	10.08_642.	POS	10.08	642.4112
**Fenpiclonil; 3-(2,3-Dichlorophenyl)-4-cyanopyrrole**	6.265_235.	NEG	6.265	235.9909
**Zuclopenthixol acetate**	5.983_442.	POS	5.983	442.1478
**Dibenzofuran**	5.978_168.	POS	5.978	168.0577
**4-Hydroxy Triamterene**	2.83_269.1	POS	2.83	269.1013
**Valerophenone**	9.364_162.	POS	9.364	162.1047
**Scilliroside**	6.056_604.	NEG	6.056	604.29
**Deoxycorticosterone acetate**	8.931_372.	POS	8.931	372.2276
**TIC10 isomer**	6.892_386.	POS	6.892	386.2095
**Tetrasul sulfoxide**	0.723_337.	NEG	0.723	337.8901
**Uncaric acid A;**	6.856_354.	POS	6.856	354.158
**2-Amino-4-methylpentanal**	0.732_115.	POS	0.732	115.0998
**NCGC00380679-01![2,3-dihydroxy-1-(7-methoxy-**	3.357_414.	POS	3.357	414.1097
**5-Feruloylquinic acid**	8.273_385.	POS	8.273	385.1372
**TRIPARANOL**	6.959_437.	POS	6.959	437.2132
**Mandelic Acid, Methyl Ester**	4.881_188.	POS	4.881	188.0451
**Mecarphon;**	0.711_271.	NEG	0.711	271.01
**PG(13:0/12:0)**	10.759_62	POS	10.759	624.4003
**Prenyletin**	5.86_263.1	POS	5.86	263.1158
**L-arabitol**	0.698_152.	NEG	0.698	152.0685
Oscillibacter_ruminantium				
**2-Phosphinomethylmalate; 2-Phosphinomethylmal**	0.668_212.	NEG	0.668	212.0087
**3-(5-Acetamido-2-hydroxyphenyl)sulfanyl-2-oxop**	2.723_269.	NEG	2.723	269.0356
**NCGC00179845-03!5-chloro-3-[(E)-3,4-dihydroxy-**	6.898_386.	POS	6.898	386.1478
**Diallyl 2,2'-oxydiethyl dicarbonate**	3.67_274.1	POS	3.67	274.1054
**Biochanin A**	5.354_284.	POS	5.354	284.066
**Isoamylamine**	2.362_87.1	POS	2.362	87.10494

Haemophilus_haemolyticus				
**2,2-Dimethyl-N-(2,4,6-trimethoxyphenyl)dodecan**	8.095_393.	POS	8.095	393.2876
Streptococcus_pneumoniae				
**Cer(d18:1/20:0)**	9.872_593.	POS	9.872	593.5752
**AdipoRon**	6.95_428.2	POS	6.95	428.2101
**2-Mercaptonicotinic acid**	5.142_155.	POS	5.142	155.0042
**N-Acetyl-S-allyl-L-cysteine**	4.406_203.	POS	4.406	203.062
**6-Hydroxycaproic acid**	2.022_132.	NEG	2.022	132.0787
**Myricetin**	2.935_318.	NEG	2.935	318.041
**Astilbin**	4.797_467.	POS	4.797	467.1431
**1-Tetradecanoyl-2-(9Z,12Z-octadecadienoyl)-3-(5**	10.058_85	POS	10.058	850.7023
**(9S,10S)-10-hydroxy-9-(phosphonooxy)octadecan**	9.918_396.	NEG	9.918	396.2277
**Lanceolatin C**	5.624_294.	POS	5.624	294.0903
**trans-Resveratrol 3,4'-disulfate**	3.774_387.	NEG	3.774	387.9925
**(2S,3R)-2-Amino-4-octadecene-3-ol**	10.031_28	POS	10.031	283.2873
**N-(3-oxo-9Z-hexadecenoyl)-homoserine lactone**	7.512_351.	POS	7.512	351.2407
**Chlomethoxyfen;**	0.703_312.	NEG	0.703	312.9915
**2-Oxo-9-methylthiononanoic acid; 9-Methylthio-2**	6.659_218.	POS	6.659	218.0976
**Gabapentin**	4.357_171.	POS	4.357	171.1262
**Karakoline; Aconitane-1,8,14-triol, 20-ethyl-16-me**	7.564_377.	POS	7.564	377.2569
**Longicaudatin**	5.829_446.	POS	5.829	446.1374
**S-Adenosylmethionine**	6.744_399.	POS	6.744	399.1432
**PA(15:1(9Z)/12:0)**	9.531_576.	NEG	9.531	576.3813
**7,10,13,16,19-Docosapentaenoic acid**	10.149_33	NEG	10.149	330.2558
**2,4,12-Octadecatrienoic acid isobutylamide**	9.986_333.	POS	9.986	333.303
**SC-1271; 1-(4'-Chlorophenyl)-1,4-dihydro-4-oxo-**	3.55_358.C	NEG	3.55	358.0723
Ruminococcus_champanellensis				
**Euphornin;**	8.791_584.	NEG	8.791	584.2999
**4-(4'-Chlorobenzyloxy)benzyl nicotinate**	2.827_353.	POS	2.827	353.0816
Campylobacter_hyointestinalis				
**Vinfosiltine**	6.933_945.	POS	6.933	945.5012
**Chembl4238926**	6.089_326.	POS	6.089	326.173
**NCGC00381435-01!13-dodecan-2-yl-6-(1-hydrox**	8.304_541.	POS	8.304	541.3704
**Alpha-hydroxymidazolam**	0.754_341.	NEG	0.754	341.0747
**Kolanone;**	7.982_502.	POS	7.982	502.3061
**bhas#30**	9.18_432.3	POS	9.18	432.3071
**Moricizine**	6.307_427.	POS	6.307	427.1546
**(2R,3R,4S,5S,6R)-2-[2-(4-methoxyphenyl)ethoxy]-**	3.305_446.	POS	3.305	446.18
**4'-ethenyl-2'-hydroxy-1,4',4a-trimethyl-5-oxospir**	7.237_372.	POS	7.237	372.1684
**Retinoate**	8.545_300.	POS	8.545	300.2087
**trans-4-Phenyl-4-piperidinocyclohexanol**	1.553_259.	POS	1.553	259.1896
**2-Iodophenol methyl ether;**	0.594_233.	POS	0.594	233.954
**TRI-P-CRESYL PHOSPHATE**	8.909_368.	POS	8.909	368.1176
**PE(18:3(6Z,9Z,12Z)/0:0)**	9.509_475.	NEG	9.509	475.2696
**3-Hydroxyisovalerylcarnitine**	1.351_261.	POS	1.351	261.1578
**Allyxycarb;**	7.107_274.	POS	7.107	274.1681
**SM(d18:0/18:1(11Z))**	11.968_73	POS	11.968	730.5995
**N-amyl arachidohoyl amine**	10.289_37	POS	10.289	373.3344
**Meranzin hydrate**	2.979_316.	POS	2.979	316.0729
**Hernandezine;**	6.788_652.	NEG	6.788	652.312
**Daidzein**	4.806_254.	NEG	4.806	254.0576
**Tauroursodeoxycholic acid**	7.388_499.	NEG	7.388	499.2963
**5-Hydroxy-2-imino-1-methylimidazolidin-4-one**	0.725_129.	POS	0.725	129.0541
**2-Iodohippuric acid**	0.689_304.	POS	0.689	304.9535
**2-[1-(2-Fluorophenyl)ethylamino]-5-methyl-5-(tri**	0.744_320.	POS	0.744	320.0599
**Temozolomide**	0.952_194.	NEG	0.952	194.0555
**[(2R)-1-[(2S)-6-oxo-2,3-dihydropyran-2-yl]propar**	5.852_337.	POS	5.852	337.1524
**(1R,7S,13S,15S)-2,15-Dihydroxy-7-methyl-6-oxabi**	8.271_280.	POS	8.271	280.1676

**Convicine**	0.634_305.	NEG	0.634	305.0857
**7,8-Didemethyl-8-hydroxy-5-deazariboflavin;**	5.25_363.1	POS	5.25	363.1068
**Imigliptin**	6.27_376.1	POS	6.27	376.1994
**Estriol 3-glucuronide**	4.885_464.	POS	4.885	464.2063
**L-aspartate**	0.65_133.C	NEG	0.65	133.0376
**Procaterol**	8.271_290.	POS	8.271	290.163
**Tauropine; N2-(D-1-Carboxyethyl)taurine;**	1.082_197.	POS	1.082	197.0361
**4-oxo-2E-nonenoic acid**	6.328_170.	POS	6.328	170.0942
**(2R)-8-ethyl-7-hydroxy-5-methoxy-2-methyl-2,3-**	4.451_236.	POS	4.451	236.1049
**3-(2,4-dihydroxyphenyl)-7-hydroxy-6,8-bis(3-met**	6.758_430.	POS	6.758	430.1738
**Glycidol**	0.661_74.C	NEG	0.661	74.03672
**Thymine**	2.543_126.	POS	2.543	126.0431
**(6R)-6-[(2-Chloro-4-fluorophenyl)sulfamoyl]cyclol**	6.793_333.	POS	6.793	333.0224
Reyranella_massiliensis				
Other				
**Carbocyclic thromboxane A2**	9.963_348.	POS	9.963	348.2664
**cis-Vitamin K1**	9.215_450.	POS	9.215	450.3498
**N(6)-Methyllysine**	5.918_160.	POS	5.918	160.1215
**L-Threonic acid**	0.66_136.C	NEG	0.66	136.0373
**DG(20:3(5Z,8Z,11Z)/18:1(11Z)/0:0)**	12.864_64	POS	12.864	644.5383
**Ampicilloyl**	5.166_365.	POS	5.166	365.1408
**Tasquinimod**	4.815_406.	POS	4.815	406.1126
**Tolrestat;**	3.528_357.	NEG	3.528	357.0632
**trans-Cinnamic acid**	2.435_148.	NEG	2.435	148.0526
**Amlexanox**	6.145_298.	POS	6.145	298.0953
**Tyr-Gly-Gly-Phe-Gly-NH2**	6.3_498.22	POS	6.3	498.2202
**Isotabtoxin; delta-Tabtoxin;**	1.312_289.	POS	1.312	289.1274
**(2S,3S)-3-hydroxy-4-methoxy-2-(2-phenylethyl)-2**	6.3_266.11	POS	6.3	266.1154
**PKHdiA-PG**	8.242_638.	POS	8.242	638.3081
**Methylsulfonyl 4-[(2-carbamimidoyl-3,4-dihydro-1**	5.18_487.1	NEG	5.18	487.1912
**N4-Acetylsulfamonomethoxine**	4.045_306.	POS	4.045	306.0794
**xi-2,3-Dihydro-2-oxo-1H-indole-3-acetic acid**	5.609_191.	POS	5.609	191.0585
**Streptolydigin**	5.132_600.	POS	5.132	600.3054
**Rhodoxanthin**	4.517_562.	POS	4.517	562.3827
**(5E)-3,4,9-trihydroxy-2-propyl-2,3,4,7,8,9-hexahyc**	2.887_244.	NEG	2.887	244.1309
**Flumetover; N-Ethyl-N-methyl-4-(trifluoromethyl)**	3.292_367.	POS	3.292	367.138
Acinetobacter_lwoffii				
**KBT 1585 hydrochloride**	4.537_461.	POS	4.537	461.1258
**(E)-N-(3-(3-(4-Fluorophenoxy)phenyl)-1-(R,S)-me**	6.401_316.	POS	6.401	316.1237
**Ambofuracin**	5.414_548.	NEG	5.414	548.1889
**Quizartinib**	7.521_560.	POS	7.521	560.223
**(E)-2-decylpent-2-enedioic acid**	6.101_287.	POS	6.101	287.2095
**Buprenorphine**	9.138_467.	POS	9.138	467.3013
**Cholesterol**	8.487_408.	POS	8.487	408.3353
**Militarinone A;**	6.99_459.2	POS	6.99	459.2626
**Congressane**	9.327_188.	POS	9.327	188.1567
**2-hydroxyquinoline**	5.077_145.	NEG	5.077	145.0529
**Seletalisib**	6.088_482.	NEG	6.088	482.0879
**2'-Carboxymethoxy-4,4'-bis(3-methyl-2-butenylo:**	5.678_450.	POS	5.678	450.2038
**NCGC00381451-01!(1S,4S,7R,10S,13S)-7,10-di(buta**	3.791_529.	POS	3.791	529.2392
**TG(18:3(9Z,12Z,15Z)/18:0/18:3(9Z,12Z,15Z))**	10.654_87	POS	10.654	878.7339
**Erythritol**	0.734_144.	POS	0.734	144.0401
**Monohydroxyperhexiline**	9.627_293.	POS	9.627	293.2719
Other				
**Niceritrol**	5.393_556.	NEG	5.393	556.1621
**1-O-(2R-hydroxy-4Z-eicosenyl)-sn-glycerol**	10.389_38	POS	10.389	386.3397
**N-(Tricosanoyl)-4E,14Z-sphingadienine**	10.251_63	POS	10.251	633.6063
**methyl 2-(2,6-dihydroxy-4-methylbenzoyl)-3,5-di**	6.559_346.	POS	6.559	346.1051

5-Hydroxy-2-(4-methoxyphenyl)-8-(3-methylbut-	7.92_676.2	POS	7.92	676.2376
Triethylene glycol dimethacrylate	5.958_286.	POS	5.958	286.1418
6-Methoxy-2(3H)-benzoxazolone	0.742_165.	NEG	0.742	165.0427
PE(17:0/20:0)	12.82_761.	POS	12.82	761.5931
Albanol A; Mulberrofuran G;	6.708_562.	POS	6.708	562.1606
cis-4-Carboxymethylenebut-2-en-4-olide; 4-Carbo	0.787_140.	POS	0.787	140.0112
LMST01060030	9.484_550.	POS	9.484	550.3881
Fluoxymesterone	10.176_33	POS	10.176	336.2089
9-(2,3-dihydroxypropoxy)-9-oxononanoic acid	5.469_262.	POS	5.469	262.1416
DG(13:0/20:4(5Z,8Z,11Z,14Z)/0:0)[iso2]	10.14_574.	POS	10.14	574.4601
Quetiapine Sulfoxide	9.167_399.	POS	9.167	399.1604
Hesperidin	5.35_610.1	POS	5.35	610.1897
2-Chlorobenzoic acid	5.602_155.	NEG	5.602	155.9979
S-(2-Carboxyethyl)-L-cysteine	0.821_193.	POS	0.821	193.0412
Stearoyl ethanolamide	10.023_32	POS	10.023	327.3136
5-(4-Phenoxybutoxy)psoralen	1.022_350.	POS	1.022	350.1149
3-[(1-Carboxyvinyl)oxy]benzoic acid	4.491_225.	POS	4.491	225.0639
2-Benzyl-2-hydroxybutanamide	5.793_193.	POS	5.793	193.1107
Argininosuccinic acid	3.587_290.	POS	3.587	290.1266
16-carboxy-17,18,19,20-tetranor-leukotriene E3	6.79_415.1	POS	6.79	415.1666
PA(20:0/22:4(7Z,10Z,13Z,16Z))	9.78_780.5	POS	9.78	780.569
Desacetyl-alacepril	5.313_364.	POS	5.313	364.1457
Perfluorooctanoyl chloride	0.707_431.	POS	0.707	431.9402
N-Acetyl-S-(2-carbamoylethyl)-L-cysteine	1.595_234.	POS	1.595	234.0676
methyl 5,6-diacetyloxy-10-hydroxy-2,4b,7,7,10a,12	7.526_576.	POS	7.526	576.2566
PS(20:3(8Z,11Z,14Z)/18:0)	10.244_81	POS	10.244	813.5524
13,14-dihydro-15-keto-tetranor Prostaglandin D2	6.809_280.	POS	6.809	280.1676
N-hydroxyl-tryptamine	3.314_176.	POS	3.314	176.0951
14,15-Leukotriene E4	7.463_439.	NEG	7.463	439.2393
Hydroxychloroquine	8.174_335.	POS	8.174	335.1762
PC(18:0/18:3(9Z,12Z,15Z))	11.634_78	POS	11.634	783.5772
Cefoperazone	5.143_645.	POS	5.143	645.1419
Nifurthiazole; 2-(2-Formylhydrazino)-4-(5-nitro-2-	3.968_254.	POS	3.968	254.0119
Trp-Lys-Tyr-Met-Val-D-Met	7.064_856.	POS	7.064	856.3994
3,4-Bis(carboxymethyl)-3,4-dihydroxyhexanedioic	0.639_294.	NEG	0.639	294.0584
Carboxypeptidase B	6.78_610.2	NEG	6.78	610.2476
3-Oxovalproic acid	4.463_158.	POS	4.463	158.0946
2,3-Dihydrothienamycin;	6.872_274.	POS	6.872	274.0976
MG(0:0/20:5(5Z,8Z,11Z,14Z,17Z)/0:0)	8.292_376.	POS	8.292	376.2612
Glycochenodeoxycholic acid 3-glucuronide	8.851_625.	POS	8.851	625.3463
20,21-Diprenylterpendole I;	9.745_589.	POS	9.745	589.3753
terracinolide C	8.73_760.2	POS	8.73	760.2953
Physcion	5.802_301.	POS	5.802	301.0951
Chloromebuform;	6.873_238.	POS	6.873	238.1226
3-Hydroxymonoethylglycinexylidide	4.765_222.	POS	4.765	222.137
apiosylskimmin	3.222_456.	NEG	3.222	456.1265
(2E)-2-Heptenal	8.908_112.	POS	8.908	112.0889
Tetranorprostanedioic acid	8.293_284.	POS	8.293	284.1988
5-Methyl-4-((1R,6R)-3-methyl-6-(1-methyletheny	10.083_25	POS	10.083	258.162
(3R)-3-Hydroxy-N-[(3S)-2-oxooxolan-3-yl]butana	2.029_187.	POS	2.029	187.0847
LysoPC(22:4(7Z,10Z,13Z,16Z)/0:0)	9.656_571.	POS	9.656	571.3642
urapidil	5.102_387.	POS	5.102	387.2255
7-Methylguanosine	2.515_297.	POS	2.515	297.1073
(5S,8S,10Ar)-N-benzhydryl-5-((S)-2-(methylamino	8.483_561.	NEG	8.483	561.3333
cyclic Melatonin	7.377_230.	POS	7.377	230.1056
2,2-Dimethyl-4-(4-methylsulfonylphenyl)-5-propo	6.322_324.	NEG	6.322	324.1032
Entospletinib	5.768_411.	POS	5.768	411.1796
Auraptene	4.2_320.13	POS	4.2	320.1371

**Cimifoetidanoside G**	8.873_662.	POS	8.873	662.4012
Corynebacterium_urealyticum				
**Kazinol Q**	6.794_462.	POS	6.794	462.2764
**(E)-4-(2-(5,6,7,8-Tetrahydro-8,8-dimethyl-2-naph**	6.867_306.	POS	6.867	306.1969
**[(3aR,4S,6E,10Z,11aR)-10-(hydroxymethyl)-6-meth**	2.585_437.	POS	2.585	437.2052
**PS(20:4(5Z,8Z,11Z,14Z)/18:1(9Z))**	0.714_809.	POS	0.714	809.5242
**3-O-alpha-L-rhamnopyranosyl-3-hydroxy-5Z-tetr**	9.898_558.	POS	9.898	558.3747
**Dichloromethotrexate**	6.589_522.	NEG	6.589	522.0933
**1-(4-Acetyl-2-pyrrolidin-1-ylmethyl-piperazin-1-y**	3.081_397.	POS	3.081	397.1343
Paraeggerthella_hongkongensis				
Thermopolyspora_flexuosa				
**[(2S,3R,4S,5S,6R)-3,4,5-trihydroxy-6-[[(E)-3-pheny**	6.637_462.	POS	6.637	462.1164
**s-Triazol-3-ol, 5-nitro-**	0.592_130.	POS	0.592	130.013
**Bunazosin**	4.461_373.	POS	4.461	373.2099
**(2R)-5,8-dihydroxy-2-(2-hydroxyphenyl)-7-metho**	6.27_302.C	POS	6.27	302.0791
**4-Methoxy-2-naphthylamine**	5.88_173.C	POS	5.88	173.0843
**5-(3',4'-dihydroxyphenyl)-gamma-valerolactone-3**	3.419_398.	NEG	3.419	398.1214
**[6-[3,4-dihydroxy-2,5-bis(hydroxymethyl)oxolan-2**	4.008_535.	POS	4.008	535.1917
**Sethoxydim;**	4.71_327.1	POS	4.71	327.1873
**Lophirone E**	2.619_372.	POS	2.619	372.0992
**Diphenyl phosphate**	9.264_250.	POS	9.264	250.0396
**methyl (2E,8E)-9-[3a-hydroxy-6,7-dimethyl-1-(2-r**	7.547_449.	POS	7.547	449.2777
**Phosphonic acid; Phosphonate; Phosphorous acid; I**	4.458_81.9	NEG	4.458	81.98211
**Indole**	3.291_117.	POS	3.291	117.0579
**Nemiralisib**	9.639_440.	NEG	9.639	440.233
**(E)-5-[(1S,4aR,8aR)-2-formyl-5,5,8a-trimethyl-1,4,**	5.425_376.	POS	5.425	376.2252
**4-[5-[[4-[5-[acetyl(hydroxy)amino]pentylamino]-4**	3.32_460.2	POS	3.32	460.2535
**1-[3,4-Dihydroxy-5-(hydroxymethyl)oxolan-2-yl]-**	3.835_259.	POS	3.835	259.0805
Slackia_isoflavoniconvertens				
**N-Methylethanolamine phosphate;**	0.696_155.	POS	0.696	155.0351
**N-(2,6-Difluorophenyl)-2-oxoglycyl-N-((1S)-1-(ca**	4.006_549.	NEG	4.006	549.0968
**trans-3-Hydroxycotinine**	3.967_192.	POS	3.967	192.0902
**PC(14:0/20:3(5Z,8Z,11Z))**	11.108_75	POS	11.108	755.5455
**Palmitoleamide**	9.405_253.	POS	9.405	253.2407
**Sorbitan palmitate**	10.293_40	NEG	10.293	402.2982
**Dihydrokainic acid**	2.681_215.	NEG	2.681	215.1158
**syringaresinol**	6.985_435.	POS	6.985	435.1894
**(2E)-2-(methoxycarbonylmethyl)but-2-enedioic ac**	0.67_188.C	NEG	0.67	188.0321
**Magnesium salicylate**	3.734_298.	NEG	3.734	298.0316
**Tesofensine**	5.909_327.	NEG	5.909	327.1141
**SAPERCONAZOLE**	6.474_672.	POS	6.474	672.3007
**Osajin**	4.777_404.	POS	4.777	404.1585
**3-methyl-3,4-dihydro-1H-1,4-benzodiazepine-2,5**	5.886_190.	POS	5.886	190.0744
**Sumarotene**	6.101_382.	POS	6.101	382.1968
**2'''-N-Acetyl-6'''-deamino-6'''-hydroxyparomomy**	4.787_658.	POS	4.787	658.2938
**2,4,6-Tris(2-pyridyl)-s-triazine**	6.567_312.	POS	6.567	312.1109
**2-Imidazolidone**	0.77_86.04	POS	0.77	86.04818
**Heliotron**	4.647_313.	POS	4.647	313.1885
**Thioperamide**	9.753_292.	POS	9.753	292.1715
**Bicuculline (+)**	4.714_389.	POS	4.714	389.0869
Pseudomonas_xanthomarina				
**Bufuralol**	5.009_261.	POS	5.009	261.1728
**Tiglylcarnitine**	5.616_243.	POS	5.616	243.147
**(R)-Equol**	5.493_242.	POS	5.493	242.0944
**N-Ethyl-N-(2-hydroxy-3-sulfopropyl)-3-toluidine**	4.11_273.1	POS	4.11	273.1035
**2-(6-hydroxyhexyl)-3-methylidenebutanedioic aci**	3.35_230.1	NEG	3.35	230.1153
**ritanserin**	0.652_477.	NEG	0.652	477.1699
**13-Deoxytedanolide;**	10.031_59	POS	10.031	594.3423

**Butyryl trihexyl citrate**	8.272_514.	POS	8.272	514.3525
Other				
**L-Tyrosine**	1.191_181.	POS	1.191	181.0741
**OA-6129 E;**	5.704_489.	POS	5.704	489.2148
**Butylparaben**	6.778_194.	NEG	6.778	194.0977
**(4-(2-((2-(3-Chlorophenyl)-2-hydroxyethyl)amino)**	6.356_363.	POS	6.356	363.1253
**Melithiazol A;**	4.266_422.	NEG	4.266	422.1325
**Diphenyl(2,4,6-trimethylbenzoyl)phosphine oxide**	0.652_348.	NEG	0.652	348.1279
**Valine**	0.988_117.	POS	0.988	117.0791
**Sulfolane**	2.174_120.	POS	2.174	120.0247
**N,N-dimethyl-Safingol**	8.553_329.	POS	8.553	329.3293
**4,5-Dinitro-7,8-dithiabicyclo[4.2.0]octa-1(6),2,4-tri**	0.635_273.	POS	0.635	273.9346
**Hesperetin**	6.376_302.	POS	6.376	302.079
**RESAZURIN**	0.761_229.	POS	0.761	229.0387
**Kaempferol 7-glucoside**	4.574_448.	NEG	4.574	448.1007
**Dihydrothymine**	0.824_128.	POS	0.824	128.0588
**Ezetimibe**	3.768_409.	POS	3.768	409.1488
**Ascorbic acid**	5.034_176.	NEG	5.034	176.0322
**Benzoic acid, 4-(4-((2R,5R)-5-((bis(phenylmethyl)a**	7.544_600.	POS	7.544	600.3007
**Clusianose;**	6.131_344.	POS	6.131	344.1336
**Neomycin**	8.912_614.	NEG	8.912	614.3123
**ZATOSETRON**	6.553_348.	POS	6.553	348.1602
**E-3174**	4.5_436.14	NEG	4.5	436.1417
Cetobacterium_somerae				
**PA(22:2(13Z,16Z)/0:0)**	7.63_490.3	POS	7.63	490.3081
**Cocculine**	4.768_271.	POS	4.768	271.1575
**1,3-Bis(tetrahydro-2-furanyl)-5-fluoro-2,4-pyrimic**	6.871_270.	POS	6.871	270.1027
**25-Azacholesterol**	10.392_38	POS	10.392	387.3502
**SNAG-delta5-CT**	6.826_780.	POS	6.826	780.3212
**1H-Imidazole, 2-(2-benzofuranyl)-4,5-dihydro-**	7.365_186.	POS	7.365	186.0796
**3-hydroxy-2-octylpentanedioic acid**	2.797_277.	POS	2.797	277.1888
**Etiocholanolone**	7.926_272.	POS	7.926	272.2141
**8-benzoyl-1,5,5-trimethyl-6,15-dioxatetracyclo[9.3**	5.49_364.1	NEG	5.49	364.167
**4-Hydroxyphenytoin glucuronide**	3.357_444.	POS	3.357	444.1164
**(Z)-2-methyl-4-[(2R,3R,4S,5S,6R)-3,4,5-trihydroxy-**	5.411_259.	POS	5.411	259.1055
**Leucadenone D**	4.955_540.	NEG	4.955	540.2165
**Nootkatone**	6.93_218.1	POS	6.93	218.1671
**Bms-707035**	5.332_410.	POS	5.332	410.1072
Clostridium_glycyrrhizinilyticum				
**Oxazepam**	3.603_286.	NEG	3.603	286.0508
**Abemaciclib**	5.895_506.	POS	5.895	506.2705
**PI(19:0/0:0)**	9.419_614.	POS	9.419	614.343
**Isorhamnetin-3-O-neohesperidoside**	6.951_624.	POS	6.951	624.1686
Campylobacter_rectus				
Acinetobacter_beijerinckii				
**Glucose lactate ketone**	4.361_384.	POS	4.361	384.0887
**NCGC00381146-01!2-[5-[2-[2-[5-(2-oxopropyl)ox**	5.963_415.	POS	5.963	415.257
**C20822; Carbapenem biosynthesis intermediate 3;**	1.531_242.	POS	1.531	242.0725
**Nitropravastatin**	5.032_469.	POS	5.032	469.2331
Enterococcus_sulfureus				
**Cer(t18:1(6OH)/15:0)**	9.724_539.	POS	9.724	539.4922
**Nicotine glucuronide**	2.847_338.	POS	2.847	338.1481
**NA**	3.716_432.	NEG	3.716	432.0838
**Fenoxycarb**	6.959_301.	POS	6.959	301.1313
**Asperlicin;**	7.058_535.	POS	7.058	535.2237
**Hydroxytyrosol 3'-glucuronide**	5.974_330.	POS	5.974	330.0961
**Propanidid**	5.414_337.	NEG	5.414	337.1889
**3-Pyridinemethanamine, 5-(3-(4,6-difluoro-1H-be**	2.659_418.	POS	2.659	418.1699

**Phosphohistidine**	2.383_235.	POS	2.383	235.0358
**N-Acetylcadaverine**	0.745_127.	POS	0.745	127.1
**ANTIMYCIN A (A1 shown)**	12.781_55(	POS	12.781	556.2373
**Prostaglandin A2**	5.773_356.	POS	5.773	356.1986
**Clethodim**	0.652_359.	NEG	0.652	359.1326
Hydrogenoanaerobacterium_saccharovorans				
**2-Pyridinecarboximidamide, N,N''-(2,5-furandiylbi:**	5.371_574.	POS	5.371	574.2668
**3,5-Diisopropylsalicylic acid**	8.241_222.	POS	8.241	222.1258
**alpha-Naphthoflavone**	4.746_272.	POS	4.746	272.0833
**Pikromycin**	8.43_525.3	NEG	8.43	525.3294
**Riddelliine N-oxide**	6.652_365.	POS	6.652	365.1473
**Clindamycin**	4.986_424.	POS	4.986	424.1783
**Chloramitriptyline**	0.994_311.	NEG	0.994	311.1456
**2,2'-Azobis(4-methoxy-2,4-dimethylvaleronitrile)**	9.75_308.2	POS	9.75	308.2202
**Motolimod**	8.652_458.	NEG	8.652	458.2704
**LMPK12120312**	0.642_392.	NEG	0.642	392.026
**(S)-N1-((1H-Benzo[d]imidazol-2-yl)methyl)-N1-(5**	8.787_349.	POS	8.787	349.2254
**Procainamide 4-hydroxylamine**	5.772_251.	POS	5.772	251.1634
**(2S)-2-[[(2S)-2-Aminopropanoyl]-naphthalen-2-yl**	5.697_344.	POS	5.697	344.1369
**Delafloxacin**	3.634_440.	POS	3.634	440.0495
**THIARABINE**	3.236_259.	POS	3.236	259.0627
**Nitromifene**	3.621_444.	POS	3.621	444.2043
**methyl (1R,18R)-17-ethyl-3,13-diazapentacyclo[13**	7.007_358.	POS	7.007	358.1639
**Fluorescein**	3.347_332.	POS	3.347	332.0681
**8-hydroxy-2,7,7,11,15-pentamethyl-5,12,16-trioxa**	8.179_414.	POS	8.179	414.1429
**L-Tyrosine methyl ester 4-sulfate;**	2.982_275.	NEG	2.982	275.0461
**1-Methylene-4-(1-methylvinyl)cyclohexane**	10.135_13(	POS	10.135	136.1254
**1alpha,25-dihydroxy-25,25-diphenyl-26,27-dinorv**	9.812_540.	POS	9.812	540.3606
**(12R)-12-Hydroxyoctadec-9-enoic acid**	9.064_298.	NEG	9.064	298.2507
**Thioctic acid**	0.682_206.	POS	0.682	206.0438
**Tridecylic acid**	8.906_214.	NEG	8.906	214.1933
**Tebupirimfos**	0.651_318.	NEG	0.651	318.1174
**6-Amino-1-naphthalenesulfonamide**	3.782_222.	POS	3.782	222.0465
**1,1-Dichloro-2-(dihydroxy-4'-chlorophenyl)-2-(4'-**	0.729_347.	POS	0.729	347.9261
Rarobacter_incanus				
**1,1,1-Trifluoroheptadecan-2-one**	9.868_308.	POS	9.868	308.2326
**Codeine**	6.897_299.	POS	6.897	299.1521
**Cassaidine;**	8.29_407.3	POS	8.29	407.3039
**Chymostatin**	7.14_607.3	POS	7.14	607.3099
**Iriomoteolide 1a; Iriomoteolide-1a;**	9.711_506.	POS	9.711	506.3266
**5-(3',5'-Dihydroxyphenyl)-gamma-valerolactone 3**	4.607_384.	NEG	4.607	384.1058
**Acetamide, N-(4-(((2,6-dimethoxy-4-pyrimidinyl)a**	1.083_352.	POS	1.083	352.0824
**PG(16:0/0:0)**	9.69_484.2	NEG	9.69	484.2802
**swertiamarin**	3.029_374.	NEG	3.029	374.1214
**Urea,3,4-tri-O-acetyl-D-ribopyranosyl)-**	4.657_409.	POS	4.657	409.0888
**Berberastine;**	7.102_352.	POS	7.102	352.1171
**N(beta)-Epoxysuccinamoyl-DAP-Val; N(beta)-Epox**	1.204_316.	POS	1.204	316.1383
**Cefpodoxime**	3.849_427.	NEG	3.849	427.062
**catechin 3-tetradecanoate**	4.786_500.	POS	4.786	500.2784
**L-Chlorozotocin**	4.646_313.	POS	4.646	313.0674
**TETRAHYDROPAPAVERINE**	6.262_343.	POS	6.262	343.1782
**(E)-2-Methylgeranyl diphosphate;**	3.241_328.	POS	3.241	328.084
**DL-Arginine**	2.441_174.	NEG	2.441	174.1092
**(+)-Syringaresinol O-beta-D-glucoside; Acanthosi(**	3.17_580.2	NEG	3.17	580.2162
**PG(18:3(9Z,12Z,15Z)/22:5(4Z,7Z,10Z,13Z,16Z))**	10.081_81	POS	10.081	818.5101
**Hippuric acid**	3.902_179.	POS	3.902	179.0584
**1,2-Dihexanoyl-sn-glycero-3-phosphoethanolami**	4.917_411.	POS	4.917	411.2023
**4-hydroxy-5-[(2S,3R,4S,5S,6R)-3,4,5-trihydroxy-6-**	4.758_378.	POS	4.758	378.0952

Vanillactic acid	4.885_212.	POS	4.885	212.0686
resmethrin	5.724_338.	POS	5.724	338.1867
3-Icosa-5,8,11,14-tetraen-2-yloxypropane-1,2-dio	10.523_36	POS	10.523	364.2977
Isobutyrylglycine	0.723_145.	NEG	0.723	145.074
Indanthrene	4.898_442.	NEG	4.898	442.0934
PHOHA-PS	5.983_637.	POS	5.983	637.3196
Succinic acid	1.276_118.	POS	1.276	118.0267
1H-Indole-3-carboxylic acid, 1-methyl-, (1-(2-((me	3.291_393.	POS	3.291	393.1723
4,5-Dihydro-4-((1-piperazinyl)acetyl)-7-(3-(trifluo	6.691_416.	POS	6.691	416.1581
N-[(2R)-4-(Hydroxymethyl)-3-oxo-1-sulfanylhex-	3.647_217.	POS	3.647	217.0775
(8S,9R,10R,13S,14S)-10,13-Dimethyl-4,5,6,7,8,9,11,	10.139_25	POS	10.139	258.2348
Nitisinone	3.221_329.	POS	3.221	329.0502
Ceanothine B;	7.416_504.	NEG	7.416	504.275
Antramycin	3.708_315.	NEG	3.708	315.1219
ROSIN	3.671_296.	POS	3.671	296.1262
3-[[(2S)-3-(4-Phenylphenyl)-2-(phosphonomethyl	5.773_406.	POS	5.773	406.131
N-palmitoyl valine	9.523_355.	POS	9.523	355.3085
Histidyltyrosine	5.313_318.	POS	5.313	318.1322
Diamfenetide	8.175_372.	NEG	8.175	372.1686
26,26,26,27,27,27-hexafluoro-1alpha,25-dihydroxy	6.683_520.	POS	6.683	520.2427
2-[2,6-bis(3-methylbut-2-enyl)-4-[(2S,3R,4S,5S,6R	2.458_466.	POS	2.458	466.2181
N-Desmethylatomoxetine	4.682_241.	POS	4.682	241.1467
N-Benzyl-9-(tetrahydro-2H-pyran-2-YL)adenine	5.093_309.	NEG	5.093	309.1576
glycolithocholic acid	8.846_433.	NEG	8.846	433.3192
[3H]Pemetrexed	6.606_427.	POS	6.606	427.1492
S-Phenyl-L-cysteine	5.622_197.	POS	5.622	197.0513
2-[(8S,9R)-9-acetyloxy-2-oxo-8,9-dihydrofuro[2,3	6.792_405.	POS	6.792	405.1784
Triflusal	0.702_248.	POS	0.702	248.03
Ancistrobrevine A;	5.283_435.	POS	5.283	435.2392
Mitomycin	3.238_334.	NEG	3.238	334.1265
4-(2-((7-Amino-2-(furan-2-yl)-[1,2,4]triazolo[1,5-	3.551_337.	POS	3.551	337.1272
L-Prolinamide,D-tyrosyl-L-arginylglycyl-4-nitro-L-	6.286_682.	POS	6.286	682.3215
Monoacetoxyscirpenol	5.413_324.	POS	5.413	324.1572
(E)-4-Chloro-N-(3-(3-(4-hydroxy-3-methoxyphen	2.438_407.	POS	2.438	407.0937
Napelline;	9.364_359.	POS	9.364	359.2461
glas#1	4.649_438.	POS	4.649	438.2113
2-Ethylhexyl diphenyl phosphate	9.263_362.	POS	9.263	362.1647
Perphenazine 4-aminobutyrate	5.827_488.	POS	5.827	488.2021
RESORCINOL MONOACETATE	5.307_152.	POS	5.307	152.0475
5-Amino-5-(4-hydroxybenzyl)-6-(D-ribitylimino)-	3.219_382.	NEG	3.219	382.1491
Leucine-beta-naphthylamide	5.483_256.	NEG	5.483	256.1574
Oxidized Renilla luciferin; Coelenteramide h; N-[3-I	5.377_395.	POS	5.377	395.1615
1-[(5-Methoxy-3-indolylidene)methylamino]-2-pe	4.117_301.	POS	4.117	301.1889
5'-Hydroxylornoxicam	6.955_386.	POS	6.955	386.975
Hexanesulfonic acid	0.72_166.0	POS	0.72	166.0666
Trichilin A;	7.823_674.	POS	7.823	674.2944
Chromopyrrolate; Chromopyrrolic acid;	1.279_385.	POS	1.279	385.1056
Skepinone-L	2.283_425.	POS	2.283	425.1439
Doxazosin	3.829_451.	POS	3.829	451.1878
Cobalt-precorrin 6; Cobalt-precorrin 6A;	5.936_952.	POS	5.936	952.2834
Estrone	5.907_270.	POS	5.907	270.1579
5-Nitroxystearic acid	7.04_345.2	POS	7.04	345.2512
Metominostrobin;	6.954_284.	POS	6.954	284.1161
7-Benzylidenenaltrexone	5.552_429.	NEG	5.552	429.1934
Sapanisertib	3.214_309.	POS	3.214	309.1324
N-[2-[5-[[3-(2-Aminoethyl)-1H-indol-5-yl]-metho	7.598_462.	NEG	7.598	462.298
UDP-L-Ara4O; UDP-4''-ketopentose; Uridine 5'-di	6.606_534.	POS	6.606	534.0285
PKOOA-PI	3.22_724.3	NEG	3.22	724.3409

**Quinocarmycin analog**	7.406_357.	POS	7.406	357.1688
**3-Indole carboxylic acid glucuronide**	3.179_337.	NEG	3.179	337.0799
Brachybacterium_muris				
**Diethyl 6-methoxy-5,7-dihydroindolo[2,3-b]carbaz**	6.27_430.1	POS	6.27	430.1544
Variovorax_paradoxus				
Syntrophococcus_sucromutans				
**Imidocarb**	6.445_348.	POS	6.445	348.1682
**gamma-Aminobutyric acid**	1.531_85.C	POS	1.531	85.05288
**trihydroxy-4-pregnen-3-one**	8.265_348.	POS	8.265	348.23
**taurohyocholic acid**	9.39_497.2	POS	9.39	497.2823
**4-Amino-4-deoxyprephenate;**	2.885_225.	POS	2.885	225.0639
**(5-D-ribonyl)hopane**	10.119_54	POS	10.119	542.4347
**Pentamethylchromanol**	7.281_220.	POS	7.281	220.1465
**(3a,5b,7a)-23-Carboxy-7-hydroxy-24-norcholan-3**	8.765_568.	POS	8.765	568.3253
Streptomyces_lavendulae				
**PS(O-18:0/20:3(8Z,11Z,14Z))**	10.381_79	POS	10.381	799.5738
**Chamuvaritin**	6.263_452.	POS	6.263	452.1634
**Chloropyramine**	6.047_289.	POS	6.047	289.1347
**Cer(t18:1(6OH)/21:0(2OH))**	12.091_63	NEG	12.091	639.582
**PG(P-20:0/17:0)**	8.209_776.	POS	8.209	776.5912
**Adarotene**	6.876_374.	POS	6.876	374.1871
**1-(3,4-Dichlorophenyl)-3-azabicyclo[3.1.0]hexane**	2.609_227.	POS	2.609	227.0258
**Coproporphyrin III**	8.328_654.	POS	8.328	654.2695
**Glisentide**	6.275_445.	POS	6.275	445.1653
**Miriquidic acid**	3.597_480.	POS	3.597	480.1749
**Lappaconitine;**	5.098_584.	POS	5.098	584.3096
**6-Dehydrotestosterone glucuronide**	7.191_462.	POS	7.191	462.226
**[5-acetyloxy-3-(hydroxymethyl)-2-oxo-6-propan-**	6.691_378.	POS	6.691	378.1426
**Etintidine**	2.775_276.	POS	2.775	276.1145
**Daltroban**	5.912_353.	POS	5.912	353.0489
**[(3R,4S)-1,1-Difluoro-3-(hexadecanoylamino)-4-hy**	7.722_519.	NEG	7.722	519.2931
**Tert-BUTYL ISOCYANIDE**	1.045_83.C	POS	1.045	83.07366
**Apixaban**	8.935_459.	POS	8.935	459.1904
**Cladinose**	5.721_176.	POS	5.721	176.1051
Other				
**(1R,3E,5R,7S,11R,12R,13S,14S)-1,13-bis(acetyloxy)-**	5.879_477.	POS	5.879	477.2731
**(**	4.26_264.1	NEG	4.26	264.1358
**Commendamide**	8.406_329.	POS	8.406	329.2566
**L-Oleandrosyl-oleandolide**	7.89_530.3	NEG	7.89	530.3101
**Propane-1,2-diol 1-phosphate;**	4.345_156.	POS	4.345	156.0189
**Verlukast**	5.24_514.1	NEG	5.24	514.1143
**Metronidazole-OH**	2.264_187.	POS	2.264	187.0595
**13-(acetyloxy)-1-hydroxy-8-(hydroxymethyl)-4,12**	9.451_472.	POS	9.451	472.2442
**NCGC00168829-02!7-[(2E)-5-hydroxy-3,7-dimethy**	5.43_336.1	POS	5.43	336.1321
**Cer(d18:1/19:0)**	12.615_57	POS	12.615	579.5596
**bhas#38**	8.81_488.3	POS	8.81	488.3731
**Sodium oleate;**	2.465_304.	POS	2.465	304.4439
**Fluorescein diacetate**	6.154_416.	POS	6.154	416.0897
**Integerrenine;**	7.869_534.	NEG	7.869	534.3231
**Eravacycline**	7.982_558.	POS	7.982	558.2112
**2-Hydroxyphenylalanine**	0.713_181.	POS	0.713	181.0718
**Descladinose roxithromycin**	6.347_678.	POS	6.347	678.4295
**2,4-bis(3-methylbut-2-enyl)-5-pentylbenzene-1,3**	7.181_316.	POS	7.181	316.2402
**BENZOYLARGININE NITROANILIDE**	5.245_398.	POS	5.245	398.1685
**Phenthoate**	4.015_320.	POS	4.015	320.0305
**Cucurbitacin F**	8.431_518.	POS	8.431	518.3236
**4-(3,4-dihydroxyphenyl)-7-hydroxy-5-[(2S,3R,4S,5**	5.53_448.1	POS	5.53	448.1007
**N6-Carboxymethyllysine**	1.219_204.	POS	1.219	204.1113

**Satigrel**	4.93_337.1	POS	4.93	337.1313
**Makisterone B;**	7.969_494.	POS	7.969	494.325
**Piperidione; 3,3-Diethyl-2,4-dioxopiperidine;**	4.752_169.	POS	4.752	169.1105
**3-Methoxy-4-Hydroxyphenylglycol sulfate**	0.726_264.	NEG	0.726	264.0301
**2H-Pyran-2-one**	0.659_96.C	NEG	0.659	96.02112
**1-(2-Hydroxyethyl)-5-nitropyrrole-2-carboxamide**	2.454_199.	POS	2.454	199.0596
**Benzamide**	4.819_121.	POS	4.819	121.0529
**(2-Carbamoyl-7-oxo-1,6-diazabicyclo[3.2.1]octan-**	3.281_265.	NEG	3.281	265.0366
**Vesatolimod**	9.953_410.	POS	9.953	410.2433
**4-(5-Fluoro-1,3-benzothiazol-2-yl)-2-methylanilir**	4.836_258.	POS	4.836	258.0617
**Guvacoline**	4.712_141.	POS	4.712	141.0792
**O-Acetylcypholophine;**	6.66_360.2	POS	6.66	360.2047
**1-Methyluric acid**	2.167_182.	POS	2.167	182.0443
**2',2'-Difluorodeoxyuridine**	5.886_264.	NEG	5.886	264.0566
**Bacteriochlorophyllide g;**	8.778_614.	POS	8.778	614.2375
**Benzenebutanoic acid, alpha-(acetylamino)-2-amir**	3.875_250.	POS	3.875	250.0954
**Etridiazole**	6.696_245.	POS	6.696	245.9177
**Naphth(1,2-b)oxirene**	5.973_142.	POS	5.973	142.042
**ARTHONIOIC ACID**	8.828_528.	POS	8.828	528.2382
**4-({4-[(2,4,6-Trimethylphenyl)amino]-1,3,5-triazin-**	6.264_330.	NEG	6.264	330.158
**Anadur**	9.764_506.	POS	9.764	506.3377
Bosea_vestrisii				
**Cholylglycyltyrosine**	8.688_628.	POS	8.688	628.3719
**Noa-Asn-Apns-Dmt-NH-tBu**	6.464_691.	NEG	6.464	691.3073
**Prolylphenylalanine**	4.23_262.1	POS	4.23	262.1317
**3-(1-Propyl-3-piperidinyl)phenol**	5.127_219.	POS	5.127	219.1623
**Torasemide**	8.596_348.	POS	8.596	348.125
**N-Acetyltryptophan**	6.943_246.	POS	6.943	246.1005
**Furafylline**	5.043_260.	POS	5.043	260.0911
**Nonanoylcarnitine**	6.369_301.	POS	6.369	301.2251
**PLATYPHYLLENONE**	5.41_318.1	POS	5.41	318.1215
Other				
**3-Indoleethanol**	5.042_193.	POS	5.042	193.1106
**Eucommin A; Medioresinol 4'-O-beta-D-glucopyra**	6.981_550.	POS	6.981	550.2034
**8-Hydroxyquinoline**	3.292_145.	POS	3.292	145.0529
Other				
**5-[(4-Ethylphenyl)methylene]-2-thioxo-4-thiazolic**	0.718_249.	NEG	0.718	249.0281
**Cer(d18:1/28:0(28OH))**	0.706_721.	POS	0.706	721.697
**(E)-3-(4-methoxyphenyl)-1-[2,4,6-trimethoxy-3-(3**	7.098_418.	POS	7.098	418.174
**PE(18:2(9Z,12Z)/0:0)**	9.387_477.	NEG	9.387	477.2852
**Tridecanamide**	9.141_213.	POS	9.141	213.2095
**Glomellic acid**	6.668_489.	POS	6.668	489.2003
**3-methoxybenzene-1,2-diol**	0.658_140.	NEG	0.658	140.0474
**3-Hydroxy-3',4'-Dimethoxyflavone**	5.985_298.	POS	5.985	298.0845
**Apricoxib**	7.146_356.	POS	7.146	356.1195
**Griseofulvic Acid**	5.34_376.C	POS	5.34	376.0134
**Parishin C**	4.861_728.	NEG	4.861	728.2165
**Gemfibrozil 1-O-beta-Glucuronide**	3.317_426.	POS	3.317	426.1904
**Penitrem B**	9.805_583.	POS	9.805	583.3297
**Lucitanib**	6.307_443.	POS	6.307	443.186
**Carbenicillin indanyl**	5.249_494.	NEG	5.249	494.1536
**PKOOA-PE**	6.244_605.	POS	6.244	605.3304
**Braxoron; 9alpha-Bromo-11-oxoprogesterone; 9al**	4.459_406.	NEG	4.459	406.1161
**4'-Hydroxy-R-phenprocoumon**	6.809_296.	POS	6.809	296.1046
**3-Methylcrotonylglycine**	3.183_157.	NEG	3.183	157.074
**PS(20:4(5Z,8Z,11Z,14Z)/18:4(6Z,9Z,12Z,15Z))**	6.693_803.	POS	6.693	803.4758
**Amdinocillin**	3.343_325.	POS	3.343	325.1462
**Ethylenediamine-N,N'-diacetic acid**	0.669_176.	NEG	0.669	176.0797

**8,8-dimethyl-2-phenylpyrano[2,3-f]chromen-4-on**	12.305_32(	POS	12.305	326.093
**Z-Ala-arg-arg-afc**	9.263_746.	POS	9.263	746.3119
**Asparagine glutamate**	0.647_277.	NEG	0.647	277.0907
**N-Glucosylnicotinate;**	4.039_286.	POS	4.039	286.0928
**Cholylmethionine**	8.362_539.	POS	8.362	539.3288
**(8R,9S,10R,13S,14S,16R,17S)-16-Ethyl-17-(2-hydro**	9.7_344.23	POS	9.7	344.2353
**Brisoral**	6.286_389.	POS	6.286	389.1046
**(+/-)11(12)-DiHET**	8.272_360.	POS	8.272	360.23
**Tesevatinib**	6.249_490.	POS	6.249	490.1347
Shewanella_baltica				
**6-Bromo-5E,9Z,13Z,24Z-heptacosatetraenoic acid**	4.74_466.2	POS	4.74	466.2431
**Revexepride**	7.047_425.	POS	7.047	425.2063
**1alpha,25-dihydroxy-2beta-(6-hydroxyhexyl)vitam**	10.014_51(	POS	10.014	516.4185
**Amibegron**	5.787_403.	POS	5.787	403.1532
**Lersivirine**	5.038_310.	POS	5.038	310.1419
**5-Methoxy-N,N-diisopropyltryptamine**	4.918_274.	POS	4.918	274.2042
**Suvorexant**	4.921_450.	POS	4.921	450.156
**Solutol HS 15**	9.956_344.	POS	9.956	344.2926
**3-((4-(2-Methoxyphenyl)piperazin-1-yl)methyl)-2,**	6.526_391.	POS	6.526	391.2028
**[3H]Rosiglitazone**	5.366_357.	POS	5.366	357.1146
**Tamoxifen-N-glucuronide**	6.845_548.	NEG	6.845	548.2623
**(L-Prolyl)adenylate;**	4.02_444.1	POS	4.02	444.1145
**(24R)-1alpha,24,25-trihydroxy-22-oxavitamin D3 /**	8.343_434.	POS	8.343	434.3036
**Gardiquimod**	4.783_313.	POS	4.783	313.1891
**Proparacaine**	6.793_294.	POS	6.793	294.1938
**[6-acetyloxy-7-(chloromethyl)-7-hydroxy-1-(3-me**	5.754_482.	POS	5.754	482.1674
**N-docosahexaenoyl histidine**	7.692_465.	NEG	7.692	465.2996
**cis-(Homo)2-aconitate;**	0.651_202.	NEG	0.651	202.0476
**Glutamine-glutamate**	0.654_291.	NEG	0.654	291.1064
**LMPK12120030**	5.517_486.	POS	5.517	486.1877
**TUCARESOL**	8.102_272.	NEG	8.102	272.0683
**4,21-Dehydrogeissoschizine;**	6.263_351.	POS	6.263	351.1713
**Spongipregnoloside A**	8.275_624.	POS	8.275	624.3484
**4-Hydroxycoumarin**	4.659_162.	NEG	4.659	162.0318
**Sinomenine**	2.932_346.	POS	2.932	346.1853
**3,5-Dihydroxyphenylvaleric acid sulfate**	3.36_290.0	POS	3.36	290.0449
**(1S,4S,9R,10S,13R,14R)-5,5,9,14-Tetramethyltetracy**	10.42_272.	POS	10.42	272.2504
**8-Hydroxy-7-methylguanine**	1.916_181.	POS	1.916	181.0603
**Acacetin 7-(2G-rhamnosyl)-rutinoside**	6.612_738.	NEG	6.612	738.2375
**Benzyloxycarbonyl-Val-Ala-Asp-fluoromethylketo**	7.131_453.	POS	7.131	453.19
**Benzenesulfonamide, N-(((1,1-dimethylethyl)amin**	5.931_406.	NEG	5.931	406.1314
**Cholylserine**	7.979_495.	POS	7.979	495.3201
**Dexelvucitabine**	5.691_227.	POS	5.691	227.0696
**Dodecanoyl-sn-glycero-3-phosphocholine**	9.72_439.2	NEG	9.72	439.2698
**Clocinnamox**	4.58_504.1	POS	4.58	504.1824
**3-[4-methoxy-2-[(2S,3R,4S,5S,6R)-3,4,5-trihydroxy**	6.371_375.	POS	6.371	375.151
**Azelastine**	9.786_381.	POS	9.786	381.1626
**3-Methoxybenzaldehyde**	4.014_136.	POS	4.014	136.0527
**[Hydroxy-(2,3,4,5,6-pentahydroxycyclohexyl)oxyph**	2.448_419.	POS	2.448	419.9618
**Nalfurafine**	4.988_476.	POS	4.988	476.2322
**Oxidized Cypridina luciferin;**	4.795_393.	POS	4.795	393.2265
**PC(22:6(4Z,7Z,10Z,13Z,16Z,19Z)/14:0)**	10.933_77	POS	10.933	777.5304
**Emerin**	2.41_316.1	POS	2.41	316.1199
**[(3aR,4S,6R,11aR)-6,9-dihydroxy-6,10-dimethyl-3-**	5.44_364.1	POS	5.44	364.1533
**Hesperetin 7-O-glucoside;**	5.76_464.1	POS	5.76	464.1321
**LMPR0104330010**	5.962_594.	POS	5.962	594.3218
**(2S)-2-Amino-3-[4-[(2S)-2-amino-3-(4-hydroxypl**	5.032_378.	POS	5.032	378.0981
Other				

N-(3-{1-[4-(3,4-Difluorophenoxy)benzyl]-4-piperi	5.913_478.	POS	5.913	478.2434
Succinylcarnitine	4.408_261.	POS	4.408	261.1215
Trilostane	4.988_329.	POS	4.988	329.1981
Tert-Butyl carbamate	0.872_117.	POS	0.872	117.0791
2-Propenoic acid, 2-(((2-methoxyphenyl)acetyl)am	5.701_235.	POS	5.701	235.0847
Norgalanthamine	6.598_273.	NEG	6.598	273.1363
Phlorizine	4.438_436.	POS	4.438	436.1349
LMPK12140327	5.275_352.	NEG	5.275	352.0254
Alfuzosin	2.597_389.	POS	2.597	389.2058
Delpazolid	2.489_308.	POS	2.489	308.13
alpha-Antiarin; Antiarigenin 3-O-beta-D-antiarosi	8.156_566.	POS	8.156	566.2743
Nalmefene	6.273_339.	POS	6.273	339.1832
1,17,19-trihydroxy-7-methyl-3-oxapentacyclo[9.8.	2.4_364.09	POS	2.4	364.094
Gepotidacin	8.025_448.	POS	8.025	448.2229
N-Oxo-3-pyridinecarboxamide	1.708_136.	POS	1.708	136.0274
PS(18:0/0:0)	9.303_525.	POS	9.303	525.3073
Cochinchinenin B	8.184_542.	POS	8.184	542.2328
3-Methyl-1-propylxanthine	3.052_208.	POS	3.052	208.0951
3-(3,3-dichloro-2-hydroxypropyl)-6,8-dimethoxyis	4.031_349.	POS	4.031	349.0486
PS(22:2(13Z,16Z)/17:2(9Z,12Z))	10.965_82	NEG	10.965	825.5549
4(1H)-Pyridinone, 3-hydroxy-1-(3-hydroxypropyl)	3.82_183.0	NEG	3.82	183.0896
2-(20-Hydroxyicosa-5,14-dienoylamino)acetic acid	7.87_381.2	POS	7.87	381.2878
LMST01080079	6.807_916.	POS	6.807	916.4681
1-Methyl-2-hydroadenosine	2.504_283.	POS	2.504	283.1279
Isovanillic acid	2.912_168.	POS	2.912	168.0425
Sinapinic acid	3.048_224.	POS	3.048	224.0663
3-O-Methyl-a-methyldopamine	4.37_181.1	NEG	4.37	181.1102
LysoPC(20:0/0:0)	10.077_55	POS	10.077	551.3962
PC(18:0/18:1(11Z))	12.895_78	POS	12.895	787.6088
AFN911	7.726_511.	POS	7.726	511.2694
Aminomethanesulfonic acid	0.587_110.	POS	0.587	110.9992
2,9-Dimethyl-4,7-diphenyl-1,10-phenanthroline	3.006_360.	POS	3.006	360.1644
25-Hydroxyvitamin D3 3-sulfate ester	9.26_480.2	POS	9.26	480.2914
Mivazerol	3.112_217.	POS	3.112	217.0853
2,5-Dimethoxy-4-(ethylthio)phenethylamine	4.772_241.	POS	4.772	241.1139
2-oxo-heneicosanoic acid	10.041_34	POS	10.041	340.2976
5'-(1,4-Dihydro-1-methyl-3-pyridinylcarbonyl)-3'-	10.076_38	POS	10.076	388.1476
para-hydroxyrosiglitazone	6.983_373.	POS	6.983	373.1083
Protosappanin A	5.933_272.	POS	5.933	272.0685
1,3-Dideacetyl-7-Deacetoxy-7-Oxokhivorin	4.345_458.	POS	4.345	458.2306
Cer(d15:1/16:0)	10.795_49	POS	10.795	495.4658
Oxidized dinoflagellate luciferin;	8.209_602.	POS	8.209	602.2753
Antimycin A	6.785_548.	NEG	6.785	548.2738
OOV-PI	7.556_696.	POS	7.556	696.3508
Iprovalicarb	7.083_320.	POS	7.083	320.21
Colforsin daropate	5.666_509.	POS	5.666	509.2964
N-Ethyl-1,1,2,2,3,3,4,4,5,5,6,6,7,7,8,8,8-heptadecafl	5.124_571.	NEG	5.124	571.0136
Calcipotriol	7.848_412.	POS	7.848	412.2977
Sunitinib	7.38_398.2	NEG	7.38	398.2129
Isrib	6.043_450.	POS	6.043	450.1132
Nervonic ceramide	10.712_64	POS	10.712	647.622
Megestrol	8.91_328.2	POS	8.91	328.2038
Petunidin 3-glucoside	5.973_479.	POS	5.973	479.1201
Elizabethkingia_miricola				
GW 6471;	5.864_635.	POS	5.864	635.2576
Isotectorigenin, 7-Methyl Ether	6.775_328.	POS	6.775	328.0945
LMGL02070004	10.083_60	POS	10.083	602.5283
N-Hydroxy phip	5.801_240.	POS	5.801	240.1

PE(16:1(9Z)/0:0)	9.598_451.	NEG	9.598	451.27
Callyspongendiol	10.379_43(	POS	10.379	436.3343
Penethamate	3.599_433.	POS	3.599	433.2032
Benzenesulfonamidothiourea	2.447_231.	POS	2.447	231.0125
N-(3-Chlorophenyl)-6,7-dimethoxyquinazolin-4-a	3.237_315.	POS	3.237	315.0775
L-Alanine 2-naphthylamide	6.591_214.	POS	6.591	214.1103
4H-Pyrido(1,2-a)pyrimidin-4-one, 9-((4-acetyl-3-h	6.961_420.	POS	6.961	420.1534
4-Fluoro-N-methyl-L-phenylalanyl-N-methyl-L-va	8.748_556.	NEG	8.748	556.343
L-2-Hydroxyphytanate;	10.421_32	POS	10.421	328.2976
Enilconazole	1.595_296.	POS	1.595	296.05
Alizapride	8.209_315.	POS	8.209	315.1708
3-Ethyl-4-(9-hydroxy-4,6,8,10-tetramethyl-7-oxo	10.071_35	POS	10.071	352.2613
Deoxybenzoin	7.949_196.	POS	7.949	196.089
2-(1-hydroxy-1-methoxy-3-methylbutyl)-6-methy	3.927_286.	POS	3.927	286.1528
6-Amino-9-benzyl-2-(2-methoxyethoxy)-9H-puri	7.636_315.	POS	7.636	315.1344
Cortisone	6.323_360.	POS	6.323	360.1935
NCGC00095690-03!(8,8-dimethyl-2-oxo-9,10-dihy	3.424_350.	POS	3.424	350.1114
[5-acetyloxy-3-(hydroxymethyl)-2-oxo-6-propan-	4.442_364.	POS	4.442	364.1271
Dehydrorabelomycin;	0.64_320.C	NEG	0.64	320.0677
Tetraethylene glycol monododecyl ether	9.492_362.	POS	9.492	362.3032
Clitidine; Chrytidine;	2.613_270.	POS	2.613	270.0851
[3,4,5-trihydroxy-6-[[(E)-3-(4-hydroxyphenyl)prop	6.271_478.	POS	6.271	478.1115
Palmitic acid	7.57_273.2	POS	7.57	273.2667
11-Mercapto-1-undecanol	2.9_204.15	POS	2.9	204.1555
Fumagillol	8.293_282.	POS	8.293	282.1831
[(2S,3S)-2-[(E,3R,4S)-3,4-dihydroxypent-1-enyl]-6	12.673_33	POS	12.673	334.0805
25-Hydroxyvitamin D2 25-(beta-glucuronide)	9.023_588.	POS	9.023	588.3668
PS(O-16:0/15:1(9Z))	8.198_705.	POS	8.198	705.4959
3-HYDROXY-4-(SUCCIN-2-YL)-CARYOLANE delta-	6.806_358.	POS	6.806	358.1527
cyclic N-Acetylserotonin glucuronide	4.202_392.	NEG	4.202	392.1221
(4-Amino-2-methylpyrimidin-5-YL)methyl dihydro	4.27_219.C	POS	4.27	219.041
Selenium Sulfide	0.706_111.	POS	0.706	111.8886
2-heptylquinolin-4(1H)-one	4.611_243.	POS	4.611	243.1622
PE(20:1(11Z)/13:0)	10.932_70	POS	10.932	703.5151
Z-Asp-Glu-Val-Asp-fluoromethylketone	7.365_626.	POS	7.365	626.2258
Glycerol 1-myristate	9.434_284.	POS	9.434	284.2351
1-(2,6-Dimethylphenoxy)-2-(3,4-dimethoxyphenyl	6.269_343.	POS	6.269	343.2146
Jaceidin 4'-glucuronide	6.248_536.	POS	6.248	536.1175
1-O-(2R-methoxy-4Z-eicosenyl)-sn-glycerol	10.232_40(	POS	10.232	400.3554
D-Sphingosine	8.509_299.	POS	8.509	299.2822
Hispolon	5.973_220.	POS	5.973	220.0737
Lacinilene C 7-methyl ether;	10.084_26(	POS	10.084	260.1413
2-[3-[2-(1,3-benzodioxol-5-yl)-7-methoxy-1-ben	4.771_521.	POS	4.771	521.1907
cortisol 21-sulfate	8.755_442.	POS	8.755	442.1664
N-Methyl-N-nitrosotoluene-4-sulphonamide (NTC	2.409_214.	POS	2.409	214.0414
Mirodenafil	5.793_531.	POS	5.793	531.2522
N-(4-bromo-benzenesulfon) arachidonoyl amine	8.233_521.	POS	8.233	521.1583
Ferutinin	8.229_358.	POS	8.229	358.2145
PG(17:1(9Z)/21:0)	8.192_804.	POS	8.192	804.5871
Benzamide, 4-chloro-N-(2-(3-oxo-4-morpholinyl)	3.966_282.	POS	3.966	282.0785
Acemetacin	5.867_415.	NEG	5.867	415.0838
TRIMETHYL PHOSPHATE	3.215_140.	POS	3.215	140.0241
(S)-2-Biphenyl-4-yl-1-(1H-tetrazol-5-yl)ethylamin	5.757_359.	POS	5.757	359.115
Cer(d18:0/20:4)	12.171_58	POS	12.171	587.5258
Phenolsulfonphthalein; Phenol red;	2.576_354.	POS	2.576	354.0556
Tazarotenic acid	3.081_323.	POS	3.081	323.0973
Oxybutynin	3.249_357.	POS	3.249	357.2311
16-Deacetylgeyerline	8.188_668.	POS	8.188	668.3332

**Pugnac**	4.16_353.1	NEG	4.16	353.1223
Succinivibrio_dextrinosolvens				
**Lappaol D;**	10.178_56	POS	10.178	568.2323
**PS(P-20:0/20:5(5Z,8Z,11Z,14Z,17Z))**	9.737_821.	NEG	9.737	821.5588
Cronobacter_dublinensis				
**Casticin 3'-glucoside**	5.239_536.	NEG	5.239	536.1554
**Desmethyl Bosentan**	4.343_537.	POS	4.343	537.1658
**2-Oxoglutaric acid**	0.639_146.	NEG	0.639	146.0217
**Phosphoric acid**	1.203_97.9	POS	1.203	97.97705
**PG(O-16:0/19:0)**	8.109_750.	POS	8.109	750.5755
**methyl 2-(2,6-dihydroxy-4-methylbenzoyl)-3-hydı**	4.967_349.	POS	4.967	349.1161
Other				
**8-Acetylneosolaniol**	7.453_424.	POS	7.453	424.1733
**dehydronifedipine**	4.81_344.1	POS	4.81	344.1006
Blautia_schinkii				
**Ikarisoside C**	8.2_824.27	POS	8.2	824.2725
**DG(20:4(8Z,11Z,14Z,17Z)/14:0/0:0)**	12.279_58	POS	12.279	588.4734
**Cumyluron;**	2.775_302.	POS	2.775	302.1173
Rubritepida_flocculans				
Saccharopolyspora_dendranthemae				
**Rostafuroxin**	8.35_374.2	POS	8.35	374.2457
**7-Oxononane-1,1,9-tricarbaldehyde**	8.224_226.	POS	8.224	226.1208
**6-Mercapto-1-hexanol**	0.75_134.0	POS	0.75	134.0767
**PE(O-16:0/19:0)**	12.485_71	POS	12.485	719.5831
**N,N-Diallyl-tyrosyl-alpha-aminoisobutyric acid-ph**	6.248_606.	POS	6.248	606.3444
**Ciclesonide**	6.576_540.	NEG	6.576	540.3111
**6-Hydroxyluteolin 6,3'-dimethyl ether 7-sulfate**	5.443_410.	NEG	5.443	410.0309
**NCGC00347618-02!3-hydroxy-N-(1-hydroxy-4-m**	3.145_359.	POS	3.145	359.1482
**Epimedin B**	8.196_808.	POS	8.196	808.2803
**Morphinan-6-ol, 7,8-didehydro-4,5-epoxy-3-meth**	8.182_299.	POS	8.182	299.1518
**LMPK12010401**	8.218_725.	POS	8.218	725.1948
**4-(Cyclohexyloxy)-2-(1-(4-[(4-methoxybenzene)sı**	8.191_510.	POS	8.191	510.2319
**Modithromycin**	8.197_840.	POS	8.197	840.4662
**Streptidine**	8.215_262.	NEG	8.215	262.1399
**dTDP-D-forosamine; dTDP-alpha-D-forosamine; d**	4.252_543.	NEG	4.252	543.1404
**4-Methyl-2-oxo-2H-chromen-7-yl sulfamate**	5.008_255.	POS	5.008	255.02
**Telenzepine**	5.032_370.	POS	5.032	370.1477
**2,2,2-Trifluoro-N-((1R,2S)-1-((1-(4-fluorophenyl)-**	5.263_487.	NEG	5.263	487.1507
**Pactimibe**	9.459_416.	POS	9.459	416.304
**(2R,3S,4S,5R,6R)-2-(hydroxymethyl)-6-[[(2R,3S,4S,**	4.293_551.	POS	4.293	551.2235
**4-[2-[5,5-Dimethyl-8-(4-methylphenyl)-6H-napht**	1.029_392.	NEG	1.029	392.1792
**2-O-(beta-D-galactopyranosyl-(1->6)-beta-D-gal**	12.679_57	POS	12.679	570.2906
**2-Acetylaminophenoxazin-3-one**	6.755_254.	NEG	6.755	254.0693
**(2R,3S,4S,5R,6S)-2-(hydroxymethyl)-6-[4-prop-2-**	3.591_491.	POS	3.591	491.2022
**BILIVERDIN**	7.614_582.	POS	7.614	582.2482
**Leucocrystal violet**	6.128_373.	POS	6.128	373.2464
**[5-[2,4-Bis((3S)-3-methylmorpholin-4-yl)pyrido[2,**	6.992_465.	POS	6.992	465.2382
**SN38 glucuronide carboxylate form;**	3.855_585.	NEG	3.855	585.1748
Other				
**Eplerenone**	6.103_414.	NEG	6.103	414.2042
**[3-hydroxy-1-(4-methoxy-7-oxofuro[3,2-g]chrom**	4.528_433.	POS	4.528	433.1734
**2-({6-Amino-3,5-dicyano-4-[4-(cyclopropylmetho**	8.183_379.	POS	8.183	379.1088
**5-hydroxy-2-(4-methoxyphenyl)-3-[(2S,3R,4S,5S,6**	7.082_608.	POS	7.082	608.1736
**Mureidomycin A; MRD A;**	4.641_840.	NEG	4.641	840.3074
**N-Acetyl-D-glucosaminate;**	0.67_237.0	NEG	0.67	237.0846
**Dinonylnaphthalene**	11.266_38	POS	11.266	380.3445
**Epiafzelechin Trimethyl Ether**	6.687_316.	NEG	6.687	316.1299
**Hymenin**	6.803_262.	POS	6.803	262.1205

**4-Hydroxybenzaldehyde**	3.901_104.	POS	3.901	104.0263
**Chelirubine;**	5.225_362.	NEG	5.225	362.1037
**PC(18:1(9Z)/17:0)**	12.43_773.	POS	12.43	773.5931
**Oxonal**	5.68_148.C	POS	5.68	148.0527
**Carfecillin**	1.192_454.	POS	1.192	454.1195
**((4-(4-Amidinophenoxy)butanoyl)aspartyl)valine**	6.988_436.	POS	6.988	436.1965
Sutterella_wadsworthensis				
**3-(((4,7-Dimethyl-1,3-benzoxazol-2-yl)methyl)ami**	4.934_311.	POS	4.934	311.1632
**NCGC00380970-01!3-hydroxy-2-[[3-(3-hydroxy-6**	5.689_399.	POS	5.689	399.262
**Cosahexaenoic acid**	8.932_300.	POS	8.932	300.2089
**(3-(2-(4-(2-(Trifluoromethyl)phenoxy)piperidin-1-**	4.58_426.C	NEG	4.58	426.0984
**PG(22:4(7Z,10Z,13Z,16Z)/20:0)**	8.221_854.	POS	8.221	854.6069
**Lysyl-aspartyl-glutamyl-leucine**	6.992_503.	POS	6.992	503.2566
**Clavamycin D;**	3.632_315.	POS	3.632	315.1431
**Nontrial**	7.284_212.	POS	7.284	212.1414
**Syringic acid**	4.732_198.	NEG	4.732	198.0528
**N-Acetyl gemifloxacin**	4.408_417.	NEG	4.408	417.1459
**norhydrocodone**	7.127_285.	POS	7.127	285.1363
**2-Furoylglycine**	2.747_169.	POS	2.747	169.0377
**Taisho**	3.333_389.	POS	3.333	389.1149
**7-[2,5-Dihydroxy-2-[3-hydroxy-4-[3-(trifluoromet**	5.721_458.	POS	5.721	458.1911
**Terephthalic acid**	10.084_16(	POS	10.084	166.0268
**Penicillanic acid**	4.436_201.	POS	4.436	201.0462
**4-[4-(3-Hydroxyphenyl)-3-(4-methylphenyl)-6-ox**	6.417_425.	POS	6.417	425.1389
**N-Acetyl-beta-D-glucosaminyl-(1->4)-D-glucosar**	9.787_382.	POS	9.787	382.1595
**Lolitrem J;**	4.798_661.	POS	4.798	661.3646
**latrunculin a**	3.601_421.	POS	3.601	421.194
**2,4-Diaminoazobenzene**	5.481_212.	POS	5.481	212.1052
**Verrucarol**	8.119_266.	POS	8.119	266.1519
**Etimicin**	8.298_477.	POS	8.298	477.3177
**N-Acetyl-S-allylcysteine**	2.597_203.	NEG	2.597	203.0617
**4-Methylumbelliferone**	6.824_193.	POS	6.824	193.0741
**3-(Hydroxymethyl)-5-oxopentanoic acid**	5.147_146.	NEG	5.147	146.058
**3-Hydroxyphenylpropionic acid glucuronide**	5.906_342.	POS	5.906	342.0951
**2-methyl-3-[(2S,3R,4S,5S,6R)-3,4,5-trihydroxy-6-(I**	2.576_310.	POS	2.576	310.0653
Other				
**Maraviroc**	8.334_513.	POS	8.334	513.3303
**PS(P-16:0/13:0)**	9.844_677.	POS	9.844	677.4627
Anaerovorax_odorimutans				
**Ac-Tyr-OEt; N-Acetyl-L-tyrosine ethyl ester; Ethyl I**	6.31_251.1	POS	6.31	251.1158
**Isousnic acid**	5.965_376.	POS	5.965	376.116
**Tremulacin;**	3.046_528.	NEG	3.046	528.1608
**2-(4-hydroxyphenyl)-7-[(2S,3R,4S,5S,6R)-3,4,5-trih**	4.621_418.	NEG	4.621	418.1266
**OON-PI**	4.72_752.4	NEG	4.72	752.4108
**3,4-Dehydrocilostazol**	5.112_367.	POS	5.112	367.1993
**Aculeacin A**	9.375_103!	POS	9.375	1035.578
**OHOOA-PA**	5.826_590.	POS	5.826	590.3234
**Butylone**	3.128_221.	POS	3.128	221.1055
**Dihydrofolic acid**	5.064_443.	NEG	5.064	443.1613
Gemmiger_formicilis				
**4-(4-Chlorophenyl)-1-[4-(4-fluorophenyl)-4-oxob**	6.095_354.	POS	6.095	354.1078
**Dopamine 4-sulfate**	3.201_233.	POS	3.201	233.036
**Sphing-6E-enine 4R-sufate**	7.483_395.	NEG	7.483	395.2342
**Bepafant**	6.27_467.1	POS	6.27	467.1188
**((2,6-Bis(1-methylethyl)phenoxy)sulfonyl)carbamic**	6.109_461.	NEG	6.109	461.2236
**Hydroxy Ritonavir**	4.988_736.	POS	4.988	736.3067
**Alangiside;**	5.871_505.	POS	5.871	505.1954
**2,2',6,6'-Tetranitro-4,4'-azoxytoluene;**	11.887_40(	POS	11.887	406.0499

**Asi-222**	8.843_519.	NEG	8.843	519.3205
Mitsuokella_jalaludinii				
Other				
**N-Phenylacetylglutamine**	4.029_264.	POS	4.029	264.1111
**(1R,2R,4S,16R,17R,20S)-13,20-dimethoxy-4,7,17,22**	7.576_516.	POS	7.576	516.2383
**Arecoline**	5.01_155.0	POS	5.01	155.095
**Pyricarbate**	4.207_253.	NEG	4.207	253.1072
**2-[(3R)-1-(2,2-Diethoxyethyl)-3-[(4-methylphenyl**	7.259_544.	NEG	7.259	544.2697
**Taurochenodeoxycholic acid sodium salt**	11.286_52	POS	11.286	521.2795
**Tetrahydrocurcumin**	6.78_372.1	NEG	6.78	372.1573
**Gatifloxacin**	4.985_375.	POS	4.985	375.1577
**1-(2-methoxy-6Z-heptadecenyl)-sn-glycero-3-ph**	10.065_52	NEG	10.065	525.3064
**Calcifediol lactone**	7.352_428.	POS	7.352	428.2922
**Thermocryptoxanthin-11;**	8.834_882.	NEG	8.834	882.6364
**(2S,3S,4S,5R,6S)-3,4,5-trihydroxy-6-(5-hydroxy-4-**	3.781_430.	NEG	3.781	430.0901
**4'-Hydroxyflurbiprofen**	4.854_260.	POS	4.854	260.0852
**Thromboxane A3**	7.05_350.2	POS	7.05	350.2089
**MeOSuc-AAPA-CMK**	5.823_474.	POS	5.823	474.1868
**26-Hydroxycastasterone;**	9.225_480.	POS	9.225	480.3456
**2,4-Oxazolidinedione**	4.185_101.	NEG	4.185	101.0113
**7,15-bis(3,4-dimethoxyphenyl)-19-oxa-4,18-diaza**	8.21_584.2	POS	8.21	584.2276
**LMPK12120481**	7.548_696.	POS	7.548	696.2704
**Benzo[a]pyrene diol**	4.637_284.	POS	4.637	284.0829
**2-Propenyl cyclohexanepentanoate**	9.022_224.	NEG	9.022	224.1776
**2-(2-Oxopropanoyloxy)butanedioic acid**	0.685_204.	NEG	0.685	204.0278
**Cucurbitacin B 2-sulfate**	10.102_63	POS	10.102	638.2743
**Methyl 2,6-dimethyl-5-nitro-4-[2-(trifluoromethyl**	5.651_356.	POS	5.651	356.0983
**4,6,11-hexadecatrienal**	9.315_234.	POS	9.315	234.1985
**PG(16:1(9Z)/0:0)**	9.943_482.	NEG	9.943	482.264
**Riddelliine**	6.794_349.	POS	6.794	349.1523
**Azulene**	8.051_128.	POS	8.051	128.0627
**Phe-Tyr**	8.547_328.	POS	8.547	328.1422
Carnobacterium_gallinarum				
**PON-PE**	9.583_607.	POS	9.583	607.386
**[(2R,3S,4R)-5-(2,4-dioxopyrimidin-1-yl)-4-hydroxy**	4.897_446.	POS	4.897	446.0722
**Methyl 3,4,5-trimethoxycinnamate**	5.752_274.	POS	5.752	274.082
**POB-PS**	6.808_581.	POS	6.808	581.297
**Daidzein 4'-sulfate**	5.958_334.	POS	5.958	334.0138
**3,4-dihydroxy-5-[(2S,3R,4S,5S,6R)-3,4,5-trihydroxy**	5.955_498.	POS	5.955	498.1756
**THYMOLPHTHALEIN**	6.645_430.	POS	6.645	430.2137
**beclomethasone propionate**	4.613_480.	POS	4.613	480.1934
**(6RS)-6,19-epidioxy-24,24-difluoro-25-hydroxy-6,**	9.136_468.	POS	9.136	468.305
**Prothoate;**	4.541_285.	POS	4.541	285.0612
**5-amino-3-[(2E)-2-(3,5-dimethyl-2-oxocyclohexyl**	7.303_298.	POS	7.303	298.1892
**2-(p-Acetamidophenyl)-2-ethylglutarimide**	4.41_274.1	POS	4.41	274.1317
**methyl 2-ethyl-4-[(3R,4R,5S)-5-hydroxy-4,5-dime**	3.28_303.1	POS	3.28	303.1681
**Lagochilin**	8.723_356.	POS	8.723	356.2563
**Cer(d14:2(4E,6E)/16:0)**	10.362_47	POS	10.362	479.4343
**PE(20:2(11Z,14Z)/18:3(6Z,9Z,12Z))**	10.837_76	POS	10.837	765.5309
**3-Cyclohexyl-1-propylsulfonic acid**	3.156_221.	POS	3.156	221.1089
**Cyproconazole**	4.704_291.	POS	4.704	291.1139
**N-Acetylglucosamine 6-sulfate**	0.668_301.	NEG	0.668	301.0465
**Gracilosulfate B**	8.41_544.3	NEG	8.41	544.3064
**Azepexole**	2.615_181.	POS	2.615	181.1218
**Mycinonic acid III**	7.39_240.1	NEG	7.39	240.1361
**Citrinin**	4.565_250.	NEG	4.565	250.084
**Musca-aurin-VII; Muscaaurin VII;**	6.809_348.	POS	6.809	348.107
**Deepoxy-deoxynivalenol**	6.316_280.	POS	6.316	280.131

**3-Methylsulfinylpropyl isothiocyanate**	4.385_163.	POS	4.385	163.0127
**Glycerol phenylbutyrate**	6.711_530.	POS	6.711	530.2666
**2,3-Diethyl-6-hexoxycarbonylbenzoic acid**	10.278_30	POS	10.278	306.1829
**Ticlopidine**	6.016_263.	NEG	6.016	263.0533
**Pirsidomine**	6.448_330.	NEG	6.448	330.1679
**Hydroxy Bosentan**	0.732_567.	POS	0.732	567.1782
**Depropionylbezitramide**	3.242_436.	POS	3.242	436.2243
**Capsaicin**	6.94_305.1	POS	6.94	305.1987
**MGMG(16:2(7Z,10Z)/0:0)**	9.824_488.	NEG	9.824	488.2987
**Ipamorelin**	6.891_711.	POS	6.891	711.385
**beta-Hydroxy-gamma-trimethylaminobutyric acid**	7.095_161.	POS	7.095	161.1054
Marinobacterium_maritimum				
**Norsolorinic acid; Norsolorinate; 2-Hexanoyl-1,3,6,**	1.005_370.	POS	1.005	370.106
**Quercetin 3'-sulfate**	5.531_381.	NEG	5.531	381.9995
**Loganoside**	3.821_390.	POS	3.821	390.1527
**Cbmida**	5.442_400.	NEG	5.442	400.1133
**Resorcinol monoacetate**	4.331_152.	POS	4.331	152.0475
**Azithromycin**	5.285_748.	POS	5.285	748.5073
**Pentachloroethane**	0.744_199.	POS	0.744	199.8522
**abietic acid**	9.738_302.	NEG	9.738	302.2244
**1-Pyrenemethanol**	3.24_232.0	POS	3.24	232.0882
**NCGC00384809-01!5-[5-hydroxy-3-(hydroxymeth**	10.118_28	POS	10.118	282.1985
**rac-Glycerol 1-myristate**	9.432_302.	POS	9.432	302.2457
**PG(20:5(5Z,8Z,11Z,14Z,17Z)/0:0)**	6.557_530.	NEG	6.557	530.2636
**Thymidine 5'-monophosphate**	6.426_322.	NEG	6.426	322.0511
**LMPR0106180020**	7.861_548.	POS	7.861	548.3725
**3,4,7-Trihydroxy-5-methoxy-8-prenylflavan 4-O-(**	5.73_666.2	POS	5.73	666.2542
**methyl 2-[(4aR,5R,6R,7S,8aR)-8a-ethenyl-5,7-dihy**	3.239_346.	POS	3.239	346.0833
**methyl (1S,2R,4R,8R,9S,10R,13R,15R)-2-(acetyloxy)**	8.933_540.	POS	8.933	540.2383
**PE(16:0/20:4(5Z,8Z,11Z,14Z))**	12.144_73	NEG	12.144	739.5175
**Bakankoside;**	3.394_357.	NEG	3.394	357.1425
**Phorbol 12,13-diacetate**	7.551_448.	POS	7.551	448.2078
**Dehydrocostus lactone**	6.212_230.	POS	6.212	230.1304
**LMST01130036**	7.131_578.	POS	7.131	578.2744
**Alogliptin**	4.165_339.	POS	4.165	339.168
**Bumetanide**	5.1_364.10	POS	5.1	364.1092
**Isopalmitic acid**	9.803_256.	POS	9.803	256.2403
Chromobacterium_haemolyticum				
Propioniciclava_tarda				
**Dioxybenzone**	5.287_244.	POS	5.287	244.0736
**Hexyl-agarose**	8.838_714.	NEG	8.838	714.2914
**7-Hydroxycoumarine**	5.043_162.	POS	5.043	162.0319
**13-Deoxydaunorubicin**	3.242_513.	POS	3.242	513.2019
**Elbasvir**	6.6_881.42	NEG	6.6	881.4261
**4-ene-Valproic acid**	9.027_142.	POS	9.027	142.0996
**Dextromethorphan**	5.864_271.	POS	5.864	271.1937
**Pregnanetriol 3a-O-b-D-glucuronide**	8.215_512.	POS	8.215	512.2991
**Simvastatin**	8.317_418.	POS	8.317	418.2719
**5'-Deoxy-5'-fluorouridine**	5.567_246.	POS	5.567	246.0641
**DG(18:1(9Z)/18:1(9Z)/0:0)**	10.028_63	POS	10.028	637.5648
**Delphinidin 3-glucoside**	5.971_465.	POS	5.971	465.1029
**(2s)-7-Amino-2-{[(R)-Hydroxy{(1r)-2-Methyl-1-[(**	6.36_426.2	POS	6.36	426.2267
**6-(furan-3-yl)-13-hydroxy-5,12,16,16-tetramethyl**	4.789_485.	POS	4.789	485.2055
**Pilocarpine**	3.079_208.	POS	3.079	208.1215
**Probenecid Glucuronide**	6.116_461.	POS	6.116	461.137
**[3-(13-methyltetradecyl)-5-sulfooxyphenyl] hydro**	12.989_51	POS	12.989	518.1397
**Kelatorphan**	1.16_294.1	NEG	1.16	294.1213
**Linoleylanilide**	8.468_355.	POS	8.468	355.2874

**Avenanthramide P**	5.984_355.	POS	5.984	355.1054
**glas#18**	8.568_494.	POS	8.568	494.2713
**Tetrahydrofuranylmethyl**	1.737_84.0	POS	1.737	84.05766
**Imidazolelactic acid**	2.08_156.0	POS	2.08	156.0537
**1,1-Diphenyl-2-(4-methoxyphenyl)propene;**	6.874_300.	POS	6.874	300.1503
**Chlorhexidine**	7.552_504.	POS	7.552	504.2009
**2-Methyl-5-nitroimidazol-1-ylacetic acid**	2.533_185.	POS	2.533	185.0439
**Borapetoside**	6.487_538.	POS	6.487	538.2032
**Dansylsarcosine**	3.326_322.	POS	3.326	322.0985
**N-a-L-Acetyl-arginine**	3.698_216.	NEG	3.698	216.1197
**2-Phosphonopropionic acid**	0.928_154.	POS	0.928	154.0033
**Nephrin**	9.748_460.	POS	9.748	460.3916
**Diminazene**	0.74_281.1	POS	0.74	281.1377
**Milciclib**	4.937_460.	POS	4.937	460.2687
**(1R,4S,7S,8S,9R,12E,20R,21R,22S,23S)-7,8,21,22,23-**	5.355_479.	POS	5.355	479.2346
**Heptadec-2-enoic acid**	9.069_268.	POS	9.069	268.2403
**3-O-(beta-D-glucopyranosyl-(1->6)-beta-D-gluco**	8.663_512.	POS	8.663	512.2481
Staphylococcus_sciuri				
**Histidinohydroxylysinonorleucine**	3.08_444.2	POS	3.08	444.2335
**5alpha-Androstane-3beta,7alpha,17beta-triol**	9.244_308.	POS	9.244	308.2351
Spirosoma_endophyticum				
Kurthia_zopfii				
Zoogloea_resiniphila				
Paenibacillus_ginsengarvi				
Flectobacillus_roseus				
Methylocystis_echinoides				
Pediococcus_damnosus				
**Manassantin B**	6.616_716.	NEG	6.616	716.323
**2-((2-Methoxy-2-(3-(trifluoromethyl)phenyl)ethyl)**	8_616.252	POS	8	616.253
**3-Amino-2,3-dihydrobenzoic acid**	2.021_139.	NEG	2.021	139.0634
**Ramifenazone**	6.254_245.	POS	6.254	245.1527
**(1R,3S,6S)-6-[18-[(1S,4S,6R)-4-Hydroxy-2,2,6-trim**	10.046_60	POS	10.046	600.4178
**NCGC00380826-01![1-(3,4-dihydroxy-5-methoxyp**	5.879_426.	POS	5.879	426.1656
**(2R,3R,4S,5S,6R)-2-[(2E)-4-ethenyl-2,5-dimethylhe**	4.447_331.	POS	4.447	331.1994
**(S)-Methyl 2,5-diamino-5-oxopentanoate**	0.67_160.0	NEG	0.67	160.0848
**(2S)-8-[(E)-3-hydroxy-3-methylbut-1-enyl]-5,7-di**	6.338_390.	POS	6.338	390.1426
**LMPK12110178**	6.634_320.	POS	6.634	320.1057
**Fosfluconazole**	0.67_386.0	NEG	0.67	386.0694
**Ximelagatran**	7.378_473.	POS	7.378	473.263
**Halobetasol**	6.232_428.	POS	6.232	428.1586
**Ethyl 3-((6-(4,5-dihydro-1H-benzo[d]azepin-3(2H)**	6.926_417.	POS	6.926	417.2182
**3-hydroxybenzoic acid-3-O-sulphate**	3.362_217.	POS	3.362	217.9879
**(2-Naphthalenyloxy)acetic acid**	4.731_202.	POS	4.731	202.0633
**S)-2-(5(((1,2-Dihydro-3-methyl-1-oxobenzo(F)qui**	7.347_500.	POS	7.347	500.17
**(2R,3R,4R,5S)-2-(Hydroxymethyl)-1-nonylpiperidir**	3.352_289.	POS	3.352	289.2255
**4-(4-(Benzo[d][1,3]dioxol-5-yl)-5-(pyridin-2-yl)-1**	6.317_384.	NEG	6.317	384.1241
**Diosgenin**	8.716_396.	POS	8.716	396.3029
**NCGC00380330-01!2-(6-methyl-7-oxooctyl)-2H-fu**	7.331_224.	POS	7.331	224.1414
**Bufogargarizin C**	6.867_362.	POS	6.867	362.1868
**6-propionyl n-caproic acid**	5.865_172.	POS	5.865	172.1102
Papillibacter_cinnamivorans				
**1,25-Dihydroxyvitamin D3 monoglucuronide**	8.78_592.3	NEG	8.78	592.3615
**11A-Acetoxyprogesterone**	7.762_389.	POS	7.762	389.2565
**26-Deoxyactein**	10.072_66	POS	10.072	660.3879
**NCGC00380621-01!4-oxo-4-(3-oxodecan-2-ylami**	7.053_253.	POS	7.053	253.1678
**N-[1-[4-[(2,4-Dimethoxyphenyl)methyl]-5-(2-phe**	10.375_58	POS	10.375	586.2698
**Amoxapine**	5.698_313.	POS	5.698	313.0981
**Riboflavin**	4.384_376.	POS	4.384	376.1383

**Tubocurarine**	8.701_609.	NEG	8.701	609.2974
**7-[(Gly-L-Phe-)Amino]-4-(trifluoromethyl)-2H-1-I**	6.91_433.1	POS	6.91	433.1228
**Sphingofungin C**	8.546_431.	NEG	8.546	431.2886
**Wighteone**	1.594_338.	POS	1.594	338.1146
**(Z)-3-(1,2-Dihydroxyethyl)-3-hydroxyhenicos-12-**	10.136_38	POS	10.136	384.3239
**(Z)-2-octylpent-2-enedioic acid**	4.412_259.	POS	4.412	259.1785
**Lomustine**	1.101_233.	POS	1.101	233.0934
**Isofuranodiene**	10.135_21	POS	10.135	216.1517
**13-Acetylphorbol**	6.488_406.	NEG	6.488	406.1993
**9-Hydroxymyristic acid**	8.94_244.2	NEG	8.94	244.2037
**sphingosine phosphate**	6.546_391.	POS	6.546	391.2106
**Methotrexate**	3.843_454.	POS	3.843	454.1708
**5,4'-Dimethyl-6-hydroxyluteolinidin**	3.579_317.	POS	3.579	317.1012
**Farnesyl pyrophosphate**	4.221_382.	NEG	4.221	382.1265
**4,7-Dioxoheptanoic acid**	3.069_158.	POS	3.069	158.0583
**Mocetinostat**	7.756_396.	NEG	7.756	396.1687
**Glycyl-L-leucine**	3.039_188.	POS	3.039	188.1163
**Roquefortine C;**	6.308_389.	POS	6.308	389.1869
**Aacocf3**	9.41_356.2	POS	9.41	356.2326
**Coumarin-3-carboxylic acid succinimidyl ester**	3.364_287.	POS	3.364	287.0416
**NCGC00385796-01![(2R,3R,4S,5S,6R)-3,5-diacetylo**	6.781_315.	POS	6.781	315.13
**2-[(2S,4aR,8aS)-2-hydroxy-4a-methyl-8-methylide**	4.675_267.	POS	4.675	267.1834
**2-Amino-3-hydroxy-3-phenylpropanoic acid**	0.646_181.	NEG	0.646	181.0739
Fonticella_tunisiensis				
**5-O-Benzyl 3-O-ethyl 2-methyl-6-phenyl-4-(2-ph**	6.629_477.	POS	6.629	477.194
**[(2R,4S,5R)-5-ethenyl-1-azabicyclo[2.2.2]octan-2-**	8.829_362.	POS	8.829	362.1406
**Hygromycin B; Antibiotic A-396-II;**	2.371_527.	POS	2.371	527.235
**Nelipepimut-S**	8.103_994.	POS	8.103	994.5805
**2-Hydroxyhexadecanoylcarnitine**	8.814_425.	POS	8.814	425.3506
**3-Pyridazinamine, N-(1-((3,4-difluorophenyl)meth**	5.031_372.	POS	5.031	372.1363
**methyl 5-hydroperoxy-6,8,9,11-bisepidioxy-12,14-**	7.368_414.	POS	7.368	414.2256
Victivallis_vadensis				
**Androstenedione**	7.74_286.1	POS	7.74	286.1932
**1-[[(2S,3S,4S,5R)-3,4-Dihydroxy-5-(hydroxymethyl**	3.204_398.	POS	3.204	398.1437
**PA(10:0/10:0)**	6.306_480.	POS	6.306	480.2837
**Pravastatin lactone**	8.246_406.	POS	8.246	406.2361
Other				
**Mifepristone**	9.289_429.	POS	9.289	429.2663
**Quinoline-4-carboxylic acid**	5.966_173.	POS	5.966	173.0478
**6-[3-(1-Adamantyl)-4-hydroxyphenyl]-2-naphthal**	7.131_398.	POS	7.131	398.1869
**(1R,2S,3R)-1-[(2E)-2-(1-Nitrosoethylidene)-1,3-di**	0.727_245.	NEG	0.727	245.101
Butyricimonas_virosa				
**methyl 3-(3-methylbut-2-enyl)-1,4-bis[[3,4,5-trihy**	6.973_610.	POS	6.973	610.2269
**PC(16:1(9Z)/16:1(9Z))**	11.041_72	POS	11.041	729.5309
**SOLASODINE**	8.64_413.3	POS	8.64	413.3293
**Phosacetim;**	0.693_373.	POS	0.693	373.9813
**silybin**	5.86_482.1	POS	5.86	482.1217
**Tetradecadienoylcarnitine**	6.896_367.	POS	6.896	367.2722
**Berberine**	5.123_335.	POS	5.123	335.1156
**Cholecalciferol**	9.246_384.	POS	9.246	384.3392
**L-Isoleucine**	1.475_131.	POS	1.475	131.0947
Providencia_thailandensis				
**Propacetamol**	4.346_264.	NEG	4.346	264.1471
**S-Oblatone B**	8.233_430.	POS	8.233	430.2725
**TRIDESACETOXYKHIVORIN**	6.83_460.2	NEG	6.83	460.2463
Corynebacterium_atrinae				
**Linusic acid**	6.837_380.	POS	6.837	380.241
**4-Amino-2-hydroxylamino-6-nitrotoluene;**	3.64_183.0	POS	3.64	183.0644

**7(1)-Hydroxychlorophyllide a;**	7.185_630.	POS	7.185	630.2327
**Limonin**	5.745_487.	POS	5.745	487.2225
**[(1S,2S,3R,5R,6S,8S)-6-hydroxy-8-methyl-3-{[(2S,3**	4.541_497.	POS	4.541	497.19
**AG 85 (pharmaceutical)**	5.777_482.	POS	5.777	482.1408
**9-phenyl-1-(2,4,6-trihydroxyphenyl)nonan-1-one**	6.736_364.	POS	6.736	364.1633
**Etalocib**	7.886_544.	POS	7.886	544.2288
Other				
**Metipamide**	3.087_339.	NEG	3.087	339.046
**PRI_303.1340_15.8**	7.261_302.	POS	7.261	302.1265
**(Betas)-beta-(((2S)-2-(4,4-Dimethyl-3-((4-((((2-me**	8.145_627.	POS	8.145	627.3072
**6-bromo-pentacosa-5E,9Z-dienoic acid**	5.371_456.	POS	5.371	456.2592
**Tauro-alpha-muricholic acid**	5.365_515.	NEG	5.365	515.2909
**Prostaglandin F2a 1,9-lactone**	7.536_353.	POS	7.536	353.2564
**Albendazole-gamma-hydroxysulphone;**	3.695_313.	POS	3.695	313.0729
**(+/-)11(12)-EET**	9.179_302.	POS	9.179	302.2245
**PE(18:0/20:3(8Z,11Z,14Z))**	11.334_76	POS	11.334	769.5617
**MG(0:0/18:1(11Z)/0:0)**	9.732_356.	NEG	9.732	356.2927
**N-Desmethyl Topotecan**	6.047_407.	NEG	6.047	407.1496
**(2S,3R,4S,5S,6R)-2-[4-[(E)-2-(3,5-dihydroxyphenyl**	5.84_420.1	POS	5.84	420.14
**Mivotilate**	0.697_330.	NEG	0.697	330.0175
**(2R,3S)-Epoxiconazole**	2.584_329.	POS	2.584	329.0746
**17(S)-HETE**	9.634_302.	POS	9.634	302.2245
**(3aR,4R,6E,10Z,11aR)-4-hydroxy-10-(hydroxymeth**	6.583_296.	POS	6.583	296.1259
**N-[2-(4-Chloro-phenyl)-acetyl]-N'-(4,7-dimethyl-**	5.167_367.	POS	5.167	367.12
**trans-isoeugenol-O-glucuronide**	5.05_340.1	POS	5.05	340.1157
**7-[(7S)-7-Amino-5-azaspiro[2.4]heptan-5-yl]-8-cł**	5.919_409.	POS	5.919	409.0997
**Chamazulene;**	7.995_184.	POS	7.995	184.1253
**reticulol**	4.393_222.	POS	4.393	222.0531
**1-Heneicosanoyl-glycero-3-phosphoserine**	9.99_567.3	NEG	9.99	567.3544
**[(4R,6aR,9S,9aR,9bR)-9-methyl-3,6-dimethylidene-**	6.675_491.	POS	6.675	491.2161
**Picrotin**	4.012_332.	POS	4.012	332.0859
**Propyl chloroformate**	1.175_122.	POS	1.175	122.0134
**2-Chloro-1-(chloromethyl)ethyl carbamate**	0.588_170.	POS	0.588	170.9847
**Guanosine, 2'-deoxy-N-(7,8,9,10-tetrahydro-7,8,9-**	4.788_569.	POS	4.788	569.1912
**11-Deoxy prostaglandin F1a**	8.834_362.	POS	8.834	362.2459
**Diethylstilbestrol**	7.545_268.	POS	7.545	268.1462
**Phenyl-agarose**	5.155_706.	NEG	5.155	706.229
**5-[(E)-3-hydroxybut-1-enyl]-4-[4-[(E)-3-oxobut-1**	8.184_332.	POS	8.184	332.1737
**Perindopril Acyl-beta-D-glucuronide**	4.609_544.	POS	4.609	544.2659
**Amrubicin**	6.658_483.	POS	6.658	483.1544
**Protoapigenone**	5.966_286.	POS	5.966	286.0478
**1,2-Ethanediol monoricinoleate**	9.349_342.	POS	9.349	342.277
**Fenpropathrin**	3.634_349.	POS	3.634	349.1663
**3-O-Acetylpinobanksin**	5.275_314.	POS	5.275	314.079
**Edulenane**	4.636_366.	POS	4.636	366.1465
**LMPK12112303**	1.094_470.	POS	1.094	470.0849
Other				
**n6-[2-(4-Aminophenyl)ethyl]adenosine**	3.147_386.	POS	3.147	386.1689
**Acetaminophen glucuronide**	0.732_327.	NEG	0.732	327.0954
**Phloretin**	5.281_274.	NEG	5.281	274.0872
**2,5-Dichloro-4-oxohex-2-enedioate**	0.681_225.	POS	0.681	225.9442
**5,9:7,10a-Dimethano-10aH-[1,3]dioxocino[6,5-d]p**	0.632_319.	NEG	0.632	319.1014
**6,8a-Seco-6,8a-deoxy-5-oxoavermectin ''1b'' agly**	7.929_554.	NEG	7.929	554.3269
**Eriojaposide B;**	12.669_51	POS	12.669	516.2557
**Fluocortolone Pivalate**	6.405_460.	POS	6.405	460.2609
**cyclopenin**	10.224_29	POS	10.224	294.1004
**4,5-dihydroxy-4,5,6-trimethyl-2,8-dioxa-13-azatri**	4.985_363.	POS	4.985	363.1068
**Valylglycine**	1.194_174.	POS	1.194	174.1008

**O-ACETYLCARNITINE**	0.743_203.	POS	0.743	203.1159
**Etomidate**	6.269_244.	NEG	6.269	244.121
**N-(4-(4-Amino-2-ethyl-imidazo(4,5-c)quinolin-1-**	6.088_361.	POS	6.088	361.1562
**Delphinidin 3,5,3'-triglucoside;**	5.931_789.	POS	5.931	789.2125
**Caspase-1 Inhibitor VI**	8.28_630.2	POS	8.28	630.2688
**Meleagrin;**	8.129_433.	POS	8.129	433.1767
**6-Chloro-2,3,4,5-tetrahydro-1H-3-benzazepine**	3.887_181.	POS	3.887	181.065
**Naproanilide;**	1.209_291.	POS	1.209	291.1253
**(-)-Asbestinine 2**	8.217_476.	POS	8.217	476.3145
**N-(9,12-octadecadienoyl)-glutamine**	9.428_408.	POS	9.428	408.2988
**Trihexosylceramide (d18:1/16:0)**	8.75_1023.	POS	8.75	1023.671
**1-Methyl-2-nonyl-4(1H)-quinolinone**	5.182_285.	POS	5.182	285.2093
**1-Methylguanine**	2.516_165.	POS	2.516	165.0653
**Iriflophenone**	4.65_246.C	POS	4.65	246.053
**Phthalamic acid**	2.96_165.C	NEG	2.96	165.0427
**5'-O-beta-D-Glucosylpyridoxine**	3.841_331.	POS	3.841	331.1281
**Cucurbitacin E**	8.612_556.	NEG	8.612	556.3056
**O-Demethylpuromycin;**	6.609_457.	NEG	6.609	457.2088
**Bilirubin diglucuronide**	10.399_93	POS	10.399	936.3291
**Lolitrem K;**	9.837_601.	POS	9.837	601.3407
**Norcotinine**	5.44_162.C	POS	5.44	162.0795
**Veratrole**	5.038_138.	POS	5.038	138.0683
**Pavoninin 1;**	10.114_66	POS	10.114	661.4196
**Disulfiram**	2.577_296.	POS	2.577	296.0499
**PG(14:1(9Z)/14:1(9Z))**	3.984_662.	POS	3.984	662.4149
**N-Methylephedrine**	3.874_179.	POS	3.874	179.1312
**Tyrosol 4-sulfate**	4.253_218.	NEG	4.253	218.0248
**(11Z)-8,18-methano-retinal**	7.963_296.	POS	7.963	296.2139
**Methotrimeprazine**	8.103_328.	NEG	8.103	328.1616
**5-Oxozaleplon**	5.089_321.	POS	5.089	321.124
**Pentaleno(1,6a-c)pyran-5-carboxylic acid, 1-(chlor**	3.713_312.	POS	3.713	312.0779
**Gracilosulfate E**	8.194_542.	POS	8.194	542.2903
**N-Glycolyl-D-mannosamine 6-phosphate;**	6.831_317.	POS	6.831	317.0518
Pseudomonas_lurida				
**(E,2S,3R)-5-[3-hydroxy-2-(hydroxymethyl)phenyl]**	5.232_241.	POS	5.232	241.1316
**Homovanillyl alcohol sulfate**	3.225_248.	POS	3.225	248.0349
Methanobrevibacter_olleyae				
**Glycodiazine**	5.986_309.	POS	5.986	309.0768
**NCGC00180209-02!4-methoxy-3-methylsulfanyl-2**	1.593_297.	POS	1.593	297.0534
**Evoxine**	6.793_347.	POS	6.793	347.1367
**(1-Hexyl-1H-indol-3-yl)(naphthalen-1-yl)methanc**	2.39_355.1	POS	2.39	355.1937
**N4-Acetylsulfamethoxazole**	3.16_295.C	POS	3.16	295.0627
**N-Acetylglycine**	3.882_99.C	POS	3.882	99.03224
**2-(4-Hydroxy-3,5-di-tert-butylphenylthio)-hexanc**	9.253_352.	POS	9.253	352.2075
**Celanide**	5.629_100	POS	5.629	1006.474
**2,4-Diamino-6-dimethylamino-1,3,5-triazine**	5.139_154.	POS	5.139	154.0971
**(-)-erythro-Anethole glycol 2-glucoside**	6.909_361.	POS	6.909	361.172
**2-bromo-decanoic acid**	3.115_250.	POS	3.115	250.0564
**PC(14:1(9Z)/20:3(8Z,11Z,14Z))**	10.838_75	POS	10.838	753.53
Bacteroides_uniformis				
**D-erythro-1-(Imidazol-4-yl)glycerol 3-phosphate;**	3.646_238.	POS	3.646	238.0363
**PHOHA-PE**	5.948_593.	POS	5.948	593.3302
**7-Sulfocholic acid**	6.924_488.	NEG	6.924	488.2441
**Famotidine**	0.725_337.	POS	0.725	337.0433
**Tezosentan**	6.193_605.	NEG	6.193	605.1782
**5,10-dimethoxy-2,2-dimethylpyrano[3,2-g]chrome**	6.793_305.	POS	6.793	305.1262
**Glutamine glutamate aspartate**	6.25_565.1	POS	6.25	565.1485
Prevotella_loescheii				

**Aspirin**	5.041_180.	POS	5.041	180.0425
**5,6-undecadien-8,10-diyn-1-ol**	10.136_16(	POS	10.136	160.089
**Ergosine**	7.449_547.	NEG	7.449	547.2807
**5,6-dihydroxy-9,12,13-trimethyl-14-(2-methylpro**	5.867_455.	POS	5.867	455.2093
**(7E,13E)-9,15-dihydroxy-4,10,16-trimethyl-1,5,11-**	6.328_342.	NEG	6.328	342.1316
**Phenylalanylphenylalanine**	5.706_312.	NEG	5.706	312.1474
**(11S,14S,17R,20S,23R)-20-Benzyl-17-[3-(diaminon**	6.723_813.	POS	6.723	813.4246
Vibrio_mimicus				
**3beta,15alpha,16alpha,24alpha-tetrahydroxy25,26,**	8.136_578.	POS	8.136	578.3457
**Triphenylsilanol; Triphenylhydroxysilane;**	4.452_276.	POS	4.452	276.0973
**s-Allyl cysteine sulfoxide**	4.412_209.	NEG	4.412	209.018
**1-(6-Aminopurin-9-yl)-5-methylpyrimidine-2,4-di**	6.142_259.	POS	6.142	259.0821
**Deoxyaminopteroic acid**	3.748_325.	NEG	3.748	325.1274
**Flamprop-M;**	3.062_321.	POS	3.062	321.0553
**cis-(Homo)3-aconitate;**	6.853_216.	NEG	6.853	216.0634
**(R)-2-Amino-3-(2-propynylthio)propanoic Acid**	5.502_159.	NEG	5.502	159.0355
**2-[4-[(3S,3aR,6S,6aR)-6-[3-methoxy-4-[3,4,5-trihy**	6.792_682.	POS	6.792	682.248
**17-Estradiol cyclooctyl acetate**	10.198_42	POS	10.198	424.2982
**2-Oxo-7-methylthioheptanoic acid; 7-(Methylthio)**	7.524_190.	POS	7.524	190.0657
Other				
**Glycine, L-gamma-glutamyl-2-(1-methyl-4-(nitros**	4.006_419.	POS	4.006	419.1493
Alistipes_timonensis				
**2,6-Diamino-7-hydroxy-azelaic acid;**	5.033_234.	POS	5.033	234.1217
**Niridazole**	3.797_214.	POS	3.797	214.0169
**Resiniferatoxin**	7.641_650.	POS	7.641	650.2591
**Tachysterol**	9.512_396.	POS	9.512	396.3393
**3-dehydroshikimate**	0.797_154.	POS	0.797	154.0268
**Isoflupredone acetate**	4.762_420.	POS	4.762	420.193
**Nateglinide**	5.872_317.	POS	5.872	317.1989
Dermabacter_hominis				
Amnibacterium_kyonggiense				
Luteococcus_sediminum				
Other				
Craurococcus_roseus				
Brumimicrobium_glaciale				
Other				
**1-(3-Fluoro-4-hydroxy-5-mercaptomethyltetrahy**	0.747_276.	POS	0.747	276.0569
**3-(1,3-benzodioxol-5-yl)-7-[(2S,3R,4S,5S,6R)-6-[[(**	5.493_606.	NEG	5.493	606.1615
**2,4-Diacetamido-2,4,6-trideoxy-D-mannopyranos**	3.11_246.1	POS	3.11	246.1214
**Caffeic acid 3-O-sulfate**	0.585_259.	POS	0.585	259.9982
**sufac#1**	12.892_33	POS	12.892	338.1756
**N-Hexadecyl-N,N-dimethyl-3-ammonio-1-propar**	7.651_391.	POS	7.651	391.3109
**2-Methylhexadecanoic acid**	10.381_27(	POS	10.381	270.2559
**20-Hydroxy-(5Z,8Z,11Z,14Z)-eicosatetraenoic acid**	8.806_320.	NEG	8.806	320.2352
**Vitexin-4''-o-glucoside**	4.637_610.	POS	4.637	610.1537
**(2,5-Dioxopyrrolidin-1-yl) 5-(2-oxo-1,3,3a,4,6,6a-l**	3.695_341.	POS	3.695	341.1045
**4-Methylcatechol 1-sulfate**	3.706_204.	NEG	3.706	204.0093
Clostridium_fimetarium				
**N-Nitrosoproline; 1-Nitroso-L-proline;**	0.718_144.	NEG	0.718	144.0536
**maesopsin**	4.513_305.	POS	4.513	305.0899
**N(4)-Octadecyl-1-arabinofuranosylcytosine**	9.596_495.	POS	9.596	495.3648
**L-Dopa**	5.518_197.	POS	5.518	197.0691
**ADRENALINE BITARTRATE**	3.846_333.	POS	3.846	333.1071
**5-Methylthioribose**	0.743_180.	POS	0.743	180.0459
**PC(18:1(9Z)/18:1(9Z))**	12.234_78	POS	12.234	785.5927
**Diacetolol**	7.529_308.	POS	7.529	308.1735
**4-Hydroxyindole**	6.713_133.	POS	6.713	133.0529
Bacteroides_eggerthii				

Perindoprilat	7.521_340.	POS	7.521	340.1996
L-Proline, 1-(2-methyl-3-(methylthio)-1-oxopropy	2.613_231.	POS	2.613	231.0931
Daunosamine	0.685_147.	NEG	0.685	147.0896
PS(O-16:0/18:2(9Z,12Z))	10.782_74	POS	10.782	745.526
1-[(2E,4E)-6,7-Dihydroxy-2,4-octadienoyl]prolyl-N	4.835_483.	POS	4.835	483.306
Imazaquin	4.377_311.	POS	4.377	311.127
Tebuconazole	2.552_307.	POS	2.552	307.1451
Histamine	0.593_111.	POS	0.593	111.0797
VIPROSTOL	8.332_392.	POS	8.332	392.2563
NCGC00347679-02!5-chloro-3-[(1E,3E)-3,5-dimetl	3.826_372.	POS	3.826	372.1103
2-Oxazolidinone, 3-[[(5-nitro-2-furanyl)methylene	0.803_225.	POS	0.803	225.038
Guanidinoproclavaminic acid; Guanidinoproclavami	1.304_244.	POS	1.304	244.1173
Cholesta-5,7,9(11)-trien-3beta-ol	9.853_382.	POS	9.853	382.3235
1,1,2-Trimethyl-1H-benzo[E]indole	3.259_209.	POS	3.259	209.1212
Thiabendazole	3.945_201.	POS	3.945	201.0364
3-[1-(3-Aminopropyl)-1H-indol-3-YL]-4-(1-methy	5.311_398.	NEG	5.311	398.1763
Malvidin	4.655_330.	POS	4.655	330.0715
Tetranor-PGF1alpha	9.667_300.	POS	9.667	300.1939
Inosine,2',3'-dideoxy-	4.075_236.	POS	4.075	236.0912
Candimine;	3.215_345.	POS	3.215	345.1215
1,7-Octadiene	10.297_11	POS	10.297	110.1097
Dietzia_lutea				
Erythromycylamine	5.32_734.4	POS	5.32	734.4927
3H-1,4-Benzodiazepin-2-amine, 7-chloro-5-pheny	3.221_285.	POS	3.221	285.0671
Frangulanine;	8.848_500.	POS	8.848	500.3353
(3R,5S)-7-[2-Cyclopropyl-4-(4-fluorophenyl)quino	5.89_421.1	POS	5.89	421.1673
(1S,3R,6R,7S,8S,9R,10S,13S,16S,17R)-8-tert-butyl-6	4.07_440.1	NEG	4.07	440.1302
(R)-2-Hydroxycaprylic acid	5.116_142.	POS	5.116	142.0996
PA(17:1(9Z)/14:0)	8.925_632.	POS	8.925	632.4401
Oxiconazole nitrate;	5.963_492.	POS	5.963	492.1404
Malonate	0.654_104.	NEG	0.654	104.011
Mimosine	3.943_215.	POS	3.943	215.0907
Gibberellin A7	2.928_330.	NEG	2.928	330.1427
2,5-Dimethyl-3(2H)-furanone	3.059_112.	POS	3.059	112.0527
(2S)-4-hydroxy-2-(2-hydroxypropan-2-yl)-7-metl	4.716_276.	NEG	4.716	276.0996
Tramadol N-Oxide	9.596_279.	POS	9.596	279.1836
Chlorothiazide	2.569_294.	NEG	2.569	294.9487
dTDP-4-oxo-2,3,6-trideoxy-D-glucose; dTDP-[(2R,	4.026_514.	NEG	4.026	514.0777
Clostridium_sporosphaeroides				
Phenylalanyl-phenylalanyl-arginine chloromethyl k	6.244_500.	POS	6.244	500.2278
Dibasic sodium phosphate; Disodium phosphate;	0.697_141.	POS	0.697	141.941
beta-Funaltrexamine	5.724_454.	POS	5.724	454.2108
VORTIOXETINE	4.683_298.	POS	4.683	298.1511
Mycobactins	8.183_591.	NEG	8.183	591.2541
1-Octen-3-ol-3-o-beta-D-xylopyranosyl(1->6)-be	4.378_422.	POS	4.378	422.2158
Cochinchinenin A	8.136_286.	POS	8.136	286.1205
Methyldienolone	7.447_286.	POS	7.447	286.1931
Leu-leu-tyr; LLY;	4.794_407.	POS	4.794	407.2419
NCGC00385530-02!(E)-5-(1,2,4a,5-tetramethyl-7-c	7.065_362.	POS	7.065	362.184
Dorsilurin D	5.905_506.	POS	5.905	506.2284
Semduramicin	9.317_872.	POS	9.317	872.5109
Rubiadin	7.56_271.0	POS	7.56	271.0845
Phenobarbital	6.382_232.	NEG	6.382	232.0846
Methyl 1-(3,4-dimethoxyphenyl)-3-(3-ethylpentyl)	12.932_52	POS	12.932	526.2575
Pirimicarb	8.551_238.	POS	8.551	238.1419
L-Threonine	0.732_119.	POS	0.732	119.0584
L-Anticapsin; Anticapsin;	3.626_199.	POS	3.626	199.0847
(1r,3r)-1-Aminocyclopentane-1,3-dicarboxylic acid	0.721_173.	NEG	0.721	173.0689

Acholeplasma_brassicae			
**LysoPC(22:1(13Z)/0:0)**	7.983_577.POS	7.983	577.4094
**S-Nitroso-L-cysteinylglycine**	3.382_207.POS	3.382	207.0322
**Caribenolide I;**	8.496_624.NEG	8.496	624.3524
**Proacacipetalin; 3-Butenenitrile, 2-(beta-D-glucop**	5.375_259.POS	5.375	259.1068
**Proxan;**	6.734_136.POS	6.734	136.0017
**3-Benzo[1,3]dioxol-5-yl-5-hydroxy-5-(4-methoxy**	5.272_506.POS	5.272	506.1587
**(+/-)-Ethylketocyclazocine**	4.94_299.1POS	4.94	299.1884
**Dixyrazine**	6.335_427.POS	6.335	427.2302
**6-Nitrochrysene**	5.971_273.POS	5.971	273.0782
**Prostaglandin D1**	9.524_371.POS	9.524	371.2672
**20-Oxo-leukotriene E4**	4.97_453.2POS	4.97	453.2187
**1-Azabicyclo[2.2.2]octan-2-yl 2-hydroxy-2,2-diph**	7.615_337.NEG	7.615	337.1668
**Ethanol, 2-(pentyl(5-(1-piperidinyl)(1,2,4)triazolo(1**	9.423_332.POS	9.423	332.2326
Actinomyces_johnsonii			
**Pantoprazole Sulfone**	6.002_399.POS	6.002	399.0701
**Leucomycin A7;**	5.703_757.POS	5.703	757.4255
**Ixoside**	3.834_388.NEG	3.834	388.1007
**3beta,7alpha-Dihydroxychol-5-en-24-oic Acid**	10.189_39POS	10.189	390.2771
**N-Phenylacetylglutamic acid**	6.645_265.POS	6.645	265.0951
**Maslinic acid**	9.953_489.POS	9.953	489.3824
**phosphatidylbutanol**	8.898_730.POS	8.898	730.5544
**Nifuradene; 1-[(5-Nitrofurfurylidene)amino]-2-imi**	3.974_224.POS	3.974	224.0542
**Isofenphos;**	1.24_345.1POS	1.24	345.117
**8-Azido-cyclic AMP**	6.53_370.CNEG	6.53	370.0523
**(**	0.74_128.CNEG	0.74	128.0474
**NCGC00381067-01!methyl 3,5-dimethoxy-2-[5-me**	6.685_442.POS	6.685	442.123
Bulleidia_extructa			
**N-(3-hydroxy-dodecanoyl)-homoserine lactone**	5.906_299.POS	5.906	299.2096
**Ectocarpen;**	7.284_148.POS	7.284	148.1255
Listeria_ivanovii			
**Propenylguaiacol**	9.849_164.POS	9.849	164.084
Marinifilum_fragile			
**Pyroglutamyl-glutamyl-proline amide**	8.652_354.POS	8.652	354.1555
Rothia_endophytica			
**(2S,4S)-Pinnatanine**	1.072_244.POS	1.072	244.1061
**Chembl4483049**	2.595_404.POS	2.595	404.1247
**(25R)-12beta-acetyl-17alpha-hydroxyspirost-4-en**	8.143_486.NEG	8.143	486.298
**Met-enkephalinamide**	4.952_572.POS	4.952	572.2419
**n-methylsphingosine**	8.567_327.POS	8.567	327.2773
**TG(18:0/18:2(9Z,12Z)/20:4(5Z,8Z,11Z,14Z))**	12.558_90POS	12.558	906.7656
**Semagacestat**	4.811_361.POS	4.811	361.2001
**D-Urobilin;**	8.204_588.POS	8.204	588.2954
**Apocynin**	3.928_166.POS	3.928	166.0631
**Ketazolam**	6.039_368.NEG	6.039	368.093
**1alpha,25-dihydroxy-2beta-(4-hydroxybutoxy)vita**	9.816_504.POS	9.816	504.3821
**Flumazenil acid**	7.21_275.CPOS	7.21	275.0712
**DG(16:1(9Z)/17:2(9Z,12Z)/0:0)[iso2]**	10.387_57POS	10.387	576.476
**1-Deoxy-1-morpholino-D-fructose**	8.173_249.POS	8.173	249.1222
**Dihydrodiethylstilbestrol**	7.733_270.POS	7.733	270.1618
**PKCbeta Inhibitor**	5.878_411.POS	5.878	411.1698
**3'-Hydroxyamobarbital**	4.353_242.POS	4.353	242.1267
**L-Glutamine**	3.316_146.NEG	3.316	146.0693
**D-(-)-Lyxose**	0.657_150.NEG	0.657	150.0527
**(5'-(but-3-en-1-yn-1-yl)-[2,2'-bithiophen]-5-yl)m**	0.789_246.NEG	0.789	246.0172
**Cer(d18:1/17:0)**	11.832_55POS	11.832	551.529
**1-[(5-Methoxy-2,3-dihydro-1H-indol-3-yl)methyl**	5.156_303.POS	5.156	303.2045
**cis-5-Tetradecenoylcarnitine**	8.106_369.POS	8.106	369.2875

**Stercobilin**	6.672_594.	NEG	6.672	594.3422
**SM(d18:1/24:1(15Z))**	10.458_81:	POS	10.458	812.6776
**5-{2-Fluoro-5-[3-(3-hydroxy-2-methoxycarbonyl-**	4.397_413.	POS	4.397	413.0897
**Glucocleomin; 2-Hydroxy-2-methylbutyl glucosino**	4.911_405.	POS	4.911	405.0749
**Tetraphenylcyclopentadienone**	4.616_384.	POS	4.616	384.1529
**Cholestanyl glucoside**	9.817_550.	POS	9.817	550.4242
**Clioquinol glucuronide**	6.633_480.	NEG	6.633	480.9405
**Fujikinetin 7-O-laminaribioside**	5.769_636.	POS	5.769	636.1675
**Spegatrine**	5.32_363.1	POS	5.32	363.1492
**(+-)-(4Ar*,10R*,10aS*)-8-Chloro-1,2,3,4,10,10a-he**	4.28_292.0	POS	4.28	292.0979
**Commodore**	5.908_449.	POS	5.908	449.1015
**Thalmidine; (+)-O-Methylthalicberine;**	6.87_622.3	NEG	6.87	622.3021
**Gemigliptin**	5.054_489.	POS	5.054	489.1429
**6-(4-Hydroxy-6-methoxy-7-methyl-3-oxo-1H-iso**	3.504_433.	NEG	3.504	433.21
**Sulfolithocholylglycine**	7.654_513.	NEG	7.654	513.2755
**Kaempferol 3-(2''-p-coumaryl-rhamnoside)-7-rha**	6.118_724.	NEG	6.118	724.2002
**Isatoribine**	4.971_316.	POS	4.971	316.049
**4-Glutathionyl cyclophosphamide**	4.443_565.	NEG	4.443	565.0929
**methyl 2,4-dihydroxy-6-[(3Z,5Z)-nona-3,5-dienyl]**	5.787_490.	POS	5.787	490.1591
**Methyl clofenapate**	5.874_304.	POS	5.874	304.0864
**BETAINE**	0.721_139.	POS	0.721	139.0611
**N-Deisopropyl-fluvastatin**	6.535_369.	POS	6.535	369.138
**Ferroxamine**	8.155_618.	POS	8.155	618.3052
**Epsilon-caprolactam**	3.483_113.	POS	3.483	113.0842
**Prorepensin**	10.511_54	POS	10.511	544.319
**6-Deoxypenciclovir**	3.304_237.	POS	3.304	237.1223
**Epoxygedunin**	5.97_515.2	POS	5.97	515.2526
**Atractyloside A;**	4.536_448.	POS	4.536	448.2328
**Bilirubin glucuronide**	8.655_760.	POS	8.655	760.2954
Other				
**Thiosulfate**	0.634_113.	NEG	0.634	113.9446
**PA(6:0/6:0)**	5.015_368.	POS	5.015	368.1583
**1,1'-(1,8-Dioxo-1,8-octanediyl)bis-2,5-pyrrolidine**	5.635_336.	POS	5.635	336.132
**XANTHOPTERIN**	5.711_179.	POS	5.711	179.0442
**1-(1-Naphthylmethyl)-1,2,3,4-tetrahydroisoquinoli**	1.597_305.	POS	1.597	305.1408
**Hydroxysteroid**	9.125_292.	POS	9.125	292.2402
**(5-Acetamido-3,4,6-trihydroxyoxan-2-yl)methyl di**	0.649_301.	NEG	0.649	301.056
**TG(12:0/22:3(10Z,13Z,16Z)/22:5(7Z,10Z,13Z,16Z,19**	12.179_90:	POS	12.179	902.7345
Cellulosilyticum_ruminicola				
**Butylate**	2.359_217.	POS	2.359	217.1509
**N-Acetyl-3-hydroxyprocainamide**	2.305_293.	POS	2.305	293.1738
**Malonylshisonin;**	4.972_843.	POS	4.972	843.1981
**N-Methylnicotinamide**	3.306_136.	NEG	3.306	136.0638
**Givinostat**	3.261_421.	POS	3.261	421.2011
**[(3aS,4S,5S,6E,10Z,11aR)-6-formyl-5-hydroxy-10-(**	6.916_379.	POS	6.916	379.163
**9alpha-Fluoro-11beta,16alpha,17alpha,21-tetrahy**	7.072_396.	POS	7.072	396.1937
**5-Hydroxy-2',4',7,8-Tetramethoxyflavone**	6.694_358.	POS	6.694	358.1049
**104-1;**	3.943_468.	POS	3.943	468.1398
**4-Aminobenzohydrazide**	1.068_151.	POS	1.068	151.0748
**PHODiA-PE**	7.54_623.3	POS	7.54	623.3408
**Morphinan-3,6-diol, 7,8-didehydro-4,5-epoxy-17-**	4.993_365.	POS	4.993	365.0933
**Isobutyramide**	0.74_87.06	POS	0.74	87.06862
**NCGC00381110-01!13-hydroxy-6-(1-hydroxyethyl**	8.861_521.	POS	8.861	521.3443
**Turkesterone 22-acetonide**	8.319_536.	POS	8.319	536.3357
**1,7-Dimethyluric acid**	2.462_196.	NEG	2.462	196.0596
**Spirolactone SC 14266**	7.482_340.	POS	7.482	340.2036
**Uracil**	3.781_112.	POS	3.781	112.0274
**Cer(d18:2/16:0)**	11.073_53	POS	11.073	535.4973

**4,7beta-Dimethyl-4-azacholestan-3-one**	10.798_41!	POS	10.798	415.3815
**Spermine NONOate**	9.232_262.	POS	9.232	262.2119
**NCGC00381435-01!13-dodecan-2-yl-6-(1-hydroxy**	9.881_523.	POS	9.881	523.3642
Undibacterium_oligocarboniphilum				
**3-Hydroxy-cis-5-tetradecenoylcarnitine**	8.442_385.	NEG	8.442	385.2832
**Avosentan**	6.687_479.	NEG	6.687	479.1251
**THTC; 2-n-Tetrahydrothiophenecarboxylic acid;**	0.829_132.	POS	0.829	132.0248
Other				
Other				
**dTDP-3-methyl-4-oxo-2,6-dideoxy-L-allose;**	4.604_544.	NEG	4.604	544.0881
**Stilbene**	8_180.094(	POS	8	180.0941
**Amoxicillin**	5.999_365.	POS	5.999	365.1042
**Rutin**	4.831_610.	NEG	4.831	610.1532
**PIM1(17:0/14:0)**	7.425_958.	NEG	7.425	958.5629
**Adibendan**	6.585_278.	POS	6.585	278.1154
**(2R,3S,4R,5R,6S)-2-[[(1R,2R,4R,5R,6S,10R)-5-Hydro**	5.905_362.	POS	5.905	362.1223
**hydroxymorphinan**	3.602_243.	POS	3.602	243.1622
**8,9-Dihydroxy-1,5,6,10B-tetrahydropyrrolo[2,1-A]i**	5.257_219.	NEG	5.257	219.0895
**7-oxo-11E-Tetradecenoic acid**	8.17_240.1	NEG	8.17	240.1725
**[(8S,9S,13S,14S,17R)-3,17-Dihydroxy-13-methyl-6,**	3.274_392.	POS	3.274	392.1982
**Xestosteryl 18-bromooctadeca-9E,17E-diene-5,7,1**	9.28_754.4	POS	9.28	754.4342
**Anhydrobrazilic Acid**	4.207_251.	POS	4.207	251.0794
**1-(2-methoxy-5Z-hexadecenyl)-sn-glycero-3-pho**	5.061_511.	POS	5.061	511.29
**Phenylalanyl-prolyl-arginine-chloromethyl ketone**	6.869_450.	POS	6.869	450.2126
**Ioxilan**	3.312_790.	POS	3.312	790.8698
**N-Acetylsphingosine**	10.099_34.	NEG	10.099	341.2929
Planifilum_composti				
**5-[1,2,4a-trimethyl-5-(3-methylbutanoyloxymethy**	8.592_423.	POS	8.592	423.3351
**5'-Hydroxystreptomycin;**	6.92_597.2	POS	6.92	597.2588
**Lamivudine sulfoxide**	2.427_245.	POS	2.427	245.0468
**PC(14:0/18:3(6Z,9Z,12Z))**	10.781_72	POS	10.781	727.5154
**LMST04030226**	8.391_478.	POS	8.391	478.2761
**(2S,3R,4S,5S,6R)-2-[4-[[(3S,4R,5S)-3-hydroxy-5-(4**	4.324_538.	NEG	4.324	538.2044
**Oxethazaine**	7.974_467.	NEG	7.974	467.3154
**Avasimibe**	7.789_501.	POS	7.789	501.2931
**7-Hydroxytrifluoperazine**	12.569_42.	POS	12.569	423.1573
Brevibacterium_senegalense				
**Diisopropylethylamine**	3.716_129.	POS	3.716	129.1519
**2-n-Propylthiazolidine-4-carboxylic acid**	3.243_175.	POS	3.243	175.0669
**(2R)-1-[4-[1,3-Benzothiazol-2-yl(methyl)amino]pi**	5.153_433.	POS	5.153	433.165
**(2R)-2-Acetamido-6-hydroxy-2-(sulfanylmethyl)h**	4.392_233.	POS	4.392	233.0723
**norsertraline**	0.642_291.	NEG	0.642	291.0587
**Cholestan-3-one**	10.385_38(	POS	10.385	386.3548
**(-)-Caryophyllene oxide**	6.961_220.	POS	6.961	220.183
Butyrivibrio_crossotus				
**Prostaglandin F2a**	8.237_336.	POS	8.237	336.2298
Alishewanella_agri				
**Soyasapogenol B 3-O-b-D-glucuronide**	9.256_634.	NEG	9.256	634.4092
**(1S,4S,5R,10S,13S,17S,19S,20R)-10-hydroxy-4,5,9,9**	8.843_454.	POS	8.843	454.3449
**Cloprednol**	7.766_392.	POS	7.766	392.1406
Clostridium_ventriculi				
**Dehydromonocrotaline**	4.147_323.	NEG	4.147	323.1369
**4-Biphenylcarboxylic acid**	10.179_19	POS	10.179	198.0683
**2-hydroxy-nonadecanoic acid**	9.996_314.	POS	9.996	314.2819
**NCGC00336197-02!(1E,4E)-1,5-bis(4-methoxypher**	4.813_316.	POS	4.813	316.1094
**Montirelin**	4.654_408.	POS	4.654	408.1562
**Stemobenoid B**	5.714_542.	POS	5.714	542.2673
**NCGC00381139-01!6-benzyl-3-butan-2-yl-9-(7-h**	5.927_592.	POS	5.927	592.3255

**Nitrendipine**	6.511_360.	POS	6.511	360.132
**D-a-Tocopherol**	11.298_43(	POS	11.298	430.3816
**Murolic acid**	9.067_402.	NEG	9.067	402.2984
Other				
Other				
**I-Bop**	5.05_512.1	POS	5.05	512.1041
**1-(5-Fluoropentyl)-1H-indole-3-carboxylic acid 8-**	3.497_376.	NEG	3.497	376.1597
**Sebacic acid**	6.43_202.1	POS	6.43	202.1207
**PKHdiA-PS**	7.185_651.	POS	7.185	651.3018
**4-hydroxytriazolam**	0.719_358.	NEG	0.719	358.0386
**(1R,7R)-7-ethenyl-1,4a,7-trimethyl-3,4,4b,5,6,9,10,**	9.632_319.	POS	9.632	319.2511
**Montalicin A**	10.583_55.	NEG	10.583	552.4382
**Bulaquine**	7.852_369.	NEG	7.852	369.2052
**Diclofenac**	8.171_295.	POS	8.171	295.0164
**Fluorofelbamate;**	4.377_256.	POS	4.377	256.0849
**5-(Hexahydro-2-oxo-1H-thieno[3,4-D]imidazol-6-**	4.162_228.	POS	4.162	228.0935
**a-Aspartylphenylalanine**	3.501_280.	POS	3.501	280.1057
**Sapienic acid**	8.617_254.	POS	8.617	254.2247
**gamma-Secretase Inhibitor XXI**	7.106_490.	POS	7.106	490.1819
**Dioxidosqualene**	9.523_442.	POS	9.523	442.3813
**Hydralazine acetone hydrazone**	4.5_200.1C	POS	4.5	200.1053
**Cucurbitacin P**	8.264_520.	POS	8.264	520.3409
**Cer(d16:2(4E,6E)/22:0)**	12.315_59.	POS	12.315	591.5596
**5-chloro-2,4-dihydroxy-6-methyl-3-[(E)-3-methyl**	4.423_406.	POS	4.423	406.1814
**Sutezolid**	2.624_353.	POS	2.624	353.1223
**Irilone**	8.815_298.	NEG	8.815	298.0475
**2E,4E,6Z-Nonatrienal**	5.342_136.	POS	5.342	136.0891
**Casimiroin;**	4.258_233.	POS	4.258	233.0691
**Benzoic acid,2-[[[(4S)-4-carboxy-4-[[4-[[(2,4-diam**	12.717_57.	POS	12.717	573.207
**4-(3-Pyridin-2-yl-[1,2,4]oxadiazol-5-yl)-butyric ac**	4.692_233.	NEG	4.692	233.08
**(9a-hydroxy-3,8a-dimethyl-5-methylidene-2-oxo-**	9.234_306.	POS	9.234	306.1471
**4-(4-chlorophenyl)-4-hydroxypiperidine**	5.051_211.	POS	5.051	211.0757
**Henatinib**	6.903_468.	NEG	6.903	468.218
**Glycochenodeoxycholate-3-sulfate**	6.772_529.	NEG	6.772	529.2707
**steviolbioside**	6.857_642.	POS	6.857	642.3268
**4-Hydroxytamoxifen**	4.972_387.	POS	4.972	387.2256
**Zileuton**	5.043_236.	POS	5.043	236.0621
**Zolantidine**	3.593_381.	POS	3.593	381.1893
**Eleclazine**	3.316_415.	POS	3.316	415.1159
**obscurolide A1**	3.545_291.	NEG	3.545	291.1106
**Luteolin 7-(6'''-acetylallosyl-(1->2)-glucoside)**	4.357_652.	NEG	4.357	652.1633
**Isosakuranetin 7-alpha-L-arabinofuranosyl-(1->6)**	6.704_580.	POS	6.704	580.1792
**PS(O-18:0/0:0)**	9.246_511.	POS	9.246	511.3279
**PG(O-16:0/19:1(9Z))**	8.899_748.	POS	8.899	748.5634
**PI(22:1(11Z)/22:6(4Z,7Z,10Z,13Z,16Z,19Z))**	8.109_964.	POS	8.109	964.6057
**NA**	0.655_194.	NEG	0.655	194.0426
**Phorbol myristate**	8.859_574.	POS	8.859	574.3876
**Manassantin A**	6.723_732.	NEG	6.723	732.3521
**Trifluridine**	0.739_296.	POS	0.739	296.0611
**Dictyostelium**	6.984_306.	POS	6.984	306.0416
**3-(2-Ethyl-5-oxohexyl)phthalic acid**	6.372_292.	POS	6.372	292.131
**Cholylhistidine**	9.523_545.	POS	9.523	545.349
**Azilsartan**	5.27_456.1	NEG	5.27	456.1451
**(E,2S,3R,4R,5S)-4-acetyloxy-2-amino-3,5,14-trihyd**	8.439_431.	NEG	8.439	431.2884
**LMPK12130012**	13.054_53.	POS	13.054	532.1193
**L-Asparagine**	0.669_132.	NEG	0.669	132.0536
**Levonordefrin**	4.314_183.	POS	4.314	183.0898
**Delphinidin 3-O-3'',6''-O-dimalonylglucoside;**	0.737_637.	NEG	0.737	637.1055

**Pasakbumin-A**	5.962_408.	POS	5.962	408.1421
Desulfovibrio_piger				
**Malabaricano**	5.263_382.	POS	5.263	382.1199
**L-Phenylalanine**	2.624_165.	POS	2.624	165.079
**(3S,5R,8S)-2-[(benzoyloxy)methyl]-8-methyl-3-{[(**	6.76_561.1	POS	6.76	561.1531
**16a-Hydroxyestrone**	3.364_286.	NEG	3.364	286.1527
**Indoline**	2.65_119.C	POS	2.65	119.0737
**NCGC00384489-01!2-phenylfuro[2,3-h]chromen-4**	3.367_262.	POS	3.367	262.0626
**ascr#4**	2.835_408.	POS	2.835	408.201
**Dihydropertusaric**	6.975_385.	POS	6.975	385.2827
**Taurolithocholic acid 3-sulfate**	7.568_563.	NEG	7.568	563.2595
**1-(2-methoxy-13-methyl-pentadecanyl)-sn-glycer**	9.452_513.	NEG	9.452	513.3064
**1,2-Distearoylphosphatidylethanolamine**	12.359_74	POS	12.359	747.5773
**Methyl (5E,17E)-18-bromo-octadeca-5,17-diene-1**	6.527_368.	POS	6.527	368.1348
**2-Hydroxyvaleric acid**	0.996_118.	NEG	0.996	118.063
**Deoxyribose triphosphate;**	0.676_373.	POS	0.676	373.9572
**Premycofactocinol**	3.467_235.	POS	3.467	235.1209
**Alilusem**	5.895_394.	POS	5.895	394.0386
**Milbemycin beta1**	8.322_544.	POS	8.322	544.3395
**purvalanol A**	5.819_388.	POS	5.819	388.1795
**BEFLOXATONE**	0.63_349.1	NEG	0.63	349.112
**Tridesilon**	5.711_416.	POS	5.711	416.22
**Arachidic acid**	9.865_312.	NEG	9.865	312.3028
**PE(20:3(8Z,11Z,14Z)/0:0)**	8.939_503.	POS	8.939	503.3017
**D-Glutamine**	0.673_146.	NEG	0.673	146.0692
**7-Isopropyl-1,4-dimethylazulene**	8.05_198.1	POS	8.05	198.1411
**NCGC00380239-01!16-hydroxy-4,4,8,10,14-pentan**	8.903_436.	POS	8.903	436.3344
**p,p'-Methoxychlor olefin; 1,1-Dichloro-2,2-bis(p-n**	3.258_308.	POS	3.258	308.0381
**13-methoxy-1,6-diazatetracyclo[7.6.1.05,**	5.104_250.	POS	5.104	250.0755
**Succinic anhydride; Dihydro-2,5-furandione;**	1.266_100.	POS	1.266	100.0162
**HC Blue No. 2; 2,2'-[[4-[(2-Hydroxyethyl)amino]-3**	3.477_285.	POS	3.477	285.1323
**Orientin 7-O-sulfate**	4.574_528.	NEG	4.574	528.0569
Kocuria_palustris				
**NCGC00384531-01!3-(2,4-dihydroxyphenyl)-7-hy**	6.972_430.	POS	6.972	430.1741
Other				
**1,2,3-Trimethylbenzene; Hemimellitene;**	8.487_120.	POS	8.487	120.094
**Alisol A 24-acetate**	8.18_532.3	POS	8.18	532.377
**5Z,7Z-Dodecadienyl acetate**	10.136_22	POS	10.136	224.1777
**Pentostatin**	3.162_134.	POS	3.162	134.0581
**N-Desmethylaminopyrine**	5.151_217.	POS	5.151	217.1226
**1-[(5-Chloro-1H-indol-2-yl)carbonyl]-4-methylpi**	2.718_277.	NEG	2.718	277.0982
**Landiolol**	4.955_509.	POS	4.955	509.2755
**LacCer(d18:1/22:0)**	8.756_945.	POS	8.756	945.7129
**(1R,5aS,6R)-4a-Hydroxy-1,4,4a,5,5a,6,9,10a-octahy**	3.739_292.	POS	3.739	292.1057
**(2-Iodo-5-nitrophenyl)(1-((1-methylpiperidin-2-yl**	4.992_503.	POS	4.992	503.0687
**Oleanilide**	10.162_35	POS	10.162	357.3034
**Polyoxin B;**	7.896_507.	POS	7.896	507.1432
**PG(21:0/18:2(9Z,12Z))**	8.813_816.	POS	8.813	816.59
**Polyribosylribitolphosphate**	3.236_232.	POS	3.236	232.0341
**2-Naphthalenecarboxamide, 3-hydroxy-4-[(4-met**	1.145_471.	POS	1.145	471.1165
**2,5-Diamino-6-(5-phospho-D-ribitylamino)pyrimi**	3.398_355.	POS	3.398	355.0905
**10S,11R-epoxy-punaglandin 4**	7.867_514.	POS	7.867	514.1957
**Fenbufen**	8.135_254.	POS	8.135	254.0944
**N-(7Z,10Z,13Z-16Z-docosatetraenoyl) dopamine**	9.584_467.	POS	9.584	467.338
**Acenaphthene**	8.061_154.	POS	8.061	154.0784
**Rigosertib**	5.394_451.	NEG	5.394	451.13
**3,5-Dichloro-L-tyrosine**	3.213_248.	POS	3.213	248.996
**S-(2-Methylbutanoyl)-dihydrolipoamide;**	5.706_291.	POS	5.706	291.1318

**Ecgonine**	4.981_185.POS	4.981	185.1056
**dihydrocytochalasin B**	9.718_481.NEG	9.718	481.2804
**floctafenine**	4.523_406.NEG	4.523	406.1161
**Arphamenine B**	4.82_336.1POS	4.82	336.1795
**Aminoadipic acid**	0.727_161.NEG	0.727	161.0689
**Neocucurbitacin E**	8.385_546.NEG	8.385	546.3212
**Pivmecillinam**	6.968_439.NEG	6.968	439.214
**Amdoxovir**	5.095_252.POS	5.095	252.0976
**N,N-(2,2-dihydroxy-ethyl) arachidonoyl amine**	7.64_391.3POS	7.64	391.3086
**1-Dodecanesulfonic acid**	8.754_250.NEG	8.754	250.1601
**Metolachlor ESA**	5.707_347.POS	5.707	347.1402
**Patellamide B;**	4.781_776.POS	4.781	776.3105
**O-7460**	6.907_500.POS	6.907	500.3097
**NCGC00347521-02!3,6,8-trihydroxy-3-methyl-2,4-**	1.084_338.POS	1.084	338.0783
Other			
**Rilmakalim**	4.378_401.POS	4.378	401.1308
**3-Phenyl-2-thioxoimidazolidin-4-one**	4.531_192.POS	4.531	192.0362
**1-(2-methoxy-nonadecanyl)-sn-glycero-3-phosph**	10.115_55NEG	10.115	555.3544
**PS(O-16:0/20:2(11Z,14Z))**	10.332_77POS	10.332	773.557
**9-Carboxymethoxymethylguanine**	2.827_239.POS	2.827	239.0653
**N-Arachidonoyl-L-serine**	8.063_391.POS	8.063	391.2721
**Fosamprenavir**	4.886_585.POS	4.886	585.1886
**methyl 2-(2,6-dihydroxy-4-methylbenzoyl)-3,5-dil**	4.038_335.POS	4.038	335.1004
**LMPK12120227**	4.003_386.POS	4.003	386.1148
**2,4,5-Triphenylimidazole**	2.4_296.13POS	2.4	296.1304
**3,4-Dihydroxyphenylglycol O-sulfate**	3.545_250.NEG	3.545	250.0145
**Butanoic acid, 3-((2-((3R)-2-oxo-3-(2-(4-piperidin**	8.608_353.POS	8.608	353.2332
**PG(18:0/0:0)**	7.461_512.NEG	7.461	512.3094
**N-oleoyl histidine**	8.908_419.POS	8.908	419.3147
**2,3',4,6-Tetrahydroxybenzophenone**	4.541_263.POS	4.541	263.0794
**Naphthoxylactic acid**	6.715_232.POS	6.715	232.0736
**8-Epiiridodial glucoside tetraacetate;**	1.594_498.POS	1.594	498.2086
**virginiamycin m1**	7.952_525.POS	7.952	525.2483
**Mosapride**	7.098_421.NEG	7.098	421.1559
**aspernigrin A**	4.409_245.POS	4.409	245.1167
**(1R,7S)-4-[4-(4-Pyrimidin-2-ylpiperazin-1-yl)butyl**	5.959_383.POS	5.959	383.2308
**Pantoprazole**	5.869_383.POS	5.869	383.0755
**N-octanoyl histidine**	5.717_281.POS	5.717	281.1738
**Lauric isopropanolamide**	10.178_25POS	10.178	257.2355
**(2S)-3-Sulfolactaldehyde;**	0.637_153.NEG	0.637	153.9937
**Styraxlignolide A**	5.768_636.POS	5.768	636.2434
**3-Benzyl-1-(1H-imidazol-4-ylmethyl)-4-(thien-2-**	3.071_489.POS	3.071	489.1316
**LMPK12020082**	5.074_436.NEG	5.074	436.1171
Other			
**Mycobilin a;**	5.747_610.POS	5.747	610.2422
**Isoimperatorin**	6.716_287.POS	6.716	287.1156
**2-[(3S,5R)-5-[6-(2,4-Dichlorophenyl)hexyl]-3-hydr**	0.648_388.NEG	0.648	388.0852
**Acetylblasticidin S;**	6.134_464.POS	6.134	464.2143
**9-(Formyloxymethyl)-9H-fluorene-2-sulfonic acid**	7.047_304.POS	7.047	304.0396
**Supidimide**	3.043_280.POS	3.043	280.0517
**E-10-Hydroxynortriptyline**	6.475_279.POS	6.475	279.1623
**Hexenal**	4.264_98.0POS	4.264	98.07316
**NCGC00380621-01!4-oxo-4-(3-oxodecan-2-ylami**	3.964_271.POS	3.964	271.1783
**Tetradecylglycidate**	8.654_284.POS	8.654	284.2351
**glas#22**	9.057_522.POS	9.057	522.3022
**Urolithin A-3-O-glucuronide**	4.871_404.POS	4.871	404.0745
Azospira_oryzae			
Sporosarcina_psychrophila			

Agromyces_marinus				
Other				
Chryseomicrobium_amylolyticum				
Bacillus_megaterium				
**Solacetal B**	7.966_446.	NEG	7.966	446.2669
**Glyceryl methylmethacrylate**	3.292_174.	POS	3.292	174.0896
**Icariin**	9.63_676.2	POS	9.63	676.2445
**PQQH2; Reduced pyrroloquinoline-quinone;**	5.903_332.	POS	5.903	332.027
**PS(19:1(9Z)/16:0)**	10.586_77!	NEG	10.586	775.5397
**N-Desmethylpromazine**	4.011_270.	POS	4.011	270.1191
**Estrone sulfate**	6.404_350.	NEG	6.404	350.1187
**N-(4-Fluorobenzyl)-3-oxo-1,1-diphenyltetrahydro**	6.923_445.	POS	6.923	445.1788
**Reduced riboflavin;**	5.85_378.1	POS	5.85	378.1528
**4-(1,3-Dihydroxy-4,4,5,5-tetramethyl-imidazolidin**	2.786_280.	POS	2.786	280.1423
**PC(18:2(9Z,12Z)/17:0)**	11.868_77.	POS	11.868	771.5773
**Pederin;**	8.444_503.	POS	8.444	503.3075
**Deltaline**	5.714_507.	POS	5.714	507.2815
**5-(3-Hydroxybenzoyl)-2-thiophenesulfonamide**	0.807_282.	POS	0.807	282.9964
**Docarpamine**	6.879_470.	POS	6.879	470.1731
**[1-(3,3-Dimethyloxiran-2-yl)-3-[(8S,10S,11S,14R)-**	8.928_514.	POS	8.928	514.3666
Deinococcus_yunweiensis				
**n-methyl-2-(4'-methylaminophenyl)-6-hydroxybe**	0.736_272.	NEG	0.736	272.0982
Phascolarctobacterium_faecium				
**Geranyl pyrophosphate ammonium*200 UG/VI AL**	3.689_314.	POS	3.689	314.0682
**PS(12:0/17:0)**	9.723_693.	POS	9.723	693.4583
Other				
**(1S,8S,9S,10S,13R)-6,9,10-trimethyl-2-oxo-4,14-di**	3.232_342.	POS	3.232	342.0884
**Propofol**	6.623_178.	NEG	6.623	178.1358
**3,4-Dihydroxybenzaldehyde**	4.544_138.	POS	4.544	138.0319
**D-Arabitol**	0.705_190.	POS	0.705	190.0246
**Methanone, 1,1'-(1,4-piperazinediyl)bis(1-(6-(1H-I**	7.363_528.	POS	7.363	528.2019
Subdoligranulum_variabile				
**Iridal**	8.683_458.	POS	8.683	458.3764
**Malaoxon**	0.662_314.	NEG	0.662	314.06
**Kanosamine**	2.315_179.	POS	2.315	179.081
**N-(3-oxododecanoyl) homoserine lactone**	7.26_297.1	POS	7.26	297.1938
**Penicisteroid F**	8.169_522.	POS	8.169	522.3563
Other				
**Clavamycin A;**	5.345_414.	POS	5.345	414.1402
**1-[6-(2-Carboxypyrrolidin-1-yl)-6-oxohexanoyl]py**	2.622_340.	POS	2.622	340.1635
**(2R,3R,4S,5R,6R)-2-[(E)-2-Amino-3-hydroxy-octad**	7.027_461.	POS	7.027	461.3355
**Urate-3-ribonucleoside; Uric acid ribonucleoside; U**	2.664_300.	POS	2.664	300.0705
**tyrosine-tyrosinate**	5.86_344.1	NEG	5.86	344.1373
**HHPAA sulfate**	3.33_247.C	NEG	3.33	247.015
**PC(14:0/16:0)**	11.352_70!	POS	11.352	705.5308
**Susalimod**	2.819_408.	POS	2.819	408.0774
**Methyprylon**	5.15_183.1	POS	5.15	183.1263
**(E)-N-(2-Amino-4-fluorophenyl)-3-(1-cinnamyl-1**	4.159_362.	POS	4.159	362.1553
**Dalfopristin**	9.047_690.	POS	9.047	690.3267
**Ethyl 4-(3,4-dimethoxyphenyl)-6,7-dimethoxy-2-(**	4.854_478.	NEG	4.854	478.1835
**Avenastenone;**	10.001_41(	POS	10.001	410.3549
**thiazoline**	4.383_87.C	POS	4.383	87.0144
**6-Hydroxyfluvastatin**	5.419_427.	NEG	5.419	427.1778
**Pyrimidodiazepine; 2-Amino-6-acetyl-3,7,8,9-tetra**	3.717_221.	POS	3.717	221.0914
**trimebutine**	6.968_387.	POS	6.968	387.2042
**Bulbocapnine**	4.368_325.	POS	4.368	325.1314
**Adenosine**	5.169_267.	NEG	5.169	267.0927
**Lurbinectedin**	5.897_784.	POS	5.897	784.2762

**(2S,3R,4R,5S,6S)-2-(Hydroxymethyl)-6-phenylmetl**	4.406_270.	NEG	4.406	270.1102
**Probucol**	8.692_516.	NEG	8.692	516.3118
**(3R,4R)-7,12-Dimethyl-3,4-dihydrobenzo(a)anthra**	3.103_290.	POS	3.103	290.13
**3-oxo-heneicosanoic acid**	10.11_340.	NEG	10.11	340.2978
**NCGC00180835-03!2-[5-[2-[2-[5-(2-hydroxybutyl)**	8.218_382.	POS	8.218	382.2355
**Formoterol**	8.193_344.	NEG	8.193	344.1737
**4-Carboxy-4-hydroxy-2-oxoadipate; 4-Hydroxy-4**	0.783_220.	POS	0.783	220.021
**DG(22:2(13Z,16Z)/14:1(9Z)/0:0)**	12.595_61	POS	12.595	618.5226
**2-Hydroxyestrone sulfate**	6.506_366.	NEG	6.506	366.1135
Opitutus_terrae				
**LMST01070001**	9.115_596.	NEG	9.115	596.393
**N-[(2S)-1-[[(E,3S)-1-(Benzenesulfonyl)-5-phenylp**	8.239_561.	NEG	8.239	561.2282
**Methylenecyclopropylglycine**	0.746_127.	POS	0.746	127.0635
**3-O-Methylfluorescein**	4.01_346.0	POS	4.01	346.0834
**1,6-Hexanediamine**	0.596_116.	POS	0.596	116.1316
**NCGC00384578-01!5-[5-(acetyloxymethyl)-1,2,4a-**	7.647_360.	POS	7.647	360.2301
**LY404039; (1R,4S,5S,6S)-4-Amino-2-thiabicyclo[3.1**	3.885_235.	POS	3.885	235.0158
**Emodic acid**	5.182_300.	NEG	5.182	300.027
**Triclosan glucuronide**	7.447_463.	NEG	7.447	463.9832
Pyramidobacter_piscolens				
**Fluocinolone acetonide 21-acetate**	8.888_494.	POS	8.888	494.2117
**Glutamyl pyruvate**	0.661_217.	NEG	0.661	217.0584
**Deoxyinosine**	2.398_252.	POS	2.398	252.0862
**gamma-L-Glutamyl-butirosin B;**	7.933_684.	POS	7.933	684.3149
**(2S)-2-[(2R,3S,7R,8R,8aS)-2,3,4'-trihydroxy-4,4,7,8**	9.244_518.	POS	9.244	518.3011
**O-Desmethylvenlafaxine glucuronide**	7.045_439.	NEG	7.045	439.2205
**DL-Alanyl-DL-leucine**	0.742_202.	POS	0.742	202.1322
**Chloric acid; Chlorate;**	0.668_83.9	NEG	0.668	83.96141
**Bonafousine;**	9.178_564.	NEG	9.178	564.3128
**Paeonolide;**	3.327_460.	NEG	3.327	460.1578
**N-Demethylansamitocin P-3; N-Demethyl-AP-3; P**	5.734_620.	POS	5.734	620.2488
**4,7-Bis(chlorosulfophenyl)-1,10-phenanthroline-2,**	0.731_647.	NEG	0.731	647.9478
Mesorhizobium_shonense				
**Acroptilin; Chlorohyssopifolin C;**	2.853_398.	POS	2.853	398.1149
**Becocalcidiol**	9.152_344.	POS	9.152	344.2715
**Nvp dpp 728 dihydrochloride**	4.663_298.	POS	4.663	298.1529
**Triangulyne G**	9.305_486.	POS	9.305	486.3478
**12-hydroxy-3Z-dodecenoic acid**	6.834_214.	POS	6.834	214.157
**indol-2-one**	6.714_131.	POS	6.714	131.0373
**N-(1,3-Benzodioxol-5-ylmethyl)-2,6-dichlorobenz**	7.007_323.	POS	7.007	323.0114
**Nitroquine**	6.237_362.	NEG	6.237	362.0463
**Amorphigenol O-glucoside**	3.204_590.	POS	3.204	590.201
**Petromylidene C**	7.866_498.	NEG	7.866	498.2641
**2,4,5-Trichloro-N-(5-methyl-1,2-oxazol-3-yl)benz**	0.638_339.	POS	0.638	339.9244
Luteibacter_anthropi				
**SANGUINARINE SULFATE**	6.497_446.	POS	6.497	446.0776
**Triamterene**	3.778_253.	POS	3.778	253.1062
**Margaroyl-EA**	8.618_313.	POS	8.618	313.2981
**5'-methylthioadenosine**	3.463_297.	POS	3.463	297.0892
**Methyl pentadecanoate**	10.025_25	POS	10.025	256.2403
**2',3'-Dideoxythymidine triphosphate**	0.714_465.	NEG	0.714	465.9921
**NCGC00169585-02!(2S,3S,4S)-3,4-dihydroxy-6-me**	3.866_279.	POS	3.866	279.147
**Urea-1,3-dicarboxylate;**	1.303_148.	POS	1.303	148.0115
**Hypercalin B;**	10.616_51	POS	10.616	518.3014
**1-Tetralone**	3.27_146.0	POS	3.27	146.0732
**Avermectin A1b aglycone;**	9.309_584.	POS	9.309	584.3353
**5-Acetylamino-6-amino-3-methyluracil**	0.787_198.	POS	0.787	198.0392
**Muronic acid**	8.877_366.	POS	8.877	366.2406

**Perfluoroheptane**	0.698_387.	NEG	0.698	387.976
**Tariquidar**	3.833_646.	POS	3.833	646.281
**6**	4.234_378.	POS	4.234	378.2041
**LMPK12050339**	6.808_458.	NEG	6.808	458.1577
**Hexosylsphingosine**	8.682_477.	POS	8.682	477.3307
**Inecalcitol**	8.171_400.	POS	8.171	400.2977
**Phenylethyl 2-Glucoside**	3.125_301.	POS	3.125	301.1524
**dehydroabietic acid**	8.194_317.	POS	8.194	317.2342
**3-Carbamyl-(3'-picolyl)-4-methoxy-1-benzamide**	4.359_285.	POS	4.359	285.1111
**Lupanine**	2.569_248.	POS	2.569	248.1888
**N-Acetylmuramic Acid**	3.678_331.	POS	3.678	331.0667
Other				
**N-palmitoyl threonine**	8.005_357.	POS	8.005	357.2877
**Acamprosate**	0.72_181.C	NEG	0.72	181.041
**Metyrosine**	6.62_195.C	POS	6.62	195.0897
**Benzylidenacetone**	4.172_146.	POS	4.172	146.0733
**(3R,7R,8R,8aS)-3,4'-dihydroxy-7'-(2-hydroxyethyl)**	7.174_467.	POS	7.174	467.206
**Dopamine**	3.066_153.	NEG	3.066	153.0791
**4'-Hydroxydiclofenac**	6.182_311.	NEG	6.182	311.0116
**Vernakalant**	7.12_349.2	POS	7.12	349.2245
**Tolfenpyrad;**	5.624_383.	POS	5.624	383.1401
**Nornefopam**	3.268_239.	POS	3.268	239.1318
**N-(2-(3,4-Dichlorophenyl)ethyl)-N-methyl-2-(dim**	6.593_274.	NEG	6.593	274.0996
Brevundimonas_vancanneytii				
**3,6,9,12-Tetraazatetradecanedioic acid, 3,6,9,12-tet**	8.22_494.1	POS	8.22	494.1838
**Tephrodin**	6.141_450.	NEG	6.141	450.1324
**6-O-myristoyl-6'-O-myristoyltrehalose**	9.172_762.	NEG	9.172	762.5137
**N-(3-Methyl-2-pyridyl)-3-phenylsuccinimide**	4.779_266.	POS	4.779	266.1058
Dietzia_cercidiphylli				
**PI(15:0/0:0)**	4.79_558.2	POS	4.79	558.2801
**(2R,3S,4R,5R)-5-(2,4-dioxopyrimidin-1-yl)-4-hydr**	2.987_422.	NEG	2.987	422.1325
**Dihydroresveratrol 3-sulfate**	4.811_310.	NEG	4.811	310.0511
**fluticasone**	6.684_444.	POS	6.684	444.1581
**Balofloxacin**	5.049_389.	POS	5.049	389.1752
**Pyridin-3-ylmethyl N,N-dimethylcarbamate**	2.766_180.	NEG	2.766	180.09
**Parecoxib**	7.011_370.	POS	7.011	370.0987
**D-(+)-Proline**	2.725_115.	POS	2.725	115.0635
**5'-xanthylate(2-)**	2.907_364.	POS	2.907	364.043
**Demethylphylloquinol;**	8.826_438.	POS	8.826	438.35
**Oryzalin**	3.186_346.	POS	3.186	346.0947
**(2,2-Dimethyl-1-nitrosopropyl)benzene**	6.756_177.	POS	6.756	177.1155
**GSH-prostaglandin A1; Glutathione-prostaglandin**	7.122_643.	POS	7.122	643.3108
Other				
Arthrobacter_arilaitensis				
Caulobacter_vibrioides				
**2-(2-(4-Phenoxy-2-propylphenoxy)ethyl)indole-5-**	7.084_429.	POS	7.084	429.1934
**Melagatran**	4.495_429.	POS	4.495	429.2364
Other				
**1,9-Heptadecadiene-4,6-diyne-3,8-diol, (3S,8S,9Z)**	10.085_26(	POS	10.085	260.1776
**p-Cresol glucuronide**	3.415_284.	NEG	3.415	284.0894
**Arachidonoylmorpholine**	8.466_373.	POS	8.466	373.2979
**PS(P-18:0/20:4(5Z,8Z,11Z,14Z))**	10.897_79	POS	10.897	795.5412
**cephalosporanic acid**	4.004_257.	NEG	4.004	257.0353
**3-Amino-4-methoxybenzanilide**	3.798_242.	POS	3.798	242.1055
**Tripeptide**	3.123_549.	POS	3.123	549.256
**N(6)-Monobutyryladenosine**	4.462_337.	POS	4.462	337.138
**24-methylene-cholesterol sulfate**	9.575_478.	POS	9.575	478.3094
**N-Benzylglucamine dithiocarbamate**	3.223_347.	POS	3.223	347.087

**CMP-2-keto-3-deoxy-D-glycero-D-galacto-nonoı**	4.773_573.	POS	4.773	573.1206
**Chloramphenicol sodium succinate;**	5.5_445.18	POS	5.5	445.1853
**PS(19:0/19:1(9Z))**	10.343_81	POS	10.343	817.5839
**Falaconitine;**	8.207_629.	POS	8.207	629.318
**14-(hydroxymethyl)-5,9-dimethyltetracyclo[11.2.1.**	10_292.24	POS	10	292.2401
**2,4-Thiazolidinedicarboxylic acid, 2-methyl-**	1.811_191.	POS	1.811	191.0255
**2,3-Dihydroxy-5-methylthio-4-pentenoic acid**	0.788_178.	NEG	0.788	178.0301
**2-hydroxyflutamide**	12.677_29	POS	12.677	292.0674
**7-(2-hydroxypropan-2-yl)-1,4a-dimethyl-2,3,4,9,1**	7.805_354.	POS	7.805	354.1579
**Fosfomycin**	0.681_138.	NEG	0.681	138.0083
**3-hydroxy-5,5,8a-trimethyl-3,4,4a,6,7,8-hexahydro**	9.042_238.	POS	9.042	238.1571
**Policapram**	4.045_111.	POS	4.045	111.0686
**RIOPROSTIL**	8.465_354.	NEG	8.465	354.2771
**3-Deoxycaryoptinol**	6.835_472.	POS	6.835	472.1848
**Diethyl 1,4-dihydro-2,6-dimethyl-3,5-pyridinedica**	3.327_253.	POS	3.327	253.1316
**Hodgkinsine;**	8.565_518.	NEG	8.565	518.3182
**Val-Met**	2.894_248.	NEG	2.894	248.1193
**PE(0:0/18:1(11Z))**	9.648_479.	POS	9.648	479.3016
**(1S,4S,5R,9S,10R,13R,14R)-14-hydroxy-5,9-dimeth**	8.156_437.	POS	8.156	437.3142
**Pro-Pro-Pro**	2.497_309.	POS	2.497	309.1687
**(E)-3-(4-acetyloxy-2,3-dihydroxy-2,5,5,8a-tetrame**	5.24_392.1	POS	5.24	392.1583
Tepidisphaera_mucosa				
Comamonas_nitrativorans				
Balneola_alkaliphila				
**PKOOA-PS**	6.541_649.	POS	6.541	649.3198
**N-Cyclopropyl-1-(3-((1-oxidopyridin-3-yl)ethynyl**	6.361_422.	NEG	6.361	422.1398
**Ergotamine**	8.171_581.	NEG	8.171	581.2658
**2beta-chloro-1alpha,25-dihydroxyvitamin D3**	8.792_450.	POS	8.792	450.2919
**N-palmitoyl tryptophan**	9.315_442.	POS	9.315	442.3201
**Kaempferol 3-glucuronide**	4.913_462.	NEG	4.913	462.08
**N-Acetyl-N-[(2S)-3-(1H-indol-3-yl)-2-[[2-(4-pipe**	8.468_573.	NEG	8.468	573.3341
**4-{[(3S)-3-{[(7-Methoxynaphthalen-2-yl)sulfonyl](**	4.681_472.	NEG	4.681	472.1246
**L-(-)-Methionine**	0.811_149.	POS	0.811	149.0513
**Ouabain**	6.264_584.	POS	6.264	584.2851
**N-palmitoyl phenylalanine**	10.034_40	POS	10.034	403.3087
**dTDP-4-oxo-alpha-D-xylose; dTDP-4-keto-alpha-**	4.347_532.	NEG	4.347	532.0516
**Bis(2-ethylhexyl) phthalate**	10.084_39	POS	10.084	390.2768
**N-(3-oxo-octanoyl)-homoserine thiolactone**	3.372_257.	POS	3.372	257.1084
**2-Amino-4-phosphonobutyric acid**	4.731_183.	NEG	4.731	183.0294
**4-hydroxy-3-[(E)-7-hydroxy-3,7-dimethyl-4-oxoo**	5.98_374.2	POS	5.98	374.2095
**Deoxyguanosine monophosphate**	1.485_347.	POS	1.485	347.0629
**Hydroxymethylphosphonate; P-(Hydroxymethyl)-p**	9.361_111.	POS	9.361	111.9927
**Val-pro-pro**	2.859_311.	NEG	2.859	311.1845
**Pelletierine Hydrochloride**	3.373_194.	POS	3.373	194.1183
**Ethylparaben sufate**	2.875_246.	POS	2.875	246.0201
**3-(4-Carboxybenzylidene)-6-hydroxycamphor**	8.195_300.	POS	8.195	300.1362
**Myxothiazol Z;**	6.713_502.	POS	6.713	502.1956
**Pazopanib**	6.883_437.	POS	6.883	437.1623
**Tyr-Gly-Gly-Phe-Gly-OH**	7.588_499.	POS	7.588	499.2048
**6-Acryloyl-2-dimethylaminonaphthalene**	3.264_225.	POS	3.264	225.1162
**4-[[2-(4-Cyanoanilino)pyrimidin-4-yl]amino]-3,5-**	7.403_340.	POS	7.403	340.1423
**3-(2,4-Dimethyl-5-(2-oxo-1,2-dihydroindol-3-ylid**	6.435_310.	POS	6.435	310.1319
**Glyphosate-isopropylammonium;**	1.132_228.	POS	1.132	228.1838
**1-Nitrosonaphthalene;**	4.87_157.0	POS	4.87	157.0529
**Phenisopham;**	7.622_342.	POS	7.622	342.1579
**Venlafaxine**	9.486_277.	POS	9.486	277.2042
Clostridium_lavalense				
**DG(14:0/18:3(9Z,12Z,15Z)/0:0)**	10.314_56	POS	10.314	562.4601

**6,7-Dimethoxy-2-(piperazin-1-yl)quinazolin-4-am**	3.237_289.	POS	3.237	289.1525
**Urolithin A**	4.865_228.	POS	4.865	228.0424
**(+/-)8-HEPE**	9.474_300.	POS	9.474	300.2089
Hungatella_hathewayi				
**6-(4-Methoxyphenyl)-2-methyl-7H-imidazo[1,2-a**	4.828_255.	POS	4.828	255.0996
**3-(2-hydroxyphenyl)propanoate**	5.353_148.	POS	5.353	148.0527
**3-Amino-L-tyrosine**	5.429_196.	NEG	5.429	196.0848
**Luteolin 3'-methyl ether 7-(6''-crotonylglucoside)**	6.934_530.	POS	6.934	530.1432
**(2R,3R,4S,5S,6R)-2-[3-hydroxy-2-(4-hydroxy-3-m**	5.783_360.	POS	5.783	360.142
**Cyclohexyl isocyanide; N-Cyclohexylisocyanide; Cy**	5.004_109.	POS	5.004	109.0895
**Ptilosaponoside B**	6.462_576.	POS	6.462	576.2595
**3,5-dihydroxy-2-(4-hydroxyphenyl)-6-(3-methylb**	3.785_518.	NEG	3.785	518.1784
**Isophorone**	6.431_138.	POS	6.431	138.1046
**O-Methylandrocymbine;**	5.977_385.	POS	5.977	385.1887
**5,9-dihydroxy-7-(hydroxymethyl)-5,7-dimethyl-4,**	8.552_282.	NEG	8.552	282.1465
**3,14-dihydro-15-keto-tetranor Prostaglandin E2**	8.816_280.	POS	8.816	280.1674
**Limonoate;**	6.568_506.	POS	6.568	506.2172
**Benzene, 1-[(4-butylphenyl)ethynyl]-4-propyl-**	1.748_276.	POS	1.748	276.1877
**7-Ethyl-3,5-dimethyl-2E,4E,6E,8E-decatetraene**	10.136_19	POS	10.136	190.1724
**PE(20:1(11Z)/18:3(6Z,9Z,12Z))**	11.194_76	POS	11.194	767.5463
**4-hydroxy-3-(3-methylbut-2-enyl)benzoic acid**	5.65_206.0	NEG	5.65	206.0942
**Hydrogenated MDI**	5.861_262.	POS	5.861	262.1681
**Lymecycline**	5.916_602.	POS	5.916	602.2596
Saccharofermentans_acetigenes				
Clostridium_bornimense				
Anaerofilum_agile				
**LL-dityrosine**	4.391_360.	POS	4.391	360.1321
**5-trans prostaglandin F2**	7.37_371.2	POS	7.37	371.2671
**Dolastatin 11;**	7.059_984.	POS	7.059	984.5908
**Lanosterol**	10.326_42	POS	10.326	426.3863
**(3As)-2,3,3a,4-tetrahydro-1H-pyrrolo[2,1-c][1,2,4]**	2.602_224.	POS	2.602	224.0625
**2(1H)-Pyrimidinone,5-[3-[(1S,2S,4R)-bicyclo[2.2.1]**	5.449_316.	POS	5.449	316.1785
**2-(2-Chlorophenyl)-4-(3-(dimethylamino)phenyl)-**	4.579_394.	POS	4.579	394.1197
**D-Ornithine**	0.591_131.	POS	0.591	131.9911
**(22R)-1alpha,22,25-trihydroxy-26,27-dimethyl-23,**	10.387_45	POS	10.387	456.3244
**Etofylline clofibrate**	6.881_420.	POS	6.881	420.1183
**LysoPC(22:5(7Z,10Z,13Z,16Z,19Z)/0:0)**	9.467_569.	POS	9.467	569.3485
**Eterobarb**	6.305_320.	POS	6.305	320.1371
**Stearoyl-oleoyl-phosphatidyl ethanolamine**	11.699_74	POS	11.699	745.5626
**[(5R,6S,7S,8aR)-8a-ethenyl-5-hydroxy-6-(3-metho**	6.496_430.	POS	6.496	430.1053
Clostridium_tarantellae				
**rac S 33138**	5.973_361.	POS	5.973	361.1781
**Traumatic acid**	6.108_228.	POS	6.108	228.1368
**3,7-dideoxy-D-threo-hepto-2,6-diulosonic acid**	0.783_190.	POS	0.783	190.0479
**NCGC00347752-02![2-(3'-ethenyl-3',5-dimethyl-2**	4.563_422.	POS	4.563	422.1326
**Bastimolide A**	12.191_78	POS	12.191	788.6027
**2-Phenylethynesulfonamide**	2.599_181.	POS	2.599	181.0202
**Monoisoamyl dimercaptosuccinic acid**	1.601_252.	POS	1.601	252.0497
**(2S,3S,9R,12R,13R,14S,15R,16R,17S)-3,12,13,15,16-**	5.727_413.	POS	5.727	413.2064
**4-O-Methylascochlorin**	8.134_418.	POS	8.134	418.1894
**Popop**	3.029_364.	POS	3.029	364.1207
**L-ethionine**	1.743_146.	POS	1.743	146.0403
**alpha-D-Ribose 1-methylphosphonate 5-triphosph**	0.675_467.	NEG	0.675	467.9366
**1,3,5[10]-Estratriene-2,3-17 beta-triol**	5.714_288.	POS	5.714	288.1726
**1-Pentanone, 3-hydroxy-1,5-diphenyl-**	8.849_292.	POS	8.849	292.0859
**N-[2-[1-(5-Tert-butyl-2-methoxyphenyl)-2-(dime**	9.549_414.	POS	9.549	414.2535
**LMPK12020254**	5.917_538.	POS	5.917	538.2007
Sphingomonas_paucimobilis				

**5-Amino-2-(4-amino-3-fluorophenyl)-6,8-difluor**	3.358_320.	POS	3.358	320.0781
**Equilin sulfate**	4.444_348.	POS	4.444	348.1034
**6-bromo-tetracosa-5E,9Z-dienoic acid**	4.971_442.	POS	4.971	442.2429
Curvibacter_lanceolatus				
Paludibacter_propionicigenes				
**Demethylalangiside;**	6.964_491.	POS	6.964	491.1795
Methanobrevibacter_thaueri				
**Morinidazole**	0.793_270.	POS	0.793	270.1329
**2-[4-[4-(4-Carbamimidoylphenyl)piperazin-1-yl]pi**	4.377_345.	POS	4.377	345.2151
**Loggerpeptin A**	7.771_992.	POS	7.771	992.5187
**Isothiazole**	6.014_84.9	NEG	6.014	84.99859
**2-Hydroxycinnamic acid**	0.8_164.04	POS	0.8	164.0475
**N-(4-Nitroanilino)-N-(2,2,4,4-tetramethyl-3H-thic**	6.979_401.	POS	6.979	401.1223
**[(3S,4S,5S,6R)-3,4,5-Trihydroxy-6-(hydroxymethyl)**	8.945_444.	POS	8.945	444.3074
**4-Nitroimidazole**	2.408_113.	NEG	2.408	113.0226
**5-Fluoroorotic acid**	9.922_174.	POS	9.922	174.0084
**Gibberellic acid**	5.173_363.	POS	5.173	363.1715
**2-[4-[(3R,3aR,6S,6aR)-3-(3,4-dimethoxyphenyl)-1,**	5.237_534.	NEG	5.237	534.2094
**2-(5-Fluoro-3,7,11-trimethyldodeca-2,6,10-trienyl**	8.767_376.	POS	8.767	376.1886
**(2-Methylphenyl) Dihydrogen Phosphate**	8.767_188.	POS	8.767	188.0241
**Cryptocyanin; Cryptocyanine iodide;**	10.082_48	POS	10.082	480.3819
**Ethamoxytriphetol**	3.256_419.	POS	3.256	419.2459
**Mycinamicin VIII;**	8.665_505.	POS	8.665	505.3408
**Diisopropylamine**	1.402_101.	POS	1.402	101.1206
**Gibberellin A4&A7**	5.282_352.	POS	5.282	352.1274
**Cimoxatone**	3.421_338.	POS	3.421	338.1257
Other				
**Nitro-prednisolone**	6.926_405.	POS	6.926	405.1787
**CYCLIC AMP**	2.416_329.	POS	2.416	329.0526
**2-(7-oxo-2,3-dihydrofuro[3,2-g]chromen-2-yl)pro**	10.389_34	POS	10.389	345.1583
**(E)-5-(2,3-dimethyl-4,5,6,7-tetrahydro-1H-tricyclo**	7.937_234.	NEG	7.937	234.1619
**(7R)-7-(4-Carboxybutanamido)cephalosporanate;**	2.743_386.	POS	2.743	386.0787
**LMPK12010044**	0.648_771.	NEG	0.648	771.2136
**SM(d18:1/20:0)**	12.789_75	POS	12.789	758.6299
**Proversilin E**	5.288_453.	POS	5.288	453.2134
**2,5-Diisothiocyanatobenzenesulfonate**	0.634_271.	POS	0.634	271.9371
**Clerodendrin A;**	7.04_606.2	POS	7.04	606.2696
**Viniferol D**	7.116_680.	POS	7.116	680.2076
**6-Oxo-2-hydroxy-7-(4'-chlorophenyl)-3,8,8,8-tetr**	0.733_415.	POS	0.733	415.8951
**1,2-Anthraquinone**	0.665_208.	NEG	0.665	208.0518
**Magnesium protoporphyrin; Magnesium protopor**	7.869_584.	POS	7.869	584.2272
**5-Methylmaleylacetate;**	0.788_172.	POS	0.788	172.0374
**Vitamin D3 sulfate**	11.429_46	POS	11.429	464.2964
**(1R,2R,4aS,6aS,6bR,9R,10R,11R,12aR)-1,10,11-trihy**	8.734_504.	NEG	8.734	504.3449
**Diphenol glucuronide**	3.22_286.0	POS	3.22	286.0702
**Jaceidin**	6.038_360.	NEG	6.038	360.0836
**Pantheric Acid C**	4.054_214.	NEG	4.054	214.1205
**Fucoxanthinol 3-palmitoleate**	10.358_85	POS	10.358	852.6258
**N-Desmethyladinazolam**	5.69_337.1	POS	5.69	337.1095
**Medifoxamine**	5.625_257.	NEG	5.625	257.1415
**9,11,12a-trihydroxy-2,3-dimethoxy-8-(3-methylbu**	5.026_445.	POS	5.026	445.1752
**Mmp inhibitor V**	6.612_416.	POS	6.612	416.1945
**Halistanol sulfonic acid H**	6.78_672.2	NEG	6.78	672.2312
**S-(2-Chloroethyl)glutathione;**	0.741_369.	POS	0.741	369.076
Bacillus_cereus				
Clostridium_ramosum				
**2',3'-Dideoxyuridine**	2.777_212.	POS	2.777	212.0799
**suloctidil**	9.748_337.	POS	9.748	337.2439

**Talampicillin**	6.202_481.POS	6.202	481.1325
**Phenylalanylleucine**	4.781_278.POS	4.781	278.1629
**Isopentenyl pyrophosphate**	3.027_246.NEG	3.027	246.0023
**Mebeverine Acid**	5.771_279.POS	5.771	279.1835
**Cannabidivarin**	7.069_286.POS	7.069	286.1933
**2-[1-[2-[(4-Carbamimidoylbenzoyl)amino]-3-(4-hy**	5.2_468.19NEG	5.2	468.1997
**Tos-Arg-CH2Cl;**	3.907_332.POS	3.907	332.1081
**Maculosin; Cyclo(L-Pro-L-Tyr);**	7.758_260.POS	7.758	260.1159
Other			
**(E)-N-[2-(3-Indolyl)ethyl]-3-(3,4-dihydroxyphenyl**	8.768_322.POS	8.768	322.1309
**Violacein**	4.257_343.NEG	4.257	343.0951
**Tamoxifen-ol**	7.531_344.POS	7.531	344.1764
**6-(2-Chlorophenyl)-2-[(4-methylpiperazin-1-yl)m**	4.677_464.POS	4.677	464.1368
**(4-{2-[(4-Carbamimidoyl-benzoyl)-methyl-amino]**	5.777_443.POS	5.777	443.135
**PE(17:1(9Z)/20:4(5Z,8Z,11Z,14Z))**	11.015_75 POS	11.015	751.5123
**2-Aminobenzothiazole**	2.851_150.POS	2.851	150.0254
**Benzofuran;**	4.316_118.NEG	4.316	118.0418
**Crocetin**	4.892_328.POS	4.892	328.1676
**Galantamine**	6.858_287.POS	6.858	287.1519
**2-Sulfamoyl-5-(5-(p-tolyl)-3-(trifluoromethyl)-1H**	7.545_425.POS	7.545	425.066
**20alpha-hydroxy cholesterol**	9.245_402.POS	9.245	402.3497
**Silandrin**	4.562_466.NEG	4.562	466.1276
**22-Hydoxy-2-hopen-1-one**	8.724_440.POS	8.724	440.3657
**Ferulate**	5.405_176.POS	5.405	176.0476
**1-Benzothiepin**	4.02_160.CPOS	4.02	160.0342
**Carboxyethyl-hydroxychroman**	4.894_222.NEG	4.894	222.0891
**Brucine N-oxide**	7.05_410.1POS	7.05	410.1842
**[(4R,5S,6R,6aS,7R,10aR,11bR)-5-acetyloxy-6-hydro**	10.615_48 POS	10.615	481.2668
**NCGC00385172-01!7-methoxy-8-(3-prop-1-en-2-**	3.762_275.POS	3.762	275.1156
**(3alpha,5beta,7alpha)-23-Carboxy-7-hydroxy-24-r**	7.782_568.NEG	7.782	568.3252
**3,5,5-Trimethyl-4-[3,7,12,16-tetramethyl-18-(2,2,6**	9.693_596.POS	9.693	596.3865
**Polygodial**	8.192_234.POS	8.192	234.1621
**5-(3-Methyl-1-triazeno)imidazole-4-carboxamide**	2.937_168.POS	2.937	168.0765
**Palmitoylcarnitine**	8.742_399.POS	8.742	399.3349
**Terpendole K;**	5.984_517.POS	5.984	517.2832
**2-methylidene-4-[(2R,3R,4S,5S,6R)-3,4,5-trihydrox**	3.86_278.1NEG	3.86	278.1012
Pedobacter_nyackensis			
Pedobacter_jejuensis			
**C-6 NBD ceramide**	7.464_597.POS	7.464	597.3452
**Intoplicine**	7.724_348.POS	7.724	348.1936
**4,10-undecadiynal**	10.373_16 POS	10.373	162.1047
**(5Z,7E)-9,10-Seco-5,7,10(19)-cholestatriene**	11.565_36 POS	11.565	368.3442
**Cyclooctane**	10.084_11 POS	10.084	112.1253
**Lexipafant**	6.641_458.POS	6.641	458.1973
**C20819; Carbapenem biosynthesis intermediate 5;**	4.82_258.1POS	4.82	258.1038
**4-Hydroxy-3-(3-methyl-2-butenyl)acetophenone**	8.861_204.POS	8.861	204.1153
**Denbufylline**	4.568_320.POS	4.568	320.1846
**Rotigaptide**	4.783_617.POS	4.783	617.2805
**Cys-Arg-Glu-Lys-Ala**	5.861_605.POS	5.861	605.2939
Bacteroides_dorei			
**Aethusin; 2,8,10-Tridecatriene-4,6-diyne;**	8.045_170.POS	8.045	170.1097
**(5E)-4-methoxy-5-[methoxy-[(2R,3S)-3-phenyloxi**	5.313_291.POS	5.313	291.1105
**[(2S)-2-Amino-3-(1H-imidazol-5-yl)propanoyl] (2**	0.745_268.POS	0.745	268.1535
Phyllobacterium_endophyticum			
**Lappaol C;**	5.978_554.POS	5.978	554.2128
**Leucyl-leucyl-norleucine;**	5.751_357.POS	5.751	357.2628
**2-amino-5-[2-[[2,3-dihydroxy-2-(1-hydroxyethyl)**	2.433_436.POS	2.433	436.1447
Other			

**[(2S,3S,5R)-3-Azido-5-(5-methyl-2,4-dioxopyrimic**	4.047_380.	POS	4.047	380.1802
**Piscerythramine**	5.721_451.	POS	5.721	451.2012
**(8R,9R,10R,13S,14S)-3-[2-(Diethylamino)ethoxy]-1**	10.082_38	POS	10.082	387.3139
**OHODA-PI**	7.545_808.	POS	7.545	808.4414
**OHODA-PE**	8.627_689.	POS	8.627	689.4265
**2-Amino-6-methylmercaptopurine**	3.147_181.	POS	3.147	181.0425
**3-O-p-Coumaroylquinic acid**	3.149_355.	POS	3.149	355.1265
**LMSL05000001**	6.669_376.	POS	6.669	376.2113
**DG(22:5(7Z,10Z,13Z,16Z,19Z)/16:0/0:0)**	12.316_64	POS	12.316	642.5227
**NVP-231**	3.975_431.	POS	3.975	431.1616
**3-Hydroxyvalproic acid**	5.034_160.	NEG	5.034	160.1101
**cyclopeptine**	4.689_297.	POS	4.689	297.1476
**Eriojaposide A;**	3.37_502.2	POS	3.37	502.2395
**Edetic Acid**	0.632_292.	NEG	0.632	292.0904
**Quinidine**	3.832_324.	POS	3.832	324.1797
**NCGC00380372-01!N-[1-(4-methoxy-6-oxopyran-**	3.425_291.	POS	3.425	291.0888
Megamonas_funiformis				
**EPITUMOMAB CITUXETAN**	5.911_686.	POS	5.911	686.256
**PA(14:1(9Z)/14:1(9Z))**	8.935_588.	POS	8.935	588.3813
**Tetrahydrocortisone**	6.75_364.2	POS	6.75	364.224
**Austrobuxusin I**	9.01_590.3	POS	9.01	590.3464
**S-(Indolylmethylthiohydroximoyl)-L-cysteine;**	5.771_293.	POS	5.771	293.0832
**Plafibride**	9.234_355.	POS	9.234	355.1314
**PI(22:6(4Z,7Z,10Z,13Z,16Z,19Z)/22:2(13Z,16Z))**	7.789_962.	POS	7.789	962.5908
**PC(18:1(9Z)/14:0)**	11.409_73	POS	11.409	731.5465
**Ethyl hydrogen sulfate**	0.663_125.	NEG	0.663	125.9988
**Muscone**	9.81_238.2	POS	9.81	238.2297
**2-Acetylacteoside**	6.98_666.2	POS	6.98	666.2147
**Clopidogrel active metabolite**	4.824_355.	POS	4.824	355.0645
**PA(P-16:0/15:1(9Z))**	9.957_616.	POS	9.957	616.4451
**Dodecanedioic acid**	3.679_230.	NEG	3.679	230.1517
**25-hydroxy-16,17,23,23,24,24-hexadehydrovitamir**	8.182_394.	POS	8.182	394.2875
**LysoPE(22:6(4Z,7Z,10Z,13Z,16Z,19Z)/0:0)**	9.366_525.	POS	9.366	525.2864
**Coenzyme B; N-(7-Mercaptoheptanoyl)threonine 3**	3.329_343.	POS	3.329	343.0838
**N-[2-(Dimethylamino)ethyl]-N-methyl-4-[({4-[4-r**	5.042_624.	POS	5.042	624.28
**1-Sulfooxymethylpyrene**	0.961_312.	POS	0.961	312.0449
**2'-Hydroxybiphenyl-2-sulfinate; 2-(2-Hydroxyphe**	0.777_234.	POS	0.777	234.0353
**Convallatoxin; Strophanthidin 3-O-alpha-L-rhamn**	7.514_550.	POS	7.514	550.2803
**N'-[(5-Formyl-3-hydroxy-2-methylpyridin-4-yl)m**	6.055_286.	POS	6.055	286.107
**3-{4-[2-Hydroxy-3-(propan-2-ylamino)propoxy]p**	6.876_281.	POS	6.876	281.1627
**Tyr-pro-leu-gly-NH2**	4.879_447.	POS	4.879	447.2471
**Schiff base**	5.736_384.	NEG	5.736	384.1898
**13-O-Tetradecanoylphorbol 12-acetate**	5.664_616.	NEG	5.664	616.3948
**2-Lysophosphatidylcholine**	10.389_52	POS	10.389	523.3648
Eubacterium_coprostanoligenes				
Treponema_berlinense				
**beta-Lumicolchicine**	6.978_399.	POS	6.978	399.1681
**Ungeremine;**	6.926_266.	POS	6.926	266.0805
**Carpachromene**	6.014_353.	POS	6.014	353.1261
Alistipes_finegoldii				
**11-beta-Hydroxyandrosterone-3-glucuronide**	3.162_482.	POS	3.162	482.2495
**6-(alpha-D-Glucosaminyl)-1D-myo-inositol 1,2-cy**	2.894_403.	POS	2.894	403.0872
**Monochlorobimane**	3.717_226.	NEG	3.717	226.0511
**PHHdiA-PI**	6.951_728.	NEG	6.951	728.3361
**NCGC00169692-02!1,7,8-trihydroxy-3-methyl-2,3,**	4.046_275.	POS	4.046	275.0826
Clostridium_methylpentosum				
**1-Hydroxymethylnaphthalene; 1-Naphthalenemetl**	8.044_158.	POS	8.044	158.0733
**Doxepin**	3.799_279.	POS	3.799	279.1631

LysoPC(20:2(11Z,14Z)/0:0)	9.721_547.	POS	9.721	547.3648
PG(22:1(11Z)/22:4(7Z,10Z,13Z,16Z))	7.61_880.6	POS	7.61	880.6169
Epiroprim	7.107_353.	POS	7.107	353.1837
5,6-dihydrouracil	0.95_131.C	POS	0.95	131.0697
2-Naphthalenecarboxamide, 3-hydroxy-N-(2-metl	2.78_383.1	POS	2.78	383.1151
4,5-Diaminofluorescein	2.845_362.	POS	2.845	362.0896
Dansylglutamyl-glycyl-arginine chloromethyl ketor	5.521_625.	POS	5.521	625.206
Menahydroquinone-4	9.56_446.3	POS	9.56	446.3186
Micronomicin	8.793_463.	POS	8.793	463.3017
Hydroxyhomo Sildenafil	4.447_504.	POS	4.447	504.2151
Cysteine thiol	0.657_152.	NEG	0.657	152.992
trans-4-Hydroxycyclohexylacetic acid	3.658_158.	NEG	3.658	158.0944
6-Hydroxymelatonin sulfate	4.34_342.C	POS	4.34	342.0515
PE(18:1(11Z)/0:0)	10.014_47!	NEG	10.014	479.3011
Cer(d18:0/16:0)	9.607_539.	POS	9.607	539.5286
Ciadox	2.614_271.	POS	2.614	271.0709
Esculeogenin A	8.568_447.	NEG	8.568	447.3349
2-Methylene-5-(2,5-Dioxotetrahydrofuran-3-Yl)-6	7.693_342.	POS	7.693	342.1217
Munetone	5.734_416.	POS	5.734	416.1616
Lyoniside	7.837_552.	POS	7.837	552.2197
5-Methoxysalicylic acid	2.22_146.C	POS	2.22	146.0582
Leucylproline	3.699_228.	POS	3.699	228.1474
L-Cysteinylglycine disulfide	0.669_297.	NEG	0.669	297.0451
Alpha-Ergocryptine	9.167_500.	POS	9.167	500.2905
Cimetidine	4.016_252.	NEG	4.016	252.1155
Benidipine	8.236_505.	POS	8.236	505.222
1,7-Dideacetoxy-1,7-Dioxo-3-Deacetylkhivorin	7.978_478.	POS	7.978	478.1956
5-(Dihydroxymethyl)furan-2-carboxylate;	0.789_158.	POS	0.789	158.0217
Methyl isothiocyanate;	2.438_72.9	POS	2.438	72.99872
Pyrophosphate	0.653_177.	NEG	0.653	177.9433
Testosterone decanoate	9.551_442.	POS	9.551	442.3425
Quercetin 7-glucuronide 3-rhamnoside	4.883_624.	POS	4.883	624.1325
chelidonine	7.931_370.	POS	7.931	370.1528
15-Deoxy-<U+0394>12,14-prostaglandin A1	8.905_318.	POS	8.905	318.2169
Cordycepin	1.474_251.	POS	1.474	251.1021
Diphenylmethane	8.049_168.	POS	8.049	168.0941
Benzamide, N-(3R)-1-azabicyclo[2.2.2]oct-3-yl-4-(	4.204_264.	NEG	4.204	264.103
Chlorimuron-ethyl; Chlorimuron ethyl;	1.458_414.	POS	1.458	414.0406
Nor-9-carboxy-d9-THC	9.965_344.	POS	9.965	344.2027
PS(16:0/0:0)	9.674_497.	NEG	9.674	497.2754
Clopidogrel carboxylic acid	6.946_307.	POS	6.946	307.0431
Parabacteroides_distasonis				
Hidroxiestilbamidina	7.158_280.	POS	7.158	280.1311
bhas#32	8.569_446.	POS	8.569	446.3229
(2S)-2-[(2-Carboxy-2-oxoethyl)amino]pentanedioi	6.956_233.	POS	6.956	233.0544
Cypendazole;	7.215_329.	POS	7.215	329.1499
NCGC00347521-02!3,6,8-trihydroxy-3-methyl-2,4-	3.759_360.	POS	3.759	360.0623
Bifidobacterium_animalis				
1,3-(8R,9R-epoxy-octadec-13Z,15Z-dien-4,6-diyn(	8.484_872.	NEG	8.484	872.526
apicidin	8.428_623.	NEG	8.428	623.3685
NCGC00385827-01!7-[[(1S,4aS,6S,8aR)-6-hydroxy-	7.089_401.	POS	7.089	401.1869
2,6-Di-tert-butylpyridine	5.53_191.1	POS	5.53	191.1676
Benzene, p-di-tert-butoxy-	10.152_22.	POS	10.152	222.1622
Naproxen	5.362_230.	POS	5.362	230.0944
2,2,3,3-Tetramethylcyclopropanecarboxamide	1.267_141.	POS	1.267	141.1155
1-Palmitoyl-2-linoleoyl-3-acetyl-rac-glycerol	10.017_63.	NEG	10.017	634.5187
(6S,7R)-3-[(5-Methyl-1,3,4-thiadiazol-2-yl)sulfany	0.778_454.	POS	0.778	454.0314
Glycocholate	7.697_930.	NEG	7.697	930.6205

Isoliquiritigenin 4,4'-diglucoside	5.287_580.	NEG	5.287	580.1797
Benzotest	8.756_398.	POS	8.756	398.2823
NCGC00347745-02! [IIN-based: Match]	5.465_534.	POS	5.465	534.1906
N-Glycolylneuraminic acid	4.368_325.	NEG	4.368	325.0983
mescaline	3.252_211.	POS	3.252	211.121
Miglitol	0.694_207.	NEG	0.694	207.1106
Myristicin	7.523_192.	POS	7.523	192.0769
Dihydroxyeicosatetraenoic acid	8.924_336.	POS	8.924	336.2301
5-(4-Acetamidophenyl)pyrazin-2(1H)-one	4.622_229.	POS	4.622	229.0854
NCGC00381317-01!(E)-1-[2-(hydroxymethyl)pyrro	9.842_281.	POS	9.842	281.1992
N(6)-6(R,S)-lipoyl-L-lysine	2.34_334.1	NEG	2.34	334.1376
Phytolaccasaponin G	6.68_664.3	NEG	6.68	664.3833
Valproic acid glucuronide	6.259_320.	NEG	6.259	320.1472
4-Prenylresveratrol;	7.53_296.1	POS	7.53	296.1409
Epitalon	0.627_390.	NEG	0.627	390.1385
5,13,15-trihydroxy-9-methyl-10-oxabicyclo[10.4.0]	5.001_308.	NEG	5.001	308.1257
Dienestrol diacetate	0.741_350.	POS	0.741	350.1511
16-bromo-7E,11E,15E-hexadecatrien-5,13-diynoic	0.661_320.	NEG	0.661	320.0412
10-Hydroxy-octadec-12Z-enoate-9-beta-D-glucu	7.514_490.	NEG	7.514	490.2778
4-Methylcatechol	2.626_106.	POS	2.626	106.042
Deacetylcephalosporin C;	3.318_373.	POS	3.318	373.0944
1,2-Naphthoquinone	5.972_158.	POS	5.972	158.037
trans,trans-2,4-Heptadienal	5.036_110.	POS	5.036	110.0733
C18-Sphingosine 1-phosphate; D-erythro-Sphingo	8.92_379.2	POS	8.92	379.2489
L-Dihydroanticapsin;	4.497_201.	POS	4.497	201.1004
5-Aminopentanamide;	3.448_116.	POS	3.448	116.0951
Desoxycorticosterone pivalate	8.219_414.	POS	8.219	414.2772
Glucuronic acid-3,6-lactone	0.666_176.	NEG	0.666	176.0321
Oleylcarnitine	10.323_41	POS	10.323	411.3715
PI(21:0/0:0)	9.285_642.	POS	9.285	642.3774
NCGC00169275-02!5-chloro-3-[(E)-3,4-dihydroxy-	5.535_384.	POS	5.535	384.1356
(2R,2'S,4'aS,5'R,5''S,6'R,8'aS)-2'-(acetyloxy)-5''-(fu	5.182_460.	NEG	5.182	460.1724
Methylcellulose	8.254_658.	NEG	8.254	658.3398
Diallyl phthalate	6.871_246.	POS	6.871	246.0893
7-(3,4-dihydroxyphenyl)-1-(4-hydroxyphenyl)-5-[	7.112_479.	POS	7.112	479.2157
Echitamine;	7.525_385.	POS	7.525	385.2136
Octocrylene	6.981_361.	POS	6.981	361.2041
Papaverine	7.16_339.1	POS	7.16	339.15
2-(4-(Piperidin-3-yl)phenyl)-2H-indazole-7-carbo	6.881_320.	POS	6.881	320.1636
25C-NBOMe	3.896_335.	POS	3.896	335.1278
Piclozotan	7.109_409.	POS	7.109	409.1555
1,10a-dihydroxy-4,4,7,11b-tetramethyl-1,2,3,4a,5,6	7.305_372.	POS	7.305	372.1683
5-(4-acetyloxy-3-hydroxy-2,5,5,8a-tetramethyl-3,4	8.245_380.	POS	8.245	380.2559
Solacetal A	8.212_476.	POS	8.212	476.2775
ascr#29	8.322_414.	POS	8.322	414.2969
Isocoumarin	7.535_146.	POS	7.535	146.037
5-Chloro-3-ethyl-N-[(1-ethylpyrrolidin-2-yl)meth	9.234_340.	POS	9.234	340.1561
C-13(2)-Carboxypyropheophorbide a; C-13(2)-Carl	4.906_578.	POS	4.906	578.2556
CE(20:5(5Z,8Z,11Z,14Z,17Z))	12.69_670.	POS	12.69	670.5673
Ac-DEVD-CHO	5.063_502.	POS	5.063	502.189
3'-Sialyl-N-acetyllactosamine	0.993_674.	POS	0.993	674.238
PS(20:0/0:0)	9.785_553.	NEG	9.785	553.3371
N-[4-[2-[Benzyl-[3-(3,4-dimethoxyphenyl)propan	7.168_573.	NEG	7.168	573.2608
Cer(d18:2/21:0)	12.742_60	POS	12.742	605.5756
N-Nitrosoguvacoline;	0.739_170.	POS	0.739	170.0694
Methyl 18-bromo-5,6-trans-endomethylene-7,11,1	2.879_422.	POS	2.879	422.1804
MIPC(t18:0/18:0(2OH))	9.377_100	POS	9.377	1003.616
Vinflunine	4.799_816.	POS	4.799	816.387

**3,17,20-Trihydroxypregnane**	9.58_336.2	POS	9.58	336.2664
**7alpha-Thiomethylspironolactone**	6.782_388.	POS	6.782	388.2072
**Monobutyl phthalate**	6.986_222.	POS	6.986	222.0893
**2-Methyl-6-phenyl-3,7-dihydroimidazo[1,2-a]pyra**	6.981_225.	POS	6.981	225.0904
**Nimodipine**	6.854_418.	POS	6.854	418.1741
**SB 228357;**	6.669_431.	NEG	6.669	431.1249
Other				
**Domoic acid**	5.269_311.	POS	5.269	311.1371
**Phosphonate analog**	3.375_315.	POS	3.375	315.0718
**4-Amino-4-deoxychorismate; ADC;**	0.654_225.	NEG	0.654	225.0636
**3-(2-Ethylhexyl)phthalic acid**	7.042_278.	POS	7.042	278.1517
**10-Hydroxy-2-oxabicyclo[6.2.2]dodeca-1(10),8,11-**	5.36_192.C	POS	5.36	192.079
**Nicardipine**	7.774_479.	POS	7.774	479.206
**PS(18:3(9Z,12Z,15Z)/18:3(9Z,12Z,15Z))**	7.004_779.	POS	7.004	779.4722
**3-[4-(1-Imidazolylmethyl)phenyl]-2-propenoic aci**	6.486_228.	POS	6.486	228.0899
**DG(15:0/18:2(9Z,12Z)/0:0)[iso2]**	9.384_578.	POS	9.384	578.4912
**gibberellin A2 O-beta-D-glucoside**	7.05_512.2	NEG	7.05	512.2255
**5-trans-Prostaglandin E2**	8.194_334.	POS	8.194	334.2142
**Rosuvastatin**	6.939_481.	POS	6.939	481.1691
**Cobyrinate c-monamide; Cobyrinic acid c-monoam**	5.643_937.	NEG	5.643	937.3517
**O-Desmethylindomethacin**	4.317_343.	NEG	4.317	343.0625
**Dibutyl adipate**	9.17_258.1	POS	9.17	258.1831
**dolichyl-4 phosphate**	9.585_440.	POS	9.585	440.3072
**O-Desmethylcarvedilol**	5.014_392.	POS	5.014	392.1734
**Bis(glutathionyl)spermine; N1,N12-Bis(glutathionyl**	8.288_780.	POS	8.288	780.3608
**(2Z)-4,6-dihydroxy-2-[(4-hydroxy-3,5-dimethoxyp**	4.441_347.	POS	4.441	347.1005
**7-Methoxycoumarin-3-carbonyl azide**	3.776_245.	NEG	3.776	245.0427
**6-Chloro-7,8-dihydroxy-3-allyl-1-phenyl-2,3,4,5-t**	5.539_329.	POS	5.539	329.1197
**Clocortolone**	5.888_410.	POS	5.888	410.1642
**N-(9Z-hexadecenoyl)-homoserine lactone**	9.522_337.	NEG	9.522	337.2618
**Propyliodone**	5.544_446.	POS	5.544	446.8833
**D-Glucuronic acid**	0.77_216.C	POS	0.77	216.0247
**Scopine**	3.2_155.09	POS	3.2	155.0949
**PS(18:1(9Z)/19:1(9Z))**	11.152_80	NEG	11.152	801.5552
**Antofloxacin**	6.611_376.	NEG	6.611	376.1557
**N-Guanylhistamine;**	2.843_153.	POS	2.843	153.1017
**Idrapril**	4.884_258.	POS	4.884	258.1218
**OR-1896**	4.525_245.	POS	4.525	245.1171
**Gericudranins A**	3.968_516.	POS	3.968	516.1422
**(E)-3-(2-(1H-Tetrazol-5-YL)vinyl)-6-fluoro-1H-inc**	2.575_229.	POS	2.575	229.0774
**LysoPE(20:0/0:0)**	9.739_509.	POS	9.739	509.3487
**Mycinamicin II; Mirosamicin;**	5.716_727.	POS	5.716	727.4158
**(S)-5-[(4-Amino-4-carboxy-1-oxobutyl)amino]-2-**	3.969_311.	POS	3.969	311.0738
**N-Cyclohexylformamide**	4.651_127.	POS	4.651	127.0999
**Taurocholic acid**	7.561_515.	POS	7.561	515.2925
**1-Hydroxyibuprofen**	5.089_222.	NEG	5.089	222.1256
**L-tryptophanamide**	4.322_203.	POS	4.322	203.107
**Detca**	7.477_400.	NEG	7.477	400.1884
**N-(3-acetamidopropyl)pyrrolidin-2-one**	3.225_184.	POS	3.225	184.1214
Chryseobacterium_hispanicum				
**Lovastatin acid**	8.163_422.	POS	8.163	422.2671
**2-Chloro-1,1,2-trifluoroethyl ethyl ether; Ethoxyflu**	0.724_162.	NEG	0.724	162.0061
**(2R,3R,4S,5S,6R)-2-[6-hydroxy-3-[(E)-3-hydroxybu**	3.218_390.	POS	3.218	390.2246
**2-Amino-1,5-dihydro-4H-imidazol-4-one**	0.716_99.C	POS	0.716	99.04343
**n-methyl-6-oxo-1,6-dihydropyridine-3-carboxami**	4.032_152.	NEG	4.032	152.0586
**Curcumin sulfate**	5.771_448.	NEG	5.771	448.0827
**Quercetin 3,4'-diglucoside**	4.571_626.	POS	4.571	626.1479
**2,4-Diamino-6-hydroxylaminotoluene;**	0.877_153.	POS	0.877	153.0905

**Sedoheptulose 7-phosphate**	0.642_290.	NEG	0.642	290.0401
**Piriprost**	8.177_425.	POS	8.177	425.2572
Anaerococcus_murdochii				
**POB-PE**	9.286_537.	NEG	9.286	537.3063
**Hydroxypioglitazone**	7.542_372.	POS	7.542	372.1142
**Hexanoylcarnitine**	4.638_259.	POS	4.638	259.1782
**Shanzhiside methyl ester**	4.457_406.	NEG	4.457	406.1489
**Omeprazole sulphone**	6.003_361.	POS	6.003	361.1094
**3,5-Dimethyl-1-(3-nitrophenyl)-1H-pyrazole-4-ca**	5.144_289.	POS	5.144	289.1056
**(1R,10R,12S,21R,22S,23R,24R)-23-(Dimethylamino)**	4.77_541.1	POS	4.77	541.1969
**Clofarabine**	2.848_303.	POS	2.848	303.0525
**PC(15:0/22:6(4Z,7Z,10Z,13Z,16Z,19Z))**	11.091_79.	POS	11.091	791.5465
**PS(17:2(9Z,12Z)/0:0)**	4.87_507.2	POS	4.87	507.2586
**1,3-Bis(4-(imidazolin-2-yl)-2-methoxyphenoxy)pr**	4.313_424.	NEG	4.313	424.2099
**N-Acetyl-L-cysteine**	7.103_163.	POS	7.103	163.0304
**Tarazepide**	5.786_448.	POS	5.786	448.1879
**NCGC00384980-01!5-[5-[3,4-dihydroxy-5-(hydrox**	6.658_513.	POS	6.658	513.248
**PS(17:1(9Z)/22:2(13Z,16Z))**	10.5_827.5	NEG	10.5	827.5698
**7-Hydroxy-5-methoxy-4-methyl-3-(4-methylpipe**	5.003_304.	NEG	5.003	304.1423
**D-Cysteine**	6.511_121.	POS	6.511	121.0199
**Sealdin**	0.638_305.	NEG	0.638	305.0745
**Cucurbitacin I**	7.602_514.	POS	7.602	514.2922
**Cytochalasin H1**	8.559_507.	POS	8.559	507.2967
**Ecalcidene**	9.133_455.	POS	9.133	455.3403
**Progabide**	0.743_334.	POS	0.743	334.0876
**Fosbretabulin**	5.768_396.	NEG	5.768	396.0994
**Valyl-leucyl-lysine 4-nitroanilide**	5.331_478.	NEG	5.331	478.2903
**Alfentanil**	7.987_416.	POS	7.987	416.2539
**TRIMETHYLCOLCHICINIC ACID**	5.065_343.	POS	5.065	343.1417
**N-Demethyl-desepoxyansamitocin P-3; N-Demeth**	6.673_604.	NEG	6.673	604.2553
**Pentaethylene glycol**	3.533_238.	POS	3.533	238.1417
**4-[2-[(1R,4aS,5R,8aS)-6-hydroxy-5-(hydroxymeth**	6.407_334.	POS	6.407	334.2139
**1H-Indazol-3-ol**	3.487_134.	POS	3.487	134.0483
**Sabine**	8.185_495.	POS	8.185	495.3201
**Dendryphiellic acid B**	5.306_170.	NEG	5.306	170.0944
**3-Methyl-1-butene**	10.085_70.	POS	10.085	70.07834
**PE(0:0/20:4;O2)**	6.922_533.	POS	6.922	533.2743
**Dimethamine;**	7.739_408.	POS	7.739	408.2512
**2-Methoxy-5-(2,4-dioxo-5-thiazolidinyl)-N-((4-(t**	6.991_422.	POS	6.991	422.1071
**METESIND**	7.446_436.	NEG	7.446	436.1557
**3-(4-Deoxy-beta-D-gluc-4-enuronosyl)-N-acetyl-**	2.826_379.	POS	2.826	379.1128
**N-Acetyl desmethyl frovatriptan**	5.442_273.	POS	5.442	273.1487
**Ceftazidime anhydrous**	3.38_546.1	POS	3.38	546.1001
**3-Hydroxypropionitrile**	1.029_71.0	POS	1.029	71.03726
Bacillus_subtilis				
**Tetraphenylporphyrin**	4.178_614.	NEG	4.178	614.2493
**N-(3-hydroxy-eicosanoyl)-homoserine lactone**	8.665_411.	POS	8.665	411.3351
**Am-PE(16:0/18:1(9Z))**	8.754_879.	POS	8.754	879.5823
Other				
**Tryptoquivaline**	8.008_546.	POS	8.008	546.2123
**PI(21:0/20:5(5Z,8Z,11Z,14Z,17Z))**	7.103_926.	NEG	7.103	926.5892
**Pterocarpanquinone**	3.212_304.	POS	3.212	304.0728
**Cimetidine sulfoxide**	2.998_268.	NEG	2.998	268.1104
**Acetyl-pepstatin**	9.291_643.	NEG	9.291	643.4133
**Dihydroxyethyldithiocarbamate**	2.766_181.	NEG	2.766	181.0232
**Isolysergol**	6.72_254.1	POS	6.72	254.1412
**5a-Pregnan-3,20-dione**	9.955_316.	POS	9.955	316.2401
**Cyclo(L-prolyl-L-valyl)**	4.323_196.	POS	4.323	196.1215

**Choline**	0.717_103.	POS	0.717	103.0998
**Tetracosahexaenic acid**	12.447_35(	POS	12.447	356.2714
**[(1R,2S,3S,4R,4aS)-2,3-dihydroxy-3,4a,8,8-tetramet**	10.321_39(	POS	10.321	390.1791
**2-Oxo-2-(3,4,5-trimethoxyphenyl)acetaldehyde**	6.8_224.06	POS	6.8	224.0685
**Pcera-1**	7.208_387.	NEG	7.208	387.1795
**Gypsogenin 3-O-b-D-glucuronide**	9.008_646.	POS	9.008	646.3718
**Tirofiban**	8.149_440.	POS	8.149	440.2349
**methyl (1R,4aS,9S,10S)-10-acetyloxy-5,9-dihydrox**	7.115_433.	POS	7.115	433.2116
**3-[7-hydroxy-8-(2-hydroxypropan-2-yl)-11-meth**	8.872_462.	POS	8.872	462.2258
Vagococcus_fluvialis				
Other				
Aquabacterium_commune				
**3-(4-Methylsulfonylphenyl)-4-phenyl-5-trifluorom**	3.378_367.	POS	3.378	367.0485
**Styrene**	8.053_104.	POS	8.053	104.0627
**Hexa-1,3,5-trienylbenzene**	8.04_156.C	POS	8.04	156.0941
Blautia_hansenii				
Other				
Helicobacter_mastomyrinus				
Helicobacter_rodentium				
Filomicrobium_fusiforme				
Pelagibacterium_halotolerans				
Clostridium_huakuii				
Winogradskyella_epiphytica				
Cerasicoccus_arenae				
Veillonella_montpellierensis				
Other				
**Gentisic acid**	1.234_154.	NEG	1.234	154.0267
**Megalomicin A;**	8.762_876.	POS	8.762	876.5533
**5-Hydroxytryptophol glucuronide**	5.765_339.	POS	5.765	339.1317
**Urdamycin B;**	5.714_696.	POS	5.714	696.2781
**8(S)-Hydroxy-(5Z,9E,11Z,14Z)-eicosatetraenoic aci**	9.24_320.2	NEG	9.24	320.2352
**Sepiapterin**	6.24_237.C	NEG	6.24	237.0857
**4-Aminophenol**	2.871_109.	NEG	2.871	109.0528
**3-[(1-Carboxyvinyl)oxy]benzoate; 3-[(1-Carboxyetl**	3.056_208.	POS	3.056	208.0372
**Myelopeptides**	9.563_680.	POS	9.563	680.3531
**bungeiside C**	3.844_430.	POS	3.844	430.1463
**5-((1-Ethyl-4-piperidinyl)oxy)-9H-pyrrolo(2,3-b:5,**	4.956_321.	POS	4.956	321.1574
**huperzine b**	8.746_256.	POS	8.746	256.1576
**1-(1-(Tert-Butoxycarbonyl)piperidin-4-yl)azetidine**	6.725_284.	POS	6.725	284.1735
**1,4-Bis((m-methoxyphenoxy)acetyl)piperazine**	4.314_414.	NEG	4.314	414.1811
**13-HODE**	9.269_296.	NEG	9.269	296.235
**1,4,8,11-Tetraoxacyclotetradecane**	4.023_204.	NEG	4.023	204.1363
**Flavan-3-ol**	7.558_226.	POS	7.558	226.0997
**Methyldopate**	6.84_239.1	POS	6.84	239.1159
**2-Methylnaphthalene; beta-Methylnaphthalene;**	8.039_142.	POS	8.039	142.0784
**Hydrangeic acid**	4.825_256.	POS	4.825	256.0736
**4'-Thiothymidine**	2.841_258.	POS	2.841	258.0675
**Chaps**	8.484_614.	NEG	8.484	614.3936
**Quercetin**	5.424_302.	NEG	5.424	302.0461
**N-[[(5S)-3-(4,6-Dihydropyrrolo[1,2-a][4,1]benzoxa**	7.494_341.	POS	7.494	341.1375
**Ethyl-4-dimethylaminobenzoate**	7.29_193.1	POS	7.29	193.1105
**Amlodipine**	5.63_408.1	NEG	5.63	408.1468
**NA**	8.334_454.	NEG	8.334	454.2926
**levan n**	6.841_504.	POS	6.841	504.1681
**Diaveridine**	4.616_260.	POS	4.616	260.1262
**(2S,3S,5S,8S,9S,10S,13S,14S,17S)-17-Acetyl-2-(2,2-**	8.568_445.	POS	8.568	445.3196
**N-palmitoyl serine**	8.73_343.2	POS	8.73	343.2722
**Ecdysterone**	7.263_480.	NEG	7.263	480.3084

**Methionine butyl ester**	4.783_205.	POS	4.783	205.114
**NORMETANEPHRINE**	4.498_183.	POS	4.498	183.0899
**2'-Methyl-4'-(5-methyl-(1,2,4)-oxadiazol-3-yl)bip**	7.385_497.	POS	7.385	497.2437
Other				
**Loliolide**	5.065_196.	POS	5.065	196.1102
**2R,3S,4S,5R,6S)-2-[[(2R,3R,4R)-3,4-dihydroxy-4-(h**	6.915_472.	POS	6.915	472.196
**Ingenol-3-angelate**	4.567_452.	POS	4.567	452.2196
**2-(4-Allyl-2,6-dimethoxyphenoxy)-1-(4-hydroxy-3**	4.325_396.	POS	4.325	396.1532
**SIMEPREVIR**	5.744_749.	POS	5.744	749.2926
**4-Methylcoumarin**	4.839_160.	NEG	4.839	160.0525
**4,4'-(Diphenylethenylidene)bis[N,N-dimethylbenze**	3.96_418.2	POS	3.96	418.2428
**3,4-Dihexylphthalic Acid**	8.844_334.	POS	8.844	334.2145
**bhas#40**	8.052_502.	NEG	8.052	502.3848
**Nicaraven**	5.73_284.1	POS	5.73	284.1264
**PS(17:1(9Z)/12:0)**	9.717_691.	POS	9.717	691.4423
**Bis(5-phenylpentyl) benzene-1,2-dicarboxylate**	7.483_458.	POS	7.483	458.2451
**LMST05040006**	8.447_557.	POS	8.447	557.3391
**2-Naphthalenesulfonic acid**	4.64_208.0	NEG	4.64	208.0194
**4-Bis(2-hydroxyethyl)amino-L-phenylalanine**	5.365_268.	POS	5.365	268.1424
**N-Acetyl-ala-ala-ala-methylester; NAc-AAA-ME;**	3.305_287.	POS	3.305	287.148
**5-(Acetoxymethyl)-2-methyl-5,6,7,7a-tetrahydro-3**	4.616_329.	POS	4.616	329.1112
**17-phenyl-trinor-PGF2alpha amide**	7.535_387.	POS	7.535	387.2408
**Kansuinine B;**	4.865_722.	POS	4.865	722.2547
**Avermectin A1a**	7.035_886.	POS	7.035	886.5113
**Icosa-5,8,11,14-tetraenal**	9.764_288.	POS	9.764	288.2454
**Visnadin**	5.873_388.	POS	5.873	388.1523
**Methyl N-[6-[(4-pyridin-2-ylpiperazine-1-carbony**	3.905_395.	POS	3.905	395.1691
**Acenaphthylene**	8.042_152.	POS	8.042	152.0627
**Dihydrorhizobitoxine; (2S)-2-Amino-4-[(2R)-2-am**	0.715_192.	POS	0.715	192.1111
**Phosphocholine**	0.685_205.	POS	0.685	205.0482
**Okanin 3',4'-diglucoside**	3.992_612.	NEG	3.992	612.1707
**Solacetal D**	7.859_508.	NEG	7.859	508.3034
**LMST05010036**	7.2_484.26	NEG	7.2	484.267
**2-Nonenal, 4-oxo-**	10.175_15	POS	10.175	154.0996
**OKHdiA-PS**	5.362_677.	POS	5.362	677.3207
**Coniferyl ferulate**	6.02_356.1	POS	6.02	356.1254
**4,6-dihydroxy-4-(hydroxymethyl)-3,4a,8,8-tetrame**	5.399_285.	POS	5.399	285.1936
**2-Thiouric acid**	3.904_184.	POS	3.904	184.0058
**(4-Methoxyphenyl)-morpholin-4-yl-sulfanyl-sulfa**	3.361_289.	POS	3.361	289.0373
**Setoperone**	5.355_401.	POS	5.355	401.1585
**Tiglylglycine**	2.638_157.	POS	2.638	157.0827
**PC(22:5(7Z,10Z,13Z,16Z,19Z)/16:0)**	12.109_80	POS	12.109	807.5763
**Capivasertib**	3.062_428.	POS	3.062	428.1734
**LMPR0103010026**	3.576_332.	POS	3.576	332.0943
**Cetirizine N-oxide**	5.789_404.	NEG	5.789	404.1505
**Isomurolic acid**	8.129_368.	NEG	8.129	368.2562
**PG(22:6(4Z,7Z,10Z,13Z,16Z,19Z)/18:4(6Z,9Z,12Z,15Z**	8.225_814.	POS	8.225	814.48
**Cyenopyrafen;**	3.246_393.	POS	3.246	393.2424
**N-Nitrosofolic acid;**	4.56_470.1	POS	4.56	470.1301
**Maduramycin**	9.306_916.	POS	9.306	916.5381
**Panipenem**	5.925_339.	POS	5.925	339.1252
**Cyclo(Pro-Phe-D-Trp-Lys-Thr-Phe)**	8.561_806.	NEG	8.561	806.4113
Methanobrevibacter_smithii				
**Artemisinic acid**	9.305_234.	POS	9.305	234.1622
**trans-Hexadec-2-enoyl carnitine**	8.491_397.	POS	8.491	397.3192
**Doramapimod**	8.885_527.	POS	8.885	527.2892
Methylobacterium_tardum				
**Vanillylmandelic acid**	2.112_180.	POS	2.112	180.0425

L-Leucine-p-nitroanilide	3.486_251.	POS	3.486	251.1273
Davallialactone	4.604_464.	POS	4.604	464.1117
Spinacetin 3-gentiobioside	3.813_670.	NEG	3.813	670.1735
Sordarin	8.196_492.	POS	8.196	492.2732
Cefluprenam	3.858_556.	POS	3.858	556.1299
CC(C)=CCCC(C)=CCc1c(O)ccc(C(=O)C=Cc2ccc(O)cc:	5.667_392.	NEG	5.667	392.1978
Deoxyloganic acid tetraacetate;	4.418_528.	NEG	4.418	528.184
Evogliptin	4.383_401.	POS	4.383	401.1915
3,5-Dihydroxycinnamic acid	8.789_180.	POS	8.789	180.0425
(2S,3S,4S,5R)-6-[[3,5-Dihydroxy-2-(4-hydroxy-3-n	5.527_480.	NEG	5.527	480.1268
Cytidine	0.67_243.C	NEG	0.67	243.087
Ilomastat	7.064_388.	POS	7.064	388.2096
6-HYDROXYANGOLENSIC ACID METHYL ESTER	5.564_508.	POS	5.564	508.2061
Sphingofungin E	3.958_417.	POS	3.958	417.2726
Phorbol-12,13-dibutyrate	9.425_504.	NEG	9.425	504.2721
4-Heptylphenol	10.185_19:	POS	10.185	192.1517
Abt-751	0.705_371.	NEG	0.705	371.0957
Tetranor-12(S)-HETE	3.974_288.	POS	3.974	288.1685
10-Deacetyl-2-debenzoylbaccatin III;	4.483_440.	NEG	4.483	440.2044
Diterpenoid EF-D; 12-Deoxy-phorbol-13-alpha-m	8.207_474.	POS	8.207	474.2621
Sarcoehrendin A	8.208_510.	POS	8.208	510.2835
Euparin	7.268_216.	POS	7.268	216.0789
Fuziline	5.932_453.	NEG	5.932	453.2725
Epothilone C	7.683_477.	POS	7.683	477.2553
Sufotidine	6.992_421.	NEG	6.992	421.2136
Fluspirilene	5.916_475.	POS	5.916	475.2434
Other				
LMPR0106150046	5.714_828.	POS	5.714	828.4504
14R-hydroxy-11E-eicosenoic acid	10.187_32	POS	10.187	326.282
7-Hydroxygliclazide	4.81_339.1	NEG	4.81	339.1254
1-Dehydro-9-fluoro-11-oxotestololactone;	9.667_332.	POS	9.667	332.1438
3-cis-Hydroxyglipizide	5.722_461.	POS	5.722	461.1735
3,6,9-Hexadecatriene	9.803_220.	POS	9.803	220.2194
Metoprolol	8.665_267.	POS	8.665	267.1835
Afrormosin 7-O-(6''-malonylglucoside)	6.248_546.	NEG	6.248	546.1365
[4,5-dihydroxy-3,4-bis(hydroxymethyl)-4a,8,8-trim	6.844_401.	POS	6.844	401.2777
1-Methyl-1H-indole-3,5,6-triol	5.33_179.C	POS	5.33	179.0585
4-Oxo-enoxacin	3.105_334.	NEG	3.105	334.1087
Kaempferol 3-sulfate	5.529_366.	NEG	5.529	366.0046
Glycine, N-[1-(phenylacetyl)-L-prolyl]-	5.905_290.	POS	5.905	290.1267
4-((4-(1H-Tetrazol-1-yl)phenoxy)methyl)-2-(1-(5-	7.103_448.	POS	7.103	448.1782
Cer(d18:0/18:0)	9.728_567.	POS	9.728	567.5594
6-Hydroxynicotinic acid	4.6_139.02	NEG	4.6	139.027
(1R,3R,6R,8S,10R,13S,16S,17R)-8-tert-butyl-6,17-d	5.298_408.	POS	5.298	408.1421
Emedastine	9.858_302.	POS	9.858	302.2096
Amsacrine	5.344_393.	POS	5.344	393.1132
Notatic acid	4.714_361.	POS	4.714	361.1182
Cer(d18:1/14:0)	11.003_50!	POS	11.003	509.4815
Anacetrapib	3.686_637.	NEG	3.686	637.1698
5-(3-(4-(2-(4-Fluorophenyl)ethoxy)phenyl)propyl)	7.017_368.	POS	7.017	368.1406
2-Amino-5-[[1-[carboxymethyl(2-hydroxypropano	2.216_379.	POS	2.216	379.1051
Thelephoric acid	0.667_352.	NEG	0.667	352.0197
Norclozapine	3.58_312.1	POS	3.58	312.1141
Pyro(l-alpha-aminoadipyl)-L-histidyl-L-thiazolidin	3.433_394.	POS	3.433	394.1412
Sebacoyl-L-carnitine	5.12_345.2	POS	5.12	345.2146
Ipragliflozin	4.576_404.	POS	4.576	404.1078
Cer(d18:0/18:1(11Z))	11.968_56!	POS	11.968	565.5439
Piperidine	1.483_85.C	POS	1.483	85.08924

**2-Phenyl-3,4-dihydro-2H-thiochromene**	4.405_226.	POS	4.405	226.082
**Topostin B553**	12.418_55	NEG	12.418	553.4701
**Halistanol sulfonic acid B**	4.892_644.	POS	4.892	644.2017
**1,3,7-Trimethyl-8-nonylpurine-2,6-dione**	9.859_320.	POS	9.859	320.2201
**PGD2 ethanolamide**	7.436_395.	POS	7.436	395.267
**4-Chloro-3-ethoxy-7-guanidinoisocoumarin**	0.623_281.	NEG	0.623	281.0567
**(Ac)2-L-Lys-D-Ala; (Ac)2-L-lysyl-D-alanine;**	3.124_301.	POS	3.124	301.1637
**hirsutanone**	6.272_328.	NEG	6.272	328.1312
**3-Hydroxybenzoic acid**	4.033_138.	POS	4.033	138.032
Clostridium_phytofermentans				
**1,4a-dimethyl-6-methylidene-5-[(2E)-3-methylpe**	8.591_324.	POS	8.591	324.2066
**Oxamniquine**	5.361_279.	POS	5.361	279.1584
**3-Hydroxypicolinic acid**	4.782_139.	NEG	4.782	139.027
**Dimethenamid**	2.43_275.0	POS	2.43	275.0736
**N-Acetyl-DL-phenylalanine**	6.612_207.	POS	6.612	207.0896
**PS(22:4(7Z,10Z,13Z,16Z)/0:0)**	8.334_573.	POS	8.334	573.3088
**Perillic acid**	6.4_166.09	NEG	6.4	166.0994
**dehydroxynocardamine**	8.21_606.3	POS	8.21	606.3345
**N-(6,8-Difluoro-2-methyl-4-quinolinyl)-N'-[4-(di**	9.633_356.	POS	9.633	356.1438
**catechin(3',4',5,7-tetraacetate)-3-dodecanoate**	8.826_640.	POS	8.826	640.2898
Acetobacter_pasteurianus				
**Tubulysin A;**	8.176_843.	POS	8.176	843.4462
**NPC**	8.195_518.	POS	8.195	518.2165
**(5R)-5-(1,2-Dihydroxyethyl)-3-hydroxyoxolane-2,**	0.789_176.	POS	0.789	176.0323
**PC(22:6(4Z,7Z,10Z,13Z,16Z,19Z)/16:0)**	11.343_80	POS	11.343	805.5618
**LMPK12113049**	0.646_334.	NEG	0.646	334.0312
Sphaerochaeta_pleomorpha				
**Persin**	9.759_380.	POS	9.759	380.2926
**Patulin**	1.194_189.	POS	1.194	189.064
**Carbutamide**	3.683_271.	POS	3.683	271.0991
Edwardsiella_ictaluri				
Desulfovibrio_vulgaris				
**L-Ascorbic acid 2-glucoside**	6.894_338.	POS	6.894	338.0833
**Pefloxacin**	3.647_333.	POS	3.647	333.1489
**Testosterone 17b-(b-D-glucuronide)**	6.803_464.	POS	6.803	464.2413
**N-(2E-dodecenoyl)-homoserine lactone**	7.057_281.	POS	7.057	281.199
**7-Hydroxyamoxapine**	3.925_329.	POS	3.925	329.0929
**Azaspiracid;**	6.832_841.	NEG	6.832	841.5017
**PC(20:4(8Z,11Z,14Z,17Z)/18:0)**	12.108_80	POS	12.108	809.5934
**Ibufenac**	6.757_192.	POS	6.757	192.1152
Other				
**Takinib**	4.157_322.	POS	4.157	322.1428
**5-hydroxy-2,2,6,6-tetramethyl-4-[2-methyl-1-[2,4**	5.539_432.	POS	5.539	432.215
**Tanariflavanone B**	9.814_490.	POS	9.814	490.2374
**(2S,6R,8aS)-6-(2-hydroxypropan-2-yl)-8a-methyl-**	10.037_23	POS	10.037	238.1934
**Costatol;**	0.8_333.92	NEG	0.8	333.9291
**Prim-O-glucosylcimifugin**	5.033_468.	POS	5.033	468.1652
**PE(22:2(13Z,16Z)/18:3(9Z,12Z,15Z))**	11.263_79	POS	11.263	793.5622
**Maculosine;**	3.006_331.	NEG	3.006	331.1062
**N-(2E,9Z-hexadecadienoyl)-homoserine lactone**	8.078_335.	POS	8.078	335.2449
**Lycoricidine;**	3.674_291.	POS	3.674	291.0742
**Diffractaic acid**	4.459_374.	NEG	4.459	374.1366
Other				
**5-Methoxyindole**	3.445_147.	NEG	3.445	147.0686
**Asenapine**	4.945_285.	POS	4.945	285.0931
**3-Chloro-2-hydroxypropanoic acid**	9.48_123.9	POS	9.48	123.9928
**Gramine**	4.974_129.	POS	4.974	129.058
**PE(0:0/18:4(6Z,9Z,12Z,15Z))**	6.718_473.	POS	6.718	473.253

**Monohydroxydiacetyltaxine**	5.53_501.1	POS	5.53	501.1408
**Isouron;**	3.098_211.	POS	3.098	211.1325
**Kadsurin A**	7.574_372.	POS	7.574	372.1575
**3-[[4-[(5-Nitropyridin-2-yl)hydrazinylidene]-3-oxo**	3.36_423.1	POS	3.36	423.1205
**Iridodial glucoside tetraacetate**	4.192_498.	NEG	4.192	498.2096
**PREGNENOLONE SULFATE**	7.67_396.1	NEG	7.67	396.1972
**Tyramine-O-sulfate**	3.541_217.	POS	3.541	217.0411
**Methohexital**	7.007_262.	POS	7.007	262.1318
**Bis(glutathionyl)spermine disulfide;**	6.994_778.	POS	6.994	778.3429
**(E)-8-(2-(4-(Diphenylamino)benzylidene)hydraziny**	10.105_45	POS	10.105	458.2332
**oscr#27**	9.625_400.	POS	9.625	400.2824
**Hippurate nitrogen**	5.471_548.	NEG	5.471	548.156
**NCGC00347765-02![(7R)-5-chloro-3-[(1E,3E,5S)-3,**	5.732_433.	POS	5.732	433.1652
**Fungichromin; Pentamycin;**	6.878_670.	POS	6.878	670.395
**Fasidotril**	5.802_443.	POS	5.802	443.1417
**Stachyflin**	8.469_385.	POS	8.469	385.2254
**NCGC00385245-01!(2E,4E,14E)-15-(1,3-benzodiox**	8.081_411.	POS	8.081	411.2771
**CPA(16:0)**	9.842_392.	NEG	9.842	392.2329
**(R)-4-(2-(2-(2-Methylpyrrolidin-1-yl)ethyl)benzof**	8.141_330.	POS	8.141	330.1731
**Histidinyl-Leucine**	2.442_268.	NEG	2.442	268.1534
**Clerosterol 3-glucoside**	10.044_57	POS	10.044	574.4216
**Olvanil**	9.824_417.	POS	9.824	417.3244
**PGF2alpha-11-acetate methyl ester**	8.955_410.	POS	8.955	410.2671
**vanilin**	5.409_152.	NEG	5.409	152.0474
**Tilmacoxib**	2.921_338.	POS	2.921	338.1113
Rhodanobacter_spathiphylli				
Mycobacterium_immunogenum				
**Coenzyme Q2**	9.859_318.	POS	9.859	318.187
**[2,2'-Biquinoline]-4,4'-dicarboxylic acid**	3.575_344.	POS	3.575	344.0791
**Tropine**	1.043_141.	POS	1.043	141.1156
**MG(0:0/16:0/0:0)**	9.805_330.	POS	9.805	330.2768
**PS(22:6(4Z,7Z,10Z,13Z,16Z,19Z)/14:1(9Z))**	8.084_777.	POS	8.084	777.4556
**Itanoxone**	4.32_300.0	NEG	4.32	300.0565
**1-Palmitoyl-2-(5-keto-8-oxo-6-octenoyl)-sn-glyc**	8.645_647.	POS	8.645	647.3802
**Carylophyllene Oxide**	9.236_206.	POS	9.236	206.1673
**Hydroxyclomipramine glucuronide**	5.72_506.1	POS	5.72	506.1839
**2-Methylthioadenosine**	3.475_313.	POS	3.475	313.0841
**Oxycodone**	4.845_315.	POS	4.845	315.1471
**(S)-ACPA; (S)-2-Amino-3-(3-carboxy-5-methyl-4-**	0.748_214.	POS	0.748	214.0594
**Diazinon;**	3.359_304.	POS	3.359	304.1002
**Methoxsalen**	3.532_216.	POS	3.532	216.0434
**Scopoloside II;**	8.623_770.	NEG	8.623	770.4121
**2-Ethyloxirene**	3.48_70.04	POS	3.48	70.04204
Methylobacterium_bullatum				
**Glycolithocholic acid sodium salt**	8.99_455.3	POS	8.99	455.3015
**Deoxycorticosterone sulfate**	6.755_410.	NEG	6.755	410.1764
**2-Linoleoyl glycerol**	12.479_33	POS	12.479	336.2664
**DG(18:0/22:6(4Z,7Z,10Z,13Z,16Z,19Z)/0:0)**	10.209_66	NEG	10.209	668.5405
**Guanidinosuccinic acid**	0.727_175.	POS	0.727	175.0595
**Cetylmannoside**	10.715_40	NEG	10.715	404.314
**Ala-Ala-Ala**	1.069_231.	POS	1.069	231.1222
**2,7-Diamino-7-iminoheptanoic acid**	2.632_173.	POS	2.632	173.1167
**1-O-alpha-D-glucopyranosyl-1,2-nonadecandiol**	8.69_462.3	POS	8.69	462.3573
**Tomaymycin hemiaminal (S.pur)**	4.214_290.	POS	4.214	290.0902
**Dapt**	3.691_432.	POS	3.691	432.1861
**p-Fluorohippuric acid**	0.656_197.	NEG	0.656	197.0498
**N-(N-L-gamma-Glutamyl-S-nitroso-L-cysteinyl)gl**	8.915_336.	POS	8.915	336.0747
**BIDISOMIDE**	7.366_407.	NEG	7.366	407.2342

**HUMULENE (alpha)**	0.751_226.	POS	0.751	226.1684
**N-(8-Amino-1-carboxyoctyl)-alanyl-proline**	8.196_357.	POS	8.196	357.2269
**N-[5-Methyl-8-(4-methylpiperazin-1-yl)-1,2,3,4-t**	8.269_448.	POS	8.269	448.2828
**Sodium ortho-phenylphenate;**	9.029_192.	POS	9.029	192.1882
**Topiramate**	2.441_339.	POS	2.441	339.0985
**Betamethasone 17 Valerate**	5.879_476.	POS	5.879	476.2561
**Cycloaltilisin 7**	5.524_406.	POS	5.524	406.1776
**Nandrolone decanoate**	9.78_428.3	POS	9.78	428.3292
**(+)-Epibatidine**	3.579_208.	POS	3.579	208.0772
**Luteolin**	5.738_286.	NEG	5.738	286.0473
**(E)-3-hydroxy-6-(4-hydroxy-6-methoxy-7-methyl**	3.459_353.	POS	3.459	353.1472
**loganic acid**	3.351_376.	NEG	3.351	376.1372
**6-Hydroxymethylpterin**	5.919_193.	POS	5.919	193.0598
**10-Nitrooleate**	10.2_344.2	POS	10.2	344.2714
**Leucine**	1.719_131.	POS	1.719	131.0948
**1,3-Benzenedicarbonitrile, 2-[[4-[[2-(acetyloxy)eth**	6.792_448.	POS	6.792	448.1844
**Diethylaminoethyl-dextran**	4.485_603.	POS	4.485	603.276
**gamma-Glutamyl-5-hydroxytryptophan**	4.891_349.	POS	4.891	349.1272
**9-Hydroxy-9-methoxycarbonyl-8-methyl-14-n-pr**	5.71_525.1	POS	5.71	525.1902
**Theophylline**	3.472_180.	POS	3.472	180.0649
**NCGC00385003-01!4-[3-methyl-5-(5,6,7-trihydrox**	8.206_424.	POS	8.206	424.2827
**5-Hydroxyindole-3-acetic acid**	3.446_145.	NEG	3.446	145.0529
**Epitestosterone glucuronide**	6.567_464.	NEG	6.567	464.2408
**Deltoidin A**	7.428_334.	POS	7.428	334.1779
**Ubiquinones**	10.285_31	POS	10.285	318.1829
**Homocastasterone**	9.792_478.	POS	9.792	478.3663
**hbas#9**	5.967_368.	POS	5.967	368.1469
**Avocadene 4-acetate**	9.521_328.	POS	9.521	328.2613
**Phthalimidinoglutarimide**	3.303_244.	POS	3.303	244.0846
**2-tridecene-4,7-diynal**	8.547_188.	POS	8.547	188.1203
**Homovanillate**	4.165_204.	POS	4.165	204.0401
**6-bromo-docosa-5E,9Z-dienoic acid**	3.067_414.	POS	3.067	414.2115
**[(2S,3R)-2-Amino-3-hydroxybutanoyl] (2S)-pyrroli**	1.178_216.	POS	1.178	216.1112
Bacteroides_massiliensis				
**5-Chloro-2'-deoxyuridine**	5.027_262.	POS	5.027	262.035
**Octyl 4-methoxycinnamic acid**	8.314_290.	POS	8.314	290.1882
**Methionine propyl ester**	4.033_191.	POS	4.033	191.0981
**Ll-2,6-diaminoheptanedioate**	3.132_172.	POS	3.132	172.0851
Desulfovibrio_simplex				
**[5-hydroxy-3-(hydroxymethyl)-2-oxo-6-propan-2**	4.277_301.	POS	4.277	301.1888
**Sildenafil**	3.163_474.	POS	3.163	474.2078
Other				
Methanosphaera_cuniculi				
**Petrosaspongiolide m**	7.853_460.	POS	7.853	460.2822
**[(2R,3R,4S,5R,6S)-6-[(3S,4S,5S,6R)-4,5-dihydroxy-6**	5.492_447.	POS	5.492	447.1754
**5-(4-methyl-5-oxo-2H-furan-3-yl)pentanoic acid**	2.455_198.	NEG	2.455	198.0892
**Ritipenem acetoxymethyl ester**	4.405_360.	POS	4.405	360.0628
**(2-Mercaptomethyl-3-phenyl-propionyl)-glycine**	3.901_253.	POS	3.901	253.0776
**17(21)-Hopen-6-one**	10.134_42	POS	10.134	424.3706
**Thiourea, (5-phenyl-1,2,4-thiadiazol-3-yl)-**	3.536_236.	POS	3.536	236.0196
**NCGC00169827-03!(2E,4E)-12-hydroxy-13-(hydro**	9.631_324.	POS	9.631	324.1946
**Scyphostatin**	8.559_485.	POS	8.559	485.3137
**N-Trifluoroacetyladriamycin**	5.464_639.	POS	5.464	639.1548
**CID 9844747**	4.907_415.	POS	4.907	415.1902
**Maltitol**	5.455_344.	POS	5.455	344.1372
**Mascaroside;**	5.731_524.	POS	5.731	524.2281
**2,6-Diaminopurine**	3.597_150.	POS	3.597	150.0659
**Denopamine**	4.849_317.	POS	4.849	317.163

**25-hydroxy-1beta-hydroxymethyl-23,23,24,24-tet**	9.386_426.	POS	9.386	426.3135
**p-Benzosemiquinone**	0.734_109.	NEG	0.734	109.029
**[(2R,3S,4S,5R,6S)-6-[4-[(E)-3-(2,4-dihydroxypheny**	5.518_564.	POS	5.518	564.1637
Massilia_aerilata				
**Bafilomycin A1(Baf-A1)**	9.662_622.	NEG	9.662	622.4099
**Scopolamine**	3.177_303.	POS	3.177	303.1468
**1,3-(8R,9R-epoxy-octadec-13Z,15Z-dien-4,6-diyn**	8.757_874.	POS	8.757	874.5381
**2-Naphthaldehyde; 2-Naphthalenecarboxaldehyde**	5.971_156.	POS	5.971	156.0577
**1,3,5-Hexatriene**	3.445_80.0	POS	3.445	80.06274
**Kanamycin A 3'-phosphate; Kanamycin 3'-phospha**	0.657_564.	NEG	0.657	564.2022
**Milbemycin A4**	8.206_542.	POS	8.206	542.3244
**N-Acetyl-D-galactosamine**	0.961_203.	POS	0.961	203.0797
**Raticate**	5.389_511.	POS	5.389	511.1908
**6-bromo-heneicosa-5E,9Z-dienoic acid**	3.214_400.	POS	3.214	400.1958
**N-Oleoyl dopamine**	9.91_417.3	NEG	9.91	417.3243
**Semaxanib**	9.01_238.1	POS	9.01	238.1109
**Asparagusate; Asparagusic acid;**	3.537_149.	POS	3.537	149.9811
**Cannabidiolic acid**	7.161_358.	POS	7.161	358.2143
**Daidzein 4'-glucuronide-7-sulfate**	3.273_510.	NEG	3.273	510.0462
**Matteucinol 7-O-glucoside**	5.035_476.	POS	5.035	476.1662
**1,2-Dioleoyl-sn-glycero-3-phosphoethanolamine**	11.284_74	POS	11.284	743.5469
**2-Cyclooctyl-2-hydroxyethylamine**	7.946_171.	POS	7.946	171.1625
**Cer(d14:1/18:1)**	10.668_50	POS	10.668	507.4658
**Methyl 4-amino-5-ethyl-3-thiophenecarboxylate**	3.148_185.	POS	3.148	185.0511
Actinocatenispora_sera				
Rhodovarius_lipocyclicus				
Brevundimonas_staleyi				
Acinetobacter_indicus				
Thiomonas_arsenitoxydans				
Halobacteriovorax_litoralis				
Polaromonas_aquatica				
Deinococcus_aerius				
**Rhizocticin C;**	6.926_464.	POS	6.926	464.2164
**Salicylic acid**	2.879_138.	NEG	2.879	138.0317
**6|A-Hydroxy-7|A-(thiomethyl)spirolactone**	6.876_404.	POS	6.876	404.2021
**6-bromo-eicosa-5E,9Z-dienoic acid**	3.15_386.1	POS	3.15	386.1803
**Eucalyptol**	7.26_136.1	POS	7.26	136.1254
Rhizobium_cellulosilyticum				
**5-Chloro-1,3-dihydro-1-(4-piperidinyl)-2H-benzir**	1.461_251.	POS	1.461	251.0828
**(2S,3R)-2-(4-{2-[(3S,4S)-3,4-Dimethylpyrrolidin-1-**	3.57_477.1	POS	3.57	477.1977
**4,4-Dimethyl-2-[3-carboxylatopropyl]-2-tridecylo**	8.518_385.	POS	8.518	385.3192
**Alpha-collatolic acid**	8.646_526.	POS	8.646	526.2226
**Dibenzo[a,c]cyclooctene, 5,6,7,8-tetrahydro-**	8.026_208.	POS	8.026	208.1253
**beta-Hederin**	5.51_734.4	POS	5.51	734.4574
**PC(18:3(9Z,12Z,15Z)/19:1(9Z))**	11.767_79	POS	11.767	795.5777
**NCGC00380809-01!(6aS)-1,2,11-trimethoxy-6-met**	3.521_363.	POS	3.521	363.1464
**4-Hydroxynonenal**	10.189_15	POS	10.189	156.1152
**Meticillin**	3.577_380.	POS	3.577	380.1041
**S-Petasin**	9.859_334.	POS	9.859	334.1594
**(2R)-2-[[(1R,3S,4S)-3-[[4-(5-Benzyl-2-ethylpyrazo**	8.329_574.	POS	8.329	574.3658
**Atorvastatin**	7.81_558.2	NEG	7.81	558.2537
**Glyoxylic acid**	0.656_74.0	NEG	0.656	74.00034
**Oxisuran**	2.649_183.	NEG	2.649	183.0355
**p-Menthan-3-ol**	3.837_173.	POS	3.837	173.1782
Herminiimonas_contaminans				
**Flurochloridone**	0.739_311.	POS	0.739	311.0081
**Ustalic acid;**	0.8_338.07	POS	0.8	338.0784
Corynebacterium_argentoratense				

Other				
**Decanoylcarnitine**	7.128_315.	POS	7.128	315.2406
**Paclobutrazol**	4.936_293.	POS	4.936	293.1296
**Pandaroside C**	7.544_768.	POS	7.544	768.3939
**1-[(3R,4R,5R)-3-Fluoro-3,4-dihydroxy-5-(1-hydro:**	3.227_276.	POS	3.227	276.0745
**reynosin**	7.765_248.	POS	7.765	248.1413
**PC(20:4(5Z,8Z,11Z,14Z)/16:1(9Z))**	11.15_779.	POS	11.15	779.546
**Tris(2-butoxyethyl) phosphate**	8.465_398.	POS	8.465	398.2435
**4-Methyl-1-pentene**	10.29_84.C	POS	10.29	84.09397
**Zaldaride**	7.119_428.	NEG	7.119	428.2232
**1-acyl-PAF**	8.831_537.	POS	8.831	537.3441
Hespellia_porcina				
**Penitrem D**	9.344_567.	POS	9.344	567.3327
**Docosanediol-1,14-disulfate**	10.261_50.	POS	10.261	502.2614
Mitsuokella_multacida				
**Acalyphin;**	6.001_360.	POS	6.001	360.1158
**Tert-Butyl 3-((3S,6S,12aS)-6-isobutyl-9-methoxy-:**	6.784_469.	POS	6.784	469.2579
**Diuron**	0.633_232.	NEG	0.633	232.0162
**5-Oxo-prolyl-glycyl-arginine-4-nitroanilide**	3.227_462.	POS	3.227	462.1966
**ZORUBICIN**	7.553_645.	POS	7.553	645.2318
**Netivudine**	4.928_282.	POS	4.928	282.0851
**4-(4-Methylcyclohexyl)-4-oxobutanoic acid**	7.194_198.	POS	7.194	198.1258
Stenotrophomonas_maltophilia				
**Methandriol dipropionate;**	10.244_41(	POS	10.244	416.293
**13-Docosenamide**	10.361_33	POS	10.361	337.3342
**PE(0:0/18:3(9Z,12Z,15Z))**	9.572_475.	POS	9.572	475.2679
**Inhibitor CC**	9.416_806.	POS	9.416	806.5396
**Formyl-l-methionyl peptide**	3.718_177.	POS	3.718	177.0461
**U-44069**	9.26_332.2	POS	9.26	332.2327
Blautia_faecis				
**Archangelolide;**	6.786_548.	POS	6.786	548.2606
**PC(14:0/20:1(11Z))**	12.04_759.	POS	12.04	759.5767
**Bacillamidin B**	6.876_357.	POS	6.876	357.2512
**PA(P-16:0/18:2(9Z,12Z))**	9.377_656.	POS	9.377	656.4761
**Penicillic acid**	4.544_170.	POS	4.544	170.0581
**Phloretin 2'-O-glucuronide**	5.463_450.	POS	5.463	450.1165
**Eudesobovatol A;**	9.536_504.	POS	9.536	504.3221
**2-O-(beta-D-galactopyranosyl-(1->6)-beta-D-gal**	8.18_514.2	POS	8.18	514.2272
**PG(P-16:0/20:5(5Z,8Z,11Z,14Z,17Z))**	5.331_752.	POS	5.331	752.5028
**2-C-Methyl-D-erythritol 4-phosphate;**	5.716_216.	POS	5.716	216.04
**Pyridinoline**	7.461_428.	POS	7.461	428.1923
**LysoPC(20:5(5Z,8Z,11Z,14Z,17Z)/0:0)**	9.147_541.	POS	9.147	541.3177
**6'-Demethoxypraecansone B**	4.365_336.	POS	4.365	336.1357
**Clorazepate**	5.41_314.C	POS	5.41	314.0465
**2,4,6-Octatriynoic acid**	7.534_132.	POS	7.534	132.0213
**SR 49498**	6.723_446.	NEG	6.723	446.2433
**3-Hydroxybiphenyl; 3-Phenylphenol;**	7.533_170.	POS	7.533	170.0734
**Dalpatein 7-O-glucoside**	5.42_504.1	POS	5.42	504.1273
**11-(hydroxymethyl)-2,6,6,14,19-pentamethyl-7,16,**	6.857_466.	POS	6.857	466.1604
Other				
**Ethyl 2-(5-(4-chlorophenyl)pentyl)oxiran-2-carbox**	7.274_296.	NEG	7.274	296.1185
**Difenoconazole**	2.993_405.	POS	2.993	405.0664
**Diphenyl sulfide**	0.968_186.	NEG	0.968	186.0504
**Kurziflavolactone A**	5.347_526.	POS	5.347	526.2006
Bacteroides_plebeius				
**Dodecanoyl-sn-glycero-3-phosphocholine (isomer**	9.383_439.	POS	9.383	439.2702
**LMST01010327**	8.227_484.	POS	8.227	484.3406
**Cysteinylglycine**	9.468_178.	NEG	9.468	178.0413

**alpha-Allocryptopine**	8.109_369.	POS	8.109	369.1596
**4-Hydroxy tolbutamide**	3.686_286.	POS	3.686	286.0986
**VAPIPROST**	9.412_477.	POS	9.412	477.286
**Ecgonine Ethyl Ester**	6.819_213.	POS	6.819	213.1368
**3,5-Dimethyl-2-(3-pyridyl)thiazolidin-4-one**	4.39_208.C	NEG	4.39	208.067
Chryseobacterium_ureilyticum				
**15(R),19(R)-Hydroxy prostaglandin F2a**	6.189_370.	NEG	6.189	370.2354
**Indole-3-methyl acetate**	6.194_189.	POS	6.194	189.0792
**ethyl ((3,4,5-trihydroxy-6-((4-(4-isopropoxybenzyl**	6.898_522.	POS	6.898	522.2585
Bacteroides_intestinalis				
**PG(O-20:0/22:4(7Z,10Z,13Z,16Z))**	8.471_840.	POS	8.471	840.6216
**Galacturonic acid**	4.368_194.	NEG	4.368	194.0402
**Hesperetin 5,7-O-diglucuronide**	4.369_654.	POS	4.369	654.1436
**Pentylone**	5.265_235.	NEG	5.265	235.1208
**Xanthurenic acid**	3.621_205.	POS	3.621	205.038
**N-(7Z-tetradecenoyl)-homoserine lactone**	8.872_309.	POS	8.872	309.2304
**Etofibrate**	4.354_363.	POS	4.354	363.0885
**12-Hydroxy-12-octadecanoylcarnitine**	8.117_443.	POS	8.117	443.361
**Glycidyl oleate;**	12.78_338.	POS	12.78	338.282
**Uniconazole**	3.789_291.	POS	3.789	291.114
**5,6-dihydro-7-oxo-19,20-epoxycytochalasin C**	9.251_523.	POS	9.251	523.2587
**Aminomethyl fluorescein**	3.12_361.C	POS	3.12	361.0943
**N-(2,4-dimethylphenyl)formamide**	4.943_149.	POS	4.943	149.0843
**Milbemycin beta2**	9.07_558.3	POS	9.07	558.356
**3-oxo-C11-HSL**	3.585_283.	POS	3.585	283.1783
**Lotamilast**	7.744_472.	NEG	7.744	472.1768
**8-hydroxy-11-(hydroxymethyl)-1,5,11-trimethyltri**	8.163_250.	POS	8.163	250.1572
**Mitoflaxone**	1.616_280.	POS	1.616	280.0728
**N-ALPHA-ACETYLLYSINE**	1.052_188.	NEG	1.052	188.1161
**N-[(Z)-1,3-Dihydroxyoctadec-4-en-2-yl]-6-[(4-nit**	8.043_575.	POS	8.043	575.3671
**(4R,5S)-4-Hydroxy-3,4,5-trimethyl-5,6,7,8-tetrahy**	6.298_246.	POS	6.298	246.1257
**Pyranocoumarin**	3.994_322.	POS	3.994	322.1195
**Lexaptepid pegol**	8.673_472.	POS	8.673	472.2167
Other				
**N-butyl-N-(1-(cyclohexylamino)-1-oxobutan-2-yl**	9.889_324.	POS	9.889	324.2776
**Aniline**	2.384_93.C	POS	2.384	93.05801
**Tuberostan**	0.738_348.	POS	0.738	348.0991
**2-Hydroxy-desipramine glucuronide**	5.712_458.	NEG	5.712	458.2064
**Avermectin B1b aglycone;**	7.924_570.	NEG	7.924	570.3217
**Chemistry 2**	8.911_572.	POS	8.911	572.3354
**N-benzyl-2-(N-butylacetamido)-2-(pyridin-3-yl)a**	4.191_424.	NEG	4.191	424.2435
**3-[4-methyl-1-(2-methylpropanoyl)-3-oxocyclohe**	6.816_285.	POS	6.816	285.1939
**PG(18:1(9Z)/0:0)**	8.134_510.	POS	8.134	510.2949
**Ptilosteroid B**	9.803_430.	POS	9.803	430.2018
**Phyllanthin**	8.266_418.	POS	8.266	418.2359
**4-Methoxyphenylacetic acid**	2.372_166.	POS	2.372	166.0631
**PE(20:1(11Z)/0:0)**	9.444_507.	POS	9.444	507.3331
**Kanokoside C;**	3.53_638.2	POS	3.53	638.2405
**Fenoxaprop-ethyl**	5.046_361.	POS	5.046	361.0732
**Betrixaban**	4.779_451.	POS	4.779	451.1416
**SN-38**	3.362_392.	POS	3.362	392.1353
**cinchonine**	7.189_316.	POS	7.189	316.1536
**2-O-alpha-L-rhamnopyranosyl-alpha-L-rhamnopy**	9.415_762.	POS	9.415	762.5125
**L-3-(3-Hydroxy-4-pivaloyloxyphenyl)alanine**	4.191_281.	NEG	4.191	281.1262
**LMST01031065**	9.436_508.	POS	9.436	508.3775
**2-Methoxyestrone**	6.212_300.	POS	6.212	300.1725
**(1S,10S,21R)-7-Ethylidene-4,21-di(propan-2-yl)-2-**	8.23_507.3	POS	8.23	507.3198
**DG(18:1(9Z)/14:0/0:0)**	12.278_56	POS	12.278	566.4919

**1-[2-methyl-6-[(2S,3R,4S,5S,6R)-3,4,5-trihydroxy-(**	6.746_312.	POS	6.746	312.1207
**(3Z,5E)-4-hydroxy-6-(4-hydroxyphenyl)hexa-3,5-c**	5.971_204.	POS	5.971	204.0788
**Dutasteride**	4.867_528.	POS	4.867	528.2233
Piscicoccus_intestinalis				
Pseudomonas_cichorii				
**D-d-Tocopherol**	9.549_402.	POS	9.549	402.3498
**N'-[(4-Oxo-4H-chromen-3-yl)methylene]nicotinol**	0.67_293.C	NEG	0.67	293.0793
**O-acetyl-l-serine**	4.786_129.	POS	4.786	129.0428
**8-Chlorodiltiazem**	4.58_448.1	POS	4.58	448.1243
**7-Deoxyloganetin;**	5.355_212.	POS	5.355	212.1051
**alpha-Methyltyrosine methyl ester**	7.06_209.1	POS	7.06	209.1054
**Pirenperone**	10.2_393.1	POS	10.2	393.184
**Mayolene-20**	12.876_58	POS	12.876	588.5118
**N-Acetylvaline**	0.748_159.	POS	0.748	159.1262
**Roridin A;**	8.218_532.	POS	8.218	532.2656
**PG(15:1(9Z)/0:0)**	8.561_468.	POS	8.561	468.2469
**Butyric acid**	1.655_88.1	POS	1.655	88.10807
**Leptomycin B**	9.192_540.	NEG	9.192	540.3477
**LID_180.1018_12.9**	4.649_179.	POS	4.649	179.0948
**Cycloxydim;**	9.821_325.	POS	9.821	325.1712
**(1R,3S,5S)-8-methyl-8-azabicyclo[3.2.1]octan-3-yl**	2.906_327.	POS	2.906	327.1253
**10,11-Dihydro-12R-hydroxy-leukotriene E4**	6.913_457.	POS	6.913	457.2497
**temephos**	8.033_465.	POS	8.033	465.9889
**Lauroyl diethanolamide**	8.245_287.	POS	8.245	287.2458
**Vitamin D3 glucosiduronate**	9.445_560.	POS	9.445	560.3718
**Quinoline**	4.368_129.	POS	4.368	129.0581
**NCGC00380747-01!7-hydroxy-3-[4-hydroxy-3-(3-**	7.002_428.	POS	7.002	428.1384
**DL-Glutamine**	4.77_146.C	POS	4.77	146.0693
**4-oxo-5-phenylpentanoic acid**	6.837_209.	POS	6.837	209.1054
**Piperacillin**	6.973_517.	POS	6.973	517.1635
**Haematommone**	8.407_314.	NEG	8.407	314.0427
**N-ACETYLMETHIONINE**	3.547_191.	POS	3.547	191.0619
**Ricinine**	1.524_164.	POS	1.524	164.0564
**Pyroglu-Glu-Asp-Ser-GlyOH**	3.393_517.	POS	3.393	517.1675
**2-Ethenyl-1-oxidopyridin-1-ium**	3.792_121.	POS	3.792	121.0528
**Staurosporine, from Streptomyces species**	4.894_466.	POS	4.894	466.1987
**2'-Deoxyuridine-5-monophosphate**	3.774_308.	NEG	3.774	308.0355
Phreatobacter_oligotrophus				
Clostridium_viride				
**Arformoterol**	7.935_344.	POS	7.935	344.1734
**(1R,19R,20S)-19-ethenyl-18-{[(2S,3R,4S,5S,6R)-3,4,**	7.591_498.	POS	7.591	498.2009
**2-(3'-Methylthio)propylmalic acid; 2-(3'-Methylthi**	3.39_222.C	POS	3.39	222.0566
**Diethyl maleate**	5.607_172.	POS	5.607	172.0738
**Cyromazine**	5.25_166.C	POS	5.25	166.0996
**5,6,7,8-Tetrahydrofolic acid**	3.554_445.	POS	3.554	445.1714
**Phenylalanine deoxycholic acid**	9.006_539.	POS	9.006	539.3623
**ginkgolide B**	5.512_424.	NEG	5.512	424.1371
**L-Alanyl-L-proline**	0.749_186.	POS	0.749	186.1007
**Asperitaconic acid A**	5.97_244.1	POS	5.97	244.1311
**3-((2'-(Benzoylaminosulfonyl)biphenyl-4-yl)methy**	4.337_524.	POS	4.337	524.1875
**OON-PS**	8.601_677.	NEG	8.601	677.392
**4-Amino-2,2,6,6-tetramethylpiperidin-1-ol**	7.031_172.	POS	7.031	172.1578
**2-Benzoyloxycinnamaldehyde**	1.328_252.	POS	1.328	252.078
**Testosterone glucuronide**	6.73_464.2	NEG	6.73	464.241
**andrastin A**	8.196_508.	POS	8.196	508.2414
**2-methylene-4-oxo-pentanedioic acid**	0.655_158.	NEG	0.655	158.0216
**DG(18:4(6Z,9Z,12Z,15Z)/20:2(11Z,14Z)/0:0)**	12.442_64	POS	12.442	640.5046
**1,6-dihydroxy-8-(hydroxymethyl)-4,12,12,15-tetra**	8.193_532.	POS	8.193	532.3028

**Quinethazone**	3.44_289.0	POS	3.44	289.0288
**1-Hydroxy-2-(N-acetylcysteinyl)-3-butene**	4.027_233.	NEG	4.027	233.0715
**4,4'-Diapolycopen-4-al;**	9.473_414.	POS	9.473	414.2917
**Glycerylphosphorylcholine**	0.716_257.	POS	0.716	257.1028
**N-Acetyl-DL-tryptophan**	3.784_246.	NEG	3.784	246.1
Variovorax_ginsengisoli				
**NCGC00347585-02!(1,9,9b-trihydroxy-6,6,9a-trime**	4.569_420.	POS	4.569	420.1932
**Carnitine (Dl) Hydrochloride**	0.662_197.	NEG	0.662	197.0816
**4-imidazoleacetic acid**	5.917_126.	POS	5.917	126.043
**Decenoylcarnitine**	5.723_313.	POS	5.723	313.2251
**Turkesterone 22-acetate**	8.094_538.	POS	8.094	538.3152
Massilia_namucuonensis				
Tenacibaculum_aestuarii				
Other				
**8-Caffeoyl-3,4-dihydro-5,7-dihydroxy-4-phenylco**	6.316_418.	POS	6.316	418.1063
**delta9-Tetrahydrocannabinol**	6.64_314.2	POS	6.64	314.2243
**Methylglucamine**	0.926_195.	NEG	0.926	195.1107
**Nitracrine**	5.864_324.	POS	5.864	324.1573
**Mozenavir**	8.268_536.	NEG	8.268	536.2802
**Neolinderatin**	9.266_530.	POS	9.266	530.3398
**6-Hydroxyetodolac**	5.45_303.1	POS	5.45	303.1473
**PC(16:1(9E)/0:0)**	9.246_493.	POS	9.246	493.3171
**N-Methylhydantoin**	0.964_132.	POS	0.964	132.0537
**7-Hydroperoxycholesterol**	8.829_418.	POS	8.829	418.3448
**Nilotinib**	8.144_529.	POS	8.144	529.1854
**Exatecan**	5.737_435.	POS	5.737	435.1578
**isopropyl unoprostone**	10.323_42	POS	10.323	424.3168
**N3-Fumaroyl-L-2,3-diaminopropanoate; N3-Fuma**	5.834_202.	POS	5.834	202.0597
Clostridium_hylemonae				
**Fenoldopam**	4.876_305.	POS	4.876	305.0833
**Pentanedioic acid, 3-((((3R,5R)-3-butyl-3-ethyl-2,3**	7.319_546.	NEG	7.319	546.2424
**LMST04040005**	8.762_462.	POS	8.762	462.3348
**Glycyrrhizin**	9.654_839.	POS	9.654	839.4299
**Spiropiperidine**	7.906_525.	NEG	7.906	525.3115
**(2R,3S,4S,5R,6R)-2-(hydroxymethyl)-6-[(E)-3-(4-hy**	4.341_312.	NEG	4.341	312.1209
Other				
**3-Hydroxy-3-methylpentanedial**	7.221_130.	POS	7.221	130.0631
**Succinobucol**	8.675_616.	POS	8.675	616.3258
Prevotella_oralis				
**PA(17:2(9Z,12Z)/12:0)**	9.633_602.	NEG	9.633	602.3969
**Alaptide**	2.469_182.	POS	2.469	182.1058
**4-hydroxy-8-methoxy-5-methyl-3-(3-methylbut-2**	4.184_296.	POS	4.184	296.1011
**3,4,5,4'-Tetramethoxystilbene**	6.722_300.	NEG	6.722	300.136
**Indoxyl glucuronide**	4.829_309.	POS	4.829	309.0848
**Cbhcy**	1.953_235.	NEG	1.953	235.0877
**15(R)-Prostaglandin D2**	7.92_334.2	POS	7.92	334.2123
**Medroxalol**	7.919_372.	POS	7.919	372.1684
**Diheptyl phthalate**	7.712_362.	POS	7.712	362.2467
**Aflatoxin G2**	4.025_330.	NEG	4.025	330.0774
**Ethyl 2-[2-(diethylamino)ethoxy]-1-phenylcyclope**	9.325_333.	POS	9.325	333.2305
**Milbemycin D**	9.415_556.	POS	9.415	556.34
**[(2R)-Oxiran-2-yl]methyl 2-methylprop-2-enoate**	4.193_142.	POS	4.193	142.063
**2-[(3S)-3-[[(2S)-1-(Carboxymethoxy)-1-oxo-4-ph**	7.886_454.	POS	7.886	454.1739
**3,5-Dinitro-4-hydroxyphenylpyruvate;**	3.358_270.	POS	3.358	270.0135
**4-(N,N-Dimethylsulfamoyl)-7-hydrazino-benzofur**	3.032_257.	POS	3.032	257.0577
**Pimozide**	3.451_461.	POS	3.451	461.2279
**Methylimidazoleacetic acid**	0.739_140.	POS	0.739	140.0588
**12-Hydroxydodecanoic acid**	8.088_216.	NEG	8.088	216.1725

**5beta-Pregnane-3alpha,20alpha-diol**	10.286_32(	POS	10.286	320.2714
**Olmesartan**	2.843_446.	POS	2.843	446.2088
**[4-[3,4,5-trihydroxy-6-(hydroxymethyl)oxan-2-yl]**	0.71_536.1	POS	0.71	536.1883
**Lexacalcitol**	10.356_46(	POS	10.356	460.3557
**OOB-PA**	6.017_520.	POS	6.017	520.2814
**2-[4-(2-Carboxy-2-methylpropyl)phenyl]propionic**	8.634_250.	POS	8.634	250.1207
**Methylprednisolone**	6.909_374.	POS	6.909	374.2091
**(6S,3'S,5'R,6'R)-6,3',5'-Trihydroxy-4,5,6',7'-tetradel**	9.86_614.3	POS	9.86	614.3992
**N-(4-(4-Methyl-1-piperazinyl)phenyl)-9-acridinan**	9.251_368.	POS	9.251	368.1987
**trichloroethyl**	0.943_130.	POS	0.943	130.9227
Thermobifida_cellulosilytica				
**3Beta-Hydroxy-23,24-Bisnorchol-5-Enic Acid**	9.807_368.	POS	9.807	368.2328
**Tilsuprost**	7.067_383.	POS	7.067	383.2133
**N-(1-Hydroxyethyl)octadecanamide**	9.027_327.	POS	9.027	327.3137
**Anthra(1,9-cd)pyrazol-6(2H)-one, 2-(2-((2-hydrox**	5.797_409.	NEG	5.797	409.2133
**zardaverine**	0.74_268.0	POS	0.74	268.067
**ibho#18**	4.591_491.	POS	4.591	491.2525
**N-(2-Hydroxyphenyl)acetamide glucuronide**	3.312_329.	POS	3.312	329.0749
**PZ-Peptide**	10.956_77(	POS	10.956	776.3957
**Celestone**	6.925_392.	POS	6.925	392.2001
**(4E,6E)-2-(hydroxymethyl)-3-(3-methoxy-3-oxopr**	4.815_301.	POS	4.815	301.1889
**Amphetamine**	5.734_135.	NEG	5.734	135.1049
**5-Hydroxypyrazine-2-carboxylic acid**	3.245_140.	POS	3.245	140.0227
**Dibenzo-P-dioxin**	7.9_402.17	POS	7.9	402.1791
**Cholesta-4,6-dien-3-one**	8.886_382.	POS	8.886	382.3236
Ornithinimicrobium_murale				
Nitrosospira_lacus				
Other				
Psychrobacter_maritimus				
**Ramipril**	9.663_416.	NEG	9.663	416.2332
**NCGC00170014-03!(E)-N-(4-acetamidobutyl)-3-(4**	5.386_634.	POS	5.386	634.2979
**1,1,2-Trimethylcyclohexane**	10.289_12(	POS	10.289	126.141
**D-RIBOSE 5-PHOSPHATE**	0.649_230.	NEG	0.649	230.0192
**Galactocerebroside**	9.847_505.	POS	9.847	505.3233
Planifilum_fulgidum				
**N-stearoyl taurine**	7.524_391.	POS	7.524	391.2756
**Nalidixic Acid**	9.428_232.	POS	9.428	232.0842
**Rphdhd**	8.62_504.3	NEG	8.62	504.3292
**Americine;**	7.932_545.	POS	7.932	545.3003
**6**	3.667_343.	POS	3.667	343.1743
**butane-1,4-disulfonic acid**	0.635_217.	NEG	0.635	217.9918
**Vanillic acid 4-O-sulfate**	1.758_247.	NEG	1.758	247.9988
**Indicumenone;**	7.977_252.	POS	7.977	252.1726
**4,4'-Diaminodiphenyl ether; 4-Aminophenyl ether;**	5.338_200.	POS	5.338	200.0953
Brucella_melitensis				
**4-hydroxybenzoate**	3.476_120.	POS	3.476	120.0212
**eurysterol A sulfonic acid**	7.979_514.	POS	7.979	514.2972
**Phosphodimethylethanolamine;**	0.663_169.	NEG	0.663	169.0505
**[4,5,8-trihydroxy-5-(hydroxymethyl)-3-(3-methox**	7.536_466.	POS	7.536	466.1793
**Propafenone glucuronide**	8.136_517.	POS	8.136	517.2316
Other				
**Pyrene-4,5-dione; 4,5-Pyrenequinone;**	2.9_232.05	POS	2.9	232.0519
**C17 Sphingosine**	8.295_285.	POS	8.295	285.2668
**(2Z)-2-[(E)-6-(hydroxymethyl)-2,4,8,10-tetramethy**	8.417_413.	POS	8.417	413.314
**Exodeconolide F**	8.066_454.	POS	8.066	454.2698
**Diethylaminoethyl-Sephacel**	8.763_378.	POS	8.763	378.2745
**Diftalone**	4.577_264.	POS	4.577	264.0911
**Valylproline**	2.69_214.1	POS	2.69	214.1318

**1-O-Hexadecyl-2-arachidonoyl-sn-glycero-3-pho**	11.869_76	POS	11.869	767.5824
**5,16-Androstadien-3beta-ol**	8.235_272.	POS	8.235	272.214
**FR901463**	10.809_54	POS	10.809	543.2618
**N2-Citryl-N6-acetyl-N6-hydroxy-L-lysine; N2-Citr**	3.857_378.	POS	3.857	378.1287
**Cholesterol sulfate**	8.192_466.	POS	8.192	466.3128
**1-(2-methoxy-octadecanyl)-sn-glycero-3-phosph**	9.908_541.	NEG	9.908	541.3371
**PGF2alpha-11-acetate**	7.807_396.	NEG	7.807	396.2512
**Telmisartan**	6.973_514.	POS	6.973	514.2377
**Norcapillene;**	8.061_140.	POS	8.061	140.0627
**bilobol**	10.136_31	POS	10.136	318.2557
**Silodosin**	8.111_495.	NEG	8.111	495.2368
**Asteriidoside L**	8.298_676.	NEG	8.298	676.3504
Howardella_ureilytica				
Eubacterium_ruminantium				
**(+)-Prosopinine;**	7.539_313.	POS	7.539	313.2616
**Stearic acid**	9.095_284.	POS	9.095	284.2715
**26,26,26,27,27,27-hexafluoro-25-hydroxy-16,17,23**	6.41_502.2	POS	6.41	502.2324
Other				
**Damme**	7.096_603.	NEG	7.096	603.2717
**(6R)-vitamin D3 6,19-sulfur dioxide adduct / (6R)-**	8.211_448.	POS	8.211	448.3021
**3,3-Bis(chloromethyl)oxetane**	1.846_153.	POS	1.846	153.9958
**Triamcinolone**	4.597_394.	POS	4.597	394.1776
**Cer(d18:0/14:0)**	9.467_511.	POS	9.467	511.4972
**Roxithromycin**	7.009_836.	POS	7.009	836.5237
**dapsone hydroxylamine**	3.916_264.	POS	3.916	264.0563
**Acitretin**	3.898_326.	POS	3.898	326.1879
**Methyl selenac; Tetrakis(dimethyldithiocarbamato)**	0.784_559.	NEG	0.784	559.8949
**3-OH-C10-HSL**	4.805_271.	POS	4.805	271.1783
**Diisopropanolamine**	9.964_133.	POS	9.964	133.1104
**L-Aspoxicillin trihydrate**	9.246_493.	POS	9.246	493.1639
**butenoylcarnitine**	2.568_229.	POS	2.568	229.1315
**1,7-bis(4-hydroxyphenyl)heptan-3-one**	5.745_336.	POS	5.745	336.1143
**Hydrobromic acid; HBr;**	0.666_79.9	NEG	0.666	79.92613
**16-epi-pretoxin**	5.825_380.	POS	5.825	380.1472
**(2S,3R,4S,5S,6R)-2-[4-(3-hydroxybutyl)phenoxy]-6**	2.577_366.	POS	2.577	366.1095
**1,25-dihydroxy-2,4-dinor-1,3-secovitamin D3 / 1,2**	9.752_390.	NEG	9.752	390.3136
**4-Methylabyssinone V**	5.766_422.	NEG	5.766	422.2089
Exiguobacterium_sibiricum				
**Itopride**	8.431_358.	NEG	8.431	358.1908
Paenibacillus_validus				
**1-(18-mercaptooctadecanoyl)-sn-glycerol 3-phosp**	3.567_470.	POS	3.567	470.2485
**alpha-GlcADAG(18:0(10Me)/16:0)**	10.368_78	POS	10.368	786.5858
**Isomigrastatin;**	9.247_489.	POS	9.247	489.271
**E-10-Hydroxydesmethylnortriptyline**	5.795_265.	POS	5.795	265.1464
**9-hydroxy-7-(2-hydroxypropan-2-yl)-1,4a-dimeth**	5.783_370.	POS	5.783	370.1561
**FLU_410.1574_18.4**	3.381_409.	POS	3.381	409.1486
**2-trans-Hydroxycyclohexyl glyburide**	6.546_509.	POS	6.546	509.1393
**4-[2-[(2-ethyl-2,3-dihydroxybutanoyl)oxymethyl]a**	3.228_370.	POS	3.228	370.174
**5alpha-Cyprinol**	10.016_45	NEG	10.016	452.3501
**Mmaf**	8.876_731.	NEG	8.876	731.4815
**Furaltadone**	2.875_324.	POS	2.875	324.1085
**PE(0:0/20:1(11Z))**	9.992_507.	NEG	9.992	507.3324
**Fenitrothion; O,O-Dimethyl O-(3-methyl-4-nitroph**	0.724_277.	POS	0.724	277.0183
**PA(18:0/0:0)**	10.191_43	POS	10.191	438.2747
**10,13-Dimethyl-7-sulfanylspiro[2,6,7,8,9,11,12,14,1**	5.439_374.	POS	5.439	374.1919
**27-Norcholestanehexol**	8.791_454.	POS	8.791	454.3297
**20-Trihydroxy-leukotriene-B4**	7.058_384.	POS	7.058	384.2148
Terrisporobacter_glycolicus				

**Putrescine**	0.588_88.1	POS	0.588	88.10009
**Monoisononyl phthalate**	8.233_292.	NEG	8.233	292.1673
**VERBENONE**	10.15_150.	POS	10.15	150.1047
**Megalomicin C1;**	7.87_960.5	NEG	7.87	960.5775
**6-Fluoropyridoxal**	3.546_185.	POS	3.546	185.048
**L-3-Hydroxykynurenine**	3.856_224.	POS	3.856	224.0798
**Glutamate carbon**	3.165_302.	POS	3.165	302.0759
**N-geranyl-L-glutamic acid**	4.844_283.	POS	4.844	283.1782
**Aldosterone 18-glucuronide**	3.384_536.	POS	3.384	536.224
**DG(12:0/18:3(9Z,12Z,15Z)/0:0)[iso2]**	10.056_53	POS	10.056	534.4293
**[(2R,3S,4S,5R,6R)-3,4,5-trihydroxy-6-[2-(4-hydrox**	9.426_495.	POS	9.426	495.1483
**Cer(d18:0/23:0)**	12.623_63	POS	12.623	637.6373
**24-(dimethoxyphosphoryl)-25,26,27-trinorvitamin**	9.816_450.	NEG	9.816	450.2919
**Ethoprophos**	4.73_242.0	POS	4.73	242.0556
**Calcium levulinate anhydrous;**	3.558_270.	POS	3.558	270.0424
**Amodiaquine**	9.631_355.	POS	9.631	355.1468
**(4R,7S,10R)-d5-8-NeuroF[8S,11S]**	7.589_394.	POS	7.589	394.2355
**phenolic phthiocerol**	12.798_57	POS	12.798	576.5123
**S-Succinylglutathione;**	3.362_407.	POS	3.362	407.0998
Other				
**Paliperidone**	7.056_426.	NEG	7.056	426.2076
**OG-PA**	3.534_550.	POS	3.534	550.2898
**Acalabrutinib**	6.508_465.	NEG	6.508	465.19
**Acetic acid, 2-(4-((5-methoxy-2-(((4-methoxy-3,5-**	5.043_559.	POS	5.043	559.1076
Bosea_thiooxidans				
**NCGC00384910-01!4,6,7-trihydroxy-5-methoxy-1,**	9.234_356.	POS	9.234	356.1284
**Glycoursodeoxycholic acid**	7.677_449.	POS	7.677	449.3139
**Alpha-Acarbose**	6.713_645.	NEG	6.713	645.2507
**Roquefortine C**	5.7_389.18	POS	5.7	389.1871
**Niraxostat**	5.555_299.	POS	5.555	299.1269
Allisonella_histaminiformans				
**N-desmethylrosiglitazone**	5.651_343.	NEG	5.651	343.099
**Mupirocin**	7.799_500.	POS	7.799	500.3007
**10,11-Dihydroxycarbamazepine**	6.871_270.	POS	6.871	270.1004
**N-Acetyl-D-alloisoleucine**	3.235_173.	NEG	3.235	173.1053
**16alpha,17alpha-epoxy-sinusta-5-en-3beta**	9.003_524.	NEG	9.003	524.3169
**[4-(3-Benzyl-4-hydroxybenzyl)-3,5-dimethylphen**	5.115_376.	POS	5.115	376.167
**Digitoxigenin bisdigitoxide**	8.685_634.	POS	8.685	634.3695
**1,7-Diphenyl-4-hepten-3-one**	9.915_286.	POS	9.915	286.1334
**Didesmethyl tocotrienol**	8.723_368.	POS	8.723	368.2715
**Kanokoside D;**	6.423_624.	NEG	6.423	624.2645
**Ignavine;**	5.429_449.	POS	5.429	449.2185
**2-(4-(2-Methylpyridin-4-yl)phenyl)-N-(4-(pyridin-**	5.801_379.	NEG	5.801	379.1666
**L-Pyroglutamyl-L-histidyl-3,3-dimethylprolinamid**	7.472_390.	POS	7.472	390.2018
Pasteurella_pneumotropica				
Other				
Novosphingobium_naphthalenivorans				
Coraliomargarita_akajimensis				
Helicobacter_cinaedi				
Bacteroides_sartorii				
Rikenella_microfusus				
Other				
Prevotella_timonensis				
Other				
Helicobacter_marmotae				
Deinococcus_antarcticus				
Sporosarcina_koreensis				
Brevibacillus_laterosporus				

**Isorhamnetin 7-alpha-D-Glucosamine**	5.48_477.1	POS	5.48	477.1275
**Amonafide**	5.191_283.	NEG	5.191	283.1319
**Dihydrotanshinone**	3.236_278.	POS	3.236	278.0935
**Lamioside;**	3.505_420.	POS	3.505	420.1644
**[3,4,5-trihydroxy-6-(hydroxymethyl)oxan-2-yl] 2,4**	4.315_435.	POS	4.315	435.153
**Phenidone**	2.98_162.C	NEG	2.98	162.0794
**2'-Dehydrokanamycin A; 2'-Oxokanamycin;**	5.801_482.	POS	5.801	482.2204
**Phenylalanine glutamate**	4.951_310.	POS	4.951	310.1165
**N-oleoyl threonine**	8.162_383.	POS	8.162	383.3034
**Avocadene 1-acetate**	9.968_328.	NEG	9.968	328.2614
**N-[(2S)-5-[(1-Amino-2-chloroethylidene)amino]-1**	10.306_49	POS	10.306	493.222
**Prebarbigerone**	4.664_410.	POS	4.664	410.1723
**Methysticin**	6.695_274.	POS	6.695	274.0842
**18:2-Glc-cholesterol**	9.33_810.6	POS	9.33	810.6375
**Vatinoxan**	4.787_418.	NEG	4.787	418.1661
**Heptyl p-hydroxybenzoate; Heptylparaben;**	7.563_236.	POS	7.563	236.1414
**Sulforaphane**	4.58_177.C	POS	4.58	177.0286
**Brasofensine**	4.02_326.C	POS	4.02	326.0937
**Taurodehydrocholic acid**	7.317_509.	NEG	7.317	509.2446
**Cer(t18:1(6OH)/23:0(2OH))**	11.046_66	NEG	11.046	667.6129
**13-Methyl-7-[9-(4,4,5,5,5-pentafluoropentylsulfiny**	8.85_606.3	POS	8.85	606.3176
**Methsuximide**	5.772_203.	POS	5.772	203.0949
**isoxicam**	5.395_335.	POS	5.395	335.0579
**NCGC00347672-02!N-[4,5-dihydroxy-2-[[4-[hydro**	5.181_537.	POS	5.181	537.1424
Other				
**Kenacourt**	7.751_478.	POS	7.751	478.1986
**3-Ethyl-1,2-benzenediol**	5.38_138.C	POS	5.38	138.0683
**2-glyceryl-PGF2alpha**	8.315_428.	POS	8.315	428.2762
**Posaconazole**	7.104_700.	POS	7.104	700.3305
**beta-Cortol**	9.098_368.	POS	9.098	368.2563
**Phenobenzuron;**	0.67_336.C	NEG	0.67	336.0419
**3-Octaprenyl-4-hydroxybenzoate; 4-Hydroxy-3-oc**	9.806_682.	POS	9.806	682.5356
**Butoctamide hydrogen succinate; Butoctamide sem**	3.35_315.2	POS	3.35	315.2046
**Roccellic acid**	9.192_300.	POS	9.192	300.23
Holdemania_filiformis				
**Desogestrel**	10.083_31	POS	10.083	310.2296
**LMSL05000005**	8.887_690.	NEG	8.887	690.4557
**N-(2,6-Dimethylphenyl)-N-(methoxyacetyl) alanine**	6.671_265.	POS	6.671	265.1313
**2-Methylbutyrylglycine**	2.235_159.	POS	2.235	159.0898
**Nonoxinol**	9.389_264.	POS	9.389	264.209
**Carpaine;**	8.993_478.	POS	8.993	478.3777
**2,3,9,10-tetramethoxy-6a,11a-dihydro-6H-[1]benz**	4.84_393.1	POS	4.84	393.1421
**N-[1-(4-methoxy-6-oxopyran-2-yl)-2-methylbuty**	6.241_270.	POS	6.241	270.1579
**Diacetyl**	3.493_172.	POS	3.493	172.074
**Turofexorate isopropyl**	3.884_438.	POS	3.884	438.1745
**4-[[4-(4-Chlorophenyl)-2-thiazolyl]amino]phenol**	0.639_302.	NEG	0.639	302.027
**(E)-1-(3,4-dihydroxyphenyl)-7-(4-hydroxyphenyl)h**	4.129_167.	POS	4.129	167.0584
**Chrysanthemic Acid**	6.53_168.1	NEG	6.53	168.1151
**Coumatetralyl**	2.707_292.	NEG	2.707	292.1092
**2-Hydroxymyristoylcarnitine**	8.005_387.	POS	8.005	387.2982
**6-beta-D-Glucopyranosyl-8-beta-D-ribopyranosyl**	4.038_564.	NEG	4.038	564.1483
Other				
**Cer(t18:1(6OH)/14:0)**	10.429_52	POS	10.429	525.4765
**5beta-scymnol sulfate**	9.33_548.3	POS	9.33	548.3033
**OOV-PS**	4.58_621.3	POS	4.58	621.3277
**Propylparaben**	3.431_180.	NEG	3.431	180.0787
**D-Kynurenine**	1.426_246.	POS	1.426	246.0417
**Taranabant**	5.479_515.	POS	5.479	515.1584

Eubacterium_cellulosolvens				
**2,6-Dichlorophenolindophenol sodium salt; 2,6-Dic**	4.849_290.	POS	4.849	290.0769
**Kaempferol 7,4'-dimethyl ether 3-O-sulfate**	6.72_394.0	NEG	6.72	394.0359
**5-(N-Hexadecanoyl)aminofluorescin**	7.533_585.	POS	7.533	585.3087
**1-[2-hydroxy-6-methyl-4-[(2S,3R,4S,5S,6R)-3,4,5-**	5.525_328.	POS	5.525	328.1174
**Isorhynchophylline**	8.923_384.	POS	8.923	384.2043
**3-Iodothyroacetic acid**	0.786_369.	NEG	0.786	369.9688
Other				
**Petasitenine;**	6.821_381.	POS	6.821	381.1785
**Delapril**	5.44_452.2	POS	5.44	452.2294
Fusobacterium_nucleatum				
**Glycerophospho-N-Arachidonoyl Ethanolamine**	9.388_501.	POS	9.388	501.2863
**Diketospirilloxanthin/ 2,2'-Diketospirilloxanthin**	4.86_624.4	POS	4.86	624.4196
**2-(6-(5-Chloro-2-methoxyphenyl)-4-oxo-2-thioxo**	3.561_325.	POS	3.561	325.0302
**DG(12:0/20:4(5Z,8Z,11Z,14Z)/0:0)[iso2]**	10.337_56(	POS	10.337	560.4445
**Diazepine**	2.965_94.0	POS	2.965	94.05319
**Benzyl phenyl ether**	8.049_184.	POS	8.049	184.089
**Mayolene-19**	12.368_57	POS	12.368	574.4965
**(E)-3-Tosylacrylonitrile**	2.589_207.	POS	2.589	207.0357
**Prolylleucine**	2.994_228.	POS	2.994	228.1475
**1-(1,3-Benzodioxol-5-ylcarbonyl)piperidine**	6.791_233.	POS	6.791	233.1052
**Moxifloxacin**	4.829_401.	POS	4.829	401.1746
**PHOSPHORYLCHOLINE**	9.464_183.	POS	9.464	183.0662
**6-Fluorohomovanillic acid**	7.539_200.	POS	7.539	200.0493
**NCGC00180705-02!(E)-5-hydroxy-1,7-diphenylhep**	6.38_299.1	POS	6.38	299.119
**PA(O-18:0/0:0)**	9.581_424.	POS	9.581	424.2955
**3,4-Epoxy-6Z,9Z-eicosadiene**	8.766_292.	POS	8.766	292.2766
**LysoPC(18:1(9Z)/0:0)**	9.626_521.	POS	9.626	521.3485
**Sulfolithocholic acid**	6.912_456.	POS	6.912	456.2525
**PKOOA-PA**	5.853_562.	POS	5.853	562.2904
**methyl 3-(3,4-dihydroxy-5-phenyloxolan-2-yl)-3-**	3.92_299.1	POS	3.92	299.1367
Micrococcus_aloeverae				
**3''-Deamino-3''-oxonicotianamine;**	3.046_302.	NEG	3.046	302.1113
**Hydroxyl frovatriptan**	4.886_261.	POS	4.886	261.1477
**6-[4-(2-carboxyethyl)phenoxy]-3,4,5-trihydroxyox**	3.211_342.	NEG	3.211	342.095
**Cancentrine;**	6.123_606.	NEG	6.123	606.2349
**LMST01031102**	8.258_474.	POS	8.258	474.3346
**Khivorin**	8.647_586.	POS	8.647	586.2796
**methyl 2-[4-ethenyl-2,6-dihydroxy-3-(3-hydroxyp**	8.599_296.	POS	8.599	296.1624
**CC(=O)Ncccccn1C=CC(=O)C(O)=C1C**	5.418_266.	POS	5.418	266.1631
**5Alpha-Cholestan-3Beta-Ol-6-One**	8.429_402.	POS	8.429	402.3496
**Pyroglutamyl-phenylalanyl-prolinamide**	7.459_372.	POS	7.459	372.1796
**[(2R,3R,4S,5S,6R)-2-(acetyloxymethyl)-3,5-dihydro**	6.277_462.	POS	6.277	462.1505
**5,10,15,20-Tetrakis(4-methoxyphenyl)-21H,23H-po**	7.094_734.	NEG	7.094	734.2866
**LMST04010421**	8.248_424.	POS	8.248	424.2828
Bdellovibrio_exovorus				
Other				
**N-oleoyl taurine**	9.238_389.	POS	9.238	389.2601
**Indoxyl-beta-D-glucoside**	3.471_295.	POS	3.471	295.1058
**LMST01160025**	7.73_488.2	POS	7.73	488.2753
**1-(O-alpha-D-glucopyranosyl)-3-keto-(1,25R,27R)**	9.385_618.	POS	9.385	618.4716
**[(1S,4aS,5R,7S)-4a,5-dihydroxy-7-methyl-1-[(2S,3I**	3.856_510.	POS	3.856	510.1717
**Corticatic acid A**	9.55_464.3	POS	9.55	464.3293
**5-(Ethylthio)-1H-tetrazole**	3.172_130.	POS	3.172	130.0315
**Adamantylamide-alanyl-isoglutamine**	3.394_350.	POS	3.394	350.2318
**LMST01080087**	9.108_594.	POS	9.108	594.3773
**THIOPHENE-2-CARBOXYLIC ACID**	2.685_127.	NEG	2.685	127.9933
**Mebendazole**	3.216_295.	POS	3.216	295.0974

**Pantetheine; (R)-Pantetheine;**	10.19_278.	NEG	10.19	278.1299
**Bisbenzimide**	8.032_452.	POS	8.032	452.2347
**Deoxycorticosterone 21-glucoside**	7.388_492.	NEG	7.388	492.2721
**Kanamycin**	8.195_506.	POS	8.195	506.2297
**Menthol glucuronide**	2.772_332.	POS	2.772	332.185
Ochrobactrum_pseudogrignonense				
**Olradipine**	3.524_486.	POS	3.524	486.1346
**Withanoside XI**	7.088_636.	POS	7.088	636.3522
**Hydrocortamate**	8.191_475.	POS	8.191	475.2937
**Fusarin C;**	3.446_431.	POS	3.446	431.1947
**4-[(S)-((2R,5S)-4-Allyl-2,5-dimethyl-piperazin-1-y**	8.009_449.	POS	8.009	449.3049
**3-Pyridinol**	0.792_95.0	POS	0.792	95.03724
**Loratadine**	5.55_382.1	NEG	5.55	382.1451
**Cotylenin F;**	8.705_626.	NEG	8.705	626.3683
**Butabarbital**	1.511_212.	POS	1.511	212.1162
**Buclizine**	7.933_432.	POS	7.933	432.2344
**Polylysine**	1.181_128.	POS	1.181	128.0952
**(2R,3R,4R,5R,6S)-2-[[(2R,3S,4S,5R,6R)-6-[1,7-bis(4-**	8.398_630.	POS	8.398	630.2689
**Am-PE(18:0/22:6(4Z,7Z,10Z,13Z,16Z,19Z))**	8.03_953.6	POS	8.03	953.603
**PSEUDO-ANISATIN**	7.934_298.	POS	7.934	298.1415
**2-hydroxy-4-(hydroxymethyl)-6-(1-hydroxy-3-me**	7.934_280.	POS	7.934	280.131
**Lixivaptan**	4.677_473.	NEG	4.677	473.1288
**Catechin 5-O-(2-feruloyl-6-p-coumaroyl-beta-D-**	6.581_774.	NEG	6.581	774.2139
**Imibenconazole;**	5.197_409.	NEG	5.197	409.9947
**Mesulergine**	8.822_362.	POS	8.822	362.1794
**Cyclo[(2Z)-2-amino-2-butenoyl-L-valyl-(3S,4E)-3-**	2.891_540.	POS	2.891	540.2077
**Calactin;**	7.896_532.	NEG	7.896	532.2695
**3-hydroxy-6-[[(E)-3-hydroxy-2,4-dimethylhept-4-**	4.842_343.	POS	4.842	343.1993
**Philanthotoxin;**	9.606_435.	POS	9.606	435.3197
**N-myristoyl glutamine**	9.303_356.	POS	9.303	356.2672
**Ridogrel**	5.351_366.	POS	5.351	366.1173
**Bay-41-8543**	8.549_420.	POS	8.549	420.1806
**Pyridoxal**	1.275_167.	NEG	1.275	167.0583
**Eugenitin**	4.736_220.	POS	4.736	220.0737
**isosakuranetin**	7.418_286.	NEG	7.418	286.084
**7-O-Methyltectorigenin 4'-O-gentiobioside**	4.601_638.	NEG	4.601	638.1848
Methylobacterium_aerolatum				
**Neoline**	8.925_437.	NEG	8.925	437.2779
**DL-Dipalmitoylphosphatidylcholine**	11.97_733.	POS	11.97	733.5624
**7-hydroxymitragynine**	9.804_414.	POS	9.804	414.2294
**steganacin**	2.871_473.	POS	2.871	473.1691
**N-coronafacoyl-valine**	5.468_307.	POS	5.468	307.1782
**Pamaqueside**	8.01_754.4	NEG	8.01	754.4174
**Atreleuton**	4.186_318.	POS	4.186	318.0827
**Glutathione**	9.576_307.	POS	9.576	307.0837
**Methyl-2-aminobenzoate**	9.956_151.	POS	9.956	151.0635
**Tanespimycin**	7.053_567.	NEG	7.053	567.2869
**Herniarin**	6.984_176.	POS	6.984	176.0476
**(-)-Camphanic acid**	2.671_198.	NEG	2.671	198.0892
**norlichexanthone**	4.847_258.	POS	4.847	258.0536
**Dasatinib**	3.568_487.	POS	3.568	487.1556
**Bisphenol A dimethacrylate**	5.88_364.1	POS	5.88	364.1673
Anoxybacillus_tepidamans				
**Balfourodinium;**	6.918_304.	POS	6.918	304.1536
Other				
**Galnon**	6.825_678.	NEG	6.825	678.3389
**Indomethacin**	5.287_357.	POS	5.287	357.0783
**Cer(d14:2(4E,6E)/20:1(11Z))**	10.766_53:	POS	10.766	533.4817

**2',3'-Cyclic CMP**	4.461_323.	POS	4.461	323.0512
**Durabolin**	11.91_406.	POS	11.91	406.2502
Methylobacterium_tarhaniae				
**PA(16:0/20:5(5Z,8Z,11Z,14Z,17Z))**	4.872_694.	POS	4.872	694.4607
**ANGOLENSIN (R)**	7.56_272.1	POS	7.56	272.1052
**Caryophyllene [T(-)]**	11.608_19	POS	11.608	190.1723
**3-Methylbenzaldehyde; m-Tolualdehyde;**	9.292_120.	POS	9.292	120.0576
Coprobacillus_cateniformis				
**Ketamine**	4.354_237.	POS	4.354	237.0922
**Niludipine**	6.153_490.	NEG	6.153	490.2328
Other				
**6-bromo-tricosa-5E,9Z-dienoic acid**	2.705_428.	NEG	2.705	428.2273
**LysoPC(16:0/0:0)**	9.559_495.	POS	9.559	495.3326
**Cyclo(L-tyrosyl-L-tyrosyl);**	7.513_326.	POS	7.513	326.1264
**3'-C-Ethynylcytidine**	3.941_267.	POS	3.941	267.0857
**6-(furan-3-yl)-12,16,21-trihydroxy-7,15-dimethyl-**	9.429_572.	POS	9.429	572.2596
**LMST01010328**	8.81_484.3	NEG	8.81	484.3399
**S-Ethyl dipropylthiocarbamate**	6.486_189.	POS	6.486	189.119
Salmonella_enterica				
Ralstonia_syzygii				
Terrimicrobium_sacchariphilum				
Other				
**Glutathione bicarbonate**	3.429_367.	POS	3.429	367.0693
**Glutathione monoisopropyl ester**	3.57_349.1	POS	3.57	349.1308
**5'-O-Desmethyl omeprazole**	5.728_331.	NEG	5.728	331.0991
**4'-O-methyl-(-)-epicatechin-3'-O-beta-glucuroni**	3.984_494.	POS	3.984	494.1446
**[5-formyl-4,8-dihydroxy-3-(3-methoxy-3-oxopro**	6.85_464.1	POS	6.85	464.1447
**Bicisate**	3.419_324.	POS	3.419	324.1176
**3-oxo-C13-HSL**	6.683_311.	POS	6.683	311.2095
**PA(16:0/18:1(11Z))**	8.04_674.4	POS	8.04	674.4866
**Isodesmosine**	7.438_526.	NEG	7.438	526.2887
**1,2-Diphenylethane**	8.041_182.	POS	8.041	182.1098
**Leucylphenylalanine**	5.402_278.	NEG	5.402	278.1629
**Ankorine;**	6.337_335.	POS	6.337	335.2095
**18-**	9.479_470.	POS	9.479	470.3399
**1-Oxononan-4-yl (2R)-2-acetamido-3-sulfanylpro**	6.915_303.	POS	6.915	303.1501
**CDP**	0.72_403.0	NEG	0.72	403.0191
**PC(20:3(5Z,8Z,11Z)/16:1(9Z))**	11.455_78	POS	11.455	781.5613
**1-[4,5-bis(hydroxymethyl)-3-methoxy-2-methylpl**	3.915_317.	POS	3.915	317.1834
**Thiamin monophosphate; Thiamine monophosphat**	6.964_345.	POS	6.964	345.0784
**(2R)-2-Acetamido-3-[[(2R)-2-acetamido-2-carbox**	3.968_324.	POS	3.968	324.0446
**Medicagenic acid 3-O-triglucoside;**	8.482_988.	POS	8.482	988.4836
**21-Hopen-3-on**	9.411_442.	POS	9.411	442.3814
**Anethole**	8.507_148.	POS	8.507	148.089
**Epinephrine**	4.026_183.	POS	4.026	183.0896
**Benzil**	6.976_210.	POS	6.976	210.0683
**Dihydroxy (3Alpha,12Alpha)Pregnan-20-One**	9.447_334.	POS	9.447	334.2508
**N-Despropyl-rotigotine**	9.247_273.	POS	9.247	273.1196
**1,7-Bis(4-hydroxy-3-methoxyphenyl)-1,4,6-heptat**	3.176_352.	POS	3.176	352.1305
**2-deoxyecdysone 22-phosphate**	8.568_528.	POS	8.568	528.2856
**bhos#34**	8.581_460.	POS	8.581	460.3415
**Leoligin**	9.556_470.	POS	9.556	470.2282
**dTDP-4-oxo-2,6-dideoxy-L-allose;**	4.367_530.	NEG	4.367	530.0728
**Acetoxolone**	9.406_512.	POS	9.406	512.3488
**(S)-7-(3-Amino-1-pyrrolidinyl)-1-cyclopropyl-6-fl**	4.8_332.12	NEG	4.8	332.1294
**Isorangiformic acid**	7.099_794.	NEG	7.099	794.5169
**L-Serine**	0.668_105.	NEG	0.668	105.0426
**Stanolone benzoate**	6.759_394.	POS	6.759	394.25

Comamonas_humi				
Other				
Dolosigranulum_pigrum				
Polynucleobacter_acidiphobus				
Povalibacter_uvarum				
**8-(3,4-Dimethoxyphenyl)-7-methyl-1,3-dipropyl-1**	6.67_386.1	POS	6.67	386.1936
**Antimony potassium tartrate;**	0.772_667.	NEG	0.772	667.8755
**Dainic Acid A**	3.382_281.	POS	3.382	281.1627
**Nicotinic acid**	1.027_123.	POS	1.027	123.0322
**Fluorene**	8.04_166.0	POS	8.04	166.0784
**24a,24b-epoxy-23-tetradehydro-24a,24b-dihomo-**	10.414_45	POS	10.414	454.3062
**2-Nitrophenyl octyl ether**	9.023_251.	NEG	9.023	251.152
**Heteropeucenin, Methyl Ether**	5.797_274.	POS	5.797	274.1205
**Tremetone**	7.734_202.	POS	7.734	202.0996
**Olodaterol**	8.169_386.	POS	8.169	386.1852
**N-(N-(3-Carboxyoxirane-2-carbonyl)leucyl)isoamy**	3.969_314.	POS	3.969	314.1841
**N-[4,5-dihydroxy-2-[[4-[hydroxy(phenyl)methyl]-!**	5.3_537.14	POS	5.3	537.1424
**(8R,9S,10S,13S,14S,17R)-16-Fluoro-17-hydroxy-10**	8.122_308.	POS	8.122	308.214
**LMST04010335**	7.967_388.	NEG	7.967	388.2616
**15-OxoEDE**	9.941_322.	POS	9.941	322.2483
Bifidobacterium_bifidum				
**NCGC00380167-01!3-(4-hydroxy-3-methoxypheny**	3.754_352.	POS	3.754	352.1269
**(2R,3S,4S,5R,6S)-2-(hydroxymethyl)-6-[4-(hydroxy**	7.097_332.	NEG	7.097	332.1833
**PE(12:0/22:0)**	11.658_71	POS	11.658	719.5476
**Cedpht**	5.502_304.	POS	5.502	304.1461
**3-Hydroxybutyric acid**	0.728_104.	NEG	0.728	104.0474
**(+)-Eudesmin; Pinoresinol dimethyl ether;**	6.893_386.	POS	6.893	386.1724
**Asulam**	3.438_230.	POS	3.438	230.0365
**Convallasaponin A;**	9.028_580.	POS	9.028	580.3596
**Thalicoside A;**	9.451_798.	POS	9.451	798.4747
**lipoamide**	5.782_205.	NEG	5.782	205.0586
**LMST05040008**	9.012_541.	NEG	9.012	541.3429
**20, 22-Dihydrodigoxigenin**	8.573_392.	NEG	8.573	392.2567
**Cholic acid glucuronide**	7.774_584.	NEG	7.774	584.3196
**Telmisartan glucuronide**	7.808_690.	NEG	7.808	690.2713
**Azobilirubin**	5.844_440.	NEG	5.844	440.1507
**N-(1-Hydroxypropan-2-yl)icosa-5,8,11,14-tetraena**	10.216_36	POS	10.216	361.2981
**Verapamil**	5.424_454.	POS	5.424	454.2816
**Prostaglandin E1**	9.128_336.	POS	9.128	336.2301
**[5-chloro-3-[(1E,3E)-3,5-dimethylhepta-1,3-dienyl**	5.778_411.	POS	5.778	411.1214
**3-Hydroxy-11Z-octadecenoylcarnitine**	8.743_441.	POS	8.743	441.3456
**LMST01031079**	8.486_528.	NEG	8.486	528.3294
**icas#18**	7.932_475.	POS	7.932	475.2574
**4-(N,N-Dimethylaminosulfonyl)-7-fluoro-2,1,3-be**	4.965_245.	POS	4.965	245.028
**Flumipropyn; S-23121;**	0.696_347.	POS	0.696	347.072
**Diisodecyl phthalate**	9.406_446.	POS	9.406	446.3399
**1-tauro-tetranor-PGE1**	5.058_405.	NEG	5.058	405.1823
**1alpha,25-dihydroxy-26,27-dimethyl-20,21,22,22,2**	9.489_438.	POS	9.489	438.3114
**Isobutyl acrylate**	5.245_128.	POS	5.245	128.0838
**PGF2alpha isopropyl ester**	9.244_396.	NEG	9.244	396.2876
**{1-[2-(4-Carbamimidoyl-benzoylamino)-propionyl**	7.03_376.1	POS	7.03	376.1731
**PCTR3**	8.818_463.	POS	8.818	463.2396
Nocardiopsis_dassonvillei				
**4-Nitroquinoline N-oxide**	2.44_190.0	NEG	2.44	190.0378
**2-[6-(4-Chlorophenoxy)hexyl]oxirane-2-carboxylic**	3.953_298.	POS	3.953	298.0986
**NeuAc(alpha->6)GalNAc(alpha1->O)Ser**	5.432_599.	POS	5.432	599.2155
**LMPK12140094**	5.888_576.	NEG	5.888	576.1467
**Isotretinoin**	7.355_300.	POS	7.355	300.2088

2-Phenylglycine	3.91_151.C	POS	3.91	151.0635
(11Z)-8,18-propano-retinal	9.048_324.	POS	9.048	324.2453
Nithiazide;	3.904_216.	POS	3.904	216.0319
Sagittatosdie B	7.12_646.2	POS	7.12	646.2275
Ipsapirone	5.766_401.	NEG	5.766	401.1509
(4E,7E,10E,13E)-Hexadeca-4,7,10,13-tetraenoic acic	9.681_248.	POS	9.681	248.1777
3-[2-(6,7-dihydroxy-1,2,4a-trimethylspiro[3,4,6,7,8	5.886_366.	NEG	5.886	366.2038
Aluminum acetoacetate;	5.758_330.	POS	5.758	330.2235
Disobutamide	8.893_407.	NEG	8.893	407.2707
4(G)-alpha-Glucopyranosyl-rutin	4.577_772.	POS	4.577	772.2065
N'-[2-(Oxidanylcarbamoyl)phenyl]-N-phenyl-octa	4.845_383.	POS	4.845	383.1843
4-Formylbenzoic acid	2.133_150.	POS	2.133	150.0319
2-Hydrazinopyridine	0.633_109.	POS	0.633	109.0642
Burkholderia_sediminicola				
(2R,4R,5R)-2,4,5,6-Tetrahydroxy-1-sulfanylhexan-3	0.71_196.C	NEG	0.71	196.0406
Phytosphingosine	8.214_317.	POS	8.214	317.2922
Heliotrine	6.915_331.	POS	6.915	331.1993
Benzoyl-DL-arginine-naphthylamide	6.227_403.	NEG	6.227	403.2028
Belnacasan	6.342_508.	POS	6.342	508.2064
9-Decenoylcarnitine	7.393_313.	NEG	7.393	313.2253
Hyodeoxycholic acid	8.591_372.	POS	8.591	372.2665
Z-PP-CHO	7.916_330.	POS	7.916	330.1578
Reduced coenzyme F420;	7.984_775.	POS	7.984	775.1974
Nebrosteroid M	9.735_446.	POS	9.735	446.3762
SQMG(16:1(9Z)/0:0)	5.468_554.	POS	5.468	554.2775
3,7-Dimethylbicyclo[4.1.0]heptane-1,7-dicarbaldeh	10.136_18	POS	10.136	180.1152
11,12-dihydroxy-13-methoxy-4,5,8-trimethyl-3-(2	8.062_468.	POS	8.062	468.3182
Galactosylhydroxylysine	0.608_324.	POS	0.608	324.1532
Amprenavir	8.707_505.	POS	8.707	505.2222
Vulpinic Acid	0.643_322.	NEG	0.643	322.0835
2-Sulfotrehalose; 2-O-Sulfo-alpha,alpha-trehalose	0.65_422.C	NEG	0.65	422.073
Baccatin III	7.817_586.	POS	7.817	586.2431
trans-2-Dodecenoylcarnitine	6.168_341.	NEG	6.168	341.2567
N-Desmethyl-O-hydroxy rosiglitazone	5.951_359.	POS	5.951	359.0939
Chapso	8.615_630.	POS	8.615	630.3888
Brucein E	7.098_412.	POS	7.098	412.1732
1-Oleoylglycerophosphocholine; 1-Oleoyl-sn-glyc	8.221_522.	NEG	8.221	522.3549
TRIFLUOROMETHANOL	0.785_85.9	NEG	0.785	85.99796
Sulfacytine	2.721_294.	POS	2.721	294.0786
Nephrosteranic acid	7.091_339.	POS	7.091	339.2406
(-)-Epigallocatechin sulfate	3.228_385.	POS	3.228	385.0237
NA	7.349_348.	POS	7.349	348.2299
Tanshinone I	5.858_276.	POS	5.858	276.0799
(2R,3R)-3-Methylglutamyl-5-semialdehyde-N6-lys	3.2_273.16	POS	3.2	273.1687
2-Octenal	7.21_126.1	POS	7.21	126.1046
(2S)-5-(Diaminomethylideneamino)-4-methyl-2-(r	5.949_202.	POS	5.949	202.1433
dTDP-4-(methylamino)-2,3,4,6-tetradeoxy-D-gluc	8.217_529.	POS	8.217	529.1249
1'-Hydroxyestragole	5.756_164.	NEG	5.756	164.0839
5alpha-Androstan-3beta,17alpha-diol disulfate	3.641_452.	POS	3.641	452.1547
Cytosine	3.217_222.	POS	3.217	222.0869
A-D-Glucopyranoside,6-O-acetyl-1,3,4-tris-O-(2-r	5.43_454.1	POS	5.43	454.1675
N2,N5-Diacetylornithine	1.026_216.	NEG	1.026	216.111
OOV-PG	10.353_60	POS	10.353	608.3323
DU 122290;	4.949_362.	POS	4.949	362.1671
Myxochelin A;	6.321_404.	POS	6.321	404.1583
3-Hydroxyhexadecanoylcarnitine	8.628_415.	POS	8.628	415.3299
Cabergoline	8.787_451.	POS	8.787	451.2952
Delcorine;	7.199_479.	POS	7.199	479.2883

**<U+0394>17-6-keto prostaglandin F1a**	8.243_368.	NEG	8.243	368.2234
**Dydrogesterone**	7.158_312.	POS	7.158	312.2088
**Meteloidine;**	5.414_255.	POS	5.414	255.147
**1-Nonadecanoyl-glycero-3-phosphoserine**	9.574_539.	NEG	9.574	539.3217
**o-Phenetidine**	3.151_137.	POS	3.151	137.0844
**Borrelidin;**	8.623_489.	POS	8.623	489.3096
Bordetella_avium				
**NCGC00385489-01!3-[4-methyl-1-(2-methylpropa**	8.357_250.	POS	8.357	250.157
**(2R,4S)-1-Tert-Butyl 2-methyl 4-aminopyrrolidine-**	4.775_244.	POS	4.775	244.1422
**Metabolite M6**	7.855_522.	POS	7.855	522.322
**Methyl sulfate**	0.669_111.	NEG	0.669	111.9831
**(9E)-11a-hydroxy-3,6,10-trimethyl-6,7,8,11-tetrahy**	8.595_264.	POS	8.595	264.1362
**1,5,9-trihydroxy-5,7,7-trimethyl-4,5a,6,8,8a,9-hexa**	8.599_282.	POS	8.599	282.1468
**N-Carbobenzoxyglycyl-prolyl-4-methylcoumarinyl**	7.4_463.17	POS	7.4	463.1749
**1,9b-dihydroxy-6,6,9a-trimethyl-1,5,5a,7,8,9-hexal**	3.88_304.1	POS	3.88	304.1091
**Isosorbide Dinitrate**	0.682_236.	NEG	0.682	236.0273
**Benorilate**	5.456_313.	NEG	5.456	313.0951
**1-Oleoyl-rac-glycerol**	9.859_373.	POS	9.859	373.3192
**(2S)-2-[2-[(5R,6R,7S,9S,16R,18S,19S)-19-amino-6-**	9.634_743.	POS	9.634	743.3472
**2-Mercaptobenzothiazole; Captax;**	5.935_166.	POS	5.935	166.9866
**Aplysinal;**	3.468_308.	POS	3.468	308.0427
**1-(7Z-Hexadecenoyl)-2-(4Z,7Z,10Z,13Z,16Z,19Z-do**	11.454_80:	POS	11.454	803.5442
**Pirbenicillin**	7.53_510.1	POS	7.53	510.1697
**methyl 8-hydroxy-4,5,7,10,14,14-hexamethyl-6,17-**	7.934_459.	POS	7.934	459.2625
**LMST01031080**	9.074_528.	POS	9.074	528.3311
**(+)-Sesamin**	5.314_376.	POS	5.314	376.0923
**2-O-Sulfo-2'-palmitoyl-alpha,alpha-trehalose;**	6.719_660.	NEG	6.719	660.3018
**8-Demethyl-8-(2,3,4-tri-O-methyl-alpha-L-rhamn**	5.466_646.	NEG	5.466	646.1914
**4-(10,13-Dimethyl-2,3,4,5,6,7,8,9,11,12,14,17-dode**	10.213_34:	POS	10.213	342.2558
**NCGC00179793-02!3,4-dimethyl-2,5-bis(3,4,5-trim**	7.935_414.	POS	7.935	414.2042
**12-hydroxyjasmonic acid 12-O-beta-D-glucoside**	3.93_386.1	POS	3.93	386.1927
**Cyproteron**	5.316_374.	POS	5.316	374.1647
**11-amino-undecanoic acid**	0.749_201.	POS	0.749	201.1732
**Hydroxypropionylcarnitine**	4.095_233.	NEG	4.095	233.1262
**Sulfamethazine**	3.933_278.	POS	3.933	278.0838
Thermicanus_aegyptius				
**6-O-(15-methyl-palmitoyl)-6'-O-oleoyltrehalose**	8.548_858.	POS	8.548	858.6053
Leifsonia_naganoensis				
**Istradefylline**	4.804_384.	NEG	4.804	384.1785
**Ascorbyl stearate**	8.553_442.	POS	8.553	442.2932
**3-Phenoxypropylene di(acetate)**	6.781_252.	POS	6.781	252.0997
**Calebassone; C-Calebassine;**	9.944_616.	NEG	9.944	616.3774
Brachybacterium_ginsengisoli				
**2,6-Ditert-butyl-4-[3-(3,5-ditert-butyl-4-hydroxyp**	10.37_484.	POS	10.37	484.3559
**(2R,3S,4S,5R,6R)-5-[(2S,3R,4R)-3,4-dihydroxy-4-(h**	4.914_432.	POS	4.914	432.1625
**Xanthumin;**	8.519_306.	POS	8.519	306.1473
**Methyl (2R,3R)-2-[(3-carbamimidoylphenyl)methyl**	6.797_446.	NEG	6.797	446.1976
**Chloramphenicol palmitate**	3.398_560.	POS	3.398	560.2447
**(8aR,12S,12aR)-12-hydroxy-4-methyl-4,5,6,7,8,8a,:**	6.105_274.	POS	6.105	274.1182
**all-trans-5,6-Epoxyretinoic acid**	8.914_316.	POS	8.914	316.2039
**2-[(4-hydroxy-3,5-dimethoxyphenyl)methoxy]-6-(**	3.893_346.	POS	3.893	346.1277
**(-)-Fusicoplagin A**	8.141_438.	POS	8.141	438.2619
**Jasmonic acid methyl ester**	7.531_224.	NEG	7.531	224.1412
**3-Methylglutaconic acid**	3.836_144.	POS	3.836	144.0788
**(4E,6E,d14:2) sphingosine**	5.663_241.	NEG	5.663	241.204
**7-Deacetoxy-7-Oxokhivorin**	6.485_542.	NEG	6.485	542.254
**2-(5-Fluoropentyl)-2-methylmalonic acid**	6.563_206.	POS	6.563	206.0946
**Predurmillone**	3.36_380.1	POS	3.36	380.1259

**(5R)-5-hydroxy-1-(4-hydroxy-3-methoxyphenyl)t**	9.338_350.	POS	9.338	350.246
**(Z)-7-Dodecenyl formate**	8.628_212.	NEG	8.628	212.1776
**3-Hydroxy-11-methyl-stearic acid**	9.795_314.	NEG	9.795	314.2821
**Descinolone acetonide; 21-Deoxytriamcinoline ace**	7.154_418.	POS	7.154	418.2139
**Ciclazindol**	3.464_298.	POS	3.464	298.087
**(+/-)13-HODE**	8.945_296.	NEG	8.945	296.235
**Oxyphencyclimine**	8.261_344.	POS	8.261	344.2099
**Perindopril**	5.442_368.	POS	5.442	368.2312
**Pitavastatin; (3R,5S,E)-7-(2-cyclopropyl-4-(4-fluor**	4.458_420.	POS	4.458	420.1609
**Pinky**	3.429_328.	NEG	3.429	328.1093
**[(1R,3aR,5R,5aR,8aR,9aR)-1,5,8a-trimethyl-2,8-dio**	6.254_306.	POS	6.254	306.1467
**Halobetasol Propionate**	6.902_484.	NEG	6.902	484.1844
Adlercreutzia_equolifaciens				
**5-Carboxy-2'-deoxyuridine**	0.66_272.0	NEG	0.66	272.0642
**2-(3,5-Dihydroxyphenyl)ethanol sulfate**	6.121_234.	POS	6.121	234.0206
**Lipstatin**	9.07_491.3	NEG	9.07	491.3611
**Colchicine**	8.097_437.	POS	8.097	437.1237
**7-(2-Chloro-6-fluorophenyl)-5-(4-chlorophenyl)-**	0.692_363.	POS	0.692	363.046
**N,N-Diethylethanolamine**	0.745_117.	POS	0.745	117.1155
Other				
**Chalepin acetate**	4.986_378.	POS	4.986	378.1426
**(8S,9S,10R,13R,14S,17R)-5,6-Dihydroperoxy-10,13-**	8.66_452.3	POS	8.66	452.3505
**2-Methylglutaric acid**	4.829_146.	NEG	4.829	146.058
**Carumonam sodium;**	8.066_510.	POS	8.066	510.3672
**grayanotoxin III**	8.545_756.	POS	8.545	756.3418
**Nivacortol**	4.779_430.	POS	4.779	430.2428
**Lunarine**	8.122_454.	POS	8.122	454.2571
**N-[(1S)-1-(Ethoxycarbonyl)-3-phenylpropyl]-L-ala**	5.75_479.2	POS	5.75	479.2272
**Murnac-tripeptide**	8.115_664.	POS	8.115	664.29
**Lasiocarpine; 7-Angelyl-9-lasiocarpylheliotridine;**	7.033_411.	POS	7.033	411.2262
**5-Tetradecenoic acid**	8.988_226.	NEG	8.988	226.1932
**Brivanib alaninate**	5.354_441.	NEG	5.354	441.1822
Other				
**Arlacel A**	8.911_410.	POS	8.911	410.3033
Lysinibacillus_xylanilyticus				
**PG(P-18:0/0:0)**	8.859_496.	NEG	8.859	496.3145
**NA**	3.481_380.	POS	3.481	380.0721
Desulfovibrio_desulfuricans				
Deinococcus_xinjiangensis				
Chryseobacterium_rhizoplanae				
Other				
Luteimonas_vadosa				
Vibrio_kanaloae				
Mucispirillum_schaedleri				
Bifidobacterium_dentium				
Lactobacillus_buchneri				
Rhodopseudomonas_pentothenatexigens				
Sphingomonas_endophytica				
Curvibacter_fontanus				
Bacteroides_salyersiae				
**Glyburide**	5.736_493.	NEG	5.736	493.1438
**N-[(1R)-1-[3-(4-Ethoxyphenyl)-4-oxopyrido[2,3-d**	8.091_603.	POS	8.091	603.208
**Carbamothioic acid, diethyl-**	3.164_133.	POS	3.164	133.0563
**1-Ethylaziridine**	0.872_71.0	POS	0.872	71.07364
**(2E,4E)-Nonadeca-2,4-dienoic acid**	10.187_29	POS	10.187	294.2557
**Estra-1,3,5(10),16-tetraen-3-ol benzoate;**	8.823_358.	POS	8.823	358.1926
**2,2-Difluoro-n-(2-hydroxyethyl)-3-(2-nitro-1h-im**	2.872_264.	POS	2.872	264.0667
**Tert-butylbenzene**	10.135_13	POS	10.135	134.1096

**Imipenem**	3.068_299.	POS	3.068	299.0943
**(8S,9S,10S,11S,13S,14S,17R)-11,17-Dihydroxy-17-(**	6.979_378.	NEG	6.979	378.2042
**2,2,3,3-Tetramethylcyclopropanecarboxylic acid**	5.655_142.	NEG	5.655	142.0995
**Amiodarone**	8.123_645.	POS	8.123	645.0233
**allo-Protolichesterinic acid**	7.485_341.	POS	7.485	341.2561
**4-Ethylphenol**	7.256_122.	POS	7.256	122.0733
Rhodococcus_jialingiae				
**3-Amino-6-{4-[(4-Methylpiperazin-1-Yl)sulfonyl]p**	4.401_453.	POS	4.401	453.1573
**L-Cysteine**	8.59_121.C	NEG	8.59	121.0198
**3beta,7alpha-Dihydroxy-5-cholestenoate**	10.393_43.	POS	10.393	432.3241
**PA(22:6(4Z,7Z,10Z,13Z,16Z,19Z)/0:0)**	7.638_482.	POS	7.638	482.2454
**Prednisolone hemisuccinate**	8.184_460.	NEG	8.184	460.2097
**cis-N-((4-Cyano-2-(trifluoromethyl)phenyl)methyl**	6.792_447.	NEG	6.792	447.2006
**7-methoxy-6-(1,2,3-trihydroxy-3-methylbutyl)chr**	7.934_294.	POS	7.934	294.1102
**Ethoxyquin**	3.861_217.	POS	3.861	217.147
**Camostat**	5.765_398.	NEG	5.765	398.1576
**Propentofylline**	5.424_306.	POS	5.424	306.1679
**Roquefortine F;**	8.195_419.	POS	8.195	419.1975
**Dehydrorotenone**	5.407_392.	POS	5.407	392.1279
Other				
**15R-PGE2 methyl ester, 15-acetate**	7.881_408.	NEG	7.881	408.2514
**N-arachidonoyl taurine**	8.92_411.2	POS	8.92	411.2446
**12-KETE**	9.224_318.	POS	9.224	318.2195
**3-(4-Hydroxy-3-methoxyphenyl)-2-methyllactic a**	5.868_226.	POS	5.868	226.0842
**Narbomycin;**	8.395_509.	POS	8.395	509.3359
**(2R,3S,4S,5S,6R)-2-[4-[7-hydroxy-3-(hydroxymeth**	4.971_514.	POS	4.971	514.1832
**Paeonimetabolin I**	5.753_198.	POS	5.753	198.0894
**Ecdysone**	8.627_464.	NEG	8.627	464.314
**enprostil**	8.133_400.	POS	8.133	400.1883
**4,7,13,16,21,24-Hexaoxa-1,10-diazabicyclo[8.8.8]h**	9.581_376.	POS	9.581	376.2589
**6,8-Di-C-arabionopyranosylluteolin**	6.08_550.1	NEG	6.08	550.1314
**Anabasamine Hydrochloride**	4.991_289.	POS	4.991	289.1347
**Retosiban**	7.251_494.	NEG	7.251	494.2514
**Prontosil**	0.748_291.	POS	0.748	291.0777
**D,L-Buthionine**	3.5_191.09	NEG	3.5	191.098
**17-Hydroxypregnenolone 3-sulfate**	7.502_412.	NEG	7.502	412.1921
**Crisnatol**	3.939_345.	POS	3.939	345.172
**(2S,3S,4S)-4-[(6S)-6-Carboxyhepta-2,4-dien-2-yl]-**	5.437_311.	POS	5.437	311.1368
**10E,12E-tetradecadiene-4,6-diynoic acid**	8.274_216.	POS	8.274	216.1154
**Aristospan**	7.052_532.	POS	7.052	532.2827
**1alpha-fluoro-25-hydroxy-16,17,23,23,24,24-hexa**	9.252_412.	POS	9.252	412.2758
**OHOOA-PG**	10.384_66	POS	10.384	664.3594
**11-Hydroxyprogesterone 11-glucuronide**	7.357_506.	POS	7.357	506.2522
**Gentian Violet**	9.728_372.	NEG	9.728	372.2432
**LysoPC(22:6(4Z,7Z,10Z,13Z,16Z,19Z)/0:0)**	9.527_567.	POS	9.527	567.3303
**(S)-4-[(S)-2-((S)-1-Formyl-4-guanidino-butylcarb**	8.476_510.	NEG	8.476	510.3188
**DL-Dopa**	3.695_197.	POS	3.695	197.0691
Soonwooa_buanensis				
**Fucoxanthinol 3-arachidonate**	8.587_902.	POS	8.587	902.641
**(1S,3R,17S,19S)-3-hydroxy-9,9,16,16-tetramethyl-**	8.791_447.	POS	8.791	447.2161
**Cer(d18:1/12:0)**	10.615_48	POS	10.615	481.45
**N-[(5Z)-5-(2,6-Dioxo-1,3-dipropylpurin-8-ylidene**	8.206_555.	POS	8.206	555.2581
Bacteroides_stercoris				
**ascr#14**	5.75_290.1	POS	5.75	290.1729
**Janthitrem B;**	10.032_58	POS	10.032	585.3453
**4-[3-Chloro-4-(pyridin-2-ylmethoxy)anilino]-6-[[(**	7.689_542.	POS	7.689	542.2177
**Cer(d18:0/20:0)**	9.836_595.	POS	9.836	595.591
**Naringin 4'-glucoside**	8.124_742.	POS	8.124	742.2344

**Anopterine;**	8.853_541.	NEG	8.853	541.3065
**Melamine**	2.791_126.	POS	2.791	126.0658
**4-Hydroxyvalsartan**	8.197_451.	POS	8.197	451.2238
**1-Naphthol**	5.96_144.0	POS	5.96	144.0577
**Phorone A**	9.445_382.	POS	9.445	382.2509
**5,7-dihydroxy-2-(4-hydroxy-3-methoxyphenyl)-3-**	5.521_624.	POS	5.521	624.1694
**Puerarin 6''-acetate**	4.378_458.	POS	4.378	458.1223
**Andrographolide**	8.259_332.	POS	8.259	332.1987
**N-Desmethyllevomepromazine**	8.215_314.	POS	8.215	314.1447
**4-Methylumbelliferyl oleate**	8.12_440.2	POS	8.12	440.2925
**(1S,2S,5R,7R,10S,11S,14R,16R,19S,20S,23R,25R)-5,1**	8.92_580.3	NEG	8.92	580.3616
**23,24-Dihydrocucurbitacin B;**	8.278_560.	POS	8.278	560.3353
**Convoline;**	3.924_307.	POS	3.924	307.1416
**N-acetyl-d-tryptophan**	4.971_246.	POS	4.971	246.1002
**trans-2-(3-Methoxy-5-methylsulfonyl-4-propoxyp**	6.705_480.	NEG	6.705	480.1816
**2-O-(beta-D-galactopyranosyl-(1->6)-beta-D-gal**	8.245_498.	NEG	8.245	498.2319
**Stigmasterol**	11.772_41	POS	11.772	412.3706
**PG(18:0/19:1(9Z))**	8.269_790.	NEG	8.269	790.5747
**methyl 10-acetyloxy-4,5,9-trihydroxy-1,4a-dimeth**	2.853_442.	POS	2.853	442.1973
**17-hydroxyprogesterone caproate**	8.796_444.	POS	8.796	444.2878
**genipin 1-gentiobioside**	8.398_567.	POS	8.398	567.2181
**Copanlisib**	3.431_480.	POS	3.431	480.2224
**Edotecarin**	5.6_608.17	NEG	5.6	608.1772
**Nebramycin 5'; Nebramycin factor 5'; 6''-O-Carbam**	7.668_510.	NEG	7.668	510.2648
**Riboprine**	3.953_335.	POS	3.953	335.1578
Pseudobutyrivibrio_ruminis				
Planktomarina_temperata				
**9Z-Dodecenal**	7.562_182.	POS	7.562	182.1675
**Thiencynonate**	8.821_363.	POS	8.821	363.1871
**1-Carboxyethylvaline**	5.658_189.	NEG	5.658	189.1002
**Linagliptin**	3.065_472.	POS	3.065	472.2326
**Allopumiliotoxin 267A;**	7.922_267.	POS	7.922	267.2199
**Dehydronorketamine**	3.918_221.	POS	3.918	221.0609
**N-[(2R)-6,7-Dihydroxy-3-oxo-1-sulfanylheptan-2-**	3.895_235.	POS	3.895	235.0879
**2-mercapto-octadecanoic acid**	7.043_316.	POS	7.043	316.2443
**1-dimethylarsinoyl-pentadecane**	5.437_332.	POS	5.437	332.2072
**DG(14:0/20:2(11Z,14Z)/0:0)**	12.48_592.	POS	12.48	592.5067
Chryseobacterium_hispalense				
Other				
Other				
Other				
Rhizobium_pusense				
Sphaerochaeta_globosa				
Other				
Other				
Bacteroides_reticulotermitis				
Other				
Stenotrophomonas_humi				
Bosea_minatitlanensis				
Dokdonia_eikasta				
Lactococcus_fujiensis				
Sediminibacterium_salmoneum				
Sandarakinorhabdus_limnophila				
Other				
Lysobacter_brunescens				
Anaerostipes_caccae				
Sphingomonas_ginsenosidimutans				
Vibrio_navarrensis				

Rubellimicrobium_aerolatum				
Other				
Pseudomonas_deceptionensis				
Streptococcus_parauberis				
**OA-6129 D;**	5.477_475.	POS	5.477	475.1991
**Mecapegfilgrastim**	5.018_536.	POS	5.018	536.1951
**Glycyrrhetinic acid**	8.858_470.	POS	8.858	470.3399
**Menadiol;**	7.517_174.	POS	7.517	174.0683
**(4aS)-6,7-dihydroxy-1,1,4a-trimethyl-3,4,10,10a-te**	10.319_27	POS	10.319	274.1569
**(3S)-3-[[(2S)-2-[[(2S)-2-Amino-3-methylbutanoyl]**	7.39_481.1	POS	7.39	481.1852
**3-(Cystein-S-yl)acetaminophen**	2.596_270.	POS	2.596	270.0676
**Canthiumine;**	9.381_552.	POS	9.381	552.2747
**(3'-sulfo)Galbeta-Cer(d18:1/16:0)**	12.866_77	NEG	12.866	779.5251
**Fosteabine**	9.028_575.	POS	9.028	575.3313
**Scutellarioside II**	5.72_486.1	POS	5.72	486.1747
**Ilexgenin A**	8.586_502.	NEG	8.586	502.329
**Paracetamol cysteine**	5.977_254.	NEG	5.977	254.0718
**Bezafibrate**	4.897_361.	POS	4.897	361.1098
**Ricolinostat**	8.196_433.	POS	8.196	433.2133
**4-trans-Hydroxyglipizide**	7.43_461.1	NEG	7.43	461.1752
**Linkckoside G**	8.43_596.3	NEG	8.43	596.3562
**Retinyl methyl ether**	8.388_300.	POS	8.388	300.2453
**24-Methylcholesterol**	11.624_40	POS	11.624	400.3706
**[6-acetyloxy-4-butanoyloxy-3,3a-dihydroxy-3,6,9-**	8.825_672.	POS	8.825	672.3153
**6-Carboxy-X-rhodamine**	7.508_534.	POS	7.508	534.2175
**Cromakalim**	6.617_286.	NEG	6.617	286.1316
**Fludarabine**	1.445_365.	POS	1.445	365.0535
**Bromovulone I**	6.791_424.	POS	6.791	424.127
**methiothepin**	7.372_356.	POS	7.372	356.1371
**7'-carboxy-N-geranyl-L-gluatamic acid**	7.556_313.	POS	7.556	313.1509
**Ethyl octadec-9-enoate**	8.989_310.	POS	8.989	310.2871
**Obscurolide A1**	6.657_273.	POS	6.657	273.1
**Phenyl acetate**	2.817_136.	POS	2.817	136.0525
**Lovastatin acetylated hydroxylated putative derivat**	7.5_481.30	POS	7.5	481.3039
**COLFORSIN**	8.197_448.	POS	8.197	448.1881
Enterorhabdus_caecimuris				
**Valproylcarnitine**	5.648_287.	NEG	5.648	287.2095
**3-(1-Azatricyclo[7.3.1.05,13]trideca-5,7,9(13)-trien**	6.567_268.	POS	6.567	268.1208
**Ethyl n-allyl-n-[(e)-2-methyl-3-[4-(4-amidinophe**	7.442_449.	POS	7.442	449.1952
**Emodin 8-glucoside**	7.987_432.	POS	7.987	432.106
**7-Methylthioheptyl-desulfoglucosinolate;**	3.92_383.1	POS	3.92	383.1455
Dialister_pneumosintes				
**Lovastatin**	8.186_404.	POS	8.186	404.2564
**2-Chloro-4-biphenylol; 2-Chloro-4-hydroxybiphe**	3.918_204.	POS	3.918	204.0344
**(3R,4S,5S,6R)-2-(6-hydroxy-2,6-dimethylocta-2,7-**	6.85_332.1	NEG	6.85	332.1835
**Ecabapide**	8.288_371.	POS	8.288	371.1844
**4,4'-Dihydrazino-biphenyl**	5.331_214.	POS	5.331	214.1209
**4'-Hydroxymethohexital**	3.012_278.	POS	3.012	278.1266
**3Alpha-Hydroxy-3-Deoxyangolensic Acid Methyl E**	7.935_489.	POS	7.935	489.2731
**3,17-Androstanediol glucuronide**	7.436_468.	NEG	7.436	468.2721
**2-(2-Chloro-4-fluorobenzyl)-3-(4-fluorophenyl)-7**	3.414_422.	POS	3.414	422.0596
**Oxymatrine**	3.888_264.	POS	3.888	264.187
**icas#22**	7.471_503.	POS	7.471	503.2867
**PE(0:0/22:5(4Z,7Z,10Z,13Z,16Z))**	9.848_527.	NEG	9.848	527.3007
**NA**	6.586_448.	NEG	6.586	448.2131
**Carnosine**	0.721_226.	POS	0.721	226.1069
**3beta-(3-methyl-butanoyloxy)-villanovane-13alph**	8.113_438.	POS	8.113	438.2978
**terpestacin**	9.59_402.2	POS	9.59	402.2771

5-(5-(2,6-Dichloro-4-(4,5-Dihydro-2-Oxazoly)phe	4.024_382.	NEG	4.024	382.0836
Cortolone-3-glucuronide	6.772_542.	NEG	6.772	542.2722
COSTUNOLIDE	7.082_232.	POS	7.082	232.1464
Parfumine	5.771_391.	POS	5.771	391.0837
(1RS,2RS)-2-(4-Hydroxyphenyl)-2-methyl-1-(9-(4,	3.528_588.	POS	3.528	588.268
SM(d18:1/22:0)	10.399_78	POS	10.399	786.6616
(+/-)-(E)-Methyl-2-[(E)-hydroxyimino]-5-nitro-6-	3.464_231.	POS	3.464	231.0857
Soybean lecithin	11.552_75	POS	11.552	757.5608
Mometasone	6.344_426.	NEG	6.344	426.1349
Amfenac	6.561_255.	POS	6.561	255.0896
(3-{[3-(2-Amino-2-Oxoethyl)-1-Benzyl-2-Ethyl-1H	5.413_430.	POS	5.413	430.1645
2,4(1H,3H)-Pyrimidinedione, 3-((2R)-2-amino-2-p	5.829_495.	POS	5.829	495.1791
6-Ethoxy-2-mercaptobenzothiazole	6.631_211.	POS	6.631	211.0128
21-hydroxy-10-methoxy-1,4,14,19,19-pentamethy	7.679_466.	POS	7.679	466.1983
Dicumarol	0.668_336.	NEG	0.668	336.0626
Orciprenaline-3-O-sulfate	0.672_291.	NEG	0.672	291.0774
Noscapine	3.912_413.	POS	3.912	413.1507
(1'S,3R,3'R,5R,6'R,7'S)-5-(furan-3-yl)-3',6',7'-trime	3.846_366.	POS	3.846	366.1425
Metconazole;	5.894_319.	POS	5.894	319.1453
Aconine	8.915_499.	POS	8.915	499.2776
Diflufenican	5.259_394.	NEG	5.259	394.0724
Delphinine;	3.902_599.	POS	3.902	599.3067
2,4-Bis(2-methoxyethoxy)-1,3,5-triazine	0.744_229.	POS	0.744	229.1064
ANTHRACENE	8.064_178.	POS	8.064	178.0784
Quinolinic acid	0.66_167.0	NEG	0.66	167.0219
Tetrahydroaldosterone	7.667_364.	NEG	7.667	364.2252
Cer(t18:0/14:0)	9.647_527.	POS	9.647	527.4921
PYROVALERONE	4.849_245.	POS	4.849	245.1781
Isovanillic acid glucuronide	0.657_344.	NEG	0.657	344.0743
Euchrenone a4	6.583_474.	POS	6.583	474.2404
13E-Tetranor-16-carboxy-LTE4	5.999_413.	NEG	5.999	413.1508
2-Aminoheptanoate	3.181_145.	POS	3.181	145.1106
Lavoltidine	5.822_359.	POS	5.822	359.2307
Pivampicillin	7.312_463.	POS	7.312	463.178
SNAG-delta5-CA	7.831_673.	NEG	7.831	673.3142
2-(2-Aminopropanoylamino)bicyclo[3.1.0]hexane-2	1.828_256.	POS	1.828	256.1059
MGMG(18:5(3Z,6Z,9Z,12Z,15Z)/0:0)	7.533_510.	NEG	7.533	510.2827
N-[4-[(4As)-1,2,4,4a,5,11-hexahydro-[1,4]oxazino[	7.734_537.	POS	7.734	537.1803
(-)-Norephedrine	3.319_151.	POS	3.319	151.1
2,4,6,8,10-dodecapentaenal	8.485_174.	POS	8.485	174.1046
Urobilinogen	8.18_590.3	POS	8.18	590.3107
Ethyl 4-methyl-5-(1-methylethoxy)-9H-pyrido(3,4	7.562_312.	POS	7.562	312.1474
2'-Fluoro-2',3'-dideoxyadenosine	3.454_253.	POS	3.454	253.0983
LysoPC(20:4(5Z,8Z,11Z,14Z)/0:0)	8.978_543.	NEG	8.978	543.33
Allopregnanolone	9.824_336.	POS	9.824	336.2663
S-[(E)-N-Hydroxy(indol-3-yl)acetimidoyl]-L-glutat	5.309_479.	POS	5.309	479.1477
Retaspimycin	9.466_587.	NEG	9.466	587.3229
Fosinoprilat;	8.024_435.	POS	8.024	435.2158
Locacorten	8.466_494.	POS	8.466	494.2457
7-Hydroxyloxapine	3.612_343.	NEG	3.612	343.1091
3-Amino-5-mercapto-1,2,4-triazole	0.736_116.	NEG	0.736	116.0156
ascr#27	10.019_40	NEG	10.019	400.2825
Hydrochlorothiazide	2.841_296.	POS	2.841	296.9645
LysoPC(20:4(8Z,11Z,14Z,17Z)/0:0)	9.364_543.	POS	9.364	543.3333
3-Hydroxyhexadecadienoylcarnitine	9.72_411.2	POS	9.72	411.2986
(2S,3R,4S,5R)-2-{[2-hydroxy-22-(2-hydroxypropan	9.422_618.	POS	9.422	618.3744
[(1R)-1-[(2S,4aR,4bS,7S,8aS)-7-hydroxy-2,4b,8,8-t	8_526.313	NEG	8	526.3139
Polysulfide; (Sulfide)n; Polysulfane;	0.677_121.	POS	0.677	121.9174

**4-Ketoalloxanthin**	4.883_578.	POS	4.883	578.378
**Phenyl 1-hydroxy-2-naphthoate**	2.084_264.	POS	2.084	264.078
**Penicisteroid G**	8.706_506.	POS	8.706	506.3614
**LMST01031106**	9.76_414.3	POS	9.76	414.3499
**4-Hydroxy-6-methyl-2-pyrone**	3.231_126.	POS	3.231	126.0308
**2-[5-[(3,5-Dimethyl-1H-pyrrol-2-yl)methylidene]-**	3.892_317.	POS	3.892	317.1536
**[(1S,3S,7R,8R,8As)-8-[2-[(4R)-4-hydroxy-6-oxooxa**	7.213_404.	NEG	7.213	404.2564
**Fluocortin butyl**	5.433_446.	POS	5.433	446.2464
**Trimethoprim**	5.746_290.	POS	5.746	290.137
**Lamivudine-triphosphate**	0.678_468.	NEG	0.678	468.9521
**Neplanocin A**	4.799_263.	POS	4.799	263.1005
**Sulfisoxazole**	3.42_267.0	POS	3.42	267.0676
**17a-Ethynylestradiol**	9.064_296.	POS	9.064	296.1776
**Pradigastat**	4.837_455.	POS	4.837	455.1808
**(1S-Endo)-5-(4-Chloro-3-methylphenyl)-1-((4-me**	8.688_475.	POS	8.688	475.2395
**Valaciclovir**	9.071_324.	POS	9.071	324.1552
**Thiophanate-methyl**	3.448_342.	POS	3.448	342.0455
**19-epi-cytochalasin P1**	8.246_541.	NEG	8.246	541.2701
Labilithrix_luteola				
**alpha-Tocopherol acetate**	8.507_494.	POS	8.507	494.3723
**11,12-Dimethoxydihydrokawain**	5.739_292.	POS	5.739	292.131
**Malathion**	5.44_330.0	POS	5.44	330.0374
**Picolinic acid**	0.661_123.	NEG	0.661	123.0321
**Artemisinin**	6.269_264.	POS	6.269	264.1362
**Cholesteryl glucoside**	10.02_548.	POS	10.02	548.4078
**Amustaline**	6.79_433.1	POS	6.79	433.1308
Other				
**Onnamide A; Onnamide;**	7.548_793.	POS	7.548	793.4509
**3-methoxy-2-(3-methylbut-2-enyl)-5-pentylphen**	9.31_262.1	POS	9.31	262.1933
**DG(20:3(8Z,11Z,14Z)/14:0/0:0)**	11.8_590.4	POS	11.8	590.4917
**Bisucaberin**	3.888_400.	POS	3.888	400.2323
**PC(17:2(9Z,12Z)/0:0)**	9.729_505.	NEG	9.729	505.3166
**p-Chlorodisopyramide**	6.889_373.	POS	6.889	373.192
**4-(Aminomethyl)benzoic acid**	9.069_151.	POS	9.069	151.0635
**N-(4-Aminobenzoyl)-L-glutamic acid**	3.209_266.	POS	3.209	266.0902
**Glyceryl monolinoleate**	9.645_354.	POS	9.645	354.2769
**1,1-Bis(4-chlorophenyl)prop-2-yn-1-ol**	0.682_276.	NEG	0.682	276.0121
**Buparlisib**	5.463_410.	POS	5.463	410.169
**NA**	5.948_272.	NEG	5.948	272.1734
**OOB-PE**	9.494_563.	NEG	9.494	563.3231
**N2-Succinyl-L-arginine; (2S)-2-(3-Carboxypropan**	9.565_274.	POS	9.565	274.1273
**1-Piperidinyloxy, 4-(((dodecyloxy)hydroxyphosphi**	8.108_420.	POS	8.108	420.2878
**Obliquine;**	8.573_448.	POS	8.573	448.2003
**Militarinone C;**	6.644_423.	POS	6.644	423.2412
**2-hydroxy-4-methoxy-3,5-bis(3-methylbut-2-eny**	7.695_443.	POS	7.695	443.2673
Acetatifactor_muris				
**Lincomycin B**	3.961_392.	POS	3.961	392.1978
**4-hydroxy-8cis-sphingenine**	8.118_315.	POS	8.118	315.2772
**(S)-3-(2-Methylphenoxy)propane-1,2-diol**	5.058_182.	POS	5.058	182.0946
**Trillin**	8.84_576.3	POS	8.84	576.3666
**Chenodeoxycholic Acid**	8.856_438.	NEG	8.856	438.2982
Other				
**Furapyrimidone**	5.047_238.	POS	5.047	238.0699
**18-Aminoabieta-8,11,13-Triene Sulfate**	8.469_383.	POS	8.469	383.2129
**1,8-Naphthyridin-2(1H)-one**	2.439_146.	NEG	2.439	146.0481
**1H-2-benzopyran-1-one, 6,8-dihydroxy-3-methyl**	3.29_192.0	NEG	3.29	192.0423
**Cer(d18:1(8E)/16:0(2OH[R]))**	10.764_55	POS	10.764	553.5074
**2'-Fluoro-2',3'-dideoxyinosine**	5.227_254.	NEG	5.227	254.0821

**2-[3-[(E)-6-acetyloxy-2-hydroxy-6-methyl-3-oxol**	8.993_612.	POS	8.993	612.2941
**Hellebrigenin-3-(14-hydroxy-6Z-tetradecenoate)**	10.016_64(	POS	10.016	640.3952
**2-trans,4-cis-Decadienoylcarnitine**	7.541_311.	POS	7.541	311.2096
**GM4(d18:1/16:0)**	9.557_990.	POS	9.557	990.665
Brachybacterium_alimentarium				
**Myxalamid S;**	9.594_419.	POS	9.594	419.3036
**PI(22:0/0:0)**	9.322_656.	POS	9.322	656.3906
**2,2'-{Oxybis[(2,1-phenylene)oxy]}bis(N,N-dicyclohe**	9.141_644.	NEG	9.141	644.416
**n6-iminoethyl-l-lysine**	3.51_187.1	POS	3.51	187.132
**1-O-Acetyl britannilactone**	7.501_308.	POS	7.501	308.1621
**Tebuquine**	7.439_465.	POS	7.439	465.1395
**7-Ketolithocholic acid**	8.301_390.	POS	8.301	390.2769
**(2E,4E)-1-[(2R,6S,14S,22S,25R)-25-(3,3-dimethylox**	7.938_530.	POS	7.938	530.2674
**LMST01010326**	3.508_500.	POS	3.508	500.333
**Eptastigmine**	8.114_359.	POS	8.114	359.2568
**6-Hydroxyazapropazone**	2.874_316.	POS	2.874	316.1531
**vamorolone**	5.89_356.1	POS	5.89	356.1987
**Annopurpuricin E**	11.73_572.	POS	11.73	572.4795
**2-Naphthalen-1-ylbutanoic acid**	3.444_214.	POS	3.444	214.0994
**Pulvomycin;**	9.321_838.	POS	9.321	838.4504
**N-(4-Amino-2,5-diethoxyphenyl)benzamide**	9.332_300.	NEG	9.332	300.1476
**Semiplenamide A**	8.749_365.	POS	8.749	365.3294
**(5S)-5-(7-Benzofuranyl)-8-chloro-3-methyl-1,2,4,!**	5.594_327.	POS	5.594	327.1039
**4H-Thieno[3,2-b]pyrrole-5-carboxylic acid**	3.457_167.	POS	3.457	167.0043
**(2Z)-2-[4-[2-[2-(3,4-dihydroxyphenyl)ethoxy]-2-o**	5.416_592.	POS	5.416	592.142
**3-dehydroecdysone**	8.916_462.	POS	8.916	462.2966
**4-Amino-5-methylamino-2',7'-difluorescein**	2.883_412.	POS	2.883	412.0881
**ISOSAFROLE**	4.94_162.C	POS	4.94	162.0682
**3'-Deoxydihydrostreptomycin 3''-phosphate;**	5.41_647.2	POS	5.41	647.2497
**Renzapride**	5.954_323.	NEG	5.954	323.1403
**(2E,4E,8E)-7,13-dihydroxy-4,8,12-trimethyltetradec**	6.441_313.	POS	6.441	313.2251
**PS(15:0/22:1(11Z))**	9.489_803.	NEG	9.489	803.5708
Pseudokineococcus_lusitanus				
Flavisolibacter_rigui				
Jeotgalicoccus_pinnipedialis				
Other				
Domibacillus_enclensis				
Other				
Roseiarcus_fermentans				
Chryseobacterium_pallidum				
Shinella_zoogloeoides				
Other				
Methylobacterium_isbiliense				
Aureimonas_altamirensis				
Ammoniibacillus_agariperforans				
Diplorickettsia_massiliensis				
Other				
Geobacillus_thermodenitrificans				
Other				
Saccharopolyspora_gloriosae				
**Cuscutic resinoside A**	9.66_632.3	POS	9.66	632.3753
**Cortisol 21-mesylate**	6.493_440.	NEG	6.493	440.1869
Other				
Pseudonocardia_antitumoralis				
**9-Hydroxymethyl-10-carbamoylacridan**	6.842_254.	POS	6.842	254.1055
**11-Dehydro thromboxane B2**	5.915_368.	NEG	5.915	368.2198
**1-Cyclohexene-1-carboxylic acid**	4.52_126.C	POS	4.52	126.0682
**2-Arachidonoyl glycerol**	9.612_378.	POS	9.612	378.277

**5-Butyl-5-cycloheptenylbarbituric acid**	8.136_292.	POS	8.136	292.1784
**Lipoxin C4**	6.813_641.	POS	6.813	641.295
**Guan-fu base A**	4.394_429.	POS	4.394	429.2152
Other				
**Lithocholic acid**	8.652_422.	NEG	8.652	422.3033
**1,5-Diphenylcarbazide**	3.53_242.1	NEG	3.53	242.1156
**4,4-Difluoro-17beta-hydroxy-17alpha-methyl-and**	9.367_338.	NEG	9.367	338.2045
**3-[1-[3-(Dimethylamino)propyl]-5-methoxy-1H-ir**	8.617_442.	NEG	8.617	442.2027
**Cysteine allyl ester**	2.188_161.	POS	2.188	161.0512
**Camphorsulfonic acid**	3.461_232.	POS	3.461	232.0773
**D-Phe-L-Pro-L-Arg-(Chloromethyl) ketone**	3.227_478.	POS	3.227	478.2103
**Bialaphos**	7.139_323.	POS	7.139	323.1302
Paraprevotella_clara				
**Clebopride**	6.179_373.	POS	6.179	373.1558
**9-oxo-7E-hexadecenoic acid**	8.832_268.	NEG	8.832	268.2036
**Epothilone A**	6.769_493.	NEG	6.769	493.2496
**Ethyl isovalerate;**	5.752_130.	NEG	5.752	130.0995
**L-Alanyl-L-glutamate;**	0.743_218.	POS	0.743	218.0905
**Lofepramine**	10.137_41	POS	10.137	418.1807
**N-(3-Oxododecanoyl)-L-homoserine lactone**	5.659_297.	POS	5.659	297.1941
**Demethylzeylasteral**	7.109_480.	POS	7.109	480.2517
**PE(16:0/20:3(8Z,11Z,14Z))**	11.685_74	POS	11.685	741.5312
**Glycylproline**	1.008_172.	POS	1.008	172.0851
Ruminococcus_lactaris				
**Caudatin**	8.243_490.	POS	8.243	490.2937
**2-[[3,4-dihydroxy-4-(hydroxymethyl)oxolan-2-yl]c**	5.85_446.2	NEG	5.85	446.2152
Microbacterium_phyllosphaerae				
**3beta-Deoxycholic acid**	8.908_414.	POS	8.908	414.2747
**Zacopride**	4.944_309.	POS	4.944	309.1245
**Tetranactin;**	9.272_792.	POS	9.272	792.4988
**Androstane-3,17-diol dipropionate**	8.854_404.	POS	8.854	404.2927
**(9E,12E)-N-(2-Hydroxyethyl)octadeca-9,12-dienan**	8.614_323.	NEG	8.614	323.2826
**Methadone**	5.733_309.	POS	5.733	309.1938
**Epilincomycin**	6.037_406.	NEG	6.037	406.214
**Osimertinib**	9.41_499.2	POS	9.41	499.2682
**Halofenozide**	8.942_330.	POS	8.942	330.1143
Sphingobacterium_faecium				
**4- Carboxyphenylglycine**	2.209_195.	NEG	2.209	195.0531
**Primary fluorescent chlorophyll catabolite;**	8.408_628.	POS	8.408	628.2895
Deinococcus_wulumuqiensis				
**Bacillamidin A**	6.236_329.	POS	6.236	329.2201
**OHODA-PA**	8.048_646.	POS	8.048	646.3828
**Sulcotrione**	4.592_328.	POS	4.592	328.0188
**di-trans,poly-cis-Pentaprenyl diphosphate; ditrans,**	8.801_518.	POS	8.801	518.2562
**Santowhite**	8.743_382.	POS	8.743	382.2872
**Fluphenazine**	4.819_437.	POS	4.819	437.1736
**LMST01031192**	9.383_408.	POS	9.383	408.3029
**3-(3-Methylbutyl)tricetin 5-neohesperidoside**	5.436_680.	POS	5.436	680.2297
**Rhizocticin B;**	7.453_450.	POS	7.453	450.1988
**Kurilensoside H**	7.827_614.	NEG	7.827	614.3682
**3,4-dimethyl-5-carboxyethylene-2-furanpentanoic**	7.24_266.1	POS	7.24	266.1154
**Monocyclohexyl phthalate**	5.741_248.	POS	5.741	248.105
**neosolaniol**	4.979_446.	POS	4.979	446.1566
**3,6-diphenylcyclohexane-1,2-diol**	5.345_306.	POS	5.345	306.1013
**3-Deoxo-3Beta-Hydroxymexicanolide 16-Enol Eth**	4.677_506.	POS	4.677	506.2303
**Calonysterone**	5.909_476.	NEG	5.909	476.2772
**4H-1-Benzopyran**	4.875_132.	POS	4.875	132.0577
**2,3-Dinor-11**	8.909_308.	POS	8.909	308.1987

**(9S)-9-Acetyl-7-[(2R,4S,6S)-4-amino-5-hydroxy-6**	7.044_497.	POS	7.044	497.1705
**icos-19-ene-1,2,4-triol**	8.62_345.3	POS	8.62	345.3243
Bilophila_wadsworthia				
**LMPK12110183**	5.228_452.	NEG	5.228	452.1482
**Corrin**	8.575_306.	NEG	8.575	306.1831
Nitrolancea_hollandica				
**3-Butylidene-4,5-dihydrophthalide**	6.819_190.	POS	6.819	190.0996
Bacteroides_ovatus				
**18-bromo-9E,17E-Octadecadiene-7,15-diynoic aci**	1.432_350.	POS	1.432	350.0893
**Cannabichromene**	10.13_314.	POS	10.13	314.2247
**(**	8.215_198.	NEG	8.215	198.162
**2-Methoxy-10H-phenothiazine**	0.726_229.	NEG	0.726	229.0562
**Pyrimidifen;**	6.419_377.	POS	6.419	377.1871
**Pregnanediol 3-O-glucuronide**	7.884_496.	NEG	7.884	496.3033
Flavobacterium_gyeonganense				
Pseudonocardia_spinosispora				
Herpetosiphon_geysericola				
Lysinibacillus_sphaericus				
Nitrospira_moscoviensis				
Paracraurococcus_ruber				
Other				
Hungatella_effluvii				
Corynebacterium_confusum				
Tetragenococcus_halophilus				
**Binapacryl;**	4.858_322.	POS	4.858	322.1164
**NCGC00169702-02!(5R)-5-hydroxy-1-(4-hydroxy-**	7.735_316.	POS	7.735	316.1647
**Alpha-linolenyl carnitine**	8.53_421.3	POS	8.53	421.3194
Acinetobacter_gandensis				
**Ethyl 4-decenoate**	8.393_198.	NEG	8.393	198.1621
**3-Hydroxy-4-aminopyridine**	0.906_110.	POS	0.906	110.0482
**1,2,3,4-Tetrahydronaphthalene**	8.452_132.	POS	8.452	132.094
**2-Hexenoylcarnitine**	4.006_257.	POS	4.006	257.1628
**Cotinine N-oxide**	1.028_192.	POS	1.028	192.0902
**Milbemectin A4**	8.869_542.	NEG	8.869	542.3269
**Sarcoehrendin D**	6.841_508.	POS	6.841	508.2686
**Metoclopramide**	8.978_299.	POS	8.978	299.1398
**2-Nonyl-1,4-dioxine**	10.185_21(	POS	10.185	210.1622
**LysoPC(18:2(9Z,12Z)/0:0)**	9.391_519.	POS	9.391	519.3328
**Rifabutin**	7.934_846.	POS	7.934	846.438
**Tetrahydrodeoxycorticosterone**	8.844_334.	POS	8.844	334.2508
**SCHRADAN**	6.571_286.	POS	6.571	286.1317
**terrein**	3.16_171.C	POS	3.16	171.0898
**[(2R,3S,4S,5R,6R)-6-[(2S,3S,4S,5R)-4-acetyloxy-3-h**	3.944_514.	POS	3.944	514.1706
**Benin**	3.925_337.	POS	3.925	337.1193
**LMPK12010431**	4.592_481.	POS	4.592	481.0983
**[D-Lys3]-GHRP-6**	7.781_929.	NEG	7.781	929.5014
**Cucurbitacin H**	8.553_534.	NEG	8.553	534.3186
**2,5-Dihydroxy-1-octadec-9-enoyloxypyrrole-3-su**	6.407_459.	NEG	6.407	459.2289
**Cholest-4-en-3-one**	11.299_38	POS	11.299	384.3393
**Istamycin C1;**	8.257_431.	POS	8.257	431.2759
**Cafestol**	8.566_316.	POS	8.566	316.2037
**STROBILACTONE A**	3.434_266.	POS	3.434	266.1506
**Dehydroepiandrosterone (DHEA)**	6.831_270.	POS	6.831	270.1984
**1-(4-hydroxyphenyl)-3-[(2R,3R,4S,5S,6R)-3,4,5-trih**	3.497_328.	POS	3.497	328.1169
**7-Hydroxystaurosporine**	6.037_482.	NEG	6.037	482.1975
**6-Methyl-1'-(2-(5-methyl-2-phenyloxazol-4-yl)et**	6.952_417.	POS	6.952	417.2052
**(1R,4aS,5R,8aS)-5-(5-hydroxy-3-methylpentyl)-1,4**	9.823_339.	POS	9.823	339.2772
Roseburia_inulinivorans				

Suberic acid	5.046_156.	POS	5.046	156.0789
Falecalcitriol	6.826_524.	NEG	6.826	524.2728
Pregnan-20-one	8.463_302.	POS	8.463	302.261
Fluroxene	0.727_126.	NEG	0.727	126.0294
Cuminaldehyde	7.934_148.	POS	7.934	148.089
Methacrifos;	0.727_240.	NEG	0.727	240.0221
(2S,3S,4S,5S)-2-(5-Amino-7-methyl-2,6,7,9,11-per	1.083_320.	NEG	1.083	320.1219
Metribuzin	8.226_214.	POS	8.226	214.0884
5,7-dihydroxy-2-phenyl-6-[3,4,5-trihydroxy-6-(hy	6.073_548.	NEG	6.073	548.1524
Nifedipine	4.61_346.1	POS	4.61	346.1162
4-[(2,4-Dihydroxy-3,3-dimethylbutanoyl)amino]bu	4.013_233.	POS	4.013	233.1265
QUINGESTANOL	8.783_366.	POS	8.783	366.2559
3-Decenoic acid	7.273_170.	NEG	7.273	170.1308
5alpha-Androstane-3beta,17beta-diol diacetate; 3l	9.776_376.	NEG	9.776	376.2616
triprenyl phenol	7.925_298.	POS	7.925	298.2296
Bilirubin	9.158_584.	NEG	9.158	584.2641
2-Hydroxymyristic acid	9.133_244.	POS	9.133	244.2038
Chenodeoxycholic acid 3-sulfate	7.016_472.	NEG	7.016	472.2494
Andolast	3.966_333.	POS	3.966	333.1074
PG(20:1(11Z)/22:2(13Z,16Z))	8.302_856.	NEG	8.302	856.6216
Octadecaneperoxoic acid	9.954_300.	POS	9.954	300.2662
OKHdiA-PG	8.473_664.	POS	8.473	664.3218
Coenzyme Q1	5.804_250.	POS	5.804	250.1207
Brevetoxin A;	7.908_866.	POS	7.908	866.4788
2-Methyl-1-hydroxypropyl-ThPP; 2-Methyl-1-hyd	4.376_497.	NEG	4.376	497.1023
D-Ribose	0.733_172.	POS	0.733	172.035
Dehydrowarfarin	2.584_306.	POS	2.584	306.0885
Quinacrine	8.173_399.	POS	8.173	399.2077
PG(18:2(9Z,12Z)/0:0)	7.426_508.	NEG	7.426	508.2781
4,4'-Diapophytoene/ Dehydrosqualene	11.607_40	POS	11.607	408.3756
Gibberellin A4 methyl ester	7.582_346.	POS	7.582	346.1781
Thiolactomycin;	1.798_210.	NEG	1.798	210.0721
Brainesteroside C	8.064_622.	POS	8.064	622.3366
(3S,4S,6aR,6bS,8R,8aR,12aS,14bR)-8-hydroxy-4,6a,	9.041_634.	POS	9.041	634.3694
10-fluoro-capric acid	5.557_190.	POS	5.557	190.136
Dexanabinol	8.853_386.	POS	8.853	386.2821
Arg-Pro-Pro-Gly-Phe-Ser-Pro	5.742_756.	POS	5.742	756.3937
NCGC00347666-02!2,4-dihydroxy-6-methyl-3-[(2E	6.795_392.	POS	6.795	392.1947
6-Chloromelatonin	3.088_266.	POS	3.088	266.0826
8-Hydroxyluteolin 8-glucoside-3'-sulfate	4.561_544.	NEG	4.561	544.0513
Ethanone, 1-(4-butylphenyl)-	8.498_176.	POS	8.498	176.1203
m-Xylene	8.495_106.	POS	8.495	106.0783
LMST04030049	8.968_448.	NEG	8.968	448.3191
PG(19:0/0:0)	8.764_526.	POS	8.764	526.3295
2-furan-octanoic acid	6.746_210.	POS	6.746	210.1259
Squalene	12.147_41(	POS	12.147	410.3913
PI(22:2(13Z,16Z)/22:1(11Z))	8.539_972.	POS	8.539	972.6653
(Z)-7-[(1R,3S,4S,5R)-3-[(E,3R)-3-Hydroxyoct-1-en	9.618_366.	NEG	9.618	366.223
Nevirapine	5.255_266.	POS	5.255	266.1155
Sphingosyl-phosphocholine;	9.048_465.	POS	9.048	465.3458
2-Methyl-3-methoxy-4-phenylbutyric acid	7.728_208.	POS	7.728	208.1102
Callytriol C;	6.551_348.	NEG	6.551	348.1719
Cholic Acid, Methyl Ester	8.853_422.	POS	8.853	422.3034
Mianserin	3.517_264.	POS	3.517	264.1634
(6Ar,10aR)-1-hydroxy-6,6-dimethyl-3-(2-methylo	8.423_400.	POS	8.423	400.2614
Milbemectin A3	7.87_528.3	NEG	7.87	528.3038
Mycalamide A;	9.527_503.	POS	9.527	503.2712
2-(3,4-Dihydro-2,2-dimethyl-6-nitro-2H-1,4-benz	4.833_301.	POS	4.833	301.1059

**Methylenediurea;**	0.742_132.	POS	0.742	132.065
**Piceatannol**	4.829_244.	POS	4.829	244.0713
**4-Pyridoxic acid**	2.872_183.	NEG	2.872	183.0532
**Propanoic acid, 3-((((2-(1,1-dimethylethyl)-5-meth**	4.609_384.	POS	4.609	384.0632
**Cianopramine**	3.426_305.	POS	3.426	305.1903
**N-(2,4-Dimethylphenyl)formamide**	6.658_149.	POS	6.658	149.0842
**13,14-Dihydro prostaglandin E1**	8.931_338.	POS	8.931	338.2456
**N-decanoyl-homoserine lactone**	6.834_255.	POS	6.834	255.1833
**Radiprodil**	5.68_397.1	POS	5.68	397.142
**NCGC00380806-01!6-(diaminomethylideneamino)-**	8.561_556.	POS	8.561	556.3589
**Pentahomomethionine; L-Pentahomomethionine;**	5.66_219.1	POS	5.66	219.1295
**Anaerobilin;**	10.269_57(	POS	10.269	576.2742
Clostridium_leptum				
**PG(22:0/22:4(7Z,10Z,13Z,16Z))**	8.985_882.	POS	8.985	882.6334
**Indole-3-carboxaldehyde**	3.793_145.	POS	3.793	145.0529
**2-[(1r,3s)-3-Hydroxycyclohexyl]-5-(2-methyloctan**	8.386_318.	POS	8.386	318.2558
**7-[(3R)-3-(Aminomethyl)-4-methoxyiminopyrrolid**	5.08_389.1	POS	5.08	389.1507
**4-(1-Isopropyl-2-methyl-1H-imidazol-5-yl)-N-(4-**	5.709_371.	POS	5.709	371.14
**Penazetidine A**	8.975_369.	POS	8.975	369.3607
**(1S,2R,5R,6R,10R,13S,15S)-5-[(2R,3E,5R)-5,6-dimet**	9.405_428.	POS	9.405	428.3292
**4-Hydroxypropranolol glucuronide**	8.194_451.	POS	8.194	451.1859
Other				
Lactococcus_garvieae				
**5-S-Cysteinyl-D-dopa**	0.642_316.	NEG	0.642	316.0728
**Taraxerone**	9.843_424.	POS	9.843	424.3706
**(2S)-2-((3-((4-(4-Fluorophenoxy)phenyl)methylcar**	3.922_451.	POS	3.922	451.1783
**3-((((-Oxoheptyl)amino)acetyl)amino)methyl-7-ox**	9.23_422.2	POS	9.23	422.2799
**[5-hydroxy-3-(hydroxymethyl)-2-oxo-6-propan-2**	9.072_298.	POS	9.072	298.1784
**Mepirizole**	3.603_234.	POS	3.603	234.1106
**Lauroylcarnitine**	7.843_343.	POS	7.843	343.272
**30:6(12Z,15Z,18Z,21Z,24Z,27Z)**	8.447_440.	POS	8.447	440.3658
**2-(Hydroxysulfanyl)hercynine; Nalpha,Nalpha,Nalp**	5.289_246.	POS	5.289	246.0924
**alpha-Tocotrienol**	9.817_424.	POS	9.817	424.3341
Prevotella_paludivivens				
Brachymonas_denitrificans				
Chthoniobacter_flavus				
**Octadeca-9,12-dienal**	9.851_264.	POS	9.851	264.2453
**Dapsone**	3.56_248.0	POS	3.56	248.0629
**Mulberrin**	3.485_422.	POS	3.485	422.1724
**Cyclopentenyl cytosine**	2.824_239.	POS	2.824	239.0908
**N-Isovalerylglycine**	2.482_159.	NEG	2.482	159.0896
**Glutathionylspermine;**	8.494_491.	NEG	8.494	491.2913
**Petromylidene A**	9.195_540.	NEG	9.195	540.3115
**Trandolapril**	8.906_430.	POS	8.906	430.2488
**2,3,4,5-tetranor-thromboxane B1**	9.071_316.	POS	9.071	316.189
Clostridium_butyricum				
**Chlorsulfuron**	3.479_357.	POS	3.479	357.0286
**(2R,3S,5R)-2-[6-[[(1S,2S)-2-Hydroxycyclopentyl]an**	3.94_351.1	POS	3.94	351.1525
**Dokdolipid B**	9.868_460.	NEG	9.868	460.3038
**Desoxycortone**	7.092_330.	POS	7.092	330.2192
**Ozolinone**	3.217_256.	POS	3.217	256.0891
Devosia_epidermidihirudinis				
**(2R)-5-methoxy-2-methyl-2,3,8,9-tetrahydrofuro[**	5.972_234.	POS	5.972	234.0893
Brevundimonas_lenta				
**(1R,4S,7S,10R,13R,16S)-13-(1H-Indol-3-ylmethyl)-**	8.526_760.	NEG	8.526	760.3354
**Xestoaminol B**	10.36_225.	POS	10.36	225.2093
**2-[5-[2-[2-[5-(2-hydroxybutyl)oxolan-2-yl]propan**	8.11_414.2	POS	8.11	414.2614
**NCGC00385003-01!4-[3-methyl-5-(5,6,7-trihydrox**	8.109_388.	POS	8.109	388.2613

**(2E,4E)-N-[2-[[(2R,3R,4R,5R,6S)-2-(1,2-Dihydroxye**	8.096_589.	POS	8.096	589.3246
Rhodococcus_cerastii				
**Cholestane-3,7,12,25-tetrol-3-glucuronide**	8.759_612.	NEG	8.759	612.3893
**7-[3-(4-Acetyl-3-hydroxy-2-propylphenoxy)-2-hy**	7.571_498.	POS	7.571	498.1905
**PS(13:0/18:3(6Z,9Z,12Z))**	8.062_715.	POS	8.062	715.4407
**PA(22:0/0:0)**	8.621_494.	NEG	8.621	494.3355
**hydrocortisone hemisuccinate**	6.767_462.	NEG	6.767	462.2253
**MG(18:2(9Z,12Z)/0:0/0:0)**	8.56_354.2	POS	8.56	354.2771
**2,10,15,20-Tetraphenyl-21,22,23,24-tetrahydropor**	8.043_696.	POS	8.043	696.2212
**Monepalin B**	8.033_758.	POS	8.033	758.1702
**6-{3-[(Pyridin-2-yl)disulfanyl]propanamido}hexan**	2.298_328.	POS	2.298	328.0922
**((1R,2R,5R)-2-(2,6-Dimethoxy-4-(2-methyloctan-2**	9.094_414.	POS	9.094	414.3135
**Nocardicin A**	3.902_500.	POS	3.902	500.1548
**N-hexanoyl histidine**	3.864_253.	POS	3.864	253.1426
**L-Proline, 1-(N2-(1-carboxy-3-phenylpropyl)-N6-**	7.356_524.	POS	7.356	524.2628
**7-hydroxy-1,4a-dimethyl-9-oxo-7-propan-2-yl-2,**	6.719_372.	POS	6.719	372.1717
**Sarcoehrendin J**	7.843_454.	POS	7.843	454.2933
**Precorrin 5;**	7.935_922.	POS	7.935	922.3493
Ruminococcus_flavefaciens				
**(2S,3R,4S,5S,6R)-2-[4-(2-hydroxyethyl)phenoxy]-6**	3.97_317.1	POS	3.97	317.1475
**Poststatin**	7.585_541.	POS	7.585	541.3451
**PI(22:6(4Z,7Z,10Z,13Z,16Z,19Z)/19:0)**	8.195_924.	POS	8.195	924.5717
**Astaxanthin**	8.999_596.	POS	8.999	596.3829
**albocycline**	8.314_330.	POS	8.314	330.1806
**8-Methyl-8-azabicyclo[3.2.1]oct-3-yl 1H-indole-3**	8.411_284.	POS	8.411	284.1525
**Meproscillarin**	8.266_544.	NEG	8.266	544.3059
**2,6-Di-tert-butyl-1,4-benzoquinone**	9.171_220.	POS	9.171	220.1466
**2-(14,15-Epoxyeicosatrienoyl) glycerol**	9.406_376.	POS	9.406	376.2614
**Lamiide**	4.495_422.	NEG	4.495	422.1439
**trans-1,4-Bis[[1-cyclohexyl-3-(4-dimethylamino pl**	12.352_63	POS	12.352	630.4629
**Amitrole**	1.03_84.04	POS	1.03	84.04369
**NPE_255.1491_11.6**	8.719_254.	POS	8.719	254.142
**PS(15:1(9Z)/16:1(9Z))**	8.118_717.	POS	8.118	717.4559
**Eltrombopag**	6.208_442.	NEG	6.208	442.1662
**AB-Pinaca**	9.071_330.	POS	9.071	330.2046
**PA(18:3(6Z,9Z,12Z)/0:0)**	4.594_432.	POS	4.594	432.2295
**Isobrucein A;**	3.434_522.	POS	3.434	522.2081
Parabacteroides_merdae				
**alpha-[3-(Nitrosoamino)propyl]-3-pyridinemethan**	3.223_195.	POS	3.223	195.101
**1,2,3,4-Tetrahydroisoquinolin-1-yl formate**	0.739_177.	POS	0.739	177.0792
**Psoralidin**	3.226_336.	POS	3.226	336.099
**Nintedanib**	8.141_539.	NEG	8.141	539.2546
**(2R)-2,5,8-Trimethyl-2-(4,8,12-trimethyltrideca-3,**	9.775_410.	POS	9.775	410.3186
**(4S,7S,12Br)-7-[[(2R)-2-acetylsulfanyl-3-phenylpr**	8.513_480.	NEG	8.513	480.1735
**N-Acetyl-D-mannosamine**	8.3_221.09	POS	8.3	221.0902
**Rafabegron**	3.908_402.	POS	3.908	402.1359
**Rehmannic acid**	10.077_55	POS	10.077	552.3802
**Arctiin**	3.909_551.	POS	3.909	551.2378
**1,2-Naphthalenedicarboxaldehyde**	4.588_184.	POS	4.588	184.0527
**(9cis)-Retinal**	9.45_284.2	POS	9.45	284.214
**2-Naphthalenol, 1-[2-[4-[2-(dimethylphenyl)diaze**	9.646_408.	POS	9.646	408.1963
**GlcCer(d16:2(4E,6E)/24:0(2OH))**	8.548_797.	POS	8.548	797.6389
**(S)-4-(4-((2-(3,5-Dimethylphenyl)pyrrolidin-1-yl)N**	3.181_418.	POS	3.181	418.2064
**Hexahomomethionine; L-Hexahomomethionine;**	6.153_233.	POS	6.153	233.1451
**N-Carbamimidoyliminomethanethioamide**	2.435_116.	POS	2.435	116.0157
**11-Ketotestosterone**	5.769_302.	POS	5.769	302.1882
**Propafenone**	4.698_341.	POS	4.698	341.1991
**4-Ethylnaphthalen-1-yl-(1-pentylindol-3-yl)metha**	3.502_369.	POS	3.502	369.2092

**Dihydromuronic acid**	8.162_386.	POS	8.162	386.2668
**Pradofloxacin**	5.437_396.	POS	5.437	396.1587
**NCGC00347585-02!(1,9,9b-trihydroxy-6,6,9a-trime**	6.296_364.	POS	6.296	364.2248
**(E)-4-[[(1R)-2-[[(2R)-2-(2-Adamantyloxycarbonyla**	9.327_612.	NEG	9.327	612.2965
**(1R,2E,7R,10E,12S,13S,15R)-12,15-dihydroxy-7-me**	7.046_280.	POS	7.046	280.1675
**Crotonic acid**	1.541_86.0	POS	1.541	86.03693
**(-)-Epigallocatechin**	4.853_306.	POS	4.853	306.0714
**Triticonazole**	5.073_317.	POS	5.073	317.1297
**5-Fluoro-3-[3-[4-(5-methoxypyrimidin-4-yl)pipera**	8.765_369.	POS	8.765	369.1973
**(3beta,5alpha,12beta)-Cholestane-3,7,12,25-tetrol**	8.682_436.	POS	8.682	436.3555
**Northiaden**	3.862_281.	POS	3.862	281.1263
**Benzoyl L-arginine methyl ester**	7.186_292.	POS	7.186	292.1536
**Phorbol 12-tiglate 13-decanoate;**	9.471_600.	POS	9.471	600.3646
**Methyl cis-9-octadecenoate**	8.552_296.	POS	8.552	296.2714
**2,3,8,9,10-pentamethoxy-6a,11a-dihydro-6H-[1]be**	5.325_396.	POS	5.325	396.1166
**Fendosal**	8.134_381.	POS	8.134	381.1357
**Cilligen**	5.318_462.	NEG	5.318	462.1922
**Zabicipril**	9.133_416.	POS	9.133	416.2329
**2-O-(2-O-isovaleryl-beta-D-glucopyranosyl) atrac**	8.047_580.	POS	8.047	580.3226
**Buspirone**	7.484_385.	POS	7.484	385.2463
Acinetobacter_seifertii				
Lactobacillus_saerimneri				
**5-hydroxy saxagliptin**	6.852_331.	POS	6.852	331.1894
**16-DHPS**	10.189_39	POS	10.189	394.1807
**Deoxycholic acid**	8.958_784.	POS	8.958	784.5853
**2-[4,7,10-Tris(carboxymethyl)-6-[4-[3-(2,5-dioxop**	8.994_626.	POS	8.994	626.2917
**Glipizide**	5.659_445.	NEG	5.659	445.1798
**1-tauro-dinor-PGE1**	6.19_433.2	NEG	6.19	433.2136
**N-(1-Carboxy-3-carboxanilidopropyl)alanylproline**	3.44_391.1	POS	3.44	391.1743
**gamma-Glutamylthreonine**	0.744_248.	POS	0.744	248.1012
**Probenecid**	6.035_285.	POS	6.035	285.1035
**NCGC00384531-01!3-(2,4-dihydroxyphenyl)-7-hyc**	3.423_408.	POS	3.423	408.1929
**Sibrafiban**	3.507_420.	POS	3.507	420.2008
**3beta-Hydroxy-5alpha-pregnan-20-one acetate; 3**	8.048_360.	POS	8.048	360.2662
**bergenin**	3.509_328.	POS	3.509	328.0789
**NCGC00385810-01! [IIN-based: Match]**	3.749_392.	POS	3.749	392.1254
**Piromidic acid**	5.9_288.12	POS	5.9	288.1224
**7-Propoxy-chromen-2-one**	7.594_204.	POS	7.594	204.0789
**Phylanthoside**	8.613_804.	POS	8.613	804.3223
**4,5-dihydroxy-3-propylcyclopent-2-en-1-one**	2.732_156.	POS	2.732	156.079
**3[N-Morpholino]propane sulfonic acid**	3.449_209.	POS	3.449	209.0727
**3,4-Dihydroxyphenylpropionic acid**	8.314_164.	POS	8.314	164.0476
Bacteroides_fragilis				
**Pyrazolo(1,5-a)pyrimidine-3-carbonitrile, 4,5-dihyc**	8.03_506.2	POS	8.03	506.2059
**punaglandin 3**	3.466_496.	POS	3.466	496.1888
**7,7-Diphenyl-2-(1-imino-2-(2-methoxyphenyl)eth**	9.646_438.	POS	9.646	438.2296
**4-Fluoro-3-phenoxybenzoic acid**	1.451_232.	POS	1.451	232.053
**Fenflumizole**	5.021_392.	POS	5.021	392.1332
**Cefoxitin**	3.956_427.	POS	3.956	427.0509
**Precocene II**	6.807_220.	POS	6.807	220.11
**Salicylamide glucuronide**	3.471_313.	POS	3.471	313.08
**2-(2-Phenylethenylsulfonyl)ethenylbenzene**	5.025_270.	POS	5.025	270.0715
**Tetramethylchromanol glucoside**	8.177_382.	POS	8.177	382.201
Cyanobium_gracile				
**Stellatate; Stellatic acid;**	9.318_370.	POS	9.318	370.287
Lentibacter_algarum				
**16-carboxy-Delta(13)-17,18,19,20-tetranor-leukot**	3.786_413.	POS	3.786	413.151
**Adenine**	1.752_135.	NEG	1.752	135.0546

**Doxercalciferol**	9.791_412.	POS	9.791	412.3342
**3-NITRO-L-TYROSINE**	3.41_226.0	POS	3.41	226.0591
**Methylenecyclohexane**	5.118_96.0	POS	5.118	96.09407
**3beta-Fluoro-5beta-pregnan-20-one;**	8.557_320.	POS	8.557	320.2504
**Myristoylcarnitine**	9.644_371.	POS	9.644	371.3035
**TOG10**	8.771_597.	POS	8.771	597.2707
**Asperitaconic acid B**	3.715_272.	NEG	3.715	272.1257
**(23R)-1alpha-Hydroxy-25,27-didehydrovitamin D3**	8.152_426.	POS	8.152	426.2771
**N-Ethyl-N-(2-hydroxy-3-sulfopropyl)-3,5-dimeth**	4.398_319.	POS	4.398	319.1091
**Glyphosate-monoammonium;**	7.272_186.	POS	7.272	186.1044
**hydroxyhexadecenoylcarnitine**	8.112_413.	POS	8.112	413.3127
Psychrobacter_faecalis				
**GALANTHAMINE HYDROBROMIDE**	5.408_384.	POS	5.408	384.1057
**TRICIRIBINE PHOSPHATE**	4.58_400.0	POS	4.58	400.0909
**Razaxaban**	5.752_528.	POS	5.752	528.1647
**(6R,7R)-6-Methoxy-3,7-dimethyl-8-oxo-5-thia-1-**	4.946_243.	POS	4.946	243.0566
**Atropine**	5.557_289.	POS	5.557	289.1711
**(5R,9S)-5,9-dimethyl-14-methylidenetetracyclo[11**	10.032_30	NEG	10.032	302.2245
**Dinitropyrene**	1.847_292.	POS	1.847	292.0472
Other				
**Hematoporphyrin**	8.855_598.	NEG	8.855	598.2796
**N-Butylbenzenesulfonamide**	3.172_213.	POS	3.172	213.0826
Devosia_limi				
Streptococcus_lactarius				
Other				
**3-(3-Hydroxyphenyl)-3-hydroxypropanoic acid**	7.734_182.	POS	7.734	182.0579
**Hydrocortisone cypionate;**	8.44_486.2	POS	8.44	486.2963
**Benzofurazan**	2.934_120.	NEG	2.934	120.0324
Other				
**4-[4-(Quinolin-2-ylmethoxy)phenyl]sulfanylbenzoi**	3.72_387.0	POS	3.72	387.0923
**Scymnol**	8.866_468.	NEG	8.866	468.3448
**Coprocholic acid**	8.865_450.	NEG	8.865	450.3344
**Fluvalinate**	7.044_502.	POS	7.044	502.1257
**Peregrine**	7.503_463.	POS	7.503	463.2931
**S-(2-Hydroxyethyl)-N-acetyl-L-cysteine;**	1.16_207.0	POS	1.16	207.0568
**Solgol**	6.424_309.	NEG	6.424	309.1939
**4-Methylstyrene**	8.485_118.	POS	8.485	118.0784
**Coproporphyrinogen I**	8.995_660.	POS	8.995	660.3165
**NCGC00385787-01!8,8-dimethyl-2-phenylpyrano[**	7.044_304.	POS	7.044	304.111
**Leukotriene D4**	7.513_496.	POS	7.513	496.2616
**Zedoarondiol**	8.774_252.	POS	8.774	252.1728
**Cyclohexyladenosine**	3.516_349.	POS	3.516	349.175
**Carnosifloside VI;**	7.108_944.	POS	7.108	944.5354
**PS(17:0/22:2(13Z,16Z))**	10.371_82	NEG	10.371	829.586
**Benzonaphthazepine**	3.495_243.	POS	3.495	243.1045
**16:3-Glc-Campesterol**	8.55_794.6	POS	8.55	794.6095
**Linkckoside J**	8.24_598.3	NEG	8.24	598.3718
**7-Dehydrocholic acid**	7.098_386.	POS	7.098	386.2455
**N-docosahexaenoyl glutamic acid**	9.284_457.	NEG	9.284	457.2829
**PA(P-16:0/13:0)**	8.601_590.	POS	8.601	590.4304
Bacteroides_vulgatus				
**2-(4-Nitrophenoxy)-1-(4-phenylphenyl)ethanone**	3.928_333.	POS	3.928	333.0997
**cis-4-Octenedioic acid**	6.214_172.	POS	6.214	172.0738
**N-Desmethyl-p-O-sulfate rosiglitazone**	3.895_439.	POS	3.895	439.0509
**Mannosyl-1beta-phosphomycoketide C33**	7.988_722.	POS	7.988	722.5441
**Protopanaxatriol**	9.041_476.	POS	9.041	476.3871
**Cyclohexyl-[cyclohexyl(sulfo)amino]sulfamic acid**	3.947_356.	POS	3.947	356.1081
**N1-Acetyl-tabtoxinine-beta-lactam;**	0.747_230.	POS	0.747	230.0903

**Gambogic acid;**	9.002_628.	POS	9.002	628.3038
**O-(2-Aminoethyl)-5Z,8Z,11Z,14Z-eicosatetraenoat**	10.136_34	POS	10.136	347.2822
**NCGC00384646-01!(4R)-4-ethenyl-4,10-dimethyl-**	8.946_332.	POS	8.946	332.1372
**PA(21:0/0:0)**	7.986_480.	POS	7.986	480.3202
**REMACEMIDE**	7.531_268.	POS	7.531	268.157
**6-hydroxy-4-tridecanolide**	8.602_228.	POS	8.602	228.1727
**1-(1-Methoxybutan-2-yl)-N-(4-methoxy-2-methy**	6.405_355.	NEG	6.405	355.1995
**PS(15:0/22:0)**	12.202_80	NEG	12.202	805.5863
**PS(17:1(9Z)/22:0)**	12.731_83	NEG	12.731	831.6018
**2-Amino-5-[[1-[carboxymethyl(sulfo)amino]-1-ox**	3.486_387.	POS	3.486	387.0416
**ITX-5061 free base**	9.635_583.	POS	9.635	583.2378
**Tephcalostan**	3.563_362.	POS	3.563	362.0784
**Halistanol sulfonic acid G**	7.9_674.24	POS	7.9	674.2443
**2-[4a-methyl-8-methylidene-4-(3-methylpentano**	6.914_348.	POS	6.914	348.2299
**Demeton-S;**	5.644_258.	POS	5.644	258.0506
**Metolachlor**	9.558_283.	POS	9.558	283.1327
**PG(O-16:0/22:6(4Z,7Z,10Z,13Z,16Z,19Z))**	8.115_780.	POS	8.115	780.5326
**PG(O-16:0/20:0)**	8.548_764.	POS	8.548	764.5907
**Succinaldehyde**	0.706_86.0	POS	0.706	86.03699
**5-O-[2-(4-Benzhydrylpiperazin-1-yl)ethyl] 3-O-m**	9.33_610.2	NEG	9.33	610.2812
**Valproic Acid beta-D-Glucuronide**	5.287_320.	POS	5.287	320.1486
**Famciclovir**	5.572_321.	POS	5.572	321.144
Other				
Parabacteroides_chartae				
Other				
Thermus_scotoductus				
Sphingomonas_yunnanensis				
**trihydroxybutyrophenone**	8.313_196.	POS	8.313	196.0738
**L-Arginine, L-asparaginylglycyl-**	8.986_345.	POS	8.986	345.1768
**4,4'-Diaminostilbene dihydrochloride;**	9.284_283.	POS	9.284	283.197
**2-Methyl-4-isothiazolin-3-one**	3.439_115.	POS	3.439	115.0094
**2-Aminoisobutyric acid**	0.739_103.	POS	0.739	103.0635
**2-(Methylamino)benzoic acid**	3.336_151.	NEG	3.336	151.0634
**Kaspar**	7.514_434.	POS	7.514	434.2666
**Glycodeoxycholic acid**	8.558_898.	POS	8.558	898.6277
**3-O-Acetyloleanolic acid**	8.938_498.	POS	8.938	498.369
**Mebeverine alcohol**	6.424_265.	NEG	6.424	265.204
**Metconazole**	5.293_319.	POS	5.293	319.1453
**25,26-dihydroxyvitamin D**	8.592_416.	POS	8.592	416.3292
**Myricetin 3-sambubioside**	3.492_612.	POS	3.492	612.1341
**Oblatone G**	9.317_406.	POS	9.317	406.3084
**2alpha-Methyl-17beta-[(tetrahydro-2H-pyran-2-y**	9.319_388.	POS	9.319	388.2977
**verruculotoxin**	3.21_261.1	POS	3.21	261.1842
**4-Ethylbenzaldehyde**	4.336_134.	POS	4.336	134.0734
**Timosaponin A-I;**	9.158_578.	NEG	9.158	578.3825
**2,6-Dimethoxy-1,4-benzoquinone**	8.284_185.	POS	8.284	185.0691
**(2R)-2-Acetamido-5-hydroxy-2-(sulfanylmethyl)p**	3.01_221.0	POS	3.01	221.0723
**SNAG-delta5-CG**	5.809_730.	NEG	5.809	730.3365
**Athidathion;**	0.729_329.	NEG	0.729	329.9915
**4-(2-Aminoethylamino)-3-[bis(carboxymethyl)ami**	5.022_393.	POS	5.022	393.1365
**Salvinorin A**	4.654_432.	NEG	4.654	432.1818
**alpha-Amylcinnamaldehyde; alpha-Pentylcinnamal**	7.841_202.	POS	7.841	202.136
**Ibrutinib**	6.714_440.	NEG	6.714	440.1947
**PS(22:6(4Z,7Z,10Z,13Z,16Z,19Z)/0:0)**	7.919_569.	POS	7.919	569.274
**Stattic**	3.99_210.9	POS	3.99	210.9939
**2-[[4-(2,4-dihydroxy-3,5,6-trimethylbenzoyl)oxy-2**	4.653_464.	POS	4.653	464.1795
**N-Methyl-2,3,7,8-tetramethoxybenzophenanthridi**	2.385_379.	POS	2.385	379.1414
**Pentadecanedioic acid**	7.889_272.	POS	7.889	272.1988

**Bufotalin**	7.847_444.	POS	7.847	444.2497
**5-[5-(acetyloxymethyl)-1,2,4a-trimethyl-7-oxo-3,4**	7.832_378.	POS	7.832	378.2402
**Cetraxate**	8.132_305.	POS	8.132	305.1641
**2Z-tridecenoic acid**	8.786_212.	POS	8.786	212.1779
**4-hydroxy-tridecanoic acid**	8.786_230.	POS	8.786	230.1884
**cyclo(tyrosyl-prolyl)**	5.583_260.	POS	5.583	260.1161
**carthamosterone**	8.464_518.	POS	8.464	518.2866
**PI(19:1(9Z)/22:6(4Z,7Z,10Z,13Z,16Z,19Z))**	7.107_922.	POS	7.107	922.5558
**Butoconazole**	3.475_410.	POS	3.475	410.0171
**Taprostene**	8.596_398.	NEG	8.596	398.2094
**p-Hydroxymandelic acid**	3.79_168.0	POS	3.79	168.0425
**Aphyllic Acid**	6.615_304.	POS	6.615	304.154
**PS(20:0/18:1(9Z))**	10.685_81	NEG	10.685	817.5859
**Puerarin**	3.942_416.	POS	3.942	416.1155
**3-[(Z)-heptadec-10-enyl]benzene-1,2-diol**	10.149_36	POS	10.149	363.3136
**SA-beta-Gal**	4.582_564.	POS	4.582	564.1976
Clostridium_acidurici				
Other				
**TRIADIMENOL**	0.737_295.	POS	0.737	295.1089
**(1R,2R,5S,8R,14R,15R,16S)-16-hydroxy-1,2,14,17,17**	9.404_486.	POS	9.404	486.3328
**Encaleret**	3.468_513.	POS	3.468	513.2081
**Bacampicillin**	5.149_465.	POS	5.149	465.157
**Diterpene alkaloid**	8.125_405.	POS	8.125	405.288
**oleandomycin**	9.275_687.	POS	9.275	687.4194
**Chenodeoxycholic acid**	8.904_784.	POS	8.904	784.5849
**27-Nor-cholestane-pentol**	8.224_438.	POS	8.224	438.3348
**LysoPC(18:3(6Z,9Z,12Z)/0:0)**	9.48_517.3	POS	9.48	517.3151
**Brigatinib**	8.549_583.	POS	8.549	583.2565
**Coenzyme M 7-mercaptoheptanoylthreonine-phos**	0.693_483.	POS	0.693	483.0475
**Agehoustin C**	5.793_448.	POS	5.793	448.1349
**3-Hydroxymethyl-picumast**	6.375_456.	NEG	6.375	456.1819
**Taxine**	8.487_641.	POS	8.487	641.3211
**Cholesterol cholate**	8.546_792.	POS	8.546	792.6232
**Bisacodyl**	3.504_361.	POS	3.504	361.1306
**peniprequinolone**	6.654_421.	POS	6.654	421.1308
**NCGC00180216-02!6a,7,8-trihydroxy-3-methyl-3,4**	3.507_350.	POS	3.507	350.1147
**3-(2,4-Cyclopentadien-1-ylidene)-5alpha-androsta**	8.486_338.	POS	8.486	338.2608
**Dihomo-<U+03B3>-linolenoyl ethanolamide**	10.209_34	POS	10.209	349.298
**(2R)-3-Cyclopentyl-2-(4-methylsulfonylphenyl)-N**	5.521_378.	POS	5.521	378.1079
**Hydroxynalidixic acid**	5.453_248.	NEG	5.453	248.0799
**1-[(4-Amino-3-methylphenyl)methyl]-5-(2,2-diph**	3.474_480.	POS	3.474	480.2148
**Dimethyl (3,3-diethylbutyl)malonate**	7.45_244.1	POS	7.45	244.1673
**(2R)-2-[[(2R)-2-amino-3-(1-methoxycarbonylindo**	7.342_641.	NEG	7.342	641.3787
**Chenodeoxycholylglycine**	8.493_413.	POS	8.493	413.2928
**(4-oxido-2,3,5,6,7,8-hexahydro-1H-pyrrolizin-4-iu**	6.949_287.	POS	6.949	287.1731
**Thermozeaxanthin-13;**	8.545_926.	POS	8.545	926.6602
**Caffeine**	3.962_194.	POS	3.962	194.0806
**O-Desmethylmethoxyphenamine**	4.945_165.	POS	4.945	165.1159
**5E,7Z-Dodecadienal**	8.811_180.	POS	8.811	180.1516
**Sorbate**	3.892_94.0	POS	3.892	94.04192
**Cer(t18:1(6OH)/19:0(2OH))**	12.505_61	NEG	12.505	611.5509
**N-desethylamiodarone**	8.071_616.	POS	8.071	616.9919
**N-arachidonoyl glutamic acid**	9.486_433.	POS	9.486	433.2831
Methanobrevibacter_woesei				
**Stieleriacine E**	8.102_459.	POS	8.102	459.3352
**Dehydrocrotonin**	8.71_314.1	POS	8.71	314.1518
**Schisantherin B**	8.492_514.	POS	8.492	514.2224
**simvastatin hydroxy acid**	7.856_436.	POS	7.856	436.2826

**6-Ethylchenodeoxycholic acid**	8.898_420.	POS	8.898	420.3241
**Rimexolone**	8.106_370.	POS	8.106	370.2507
**Stieleriacine D**	8.563_457.	POS	8.563	457.3194
**Cladosporacid E**	3.391_200.	NEG	3.391	200.1049
**Nordeoxycholic acid**	9.611_400.	POS	9.611	400.2591
Maricaulis_virginensis				
**1,2-Epoxyhexadecane;**	8.698_240.	POS	8.698	240.2454
**Bufadienolide**	8.44_354.2	POS	8.44	354.2557
**N-(3beta,7beta,dihydroxycholest-5-en-24-oyl) gly**	8.206_447.	NEG	8.206	447.2986
**o6-Carboxymethyl-2'-deoxy-guanosine**	3.055_325.	POS	3.055	325.1024
**Neolinderatone**	8.692_528.	POS	8.692	528.3265
**NCGC00380977-01!6-[(1E,3E,5E,7E)-8-[(2R,3S,4S,5S**	3.494_424.	POS	3.494	424.1881
**Diallyl sulfone**	1.352_146.	POS	1.352	146.0403
**Y1 Receptor antagonist 1**	7.24_487.2	NEG	7.24	487.2599
**Vobtusine;**	6.363_718.	NEG	6.363	718.3727
**2-Oxo-turkesterone**	7.756_494.	NEG	7.756	494.2878
**NCGC00347805-02!(2E,6E)-3,7,11,15,19,23,27,31,35**	9.657_396.	POS	9.657	396.2878
**Acetaminophen**	2.877_151.	POS	2.877	151.0635
**Brivaracetam**	3.291_212.	POS	3.291	212.1527
**(+/-)-6-Acetonyldihydrochelerythrine; 6-Acetonyl-**	5.056_405.	POS	5.056	405.157
**Kaempferol**	9.316_286.	NEG	9.316	286.0476
**Monodechloroaminopyrrolnitrin;**	3.415_192.	POS	3.415	192.0459
**triterpenoids**	9.094_472.	POS	9.094	472.3169
**NCGC00384658-01!7-methoxy-8-(3-methyl-5-oxo**	1_294.052	POS	1	294.0521
**PI(17:2(9Z,12Z)/0:0)**	8.6_582.28	POS	8.6	582.2808
**Avermectin A2a aglycone;**	9.714_616.	POS	9.714	616.3587
**25-Hydroxycholesterol**	9.432_384.	POS	9.432	384.3392
**rabelomycin**	0.628_338.	NEG	0.628	338.0784
**Descarboxyethoxyloratadine**	6.592_426.	NEG	6.592	426.1713
Other				
**p-Hydroxyphenyl-phenylhydantoin**	3.462_268.	POS	3.462	268.0849
**Strophanthin**	8.508_710.	POS	8.508	710.3507
**Becatecarin**	3.458_668.	POS	3.458	668.1835
**N-oleoyl asparagine**	5.407_396.	POS	5.407	396.2987
**Glucose lactate lactate**	0.655_356.	NEG	0.655	356.0953
**5-hydroxy-2-(4-hydroxyphenyl)-7-(3,4,5-trihydrox**	6.472_564.	POS	6.472	564.1479
**Crustecdysone**	8.151_480.	NEG	8.151	480.3088
**(2R)-1-(4-Iodophenyl)-N-propan-2-ylpropan-2-ar**	4.582_303.	POS	4.582	303.0488
**N-[(4-Chlorophenyl)methyl]-N-ethyl-2-pyridinam**	5.75_246.0	POS	5.75	246.0926
**Didesethyl Chloroquine**	5.751_263.	POS	5.751	263.1192
**Cloranolol**	3.402_291.	POS	3.402	291.0779
**LMPR0106140010**	8.532_486.	POS	8.532	486.3349
**Dathf**	5.81_443.1	POS	5.81	443.1795
**Benzyl alcohol, 4-(2,4-diamino-5-pyrimidinylmethy**	7.561_318.	POS	7.561	318.1698
**Mometasone furoate**	9.378_520.	POS	9.378	520.1436
**Carbamorph;**	5.763_220.	POS	5.763	220.0712
**NCGC00180837-02!3,8-dihydroxy-3-methyl-2,4-di**	2.252_322.	POS	2.252	322.0834
**Cephalexin**	8.305_694.	NEG	8.305	694.1859
**Pinane**	9.028_138.	POS	9.028	138.141
**Sarpicillin**	7.621_433.	NEG	7.621	433.1673
**Furylacryloylalanyllysine**	3.449_337.	POS	3.449	337.1638
Other				
**Carbaprostacyclin**	7.94_332.2	POS	7.94	332.235
**Cer(t18:1(6OH)/24:0(2OH))**	12.742_68	NEG	12.742	681.6303
**11-Dehydrocorticosterone**	7.344_344.	POS	7.344	344.1985
**Alanyltryptophan**	7.811_275.	POS	7.811	275.127
**4-heptanoyl butyric acid**	7.728_200.	POS	7.728	200.1415
Anaeroplasma_abactoclasticum				

**1-(1,3-Benzodioxol-5-yl)-4,4-dimethyl-1-penten-**	8.438_234.	POS	8.438	234.126
**N-Desmethylzopiclone**	3.465_374.	POS	3.465	374.0895
**2-Aminobiphenyl**	7.761_169.	POS	7.761	169.0893
**18-Hydroxycortisol**	7.454_378.	POS	7.454	378.2041
**Cholic acid**	8.443_425.	POS	8.443	425.3143
**FA 15 (antioxidant)**	8.99_467.3	POS	8.99	467.3766
**L-Cysteine-S-sulfate**	0.907_200.	POS	0.907	200.9769
**Ixabepilone**	8.629_506.	POS	8.629	506.2822
**Streptothricin F; Racemomycin A;**	8.461_502.	POS	8.461	502.2508
Other				
**Pregnenolone**	8.683_316.	POS	8.683	316.2402
**Zileuton sulfoxide**	3.117_252.	POS	3.117	252.057
**(1S,2R,3R,4R,5R,6S,7S,8R,9R,10R,13R,14R,16S,17S,1**	7.906_485.	POS	7.906	485.2644
**Dinonyl phthalate**	8.038_418.	POS	8.038	418.3083
**Brainesteroside D**	8.586_610.	POS	8.586	610.372
**Butanoyl (2S)-2-amino-5-(diaminomethylideneam**	8.222_244.	POS	8.222	244.1526
**1-Archaetidyl-1D-myo-inositol 3-phosphate; 1-Sa**	8.527_974.	POS	8.527	974.6632
**Pentalinonsterol**	8.632_380.	POS	8.632	380.308
**6,15-Diketo,13,14-dihydro-PGF1a**	6.44_368.2	NEG	6.44	368.2201
**(1'S,2'S)-3',11'-dihydroxy-1',2',5'-trimethyl-8'-oxa**	3.324_297.	POS	3.324	297.1576
**(R)-N1-((S)-3,3-Dimethyl-1-(methylamino)-1-oxo**	3.932_315.	POS	3.932	315.2159
**5-Amino-2-[(2-amino-3-carboxypropanoyl)oxyam**	0.74_263.1	POS	0.74	263.1118
**N-lauroyl glycine**	2.882_257.	POS	2.882	257.1991
Other				
**7,12-diketolithocholic acid**	7.567_404.	POS	7.567	404.2564
**(Z)-N-(3-hydroxyhexadec-9-enoyl)-L-phenylalani**	8.103_417.	POS	8.103	417.2878
**Cinobufagin**	8.474_442.	POS	8.474	442.236
Chryseobacterium_indoltheticum				
**N-Acetyl-D-glucosamine**	8.288_203.	POS	8.288	203.0796
**Demonomethylchlorpromazine**	3.213_304.	POS	3.213	304.0796
**4-Heptyloxyphenol**	8.906_208.	POS	8.906	208.1466
**PA(20:5(5Z,8Z,11Z,14Z,17Z)/13:0)**	8.97_652.4	POS	8.97	652.4129
**N-((Hexahydro-1-azepinyl)carbonyl)-leucyl(1-met**	8.919_619.	POS	8.919	619.3356
**(6Z,10E)-4-hydroxy-6-(hydroxymethyl)-10-methyl**	7.124_264.	POS	7.124	264.1361
**Methylecgonine**	6.146_199.	POS	6.146	199.1211
**(+/-)-(e)-4-Ethyl-2-[(z)-hydroxyimino]-5-nitro-3-**	4.546_306.	POS	4.546	306.1329
**(-)-Huperzine A (HupA)**	5.749_264.	POS	5.749	264.1229
Bacillus_endoradicis				
**Libenzapril**	3.904_363.	POS	3.904	363.1789
**NCGC00381075-01!3-(2,4-dihydroxypentyl)-6,8-di**	6.967_308.	POS	6.967	308.1259
**alpha-curcumene**	7.801_202.	POS	7.801	202.1723
**Ecgonine methyl ester**	6.653_199.	POS	6.653	199.1212
**1alpha-hydroxy-18-(4-hydroxy-4-methyl-2-penty**	8.564_442.	POS	8.564	442.3086
**Jamaicamide C;**	9.318_490.	POS	9.318	490.2613
Prevotella_oulorum				
**N-(dodecanoyl)-homoserine lactone**	8.55_283.2	POS	8.55	283.2147
**L-Urobilinogen; L-Stercobilinogen; Stercobilinogen**	8.273_596.	POS	8.273	596.3567
**N-(9,12-octadecadienoyl)-glutamic acid**	9.493_409.	POS	9.493	409.283
**OKOHA-PE**	9.029_617.	POS	9.029	617.3299
**Schizonepetoside E;**	9.393_348.	NEG	9.393	348.1789
**Hydroxyphenylacetylglycine**	1.981_209.	NEG	1.981	209.0687
**12-Ethyl-8-isobutylbacteriochlorophyllide d;**	8.546_616.	POS	8.546	616.2897
**DIGOXIGENIN**	8.492_408.	POS	8.492	408.2513
**N-(7alpha-Hydroxy-3-oxochol-4-en-24-oyl)-tauri**	7.359_495.	NEG	7.359	495.2653
**a-Lapachone**	1.783_264.	POS	1.783	264.078
**Zizyphine A;**	8.059_611.	NEG	8.059	611.3683
**N-Tert-butyl-1-(2-sulfophenyl)methanimine oxide**	4.414_257.	POS	4.414	257.0723
**Vindoline**	6.971_456.	POS	6.971	456.2294

**Phenylheptatriyne; 1-Phenylhepta-1,3,5-triyne;**	8.072_164.	POS	8.072	164.0628
**Talatisamine**	9.299_421.	POS	9.299	421.2829
Other				
**viloxazine**	7.066_237.	POS	7.066	237.1366
**5-O-Methylvisamminol**	6.93_290.1	POS	6.93	290.1153
**4,4'-Dihydroxytetraphenylmethane**	3.519_352.	POS	3.519	352.1458
**5alpha-Androstane-2alpha-fluoro-17beta-ol-3-on**	7.109_350.	POS	7.109	350.224
**L-Pyrrolysine; Pyrrolysine;**	5.682_255.	POS	5.682	255.1583
**2-Ethyl-2-methyl-N-(6-chloro-4-sulfamoyl-o-toly**	0.648_344.	NEG	0.648	344.0603
**Artefenomel**	8.422_469.	POS	8.422	469.2807
**2,6-Dihydroxy-4-Methoxytoluene**	3.176_154.	POS	3.176	154.0633
**1-oxoprevitamin D3 / 1-oxoprecholecalciferol**	8.593_398.	POS	8.593	398.3185
**Difenzoquat;**	5.764_249.	POS	5.764	249.14
**2-Acetamido-2-deoxy-beta-D-glucosylamine**	0.742_220.	POS	0.742	220.106
**Digoxigenin bisdigitoxoside**	8.555_650.	POS	8.555	650.3645
**Chloroquine**	7.995_319.	POS	7.995	319.1816
**4-Hydroxyphenylglycine**	3.854_167.	POS	3.854	167.0584
**Tetrahydroaldosterone-3-glucuronide**	6.454_540.	POS	6.454	540.2579
**6alpha-Fluoropregn-4-ene-3,20-dione; 6alpha-Flu**	7.109_332.	POS	7.109	332.2136
**PI(O-20:0/22:6(4Z,7Z,10Z,13Z,16Z,19Z))**	8.719_924.	POS	8.719	924.6116
**Proclavaminic acid; Proclavaminate;**	0.745_202.	POS	0.745	202.0957
**5-(8,11-Pentadecadienyl)-1,3-benzenediol**	8.549_338.	POS	8.549	338.2221
**(+)-Isofebrifugine**	4.434_301.	POS	4.434	301.1427
**Doramectin**	8.571_898.	POS	8.571	898.5108
**(1S,4aS,7S,7aS)-7-hydroxy-7-methyl-1-[(2S,3R,4S,**	4.863_376.	POS	4.863	376.1379
**3,7,11-Trimethyldodeca-2,6,10-trienoic acid**	10.134_23	POS	10.134	236.1778
**Orotidine**	3.517_288.	POS	3.517	288.0594
**Trigonelline**	8.278_137.	POS	8.278	137.0478
**26:6(8Z,11Z,14Z,17Z,20Z,23Z)**	8.897_384.	POS	8.897	384.3028
Other				
**Methyl (2E)-2-(10,13-dimethyl-11-oxo-3-pyrrolidi**	7.981_409.	POS	7.981	409.2617
**11alpha-Hemiglutaryloxy-1,25-dihydroxyvitamin D**	9.969_544.	POS	9.969	544.3749
**falcarindiol**	9.466_260.	POS	9.466	260.1777
**PI(20:0/22:4(7Z,10Z,13Z,16Z))**	7.983_942.	POS	7.983	942.6181
**PA(12:0/22:4(7Z,10Z,13Z,16Z))**	9.163_668.	NEG	9.163	668.4443
**Diethylene glycol dimethacrylate**	5.742_242.	POS	5.742	242.1155
**PCTR2**	8.999_520.	POS	8.999	520.2616
**(6AR,10AR)-3-(1,1-Dimethylbutyl)-6A,7,10,10A-tet**	6.919_312.	POS	6.919	312.2452
**Parenabol**	9.154_452.	POS	9.154	452.3294
**Acetamide, 2-(4-(2-((2-hydroxy-3-phenoxypropyl)**	6.94_418.2	POS	6.94	418.2083
**Neolinderachalcone**	8.898_528.	NEG	8.898	528.3226
**2-deoxyecdysone**	8.551_448.	POS	8.551	448.3192
**Decaglycerol**	7.934_758.	POS	7.934	758.3771
**Hildgardtene**	4.8_320.14	NEG	4.8	320.1406
**9-Bromo-17beta-hydroxy-17-methylandrost-4-en**	8.537_394.	POS	8.537	394.1144
**Certonardosterol K**	8.427_466.	NEG	8.427	466.3293
**(8Z,11Z,14Z,17Z,20Z)-Hexacosapentaenoic acid; 8Z**	8.661_386.	POS	8.661	386.3186
**Bisphenol A**	6.718_228.	NEG	6.718	228.115
**N-Monodemethyl roxithromycin**	8.056_808.	POS	8.056	808.4902
**Mosapramine**	7.131_478.	POS	7.131	478.2516
**3beta-Hydroxy-7-oxochol-5-en-24-oyl-glycine**	7.985_445.	POS	7.985	445.283
**2-methyl-hexadecanedioic acid**	9.052_300.	POS	9.052	300.23
Enterococcus_saccharolyticus				
**Timegadine**	8.123_365.	POS	8.123	365.1661
**Actodigin**	8.042_536.	POS	8.042	536.2972
**PS(15:0/0:0)**	7.104_483.	POS	7.104	483.2605
**Mycinamicin VII;**	8.425_521.	POS	8.425	521.3356
**icas#16**	8.216_461.	POS	8.216	461.2416

**Chlorpheniramine; Chlorphenamine;**	7.074_274.	POS	7.074	274.1238
**PG(O-16:0/0:0)**	9.047_470.	POS	9.047	470.3012
Other				
**gemeprost**	7.657_394.	NEG	7.657	394.272
**Hecogenin**	8.721_430.	POS	8.721	430.3084
**Teleocidin B 2**	8.418_451.	POS	8.418	451.3204
**Sdz mrl 953**	8.545_937.	POS	8.545	937.6266
**Rifampicin**	8.557_822.	POS	8.557	822.4037
**Ibutamoren**	8.515_528.	POS	8.515	528.2431
**Pyrodone**	4.81_275.1	POS	4.81	275.1885
**Arbutin**	0.999_310.	POS	0.999	310.047
**(2R,3R,4S,5S,6R)-2-[1,7-bis(4-hydroxyphenyl)hepta**	8.388_479.	POS	8.388	479.2535
**13,14-Dihydro-15-keto Prostaglandin A2**	6.884_356.	POS	6.884	356.1988
**Saredutant**	8.539_551.	POS	8.539	551.2103
**Protoporphyrin IX**	10.04_562.	POS	10.04	562.2582
**Hemiasterlin**	8.394_526.	POS	8.394	526.3521
**1,25-Dihydroxy-19-norvitamin D3**	8.688_404.	NEG	8.688	404.3293
**Staphylopine; (2S)-4-{[(1R)-1-Carboxy-2-(1H-imid**	5.516_328.	POS	5.516	328.1397
**Naldemedine**	8.554_570.	POS	8.554	570.2482
**PG(O-20:0/0:0)**	10.004_52(	POS	10.004	526.3643
**6-acetyloxy-7-ethenyl-1,4a,7-trimethyl-3,4,4b,5,6,**	7.472_398.	POS	7.472	398.1874
**(2R)-2,8-Dimethyl-2-(4,8,12-trimethyltrideca-3,7,1**	8.166_396.	POS	8.166	396.3027
**2-(2-hydroxybut-3-en-2-yl)-3a,6,6,9a-tetramethyl**	7.54_376.2	POS	7.54	376.2032
**NCGC00180657-03!(3S,6R,6aS,7S)-6,6a,7,8-tetrahy**	2.905_308.	POS	2.905	308.1041
**5,6,7,8-Tetrahydro-2-naphthoic acid;**	9.691_176.	POS	9.691	176.084
**Epicatechin 3-O-(2-trans-cinnamoyl-beta-D-allop**	7.594_582.	POS	7.594	582.176
**Minabeolide-7**	8.226_456.	POS	8.226	456.2869
**3-ureidopropionate**	1.671_114.	POS	1.671	114.0431
**Citiolone**	8.667_159.	POS	8.667	159.0357
**Varanic acid**	8.041_436.	POS	8.041	436.3192
**OKODA-PI**	8.491_806.	POS	8.491	806.4239
**LMST01010293**	8.537_490.	POS	8.537	490.3305
**LMST01010288**	9.11_354.2	POS	9.11	354.2922
**Estradiol-17-phenylpropionate; Estra-1,3,5(10)-trie**	8.151_404.	POS	8.151	404.2345
**Estradiol enanthate**	8.558_384.	POS	8.558	384.2666
**Glimepiride**	8.06_490.2	POS	8.06	490.2251
**Adiantifoline;**	7.937_726.	POS	7.937	726.3516
**14:4(2E,4E,8E,10E)(6Me[S],9Me,12Me[S])**	8.488_262.	POS	8.488	262.1934
**PG(18:3(9Z,12Z,15Z)/22:6(4Z,7Z,10Z,13Z,16Z,19Z))**	8.538_816.	POS	8.538	816.4961
**Erdafitinib**	8.464_446.	POS	8.464	446.2435
**D-Pantethine**	6.333_554.	NEG	6.333	554.2355
**Belotecan**	8.529_433.	POS	8.529	433.2003
**PS(20:5(5Z,8Z,11Z,14Z,17Z)/18:4(6Z,9Z,12Z,15Z))**	8.509_801.	POS	8.509	801.459
**TG(18:0(11S-acetoxy)/2:0/2:0)**	8.32_500.3	NEG	8.32	500.3341
**2,2'-Methylenebis(4-methyl-6-tert-butylphenol)**	8.001_340.	POS	8.001	340.2401
**Menatetrenone Epoxide**	8.95_460.2	POS	8.95	460.2975
**Antcin K**	8.725_488.	POS	8.725	488.3119
**Stanolone enanthate**	8.431_402.	POS	8.431	402.3134
**(2R,5S)-5-Amino-1,2,6,7-tetrahydroxyoctane-3,4-c**	7.545_221.	POS	7.545	221.0901
**LMGP06020069**	8.534_928.	POS	8.534	928.6396
**Testosterone isobutyrate**	8.003_358.	POS	8.003	358.2507
**LMSP01080031**	8.511_313.	POS	8.511	313.3341
**Drazoxolon;**	0.627_237.	NEG	0.627	237.0306
**Glycyrrhetinic Acid**	8.555_470.	POS	8.555	470.3396
**PG(18:3(6Z,9Z,12Z)/21:0)**	8.51_814.5	POS	8.51	814.5734
**Adipoylglycine**	8.174_203.	POS	8.174	203.0797
**PA(18:4(6Z,9Z,12Z,15Z)/12:0)**	8.52_612.3	POS	8.52	612.3768
**Naphthoquine**	8.549_409.	POS	8.549	409.1922

Other
Other
Other
Neisseria_elongata
Halothiobacillus_neapolitanus
Other
Prevotella_denticola
Thermobispora_bispora
Psychrobacter_nivimaris
Other
Other
Capnocytophaga_ochracea
Chiayiivirga_flava
Other
Other
Anaeromyxobacter_dehalogenans
Bacteroides_nordii
Deinococcus_aquiradiocola
Peptoniphilus_lacrimalis
Weissella_paramesenteroides
Solirubrobacter_ginsenosidimutans
Other
Other
Photobacterium_kishitanii
Rothia_terrae
Other
Hydrotalea_sandarakina
Scardovia_wiggsiae
Propionimicrobium_lymphophilum
Sphingomonas_daechungensis
Dietzia_timorensis
Clostridium_sphenoides
Pseudohongiella_spirulinae
Aridibacter_famidurans
Acinetobacter_radioresistens
Other
Legionella_waltersii
Rhodobacter_blasticus
Other
Deinococcus_aetherius
Arcanobacterium_phocae
Rubellimicrobium_thermophilum
Actinomyces_radingae
Xanthomonas_citri
Bavariicoccus_seileri
Planctopirus_limnophila
Rubellimicrobium_mesophilum
Bacteroides_graminisolvens
Moraxella_nonliquefaciens
Other
Calidifontibacter_indicus
Malikia_spinosa
Dechloromonas_agitata
Conexibacter_arvalis
Other
Bacteroides_acidifaciens
Other
Clostridium_spiroforme

Other
Sphingomonas_sediminicola
Pelomonas_puraquae
Arcobacter_mytili
Sphingomonas_ginsengisoli
Other
Sutterella_parvirubra
Other
Caldilinea_aerophila
Other
Prevotella_nigrescens
Mucilaginibacter_jinjuensis
Pseudochelatococcus_lubricantis
Other
Clostridium_sardiniense
Bifidobacterium_magnum
Azospirillum_doebereinerae
Pseudonocardia_xishanensis
Ruminococcus_gauvreauii
Methanocella_arvoryzae
Erysipelothrix_inopinata
Defluviicoccus_vanus
Simkania_negevensis
Lactobacillus_acetotolerans
Butyrivibrio_fibrisolvens
Altererythrobacter_dongtanensis
Lysobacter_mobilis
Other
Other
Eubacterium_aggregans
Paracoccus_sulfuroxidans
Micrococcus_flavus
Fibrella_aestuarina
Microbacterium_murale
Parabacteroides_faecis
Other
Other
Other
Jeotgalicoccus_huakuii
Aureimonas_ureilytica
Tolumonas_auensis
Other
Chryseobacterium_xinjiangense
Flavobacterium_cutihirudinis
Other
Other
Ochrobactrum_intermedium
Other
Corynebacterium_aquatimens
Dietzia_maris
Other
Mesorhizobium_mediterraneum
Other
Sphingomonas_cynarae
Mycoplasma_yeatsii
Geodermatophilus_nigrescens
Bifidobacterium_cuniculi
Cloacibacterium_haliotis

Other
Myroides_odoratimimus
Sphingobacterium_wenxiniae
Brochothrix_thermosphacta
Camelimonas_lactis
Methylophilus_leisingeri
Pseudomonas_cedrina
Other
Prosthecobacter_fusiformis
Agromyces_italicus
Hymenobacter_flocculans
Facklamia_tabacinasalis
Other
Other
Fusobacterium_varium
Novosphingobium_fuchskuhlense
Aquicella_siphonis
Arcicella_rigui
Terribacillus_halophilus
Paenibacillus_endophyticus
Lactobacillus_pantheris
Lachnoanaerobaculum_saburreum
Sphingomonas_kaistensis
Rivicola_pingtungensis
Helicobacter_pylori
Other
Halomicrobium_katesii
Other
Dermacoccus_nishinomiyaensis
Other
Myroides_phaeus
Salsuginibacillus_kocurii
Paracoccus_stylophorae
Azospirillum_brasilense
Hydrogenophaga_pseudoflava
Sulfuriferula_plumbophilus
Other
Agrococcus_citreus
Phytohabitans_flavus
Other
Bacteroides_propionicifaciens
Anabaena_flos-aquae
Anabaena_sp
Deinococcus_ficus
Ureibacillus_thermosphaericus
Halocella_cellulosilytica
Aquabacter_spiritensis
Other
Methyloparacoccus_murrellii
Cellvibrio_fulvus
Cellvibrio_gandavensis
uncultivated_soil
Chryseobacterium_formosense
Tolypothrix_sp
Acetoanaerobium_noterae
Gimesia_maris
Rickettsia_sibirica
Hydrogenophaga_defluvii

Acidithiobacillus_ferrivorans
Marinobacter_lutaoensis
Other
Other

Formula	Delta Mass	Delta Mass	Match	Level	Database	KEGG ID	HMDB ID	Super Clas

C7 H8	9.84E-05	1.068439	NA	level4	HMDB	NA	HMDB003	Benzenoid
C12 H14 C	0.000163	0.683179	50.00182	level4	Public	NA	HMDB000	Phenylpro
C9 H10 O4	-3.98E-06	-0.01857	NA	level4	HMDB	NA	HMDB017	Organic ac
C8 H8 O4	1.09E-05	0.054355	92.02768	level1	Decode	NA	HMDB006	Organic ac
C25 H24 F	-0.00127	-2.98441	NA	level4	HMDB	NA	HMDB025	NA
C26 H29 N	0.002037	4.595902	NA	level4	HMDB	NA	HMDB025	NA
C6 H10 O4	-18.0104	-123310	66.28255	level1	BMDB	NA	HMDB000	Lipids and
C33 H54 C	-0.0027	-4.09794	NA	level4	NA	NA	NA	NA
C12 H16 C	4.78E-05	0.199063	NA	level4	HMDB	NA	HMDB006	Lipids and
C12 H16 C	-0.0002	-0.76811	22.80607	level4	Public	NA	NA	NA
C13 H13 N	-0.00132	-4.85675	NA	level4	HMDB	NA	HMDB025	NA
C22 H25 N	-0.00015	-0.40563	NA	level4	HMDB	NA	HMDB025	NA
C6 H12 N2	0.000311	1.945547	0.440278	level4	Public	NA	NA	NA
C16 H32 C	-0.00035	-1.14229	NA	level4	NA	NA	NA	NA
C13 H9 N	0.00025	1.397692	62.48088	level2	Public	NA	NA	NA
C42 H71 C	0.000463	0.603612	NA	level4	NA	NA	NA	NA
C26 H31 N	-0.00013	-0.2731	NA	level4	HMDB	NA	HMDB025	NA
C7 H6 O4	9.68E-05	0.628323	81.46645	level2	mzCloud	NA	HMDB002	Benzenoid
C21 H24 N	8.55E-05	0.232196	NA	level4	HMDB	NA	HMDB025	NA
C22 H25 N	-0.00023	-0.65338	NA	level4	HMDB	NA	HMDB024	NA
C50 H87 N	0.000491	0.476179	NA	level4	NA	NA	NA	NA
C5 H11 N	-18.0105	-153832	89.37118	level2	BMDB	C00431	HMDB000	Organic ac
C36 H56 C	0.001172	2.005082	NA	level4	HMDB	NA	HMDB024	NA
C25 H35 N	-0.00015	-0.36822	NA	level4	HMDB	NA	HMDB006	Benzenoid
C19 H18 N	-0.00134	-3.65733	NA	level4	HMDB	NA	HMDB025	NA
C16 H17 N	-9.87E-05	-0.36404	NA	level4	HMDB	NA	HMDB025	NA
C8 H8 O5	0.000229	1.243172	NA	level4	HMDB	NA	HMDB025	NA
C28 H33 N	0.001149	2.116031	NA	level4	NA	C18642	NA	NA
C10 H20 C	4.71E-05	0.250301	88.00146	level1	Decode	C02774	HMDB024	NA
C21 H24 N	17.02428	36678.95	4.276763	level4	Public	NA	NA	NA
C19 H28 C	-8.41E-05	-0.23875	NA	level4	HMDB	NA	HMDB025	NA
C5 H9 N C	8.62E-05	0.869944	53.95009	level3	Public	NA	NA	NA
C8 H12 O5	0.000248	1.317039	NA	level4	HMDB	NA	HMDB025	NA
C H4 O3 S	4.59E-05	0.477813	NA	level4	NA	C11145	NA	Organic ac
C25 H40 N	-0.00132	-2.77493	NA	level4	HMDB	NA	HMDB025	NA
C26 H34 C	-5.64E-05	-0.12754	NA	level4	HMDB	NA	HMDB025	NA
C9 H11 N	8.08E-05	0.309642	NA	level4	HMDB	NA	HMDB015	Organic ac
C10 H11 N	6.13E-06	0.024605	NA	level4	HMDB	NA	HMDB024	NA
C12 H16 C	-7.44E-05	-0.30972	NA	level4	HMDB	NA	HMDB003	Phenylpro
C37 H58 C	0.000451	0.664328	NA	level4	NA	NA	NA	NA
C17 H28 N	-0.00092	-2.62525	NA	level4	HMDB	NA	HMDB025	NA
C8 H15 N	6.43E-05	0.339967	41.50984	level3	NA	NA	NA	NA
C28 H31 C	0.001513	2.528451	NA	level4	HMDB	NA	HMDB001	Organic ox
C8 H18 N4	0.000251	1.242765	52.83531	level3	mzCloud	NA	HMDB000	Organic ac
C42 H68 N	-0.00127	-1.62278	NA	level4	HMDB	NA	HMDB024	NA
C10 H18 N	0.000172	0.802857	NA	level4	HMDB	NA	HMDB025	NA
C21 H18 C	0.000219	0.491961	NA	level4	HMDB	NA	HMDB004	Phenylpro
C25 H34 C	4.96E-05	0.11525	NA	level4	HMDB	NA	HMDB025	NA
C25 H38 C	-0.00085	-1.42921	NA	level4	NA	C17565	NA	NA
C40 H48 C	-4.87E-05	-0.08218	NA	level4	HMDB	NA	HMDB024	NA
C33 H37 N	0.002561	4.390521	NA	level4	HMDB	NA	HMDB001	Alkaloids a
C12 H21 N	-8.76E-05	-0.28895	NA	level4	HMDB	NA	HMDB025	NA
C22 H28 N	-0.00017	-0.46339	NA	level4	HMDB	NA	HMDB025	NA

C27 H47 C	-0.00122	-2.60621	NA	level4	HMDB	NA	HMDB025	NA
C13 H16 C	0.000329	1.609767	NA	level4	HMDB	NA	HMDB024	NA
C15 H10 C	-0.0001	-0.37957	94.56216	level2	mzCloud	C10343	HMDB003	Benzenoid
C17 H22 N	-0.00136	-4.32143	NA	level4	HMDB	NA	HMDB025	NA
C14 H19 N	-0.00031	-1.04151	NA	level4	HMDB	NA	HMDB001	Organic o
C6 H12 O5	-4.25E-05	-0.25929	50.53514	level3	mzCloud	C01018	NA	Carbohydr
C6 H12 O5	46.00543	280403.9	31.46221	level3	Decode	C07326	HMDB000	Organic o
C46 H56 N	-0.00095	-1.20318	NA	level4	NA	C11641	NA	Alkaloids
C11 H14 N	-0.00078	-3.18602	NA	level4	HMDB	NA	HMDB025	NA
C40 H59 N	0.00243	3.331556	NA	level4	HMDB	NA	HMDB025	NA
C24 H34 C	-0.00017	-0.41359	3.499969	level4	BMDB2	NA	NA	NA
C10 H12 C	21.98208	112111.4	12.06354	level4	Public	NA	NA	NA
C24 H30 N	0.001211	2.789156	NA	level4	HMDB	NA	HMDB025	NA
C21 H24 N	-0.00073	-1.90856	NA	level4	HMDB	NA	HMDB025	NA
C23 H24 N	0.000844	1.700062	NA	level4	HMDB	NA	HMDB024	NA
C30 H48 C	0.000603	1.321823	NA	level4	HMDB	NA	HMDB025	NA
C21 H30 C	-1.73E-05	-0.0477	NA	level4	HMDB	NA	HMDB026	NA
C28 H48 C	2.60E-05	0.058064	NA	level4	HMDB	NA	HMDB024	NA
C10 H12 C	4.06E-06	0.020693	68.16358	level2	Decode	NA	HMDB006	Phenylpro
C9 H18 N2	0.000266	1.429531	NA	level4	HMDB	NA	HMDB025	NA
C25 H45 F	0.000434	0.707472	NA	level4	KEGG	C07597	NA	Lipids and
C50 H89 C	-0.00057	-0.60998	NA	level4	NA	NA	NA	NA
C28 H50 C	0.000165	0.354152	NA	level4	NA	NA	NA	NA
C12 H18 N	7.17E-05	0.231185	NA	level4	HMDB	NA	HMDB024	NA
C53 H83 C	0.00194	2.023661	NA	level4	NA	NA	NA	NA
C34 H62 N	-0.00034	-0.48239	NA	level4	NA	NA	NA	NA
C18 H30 C	-0.00019	-0.63266	92.07005	level2	mzCloud	C16316	NA	FA Fatty a
C32 H50 C	-0.00164	-2.92301	4.027656	level4	Public	NA	NA	NA
C10 H9 N5	21.98098	102198.7	71.50995	level2	mzCloud	C08272	HMDB001	Organohe
C13 H19 N	0.000112	0.545631	NA	level4	HMDB	NA	HMDB006	Benzenoid
C14 H12 N	0.000213	0.796114	NA	level4	NA	C19236	NA	NA
C50 H78 C	-0.00433	-4.20291	NA	level4	NA	NA	NA	NA
C31 H34 C	-0.0011	-1.9412	NA	level4	NA	C12405	NA	NA
C17 H21 N	-0.00024	-0.83709	NA	level4	HMDB	NA	HMDB025	NA
C20 H25 N	-0.00046	-1.30473	NA	level4	HMDB	NA	HMDB025	NA
C18 H23 N	-6.02E-05	-0.15962	NA	level4	HMDB	NA	HMDB024	NA
C48 H79 C	-0.00094	-1.05058	NA	level4	NA	NA	NA	NA
C28 H50 N	-0.0014	-2.52582	NA	level4	HMDB	NA	HMDB025	NA
C26 H28 C	-0.0021	-4.99277	NA	level4	NA	NA	NA	NA
C25 H50 C	0.000239	0.599558	NA	level4	NA	NA	NA	NA
C20 H34 C	-18.0133	-50850.4	53.27989	level3	mzCloud	NA	NA	NA
C4 H8 N4	-0.00058	-3.27578	NA	level4	KEGG	C00499	NA	Organic a
C23 H37 N	-4.10E-05	-0.09006	NA	level4	KEGG	C03577	NA	FA Fatty a
C10 H21 N	6.86E-05	0.36648	5.084315	level4	Public	NA	NA	NA
C21 H34 C	9.79E-05	0.256227	NA	level4	NA	C17770	NA	NA
C6 H8 O9	-0.00019	-0.74276	90.28064	level2	mzCloud	NA	NA	NA
C12 H20 N	0.000238	0.991877	NA	level4	HMDB	NA	HMDB001	Organohe
C30 H59 C	-0.00125	-2.15765	NA	level4	NA	NA	NA	NA
C23 H38 C	3.77E-05	0.108751	NA	level4	HMDB	NA	HMDB025	NA
C25 H39 N	-18.0106	-40087.9	18.90446	level4	Public	NA	NA	NA
C34 H58 C	0.001322	1.87161	4.273019	level4	Public	NA	NA	NA
C5 H4 N4	4.51E-05	0.296899	96.93812	level1	Decode	C07599	HMDB000	Organohe
C9 H16 N2	8.71E-05	0.57265	32.87197	level3	mzCloud	NA	NA	NA

C9 H12 N	-4.17E-05	-0.17079	NA	level4	HMDB	NA	HMDB025	NA
C30 H29 N	-0.00076	-1.53942	NA	level4	HMDB	NA	HMDB025	NA
C8 H8 O4	0.000214	1.272905	17.47189	level4	Public	NA	HMDB002	Organic o
C13H15N	0.000746	2.082037	NA	level4	NA	C13678	NA	NA
C20 H22 N	0.000482	1.249515	NA	level4	HMDB	NA	HMDB024	NA
C18 H18 C	-0.00017	-0.54787	NA	level4	NA	NA	NA	NA
C22 H20 C	0.000684	1.539063	NA	level4	HMDB	NA	HMDB004	Phenylpro
C22 H32 C	-3.97E-05	-0.1211	NA	level4	HMDB	NA	HMDB025	NA
C6 H9 N C	0.000254	1.599437	NA	level4	HMDB	NA	HMDB024	NA
C9 H12 N	-0.00021	-0.86241	86.42993	level2	mzCloud	C02067	HMDB000	Nucleosid
C5 H11 N	8.38E-05	0.828777	41.11566	level3	Public	C01842	HMDB025	NA
C27 H30 C	0.001212	1.79748	NA	level4	NA	NA	NA	NA
C27 H58 N	-0.00252	-4.66297	NA	level4	NA	NA	NA	NA
C21 H23 N	-0.00098	-2.26244	NA	level4	HMDB	NA	HMDB004	Organic o
C14 H16 C	-0.00025	-1.06429	NA	level4	NA	NA	NA	NA
C24 H51 C	-0.00087	-2.00395	NA	level4	HMDB	NA	HMDB025	NA
C10 H16 C	0.000192	0.961789	NA	level4	NA	NA	NA	NA
C10 H10 C	0.000304	1.564279	77.95087	level2	mzCloud	C10470	HMDB000	Phenylpro
C8 H8 O4	0.000207	1.233777	92.77152	level1	Decode	C06672	HMDB000	Benzenoid
C15 H10 C	0.00017	0.485446	NA	level4	HMDB	NA	HMDB024	Phenylpro
C34 H48 C	0.001986	2.989265	1.273185	level4	Public	NA	NA	NA
C14 H21 N	55.96496	189613.3	9.356604	level4	Public	NA	NA	NA
C11 H19 N	-4.57E-05	-0.15811	7.075108	level3	BMDB	C21016	HMDB000	Organic a
C15 H20 C	21.98197	88586.51	15.28967	level4	Public	NA	HMDB025	NA
C39 H47 N	0.001135	1.609218	NA	level4	HMDB	NA	HMDB025	NA
C21 H34 C	-18.0108	-45227	17.41691	level4	Public	NA	NA	NA
C28 H46 C	0.000104	0.233975	NA	level4	HMDB	NA	HMDB024	NA
C11 H11 N	0.000215	1.136552	93.5989	level1	BMDB2	NA	HMDB000	Organohe
C9 H8 O3	0.000121	0.738523	59.38156	level3	mzCloud	C00166	HMDB000	Benzenoid
C13 H19 N	0.001115	3.607981	NA	level4	HMDB	NA	HMDB024	NA
C8 H14 N	-0.00391	-15.6487	47.23192	level3	mzCloud	C00669	HMDB000	Organic a
C24 H30 C	18.01045	49179.4	42.65402	level3	Public	NA	NA	NA
C18 H22 N	-0.00027	-0.86042	NA	level4	HMDB	NA	HMDB025	NA
C21 H22 C	21.98099	56925.43	52.00313	level4	Public	NA	NA	NA
C28 H41 N	0.000352	0.774174	NA	level4	NA	NA	NA	NA
C25 H29 C	0.000175	0.398273	NA	level4	HMDB	NA	HMDB025	NA
C17 H10 C	6.29E-05	0.178522	NA	level4	HMDB	NA	HMDB025	NA
C25 H48 C	0.000283	0.687251	NA	level4	HMDB	NA	HMDB025	NA
C28 H42 N	-0.00173	-3.56692	NA	level4	NA	NA	NA	NA
C26 H32 C	2.97E-05	0.072833	NA	level4	NA	NA	NA	NA
C22 H37 N	9.74E-05	0.256882	NA	level4	NA	NA	NA	NA
C15 H15 N	-1.41E-05	-0.055	29.64123	level4	Public	NA	NA	NA
C23 H24 N	0.00253	4.790296	NA	level4	HMDB	NA	HMDB024	NA
C18 H29 N	-0.00045	-1.39318	NA	level4	HMDB	NA	HMDB025	NA
C29 H38 N	-0.00157	-3.09851	NA	level4	NA	C10009	NA	NA
C23 H27 C	0.000331	0.739371	NA	level4	NA	C12564	NA	Organohe
C19 H19 F	0.001353	3.655778	NA	level4	HMDB	NA	HMDB024	NA
C24 H38 C	0.000765	1.683856	NA	level4	KEGG	C09180	NA	Terpenoid
C23 H35 N	0.000286	0.766518	NA	level4	NA	C12161	NA	NA

C19 H20 F	0.000362	1.099454	NA	level4	HMDB	NA	HMDB001	Organohe
C17 H11 C	0.000439	1.36457	NA	level4	HMDB	NA	HMDB025	NA
C39 H62 C	0.000742	0.943675	NA	level4	NA	NA	NA	NA
C6 H12 O5	21.98221	133981.9	42.72428	level3	Public	NA	HMDB006	Lipids and
C19 H36 C	-18.0106	-54866.5	44.84178	level4	Public	NA	HMDB003	Lipids and
C23 H24 F	-0.00132	-3.31524	NA	level4	HMDB	NA	HMDB025	NA
C19 H30 C	2.41E-05	0.078846	NA	level4	HMDB	NA	HMDB000	Lipids and
C35 H41 N	-0.0028	-4.25405	NA	level4	HMDB	NA	HMDB024	NA
C18 H36 C	-0.00038	-1.10319	NA	level4	NA	NA	NA	NA
C15 H17 F	0.001294	4.68618	NA	level4	HMDB	NA	HMDB024	NA
C10 H13 N	0.001178	3.098544	NA	level4	HMDB	NA	HMDB006	Nucleosid
C21 H32 C	7.66E-06	0.02015	NA	level4	HMDB	NA	HMDB024	NA
C29 H52 C	0.000853	1.158129	NA	level4	HMDB	NA	HMDB025	NA
C25 H37 N	2.82E-05	0.073662	NA	level4	HMDB	NA	HMDB025	NA
C20 H30 C	0.000333	0.719901	NA	level4	NA	NA	NA	NA
C30 H50 C	-0.00012	-0.2425	NA	level4	HMDB	NA	HMDB024	NA
C16 H13 N	-0.00017	-0.56363	NA	level4	NA	C11326	NA	Benzenoid
C44 H81 C	0.002558	3.260818	NA	level4	NA	NA	NA	NA
C22 H34 C	21.98206	58116.61	10.52697	level4	Public	NA	NA	NA
C27 H38 C	-0.00212	-4.47537	5.152031	level4	Public	NA	NA	NA
C29 H31 N	-0.00061	-1.23046	4.615031	level4	Public	NA	HMDB001	Benzenoid
C26 H39 F	-0.00138	-3.13325	NA	level4	HMDB	NA	HMDB024	NA
C17 H32 C	3.84E-05	0.143297	14.05331	level4	Public	NA	NA	NA
C5 H12 N2	7.12E-05	0.538945	87.81534	level1	Public	C00077	HMDB000	Organic ac
C51 H89 C	-0.00235	-2.495	NA	level4	NA	NA	NA	NA
C32 H54 C	-0.00042	-0.8043	NA	level4	NA	NA	NA	NA
C8 H18 N4	1.26E-05	0.062134	57.38853	level3	Public	NA	HMDB024	NA
C24 H29 N	0.0005	1.227621	NA	level4	HMDB	NA	HMDB024	NA
C12 H13 N	-0.0001	-0.39568	NA	level4	HMDB	NA	HMDB025	NA
C5 H9 N S	0.000218	1.48138	NA	level4	HMDB	NA	HMDB025	NA
C8 H14 O2	-0.00107	-5.18439	39.76136	level3	mzCloud	C16241	HMDB000	Organohe
C16 H18 N	-0.00136	-4.37149	NA	level4	HMDB	NA	HMDB025	NA
C7 H12 O4	-18.0104	-112513	66.81373	level2	BMDB2	C02656	HMDB000	Lipids and
C13 H10 C	0.000136	0.589701	NA	level4	HMDB	NA	HMDB025	NA
C14 H17 N	0.000178	0.413152	NA	level4	NA	C10952	NA	NA
C24 H50 N	0.000532	1.10993	NA	level4	HMDB	NA	HMDB001	Lipids and
C5 H10 N2	0.000394	2.033154	NA	level4	HMDB	NA	HMDB025	NA
C18 H34 C	0.000108	0.306023	NA	level4	NA	NA	NA	NA
C13 H22 C	2.59E-05	0.106824	NA	level4	NA	NA	NA	NA
C20 H34 C	-18.0132	-50850.1	55.04167	level3	mzCloud	NA	NA	NA
C16 H32 C	-8.75E-05	-0.30353	NA	level4	NA	NA	NA	NA
C20 H34 C	2.40E-05	0.06767	39.66549	level3	mzCloud	NA	NA	NA
C28 H41 N	-0.00029	-0.54896	NA	level4	HMDB	NA	HMDB024	NA

C50 H87 C	-0.00197	-2.24643	NA	level4	NA	NA	NA	NA
C20 H21 F	-0.00139	-4.30014	NA	level4	HMDB	NA	HMDB000	Benzenoid
C14 H28 C	0.000291	1.119707	NA	level4	NA	NA	NA	NA
C12 H21 N	0.001486	3.839568	NA	level4	KEGG	C08403	NA	Amino aci
C19 H31 N	-8.44E-05	-0.27638	NA	level4	HMDB	NA	HMDB024	NA
C6 H12 N2	0.000129	0.732832	NA	level4	HMDB	NA	HMDB025	NA
C22 H36 C	0.000245	0.593166	21.16849	level4	Public	NA	NA	NA
C20 H28 C	21.98213	66171.62	25.29644	level4	Public	NA	NA	NA
C13 H12 C	0.000799	2.362292	NA	level4	HMDB	NA	HMDB025	NA
C18 H34 C	-18.0107	-57313.9	90.25226	level2	mzCloud	C14829	HMDB000	Lipids and
C25 H36 C	-9.25E-05	-0.22212	NA	level4	HMDB	NA	HMDB025	NA
C10 H8 N2	0.000132	0.844155	15.88529	level4	Public	NA	HMDB024	NA
C30 H53 C	-0.00138	-2.416	NA	level4	NA	NA	NA	NA
C16 H21 N	-0.00163	-4.24757	NA	level4	HMDB	NA	HMDB025	NA
C26 H32 C	21.98098	46549.19	2.26566	level4	Public	NA	NA	NA
C19 H19 N	-7.50E-05	-0.19465	NA	level4	HMDB	NA	HMDB001	Organohe
C21 H39 N	0.000254	0.632542	NA	level4	HMDB	NA	HMDB024	NA
C25 H26 C	-0.00063	-1.21176	NA	level4	NA	C16224	NA	Flavonoids
C19 H18 C	2.20E-05	0.071092	17.63862	level4	Public	NA	NA	NA
C29 H50 C	0.000502	0.683111	NA	level4	HMDB	NA	HMDB025	NA
C29 H34 C	0.000796	1.609655	NA	level4	KEGG	C09510	NA	Terpenoid
C50 H85 C	-0.00242	-2.75634	NA	level4	NA	NA	NA	NA
C4 H6 O6	2.87E-05	0.191029	95.89463	level2	mzCloud	C00898	HMDB000	Organic ox
C30 H46 C	0.000324	0.712625	NA	level4	HMDB	NA	HMDB024	NA
C22 H34 C	0.000955	2.423705	NA	level4	NA	NA	NA	NA
C12 H15 C	-3.01E-05	-0.10517	NA	level4	NA	C15439	NA	NA
C10 H14 N	0.000272	1.293892	NA	level4	HMDB	NA	HMDB024	NA
C33 H50 C	0.002425	4.000019	NA	level4	HMDB	NA	HMDB001	Lipids and
C25 H40 N	0.000282	0.551006	NA	level4	NA	NA	NA	NA
C24 H34 C	0.001925	4.27564	NA	level4	HMDB	NA	HMDB025	NA
C20 H20 N	-0.00145	-3.98324	NA	level4	HMDB	NA	HMDB025	NA
C22 H32 N	-0.0012	-3.11581	NA	level4	HMDB	NA	HMDB024	NA
C25 H28 C	-0.00214	-4.68297	NA	level4	NA	NA	NA	NA
C32 H44 C	0.001417	2.172351	NA	level4	NA	C19869	NA	NA
C17 H20 C	17.02801	59097.1	10.64163	level4	Public	NA	NA	NA
C12 H21 N	0.000289	1.370836	NA	level4	NA	C10592	NA	Alkaloids
C17 H22 C	21.98201	75760.37	15.3074	level4	Public	NA	NA	NA
C32 H39 C	0.001089	1.497157	NA	level4	NA	NA	NA	NA
C17 H28 C	2.36E-05	0.072029	12.15204	level4	Public	NA	NA	NA
C11 H14 N	0.000813	2.727142	NA	level4	HMDB	NA	HMDB024	NA
C11 H11 N	-6.28E-05	-0.30603	95.99985	level1	BMDB2	NA	NA	NA
C34 H61 C	-0.00078	-1.09442	NA	level4	NA	NA	NA	NA
C17 H16 C	-0.00011	-0.37127	NA	level4	HMDB	NA	HMDB024	NA
C10 H12 C	0.000141	0.782691	NA	level4	HMDB	NA	HMDB025	NA
C12 H13 C	0.00038	1.530438	NA	level4	HMDB	NA	HMDB001	Organohe
C20 H32 C	7.66E-06	0.020807	NA	level4	HMDB	NA	HMDB024	NA
C36 H68 N	-0.00224	-3.04644	NA	level4	HMDB	NA	HMDB025	NA
C27 H32 C	-0.00212	-3.91313	NA	level4	HMDB	NA	HMDB025	NA
C26 H24 N	-0.0011	-2.89884	NA	level4	HMDB	NA	HMDB025	NA
C7 H13 Cl	0.000881	4.363597	NA	level4	HMDB	NA	HMDB024	NA
C27 H30 C	8.00E-05	0.171587	NA	level4	HMDB	NA	HMDB025	NA
C34 H40 N	0.002525	4.443353	NA	level4	HMDB	NA	HMDB026	NA
C27 H26 C	-6.91E-05	-0.11102	NA	level4	HMDB	NA	HMDB004	Phenylpro
C12 H16 N	4.44E-05	0.176033	37.88737	level3	mzCloud	NA	NA	NA

C9 H20 N2	0.000223	1.184467	89.83862	level1	BMDB	C03793	HMDB000	Organic ac
C18 H32 C	-0.00024	-0.73729	NA	level4	NA	C14795	NA	FA Fatty a
C16 H25 N	-0.00012	-0.40119	NA	level4	HMDB	NA	HMDB001	Benzenoid
C19 H39 N	0.001415	3.149753	NA	level4	HMDB	NA	HMDB025	NA
C8 H14 N2	0.000137	0.586688	NA	level4	HMDB	NA	HMDB024	NA
C8 H11 N	0.00019	1.122014	NA	level4	HMDB	NA	HMDB000	Benzenoid
C5 H6 O4	7.89E-05	0.606674	82.55512	level1	BMDB	C00490	HMDB000	Lipids and
C19 H41 C	9.46E-05	0.238818	NA	level4	NA	NA	NA	NA
C18 H16 N	0.000216	0.749308	NA	level4	HMDB	NA	HMDB025	NA
C21 H27 N	-0.00143	-3.35256	NA	level4	HMDB	NA	HMDB024	NA
C5 H5 N5	0.00022	1.454531	93.28451	level2	mzCloud	C00242	HMDB000	Organohe
C27 H32 N	0.002084	4.733391	NA	level4	HMDB	NA	HMDB024	NA
C16 H20 C	21.98201	59074.64	52.58266	level3	Public	NA	NA	NA
C35 H58 C	0.000248	0.388612	NA	level4	HMDB	NA	HMDB025	NA
C21 H22 N	0.001389	3.673899	NA	level4	HMDB	NA	HMDB025	NA
C21 H20 C	-0.00026	-0.50086	NA	level4	NA	NA	NA	NA
C15 H30 C	5.35E-05	0.195123	NA	level4	HMDB	NA	HMDB024	NA
C29 H25 N	0.000315	0.598079	NA	level4	HMDB	NA	HMDB024	NA
C8 H Cl3 N	-0.00016	-0.66828	66.31855	level2	Public	NA	HMDB024	Benzenoid
C27 H52 C	0.000111	0.252508	NA	level4	HMDB	NA	HMDB001	Lipids and
C8 H12 O	0.000131	1.053821	NA	level4	NA	NA	NA	NA
C8 H16 O2	8.56E-05	0.593989	87.83081	level1	Decode	C07185	HMDB000	Lipids and
C56 H85 N	0.004334	4.424338	NA	level4	NA	C20023	NA	Marine bio
C8 H5 F3 (	-0.00047	-2.09567	NA	level4	HMDB	NA	HMDB024	NA
C17 H14 F	-8.97E-05	-0.23539	0.501142	level4	Public	C07589	HMDB000	Organohe
C14 H26 C	-0.00033	-1.21589	NA	level4	HMDB	NA	HMDB000	Lipids and
C13 H17 C	0.001606	4.806756	NA	level4	HMDB	NA	HMDB025	NA
C6 H12 O2	-18.1067	-155980	83.93654	level2	Decode	C01585	HMDB000	Lipids and
C6 H10 O4	0.00015	1.030042	91.84025	level2	BMDB	CC06104	HMDB000	Lipids and
C8 H17 N	0.000176	1.103213	69.10539	level2	mzCloud	NA	HMDB000	Organic ac
C25 H32 C	-0.00229	-4.6623	NA	level4	NA	C17507	NA	Terpenoid
C23 H38 C	3.04E-05	0.0803	NA	level4	HMDB	NA	HMDB001	Lipids and
C27 H42 C	0.000271	0.628953	NA	level4	HMDB	NA	HMDB025	NA
C18 H30 C	-7.50E-05	-0.22999	69.08874	level2	Public	NA	HMDB005	Benzenoid
C28 H47 N	0.000964	1.83485	NA	level4	NA	C20739	NA	NA
C29 H48 C	0.000717	1.409563	NA	level4	HMDB	NA	HMDB025	NA
C31 H46 C	-0.00147	-2.76321	NA	level4	HMDB	NA	HMDB006	Lipids and
C19 H20 C	21.9802	70418.64	19.53724	level4	Public	NA	NA	NA
C18 H36 C	-0.00012	-0.36558	NA	level4	NA	C19622	NA	NA
C13 H18 N	0.000152	0.65014	NA	level4	HMDB	NA	HMDB025	NA
C14 H12 N	0.000262	1.259509	69.17206	level2	mzCloud	NA	NA	NA
C19 H22 N	-0.00125	-3.52396	NA	level4	HMDB	NA	HMDB024	NA
C21 H25 C	0.000411	1.058422	46.77669	level3	Public	C07778	HMDB000	Benzenoid
C9 H14 N5	0.001121	4.690575	NA	level4	HMDB	NA	HMDB025	NA
C10 H18 C	-0.00011	-0.50143	NA	level4	HMDB	NA	HMDB000	Organic ac
C27 H21 N	-0.00076	-1.63694	NA	level4	HMDB	NA	HMDB025	NA
C9 H11 N	0.000238	1.791797	NA	level4	HMDB	NA	HMDB001	Organic ni
C26 H34 C	0.000285	0.581755	NA	level4	HMDB	NA	HMDB006	Lipids and
C16 H30 C	2.45E-05	0.090735	NA	level4	HMDB	NA	HMDB024	NA
C19 H25 N	0.000536	1.701555	NA	level4	HMDB	NA	HMDB001	Benzenoid
C7 H7 N C	0.000225	1.469947	NA	level4	HMDB	NA	HMDB026	NA
C32 H61 C	-0.00113	-1.87662	NA	level4	NA	NA	NA	NA

C21 H26 N	0.001754	4.54112	NA	level4	HMDB	NA	HMDB024	NA
C18 H32 C	296.2355	1000001	82.37121	level2	mzCloud	NA	NA	NA
C23 H22 C	21.98072	55768.59	24.07201	level4	Public	NA	NA	NA
C5 H9 N C	0.000212	1.614882	43.62094	level3	BMDB	NA	HMDB000	Organic ac
C6 H14 N2	0.000202	1.384902	NA	level4	HMDB	NA	HMDB024	NA
C12 H11 C	0.000492	1.850836	NA	level4	HMDB	NA	HMDB024	NA
C35 H36 N	-0.00041	-0.68131	NA	level4	NA	C21192	NA	NA
C19 H34 C	-2.73E-05	-0.07967	NA	level4	HMDB	NA	HMDB024	NA
C17 H24 N	-0.00136	-3.57721	NA	level4	HMDB	NA	HMDB024	NA
C36 H59 N	0.001671	2.522378	NA	level4	HMDB	NA	HMDB025	NA
C20 H32 C	-9.24E-07	-0.00231	NA	level4	NA	C17648	NA	NA
C19 H18 C	-0.00021	-0.54852	NA	level4	HMDB	NA	HMDB025	NA
C32 H64 C	0.000601	1.042534	NA	level4	NA	NA	NA	NA
C9 H15 O1	-0.0009	-2.4882	NA	level4	HMDB	NA	HMDB025	NA
C16 H30 C	-0.0008	-2.28881	NA	level4	NA	NA	NA	NA
C22 H38 C	-8.82E-05	-0.26373	83.72374	level2	mzCloud	NA	HMDB000	Lipids and
C9 H13 N	0.000219	1.019466	NA	level4	HMDB	NA	HMDB025	NA
C44 H84 N	0.000378	0.471965	NA	level4	NA	NA	NA	NA
C15 H22 C	21.98044	82586.08	15.95256	level4	Public	NA	NA	NA
C14 H23 N	-0.00011	-0.40981	NA	level4	NA	NA	NA	NA
C12 H22 N	-0.00023	-0.64387	NA	level4	NA	C17950	NA	NA
C26 H52 C	0.000214	0.518414	NA	level4	NA	NA	NA	NA

C10 H18 C	1.05E-05	0.048159	NA	level4	HMDB	NA	HMDB000	Organic ac
C19 H35 N	-0.00031	-1.01273	NA	level4	HMDB	NA	HMDB001	Organic ac
C26 H42 C	0.000114	0.273556	NA	level4	HMDB	NA	HMDB024	NA
C25 H42 C	0.000199	0.470214	NA	level4	HMDB	NA	HMDB025	NA
C26 H34 C	21.98067	47968.6	10.20053	level4	Public	NA	NA	NA
C12 H15 N	0.00122	4.371949	NA	level4	KEGG	C02892	NA	NA
C11 H11 N	0.000204	0.923082	NA	level4	KEGG	C05657	NA	NA
C22 H23 N	-0.00158	-4.24328	NA	level4	HMDB	NA	HMDB025	NA
C41 H52 N	0.002046	2.937707	NA	level4	HMDB	NA	HMDB024	NA
C20 H14 C	-0.00059	-1.95459	NA	level4	NA	C14853	NA	Benzenoid
C13 H23 N	5.50E-05	0.228066	0.727626	level4	Public	NA	NA	NA
C19 H28 N	-0.00042	-1.21307	NA	level4	HMDB	NA	HMDB001	Organohe
C15 H24 N	0.000563	1.580271	NA	level4	HMDB	NA	HMDB025	NA
C44 H80 N	0.000161	0.202033	NA	level4	NA	NA	NA	NA
C15 H10 C	0.000111	0.411963	NA	level4	HMDB	NA	HMDB000	Phenylpro
C22 H22 C	0.000582	1.042701	NA	level4	NA	NA	NA	NA
C19 H21 N	21.98015	67187.37	8.288274	level4	Public	NA	HMDB003	Alkaloids a

C6 H6 O4	4.78E-05	0.274473	NA	level4	KEGG	C00850	NA	NA
C33 H36 (	0.00199	3.655836	NA	level4	NA	C10085	NA	Organosul
C16 H16 (	-0.00041	-1.26386	76.93141	level2	Public	NA	HMDB024	NA
C34 H54 (	0.000137	0.232352	NA	level4	HMDB	NA	HMDB025	NA
C22 H26 (	-0.00223	-4.78526	13.42536	level4	Public	NA	HMDB003	Lipids and
C15 H20 N	-0.00025	-0.79595	NA	level4	HMDB	NA	HMDB025	NA
C21 H24 F	0.001627	3.220388	NA	level4	HMDB	NA	HMDB024	NA
C7 H8 O6	-0.00017	-0.7647	NA	level4	HMDB	NA	HMDB024	NA
C8 H8 N2	6.44E-05	0.357689	80.1574	level2	mzCloud	C05380	HMDB000	Organic ac
C9 H11 N5	-0.00077	-3.03522	51.08721	level3	mzCloud	C05926	HMDB000	Organohe
C26 H50 (	0.000192	0.449273	NA	level4	HMDB	NA	HMDB025	NA
C28 H47 (	1.32E-05	0.022292	NA	level4	NA	NA	NA	NA
C6 H7 N (	0.000238	1.513668	12.14668	level4	Public	NA	HMDB024	NA
C16 H23 N	-0.0009	-2.63402	NA	level4	HMDB	NA	HMDB024	NA
C20 H26 (	-0.00192	-4.8669	NA	level4	HMDB	NA	HMDB025	NA
C18 H18 (	-1.69E-05	-0.06357	NA	level4	HMDB	NA	HMDB001	Phenylpro
C27 H42 (	0.000473	0.988486	NA	level4	NA	NA	NA	NA
C21 H30 (	21.98207	49712.52	13.56713	level4	Public	NA	NA	NA
C12 H11 I	-0.00122	-1.89828	NA	level4	HMDB	NA	HMDB025	NA
C26 H32 (	0.002058	3.956591	NA	level4	HMDB	NA	HMDB024	NA
C48 H76 (	0.000889	0.913897	NA	level4	HMDB	NA	HMDB003	Lipids and
C10 H19 N	4.77E-05	0.23698	88.09842	level1	Decode	NA	HMDB000	Organic ac

C8 H6 N2	0.000214	1.462966	55.54406	level4	Public	NA	HMDB024	NA
C8 H14 N2	0.000259	1.681122	NA	level4	HMDB	NA	HMDB025	NA
C22 H36 N	17.0255	37645.1	12.31821	level4	Public	NA	NA	NA
C26 H42 N	-0.0007	-1.26825	NA	level4	HMDB	NA	HMDB025	NA
C18 H23 N	-0.00042	-1.20686	NA	level4	NA	C18480	NA	NA
C23 H16 (	-0.00064	-1.89624	NA	level4	HMDB	NA	HMDB025	NA
C32 H43 (	-0.00223	-3.50996	NA	level4	HMDB	NA	HMDB024	NA
C24 H36 (	0.00012	0.337185	NA	level4	HMDB	NA	HMDB024	NA
C55 H89 (	0.00427	4.698996	NA	level4	NA	C20369	NA	NA
C22 H33 N	-0.00029	-0.72867	NA	level4	KEGG	C08686	NA	NA
C16 H28 N	-0.00012	-0.39631	NA	level4	HMDB	NA	HMDB001	Organic ac
C18 H32 (	-1.61E-05	-0.05731	NA	level4	HMDB	NA	HMDB024	NA
H Cl O4	-9.67E-07	-0.00968	NA	level4	HMDB	NA	HMDB025	NA
C16 H19 N	-0.00063	-2.0782	NA	level4	HMDB	NA	HMDB025	NA
C28 H32 F	0.000484	0.972578	NA	level4	HMDB	NA	HMDB025	NA

C30 H44 C	0.000708	1.461903	NA	level4	KEGG	C08971	NA	Terpenoid
C22 H21 C	1.41E-05	0.033633	NA	level4	HMDB	NA	HMDB001	Benzenoid
C18 H23 N	-0.00144	-4.22644	NA	level4	HMDB	NA	HMDB025	NA
C11 H17 N	0.000324	1.659104	NA	level4	HMDB	NA	HMDB025	NA
C11 H15 N	0.00011	0.45473	NA	level4	HMDB	NA	HMDB001	Benzenoid
C19 H18 C	-0.00417	-14.1723	45.06173	level3	mzCloud	NA	NA	NA
C22 H28 C	6.04E-05	0.177454	NA	level4	HMDB	NA	HMDB000	Lipids and
C22 H28 N	0.000829	1.786562	NA	level4	HMDB	NA	HMDB025	NA
C53 H83 N	-0.00105	-1.09345	NA	level4	HMDB	NA	HMDB001	Phenylpro
C20 H36 N	21.98424	46161.3	11.65734	level4	Public	NA	NA	NA
C12 H18 C	0.000184	1.030853	NA	level4	HMDB	NA	HMDB024	NA
C15 H22 N	17.026	52203.32	11.52881	level4	Public	NA	NA	NA
C18 H36 C	-0.0002	-0.59732	NA	level4	NA	NA	NA	NA
C23 H37 N	-6.62E-05	-0.16253	NA	level4	NA	NA	NA	NA
C13 H9 Cl	-4.61E-05	-0.15113	NA	level4	HMDB	NA	HMDB024	NA
C19 H24 N	0.001059	3.228135	1.208416	level4	Public	C07063	HMDB001	Benzenoid
C36 H72 C	0.000441	0.680163	NA	level4	NA	NA	NA	NA
C9 H12 N2	-0.00026	-1.04962	92.96277	level1	BMDB	C00299	HMDB000	Nucleoside
C12 H18 N	-0.00094	-3.33849	NA	level4	HMDB	NA	HMDB024	NA
C34 H44 C	0.00182	2.406027	NA	level4	HMDB	NA	HMDB025	NA
C9 H18 O2	156.1149	987252.1	27.79651	level3	BMDB2	C01601	HMDB000	Lipids and
C13 H9 N	9.57E-05	0.534317	NA	level4	HMDB	NA	HMDB024	NA
C19 H21 N	4.79E-05	0.136416	NA	level4	HMDB	NA	HMDB001	Organohe
C18 H25 N	0.000156	0.489493	NA	level4	NA	NA	NA	NA
C20 H34 C	21.98024	59368.25	40.45758	level3	mzCloud	C05963	HMDB000	Lipids and
C18 H36 C	-3.72E-05	-0.11772	NA	level4	NA	NA	NA	NA
C14 H17 N	17.02642	57696.03	6.282173	level4	Public	NA	HMDB003	Organic o
C24 H23 N	6.18E-05	0.138843	NA	level4	HMDB	NA	HMDB025	NA
C8 H15 N	9.67E-05	0.558629	NA	level4	HMDB	NA	HMDB024	NA

C32 H40 N	0.00214	4.051367	NA	level4	HMDB	NA	HMDB025	NA
C23 H40 C	0.000734	1.320317	NA	level4	NA	C21969	NA	NA
C17 H23 N	-1.38E-06	-0.00415	NA	level4	NA	C11774	NA	NA
C21 H23 N	0.000735	1.909461	NA	level4	HMDB	NA	HMDB024	NA
C6 H10 O	-1.06E-05	-0.05026	53.10198	level3	mzCloud	C00818	HMDB000	Organic o
C42 H50 N	0.002437	3.450111	2.543898	level4	Public	NA	NA	NA
C21 H43 N	21.98105	38343.41	4.311812	level4	Public	NA	NA	NA
C15 H21 N	-0.00015	-0.47766	NA	level4	HMDB	NA	HMDB025	NA
C19 H21 N	0.000625	2.116485	NA	level4	HMDB	NA	HMDB025	NA
C28 H37 C	0.000816	1.698228	NA	level4	HMDB	NA	HMDB025	NA
C33 H42 N	-0.00087	-1.76561	NA	level4	KEGG	C09043	NA	NA
C13 H14 N	-0.00117	-4.83592	NA	level4	HMDB	NA	HMDB024	NA
C11 H11 N	0.000201	0.981586	97.08229	level1	Decode	C02043	HMDB000	Organohe
C21 H22 C	-0.00408	-12.0634	32.51301	level3	mzCloud	NA	NA	NA
C32 H50 C	0.000961	1.759425	NA	level4	NA	NA	NA	NA
C7 H6 N2	0.000187	1.586776	70.72551	level2	Public	NA	HMDB024	NA
C12 H11 N	0.000201	1.000298	NA	level4	HMDB	NA	HMDB025	NA
C18 H20 C	-18.0106	-56971.8	3.24395	level4	Public	NA	HMDB003	Benzenoid
C35 H38 N	0.000174	0.278368	NA	level4	NA	C18022	NA	NA
C25 H30 C	0.001954	4.119688	NA	level4	KEGG	C09968	NA	NA
C19 H30 C	0.000151	0.406682	NA	level4	HMDB	NA	HMDB000	Lipids and
C10 H8 N	0.0013	4.922101	NA	level4	HMDB	NA	HMDB025	NA
C24 H44 C	-0.00158	-3.02266	NA	level4	HMDB	NA	HMDB025	NA
C25 H49 C	0.000786	1.373959	NA	level4	HMDB	NA	HMDB006	Lipids and
C27 H43 N	0.000223	0.452994	NA	level4	NA	C10831	NA	Alkaloids
C24 H22 C	-8.07E-05	-0.1986	NA	level4	HMDB	NA	HMDB025	NA
C36 H48 N	-0.00053	-0.7676	NA	level4	HMDB	NA	HMDB024	NA
C16 H23 N	-2.13E-05	-0.07682	NA	level4	HMDB	NA	HMDB024	NA
C4 H6 O5	7.63E-05	0.56914	95.23674	level1	BMDB	C00497	HMDB003	Organic a
C14 H12 N	-0.00123	-3.9394	NA	level4	HMDB	NA	HMDB025	NA
C20 H26 C	-0.00045	-1.51865	26.65782	level4	Public	NA	NA	NA
C26 H34 C	18.01079	40726.68	3.89172	level4	Public	NA	NA	NA
C17 H22 N	-0.0002	-0.5361	NA	level4	HMDB	NA	HMDB025	NA
C22 H22 C	-18.0079	-45230.5	5.444755	level4	Public	NA	NA	NA
C11 H14 C	4.77E-05	0.268025	NA	level4	HMDB	NA	HMDB025	NA
C17 H19 N	-9.53E-05	-0.33424	96.02356	level2	mzCloud	C03882	HMDB002	Alkaloids a
C46 H82 N	0.000676	0.820607	NA	level4	NA	NA	NA	NA
C8 H15 N	0.00018	1.144334	60.74559	level2	Decode	C08283	HMDB003	Organic a
C20 H30 C	-0.00019	-0.65248	NA	level4	HMDB	NA	HMDB000	Lipids and
C11 H10 N	-0.00125	-4.77752	NA	level4	HMDB	NA	HMDB025	NA
C17 H14 F	-0.00016	-0.40742	NA	level4	HMDB	NA	HMDB001	Organohe
C16 H28 C	8.35E-05	0.331135	NA	level4	HMDB	NA	HMDB024	NA
C23 H31 N	-0.00085	-2.30018	NA	level4	HMDB	NA	HMDB025	NA
C8 H13 N	0.000657	2.660725	NA	level4	HMDB	NA	HMDB001	Organohe
C23 H37 N	0.000146	0.373517	NA	level4	NA	NA	NA	NA
C17 H19 N	-0.00136	-3.00954	NA	level4	HMDB	NA	HMDB024	NA

C18 H32 C	-0.00014	-0.40937	NA	level4	NA	NA	NA	NA
C22 H36 C	8.84E-05	0.214314	NA	level4	HMDB	NA	HMDB024	NA
C15 H28 C	0.000242	0.94307	NA	level4	NA	NA	NA	NA
C18 H31 N	3.33E-05	0.120036	NA	level4	HMDB	NA	HMDB025	NA
C19 H34 C	-0.00112	-2.8765	NA	level4	NA	C17451	NA	NA
C27 H36 C	21.98216	48179.95	1.752239	level4	Public	NA	NA	NA
C15 H14 C	-0.00153	-4.15724	NA	level4	HMDB	NA	HMDB001	Organic ac
C7 H14 N2	0.011522	66.18289	84.22008	level1	Decode	C01047	HMDB003	Organic ac
C12 H11 N	-0.00123	-4.793	NA	level4	HMDB	NA	HMDB025	NA
C17 H28 C	8.50E-05	0.236093	NA	level4	NA	NA	NA	NA
C13 H13 N	-0.00024	-0.8149	NA	level4	HMDB	NA	HMDB025	NA
C22 H30 N	-7.11E-06	-0.01768	NA	level4	HMDB	NA	HMDB006	Organic ac
C18 H34 C	17.02651	60322.95	78.74602	level1	BMDB	C00712	HMDB000	Lipids and
C4 H10 O3	0.000107	1.012536	NA	level4	HMDB	NA	HMDB025	NA
C18 H29 N	-8.54E-05	-0.29337	NA	level4	HMDB	NA	HMDB025	NA
C7 H11 N	0.000225	1.435189	25.2748	level4	Public	NA	HMDB024	NA
C27 H29 N	0.002095	4.769476	NA	level4	HMDB	NA	HMDB025	NA
C11 H16 N	4.97E-05	0.185334	NA	level4	HMDB	NA	HMDB025	NA
C6 H14 O6	3.38E-05	0.185615	68.02448	level1	BMDB	C00392	HMDB000	Organic ox
C40 H47 N	0.003705	4.944719	NA	level4	NA	C16524	NA	Terpenoid
C22 H47 N	8.75E-05	0.215944	NA	level4	HMDB	NA	HMDB024	NA
C11 H13 N	0.000397	1.781508	92.89191	level2	mzCloud	NA	HMDB000	Organic ac
C24 H47 N	0.000106	0.256145	NA	level4	HMDB	NA	HMDB000	Lipids and
C H3 Hg	8.74E-05	0.402688	NA	level4	NA	C18672	NA	NA
C36 H75 N	0.000866	0.908045	NA	level4	HMDB	NA	HMDB025	NA
C21 H20 C	-0.00034	-0.81091	NA	level4	HMDB	NA	HMDB025	NA
C14 H14 N	-4.44E-05	-0.15301	NA	level4	KEGG	C03414	NA	NA
C29 H30 C	0.000503	1.027123	NA	level4	NA	NA	NA	NA
C16 H18 N	-0.00055	-1.46923	NA	level4	HMDB	NA	HMDB024	NA
C13 H16 C	3.58E-05	0.141409	NA	level4	HMDB	NA	HMDB006	Benzenoid
C37 H71 C	0.00014	0.197627	NA	level4	NA	NA	NA	NA
C13 H11 C	-0.00057	-2.16437	NA	level4	HMDB	NA	HMDB025	NA
C12 H8 O3	0.000113	0.562899	NA	level4	NA	C19444	NA	NA
C14 H20 C	0.000179	0.667535	NA	level4	NA	NA	NA	NA
C2 H6 O2	0.000137	1.457805	93.80898	level1	Decode	C11142	HMDB000	Organosul
C11 H19 N	-1.63E-05	-0.07136	NA	level4	HMDB	NA	HMDB001	Lipids and
C20 H28 C	-0.0001	-0.35418	NA	level4	HMDB	NA	HMDB000	Lipids and
C20 H25 N	-0.00379	-8.24417	40.58294	level3	mzCloud	D09353	NA	NA

C12 H13 N	-0.00111	-4.04981	NA	level4	HMDB	NA	HMDB025	NA
C32 H64 N	0.000701	1.189832	NA	level4	NA	NA	NA	NA
C16 H22 C	0.00013	0.528388	NA	level4	NA	NA	NA	NA
C10 H7 N	0.000169	0.974718	82.16389	level1	BMDB	C06325	HMDB000	Organohe
C24 H46 N	-0.00042	-0.79584	NA	level4	NA	NA	NA	NA
C32 H56 C	-18.0106	-27778.1	5.574423	level4	Public	NA	NA	NA
C21 H23 C	-0.00178	-4.40448	NA	level4	HMDB	NA	HMDB024	NA
C11 H15 N	1.51E-06	0.005369	87.50102	level1	BMDB	C02494	HMDB000	Nucleosid
C10 H14 N	0.001596	7.058841	50.34836	level3	mzCloud	C00931	HMDB000	Organic ni
C4 H8 N2	0.000303	1.845565	NA	level4	HMDB	NA	HMDB025	NA
C27 H28 C	0.000587	0.964451	NA	level4	NA	NA	NA	NA
C37 H73 N	-0.0004	-0.58948	NA	level4	NA	NA	NA	NA
C18 H20 N	-2.09E-05	-0.06355	NA	level4	HMDB	NA	HMDB025	NA
C7 H15 N	0.000211	1.456884	50.40412	level3	Decode	C01181	HMDB000	Lipids and
C36 H56 C	0.000969	1.457841	NA	level4	KEGG	C08968	NA	Terpenoid
C19 H18 C	8.88E-06	0.025949	7.330743	level4	Public	NA	NA	NA
C3 H9 N C	0.000117	1.563898	93.62072	level1	Decode	C01104	HMDB000	Organic ni
C18 H34 C	-0.00016	-0.43388	NA	level4	NA	NA	NA	NA
C25 H28 C	17.0275	33773.79	11.91667	level4	Public	NA	NA	NA
C25 H37 N	-7.03E-05	-0.17606	NA	level4	HMDB	NA	HMDB025	NA
C26 H32 C	-0.00136	-2.45695	4.911912	level4	Public	NA	NA	NA
C36 H59 N	-0.00093	-1.36022	NA	level4	NA	C15681	NA	NA
C14 H16 C	21.98048	94698.57	16.59959	level4	Public	NA	NA	NA
C18 H34 C	-1.12E-05	-0.03377	NA	level4	HMDB	NA	HMDB000	Lipids and
C25 H34 C	-7.63E-05	-0.19162	NA	level4	NA	C15046	NA	NA
C26 H50 C	2.26E-05	0.049388	NA	level4	NA	NA	NA	NA
C8 H11 N	0.000173	1.129733	63.54727	level2	mzCloud	D08286	NA	NA
C12 H22 N	0.000214	0.948221	NA	level4	KEGG	C04277	NA	NA
C9 H17 N	0.000231	1.489634	NA	level4	HMDB	NA	HMDB025	NA
C28 H52 C	-0.00055	-0.9715	NA	level4	HMDB	NA	HMDB024	NA
C4 H9 N3	7.91E-05	0.603785	87.04618	level1	BMDB	C00300	HMDB000	Organic ac
C4 H7 N3	9.83E-05	0.869305	92.08635	level1	BMDB	C00791	HMDB000	Organic ac
C26 H25 F	0.000964	1.878407	NA	level4	HMDB	NA	HMDB025	NA
C13 H18 C	-0.00026	-1.03751	NA	level4	NA	NA	NA	NA
C21 H25 C	0.001535	3.620047	NA	level4	HMDB	NA	HMDB025	NA
C27 H29 N	0.00083	1.540139	NA	level4	KEGG	C06450	NA	NA
C11 H22 C	0.000119	0.546161	NA	level4	HMDB	NA	HMDB025	NA
C23 H39 N	0.0007	1.585839	NA	level4	NA	NA	NA	NA
C34 H29 N	0.002553	4.069755	NA	level4	HMDB	NA	HMDB025	NA
C25 H28 N	-0.00022	-0.52464	77.52852	level2	Public	C07469	HMDB001	Benzenoid
C42 H36 N	0.00163	2.867839	NA	level4	HMDB	NA	HMDB025	NA
C27 H33 C	0.001105	2.173361	NA	level4	HMDB	NA	HMDB025	NA
C29 H43 N	0.000163	0.286781	NA	level4	HMDB	NA	HMDB024	NA
C6 H9 N3	0.000113	0.728756	96.21175	level1	BMDB	C00135	HMDB000	Organic ac

C16 H14 C	-21.9819	-86516.8	57.64253	level4	Public	NA	NA	NA
C23 H28 C	0.000888	1.848567	NA	level4	HMDB	NA	HMDB025	NA
C38 H50 N	-7.35E-05	-0.09597	NA	level4	HMDB	NA	HMDB025	NA
C20 H30 N	-0.00137	-3.82921	NA	level4	HMDB	NA	HMDB025	NA
C13 H12 N	-0.00011	-0.53706	68.3817	level2	mzCloud	C06538	HMDB003	Alkaloids a
C11 H17 N	0.000119	0.524251	NA	level4	NA	NA	NA	NA
C17 H19 N	-0.00026	-0.81723	NA	level4	NA	C12168	NA	NA
C24 H34 C	0.000377	0.900487	NA	level4	NA	C15180	NA	NA
C6 H8 F N	0.000437	2.312743	NA	level4	HMDB	NA	HMDB025	NA
C23 H27 N	-0.00118	-2.61308	NA	level4	HMDB	NA	HMDB024	NA
C16 H22 N	1.44E-05	0.041166	NA	level4	NA	C18497	NA	NA
C6 H6 O6	-7.67E-05	-0.37218	93.7898	level1	NA	NA	NA	NA
C20 H32 C	-0.00017	-0.50391	NA	level4	HMDB	NA	HMDB024	NA
C12 H14 C	6.27E-05	0.282159	44.86834	level3	Public	NA	NA	NA
C18 H16 C	17.02657	57501.82	8.700574	level4	Public	NA	NA	NA
C12 H15 N	7.80E-05	0.308122	NA	level4	HMDB	NA	HMDB006	Organic ac
C21 H22 C	-5.27E-05	-0.12612	NA	level4	HMDB	NA	HMDB004	Phenylpro
C22 H38 C	0.000109	0.286049	NA	level4	HMDB	NA	HMDB024	NA
C30 H60 N	0.00025	0.445222	NA	level4	NA	NA	NA	NA
C24 H29 N	0.000148	0.340839	73.3217	level2	Public	NA	HMDB001	Organic ac
C20 H26 C	0.001789	4.362315	NA	level4	HMDB	NA	HMDB025	NA
C26 H45 N	0.000602	1.039868	NA	level4	HMDB	NA	HMDB000	Lipids and
C22 H31 N	0.001912	4.110777	NA	level4	HMDB	NA	HMDB024	NA
C21 H24 C	-0.00014	-0.40341	7.643963	level4	Public	NA	NA	NA
C24 H41 N	-0.00033	-0.90743	NA	level4	NA	NA	NA	NA
C26 H25 F	-0.00168	-3.00234	NA	level4	HMDB	NA	HMDB024	NA
C26 H30 C	-0.00218	-4.62845	NA	level4	KEGG	C09009	NA	NA
C28 H46 C	0.001176	2.304956	NA	level4	NA	NA	NA	NA
C18 H34 C	-0.00014	-0.47815	NA	level4	HMDB	NA	HMDB024	NA
C18 H24 C	6.21E-05	0.186386	NA	level4	HMDB	NA	HMDB025	NA
C6 H14 O	-0.00147	-4.76532	NA	level4	NA	NA	NA	NA
C7 H11 N	0.000235	1.497852	63.52852	level1	BMDB	NA	NA	NA
C11 H15 N	-6.03E-05	-0.21467	NA	level4	HMDB	NA	HMDB025	NA
C20 H23 N	-0.00014	-0.3734	NA	level4	HMDB	NA	HMDB025	NA
C29 H44 C	-0.00195	-3.99095	NA	level4	HMDB	NA	HMDB025	NA
C21 H27 N	-8.24E-05	-0.23082	NA	level4	HMDB	NA	HMDB001	Benzenoid
C31 H50 C	0.000698	1.345875	NA	level4	NA	NA	NA	NA
C20 H35 N	-8.97E-05	-0.27912	68.45977	level2	mzCloud	NA	HMDB001	Organic ni
C27 H30 C	0.000863	1.452411	8.650847	level4	Public	NA	HMDB030	NA
C25 H37 N	3.56E-05	0.085667	NA	level4	HMDB	NA	HMDB024	NA
C19 H20 N	9.84E-05	0.253501	NA	level4	HMDB	NA	HMDB024	NA
C5 H4 O3	8.42E-05	0.751613	88.44213	level2	mzCloud	NA	HMDB000	Organohe
C19 H26 N	0.000127	0.357511	NA	level4	HMDB	NA	HMDB024	NA
C24 H40 C	7.00E-05	0.194307	58.79905	level3	Public	NA	NA	NA
C21 H23 N	0.000248	0.644052	NA	level4	HMDB	NA	HMDB025	NA
C18 H36 C	5.65E-05	0.170065	NA	level4	NA	NA	NA	NA
C21 H20 C	-6.38E-05	-0.14765	78.06659	level2	mzCloud	C09126	HMDB003	Phenylpro
C14 H17 N	0.000215	0.841297	NA	level4	HMDB	NA	HMDB024	NA

C5 H4 N4	0.000213	1.562401	95.03715	level1	Decode	NA	HMDB001	Organohe
C12 H22 (	0.000116	0.470898	NA	level4	HMDB	NA	HMDB000	Organic ac
C20 H21 N	-0.00111	-2.6965	NA	level4	HMDB	NA	HMDB026	NA
C10 H13 N	0.001086	2.983773	NA	level4	HMDB	NA	HMDB006	Nucleosid
C26 H28 N	-0.0008	-1.80892	NA	level4	HMDB	NA	HMDB025	NA
C43 H85 N	0.002991	4.299404	NA	level4	NA	NA	NA	NA
C44 H77 (	0.001429	1.869479	NA	level4	NA	NA	NA	NA
C30 H50 (	37.95463	85796.22	35.22137	level4	Public	C08618	HMDB003	Lipids and
C45 H67 (	-0.00151	-1.96694	NA	level4	NA	NA	NA	NA
C36 H66 N	-0.00232	-3.3015	NA	level4	NA	NA	NA	NA
C9 H9 N	0.000129	0.980647	NA	level4	HMDB	NA	HMDB024	NA
C19 H34 (	1.57E-05	0.050631	NA	level4	HMDB	NA	HMDB024	NA
C9 H11 N	0.000146	0.595996	NA	level4	HMDB	NA	HMDB025	NA
C9 H11 N	0.000301	1.413508	NA	level4	HMDB	NA	HMDB001	Organic ac
C20 H38 (	-6.83E-05	-0.21998	78.48354	level2	mzCloud	C16526	HMDB000	Lipids and
C20 H26 (	37.95418	114948.9	7.537905	level4	Public	NA	NA	NA
C14 H26 (	0.00026	1.147286	NA	level4	HMDB	NA	HMDB025	NA
C19 H29 N	-0.0003	-0.8431	NA	level4	HMDB	NA	HMDB001	Benzenoid
C6 H11 Cl	0.000122	0.571501	NA	level4	HMDB	NA	HMDB025	NA
C18 H24 I	-0.00017	-0.21021	NA	level4	HMDB	NA	HMDB025	NA
C18 H16 (	0.000339	0.940497	1.503214	level4	Public	C17957	HMDB030	NA
C24 H38 (	15.99481	40983.24	95.38862	level1	BMDB2	NA	HMDB000	Lipids and
C16 H17 N	-0.00156	-4.30569	4.459406	level4	Public	NA	NA	NA
C6 H14 N	0.000249	1.432472	NA	level4	HMDB	NA	HMDB024	NA
C21 H24 (	-0.00029	-0.80731	3.279259	level4	Public	NA	NA	NA
C12 H26 (	0.000139	0.595568	NA	level4	HMDB	NA	HMDB025	NA
C20 H26 (	21.98034	66570.15	5.273865	level4	Public	NA	NA	NA
C11 H22 N	3.24E-05	0.118103	NA	level4	HMDB	NA	HMDB025	NA
C20 H24 N	-0.00059	-1.65273	NA	level4	HMDB	NA	HMDB025	NA
C27 H33 N	0.000817	1.548946	NA	level4	HMDB	NA	HMDB001	Phenylpro
C8 H9 N (	0.000195	1.442104	63.35925	level2	Public	NA	NA	NA
C32 H48 (	0.002265	3.824728	NA	level4	HMDB	NA	HMDB024	NA
C10 H10 N	0.000246	1.195129	NA	level4	NA	C16516	NA	NA
C27 H30 (	0.000449	0.755032	NA	level4	HMDB	NA	HMDB025	NA
C18 H25 N	-0.00031	-0.9327	NA	level4	HMDB	NA	HMDB024	NA
C9 H9 N (	0.000203	1.040481	93.31264	level2	mzCloud	NA	HMDB000	Benzenoid
C27 H32 (	21.98103	43944.08	45.23724	level4	Public	NA	NA	NA
C17 H27 N	0.001679	4.548883	NA	level4	HMDB	NA	HMDB001	Benzenoid
C13 H13 N	-9.62E-06	-0.03655	NA	level4	HMDB	NA	HMDB024	NA
C26 H34 (	0.000743	1.515547	53.23404	level4	Public	NA	NA	NA
C9 H12 N	0.000199	1.214651	30.40351	level4	Public	NA	NA	NA
C7 H8 N2	0.000339	2.490092	32.0096	level3	mzCloud	NA	NA	NA
C20 H20 (	-0.00069	-2.25183	NA	level4	NA	NA	NA	NA
C16 H29 N	0.002321	4.604519	NA	level4	NA	C21152	NA	NA
C19 H22 (	-9.41E-06	-0.02386	8.98811	level4	Public	NA	HMDB024	NA
C22 H36 N	0.001763	4.155129	NA	level4	HMDB	NA	HMDB025	NA
C15 H22 N	-0.00029	-0.889	NA	level4	HMDB	NA	HMDB025	NA
C26 H50 N	0.001504	2.728486	NA	level4	NA	NA	NA	NA
C6 H8 O7	0.000179	0.931848	95.33734	level1	BMDB	C00158	HMDB000	Organic ac
C21 H22 (	-0.00016	-0.44747	NA	level4	HMDB	NA	HMDB025	NA
C27 H30 (	0.000382	0.642141	39.63451	level4	Public	NA	NA	NA
C23 H38 (	17.03489	39963.46	30.60473	level3	mzCloud	NA	NA	NA
C16 H24 (	5.79E-05	0.195507	NA	level4	NA	NA	NA	NA
C27 H53 (	0.000334	0.556264	NA	level4	HMDB	NA	HMDB024	Lipids and

C26 H30 C	21.98054	50160.49	23.71664	level4	Public	NA	NA	NA
C7 H15 N	0.000185	1.043548	NA	level4	NA	C17217	NA	NA
C35 H52 N	0.00019	0.26497	NA	level4	HMDB	NA	HMDB025	NA
C17 H12 F	-2.77E-05	-0.06733	NA	level4	HMDB	NA	HMDB006	Organohe
C19 H28 C	0.001601	3.84802	15.25155	level4	Public	NA	NA	NA
C11 H12 N	9.81E-05	0.445689	76.34111	level2	Public	NA	NA	NA
C29 H54 N	-0.00124	-1.94137	NA	level4	NA	NA	NA	NA
C23 H36 N	-0.00126	-2.8108	NA	level4	NA	C11297	NA	NA
C22 H34 C	37.95405	100343.7	5.862494	level4	Public	NA	NA	NA
C15 H21 N	-1.35E-05	-0.04833	NA	level4	HMDB	NA	HMDB024	NA
C26 H28 C	-0.00222	-4.74637	NA	level4	NA	NA	NA	NA
C12 H10 C	0.000367	1.487291	22.17437	level4	Public	C18604	HMDB025	NA
C15 H14 N	-1.18E-05	-0.03526	NA	level4	HMDB	NA	HMDB024	NA
C20 H20 N	-0.00145	-4.12136	NA	level4	HMDB	NA	HMDB025	NA
C19 H18 C	0.000296	0.680018	NA	level4	HMDB	NA	HMDB001	Organic ac
C28 H30 C	0.001253	1.915956	NA	level4	HMDB	NA	HMDB004	Phenylpro
C24 H36 C	-3.02E-05	-0.08124	NA	level4	HMDB	NA	HMDB001	Lipids and
C23 H24 N	-0.0017	-4.92575	NA	level4	NA	C20168	NA	NA
C29 H32 N	17.02639	33238.21	4.249955	level4	Public	NA	NA	NA
C29 H38 C	0.002011	3.576262	NA	level4	KEGG	C08865	NA	Terpenoid
C6 H6 O	-6.11E-05	-0.64955	94.74363	level2	mzCloud	C00146	HMDB000	Benzenoid
C12 H8 N2	0.00011	0.517193	NA	level4	HMDB	NA	HMDB024	NA
C10 H14 N	0.001351	4.754142	NA	level4	HMDB	NA	HMDB006	Nucleoside
C20 H36 C	0.00108	2.159846	NA	level4	HMDB	NA	HMDB025	NA
C18 H32 C	0.000174	0.529845	NA	level4	NA	NA	NA	NA
C20 H41 N	-18.0107	-55025.7	52.9265	level3	mzCloud	C13914	HMDB001	Organic ni
C17 H19 N	2.56E-05	0.070935	NA	level4	HMDB	NA	HMDB025	NA
C5 H13 N2	0.000158	0.694176	NA	level4	KEGG	C03872	NA	NA
C22 H25 N	17.02639	39489.91	8.758585	level4	Public	NA	NA	NA
C12 H14	0.000193	1.218164	NA	level4	HMDB	NA	HMDB024	NA
C11 H21 N	0.000309	1.435932	NA	level4	HMDB	NA	HMDB024	NA
C21 H20 C	0.000141	0.325782	20.94751	level4	Public	NA	NA	NA
C17 H15 N	-0.00152	-4.78264	NA	level4	HMDB	NA	HMDB025	NA
C25 H22 C	-0.00083	-2.0703	NA	level4	NA	NA	NA	NA
C8 H15 N	0.000293	1.549635	40.08508	level3	Decode	NA	HMDB009	Organic ac
C10 H14 N	-3.08E-05	-0.10907	NA	level4	KEGG	C00939	NA	NA
C25 H33 N	-0.00058	-1.18304	NA	level4	NA	C15674	NA	NA
C21 H24 N	17.02656	48346.35	5.076844	level4	Public	NA	NA	NA
C30 H44 C	0.002732	4.98358	45.0558	level3	mzCloud	C08859	NA	Steroids
C27 H44 C	-0.00016	-0.40758	NA	level4	HMDB	NA	HMDB024	NA
C11 H10 N	-0.00196	-10.5565	62.59302	level2	mzCloud	NA	NA	NA
C30 H58 N	0.000405	0.703736	NA	level4	HMDB	NA	HMDB001	Lipids and
C21 H26 C	0.000232	0.599774	NA	level4	HMDB	NA	HMDB025	NA
C35 H54 C	-0.00061	-1.01348	NA	level4	HMDB	NA	HMDB025	NA
C16 H26 C	2.26E-05	0.065201	14.86545	level4	Public	NA	NA	NA
C15 H12 C	0.00018	0.804759	NA	level4	HMDB	NA	HMDB025	NA
C33 H64 N	0.000586	0.87995	NA	level4	NA	NA	NA	NA
C6 H9 N C	7.81E-05	0.446012	83.3001	level2	mzCloud	C01042	HMDB000	Organic ac

C12 H17 N	0.000235	0.797417	NA	level4	HMDB	NA	HMDB025	NA
C23 H45 N	4.957712	12414.92	6.866316	level4	Public	NA	HMDB024	NA
C5 H8 O3	-4.07E-06	-0.03507	85.68858	level1	Decode	NA	HMDB000	Organic ac
C28 H55 A	-0.00094	-1.72814	NA	level4	NA	NA	NA	NA
C18 H19 F	0.001187	2.907917	NA	level4	HMDB	NA	HMDB025	NA
C28 H35 N	0.000796	1.257213	NA	level4	HMDB	NA	HMDB025	NA
C28 H32 F	-0.00044	-0.98375	NA	level4	HMDB	NA	HMDB025	NA
C20 H17 F	-0.00117	-3.14587	NA	level4	HMDB	NA	HMDB006	Benzenoid
C37 H45 N	-0.00024	-0.39796	NA	level4	HMDB	NA	HMDB025	NA
C8 H7 Cl C	-1.27E-06	-0.00824	NA	level4	HMDB	NA	HMDB024	NA
C24 H30 C	-1.73E-05	-0.04339	NA	level4	HMDB	NA	HMDB024	NA
C6 H13 N5	0.000231	1.347277	NA	level4	HMDB	NA	HMDB025	NA
C29 H37 N	-0.00095	-1.98471	NA	level4	HMDB	NA	HMDB025	NA
C25 H29 N	-0.00011	-0.26401	NA	level4	HMDB	NA	HMDB024	NA
C25 H23 N	0.001765	3.762256	NA	level4	HMDB	NA	HMDB024	NA
C30 H42 C	4.33E-05	0.089687	NA	level4	NA	NA	NA	NA
C23 H24 N	0.001657	4.453042	NA	level4	HMDB	NA	HMDB025	NA
C12 H17 N	-3.54E-05	-0.11364	NA	level4	HMDB	NA	HMDB000	Nucleoside
C15 H24	0.000296	1.450553	NA	level4	HMDB	NA	HMDB024	NA
C46 H84 N	0.000138	0.163492	NA	level4	NA	NA	NA	NA
C27 H36 C	0.000462	0.979165	NA	level4	HMDB	NA	HMDB025	NA
C22 H32 N	-0.00098	-2.35765	NA	level4	HMDB	NA	HMDB025	NA
C15 H18 C	17.02655	69178.31	25.5746	level4	Public	NA	NA	NA
C18 H19 N	-0.00141	-3.51983	NA	level4	HMDB	NA	HMDB025	NA
C16 H14 C	-0.00011	-0.39368	76.65874	level2	mzCloud	NA	NA	NA
C26 H48 N	0.001036	1.886448	NA	level4	NA	NA	NA	NA
C11 H10 N	-0.00217	-10.7572	46.28331	level3	mzCloud	C10744	NA	Alkaloids
C33 H49 N	0.000607	1.126183	2.404634	level4	Public	NA	HMDB024	NA
C24 H48 N	-0.00028	-0.57606	NA	level4	HMDB	NA	HMDB001	Lipids and
C17 H22 N	-3.84E-05	-0.10953	NA	level4	HMDB	NA	HMDB025	NA
C20 H24 C	0.001165	3.088244	NA	level4	HMDB	NA	HMDB024	NA
C11 H16 C	0.000259	1.576509	NA	level4	HMDB	NA	HMDB024	NA
C15 H14 C	1.57E-05	0.064757	NA	level4	HMDB	NA	HMDB000	Phenylpro
C35 H37 N	-0.00109	-2.06051	NA	level4	HMDB	NA	HMDB025	NA
C22 H30 C	-0.00041	-1.13262	NA	level4	HMDB	NA	HMDB024	NA
C11 H19 N	-3.90E-05	-0.15918	NA	level4	HMDB	NA	HMDB024	NA
C24 H34 C	18.01061	43063.31	3.37074	level4	Public	NA	NA	NA
C9 H16 N4	0.000575	2.709504	NA	level4	HMDB	NA	HMDB024	NA
C25 H28 C	21.98088	48184.83	20.35358	level4	Public	NA	NA	NA
C31 H48 C	-0.00202	-3.58721	NA	level4	NA	NA	NA	NA
C8 H8 Cl2	-0.00059	-2.84139	NA	level4	NA	C11252	NA	NA
C9 H9 N C	-5.61E-05	-0.28739	84.90857	level1	Decode	NA	HMDB000	Benzenoid
C16 H22 C	9.14E-05	0.280277	NA	level4	HMDB	NA	HMDB025	NA
C30 H39 N	-0.00124	-2.28852	NA	level4	NA	NA	NA	NA
C6 H10 O3	9.20E-05	0.707711	49.92116	level4	Public	NA	NA	NA
C21 H22 C	0.000172	0.426979	85.41876	level2	mzCloud	C10112	HMDB002	Phenylpro
C12 H11 N	0.000326	1.62087	8.755465	level4	Public	C07491	HMDB024	NA
C17 H22 N	-0.0003	-0.95086	NA	level4	HMDB	NA	HMDB025	NA
C39 H77 N	-8.01E-05	-0.11427	NA	level4	NA	NA	NA	NA
C19 H32 C	-9.60E-05	-0.29597	NA	level4	NA	C15495	NA	NA
C3 H6 O6	6.52E-05	0.383823	NA	level4	NA	C11499	NA	Organic ac
C6 H14 N4	0.000253	1.451009	96.9403	level1	BMDB	C00062	HMDB000	Organic ac
C25 H46 C	-0.00209	-4.72581	NA	level4	NA	NA	NA	NA
C21 H26 C	0.001445	3.703783	NA	level4	HMDB	NA	HMDB024	NA

C40 H72 N	0.000249	0.342786	NA	level4	NA	NA	NA	NA
C7 H8 O	0.000159	1.46884	49.54242	level3	BMDB	C00556	HMDB000	Benzenoid
C16 H17 N	0.000439	1.530571	60.28823	level2	mzCloud	NA	HMDB006	Benzenoid
C10 H12 N	0.000292	1.2167	NA	level4	NA	C14302	NA	Benzenoid
C6 H8 N C	0.000236	1.365907	NA	level4	HMDB	NA	HMDB024	NA
C44 H88 N	0.002658	3.29977	NA	level4	NA	NA	NA	NA
C17 H14 C	21.98364	69993.97	34.87832	level4	Public	C17785	HMDB025	NA
C8 H11 N	0.000187	1.365717	41.01496	level3	BMDB	C00483	HMDB000	Benzenoid
C13 H18 B	6.63E-05	0.17638	94.0762	level2	mzCloud	NA	NA	NA
C21 H31 C	-0.00149	-3.37087	NA	level4	HMDB	NA	HMDB025	NA
C31 H37 N	0.001976	3.584375	NA	level4	HMDB	NA	HMDB006	Phenylpro
C29 H46 F	0.001869	3.890488	NA	level4	NA	NA	NA	NA
C37 H54 C	-0.00275	-4.07509	NA	level4	HMDB	NA	HMDB025	NA
C22 H40 C	-0.00189	-3.93054	NA	level4	NA	NA	NA	NA
C6 H4 O5	0.000193	1.237563	NA	level4	HMDB	NA	HMDB000	Organohe
C7 H14 O2	-2.01549	-15491.9	71.58295	level2	BMDB2	C17714	HMDB000	Lipids and
C2 H4 O5	0.00011	0.787707	NA	level4	HMDB	NA	HMDB025	NA
C18 H16 N	0.000233	0.600733	NA	level4	NA	C17753	NA	NA
C23 H27 N	-0.0002	-0.43864	NA	level4	HMDB	NA	HMDB004	Alkaloids a
C18 H34 C	1.87E-05	0.051698	NA	level4	NA	NA	NA	NA
C19 H27 C	-18.009	-46637.2	17.91199	level4	Public	NA	NA	NA
C15 H12 C	7.52E-05	0.313076	NA	level4	HMDB	NA	HMDB024	NA
C20 H44 N	0.001973	4.470463	NA	level4	NA	NA	NA	NA
C21 H22 N	-0.00139	-3.26251	NA	level4	HMDB	NA	HMDB024	NA
C17 H17 N	2.34E-05	0.082546	NA	level4	HMDB	NA	HMDB025	NA
C20 H26 N	37.95623	116359	16.65377	level4	Public	NA	HMDB024	NA
C23 H22 C	17.02763	43201.8	9.913154	level4	Public	C07593	HMDB003	Phenylpro
C10 H10 C	4.99E-05	0.236608	NA	level4	NA	C14266	NA	NA
C22 H20 N	-0.00144	-4.05086	NA	level4	HMDB	NA	HMDB024	NA
C22 H20 C	0.000926	1.666014	NA	level4	NA	NA	NA	NA
C25 H30 N	0.00062	1.342139	NA	level4	HMDB	NA	HMDB024	NA
C21 H18 C	6.32E-05	0.141775	60.9538	level2	Public	C10025	HMDB004	Phenylpro
C41 H42 N	-0.00178	-2.01927	NA	level4	HMDB	NA	HMDB025	NA
C35 H37 N	-0.00254	-4.78489	NA	level4	HMDB	NA	HMDB025	NA
C22 H24 C	0.000956	2.2475	0.237179	level4	Public	NA	HMDB001	Organohe
C16 H16 N	-0.00017	-0.51802	NA	level4	HMDB	NA	HMDB025	NA
C17 H26 C	-18.0107	-61222.7	20.00028	level4	Public	NA	NA	NA
C30 H39 N	5.18E-05	0.083438	NA	level4	KEGG	C02334	NA	NA
C18 H34 C	41.02617	130554.4	55.24493	level4	Public	NA	NA	NA
C21 H30 N	0.001464	2.762151	NA	level4	HMDB	NA	HMDB024	NA
C9 H14 N2	0.000315	1.470819	NA	level4	HMDB	NA	HMDB000	Organohe
C24 H26 C	-0.00078	-2.36954	NA	level4	NA	NA	NA	NA
C5 H9 N C	0.000126	0.859971	69.76802	level1	BMDB	C00025	HMDB000	Organic ac

C34 H52 C	37.9554	66312.56	0.824649	level4	Public	NA	NA	NA
C32 H30 C	0.001082	1.614607	NA	level4	NA	NA	NA	NA
C11 H12 C	0.000237	1.13917	11.28309	level4	Public	NA	NA	NA
C19 H20 N	-0.00038	-1.24932	10.47462	level4	Public	C07440	HMDB001	Benzenoid
C14 H8 O2	0.000185	0.887647	NA	level4	HMDB	NA	HMDB025	NA
C14 H11 N	0.000121	0.580875	NA	level4	HMDB	NA	HMDB006	Organohe
C20 H41 N	-0.00017	-0.5004	NA	level4	HMDB	NA	HMDB024	NA
C22 H24 F	-0.00039	-1.01082	NA	level4	HMDB	NA	HMDB024	NA
C26 H45 N	0.000395	0.662927	NA	level4	HMDB	NA	HMDB000	Lipids and
C18 H29 N	-4.92E-06	-0.01601	NA	level4	HMDB	NA	HMDB001	Benzenoid
C20 H29 N	-0.00018	-0.56148	4.101669	level4	Public	NA	NA	NA
C18 H36 N	-0.00137	-3.06535	NA	level4	NA	C16566	NA	NA
C20 H18 C	-0.00065	-2.35923	NA	level4	HMDB	NA	HMDB025	NA
C22 H40 C	17.02474	34306.64	5.096503	level4	Public	NA	NA	NA
C20 H23 F	-0.0012	-3.81589	NA	level4	HMDB	NA	HMDB024	NA
C18 H34 C	-7.87E-05	-0.25046	93.53119	level2	mzCloud	NA	NA	NA
C18 H18 C	0.000337	0.98092	NA	level4	HMDB	NA	HMDB024	NA
C14 H11 C	-0.00023	-0.73645	85.95664	level2	mzCloud	NA	HMDB006	Benzenoid
C15 H19 N	0.001866	4.797186	NA	level4	HMDB	NA	HMDB024	NA
C24 H30 C	21.98018	57507.46	8.100559	level4	Public	NA	HMDB002	Phenylpro
C21 H36 C	0.001805	4.028034	9.229803	level4	Public	NA	NA	NA
C16 H32 C	-0.00018	-0.6288	NA	level4	KEGG	C08285	NA	FA Fatty a
C24 H32 N	-0.00082	-1.79712	NA	level4	HMDB	NA	HMDB025	NA
C21 H22 C	-0.0008	-1.91794	NA	level4	HMDB	NA	HMDB025	NA
C10 H20 C	2.94E-05	0.143801	46.51902	level3	Public	NA	NA	NA
C15 H19 N	-0.00131	-4.85493	NA	level4	HMDB	NA	HMDB001	Organohe
C12 H24 C	0.000127	0.586551	NA	level4	HMDB	NA	HMDB024	NA
C10 H13 N	-0.00018	-0.64109	NA	level4	HMDB	NA	HMDB024	NA
C11 H17 N	17.02711	62803.58	1.522254	level4	Public	NA	NA	NA
C22 H19 C	0.001933	4.657414	NA	level4	HMDB	NA	HMDB024	NA
C26 H30 C	-0.0024	-4.80381	NA	level4	HMDB	NA	HMDB001	Benzenoid
C10 H10 N	0.000298	1.569062	NA	level4	HMDB	NA	HMDB024	NA
C22 H28 N	-0.00137	-3.32486	NA	level4	HMDB	NA	HMDB025	NA
C17 H25 N	5.00E-05	0.154786	NA	level4	NA	C18772	NA	NA
C42 H51 N	0.00075	0.927121	NA	level4	HMDB	NA	HMDB024	NA
C25 H25 N	-0.00129	-2.7312	NA	level4	HMDB	NA	HMDB025	NA
C20 H22 N	-0.00458	-14.2043	38.331	level3	mzCloud	NA	NA	NA

C23 H22 F	0.002119	4.995071	NA	level4	HMDB	NA	HMDB025	NA
C20 H30 C	-0.00103	-2.57901	NA	level4	HMDB	NA	HMDB024	NA
C31 H48 C	0.002104	3.837885	NA	level4	NA	NA	NA	NA
C6 H12 O6	1.25E-05	0.069683	85.01796	level1	BMDB	C06468	NA	Carbohydr
C6 H14 N	-0.00023	-0.89657	NA	level4	HMDB	NA	HMDB025	NA
C14 H16 N	-0.00156	-4.81955	NA	level4	HMDB	NA	HMDB025	NA
C16 H10 C	-0.00036	-1.66644	NA	level4	NA	C14519	NA	Benzenoid
C11 H6 O7	-0.00053	-1.86667	NA	level4	HMDB	NA	HMDB024	Phenylpro
C20 H18 C	0.000272	0.676569	NA	level4	NA	NA	NA	NA
C52 H82 N	0.000891	0.878547	NA	level4	HMDB	NA	HMDB025	NA
C32 H58 N	-0.00294	-4.42452	NA	level4	NA	C13900	NA	GP Glycer
C31 H44 C	-0.00155	-2.92496	NA	level4	HMDB	NA	HMDB024	NA
C16 H30 C	-0.00034	-1.0309	NA	level4	NA	NA	NA	NA
C21 H30 C	0.0034	10.81884	43.03951	level3	mzCloud	C06972	HMDB001	Organohe
C14 H26 C	0.001526	4.735539	NA	level4	NA	NA	NA	NA
C11 H20 C	0.000129	0.596206	40.53574	level3	Public	NA	NA	NA
C20 H24 N	2.25E-05	0.055776	NA	level4	HMDB	NA	HMDB025	NA
C8 H16 O2	7.88E-05	0.546812	84.25611	level1	Decode	C06423	HMDB000	Lipids and
C27 H29 N	-0.00187	-4.0412	NA	level4	HMDB	NA	HMDB024	NA
C41 H43 N	0.001812	2.512118	NA	level4	HMDB	NA	HMDB025	NA
C31 H42 N	0.002294	4.079554	NA	level4	HMDB	NA	HMDB025	NA
C36 H70 N	-0.00058	-0.8593	NA	level4	HMDB	NA	HMDB000	Lipids and
C20 H30 N	0.000296	0.85494	NA	level4	KEGG	C04170	NA	Alkaloids
C32 H62 N	0.002144	3.461792	NA	level4	NA	NA	NA	NA
C14 H26 C	7.94E-05	0.328041	NA	level4	NA	NA	NA	NA
C5 H11 N3	0.000215	1.483873	70.48627	level2	mzCloud	C01035	HMDB000	Organic ac
C8 H7 N C	0.000256	1.552466	NA	level4	HMDB	NA	HMDB024	NA
C20 H21 C	6.41E-06	0.015408	NA	level4	HMDB	NA	HMDB025	NA
C30 H46 C	-18.0105	-37033.1	14.90988	level4	Public	NA	NA	NA
C6 H11 N	0.00018	1.395757	92.18877	level1	BMDB	C00408	HMDB000	Organic ac
C6 H6 N4	-0.00124	-7.46074	54.61528	level3	mzCloud	C16353	HMDB000	Organohe
C24 H30 C	0.000116	0.303044	NA	level4	NA	NA	NA	NA
C21 H22 C	-4.82E-05	-0.14244	2.447236	level4	Public	NA	NA	NA
C12 H12 C	0.000226	1.202176	NA	level4	HMDB	NA	HMDB024	NA
C26 H46 N	37.95801	64738.01	1.745098	level4	Public	NA	NA	NA
C19 H34 C	0.001141	2.80794	NA	level4	NA	NA	NA	NA
C27 H22 F	0.00116	2.078273	NA	level4	HMDB	NA	HMDB025	NA
C11 H8 N8	-0.00129	-4.08471	NA	level4	HMDB	NA	HMDB026	NA
C14 H25 N	0.00014	0.627385	NA	level4	NA	NA	NA	NA
C28 H48 N	-0.0011	-1.9706	NA	level4	HMDB	NA	HMDB025	NA
C27 H30 C	6.09E-05	0.102505	44.95133	level4	Public	NA	NA	NA
C20 H24 C	-0.00058	-1.49169	NA	level4	HMDB	NA	HMDB025	NA
C19 H21 N	-0.00038	-0.8494	NA	level4	HMDB	NA	HMDB024	NA
C30 H44 C	0.002602	4.887916	NA	level4	NA	NA	NA	NA
C26 H43 N	0.000124	0.266916	96.05147	level1	BMDB2	NA	HMDB024	Lipids and
C14 H29 N	-0.00093	-2.93239	9.791968	level4	Public	NA	NA	NA
C23 H22 N	-0.00121	-2.91557	NA	level4	HMDB	NA	HMDB025	NA
C34 H65 N	0.001081	1.960397	NA	level4	NA	NA	NA	NA
C31 H34 C	0.001567	2.847817	NA	level4	NA	C17685	NA	NA
C31 H61 C	-0.00131	-2.27055	NA	level4	NA	NA	NA	NA
C16 H17 N	8.66E-05	0.246766	NA	level4	HMDB	NA	HMDB006	Organic ox
C9 H11 N3	0.000237	1.338777	NA	level4	HMDB	NA	HMDB026	NA
C8 H9 N C	0.000154	1.139835	NA	level4	HMDB	NA	HMDB024	NA
C21 H34	-0.00014	-0.48177	NA	level4	HMDB	NA	HMDB025	NA

C30 H31 N	-0.00063	-1.3995	NA	level4	HMDB	NA	HMDB025	NA
C15 H18 O	0.000115	0.321325	NA	level4	HMDB	NA	HMDB004	Organic ox
C9 H17 N	0.000255	1.253936	33.91602	level3	mzCloud	NA	NA	NA
C20 H37 N	0.000158	0.446034	NA	level4	HMDB	NA	HMDB025	NA
C28 H39 N	0.000481	0.883017	NA	level4	HMDB	NA	HMDB025	NA
C H6 O6 P	-0.00051	-2.91634	NA	level4	HMDB	NA	HMDB025	NA
C13 H18 N	1.52E-05	0.060633	25.88079	level4	Public	NA	HMDB025	NA
C19 H23 N	-0.00146	-3.83282	NA	level4	HMDB	NA	HMDB024	NA
C26 H45 N	0.0005	1.002092	90.86598	level1	BMDB2	C05463	HMDB000	Lipids and
C9 H10 N	0.000244	1.670153	62.91514	level2	mzCloud	C03114	HMDB000	Organohe
C18 H26 N	-0.00015	-0.43425	NA	level4	HMDB	NA	HMDB025	NA
C2 H7 N O	21.98216	175836.7	15.2669	level4	BMDB	C00245	HMDB000	Organic ac
C15 H21 O	0.000685	2.089019	NA	level4	HMDB	NA	HMDB025	NA
C17 H22 N	4.11E-05	0.143505	NA	level4	HMDB	NA	HMDB006	Phenylpro
C14 H21 N	0.000116	0.373159	NA	level4	HMDB	NA	HMDB024	NA
C7 H10 N	0.000181	1.173578	NA	level4	HMDB	NA	HMDB025	NA
C15 H18 N	-0.00019	-0.61165	NA	level4	KEGG	C01632	NA	NA
C6 H12 O	-0.00074	-4.11273	NA	level4	HMDB	NA	HMDB025	NA
C18 H24 N	-0.00024	-0.77297	30.20068	level4	Public	NA	NA	NA
C9 H16 O	3.03E-05	0.160823	95.37326	level1	BMDB	C08261	HMDB000	Lipids and
C5 H4 N4	8.38E-05	0.615679	96.77427	level1	BMDB	C00262	HMDB000	Organohe
C13 H22 N	0.00101	3.214394	NA	level4	HMDB	NA	HMDB000	Organic ni
C25 H36 O	0.000291	0.605215	NA	level4	HMDB	NA	HMDB001	Lipids and
C10 H16 N	0.000227	0.993774	NA	level4	HMDB	NA	HMDB025	NA
C12 H16 N	3.33E-05	0.132112	55.64589	level3	mzCloud	NA	NA	NA
C12 H8 O	0.000101	0.506818	NA	level4	NA	C21149	NA	NA
C10 H17 N	0.000113	0.487364	NA	level4	HMDB	NA	HMDB000	Organic ac
C11 H20 O	2.14E-05	0.086083	NA	level4	NA	NA	NA	NA
C27 H30 N	0.001793	3.781427	NA	level4	HMDB	NA	HMDB025	NA
C8 H12 O	0.000173	1.23799	NA	level4	HMDB	NA	HMDB006	Lipids and
C41 H48 N	0.003483	4.889901	NA	level4	HMDB	NA	HMDB025	NA
C10 H14 F	0.001147	4.643335	NA	level4	HMDB	NA	HMDB025	NA
C18 H14 N	-0.00017	-0.54077	NA	level4	HMDB	NA	HMDB025	NA
C6 H12 O	-18.0105	-109774	29.03319	level3	BMDB	C01019	HMDB000	Organic ox
C24 H34 O	0.001693	3.899971	NA	level4	HMDB	NA	HMDB006	Organohe
C26 H26 N	-0.00091	-1.88073	NA	level4	HMDB	NA	HMDB025	NA
C8 H5 N3	-0.00057	-3.26114	NA	level4	HMDB	NA	HMDB024	NA
C30 H46 O	-0.00011	-0.26655	NA	level4	NA	NA	NA	NA
C33 H34 N	0.001011	1.893061	NA	level4	HMDB	NA	HMDB025	NA
C24 H36 O	2.12E-05	0.056857	3.553155	level4	Public	NA	NA	NA
C26 H45 N	-0.0012	-2.40324	91.16807	level2	mzCloud	C05465	HMDB000	Lipids and
C30 H45 O	0.002061	3.843826	NA	level4	HMDB	NA	HMDB025	NA
C11 H8 O	4.79E-05	0.254486	63.31675	level2	Public	C10387	HMDB025	NA
C40 H54 N	0.00358	4.529085	NA	level4	NA	C15724	NA	NA
C2 H4 O4	0.000116	0.934903	NA	level4	HMDB	NA	HMDB025	NA
C12 H15 N	0.00038	1.852178	NA	level4	HMDB	NA	HMDB024	NA
C20 H27 N	-0.00014	-0.35449	NA	level4	NA	C17309	NA	NA
C21 H42 O	0.000114	0.332393	NA	level4	HMDB	NA	HMDB025	NA
C40 H70 N	0.000231	0.318686	NA	level4	NA	NA	NA	NA
C23 H29 O	-0.00198	-4.53502	NA	level4	HMDB	NA	HMDB025	NA
C11 H15 N	0.000151	0.670169	NA	level4	HMDB	NA	HMDB025	NA
C13 H14 N	0.000277	1.025446	NA	level4	HMDB	NA	HMDB025	NA

C9 H15 N5	0.000101	0.392879	NA	level4	NA	C15522	NA	Organohe
C16 H17 N	0.000964	4.318269	NA	level4	HMDB	NA	HMDB024	NA
C24 H34 N	0.001543	3.72594	NA	level4	HMDB	NA	HMDB025	NA
C27 H32 O	-0.00107	-2.54361	NA	level4	HMDB	NA	HMDB024	NA
C23 H18 O	6.20E-05	0.15256	NA	level4	NA	NA	NA	NA
C20 H23 N	0.001188	3.483311	NA	level4	HMDB	NA	HMDB025	NA
C12 H23 N	-3.38E-05	-0.13786	50	level3	Masslist	NA	HMDB000	Lipids and
C23 H29 N	0.000672	1.503446	NA	level4	HMDB	NA	HMDB006	Alkaloids a
C15 H23 O	0.000561	1.678216	NA	level4	NA	C19019	NA	NA
C26 H26 N	0.00215	4.906417	NA	level4	HMDB	NA	HMDB025	NA
C12 H12 F	0.001136	3.876211	NA	level4	HMDB	NA	HMDB025	NA
C10 H16 N	-3.98E-05	-0.14827	NA	level4	HMDB	NA	HMDB025	NA
C10 H14 N	0.000251	1.002336	NA	level4	HMDB	NA	HMDB025	NA
C14 H18 N	-0.00013	-0.42694	NA	level4	HMDB	NA	HMDB025	NA
C26 H45 N	-18.0101	-37264.8	71.97985	level2	Public	NA	NA	NA
C17 H24 O	0.001562	4.591209	20.96278	level4	Public	NA	NA	NA
C31 H44 O	-0.00143	-2.53897	NA	level4	HMDB	NA	HMDB024	NA
C27 H44 O	0.000283	0.680595	47.1723	level3	mzCloud	C01673	HMDB000	Lipids and
C23 H30 N	0.000113	0.232181	NA	level4	HMDB	NA	HMDB024	NA
C38 H72 N	-9.69E-05	-0.13813	NA	level4	HMDB	NA	HMDB000	Lipids and
C26 H45 N	0.000455	0.941848	90.24447	level1	BMDB	C02592	HMDB000	Lipids and
C15 H9 Cl	0.00057	1.876224	NA	level4	NA	C10311	NA	Polyketide
C32 H53 N	0.002476	3.666387	NA	level4	HMDB	NA	HMDB024	NA
C19 H28 O	-0.0003	-0.78799	NA	level4	HMDB	NA	HMDB025	NA
C24 H40 O	0.000125	0.305149	NA	level4	HMDB	NA	HMDB024	NA
C17 H34 O	-7.92E-05	-0.27656	NA	level4	HMDB	NA	HMDB025	NA
C27 H28 O	0.000599	1.012139	NA	level4	HMDB	NA	HMDB004	Phenylpro
C21 H30 N	4.93E-05	0.121298	NA	level4	NA	NA	NA	NA
C9 H14 N2	-4.31E-05	-0.21759	NA	level4	HMDB	NA	HMDB001	Organohe
C14 H26 N	-0.00078	-1.88864	NA	level4	NA	C21219	NA	NA
C20 H25 N	-0.00028	-0.78526	NA	level4	HMDB	NA	HMDB024	NA
C29 H46 O	-0.00027	-0.54084	NA	level4	NA	NA	NA	NA
C16 H20 O	0.000266	1.08749	NA	level4	NA	C10351	NA	NA
C27 H45 B	-0.00014	-0.30034	NA	level4	NA	NA	NA	NA
C19 H28 O	37.95397	107766	19.53776	level4	Public	NA	NA	NA
C19 H30 O	-0.0001	-0.23018	NA	level4	HMDB	NA	HMDB024	Lipids and
C16 H27 N	-18.0106	-64049.3	20.9342	level4	Public	NA	NA	NA
C38 H50 O	0.000576	0.955538	NA	level4	HMDB	NA	HMDB025	NA
C25 H46 N	-0.00124	-2.01239	NA	level4	NA	C17969	NA	NA
C20 H19 F	0.000782	1.938798	NA	level4	HMDB	NA	HMDB025	NA
C19 H18 O	-0.00035	-0.9683	NA	level4	HMDB	NA	HMDB025	NA
C14 H26 O	-5.14E-05	-0.19925	76.23586	level2	mzCloud	NA	HMDB000	Lipids and
C22 H22 O	-0.00013	-0.26779	25.04485	level4	Public	NA	NA	NA
C17 H21 N	0.000409	1.281383	NA	level4	HMDB	NA	HMDB025	NA
C25 H34 N	0.001754	3.578889	NA	level4	HMDB	NA	HMDB026	NA
C19 H18 N	0.000753	2.253941	NA	level4	HMDB	NA	HMDB025	NA
C32 H45 N	0.002671	4.101624	NA	level4	NA	NA	NA	NA
C9 H10 O4	0.000249	1.36768	72.74647	level2	mzCloud	NA	NA	NA
C15 H26 O	0.000574	1.718233	NA	level4	HMDB	NA	HMDB024	NA
C21 H28 F	0.001007	2.874611	NA	level4	NA	C15152	NA	NA
C40 H64 O	0.002595	3.24154	NA	level4	NA	NA	NA	NA
C11 H13 N	-0.00015	-0.5556	NA	level4	HMDB	NA	HMDB025	NA
C16 H24 O	-0.00014	-0.43644	21.57003	level4	Public	NA	NA	NA

C16 H12 C	-0.0006	-2.09617	88.45451	level2	mzCloud	C10197	NA	Flavonoids
C18 H18 N	0.000862	1.590537	NA	level4	HMDB	NA	HMDB001	Organohe
C25 H34 N	-0.00038	-0.80854	NA	level4	HMDB	NA	HMDB025	NA
C21 H27 N	-8.43E-05	-0.17816	NA	level4	HMDB	NA	HMDB025	NA
C15 H18 N	0.000782	2.207323	NA	level4	KEGG	C01313	NA	NA
C22 H22 C	37.95476	95095.21	5.089515	level4	Public	NA	NA	NA
C13 H13 N	-0.0008	-2.08585	NA	level4	HMDB	NA	HMDB001	Organohe
C17 H24 C	17.02478	43862.82	4.356767	level4	Public	NA	HMDB025	NA
C23 H34 N	-0.00116	-2.60715	NA	level4	HMDB	NA	HMDB024	NA
C16 H22 C	0.001432	3.826547	12.13033	level4	Public	C11673	HMDB003	Lipids and
C10 H20 N	0.000257	1.283216	NA	level4	HMDB	NA	HMDB025	NA
C11 H12 C	-0.00216	-10.383	65.12578	level2	mzCloud	NA	HMDB003	Phenylpro
C7 H11 N3	0.000122	0.79595	68.42544	level2	mzCloud	C05135	HMDB001	Organic ac
C26 H44 C	0.001073	2.078571	NA	level4	NA	NA	NA	NA
C23 H43 N	-3.76E-05	-0.09849	NA	level4	HMDB	NA	HMDB025	NA
C5 H6 N2	-1.77E-05	-0.18794	NA	level4	HMDB	NA	HMDB024	NA
C6 H12 N2	-0.00012	-0.51228	94.61702	level1	BMDB	C01420	HMDB000	Organic ac
C23 H25 N	0.001729	4.657299	NA	level4	HMDB	NA	HMDB025	NA
C16 H26 C	-18.0104	-67660.5	92.18036	level2	mzCloud	NA	NA	NA
C39 H40 N	-0.00282	-4.46334	NA	level4	KEGG	C09608	NA	Alkaloids
C22 H20 C	0.000426	0.894039	50.95823	level3	Public	NA	NA	NA
C29 H58 N	-0.00159	-2.74384	NA	level4	NA	NA	NA	NA
C11 H20 C	0.000286	1.552293	NA	level4	HMDB	NA	HMDB003	Lipids and
C9 H13 Cl	0.000856	3.14514	NA	level4	HMDB	NA	HMDB025	NA
C6 H6 O6	8.32E-05	0.477985	85.70483	level1	BMDB	C02341	HMDB000	Organic ac
C19 H24 C	-0.0018	-4.5438	NA	level4	NA	C16910	NA	NA
C10 H14 N	0.000183	1.127898	95.33705	level2	mzCloud	C00745	HMDB000	Organohe
C8 H19 N	-7.85E-05	-0.27362	NA	level4	HMDB	NA	HMDB025	NA
C37 H67 C	-0.00146	-2.22415	NA	level4	NA	NA	NA	NA
C9 H19 N	0.000268	1.546558	NA	level4	HMDB	NA	HMDB024	NA
C7 H5 N C	-4.73E-05	-0.22023	74.14427	level2	Public	NA	NA	NA
C6 H10 O8	21.98207	104657.8	36.7087	level4	Public	C00879	HMDB000	Organic ox
C15 H22 F	-0.0011	-3.05464	NA	level4	HMDB	NA	HMDB001	Nucleoside
C13 H13 N	6.80E-05	0.275399	NA	level4	NA	C20677	NA	NA
C5 H4 N4	0.000222	1.461009	90.57074	level1	BMDB	C00385	HMDB000	Organohe
C6 H15 O4	0.000279	1.533984	71.80401	level2	Public	NA	HMDB025	NA
C22 H20 C	37.95633	88660.08	9.216356	level4	Public	NA	NA	NA
C27 H38 C	0.000553	1.127158	18.46846	level4	Public	NA	NA	NA
C19 H23 C	-0.00034	-1.02313	NA	level4	HMDB	NA	HMDB006	Organohe
C12 H17 N	0.000163	0.636983	NA	level4	KEGG	C03142	NA	NA

C6 H13 N	0.000138	0.771123	62.27468	level2	Public	C02824	HMDB003	Organic ac
C15 H14 N	0.000119	0.416769	NA	level4	HMDB	NA	HMDB001	Organohe
C19 H22 C	-0.00011	-0.28884	NA	level4	KEGG	C09477	NA	Terpenoid
C20 H32 C	-0.00073	-1.99158	NA	level4	HMDB	NA	HMDB024	NA
C22 H36 N	0.001854	4.726649	NA	level4	HMDB	NA	HMDB024	NA
C41 H81 N	0.000197	0.270501	NA	level4	HMDB	NA	HMDB001	Lipids and
C8 H8 O3	0.000107	0.70622	89.9935	level1	BMDB	C01984	HMDB000	Benzenoid
C13 H12 N	-1.10E-05	-0.04239	NA	level4	HMDB	NA	HMDB025	NA
C30 H38 C	0.000274	0.477208	NA	level4	HMDB	NA	HMDB025	NA
C17 H27 N	-0.00021	-0.63676	NA	level4	HMDB	NA	HMDB026	NA
C23 H39 N	-0.00032	-0.91327	NA	level4	NA	C10810	NA	Alkaloids
C14 H14 N	0.000102	0.45101	NA	level4	HMDB	NA	HMDB001	Organic ox
C13 H18 N	0.001528	4.68613	NA	level4	KEGG	C04332	NA	Organohe
C18 H32 C	-2.94E-06	-0.01051	40.26704	level3	Public	NA	NA	NA
C26 H42 C	0.000593	1.228959	NA	level4	KEGG	C09093	NA	Terpenoid
C17 H32 C	5.34E-05	0.211707	NA	level4	NA	NA	NA	NA
C29 H56 N	-0.00027	-0.4488	NA	level4	HMDB	NA	HMDB025	NA
C12 H14 N	0.003558	16.31292	35.53568	level3	mzCloud	C00978	HMDB000	Organohe
C19 H26 C	55.96469	167469.7	3.241243	level4	Public	NA	NA	NA
C21 H20 C	17.02732	46254.07	4.369959	level4	Public	C10443	HMDB000	Phenylpro
C21 H33 F	-0.00159	-4.63827	NA	level4	HMDB	NA	HMDB025	NA
C18 H18 C	-0.00065	-1.88532	NA	level4	HMDB	NA	HMDB025	NA
C11 H16 N	8.14E-05	0.299205	NA	level4	HMDB	NA	HMDB024	NA
C42 H72 N	-0.0004	-0.52959	NA	level4	NA	NA	NA	NA
C29 H40 C	-0.00191	-4.08054	NA	level4	HMDB	NA	HMDB024	NA
C36 H56 C	0.001745	2.760004	NA	level4	HMDB	NA	HMDB025	NA
C35 H46 C	-0.00286	-4.56866	NA	level4	NA	NA	NA	NA
C8 H14 N2	-1.86E-05	-0.06589	NA	level4	HMDB	NA	HMDB024	NA
C11 H16 I	8.94E-05	0.278591	NA	level4	HMDB	NA	HMDB025	NA
C10 H16 N	9.25E-05	0.563825	NA	level4	HMDB	NA	HMDB024	NA
C31 H45 N	0.000719	1.405306	NA	level4	NA	NA	NA	NA
C5 H8 O2	0.000177	1.769549	NA	level4	HMDB	NA	HMDB025	NA
C31 H52 N	0.000279	0.493737	NA	level4	HMDB	NA	HMDB025	NA
C33 H34 N	0.000516	0.885845	NA	level4	HMDB	NA	HMDB024	NA
C14 H16 N	-0.00116	-4.81969	NA	level4	HMDB	NA	HMDB024	NA
C22 H24 C	21.98031	62414.37	15.71681	level4	Public	NA	NA	NA
C11 H6 O4	0.000265	1.313318	NA	level4	HMDB	NA	HMDB001	Phenylpro
C22 H28 N	0.001789	4.657224	NA	level4	HMDB	NA	HMDB025	NA
C29 H36 C	-0.0005	-1.12579	NA	level4	HMDB	NA	HMDB024	NA
C8 H6 O4	5.42E-05	0.326174	32.50909	level4	Public	NA	HMDB003	Organohe
C28 H48 C	0.000102	0.235195	NA	level4	NA	NA	NA	NA
C21 H25 N	0.00037	1.041332	NA	level4	HMDB	NA	HMDB024	NA
C18 H34 C	0.000132	0.379986	NA	level4	NA	NA	NA	NA
C15 H24 C	0.000107	0.484042	NA	level4	HMDB	NA	HMDB025	NA
C9 H18 N2	0.000183	0.839536	NA	level4	HMDB	NA	HMDB001	Organic ac
C20 H13 F	-0.00214	-4.98544	NA	level4	HMDB	NA	HMDB024	NA
C12 H18 N	0.000145	0.705638	73.77651	level2	Public	C16561	HMDB006	Organic ac
C30 H42 C	18.01093	33964.42	21.99473	level4	Public	NA	NA	NA
C9 H19 N	0.000245	1.736006	NA	level4	HMDB	NA	HMDB025	NA
C31 H48 C	0.001169	2.196786	NA	level4	NA	NA	NA	NA
C24 H34 C	-0.00018	-0.45585	NA	level4	HMDB	NA	HMDB025	NA
C36 H55 N	-0.00071	-1.05909	3.16188	level4	Public	NA	NA	NA
C18 H18 N	0.000525	1.414015	NA	level4	HMDB	NA	HMDB025	NA
C8 H10 N6	0.000469	2.110298	NA	level4	HMDB	NA	HMDB025	NA
C18 H28 C	0.001428	3.680015	NA	level4	NA	NA	NA	NA

C17 H14 N	-0.00022	-0.61173	NA	level4	NA	C12478	NA	NA
C26 H45 N	-36.0207	-72142.8	63.74557	level2	BMDB2	NA	NA	NA
C11 H14 C	0.000177	0.995049	84.11293	level2	mzCloud	NA	HMDB004	Benzenoid
C24 H23 N	0.002567	4.889016	NA	level4	HMDB	NA	HMDB025	NA
C5 H10 O:	-18.0104	-120041	76.75443	level2	BMDB	NA	HMDB003	Lipids and
C25 H43 N	-0.00047	-0.90571	NA	level4	NA	C17009	NA	NA
C25 H43 N	5.63E-05	0.124284	NA	level4	NA	C11994	NA	PK Polyke
C14 H22 C	-6.53E-05	-0.25696	NA	level4	HMDB	NA	HMDB024	NA
C21 H26 F	0.000175	0.440927	NA	level4	HMDB	NA	HMDB006	Lipids and
C26 H44 C	0.002438	4.721484	NA	level4	NA	C11991	NA	PK Polyke
C40 H74 N	0.000549	0.737833	NA	level4	NA	NA	NA	NA
C26 H40 C	0.002235	4.654035	18.2451	level4	Public	NA	HMDB025	NA
C35 H54 C	-0.00031	-0.5279	NA	level4	KEGG	C08957	NA	Terpenoid
C10 H18 C	0.000348	1.722717	NA	level4	HMDB	NA	HMDB024	NA
C14 H23 N	-0.00109	-3.40154	NA	level4	HMDB	NA	HMDB025	NA
C29 H36 N	0.000509	1.017609	NA	level4	HMDB	NA	HMDB025	NA
C29 H50 C	0.000351	0.75834	NA	level4	HMDB	NA	HMDB025	NA
C15 H24 C	0.001344	3.859382	NA	level4	HMDB	NA	HMDB024	NA
C18 H26 C	9.60E-05	0.371845	NA	level4	HMDB	NA	HMDB024	NA
C12 H17 N	0.0002	0.852683	NA	level4	HMDB	NA	HMDB026	NA
C17 H20 N	-2.82E-05	-0.0991	70.82179	level2	Public	C07404	HMDB001	Organohe
C14 H18 C	-0.00037	-1.39635	26.21561	level4	Public	NA	NA	NA
C23 H30 N	-0.00109	-2.50475	NA	level4	HMDB	NA	HMDB025	NA
C20 H22 C	-0.00017	-0.5469	NA	level4	HMDB	NA	HMDB025	NA
C13 H10 S	0.000432	2.178965	NA	level4	HMDB	NA	HMDB025	NA
C16 H31 C	-0.00012	-0.4112	NA	level4	HMDB	NA	HMDB024	NA
C39 H50 C	0.001372	1.668599	NA	level4	HMDB	NA	HMDB024	NA
C37 H56 N	-0.00264	-3.55259	NA	level4	HMDB	NA	HMDB024	NA
C18 H24 N	7.47E-05	0.224873	45.23808	level3	Public	C18504	HMDB025	NA
C27 H54 N	0.001104	2.062048	NA	level4	NA	NA	NA	NA
C4 H9 N C	-18.0104	-151274	55.57677	level3	BMDB	C00263	HMDB000	Organic ac
C18 H31 N	-0.0001	-0.31378	NA	level4	HMDB	NA	HMDB000	Lipids and
C14 H17 F	-0.0011	-3.45268	NA	level4	HMDB	NA	HMDB006	Benzenoid
C26 H46 C	-0.00147	-2.75411	NA	level4	NA	NA	NA	NA
C61 H106	-0.00086	-0.9184	NA	level4	NA	NA	NA	NA
C53 H87 C	0.002893	3.005573	NA	level4	NA	NA	NA	NA
C13 H14 N	-9.12E-05	-0.39634	NA	level4	HMDB	NA	HMDB025	NA
C20 H26 N	-0.00554	-16.9987	34.58535	level3	mzCloud	NA	NA	NA
C26 H38 C	0.002058	4.451323	NA	level4	HMDB	NA	HMDB001	Lipids and
C16 H22 N	-0.0002	-0.65089	2.555283	level4	Public	NA	NA	NA
C20 H16 C	21.98024	62427.07	16.09609	level4	Public	NA	NA	NA
C7 H5 N	6.95E-05	0.674559	NA	level4	KEGG	C09814	NA	NA
C23 H30 C	0.001664	3.339486	5.398685	level4	Public	NA	NA	NA
C14 H14 C	5.27E-05	0.228972	11.15241	level4	Public	NA	NA	NA
C29 H32 C	-0.00277	-4.24549	NA	level4	NA	NA	NA	NA
C23 H25 N	37.95745	100104.5	1.957041	level4	Public	NA	NA	NA
C2 H6 O S	0.000139	1.78156	NA	level4	HMDB	NA	HMDB024	NA
C42 H47 C	0.003042	3.428111	NA	level4	NA	NA	NA	NA
C15 H26 N	-0.00014	-0.50376	NA	level4	NA	C10756	NA	NA
C9 H8	4.28E-05	0.36834	NA	level4	HMDB	NA	HMDB025	NA
C20 H23 F	0.000982	2.542196	NA	level4	HMDB	NA	HMDB025	NA

C19 H28 (	0.000167	0.453696	76.26278	level2	mzCloud	NA	HMDB000	Lipids and
C35 H50 (	-0.00146	-2.44113	NA	level4	NA	C11980	NA	NA
C40 H71 (	0.001953	2.630757	NA	level4	NA	NA	NA	NA
C20 H36 (	-0.00026	-0.68814	NA	level4	NA	NA	NA	NA
C16 H10 S	-0.00071	-3.04577	NA	level4	NA	C20124	NA	NA
C25 H26 (	-0.00238	-5.85863	3.841	level4	Public	NA	HMDB030	NA
C27 H39 N	8.34E-05	0.196109	NA	level4	HMDB	NA	HMDB025	NA
C20 H30 (	21.98416	72741.15	38.13721	level3	BMDB	C06428	HMBD019	NA
C5 H11 N3	0.000198	1.535419	NA	level4	NA	C19778	NA	NA
C21 H20 F	-0.00175	-3.80536	NA	level4	HMDB	NA	HMDB025	NA
C21 H22 (	6.35E-05	0.187726	NA	level4	HMDB	NA	HMDB024	NA
C27 H45 N	0.000355	0.739737	NA	level4	HMDB	NA	HMDB025	NA
C11 H21 N	0.000104	0.419869	NA	level4	HMDB	NA	HMDB001	Lipids and
C20 H30 N	0.00031	0.756153	NA	level4	HMDB	NA	HMDB024	NA
C9 H12 O3	0.000238	1.418854	NA	level4	HMDB	NA	HMDB002	Benzenoid
C11 H16 N	0.000167	0.801405	NA	level4	HMDB	NA	HMDB024	NA
C10 H13 N	0.000256	1.431146	46.03661	level3	Public	NA	NA	NA
C14 H24 (	7.32E-05	0.217712	NA	level4	HMDB	NA	HMDB024	NA
C12 H12 (	0.000215	0.91105	66.5048	level2	mzCloud	NA	NA	NA
C40 H43 N	-0.00237	-3.09212	NA	level4	HMDB	NA	HMDB025	NA
C6 H6 O5	-1.43E-05	-0.07526	93.1561	level1	Decode	NA	HMDB024	Organic ac
C17 H22 N	0.000165	0.451653	NA	level4	HMDB	NA	HMDB024	Organohe
C13 H14 (	-9.89E-06	-0.03953	NA	level4	HMDB	NA	HMDB024	NA
C15 H22 F	-0.00126	-3.85701	NA	level4	HMDB	NA	HMDB025	NA
C29 H42 (	0.000385	0.818665	NA	level4	NA	NA	NA	NA
C21 H22 (	0.000219	0.548722	NA	level4	HMDB	NA	HMDB025	NA
C5 H10 N2	8.19E-05	0.629608	NA	level4	HMDB	NA	HMDB024	NA
C10 H15 N	-8.34E-05	-0.34584	NA	level4	HMDB	NA	HMDB006	Nucleoside
C12 H13 N	-0.00018	-0.72061	NA	level4	HMDB	NA	HMDB024	NA
C25 H39 N	0.000108	0.234996	NA	level4	HMDB	NA	HMDB024	NA
C17 H22 N	-1.45E-05	-0.0481	NA	level4	HMDB	NA	HMDB025	NA
C53 H76 N	-564.292	-500001	28.63534	level4	Public	NA	NA	NA
C14 H13 (	1.30E-05	0.060002	NA	level4	HMDB	NA	HMDB024	NA
C12 H11 N	-0.00019	-0.7557	NA	level4	HMDB	NA	HMDB025	NA
C8 H12 N6	0.000337	1.502874	NA	level4	HMDB	NA	HMDB024	NA
C29 H46 N	0.000706	1.005862	NA	level4	HMDB	NA	HMDB024	NA
C27 H28 N	-0.00073	-1.48035	NA	level4	HMDB	NA	HMDB025	NA
C15 H26 N	0.000246	0.631665	NA	level4	NA	C20925	NA	NA
C27 H52 (	0.000572	1.254083	NA	level4	NA	NA	NA	NA
C9 H12 F3	0.000432	1.936013	NA	level4	HMDB	NA	HMDB026	NA
C32 H52 (	0.000459	0.888822	NA	level4	NA	NA	NA	NA
C18 H22 N	-0.00018	-0.45503	NA	level4	HMDB	NA	HMDB006	Organic ox
C24 H40 N	0.000952	1.887231	NA	level4	HMDB	NA	HMDB001	Organic ni
C8 H15 N	0.000313	1.417409	48.59977	level3	Public	C05021	HMDB000	Organic ox
C16 H16 N	0.000328	0.940338	NA	level4	HMDB	NA	HMDB024	NA
C12 H19 N	0.00031	1.403142	NA	level4	HMDB	NA	HMDB001	Benzenoid

C6 H6 O3	18.01064	142905.6	56.11265	level4	Public	C11918	HMDB003	Organohe
C30 H54 N	-0.00109	-1.71259	NA	level4	NA	NA	NA	NA
C10 H17 N	-0.00026	-0.95076	50	level3	Masslist	C05283	HMDB001	Organic ac
C20 H35 N	-0.00136	-2.65227	NA	level4	NA	C21293	NA	NA
C14 H16 C	0.000194	0.69877	3.716216	level4	Public	C10948	HMDB025	NA
C4 H9 N C	0.000293	2.169431	NA	level4	HMDB	NA	HMDB000	Organic ac
C17 H14 N	-0.00089	-2.49444	NA	level4	HMDB	NA	HMDB024	NA
C23 H30 C	-3.69E-05	-0.10418	NA	level4	HMDB	NA	HMDB025	NA
C10 H14 N	-0.00029	-1.01609	NA	level4	HMDB	NA	HMDB025	NA
C16 H24 N	-0.0015	-3.8817	NA	level4	HMDB	NA	HMDB006	Organic ox
C34 H67 N	0.000704	1.310634	NA	level4	HMDB	NA	HMDB000	Lipids and
C21 H28 C	0.001499	3.663665	NA	level4	HMDB	NA	HMDB006	Organohe
C25 H30 C	-0.00206	-4.33626	2.155261	level4	Public	NA	NA	NA
C9 H17 N	21.98205	108224.3	9.881533	level4	Public	NA	HMDB024	NA
C15 H22 N	-0.00022	-0.85392	NA	level4	HMDB	NA	HMDB025	NA
C20 H32 C	-18.0106	-53566.4	78.07379	level2	mzCloud	NA	NA	NA
C20 H44 A	0.000679	1.810369	NA	level4	NA	NA	NA	NA
C23 H31 F	-0.00198	-4.69927	NA	level4	HMDB	NA	HMDB025	NA
C15 H21 N	0.001017	2.105255	6.437915	level4	Public	NA	NA	NA
C18 H24 F	0.000853	2.237487	NA	level4	NA	C18415	NA	NA
C18 H26 C	-0.00013	-0.45915	NA	level4	HMDB	NA	HMDB025	NA
C10 H12 N	0.000136	0.707216	NA	level4	HMDB	NA	HMDB025	NA
C15 H11 N	-0.00056	-2.36968	4.544898	level4	Public	NA	NA	NA
C18 H24 C	0.000267	0.764358	NA	level4	HMDB	NA	HMDB025	NA
C10 H18 N	0.000511	2.259676	NA	level4	NA	C11801	NA	NA
C6 H10	5.04E-05	0.613998	NA	level4	HMDB	NA	HMDB025	NA
C15 H20 F	0.000104	0.274239	NA	level4	HMDB	NA	HMDB025	NA
C22 H36 C	6.15E-05	0.184987	NA	level4	HMDB	NA	HMDB000	Lipids and
C18 H32 C	-8.46E-05	-0.27104	89.53926	level2	mzCloud	NA	NA	NA
C20 H24 C	0.00026	0.590737	NA	level4	HMDB	NA	HMDB024	NA
C32 H58 C	-0.0024	-3.88502	NA	level4	NA	NA	NA	NA
C19 H36 C	-6.86E-07	-0.00182	NA	level4	NA	NA	NA	NA
C17 H13 N	-0.0015	-4.7	NA	level4	HMDB	NA	HMDB024	NA
C22 H20 C	-7.02E-06	-0.01526	67.61213	level2	Public	NA	NA	NA
C29 H27 C	4.90E-05	0.087716	NA	level4	HMDB	NA	HMDB025	NA
C22 H36 N	0.000718	1.483358	NA	level4	HMDB	NA	HMDB025	NA
C29 H41 N	-0.00163	-3.49058	NA	level4	HMDB	NA	HMDB026	NA
C21 H23 C	0.001439	3.570315	NA	level4	HMDB	NA	HMDB025	NA
C10 H13 N	0.000219	0.965592	NA	level4	KEGG	C00826	NA	NA
C35 H70 N	0.002081	3.218928	NA	level4	NA	NA	NA	NA
C16 H12 C	-4.38E-06	-0.01633	NA	level4	HMDB	NA	HMDB024	NA
C26 H32 C	0.000463	0.948037	32.9553	level4	Public	NA	NA	NA
C17 H26 N	-0.00023	-0.76328	NA	level4	HMDB	NA	HMDB025	NA
C25 H37 N	0.000178	0.412611	NA	level4	HMDB	NA	HMDB006	Benzenoid
C22 H29 N	9.12E-06	0.020948	NA	level4	HMDB	NA	HMDB025	NA
C7 H10 N	0.000286	2.070861	NA	level4	HMDB	NA	HMDB024	NA
C17 H22 N	-0.00025	-0.737	14.09858	level4	Public	NA	NA	NA
C10 H13 C	-0.00017	-0.72448	NA	level4	NA	C19026	NA	NA

C36 H69 N	0.00054	0.958093	NA	level4	HMDB	NA	HMDB000	Lipids and	
C47 H80 N	0.00406	4.778777	NA	level4	HMDB	NA	HMDB025	NA	
C12 H19 N	0.00041	1.82244	NA	level4	HMDB	NA	HMDB001	Benzenoid	
C32 H42 C	0.001411	2.169744	3.531272	level4	Public	NA	NA	NA	
C14 H24 C	0.000129	0.538252	NA	level4	NA	NA	NA	NA	
C10 H13 F	-0.00109	-4.47285	NA	level4	HMDB	NA	HMDB024	NA	
C24 H38 N	-0.00037	-0.62434	NA	level4	HMDB	NA	HMDB025	NA	
C25 H52 N	0.000913	1.851456	NA	level4	NA	NA	NA	NA	
C23 H29 N	0.002145	4.883273	NA	level4	HMDB	NA	HMDB025	NA	
C10 H18 N	0.000305	1.53838	NA	level4	HMDB	NA	HMDB024	NA	
C26 H54 N	0.000654	1.289825	NA	level4	HMDB	NA	HMDB001	Lipids and	
C20 H31 F	-0.00122	-3.62043	NA	level4	NA	C15444	NA	NA	
C28 H32 C	-0.00146	-2.84998	NA	level4	NA	NA	NA	NA	
C17 H25 N	-5.74E-05	-0.18682	NA	level4	HMDB	NA	HMDB025	NA	
C21 H22 C	1.51E-05	0.034787	NA	level4	HMDB	NA	HMDB006	Benzenoid	
C20 H31 N	37.95413	99560.92	13.34065	level4	Public	NA	NA	NA	
C22 H25 C	-0.00149	-3.71442	NA	level4	HMDB	NA	HMDB025	NA	
C14 H22 C	0.000231	1.037852	9.291594	level4	Public	NA	NA	NA	
C17 H29 N	0.000548	1.294185	NA	level4	KEGG	C08336	NA	Amino aci	
C14 H20 C	-0.00021	-0.74549	NA	level4	NA	NA	NA	NA	
C22 H24 C	1.05E-05	0.03449	NA	level4	NA	NA	NA	NA	
C16 H14 N	-0.00025	-0.72112	NA	level4	HMDB	NA	HMDB024	NA	
C29 H34 C	-0.00036	-0.87618	NA	level4	HMDB	NA	HMDB025	NA	
C29 H47 C	-0.00019	-0.31067	NA	level4	HMDB	NA	HMDB025	NA	
C12 H17 N	-17.0263	-71205.4	15.78659	level4	Public	NA	NA	NA	
C9 H13 Br	0.001002	3.853858	NA	level4	NA	C10911	NA	NA	
C9 H10 O	-0.00028	-1.07374	NA	level4	HMDB	NA	HMDB004	Organic ac	
C30 H42 C	0.002033	3.420337	NA	level4	NA	NA	NA	NA	
C10 H8 N	0.000369	1.962551	NA	level4	HMDB	NA	HMDB024	NA	
C28 H41 N	-0.00241	-5.4893	56.05785	level3	mzCloud	C12271	NA	FA Fatty a	
C16 H24 C	-18.0068	-54873.3	45.85152	level3	mzCloud	NA	NA	NA	
C42 H80 N	0.003912	4.954259	NA	level4	HMDB	NA	HMDB001	Lipids and	
C7 H5 N C	0.00018	1.190012	83.09829	level2	mzCloud	NA	NA	NA	
C40 H46 N	-0.0008	-0.94063	NA	level4	KEGG	C01024	NA	Organohe	
C10 H10 C	0.000297	1.528187	14.26181	level4	Public	NA	NA	NA	
C39 H79 N	-7.23E-05	-0.1029	91.15983	level2	mzCloud	NA	NA	NA	
C18 H30 C	-0.00034	-1.10226	NA	level4	HMDB	NA	HMDB001	Lipids and	
C7 H5 N S	7.16E-05	0.530309	NA	level4	HMDB	NA	HMDB024	NA	
C24 H26 F	-0.00169	-3.96097	NA	level4	HMDB	NA	HMDB025	NA	
C22 H35 N	-0.00061	-1.47884	NA	level4	NA	NA	NA	NA	
C8 H F17 (	-0.00026	-0.51035	11.30065	level4	Public	NA	NA	NA	
C13 H25 N	5.36E-05	0.194958	NA	level4	HMDB	NA	HMDB001	Lipids and	
C40 H52 C	0.000106	0.187348	69.97529	level2	Public	C08583	HMDB000	Lipids and	
C22 H45 C	-0.00022	-0.46565	NA	level4	NA	NA	NA	NA	
C20 H22 C	21.98035	61373.35	16.60246	level4	Public	NA	NA	NA	

C29 H33 C	0.000742	1.557612	41.89386	level4	Public	C07080	HMDB000	Benzenoid
C10 H10 C	-0.00024	-0.87786	NA	level4	HMDB	NA	HMDB002	Phenylpro
C6 H10 N2	0.000206	1.184812	22.76715	level3	BMDB	NA	HMDB000	Organic ac
C16 H16 C	-5.81E-05	-0.17278	NA	level4	NA	C10296	NA	NA
C8 H10 O4	3.34E-06	0.016545	93.61789	level1	Decode	NA	HMDB006	Organic ac
C29 H54 N	-0.00303	-4.98321	NA	level4	NA	NA	NA	NA
C10 H13 N	0.000222	1.23876	NA	level4	HMDB	NA	HMDB024	NA
C34 H35 C	0.001249	2.131468	NA	level4	HMDB	NA	HMDB025	NA
C16 H24 N	-0.00034	-1.0462	NA	level4	HMDB	NA	HMDB025	NA
C7 H11 N	-18.0104	-95260.8	43.00675	level3	BMDB	C00624	HMDB000	Organic ac
C46 H84 N	0.002185	2.646633	NA	level4	NA	NA	NA	NA
C18 H32 C	-6.10E-05	-0.19528	30.53508	level4	Public	NA	NA	NA
C18 H34 C	-5.41E-05	-0.19179	NA	level4	HMDB	NA	HMDB000	Lipids and
C8 H7 N C	0.000236	1.582292	NA	level4	HMDB	NA	HMDB026	NA
C21 H27 C	-0.0001	-0.25495	NA	level4	HMDB	NA	HMDB001	Lipids and
C13 H12 C	17.02769	68641.07	16.66811	level4	Public	NA	NA	NA
C11 H12 C	-0.00012	-0.42445	NA	level4	HMDB	NA	HMDB002	Organic ac
C38 H77 N	-6.70E-05	-0.09729	NA	level4	NA	NA	NA	NA
C40 H58 C	0.002103	3.40106	NA	level4	NA	NA	NA	NA
C24 H40 C	37.95596	96754.2	14.40194	level4	Public	NA	NA	NA
C27 H56 N	0.000705	1.312845	NA	level4	NA	NA	NA	NA
C10 H13 N	0.0003	1.536146	58.00812	level3	mzCloud	C03404	HMDB002	Organic ac
C10 H12 N	0.000211	0.824235	NA	level4	HMDB	NA	HMDB025	NA
C16 H25 N	-0.00015	-0.44201	NA	level4	NA	C11775	NA	NA
C25 H26 N	0.001912	4.247638	NA	level4	HMDB	NA	HMDB025	NA
C14 H10 N	0.001791	6.731725	37.46268	level3	mzCloud	NA	NA	NA
C22 H16 C	-7.65E-06	-0.02126	NA	level4	HMDB	NA	HMDB024	NA
C24 H28 N	-0.00136	-3.10747	NA	level4	HMDB	NA	HMDB025	NA
C21 H30 C	0.00394	11.37903	53.01206	level3	mzCloud	C02140	HMDB000	Lipids and
C4 H8 O	72.05766	1000002	20.07419	level3	BMDB	C03219	HMDB003	Organic ox
C18 H31 N	1.04E-05	0.035503	NA	level4	NA	NA	NA	NA
C19 H20 C	0.005643	19.055	42.01944	level3	mzCloud	NA	NA	NA
C24 H26 C	21.98206	53592.17	10.45731	level4	Public	NA	NA	NA
C21 H44 N	1.70E-05	0.038775	NA	level4	HMDB	NA	HMDB001	Lipids and
C19 H36 C	-0.00038	-1.04969	NA	level4	NA	NA	NA	NA
C23 H35 N	-0.00136	-3.65597	NA	level4	NA	C11689	NA	NA
C21 H26 F	0.001401	2.99984	NA	level4	HMDB	NA	HMDB006	Organohe
C32 H54 C	0.000374	0.699537	NA	level4	NA	NA	NA	NA
C8 H13 N	0.000187	1.345182	NA	level4	NA	C16697	NA	NA

C16 H12 C	-8.55E-05	-0.30082	90.29857	level2	mzCloud	C14536	HMDB000	Phenylpro
C18 H24 C	0.001259	4.139572	NA	level4	HMDB	NA	HMDB024	NA
C15 H23 N	-0.00137	-4.07064	NA	level4	KEGG	C02993	NA	Organic ac
C23 H37 N	-6.21E-05	-0.17283	NA	level4	NA	NA	NA	NA
C19 H27 F	-0.00105	-3.24762	NA	level4	NA	C15164	NA	NA
C30 H44 C	0.001139	2.276651	NA	level4	KEGG	C08807	NA	Terpenoid
C14 H12 C	-6.01E-05	-0.23117	1.093047	level4	Public	C09010	HMDB025	NA
C5 H3 Cl3	0.000822	3.808088	NA	level4	HMDB	NA	HMDB024	NA
C13 H14 N	-0.00135	-4.65321	NA	level4	HMDB	NA	HMDB025	NA
C24 H40 C	0.000104	0.289067	NA	level4	HMDB	NA	HMDB024	NA
C30 H56 C	-0.00024	-0.45988	NA	level4	HMDB	NA	HMDB025	NA
C42 H38 C	-0.00157	-2.28647	NA	level4	NA	NA	NA	NA
C30 H48 C	8.87E-05	0.187801	NA	level4	HMDB	NA	HMDB025	NA
C16 H16 N	-0.00135	-4.39037	NA	level4	HMDB	NA	HMDB025	NA
C31 H40 N	-0.00091	-1.61967	NA	level4	HMDB	NA	HMDB025	NA
C16 H28 N	0.0061	16.38966	39.06	level3	mzCloud	C05552	HMDB000	Organic ac
C21 H22 C	-0.00022	-0.58905	NA	level4	HMDB	NA	HMDB006	Organohe
C30 H62 N	0.00066	1.139026	NA	level4	HMDB	NA	HMDB001	Lipids and
C25 H28 C	21.98087	48184.8	40.64838	level4	Public	NA	NA	NA
C20 H25 F	-0.00099	-2.98269	NA	level4	NA	C14885	NA	NA
C28 H28 C	0.001764	2.772724	NA	level4	NA	NA	NA	NA
C17 H19 N	0.000115	0.31381	NA	level4	HMDB	NA	HMDB006	Alkaloids a
C7 H15 Cl	0.001015	4.924789	NA	level4	HMDB	NA	HMDB024	NA
C17 H24 C	-0.00049	-1.49682	NA	level4	KEGG	C09560	NA	Terpenoid
C22 H26 F	0.001382	3.328683	NA	level4	HMDB	NA	HMDB025	NA
C11 H21 N	0.001328	3.53967	NA	level4	KEGG	C08407	NA	Amino aci
C16 H14 C	17.02645	59515.52	1.615083	level4	Public	NA	NA	NA
C12 H12 N	0.000356	1.935276	NA	level4	HMDB	NA	HMDB024	NA
C12 H16 N	0.000287	1.302727	NA	level4	HMDB	NA	HMDB025	NA
C9 H16 N6	-0.00119	-3.24088	NA	level4	NA	C20022	NA	Marine bio
C29 H53 C	0.001278	1.996021	NA	level4	NA	NA	NA	NA
C16 H26 C	-18.0104	-63825.4	12.93522	level4	Public	NA	NA	NA
C42 H83 C	-0.00206	-2.70137	NA	level4	NA	NA	NA	NA
C19 H30 N	-0.0014	-3.50702	NA	level4	HMDB	NA	HMDB025	NA
C11 H13 N	0.000263	1.269052	NA	level4	HMDB	NA	HMDB025	NA
C6 H7 N3	0.000115	0.752818	NA	level4	HMDB	NA	HMDB024	NA
C21 H41 N	-0.00092	-1.9459	NA	level4	HMDB	NA	HMDB001	Organic ox
C11 H11 N	-0.00369	-15.0595	51.05488	level3	mzCloud	C19897	NA	NA
C10 H14 N	0.000238	1.335308	NA	level4	HMDB	NA	HMDB025	NA
C42 H76 N	-0.00013	-0.15973	NA	level4	HMDB	NA	HMDB001	Lipids and
C30 H54 N	-0.00022	-0.33195	NA	level4	NA	NA	NA	NA
C20 H19 C	4.29E-05	0.091092	NA	level4	HMDB	NA	HMDB006	Organic ox
C7 H14 N	-1.00765	-6992.58	93.50411	level1	Decode	C10172	HMDB000	Organic ac
C21 H22 C	21.98035	59383.63	8.932819	level4	Public	NA	NA	NA
C24 H26 C	0.002092	4.753685	NA	level4	HMDB	NA	HMDB001	Organohe
C28 H48 N	0.003331	4.549276	NA	level4	HMDB	NA	HMDB024	NA
C32 H43 N	-0.00146	-2.88095	NA	level4	HMDB	NA	HMDB024	NA

C18 H17 F	0.000387	1.060735	NA	level4	HMDB	NA	HMDB025	NA
C30 H55 C	0.00209	3.540794	NA	level4	NA	NA	NA	NA
C8 H13 N	8.26E-05	0.594044	NA	level4	NA	C10400	NA	Alkaloids
C10 H16 N	0.000762	2.759731	NA	level4	HMDB	NA	HMDB024	NA
C18 H22 C	-0.00019	-0.59062	30.98267	level4	Public	NA	NA	NA
C19 H28 C	17.02649	55970.67	32.27872	level3	mzCloud	C05139	HMDB000	Lipids and
C14 H16 C	37.95816	152992.4	15.24283	level4	Public	NA	NA	NA
C20 H21 N	-3.22E-06	-0.00918	NA	level4	NA	C10635	NA	Alkaloids
C40 H50 N	-0.00125	-1.6933	NA	level4	HMDB	NA	HMDB025	NA
C11 H16 N	0.000956	3.184725	NA	level4	HMDB	NA	HMDB025	NA
C4 H11 N	0.000342	2.822565	NA	level4	KEGG	C07182	NA	Organic ni
C19 H40 C	-0.00016	-0.5149	NA	level4	NA	C13859	NA	FA Fatty a
C18 H18 N	-0.00012	-0.34289	NA	level4	HMDB	NA	HMDB025	NA
C9 H12 O	-0.00035	-1.33169	NA	level4	HMDB	NA	HMDB025	NA
C22 H21 C	21.98527	47681	42.85955	level3	mzCloud	NA	NA	NA
C16 H26 C	-1.72E-05	-0.0608	4.631137	level4	Public	NA	NA	NA
C9 H12 N	-5.52E-05	-0.19984	NA	level4	HMDB	NA	HMDB024	NA
C19 H38 N	-0.00139	-3.2822	NA	level4	NA	NA	NA	NA
C27 H48 N	0.002892	4.81757	5.476927	level4	Public	NA	NA	NA
C20 H14 C	5.01E-05	0.143099	NA	level4	NA	C12377	NA	NA
C14 H16 N	-0.00117	-4.12135	NA	level4	HMDB	NA	HMDB025	NA
C18 H25 N	-0.00066	-1.87743	NA	level4	HMDB	NA	HMDB024	NA
C45 H84 N	-0.00062	-0.77797	NA	level4	HMDB	NA	HMDB025	NA
C33 H38 C	0.000142	0.176526	NA	level4	HMDB	NA	HMDB003	Phenylpro
C20 H37 N	0.00114	2.162584	NA	level4	KEGG	C01688	NA	NA
C19 H18 N	-0.00013	-0.49184	NA	level4	HMDB	NA	HMDB025	NA
C20 H32 C	-8.39E-05	-0.26198	38.67702	level3	mzCloud	NA	NA	NA
C24 H52 N	0.000402	0.83485	NA	level4	HMDB	NA	HMDB024	NA
C8 H8 N2	7.92E-05	0.482496	NA	level4	HMDB	NA	HMDB025	NA
C15 H13 C	0.001544	4.57997	NA	level4	NA	C10895	NA	NA
C9 H16 N	7.26E-05	0.312769	NA	level4	HMDB	NA	HMDB024	Organic ac
C11 H10 C	17.02667	76678.45	6.843768	level4	Public	C17480	HMDB025	NA
C27 H26 C	0.000993	1.957586	NA	level4	HMDB	NA	HMDB025	NA
C20 H30 C	37.95409	108375.4	17.67509	level4	Public	NA	NA	NA
C13 H24 C	-0.0002	-0.72748	NA	level4	HMDB	NA	HMDB025	NA
C27 H53 N	0.00047	1.032576	NA	level4	HMDB	NA	HMDB000	Lipids and
C24 H42 C	-0.00022	-0.45994	NA	level4	HMDB	NA	HMDB025	NA
C27 H29 N	0.000442	0.900748	NA	level4	HMDB	NA	HMDB026	NA
C15 H18 C	21.98053	83856.59	20.34973	level4	Public	NA	NA	NA
C12 H14 N	0.000452	1.72448	NA	level4	HMDB	NA	HMDB024	NA
C30 H27 F	0.00156	2.581908	NA	level4	HMDB	NA	HMDB026	NA
C22 H32 N	0.000459	1.207621	NA	level4	HMDB	NA	HMDB024	NA
C15 H10 C	0.000168	0.661517	NA	level4	HMDB	NA	HMDB025	NA
C16 H22 N	-8.87E-05	-0.28967	NA	level4	HMDB	NA	HMDB025	NA
C13 H21 N	0.004055	10.69667	46.09345	level3	mzCloud	C03451	HMDB000	Organic ac
C21 H25 N	0.001027	2.198364	NA	level4	HMDB	NA	HMDB024	NA
C16 H12 N	-0.00149	-4.58861	NA	level4	HMDB	NA	HMDB024	NA
C22 H26 C	-8.70E-05	-0.19326	38.93774	level4	Public	NA	NA	NA
C13 H13 N	0.000119	0.433595	NA	level4	HMDB	NA	HMDB025	NA

C16 H26 (	-18.0105	-54549.4	37.34759	level3	mzCloud	NA	NA	NA
C26 H45 N	-36.0183	-72138.1	40.93109	level4	Public	NA	NA	NA
C24 H24 (	0.001745	4.113654	NA	level4	NA	NA	NA	NA
C17 H17 N	1.22E-05	0.038746	NA	level4	HMDB	NA	HMDB024	NA
C16 H20 (	-0.00025	-0.703	24.67317	level4	Public	NA	NA	NA
C22 H24 N	0.00183	4.714471	NA	level4	HMDB	NA	HMDB024	NA
C29 H56 N	0.000651	1.096372	NA	level4	HMDB	NA	HMDB025	NA
C20 H21 N	0.001057	2.379956	NA	level4	HMDB	NA	HMDB025	NA
C10 H15 N	0.000252	1.389064	32.2694	level4	Public	NA	NA	NA
C14 H20 N	-0.00039	-1.27909	NA	level4	HMDB	NA	HMDB024	NA
C18 H17 (	0.001269	3.228088	NA	level4	HMDB	NA	HMDB025	NA
C35 H44 (	-0.00253	-4.15201	NA	level4	KEGG	C09068	NA	Terpenoid
C8 H13 N3	0.0003	1.505495	NA	level4	HMDB	NA	HMDB024	NA
C26 H30 N	-0.00136	-2.86755	NA	level4	HMDB	NA	HMDB025	NA
C9 H9 N (	0.000243	1.246276	77.18189	level1	Decode	C07588	HMDB000	Benzenoid
C36 H45 N	-0.00232	-3.52621	NA	level4	HMDB	NA	HMDB024	NA
C18 H20 F	0.000336	0.931953	NA	level4	NA	C13487	NA	NA
C18 H20 F	-0.00066	-1.81578	NA	level4	HMDB	NA	HMDB000	Benzenoid
C11 H10 N	-4.11E-05	-0.20324	21.88407	level4	Public	NA	HMDB025	NA
C15 H18 N	-0.00024	-0.9115	NA	level4	HMDB	NA	HMDB024	NA
C11 H15 N	0.001345	4.003192	NA	level4	KEGG	C01185	NA	Alkaloids
C12 H14 N	0.000233	1.067645	NA	level4	HMDB	NA	HMDB025	NA
C18 H14 (	-0.00081	-2.16704	NA	level4	NA	NA	NA	NA
C13 H26 (	0.000219	1.020277	NA	level4	NA	NA	NA	NA
C20 H20 N	-0.00134	-3.25417	NA	level4	HMDB	NA	HMDB025	NA
C12 H12 N	0.000156	0.720921	NA	level4	HMDB	NA	HMDB024	NA
C10 H6 Cl	0.000548	2.142119	NA	level4	NA	C12491	NA	NA
C13 H14 (	3.92E-05	0.124842	NA	level4	HMDB	NA	HMDB012	Organic o
C20 H26 N	1.23E-06	0.003027	NA	level4	HMDB	NA	HMDB024	NA
C21 H24 (	-0.00166	-4.05081	NA	level4	NA	C18553	NA	NA
C21 H30 N	-0.00125	-3.37274	NA	level4	HMDB	NA	HMDB025	NA
C6 H12 O	-0.00023	-1.17085	18.35215	level3	BMDB	C00257	HMDB000	Organic o
C9 H10 F2	-0.00084	-3.17259	NA	level4	HMDB	NA	HMDB024	NA
C8 H17 N	0.000298	1.701846	NA	level4	HMDB	NA	HMDB025	NA
C9 H10 O	-0.00026	-0.94573	NA	level4	HMDB	NA	HMDB013	Benzenoid
C11 H13 N	0.000124	0.600514	95.60731	level1	BMDB	C03519	HMDB000	Organic a
C26 H19 N	-0.00095	-2.1037	NA	level4	HMDB	NA	HMDB024	NA
C27 H38 (	-4.29E-05	-0.11329	NA	level4	NA	NA	NA	NA
C12 H14 N	4.80E-05	0.161203	NA	level4	HMDB	NA	HMDB006	Organic a
C15 H23 N	17.02642	57297.59	7.632461	level4	Public	NA	NA	NA
C23 H24 (	21.9803	55483.78	10.42237	level4	Public	NA	NA	NA
C10 H11 N	5.35E-05	0.277298	76.81225	level2	mzCloud	NA	HMDB001	Benzenoid
C15 H12	-0.00032	-1.64708	NA	level4	NA	C19457	NA	NA
C34 H68 N	0.00036	0.55452	NA	level4	NA	NA	NA	NA
C12 H27 (	0.001279	4.073126	NA	level4	HMDB	NA	HMDB025	NA
C13 H17 N	-3.19E-05	-0.11962	NA	level4	HMDB	NA	HMDB025	NA
C20 H25 N	5.34E-05	0.132441	NA	level4	NA	C12221	NA	NA
C11 H19 N	9.00E-05	0.37306	NA	level4	HMDB	NA	HMDB024	NA
C14 H13 N	3.19E-05	0.12327	NA	level4	HMDB	NA	HMDB025	NA
C20 H27 N	17.02419	37239.16	4.536007	level4	Public	NA	NA	NA

C18 H22 C	7.93E-05	0.207643	NA	level4	HMDB	NA	HMDB024	NA
C36 H36 N	0.001993	3.387424	NA	level4	HMDB	NA	HMDB025	NA
C9 H18 N4	0.000238	0.967991	NA	level4	HMDB	NA	HMDB026	NA
C25 H24 F	-0.00062	-1.25883	NA	level4	NA	C10994	NA	NA
C6 H6 O	-3.17E-06	-0.03376	NA	level4	HMDB	NA	HMDB024	NA
C2 H7 N C	0.000106	0.755208	NA	level4	HMDB	NA	HMDB024	NA
C28 H23 N	-0.00124	-2.26241	NA	level4	KEGG	C06777	NA	NA
C29 H58 N	-0.00167	-2.96465	NA	level4	NA	NA	NA	NA
C21 H28 C	-0.00018	-0.48722	68.46974	level2	mzCloud	C07369	HMDB001	Lipids and
C17 H33 I	0.000532	1.343156	NA	level4	HMDB	NA	HMDB024	NA
C9 H8 N2	0.000146	0.912946	32.37114	level4	Public	NA	HMDB024	NA
C12 H14 N	0.000392	2.107611	25.27217	level4	Public	NA	HMDB002	Organohe
C8 H11 N	0.000183	1.333434	68.29683	level2	mzCloud	C02735	HMDB000	Organic ni
C8 H8 O4	8.26E-05	0.491434	88.00096	level2	mzCloud	C00544	HMDB000	Benzenoid
C21 H20 C	0.000388	0.865094	16.15805	level4	Public	NA	HMDB025	NA
C36 H33 N	0.002717	4.625929	NA	level4	HMDB	NA	HMDB024	NA
C36 H44 N	0.00313	4.768436	NA	level4	HMDB	NA	HMDB026	NA
C12 H14 N	0.00016	0.640072	NA	level4	HMDB	NA	HMDB025	NA
C24 H30 C	-0.00029	-0.55551	38.11678	level4	Public	NA	NA	NA
C30 H33 N	-0.0008	-1.69445	NA	level4	HMDB	NA	HMDB025	NA
C21 H27 N	-0.0005	-1.15709	NA	level4	HMDB	NA	HMDB025	NA
UnkownFc	289.095	Inf	10.37938	level4	Public	NA	NA	NA
C20 H26 C	17.02543	54188.64	39.00803	level4	Public	NA	NA	NA
C6 H12 O6	21.98219	122080.3	57.22498	level3	BMDB	C00137	HMDB000	Organic ox
C11 H20 C	8.75E-05	0.520389	NA	level4	NA	NA	NA	NA
C10 H15 N	0.000187	0.830929	NA	level4	NA	C19577	NA	NA
C14 H28 C	-5.44E-05	-0.23835	69.13685	level2	mzCloud	C06424	HMDB000	Lipids and
C5 H4 N4	-0.0013	-10.831	39.10085	level3	mzCloud	C15587	HMDB000	Organohe
C9 H18 N2	0.000281	1.392589	NA	level4	HMDB	NA	HMDB025	NA
C15 H14 C	-0.00303	-10.4535	57.90872	level3	mzCloud	C17590	HMDB012	Phenylpro
C41 H74 N	0.000285	0.385338	NA	level4	NA	NA	NA	NA
C8 H13 N6	0.000341	1.184959	NA	level4	HMDB	NA	HMDB025	NA
C7 H7 N	0.000167	1.585016	NA	level4	HMDB	NA	HMDB024	NA
C15 H17 N	17.02646	61888.37	0.732355	level4	Public	NA	NA	NA
C6 H6 N4	0.000189	1.25858	NA	level4	HMDB	NA	HMDB024	NA
C21 H35 N	17.02449	39663.92	8.540779	level4	Public	NA	NA	NA
C30 H23 B	-0.00019	-0.3573	28.21902	level4	Public	NA	NA	NA
C9 H7 I O2	-1.63E-05	-0.05335	NA	level4	HMDB	NA	HMDB025	NA
C46 H88 N	0.00042	0.516148	NA	level4	NA	NA	NA	NA
C44 H76 N	-5.46E-05	-0.06876	NA	level4	NA	NA	NA	NA
C30 H51 N	0.000387	0.742729	NA	level4	NA	NA	NA	NA
C9 H7 N C	0.00016	0.995023	NA	level4	HMDB	NA	HMDB025	NA
C18 H24 C	-5.05E-05	-0.1973	NA	level4	HMDB	NA	HMDB024	NA
C8 H12 N2	-4.80E-05	-0.2399	NA	level4	HMDB	NA	HMDB024	NA
C35 H38 N	-0.00012	-0.19159	NA	level4	HMDB	NA	HMDB025	NA
C32 H54 C	0.000847	1.685988	NA	level4	NA	NA	NA	NA
C8 H10 N2	7.68E-05	0.51151	NA	level4	HMDB	NA	HMDB024	NA
C16 H18 C	15.97483	61887.8	9.220068	level4	Public	NA	NA	NA
C16 H30 C	9.19E-05	0.361405	87.27751	level1	BMDB	C08362	HMDB000	Lipids and
C20 H32 N	9.89E-05	0.226633	NA	level4	HMDB	NA	HMDB025	NA
C16 H26 C	4.13E-05	0.125053	9.278365	level4	Public	NA	NA	NA

C13 H14 C	0.00111	4.740777	NA	level4	NA	C10426	NA	Phenylpro
C30 H44 C	-0.00212	-4.51649	NA	level4	KEGG	C02943	NA	NA
C14 H20 C	0.001491	4.950993	NA	level4	HMDB	NA	HMDB006	Benzenoid
C11 H12 N	0.000212	0.963695	60.68897	level2	mzCloud	C00643	HMDB000	Organohe
C20 H30 C	21.98176	55203.63	20.95257	level4	Public	NA	NA	NA
C6 H13 N	21.98198	112685.2	11.37275	level4	Public	NA	HMDB025	NA
C9 H12 N2	0.000179	0.734098	NA	level4	HMDB	NA	HMDB024	NA
C10 H18 C	2.14E-05	0.091197	NA	level4	HMDB	NA	HMDB026	NA
C17 H30 C	32.02602	107392.6	48.00401	level4	Public	NA	NA	NA
C28 H32 N	21.98105	45020.78	16.50492	level4	Public	NA	NA	NA
C19 H33 N	-0.00118	-2.77171	NA	level4	HMDB	NA	HMDB025	NA
C26 H36 F	21.97158	41358.7	31.43738	level3	mzCloud	NA	NA	NA
C28 H37 N	-0.002	-4.43581	NA	level4	NA	C20586	NA	NA
C19 H17 C	-2.92E-05	-0.06235	NA	level4	HMDB	NA	HMDB001	Organic ac
C32 H36 N	0.000455	0.77373	NA	level4	HMDB	NA	HMDB024	NA
C27 H38 C	0.000495	1.010545	NA	level4	HMDB	NA	HMDB025	NA
C10 H9 N	6.52E-05	0.455463	NA	level4	HMDB	NA	HMDB024	NA
C11 H13 N	-0.00018	-0.75744	NA	level4	HMDB	NA	HMDB025	NA
C24 H43 C	0.000711	1.403581	NA	level4	NA	NA	NA	NA
C22 H18 N	-0.00147	-4.75148	NA	level4	HMDB	NA	HMDB001	Benzenoid
C18 H28 N	-0.00133	-3.87166	NA	level4	NA	C11804	NA	NA
C16 H23 N	0.00015	0.389666	NA	level4	HMDB	NA	HMDB006	Organic ac
C35 H53 N	-0.00141	-2.64214	NA	level4	HMDB	NA	HMDB025	NA
C13 H12 C	17.0266	73367.26	10.49262	level4	Public	NA	NA	NA
C17 H14 C	-6.71E-05	-0.20763	NA	level4	HMDB	NA	HMDB006	Organohe
C29 H34 C	-0.00171	-2.68679	NA	level4	NA	NA	NA	NA
C22 H27 N	-4.87E-06	-0.0118	8.698194	level4	Public	NA	NA	NA
C14 H19 N	7.88E-05	0.297316	NA	level4	HMDB	NA	HMDB024	NA
C15 H15 N	0.00091	3.774463	28.55419	level4	Public	C02168	HMDB001	Benzenoid
C18 H16 C	-0.00044	-1.28967	16.54511	level4	Public	NA	HMDB025	NA
C37 H57 N	0.001293	1.748591	NA	level4	NA	C15822	NA	NA
C27 H29 N	0.000459	0.831946	NA	level4	HMDB	NA	HMDB001	Organohe
C12 H13 F	9.19E-05	0.33544	NA	level4	HMDB	NA	HMDB025	NA
C9 H9 Cl2	-0.00086	-3.5229	NA	level4	HMDB	NA	HMDB006	Benzenoid
C6 H13 N	0.000119	0.810846	NA	level4	HMDB	NA	HMDB006	Organohe
C12 H10 S	0.000823	3.77589	NA	level4	NA	C14569	NA	NA
C5 H7 N3	17.02602	98391.48	14.3617	level4	Public	NA	NA	NA
C6 H8 O7	0.000304	1.584126	NA	level4	HMDB	NA	HMDB000	Organic ac
C27 H39 N	21.98067	51683.6	25.03451	level4	Public	NA	NA	NA
C18 H14 N	-0.00072	-2.49022	NA	level4	HMDB	NA	HMDB024	NA
C6 H10 O2	0.000191	1.677137	67.10419	level2	mzCloud	NA	NA	NA
C21 H39 N	-0.00229	-3.91919	NA	level4	NA	C17573	NA	NA
C23 H24 C	-0.00138	-3.62062	NA	level4	NA	C15050	NA	NA
C19 H34 C	-0.00047	-1.42831	51.2846	level3	Public	NA	HMDB003	Lipids and
C32 H64 N	-0.0002	-0.32198	NA	level4	HMDB	NA	HMDB024	NA
C12 H22 C	-0.00114	-3.34073	NA	level4	HMDB	NA	HMDB026	NA
C20 H32 C	21.98393	68649.33	16.29331	level4	Public	NA	NA	NA
C17 H24 C	-0.00119	-2.95026	4.783833	level4	Public	NA	NA	NA
C21 H30 C	21.97946	46353.18	29.04954	level4	Public	NA	NA	NA
C24 H35 N	-2.78E-05	-0.0787	NA	level4	HMDB	NA	HMDB025	NA

C10 H16 N	0.00396	16.49336	43.92259	level3	mzCloud	C01262	HMDB000	Organic ac
C10 H13 N	0.000254	1.044977	NA	level4	HMDB	NA	HMDB024	NA
C47 H72 C	0.00237	2.477443	NA	level4	HMDB	NA	HMDB003	Lipids and
C21 H40 C	-7.07E-05	-0.21809	NA	level4	NA	NA	NA	NA
C4 H6 O5	21.9822	164019.9	3.844401	level4	Decode	C00149	HMDB000	Organic ac
C11 H9 N	1.98E-05	0.105827	77.05264	level1	NA	NA	NA	NA
C16 H21 N	1.42E-05	0.051681	56.86891	level4	Public	NA	NA	NA
C18 H24 N	-0.00135	-3.46806	NA	level4	HMDB	NA	HMDB024	NA
C6 H14 N	-0.00013	-0.52218	NA	level4	NA	C21416	NA	NA
C11 H12 C	-0.00074	-2.73164	NA	level4	HMDB	NA	HMDB005	Organic ac
C22 H33 N	0.000118	0.280993	NA	level4	HMDB	NA	HMDB024	NA
C17 H27 N	0.000164	0.44982	NA	level4	NA	C20973	NA	NA
C7 H10 N2	0.000123	0.56435	NA	level4	HMDB	NA	HMDB024	NA
C33 H38 C	0.003576	4.642936	NA	level4	NA	NA	NA	NA
C29 H32 F	0.00096	1.963218	NA	level4	HMDB	NA	HMDB025	NA
C50 H82 N	0.001776	2.001491	NA	level4	NA	NA	NA	NA
C10 H12 F	-0.00104	-4.5529	NA	level4	HMDB	NA	HMDB024	NA
C23 H28 C	-0.00056	-1.35422	NA	level4	HMDB	NA	HMDB025	NA
C9 H10 F N	7.52E-05	0.377717	NA	level4	HMDB	NA	HMDB024	NA
C8 H18 N2	0.000696	2.303249	NA	level4	HMDB	NA	HMDB025	NA
C11 H15 N	0.000213	0.964858	NA	level4	HMDB	NA	HMDB025	NA
C17 H25 N	-0.00073	-1.84135	NA	level4	NA	C18414	NA	NA
C18 H34 C	-0.0003	-0.85367	NA	level4	NA	NA	NA	NA
C11 H17 N	0.000118	0.493177	NA	level4	HMDB	NA	HMDB025	NA
C10 H12 C	0.000232	1.413648	94.23873	level2	mzCloud	C21793	HMDB000	Benzenoid
C19 H17 N	0.001461	4.208482	NA	level4	HMDB	NA	HMDB025	NA
C18 H21 B	0.001618	4.647584	NA	level4	NA	NA	NA	NA
C16 H16 N	-0.00016	-0.61456	NA	level4	NA	C11571	NA	NA
C16 H30 N	0.001764	4.520042	NA	level4	NA	NA	NA	NA
C16 H22 C	3.52E-05	0.098275	NA	level4	HMDB	NA	HMDB025	NA
C20 H30 C	-36.0216	-102857	6.030487	level4	Public	NA	NA	NA
C11 H14 C	-6.46E-05	-0.28557	NA	level4	HMDB	NA	HMDB004	Organic ac
C9 H15 N5	8.74E-05	0.272163	NA	level4	KEGG	C05252	NA	Organohe
C17 H32 C	0.001076	2.955205	NA	level4	NA	NA	NA	NA
C19 H17 C	0.00018	0.433191	NA	level4	HMDB	NA	HMDB024	NA
C18 H19 N	-4.62E-05	-0.14201	NA	level4	HMDB	NA	HMDB024	NA
C16 H21 N	-0.00011	-0.34716	NA	level4	HMDB	NA	HMDB025	NA
C15 H26	0.000188	0.913053	NA	level4	HMDB	NA	HMDB026	NA
C16 H12 C	0.000167	0.586323	NA	level4	HMDB	NA	HMDB025	NA
C5 H7 N C	9.64E-05	0.746972	91.7139	level2	mzCloud	C01877	NA	NA
C7 H7 N C	0.000209	1.365703	87.40671	level2	mzCloud	C00632	HMDB000	Benzenoid
C27 H47 N	-0.00198	-4.00421	NA	level4	HMDB	NA	HMDB025	NA
C20 H17 C	0.000186	0.431304	NA	level4	NA	C18299	NA	NA
C7 H13 N	0.000121	0.848561	66.72846	level2	mzCloud	NA	NA	NA
C21 H26 N	21.98045	62057.59	18.33322	level4	Public	NA	HMDB025	NA
C19 H32 N	0.001401	2.734892	NA	level4	HMDB	NA	HMDB025	NA
C13 H16 N	-5.98E-05	-0.25767	69.35304	level2	mzCloud	C01598	HMDB000	Organohe
C22 H23 N	37.95604	95570.43	1.988759	level4	Public	NA	NA	NA
C22 H18 F	-0.00037	-0.87994	NA	level4	HMDB	NA	HMDB025	NA
C18 H28	3.70E-05	0.151431	NA	level4	HMDB	NA	HMDB026	NA
C21 H30 C	21.98022	63487.3	22.53643	level4	Public	NA	NA	NA
C18 H14 C	-0.00162	-4.10026	26.31636	level4	Public	NA	NA	NA
C30 H34 C	-18.0097	-30722.6	0.954666	level4	Public	NA	NA	NA

C37 H63 N	-0.00106	-1.53363	4.477634	level4	Public	NA	NA	NA
C21 H26 C	21.98027	61366.88	5.864657	level4	Public	NA	NA	NA
C26 H28 C	-0.00012	-0.20144	NA	level4	NA	NA	NA	NA
C15 H16 N	-0.00115	-3.15707	NA	level4	HMDB	NA	HMDB025	NA
C25 H38 C	0.000226	0.500885	NA	level4	KEGG	C09697	NA	Terpenoid
C12 H9 Cl	0.000594	3.15913	NA	level4	HMDB	NA	HMDB024	NA
C11 H14 N	8.73E-05	0.366688	NA	level4	HMDB	NA	HMDB001	Benzenoid
C18 H25 N	-7.98E-05	-0.25318	NA	level4	HMDB	NA	HMDB001	Organic ac
C10 H12 N	0.000246	1.183852	51.18344	level3	BMDB	C00328	HMDB000	Organic ox
C52 H89 C	-0.00388	-4.07432	NA	level4	NA	NA	NA	NA
C11 H10 C	0.000175	0.654582	NA	level4	NA	C18550	NA	NA
C6 H10 N6	0.000532	2.919893	NA	level4	HMDB	NA	HMDB025	NA
C41 H47 C	-0.00132	-1.83998	NA	level4	HMDB	NA	HMDB024	NA
C33 H48 C	-0.00196	-3.98133	NA	level4	NA	NA	NA	NA
C8 H12 N2	0.000637	2.746584	NA	level4	HMDB	NA	HMDB025	NA
C8 H9 N C	0.000199	1.473794	61.07357	level2	mzCloud	C15561	NA	NA
C12 H18 N	0.000101	0.398134	50	level3	Masslist	NA	HMDB003	Organic ac
C13 H16 N	-0.00063	-2.13633	NA	level4	HMDB	NA	HMDB025	NA
C18 H26 N	-0.00022	-0.66051	NA	level4	HMDB	NA	HMDB025	NA
C12 H20 N	-0.00122	-4.82428	NA	level4	HMDB	NA	HMDB025	NA
C14 H19 N	-8.67E-05	-0.32717	29.73225	level4	Public	NA	NA	NA
C6 H8 O4	0.000101	0.702442	NA	level4	HMDB	NA	HMDB024	NA
C10 H13 N	0.000193	0.912138	NA	level4	HMDB	NA	HMDB025	NA
C18 H34 N	-0.00026	-0.64614	NA	level4	HMDB	NA	HMDB001	Organic ac
C24 H22 C	0.000431	0.806304	NA	level4	HMDB	NA	HMDB003	Phenylpro
C12 H24 C	-0.00025	-1.01103	NA	level4	NA	NA	NA	NA
C29 H31 N	-0.00052	-1.01837	NA	level4	HMDB	NA	HMDB025	NA
C49 H102	0.002886	3.577643	NA	level4	HMDB	NA	HMDB025	NA
C10 H8 Cl	-2.19E-06	-0.00967	NA	level4	NA	C21109	NA	NA
C31 H48 C	37.95768	73512.23	24.71641	level4	Public	C06694	HMDB001	Lipids and
C17 H14 C	-0.00041	-1.29798	40.11313	level4	Public	NA	NA	NA
C22 H20 F	-0.00023	-0.62785	NA	level4	HMDB	NA	HMDB025	NA
C7 H11 N3	0.000176	0.812142	NA	level4	NA	C20967	NA	NA
C19 H20 C	-1.15E-05	-0.0409	6.21247	level4	Public	NA	NA	NA
C22 H18 C	0.002277	4.905905	NA	level4	HMDB	NA	HMDB025	NA
C21 H32 C	-0.00081	-1.96655	NA	level4	HMDB	NA	HMDB000	Lipids and
C20 H19 N	-0.00129	-3.3219	NA	level4	HMDB	NA	HMDB024	NA
C8 H12 N2	-0.00221	-13.1321	60.82257	level2	mzCloud	C00534	HMDB000	Organohe
C25 H43 N	0.000824	1.81808	NA	level4	NA	NA	NA	NA
C23 H27 N	0.000169	0.370072	39.3767	level3	mzCloud	C07225	HMDB001	Phenylpro
C14 H8 O7	-0.0007	-2.20006	NA	level4	HMDB	NA	HMDB024	NA
C16 H16 N	0.000181	0.426722	NA	level4	HMDB	NA	HMDB024	NA
C21 H30 C	17.02649	49179.04	7.529982	level4	Public	NA	NA	NA
C12 H19 N	18.01062	74692.15	8.207996	level4	Public	NA	NA	NA
C19 H36 C	-0.0001	-0.30499	NA	level4	HMDB	NA	HMDB024	NA

C24 H32 C	-3.39E-05	-0.07841	NA	level4	HMDB	NA	HMDB025	NA
C46 H78 N	0.00043	0.525052	NA	level4	NA	NA	NA	NA
C9 H16 O	0.000272	1.444073	NA	level4	HMDB	NA	HMDB001	Lipids and
C12 H18 C	0.000295	1.402941	76.39338	level2	mzCloud	C08491	HMDB003	Lipids and
C22 H19 N	0.001762	4.774278	NA	level4	HMDB	NA	HMDB025	NA
C23 H29 N	0.000212	0.457944	NA	level4	HMDB	NA	HMDB006	Alkaloids a
C18 H13 N	0.001264	4.341194	NA	level4	NA	C18869	NA	NA
C37 H56 C	0.002395	3.540485	NA	level4	HMDB	NA	HMDB024	NA
C16 H15 N	0.000136	0.536367	NA	level4	HMDB	NA	HMDB025	NA
C26 H54 N	0.001626	3.013826	NA	level4	NA	NA	NA	NA
C15 H12 C	-8.51E-05	-0.31263	90.42604	level2	mzCloud	NA	NA	NA
C18 H17 N	0.001374	4.720278	NA	level4	HMDB	NA	HMDB025	NA
C11 H13 N	-0.00025	-1.31436	NA	level4	HMDB	NA	HMDB025	NA
C38 H54 C	0.00011	0.143444	NA	level4	NA	NA	NA	NA
C9 H10 O	-0.00027	-1.0984	NA	level4	HMDB	NA	HMDB009	Organic ac
C24 H44 C	0.000287	0.754879	NA	level4	NA	NA	NA	NA
C17 H27 N	-0.00334	-8.67744	53.49957	level3	mzCloud	NA	NA	NA
C19 H16 N	0.00164	4.656373	NA	level4	HMDB	NA	HMDB025	NA
C26 H37 N	21.98033	53444.06	48.14724	level3	Public	NA	NA	NA
C14 H22 N	-0.00015	-0.64033	62.91416	level2	Public	C07073	HMDB001	Benzenoid
C32 H56 C	-332.183	-500000	8.821144	level4	Public	NA	NA	NA
C15 H16 C	17.02668	58291.64	13.70583	level4	Public	NA	NA	NA
C16 H22 N	-0.00167	-4.87524	NA	level4	HMDB	NA	HMDB025	NA
C28 H36 C	0.002647	4.827853	NA	level4	KEGG	C08749	NA	Terpenoid
C14 H17 N	0.000401	1.080458	NA	level4	HMDB	NA	HMDB024	NA
C16 H21 N	-0.00028	-0.96833	NA	level4	HMDB	NA	HMDB024	NA
C16 H26 C	9.65E-05	0.385567	NA	level4	HMDB	NA	HMDB024	NA
C16 H26 N	-0.00144	-4.38049	NA	level4	NA	C12869	NA	NA
C11 H16 N	5.64E-05	0.293776	NA	level4	HMDB	NA	HMDB001	Benzenoid
C25 H39 N	0.000453	0.940763	NA	level4	HMDB	NA	HMDB025	NA
C10 H9 N	0.000232	1.327032	88.72639	level1	BMDB	C00954	HMDB000	Organohe
C19 H19 N	-0.00153	-4.6027	NA	level4	HMDB	NA	HMDB024	NA
C28 H44 C	-0.00036	-0.8194	NA	level4	NA	NA	NA	NA
C16 H18 C	21.98196	59396.26	33.80013	level4	Public	NA	NA	NA
C11 H20 N	0.000284	1.244691	NA	level4	HMDB	NA	HMDB001	Organic ac
C16 H25 N	-0.00016	-0.598	NA	level4	HMDB	NA	HMDB024	NA
C25 H40 C	0.000419	0.926245	NA	level4	NA	NA	NA	NA
C12 H22 C	0.000245	1.237867	NA	level4	NA	NA	NA	NA
C27 H30 F	4.11E-05	0.087812	NA	level4	HMDB	NA	HMDB025	NA
C14 H18 N	-3.92E-06	-0.01592	66.22344	level2	Public	C09213	HMDB006	Organic ac
C18 H30 C	-8.07E-05	-0.26011	50.29683	level3	Public	NA	NA	NA
C23 H29 N	3.84E-05	0.083002	NA	level4	HMDB	NA	HMDB006	Organic ox
C25 H43 N	9.49E-05	0.2342	NA	level4	HMDB	NA	HMDB025	NA
C20 H20 N	-0.00128	-3.775	NA	level4	KEGG	C01795	NA	Alkaloids
C10 H26 N	0.000242	1.198562	95.97095	level1	BMDB	C00750	HMDB000	Organic ni
C11 H9 N	0.000199	0.906824	50	level3	Masslist	C05830	HMDB006	Organohe
C26 H28 C	-0.00043	-0.98696	9.93309	level4	Public	NA	NA	NA
C7 H8 N2	0.000208	1.235074	NA	level4	HMDB	NA	HMDB001	Benzenoid
C32 H36 N	0.002323	3.645552	NA	level4	HMDB	NA	HMDB025	NA
C24 H45 N	0.000121	0.293245	NA	level4	NA	NA	NA	NA
C33 H49 N	0.000611	1.09934	NA	level4	NA	NA	NA	NA
C16 H18 N	-0.00013	-0.39844	NA	level4	HMDB	NA	HMDB025	NA

C40 H75 C	-0.00142	-1.51297	NA	level4	NA	NA	NA	NA
C7 H15 N3	0.000892	4.034736	NA	level4	HMDB	NA	HMDB024	NA
C28 H46 C	0.000688	1.391868	10.91525	level4	Public	NA	NA	NA
C9 H11 N5	-0.00085	-4.16076	NA	level4	HMDB	NA	HMDB024	NA
C8 H9 N C	0.000263	1.138915	NA	level4	HMDB	NA	HMDB005	Organic ac
C9 H13 N3	9.43E-05	0.415097	NA	level4	HMDB	NA	HMDB025	NA
C11 H12 N	-0.00016	-0.67622	NA	level4	HMDB	NA	HMDB024	NA
C22 H21 C	0.000228	0.495079	NA	level4	NA	C18297	NA	NA
C31 H39 N	-0.00199	-3.82595	NA	level4	HMDB	NA	HMDB024	NA
C8 H9 N C	0.000212	1.271744	NA	level4	HMDB	NA	HMDB024	NA
C19 H19 N	-0.00148	-4.28933	NA	level4	HMDB	NA	HMDB024	NA
C20 H12 C	-0.00066	-2.47352	NA	level4	HMDB	NA	HMDB024	NA
C9 H12 N2	1.68E-05	0.064775	NA	level4	HMDB	NA	HMDB025	NA
C9 H10 O	1.44E-05	0.107378	66.93737	level2	mzCloud	C02394	HMDB002	Phenylpro
C14 H15 N	0.000239	1.211101	NA	level4	HMDB	NA	HMDB024	NA
C11 H11 N	0.000117	0.460659	NA	level4	HMDB	NA	HMDB025	NA
C8 H9 F3 N	0.000788	2.792628	NA	level4	HMDB	NA	HMDB025	NA
C16 H17 N	0.000864	3.387078	7.392473	level4	Public	NA	NA	NA
C21 H26 C	-0.0002	-0.58211	NA	level4	HMDB	NA	HMDB025	NA
C29 H36 C	-0.00198	-3.63576	NA	level4	HMDB	NA	HMDB024	NA
C8 H6 O3	6.15E-05	0.409733	NA	level4	HMDB	NA	HMDB024	NA
C15 H18 C	18.01099	68712.61	20.51538	level4	Public	NA	NA	NA
C29 H52 C	-0.00204	-4.55157	NA	level4	NA	NA	NA	NA
C36 H56 C	0.000229	0.321476	NA	level4	NA	NA	NA	NA
C24 H42 C	-6.22E-05	-0.15767	NA	level4	NA	NA	NA	NA
C21 H28 C	21.98084	61025.07	0.245298	level4	Public	NA	NA	NA
C20 H23 F	-0.00138	-3.63631	NA	level4	HMDB	NA	HMDB025	NA
C17 H31 N	5.40E-06	0.018171	NA	level4	HMDB	NA	HMDB025	NA
C10 H11 B	0.000929	2.196722	NA	level4	NA	C13817	NA	NA
C21 H35 N	-0.00012	-0.36553	NA	level4	HMDB	NA	HMDB024	NA
C28 H44 C	-0.00196	-4.25829	NA	level4	NA	NA	NA	NA
C18 H19 C	-0.00144	-3.76552	NA	level4	HMDB	NA	HMDB001	Organohe
C12 H19 N	0.001439	3.385363	NA	level4	HMDB	NA	HMDB000	Organohe
C17 H16 C	17.02681	59931.43	11.85389	level4	Public	NA	NA	NA
C6 H8 O	0.000175	1.819455	NA	level4	HMDB	NA	HMDB024	NA
C54 H109	-0.00075	-0.88268	NA	level4	NA	NA	NA	NA
C5 H4 N4	0.000136	0.812335	92.33172	level1	Decode	C00366	HMDB000	Organohe
C9 H14 N4	9.00E-05	0.371786	NA	level4	HMDB	NA	HMDB024	NA
C26 H43 N	-2.01593	-4486.67	90.21395	level1	BMDB2	NA	HMDB030	NA
C33 H40 N	-0.01016	-16.7036	41.37966	level3	mzCloud	C06539	HMDB001	Alkaloids a
C6 H10 N2	0.000268	1.542349	NA	level4	HMDB	NA	HMDB024	NA
C13 H9 Cl	0.000789	2.739864	NA	level4	HMDB	NA	HMDB025	NA
C12 H21 N	0.00031	1.587961	NA	level4	HMDB	NA	HMDB006	Organic ox
C21 H19 F	0.000763	1.877393	NA	level4	HMDB	NA	HMDB025	NA
C7 H14 O6	37.95592	195569.4	25.80496	level4	Public	NA	NA	NA
C23 H25 N	0.001986	4.925244	NA	level4	HMDB	NA	HMDB024	NA
C19 H25 N	-0.00137	-3.53071	NA	level4	HMDB	NA	HMDB001	Organohe
C24 H35 N	-0.00022	-0.53742	NA	level4	HMDB	NA	HMDB025	NA
C11 H18 N	0.000271	1.196796	NA	level4	HMDB	NA	HMDB001	Organohe
C14 H11 C	-0.00033	-1.01377	NA	level4	HMDB	NA	HMDB001	Benzenoid
C14 H16 N	-0.00011	-0.36027	NA	level4	HMDB	NA	HMDB024	NA
C18 H20 F	0.001222	3.500527	NA	level4	HMDB	NA	HMDB001	Benzenoid
C12 H10 N	0.000208	0.859125	4.302591	level4	BMDB	C01727	NA	NA
C5 H9 N C	21.98207	149483.8	20.01172	level3	BMDB	NA	HMDB000	Organic ac
C17 H33 N	-0.00026	-0.90953	NA	level4	NA	NA	NA	NA
C22 H29 N	-0.00086	-2.1528	NA	level4	HMDB	NA	HMDB001	Organohe

C17 H30 C	-1.47E-05	-0.05518	NA	level4	HMDB	NA	HMDB025	NA
C20 H27 N	-0.00118	-2.63554	NA	level4	HMDB	NA	HMDB001	Benzenoid
C7 H9 N3	0.00028	1.301598	NA	level4	HMDB	NA	HMDB025	NA
C17 H18 N	-0.00074	-1.95906	NA	level4	HMDB	NA	HMDB001	Organic ac
C14 H16 N	-0.00025	-0.90717	NA	level4	HMDB	NA	HMDB006	Organic ac
C29 H39 C	0.000225	0.358923	NA	level4	HMDB	NA	HMDB024	NA
C28 H34 C	0.002257	4.256598	NA	level4	HMDB	NA	HMDB025	NA
C18 H17 N	0.001223	2.891007	NA	level4	NA	C21337	NA	NA
C26 H41 N	-4.65E-05	-0.12143	NA	level4	HMDB	NA	HMDB025	NA
C26 H30 C	21.98096	46748.69	28.96975	level4	Public	NA	NA	NA
C35 H63 N	-0.00017	-0.24297	NA	level4	HMDB	NA	HMDB006	Organic ox
C17 H17 N	-0.00026	-0.877	7.349187	level4	Public	NA	NA	NA
C16 H20 C	0.000298	0.803622	NA	level4	HMDB	NA	HMDB024	NA
C30 H33 N	21.9813	42334.76	24.04896	level4	Public	NA	NA	NA
C25 H26 C	0.000585	1.096023	NA	level4	HMDB	NA	HMDB025	NA
C24 H35 N	-5.32E-05	-0.1381	NA	level4	HMDB	NA	HMDB025	NA
C38 H40 N	-0.00161	-2.75473	NA	level4	KEGG	C09100	NA	NA
C12 H29 N	0.000559	1.619865	NA	level4	HMDB	NA	HMDB025	NA
C15 H27 N	0.001768	4.497656	NA	level4	HMDB	NA	HMDB025	NA
C29 H44 C	-0.00167	-3.7982	NA	level4	HMDB	NA	HMDB026	NA
C26 H48 C	-0.00011	-0.25854	NA	level4	HMDB	NA	HMDB024	NA
C21 H32 C	21.98236	55480.91	13.17521	level4	Public	NA	NA	NA
C21 H22 C	17.02722	48079.57	4.188921	level4	Public	NA	NA	NA
C18 H17 N	-0.00072	-2.15393	NA	level4	HMDB	NA	HMDB025	NA
C12 H17 N	0.000222	1.071843	43.60512	level4	Public	NA	HMDB025	NA
C6 H12 O3	0.00024	1.819226	NA	level4	HMDB	NA	HMDB025	NA
C10 H12 N	-0.00029	-1.08657	92.00335	level1	BMDB	C00294	HMDB000	Nucleoside
C21 H27 N	0.000309	0.740096	NA	level4	NA	C10577	NA	Organic ox
C34 H56 C	21.98087	39222.99	0.743132	level4	Public	NA	NA	NA
C10 H12 N	-0.00024	-0.80386	NA	level4	HMDB	NA	HMDB001	Organic ac
C15 H16 C	17.02657	65460.4	10.92656	level4	Public	NA	NA	NA
C14 H20 N	0.001566	4.198154	14.28904	level4	Public	C18761	HMDB025	NA
C14 H18 C	0.000181	0.831829	NA	level4	NA	NA	NA	NA
C19 H21 C	-0.00039	-0.98089	NA	level4	HMDB	NA	HMDB024	NA
C15 H27 N	-0.00131	-3.67189	NA	level4	HMDB	NA	HMDB025	NA
C25 H38 N	-0.00063	-1.26125	NA	level4	HMDB	NA	HMDB024	NA
C9 H10 Cl	0.000523	2.627392	NA	level4	HMDB	NA	HMDB024	NA
C19 H32 C	-9.23E-05	-0.28479	NA	level4	NA	NA	NA	NA
C26 H42 N	-0.00139	-2.84382	NA	level4	HMDB	NA	HMDB025	NA
C24 H40 C	-7.05E-05	-0.17276	NA	level4	HMDB	NA	HMDB000	Lipids and
C15 H23 N	0.000345	1.300758	NA	level4	HMDB	NA	HMDB001	Benzenoid
C9 H8 O4	9.64E-05	0.535153	80.61661	level1	Decode	C01179	HMDB000	Benzenoid
C34 H50 C	0.001225	1.930764	NA	level4	NA	NA	NA	NA
C47 H82 N	0.000735	0.896693	NA	level4	HMDB	NA	HMDB024	Lipids and
C19 H19 C	0.001342	3.202947	NA	level4	HMDB	NA	HMDB025	NA
C18 H21 C	-0.00141	-4.46443	NA	level4	HMDB	NA	HMDB006	Organohe
C33 H38 N	0.000698	1.190568	NA	level4	HMDB	NA	HMDB024	NA

C29 H57 (	-0.00198	-3.51222	NA	level4	NA	NA	NA	NA
C27 H43 N	6.39E-05	0.148858	NA	level4	HMDB	NA	HMDB025	NA
C17 H23 N	-0.00013	-0.51655	NA	level4	HMDB	NA	HMDB025	NA
C33 H34 N	0.000645	1.077541	NA	level4	NA	C20667	NA	NA
C25 H27 N	-0.00063	-1.33577	NA	level4	HMDB	NA	HMDB006	Organohe
C5 H7 N3	0.000277	1.964908	NA	level4	HMDB	NA	HMDB024	NA
C22 H44 N	-0.00128	-2.75363	NA	level4	HMDB	NA	HMDB001	Lipids and
C46 H82 N	0.00059	0.702622	NA	level4	NA	NA	NA	NA
C9 H12 N2	-0.00016	-0.64217	NA	level4	HMDB	NA	HMDB025	NA
C37 H38 F	-0.00306	-4.3092	NA	level4	HMDB	NA	HMDB025	NA
C12 H22 (	21.98193	64252.81	41.85372	level3	mzCloud	C01971	NA	NA
C6 H10 N2	8.00E-05	0.421138	NA	level4	HMDB	NA	HMDB001	Organic ac
C12 H20 (	0.000159	0.749394	25.06256	level4	Public	NA	HMDB003	Lipids and
C12 H21 N	-0.0001	-0.45457	NA	level4	HMDB	NA	HMDB025	NA
C17 H20 (	37.95392	103104.6	20.72173	level4	Public	NA	NA	NA
C24 H44 (	-3.27E-05	-0.08251	NA	level4	NA	NA	NA	NA
C9 H9 N (	0.000423	2.170198	NA	level4	HMDB	NA	HMDB024	NA
C15 H10 (	-18.0106	-62963.4	14.77017	level4	Public	NA	NA	NA
C14 H21 N	5.76E-05	0.233237	NA	level4	HMDB	NA	HMDB025	NA
C22 H38 (	-8.44E-05	-0.25251	NA	level4	HMDB	NA	HMDB024	NA
C23 H20 F	-0.00196	-4.61049	NA	level4	HMDB	NA	HMDB024	NA
C3 H6 N6	0.000268	1.205906	NA	level4	HMDB	NA	HMDB025	NA
C20 H22 (	3.04E-05	0.081182	25.34731	level4	Public	NA	NA	NA
C14 H26 N	-0.00021	-0.74856	NA	level4	NA	C11333	NA	NA
C8 H12 O3	0.000185	1.18246	NA	level4	NA	NA	NA	NA
C25 H33 (	-0.002	-3.90925	NA	level4	NA	NA	NA	NA
C11 H13 N	9.87E-06	0.03868	0.988886	level4	Public	NA	NA	NA
C8 H15 N	0.000181	1.444363	NA	level4	HMDB	NA	HMDB025	NA
C19 H26 (	17.02646	46499.06	2.047636	level4	Public	NA	NA	NA
C41 H79 (	0.002752	3.608748	NA	level4	NA	NA	NA	NA
C18 H19 N	0.000624	2.219655	NA	level4	HMDB	NA	HMDB006	Organohe
C19 H20 F	0.000661	1.872185	NA	level4	HMDB	NA	HMDB024	NA
C20 H14 (	-0.00029	-0.92441	NA	level4	HMDB	NA	HMDB025	NA
C15 H18 N	-0.00133	-4.92088	NA	level4	HMDB	NA	HMDB025	NA
C17 H19 N	-0.00018	-0.55778	NA	level4	HMDB	NA	HMDB024	NA
C22 H22 (	37.95493	85077.6	8.442781	level4	Public	NA	NA	NA
C9 H6 F3 I	0.000202	0.930236	NA	level4	HMDB	NA	HMDB024	NA
C10 H4 Cl	0.000583	2.208738	NA	level4	HMDB	NA	HMDB024	NA
C23 H32 (	21.98279	40101.83	4.382159	level4	Public	NA	NA	NA
C19 H23 N	0.000401	1.109377	NA	level4	HMDB	NA	HMDB024	NA
C11 H10 (	0.001337	4.987923	NA	level4	HMDB	NA	HMDB025	NA
C27 H32 F	0.001656	3.196447	NA	level4	NA	NA	NA	NA
C23 H31 B	-0.00206	-4.92932	NA	level4	NA	NA	NA	NA
C21 H25 N	0.001188	3.132828	NA	level4	HMDB	NA	HMDB024	NA
C17 H19 N	-0.0014	-4.77659	NA	level4	HMDB	NA	HMDB001	Benzenoid
C43 H78 N	0.003043	3.805618	NA	level4	NA	NA	NA	NA
C25 H18 F	0.001215	2.449967	NA	level4	HMDB	NA	HMDB025	NA
C11 H17 N	2.63E-05	0.086757	NA	level4	NA	C20026	NA	Marine bic
C31 H60 N	0.001035	1.623287	NA	level4	NA	NA	NA	NA
C15 H17 N	-0.00015	-0.45379	NA	level4	HMDB	NA	HMDB001	Organic o:
C15 H21 F	-0.00086	-2.71185	7.416206	level4	Public	NA	NA	NA
C12 H16 N	0.000329	1.610343	44.94368	level3	mzCloud	C08299	HMDB004	Organohe
C27 H41 F	-0.00144	-3.16442	NA	level4	NA	NA	NA	NA
C35 H42 N	-0.00041	-0.66263	NA	level4	HMDB	NA	HMDB024	NA
C30 H50 (	-0.00207	-4.21811	NA	level4	HMDB	NA	HMDB025	NA
C10 H8 O2	0.000205	1.279116	NA	level4	HMDB	NA	HMDB025	NA

C7 H9 N C	0.000169	1.092408	NA	level4	HMDB	NA	HMDB025	NA
C13 H13 N	-0.00019	-0.77197	NA	level4	NA	NA	NA	NA
C49 H96 A	0.00177	1.744386	NA	level4	NA	NA	NA	NA
C21 H18 N	0.000825	2.30315	NA	level4	NA	C22145	NA	NA
C14 H23 N	3.97E-05	0.156984	NA	level4	HMDB	NA	HMDB024	NA
C35 H56 N	-0.00052	-0.61099	NA	level4	HMDB	NA	HMDB025	NA
C31 H46 C	0.000937	1.619469	NA	level4	NA	NA	NA	NA
C7 H15 N	0.000166	1.147096	96.40689	level2	mzCloud	C01996	HMDB000	Organic ni
C17 H14 C	-0.00049	-1.65046	NA	level4	HMDB	NA	HMDB025	NA
C14 H25 N	0.001087	2.085652	NA	level4	NA	C11435	NA	NA
C24 H50 N	-0.00029	-0.58933	NA	level4	HMDB	NA	HMDB025	NA
C12 H17 N	0.00114	4.301853	NA	level4	HMDB	NA	HMDB000	Organohe
C8 H11 N3	2.93E-05	0.127764	NA	level4	HMDB	NA	HMDB001	Nucleosid
C15 H24 C	18.01068	76259.05	26.5795	level4	Public	NA	NA	NA
C5 H4 O4	0.000189	1.474161	NA	level4	HMDB	NA	HMDB025	NA
C17 H9 N	-0.00085	-3.10099	NA	level4	HMDB	NA	HMDB025	NA
C24 H30 C	-0.00033	-0.75889	NA	level4	HMDB	NA	HMDB025	NA
C10 H11 N	0.000249	1.288968	NA	level4	HMDB	NA	HMDB006	Benzenoid
C18 H20 C	0.00013	0.457846	NA	level4	HMDB	NA	HMDB024	NA
C54 H87 N	-0.00249	-2.4833	NA	level4	KEGG	C05845	NA	NA
C11 H14 C	0.000221	1.243351	NA	level4	HMDB	NA	HMDB025	NA
C20 H17 F	0.001413	3.277751	NA	level4	HMDB	NA	HMDB025	NA
C26 H34 N	0.002449	4.121849	NA	level4	HMDB	NA	HMDB025	NA
C45 H89 N	-0.00017	-0.21672	NA	level4	HMDB	NA	HMDB001	Lipids and
C12 H14 C	0.000179	0.805887	NA	level4	HMDB	NA	HMDB000	Benzenoid
C16 H18 N	-151.063	-499999	29.07641	level4	Public	NA	NA	NA
C16 H12 C	-0.00043	-1.82146	NA	level4	NA	C18255	NA	NA
C9 H10 N2	-3.70E-05	-0.19042	88.54909	level2	mzCloud	D01421	HMDB000	Benzenoid
C12 H14 N	-0.00019	-0.8225	48.74711	level3	mzCloud	NA	NA	NA
C15 H27 N	0.001413	4.95422	NA	level4	HMDB	NA	HMDB001	Lipids and
C10 H16 C	0.000176	0.956764	NA	level4	HMDB	NA	HMDB025	NA
C11 H14 N	0.000166	0.624964	NA	level4	HMDB	NA	HMDB025	NA
C11 H11 N	0.000308	1.503818	75.1652	level2	mzCloud	C05660	HMDB000	Organohe
C22 H20 C	0.000531	1.114595	NA	level4	NA	NA	NA	NA
C23 H31 N	-0.00062	-1.32791	NA	level4	HMDB	NA	HMDB024	NA
C17 H24 N	-0.00033	-1.08239	NA	level4	HMDB	NA	HMDB025	NA
C19 H40 N	0.000179	0.421527	NA	level4	NA	NA	NA	NA
C26 H47 C	0.002433	4.554176	NA	level4	NA	NA	NA	NA
C7 H15 N	0.000284	1.603401	9.903003	level4	Public	NA	NA	NA
C19 H32 N	-0.002	-4.05939	NA	level4	HMDB	NA	HMDB024	NA
C24 H21 F	-0.00037	-0.9048	NA	level4	HMDB	NA	HMDB025	NA
C30 H57 N	-0.0026	-4.5501	NA	level4	HMDB	NA	HMDB025	NA
C14 H26 N	-0.00019	-0.54662	NA	level4	NA	C21160	NA	NA
C13 H16 N	0.000886	3.354436	40.10921	level3	mzCloud	C05642	HMDB000	Organic ox
C14 H17 N	0.00115	4.436308	NA	level4	HMDB	NA	HMDB006	Organohe
C26 H28 C	0.000546	0.967311	NA	level4	NA	NA	NA	NA
C10 H6 O3	0.000382	2.193893	NA	level4	HMDB	NA	HMDB025	NA
C5 H12 O9	-0.00053	-1.89776	NA	level4	NA	C11453	NA	NA
C30 H40 N	-0.00049	-0.80842	NA	level4	HMDB	NA	HMDB024	NA
C11 H14 N	0.000273	1.004547	NA	level4	HMDB	NA	HMDB006	Organic ox
C30H21N3	0.000913	1.269374	NA	level4	KEGG	C06230	NA	NA
C22 H24 N	0.000394	0.833596	NA	level4	HMDB	NA	HMDB025	NA
C24 H44 N	0.001815	4.276462	29.94361	level4	Public	NA	NA	NA

C14 H16 C	9.71E-05	0.36769	NA	level4	HMDB	NA	HMDB024	NA
C10 H11 N	0.00014	0.724005	49.50054	level3	mzCloud	C05598	HMDB000	Organic ac
C22 H15 C	-0.00079	-2.1974	NA	level4	HMDB	NA	HMDB024	NA
C10 H14 C	0.000255	1.698612	6.836851	level4	Public	C09908	HMDB000	Lipids and
C21 H24 F	-0.00116	-2.42757	NA	level4	HMDB	NA	HMDB025	NA
C28 H24 N	0.0019	4.895703	NA	level4	HMDB	NA	HMDB025	NA
C18 H15 F	0.001431	3.825286	NA	level4	HMDB	NA	HMDB025	NA
C13 H15 N	-3.50E-05	-0.13401	43.19005	level4	Public	C18864	HMDB025	NA
C14 H19 N	-0.0009	-2.51341	NA	level4	KEGG	C06565	NA	NA
C12 H20 N	3.03E-05	0.11131	NA	level4	HMDB	NA	HMDB001	Benzenoid
C20 H36 C	-8.31E-05	-0.26972	81.12825	level2	mzCloud	C16525	HMDB000	Lipids and
C24 H32 C	-0.00033	-0.78812	6.94625	level4	Public	NA	NA	NA
C22 H27 N	0.000571	1.167699	NA	level4	HMDB	NA	HMDB025	NA
C14 H18 N	-0.00027	-0.82538	NA	level4	HMDB	NA	HMDB025	NA
C20 H31 N	0.001421	3.578056	NA	level4	HMDB	NA	HMDB025	NA
C16 H22 C	0.000251	0.70132	NA	level4	NA	NA	NA	NA
C22 H37 N	1.69E-05	0.046552	NA	level4	HMDB	NA	HMDB001	Organic ni
C18 H18 C	21.98034	82592.28	4.801115	level4	Public	NA	NA	NA
C6 H12 N2	8.16E-05	0.728155	NA	level4	HMDB	NA	HMDB024	NA
C18 H28 C	2.02E-05	0.069082	NA	level4	NA	NA	NA	NA
C17 H22 C	-4.76E-05	-0.17346	NA	level4	HMDB	NA	HMDB025	NA
C21 H21 N	-0.00033	-0.86225	NA	level4	HMDB	NA	HMDB025	NA
C16 H20 C	-4.23E-05	-0.11377	NA	level4	HMDB	NA	HMDB004	Organic ox
C9 H9 N C	0.000182	1.114552	NA	level4	HMDB	NA	HMDB024	NA
C12 H19 N	0.000116	0.480511	NA	level4	HMDB	NA	HMDB024	NA
C21 H14 C	-3.64E-05	-0.09628	NA	level4	NA	NA	NA	NA
C18 H34 C	-1.05E-06	-0.0037	74.21217	level2	mzCloud	NA	NA	NA
C17 H16 C	17.02627	56735.38	7.477495	level4	Public	NA	NA	NA
C21 H14	0.000824	3.09578	NA	level4	NA	C19391	NA	NA
C30 H42 C	0.002588	4.737108	NA	level4	KEGG	C08860	NA	Terpenoid
C43 H59 N	2.50E-05	0.029614	NA	level4	HMDB	NA	HMDB024	NA
C30 H32 C	0.00166	3.006563	10.09695	level4	Public	NA	NA	NA
C21 H18 C	2.61E-06	0.006836	9.953228	level4	Public	NA	NA	NA
C18 H20 N	0.001328	4.740073	NA	level4	HMDB	NA	HMDB024	NA
C18 H30 C	-7.98E-05	-0.28684	87.33672	level2	BMDB	C06426	HMDB000	Lipids and
C18 H28 N	-0.00015	-0.43943	NA	level4	HMDB	NA	HMDB001	Organic ox
C8 H17 N	0.000206	1.43646	NA	level4	HMDB	NA	HMDB025	NA
C18 H29 N	8.66E-05	0.281997	8.494294	level4	Public	C16952	HMDB003	Benzenoid
C37 H75 N	-0.00042	-0.61971	NA	level4	HMDB	NA	HMDB001	Lipids and
C16 H31 F	0.001209	3.948644	NA	level4	HMDB	NA	HMDB024	NA
C24 H34 C	-18.0105	-41476.9	27.5985	level4	Public	NA	NA	NA
C28 H49 C	0.002089	3.728198	NA	level4	NA	NA	NA	NA
C31 H48 C	-0.00203	-4.33374	NA	level4	HMDB	NA	HMDB025	NA
C13 H18 C	0.000185	0.972913	NA	level4	NA	NA	NA	NA
C26 H56 N	-0.00219	-4.17703	NA	level4	NA	NA	NA	NA
C22 H41 N	-2.83E-05	-0.07712	NA	level4	NA	NA	NA	NA
C14 H15 N	-5.23E-05	-0.17131	NA	level4	HMDB	NA	HMDB025	NA

C22 H39 N	-0.00034	-0.85873	NA	level4	HMDB	NA	HMDB024	NA
C15 H12 C	-0.00023	-0.88581	NA	level4	HMDB	NA	HMDB024	NA
C23 H35 N	-6.88E-05	-0.19266	NA	level4	HMDB	NA	HMDB025	NA
C21 H22 C	3.88E-05	0.092842	5.098048	level4	Public	NA	NA	NA
C17 H35 N	3.46E-05	0.128635	NA	level4	NA	C17515	NA	Organic ac
C44 H54 N	-0.00105	-1.37061	NA	level4	HMDB	NA	HMDB026	NA

C10 H12 N	0.000283	1.179376	NA	level4	HMDB	NA	HMDB024	NA
C10 H11 N	-0.00101	-4.67345	NA	level4	HMDB	NA	HMDB025	NA
C30 H46 C	0.000547	0.966635	NA	level4	NA	NA	NA	NA
C17 H19 N	-0.00106	-3.24826	NA	level4	HMDB	NA	HMDB025	NA
C30 H37 N	-0.00222	-4.66887	NA	level4	HMDB	NA	HMDB025	NA
C29 H46 C	0.000288	0.627344	NA	level4	HMDB	NA	HMDB025	NA
C22 H40 C	-3.90E-05	-0.11583	NA	level4	HMDB	NA	HMDB024	NA
C17 H16 N	0.000176	0.594287	NA	level4	HMDB	NA	HMDB024	NA
C34 H58 C	0.000781	1.38777	NA	level4	NA	NA	NA	NA
C18 H30 C	1.51E-05	0.046165	70.42458	level2	mzCloud	NA	NA	NA
C5 H7 N C	-0.01754	-120.955	65.52939	level1	Decode	C00940	HMDB000	Organic ac
C6 H11 N	0.000196	1.349246	82.48584	level2	BMDB	C02946	HMDB000	Organic ac
C15 H15 N	-0.0005	-1.46858	NA	level4	HMDB	NA	HMDB001	Organic ox
C20 H18 C	37.95697	117834.7	21.47704	level4	Public	NA	HMDB025	NA
C21 H26 C	17.02587	45502.68	20.25587	level4	Public	NA	NA	NA
C10 H9 N	0.000357	1.867032	91.29064	level1	BMDB	C05635	HMDB000	Organohe
C36 H63 C	0.002496	3.398105	NA	level4	NA	NA	NA	NA
C26 H40 C	0.000396	0.882709	21.62042	level4	Public	NA	NA	NA
C20 H20 C	17.02595	47808.77	29.28583	level4	Public	NA	NA	NA
C14 H29 N	0.001956	4.006276	NA	level4	KEGG	C04767	NA	NA
C34 H69 C	0.000633	0.970895	NA	level4	NA	NA	NA	NA
C32 H34 C	37.9563	65643.9	2.770804	level4	Public	NA	NA	NA
C19 H18 F	3.99E-05	0.096952	NA	level4	HMDB	NA	HMDB026	NA
C5 H7 N C	0.000187	1.649953	84.00986	level1	Decode	C03912	HMDB000	Organic ac
C25 H24 N	-0.00129	-2.86896	NA	level4	HMDB	NA	HMDB025	NA
C17 H31 N	-0.00106	-3.11244	NA	level4	HMDB	NA	HMDB025	NA
C6 H15 N3	0.000198	1.229466	NA	level4	HMDB	NA	HMDB025	NA
C20 H26 N	-0.00098	-2.31797	NA	level4	NA	C15712	NA	NA
C10 H11 N	0.000221	1.143137	46.01082	level3	mzCloud	NA	HMDB000	Organic ac
C21 H23 N	-0.00099	-2.32753	NA	level4	HMDB	NA	HMDB025	NA
C20 H32 C	-9.23E-06	-0.03034	40.20004	level3	Public	NA	HMDB003	Lipids and
C15 H16 N	-6.69E-05	-0.26104	NA	level4	HMDB	NA	HMDB025	NA
C15 H18 C	17.02647	54905.46	17.05626	level4	Public	C04164	HMDB030	NA
C19 H16 C	17.02646	47815.1	3.273117	level4	Public	NA	NA	NA
C26 H46 N	37.95778	63018.51	14.60013	level4	Public	NA	NA	NA
C43 H48 C	0.000429	0.458335	NA	level4	NA	C11539	NA	NA
C13 H8 O	4.46E-05	0.210391	NA	level4	HMDB	NA	HMDB025	NA
C18 H35 C	-1.37E-05	-0.04317	NA	level4	HMDB	NA	HMDB024	NA
C18 H15 F	0.000708	2.699495	NA	level4	HMDB	NA	HMDB025	NA
C44 H56 C	0.003391	4.869487	NA	level4	HMDB	NA	HMDB025	NA
C12 H16 N	3.35E-05	0.132806	NA	level4	HMDB	NA	HMDB001	Organohe
C12 H17 N	0.000256	1.146512	NA	level4	HMDB	NA	HMDB001	Organohe
C8 H19 O3	0.000458	1.58032	NA	level4	NA	C19009	NA	NA
C34 H53 N	0.001162	1.784681	NA	level4	HMDB	NA	HMDB025	NA
C14 H20 C	0.000351	1.717002	NA	level4	NA	NA	NA	NA
C17 H33 C	-0.00133	-3.51076	NA	level4	NA	NA	NA	NA
C7 H11 N	0.000212	1.505908	NA	level4	HMDB	NA	HMDB025	NA
C9 H17 N	0.000274	1.347252	NA	level4	HMDB	NA	HMDB006	Organic ac
C17 H27 N	-0.00012	-0.39576	NA	level4	HMDB	NA	HMDB001	Benzenoid
C15 H21 N	-0.00144	-3.99791	NA	level4	HMDB	NA	HMDB001	Organic ox

C17 H28 (	-0.00016	-0.54036	NA	level4	NA	C11993	NA	PK Polyke
C10 H10 N	4.79E-05	0.275215	42.12996	level3	BMDB	C02693	HMDB002	Organohe
C25 H47 N	1.46E-05	0.035708	NA	level4	HMDB	NA	HMDB025	NA
C11 H18 N	-3.03E-05	-0.13393	NA	level4	HMDB	NA	HMDB025	NA
C27 H48 (	-0.00055	-1.0263	NA	level4	NA	NA	NA	NA
C20 H32 (	3.60E-05	0.107082	80.65651	level2	mzCloud	NA	NA	NA
C23 H21 F	0.000646	1.774687	NA	level4	HMDB	NA	HMDB025	NA
C8 H11 N	0.000233	1.376744	92.83481	level1	BMDB	C00314	HMDB000	Organohe

C23 H23 (	0.001286	2.706571	NA	level4	NA	NA	NA	NA
C14 H12 N	-0.0014	-4.9949	NA	level4	HMDB	NA	HMDB001	Benzenoid
C14 H15 N	9.10E-05	0.220325	NA	level4	HMDB	NA	HMDB004	Organohe
C9 H10 O	0.000112	0.838079	12.57528	level4	Public	C01710	HMDB005	Benzenoid

C20 H34 (	-9.53E-05	-0.31126	NA	level4	HMDB	NA	HMDB025	NA
C16 H25 N	0.000866	3.103694	NA	level4	NA	C18898	NA	NA
C20 H31 N	-0.00135	-2.19071	NA	level4	HMDB	NA	HMDB026	NA
C26 H30 (	-0.00122	-2.50164	13.54101	level4	Public	NA	NA	NA
C23 H31 (	-0.00165	-3.76562	NA	level4	HMDB	NA	HMDB001	Lipids and

C23 H25 N	-0.00035	-0.96791	NA	level4	HMDB	NA	HMDB025	NA
C18 H30 (	-0.00021	-0.71427	92.26157	level2	mzCloud	C14766	HMDB000	Lipids and
C16 H27 N	0.001244	3.040662	NA	level4	KEGG	C08333	NA	Amino aci
C24 H17 N	-0.00106	-2.40867	NA	level4	HMDB	NA	HMDB025	NA

C19 H25 N	-0.00059	-1.69144	NA	level4	HMDB	NA	HMDB025	NA
C15 H25 N	-0.00177	-3.43163	NA	level4	NA	C21354	NA	NA
C22 H23 (	0.000577	1.366064	41.07541	level3	Public	C07072	HMDB001	Benzenoid
C19 H24 N	-0.00119	-3.238	NA	level4	HMDB	NA	HMDB024	NA

C22 H26 F	-0.00146	-3.23165	NA	level4	HMDB	NA	HMDB025	NA
C11 H17 N	0.000304	1.1898	NA	level4	NA	C19247	NA	NA
C14 H25 N	0.000137	0.50659	13.83664	level4	Public	NA	NA	NA
C17 H20 N	-0.00153	-3.76101	NA	level4	HMDB	NA	HMDB025	NA
C11 H16 N	0.000212	0.944438	28.06694	level4	Public	NA	NA	NA
C5 H7 N (	0.000198	1.531225	NA	level4	HMDB	NA	HMDB025	NA
C34 H60 (	17.02603	25171.42	4.437237	level4	Public	NA	NA	NA
C16 H26 N	0.000836	1.886544	NA	level4	HMDB	NA	HMDB024	NA
C20 H20 (	-0.00114	-2.43115	1.037496	level4	Public	NA	NA	NA
C8 H12 N2	0.000386	1.927931	25.73212	level4	Public	NA	NA	NA
C28 H46 (	0.001641	3.549696	NA	level4	NA	NA	NA	NA
C15 H18 N	-0.0002	-0.73148	NA	level4	HMDB	NA	HMDB024	NA
C22 H43 N	-5.83E-05	-0.15777	NA	level4	NA	NA	NA	NA
C10 H9 N	0.000158	0.761774	NA	level4	HMDB	NA	HMDB024	NA
C19 H30 N	0.00157	3.024228	NA	level4	HMDB	NA	HMDB025	NA
C8H10N3.	0.000937	3.988363	NA	level4	NA	C13681	NA	NA
C8 H7 N (	0.000154	1.156902	83.65222	level2	mzCloud	C12312	HMDB006	Organohe
C20 H20 (	-0.00052	-1.40922	4.288352	level4	Public	C10190	HMDB003	Phenylpro
C23 H28 F	-0.00183	-3.50191	NA	level4	HMDB	NA	HMDB001	Organohe
C30 H32 (	-0.00237	-4.85992	NA	level4	NA	NA	NA	NA
C5 H12 N2	21.98295	122088.9	47.38375	level3	BMDB	C03510	HMDB002	Organic ac
C5 H6 O2	0.000209	2.131036	NA	level4	HMDB	NA	HMDB024	NA
C17 H22 (	-9.16E-05	-0.31567	NA	level4	NA	C17746	NA	NA
C12 H20 N	-0.00023	-0.74993	NA	level4	NA	C20962	NA	NA
C20 H28 (	0.000105	0.245679	NA	level4	HMDB	NA	HMDB025	NA
C15 H16 (	17.02645	52537.07	7.458907	level4	Public	NA	HMDB025	NA
C39 H64 (	-0.0019	-2.57161	NA	level4	NA	C17075	NA	NA

C23 H45 N	1.16E-05	0.03037	NA	level4	NA	NA	NA	NA
C21 H39 N	-17.0262	-40803.6	2.119073	level4	Public	NA	NA	NA
C24 H30 C	-0.00223	-4.0705	NA	level4	HMDB	NA	HMDB025	NA
C21 H30 C	0.00034	1.031091	NA	level4	HMDB	NA	HMDB024	NA
C23 H32 N	-0.0014	-3.27387	NA	level4	HMDB	NA	HMDB025	NA
C21 H20 C	-0.00013	-0.30361	NA	level4	HMDB	NA	HMDB004	Phenylpro
C9 H7 N C	0.00053	2.994422	NA	level4	HMDB	NA	HMDB024	NA

C30 H41 F	-0.0006	-0.90005	NA	level4	HMDB	NA	HMDB025	NA
C29 H40 C	0.000619	1.277655	NA	level4	NA	C12150	NA	NA
C16 H23 N	0.001014	3.554989	NA	level4	HMDB	NA	HMDB025	NA
C20 H36 C	0.006552	14.55308	37.96597	level3	mzCloud	C05847	HMDB000	Lipids and
C9 H17 N	0.000225	1.20327	NA	level4	HMDB	NA	HMDB025	NA
C25 H40 N	-0.00075	-1.4688	NA	level4	NA	C15710	NA	NA
C17 H24 C	37.95411	117085.5	33.93684	level4	Public	NA	NA	NA
C20 H24 C	17.02662	51883.94	14.93093	level4	Public	NA	NA	NA
C22 H20 N	-0.00117	-2.71677	NA	level4	HMDB	NA	HMDB025	NA
C36 H70 C	0.00013	0.161292	NA	level4	HMDB	NA	HMDB024	NA
C9 H16 O	0.000222	1.587763	NA	level4	HMDB	NA	HMDB003	Organic o:
C19 H26 C	0.001137	2.746167	29.79627	level4	Public	NA	NA	NA
C28 H40 C	0.000144	0.365813	NA	level4	HMDB	NA	HMDB024	NA
C17 H30 C	2.99E-05	0.105879	NA	level4	NA	NA	NA	NA
C15 H26 C	6.44E-05	0.238473	NA	level4	NA	C16506	NA	NA
C23 H50 N	-0.00013	-0.27452	NA	level4	NA	NA	NA	NA
C23 H30 C	6.69E-05	0.188795	28.38912	level4	Public	NA	NA	NA
C4 H9 N C	0.000138	1.585561	59.93842	level3	Public	C14452	HMDB003	Organohe
C20 H25 C	-0.0003	-0.74119	NA	level4	HMDB	NA	HMDB025	NA
C8 H13 N3	-0.00015	-0.62396	NA	level4	HMDB	NA	HMDB025	NA
C15 H18 C	8.42E-06	0.025828	NA	level4	HMDB	NA	HMDB024	NA
C25 H29 N	-0.00018	-0.43206	NA	level4	HMDB	NA	HMDB025	NA
C27 H41 N	0.000143	0.348876	NA	level4	HMDB	NA	HMDB025	NA
C35 H46 C	0.000963	1.394589	NA	level4	HMDB	NA	HMDB025	NA
C11 H18 C	21.98202	95526.06	60.04829	level2	Public	NA	NA	NA
C6 H11 N	0.000282	1.592933	NA	level4	HMDB	NA	HMDB025	NA
C21 H31 N	-0.00013	-0.31353	NA	level4	HMDB	NA	HMDB000	Organic ac
C6 H12 O8	0.00075	3.074608	NA	level4	NA	C22078	NA	NA
C21 H24 C	-0.002	-4.76125	NA	level4	HMDB	NA	HMDB024	NA
C43 H83 C	-0.0018	-2.19173	NA	level4	NA	NA	NA	NA
C17 H26 C	1.30E-05	0.030804	NA	level4	NA	C11644	NA	NA
C3 H8 N2	0.000113	1.284085	50.26838	level4	Public	NA	NA	NA
C8 H6 N2	0.000119	0.737313	NA	level4	HMDB	NA	HMDB024	NA
C27 H35 N	-0.00142	-3.30243	NA	level4	HMDB	NA	HMDB025	NA
C22 H30 N	-0.00046	-1.03221	NA	level4	HMDB	NA	HMDB024	NA
C23 H20 C	-0.00064	-1.22248	NA	level4	HMDB	NA	HMDB003	Lipids and
C7 H6 N2	0.000755	3.069631	NA	level4	HMDB	NA	HMDB025	NA
C20 H30 C	21.98066	62764.34	7.39451	level4	Public	NA	NA	NA

C20 H13 F	0.001509	3.189599	NA	level4	HMDB	NA	HMDB025	NA
C18 H18	0.000931	3.977242	NA	level4	HMDB	NA	HMDB025	NA
C17 H16 C	17.0258	59927.87	9.107759	level4	Public	NA	NA	NA
C29 H50 C	21.98051	53043.54	48.31577	level3	Public	C01753	HMDB000	Lipids and
C20 H18 C	17.0265	46004.47	7.778601	level4	Public	NA	NA	NA
C21 H24 C	21.98029	59061.84	4.61002	level4	Public	C10545	HMDB003	Lignans, n
C12 H18 N	-0.00011	-0.44573	41.81249	level3	Public	NA	HMDB001	Organohe
C24 H26 C	-0.00025	-0.51497	NA	level4	NA	NA	NA	NA
C7 H6 O	0.000261	2.458411	NA	level4	HMDB	NA	HMDB000	Benzenoid
C14 H16 C	0.001142	3.993967	NA	level4	HMDB	NA	HMDB025	NA
C18 H25 N	0.00062	1.764814	NA	level4	HMDB	NA	HMDB025	NA
C20 H40 C	-4.10E-05	-0.13846	NA	level4	NA	NA	NA	NA
C14 H22 C	0.000225	1.090895	NA	level4	HMDB	NA	HMDB024	NA
C15 H21 N	-0.00031	-0.95736	NA	level4	HMDB	NA	HMDB001	Benzenoid
C17 H17 C	-0.00063	-1.55611	NA	level4	HMDB	NA	HMDB006	Organohe
C5 H6 N2	180.0132	1139088	10.3479	level4	Public	C00337	HMDB000	Organic ac
C26 H28 C	-1.86E-05	-0.03301	9.154972	level4	Public	NA	NA	NA
C24 H48 N	-0.00017	-0.33213	NA	level4	NA	NA	NA	NA
C19 H26 C	0.000179	0.468952	NA	level4	HMDB	NA	HMDB001	Organic ox
C28 H41 N	-0.00203	-4.61117	NA	level4	HMDB	NA	HMDB024	NA
C21 H34 C	17.02673	38158.12	31.3426	level4	Public	NA	NA	NA
C10 H8 N2	0.000187	1.195559	53.99353	level3	mzCloud	C02938	HMDB000	Organohe
C9 H13 Cl	0.001012	4.68578	NA	level4	NA	C10963	NA	NA
C42 H83 C	-0.00123	-1.64288	NA	level4	NA	NA	NA	NA
C18 H17 N	-4.29E-05	-0.11952	41.92277	level3	mzCloud	NA	NA	NA
C6 H14 N2	0.000172	1.177228	89.30661	level1	BMDB	C00047	HMDB000	Organic ac
C19 H20 N	-0.00016	-0.48907	NA	level4	HMDB	NA	HMDB025	NA
C20 H30 C	-0.00054	-1.69221	31.60948	level3	mzCloud	C14732	HMDB001	Lipids and
C20 H26 C	-0.00016	-0.50514	NA	level4	HMDB	NA	HMDB024	NA
C13 H4 Cl	-0.00115	-2.38601	NA	level4	HMDB	NA	HMDB024	NA
C15 H17 C	-0.00147	-4.31092	NA	level4	HMDB	NA	HMDB025	NA
C13 H13 C	-0.00058	-2.33191	NA	level4	HMDB	NA	HMDB024	NA
C21 H28 C	17.02623	43413.94	8.720171	level4	Public	NA	NA	NA
C24 H29 N	-0.00153	-3.42356	NA	level4	HMDB	NA	HMDB025	NA
C14 H20 C	-0.00023	-0.87093	NA	level4	NA	NA	NA	NA
C14 H14 N	-2.83E-06	-0.01034	NA	level4	HMDB	NA	HMDB025	NA
C18 H33 N	-0.0002	-0.72306	NA	level4	NA	NA	NA	NA
C10 H8 O	-0.00013	-0.46989	NA	level4	NA	C16198	NA	NA
C20 H29 N	0.001724	3.819851	NA	level4	HMDB	NA	HMDB024	NA
C16 H17 N	0.000767	3.005684	NA	level4	HMDB	NA	HMDB024	NA
C42 H85 C	-0.00272	-3.71109	NA	level4	NA	NA	NA	NA
C24 H46 C	0.000158	0.431112	92.40052	level2	mzCloud	C08323	HMDB000	Lipids and
C12 H24 C	6.33E-05	0.255185	NA	level4	NA	NA	NA	NA
C24 H40 C	-251.141	-615107	56.89231	level3	BMDB2	NA	HMDB000	Lipids and
C30 H42 C	-0.00016	-0.28772	NA	level4	NA	NA	NA	NA
C28 H50 N	-0.0027	-4.57359	NA	level4	NA	NA	NA	NA
C19 H22 N	0.002261	4.892284	NA	level4	HMDB	NA	HMDB024	NA
C10 H19 N	0.00017	1.002356	NA	level4	NA	C10773	NA	Alkaloids
C10 H10 N	6.02E-05	0.345874	NA	level4	HMDB	NA	HMDB024	NA
C30 H42 C	-288.622	-485677	0.563544	level4	Public	NA	NA	NA
C20 H32 N	0.000277	0.727639	NA	level4	HMDB	NA	HMDB024	NA
C18 H20 C	17.02627	56728.5	9.699914	level4	Public	NA	NA	NA
C15 H19 N	-7.45E-05	-0.24406	NA	level4	HMDB	NA	HMDB025	NA
C18 H21 N	0.000658	2.087283	NA	level4	HMDB	NA	HMDB024	NA

C18 H18 (	0.000132	0.40485	NA	level4	HMDB	NA	HMDB001	Organohe
C10 H9 N	0.000312	1.633983	NA	level4	HMDB	NA	HMDB025	NA
C22 H26 N	17.02644	44549.77	6.712392	level4	Public	NA	NA	NA
C6 H4 N2	0.000163	1.361286	NA	level4	HMDB	NA	HMDB025	NA
C25 H26 (	-0.0007	-1.79078	NA	level4	HMDB	NA	HMDB025	NA
C9 H12 F N	-0.00113	-4.62924	NA	level4	HMDB	NA	HMDB006	Nucleosid
C36 H67 N	0.000442	0.787574	NA	level4	NA	NA	NA	NA
C16 H22 (	-0.00021	-0.76296	94.1244	level2	mzCloud	C03343	HMDB001	Benzenoid
C40 H86 N	0.001994	2.534687	NA	level4	KEGG	C04862	NA	NA
C21 H20 (	0.000211	0.488815	23.43138	level4	Public	C04608	HMDB003	Phenylpro
C31 H46 (	0.000198	0.425493	NA	level4	HMDB	NA	HMDB024	NA
C12 H22 (	46.00539	134473	81.46387	level2	mzCloud	C00243	HMDB000	Organic o:
C30 H39 N	-0.00015	-0.27254	NA	level4	HMDB	NA	HMDB025	NA
C9 H10 O2	0.000223	1.48661	82.32954	level2	mzCloud	NA	NA	NA
C8 H18 N2	0.000244	1.399902	NA	level4	HMDB	NA	HMDB025	NA
C10 H19 N	0.001622	4.49244	NA	level4	KEGG	C08418	NA	Amino aci
C10 H10 (	0.000142	0.875534	87.1478	level2	mzCloud	C06358	HMDB003	Phenylpro
C44 H82 N	0.000649	0.796308	NA	level4	NA	NA	NA	NA
C8 H20 O5	-0.00136	-4.21602	NA	level4	HMDB	NA	HMDB025	NA
C43 H74 (	-0.00106	-1.38088	NA	level4	NA	NA	NA	NA
C16 H22 (	0.000146	0.494855	NA	level4	HMDB	NA	HMDB025	NA
C32 H38 N	0.002857	4.940053	NA	level4	HMDB	NA	HMDB001	Alkaloids a
C18 H21 N	-0.0014	-3.60432	NA	level4	HMDB	NA	HMDB024	NA
C22 H28 (	17.0266	42125.91	7.957208	level4	Public	NA	NA	NA
C23 H24 N	-0.00114	-2.75039	NA	level4	HMDB	NA	HMDB025	NA
C23 H34 (	37.95749	101424	5.112341	level4	Public	NA	NA	NA
C11 H16 F	-0.00116	-4.26119	NA	level4	HMDB	NA	HMDB024	NA
C18 H30 (	-3.70E-06	-0.01409	NA	level4	HMDB	NA	HMDB024	NA
UnkownFc	376.2053	Inf	2.485243	level4	Public	NA	NA	NA
C19 H22 N	0.000388	0.990472	NA	level4	HMDB	NA	HMDB001	Organohe
C21 H27 N	-0.0005	-1.40875	96.54989	level2	mzCloud	C09558	HMDB003	Organohe
C24 H38 (	4.55E-06	0.011205	NA	level4	HMDB	NA	HMDB024	NA
C5 H12 O4	-0.00031	-1.54146	NA	level4	NA	C11498	NA	NA
C8 H7 N (	0.000168	1.264515	NA	level4	HMDB	NA	HMDB024	NA
C11 H12 (	0.0007	2.301355	NA	level4	HMDB	NA	HMDB005	Organic a
C10 H16 N	21.98207	90057.93	64.91641	level2	mzCloud	C00120	HMDB000	Organohe
C7 H11 N	0.000185	0.976791	NA	level4	HMDB	NA	HMDB000	Organic a
C18 H24 (	-0.00023	-0.62504	13.49183	level4	Public	NA	NA	NA
C12 H8 Cl	0.000679	2.121074	NA	level4	NA	C19533	NA	NA
C19 H28 (	-9.11E-05	-0.31594	54.66163	level3	mzCloud	NA	HMDB000	Lipids and
C22 H36 (	0.000112	0.283167	NA	level4	NA	NA	NA	NA
C6 H5 N3	5.19E-05	0.436096	NA	level4	HMDB	NA	HMDB025	NA
C20 H34 (	-0.00039	-1.34178	NA	level4	HMDB	NA	HMDB024	NA
C26 H32 (	0.00084	1.779046	NA	level4	NA	NA	NA	NA
C17 H26 (	-18.0105	-61222.1	91.77605	level2	mzCloud	C10462	NA	Skimate /
C18 H25 N	-0.00029	-1.00104	NA	level4	NA	C10153	NA	Alkaloids
C26 H26 (	-0.00161	-3.23044	NA	level4	NA	NA	NA	NA
C21 H33 N	-0.00016	-0.43395	NA	level4	KEGG	C08667	NA	Alkaloids
C20 H33 N	8.46E-05	0.212017	NA	level4	HMDB	NA	HMDB024	NA
C24 H37 N	-0.00022	-0.60082	NA	level4	HMDB	NA	HMDB025	NA
C17 H14 (	-3.35E-05	-0.07865	NA	level4	NA	NA	NA	NA
C21 H20 (	21.98031	65391.08	19.95889	level4	Public	NA	NA	NA
C27 H30 (	0.000538	1.046116	NA	level4	HMDB	NA	HMDB024	NA

C20 H29 N	-0.00095	-2.86678	NA	level4	HMDB	NA	HMDB026	NA
C33 H40 N	-0.00325	-4.95262	NA	level4	HMDB	NA	HMDB025	NA
C18 H26 N	-0.00018	-0.48611	NA	level4	HMDB	NA	HMDB024	NA
C18 H22 N	-0.00169	-4.72897	NA	level4	HMDB	NA	HMDB025	NA
C31 H26 N	0.001933	4.076464	NA	level4	HMDB	NA	HMDB024	NA
C20H2Cl4I	0.004949	4.863032	NA	level4	NA	C20347	NA	NA
C19 H27 N	-2.50E-05	-0.07514	NA	level4	HMDB	NA	HMDB025	NA
C10 H6 O3	4.62E-05	0.265241	88.53262	level2	mzCloud	C10368	NA	Others
C17 H17 C	3.05E-05	0.065945	NA	level4	HMDB	NA	HMDB025	NA
C10 H12 N	4.70E-05	0.267052	5.892591	level4	Decode	NA	HMDB000	Organohe
C9 H8 Cl N	-0.00051	-1.68628	NA	level4	NA	C20939	NA	NA
C43 H75 C	0.002123	2.60702	NA	level4	NA	NA	NA	NA
C17 H19 N	-0.00045	-1.42509	NA	level4	HMDB	NA	HMDB025	NA
C13 H18 N	0.000189	0.866387	40.6468	level4	Public	NA	HMDB024	NA
C20 H16 N	-0.00155	-4.49156	NA	level4	HMDB	NA	HMDB025	NA
C30 H36 N	-0.00196	-3.14138	NA	level4	HMDB	NA	HMDB024	NA
C27 H21 N	-0.0003	-0.66792	3.836794	level4	Public	NA	NA	NA
C16 H12 C	-0.0002	-0.67285	NA	level4	HMDB	NA	HMDB025	NA
C30 H50 C	-18.0102	-36728	19.09927	level4	Public	NA	NA	NA
C21 H24 C	-0.00066	-1.9401	NA	level4	HMDB	NA	HMDB024	NA
C21 H39 C	0.001766	4.222192	NA	level4	NA	NA	NA	NA
C5 H10 O2	6.81E-05	0.666852	NA	level4	HMDB	NA	HMDB025	NA
C14 H28 C	0.000685	2.222456	NA	level4	HMDB	NA	HMDB025	NA
C29 H38 C	18.01098	39999.78	9.813951	level4	Public	NA	HMDB000	Lipids and
C16 H12 C	-0.00027	-1.01734	87.91961	level2	mzCloud	C00858	HMDB000	Phenylpro
C9 H10 O4	6.94E-05	0.381242	89.55899	level1	BMDB	C03672	HMDB000	Phenylpro
C12 H11 C	0.000279	0.920256	NA	level4	HMDB	NA	HMDB024	NA
C15 H12 C	3.20E-05	0.13316	1.961699	level4	Public	NA	NA	NA
C18 H15 N	0.000376	1.088735	NA	level4	HMDB	NA	HMDB025	NA
C18 H22 F	-0.00031	-0.89811	NA	level4	HMDB	NA	HMDB024	NA
C15 H19 N	-6.27E-05	-0.2402	NA	level4	HMDB	NA	HMDB025	NA
C18 H21 N	-0.00121	-3.03749	NA	level4	HMDB	NA	HMDB024	NA
C23 H19 N	0.001843	4.64048	NA	level4	HMDB	NA	HMDB025	NA
C16 H20 C	-3.01E-06	-0.01091	NA	level4	NA	C17745	NA	NA
C12 H21 N	6.74E-05	0.237979	NA	level4	HMDB	NA	HMDB025	NA
C16 H14 N	-0.00023	-0.68206	NA	level4	HMDB	NA	HMDB024	NA
C8 H15 N	0.000265	1.292499	1.989685	level3	BMDB	C00248	HMDB000	Organohe
C22 H27 N	-0.00126	-2.96325	NA	level4	HMDB	NA	HMDB025	NA
C33 H40 C	0.001323	1.911131	NA	level4	NA	NA	NA	NA
C29 H28 C	-0.00135	-2.59263	NA	level4	HMDB	NA	HMDB025	NA
C16 H19 N	0.00017	0.756794	NA	level4	HMDB	NA	HMDB025	NA
C43 H75 C	0.003569	4.56033	NA	level4	NA	NA	NA	NA
C18 H35 C	-0.00182	-4.61072	NA	level4	NA	NA	NA	NA
C32 H44 C	0.002346	3.882309	NA	level4	KEGG	C08873	NA	Terpenoid
C36 H70 C	0.003191	4.935858	NA	level4	NA	NA	NA	NA
C18 H37 N	3.95E-06	0.010861	NA	level4	NA	NA	NA	NA
C12 H24 N	3.85E-06	0.015779	NA	level4	HMDB	NA	HMDB025	NA
C8 H14 O3	18.01094	113925.3	14.60148	level3	Decode	NA	HMDB001	Organic ac
C36 H54 C	-0.00333	-4.90746	NA	level4	HMDB	NA	HMDB003	Lipids and
C6 H6 N2	0.000156	1.130933	92.58817	level1	BMDB	C00785	HMDB000	Organohe
C20 H38 N	0.001008	2.36376	NA	level4	HMDB	NA	HMDB025	NA

C8 H12 N2	-1.04E-05	-0.04825	NA	level4	HMDB	NA	HMDB000	Organic ac
C20 H34 C	17.02655	48064.92	9.967892	level4	Public	NA	NA	NA
C14 H18 N	-0.00126	-3.15223	NA	level4	HMDB	NA	HMDB025	NA
C31 H46 C	0.000602	1.24829	NA	level4	HMDB	NA	HMDB025	NA
C8 H16 N2	0.000271	1.735313	NA	level4	HMDB	NA	HMDB024	NA
C19 H37 A	0.000165	0.425469	NA	level4	NA	NA	NA	NA
C19 H12 F	-0.00078	-1.85556	NA	level4	HMDB	NA	HMDB026	NA
C7 H14 N2	2.97E-05	0.170316	79.33895	level2	mzCloud	NA	NA	NA
C30 H32 C	0.001743	3.30649	NA	level4	HMDB	NA	HMDB001	Benzenoid
C6 H6 O3	4.51E-05	0.357464	72.16038	level2	mzCloud	C02183	HMDB001	Benzenoid
C24 H47 B	1.996165	3399.335	6.761681	level4	Public	NA	NA	NA
C9 H7 N C	9.44E-05	0.651126	39.46675	level4	Public	C06343	HMDB024	NA
C16 H31 B	-0.00162	-4.83845	NA	level4	NA	NA	NA	NA
C19 H21 C	0.001591	4.569615	NA	level4	HMDB	NA	HMDB025	NA
C15 H15 C	0.000122	0.388384	NA	level4	NA	C18613	NA	NA
C6 H10 O2	0.000108	0.945357	76.507	level1	BMDB2	NA	HMDB003	Lipids and
C30 H52 C	-0.00019	-0.38478	NA	level4	NA	NA	NA	NA
C26 H26 C	-0.00036	-0.87017	NA	level4	NA	NA	NA	NA
C14 H18 N	0.000135	0.519665	NA	level4	NA	C21112	NA	NA
C21 H38 C	21.98157	54644.94	25.51269	level4	Public	NA	NA	NA
C19 H21 F	-0.00031	-0.95974	NA	level4	HMDB	NA	HMDB025	NA
C16 H17 N	-7.38E-05	-0.26083	NA	level4	HMDB	NA	HMDB025	NA
C19 H32 C	37.95397	106546.5	16.14553	level4	Public	NA	NA	NA
C40 H56 C	0.000347	0.610777	NA	level4	HMDB	NA	HMDB024	NA
C16 H28 C	-3.12E-05	-0.11626	NA	level4	NA	NA	NA	NA
C18 H10 N	-0.00065	-2.41422	NA	level4	HMDB	NA	HMDB025	NA
C14 H15 N	-3.27E-05	-0.11154	NA	level4	NA	C12256	NA	NA
C21 H16 C	-0.00131	-2.71848	NA	level4	HMDB	NA	HMDB006	Organic ox
C19 H39 N	-8.15E-06	-0.02475	NA	level4	NA	NA	NA	NA
C17 H23 N	-4.78E-05	-0.17496	6.451919	level4	Public	NA	NA	NA
C12 H16 N	-3.68E-05	-0.13736	NA	level4	HMDB	NA	HMDB025	NA
C26 H45 N	0.000939	1.821713	NA	level4	HMDB	NA	HMDB025	NA
C7 H13 N3	-0.00011	-0.5239	NA	level4	KEGG	C04673	NA	NA
C21 H26 C	-0.00033	-0.75576	14.22283	level4	Public	NA	NA	NA
C9 H13 N5	-0.00357	-13.9817	30.78085	level3	mzCloud	C04874	HMDB000	Organohe
C32 H40 C	-0.00122	-1.75381	NA	level4	NA	NA	NA	NA
C14 H24 C	-0.00011	-0.36689	NA	level4	NA	NA	NA	NA
C39 H37 N	0.000223	0.313213	NA	level4	HMDB	NA	HMDB006	Benzenoid
C32 H43 C	0.000918	1.656556	NA	level4	HMDB	NA	HMDB025	NA
C8 H6 O5	9.33E-05	0.512587	NA	level4	HMDB	NA	HMDB024	NA
C17 H19 N	-0.00016	-0.47793	76.54572	level2	Public	C07324	HMDB000	Organohe
C13 H20 C	-5.56E-05	-0.20432	NA	level4	NA	NA	NA	NA
C20 H16 N	-0.00063	-1.80905	NA	level4	HMDB	NA	HMDB024	NA
C24 H30 C	0.000285	0.639427	6.037603	level4	Public	NA	HMDB025	NA
C21 H22 C	0.000505	1.121999	7.855404	level4	Public	NA	NA	NA
C22 H33 N	0.00025	0.554797	NA	level4	NA	C11221	NA	NA
C9 H12 N2	-0.00029	-1.12247	NA	level4	HMDB	NA	HMDB024	NA
C22 H22 F	0.001591	3.869282	NA	level4	HMDB	NA	HMDB024	NA
C12 H14 C	-0.00013	-0.52754	30.4524	level4	Public	NA	NA	NA
C33 H38 N	-0.00051	-0.82382	NA	level4	HMDB	NA	HMDB006	Organic ox
C23 H39 N	0.000534	1.256783	NA	level4	NA	NA	NA	NA
C20 H30 C	17.02834	35611.57	0.577896	level4	Public	NA	NA	NA
C17 H37 N	-9.04E-05	-0.31479	NA	level4	NA	NA	NA	NA
C22 H24 C	21.98029	59702.72	39.03473	level4	Public	NA	NA	NA

C20 H14	-0.00017	-0.6832	NA	level4	HMDB	NA	HMDB026	NA	
C30 H38 C	-0.00027	-0.56249	NA	level4	HMDB	NA	HMDB001	Lipids and	
C22 H29 N	-1.81E-05	-0.05149	NA	level4	HMDB	NA	HMDB024	NA	
C11 H18 N	0.002175	4.749497	NA	level4	NA	C20183	NA	Nucleosid	
C22 H31 C	37.95756	69880.73	3.374823	level4	Public	NA	NA	NA	
C24 H39 N	-4.46E-05	-0.11466	NA	level4	NA	NA	NA	NA	
C35 H57 N	0.000624	0.935089	NA	level4	NA	C15682	NA	NA	
C8 H16 N2	-1.72E-05	-0.09147	NA	level4	HMDB	NA	HMDB024	NA	
C22 H34 C	-4.03E-05	-0.09819	NA	level4	HMDB	NA	HMDB025	NA	
C28 H29 N	-0.0007	-1.69201	NA	level4	HMDB	NA	HMDB024	NA	
C19 H31 N	-0.00109	-2.58756	NA	level4	HMDB	NA	HMDB025	NA	
C20 H22 C	-1.44E-07	-0.00039	NA	level4	HMDB	NA	HMDB025	NA	
C15 H8 O	-0.00021	-0.75358	NA	level4	HMDB	NA	HMDB025	NA	
C8 H15 N3	-0.00029	-1.33782	NA	level4	HMDB	NA	HMDB024	NA	
C20 H30 C	0.001148	2.669201	NA	level4	NA	NA	NA	NA	
C15 H22 N	0.00166	4.839432	NA	level4	NA	NA	NA	NA	
C15 H25 N	-0.00019	-0.66369	NA	level4	HMDB	NA	HMDB006	Benzenoid	
C8 H9 F N	-0.00082	-4.12014	NA	level4	HMDB	NA	HMDB024	NA	
C25 H26 C	0.000797	1.448305	NA	level4	NA	NA	NA	NA	
C20 H19 C	-0.00126	-3.37041	NA	level4	NA	C18571	NA	NA	
C10 H15 N	0.00019	0.889845	NA	level4	HMDB	NA	HMDB025	NA	
C21 H30 N	-0.00175	-4.35625	NA	level4	HMDB	NA	HMDB001	Benzenoid	
C15 H16	0.000196	0.998734	NA	level4	NA	C14581	NA	Phenylpro	
C11 H16 N	0.003996	14.90282	45.47609	level3	mzCloud	NA	NA	NA	
C23 H29 F	-0.00196	-4.88671	NA	level4	HMDB	NA	HMDB024	NA	
C23 H31 F	-0.00185	-4.72959	NA	level4	NA	C15310	NA	NA	
C27 H32 N	0.00041	0.826054	NA	level4	NA	NA	NA	NA	
C20 H28 C	-2.53E-05	-0.06392	NA	level4	HMDB	NA	HMDB025	NA	
C29 H46 C	-0.00055	-1.29092	NA	level4	NA	C22118	NA	NA	
C26 H51 C	0.00092	1.709097	NA	level4	NA	NA	NA	NA	
C27 H31 C	-0.00274	-4.97385	NA	level4	HMDB	NA	HMDB006	Organic o	
C23 H22 C	2.52E-05	0.049782	NA	level4	NA	NA	NA	NA	
C26 H34 C	17.02913	32609.73	8.345025	level4	Public	NA	NA	NA	
C14 H12 N	0.000545	2.161455	NA	level4	HMDB	NA	HMDB024	NA	
C27 H38 C	0.000499	0.954896	NA	level4	KEGG	C09563	NA	Terpenoid	
C11 H15 N	0.001317	4.40438	NA	level4	NA	C16352	NA	Alkaloids	
C36 H54 C	-0.00258	-4.19323	NA	level4	KEGG	C09192	NA	Terpenoid	
C33 H44 N	0.001762	2.975248	NA	level4	KEGG	C05790	NA	Organohe	
C41 H81 C	-0.00184	-2.30847	NA	level4	NA	NA	NA	NA	
C27 H36 F	0.000843	1.762333	NA	level4	HMDB	NA	HMDB025	NA	
C30 H48 C	0.000917	1.877444	32.50047	level4	Public	NA	HMDB003	Lipids and	
C19 H16 I	0.000845	1.547928	NA	level4	NA	C15143	NA	NA	
C20 H20 F	0.001501	4.021613	NA	level4	HMDB	NA	HMDB001	Organohe	
C32 H60 C	0.00067	1.079536	NA	level4	NA	NA	NA	NA	
C20 H34 C	17.02661	50337.98	43.78522	level3	mzCloud	NA	NA	NA	
C11 H21 N	-0.00016	-0.69157	NA	level4	HMDB	NA	HMDB000	Lipids and	
C22 H43 N	-4.61E-05	-0.13058	NA	level4	NA	NA	NA	NA	
C26 H44 N	0.000824	1.581206	88.07255	level2	BMDB2	NA	NA	NA	
C21 H36 N	-0.00079	-1.66516	NA	level4	HMDB	NA	HMDB025	NA	
C13 H11 N	0.000133	0.543894	NA	level4	HMDB	NA	HMDB025	NA	
C18 H30 C	-0.00013	-0.40993	NA	level4	HMDB	NA	HMDB024	NA	
C18 H32 C	-0.00017	-0.5676	NA	level4	HMDB	NA	HMDB024	NA	
C15 H25 N	8.30E-05	0.277293	NA	level4	HMDB	NA	HMDB025	NA	
C11 H20 N	-0.00038	-1.47221	NA	level4	HMDB	NA	HMDB001	Organic a	
C12 H25 N	0.000308	1.54591	NA	level4	HMDB	NA	HMDB025	NA	
C5 H10 O	3.04E-05	0.257728	84.61271	level2	mzCloud	NA	HMDB000	Lipids and	

C19 H23 C	0.000533	1.185124	NA	level4	HMDB	NA	HMDB025	NA
C25 H38 C	18.01082	41474.09	31.41473	level4	Public	NA	NA	NA
C32 H54 C	-0.0017	-2.56938	NA	level4	NA	NA	NA	NA
C23 H27 N	0.001572	3.805352	NA	level4	NA	C16787	NA	Organic ac
C30 H23 B	-0.00069	-1.30729	NA	level4	NA	C18596	NA	NA
C21 H30 C	17.02845	35911.85	1.810716	level4	Public	NA	NA	NA
C6 H7 N	0.000119	1.282938	NA	level4	HMDB	NA	HMDB024	NA
C29 H50 C	0.000205	0.476379	NA	level4	HMDB	NA	HMDB024	NA
C28 H30 C	-0.00082	-1.77794	NA	level4	HMDB	NA	HMDB024	NA
C22 H33 N	-0.00091	-1.96341	NA	level4	HMDB	NA	HMDB025	NA
C24 H24 C	21.98239	49943.27	28.87396	level4	Public	NA	NA	NA
C22 H20 F	0.001483	3.018724	NA	level4	HMDB	NA	HMDB024	NA
C18 H35 N	-0.00015	-0.53983	86.80999	level2	mzCloud	C19670	HMDB000	Lipids and
C15 H14 N	-6.73E-05	-0.24912	2.476104	level4	Public	NA	NA	NA
C26 H28 C	0.000485	0.859121	12.60352	level4	Public	NA	NA	NA
C17 H19 F	0.001076	2.830006	NA	level4	HMDB	NA	HMDB025	NA
C7 H5 N C	-0.00043	-2.35468	NA	level4	HMDB	NA	HMDB002	Organohe
C7 H16 N	-1.85E-05	-0.0719	NA	level4	NA	C21216	NA	NA
C22 H30 F	0.001509	3.196503	NA	level4	HMDB	NA	HMDB024	NA
C11 H12 N	0.001313	4.142263	NA	level4	HMDB	NA	HMDB025	NA
C13 H20	0.000251	1.426584	NA	level4	NA	NA	NA	NA
C17 H15 N	-0.00022	-0.77408	NA	level4	HMDB	NA	HMDB025	NA
C19 H39 N	-7.40E-05	-0.24877	NA	level4	NA	C11285	NA	Organohe
C24 H41 N	-0.00047	-1.2484	NA	level4	HMDB	NA	HMDB025	NA
C47 H70 C	-0.00152	-2.17335	NA	level4	NA	NA	NA	NA
C6 H11 N3	0.000107	0.682772	NA	level4	HMDB	NA	HMDB025	NA
C19 H30 C	0.000965	2.606831	NA	level4	HMDB	NA	HMDB025	NA
C33 H52 C	-0.00253	-3.76075	NA	level4	NA	NA	NA	NA
C16 H24 C	18.01062	54884.97	23.65873	level4	Public	NA	NA	NA
C32 H31 F	0.000445	0.843309	NA	level4	HMDB	NA	HMDB026	NA
C20 H24 C	-7.09E-05	-0.18846	NA	level4	HMDB	NA	HMDB025	NA
C31 H38 C	0.000315	0.451484	NA	level4	NA	NA	NA	NA
C24 H40 C	1.25E-05	0.031891	NA	level4	HMDB	NA	HMDB024	NA
C26 H26 F	-0.00174	-4.30685	NA	level4	HMDB	NA	HMDB001	Benzenoid
C13 H15 N	9.35E-05	0.332728	NA	level4	KEGG	C05595	NA	NA
C39 H68 C	-0.00235	-3.81989	NA	level4	HMDB	NA	HMDB000	Lipids and
C41 H70 N	-0.00244	-3.1761	NA	level4	NA	NA	NA	NA
C10 H19 N	0.00025	1.149295	96.58181	level2	mzCloud	C03017	HMDB000	Lipids and
C6 H15 O8	-6.43E-05	-0.26134	NA	level4	KEGG	C03274	NA	NA
C21 H15 F	0.001063	2.490004	NA	level4	HMDB	NA	HMDB025	NA
C15 H17 N	17.0264	58487.71	16.32367	level4	Public	NA	NA	NA
C23 H26 F	-0.00102	-2.12298	NA	level4	HMDB	NA	HMDB025	NA
C14H13IN	0.001012	1.911509	NA	level4	NA	C18431	NA	NA
C39 H76 N	0.00266	3.625989	NA	level4	NA	NA	NA	NA
C17 H20 C	-0.00143	-4.30726	NA	level4	NA	C18503	NA	NA
C22 H22 N	8.14E-05	0.198379	NA	level4	HMDB	NA	HMDB025	NA
C13 H18 C	-6.89E-05	-0.27116	49.22366	level3	Public	NA	NA	NA
C16 H16 C	-0.00012	-0.36231	NA	level4	HMDB	NA	HMDB001	Organic ac
C17 H26 N	3.47E-05	0.098065	NA	level4	HMDB	NA	HMDB025	NA
C10 H17 N	0.000685	2.290522	NA	level4	NA	C13757	NA	Cyanotoxi
C21 H26 N	-0.00046	-1.2342	NA	level4	HMDB	NA	HMDB025	NA
C29 H34 C	-0.00283	-4.92889	NA	level4	NA	NA	NA	NA
C19 H22 N	0.000194	0.590653	NA	level4	KEGG	C06167	NA	NA
C15 H18 C	-2.70E-05	-0.09165	NA	level4	NA	NA	NA	NA

C18 H35 N	-7.34E-06	-0.02343	NA	level4	NA	NA	NA	NA	
C8 H9 N2	0.001134	4.973864	NA	level4	HMDB	NA	HMDB024	NA	
C8 H10 N2	0.000201	0.940781	NA	level4	HMDB	NA	HMDB024	NA	
C29 H51 C	0.002626	4.22047	NA	level4	NA	NA	NA	NA	
C19 H18 C	0.000368	0.950132	NA	level4	HMDB	NA	HMDB025	NA	
C16 H25 N	-0.00017	-0.58845	NA	level4	HMDB	NA	HMDB025	NA	
C24 H40 N	-0.00109	-2.1093	NA	level4	NA	NA	NA	NA	
C11 H15 N	-0.00012	-0.42405	90.97986	level2	mzCloud	NA	HMDB000	Nucleoside	
C12 H18 C	-18.0103	-92774.3	77.33242	level2	mzCloud	C17002	HMDB003	Organohe	
C5 H10 O3	0.000119	1.008252	NA	level4	HMDB	NA	HMDB025	NA	
C9 H14 N2	0.000126	0.513498	NA	level4	HMDB	NA	HMDB025	NA	
C15 H23 N	-0.00015	-0.51686	43.3021	level3	mzCloud	C06685	NA	Antibiotics	
C17 H26 C	0.000601	1.938793	NA	level4	NA	NA	NA	NA	
C19 H22 C	-8.77E-05	-0.31072	6.815676	level4	Public	NA	NA	NA	
C17 H27 N	-0.00012	-0.32352	NA	level4	NA	C10412	NA	Alkaloids	
C9 H13 F N	0.00115	4.635431	NA	level4	HMDB	NA	HMDB025	NA	
C23 H46 C	-8.58E-05	-0.25371	NA	level4	HMDB	NA	HMDB025	NA	
C25 H26 C	0.000355	0.754876	NA	level4	NA	NA	NA	NA	
C5 H11 N	5.21E-05	0.3916	NA	level4	HMDB	NA	HMDB025	NA	
C21 H18 C	-0.0003	-0.56936	NA	level4	HMDB	NA	HMDB024	Phenylpro	
C18 H31 F	0.001264	4.239687	NA	level4	NA	NA	NA	NA	
C12 H13 N	5.25E-06	0.025832	79.80005	level2	mzCloud	C11284	HMDB000	Organohe	
C37 H46 N	-0.00178	-3.16453	NA	level4	HMDB	NA	HMDB025	NA	
C30 H48 C	-0.00162	-3.30932	NA	level4	HMDB	NA	HMDB025	NA	
C7 H9 N C	0.000272	1.952603	NA	level4	HMDB	NA	HMDB024	NA	
C31 H50 C	0.000629	1.29423	NA	level4	HMDB	NA	HMDB025	NA	
C11 H16 N	-0.00028	-1.04061	NA	level4	HMDB	NA	HMDB025	NA	
C11 H19 N	-0.00014	-0.44116	93.87419	level1	BMDB	C00270	HMDB000	Organic ox	
C14 H10	0.00088	4.941466	NA	level4	HMDB	NA	HMDB025	NA	
C22 H25 N	0.00052	1.252177	NA	level4	NA	C20297	NA	NA	
C12 H22 C	4.82E-05	0.140969	93.08597	level1	BMDB	C05402	HMDB000	Organic ox	
C46 H58 C	-6.58E-05	-0.07804	NA	level4	HMDB	NA	HMDB025	NA	
C14 H12 C	0.000768	2.782236	NA	level4	KEGG	C04485	NA	Organohe	
C24 H30 C	21.98022	62760.35	5.421686	level4	Public	NA	NA	NA	
C17 H26 N	-0.00013	-0.46433	NA	level4	HMDB	NA	HMDB001	Benzenoid	
C14 H16 C	0.001506	3.871775	NA	level4	HMDB	NA	HMDB001	Organohe	
C9 H16 O3	8.50E-05	0.494077	NA	level4	NA	NA	NA	NA	
C18 H18 C	-0.00012	-0.42359	NA	level4	HMDB	NA	HMDB025	NA	
C22 H32 C	9.74E-05	0.282869	55.20652	level3	mzCloud	C07119	HMDB000	Lipids and	
C17 H36 N	-0.00168	-4.59354	NA	level4	NA	NA	NA	NA	
C20 H32 C	37.95411	112881.4	12.95846	level4	Public	NA	NA	NA	
C26 H30 C	-0.00096	-1.75161	24.66353	level4	Public	NA	HMDB003	Phenylpro	
C16 H22 C	-0.00123	-3.16437	NA	level4	HMDB	NA	HMDB025	NA	
C9 H10 N2	0.000167	0.935192	NA	level4	HMDB	NA	HMDB025	NA	

C10 H18 C	0.000178	0.957219	NA	level4	HMDB	NA	HMDB024	NA
C21 H24 F	0.000777	1.507523	NA	level4	HMDB	NA	HMDB026	NA
C11 H14	0.0001	0.685831	NA	level4	HMDB	NA	HMDB024	NA
C16 H12 C	-0.0002	-0.66487	10.99891	level4	Public	NA	NA	NA
C17 H20 N	-0.0001	-0.33395	NA	level4	HMDB	NA	HMDB024	NA
C25 H42 C	-0.00136	-2.62989	NA	level4	HMDB	NA	HMDB025	NA
C22 H41 N	-36.0211	-83519.9	36.3146	level4	Public	NA	NA	NA
C18 H21 C	2.56E-05	0.062601	NA	level4	HMDB	NA	HMDB025	NA
C33 H58 C	0.000234	0.371661	NA	level4	NA	NA	NA	NA
C25 H34 C	-0.00045	-1.01557	NA	level4	HMDB	NA	HMDB025	NA
C19 H28 C	-3.02E-05	-0.10494	NA	level4	HMDB	NA	HMDB026	NA
C11 H19 N	0.000275	1.395976	NA	level4	HMDB	NA	HMDB025	NA
C22 H30 C	4.63E-05	0.142068	NA	level4	HMDB	NA	HMDB006	Lipids and
C41 H60 N	-0.00546	-6.75637	0.623587	level4	Public	NA	NA	NA
C17 H25 N	-0.0009	-2.76996	NA	level4	HMDB	NA	HMDB024	NA
C21 H26 N	-0.00096	-2.49424	NA	level4	HMDB	NA	HMDB001	Organohe
C18 H32 C	-2.01568	-7192.67	87.25368	level1	BMDB2	C01595	HMDB000	Lipids and
C18 H39 N	1.96E-05	0.065147	98.29843	level1	BMDB	C00836	HMDB000	Organic ni
C13 H11 N	-0.00072	-2.04126	NA	level4	HMDB	NA	HMDB001	Organohe
C30 H34 N	-0.00227	-4.47441	NA	level4	HMDB	NA	HMDB024	NA
C32 H31 N	0.002346	3.288907	NA	level4	HMDB	NA	HMDB025	NA
C12 H16 C	0.00025	1.116716	NA	level4	HMDB	NA	HMDB025	NA
C7 H8 O4	8.67E-06	0.046122	88.90791	level1	NA	NA	NA	NA
C9 H10 N2	5.10E-05	0.2865	NA	level4	HMDB	NA	HMDB001	Benzenoid
C13 H17 C	0.001333	4.691265	NA	level4	HMDB	NA	HMDB025	NA
C6 H14 O4	0.000185	1.23272	NA	level4	HMDB	NA	HMDB025	NA
C17 H11 N	-0.00088	-3.16887	NA	level4	HMDB	NA	HMDB025	NA
C27 H26 C	0.000849	1.401002	NA	level4	HMDB	NA	HMDB024	Phenylpro
C22 H23 N	-0.00071	-2.13751	NA	level4	NA	C15458	NA	NA
C21 H26 N	-0.00127	-3.47958	NA	level4	HMDB	NA	HMDB025	NA
C15 H10 C	-0.0003	-1.12441	96.58679	level2	mzCloud	C06563	HMDB000	Phenylpro
C16 H21 N	-0.00157	-3.7758	NA	level4	NA	C21563	NA	NA
C10 H13 N	-0.00135	-4.77803	NA	level4	HMDB	NA	HMDB025	NA
C40 H79 N	0.001601	2.44981	NA	level4	NA	NA	NA	NA
C34 H32 N	0.001284	2.15338	NA	level4	NA	C17906	NA	NA
C9 H17 N	0.000147	0.670644	93.93964	level1	BMDB	C00864	HMDB000	Organic o
C15 H14 C	0.001153	2.805079	NA	level4	HMDB	NA	HMDB024	NA
C26 H40 C	0.000681	1.576258	NA	level4	HMDB	NA	HMDB001	Lipids and
C13 H23 N	-0.0001	-0.34665	NA	level4	HMDB	NA	HMDB000	Lipids and
C35 H35 C	-0.00263	-3.62034	NA	level4	NA	NA	NA	NA
C18 H15 C	-0.00088	-2.83074	NA	level4	NA	C11096	NA	NA
C24 H39 A	-0.00136	-3.24211	NA	level4	NA	NA	NA	NA
C11 H20 C	-4.70E-05	-0.20244	NA	level4	NA	C17232	NA	FA Fatty a
C21 H23 N	0.000156	0.393524	NA	level4	HMDB	NA	HMDB024	NA
C29 H48 C	0.000148	0.345241	NA	level4	HMDB	NA	HMDB024	NA
C22 H30 C	18.01031	50278.03	10.6811	level4	Public	NA	NA	NA
C14 H23 N	0.001399	3.939382	NA	level4	HMDB	NA	HMDB000	Nucleoside
C38 H59 F	-0.00185	-2.92918	NA	level4	HMDB	NA	HMDB025	NA
C18 H22 C	0.001384	4.142502	8.495547	level4	Public	NA	NA	NA
H K2 O4 P	0.00021	1.205349	NA	level4	NA	C13197	NA	NA
C16 H15 F	-0.00308	-7.55872	36.23911	level3	mzCloud	D08516	HMDB001	Organic ac
C24 H23 N	0.001321	3.07887	NA	level4	HMDB	NA	HMDB025	NA
C42 H82 N	0.001904	2.455179	NA	level4	NA	NA	NA	NA

C4 H6 O3	-7.02E-05	-0.52417	NA	level4	HMDB	NA	HMDB025	NA
C8 H13 N	2.17E-05	0.087429	NA	level4	NA	C19036	NA	NA
C8 H6 O	0.000254	2.147637	NA	level4	NA	NA	NA	NA
C22 H23	0.000482	1.17706	NA	level4	HMDB	NA	HMDB025	NA
C17 H20	-0.00101	-2.45026	NA	level4	HMDB	NA	HMDB001	Benzenoid
C31 H52	0.000481	0.984951	NA	level4	HMDB	NA	HMDB024	NA
C30 H48	0.000819	1.637138	NA	level4	NA	C15688	NA	NA
C14 H20	0.00144	4.913447	NA	level4	NA	C18544	NA	NA
C5 H4 N4	0.000269	1.461714	NA	level4	NA	C11821	NA	NA
C34 H69	0.000581	1.046723	NA	level4	NA	NA	NA	NA
C6 H14 O	-0.00046	-1.85865	NA	level4	HMDB	NA	HMDB001	Organic a
C7 H8 N4	0.000386	2.141593	NA	level4	HMDB	NA	HMDB000	Organohe
C5 H11 N	-0.00065	-3.59949	NA	level4	HMDB	NA	HMDB025	NA
C13 H20	-0.00098	-3.25475	NA	level4	HMDB	NA	HMDB025	NA
C17 H10	0.001504	4.613349	NA	level4	NA	NA	NA	NA
C17 H16	-0.00014	-0.48206	NA	level4	HMDB	NA	HMDB024	NA
C12 H21	0.00076	2.375406	NA	level4	HMDB	NA	HMDB025	NA
H I	8.96E-05	0.700733	NA	level4	KEGG	C05590	NA	Homogen
C11 H9 N	-0.00019	-0.81312	NA	level4	HMDB	NA	HMDB024	NA
C10 H14	-0.00071	-3.88099	NA	level4	HMDB	NA	HMDB025	NA
C10 H8 O	0.000231	1.311609	NA	level4	HMDB	NA	HMDB025	NA
C12 H17	0.000338	1.768818	72.85824	level2	Public	C10935	HMDB025	NA
C19 H32	-0.00135	-3.7798	NA	level4	NA	C14960	NA	NA
C22 H25	-0.00022	-0.65164	NA	level4	HMDB	NA	HMDB024	NA
C11 H19	-7.57E-05	-0.25821	NA	level4	KEGG	C02713	NA	Carbohyd
C21 H25	-6.84E-05	-0.18434	NA	level4	HMDB	NA	HMDB025	NA
C8 H16 N	0.000764	3.031756	NA	level4	NA	C17242	NA	NA
C6 H11 N	0.000209	1.67267	49.4597	level3	BMDB	NA	HMDB000	Organic ni
C32 H36	37.95369	71846.28	9.050785	level4	Public	NA	NA	NA
C8 H14 O	0.000194	1.023009	NA	level4	HMDB	NA	HMDB000	Organic a
C14 H27	-0.00145	-4.49179	NA	level4	HMDB	NA	HMDB024	NA
C17 H15	0.000743	2.534068	NA	level4	HMDB	NA	HMDB026	NA
C26 H42	0.001166	2.199496	28.87079	level4	Public	NA	NA	NA
C19 H15	-2.76E-05	-0.09919	NA	level4	HMDB	NA	HMDB024	NA
C26 H29	0.001986	4.161894	NA	level4	HMDB	NA	HMDB001	Benzenoid
C18 H22	0.001192	3.933336	NA	level4	HMDB	NA	HMDB024	NA
C15 H14	17.02659	58696.39	13.62437	level4	Public	NA	NA	NA
C15 H15	-4.34E-05	-0.15015	NA	level4	NA	C10631	NA	Alkaloids
C23 H20	0.001346	3.157574	NA	level4	HMDB	NA	HMDB025	NA
C21 H22	-0.00132	-2.89096	NA	level4	HMDB	NA	HMDB006	Organic o
C15 H23	-0.00013	-0.39561	NA	level4	HMDB	NA	HMDB025	NA
C15 H15	-0.0004	-1.15414	NA	level4	HMDB	NA	HMDB025	NA
C7 H14 O	-0.00014	-0.6878	NA	level4	HMDB	NA	HMDB025	NA
C9 H18 N	-5.53E-05	-0.1855	NA	level4	HMDB	NA	HMDB024	Organic a
C11 H21	6.90E-05	0.298572	9.448587	level4	Public	NA	NA	NA
C22 H29	-0.0019	-4.47298	NA	level4	HMDB	NA	HMDB024	NA
C18 H20	-0.00142	-4.87648	NA	level4	HMDB	NA	HMDB025	NA
C24 H21	-0.00082	-1.96307	NA	level4	NA	C18680	NA	NA
C13 H12	-6.72E-05	-0.23022	NA	level4	HMDB	NA	HMDB024	NA
C17 H24	37.95664	137438.1	6.668267	level4	Public	NA	NA	NA
C10 H6 Cl	0.000696	3.119882	NA	level4	HMDB	NA	HMDB024	NA
C17 H35	-0.00154	-4.03161	NA	level4	NA	NA	NA	NA
C20 H20	21.9803	67811.94	6.534062	level4	Public	NA	NA	NA
C27 H26	5.56E-05	0.096828	NA	level4	NA	NA	NA	NA

C23 H22 C	6.67E-05	0.13619	NA	level4	HMDB	NA	HMDB004	Phenylpro
C14 H22 C	6.11E-05	0.256654	NA	level4	NA	NA	NA	NA
C7 H8 O3	0.000257	1.837399	44.96034	level3	mzCloud	NA	NA	NA
C9 H8 N2	2.09E-05	0.118539	NA	level4	HMDB	NA	HMDB024	NA
C19 H24 C	-6.39E-05	-0.23835	NA	level4	NA	NA	NA	NA
C15 H16 F	-0.00113	-3.70832	NA	level4	HMDB	NA	HMDB025	NA
C9 H10 O2	0.000175	1.16531	NA	level4	HMDB	NA	HMDB000	Phenylpro
C10 H7 N	6.00E-05	0.317381	84.66333	level1	BMDB2	C01717	HMDB000	Organohe
C23 H24 C	-8.31E-06	-0.01746	NA	level4	NA	NA	NA	NA
C21 H31 N	5.13E-06	0.011726	NA	level4	HMDB	NA	HMDB025	NA
C26 H25 N	-0.00085	-2.04935	NA	level4	HMDB	NA	HMDB024	NA
C23 H22 C	21.98068	53593.57	15.95813	level4	Public	NA	NA	NA
C5 H10 N2	0.000113	0.698705	NA	level4	HMDB	NA	HMDB025	NA
C21 H25 N	4.04E-05	0.098139	NA	level4	HMDB	NA	HMDB025	NA
C18 H18 C	0.000375	1.034613	NA	level4	NA	NA	NA	NA
C14 H23 N	0.00026	1.176332	NA	level4	HMDB	NA	HMDB024	NA
C42 H36 C	0.002617	4.113611	NA	level4	HMDB	NA	HMDB024	NA
C24 H25 F	0.001907	4.293931	NA	level4	HMDB	NA	HMDB024	NA
C46 H80 N	0.000185	0.224797	NA	level4	NA	NA	NA	NA
C28 H56 N	0.001062	1.933163	NA	level4	HMDB	NA	HMDB001	Lipids and
C13 H16 N	5.21E-06	0.016265	NA	level4	HMDB	NA	HMDB024	NA
C9 H19 O1	-0.0001	-0.30857	NA	level4	KEGG	C04703	NA	NA
C26 H36 C	0.001536	2.761187	NA	level4	NA	C11667	NA	PR Prenol
C14 H22 N	4.26E-05	0.170289	96.87478	level2	mzCloud	NA	NA	NA

C13 H18 N	-5.68E-05	-0.20147	NA	level4	HMDB	NA	HMDB006	Organic ac
C21 H24 C	21.98143	52319.05	5.557404	level4	Public	NA	NA	NA
C16 H32 C	0.000476	1.566146	15.06709	level4	Public	NA	NA	NA
C21 H27 N	-0.00056	-1.44477	NA	level4	NA	NA	NA	NA
C17 H14 C	-0.00074	-2.973	NA	level4	HMDB	NA	HMDB024	NA
C18 H30 C	-2.51E-05	-0.09039	NA	level4	HMDB	NA	HMDB025	NA
C21 H18 C	-0.00067	-1.8339	NA	level4	HMDB	NA	HMDB024	NA
C17 H20 C	0.001925	3.864173	NA	level4	HMDB	NA	HMDB025	NA
C17 H25 N	-0.0015	-4.52166	NA	level4	HMDB	NA	HMDB025	NA
C8 H12 N2	0.000449	2.246461	NA	level4	HMDB	NA	HMDB025	NA
C20 H24 C	21.98024	70410.56	23.9926	level4	Public	NA	NA	NA
C5 H12 N2	0.000622	3.454249	NA	level4	HMDB	NA	HMDB025	NA
C10 H13 N	-0.00022	-0.79225	95.65605	level1	BMDB	C00387	HMDB000	Nucleoside
C10 H10 C	-0.0005	-1.94425	NA	level4	HMDB	NA	HMDB024	NA
C11 H15 N	0.00027	0.910007	NA	level4	HMDB	NA	HMDB025	NA
C23 H28 N	5.76E-05	0.129614	NA	level4	HMDB	NA	HMDB025	NA
C13 H10 I	0.000935	2.894026	NA	level4	NA	C18907	NA	NA
C32 H42 C	0.001203	2.170467	NA	level4	KEGG	C09916	NA	NA
C19 H32 C	-18.0103	-50559.6	33.00909	level4	Public	NA	NA	NA
C26 H34 C	0.00208	4.109007	21.08109	level4	Public	NA	NA	NA
C23 H43 C	0.002302	4.657489	NA	level4	NA	NA	NA	NA
C25 H44 N	-0.00111	-2.14421	NA	level4	NA	NA	NA	NA
C9 H10 F I	1.51E-05	0.070154	NA	level4	HMDB	NA	HMDB024	NA
C20 H23 N	0.000311	0.870636	NA	level4	NA	C14917	NA	NA
C16 H12 C	9.46E-05	0.299432	6.145557	level4	Public	NA	NA	NA
C23 H22 F	-0.00151	-3.80934	NA	level4	HMDB	NA	HMDB025	NA
C21 H24 C	21.98381	61724.13	25.42293	level4	Public	NA	NA	NA
C10 H13 N	0.000147	0.752077	41.419	level3	mzCloud	NA	HMDB004	Benzenoid
C22 H31 N	0.001788	4.286608	NA	level4	HMDB	NA	HMDB001	Organic ac

C15 H27 N	-7.76E-05	-0.24473	NA	level4	HMDB	NA	HMDB024	NA
C18 H18 F	-0.00166	-4.35541	NA	level4	HMDB	NA	HMDB025	NA
C12 H21 N	0.000141	0.579948	12.80576	level4	Public	NA	NA	NA
C16 H20 C	21.9814	71339.01	46.11707	level4	Public	NA	NA	NA
C15 H22 C	7.44E-05	0.297536	51.36721	level4	Public	NA	NA	NA
C24 H42 N	-0.00082	-1.58008	NA	level4	NA	NA	NA	NA
C16 H21 N	-4.31E-05	-0.12711	NA	level4	HMDB	NA	HMDB025	NA
C28 H29 C	0.000195	0.399543	NA	level4	HMDB	NA	HMDB025	NA
C33 H35 F	0.000302	0.525355	NA	level4	HMDB	NA	HMDB006	Organohe
C27 H44 C	0.001329	2.516151	NA	level4	NA	NA	NA	NA
C18 H33 N	-5.87E-05	-0.18848	NA	level4	NA	NA	NA	NA
C28 H40 N	0.00121	2.606626	NA	level4	HMDB	NA	HMDB025	NA
C22 H30 N	-0.00113	-1.8919	NA	level4	HMDB	NA	HMDB024	NA
C36 H36 N	-0.00154	-2.67687	NA	level4	KEGG	C09667	NA	Alkaloids
C6 H5 N3	0.000155	1.300026	72.24039	level2	Public	NA	HMDB024	NA
C23 H24 C	0.000347	0.704827	NA	level4	NA	C10303	NA	Polyketide
C33 H33 F	0.000105	0.193755	NA	level4	HMDB	NA	HMDB024	NA
C23 H41 N	-0.00108	-2.17227	NA	level4	HMDB	NA	HMDB026	NA
C27 H48 N	-0.00204	-3.39444	2.040996	level4	Public	NA	NA	NA
C27 H45 C	0.002113	3.771983	NA	level4	NA	NA	NA	NA
C25 H43 C	0.001973	4.057817	NA	level4	NA	NA	NA	NA
C20 H34 C	21.98043	64983.61	14.45887	level4	Public	NA	NA	NA
C17 H22 C	-0.00242	-6.83946	1.64527	level4	Public	NA	NA	NA
C28 H52 N	-0.00247	-4.04845	NA	level4	NA	NA	NA	NA
C6 H6 N4	0.00038	2.289019	83.14	level2	mzCloud	NA	NA	NA
C6 H3 Cl3	-0.00019	-0.7718	NA	level4	HMDB	NA	HMDB025	NA
C24 H38 C	17.02672	43627.27	89.67639	level1	BMDB2	NA	NA	NA
C27 H17 N	-0.00132	-2.66459	NA	level4	HMDB	NA	HMDB026	NA
C19 H35 N	4.18E-07	0.001225	NA	level4	HMDB	NA	HMDB025	NA
C37 H38 N	-0.00298	-4.91636	NA	level4	HMDB	NA	HMDB024	NA
C24 H28 N	-0.00116	-2.85162	NA	level4	HMDB	NA	HMDB024	NA
C16 H20 F	0.001615	4.790207	NA	level4	HMDB	NA	HMDB001	Organohe
C12 H15 N	1.92E-05	0.077094	NA	level4	HMDB	NA	HMDB025	NA
C14 H24 N	0.000843	2.218243	NA	level4	HMDB	NA	HMDB025	NA
C21 H39 N	-6.92E-05	-0.19583	NA	level4	HMDB	NA	HMDB025	NA
C40 H54 C	0.000528	0.906664	NA	level4	NA	NA	NA	NA
C11 H14 N	-5.11E-05	-0.24775	NA	level4	HMDB	NA	HMDB024	NA
C27 H48 C	0.000814	1.576418	NA	level4	NA	NA	NA	NA
C15 H28 C	-5.26E-05	-0.18255	16.15068	level4	Public	NA	NA	NA
C10 H10 C	0.000347	1.948444	NA	level4	HMDB	NA	HMDB024	NA
C20 H21 N	0.000653	1.838937	NA	level4	HMDB	NA	HMDB025	NA
C22 H29 C	0.000961	2.353594	NA	level4	HMDB	NA	HMDB001	Lipids and
C11 H13 C	0.001607	6.582662	19.51604	level4	Public	NA	NA	NA
C12 H20 N	0.000243	0.730595	NA	level4	HMDB	NA	HMDB001	Organic ac
C5 H8 O2	4.13E-05	0.412297	NA	level4	HMDB	NA	HMDB025	NA
C19 H23 N	-0.00045	-1.24913	NA	level4	KEGG	C08514	NA	Alkaloids
C32 H54 C	0.000798	1.540018	NA	level4	NA	NA	NA	NA
C19 H22 C	-18.0109	-52033.2	17.20169	level4	Public	NA	NA	NA
C21 H41 C	-8.48E-05	-0.20186	NA	level4	NA	NA	NA	NA
C22 H28 C	17.0263	42125.17	5.169915	level4	Public	NA	NA	NA
C6 H12 F C	0.00119	4.542386	NA	level4	HMDB	NA	HMDB024	NA
C22 H25 N	-0.00131	-2.88593	NA	level4	HMDB	NA	HMDB024	NA
C9 H10 Cl	7.85E-05	0.365152	NA	level4	HMDB	NA	HMDB000	Organic ac
C20 H40 N	-0.00186	-4.41756	NA	level4	NA	NA	NA	NA
C15 H21 N	-4.54E-05	-0.15593	NA	level4	KEGG	C09176	NA	Alkaloids

C24 H30 C	17.02686	34454.86	1.178905	level4	Public	NA	NA	NA	
C34 H52 C	0.000378	0.661108	NA	level4	HMDB	NA	HMDB001	Lipids and	
C28 H35 F	0.001745	3.137206	NA	level4	HMDB	NA	HMDB025	NA	
C40 H54 C	0.000985	1.788736	NA	level4	HMDB	NA	HMDB024	NA	
C37 H68 C	0.000167	0.274335	NA	level4	NA	NA	NA	NA	
C4 H4 N2	-0.00055	-3.79548	NA	level4	HMDB	NA	HMDB024	NA	
C23 H22 C	17.02672	41514.76	22.11097	level4	Public	NA	NA	NA	
C23 H32 C	0.000116	0.326051	NA	level4	HMDB	NA	HMDB006	Lipids and	
C20 H32 N	-0.00031	-0.88701	NA	level4	HMDB	NA	HMDB024	NA	
C3 H5 O7	-0.00033	-1.7886	NA	level4	HMDB	NA	HMDB025	NA	
C9 H16 N2	-6.65E-05	-0.25169	NA	level4	HMDB	NA	HMDB025	NA	
C20 H16 C	-0.00062	-1.76719	NA	level4	NA	C19562	NA	Benzenoid	
C13 H16 C	0.000146	0.618283	NA	level4	HMDB	NA	HMDB006	Phenylpro	
C20 H20 C	2.81E-05	0.072386	60.72443	level2	Public	NA	HMDB003	Phenylpro	
C12 H16 N	8.42E-05	0.356423	NA	level4	HMDB	NA	HMDB001	Organohe	
C13 H21 N	-0.00014	-0.52858	NA	level4	HMDB	NA	HMDB024	NA	
C32 H62 N	0.000393	0.603175	NA	level4	NA	NA	NA	NA	
C8 H10 F N	-0.00086	-4.5738	NA	level4	HMDB	NA	HMDB024	NA	
C9 H13 N	0.00022	1.317797	8.860614	level4	Public	C04548	HMDB000	Benzenoid	
C10 H12 C	0.000909	3.294917	NA	level4	HMDB	NA	HMDB004	Organic ac	
C28 H45 F	-0.00087	-1.94666	NA	level4	NA	NA	NA	NA	
C5 H6 O	2.06E-05	0.251512	NA	level4	HMDB	NA	HMDB024	NA	
C23 H30 N	6.88E-05	0.154087	NA	level4	HMDB	NA	HMDB025	NA	
C24 H50 N	0.000627	1.266294	NA	level4	HMDB	NA	HMDB024	NA	
C9 H14 O4	17.02699	91499.07	29.15445	level4	Public	NA	NA	NA	
C14 H15 N	-0.00021	-0.60772	NA	level4	HMDB	NA	HMDB024	NA	
C2 Cl F3	-0.00023	-1.96422	NA	level4	HMDB	NA	HMDB025	NA	
C15 H15 C	0.000817	2.815937	NA	level4	HMDB	NA	HMDB006	Organohe	
C7 H14 N2	0.000846	4.450284	NA	level4	HMDB	NA	HMDB024	NA	
C19 H14 C	-0.00098	-3.39515	NA	level4	HMDB	NA	HMDB024	NA	
C19 H29 I	-0.00171	-4.10682	NA	level4	HMDB	NA	HMDB001	Benzenoid	
C23 H28 N	-0.0013	-3.31267	NA	level4	HMDB	NA	HMDB025	NA	
C12 H9 N5	-4.32E-06	-0.01808	NA	level4	HMDB	NA	HMDB024	NA	
C21 H25 N	-4.71E-05	-0.1325	41.6048	level3	mzCloud	C09446	NA	Alkaloids	
C27 H27 N	-0.00028	-0.60197	NA	level4	HMDB	NA	HMDB024	NA	
C13 H10 C	0.000103	0.480257	NA	level4	HMDB	NA	HMDB024	NA	
C20 H16 N	-0.00053	-1.52064	50.25266	level3	mzCloud	C01897	NA	Alkaloids	
C9 H13 N	1.54E-05	0.077595	NA	level4	NA	NA	NA	NA	
C17 H20 N	-0.0001	-0.34602	NA	level4	HMDB	NA	HMDB001	Organohe	
C9 H14 O4	2.28E-05	0.122339	69.1143	level2	Public	NA	NA	NA	
C18 H32 C	37.95545	128126.1	5.05009	level4	Public	NA	NA	NA	
C28 H48 C	-0.00212	-4.56613	NA	level4	HMDB	NA	HMDB000	Lipids and	
C14 H17 N	-1.65E-05	-0.04298	NA	level4	HMDB	NA	HMDB000	Nucleoside	
C8 H13 N3	0.0004	1.85804	NA	level4	HMDB	NA	HMDB024	NA	
C21 H30 C	-0.00164	-3.57584	NA	level4	NA	C10286	NA	Alkaloids	
C15 H22 C	-2.44E-05	-0.09175	NA	level4	HMDB	NA	HMDB006	Organohe	
C7 H12 O2	7.75E-05	0.60528	NA	level4	HMDB	NA	HMDB024	NA	
C20 H16 C	-6.65E-05	-0.18892	NA	level4	HMDB	NA	HMDB025	NA	
C19 H14 C	0.000344	0.972216	NA	level4	NA	NA	NA	NA	
C11 H14 N	8.40E-05	0.441864	NA	level4	HMDB	NA	HMDB025	NA	
C16 H12 C	-0.0039	-16.515	31.85135	level3	mzCloud	NA	NA	NA	
C41 H53 N	-0.00026	-0.29299	NA	level4	NA	C15927	NA	NA	
C13 H20 N	0.000112	0.473859	NA	level4	HMDB	NA	HMDB001	Benzenoid	
C19 H28 C	-0.00031	-0.69977	NA	level4	HMDB	NA	HMDB024	NA	
C19 H21 N	-3.67E-05	-0.11232	22.88317	level4	Public	C11781	HMDB004	Alkaloids a	
C18 H27 N	-0.00022	-0.73395	NA	level4	HMDB	NA	HMDB024	NA	
C30 H34 C	0.001793	3.058999	2.805245	level4	Public	NA	NA	NA	

C44 H74 N	-0.00011	-0.14365	NA	level4	NA	NA	NA	NA
C18 H28 N	-0.00168	-2.6719	NA	level4	NA	C20672	NA	NA
C18 H14 C	-0.00038	-1.15662	NA	level4	HMDB	NA	HMDB025	NA
C16 H16 C	-1.40E-05	-0.03839	NA	level4	HMDB	NA	HMDB001	Benzenoid
C26 H41 N	-0.00017	-0.43765	NA	level4	NA	NA	NA	NA
C20 H23 F	-0.00183	-4.82387	NA	level4	HMDB	NA	HMDB025	NA
C29 H33 F	-0.00039	-0.78938	NA	level4	HMDB	NA	HMDB024	NA
C25 H26 C	-0.00053	-1.24951	NA	level4	HMDB	NA	HMDB025	NA
C18 H23 N	-0.00059	-1.46777	NA	level4	NA	C21647	NA	NA
C9 H9 N C	0.000212	1.185152	78.39127	level2	mzCloud	C19569	HMDB000	Organic ac
C8 H8 O3	0.000173	1.137518	62.93995	level2	mzCloud	D01400	HMDB003	Benzenoid
C10 H13 N	0.000188	1.049608	54.79174	level3	mzCloud	NA	HMDB000	Organohe
C30 H42 C	-0.00034	-0.61337	33.48586	level4	Public	NA	NA	NA
C22 H26 C	-0.00067	-1.80741	NA	level4	HMDB	NA	HMDB024	NA
C37 H67 N	-0.00044	-0.59889	93.80408	level2	mzCloud	C01912	HMDB001	Organohe
C13 H24 C	0.000979	2.879095	NA	level4	KEGG	C04698	NA	NA
C25 H26 C	-0.00193	-4.02573	NA	level4	HMDB	NA	HMDB024	NA
C38 H40 N	0.001772	2.589511	3.753144	level4	Public	NA	NA	NA
C12 H9 Cl	0.000815	3.23483	NA	level4	KEGG	C06587	NA	NA
C17 H22 N	0.001668	4.366181	NA	level4	HMDB	NA	HMDB024	NA
C19 H24 C	0.001188	3.412552	14.38634	level4	Public	NA	NA	NA
C44 H60 N	0.003717	4.620041	NA	level4	HMDB	NA	HMDB025	NA
C22 H29 F	-0.00132	-3.65221	NA	level4	NA	C15148	NA	NA
C28 H36 C	0.000382	0.740356	NA	level4	NA	NA	NA	NA
C13 H16 N	-0.00015	-0.59453	61.56253	level2	mzCloud	C05643	HMDB000	Organohe
C19 H17 N	0.000252	0.488772	NA	level4	HMDB	NA	HMDB024	NA
C6 H15 N3	0.000397	2.461914	NA	level4	HMDB	NA	HMDB025	NA
C17 H12 C	0.000309	0.940578	NA	level4	NA	C16756	NA	Fungal tox
C17 H16 C	-0.00142	-4.01257	NA	level4	HMDB	NA	HMDB025	NA
C24 H25 N	-0.00034	-0.95952	NA	level4	HMDB	NA	HMDB006	Phenylpro
C28 H45 C	0.001785	3.208513	NA	level4	NA	NA	NA	NA
C24 H51 C	0.000654	1.31277	NA	level4	NA	NA	NA	NA
C7 H14 N2	0.000356	1.725771	NA	level4	HMDB	NA	HMDB025	NA
C10 H11 N	0.001221	4.471968	NA	level4	HMDB	NA	HMDB001	Organic ac
C23 H30 N	-0.00163	-4.43993	NA	level4	HMDB	NA	HMDB001	Organohe
C15 H21 N	-0.00027	-0.75453	NA	level4	HMDB	NA	HMDB024	NA
C14 H15 C	-0.00139	-4.38426	NA	level4	HMDB	NA	HMDB024	NA
C4 H2 F6	0.000448	2.731943	NA	level4	HMDB	NA	HMDB024	NA
C12 H15 N	-0.00129	-4.66141	NA	level4	HMDB	NA	HMDB025	NA
C15 H19 N	-0.00135	-4.73534	NA	level4	HMDB	NA	HMDB006	Organohe
C24 H34 C	-6.17E-06	-0.01598	23.54137	level4	Public	NA	NA	NA
C17 H12 C	-0.00019	-0.53338	NA	level4	HMDB	NA	HMDB006	Phenylpro
C13 H20 N	-0.0001	-0.40104	NA	level4	HMDB	NA	HMDB001	Organohe
C29 H27 N	-0.00045	-0.96603	NA	level4	NA	C13771	NA	NA
C17 H18 N	-0.00138	-4.50589	NA	level4	HMDB	NA	HMDB024	NA
C32 H38 C	0.000933	1.755663	NA	level4	HMDB	NA	HMDB024	NA
C16 H28 C	21.98171	66173.38	20.67066	level4	Public	NA	NA	NA
C15 H13 N	1.93E-05	0.086364	NA	level4	HMDB	NA	HMDB025	NA
C18 H18 C	-3.05E-05	-0.09252	NA	level4	HMDB	NA	HMDB024	NA
C20 H20 C	-0.00027	-0.67262	NA	level4	HMDB	NA	HMDB004	Phenylpro
C14 H14 N	-0.00069	-1.98356	NA	level4	HMDB	NA	HMDB025	NA
C13 H18 C	0.000548	1.76707	NA	level4	HMDB	NA	HMDB025	NA

C9 H8 O3	0.000201	1.225475	NA	level4	HMDB	NA	HMDB025	NA
C17 H18 F	7.12E-05	0.204968	NA	level4	HMDB	NA	HMDB025	NA
C14 H26 C	-7.51E-05	-0.31007	NA	level4	NA	NA	NA	NA
C11 H13 N	0.000106	0.513036	45.06714	level3	Public	C20126	HMDB025	NA
C13 H14 C	-0.00012	-0.45573	30.43517	level4	Public	NA	NA	NA
C19 H32	-0.00063	-2.4271	NA	level4	HMDB	NA	HMDB024	NA
C37 H42 N	0.002829	4.634737	NA	level4	HMDB	NA	HMDB025	NA
C10 H14 F	-0.00028	-1.10439	NA	level4	HMDB	NA	HMDB024	NA
C19 H14 N	-0.00137	-4.60319	NA	level4	HMDB	NA	HMDB025	NA
C19 H28 C	37.95412	103084.8	7.953869	level4	Public	NA	NA	NA
C16 H33 N	8.98E-05	0.351637	NA	level4	NA	NA	NA	NA
C17 H20 C	37.9542	118560.2	10.99556	level4	Public	C20380	HMDB001	Organohe
C13 H14 C	0.000549	1.719811	NA	level4	HMDB	NA	HMDB024	NA
C22 H30 C	37.9546	89898.47	10.96858	level4	Public	NA	NA	NA
C5 H8 O4	0.000115	0.872895	42.56593	level3	BMDB	C00489	HMDB000	Organic ac
C5 H6 O3	0.000148	1.30222	NA	level4	HMDB	NA	HMDB025	NA
C19 H14 C	-0.00025	-0.60271	NA	level4	HMDB	NA	HMDB025	NA
C12 H22 N	0.000732	2.114427	NA	level4	HMDB	NA	HMDB024	NA
C10 H10 N	0.000291	1.31235	NA	level4	HMDB	NA	HMDB025	NA
C32 H30 C	-0.00024	-0.40001	NA	level4	HMDB	NA	HMDB025	NA
C7 H14 O(	21.9821	113263.7	23.05567	level4	Public	NA	HMDB024	NA
C42 H78 N	-0.00059	-0.77054	NA	level4	NA	NA	NA	NA
C23 H40 C	21.98199	55469.8	62.67899	level2	Public	NA	NA	NA
C20 H24 N	-0.0011	-2.87219	NA	level4	HMDB	NA	HMDB025	NA
C11 H15 N	0.000148	0.622887	NA	level4	HMDB	NA	HMDB025	NA
C9 H14 O	5.61E-05	0.406105	NA	level4	HMDB	NA	HMDB024	NA
C18 H28 C	-3.32E-05	-0.12017	NA	level4	HMDB	NA	HMDB000	Lipids and
C11 H18 C	3.50E-05	0.210908	NA	level4	NA	NA	NA	NA
C14 H20 N	0.000457	1.840647	NA	level4	HMDB	NA	HMDB001	Organohe
C25 H25 N	0.000545	1.176089	NA	level4	HMDB	NA	HMDB025	NA
C7 H8 O	6.23E-05	0.576997	84.54117	level2	mzCloud	C01468	HMDB000	Benzenoid
C28 H34 C	-0.00179	-2.85183	NA	level4	NA	NA	NA	NA
C37 H71 N	0.000678	1.143125	NA	level4	NA	NA	NA	NA
C19 H26 N	-6.86E-05	-0.20782	NA	level4	HMDB	NA	HMDB026	NA
C20 H28 C	-0.00046	-1.44979	7.297997	level4	Public	NA	NA	NA
C17 H19 N	17.02651	53689.99	7.271412	level4	Public	C10166	HMDB003	Phenylpro
C46 H84 N	-7.41E-05	-0.09341	NA	level4	HMDB	NA	HMDB001	Lipids and
C25 H32 C	-0.00141	-2.94676	NA	level4	NA	C20134	NA	NA
C17 H20 C	0.000183	0.495656	NA	level4	HMDB	NA	HMDB025	NA
C25 H27 C	8.09E-05	0.207319	NA	level4	HMDB	NA	HMDB001	Benzenoid
C28 H30 C	-0.00103	-1.89889	NA	level4	NA	NA	NA	NA
C27 H44 C	-0.00013	-0.26408	12.13144	level4	Public	NA	NA	NA
C22 H26 N	-5.96E-05	-0.14379	NA	level4	HMDB	NA	HMDB001	Organohe
C17 H22 C	15.99614	49655.6	5.168108	level4	Public	NA	NA	NA
C17 H18 C	35.03714	115972.7	6.622227	level4	Public	NA	NA	NA
C31 H36 C	0.001953	3.253319	NA	level4	NA	C12408	NA	NA
C24 H28 N	-0.00031	-0.734	NA	level4	HMDB	NA	HMDB001	Organic ac
C18 H23 I	-0.00116	-2.91249	NA	level4	HMDB	NA	HMDB025	NA
C32 H40 B	0.001239	1.896652	NA	level4	HMDB	NA	HMDB001	Organic ac
C6 H7 N C	0.000189	1.094212	38.33741	level3	BMDB	C06333	NA	NA
C22 H22 C	-0.00022	-0.48497	NA	level4	HMDB	NA	HMDB024	NA

C14 H12 C	21.98239	90064.62	3.508434	level4	Public	NA	NA	NA
C11 H10 C	-0.00109	-3.99142	NA	level4	NA	C21868	NA	NA
C20 H25 N	-0.00103	-2.74868	NA	level4	HMDB	NA	HMDB024	NA
C8 H8 O5	-36.0209	-195726	43.95406	level3	Public	C05580	HMDB000	Benzenoid
C8 H10 O2	0.000125	0.908313	65.69455	level2	mzCloud	C06044	HMDB000	Benzenoid
C41 H80 N	0.003608	4.640582	NA	level4	NA	NA	NA	NA
C2 H7 O4	0.000184	1.458457	NA	level4	HMDB	NA	HMDB006	Organic ac
C21 H22 C	-0.00028	-0.74881	NA	level4	HMDB	NA	HMDB024	NA
C27 H46 C	-8.92E-05	-0.18485	NA	level4	HMDB	NA	HMDB025	NA
C19 H18 C	21.98026	70876.52	10.37044	level4	Public	NA	NA	NA
C4 H8 O S	0.000171	1.642908	NA	level4	HMDB	NA	HMDB025	NA
C21 H32 C	17.02788	37001.48	1.107095	level4	Public	NA	NA	NA
C23 H29 N	0.00125	3.261698	NA	level4	HMDB	NA	HMDB025	NA
C22 H26 C	-0.00211	-4.52339	NA	level4	HMDB	NA	HMDB025	NA
C18 H22 C	-0.00127	-2.9555	NA	level4	HMDB	NA	HMDB024	NA
C32 H50 C	21.98072	47130.39	37.52211	level4	Public	NA	NA	NA
C26 H41 N	0.0002	0.482112	NA	level4	NA	NA	NA	NA
C7 H11 O8	0.001118	4.400002	NA	level4	HMDB	NA	HMDB025	NA
C24 H24 C	-0.00023	-0.58351	NA	level4	NA	C11622	NA	NA
C33 H54 C	0.001069	1.585357	NA	level4	NA	NA	NA	NA
C24 H22 N	-0.00139	-4.09627	NA	level4	KEGG	C00915	NA	NA
C21 H46 N	-5.51E-05	-0.12094	NA	level4	NA	NA	NA	NA
C28 H35 N	0.001957	4.065844	NA	level4	HMDB	NA	HMDB025	NA
C9 H19 N	-5.64E-05	-0.23791	NA	level4	HMDB	NA	HMDB024	NA
C24 H29 N	9.39E-05	0.221905	NA	level4	NA	C17728	NA	NA
C28 H37 F	-0.00138	-2.73666	NA	level4	HMDB	NA	HMDB024	NA
C42 H78 N	-0.00022	-0.27781	NA	level4	HMDB	NA	HMDB001	Lipids and
C25 H28 N	0.000151	0.328065	NA	level4	HMDB	NA	HMDB024	NA
C24 H38 C	-0.00012	-0.27238	NA	level4	NA	NA	NA	NA
C15 H11 F	-0.00144	-4.67219	NA	level4	HMDB	NA	HMDB025	NA
C21 H28 C	-0.00019	-0.60858	36.2211	level4	Public	NA	HMDB024	NA
C32 H50 C	0.001669	2.968529	NA	level4	HMDB	NA	HMDB024	NA
C27 H33 N	0.00062	1.212912	NA	level4	NA	C20045	NA	Fungal tox
C19 H20 C	7.79E-05	0.24964	27.35186	level4	Public	NA	NA	NA
C10 H16 C	-0.00135	-4.83602	NA	level4	HMDB	NA	HMDB025	NA
C20 H24 N	0.001889	4.322337	NA	level4	HMDB	NA	HMDB024	NA
C17 H32 C	-0.00022	-0.80807	77.88285	level2	mzCloud	NA	NA	NA
C12 H22 N	-0.0002	-0.62909	NA	level4	HMDB	NA	HMDB025	NA
C34 H50 C	0.000393	0.670167	NA	level4	HMDB	NA	HMDB024	NA
C13 H18 N	-8.25E-05	-0.30988	NA	level4	NA	C20926	NA	NA
C18 H14 N	-0.00102	-3.02244	NA	level4	HMDB	NA	HMDB024	NA
C15 H20 C	-9.02E-05	-0.32217	24.29751	level4	Public	NA	NA	NA
C9 H14 O5	-1.88E-05	-0.09297	NA	level4	KEGG	C04067	NA	Organic ac
C20 H24 C	0.000541	1.229189	NA	level4	KEGG	C07603	NA	Terpenoid
C24 H16 F	8.91E-05	0.17598	NA	level4	HMDB	NA	HMDB025	NA
C35 H58 C	0.001242	1.898248	NA	level4	HMDB	NA	HMDB024	NA
C12 H20 C	0.000233	1.186264	NA	level4	NA	NA	NA	NA
C9 H12 N2	0.000312	1.591717	NA	level4	HMDB	NA	HMDB024	NA
C10 H13 N	0.004507	13.57296	35.61505	level3	mzCloud	C06196	HMDB000	Nucleoside
C17 H17 N	17.02651	60138.66	8.543379	level4	Public	NA	NA	NA
C17 H21 N	17.02649	59294.31	15.13946	level4	Public	NA	NA	NA

C11 H18 C	17.02661	73991.62	8.1325	level4	Public	NA	NA	NA	
C17 H32 N	-0.0004	-0.96207	NA	level4	HMDB	NA	HMDB025	NA	
C15 H10 C	-0.00033	-1.28631	37.20998	level3	mzCloud	C10028	HMDB003	Phenylpro	
C19 H35 N	-0.0003	-0.81587	NA	level4	HMDB	NA	HMDB001	Organic ac	
C23 H31 N	-0.00028	-0.62139	NA	level4	NA	C16786	NA	NA	
C15 H28 C	18.01062	70298.01	25.31203	level4	Public	NA	NA	NA	
C10 H9 N	8.63E-05	0.386825	NA	level4	NA	C19686	NA	NA	
C21 H44 N	0.000149	0.329172	92.09653	level2	mzCloud	NA	NA	NA	
C18 H30 C	21.98113	58742.6	35.50694	level4	Public	NA	NA	NA	
C44 H73 C	0.003499	4.505929	NA	level4	NA	NA	NA	NA	
C10 H14 N	2.31E-05	0.095432	NA	level4	HMDB	NA	HMDB000	Nucleosid	
C9 H11 N5	-0.00033	-1.3755	NA	level4	HMDB	NA	HMDB025	NA	
C20 H27 N	-0.0003	-0.74432	NA	level4	HMDB	NA	HMDB025	NA	
C22 H30 N	-0.00026	-0.55083	NA	level4	HMDB	NA	HMDB001	Organic ac	
C19 H24 C	-0.00014	-0.47662	NA	level4	HMDB	NA	HMDB025	NA	
C14 H24 C	-6.14E-05	-0.27374	54.47419	level3	Public	NA	NA	NA	
C31 H50 C	0.001611	2.843825	NA	level4	NA	NA	NA	NA	
C27 H36 C	-0.00031	-0.7704	NA	level4	HMDB	NA	HMDB025	NA	
C6 H10 O8	-0.00077	-3.1843	NA	level4	NA	C21064	NA	NA	
C17 H26 C	-0.00019	-0.69324	NA	level4	HMDB	NA	HMDB024	NA	
C14 H12 N	-0.00011	-0.47755	NA	level4	HMDB	NA	HMDB024	NA	
C16 H19 N	-8.22E-05	-0.31969	NA	level4	HMDB	NA	HMDB025	NA	
C26 H30 N	0.00038	0.763523	NA	level4	HMDB	NA	HMDB025	NA	
C16 H19 N	6.19E-05	0.22646	NA	level4	HMDB	NA	HMDB025	NA	
C15 H18 C	0.000116	0.32495	NA	level4	HMDB	NA	HMDB024	Organic ox	
C18 H19 N	5.11E-05	0.15536	NA	level4	NA	C10722	NA	Alkaloids	
C20 H39 N	-2.52E-05	-0.07746	94.74856	level2	mzCloud	C20792	HMDB000	Organic ni	
C18 H24 N	-8.88E-05	-0.22632	NA	level4	HMDB	NA	HMDB025	NA	
C22 H25 C	-0.00045	-1.13038	NA	level4	HMDB	NA	HMDB001	Organohe	
C5 H8 N4	0.001394	4.412375	NA	level4	HMDB	NA	HMDB025	NA	
C19 H34 C	-0.00013	-0.39952	37.91192	level4	Public	NA	NA	NA	
C10 H12 C	-4.36E-05	-0.19127	NA	level4	KEGG	C06581	NA	NA	
C29 H42 C	0.002443	4.57295	NA	level4	NA	C17525	NA	NA	
C22 H24 F	7.99E-05	0.169563	NA	level4	HMDB	NA	HMDB006	Organic ox	
C12 H16 C	-0.00049	-1.7038	NA	level4	HMDB	NA	HMDB025	NA	
C21 H39 N	-0.00013	-0.37552	45.22365	level4	Public	NA	NA	NA	
C25 H32 C	0.000418	0.890249	NA	level4	HMDB	NA	HMDB001	Organohe	
C19 H22 C	4.953909	14311.8	22.05308	level4	Public	NA	NA	NA	
C32 H58 C	0.000924	1.767749	NA	level4	NA	NA	NA	NA	
C29 H58 A	-0.00151	-2.09285	NA	level4	NA	NA	NA	NA	
C12 H10 C	5.77E-05	0.246728	NA	level4	HMDB	NA	HMDB024	NA	
C24 H26 N	-0.00154	-3.21743	NA	level4	HMDB	NA	HMDB024	NA	
C30 H55 C	-0.00039	-0.60353	NA	level4	NA	NA	NA	NA	
C12 H21 N	275.1395	1000010	19.08596	level3	BMDB	NA	HMDB001	Lipids and	
C24 H34 N	0.002385	4.464725	15.95445	level4	Public	NA	NA	NA	
C23 H39 N	-0.00144	-2.26771	NA	level4	HMDB	NA	HMDB025	NA	
C19 H24 N	-0.00056	-1.62168	NA	level4	HMDB	NA	HMDB025	NA	
C16 H20 N	5.51E-05	0.191191	NA	level4	NA	C11495	NA	NA	
C25 H48 C	-0.00015	-0.31812	NA	level4	NA	NA	NA	NA	
C18 H32 C	17.02584	33770.1	0.419025	level4	Public	C08243	HMDB001	Organic ox	
C5 F12	-0.00028	-0.96475	NA	level4	HMDB	NA	HMDB025	NA	
C16 H20 N	-9.37E-05	-0.34425	NA	level4	HMDB	NA	HMDB025	NA	
C19 H30 C	-2.59E-05	-0.08443	30.71528	level3	mzCloud	C14606	HMDB000	Lipids and	
C35 H58 C	0.000336	0.602252	NA	level4	NA	NA	NA	NA	
C45 H52 C	-0.00383	-3.97167	NA	level4	NA	C16242	NA	NA	

C25 H30 C	-0.00127	-2.43439	NA	level4	KEGG	C09501	NA	Terpenoid
C25 H26 C	-0.00064	-1.57208	3.209092	level4	Public	NA	NA	NA
C21 H32 N	-0.00033	-0.76578	NA	level4	NA	C15676	NA	NA
C21 H38 C	-18.0104	-46626.9	35.85492	level3	mzCloud	NA	NA	NA
C14 H20 C	-9.72E-05	-0.32372	21.59434	level4	Public	NA	NA	NA
C18 H19 N	0.000117	0.288629	NA	level4	HMDB	NA	HMDB001	Organohe
C15 H20 C	0.000233	1.002305	NA	level4	HMDB	NA	HMDB024	NA
C19 H24 C	21.98028	66173.31	10.18229	level4	Public	NA	NA	NA
C6 H13 N	0.001146	5.872994	27.97995	level3	BMDB	C03752	NA	Carbohydı
C24 H30 C	0.00112	2.604187	8.394678	level4	Public	NA	NA	NA
C5 H9 N C	-1.38E-05	-0.13928	50	level3	Masslist	NA	HMDB001	Organohe
C38 H40 N	-0.00056	-0.88224	NA	level4	KEGG	C09661	NA	Alkaloids
C30 H52 C	0.000689	1.4002	NA	level4	NA	NA	NA	NA
C34 H34 F	0.003097	4.898518	NA	level4	HMDB	NA	HMDB025	NA
C30 H30 N	-0.00055	-1.08378	NA	level4	HMDB	NA	HMDB025	NA
C9 H14 O4	0.00028	1.503816	NA	level4	HMDB	NA	HMDB025	NA
C20 H20 C	-0.0007	-1.6578	NA	level4	NA	NA	NA	NA
C27 H34 C	-0.0009	-1.5444	1.443028	level4	Public	NA	NA	NA
C20 H28 C	37.95564	88646.55	1.467471	level4	Public	NA	NA	NA
C9 H8 O4	6.31E-05	0.350317	41.02652	level3	mzCloud	NA	HMDB000	Benzenoid
C14 H8 O4	17.02593	70928.91	27.41192	level4	Public	NA	NA	NA
C30 H28 C	0.001177	2.280494	NA	level4	HMDB	NA	HMDB024	NA
C21 H34 C	17.02627	46489.29	35.17895	level3	mzCloud	NA	HMDB000	Lipids and
C46H58N4	0.004054	4.459666	NA	level4	NA	C11626	NA	NA
C16 H16 C	-0.00013	-0.41945	9.654901	level4	Public	NA	HMDB025	NA
C19 H18 C	0.000615	1.752133	NA	level4	HMDB	NA	HMDB001	Organohe
C6 H14 N	0.000213	0.821433	NA	level4	HMDB	NA	HMDB025	NA
C18 H18 C	-0.00065	-1.70921	NA	level4	HMDB	NA	HMDB024	NA
C17 H25 N	-4.64E-05	-0.15918	NA	level4	HMDB	NA	HMDB001	Benzenoid
C29 H47 N	17.02758	29492.76	20.44329	level4	Public	NA	NA	NA
C30 H60 N	-0.00124	-2.08488	NA	level4	NA	NA	NA	NA
C9 H14 N4	-0.0001	-0.44644	NA	level4	HMDB	NA	HMDB025	NA
C20 H39 N	-2.02E-05	-0.05907	NA	level4	HMDB	NA	HMDB024	NA
C20 H37 N	-7.63E-05	-0.23605	88.55264	level2	mzCloud	NA	NA	NA
C22 H38 C	-0.00022	-0.62098	NA	level4	NA	NA	NA	NA
C35 H60 C	0.000511	0.887021	NA	level4	NA	C20785	NA	NA
C27 H46 C	0.000237	0.544962	NA	level4	HMDB	NA	HMDB024	NA
C37 H40 C	0.000815	1.125574	NA	level4	NA	NA	NA	NA
C15 H20 N	-3.08E-05	-0.11835	NA	level4	HMDB	NA	HMDB025	NA
C11 H16 N	-0.00106	-3.7374	NA	level4	HMDB	NA	HMDB025	NA
C31 H54 N	-0.0026	-3.83006	NA	level4	NA	NA	NA	NA
C20 H17 N	0.000162	0.423737	NA	level4	NA	C10602	NA	Flavonoids
C5 H8 N2	3.73E-05	0.291381	35.55546	level3	mzCloud	NA	NA	NA
C19 H34 C	-0.00012	-0.32062	28.49443	level4	Public	NA	NA	NA
C16 H15 C	0.000754	1.548502	NA	level4	HMDB	NA	HMDB024	NA
C10 H8 N2	0.000249	1.448345	NA	level4	HMDB	NA	HMDB025	NA
C18 H25 N	-0.00011	-0.40311	45.45574	level4	Public	NA	NA	NA
C20 H30 C	17.02655	53505.66	21.1861	level4	Public	NA	NA	NA
C39 H64 C	-0.00253	-4.12638	NA	level4	NA	NA	NA	NA
C11 H8 N2	0.000228	1.236106	NA	level4	HMDB	NA	HMDB025	NA
C28 H56 N	0.000191	0.327962	NA	level4	NA	NA	NA	NA
C26 H31 N	0.000925	2.114523	NA	level4	NA	C10589	NA	NA
C21 H24 N	-0.00115	-3.30337	NA	level4	HMDB	NA	HMDB024	NA
C22 H35 C	-0.00164	-3.25842	NA	level4	HMDB	NA	HMDB025	NA

C5 H6 N2	0.000117	0.826252	NA	level4	HMDB	NA	HMDB024	NA
C28 H42 C	8.38E-05	0.160405	NA	level4	NA	NA	NA	NA
C16 H24 N	-0.00028	-1.09359	NA	level4	HMDB	NA	HMDB001	Organohe
C11 H12 N	-0.00012	-0.52481	NA	level4	HMDB	NA	HMDB025	NA
C19 H29 N	-0.00091	-3.00719	NA	level4	HMDB	NA	HMDB024	NA
C24 H46 C	0.000173	0.452313	NA	level4	NA	NA	NA	NA
C16 H12 F	-7.31E-05	-0.21813	NA	level4	HMDB	NA	HMDB024	NA
C16 H17 N	-0.00012	-0.42104	NA	level4	HMDB	NA	HMDB025	NA
C34 H38 C	0.001739	2.317406	NA	level4	NA	NA	NA	NA
C29 H39 N	0.002643	4.99462	20.31057	level4	Public	NA	NA	NA
C19 H18 N	-0.00149	-3.89448	NA	level4	HMDB	NA	HMDB025	NA
C24 H28 C	-0.00037	-1.00734	NA	level4	HMDB	NA	HMDB025	NA
C8 H18 N2	0.000202	1.277516	33.27127	level4	Public	C19277	HMDB025	NA
C19 H26 N	-0.00043	-1.17618	NA	level4	HMDB	NA	HMDB025	NA
C28 H32 N	0.001699	3.723184	NA	level4	HMDB	NA	HMDB025	NA
C2 H5 N C	-6.41E-05	-0.85463	50	level3	Masslist	C00037	HMDB000	Organic ac
C34 H60 C	0.001077	1.964637	NA	level4	NA	NA	NA	NA
C19 H27 N	-9.09E-05	-0.30177	NA	level4	HMDB	NA	HMDB024	NA
C28 H37 N	0.000552	1.192058	NA	level4	HMDB	NA	HMDB025	NA
C19 H25 N	-0.00017	-0.47402	NA	level4	HMDB	NA	HMDB025	NA
C17 H26 N	6.25E-05	0.176528	NA	level4	HMDB	NA	HMDB025	NA
C26 H30 C	-0.00102	-2.42619	NA	level4	NA	NA	NA	NA
C8 H15 N	0.000296	1.168481	NA	level4	HMDB	NA	HMDB024	NA
C18 H30	0.000162	0.656158	NA	level4	HMDB	NA	HMDB025	NA
C20 H23 C	-0.00174	-3.78719	NA	level4	NA	C15693	NA	NA
C11 H17 N	0.000186	0.820765	13.6931	level4	Public	NA	NA	NA
C22 H29 N	0.000104	0.246966	NA	level4	NA	NA	NA	NA
C32 H30 C	0.000169	0.271784	NA	level4	NA	NA	NA	NA
C28 H34 C	0.001709	3.039048	NA	level4	NA	NA	NA	NA
C15 H26 C	-2.98E-05	-0.13424	39.9459	level4	Public	NA	NA	NA
C8 H8 O3	6.31E-05	0.415145	96.44196	level2	mzCloud	C05852	HMDB000	Benzenoid
C26 H35 F	0.00226	4.648225	NA	level4	HMDB	NA	HMDB024	NA
C27 H32 C	0.001741	3.373264	NA	level4	HMDB	NA	HMDB025	NA
C12 H18 C	0.000137	0.321201	NA	level4	NA	C17388	NA	NA
C32 H50 C	-0.00119	-2.25213	NA	level4	NA	NA	NA	NA
C7 H7 N C	6.60E-05	0.431442	NA	level4	HMDB	NA	HMDB025	NA
C40 H46 N	-0.00243	-3.56367	NA	level4	KEGG	C09441	NA	Alkaloids
C13 H18 N	-0.00146	-4.52754	NA	level4	HMDB	NA	HMDB025	NA
C7 H14 O	0.000109	0.955486	NA	level4	HMDB	NA	HMDB003	Organic ox
C20 H24 N	0.001165	3.270639	NA	level4	HMDB	NA	HMDB024	NA
C23 H26 C	17.02694	41111.22	3.30949	level4	Public	NA	NA	NA
C19 H37 N	-0.00127	-3.04723	NA	level4	HMDB	NA	HMDB025	NA
C19 H30 C	17.02653	58666.76	63.48996	level2	mzCloud	C00523	HMDB000	Lipids and
C19 H23 C	-0.00068	-2.1645	NA	level4	HMDB	NA	HMDB001	Organohe
C21 H26 C	0.001695	3.589217	NA	level4	KEGG	C06491	NA	NA
C19 H14 C	-0.00049	-1.51571	NA	level4	NA	NA	NA	NA
C19 H20 N	-0.00027	-0.87478	NA	level4	HMDB	NA	HMDB025	NA
C14 H11 N	-5.67E-05	-0.23712	NA	level4	NA	C21295	NA	NA
C20 H18 C	-0.00182	-4.72062	NA	level4	HMDB	NA	HMDB025	NA
C11 H17 N	-0.00014	-0.44866	NA	level4	HMDB	NA	HMDB025	NA
C13 H16 C	0.000147	0.548711	NA	level4	HMDB	NA	HMDB024	NA
C17 H21 N	-6.09E-05	-0.17339	NA	level4	HMDB	NA	HMDB024	NA
C27 H30 C	0.000415	0.698199	17.47633	level4	Public	NA	NA	NA
C15 H19 N	-0.00164	-4.3989	NA	level4	HMDB	NA	HMDB002	Organic ox
C20 H32 C	-3.51E-05	-0.10435	85.23976	level2	mzCloud	NA	NA	NA
C9 H16 N2	-0.00018	-0.73196	NA	level4	HMDB	NA	HMDB025	NA
C14 H24 C	2.96E-05	0.142009	NA	level4	NA	NA	NA	NA
C18 H18 C	-0.00393	-14.7719	38.24123	level3	mzCloud	C10651	NA	Phenylpro

C5 H7 N C	0.00019	1.473575	94.41561	level1	Decode	C01879	HMDB000	Organic ac
C20 H21 C	-0.00014	-0.34998	NA	level4	HMDB	NA	HMDB025	NA
C21 H27 F	-0.00171	-4.51977	NA	level4	HMDB	NA	HMDB025	NA
C9 H8 O6	-0.00022	-0.91632	NA	level4	HMDB	NA	HMDB012	Phenylpro
C9 H11 N	0.000171	1.033573	NA	level4	HMDB	NA	HMDB025	NA
C28 H36 C	0.002381	4.473716	NA	level4	HMDB	NA	HMDB025	NA
C36 H58 C	0.000226	0.36504	NA	level4	HMDB	NA	HMDB025	NA
C39 H63 N	-0.00293	-3.81155	NA	level4	NA	C12152	NA	NA
C20 H43 A	0.001279	3.416834	NA	level4	NA	NA	NA	NA
C17 H18 N	-0.00017	-0.59405	NA	level4	HMDB	NA	HMDB024	NA
C27 H34 F	0.001273	2.504935	NA	level4	HMDB	NA	HMDB001	Lipids and
C13 H23 N	7.58E-05	0.265724	NA	level4	KEGG	C01833	NA	NA
C10 H11 N	0.000244	1.290304	NA	level4	HMDB	NA	HMDB025	NA
C23 H39 N	-9.97E-05	-0.26426	NA	level4	NA	NA	NA	NA
C36 H72 N	-0.0004	-0.59473	NA	level4	HMDB	NA	HMDB024	NA
C26 H28 C	0.000239	0.422868	NA	level4	NA	NA	NA	NA
C24 H20 C	0.000247	0.546294	NA	level4	NA	NA	NA	NA
C15 H13 N	-0.00138	-4.20782	NA	level4	HMDB	NA	HMDB025	NA
C26 H21 N	0.001596	3.634717	NA	level4	HMDB	NA	HMDB025	NA
C34 H52 C	0.001398	2.142293	NA	level4	NA	NA	NA	NA
C19 H18 C	-0.00036	-0.9564	61.13754	level2	Public	NA	NA	NA
C16 H27 N	0.000616	1.968058	NA	level4	NA	NA	NA	NA
C31 H41 N	0.000355	0.746971	NA	level4	NA	NA	NA	NA
C26 H36 C	0.000399	0.784646	7.255057	level4	Public	NA	NA	NA
C33 H40 C	-0.00023	-0.31069	NA	level4	HMDB	NA	HMDB025	NA
C32 H36 N	-0.00261	-4.93858	NA	level4	NA	NA	NA	NA
C14 H29 N	0.000165	0.724086	NA	level4	NA	NA	NA	NA
C22 H27 N	0.000312	0.747143	NA	level4	NA	NA	NA	NA
C21 H20 C	0.00038	0.819462	NA	level4	HMDB	NA	HMDB025	NA
C26 H45 N	-0.00186	-2.69327	NA	level4	HMDB	NA	HMDB024	NA
C9 H11 N	-0.00017	-0.61919	NA	level4	HMDB	NA	HMDB000	Organic ac
C24 H30 N	0.000978	2.133935	NA	level4	HMDB	NA	HMDB024	NA
C15 H16 N	-6.12E-05	-0.21229	NA	level4	HMDB	NA	HMDB024	NA
C21 H35 N	-7.79E-05	-0.23373	47.2829	level4	Public	NA	NA	NA
C22 H17 C	-0.00121	-2.90711	NA	level4	HMDB	NA	HMDB025	NA
C8 H11 N	0.000255	1.37982	NA	level4	HMDB	NA	HMDB024	NA
C18 H12 C	0.000964	3.129118	NA	level4	NA	NA	NA	NA
C23 H28 C	17.02814	37994.53	0.627337	level4	Public	NA	NA	NA
C13 H27 N	0.001104	3.275585	NA	level4	NA	C17982	NA	NA
C37 H40 N	-0.00065	-0.99702	NA	level4	KEGG	C09208	NA	NA
C19 H23 N	-0.00024	-0.86551	NA	level4	HMDB	NA	HMDB024	NA
C25 H30 C	0.001195	2.51989	8.98397	level4	Public	NA	NA	NA
C20 H14 C	-0.00052	-1.55261	NA	level4	HMDB	NA	HMDB025	NA
C21 H25 F	-0.00176	-4.7977	NA	level4	NA	C15340	NA	NA
C15 H16 N	-0.00021	-0.83069	NA	level4	HMDB	NA	HMDB024	NA
C9 H15 N3	9.87E-05	0.430718	NA	level4	HMDB	NA	HMDB000	Organic ac
C13 H14 C	0.00121	4.546634	NA	level4	HMDB	NA	HMDB025	NA
C26 H45 C	-0.00078	-1.47	NA	level4	NA	NA	NA	NA
C6 H5 F O	4.22E-05	0.263942	NA	level4	NA	C16477	NA	Organohe
C23 H25 N	-0.00149	-4.49624	NA	level4	KEGG	C09220	NA	Organohe
C5 H8 O5	-4.12E-05	-0.27863	92.0259	level2	mzCloud	C01087	HMDB000	Organic ac
UnkownFo	302.1655	Inf	14.76112	level4	Public	NA	NA	NA
C23 H41 N	-6.69E-06	-0.01763	NA	level4	NA	NA	NA	NA
C18 H26 N	37.9572	95329.39	3.114465	level4	Public	NA	NA	NA

C10 H14 N	0.000114	0.415657	NA	level4	HMDB	NA	HMDB024	NA
C28 H42 C	0.000247	0.539111	NA	level4	NA	NA	NA	NA
C13 H10 C	-0.00032	-1.37429	NA	level4	KEGG	C06641	NA	NA
C14 H12 C	3.20E-05	0.140166	NA	level4	HMDB	NA	HMDB025	NA
C11 H21 N	0.000196	1.06785	NA	level4	KEGG	C09989	NA	NA
C22 H30 F	-0.00086	-1.83581	NA	level4	HMDB	NA	HMDB024	NA
C10 H20 C	9.16E-05	0.53234	96.15867	level2	mzCloud	C01571	HMDB000	Lipids and
C22 H32 C	-4.63E-05	-0.14092	89.48461	level2	mzCloud	C06429	HMDB000	Lipids and
C35 H56 C	21.98072	35430.35	4.278229	level4	Public	NA	NA	NA
C24 H16 N	0.002055	4.244269	NA	level4	HMDB	NA	HMDB025	NA
C17 H23 N	-2.13E-05	-0.07363	NA	level4	HMDB	NA	HMDB001	Benzenoid
C24 H32 C	1.03E-05	0.027837	NA	level4	HMDB	NA	HMDB025	NA
C21 H27 N	2.90E-05	0.052015	NA	level4	HMDB	NA	HMDB024	NA
C18 H19 N	-0.00025	-0.82733	NA	level4	HMDB	NA	HMDB025	NA
C15 H23 N	-0.00062	-1.81864	NA	level4	HMDB	NA	HMDB001	Benzenoid
C26 H32 C	0.000432	0.885527	NA	level4	HMDB	NA	HMDB025	NA
C35 H36 N	-0.00041	-0.64604	NA	level4	NA	C18154	NA	NA
C9 H8 N2	0.000235	1.469505	NA	level4	HMDB	NA	HMDB024	NA
C14 H11 N	-0.00014	-0.50407	NA	level4	HMDB	NA	HMDB024	NA
C33 H50 N	0.001162	1.842679	NA	level4	HMDB	NA	HMDB001	Organic ac
C15 H12 C	17.02652	62581.76	9.154342	level4	Public	NA	NA	NA
C5 H8 N2	7.01E-05	0.39823	NA	level4	KEGG	C04209	NA	Organic ac
C29 H46 C	0.00105	2.009415	12.95988	level4	Public	NA	NA	NA
C24 H24 C	0.000338	0.716778	2.906588	level4	Public	NA	NA	NA
C21 H23 F	0.001339	3.097766	NA	level4	HMDB	NA	HMDB025	NA
C17 H20 N	-0.00162	-4.13955	NA	level4	HMDB	NA	HMDB024	NA
C7 H5 N C	-2.14E-05	-0.18016	NA	level4	HMDB	NA	HMDB024	NA
C11 H16 N	0.00023	1.305108	NA	level4	HMDB	NA	HMDB024	NA
C15 H20 C	0.000163	0.658419	37.90535	level4	Public	NA	NA	NA
C42 H82 N	0.000857	1.152981	NA	level4	HMDB	NA	HMDB001	Lipids and
C9 H8 N2	0.00033	2.29134	NA	level4	HMDB	NA	HMDB024	NA
C44 H82 N	0.003105	3.80731	NA	level4	NA	NA	NA	NA
C20 H28 C	21.9816	57818.4	5.6452	level4	Public	NA	NA	NA
C25 H26 C	17.02754	38860.76	2.855325	level4	Public	NA	NA	NA
C13 H11 C	-0.00022	-1.02206	64.41462	level2	Public	NA	HMDB024	NA
C15 H16 N	-1.42E-05	-0.05207	NA	level4	HMDB	NA	HMDB024	NA
C16 H15 N	0.000835	3.523224	NA	level4	HMDB	NA	HMDB025	NA
C18 H37 N	-0.00369	-10.163	44.29509	level3	mzCloud	NA	NA	NA
C16 H22 N	324.1569	1072748	8.411561	level4	Public	NA	NA	NA
C16 H27 N	-0.00028	-0.85953	NA	level4	HMDB	NA	HMDB025	NA
C14 H22 C	0.000118	0.464164	36.95424	level4	Public	NA	NA	NA
C9 H9 N C	5.51E-05	0.374497	NA	level4	HMDB	NA	HMDB025	NA
C19 H34 C	0.000923	2.033153	NA	level4	NA	NA	NA	NA
C28 H34 C	-0.00039	-0.87066	20.46168	level4	Public	NA	NA	NA
C20 H36 C	-2.53E-05	-0.07442	NA	level4	NA	NA	NA	NA
C43 H58 N	0.003022	3.604992	NA	level4	NA	C21143	NA	NA
C22 H32 C	0.000339	0.864115	NA	level4	NA	NA	NA	NA
C6 H14 O5	0.000123	0.742236	NA	level4	HMDB	NA	HMDB024	NA
C7 H8 N4	0.000228	1.264587	97.53462	level1	BMDB	C07480	HMDB000	Organohe
C4 H10 O2	9.35E-05	0.766059	NA	level4	HMDB	NA	HMDB025	NA
C35 H56 C	0.000808	1.336046	NA	level4	KEGG	C08632	NA	Terpenoid
C22 H18 C	0.000103	0.283237	NA	level4	NA	NA	NA	NA
C10 H13 F	-0.0011	-4.2172	NA	level4	HMDB	NA	HMDB025	NA
C11 H16 N	8.18E-05	0.268902	90.61937	level2	mzCloud	C12270	HMDB000	Organic ac

C19 H24 (	-0.00013	-0.37693	NA	level4	HMDB	NA	HMDB025	NA
C28 H46 (	0.000114	0.247246	NA	level4	NA	NA	NA	NA
C19 H22 (	17.02774	41518.79	3.473418	level4	Public	NA	NA	NA
C17 H11 (	0.001563	4.04783	NA	level4	HMDB	NA	HMDB001	Organohe
C8 H9 N C	9.31E-05	0.688933	NA	level4	HMDB	NA	HMDB024	NA
C9 H12 N2	0.001013	4.441382	NA	level4	HMDB	NA	HMDB024	NA
C28 H30 F	0.001807	3.802101	NA	level4	HMDB	NA	HMDB025	NA
C15 H29 N	-0.00022	-0.6765	NA	level4	HMDB	NA	HMDB001	Organic o)
C17 H18 (	37.9574	119321.5	13.84222	level4	Public	NA	NA	NA
C6 H12 O4	0.00017	1.146607	62.51481	level2	mzCloud	C00418	HMDB000	Lipids and
C16 H22 N	-0.00166	-4.57489	NA	level4	HMDB	NA	HMDB025	NA
C11 H14 N	0.000193	1.014456	67.21627	level2	mzCloud	C10763	NA	Alkaloids
C8 H11 N	-18.0104	-117654	96.3086	level1	BMDB	C04227	HMDB000	Benzenoid
C5 H7 N3	0.000134	1.070589	NA	level4	HMDB	NA	HMDB024	NA
C46 H86 N	-5.50E-05	-0.06772	NA	level4	HMDB	NA	HMDB000	Lipids and
C26 H44 (	-0.00032	-0.65903	9.297452	level4	Public	NA	NA	NA
C29 H39 N	0.002661	4.881053	NA	level4	HMDB	NA	HMDB025	NA
C23 H38 (	4.55E-06	0.011546	NA	level4	HMDB	NA	HMDB025	NA
C15 H15 N	-0.00042	-1.09648	NA	level4	HMDB	NA	HMDB003	Organohe
C41 H68 (	-0.00239	-3.04291	NA	level4	HMDB	NA	HMDB024	NA
C22 H28 (	0.00013	0.348884	NA	level4	HMDB	NA	HMDB024	NA
C29 H33 F	-0.00095	-1.8217	NA	level4	HMDB	NA	HMDB025	NA
C6 H11 N	8.81E-05	0.497637	NA	level4	HMDB	NA	HMDB024	NA
C10 H11 N	37.95459	149987.2	31.78485	level3	mzCloud	C07315	HMDB001	Benzenoid
C23 H32 (	-0.00241	-4.63485	NA	level4	HMDB	NA	HMDB025	NA
C21 H18 (	21.98018	65785.18	4.702157	level4	Public	NA	NA	NA
C14H17N2	-0.00114	-3.0495	NA	level4	NA	C13677	NA	NA
C23 H30 (	-0.00178	-4.79793	NA	level4	HMDB	NA	HMDB025	NA
C5 H9 N C	-18.0104	-137423	86.85079	level1	BMDB	C00430	HMDB000	Organic ac
C45 H77 (	0.002211	2.734229	NA	level4	NA	NA	NA	NA
C5 H10 N2	-0.00028	-1.01921	NA	level4	HMDB	NA	HMDB001	Organic ac
C11 H8 O2	0.000175	1.018603	NA	level4	HMDB	NA	HMDB024	NA
C19 H38 (	-18.0106	-54531.9	42.07694	level4	Public	NA	NA	NA
C13 H17 (	8.89E-05	0.331762	NA	level4	HMDB	NA	HMDB001	Benzenoid
C43 H82 N	0.00206	2.465897	NA	level4	NA	NA	NA	NA
C25 H34 (	0.000696	1.407365	NA	level4	KEGG	C08763	NA	Terpenoid
C21 H18 (	0.000125	0.27935	66.52505	level2	Public	NA	HMDB024	Organic ac
C13 H18 (	0.000228	1.105971	75.95698	level2	Decode	C01588	HMDB000	Phenylpro
C33 H40 (	0.002393	4.364293	5.004929	level4	Public	NA	NA	NA
C7 H10 Cl	-0.00018	-0.78181	NA	level4	HMDB	NA	HMDB024	NA
C27 H24 N	-0.00226	-4.9907	NA	level4	KEGG	C07349	NA	NA
C15 H13 N	-9.01E-05	-0.33249	NA	level4	HMDB	NA	HMDB006	Organic o)
C10 H14 N	-0.00037	-1.02858	NA	level4	HMDB	NA	HMDB000	Nucleosid
C19 H23 N	-0.00155	-3.44012	NA	level4	HMDB	NA	HMDB025	NA
C33 H30 (	-0.00211	-3.17303	NA	level4	HMDB	NA	HMDB006	Organohe
C12 H12 N	6.33E-05	0.255339	NA	level4	HMDB	NA	HMDB006	Organohe
C20 H22 N	0.000246	0.588052	NA	level4	KEGG	C06446	NA	NA
C25 H28 (	17.02583	31755.54	0.579671	level4	Public	NA	NA	NA
C H Cl O2	-0.0002	-2.54449	NA	level4	HMDB	NA	HMDB025	NA
C10 H15 N	0.000243	1.47289	87.26182	level2	mzCloud	C01575	HMDB001	Benzenoid
C24 H28 (	21.98029	55478.65	25.45052	level4	Public	NA	NA	NA
C11 H17 N	0.000306	1.477924	NA	level4	HMDB	NA	HMDB025	NA
C16 H14 (	0.000211	0.551576	NA	level4	HMDB	NA	HMDB002	Phenylpro
C24 H51 N	-0.00047	-0.95009	NA	level4	KEGG	C04102	NA	GP Glycer
C20 H27 F	-0.0012	-3.58759	NA	level4	NA	C15092	NA	NA
C11 H11 N	0.000119	0.5788	NA	level4	HMDB	NA	HMDB024	NA
C16 H18 (	-3.33E-05	-0.1087	NA	level4	HMDB	NA	HMDB024	NA

C22 H24 N	0.000371	1.019231	NA	level4	NA	C12073	NA	NA
C15 H24 C	5.79E-05	0.203764	NA	level4	HMDB	NA	HMDB006	Organic o:
C14 H23 N	-6.84E-05	-0.2077	NA	level4	KEGG	C06668	NA	NA
C16 H12 C	0.000158	0.453307	NA	level4	HMDB	NA	HMDB024	Phenylpro
C24 H29 N	0.000448	0.94209	NA	level4	HMDB	NA	HMDB006	Alkaloids a
C25 H25 F	9.08E-05	0.170016	NA	level4	HMDB	NA	HMDB025	NA
C32 H64 N	0.002472	3.877955	NA	level4	NA	NA	NA	NA
C12 H18 C	0.000311	1.603642	NA	level4	HMDB	NA	HMDB001	Benzenoid
C14 H15 N	-0.00106	-3.92403	NA	level4	HMDB	NA	HMDB025	NA
C17 H21 N	17.02674	59295.18	18.76327	level4	Public	NA	HMDB003	Organohe
C7 H11 N3	0.00023	1.359612	93.40321	level1	BMDB	C01152	HMDB000	Organic ac
C10 H16 N	0.000115	0.469199	NA	level4	HMDB	NA	HMDB024	NA
C18 H11 N	0.003188	10.5897	38.18209	level3	mzCloud	NA	NA	NA
C40 H78 N	-0.00055	-0.7411	NA	level4	HMDB	NA	HMDB025	NA
C6 H13 N	0.000318	1.630539	NA	level4	HMDB	NA	HMDB024	NA
C13 H6 F2	0.000176	0.677512	NA	level4	HMDB	NA	HMDB025	NA
C24 H23 N	-0.0006	-1.2598	NA	level4	HMDB	NA	HMDB024	NA
C10 H19 N	0.000146	0.727512	NA	level4	HMDB	NA	HMDB025	NA
C21 H22 C	0.000327	0.677653	NA	level4	HMDB	NA	HMDB025	NA
C20 H32 C	4.92E-05	0.139725	NA	level4	HMDB	NA	HMDB000	Lipids and
C33 H38 N	0.000692	1.063836	NA	level4	HMDB	NA	HMDB024	NA
C8 H15 N	0.000152	0.964592	NA	level4	HMDB	NA	HMDB024	NA
C9 H6 N2	-0.00086	-3.3989	NA	level4	HMDB	NA	HMDB025	NA
C26 H27 F	0.00049	0.860638	NA	level4	HMDB	NA	HMDB025	NA
C6 H6 O2	0.000172	1.560891	32.59187	level4	BMDB	C00530	HMDB000	Benzenoid
C22 H18 C	0.000494	1.078653	3.694193	level4	Public	C09731	HMDB000	Phenylpro
C11 H12 N	-0.00158	-7.74452	90.20601	level2	mzCloud	C00806	HMDB003	Organohe
C33 H38 C	-0.0005	-0.65375	NA	level4	NA	NA	NA	NA
C24 H26 C	7.84E-05	0.165433	NA	level4	NA	NA	NA	NA
C46 H80 N	-0.00053	-0.63302	NA	level4	HMDB	NA	HMDB001	Lipids and
C7 H13 N	0.000303	1.584641	NA	level4	HMDB	NA	HMDB025	NA
C9 H12 O4	-18.0104	-97843.3	64.81872	level2	Public	C05594	HMDB000	Benzenoid
C24 H20 A	0.001022	2.666907	NA	level4	NA	C11316	NA	NA
C18 H21 N	-0.00158	-4.45023	NA	level4	HMDB	NA	HMDB025	NA
C23 H47 C	-0.00119	-2.65147	NA	level4	NA	NA	NA	NA
C33 H54 C	-0.00018	-0.33394	NA	level4	HMDB	NA	HMDB025	NA
C15 H24 N	-0.00017	-0.53312	NA	level4	HMDB	NA	HMDB025	NA
C22 H41 C	0.003033	4.512727	NA	level4	NA	NA	NA	NA
C28 H32 C	30.01292	58595.57	30.69238	level4	Public	NA	NA	NA
C16 H13 C	-0.00088	-2.94539	NA	level4	HMDB	NA	HMDB025	NA
C19 H18 N	0.001672	3.303984	NA	level4	HMDB	NA	HMDB001	Organohe
C20 H16 C	0.000112	0.274832	NA	level4	HMDB	NA	HMDB025	NA
C17 H30 N	0.000214	0.641178	NA	level4	HMDB	NA	HMDB024	NA
C25 H26 F	-0.00155	-3.31748	NA	level4	HMDB	NA	HMDB025	NA
C22 H32 C	0.001491	3.514263	NA	level4	HMDB	NA	HMDB025	NA
C22 H27 N	-0.00192	-4.47781	NA	level4	HMDB	NA	HMDB024	NA
C20 H36 C	7.98E-05	0.246007	25.22952	level4	Public	NA	NA	NA
C22 H43 N	1.94E-05	0.052571	NA	level4	NA	NA	NA	NA
C16 H30 C	-0.00015	-0.53749	85.53402	level2	mzCloud	C19615	HMDB000	Lipids and
C20 H36 C	6.51E-06	0.019119	NA	level4	NA	NA	NA	NA
C11 H18 N	9.15E-05	0.435549	NA	level4	HMDB	NA	HMDB003	Organic ac
C11 H14 C	-1.85E-05	-0.06049	NA	level4	HMDB	NA	HMDB012	Lipids and
C16 H15 C	0.000348	1.053667	NA	level4	HMDB	NA	HMDB024	NA
C12 H20 N	-0.00069	-2.379	NA	level4	KEGG	C07558	NA	NA

C5 H7 N C	0.000278	2.859817	NA	level4	HMDB	NA	HMDB024	NA
C14 H17 N	-0.00175	-4.87423	NA	level4	KEGG	C08342	NA	Amino aci
C20 H22 N	0.001656	3.629695	NA	level4	KEGG	C00445	NA	NA
C31 H38 C	-0.00148	-2.27476	NA	level4	NA	NA	NA	NA
C20 H34 C	-0.00012	-0.39788	89.07469	level2	mzCloud	C03242	HMDB000	Lipids and
C16 H16 C	0.000211	0.822813	NA	level4	HMDB	NA	HMDB025	NA
C26 H50 C	-54.0295	-97468.1	8.318342	level4	Public	NA	NA	NA
C24 H26 F	-0.00165	-4.01624	NA	level4	HMDB	NA	HMDB001	Organohe
C27 H24 C	0.002202	3.439367	NA	level4	HMDB	NA	HMDB006	Organic o:
C22 H24 C	21.98022	62414.13	7.813836	level4	Public	NA	NA	NA
C38 H51 N	-0.00245	-3.9683	NA	level4	NA	C20585	NA	NA
C5 H5 N C	0.000306	2.138789	NA	level4	NA	C18232	NA	NA
C26 H36 C	-0.00191	-3.87339	NA	level4	HMDB	NA	HMDB006	Lipids and
C16 H20 N	-0.00012	-0.39635	47.75748	level4	Public	NA	NA	NA
C23 H26 N	-0.00161	-3.30438	NA	level4	NA	C17351	NA	NA
C24 H32 C	-4.23E-05	-0.10579	NA	level4	HMDB	NA	HMDB025	NA
C35 H30 N	-0.00074	-1.21701	NA	level4	NA	C11831	NA	NA
C17 H18 N	0.001294	2.869505	NA	level4	KEGG	C06433	NA	NA
C48 H84 N	0.000367	0.440537	NA	level4	HMDB	NA	HMDB000	Lipids and
C5 H5 N C	0.000158	1.247544	NA	level4	KEGG	C03458	NA	NA
C16 H18 F	-0.00051	-1.60447	NA	level4	HMDB	NA	HMDB001	Organohe
C21 H26 C	-2.23E-05	-0.05098	NA	level4	HMDB	NA	HMDB025	NA
C20 H37 N	0.000206	0.531202	NA	level4	NA	NA	NA	NA
C16 H21 N	0.000573	1.711122	NA	level4	HMDB	NA	HMDB025	NA
C10 H20 N	0.000214	0.922176	NA	level4	HMDB	NA	HMDB025	NA
C24 H47 N	1.37E-05	0.034431	NA	level4	HMDB	NA	HMDB024	NA
C11 H12 N	6.47E-05	0.273875	47.6973	level3	BMDB2	C02406	HMDB000	Organic o:
C15 H22 C	-3.23E-05	-0.1291	31.79584	level4	Public	NA	NA	NA
C28 H32 C	0.002035	3.853518	6.608184	level4	Public	NA	NA	NA
C15 H20 C	-6.66E-05	-0.2135	NA	level4	KEGG	C05855	NA	Phenylpro
C39 H77 N	0.000887	1.459702	NA	level4	HMDB	NA	HMDB024	NA
C16 H20 N	-8.67E-05	-0.30095	NA	level4	NA	C11494	NA	NA
C28 H38 N	0.000475	0.903249	NA	level4	HMDB	NA	HMDB025	NA
C7 H15 N	0.000168	1.04203	69.90449	level1	BMDB	C00318	HMDB000	Organic ni
C15 H19 N	0.000137	0.473578	NA	level4	HMDB	NA	HMDB025	NA
C51 H82 C	-0.0029	-3.05038	NA	level4	NA	NA	NA	NA
C32 H42 C	-0.00244	-3.94999	NA	level4	KEGG	C08853	NA	Terpenoid
C55 H70 N	-0.00141	-1.5526	NA	level4	KEGG	C05307	NA	Organohe
C15 H16 C	-0.00014	-0.60613	NA	level4	HMDB	NA	HMDB024	NA
C9 H13 N	0.00093	3.762577	NA	level4	KEGG	C05931	NA	NA
C17 H14 C	-0.00192	-4.60394	NA	level4	NA	C10992	NA	NA
C5 H10 N6	0.00048	2.823897	NA	level4	HMDB	NA	HMDB025	NA
C37 H52 C	0.000309	0.567546	NA	level4	NA	NA	NA	NA
C19 H29 N	-0.00013	-0.33192	NA	level4	HMDB	NA	HMDB026	NA
C51 H85 C	0.002918	3.115818	NA	level4	NA	NA	NA	NA
C13 H8 O3	0.000209	0.987188	45.68249	level4	Public	NA	HMDB001	Phenylpro
C30 H32 C	0.000979	1.675905	NA	level4	HMDB	NA	HMDB024	NA
C25 H30 C	-0.00262	-5.71053	32.72614	level4	Public	NA	NA	NA
C44 H78 N	-0.00024	-0.29571	NA	level4	HMDB	NA	HMDB001	Lipids and
C5 H14 N2	0.000444	3.312911	NA	level4	HMDB	NA	HMDB025	NA
C36 H74 N	0.000985	1.485157	NA	level4	HMDB	NA	HMDB002	Lipids and
C34 H47 N	0.00191	2.888578	NA	level4	KEGG	C08655	NA	Alkaloids
C7 H7 N C	0.000188	1.37311	78.81707	level1	Decode	C00108	HMDB000	Benzenoid

C14 H12 N	3.59E-06	0.014946	NA	level4	HMDB	NA	HMDB025	NA
C15 H19 N	-0.00063	-1.94917	NA	level4	HMDB	NA	HMDB026	NA
C22 H18 C	-0.00028	-0.54348	NA	level4	HMDB	NA	HMDB025	NA
C40 H76 C	-0.00372	-4.76688	NA	level4	NA	NA	NA	NA
C17 H26 C	-9.29E-05	-0.29935	NA	level4	KEGG	C09622	NA	PR Prenol
C12 H26 C	-0.00021	-0.7992	51.47051	level3	Public	NA	NA	NA
C16 H19 N	-5.34E-05	-0.16034	NA	level4	HMDB	NA	HMDB025	NA
C31 H36 N	-0.00038	-0.6144	NA	level4	HMDB	NA	HMDB025	NA
C16 H10	-0.00052	-2.57433	NA	level4	HMDB	NA	HMDB025	NA
C12 H16 C	0.000123	0.546969	NA	level4	NA	C21712	NA	Benzenoid
C16 H14 C	8.84E-05	0.295688	NA	level4	HMDB	NA	HMDB001	Organohe
C8 H13 N	0.000142	0.915161	NA	level4	HMDB	NA	HMDB025	NA
C24 H28 C	17.0268	34595.84	4.951401	level4	Public	NA	NA	NA
C30 H48 C	-252.173	-500000	31.18393	level4	Public	NA	NA	NA
C20 H29 N	-5.18E-05	-0.13106	NA	level4	HMDB	NA	HMDB025	NA
C16 H13 C	3.04E-05	0.086227	NA	level4	HMDB	NA	HMDB024	NA
C15 H14 N	0.000823	2.588228	NA	level4	HMDB	NA	HMDB024	NA
C49 H78 N	-0.0043	-4.56599	NA	level4	HMDB	NA	HMDB025	NA
C10 H10 C	9.98E-05	0.56066	59.95884	level3	mzCloud	NA	HMDB000	Phenylpro
C18 H27 N	2.33E-05	0.060411	NA	level4	NA	NA	NA	NA
C9 H21 N3	0.00029	1.552017	86.98728	level2	mzCloud	C01029	HMDB000	Organic ac
C16 H17 N	-1.25E-05	-0.03907	NA	level4	HMDB	NA	HMDB024	NA
C17 H20 C	0.000121	0.329494	63.08346	level2	Public	NA	NA	NA
C16 H20 N	0.000182	0.469322	NA	level4	HMDB	NA	HMDB025	NA
C20 H30 C	17.02527	36837.37	5.095107	level4	Public	NA	NA	NA
C42 H69 C	0.003461	4.526809	NA	level4	NA	NA	NA	NA
C44 H57 N	0.00094	1.206099	NA	level4	HMDB	NA	HMDB025	NA
C20 H20 C	-0.00029	-0.84881	NA	level4	HMDB	NA	HMDB024	NA
C7 H6 O2	17.02675	139521.5	44.93	level3	BMDB	C00180	HMDB000	Benzenoid
C20 H31 N	0.000118	0.267676	NA	level4	HMDB	NA	HMDB024	NA
C15 H26 N	-0.00121	-3.48353	NA	level4	NA	C17970	NA	NA
C6 H7 N C	9.39E-05	0.497064	NA	level4	HMDB	NA	HMDB000	Organic ac
C34 H31 N	-0.00027	-0.49264	NA	level4	HMDB	NA	HMDB024	NA
C21 H44 N	0.000892	1.838618	NA	level4	NA	NA	NA	NA
C41 H78 N	0.00178	2.343788	NA	level4	NA	NA	NA	NA
C30 H34 C	17.02558	32601.97	12.29209	level4	Public	NA	NA	NA
C33 H42 C	0.000712	1.049824	NA	level4	NA	NA	NA	NA
C6 H5 Br C	-0.00074	-4.29244	NA	level4	NA	C14453	NA	Benzenoid
C10 H13 N	0.000215	1.018753	96.98954	level1	BMDB	NA	HMDB000	Organic ac
C34 H36 N	0.002383	4.223181	NA	level4	HMDB	NA	HMDB026	NA
C5 H8 O6	0.000119	0.727352	NA	level4	KEGG	C02928	NA	NA
C29 H53 N	0.000193	0.345162	4.079825	level4	Public	NA	NA	NA
C26 H39 N	0.000213	0.51538	NA	level4	NA	NA	NA	NA
C43 H71 C	0.001971	2.531443	NA	level4	NA	NA	NA	NA
C32 H34 C	-0.00241	-3.4101	NA	level4	NA	NA	NA	NA
C19 H40 N	0.000221	0.520032	NA	level4	NA	NA	NA	NA
C26 H38 N	-0.00246	-4.27902	NA	level4	HMDB	NA	HMDB025	NA
C10 H16 N	0.001606	4.939864	NA	level4	NA	C18658	NA	NA
C30 H46 C	-0.00196	-3.46581	NA	level4	NA	NA	NA	NA
C20 H39 N	-0.00016	-0.41956	NA	level4	NA	NA	NA	NA
C15 H14 C	-0.001	-4.11572	NA	level4	NA	C15045	NA	NA
C20 H24 C	21.98032	66978.99	33.33612	level4	Public	NA	NA	NA
C19 H37 N	-0.00031	-0.86938	NA	level4	HMDB	NA	HMDB006	Lipids and

C10 H15 (	0.000891	3.621571	NA	level4	NA	C18987	NA	NA
C17 H30 N	0.001403	4.099994	NA	level4	HMDB	NA	HMDB024	NA
C10 H9 N	4.10E-05	0.214412	NA	level4	NA	C11048	NA	NA
C32 H44 (	0.001195	1.926373	NA	level4	NA	NA	NA	NA
C22 H23 N	-4.05E-05	-0.09804	9.414274	level4	Public	C09592	HMDB003	Alkaloids a
C27 H46 N	0.0001	0.144503	NA	level4	HMDB	NA	HMDB025	NA
C20 H18 (	-0.00027	-0.7757	20.79655	level4	Public	NA	NA	NA
C18 H26 (	-0.00034	-1.24565	NA	level4	HMDB	NA	HMDB000	Lipids and
C6 H12 O!	-0.00031	-1.18976	NA	level4	KEGG	C01067	NA	NA
C22 H14 F	0.00109	2.123989	NA	level4	HMDB	NA	HMDB025	NA
C21 H27 F	-0.00111	-3.19878	NA	level4	NA	C15327	NA	NA
C20 H30 (	37.95401	108375.2	7.204008	level4	Public	NA	NA	NA
C9 H19 Cl	0.000524	1.664182	NA	level4	HMDB	NA	HMDB025	NA
C22 H21 N	0.001438	4.142548	NA	level4	HMDB	NA	HMDB025	NA
C26 H25 F	0.001966	3.695043	NA	level4	HMDB	NA	HMDB025	NA
C16 H28 N	-0.0001	-0.24763	NA	level4	HMDB	NA	HMDB025	NA
C4 H5 N3	5.14E-05	0.40421	NA	level4	HMDB	NA	HMDB006	Organohe
C19 H15 N	-0.00117	-3.24912	NA	level4	HMDB	NA	HMDB026	NA
C27 H30 (	-0.00034	-0.62007	NA	level4	KEGG	C04194	NA	NA
C22 H28 (	21.98045	56623.13	11.43625	level4	Public	NA	NA	NA
C21 H22 (	0.00013	0.300105	20.3698	level4	Public	NA	NA	NA
C23 H29 (	0.000294	0.705837	NA	level4	HMDB	NA	HMDB025	NA
C20 H27 N	0.001262	3.347112	NA	level4	HMDB	NA	HMDB024	NA
C6 H14 O:	9.89E-05	0.737747	NA	level4	HMDB	NA	HMDB025	NA
C23 H32 N	-0.00242	-4.95133	NA	level4	HMDB	NA	HMDB001	Benzenoid
C40 H54 (	0.001146	2.022583	NA	level4	HMDB	NA	HMDB000	Lipids and
C23 H41 N	-0.00019	-0.43946	NA	level4	NA	NA	NA	NA
C14 H19 N	-0.00101	-3.71444	NA	level4	HMDB	NA	HMDB025	NA
C54 H82 (	0.000297	0.358922	NA	level4	NA	NA	NA	NA
C17 H18 N	-0.00015	-0.43975	NA	level4	KEGG	C08556	NA	Amino aci
C18 H21 N	-0.00021	-0.70189	45.18283	level3	mzCloud	C08024	HMDB001	Alkaloids a
C10 H20 N	2.50E-05	0.084333	NA	level4	HMDB	NA	HMDB024	NA
C7 H14 N4	0.00033	1.632442	NA	level4	HMDB	NA	HMDB024	NA
C8 H14 N2	-0.00011	-0.50806	NA	level4	KEGG	C03738	NA	NA
C13 H15 N	1.24E-05	0.042281	NA	level4	HMDB	NA	HMDB025	NA
C15 H20 (	17.02671	68617.01	17.4881	level4	Public	NA	HMDB003	Lipids and
C6 H13 N	-18.0098	-69526	7.951696	level3	BMDB	C04132	HMDB000	Organic ox
C20 H22 (	21.98008	61372.6	12.06783	level4	Public	NA	NA	NA
C10 H11 N	7.99E-05	0.301552	NA	level4	HMDB	NA	HMDB024	NA
C6 H16 O1	-0.00057	-1.14603	NA	level4	HMDB	NA	HMDB024	NA
C26 H32 F	0.000404	0.798265	NA	level4	NA	NA	NA	NA
C13 H22 N	-4.27E-05	-0.11801	NA	level4	NA	C17362	NA	NA
C8 H17 N	0.000157	0.820676	NA	level4	HMDB	NA	HMDB025	NA
C31 H52 (	-0.00247	-3.81014	NA	level4	NA	NA	NA	NA
C6 H7 N C	0.000119	0.757827	NA	level4	HMDB	NA	HMDB025	NA
C35 H42 N	-0.01226	-19.3277	36.03602	level3	mzCloud	NA	NA	NA
C22 H24 (	-0.00205	-4.77263	NA	level4	HMDB	NA	HMDB024	NA
C20 H13 (	0.001358	3.721027	NA	level4	HMDB	NA	HMDB025	NA
C15 H24 N	-0.00013	-0.46466	NA	level4	HMDB	NA	HMDB024	NA
C18 H28 (	18.01052	61636.85	83.93736	level3	mzCloud	C01226	NA	FA Fatty a
C12 H19 N	-7.93E-05	-0.26328	NA	level4	HMDB	NA	HMDB025	NA
C46 H86 N	2.05E-05	0.025771	NA	level4	HMDB	NA	HMDB001	Lipids and
C19 H20 N	2.36E-06	0.007662	NA	level4	HMDB	NA	HMDB024	NA
C10 H9 N	-6.78E-05	-0.28365	NA	level4	HMDB	NA	HMDB024	NA
C10 H16 N	0.00013	0.501413	NA	level4	HMDB	NA	HMDB024	NA

C14 H18 C	-0.00015	-0.59792	64.70773	level2	mzCloud	NA	NA	NA
C7 H11 N	0.000282	2.000737	37.56447	level3	mzCloud	NA	NA	NA
C13 H28 N	0.001678	4.99046	NA	level4	NA	C17983	NA	NA
C27 H32 C	-0.0001	-0.16775	NA	level4	NA	NA	NA	NA
C34 H44 C	0.001089	1.649946	NA	level4	NA	NA	NA	NA
C17 H33 N	-0.00036	-1.08601	NA	level4	HMDB	NA	HMDB006	Lipids and
C16 H22 N	0.000215	0.644155	NA	level4	HMDB	NA	HMDB025	NA
C16 H19 N	-0.00023	-0.67199	70.33333	level2	Public	NA	NA	NA
C15 H14 C	-0.00012	-0.46391	NA	level4	HMDB	NA	HMDB025	NA
C21 H34 C	-3.43E-05	-0.10775	NA	level4	HMDB	NA	HMDB024	NA
C20 H28 N	0.00016	0.35464	NA	level4	HMDB	NA	HMDB025	NA
C28 H53 C	0.001444	2.559278	NA	level4	NA	NA	NA	NA
C26 H28 C	-0.00109	-2.40174	NA	level4	NA	NA	NA	NA
C9 H8 N2	8.24E-05	0.5146	NA	level4	HMDB	NA	HMDB024	NA
C5 H8 N4	-0.00041	-1.72546	NA	level4	HMDB	NA	HMDB001	Organohe
C42 H84 N	0.000597	0.800102	NA	level4	HMDB	NA	HMDB001	Lipids and
C29 H36 C	-0.00078	-1.67263	NA	level4	NA	NA	NA	NA
C6 H6 O3	6.35E-05	0.401659	69.80353	level2	Public	NA	HMDB024	NA
C4 H8 O	9.74E-05	1.351578	NA	level4	HMDB	NA	HMDB000	Organohe
C4 H9 N S	0.000231	2.237259	NA	level4	HMDB	NA	HMDB025	NA
C28 H31 N	0.000944	1.658851	NA	level4	HMDB	NA	HMDB024	NA
C26 H29 F	0.000604	1.437604	NA	level4	HMDB	NA	HMDB025	NA
C11 H15 N	-0.00388	-13.0508	48.4801	level3	mzCloud	NA	NA	NA
C20 H20 C	17.02623	50057.85	19.47715	level4	Public	NA	NA	NA
C21 H24 C	17.02656	43865.67	11.62499	level4	Public	NA	NA	NA
C17 H17 C	17.02808	48365.41	5.188456	level4	Public	C06686	HMDB001	Organohe
C19 H21 N	-0.00012	-0.33141	NA	level4	HMDB	NA	HMDB001	Organohe
C25 H43 N	-0.00052	-0.81172	NA	level4	HMDB	NA	HMDB024	NA
C23 H20 C	0.000619	1.190292	NA	level4	HMDB	NA	HMDB003	Phenylpro
C19 H22 F	-0.00017	-0.4606	NA	level4	HMDB	NA	HMDB001	Organohe
C7 H9 N C	0.000214	1.25034	NA	level4	KEGG	C03972	NA	Organic ac
C8 H8 O3	-18.0104	-118452	83.84954	level2	mzCloud	NA	HMDB003	Benzenoid
C9 H6 O2	0.000215	1.470393	85.41238	level2	mzCloud	C05851	HMDB000	Phenylpro
C57 H100	-0.00219	-2.48328	NA	level4	HMDB	NA	HMDB000	Lipids and
C26 H53 C	-0.00067	-1.2705	NA	level4	NA	NA	NA	NA
C15 H29 N	-0.00026	-0.84411	NA	level4	HMDB	NA	HMDB006	Lipids and
C25 H28 N	0.001229	2.241704	NA	level4	HMDB	NA	HMDB024	NA
C13 H13 N	2.48E-05	0.095855	NA	level4	HMDB	NA	HMDB001	Organohe
C7 H19 N3	0.000188	1.294157	94.77036	level1	BMDB	C00315	HMDB000	Organic ni
C48 H88 N	-0.00056	-0.67228	NA	level4	HMDB	NA	HMDB000	Lipids and
C40 H46 N	0.002117	2.980571	43.56555	level3	mzCloud	NA	NA	NA
C23 H26 N	0.000338	0.975364	NA	level4	HMDB	NA	HMDB025	NA
C30 H33 F	-0.00235	-3.99638	NA	level4	HMDB	NA	HMDB025	NA
C18 H30 C	-0.00019	-0.69084	NA	level4	HMDB	NA	HMDB024	NA
C11 H14 N	0.000184	0.828828	NA	level4	NA	C20843	NA	NA
C30 H33 C	-0.00145	-2.40613	NA	level4	HMDB	NA	HMDB025	NA
C26 H45 N	-5.90E-05	-0.14064	NA	level4	NA	NA	NA	NA
C19 H26 C	-0.00182	-4.9559	NA	level4	HMDB	NA	HMDB024	NA
C15 H17 N	-8.47E-05	-0.25287	NA	level4	NA	C21162	NA	NA
C31 H28 C	-0.00011	-0.17315	NA	level4	NA	NA	NA	NA
C19 H22 C	17.02808	46893.32	5.958211	level4	Public	NA	NA	NA
C10 H12 N	0.000175	0.780138	NA	level4	HMDB	NA	HMDB001	Nucleoside
C17 H18 N	-0.00032	-1.03182	NA	level4	HMDB	NA	HMDB025	NA
C20 H26 C	-1.79E-05	-0.04941	70.26795	level2	Public	NA	HMDB025	NA
C22 H23 N	0.000972	2.576587	NA	level4	HMDB	NA	HMDB025	NA
C34 H41 N	0.00186	2.216199	NA	level4	HMDB	NA	HMDB025	NA
C55 H98 C	-0.0021	-2.45813	NA	level4	HMDB	NA	HMDB001	Lipids and

C10 H12 C	0.000161	1.086182	NA	level4	HMDB	NA	HMDB024	NA
C13 H22 N	-0.00024	-0.65845	NA	level4	NA	C17363	NA	NA
C10 H10 N	0.00026	1.260742	NA	level4	HMDB	NA	HMDB024	NA
C8 H5 N C	0.000189	1.28456	87.93868	level1	Decode	C11129	HMDB006	Organohe
C9 H15 N	0.000232	1.154323	NA	level4	NA	NA	NA	NA
C25 H31 N	0.000525	1.072559	NA	level4	NA	C22046	NA	NA
C26 H40 N	0.002782	4.761279	NA	level4	HMDB	NA	HMDB025	NA
C35 H53 N	-0.00014	-0.20934	15.55775	level4	Public	NA	NA	NA
C47 H66 N	-493.255	-500001	20.60899	level4	Public	NA	NA	NA
C5 H10 O2	-3.31E-05	-0.32415	NA	level4	HMDB	NA	HMDB000	Lipids and
C22 H28 C	8.83E-05	0.227465	NA	level4	HMDB	NA	HMDB025	NA
C12 H10 N	-0.00096	-4.58532	NA	level4	HMDB	NA	HMDB025	NA
C21 H16 F	-0.00111	-2.90793	NA	level4	HMDB	NA	HMDB025	NA
C44 H60 N	-472.231	-500000	37.33589	level4	Public	NA	NA	NA
C12 H6 N4	-0.00088	-4.28238	NA	level4	HMDB	NA	HMDB024	NA
C20 H34 C	-18.0105	-46631.5	3.518625	level4	Public	NA	NA	NA
C22 H22 C	0.000268	0.632312	NA	level4	HMDB	NA	HMDB024	NA
C15 H18 N	-0.00156	-4.45636	NA	level4	HMDB	NA	HMDB024	NA
C15 H21 N	-0.00132	-3.57009	NA	level4	HMDB	NA	HMDB024	NA
C12 H20 N	-0.00032	-1.06412	NA	level4	HMDB	NA	HMDB024	NA
C26 H28 N	-0.00159	-4.30817	NA	level4	HMDB	NA	HMDB001	Benzenoid
C13 H10 N	-0.0002	-0.83067	NA	level4	HMDB	NA	HMDB024	NA
C28 H50 C	-0.00027	-0.60787	NA	level4	NA	NA	NA	NA
C16 H24 C	-1.81E-05	-0.05027	NA	level4	NA	C11649	NA	NA
C33 H54 C	-0.00098	-1.74424	NA	level4	HMDB	NA	HMDB001	Lipids and
C6 H11 Cl	-8.09E-05	-0.24232	NA	level4	NA	C18677	NA	NA
C33 H30 C	-0.00012	-0.21696	NA	level4	NA	C17870	NA	NA
C7 H15 N	-0.00064	-2.8618	NA	level4	HMDB	NA	HMDB025	NA
C16 H16 N	-0.00022	-0.73787	NA	level4	HMDB	NA	HMDB025	NA
C50 H79 C	0.001687	1.938252	NA	level4	NA	NA	NA	NA
C14 H19 N	-1.53E-05	-0.04891	NA	level4	HMDB	NA	HMDB001	Organic o
C11 H20 C	0.000184	0.849483	NA	level4	HMDB	NA	HMDB000	Lipids and
C32 H40 C	0.00175	2.6347	NA	level4	NA	NA	NA	NA
C4 H8 O2	-1.71E-05	-0.19426	NA	level4	HMDB	NA	HMDB024	NA
C18 H20 F	0.000899	2.310466	NA	level4	HMDB	NA	HMDB001	Organohe
C6 H11 N3	0.000118	0.626117	NA	level4	HMDB	NA	HMDB024	NA
C22 H20 C	0.000731	1.773945	1.919102	level4	Public	NA	NA	NA
C22 H36 C	-7.44E-05	-0.22392	NA	level4	HMDB	NA	HMDB026	NA
C19 H14 C	0.000314	0.886026	2.272611	level4	Public	NA	NA	NA
C11 H18 N	-0.00011	-0.39427	NA	level4	HMDB	NA	HMDB025	NA
C13 H19 N	-0.00018	-0.66545	NA	level4	NA	C11085	NA	NA
C8 H7 N	2.16E-05	0.184182	NA	level4	HMDB	NA	HMDB024	NA
C16 H17 N	-3.93E-05	-0.11874	NA	level4	HMDB	NA	HMDB024	NA
C20 H34 C	0.000184	0.544437	NA	level4	HMDB	NA	HMDB000	Lipids and
C31 H54 C	0.001742	3.440426	NA	level4	NA	NA	NA	NA
C32 H50 C	-0.00214	-4.43201	NA	level4	NA	NA	NA	NA
C4 H10 N	-6.28E-05	-0.37588	NA	level4	NA	C17962	NA	FA Fatty a
C15 H12 C	-0.0002	-0.76387	33.38193	level4	Public	C08650	HMDB003	Phenylpro
C18 H19 N	37.95734	121218.6	12.37383	level4	Public	C02717	HMDB002	Benzenoid
C16 H20 N	-0.00104	-3.50593	NA	level4	HMDB	NA	HMDB024	NA
C6 H8 O2	0.000175	1.216004	NA	level4	HMDB	NA	HMDB024	NA
C32 H42 C	-0.00204	-4.29601	NA	level4	NA	NA	NA	NA
C22 H26 C	-6.34E-05	-0.17911	NA	level4	HMDB	NA	HMDB025	NA
C23 H26 C	21.98029	57513.22	10.34049	level4	Public	NA	NA	NA
C24 H33 N	37.95468	95067.09	3.363436	level4	Public	NA	NA	NA
C22 H23 F	0.0012	3.232217	NA	level4	HMDB	NA	HMDB025	NA

C6 H6 N4	-0.00046	-1.99696	NA	level4	HMDB	NA	HMDB024	NA
C12 H18 N	-6.88E-05	-0.25467	NA	level4	HMDB	NA	HMDB001	Benzenoid
C21 H21 N	0.000265	0.663444	NA	level4	KEGG	C09593	NA	Alkaloids
C14 H12 C	0.000111	0.361613	NA	level4	HMDB	NA	HMDB004	Phenylpro
C33 H56 C	0.001668	2.409382	NA	level4	NA	NA	NA	NA
C40 H68 C	-0.0026	-3.59475	NA	level4	HMDB	NA	HMDB025	NA
C7 H16 O3	0.00023	1.555751	NA	level4	HMDB	NA	HMDB025	NA
C15 H24 C	37.95786	104240.6	7.437933	level4	Public	NA	NA	NA
C29 H40 C	-0.00053	-1.0339	NA	level4	NA	NA	NA	NA
C32 H45 N	-0.00205	-3.5141	NA	level4	HMDB	NA	HMDB025	NA
C24 H21 C	0.001086	2.167933	NA	level4	NA	NA	NA	NA
C21 H28 C	-17.0244	-36056.5	1.035506	level4	Public	NA	NA	NA
C16 H14 N	-9.21E-05	-0.32641	NA	level4	HMDB	NA	HMDB024	NA
C15 H14 C	0.000411	1.954759	NA	level4	HMDB	NA	HMDB025	NA
C27 H32 C	-0.00218	-4.49593	NA	level4	HMDB	NA	HMDB025	NA
C44 H56 N	0.000119	0.146918	NA	level4	HMDB	NA	HMDB025	NA
C13 H18 C	3.73E-05	0.156668	NA	level4	HMDB	NA	HMDB025	NA
C11 H16 C	-0.00186	-11.3495	81.84991	level2	mzCloud	C14205	HMDB001	Benzenoid
C41 H69 C	0.00394	4.921697	NA	level4	NA	NA	NA	NA
C15 H10 C	-0.00075	-3.63372	NA	level4	HMDB	NA	HMDB024	NA
C8 H5 F3 (	0.000141	0.809577	NA	level4	HMDB	NA	HMDB024	NA
C31 H41 N	-0.00167	-3.39406	NA	level4	HMDB	NA	HMDB024	NA
C17 H13 C	-4.33E-05	-0.13376	NA	level4	HMDB	NA	HMDB006	Organohe
C6 H8 N2	4.92E-05	0.285897	NA	level4	KEGG	C05565	NA	Organohe
C14 H25 N	9.54E-05	0.262693	NA	level4	KEGG	C05556	NA	NA
C45 H79 C	0.002323	2.865566	NA	level4	NA	NA	NA	NA
C26 H32 N	0.002387	4.930146	NA	level4	HMDB	NA	HMDB025	NA
C19 H19 N	0.001545	4.871086	NA	level4	NA	C13794	NA	NA
C16 H16 N	-5.06E-05	-0.16869	NA	level4	HMDB	NA	HMDB024	NA
C18 H17 N	17.02639	52050.85	3.899672	level4	Public	NA	NA	NA
C12 H14 C	0.000147	0.657388	92.0307	level2	mzCloud	NA	NA	NA
C23 H48 N	0.000239	0.514459	NA	level4	NA	NA	NA	NA
C10 H18 C	3.37E-05	0.166639	47.76915	level3	Public	NA	NA	NA
C20 H30	6.84E-05	0.253177	NA	level4	HMDB	NA	HMDB024	NA
C32 H45 N	-0.00087	-1.54082	NA	level4	HMDB	NA	HMDB001	Organic ac
C13 H21 N	-8.83E-05	-0.27665	NA	level4	HMDB	NA	HMDB006	Organic ac
C3 H9 O6	0.000269	1.561978	76.79565	level1	BMDB	C00093	HMDB000	Lipids and
C15 H17 N	-0.00032	-1.1761	NA	level4	HMDB	NA	HMDB025	NA
C24 H26 C	-0.00078	-2.06894	NA	level4	KEGG	C08732	NA	Flavonoids
C38 H44 N	-0.00289	-4.41072	NA	level4	NA	C16954	NA	NA
C41 H81 N	0.00023	0.362265	NA	level4	HMDB	NA	HMDB000	Lipids and
C20 H29 N	-0.00102	-3.23013	55.19673	level4	Public	NA	NA	NA
C11 H18 C	0.000244	1.342371	NA	level4	HMDB	NA	HMDB003	Lipids and
C20 H25 C	8.10E-05	0.217509	NA	level4	HMDB	NA	HMDB024	NA
C10 H15 N	2.24E-05	0.085873	NA	level4	HMDB	NA	HMDB025	NA
C17 H24 C	17.02462	45747.64	1.359791	level4	Public	NA	NA	NA
C11 H20 N	0.000764	1.712684	NA	level4	KEGG	C00570	NA	Nucleoside
C33 H52 C	-0.00245	-4.03493	NA	level4	NA	NA	NA	NA
C26 H34 F	0.001387	2.729569	NA	level4	HMDB	NA	HMDB026	NA
C20 H37 N	-0.00018	-0.53867	NA	level4	HMDB	NA	HMDB001	Organic ac
C22 H26 C	-0.0002	-0.50271	24.7671	level4	Public	NA	NA	NA
C10 H13 N	-0.00029	-1.06098	NA	level4	NA	C20911	NA	NA
C31 H24 C	-0.00065	-1.46891	NA	level4	HMDB	NA	HMDB025	NA
C16 H14 C	0.000526	2.068947	5.94718	level4	Public	NA	NA	NA
C33 H60 N	0.002269	3.43118	NA	level4	NA	NA	NA	NA
C33 H36 N	0.001028	1.759892	NA	level4	HMDB	NA	HMDB025	NA
C10 H16 N	-0.00027	-0.98002	NA	level4	HMDB	NA	HMDB025	NA

C9 H17 N	21.98203	100323.9	47.93629	level3	Public	NA	HMDB025	NA
C18 H29 N	-0.00028	-0.77547	NA	level4	HMDB	NA	HMDB001	Benzenoid
C6 H10 O3	0.00019	1.171843	NA	level4	NA	C15606	NA	Organic ox
C19 H20 F	-0.00155	-4.05078	NA	level4	HMDB	NA	HMDB025	NA
C26 H37 N	0.0002	0.48633	NA	level4	HMDB	NA	HMDB001	Benzenoid
C14 H18 N	-0.00105	-3.68634	NA	level4	HMDB	NA	HMDB025	NA
C7 H7 N C	-18.0103	-131417	89.22547	level2	Decode	C00568	HMDB000	Benzenoid
C22 H34 N	-0.00164	-2.71018	NA	level4	HMDB	NA	HMDB024	NA
C10 H19 C	-0.00026	-1.09628	NA	level4	NA	C21203	NA	Lipids and
C6 H10 O5	21.98222	135648.5	68.84862	level1	BMDB	C03761	HMDB000	Lipids and
C6 H13 N3	21.98465	125557.9	42.51344	level3	mzCloud	C00327	HMDB000	Organic ac
C18 H14 C	-0.0007	-2.66776	NA	level4	NA	C18260	NA	Benzenoid
C10 H16 N	0.000729	1.911975	NA	level4	NA	C15563	NA	NA
C33 H52 C	0.001061	2.07045	NA	level4	NA	NA	NA	NA
C20 H14 N	-0.00076	-2.29737	NA	level4	HMDB	NA	HMDB025	NA
C23 H32 N	-0.00142	-3.89527	NA	level4	HMDB	NA	HMDB025	NA
C11 H8 N2	8.81E-05	0.440216	NA	level4	HMDB	NA	HMDB025	NA
C8 H7 N C	-7.66E-05	-0.35959	96.99866	level2	mzCloud	NA	HMDB000	Organic ac
C11 H14 N	-9.02E-05	-0.35485	NA	level4	HMDB	NA	HMDB006	Lipids and
C35 H36 N	0.000581	0.981252	NA	level4	HMDB	NA	HMDB025	NA
C14 H16 C	21.9819	67001.75	2.363338	level4	Public	NA	NA	NA
C23 H44 N	-0.00011	-0.26814	NA	level4	NA	NA	NA	NA
C12 H12 C	3.70E-05	0.137197	NA	level4	HMDB	NA	HMDB025	NA
C13 H18 C	21.98106	72760.84	10.81197	level4	Public	NA	NA	NA
C18 H21 F	-1.88E-05	-0.06923	NA	level4	NA	C15463	NA	NA
C24 H40 C	0.002682	4.857476	NA	level4	NA	NA	NA	NA
C5 H14 N	5.98E-06	0.027797	NA	level4	KEGG	C01233	NA	NA
C31 H34 N	-0.00243	-4.73478	NA	level4	NA	C21145	NA	NA
C16 H12 C	17.02673	51277.1	26.46242	level4	Public	NA	NA	NA
C27 H26 N	0.000522	1.039945	NA	level4	HMDB	NA	HMDB026	NA
C14 H15 C	9.74E-05	0.283842	NA	level4	NA	C18888	NA	NA
C27 H29 F	0.000859	2.064472	NA	level4	HMDB	NA	HMDB025	NA
C14 H21 F	-0.00149	-4.72037	NA	level4	NA	NA	NA	NA
C32 H46 C	-0.00225	-3.90996	NA	level4	NA	NA	NA	NA
C22 H41 N	-0.00014	-0.43192	NA	level4	NA	NA	NA	NA
C33 H41 C	-0.00123	-1.90338	NA	level4	HMDB	NA	HMDB025	NA
C23 H39 N	-5.13E-05	-0.14202	66.54933	level2	mzCloud	NA	NA	NA
C19 H16 C	-0.00015	-0.45367	NA	level4	HMDB	NA	HMDB001	Phenylpro
C8 H13 N3	0.000109	0.41388	NA	level4	HMDB	NA	HMDB025	NA
C27 H44 C	4.34E-05	0.108429	47.51009	level3	Public	NA	NA	NA
C18 H19 C	-0.00055	-1.64821	NA	level4	HMDB	NA	HMDB025	NA
C23 H29 N	0.000253	0.571254	NA	level4	KEGG	C02203	NA	NA
C25 H22 N	-0.00089	-1.76552	NA	level4	HMDB	NA	HMDB026	NA
C29 H29 F	-0.00124	-2.12661	NA	level4	HMDB	NA	HMDB024	NA
C12 H22 C	-8.90E-05	-0.24846	NA	level4	HMDB	NA	HMDB025	NA

C27 H30 I	0.001199	1.830311	NA	level4	HMDB	NA	HMDB024	NA
C22 H22 N	0.001951	4.578665	NA	level4	KEGG	C02811	NA	PK Polyke
C24 H38 C	8.77E-05	0.234204	NA	level4	HMDB	NA	HMDB000	Lipids and
C16 H27 N	-0.00051	-1.50125	NA	level4	HMDB	NA	HMDB025	NA
C15 H17 I	-0.00038	-0.98678	NA	level4	HMDB	NA	HMDB024	NA
C21 H41 N	0.00043	0.798114	NA	level4	HMDB	NA	HMDB024	NA
C18 H24 N	-0.00141	-2.99548	NA	level4	HMDB	NA	HMDB024	NA
C36 H60 C	-0.00205	-3.22859	NA	level4	NA	NA	NA	NA
C9 H14 O	-9.76E-05	-0.41706	NA	level4	HMDB	NA	HMDB025	NA
C28 H23 N	-0.00032	-0.68858	0.322868	level4	Public	NA	NA	NA
C12 H8 N2	0.000175	0.893883	NA	level4	NA	C21477	NA	NA
C23 H34 N	0.000415	0.922544	NA	level4	HMDB	NA	HMDB025	NA
C9 H6 N2	5.48E-05	0.314959	NA	level4	HMDB	NA	HMDB024	NA
C25 H35 N	0.000567	1.18731	NA	level4	NA	NA	NA	NA
C32 H45 N	0.003735	6.726649	35.3574	level3	mzCloud	NA	NA	NA
C9 H19 I	-0.00091	-3.57356	NA	level4	HMDB	NA	HMDB024	NA
C13 H23 N	0.000192	0.797499	NA	level4	NA	C10869	NA	Alkaloids
C12 H24 C	-18.0103	-89971.8	89.00048	level1	Decode	C02679	HMDB000	Lipids and
C16 H32 C	-0.00021	-0.76086	91.43554	level2	mzCloud	C18218	HMDB000	Lipids and
C20 H32 C	17.02647	53168.65	40.36439	level4	Public	NA	NA	NA
C19 H24 N	-0.00015	-0.36073	NA	level4	HMDB	NA	HMDB025	NA
C40 H52 C	0.002023	3.48504	NA	level4	HMDB	NA	HMDB025	NA
C25 H35 N	-1.27E-05	-0.02889	NA	level4	HMDB	NA	HMDB024	NA
C5 H9 N C	0.000147	1.276615	89.70637	level1	BMDB	C00148	HMDB000	Organic ac
C8 H16 O	0.000226	1.411464	NA	level4	HMDB	NA	HMDB024	NA
C24 H31 F	-18.0083	-44772.2	48.40027	level3	mzCloud	NA	NA	NA
C34 H60 C	0.000328	0.581477	NA	level4	NA	NA	NA	NA
C18 H35 N	-3.73E-06	-0.01254	NA	level4	HMDB	NA	HMDB025	NA
C3 H6 Cl N	0.000171	1.134414	NA	level4	HMDB	NA	HMDB024	NA
C16 H10 C	-6.70E-05	-0.21345	NA	level4	HMDB	NA	HMDB025	NA
C29 H39 N	0.000308	0.710971	NA	level4	HMDB	NA	HMDB025	NA
C9 H9 N	0.00018	1.372821	19.16199	level4	Public	NA	HMDB024	NA
C18 H24 C	3.84E-05	0.126312	31.23647	level4	Public	NA	NA	NA
C19 H22 C	37.95726	120824.6	17.67463	level4	Public	NA	NA	NA
C14 H25 C	0.000784	1.998536	NA	level4	HMDB	NA	HMDB025	NA
C10 H18 C	37.95682	246255.8	3.799937	level4	Public	C09871	HMDB000	Lipids and
C18 H28 C	-1.17E-05	-0.03796	NA	level4	NA	NA	NA	NA
C18 H15 C	-9.17E-05	-0.28109	74.82617	level2	Public	C14235	HMDB025	NA
C20 H22 C	-9.79E-05	-0.23195	41.73067	level4	Public	NA	NA	NA
C25 H29 N	0.000711	1.41359	NA	level4	HMDB	NA	HMDB025	NA
C26 H30 C	0.001512	2.748011	NA	level4	HMDB	NA	HMDB024	NA
C43 H84 A	0.002653	2.850775	NA	level4	NA	NA	NA	NA
C17 H16 C	0.000558	1.766019	NA	level4	NA	C14969	NA	NA
C10 H11 N	0.000188	0.777878	NA	level4	KEGG	C04204	NA	NA
C40 H52 C	0.000278	0.491839	NA	level4	HMDB	NA	HMDB000	Lipids and
C28 H44 C	17.02628	32474.41	23.38305	level4	Public	NA	NA	NA
C8 H14 O	0.000263	1.51352	31.23998	level3	mzCloud	NA	HMDB003	Lipids and
C2 H7 N3	9.91E-05	1.356973	NA	level4	HMDB	NA	HMDB000	Organic ni
C26 H52 N	0.000718	1.420127	NA	level4	HMDB	NA	HMDB001	Lipids and
C25 H39 N	0.00021	0.466606	NA	level4	KEGG	C08671	NA	Alkaloids
C27 H44 C	0.000695	1.356274	NA	level4	NA	NA	NA	NA

C24 H23 N	-0.00105	-2.21113	NA	level4	HMDB	NA	HMDB024	NA
C10 H19 C	0.001451	4.869386	NA	level4	HMDB	NA	HMDB025	NA
C18 H21 N	-0.00014	-0.42391	NA	level4	NA	C12237	NA	NA
C21 H23 F	0.000772	2.150669	NA	level4	HMDB	NA	HMDB025	NA
C18 H23 N	-0.00036	-1.18534	NA	level4	HMDB	NA	HMDB001	Benzenoid
C11 H18 N	0.000207	1.068225	NA	level4	HMDB	NA	HMDB024	NA
C15 H26 C	7.80E-05	0.306693	NA	level4	NA	C18538	NA	NA
C10 H17 N	0.000244	1.223703	54.96364	level4	Public	NA	NA	NA
C26 H36 C	0.000546	1.146105	17.25894	level4	Public	NA	NA	NA
C22 H44 C	8.08E-05	0.237373	68.19001	level2	mzCloud	C08281	HMDB000	Lipids and
C15 H26 C	0.000142	0.597173	26.40717	level4	Public	NA	NA	NA
C21 H38 C	5.89E-05	0.159002	NA	level4	HMDB	NA	HMDB025	NA
C28 H38 N	0.001802	3.864944	NA	level4	HMDB	NA	HMDB024	NA
C12 H21 N	-6.90E-05	-0.20589	NA	level4	HMDB	NA	HMDB025	NA
C24 H21 N	0.001657	4.369671	NA	level4	HMDB	NA	HMDB025	NA
C14 H21 N	-0.00015	-0.51825	NA	level4	HMDB	NA	HMDB025	NA
C19 H38 C	-3.69E-05	-0.12357	NA	level4	HMDB	NA	HMDB000	Lipids and
C14 H20 N	0.000127	0.480123	30.08134	level4	Public	NA	NA	NA
C34 H48 N	-0.00269	-3.84062	NA	level4	HMDB	NA	HMDB025	NA
C20 H18 N	-0.00112	-3.21063	NA	level4	HMDB	NA	HMDB025	NA
C15 H15 F	0.000105	0.367722	NA	level4	HMDB	NA	HMDB026	NA
C22 H26 C	17.02613	42335.88	11.51922	level4	Public	NA	NA	NA
C10 H12 C	-0.00213	-10.8415	60.81153	level2	mzCloud	C16778	NA	NA
C13 H14 C	0.000417	1.493482	NA	level4	HMDB	NA	HMDB025	NA
C12 H14 N	0.000114	0.486829	NA	level4	HMDB	NA	HMDB025	NA
C25 H46 N	-0.00242	-3.21871	5.530332	level4	Public	NA	NA	NA
C27 H41 N	0.001972	3.269439	NA	level4	HMDB	NA	HMDB025	NA
C13 H22 N	0.000738	2.435292	35.16182	level3	mzCloud	C18659	HMDB003	Benzenoid
C48 H91 C	-0.0003	-0.30023	NA	level4	NA	NA	NA	NA
C52 H86 C	-0.00355	-4.39598	NA	level4	NA	NA	NA	NA
C11 H19 N	0.001301	4.74699	NA	level4	HMDB	NA	HMDB024	NA
C42 H83 N	0.000166	0.255988	NA	level4	HMDB	NA	HMDB000	Lipids and
C34 H56 C	-0.00111	-2.03154	NA	level4	NA	NA	NA	NA
C27 H31 N	-0.00124	-2.55887	NA	level4	HMDB	NA	HMDB024	NA
C22 H37 N	0.000132	0.310029	NA	level4	NA	NA	NA	NA
C15 H12 C	0.000211	0.941857	63.96118	level2	mzCloud	C00766	NA	NA
C22 H32 C	-2.06E-05	-0.05053	12.8065	level4	Public	NA	NA	NA
C29 H48 C	0.000702	1.580756	NA	level4	KEGG	C08631	NA	Terpenoid
C30 H39 F	-0.00059	-1.09485	NA	level4	HMDB	NA	HMDB025	NA
C14 H10 C	0.001503	4.725803	NA	level4	HMDB	NA	HMDB024	NA
C8 H8 O3	0.00017	1.119392	65.29178	level2	mzCloud	C00755	HMDB001	Benzenoid
C27 H45 N	-0.00028	-0.67038	NA	level4	HMDB	NA	HMDB025	NA
C22 H22 F	-0.00142	-3.97244	NA	level4	HMDB	NA	HMDB001	Benzenoid
C19 H24 C	37.95737	120054.7	8.839552	level4	Public	NA	NA	NA
C27 H50 N	0.000562	0.932338	NA	level4	NA	NA	NA	NA
C13 H8 N2	-3.54E-06	-0.01582	NA	level4	NA	C21442	NA	NA
C38 H46 C	0.00283	4.607107	NA	level4	NA	NA	NA	NA
C8 H4 O3	0.000147	0.996295	NA	level4	HMDB	NA	HMDB025	NA
C17 H18 C	17.02671	48633.83	14.59762	level4	Public	NA	NA	NA
C7 H9 N C	0.000224	1.819808	NA	level4	HMDB	NA	HMDB024	NA
C14 H12 N	0.000183	0.715118	NA	level4	HMDB	NA	HMDB024	NA
C19 H21 N	-0.00143	-3.77373	NA	level4	HMDB	NA	HMDB025	NA
C13 H22	0.000184	1.032238	NA	level4	NA	NA	NA	NA
C22 H20 C	0.000314	0.5484	NA	level4	NA	NA	NA	NA
C24 H24 C	0.000733	1.408434	NA	level4	NA	NA	NA	NA
C21 H38 C	-8.64E-05	-0.26802	NA	level4	NA	NA	NA	NA

C8 H8 O6	-0.00051	-2.19771	NA	level4	HMDB	NA	HMDB012	Organic ac
C10 H14 N	0.000224	1.151581	NA	level4	HMDB	NA	HMDB024	NA
C26 H46 C	-0.00012	-0.31668	NA	level4	NA	NA	NA	NA
C19 H16 C	-0.00112	-2.95178	NA	level4	HMDB	NA	HMDB024	NA
C21 H34 C	17.02643	53499.18	25.97889	level4	Public	NA	NA	NA
C20 H24 C	37.95395	100900.5	20.63749	level4	Public	NA	NA	NA
C42 H45 N	0.001911	2.733061	NA	level4	KEGG	C09995	NA	NA
C12 H15 N	0.000122	0.513507	31.27554	level4	Public	NA	NA	NA
C12 H13 N	0.000168	0.767285	NA	level4	HMDB	NA	HMDB001	Organohe
C6 H7 N C	0.000109	0.627751	NA	level4	HMDB	NA	HMDB025	NA
C12 H18 N	0.000563	1.536966	NA	level4	HMDB	NA	HMDB025	NA
C12 H16 C	-6.33E-05	-0.28259	53.74118	level3	Public	NA	NA	NA
C12 H12 C	17.02664	72125.84	5.176167	level4	Public	NA	NA	NA
C26 H17 N	-0.00197	-4.84213	NA	level4	HMDB	NA	HMDB025	NA
C30 H39 N	-0.00178	-2.48775	NA	level4	KEGG	C04489	NA	Lipids and
C18 H34 C	1.27E-05	0.047885	NA	level4	HMDB	NA	HMDB025	NA
C10 H16 C	0.000216	1.283538	NA	level4	HMDB	NA	HMDB024	NA
C22 H36 C	-0.00012	-0.32377	41.7732	level4	Public	NA	NA	NA
C9 H8 N2	3.52E-05	0.183456	NA	level4	HMDB	NA	HMDB024	NA
C19 H28 C	-0.00035	-1.08493	NA	level4	HMDB	NA	HMDB025	NA
C39 H76 N	-5.35E-05	-0.07455	NA	level4	NA	NA	NA	NA
C17 H28 C	7.78E-05	0.27759	NA	level4	HMDB	NA	HMDB024	NA
C27 H33 N	0.000742	1.355408	NA	level4	HMDB	NA	HMDB006	Organic ox
C22 H32 C	37.95433	89470.68	16.10945	level4	Public	NA	NA	NA
C10 H10	0.000148	1.140796	NA	level4	HMDB	NA	HMDB025	NA
C30 H48 C	-0.00072	-1.29964	NA	level4	HMDB	NA	HMDB000	Lipids and
C31 H29 N	0.001618	2.700713	NA	level4	HMDB	NA	HMDB025	NA
C14 H23 N	0.000264	1.114807	NA	level4	HMDB	NA	HMDB025	NA
C16 H22 N	9.22E-05	0.272623	NA	level4	HMDB	NA	HMDB024	NA
C12 H23 B	-0.00029	-1.04551	NA	level4	NA	NA	NA	NA
C45 H72 N	0.002607	3.189385	NA	level4	NA	NA	NA	NA
C46 H87 N	-0.00018	-0.20477	NA	level4	NA	NA	NA	NA
C27 H38 C	0.000798	1.804341	NA	level4	HMDB	NA	HMDB025	NA
C33 H63 N	0.000759	1.455943	NA	level4	NA	NA	NA	NA
C33 H61 N	-0.0026	-3.86721	NA	level4	NA	C11610	NA	NA
C16 H30 C	-0.00029	-0.96601	NA	level4	HMDB	NA	HMDB025	NA
C7 H8 O4	0.000246	1.577521	NA	level4	KEGG	C04112	NA	NA
C7 H11 N3	-0.0002	-0.8422	NA	level4	HMDB	NA	HMDB025	NA
C17 H17 N	0.001616	4.682499	NA	level4	NA	C17757	NA	Amino aci
C8 H10 O6	-0.00016	-0.67128	NA	level4	HMDB	NA	HMDB024	Organic ac
C15 H13 C	0.000222	0.684077	NA	level4	HMDB	NA	HMDB025	NA
C6 H15 N	0.000223	1.494183	NA	level4	KEGG	C06771	NA	Organic ni
C24 H33 N	2.11E-05	0.049321	NA	level4	HMDB	NA	HMDB001	Benzenoid
C28 H36 N	17.02756	36676.19	4.904605	level4	Public	NA	NA	NA
C17 H20 N	0.001061	3.399714	NA	level4	HMDB	NA	HMDB025	NA
C12 H22 C	0.001393	4.734715	NA	level4	NA	NA	NA	NA
C10 H15 N	0.000282	1.322451	42.38319	level3	Public	NA	NA	NA
C15 H12 C	0.000217	0.845781	15.42374	level4	Public	NA	NA	NA
C34 H56 C	0.001765	2.688775	NA	level4	NA	NA	NA	NA
C36 H64 C	-0.00225	-2.8726	NA	level4	NA	NA	NA	NA
C32 H53 N	0.000805	1.221268	NA	level4	NA	NA	NA	NA
C11 H14 C	0.001229	4.743912	NA	level4	HMDB	NA	HMDB025	NA

C43 H78 N	-0.00015	-0.20072	NA	level4	NA	NA	NA	NA
C16 H23 N	1.47E-05	0.045898	NA	level4	HMDB	NA	HMDB025	NA
C20 H28 C	-0.00019	-0.57587	12.97598	level4	Public	NA	NA	NA
C31 H37 N	0.002222	3.972223	NA	level4	HMDB	NA	HMDB025	NA
C9 H11 N	0.00017	1.13738	NA	level4	HMDB	NA	HMDB024	NA
C21 H27 N	-0.00015	-0.46718	NA	level4	HMDB	NA	HMDB025	NA
C11 H13 N	0.000216	0.861581	NA	level4	HMDB	NA	HMDB024	NA
C13 H8 O2	0.000234	1.191521	NA	level4	HMDB	NA	HMDB025	NA
C10 H13 N	0.001416	4.735281	NA	level4	HMDB	NA	HMDB000	Nucleosid
C43 H74 N	-0.00078	-1.01884	NA	level4	NA	NA	NA	NA
C15 H19 N	1.89E-05	0.052917	NA	level4	HMDB	NA	HMDB003	Organic o
C23 H33 N	4.33E-06	0.010337	NA	level4	HMDB	NA	HMDB024	NA
C10 H9 N	0.000147	1.025029	86.49986	level2	mzCloud	NA	HMDB003	Organohe
C6 H15 N2	-0.00011	-0.48353	NA	level4	HMDB	NA	HMDB025	NA
C17 H17 N	0.000615	2.449262	NA	level4	HMDB	NA	HMDB006	Organohe
C19 H28 C	0.006104	20.06611	54.68774	level3	mzCloud	NA	NA	NA
C31 H50 C	21.98544	37753.59	30.14283	level4	Public	NA	NA	NA
C19 H16 C	0.001459	4.277798	NA	level4	HMDB	NA	HMDB024	NA
C11 H18 C	0.000638	2.170237	NA	level4	NA	NA	NA	NA
C19 H17 N	-0.00069	-2.13412	NA	level4	HMDB	NA	HMDB025	NA
C17 H20 C	0.000846	2.51756	NA	level4	HMDB	NA	HMDB025	NA
C11 H15 N	-8.72E-05	-0.29354	88.60569	level2	mzCloud	NA	HMDB000	Nucleosid
C8 H15 N	0.00011	0.635549	81.07651	level1	Decode	NA	HMDB000	Organic a
C9 H16	0.000146	1.174299	NA	level4	HMDB	NA	HMDB025	NA
C17 H31 C	-0.0011	-1.97684	NA	level4	NA	C19774	NA	NA
C28 H50 C	-6.20E-05	-0.12857	NA	level4	NA	NA	NA	NA
C16 H18 N	-0.00026	-0.90603	NA	level4	HMDB	NA	HMDB024	NA
C36 H56 C	0.001499	2.370102	63.85541	level2	Public	NA	NA	NA
C19 H16 F	0.000683	1.603206	NA	level4	HMDB	NA	HMDB025	NA
C24 H45 N	-5.40E-05	-0.13658	NA	level4	NA	NA	NA	NA
C14 H11 F	0.000167	0.535137	NA	level4	HMDB	NA	HMDB024	NA
C48 H86 N	-0.00075	-0.89835	NA	level4	HMDB	NA	HMDB000	Lipids and
C28 H46 C	-8.91E-05	-0.18634	NA	level4	NA	NA	NA	NA
C17 H18 F	0.00128	4.062926	NA	level4	HMDB	NA	HMDB025	NA
C30 H38 C	0.002144	3.536689	NA	level4	KEGG	C08750	NA	Terpenoid
C14H14Cl2	-0.00139	-3.85988	NA	level4	NA	C11219	NA	NA
C22 H30 N	-0.00111	-2.60799	NA	level4	HMDB	NA	HMDB025	NA
C22 H26 C	21.97959	48826.98	22.94062	level4	Public	NA	NA	NA
C28 H50 C	-0.00132	-2.48725	NA	level4	NA	NA	NA	NA
C7 H6 N2	0.000254	1.529237	NA	level4	HMDB	NA	HMDB024	NA
C10 H17 N	0.000219	0.886851	NA	level4	HMDB	NA	HMDB000	Lipids and
C20 H29 N	0.000453	1.102364	NA	level4	HMDB	NA	HMDB025	NA
C22 H32 F	0.001149	3.137162	NA	level4	NA	C15322	NA	NA
C16 H16 N	-0.00023	-0.70299	NA	level4	HMDB	NA	HMDB024	NA
C25 H45 N	7.57E-05	0.185956	NA	level4	NA	NA	NA	NA
C21 H41 N	8.07E-05	0.142339	NA	level4	KEGG	C03755	NA	NA
C20 H40 N	-0.00129	-2.95513	NA	level4	NA	NA	NA	NA
C26 H28 C	2.33E-05	0.041311	48.74952	level3	Public	NA	NA	NA
C9 H13 N	3.45E-05	0.228501	NA	level4	HMDB	NA	HMDB025	NA
C18 H30 C	-4.01E-06	-0.01028	NA	level4	NA	NA	NA	NA
C24 H23 C	-0.00133	-2.56227	NA	level4	NA	C12642	NA	Flavonoids
C21 H29 F	0.001542	4.234023	NA	level4	HMDB	NA	HMDB025	NA
C10 H17 N	-0.00038	-1.28202	NA	level4	NA	NA	NA	NA
C35 H56 C	0.001556	2.445767	NA	level4	NA	NA	NA	NA
C18 H39 N	21.98248	37821.19	14.39182	level4	Public	NA	NA	NA

C7 H5 N C	0.000235	1.554462	NA	level4	HMDB	NA	HMDB024	NA
C30 H52 N	-0.00281	-4.22211	NA	level4	NA	NA	NA	NA
C30 H22 N	-0.00129	-2.49408	NA	level4	NA	C12116	NA	NA
C16 H19 N	-0.00027	-0.75941	NA	level4	HMDB	NA	HMDB001	Organic ac
C21 H30 C	-3.68E-05	-0.10618	NA	level4	HMDB	NA	HMDB025	NA
C18 H14 N	-0.0015	-4.70508	NA	level4	HMDB	NA	HMDB025	NA
C5 H9 N C	0.000189	1.284083	NA	level4	HMDB	NA	HMDB024	NA
C26 H36 N	0.00026	0.569379	NA	level4	HMDB	NA	HMDB025	NA
C20 H32 C	-18.013	-51140.7	58.61671	level3	mzCloud	NA	NA	NA
C30 H40 C	21.9841	47349.66	40.51137	level4	Public	NA	NA	NA
C16 H22 N	-2.74E-05	-0.08199	NA	level4	HMDB	NA	HMDB025	NA
C18 H32 N	-36.0194	-82574.3	25.3959	level4	Public	NA	NA	NA
C25 H26 N	0.002049	4.807985	NA	level4	HMDB	NA	HMDB025	NA
C26 H43 C	0.000725	1.555095	NA	level4	NA	NA	NA	NA
C35 H44 C	0.0017	2.360348	9.388341	level4	Public	NA	NA	NA
C17 H21 N	0.000487	1.524913	NA	level4	HMDB	NA	HMDB026	NA
C21 H41 N	0.000103	0.238403	NA	level4	NA	NA	NA	NA
C22 H28 N	0.000906	2.263164	NA	level4	KEGG	C04318	NA	NA
C25 H34 C	0.000116	0.279324	NA	level4	HMDB	NA	HMDB025	NA
C40 H79 N	0.000102	0.164573	NA	level4	HMDB	NA	HMDB000	Lipids and
C12 H10 C	17.02672	78083.48	14.89031	level4	Public	NA	NA	NA
C18 H24 I	0.003401	4.300054	NA	level4	HMDB	NA	HMDB024	NA
C15 H21 N	-9.07E-05	-0.32968	NA	level4	HMDB	NA	HMDB001	Organohe
C19 H17 C	0.000314	0.935089	NA	level4	NA	C18461	NA	NA
C26 H22 N	-0.00029	-0.74746	NA	level4	HMDB	NA	HMDB025	NA
C20 H28 C	17.02556	53843.62	28.01198	level4	Public	NA	NA	NA
C11 H11 I	0.000168	0.534315	NA	level4	HMDB	NA	HMDB025	NA
C11 H9 N	0.000346	1.470678	NA	level4	NA	C11468	NA	NA
C6 H9 N3	-7.65E-05	-0.40884	NA	level4	HMDB	NA	HMDB024	NA
C15 H20 N	0.001117	2.805776	NA	level4	KEGG	C04425	NA	NA
C18 H23 N	0.000304	0.913316	NA	level4	HMDB	NA	HMDB025	NA
C20 H36 N	-21.9785	-46149.3	5.888322	level4	Public	NA	NA	NA
C18 H12 C	0.000145	0.406225	NA	level4	NA	NA	NA	NA
C20 H23 N	-0.00028	-0.81037	47.46853	level4	Public	C09549	HMDB003	Alkaloids a
C21 H22 C	-0.00395	-11.1511	45.88832	level3	mzCloud	C16417	HMDB003	Phenylpro
C10 H17 N	-0.00026	-0.75625	NA	level4	HMDB	NA	HMDB025	NA
C2 H4 O3	0.000121	1.596406	NA	level4	HMDB	NA	HMDB000	Organic ac
C20 H36 C	17.02643	47792.64	60.29043	level2	mzCloud	NA	NA	NA
C10 H18 N	17.02801	56180.81	4.97623	level4	Public	NA	NA	NA
C23 H34 C	-0.00031	-0.79832	NA	level4	HMDB	NA	HMDB025	NA
C28 H31 C	-0.0005	-1.08866	NA	level4	HMDB	NA	HMDB025	NA
C6 H10 O4	0.000191	1.310257	87.34324	level2	mzCloud	NA	HMDB003	Lipids and
C26 H36 F	-0.00169	-3.92875	NA	level4	HMDB	NA	HMDB025	NA
C42 H59 N	-0.00093	-1.21476	NA	level4	HMDB	NA	HMDB024	NA
C15 H14 C	0.000124	0.375941	NA	level4	HMDB	NA	HMDB024	NA
C10 H16 N	6.29E-05	0.178665	NA	level4	HMDB	NA	HMDB025	NA
C15 H22 C	17.02483	51569.85	3.676006	level4	Public	NA	NA	NA
C28 H44 C	0.002242	4.871491	NA	level4	NA	NA	NA	NA
C10 H15 N	0.000294	1.49027	NA	level4	HMDB	NA	HMDB006	Benzenoid
C11 H13 N	-0.00105	-4.87645	NA	level4	HMDB	NA	HMDB025	NA
C20 H26 N	-0.00031	-0.91247	NA	level4	NA	C21491	NA	NA
C23 H33 N	0.001238	2.695121	NA	level4	HMDB	NA	HMDB024	NA
C6 H10 N2	0.000161	1.13257	40.85065	level4	Public	C06231	HMDB024	Organic ac
C4 H4 N2	-1.53E-05	-0.15904	NA	level4	HMDB	NA	HMDB025	NA
C13 H18 N	0.000242	0.769828	NA	level4	HMDB	NA	HMDB025	NA
C34 H38 N	-0.00244	-3.86651	NA	level4	HMDB	NA	HMDB025	NA

C18 H21 N	17.02516	54714.54	5.64284	level4	Public	NA	NA	NA	
H2 O4 S	-1.26E-05	-0.12851	NA	level4	HMDB	NA	HMDB000	Homogen	
C32 H51 N	0.000333	0.519311	NA	level4	HMDB	NA	HMDB000	Organic ni	
C25 H32 N	0.000343	0.726348	NA	level4	HMDB	NA	HMDB006	Lipids and	
C11 H21 N	-0.00131	-4.29393	NA	level4	NA	C18469	NA	NA	
C10 H10 C	0.000171	1.055182	72.46033	level2	mzCloud	C10475	HMDB003	Phenylpro	
C18 H20 F	0.000451	1.263859	NA	level4	HMDB	NA	HMDB025	NA	
C11 H12 C	2.93E-05	0.141003	NA	level4	HMDB	NA	HMDB002	Benzenoid	
C44 H86 N	-0.00021	-0.27145	NA	level4	HMDB	NA	HMDB000	Lipids and	
C8 H8 N2	0.000269	1.495581	74.78649	level1	Decode	NA	HMDB005	Organic ac	
C22 H35 N	0.000126	0.348136	NA	level4	HMDB	NA	HMDB000	Organic ac	
C20 H20 C	17.02646	52528.71	33.5258	level4	Public	NA	NA	NA	
C11 H10 C	0.000213	1.120108	NA	level4	HMDB	NA	HMDB024	NA	
C6 H13 N	0.000178	1.20738	NA	level4	HMDB	NA	HMDB025	NA	
C26 H31 C	-0.00056	-1.05794	NA	level4	HMDB	NA	HMDB001	Organohe	
C10 H21 N	0.000374	1.70754	NA	level4	HMDB	NA	HMDB001	Organohe	
C12 H15 N	6.55E-05	0.232989	NA	level4	NA	C21883	NA	NA	
C5 H10 N6	0.000603	3.241471	NA	level4	NA	C19409	NA	NA	
C14 H22 N	0.000194	0.566588	NA	level4	HMDB	NA	HMDB025	NA	
C10 H23 N	0.000194	0.830381	NA	level4	NA	NA	NA	NA	
C38 H54 C	0.001755	2.15551	NA	level4	NA	C17513	NA	Terpenoid	
C16 H18 N	-0.00013	-0.50193	NA	level4	HMDB	NA	HMDB024	NA	
C12 H19 N	0.000318	1.648352	NA	level4	HMDB	NA	HMDB024	NA	
C18 H32 C	-0.00104	-2.12568	NA	level4	HMDB	NA	HMDB024	NA	
C22 H26 C	21.98011	56917.78	13.83858	level4	Public	NA	NA	NA	
C28 H43 N	0.000154	0.349357	NA	level4	NA	NA	NA	NA	
C26 H36 C	0.000248	0.602392	12.42313	level4	Public	NA	NA	NA	
C7 H8 N2	0.000204	1.342733	94.72019	level1	Decode	C05842	HMDB000	Organohe	
C13 H9 F N	0.000508	1.953331	NA	level4	HMDB	NA	HMDB024	NA	
C13 H18 C	0.000218	0.913816	11.55045	level4	Public	NA	NA	NA	
C4 H11 O7	0.000245	1.213451	NA	level4	NA	C21294	NA	NA	
C16 H20 I3	0.000635	0.850245	NA	level4	HMDB	NA	HMDB024	NA	
C16 H20 N	-0.00094	-2.67375	NA	level4	HMDB	NA	HMDB006	Organic ac	
C18 H25 C	0.000804	2.125301	NA	level4	HMDB	NA	HMDB024	NA	
C28 H28 C	-0.00029	-0.67384	NA	level4	HMDB	NA	HMDB025	NA	
C5 H3 N5	-0.00023	-1.36348	NA	level4	HMDB	NA	HMDB025	NA	
C10 H15 N	-0.00204	-10.3742	40.60261	level3	mzCloud	C05588	HMDB006	Benzenoid	
C12 H19 N	3.43E-05	0.127605	NA	level4	HMDB	NA	HMDB026	NA	
C21 H13 F	0.001627	3.929919	NA	level4	HMDB	NA	HMDB025	NA	
C27 H41 N	-0.00016	-0.37491	NA	level4	HMDB	NA	HMDB025	NA	
C20 H28 C	21.98043	69513.49	38.81163	level3	mzCloud	NA	NA	NA	
C28 H43 N	0.000841	1.248697	NA	level4	KEGG	C08442	NA	NA	
C33 H60 C	-0.00014	-0.22993	NA	level4	HMDB	NA	HMDB001	Organic ox	
C15 H31 A	0.001335	3.996283	NA	level4	NA	NA	NA	NA	
C10 H16 C	0.000141	0.928536	NA	level4	HMDB	NA	HMDB025	NA	
C28 H31 N	-1.89E-05	-0.04584	NA	level4	HMDB	NA	HMDB025	NA	
C15 H26 C	0.000234	0.981536	NA	level4	HMDB	NA	HMDB025	NA	
C10 H10 N	0.000153	0.966584	NA	level4	HMDB	NA	HMDB024	NA	
C19 H34 C	21.98189	67378.33	56.54499	level3	Public	NA	HMDB003	Lipids and	
C22 H40 C	-2.14E-05	-0.06066	NA	level4	NA	NA	NA	NA	
C23 H22 C	-0.00044	-1.02885	NA	level4	NA	NA	NA	NA	
C16 H16 C	-0.00288	-8.17133	42.25031	level3	mzCloud	C11584	HMDB024	Phenylpro	

C14 H26 C	0.001423	4.903874	NA	level4	NA	NA	NA	NA
C19 H20 C	21.98361	70429.56	45.98674	level4	Public	NA	NA	NA
C8 H8 O2	8.94E-05	0.657381	51.75661	level3	mzCloud	C01454	HMDB002	Benzenoid
C25 H32 C	21.98114	46159.03	10.2717	level4	Public	NA	NA	NA
C3 H7 N C	0.0002	1.460887	NA	level4	HMDB	NA	HMDB025	NA
C11 H13 N	0.000987	3.995755	NA	level4	HMDB	NA	HMDB024	NA
C20 H23 C	0.001195	3.619213	NA	level4	NA	C15073	NA	NA
C20 H38 C	0.000179	0.42418	NA	level4	HMDB	NA	HMDB025	NA
C11 H9 N	0.000222	1.186029	94.33101	level1	Decode	NA	HMDB000	Organohe
C22 H27 N	21.98035	65184.09	2.237233	level4	Public	NA	NA	NA
C24 H40 C	-0.00027	-0.63293	NA	level4	NA	NA	NA	NA
C21 H30 C	-0.00026	-0.72401	86.24871	level2	mzCloud	C00735	HMDB000	Lipids and
C13 H16 N	-2.28E-05	-0.077	NA	level4	KEGG	C03995	NA	NA
C16 H24 C	17.02647	54544.51	32.08859	level4	Public	NA	NA	NA
C18 H27 C	-0.00157	-4.38503	NA	level4	HMDB	NA	HMDB025	NA
C44 H80 N	-0.00063	-0.81893	NA	level4	HMDB	NA	HMDB000	Lipids and
C10 H9 N	0.000172	0.983103	NA	level4	HMDB	NA	HMDB025	NA
C12 H13 N	-8.59E-05	-0.34216	NA	level4	HMDB	NA	HMDB025	NA
C20 H24 N	-0.00152	-3.8323	NA	level4	HMDB	NA	HMDB024	NA
C24 H32 C	21.98038	57206.31	9.855574	level4	Public	NA	NA	NA
C11 H11 C	-0.00101	-3.74232	NA	level4	HMDB	NA	HMDB025	NA
C22 H45 N	-1.51E-05	-0.04251	NA	level4	HMDB	NA	HMDB024	NA
C25 H26 C	-0.00223	-4.43962	NA	level4	HMDB	NA	HMDB025	NA
C25 H29 N	0.000281	0.423976	NA	level4	HMDB	NA	HMDB024	NA
C24 H19 F	-0.00081	-2.01704	NA	level4	HMDB	NA	HMDB025	NA
C22 H23 F	0.000153	0.421587	NA	level4	HMDB	NA	HMDB024	NA
C12 H17 N	0.00012	0.450875	NA	level4	HMDB	NA	HMDB006	Benzenoid
C19 H30 C	-0.00136	-4.22593	NA	level4	HMDB	NA	HMDB025	NA
C28 H48 C	0.000401	0.962232	NA	level4	HMDB	NA	HMDB000	Lipids and
C4 H6 N2	0.000124	1.088958	85.99433	level2	mzCloud	C02777	NA	NA
C30 H30 C	0.000189	0.307048	NA	level4	NA	NA	NA	NA
C9 H15 N	3.15E-05	0.170414	NA	level4	HMDB	NA	HMDB024	NA
C17 H27 N	-0.00012	-0.36447	14.64497	level4	Public	NA	NA	NA
C17 H21 N	0.004316	9.462469	33.47967	level3	mzCloud	C00061	HMDB000	Nucleoside
C8 H12 O4	0.000139	0.809995	18.53726	level4	Public	NA	NA	NA
C7 H8 O3	0.000145	0.844179	NA	level4	KEGG	C06677	NA	Benzenoid
C19 H20 F	-0.0016	-4.55633	NA	level4	HMDB	NA	HMDB024	NA
C15 H20 C	0.00159	4.619904	NA	level4	HMDB	NA	HMDB024	Organic ox
C19 H28 C	7.05E-05	0.183561	NA	level4	HMDB	NA	HMDB006	Lipids and
C19 H25 N	-0.00043	-1.29104	NA	level4	HMDB	NA	HMDB025	NA
C42 H76 N	-0.00043	-0.56268	NA	level4	NA	NA	NA	NA
C17 H24 C	-0.00035	-1.25745	NA	level4	HMDB	NA	HMDB001	Benzenoid
C20 H21 N	-0.00014	-0.44858	NA	level4	HMDB	NA	HMDB025	NA
C24 H21 C	0.000618	1.195181	NA	level4	HMDB	NA	HMDB024	NA
C52 H48 N	-0.00032	-0.37141	NA	level4	HMDB	NA	HMDB025	NA
C9 H14 N2	-9.65E-06	-0.0392	NA	level4	HMDB	NA	HMDB000	Organic ox
C10 H16 N	-0.00056	-1.9183	NA	level4	HMDB	NA	HMDB025	NA
C7 H9 N	0.000147	1.371136	NA	level4	HMDB	NA	HMDB003	Benzenoid
C30 H38 N	0.00249	4.804451	NA	level4	NA	C10013	NA	NA
C28 H58 N	0.000288	0.493118	NA	level4	NA	NA	NA	NA
C16 H18 C	-0.00074	-2.40459	1.412979	level4	Public	NA	NA	NA
C12 H20 N	-1.13E-05	-0.04397	NA	level4	NA	C20515	NA	NA

C12 H21 N	7.32E-05	0.282663	NA	level4	HMDB	NA	HMDB024	NA
C22 H40 C	21.9799	45766.95	24.87213	level4	Public	NA	NA	NA
C13 H21 N	-1.38E-05	-0.05174	NA	level4	HMDB	NA	HMDB024	NA
C22 H30 F	0.000757	1.990937	NA	level4	NA	C15112	NA	NA
C5 H12 O5	21.98219	144554.5	56.34233	level3	BMDB	C00379	HMDB000	Organic ox
C5 H12 O5	0.000191	1.253547	24.37641	level3	BMDB	C00474	HMDB000	Organic ox
C15 H33 N	8.80E-05	0.387291	NA	level4	HMDB	NA	HMDB024	NA
C8 H12 N2	0.000375	1.734128	NA	level4	HMDB	NA	HMDB025	NA
C25 H24 F	-0.00094	-1.78797	NA	level4	HMDB	NA	HMDB025	NA
C17 H23 F	0.001406	3.623348	NA	level4	HMDB	NA	HMDB025	NA
C25 H20 C	-0.00029	-0.72153	NA	level4	NA	NA	NA	NA
C20 H30 C	37.95402	108375.3	4.331441	level4	Public	NA	NA	NA
C7 H11 N3	0.00029	1.567703	NA	level4	HMDB	NA	HMDB025	NA
C22 H41 N	-4.53E-05	-0.12886	NA	level4	NA	NA	NA	NA
C28 H30 N	-0.00036	-0.69365	NA	level4	HMDB	NA	HMDB025	NA
C20 H38 C	0.00024	0.512866	NA	level4	NA	C21658	NA	NA
C9 H10 O4	0.000183	1.006342	67.04244	level2	mzCloud	NA	HMDB000	Benzenoid
C22 H30 C	6.27E-05	0.192058	NA	level4	HMDB	NA	HMDB025	NA
C18 H30 C	17.02638	52194.66	57.42997	level3	mzCloud	C14794	NA	FA Fatty a
C9 H9 F6 N	-0.00163	-4.83288	NA	level4	HMDB	NA	HMDB024	NA
C20 H22 C	-0.00025	-0.53787	NA	level4	HMDB	NA	HMDB024	Phenylpro
C19 H28 N	0.000102	0.25986	NA	level4	HMDB	NA	HMDB025	NA
C17 H15 N	0.000562	1.646752	NA	level4	HMDB	NA	HMDB024	NA
C20 H32 C	-0.00014	-0.47027	98.23805	level2	mzCloud	C00219	HMDB000	Lipids and
C27 H31 N	-0.00129	-2.64845	NA	level4	HMDB	NA	HMDB024	NA
C30 H53 N	21.9842	37428.4	10.69074	level4	Public	NA	NA	NA
C23 H37 N	-1.36E-05	-0.0362	NA	level4	NA	NA	NA	NA
C8 H15 N	0.00023	1.326766	68.81968	level1	BMDB	C02710	HMDB001	Organic ac
C20 H34 C	35.97664	101559.9	34.7817	level3	mzCloud	NA	NA	NA
C26 H36 N	0.002721	4.071795	NA	level4	HMDB	NA	HMDB024	NA
C3 H7 N C	-1.88E-05	-0.21068	78.14914	level2	mzCloud	C00099	HMDB000	Organic ac
C14 H16 N	-0.00016	-0.49944	NA	level4	KEGG	C08564	NA	Amino aci
C22 H28 C	43.96121	113247	19.08589	level4	Public	NA	NA	NA
C38 H62 C	-0.00161	-2.68761	NA	level4	NA	NA	NA	NA
C22 H38 N	0.000614	1.231942	NA	level4	HMDB	NA	HMDB025	NA
C23 H25 N	-7.77E-05	-0.17684	NA	level4	HMDB	NA	HMDB025	NA
C15 H10 C	-0.00072	-3.2501	3.552545	level4	Public	C15608	HMDB000	Phenylpro
C30 H30 C	17.02464	28461.28	5.68389	level4	Public	NA	NA	NA
C20 H30 N	0.00156	4.305464	NA	level4	HMDB	NA	HMDB024	NA
C18 H16 C	0.000136	0.319713	NA	level4	NA	NA	NA	NA
C7 H8 N4	0.000267	1.36021	NA	level4	HMDB	NA	HMDB000	Organohe
C18 H13 C	0.001587	4.881073	NA	level4	HMDB	NA	HMDB001	Organohe
C32 H32 N	0.000572	1.065754	NA	level4	HMDB	NA	HMDB025	NA
C24 H28 F	0.002156	4.654294	NA	level4	HMDB	NA	HMDB025	NA
C10 H9 N	0.000234	1.472067	86.15969	level1	BMDB	C00637	HMDB000	Organohe
C22 H37 N	-0.00178	-3.0476	NA	level4	HMDB	NA	HMDB025	NA
C15 H16 C	-0.00013	-0.46531	NA	level4	KEGG	C09584	NA	Terpenoid
C11 H20 N	-7.45E-05	-0.28627	NA	level4	HMDB	NA	HMDB001	Organic ac
C23 H33 N	0.000869	1.659982	NA	level4	HMDB	NA	HMDB024	NA
C10 H10 C	-18.0103	-101146	61.94522	level2	mzCloud	NA	NA	NA
C26 H27 N	0.001882	3.815966	NA	level4	HMDB	NA	HMDB025	NA
C5 H9 N C	18.01067	100604.1	0.869726	level4	BMDB	C03727	HMDB002	Organic ac
C30 H42 C	-0.00054	-1.02366	6.161905	level4	Public	NA	NA	NA
C25 H20 C	-0.00201	-4.31655	NA	level4	HMDB	NA	HMDB025	NA

C30 H52 C	0.001436	2.449977	NA	level4	NA	C17434	NA	NA
C11 H9 N	-0.00212	-10.4592	50.57605	level3	mzCloud	C00331	HMDB006	Organohe
C25 H26 N	8.99E-05	0.189593	NA	level4	HMDB	NA	HMDB025	NA
C8 H8 O2	0.000263	1.931181	NA	level4	HMDB	NA	HMDB000	Benzenoid
C10 H8 Cl	0.000965	3.815793	NA	level4	KEGG	C04798	NA	NA
C19 H20 F	0.002335	4.082192	NA	level4	HMDB	NA	HMDB025	NA
C8 H8 O	2.22E-05	0.18525	NA	level4	NA	NA	NA	NA
C9 H10 O3	5.37E-05	0.32312	94.4164	level1	Decode	C01479	HMDB000	Phenylpro
C3 H6 O2	0.000143	1.924815	NA	level4	HMDB	NA	HMDB025	NA
C13 H14 N	0.000152	0.619661	50.63993	level3	Public	NA	NA	NA
C32 H60 N	0.000438	0.674954	NA	level4	NA	NA	NA	NA
C16 H21 N	1.35E-05	0.052188	NA	level4	HMDB	NA	HMDB000	Benzenoid
C22 H46 N	-1.26E-05	-0.02692	NA	level4	HMDB	NA	HMDB001	Lipids and
C14 H17 N	-0.0001	-0.35551	31.35888	level4	Public	NA	NA	NA
C17 H16 C	17.02836	53871.09	6.233496	level4	Public	NA	NA	NA
C35 H60 C	0.00133	2.077235	NA	level4	NA	C17024	NA	NA
C33 H40 C	0.001636	2.478367	NA	level4	NA	NA	NA	NA
C26 H49 C	0.000636	1.185777	NA	level4	NA	NA	NA	NA
C17 H12 C	-0.00128	-3.87604	NA	level4	NA	C11226	NA	Benzenoid
C6 H11 N	0.00018	1.392144	75.99705	level2	Decode	NA	HMDB009	Organic ac
C12 H10 C	2.12E-05	0.114048	58.37219	level3	Public	NA	NA	NA
C11 H15 N	2.20E-05	0.105105	NA	level4	HMDB	NA	HMDB025	NA
C36 H62 C	0.000177	0.316654	NA	level4	NA	NA	NA	NA
C23 H32 C	-0.00011	-0.31057	NA	level4	HMDB	NA	HMDB025	NA
C34 H56 C	0.001601	2.563417	NA	level4	NA	NA	NA	NA
C23 H28 C	0.000432	1.000603	NA	level4	HMDB	NA	HMDB024	NA
C40 H50 C	-0.00016	-0.26528	NA	level4	NA	NA	NA	NA
C15 H20 C	-0.00013	-0.47356	27.1613	level4	Public	NA	HMDB003	Lipids and
C22 H38 C	0.001502	3.491832	9.38479	level4	Public	NA	NA	NA
C22 H34 F	-0.00093	-1.95005	NA	level4	HMDB	NA	HMDB025	NA
C22 H18 N	0.000665	1.531631	NA	level4	HMDB	NA	HMDB025	NA
C8 H12 N2	-0.00026	-1.22553	NA	level4	NA	C17359	NA	NA
C6 H4 O2	0.000265	2.456137	NA	level4	HMDB	NA	HMDB025	NA
C17 H23 N	0.000433	1.13619	NA	level4	HMDB	NA	HMDB024	NA
C17 H21 N	-0.00142	-4.3309	NA	level4	HMDB	NA	HMDB024	NA
C14 H12 C	-0.0002	-0.7487	NA	level4	HMDB	NA	HMDB025	NA
C13 H9 N	0.000269	1.274323	NA	level4	HMDB	NA	HMDB025	NA
C12 H27 N	0.000251	1.355442	NA	level4	HMDB	NA	HMDB025	NA
C19 H23 N	-0.00135	-3.64363	NA	level4	HMDB	NA	HMDB001	Organohe
C24 H36 C	-5.09E-05	-0.13111	NA	level4	NA	NA	NA	NA
C23 H42 C	17.02489	33364.63	2.716466	level4	Public	NA	NA	NA
C14 H15 N	3.29E-05	0.082836	NA	level4	HMDB	NA	HMDB024	NA
C21 H44 N	0.000245	0.539732	NA	level4	HMDB	NA	HMDB025	NA
C21 H32 C	-0.00013	-0.39929	NA	level4	HMDB	NA	HMDB000	Lipids and
C21 H22 N	-0.00159	-4.25795	NA	level4	HMDB	NA	HMDB025	NA
C26 H43 N	0.001006	1.90147	NA	level4	HMDB	NA	HMDB025	NA
C37 H41 N	0.002493	4.131996	NA	level4	HMDB	NA	HMDB025	NA
C26 H43 N	0.000722	1.324873	NA	level4	HMDB	NA	HMDB024	NA
C42 H66 N	-0.00224	-2.8937	NA	level4	NA	NA	NA	NA

C22 H44 N	-0.00033	-0.59654	NA	level4	HMDB	NA	HMDB001	Organic o:
C23 H29 F	-0.00196	-4.84461	NA	level4	HMDB	NA	HMDB026	NA
C13 H17 N	-2.90E-05	-0.11719	NA	level4	HMDB	NA	HMDB025	NA
C46 H86 N	0.000294	0.354775	NA	level4	NA	NA	NA	NA
C26 H26 N	0.00331	4.795779	NA	level4	HMDB	NA	HMDB024	NA
C22 H45 N	-8.46E-05	-0.24932	NA	level4	NA	NA	NA	NA
C40 H81 N	0.001205	1.932411	NA	level4	HMDB	NA	HMDB001	Lipids and
C40 H77 N	9.94E-05	0.160406	NA	level4	NA	NA	NA	NA
C5 H10 O3	21.98218	186190.3	70.42397	level1	BMDB2	NA	HMDB000	Lipids and
C21 H20 C	0.000378	0.842449	NA	level4	HMDB	NA	HMDB025	NA
C16 H14 C	-5.83E-05	-0.24506	NA	level4	NA	NA	NA	NA
C26 H38 C	-0.00028	-0.62999	35.68192	level4	Public	NA	NA	NA
C8 H7 N C	-0.00032	-1.31282	NA	level4	HMDB	NA	HMDB024	Organohe
C17 H12 C	-9.95E-05	-0.28921	NA	level4	NA	NA	NA	NA
C22 H45 C	-0.00024	-0.53291	NA	level4	NA	NA	NA	NA
C26 H43 N	-8.27E-06	-0.01908	NA	level4	HMDB	NA	HMDB000	Lipids and
C24 H22 C	-0.00128	-2.63972	NA	level4	HMDB	NA	HMDB025	NA
C8 H10 O4	-18.0105	-105908	94.47663	level2	BMDB	C05576	HMDB000	Benzenoid
C27 H45 C	0.002643	4.856291	NA	level4	NA	NA	NA	NA
C16 H21 N	-0.00013	-0.4009	NA	level4	HMDB	NA	HMDB025	NA
C26 H43 N	-9.87E-06	-0.02197	93.89183	level1	BMDB2	C05466	HMDB000	Lipids and
C14 H21 N	0.00093	3.956208	NA	level4	HMDB	NA	HMDB006	Benzenoid
C5 H8 O2	0.000134	1.341664	87.74582	level1	BMDB	C02240	NA	NA
C35 H62 C	0.000669	1.157151	NA	level4	NA	NA	NA	NA
C20 H30 C	0.00055	1.150115	3.87775	level4	Public	NA	NA	NA
C24 H33 N	-0.0009	-1.62439	NA	level4	HMDB	NA	HMDB024	NA
C25 H39 N	-0.00017	-0.4327	NA	level4	HMDB	NA	HMDB024	NA
C17 H16 C	17.02632	59929.69	38.84245	level4	Public	NA	NA	NA
C20 H28 C	-0.00019	-0.61628	29.17818	level4	Public	NA	NA	NA
C15 H23 N	0.004767	12.5056	41.94463	level3	mzCloud	D05846	NA	NA
C9 H9 N C	-4.68E-06	-0.03184	30.4735	level4	Public	C02366	HMDB030	NA
C29 H48 C	0.000507	1.064272	NA	level4	NA	NA	NA	NA
C9 H10 N2	0.000234	1.316844	NA	level4	HMDB	NA	HMDB024	NA
C22 H20 F	-0.00163	-3.4674	NA	level4	HMDB	NA	HMDB025	NA
C18 H38 C	-9.38E-05	-0.29456	NA	level4	NA	NA	NA	NA
C18 H25 N	0.00329	9.816015	48.54435	level3	mzCloud	C06176	NA	Alkaloids
C19 H22 N	-4.25E-05	-0.10477	NA	level4	HMDB	NA	HMDB024	NA
C23 H23 N	0.000478	0.986022	NA	level4	NA	C17353	NA	NA
C8 H11 N	-5.09E-05	-0.20443	NA	level4	HMDB	NA	HMDB000	Organic ac
C19 H30 C	-7.41E-06	-0.02551	NA	level4	HMDB	NA	HMDB025	NA
C11 H18 N	-0.00059	-1.6157	NA	level4	HMDB	NA	HMDB024	NA
C26 H34 N	-0.00013	-0.24433	NA	level4	HMDB	NA	HMDB025	NA
C27 H36 C	-0.00217	-4.3001	NA	level4	NA	NA	NA	NA
C8 H10 N6	0.00038	1.598027	NA	level4	HMDB	NA	HMDB025	NA
C16 H18 N	-0.00022	-0.79891	NA	level4	HMDB	NA	HMDB025	NA
C7 H9 N3	-0.00017	-0.73728	NA	level4	HMDB	NA	HMDB025	NA
C25 H40 C	-6.98E-05	-0.18755	NA	level4	NA	NA	NA	NA
C20 H30 C	-18.0106	-53889.4	85.0042	level2	mzCloud	NA	NA	NA
C19 H21 N	-5.17E-05	-0.13214	NA	level4	NA	C10686	NA	Alkaloids
C9 H18 N2	-0.00017	-0.79734	NA	level4	HMDB	NA	HMDB025	NA
C20 H30 C	17.02649	50944.82	33.12625	level3	mzCloud	C05957	HMDB000	Lipids and
C18 H34 C	-36.0212	-114627	65.75418	level2	Public	NA	NA	NA
C28 H35 F	0.00241	4.799188	NA	level4	HMDB	NA	HMDB001	Lipids and

C21 H24 N	21.98022	59700.69	29.52044	level4	Public	NA	NA	NA
C21 H18 C	-0.00084	-2.5023	22.65824	level4	Public	NA	NA	NA
C22 H28 C	6.57E-05	0.20262	NA	level4	HMDB	NA	HMDB001	Lipids and
C57 H96 C	-0.00226	-2.57676	NA	level4	NA	NA	NA	NA
C8 H13 N	0.000291	1.702494	NA	level4	HMDB	NA	HMDB024	NA
C25 H30 N	0.000515	0.99361	NA	level4	HMDB	NA	HMDB025	NA
C8 H15 N	-3.40E-05	-0.17972	NA	level4	HMDB	NA	HMDB025	NA
C32 H47 N	0.000477	0.717721	NA	level4	NA	NA	NA	NA
C8 H9 N5	-0.00052	-2.1807	NA	level4	HMDB	NA	HMDB024	NA
C29 H36 C	0.00228	4.188828	10.12998	level4	Public	NA	NA	NA
C4 H5 N3	0.000102	0.803086	NA	level4	HMDB	NA	HMDB024	NA
C11 H7 N	0.000131	0.774683	NA	level4	HMDB	NA	HMDB024	NA
C37 H44 C	0.002487	4.136153	NA	level4	NA	C20795	NA	Fungal tox
C28 H48 C	0.00069	1.390724	NA	level4	NA	C19874	NA	NA
C12 H20 N	4.45E-05	0.132266	NA	level4	HMDB	NA	HMDB006	Organic ac
C34 H60 N	-0.00042	-0.589	NA	level4	NA	NA	NA	NA
C24 H44 C	-6.80E-05	-0.18675	NA	level4	HMDB	NA	HMDB025	NA
C28 H46 C	0.00018	0.419047	NA	level4	HMDB	NA	HMDB025	NA
C22 H18 N	-0.00071	-1.8971	NA	level4	NA	C11564	NA	NA
C12 H11 K	-0.00185	-2.94991	NA	level4	HMDB	NA	HMDB025	NA
C23 H38 N	-0.00117	-2.43889	NA	level4	HMDB	NA	HMDB025	NA
C12 H14 C	21.98221	115635.4	3.610244	level4	Public	C16987	HMDB030	NA
C31 H40 N	0.000268	0.462514	NA	level4	HMDB	NA	HMDB026	NA
C21 H30 C	-0.00016	-0.48644	65.29635	level2	mzCloud	C01176	HMDB000	Lipids and
C24 H22 N	0.000169	0.407206	NA	level4	HMDB	NA	HMDB025	NA
C16 H26 N	0.002386	4.622548	NA	level4	NA	C12447	NA	NA
C27 H41 N	-0.00139	-2.98494	NA	level4	HMDB	NA	HMDB024	NA
C26 H45 N	-18.0101	-36071	96.35277	level2	mzCloud	NA	NA	NA
C7 H9 N5	0.000192	1.069748	NA	level4	HMDB	NA	HMDB024	NA
C22 H28 C	-0.0041	-11.0115	37.16489	level3	mzCloud	D06673	NA	NA
C4 H6 O3	0.000166	1.630158	49.25521	level3	BMDB	C00164	HMDB000	Organic ac
C20 H28 C	17.02618	51252.98	13.26241	level4	Public	NA	NA	NA
C17 H26 C	0.00038	1.290254	NA	level4	HMDB	NA	HMDB025	NA
C8 H16 N2	0.000307	1.629876	41.79864	level3	mzCloud	C02727	HMDB000	Organic ac
C9 H16 O9	0.001225	4.570981	NA	level4	HMDB	NA	HMDB025	NA
C17 H22 C	0.001167	3.622427	NA	level4	HMDB	NA	HMDB024	NA
C18 H32 C	21.98278	43602.01	88.29622	level1	BMDB	C00492	HMDB000	Organic ox
C13 H15 N	17.02649	78423.22	20.5726	level4	Public	NA	NA	NA
C26 H45 N	0.003185	4.716116	NA	level4	HMDB	NA	HMDB025	NA
C22 H34 C	-0.00012	-0.35376	NA	level4	HMDB	NA	HMDB000	Lipids and
C11 H16 N	0.000198	1.028115	NA	level4	HMDB	NA	HMDB025	NA
C46 H66 N	-0.00429	-4.60923	NA	level4	HMDB	NA	HMDB000	Organic ac
C20 H19 N	-0.00116	-3.27277	NA	level4	HMDB	NA	HMDB025	NA
C10 H13 N	9.72E-06	0.0325	NA	level4	HMDB	NA	HMDB024	NA
C28 H31 F	0.001033	2.255315	NA	level4	HMDB	NA	HMDB001	Organohe
C21 H24 N	0.000661	1.78549	NA	level4	NA	C21586	NA	NA
C42 H81 C	-0.00131	-1.75455	NA	level4	NA	NA	NA	NA
C43 H68 C	-0.00139	-1.71546	NA	level4	HMDB	NA	HMDB001	Phenylpro
C11 H21 N	-0.00019	-0.68416	NA	level4	HMDB	NA	HMDB000	Organic ac
C18 H39 N	2.07E-05	0.065176	59.12195	level3	mzCloud	C12144	HMDB000	Organic ni
C28 H46 C	0.000961	1.826194	NA	level4	NA	NA	NA	NA

C21 H23 N	0.001302	3.451658	NA	level4	HMDB	NA	HMDB025	NA
C26 H36 C	0.001897	3.732638	NA	level4	NA	NA	NA	NA
C22 H16 C	-0.0008	-2.42708	NA	level4	HMDB	NA	HMDB025	NA
C29 H39 N	0.000149	0.2543	NA	level4	HMDB	NA	HMDB001	Phenylpro
C17 H16 C	0.001459	3.673651	NA	level4	HMDB	NA	HMDB025	NA
C26 H29 N	-0.00202	-4.05513	NA	level4	HMDB	NA	HMDB025	NA
C45 H70 N	-0.00357	-4.55806	NA	level4	NA	NA	NA	NA
C22 H36 C	17.02473	34587.6	4.383943	level4	Public	NA	NA	NA
C20 H10 F	-0.00044	-0.99544	NA	level4	HMDB	NA	HMDB025	NA
C27 H31 N	-0.00055	-1.08133	NA	level4	HMDB	NA	HMDB025	NA
C24 H27 N	37.95789	80901.77	9.244641	level4	Public	NA	NA	NA
C26 H22 F	0.00221	4.499041	NA	level4	HMDB	NA	HMDB024	NA
C5 H11 N	7.97E-05	0.598667	NA	level4	HMDB	NA	HMDB024	NA
C7 H6 O5	-3.67E-05	-0.18187	NA	level4	HMDB	NA	HMDB024	NA
C12 H13 N	-0.0001	-0.35304	NA	level4	HMDB	NA	HMDB024	NA
C29 H40 C	-0.00108	-2.08934	17.82303	level4	Public	NA	NA	NA
C10 H13 N	-0.00535	-14.5323	41.91193	level3	mzCloud	C01103	HMDB000	Nucleosid
C18 H30 C	-36.0211	-105262	11.75467	level4	Public	NA	NA	NA
C12 H20 C	0.001173	4.016377	11.66165	level4	Public	NA	NA	NA
C16 H13 F	0.000257	0.786829	NA	level4	HMDB	NA	HMDB025	NA
C9 H15 N4	-0.00027	-0.75655	NA	level4	KEGG	C01268	NA	NA
C17 H18 N	-0.00099	-3.16676	NA	level4	NA	C12767	NA	NA
C21 H37 N	-0.00016	-0.46277	NA	level4	HMDB	NA	HMDB024	NA
C9 H21 O4	0.000237	1.058548	NA	level4	HMDB	NA	HMDB025	NA
C16 H29 C	-0.0018	-4.94407	NA	level4	NA	C16507	NA	NA
C14 H21 N	-0.0001	-0.39285	NA	level4	HMDB	NA	HMDB026	NA
C19 H27 N	17.02571	36919.84	1.020455	level4	Public	NA	NA	NA
C26 H38 N	-0.00055	-1.13474	NA	level4	HMDB	NA	HMDB025	NA
C17 H31 N	-5.90E-05	-0.23674	NA	level4	NA	NA	NA	NA
C29 H44 C	-0.00019	-0.3383	NA	level4	NA	NA	NA	NA
C30 H26 C	0.000322	0.556617	NA	level4	HMDB	NA	HMDB003	Phenylpro
C25 H48 C	0.002043	3.672378	NA	level4	NA	NA	NA	NA
C19 H15 N	-0.00098	-2.91758	NA	level4	HMDB	NA	HMDB025	NA
C31 H34 C	21.98029	38819.67	9.763571	level4	Public	NA	NA	NA
C9 H11 N3	0.000203	0.903388	NA	level4	HMDB	NA	HMDB024	NA
C20 H23 N	0.001146	3.569543	NA	level4	HMDB	NA	HMDB024	NA
C39 H78 N	0.002444	3.323279	NA	level4	NA	NA	NA	NA
C31 H48 N	21.98507	38411.44	22.4835	level4	Public	NA	NA	NA
C19 H23 C	-0.00151	-3.53287	NA	level4	HMDB	NA	HMDB025	NA
C21 H34 C	-8.69E-05	-0.24805	NA	level4	HMDB	NA	HMDB024	NA
C22 H43 C	9.27E-05	0.205863	NA	level4	NA	NA	NA	NA
C23 H27 N	0.00032	0.69419	NA	level4	HMDB	NA	HMDB006	Alkaloids a
C24 H32 C	-9.28E-05	-0.26358	NA	level4	HMDB	NA	HMDB024	NA
C26 H29 N	-0.00062	-1.68016	NA	level4	HMDB	NA	HMDB001	Phenylpro
C16 H10 C	0.001065	2.703672	NA	level4	HMDB	NA	HMDB024	Phenylpro
C29 H36 C	-21.9786	-39231.6	8.248921	level4	Public	NA	NA	NA
C22 H28 C	21.98032	56622.78	12.24886	level4	Public	NA	NA	NA
C14 H25 N	-0.00022	-0.56784	NA	level4	HMDB	NA	HMDB024	NA
C40 H80 N	0.001693	2.258191	NA	level4	NA	NA	NA	NA
C7 H5 F3 I	-2.51E-05	-0.12166	NA	level4	HMDB	NA	HMDB024	NA
C21 H30 N	0.000279	0.538195	NA	level4	HMDB	NA	HMDB026	NA
C19 H17 N	0.000722	2.263101	49.57054	level4	Public	NA	NA	NA

C9 H9 N3	-0.00043	-2.24336	NA	level4	NA	C10897	NA	Organohe
C25 H48 N	-0.00194	-2.94887	NA	level4	KEGG	C02725	NA	NA
C15 H20 C	0.00157	4.785739	NA	level4	NA	C10717	NA	NA
C24 H35 N	37.95437	94588.78	9.043957	level4	Public	NA	NA	NA
C20 H23 N	-0.00035	-0.97587	NA	level4	HMDB	NA	HMDB025	NA
C26 H38 C	37.95471	79360.54	23.3486	level4	Public	NA	NA	NA
C27 H31 F	-1.75E-05	-0.02866	NA	level4	HMDB	NA	HMDB025	NA
C12 H18 C	-0.00017	-0.45563	NA	level4	KEGG	C02273	NA	Organic ox
C27 H30 C	0.000164	0.275474	66.03266	level2	Public	NA	HMDB003	Phenylpro
C30 H60 N	-0.00063	-1.05816	NA	level4	NA	NA	NA	NA
C15 H22 C	-6.96E-05	-0.24677	NA	level4	HMDB	NA	HMDB025	NA
C17 H24 C	37.95406	137428.8	31.3248	level4	Public	NA	NA	NA
C9 H10 O3	0.000162	0.974584	20.01905	level4	Public	NA	HMDB004	Organic ox
C14 H31 N	0.000107	0.468067	73.01487	level2	Public	NA	NA	NA
C19 H30 C	1.69E-05	0.058336	71.88908	level2	mzCloud	C03917	HMDB000	Lipids and
C11 H14 N	0.000171	0.830739	64.62954	level2	mzCloud	C07499	NA	NA
C25 H41 N	-2.29E-05	-0.06157	NA	level4	NA	NA	NA	NA
C19 H27 N	2.94E-05	0.074886	NA	level4	HMDB	NA	HMDB025	NA
C26 H26 N	-0.00148	-3.85964	1.527089	level4	Public	NA	NA	NA
C21 H14 C	-0.00047	-1.68221	NA	level4	NA	C14657	NA	NA
C12 H15 N	-4.67E-05	-0.21142	NA	level4	HMDB	NA	HMDB001	Benzenoid
C10 H14 C	8.89E-06	0.041533	NA	level4	NA	NA	NA	NA
C18 H34 C	-8.99E-05	-0.28598	NA	level4	HMDB	NA	HMDB000	Lipids and
C17 H17 N	3.59E-06	0.008169	NA	level4	HMDB	NA	HMDB006	Organic ac
C26 H28 C	-0.00098	-2.24631	NA	level4	NA	NA	NA	NA
C16 H19 N	0.003527	12.19923	41.37752	level3	mzCloud	C10847	HMDB004	Benzenoid
C24 H21 N	-0.00072	-1.50755	NA	level4	HMDB	NA	HMDB026	NA
C28 H28 N	0.001962	4.339591	NA	level4	HMDB	NA	HMDB025	NA
C10 H12 N	0.000197	0.693802	NA	level4	HMDB	NA	HMDB025	NA
C20 H32 C	55.96473	174761.4	24.44957	level4	Public	NA	NA	NA
C16 H20 N	-0.00011	-0.31557	NA	level4	HMDB	NA	HMDB000	Nucleoside
C26 H41 N	4.65E-05	0.111854	NA	level4	NA	C12158	NA	NA
C19 H25 N	-0.00048	-1.27704	NA	level4	NA	C21070	NA	NA
C16 H24 N	-0.00011	-0.34156	NA	level4	HMDB	NA	HMDB006	Organohe
C11 H16 F	-0.00122	-4.74258	NA	level4	HMDB	NA	HMDB024	NA
C23 H30 N	0.007152	17.95871	40.92962	level3	mzCloud	C09226	HMDB004	Alkaloids a
C18 H24 C	37.95414	118546.5	30.35282	level4	Public	NA	HMDB003	Benzenoid
C35 H39 N	-0.0007	-0.99849	NA	level4	KEGG	C06769	NA	NA
C6 H9 N3	0.000169	0.989719	71.93796	level1	Decode	C07203	HMDB001	Organohe
C16 H12 F	-0.0016	-5.09932	19.21741	level4	Public	NA	HMDB001	Organohe
C6 H6 N2	0.000138	1.131584	90.70231	level2	mzCloud	C00153	HMDB000	Organohe
C11 H10 C	-0.00071	-4.05612	1.939905	level4	Public	NA	NA	NA
C45 H73 N	-0.00058	-0.6538	NA	level4	HMDB	NA	HMDB025	NA
C4 H4 O2	0.000143	1.704861	NA	level4	HMDB	NA	HMDB024	NA
C10 H8 O4	-0.00212	-11.0222	46.72592	level3	mzCloud	C01752	HMDB003	Phenylpro
C8 H14 Cl	0.000879	4.086408	NA	level4	KEGG	C06551	NA	Organohe
C26 H29 N	-0.00177	-3.60396	NA	level4	HMDB	NA	HMDB025	NA
C11 H21 B	0.001164	4.406204	NA	level4	NA	NA	NA	NA
C12 H Cl7	-0.00166	-4.0958	NA	level4	HMDB	NA	HMDB024	NA
C15 H12 F	0.001566	4.787516	NA	level4	HMDB	NA	HMDB025	NA
C8 H10 I N	0.000139	0.503928	NA	level4	HMDB	NA	HMDB025	NA
C19 H26 C	6.64E-05	0.245612	NA	level4	HMDB	NA	HMDB024	NA
C24 H32 C	-0.00211	-4.3975	NA	level4	NA	C16752	NA	Organic ac
C23 H22 C	-0.00021	-0.52065	NA	level4	NA	C18522	NA	NA
C24 H32 C	-0.00183	-3.57599	5.029789	level4	Public	NA	NA	NA
C24 H23 N	0.001834	4.618487	NA	level4	HMDB	NA	HMDB024	NA

C34 H50 (	0.001351	2.077818	NA	level4	HMDB	NA	HMDB025	NA
C10 H18 (	0.000177	1.041072	NA	level4	HMDB	NA	HMDB025	NA
C14 H23 F	0.001423	4.110805	NA	level4	HMDB	NA	HMDB025	NA
C25 H22 (	-0.00085	-2.20651	NA	level4	NA	NA	NA	NA
C24 H26 N	-0.0015	-3.68293	NA	level4	HMDB	NA	HMDB001	Organohe
C22 H32 (	0.000251	0.728474	NA	level4	HMDB	NA	HMDB006	Lipids and
C16 H12 (	17.02754	56746.47	14.30665	level4	Public	NA	NA	NA
C19 H26 I	-0.00026	-0.31524	NA	level4	HMDB	NA	HMDB001	Benzenoid
C12 H16 (	0.000209	1.001884	40.7769	level3	mzCloud	C10430	NA	NA
C39 H74 N	8.11E-05	0.113368	NA	level4	NA	NA	NA	NA
C29 H46 (	0.001822	3.194334	11.09718	level4	Public	NA	NA	NA
C15 H30 N	0.001495	4.696925	NA	level4	HMDB	NA	HMDB025	NA
C26 H23 (	0.000203	0.417723	NA	level4	HMDB	NA	HMDB025	NA
C16 H20 (	35.03737	119937.1	14.59673	level4	Public	NA	NA	NA
C6 H12 O	0.000131	1.132001	NA	level4	HMDB	NA	HMDB024	NA
C9 H11 N!	4.43E-05	0.175132	NA	level4	HMDB	NA	HMDB024	NA
C16 H22 (	0.000263	0.855795	NA	level4	HMDB	NA	HMDB025	NA
C11 H12 N	0.000288	1.413284	NA	level4	HMDB	NA	HMDB006	Organohe
C25 H38 (	-8.26E-05	-0.17725	82.34773	level2	mzCloud	C11135	HMDB000	Lipids and
C11 H19 N	0.001184	3.172997	NA	level4	KEGG	C08415	NA	Amino aci
C9 H10 O	17.02691	93524.71	15.67871	level4	Public	NA	NA	NA
C26 H36 (	7.93E-05	0.200016	NA	level4	HMDB	NA	HMDB025	NA
C19 H38 N	0.002107	4.129424	3.662445	level4	Public	NA	NA	NA
C17 H12 (	-4.69E-05	-0.15034	NA	level4	HMDB	NA	HMDB000	Phenylpro
C31 H38 (	-0.00179	-2.55922	NA	level4	NA	NA	NA	NA
C13 H22 N	-0.00154	-3.75458	NA	level4	NA	C20928	NA	NA
C8 H6 Cl N	0.000424	2.366881	NA	level4	HMDB	NA	HMDB025	NA
C28 H52 N	-0.00134	-2.45682	NA	level4	HMDB	NA	HMDB001	Lipids and
C5 H11 N	0.000185	1.123195	89.7777	level2	mzCloud	C02989	HMDB000	Organic ac
C19 H21 N	345.1429	1068014	12.26744	level4	Public	NA	NA	NA
C16 H26 N	0.001032	2.880506	NA	level4	HMDB	NA	HMDB024	NA
C45 H44 (	-0.0004	-0.52037	NA	level4	NA	C10100	NA	Flavonoids
C37 H66 (	0.00068	1.121666	NA	level4	NA	NA	NA	NA
C11 H18 (	0.000251	1.171426	34.0446	level4	Public	NA	NA	NA
C16 H18 (	-0.00029	-1.01396	16.09977	level4	Public	NA	NA	NA
C31 H45 (	-0.00233	-2.8556	NA	level4	KEGG	C08443	NA	NA
C7 H12 N	-0.00013	-0.60614	NA	level4	HMDB	NA	HMDB025	NA
C20 H22 (	-6.38E-05	-0.15107	NA	level4	HMDB	NA	HMDB025	NA
C27 H45 N	-6.06E-06	-0.01405	NA	level4	HMDB	NA	HMDB025	NA
C16 H20 N	0.001434	3.896099	NA	level4	HMDB	NA	HMDB000	Organic o:
C11 H12 N	4.57E-05	0.224004	97.82608	level1	BMDB	C00078	HMDB000	Organohe
C10 H10 N	-0.00085	-4.18416	0.840624	level4	Public	C10930	HMDB025	NA
C10 H18 N	-0.00107	-3.83035	NA	level4	HMDB	NA	HMDB025	NA
C21 H42 (	-8.28E-05	-0.231	86.70289	level2	mzCloud	D01947	NA	NA
C20 H26 (	37.95738	104804.6	32.91448	level4	Public	NA	NA	NA
C20 H36 (	17.03272	50057.77	35.10158	level3	mzCloud	NA	NA	NA
C18 H35 A	0.001378	3.683251	NA	level4	NA	NA	NA	NA
C28 H29 N	-0.00075	-1.55527	NA	level4	HMDB	NA	HMDB001	Benzenoid
C16 H12 (	2.94E-05	0.092483	NA	level4	HMDB	NA	HMDB024	NA
C45 H86 N	9.04E-05	0.113104	NA	level4	HMDB	NA	HMDB025	NA
C52 H88 (	-0.00344	-4.2547	NA	level4	NA	NA	NA	NA
C25 H32 (	37.95491	104202.9	4.24698	level4	Public	NA	NA	NA
C9 H13 N	0.00046	2.027717	15.2166	level4	BMDB	C00881	HMDB000	Nucleosid
C43 H46 (	-0.00343	-3.67076	NA	level4	NA	C17401	NA	NA
C32 H28 N	-0.00147	-2.36249	NA	level4	HMDB	NA	HMDB024	NA
C16 H22 (	0.000105	0.40065	NA	level4	HMDB	NA	HMDB025	NA

C29 H36 C	-0.00047	-0.72029	NA	level4	NA	C10466	NA	NA
C17 H26 N	-0.00122	-3.84373	NA	level4	HMDB	NA	HMDB006	Organohe
C11 H11 N	0.000243	1.18705	85.11291	level2	mzCloud	NA	HMDB001	Organic ac
C13 H8 Cl:	-0.001	-3.0627	NA	level4	HMDB	NA	HMDB001	Benzenoid
C12 H15 N	0.000108	0.45703	NA	level4	HMDB	NA	HMDB025	NA
C23 H22 C	-0.00038	-0.74126	61.90769	level2	Public	NA	NA	NA
C27 H34 N	0.001148	2.303694	NA	level4	HMDB	NA	HMDB001	Organic ac
C14 H23 N	-0.00014	-0.48584	NA	level4	HMDB	NA	HMDB024	NA
C11 H11 F	0.000558	2.655909	NA	level4	HMDB	NA	HMDB025	NA
C22 H24 C	17.02643	42549.89	5.978365	level4	Public	NA	NA	NA
C21 H30 C	-0.00015	-0.48851	86.45268	level2	mzCloud	C00410	HMDB000	Lipids and
C46 H91 N	0.000438	0.548409	NA	level4	HMDB	NA	HMDB001	Lipids and
C15 H24 C	-0.00016	-0.59839	NA	level4	HMDB	NA	HMDB006	Organohe
C30 H51 N	0.000669	1.322888	51.16913	level4	Public	NA	HMDB024	NA
C17 H26 C	0.001508	3.573118	12.72551	level4	Public	NA	NA	NA
C21 H21 N	-0.00146	-4.16316	NA	level4	HMDB	NA	HMDB001	Lipids and
C16 H14 N	-8.20E-05	-0.26117	NA	level4	NA	C21552	NA	Benzenoid
C17 H20 C	-0.00024	-0.86763	NA	level4	NA	C15153	NA	NA
C11 H18 N	8.06E-06	0.031714	NA	level4	HMDB	NA	HMDB025	NA
C28 H45 N	0.000541	1.066791	NA	level4	HMDB	NA	HMDB025	NA
C9 H7 Cl2	0.000148	0.581174	NA	level4	HMDB	NA	HMDB025	NA
C40 H52 C	0.001169	2.131532	NA	level4	NA	NA	NA	NA
C31 H57 C	0.000354	0.542651	NA	level4	NA	NA	NA	NA
C7 H11 N3	0.000251	1.246302	NA	level4	HMDB	NA	HMDB025	NA
C12 H10 C	0.000176	0.872647	NA	level4	HMDB	NA	HMDB025	NA
C25 H24 C	-0.00057	-1.31725	18.42557	level4	Public	NA	NA	NA
C26 H32 C	0.000365	0.799461	NA	level4	NA	NA	NA	NA
C8 H12	0.00018	1.660744	NA	level4	HMDB	NA	HMDB024	NA
C23 H37 C	0.001179	2.583314	NA	level4	NA	NA	NA	NA
C41 H37 C	0.003331	4.574379	NA	level4	HMDB	NA	HMDB024	NA
C19 H27 C	0.000122	0.283956	NA	level4	HMDB	NA	HMDB025	NA
C30 H27 C	0.002446	4.222574	NA	level4	NA	NA	NA	NA
C27 H50 C	0.000252	0.536119	NA	level4	HMDB	NA	HMDB025	NA
C10 H11 C	-0.0007	-2.69635	NA	level4	HMDB	NA	HMDB024	NA
C18 H18 C	0.000208	0.663033	1.37445	level4	Public	NA	NA	NA
C23 H32 C	37.95415	93894.81	1.393284	level4	Public	NA	NA	NA
C7 H14 O3	8.98E-05	0.614354	NA	level4	HMDB	NA	HMDB025	NA
C8 H12 N4	-3.01E-05	-0.14195	NA	level4	HMDB	NA	HMDB025	NA
C37 H70 C	0.001221	2.05379	NA	level4	HMDB	NA	HMDB000	Lipids and
C20 H22 C	21.98106	54123.69	10.07139	level4	Public	NA	NA	NA
C11 H12 C	-0.00012	-0.66922	NA	level4	KEGG	C06359	NA	NA
C28 H38 N	17.0289	34175.85	19.47691	level4	Public	NA	NA	NA
C13 H14 C	0.000205	0.941212	NA	level4	HMDB	NA	HMDB025	NA
C28 H52 N	-0.00075	-1.30863	NA	level4	HMDB	NA	HMDB025	NA
C13 H22 C	0.000255	1.310827	NA	level4	KEGG	C03527	NA	Lipids and
C20 H18 C	-0.00037	-1.03424	NA	level4	HMDB	NA	HMDB024	NA
C6 H10 N2	0.000254	1.60881	NA	level4	HMDB	NA	HMDB024	NA
C20 H20 C	0.000544	1.461123	NA	level4	HMDB	NA	HMDB024	NA
C20 H24 N	-0.00012	-0.32017	NA	level4	HMDB	NA	HMDB001	Organohe
C21 H32 C	-6.71E-05	-0.16946	NA	level4	HMDB	NA	HMDB006	Lipids and
C4 H11 N	0.000116	1.590236	47.86115	level4	Public	NA	HMDB003	Organic ni
C21 H30 C	21.98025	69950.77	10.40124	level4	Public	NA	NA	NA
C23 H30 N	-0.00162	-3.74359	NA	level4	HMDB	NA	HMDB006	Organic ac
C25 H26 F	-0.00103	-2.06702	NA	level4	HMDB	NA	HMDB025	NA

C22 H22 C	-0.00114	-2.65612	10.55862	level4	Public	NA	NA	NA
C7 H5 N5	-0.00022	-1.16834	NA	level4	HMDB	NA	HMDB024	NA
C12 H21 N	-0.00155	-3.84343	NA	level4	KEGG	C08416	NA	Amino aci
C26 H34 N	5.17E-05	0.118062	NA	level4	HMDB	NA	HMDB024	NA
C19 H22 N	0.000463	1.292789	NA	level4	HMDB	NA	HMDB024	NA
C31 H44 C	0.000642	1.337248	NA	level4	HMDB	NA	HMDB026	NA
C20 H28 N	-0.00155	-4.55215	NA	level4	HMDB	NA	HMDB025	NA
C19 H34 C	0.0015	3.301764	NA	level4	NA	NA	NA	NA
C36 H51 N	0.001512	2.193676	NA	level4	KEGG	C08704	NA	Alkaloids
C22 H22 C	0.000647	1.352368	NA	level4	HMDB	NA	HMDB004	Phenylpro
C15 H18 N	0.000152	0.589694	NA	level4	HMDB	NA	HMDB025	NA
C7 H10 N4	-5.08E-05	-0.25659	41.1685	level4	Public	NA	NA	NA
C22 H40 C	-0.00011	-0.2976	NA	level4	NA	NA	NA	NA
C13 H13 N	-1.21E-05	-0.04974	NA	level4	HMDB	NA	HMDB024	NA
C23 H34 C	18.01071	46152.83	35.76671	level3	mzCloud	NA	NA	NA
C24 H34 C	-0.0003	-0.77801	NA	level4	HMDB	NA	HMDB024	NA
C20 H10 C	-0.00071	-2.5176	NA	level4	HMDB	NA	HMDB024	NA
C15 H15 N	4.37E-05	0.162495	NA	level4	HMDB	NA	HMDB025	NA
C20 H27 N	-1.39E-05	-0.0494	NA	level4	HMDB	NA	HMDB001	Benzenoid
C18 H30 C	21.98038	74707.43	32.80798	level3	mzCloud	C14765	HMDB000	Lipids and
C14 H18 C	-0.00038	-1.26556	14.83278	level4	Public	NA	NA	NA
C19 H32 C	0.000191	0.513501	NA	level4	HMDB	NA	HMDB024	NA
C25 H43 N	0.00208	4.322204	NA	level4	HMDB	NA	HMDB025	NA
C18 H25 N	-0.00027	-0.7677	74.48391	level2	mzCloud	C10364	NA	Alkaloids
C6 H7 N5	0.000265	1.607159	90.1859	level2	mzCloud	C02242	HMDB000	Organohe
C38 H58 C	-0.00193	-2.99979	NA	level4	NA	NA	NA	NA
C11 H6 Cl	0.000116	0.489969	NA	level4	NA	C14268	NA	NA
C24 H27 C	-0.00038	-0.86902	NA	level4	HMDB	NA	HMDB026	NA
C12 H8 O	0.000226	1.346147	NA	level4	HMDB	NA	HMDB025	NA
C12 H11 N	-0.00118	-4.36968	NA	level4	HMDB	NA	HMDB024	NA
C11 H14 C	0.000206	1.271657	50.57796	level3	mzCloud	NA	HMDB003	Organic o:
C32 H44 C	0.00163	2.696649	NA	level4	NA	NA	NA	NA
C23 H32 C	-0.00248	-6.65333	45.228	level3	mzCloud	C14554	NA	NA
C24 H26 N	-0.00114	-2.9547	NA	level4	HMDB	NA	HMDB025	NA
C12 H6 Cl	0.00071	2.100435	NA	level4	HMDB	NA	HMDB025	NA
C20 H22 N	4.93E-05	0.139242	NA	level4	NA	C17874	NA	NA
C6 H13 N	7.32E-05	0.635912	NA	level4	HMDB	NA	HMDB024	NA
C20 H24 C	37.95752	100910	19.50344	level4	Public	NA	NA	NA
C17 H20 C	17.02646	46253.64	24.19082	level4	Public	NA	HMDB024	Organic o:
C27 H32 C	0.001036	2.370116	NA	level4	HMDB	NA	HMDB025	NA
C9 H10 O:	21.98215	132372.3	22.61344	level4	Public	NA	NA	NA
C7 H14 N	-0.00023	-0.85185	NA	level4	NA	C19005	NA	NA
C31 H61 C	2.08E-05	0.033277	NA	level4	NA	NA	NA	NA
C14 H14 C	17.02658	69188.63	28.48146	level4	Public	NA	NA	NA
C5 H12 O!	5.88E-05	0.386848	95.60952	level1	BMDB	C00532	HMDB000	Organic o:
C5 H9 O7	7.62E-05	0.359466	NA	level4	NA	C17947	NA	NA
C11 H11 N	-0.00023	-0.84229	NA	level4	HMDB	NA	HMDB025	NA
C19 H27 C	-0.00187	-4.83431	8.076281	level4	Public	NA	NA	NA
C12 H18 C	0.000125	0.454498	NA	level4	HMDB	NA	HMDB024	NA
C16 H12 C	-0.00243	-8.55019	57.9118	level3	mzCloud	C00814	HMDB000	Phenylpro
C5 H13 N	0.000137	1.573755	82.41328	level2	mzCloud	C02640	HMDB003	Organic ni

C23 H39 N	-0.00032	-0.80649	NA	level4	HMDB	NA	HMDB025	NA
C38 H75 N	0.000488	0.822073	NA	level4	HMDB	NA	HMDB000	Lipids and
C27 H28 N	0.000107	0.250903	NA	level4	HMDB	NA	HMDB024	NA
C6 H5 N C	6.84E-05	0.441355	NA	level4	HMDB	NA	HMDB024	NA
C8 H13 N	0.000367	1.805497	90.65732	level2	mzCloud	NA	NA	NA
C6 H12 O3	3.91E-05	0.296378	89.97895	level2	mzCloud	C06103	HMDB001	Organic ac
C15 H10 C	0.00338	10.62864	54.06268	level3	mzCloud	C10107	HMDB000	Phenylpro
C21 H22 C	17.02689	37827.76	4.107683	level4	Public	C17449	HMDB003	Phenylpro
C55 H94 C	-0.00272	-3.20008	NA	level4	HMDB	NA	HMDB025	NA
C18 H37 C	-4.27E-05	-0.10784	NA	level4	NA	NA	NA	NA
C18 H14 C	0.001109	3.769943	NA	level4	HMDB	NA	HMDB025	NA
C14 H12 C	0.000263	0.679085	NA	level4	HMDB	NA	HMDB004	Phenylpro
C18 H37 N	-0.00019	-0.68823	NA	level4	HMDB	NA	HMDB024	NA
C20 H33 N	-0.00026	-0.73286	NA	level4	NA	NA	NA	NA
C13 H9 Cl	0.000666	2.129422	NA	level4	NA	C19066	NA	NA
C10 H18 C	-2.22E-05	-0.10196	NA	level4	NA	C17228	NA	FA Fatty a
C9 H17 N	0.000295	1.723896	50	level3	Masslist	NA	HMDB000	Organic ac
C22 H35 N	0.000336	0.890936	NA	level4	KEGG	C08693	NA	Alkaloids
C26 H22 C	0.000885	1.983396	NA	level4	NA	NA	NA	NA
C15 H23 N	-0.00191	-4.77547	NA	level4	HMDB	NA	HMDB000	Nucleoside
C30 H57 C	0.002195	3.808357	NA	level4	NA	NA	NA	NA
C22 H34 C	-4.37E-05	-0.13245	NA	level4	HMDB	NA	HMDB024	NA
C22 H39 N	-0.00012	-0.37436	42.0862	level3	Public	NA	HMDB003	Lipids and
C18 H15 C	0.000306	0.853338	NA	level4	NA	C10969	NA	NA
C33 H44 C	0.001407	2.408844	NA	level4	NA	C19991	NA	Terpenoid
C20 H16 C	-0.00031	-0.88581	NA	level4	HMDB	NA	HMDB025	NA
C51 H72 N	-0.00047	-0.49963	NA	level4	HMDB	NA	HMDB025	NA
C18 H22 N	-0.00125	-3.84653	NA	level4	HMDB	NA	HMDB025	NA
C28 H51 N	-0.00229	-4.23334	4.136432	level4	Public	NA	NA	NA
C18 H13 C	0.001572	4.609188	NA	level4	HMDB	NA	HMDB006	Organohe
C33 H42 C	-0.00221	-4.39478	NA	level4	KEGG	C09948	NA	NA
C23 H44 C	-0.00164	-3.78625	NA	level4	NA	NA	NA	NA
C22 H25 N	-0.00199	-4.65461	NA	level4	HMDB	NA	HMDB001	Organohe
C20 H30 C	0.001137	2.54878	21.63981	level4	Public	NA	NA	NA
C20 H30 C	37.95396	113561.7	10.66302	level4	Public	NA	NA	NA
C20 H28 C	-0.00026	-0.88034	71.06498	level1	BMDB	C00777	HMDB000	Lipids and
C17 H25 N	-0.00404	-15.5869	42.49248	level3	mzCloud	NA	NA	NA
C7 H7 I O	-0.00012	-0.5242	NA	level4	KEGG	C03575	NA	NA
C21 H21 C	-0.00011	-0.3089	NA	level4	HMDB	NA	HMDB025	NA
C23 H42 N	-0.0003	-0.62914	NA	level4	NA	NA	NA	NA
C12 H23 N	0.00014	0.535089	NA	level4	HMDB	NA	HMDB006	Lipids and
C16 H22 N	-1.10E-05	-0.04002	NA	level4	NA	C18947	NA	NA
C41 H83 N	0.000575	0.786849	NA	level4	HMDB	NA	HMDB001	Lipids and
C25 H43 N	-2.83E-05	-0.07581	NA	level4	NA	NA	NA	NA
C15 H18 C	37.95747	136481	24.69976	level4	Public	NA	HMDB025	NA
C39 H44 N	-0.00283	-4.33281	NA	level4	KEGG	C09461	NA	Alkaloids
C15 H10 C	-0.00031	-1.21123	96.00195	level2	mzCloud	C10208	HMDB000	Phenylpro
C26 H45 N	-0.0005	-1.00616	87.26968	level2	mzCloud	C16868	HMDB000	Lipids and
C4 H7 N3	0.000277	2.144618	NA	level4	HMDB	NA	HMDB024	NA
C9 H8 I N	-0.00142	-4.6616	NA	level4	HMDB	NA	HMDB024	NA
C13 H12 F	-0.00071	-2.20674	NA	level4	HMDB	NA	HMDB025	NA
C6 H6 N6	0.000286	1.475262	NA	level4	HMDB	NA	HMDB001	Organohe
C17 H20 C	17.02643	53186.67	17.22849	level4	Public	NA	NA	NA
C16 H24 C	0.000104	0.37027	NA	level4	HMDB	NA	HMDB024	NA

C10 H15 N	-0.00024	-0.77998	NA	level4	HMDB	NA	HMDB025	NA
C16 H17 N	0.000124	0.341938	NA	level4	NA	C19154	NA	NA
C21 H24 N	-0.00177	-4.70735	NA	level4	HMDB	NA	HMDB025	NA
C24 H32 O	0.001653	3.560976	NA	level4	HMDB	NA	HMDB025	NA
C4 H7 N O	8.84E-05	0.664821	95.27661	level1	BMDB	C00049	HMDB000	Organic ac
C16 H22 N	-9.20E-05	-0.3172	NA	level4	HMDB	NA	HMDB001	Organohe
C5 H11 N	0.000288	1.460074	NA	level4	KEGG	C01616	NA	NA
C9 H14 O	-0.00012	-0.67705	NA	level4	NA	NA	NA	NA
C13 H16 O	6.24E-05	0.264145	29.71023	level4	Public	NA	NA	NA
C25 H28 O	21.98014	53847.33	4.942615	level4	Public	NA	NA	NA
C3 H6 O2	-5.58E-05	-0.75384	NA	level4	HMDB	NA	HMDB025	NA
C5 H6 N2	0.000176	1.397746	69.03687	level2	mzCloud	C00178	HMDB000	Organohe
C13 H13 O	-0.00137	-4.10606	NA	level4	HMDB	NA	HMDB025	NA
C22 H36 O	-7.93E-05	-0.22777	NA	level4	HMDB	NA	HMDB024	NA
C31 H46 O	3.12E-05	0.069178	NA	level4	HMDB	NA	HMDB025	NA
C7 H16 N	0.000284	1.77424	50	level3	Masslist	C02728	HMDB000	Organic ac
C4 H8 O5	9.87E-05	0.725315	85.69334	level1	NA	NA	NA	NA
C41 H72 O	0.000347	0.537619	NA	level4	HMDB	NA	HMDB000	Lipids and
C17 H23 N	-0.0001	-0.27829	NA	level4	HMDB	NA	HMDB024	NA
C20 H17 F	-0.00148	-3.63349	NA	level4	HMDB	NA	HMDB025	NA
C16 H14 F	-0.00146	-4.09922	NA	level4	KEGG	C01621	NA	NA
C9 H8 O2	0.000158	1.064528	92.42791	level2	mzCloud	C00423	HMDB000	Phenylpro
C16 H14 N	-9.91E-05	-0.33235	NA	level4	HMDB	NA	HMDB001	Organohe
C24 H30 N	-0.00245	-4.91865	NA	level4	HMDB	NA	HMDB025	NA
C11 H19 N	3.51E-05	0.121313	NA	level4	NA	C20919	NA	NA
C14 H16 O	18.01051	72592.35	10.7274	level4	Public	NA	NA	NA
C29 H51 O	0.001364	2.137205	NA	level4	NA	NA	NA	NA
C23 H29 N	0.002205	4.526926	NA	level4	HMDB	NA	HMDB025	NA
C13 H14 N	0.000706	2.305187	NA	level4	HMDB	NA	HMDB025	NA
C10 H9 N	0.000229	1.196866	8.897969	level3	Decode	NA	HMDB003	Organohe
C32 H44 N	0.000738	1.228601	NA	level4	HMDB	NA	HMDB025	NA
C40 H50 O	0.001611	2.865307	NA	level4	NA	NA	NA	NA
C12 H20 O	-0.00022	-0.88492	44.04921	level3	Public	NA	NA	NA
C19 H20 F	-0.00149	-4.05701	NA	level4	NA	C10939	NA	NA
C21 H23 N	0.00017	0.369651	NA	level4	HMDB	NA	HMDB024	NA
C17 H17 F	0.00136	4.302371	NA	level4	HMDB	NA	HMDB024	NA
C27 H32 O	-0.00052	-0.94847	NA	level4	NA	NA	NA	NA
C29 H32 N	0.002374	4.237473	NA	level4	HMDB	NA	HMDB025	NA
C15 H26 O	17.02638	63017.93	7.83682	level4	Public	NA	NA	NA
C29 H41 N	-0.00222	-4.76105	29.4116	level4	Public	C08007	HMDB001	Benzenoid
C27 H46 O	21.98042	56891.79	16.62355	level4	Public	C00187	HMDB000	Lipids and
C26 H37 N	0.000533	1.160821	NA	level4	NA	C12333	NA	NA
C14 H20	0.000209	1.11065	NA	level4	HMDB	NA	HMDB025	NA
C9 H7 N O	9.06E-05	0.624332	59.23053	level3	Public	NA	NA	NA
C23 H14 O	0.000902	1.871517	NA	level4	HMDB	NA	HMDB025	NA
C27 H30 O	-0.00047	-1.03633	NA	level4	HMDB	NA	HMDB024	NA
C23 H39 N	-1.62E-05	-0.03062	6.181448	level4	Public	NA	NA	NA
C57 H98 O	-0.00247	-2.80775	NA	level4	HMDB	NA	HMDB001	Lipids and
C4 H10 O	21.98218	180096.3	16.71918	level3	BMDB	C00503	HMDB000	Organic o
C19 H35 N	-1.76E-05	-0.05997	NA	level4	HMDB	NA	HMDB006	Organic o
C29 H24 N	0.002648	4.761704	NA	level4	HMDB	NA	HMDB025	NA
C23 H46 O	0.000128	0.331745	NA	level4	NA	NA	NA	NA
C41 H79 N	0.000254	0.400234	NA	level4	HMDB	NA	HMDB025	NA
C18 H18 O	-0.00013	-0.38465	4.357645	level4	Public	NA	NA	NA

C33 H40 C	0.000917	1.355314	NA	level4	HMDB	NA	HMDB024	NA
C14 H22 C	0.000159	0.554683	NA	level4	HMDB	NA	HMDB024	NA
C8 H7 N C	0.00013	0.786385	NA	level4	HMDB	NA	HMDB003	Organohe
C42 H84 N	-0.00035	-0.45395	NA	level4	NA	NA	NA	NA
C34 H26 C	-0.00214	-3.80049	NA	level4	KEGG	C08738	NA	NA
C6 H4 O4	0.000203	1.452791	NA	level4	KEGG	C04431	NA	Organohe
C32 H54 C	0.001155	2.099054	NA	level4	NA	NA	NA	NA
C20 H29 F	-0.00122	-3.63457	NA	level4	HMDB	NA	HMDB001	Lipids and
C12 H22 C	-2.01E-05	-0.07654	14.68382	level4	Public	NA	NA	NA
C36 H62 C	0.000352	0.612658	NA	level4	NA	NA	NA	NA
C21 H25 N	-0.00124	-3.11851	NA	level4	HMDB	NA	HMDB025	NA
C28 H34 C	-2.52E-05	-0.04124	4.368887	level4	Public	NA	NA	NA
C7 H5 Cl C	0.000116	0.74323	NA	level4	HMDB	NA	HMDB024	NA
C6 H11 N	0.000289	1.499329	NA	level4	HMDB	NA	HMDB024	NA
C20 H41 N	-0.00012	-0.36937	93.32127	level2	mzCloud	NA	NA	NA
C21 H18 C	-0.00051	-1.45034	NA	level4	HMDB	NA	HMDB024	NA
C10 H8 O5	17.02675	81844.73	5.835642	level4	Public	NA	NA	NA
C11 H15 N	0.000456	2.362353	NA	level4	HMDB	NA	HMDB025	NA
C10 H18 N	0.003937	13.57117	36.50742	level3	mzCloud	C03406	HMDB000	Organic ac
C19 H29 N	0.00017	0.410143	NA	level4	NA	NA	NA	NA
C45 H81 C	0.002112	2.705195	NA	level4	NA	NA	NA	NA
C18 H24 N	-3.09E-05	-0.08476	NA	level4	HMDB	NA	HMDB025	NA
C8 Cl F15	0.000342	0.790853	NA	level4	HMDB	NA	HMDB025	NA
C8 H14 N2	0.000186	0.794586	NA	level4	HMDB	NA	HMDB024	NA
C30 H40 C	-0.00042	-0.73303	2.843428	level4	Public	NA	NA	NA
C44 H80 N	0.000419	0.514505	NA	level4	HMDB	NA	HMDB001	Lipids and
C16 H26 C	-18.0104	-60401.5	32.94396	level3	mzCloud	NA	NA	NA
C10 H12 N	0.000147	0.83196	NA	level4	HMDB	NA	HMDB025	NA
C23 H37 N	2.73E-05	0.062068	71.35494	level2	mzCloud	NA	NA	NA
C18 H26 C	-0.00021	-0.64	NA	level4	HMDB	NA	HMDB001	Organohe
C44 H82 N	-0.00065	-0.8277	NA	level4	HMDB	NA	HMDB000	Lipids and
C25 H27 N	-0.00053	-0.81511	NA	level4	HMDB	NA	HMDB001	Organic ac
C8 H6 N4	0.000944	3.714984	NA	level4	NA	C19240	NA	NA
C41 H60 N	0.001849	2.158596	NA	level4	HMDB	NA	HMDB024	NA
C10 H14 C	-0.00027	-0.9261	NA	level4	HMDB	NA	HMDB025	NA
C31 H38 N	0.001439	2.357994	NA	level4	HMDB	NA	HMDB025	NA
C8 H14 O3	0.000327	2.065969	NA	level4	HMDB	NA	HMDB006	Organic ac
C11 H18 N	-0.00114	-4.15198	NA	level4	NA	C20820	NA	NA
C23 H36 C	-0.0002	-0.54083	NA	level4	NA	NA	NA	NA
C32 H51 N	9.17E-05	0.146648	NA	level4	HMDB	NA	HMDB000	Lipids and
C37 H51 N	-0.00143	-2.42935	NA	level4	NA	C20532	NA	NA
C36 H50 C	21.98539	29777.99	0.225756	level4	Public	NA	NA	NA
C16 H12 C	17.02661	59938.4	5.235768	level4	Public	NA	NA	NA
C13 H19 C	-0.00105	-4.39967	NA	level4	NA	C19037	NA	NA
C12 H18 N	0.000131	0.588117	NA	level4	HMDB	NA	HMDB006	Organic ac
C20 H24 C	-0.00027	-0.58561	7.774929	level4	Public	NA	NA	NA
C7 H12 O	0.000125	1.115994	NA	level4	HMDB	NA	HMDB003	Organic ox
C16 H28 C	6.93E-05	0.243833	NA	level4	HMDB	NA	HMDB025	NA
C17 H22 C	5.50E-05	0.213174	NA	level4	HMDB	NA	HMDB025	NA
C8 H13 N	0.000194	1.034396	NA	level4	HMDB	NA	HMDB024	NA
C30 H54 N	0.000429	0.751513	NA	level4	HMDB	NA	HMDB001	Lipids and
C20 H29 N	-0.00151	-3.8972	NA	level4	HMDB	NA	HMDB025	NA
C11 H15 N	-7.24E-05	-0.24356	91.17245	level2	mzCloud	C20674	HMDB000	Nucleoside
C32 H43 N	0.001766	3.145375	NA	level4	HMDB	NA	HMDB024	NA
C13 H14 N	8.30E-05	0.360556	NA	level4	HMDB	NA	HMDB006	Organohe
C16 H20 C	4.91E-05	0.151427	NA	level4	HMDB	NA	HMDB025	NA
C23 H21 N	-0.0012	-2.92081	NA	level4	HMDB	NA	HMDB025	NA
C19 H22 C	21.98022	73720.32	16.14073	level4	Public	NA	HMDB003	Lipids and

C37 H58 C	-0.00184	-2.7715	NA	level4	NA	NA	NA	NA
C30 H38 C	-0.00062	-1.3424	NA	level4	NA	NA	NA	NA
C22 H26 C	-0.00151	-4.93121	NA	level4	HMDB	NA	HMDB025	NA
C22 H28 C	17.02675	40522.67	23.08958	level4	Public	NA	NA	NA
C44 H76 N	0.003534	4.365214	NA	level4	HMDB	NA	HMDB001	Lipids and
C30 H54 C	-0.00204	-3.65453	NA	level4	NA	NA	NA	NA
C20 H20 C	-9.58E-05	-0.18356	NA	level4	HMDB	NA	HMDB025	NA
C19 H25 C	0.001902	4.788373	NA	level4	HMDB	NA	HMDB025	NA
C22 H22 C	0.000228	0.49327	18.55822	level4	Public	NA	NA	NA
C2 H2 N4	0.000308	2.365433	NA	level4	HMDB	NA	HMDB025	NA
C19 H27 N	-0.00147	-3.94585	NA	level4	HMDB	NA	HMDB024	NA
C16 H14 C	1.12E-05	0.036966	7.832441	level4	Public	NA	NA	NA
C11 H11 N	0.000243	1.402162	NA	level4	HMDB	NA	HMDB024	NA
C18 H22 C	0.000116	0.290646	NA	level4	HMDB	NA	HMDB002	Benzenoid
C22 H30 C	17.02814	32862.49	1.362492	level4	Public	NA	NA	NA
C17 H29 N	0.000503	1.538668	NA	level4	NA	C18539	NA	NA
C23 H16 C	-0.00054	-1.46133	NA	level4	NA	NA	NA	NA
C12 H11 C	5.66E-05	0.226292	75.41643	level2	Public	NA	HMDB025	NA
C25 H39 N	-1.07E-05	-0.0239	17.7452	level4	Public	NA	NA	NA
H3 O3 P	0.000134	1.634457	NA	level4	KEGG	C06701	NA	NA
C8 H7 N	9.01E-05	0.769675	87.54517	level2	mzCloud	C00463	HMDB000	Organohe
C26 H28 N	0.000495	1.12368	NA	level4	HMDB	NA	HMDB025	NA
C22 H32 C	0.000202	0.536388	20.33799	level4	Public	NA	NA	NA
C20 H36 N	0.000213	0.462164	25.10508	level4	Public	NA	NA	NA
C9 H13 N3	2.64E-05	0.101965	NA	level4	HMDB	NA	HMDB025	NA
C3 H10 N	0.000302	1.94747	NA	level4	KEGG	C01210	NA	Organic ac
C22 H17 F	-0.00026	-0.46462	NA	level4	HMDB	NA	HMDB025	NA
C10 H12 N	0.000284	1.476696	50.70106	level3	mzCloud	NA	NA	NA
C42 H78 N	-0.001	-1.32105	NA	level4	HMDB	NA	HMDB000	Lipids and
C16 H31 N	8.25E-05	0.325735	NA	level4	HMDB	NA	HMDB025	NA
C22 H42 C	0.000103	0.255456	NA	level4	NA	NA	NA	NA
C10 H17 N	-6.55E-06	-0.03044	NA	level4	HMDB	NA	HMDB025	NA
C22 H26 C	17.0266	40717.63	14.09128	level4	Public	NA	NA	NA
C7 H8 O6	1.48E-05	0.078922	NA	level4	NA	NA	NA	NA
C14 H10 N	-0.00122	-4.09819	NA	level4	HMDB	NA	HMDB001	Benzenoid
C17 H23 C	-0.00156	-4.77574	NA	level4	HMDB	NA	HMDB025	NA
C35 H38 F	0.002239	3.330511	NA	level4	HMDB	NA	HMDB025	NA
C25 H24 C	-0.00392	-9.70476	30.59251	level3	mzCloud	C10511	NA	PK Polyke
C10 H10 N	0.000193	1.017003	45.00405	level4	Public	NA	NA	NA
C24 H30 C	0.000113	0.295914	NA	level4	HMDB	NA	HMDB025	NA
C25 H46 N	0.002929	4.449121	NA	level4	NA	C21261	NA	NA
C18 H12 N	-0.0014	-4.49747	NA	level4	HMDB	NA	HMDB024	NA
C3 H6 N2	0.000169	1.960055	NA	level4	HMDB	NA	HMDB024	NA
C16 H27 N	-0.00038	-1.225	NA	level4	HMDB	NA	HMDB025	NA
C15 H24 N	-0.0007	-2.39296	NA	level4	HMDB	NA	HMDB025	NA
C20 H17 N	21.98134	59877.43	12.31246	level4	Public	NA	NA	NA
C16 H23 N	-0.0001	-0.38981	NA	level4	HMDB	NA	HMDB024	NA
C12 H21 N	-3.35E-05	-0.13782	50	level3	Masslist	NA	HMDB000	Lipids and
C15 H14 C	0.000116	0.47853	93.56546	level2	mzCloud	NA	NA	NA
C12 H19 N	-3.47E-06	-0.01272	NA	level4	HMDB	NA	HMDB025	NA
C11 H18 C	-0.00015	-0.64209	3.441818	level4	Public	NA	NA	NA
C27 H25 F	0.001265	2.651796	NA	level4	HMDB	NA	HMDB025	NA
C32 H50 C	0.001874	3.153428	NA	level4	NA	C16861	NA	NA

C28 H50 C	0.00193	3.752056	NA	level4	HMDB	NA	HMDB024	NA
C9 H11 N	0.000208	1.147254	97.16115	level1	BMDB	C01536	HMDB000	Organic ac
C21 H35 N	0.000345	0.704708	NA	level4	NA	C17376	NA	NA
C11 H14 C	0.003369	17.35823	36.47227	level3	mzCloud	D01420	HMDB003	Benzenoid
C19 H22 C	0.001556	4.284956	NA	level4	HMDB	NA	HMDB024	NA
C20 H26 N	-0.00088	-2.07946	NA	level4	NA	C15696	NA	NA
C22 H21 C	-3.92E-06	-0.01127	NA	level4	HMDB	NA	HMDB024	NA
C5 H11 N	0.000115	0.983721	89.9358	level2	mzCloud	C00183	HMDB000	Organic ac
C4 H8 O2	0.000189	1.577487	NA	level4	HMDB	NA	HMDB025	NA
C20 H43 N	-0.00012	-0.34969	NA	level4	NA	NA	NA	NA
C7 H2 N2	-0.00078	-2.85979	NA	level4	HMDB	NA	HMDB025	NA
C16 H14 C	-5.18E-05	-0.17143	89.03779	level2	mzCloud	C01709	HMDB000	Phenylpro
C12 H7 N	0.001145	4.99876	NA	level4	HMDB	NA	HMDB025	NA
C21 H20 C	0.000148	0.330553	40.85348	level4	Public	NA	HMDB030	NA
C5 H8 N2	0.000171	1.332502	48.68012	level3	mzCloud	C00906	HMDB000	Organohe
C24 H21 F	-0.0002	-0.48531	NA	level4	HMDB	NA	HMDB001	Organohe
C6 H8 O6	6.23E-05	0.353663	10.74465	level4	Public	C01041	HMDB000	Organohe
C36 H44 N	-0.00147	-2.45152	NA	level4	HMDB	NA	HMDB024	NA
C12 H24 C	0.001701	4.941923	NA	level4	KEGG	C08254	NA	NA
C23 H46 N	-2.53E-05	-0.04119	NA	level4	HMDB	NA	HMDB001	Organic ox
C19 H25 C	-0.00025	-0.72326	NA	level4	HMDB	NA	HMDB025	NA
C22 H21 C	0.000225	0.514774	NA	level4	HMDB	NA	HMDB006	Benzenoid
C25 H47 C	0.002119	4.322604	NA	level4	NA	NA	NA	NA
C17 H21 N	0.000237	0.87327	NA	level4	HMDB	NA	HMDB025	NA
C12 H15 F	0.001093	4.047196	NA	level4	HMDB	NA	HMDB025	NA
C26 H45 N	8.55E-05	0.220812	NA	level4	NA	NA	NA	NA
C34 H56 N	0.003886	4.979498	NA	level4	NA	NA	NA	NA
C11 H10 N	0.000241	1.294215	NA	level4	HMDB	NA	HMDB024	NA
C13 H24 C	17.0264	65445.27	10.91445	level4	Public	NA	NA	NA
C19 H30 C	-18.0105	-62057.2	64.45386	level2	mzCloud	C04373	HMDB000	Lipids and
C23 H24 C	-0.00048	-1.31683	43.87417	level4	Public	NA	NA	NA
C21 H20 N	-0.00044	-0.98706	NA	level4	HMDB	NA	HMDB006	Organohe
C11 H17 N	-0.00013	-0.49572	16.4508	level4	Public	NA	NA	NA
C33 H32 C	0.001702	3.150572	NA	level4	NA	NA	NA	NA
C15 H22 C	2.77E-05	0.12702	39.5355	level3	mzCloud	C17914	HMDB001	Lipids and
C17 H19 F	0.001224	2.985727	NA	level4	HMDB	NA	HMDB024	NA
C15 H11 C	-8.00E-05	-0.27973	NA	level4	HMDB	NA	HMDB001	Organohe
C27 H32 F	-0.0013	-2.57765	NA	level4	HMDB	NA	HMDB024	NA
C28 H55 C	-0.00012	-0.19626	NA	level4	NA	NA	NA	NA
C28 H32 C	-0.00047	-0.75125	NA	level4	HMDB	NA	HMDB025	NA
C13 H20 C	-0.00172	-4.4775	NA	level4	HMDB	NA	HMDB025	NA
C21 H34 C	17.02659	42755.62	8.946179	level4	Public	NA	NA	NA
C10 H14 N	-3.19E-05	-0.13161	NA	level4	NA	C20822	NA	NA
C23 H35 N	0.001868	3.980656	NA	level4	HMDB	NA	HMDB025	NA
C33 H65 N	0.000861	1.596713	NA	level4	NA	NA	NA	NA
C16 H22 N	0.000272	0.803148	NA	level4	HMDB	NA	HMDB000	Organic ox
C18 H24 C	-0.00751	-17.3701	37.98791	level3	mzCloud	NA	HMDB004	Lipids and
C17 H19 N	-9.01E-05	-0.29915	NA	level4	HMDB	NA	HMDB025	NA
C31 H29 N	0.001726	3.225076	NA	level4	NA	C11688	NA	NA
C14 H18 C	0.001023	3.098499	NA	level4	HMDB	NA	HMDB024	Organic ox
C18 H27 N	-5.56E-06	-0.01647	NA	level4	HMDB	NA	HMDB025	NA
C23 H20 F	-0.00184	-4.39228	NA	level4	HMDB	NA	HMDB024	NA

C6 H10 N	-3.84E-05	-0.16332	NA	level4	HMDB	NA	HMDB025	NA
C7 H16 N	-17.0263	-118135	5.14813	level3	Decode	NA	HMDB000	Organic a
C27 H38 N	21.97956	41140.37	1.132187	level4	Public	NA	NA	NA
C20 H30 C	21.98423	65778.82	70.5652	level2	mzCloud	C05953	HMDB000	Lipids and
C17 H26 C	0.000359	1.00049	NA	level4	HMDB	NA	HMDB025	NA
C34 H34 N	-0.00247	-4.29694	NA	level4	HMDB	NA	HMDB025	NA
C13 H18 C	0.000216	0.97327	NA	level4	HMDB	NA	HMDB024	NA
C19 H12 C	-0.00043	-1.58046	49.12635	level4	Public	NA	HMDB024	NA
C28 H47 N	-0.00073	-1.38939	NA	level4	NA	NA	NA	NA
C18 H23 N	-0.00013	-0.36926	NA	level4	HMDB	NA	HMDB025	NA
C18 H33 C	-0.00157	-3.70038	NA	level4	HMDB	NA	HMDB001	Organic a
C20 H22 C	0.001474	4.738694	NA	level4	HMDB	NA	HMDB025	NA
C16 H28 N	-0.00107	-3.45839	NA	level4	HMDB	NA	HMDB024	NA
C28 H34 N	0.002186	4.770296	NA	level4	HMDB	NA	HMDB025	NA
C18 H17 B	1.18E-05	0.030152	NA	level4	NA	NA	NA	NA
C21 H27 N	-0.00126	-3.59773	NA	level4	HMDB	NA	HMDB024	NA
C13 H21 N	-1.44E-05	-0.05742	NA	level4	HMDB	NA	HMDB025	NA
C18 H20 N	-0.00031	-0.89927	NA	level4	HMDB	NA	HMDB026	NA
C18 H12 C	-0.0004	-0.91007	NA	level4	HMDB	NA	HMDB025	NA
C9 H13 N	-6.83E-06	-0.02637	NA	level4	HMDB	NA	HMDB025	NA
C27 H28 N	-0.00057	-1.29441	NA	level4	HMDB	NA	HMDB025	NA
C21 H24 N	21.98016	65381.39	9.055294	level4	Public	NA	NA	NA
C20 H12 C	-0.00037	-1.11896	NA	level4	HMDB	NA	HMDB001	Organohe
C21 H28 C	37.95428	100891.6	10.24	level4	Public	NA	NA	NA
C10 H13 N	-0.00023	-0.83129	NA	level4	KEGG	C04201	NA	NA
C10 H16	0.000204	1.499404	NA	level4	HMDB	NA	HMDB025	NA
C37 H48 C	0.000221	0.408233	NA	level4	NA	NA	NA	NA
C18 H34 C	-0.00013	-0.42983	NA	level4	HMDB	NA	HMDB024	NA
C8 H14 O	0.000274	1.327472	NA	level4	HMDB	NA	HMDB025	NA
C13 H26 C	1.41E-05	0.065997	81.80069	level2	mzCloud	C17076	HMDB000	Lipids and
C13 H23 N	0.000689	2.166337	NA	level4	HMDB	NA	HMDB025	NA
C10 H10 N	0.000226	1.017302	NA	level4	HMDB	NA	HMDB024	NA
C14 H8 Cl	-0.00171	-4.90396	NA	level4	KEGG	C06644	NA	NA
C17 H31 F	-7.99E-05	-0.25936	NA	level4	HMDB	NA	HMDB025	NA
C18 H21 N	-3.74E-05	-0.12488	NA	level4	HMDB	NA	HMDB000	Alkaloids a
C24 H41 N	0.000348	0.853394	NA	level4	KEGG	C08669	NA	Alkaloids
C31 H41 N	-0.00191	-3.14295	NA	level4	HMDB	NA	HMDB024	NA
C29 H46 C	0.002202	4.348156	NA	level4	NA	C20009	NA	Marine bic
C17 H20 C	0.00011	0.287042	NA	level4	HMDB	NA	HMDB004	Organic o
C14 H16 N	-0.00173	-4.90124	NA	level4	HMDB	NA	HMDB024	NA
C22 H45 C	4.66E-05	0.096202	NA	level4	NA	NA	NA	NA
C16 H22 C	7.91E-05	0.21151	32.56111	level4	Public	NA	HMDB025	NA
C14 H20 C	-4.45E-05	-0.10881	NA	level4	HMDB	NA	HMDB024	NA
C20 H18 N	-0.00145	-4.10531	NA	level4	KEGG	C09360	NA	Alkaloids
C12 H20 N	-2.25E-05	-0.07117	NA	level4	NA	C20965	NA	NA
C15 H17 N	6.55E-07	0.001534	NA	level4	HMDB	NA	HMDB001	Organohe
C29 H40 C	0.001026	2.049935	NA	level4	NA	NA	NA	NA
C9 H16 Cl	-0.00028	-0.88638	NA	level4	HMDB	NA	HMDB024	NA
C20 H25 N	-0.00015	-0.434	NA	level4	HMDB	NA	HMDB025	NA
C11 H22 C	-0.0001	-0.31983	NA	level4	NA	C20235	NA	NA
C6 H14 N	-0.00252	-14.4849	57.48909	level3	mzCloud	C02385	NA	NA
C28 H36 C	0.000609	1.049765	NA	level4	NA	C10890	NA	Phenylpro
C46 H75 C	0.000267	0.32616	NA	level4	HMDB	NA	HMDB001	Lipids and
C9 H9 N C	0.000176	0.983403	94.07392	level1	Decode	C01586	HMDB000	Benzenoid
C17 H34 N	0.000132	0.319858	NA	level4	HMDB	NA	HMDB024	NA
C18 H18 C	0.00015	0.396659	3.791264	level4	Public	NA	NA	NA

C10 H12 C	0.000171	0.807665	NA	level4	HMDB	NA	HMDB000	Phenylpro
C22 H26 C	-0.00147	-4.35864	NA	level4	HMDB	NA	HMDB025	NA
C23 H40 C	-4.76E-05	-0.13057	NA	level4	HMDB	NA	HMDB026	NA
C6 H11 N	0.000121	0.836864	NA	level4	HMDB	NA	HMDB000	Organic ac
C28 H14 N	-0.00196	-4.43249	NA	level4	HMDB	NA	HMDB025	NA
C29 H52 N	-0.00315	-4.93569	NA	level4	NA	NA	NA	NA
C4 H6 O4	0.000135	1.14797	NA	level4	HMDB	NA	HMDB000	Organic ac
C19 H27 N	3.14E-05	0.079792	NA	level4	HMDB	NA	HMDB025	NA
C20 H19 F	0.000885	2.126296	NA	level4	HMDB	NA	HMDB025	NA
C9 H15 N	0.000222	1.022646	NA	level4	HMDB	NA	HMDB026	NA
C19 H30	6.84E-05	0.264942	NA	level4	HMDB	NA	HMDB026	NA
C14 H10 F	-0.00089	-2.69992	NA	level4	HMDB	NA	HMDB001	Organic ox
C29 H36 N	0.001291	2.559776	NA	level4	NA	C10001	NA	Organic ac
C16 H17 N	1.50E-05	0.047747	NA	level4	HMDB	NA	HMDB024	NA
C15 H20 C	0.000189	0.636686	NA	level4	HMDB	NA	HMDB025	NA
C19 H23 N	0.001656	4.078119	NA	level4	HMDB	NA	HMDB024	NA
C21 H41 N	-0.00016	-0.43703	NA	level4	NA	NA	NA	NA
C15 H18 N	-0.00065	-2.03297	50	level3	Masslist	NA	HMDB002	Organic ac
C20 H24 N	0.000107	0.288778	NA	level4	HMDB	NA	HMDB025	NA
C27 H34 F	0.001467	2.818982	NA	level4	NA	NA	NA	NA
C24 H34 C	-0.00215	-4.6121	8.553568	level4	Public	NA	NA	NA
C16 H19 N	4.22E-05	0.175142	NA	level4	HMDB	NA	HMDB025	NA
C17 H19 N	-0.00141	-4.57689	NA	level4	HMDB	NA	HMDB024	NA
C26 H43 N	-2.29E-05	-0.05286	62.95843	level2	Public	NA	NA	NA
C20 H21 N	3.43E-05	0.080254	NA	level4	HMDB	NA	HMDB024	NA
C9 H11 N	0.000258	1.308497	NA	level4	HMDB	NA	HMDB025	NA
C21 H24 C	17.02619	43864.72	2.203299	level4	Public	NA	NA	NA
C10 H7 F3	0.000386	1.556742	NA	level4	HMDB	NA	HMDB025	NA
C27 H33 N	-0.00181	-4.14845	NA	level4	NA	C12342	NA	NA
C15 H18 N	-0.00124	-3.7149	2.467403	level4	Public	C06681	HMDB001	Organohe
C16 H15 N	-0.00151	-4.46865	NA	level4	HMDB	NA	HMDB024	NA
C31 H42 N	0.002836	4.156601	NA	level4	HMDB	NA	HMDB024	NA
C17 H24 C	-9.09E-05	-0.28054	12.33742	level4	Public	NA	HMDB003	Alkaloids a
C23 H18 C	0.001209	2.970728	NA	level4	HMDB	NA	HMDB024	NA
C22 H33 N	3.65E-05	0.10164	NA	level4	KEGG	C08700	NA	Alkaloids
C19 H34 C	0.001209	2.75932	NA	level4	NA	NA	NA	NA
C20 H27 C	-1.85E-05	-0.05099	NA	level4	HMDB	NA	HMDB024	NA
C25 H33 C	0.00083	1.69988	NA	level4	HMDB	NA	HMDB025	NA
C8 H8 O3	0.000189	1.245478	NA	level4	HMDB	NA	HMDB025	NA
C16 H22 N	0.000212	0.555904	NA	level4	NA	C21971	NA	NA
C16 H20 N	-0.00013	-0.49801	NA	level4	HMDB	NA	HMDB024	NA
C25 H21 N	-0.00192	-4.86323	NA	level4	KEGG	C03717	NA	NA
C16 H23 N	-0.00138	-4.58481	NA	level4	HMDB	NA	HMDB024	NA
C13 H10 C	-4.67E-05	-0.12079	NA	level4	HMDB	NA	HMDB001	Organic ac
C6 H14 O3	0.000226	1.361463	NA	level4	HMDB	NA	HMDB025	NA
C35 H46 C	0.000593	0.879662	NA	level4	KEGG	C08786	NA	Terpenoid
C22 H15 N	-0.00068	-1.76179	NA	level4	NA	C21125	NA	NA
C24 H21 F	1.25E-05	0.029412	NA	level4	HMDB	NA	HMDB025	NA
C23 H25 N	0.002192	4.859335	NA	level4	HMDB	NA	HMDB001	Organohe
C44 H53 C	0.004577	4.806816	NA	level4	NA	C11542	NA	NA
C18 H22 C	-0.00404	-14.9651	33.3033	level3	mzCloud	C00468	HMDB000	Lipids and
C18 H35 N	-0.0003	-0.87222	NA	level4	HMDB	NA	HMDB024	NA
C16 H16 N	-4.08E-05	-0.14368	NA	level4	NA	C18559	NA	NA
C27 H27 N	-0.00057	-1.3234	NA	level4	HMDB	NA	HMDB024	NA
C15 H15 N	-0.0014	-4.53535	NA	level4	HMDB	NA	HMDB024	NA
C28 H38 N	-0.00147	-3.17079	NA	level4	HMDB	NA	HMDB026	NA
C14 H20 N	-0.00034	-0.6356	NA	level4	NA	C16155	NA	NA
C33 H57 C	-0.00263	-3.62546	NA	level4	NA	NA	NA	NA

C19 H23 N	-7.68E-05	-0.2151	NA	level4	HMDB	NA	HMDB025	NA
C15 H15 N	0.000112	0.331371	NA	level4	HMDB	NA	HMDB001	Lipids and
C25 H22 N	0.001503	3.494854	NA	level4	HMDB	NA	HMDB025	NA
C19 H20 N	-0.00164	-4.70328	NA	level4	HMDB	NA	HMDB025	NA
C4 H9 N C	-18.0104	-174751	68.88613	level2	BMDB	C00334	HMDB000	Organic ac
C21 H32 C	-3.61E-05	-0.10353	NA	level4	HMDB	NA	HMDB025	NA
C26 H45 N	-18.0094	-34949.9	31.92189	level4	Public	NA	NA	NA
C10 H11 N	0.000138	0.611003	NA	level4	NA	C22210	NA	NA
C35 H58 C	0.001169	2.154728	NA	level4	NA	NA	NA	NA
C14 H20 C	0.000154	0.699967	NA	level4	HMDB	NA	HMDB025	NA
C30 H48 C	0.00058	1.021262	NA	level4	HMDB	NA	HMDB000	Lipids and
C44 H82 N	0.001059	1.324079	NA	level4	NA	NA	NA	NA
C29 H24 C	0.001034	2.287816	NA	level4	NA	NA	NA	NA
C16 H20 C	0.000135	0.467192	NA	level4	HMDB	NA	HMDB001	Benzenoid
C39 H77 N	0.00187	2.924397	NA	level4	NA	NA	NA	NA
C43 H85 C	-0.00192	-2.47041	NA	level4	NA	NA	NA	NA
C25 H26 C	-0.0011	-2.9524	NA	level4	HMDB	NA	HMDB024	NA
C11 H11 C	-0.0011	-4.82798	NA	level4	HMDB	NA	HMDB024	NA
C36 H38 N	0.000506	0.772991	NA	level4	HMDB	NA	HMDB000	Organohe
C22 H27 N	-0.00186	-4.18884	NA	level4	HMDB	NA	HMDB025	NA
C25 H30 C	21.98083	47972.76	32.209	level4	Public	NA	NA	NA
C32 H44 N	-0.00015	-0.25366	NA	level4	KEGG	C08694	NA	Alkaloids
C25 H34 C	0.000586	1.268169	NA	level4	HMDB	NA	HMDB001	Organohe
C18 H28 C	37.95405	111567.7	22.31353	level4	Public	NA	NA	NA
C12 H16 N	-0.00118	-4.28195	NA	level4	HMDB	NA	HMDB025	NA
C16 H16 C	5.00E-05	0.141486	NA	level4	HMDB	NA	HMDB025	NA
C26 H44 F	0.000547	1.053924	NA	level4	HMDB	NA	HMDB025	NA
C5 H9 N	0.000164	1.978327	NA	level4	HMDB	NA	HMDB025	NA
C25 H25 N	-0.00024	-0.5181	NA	level4	HMDB	NA	HMDB024	NA
C8 H16 O4	0.000203	1.150087	NA	level4	HMDB	NA	HMDB025	NA
C26 H36 C	17.02695	36995.33	31.6838	level4	Public	NA	NA	NA
C15 H20 C	-0.00034	-1.29654	62.27771	level2	mzCloud	NA	NA	NA
C18 H35 N	-6.27E-05	-0.19045	NA	level4	NA	NA	NA	NA
C27 H46 C	0.000982	1.852002	NA	level4	NA	NA	NA	NA
C3 H9 O5	0.000132	0.848765	NA	level4	KEGG	C03894	NA	NA
C26 H27 C	-0.00083	-1.61329	NA	level4	HMDB	NA	HMDB025	NA
C6 H9 N3	0.000168	0.896044	95.67789	level2	mzCloud	NA	NA	NA
C27 H36 C	-0.00195	-4.1344	34.65918	level4	Public	NA	NA	NA
C19 H22 C	21.98029	69967.1	17.88446	level4	Public	NA	NA	NA
C37 H73 N	0.00055	0.949503	NA	level4	NA	NA	NA	NA
C27 H52 C	0.001821	3.729174	NA	level4	NA	NA	NA	NA
C18H33O2	0.000726	2.383901	NA	level4	NA	C18601	NA	NA
C24 H16 C	7.10E-05	0.170672	NA	level4	HMDB	NA	HMDB025	NA
C31 H42 N	0.002528	4.731061	NA	level4	NA	C10005	NA	NA
C27 H31 F	-0.00142	-2.54527	NA	level4	HMDB	NA	HMDB025	NA
C9 H11 N	-0.00205	-11.3381	84.69373	level2	mzCloud	NA	NA	NA
C33 H62 N	-0.00082	-1.20395	NA	level4	HMDB	NA	HMDB006	Organic ox
C21 H32 C	-2.35E-05	-0.07433	15.49781	level4	Public	NA	NA	NA
C19 H22 N	-0.00175	-4.39444	NA	level4	HMDB	NA	HMDB024	NA
C12 H17 C	-8.45E-05	-0.26399	NA	level4	HMDB	NA	HMDB025	NA
C30 H46 C	-0.00076	-1.46331	NA	level4	NA	NA	NA	NA
C21 H20 C	9.27E-05	0.206953	26.91248	level4	Public	NA	NA	NA
C8 H16 N2	0.000288	1.412176	NA	level4	HMDB	NA	HMDB024	Organic ac

C20 H19 N	-7.38E-05	-0.21879	NA	level4	HMDB	NA	HMDB025	NA	
C28 H46 C	0.000644	1.302231	NA	level4	KEGG	C08829	NA	Terpenoid	
C9 H15 N	0.000209	1.236773	NA	level4	NA	C17730	NA	NA	
C9 H12 O	-0.00027	-1.02065	NA	level4	HMDB	NA	HMDB000	Benzenoid	
C5 H4 O2	-6.32E-06	-0.06582	NA	level4	HMDB	NA	HMDB024	NA	
C7 H9 N3	0.000282	1.415225	NA	level4	HMDB	NA	HMDB025	NA	
C7 H7 N C	0.000102	0.841945	57.15356	level3	Public	C09815	HMDB000	Benzenoid	
C7 H11 N	-0.00028	-1.06447	NA	level4	HMDB	NA	HMDB024	NA	
C22 H30 N	0.00031	0.754727	NA	level4	HMDB	NA	HMDB025	NA	
C14 H11 F	-0.00099	-3.83645	NA	level4	HMDB	NA	HMDB024	NA	
C7 H11 N	0.000253	1.790826	32.22529	level3	mzCloud	C16821	NA	NA	
C20 H28 N	-0.00018	-0.49487	NA	level4	NA	C10564	NA	NA	
C6 H6 N4	0.00031	1.700217	76.86201	level2	mzCloud	C16359	HMDB000	Organohe	
C9 H10 F2	0.000846	3.203315	NA	level4	HMDB	NA	HMDB006	Nucleosid	
C35 H34 N	-0.00048	-0.778	NA	level4	NA	C21441	NA	NA	
C12 H14 N	6.47E-06	0.025874	NA	level4	HMDB	NA	HMDB024	NA	
C5 H5 Cl3	-0.00111	-4.49493	NA	level4	HMDB	NA	HMDB025	NA	
C10 H6 O	0.000175	1.232005	NA	level4	HMDB	NA	HMDB025	NA	
C29 H36 C	0.002214	4.191255	5.982994	level4	Public	NA	NA	NA	
C19 H18 N	-0.00127	-3.85349	NA	level4	HMDB	NA	HMDB024	NA	
C33 H46 C	-0.00189	-3.72317	NA	level4	HMDB	NA	HMDB024	NA	
C35 H52 N	-0.00043	-0.6868	NA	level4	HMDB	NA	HMDB025	NA	
C36 H45 N	0.003309	4.786863	NA	level4	HMDB	NA	HMDB025	NA	
C14 H18 N	-3.95E-05	-0.15062	20.52711	level4	Public	NA	NA	NA	
C14 H21 N	-5.05E-05	-0.23022	NA	level4	HMDB	NA	HMDB024	NA	
C16 H20 N	-0.00063	-1.79966	NA	level4	HMDB	NA	HMDB001	Organohe	
C13 H14 N	7.74E-05	0.314613	50	level3	Masslist	NA	HMDB001	Organic ac	
C12 H12 N	0.000202	0.778187	NA	level4	HMDB	NA	HMDB025	NA	
C16 H31 N	-0.00019	-0.64185	NA	level4	HMDB	NA	HMDB001	Lipids and	
C19 H20 C	21.98022	74222.09	24.98739	level4	Public	NA	NA	NA	
C10 H11 N	32.02655	198818.8	57.51111	level3	BMDB2	C00955	HMDB000	Organohe	
C27 H34 C	-0.00167	-3.03619	NA	level4	NA	C10560	NA	Phenylpro	
C9 H7 N C	0.000138	0.953076	94.00575	level2	mzCloud	C19434	NA	NA	
C12 H11 N	-0.00013	-0.51768	NA	level4	HMDB	NA	HMDB024	NA	
C46 H91 N	0.00214	2.965766	NA	level4	NA	NA	NA	NA	
C24 H28 C	21.98031	55478.71	3.06771	level4	Public	NA	NA	NA	
C23 H44 N	-0.00031	-0.64592	NA	level4	NA	NA	NA	NA	
C13 H27 N	0.000241	1.131587	NA	level4	NA	NA	NA	NA	
C25 H28 C	17.02692	36060.73	2.818728	level4	Public	NA	NA	NA	
C7 H8 O3	7.85E-05	0.560268	NA	level4	HMDB	NA	HMDB012	Benzenoid	
C17 H14 C	0.000336	1.126494	15.51426	level4	Public	NA	NA	NA	
C19 H20 N	-2.28E-07	-0.00064	NA	level4	HMDB	NA	HMDB024	NA	
C16 H15 C	37.9577	112282.4	9.101702	level4	Public	NA	NA	NA	
C32 H40 C	0.000127	0.173775	NA	level4	HMDB	NA	HMDB025	NA	
C21 H30 C	0.001403	3.292961	NA	level4	HMDB	NA	HMDB024	NA	
C37 H45 N	-0.0001	-0.17351	NA	level4	HMDB	NA	HMDB025	NA	
C26 H25 N	0.001455	3.281985	NA	level4	HMDB	NA	HMDB025	NA	
C26 H26 N	0.002397	4.851686	NA	level4	HMDB	NA	HMDB024	NA	
C29 H52 N	-0.00251	-4.15177	NA	level4	NA	NA	NA	NA	
C21 H27 B	0.001776	4.37222	NA	level4	NA	C14647	NA	NA	
C18 H16 C	-0.00023	-0.76848	NA	level4	HMDB	NA	HMDB006	Phenylpro	
C7 H11 N	0.000134	0.855602	48.7823	level3	mzCloud	C20828	HMDB000	Organic ac	
C44 H70 N	0.002105	2.619435	NA	level4	NA	NA	NA	NA	
C15 H23 N	0.000215	0.660005	NA	level4	HMDB	NA	HMDB001	Organic ac	
C6 H12 N	2.76E-07	0.001568	NA	level4	HMDB	NA	HMDB024	NA	

C20 H16 C	21.98306	72286.55	21.31005	level4	Public	NA	NA	NA
C33 H41 F	0.000731	0.978918	NA	level4	HMDB	NA	HMDB025	NA
C9 H15 N3	-0.00028	-1.02049	NA	level4	HMDB	NA	HMDB024	NA
C12 H16 N	0.000144	0.504544	NA	level4	KEGG	C03003	NA	NA
C29 H49 N	0.00073	1.353756	4.357066	level4	Public	NA	HMDB024	NA
C22 H32 C	0.000102	0.295738	NA	level4	HMDB	NA	HMDB026	NA
C18 H19 N	1.14E-05	0.029241	NA	level4	HMDB	NA	HMDB025	NA
C20 H34 C	21.98426	64994.93	56.74113	level3	mzCloud	NA	NA	NA
C24 H25 C	0.000832	1.696666	NA	level4	HMDB	NA	HMDB025	NA
C26 H43 B	-0.00156	-3.3538	NA	level4	NA	NA	NA	NA
C21 H32 C	-0.00188	-4.41947	NA	level4	HMDB	NA	HMDB025	NA
C33 H56 C	0.000678	1.312506	NA	level4	NA	NA	NA	NA
C22 H26 C	-0.00182	-4.5158	NA	level4	HMDB	NA	HMDB024	NA
C17 H18 N	-0.00111	-3.58856	NA	level4	HMDB	NA	HMDB025	NA
C17 H26 N	-0.00033	-1.21541	NA	level4	HMDB	NA	HMDB024	NA
C23 H23 C	-0.00114	-2.53006	NA	level4	HMDB	NA	HMDB025	NA
C20 H40 C	-9.11E-05	-0.26465	NA	level4	HMDB	NA	HMDB025	NA
C22 H25 N	0.001933	4.941764	NA	level4	HMDB	NA	HMDB025	NA
C18 H19 N	-0.0001	-0.2842	NA	level4	HMDB	NA	HMDB024	NA
C32 H38 N	-0.00255	-4.64343	NA	level4	HMDB	NA	HMDB006	Phenylpro
C15 H21 N	-0.00136	-3.06097	NA	level4	NA	C20656	NA	NA
C26 H42 C	0.000382	0.878829	NA	level4	NA	NA	NA	NA
C17 H23 N	-0.00115	-3.66262	NA	level4	HMDB	NA	HMDB025	NA
C16 H26 N	-0.00057	-1.93547	NA	level4	HMDB	NA	HMDB001	Benzenoid
C22 H33 C	21.98101	47765.45	2.520914	level4	Public	NA	NA	NA
C28 H39 N	0.000451	0.968653	NA	level4	NA	NA	NA	NA
C8 H10 O6	-0.00013	-0.64203	NA	level4	NA	C20581	NA	NA
C10 H17 N	-0.00027	-0.92529	NA	level4	HMDB	NA	HMDB025	NA
C26 H30 C	-0.00126	-2.59711	NA	level4	NA	NA	NA	NA
C15 H12 C	-0.00016	-0.59387	NA	level4	HMDB	NA	HMDB025	NA
C21 H23 N	0.000445	1.266469	NA	level4	KEGG	C03677	NA	Alkaloids
C33 H52 C	-0.00253	-4.04564	NA	level4	NA	NA	NA	NA
C9 H6 O3	8.82E-05	0.544252	83.83924	level2	mzCloud	C20414	HMDB013	Phenylpro
C19 H23 N	17.02261	51714.88	42.87016	level3	mzCloud	C09643	NA	Alkaloids
C11 H14 C	-0.00109	-3.75689	NA	level4	HMDB	NA	HMDB024	Lipids and
C20 H32	-3.93E-05	-0.14433	NA	level4	HMDB	NA	HMDB026	NA
C6 H7 N5	0.0003	1.657626	NA	level4	HMDB	NA	HMDB000	Organohe
C34 H42 C	0.000332	0.449626	NA	level4	NA	NA	NA	NA
C21 H28 F	-0.00117	-2.57722	NA	level4	HMDB	NA	HMDB025	NA
C18 H22 N	0.000301	0.741781	NA	level4	HMDB	NA	HMDB024	NA
C27 H45 N	0.000486	0.980933	57.68945	level3	Public	NA	HMDB024	NA
C9 H10 F N	-0.00102	-4.50147	NA	level4	HMDB	NA	HMDB025	NA
C20 H42 N	-4.63E-05	-0.10545	NA	level4	HMDB	NA	HMDB024	NA
C29 H29 C	0.000806	1.598567	NA	level4	HMDB	NA	HMDB025	NA
C16 H22 C	17.02464	47538.09	1.955067	level4	Public	NA	NA	NA
C22 H24 C	0.001792	4.702469	NA	level4	HMDB	NA	HMDB001	Organohe
C8 H8 O2	0.000225	1.656934	89.76687	level2	mzCloud	NA	HMDB003	Benzenoid
C6 H15 O3	-0.00057	-1.35699	NA	level4	HMDB	NA	HMDB025	NA
C28 H32 N	0.001042	2.188702	NA	level4	HMDB	NA	HMDB025	NA
C21 H27 N	-0.00119	-3.01935	NA	level4	KEGG	C03887	NA	NA
C44 H76 N	-0.00043	-0.55219	NA	level4	HMDB	NA	HMDB000	Lipids and
C20 H16 N	-0.00132	-4.16954	NA	level4	HMDB	NA	HMDB025	NA
C19 H24 C	0.001104	3.031551	5.129839	level4	Public	NA	NA	NA
C22 H24 C	0.000205	0.442356	NA	level4	NA	C16422	NA	Flavonoids
C35 H46 C	0.002557	4.301985	NA	level4	NA	NA	NA	NA
C18 H19 C	-0.00015	-0.39329	NA	level4	HMDB	NA	HMDB026	NA

C29 H32 F	0.000253	0.529277	NA	level4	HMDB	NA	HMDB025	NA
C11 H19 N	0.000209	0.801714	NA	level4	HMDB	NA	HMDB006	Lipids and
C20 H27 N	-0.00104	-3.14797	NA	level4	HMDB	NA	HMDB001	Lipids and
C5 H11 N	0.000125	1.06633	NA	level4	HMDB	NA	HMDB025	NA
C12 H13 N	0.000276	1.174378	NA	level4	HMDB	NA	HMDB024	NA
C16 H19 N	-0.00018	-0.67021	NA	level4	HMDB	NA	HMDB025	NA
C21 H24 C	-0.00204	-4.68191	NA	level4	HMDB	NA	HMDB025	NA
C15 H12 C	0.000112	0.317139	NA	level4	NA	NA	NA	NA
C19 H27 N	-0.00048	-1.22452	NA	level4	HMDB	NA	HMDB001	Organohe
C14 H17 F	0.001523	4.942505	NA	level4	HMDB	NA	HMDB025	NA
C29 H42 C	0.001628	2.874447	NA	level4	KEGG	C08847	NA	Terpenoid
C21 H25 N	-0.00023	-0.6765	NA	level4	HMDB	NA	HMDB025	NA
C19 H18 C	21.98367	64259.01	36.37668	level4	Public	NA	NA	NA
C24 H28 N	0.000559	1.246348	NA	level4	HMDB	NA	HMDB025	NA
C6 H4 N2	8.13E-05	0.597532	NA	level4	HMDB	NA	HMDB025	NA
C24 H48 N	0.00063	1.199728	NA	level4	NA	NA	NA	NA
C33 H34 C	0.002321	4.281242	NA	level4	HMDB	NA	HMDB025	NA
C9 H12 N4	-0.00093	-4.49143	NA	level4	HMDB	NA	HMDB024	NA
C14 H14 C	17.02675	51282.01	3.265171	level4	Public	NA	NA	NA
C45 H80 N	0.0029	3.512985	NA	level4	NA	NA	NA	NA
C9 H13 N	2.41E-05	0.131572	NA	level4	HMDB	NA	HMDB025	NA
C22 H39 N	-0.00013	-0.34409	NA	level4	HMDB	NA	HMDB025	NA
C45 H72 C	0.001286	1.402786	NA	level4	NA	NA	NA	NA
C11 H17 N	-0.00017	-0.59403	NA	level4	HMDB	NA	HMDB024	NA
C8 H8 O4	0.000189	1.127471	90.22484	level2	mzCloud	NA	HMDB006	Benzenoid
C11 H12 C	-0.00223	-9.93369	54.30006	level3	mzCloud	C00482	HMDB003	Phenylpro
C10 H15 N	-4.45E-05	-0.24589	NA	level4	HMDB	NA	HMDB006	Benzenoid
C28 H58 N	0.001137	2.062673	NA	level4	HMDB	NA	HMDB001	Lipids and
C44 H86 N	-0.00033	-0.41322	NA	level4	HMDB	NA	HMDB000	Lipids and
C29 H33 N	-0.00022	-0.43078	NA	level4	HMDB	NA	HMDB001	Organohe
C H5 N O3	0.00014	1.261725	NA	level4	HMDB	NA	HMDB024	NA
C26 H20 N	0.001769	4.91072	NA	level4	HMDB	NA	HMDB024	NA
C27 H44 C	0.000471	0.980743	NA	level4	HMDB	NA	HMDB024	NA
C11 H11 N	0.000157	0.721627	NA	level4	HMDB	NA	HMDB025	NA
C12 H19 N	0.000229	0.948454	NA	level4	HMDB	NA	HMDB024	NA
C21 H40 C	-0.00017	-0.49604	NA	level4	NA	NA	NA	NA
C17 H20 N	-0.00192	-4.94016	NA	level4	HMDB	NA	HMDB024	NA
C18 H19 N	-0.00136	-3.65509	NA	level4	HMDB	NA	HMDB006	Benzenoid
C15 H12 C	7.07E-05	0.260041	NA	level4	HMDB	NA	HMDB025	NA
C26 H34 C	0.000185	0.40414	4.862537	level4	Public	NA	NA	NA
C31 H61 N	0.000639	1.290542	NA	level4	NA	NA	NA	NA
C33 H38 N	0.001208	2.005286	NA	level4	NA	C19705	NA	NA
C28 H40 N	0.000437	0.79624	NA	level4	HMDB	NA	HMDB024	NA
C32 H57 C	0.002163	3.106184	NA	level4	NA	NA	NA	NA
C18 H28 N	-4.51E-05	-0.141	NA	level4	HMDB	NA	HMDB025	NA
C27 H43 N	-0.00243	-4.77673	NA	level4	HMDB	NA	HMDB025	NA
C12 H10 F	0.00257	4.500878	NA	level4	HMDB	NA	HMDB024	NA
C27 H40 C	-5.76E-05	-0.13981	NA	level4	HMDB	NA	HMDB001	Lipids and
C22 H27 F	0.001068	2.682755	NA	level4	HMDB	NA	HMDB001	Organohe
C22 H24 C	0.001834	4.073511	NA	level4	HMDB	NA	HMDB025	NA
C42 H81 N	0.000368	0.568206	89.64435	level2	mzCloud	NA	HMDB000	Lipids and
C21 H28 C	-6.17E-05	-0.18799	NA	level4	HMDB	NA	HMDB001	Lipids and
C22 H23 C	0.001153	2.40619	NA	level4	HMDB	NA	HMDB003	Phenylpro
C35 H36 F	-0.00309	-4.86372	NA	level4	NA	C15620	NA	NA
C18 H16 C	-0.00018	-0.53795	43.23349	level3	Public	NA	NA	NA
C39 H70 C	0.000913	1.515816	NA	level4	NA	NA	NA	NA
C13 H12 N	-0.00116	-4.84829	NA	level4	HMDB	NA	HMDB025	NA

C21 H42 N	0.000113	0.249741	NA	level4	NA	NA	NA	NA
C30 H44 C	0.000115	0.263713	NA	level4	NA	NA	NA	NA
C22 H31 N	-0.00031	-0.72576	NA	level4	HMDB	NA	HMDB025	NA
C7 H9 N3	-0.00109	-4.71224	NA	level4	HMDB	NA	HMDB024	NA
C16 H14 C	2.38E-05	0.075646	NA	level4	HMDB	NA	HMDB024	NA
C13 H14 N	-0.00029	-1.3552	NA	level4	HMDB	NA	HMDB025	NA
C21 H20 N	-0.00125	-2.98391	NA	level4	HMDB	NA	HMDB024	NA
C31 H45 F	0.000486	0.873633	NA	level4	HMDB	NA	HMDB024	NA
C20 H40 C	-0.00013	-0.38357	NA	level4	KEGG	C02982	NA	NA
C14 H14 C	0.001665	5.625317	43.05987	level3	mzCloud	C18739	HMDB003	Benzenoid
C16 H21 N	0.001224	3.883434	NA	level4	HMDB	NA	HMDB001	Organohe
C21 H36 C	-2.18E-05	-0.06188	NA	level4	HMDB	NA	HMDB025	NA
C14 H12 C	0.000165	0.843401	NA	level4	HMDB	NA	HMDB024	NA
C13 H19 N	17.02651	63265.88	9.144351	level4	Public	NA	NA	NA
C15 H17 N	0.001283	4.072534	NA	level4	HMDB	NA	HMDB024	NA
C21 H28 C	-0.00021	-0.58178	2.03579	level4	BMDB	C00762	HMDB000	Lipids and
C19 H20 C	21.98028	66986.28	43.78296	level3	Public	NA	NA	NA
C17 H26 C	37.95411	116361.9	4.444607	level4	Public	NA	NA	NA
C19 H12 C	-0.0008	-2.5071	NA	level4	NA	C12390	NA	NA
C20 H42 C	-4.34E-07	-0.0012	NA	level4	HMDB	NA	HMDB024	NA
C11 H14 N	-5.54E-05	-0.20521	NA	level4	NA	C19977	NA	Fungal tox
C22 H22 C	0.000372	0.777903	8.34783	level4	Public	NA	NA	NA
C16 H32 C	17.02644	66447.17	93.64248	level1	BMDB2	C00249	HMDB000	Lipids and
C11 H24 C	0.000755	3.695826	NA	level4	HMDB	NA	HMDB024	NA
C16 H26 C	2.97E-05	0.105264	NA	level4	HMDB	NA	HMDB025	NA
C15 H20 C	37.9545	128170.1	8.271094	level4	Public	NA	NA	NA
C34 H52 C	0.000584	0.992888	NA	level4	HMDB	NA	HMDB001	Lipids and
C37 H72 N	0.001464	2.075839	NA	level4	NA	NA	NA	NA
C19 H28 C	37.95397	118532.5	10.29184	level4	Public	NA	NA	NA
C18 H20 N	0.000177	0.452339	NA	level4	HMDB	NA	HMDB006	Organic ox
C6 H10 N3	0.000103	0.470917	NA	level4	HMDB	NA	HMDB024	NA
S Se	2.77E-06	0.0248	NA	level4	HMDB	NA	HMDB001	Homogen
C16 H21 N	-0.00013	-0.53535	41.20362	level4	Public	NA	NA	NA
C38 H74 N	-9.11E-05	-0.12948	NA	level4	NA	NA	NA	NA
C27 H35 F	0.002205	3.521341	NA	level4	HMDB	NA	HMDB025	NA
C17 H34 C	-18.0106	-59589.2	64.07035	level2	Public	NA	HMDB030	NA
C21 H29 N	-0.00013	-0.37	NA	level4	HMDB	NA	HMDB024	NA
C24 H24 C	0.000844	1.5752	NA	level4	HMDB	NA	HMDB003	Phenylpro
C24 H48 C	0.000164	0.410573	NA	level4	NA	NA	NA	NA
C18 H37 N	-0.00019	-0.64663	96.04174	level2	mzCloud	C00319	HMDB000	Organic ni
C12 H12 C	9.72E-05	0.441794	NA	level4	HMDB	NA	HMDB025	NA
C16 H20 C	5.53E-05	0.212589	NA	level4	KEGG	C09695	NA	Terpenoid
C25 H28 C	17.02756	33773.91	7.214684	level4	Public	NA	NA	NA
C21 H30 C	0.000291	0.658179	NA	level4	NA	NA	NA	NA
C8 H10 N2	0.000215	1.004211	3.014961	level4	Public	NA	NA	NA
C26 H37 N	0.000612	1.151522	NA	level4	HMDB	NA	HMDB025	NA
C26 H36 B	-0.00162	-3.09968	NA	level4	NA	NA	NA	NA
C22 H30 C	5.48E-05	0.152961	NA	level4	HMDB	NA	HMDB025	NA
C44 H85 C	-0.00095	-1.18238	NA	level4	NA	NA	NA	NA
C13 H15 C	0.001388	4.921818	NA	level4	HMDB	NA	HMDB025	NA
C21 H18 C	0.00155	3.733901	NA	level4	HMDB	NA	HMDB024	NA
C3 H9 O4	0.00025	1.787513	NA	level4	HMDB	NA	HMDB025	NA
C16 H18 N	0.000312	0.868756	NA	level4	HMDB	NA	HMDB024	NA
C38 H69 N	-0.00194	-3.30273	NA	level4	HMDB	NA	HMDB024	NA
C19 H14 C	-0.0006	-1.70747	NA	level4	NA	C12600	NA	NA
C19 H17 N	-0.00075	-2.3316	NA	level4	HMDB	NA	HMDB006	Organohe
C22 H31 N	0.000733	2.051786	3.016901	level4	Public	C07360	HMDB001	Benzenoid
C36 H48 N	0.002343	3.505848	NA	level4	HMDB	NA	HMDB024	NA

C15 H19 N	-2.39E-06	-0.00676	NA	level4	HMDB	NA	HMDB025	NA
C31 H36 C	0.001439	2.532911	NA	level4	NA	C17812	NA	NA
C46 H80 N	0.001774	2.159734	NA	level4	NA	NA	NA	NA
C25 H28 C	0.002418	4.510493	NA	level4	NA	NA	NA	NA
C26 H27 N	-0.00239	-4.44002	NA	level4	HMDB	NA	HMDB025	NA
C5 H6 O5	0.000186	1.273428	69.68071	level2	mzCloud	C00026	HMDB000	Organic ac
H3 O4 P	0.000158	1.60858	85.44635	level1	Decode	C00009	HMDB000	Homogen
C41 H83 C	-0.00198	-2.64243	NA	level4	NA	NA	NA	NA
C17 H16 C	17.02645	51270.65	17.20367	level4	Public	NA	NA	NA
C21 H28 C	-4.51E-05	-0.10633	NA	level4	HMDB	NA	HMDB024	NA
C17 H16 N	-0.00025	-0.71907	NA	level4	HMDB	NA	HMDB006	Organohe
C38 H48 C	-0.00137	-1.66131	NA	level4	NA	NA	NA	NA
C37 H64 C	-0.002	-3.39809	NA	level4	HMDB	NA	HMDB000	Lipids and
C17 H19 C	-0.00133	-4.40621	NA	level4	NA	C18425	NA	NA
C23 H34 C	-1.32E-05	-0.03535	NA	level4	HMDB	NA	HMDB025	NA
C12 H18 C	0.000281	1.244675	NA	level4	HMDB	NA	HMDB026	NA
C6 H14 O	0.000205	1.529044	NA	level4	HMDB	NA	HMDB024	NA
C40 H82 N	0.000209	0.289991	NA	level4	NA	NA	NA	NA
C34 H46 N	0.002673	4.40834	NA	level4	HMDB	NA	HMDB025	NA
C32 H44 C	0.002368	4.383507	NA	level4	HMDB	NA	HMDB001	Lipids and
C17 H14 C	9.96E-05	0.242903	NA	level4	NA	NA	NA	NA
C18 H27 N	37.95417	118165.9	42.72632	level3	Public	NA	NA	NA
C38 H48 C	0.001342	1.660548	NA	level4	HMDB	NA	HMDB025	NA
C18 H21 N	-0.00036	-1.21157	32.00217	level4	Public	NA	HMDB024	NA
C32 H37 C	0.001846	2.545843	NA	level4	NA	NA	NA	NA
C27 H34 N	0.001783	3.494487	NA	level4	HMDB	NA	HMDB024	NA
C43 H64 N	0.002928	3.483308	NA	level4	HMDB	NA	HMDB025	NA
C8 H18 N6	0.00093	3.547255	NA	level4	HMDB	NA	HMDB025	NA
C18 H31 N	0.002098	3.86306	NA	level4	NA	C18034	NA	NA
C10 H9 N	-0.00014	-0.53676	NA	level4	HMDB	NA	HMDB025	NA
C19 H22 N	0.001315	3.551444	NA	level4	HMDB	NA	HMDB025	NA
C25 H21 F	-0.00116	-2.39035	NA	level4	HMDB	NA	HMDB024	NA
C25 H40 N	0.00011	0.26343	NA	level4	HMDB	NA	HMDB025	NA
C23 H34 C	17.02862	31877.16	6.862674	level4	Public	NA	NA	NA
C28 H24 C	0.001583	4.035786	NA	level4	HMDB	NA	HMDB024	NA
C25 H46 C	0.001829	3.206564	NA	level4	NA	NA	NA	NA
C14 H10 N	0.000116	0.455511	NA	level4	HMDB	NA	HMDB024	Organohe
C21 H30 C	17.0285	35911.94	1.901547	level4	Public	NA	NA	NA
C33 H34 N	0.000382	0.65593	62.1032	level2	BMDB	C00500	HMDB000	Organohe
C25 H31 N	-0.00542	-14.5196	32.72571	level3	mzCloud	NA	NA	NA
C25 H31 N	0.000572	1.229309	NA	level4	HMDB	NA	HMDB024	NA
C28 H29 N	0.002708	4.627748	NA	level4	NA	C11375	NA	NA
C24 H30 C	-6.25E-05	-0.15079	NA	level4	HMDB	NA	HMDB001	Lipids and
C22 H24 C	17.02629	40914.11	3.238293	level4	Public	NA	NA	NA
C19 H17 N	-0.00148	-3.91225	NA	level4	HMDB	NA	HMDB024	NA
C28 H32 C	-0.00049	-0.80304	1.923723	level4	Public	NA	NA	NA
C38 H48 N	-0.00385	-4.58502	NA	level4	NA	C12007	NA	NA
C8 H15 N	-0.00021	-0.89824	NA	level4	KEGG	C01133	NA	NA
C28 H44	0.000184	0.484534	NA	level4	HMDB	NA	HMDB025	NA
C18 H20 C	-0.00121	-3.82075	5.889312	level4	Public	NA	NA	NA
C15 H18 C	-1.27E-05	-0.04854	NA	level4	HMDB	NA	HMDB025	NA

C7 H6 O2	-18.0104	-147582	96.90427	level1	BMDB	C00633	HMDB001	Organic o
C21 H16 N	0.000865	2.387987	NA	level4	KEGG	C06327	NA	Alkaloids
C43 H84 N	-0.00034	-0.43354	NA	level4	HMDB	NA	HMDB025	NA
C9 H8 O2	0.00023	1.552183	NA	level4	HMDB	NA	HMDB025	NA
C23 H22 N	-0.00039	-0.86781	NA	level4	HMDB	NA	HMDB024	NA
C20 H28 N	0.00069	1.582315	NA	level4	HMDB	NA	HMDB024	NA
C18 H21 N	-0.00018	-0.58576	NA	level4	HMDB	NA	HMDB024	NA
C21 H39 N	-18.0106	-43162.7	9.112441	level4	Public	NA	NA	NA
C20 H28 O	-2.01E-05	-0.06711	NA	level4	HMDB	NA	HMDB025	NA
C18 H17 F	0.001057	2.4795	NA	level4	HMDB	NA	HMDB024	NA
C48 H87 O	0.003174	3.714005	NA	level4	NA	NA	NA	NA
C21 H37 N	-0.00249	-4.93954	NA	level4	HMDB	NA	HMDB024	NA
C13 H21 N	8.17E-05	0.259322	NA	level4	NA	C17364	NA	NA
C12 H20 O	0.000105	0.493964	NA	level4	HMDB	NA	HMDB025	NA
C9 H10 O5	-4.11E-05	-0.2076	82.17247	level2	mzCloud	C10833	HMDB000	Benzenoid
C19 H20 F	0.001068	2.560792	NA	level4	HMDB	NA	HMDB006	Benzenoid
C17 H19 N	-0.00018	-0.6147	NA	level4	HMDB	NA	HMDB006	Alkaloids a
C7 H7 N O	0.000165	0.974412	50	level3	Masslist	NA	HMDB000	Organic ac
C17 H19 N	-0.00091	-2.34108	NA	level4	HMDB	NA	HMDB025	NA
C23 H29 F	-0.00054	-1.1686	NA	level4	HMDB	NA	HMDB026	NA
C8 H6 O4	0.000215	1.29354	NA	level4	HMDB	NA	HMDB000	Benzenoid
C8 H11 N	0.000272	1.353488	NA	level4	HMDB	NA	HMDB025	NA
C25 H19 N	0.001349	3.174248	NA	level4	HMDB	NA	HMDB025	NA
C14 H26 N	0.000711	1.859556	NA	level4	NA	C21747	NA	NA
C39 H51 N	0.003143	4.752692	NA	level4	NA	C20549	NA	NA
C22 H31 N	0.001663	3.947485	NA	level4	HMDB	NA	HMDB025	NA
C12 H12 N	-0.00105	-4.93338	NA	level4	HMDB	NA	HMDB024	NA
C15 H22 O	4.01E-05	0.150664	47.22125	level3	mzCloud	NA	NA	NA
C21 H43 N	0.001468	3.075992	NA	level4	HMDB	NA	HMDB025	NA
C8 H13 N	6.03E-05	0.297068	NA	level4	HMDB	NA	HMDB024	Organic ac
C10 H8 O3	17.02673	96716.79	27.95239	level4	Public	C03081	HMDB005	Phenylpro
C6 H10 O4	5.76E-05	0.394551	NA	level4	HMDB	NA	HMDB025	NA
C15 H18 O	-3.31E-05	-0.09685	NA	level4	HMDB	NA	HMDB024	Organic o
C12 H16 O	21.98075	76299.64	3.99432	level4	Public	NA	NA	NA
C29 H41 F	0.002392	4.658989	NA	level4	HMDB	NA	HMDB001	Alkaloids a
C35 H68 N	-0.00044	-0.64597	NA	level4	NA	NA	NA	NA
C13 H17 N	-5.15E-06	-0.02052	NA	level4	KEGG	C01657	NA	NA
C18 H16 O	32.02639	93075.73	28.07604	level4	Public	NA	NA	NA
C27 H28 O	-0.00238	-4.50882	NA	level4	KEGG	C09973	NA	NA
C21 H22 O	0.000185	0.443176	25.90862	level4	Public	NA	NA	NA
C36 H65 O	-0.00035	-0.46752	NA	level4	NA	NA	NA	NA
C20 H25 N	-0.00158	-4.29985	NA	level4	HMDB	NA	HMDB024	NA
C50 H81 N	0.003977	3.840399	NA	level4	HMDB	NA	HMDB024	NA
C29 H51 O	0.001367	2.315315	NA	level4	NA	NA	NA	NA
C12 H15 N	0.000294	1.328	23.12465	level4	Public	NA	HMDB024	NA
C19 H21 N	0.005967	13.46571	47.11937	level3	mzCloud	C00415	HMDB000	Organic ac
C21 H18 O	0.001706	4.816755	NA	level4	HMDB	NA	HMDB006	Organic o
C8 H11 N	0.000175	0.74943	NA	level4	HMDB	NA	HMDB000	Organic ac
C18 H37 N	4.69E-05	0.118742	NA	level4	NA	NA	NA	NA
C23 H22 O	0.000549	1.174741	NA	level4	HMDB	NA	HMDB024	NA
C25 H35 N	3.02E-05	0.065504	NA	level4	HMDB	NA	HMDB024	NA
C37 H48 N	-0.00099	-1.33942	NA	level4	HMDB	NA	HMDB006	Organic ac
C25 H31 N	0.000605	1.1968	NA	level4	KEGG	C09330	NA	Alkaloids
C14 H10 N	-0.00107	-2.64357	NA	level4	NA	C16413	NA	NA

C29 H45 N	0.000861	1.657919	NA	level4	HMDB	NA	HMDB026	NA
C13 H16 N	0.000118	0.448606	96.52022	level2	mzCloud	C04148	HMDB000	Organic ac
C28 H36 C	0.002342	4.536879	9.189743	level4	Public	NA	NA	NA
C8 H13 N	0.000412	2.655038	60.58206	level2	mzCloud	C10129	HMDB003	Alkaloids a
C11 H15 N	0.000911	3.597598	NA	level4	HMDB	NA	HMDB025	NA
C31 H36 N	0.001132	2.08067	NA	level4	HMDB	NA	HMDB024	NA
C26 H44 N	0.00075	1.43807	86.71979	level2	BMDB2	NA	NA	NA
C21 H24 C	-1.78E-05	-0.04781	NA	level4	HMDB	NA	HMDB000	Phenylpro
C19 H22 F	-0.00177	-4.71236	NA	level4	HMDB	NA	HMDB001	Organohe
C24 H48 N	-0.00028	-0.52928	NA	level4	NA	NA	NA	NA
C27 H40 C	-0.00046	-1.07282	NA	level4	HMDB	NA	HMDB024	NA
C57 H86 C	-0.00098	-1.11022	NA	level4	NA	C15952	NA	NA
C21 H18 C	0.000113	0.262247	67.6849	level2	Public	NA	NA	NA
C15 H13 F	0.000289	1.110115	NA	level4	HMDB	NA	HMDB006	Benzenoid
C20 H30 C	-0.00038	-1.09503	NA	level4	HMDB	NA	HMDB025	NA
C20 H31 C	-0.00134	-2.83048	NA	level4	HMDB	NA	HMDB025	NA
C28 H48 C	0.000529	1.101613	NA	level4	NA	C19873	NA	NA
C3 H3 N C	1.97E-05	0.194926	NA	level4	HMDB	NA	HMDB024	NA
C32 H38 N	37.95458	69479.14	11.31953	level4	Public	NA	NA	NA
C44 H40 C	-0.00195	-2.79446	NA	level4	NA	NA	NA	NA
C20 H12 C	-0.00083	-2.91022	NA	level4	HMDB	NA	HMDB024	NA
C14 H24 C	-3.47E-05	-0.15473	NA	level4	NA	NA	NA	NA
C7 H8 O7	0.000766	3.752147	NA	level4	HMDB	NA	HMDB025	NA
C32 H46 C	-0.00179	-2.79765	NA	level4	HMDB	NA	HMDB025	NA
C16 H15 F	-4.67E-05	-0.13126	NA	level4	HMDB	NA	HMDB024	NA
C16 H26 C	0.000176	0.751393	NA	level4	NA	NA	NA	NA
C22 H43 C	-0.00051	-1.05587	NA	level4	NA	NA	NA	NA
C18 H23 N	-0.00026	-0.73353	NA	level4	HMDB	NA	HMDB025	NA
C10 H8	9.35E-05	0.730404	NA	level4	HMDB	NA	HMDB024	NA
C18 H20 N	-9.75E-05	-0.29726	NA	level4	HMDB	NA	HMDB025	NA
C30 H58 N	0.001062	1.749284	NA	level4	NA	NA	NA	NA
C18 H20 N	37.95528	93001.01	4.498836	level4	Public	NA	NA	NA
C13 H16 C	21.98221	87196.48	9.779308	level4	Public	NA	HMDB003	Phenylpro
C26 H48 N	0.000547	0.941205	NA	level4	NA	NA	NA	NA
C15 H10 C	-0.00091	-2.73097	NA	level4	HMDB	NA	HMDB024	NA
C23 H30 C	0.001821	3.655873	8.144316	level4	Public	NA	NA	NA
C28 H30 C	-0.00074	-1.71697	NA	level4	HMDB	NA	HMDB025	NA
C25 H33 C	0.001926	4.011054	NA	level4	HMDB	NA	HMDB024	NA
C27 H42 F	-7.77E-05	-0.16585	NA	level4	NA	NA	NA	NA
C9 H20 N	-0.00099	-3.45878	NA	level4	NA	C19014	NA	NA
C15 H23 N	17.02648	60557.41	23.38306	level4	Public	NA	NA	NA
C15 H18 N	-2.89E-05	-0.10561	NA	level4	HMDB	NA	HMDB024	NA
C14 H22 C	17.02647	59503.63	16.45682	level4	Public	NA	NA	NA
C20 H36 C	1.85E-06	0.00519	43.93631	level3	mzCloud	NA	NA	NA
C30 H57 N	0.000454	0.94618	NA	level4	NA	NA	NA	NA
C43 H76 N	-8.38E-06	-0.01095	NA	level4	NA	NA	NA	NA
C9 H19 N	0.00034	1.536439	NA	level4	HMDB	NA	HMDB024	NA
C15 H18 C	7.80E-06	0.026785	NA	level4	HMDB	NA	HMDB025	NA
C8 H15 N	-0.00026	-0.8631	NA	level4	HMDB	NA	HMDB000	Organic ox
C28 H48 C	-0.0006	-1.102	NA	level4	NA	NA	NA	NA
C9 H15 N3	0.000247	1.3635	NA	level4	HMDB	NA	HMDB024	NA
C13 H20 C	-7.56E-05	-0.3149	NA	level4	NA	NA	NA	NA
C13 H14 C	-0.00013	-0.50279	58.35939	level3	mzCloud	C16765	HMDB004	Organohe
C15 H16 N	-3.34E-05	-0.09599	NA	level4	KEGG	C08562	NA	Amino aci
C15 H20 C	-8.78E-05	-0.31358	NA	level4	HMDB	NA	HMDB025	NA

C5 H9 N C	0.000155	0.953809	NA	level4	HMDB	NA	HMDB000	Organosul
C33 H38 C	-0.00026	-0.48526	NA	level4	HMDB	NA	HMDB025	NA
C18 H26 C	-0.00024	-0.78724	NA	level4	HMDB	NA	HMDB025	NA
C14 H14 C	-0.00028	-1.06898	NA	level4	HMDB	NA	HMDB001	Organohe
C17 H22 N	-0.00134	-4.06924	NA	level4	HMDB	NA	HMDB025	NA
C27 H29 N	-0.00062	-1.09418	NA	level4	HMDB	NA	HMDB025	NA
C28 H28 N	-0.00199	-4.56224	NA	level4	HMDB	NA	HMDB025	NA
C18 H27 N	-0.00038	-1.23391	30.00737	level4	Public	C06866	HMDB000	Benzenoid
C25 H44 C	0.000126	0.257519	NA	level4	NA	NA	NA	NA
C38 H49 N	-0.00067	-0.94063	NA	level4	HMDB	NA	HMDB025	NA
C7 H15 N	0.000177	1.097004	NA	level4	HMDB	NA	HMDB024	NA
C20 H18 C	0.000782	2.113987	NA	level4	NA	C20452	NA	NA
C15 H10 C	-2.87E-06	-0.00751	NA	level4	HMDB	NA	HMDB004	Phenylpro
C17 H26 C	0.000128	0.328332	NA	level4	HMDB	NA	HMDB024	NA
C16 H20 N	0.00147	3.672921	NA	level4	HMDB	NA	HMDB024	NA
C8 H8 O3	0.000177	1.16482	92.5567	level1	BMDB	C12064	NA	NA
C38 H72 N	-0.00121	-1.62197	69.82785	level2	Public	C06838	HMDB001	Organic ox
C2 H Cl5	0.000119	0.596395	NA	level4	HMDB	NA	HMDB025	NA
C20 H30 C	-0.00015	-0.5071	58.30836	level4	Public	NA	HMDB024	NA
C17 H12 C	-0.00066	-2.8572	NA	level4	HMDB	NA	HMDB024	NA
C20 H34 C	-72.0422	-203371	16.87283	level4	Public	NA	NA	NA
C17 H34 C	-4.82E-05	-0.1596	92.57043	level1	BMDB	NA	NA	NA
C26 H43 C	-0.00084	-1.59336	NA	level4	NA	NA	NA	NA
C10 H15 N	-0.00547	-16.9895	31.25656	level3	mzCloud	C00364	HMDB000	Nucleoside
C32 H52 C	0.00118	2.152469	NA	level4	NA	NA	NA	NA
C32 H42 C	0.001781	2.672866	NA	level4	NA	NA	NA	NA
C16 H20 C	37.95731	123187.6	7.687808	level4	Public	NA	NA	NA
C30 H36 C	0.002385	4.415528	5.72943	level4	Public	NA	NA	NA
C41 H74 N	0.002326	3.145326	NA	level4	NA	NA	NA	NA
C16 H23 N	0.000164	0.45933	NA	level4	KEGG	C09914	NA	NA
C24 H32 C	-0.00194	-4.32168	NA	level4	HMDB	NA	HMDB025	NA
C15 H18 C	-0.00027	-1.15719	NA	level4	HMDB	NA	HMDB025	NA
C30 H42 C	0.00166	2.871227	NA	level4	NA	NA	NA	NA
C18 H21 N	-0.00155	-4.58076	NA	level4	HMDB	NA	HMDB024	NA
C17 H20 N	-5.28E-05	-0.14506	NA	level4	HMDB	NA	HMDB001	Benzenoid
C16 H32 C	3.40E-05	0.132637	19.38557	level4	Public	NA	HMDB003	Lipids and
C14 H12 C	2.09E-05	0.085766	NA	level4	HMDB	NA	HMDB025	NA
C30 H50 C	-0.00323	-4.52701	NA	level4	HMDB	NA	HMDB025	NA
C9 H6 O3	0.000199	1.229405	76.17083	level2	mzCloud	C09315	HMDB002	Phenylpro
C27 H31 N	0.00201	3.915668	NA	level4	NA	NA	NA	NA
C49 H55 N	0.003699	4.197072	NA	level4	HMDB	NA	HMDB025	NA
C8 H14 O2	0.000171	1.200091	NA	level4	HMDB	NA	HMDB001	Lipids and
C18 H25 N	5.62E-05	0.207189	NA	level4	HMDB	NA	HMDB000	Alkaloids a
C27 H44 C	0.000591	1.153103	NA	level4	HMDB	NA	HMDB024	NA
C25 H38 C	-1.46E-05	-0.03481	NA	level4	HMDB	NA	HMDB000	Organohe
C9 H11 F N	-0.00112	-4.57139	NA	level4	HMDB	NA	HMDB024	NA
C39 H72 C	17.02678	27438.75	0.450638	level4	Public	NA	HMDB000	Lipids and
C21 H21 C	-0.00038	-0.81377	NA	level4	HMDB	NA	HMDB003	Phenylpro
C21 H35 N	-0.00162	-3.81192	NA	level4	HMDB	NA	HMDB024	NA
C26 H28 C	17.02704	36368.71	20.67364	level4	Public	NA	NA	NA
C11 H16 N	0.000315	1.511244	71.59663	level2	mzCloud	C07474	HMDB001	Alkaloids a
C19 H27 N	0.001383	2.998975	NA	level4	HMDB	NA	HMDB025	NA
C21 H36 C	37.95453	79041.44	8.324153	level4	Public	NA	NA	NA
C14 H18 N	-0.00028	-0.95845	NA	level4	HMDB	NA	HMDB025	NA
C24 H37 N	-0.00013	-0.36499	NA	level4	HMDB	NA	HMDB025	NA

C19 H17 N	-0.00014	-0.40246	NA	level4	HMDB	NA	HMDB024	NA
C23 H42 C	-0.00145	-2.94282	NA	level4	NA	NA	NA	NA
C5 H8 O	0.00015	1.783734	NA	level4	HMDB	NA	HMDB024	NA
C6 H8 N2	0.000196	1.258762	45.43006	level3	mzCloud	NA	HMDB000	Organohe
C22 H20 C	-0.00112	-3.7348	NA	level4	NA	C15179	NA	NA
C22 H30 C	-0.00225	-4.46545	NA	level4	HMDB	NA	HMDB001	Benzenoid
C6 H7 N3	0.000215	1.163082	NA	level4	HMDB	NA	HMDB024	NA
C26 H34 C	-0.00185	-3.4441	NA	level4	HMDB	NA	HMDB024	NA
C15 H18 N	-0.00025	-0.78886	NA	level4	HMDB	NA	HMDB025	NA
C8 H16 N4	-0.0025	-11.5864	63.62766	level2	mzCloud	NA	HMDB000	Organic ac
C3 H7 O5	0.000157	1.022598	NA	level4	HMDB	NA	HMDB024	NA
C30 H52 C	-3.04E-05	-0.06602	NA	level4	HMDB	NA	HMDB025	NA
C14 H15 N	-0.00116	-4.12375	NA	level4	HMDB	NA	HMDB025	NA
C25 H32 N	-0.00124	-2.7033	NA	level4	HMDB	NA	HMDB025	NA
C21 H34 C	17.02445	36832.71	11.41411	level4	Public	NA	NA	NA
C17 H32 C	2.20E-05	0.082037	NA	level4	HMDB	NA	HMDB025	NA
C22 H40 C	0.001231	2.402868	NA	level4	NA	NA	NA	NA
C18 H32 N	0.00026	0.585056	NA	level4	HMDB	NA	HMDB025	NA
C19 H32 C	-8.16E-05	-0.26473	NA	level4	HMDB	NA	HMDB024	NA

C41 H48 C	0.003349	4.67592	NA	level4	HMDB	NA	HMDB025	NA
C36 H35 F	-0.00191	-3.10367	NA	level4	HMDB	NA	HMDB025	NA
C7 H9 N C	7.42E-05	0.53332	NA	level4	HMDB	NA	HMDB025	NA
C14 H19 N	-0.00013	-0.53401	NA	level4	HMDB	NA	HMDB025	NA
C40 H56 C	-0.00011	-0.19051	NA	level4	HMDB	NA	HMDB026	NA
C22 H28 C	21.98205	54386.3	50.38671	level3	Public	NA	NA	NA
C16 H26 C	17.02647	54194.58	42.87717	level4	Public	NA	NA	NA
C6 H12 N2	-2.35E-05	-0.1467	NA	level4	HMDB	NA	HMDB024	NA
C22 H24 C	21.98027	59702.65	8.712029	level4	Public	NA	NA	NA
C20 H16 C	0.000842	2.629983	NA	level4	NA	NA	NA	NA
C13 H13 F	-0.00102	-2.6311	NA	level4	HMDB	NA	HMDB025	NA
C24 H35 N	-0.00086	-1.82251	NA	level4	HMDB	NA	HMDB001	Organic ac
C22 H27 C	0.001963	4.583789	NA	level4	HMDB	NA	HMDB025	NA
C24 H27 N	0.001743	4.177076	NA	level4	HMDB	NA	HMDB025	NA
C7 H6 O6	-0.00058	-2.67419	NA	level4	HMDB	NA	HMDB005	Organic ac
C12 H10 C	0.00028	1.387305	62.90727	level2	Public	C18750	HMDB003	Benzenoid
C27 H24 N	0.00045	0.898809	NA	level4	HMDB	NA	HMDB024	NA
C15 H31 N	0.000167	0.575931	NA	level4	HMDB	NA	HMDB024	NA
C22 H16 N	0.001884	4.904025	NA	level4	HMDB	NA	HMDB024	NA
C27 H42 C	-18.0105	-43470.7	49.79405	level3	mzCloud	C08898	NA	Steroids
C13 H20 C	0.000111	0.494821	46.62435	level4	Public	NA	NA	NA
C24 H26 C	-0.00135	-3.7327	NA	level4	NA	NA	NA	NA
C9 H16 O3	0.000299	1.739258	NA	level4	NA	NA	NA	NA
C33 H52 C	0.000388	0.65497	NA	level4	HMDB	NA	HMDB024	NA
C23 H32 C	17.02641	45741.64	25.38179	level4	Public	NA	NA	NA
C37 H56 C	0.000517	0.783315	NA	level4	HMDB	NA	HMDB024	NA
C14 H25 N	-18.0106	-66416.1	8.34732	level4	Public	NA	NA	NA
C35 H34 N	0.000584	0.996401	NA	level4	HMDB	NA	HMDB025	NA
C17 H16 C	-5.87E-05	-0.18747	NA	level4	HMDB	NA	HMDB001	Organohe
C17 H20 N	-3.04E-05	-0.08087	68.34084	level1	BMDB	C00255	HMDB000	Organohe

C37 H41 N	0.000976	1.601737	NA	level4	HMDB	NA	HMDB001	Organic o:
C21 H18 F	-0.00212	-4.89636	NA	level4	HMDB	NA	HMDB025	NA
C22 H41 N	0.00026	0.602843	NA	level4	NA	NA	NA	NA
C20 H18 C	-0.00081	-2.40439	NA	level4	NA	NA	NA	NA
C23 H44 C	-2.48E-05	-0.06454	NA	level4	HMDB	NA	HMDB025	NA
C13 H22 C	17.02664	70313.9	14.32817	level4	Public	NA	NA	NA
C9 H16 Cl	0.000281	1.206477	NA	level4	HMDB	NA	HMDB001	Organic ac
C15 H20 C	0.000235	1.087812	NA	level4	HMDB	NA	HMDB025	NA
C22 H30 C	0.000177	0.436858	NA	level4	HMDB	NA	HMDB024	NA
C14 H28 C	-0.00015	-0.61172	NA	level4	NA	NA	NA	NA
C18 H34 N	-0.00175	-4.46684	NA	level4	HMDB	NA	HMDB025	NA
C20 H22 N	-0.00049	-1.08264	NA	level4	HMDB	NA	HMDB001	Organic ac
C17 H17 C	-0.00136	-4.29131	NA	level4	NA	NA	NA	NA
C15 H28 C	-0.00456	-11.9455	55.0092	level3	mzCloud	C00448	HMDB000	Lipids and
C7 H10 O	0.000412	2.608681	NA	level4	HMDB	NA	HMDB024	NA
C23 H20 N	-0.00118	-2.98655	NA	level4	HMDB	NA	HMDB025	NA
C8 H16 N	0.000182	0.966388	96.05288	level2	mzCloud	C02155	HMDB000	Organic ac
C22 H23 N	0.001738	4.46688	NA	level4	NA	C22165	NA	Organohe
C21 H31 F	-0.0001	-0.28386	NA	level4	HMDB	NA	HMDB024	NA
C14 H9 N	-0.00137	-4.78792	NA	level4	HMDB	NA	HMDB024	NA
C28 H48 C	-277.179	-467964	2.005446	level4	Public	NA	NA	NA
C15 H22 C	17.02648	68063.22	32.71473	level4	Public	NA	NA	NA
C9 H11 N	3.39E-05	0.187165	NA	level4	HMDB	NA	HMDB024	NA
C31 H27 N	-2.14E-05	-0.04487	NA	level4	HMDB	NA	HMDB024	NA
C20 H24 N	37.95677	117084.1	20.26079	level4	Public	NA	NA	NA
C20 H37 N	0.002355	4.467119	NA	level4	KEGG	C01925	NA	Organic o:
C50 H78 N	-0.00468	-4.70392	NA	level4	HMDB	NA	HMDB025	NA
C25 H47 N	4.45E-05	0.104561	NA	level4	HMDB	NA	HMDB001	NA
C17 H17 F	-0.00109	-2.92895	NA	level4	HMDB	NA	HMDB025	NA
C21 H34 C	0.000202	0.486736	NA	level4	NA	NA	NA	NA
C19 H26 C	-6.66E-05	-0.2327	NA	level4	HMDB	NA	HMDB000	Lipids and
C15 H26 C	0.001268	3.184411	NA	level4	HMDB	NA	HMDB026	NA
C23 H45 C	-0.00154	-3.19942	NA	level4	NA	NA	NA	NA
C23 H34 C	0.000532	1.309383	NA	level4	HMDB	NA	HMDB025	NA
C29 H35 N	-0.00048	-1.11997	NA	level4	HMDB	NA	HMDB001	Lipids and
C10 H7 N	0.000129	0.746549	NA	level4	HMDB	NA	HMDB025	NA
C27 H26 C	-0.00126	-3.15766	NA	level4	HMDB	NA	HMDB024	NA
C9 H15 N	-0.00017	-0.71129	NA	level4	HMDB	NA	HMDB025	NA
C29 H38 C	0.000779	1.277264	1.375942	level4	Public	NA	NA	NA
C40 H76 N	1.96E-05	0.026884	NA	level4	HMDB	NA	HMDB000	Lipids and
C27 H43 N	-5.59E-05	-0.13521	34.60782	level4	Public	NA	NA	NA
C14 H13 C	8.84E-05	0.236465	NA	level4	NA	C19140	NA	NA
C25 H22 C	0.000363	0.753913	6.214207	level4	Public	NA	NA	NA
C21 H37 N	-5.77E-05	-0.15721	NA	level4	HMDB	NA	HMDB025	NA
C20 H17 N	-0.00013	-0.38186	90.54972	level2	mzCloud	C00757	HMDB000	Alkaloids a
C27 H44 C	-7.17E-06	-0.01864	91.46437	level2	mzCloud	C05443	HMDB000	Lipids and
C6 H13 N	9.36E-05	0.714255	96.60017	level1	BMDB	C00407	HMDB000	Organic ac
C14 H20 N	-0.0003	-1.118	NA	level4	HMDB	NA	HMDB025	NA
C26 H38 C	0.000581	1.349472	NA	level4	NA	NA	NA	NA
C26 H36 C	0.000159	0.344409	22.16834	level4	Public	NA	NA	NA
C18 H36 C	-5.17E-05	-0.13599	NA	level4	NA	NA	NA	NA
C7 H9 N3	4.79E-05	0.261445	NA	level4	NA	C16403	NA	NA

C35 H34 N	-0.00022	-0.35587	NA	level4	NA	C16540	NA	NA
C26 H30 C	17.02839	36215.67	52.39342	level3	mzCloud	C03514	HMDB003	Lipids and
C23 H28 C	17.02683	35460.51	50.8949	level3	Public	NA	NA	NA
C27 H22 N	-0.0005	-1.03086	NA	level4	HMDB	NA	HMDB024	NA
C21 H26 C	21.98023	64235.27	11.38146	level4	Public	NA	NA	NA
C33 H33 F	0.00263	4.831685	NA	level4	HMDB	NA	HMDB025	NA
C14 H14 C	0.00156	4.601046	NA	level4	HMDB	NA	HMDB025	NA
C16 H18 N	-0.00017	-0.56447	43.79929	level3	Public	NA	NA	NA
C35 H41 N	0.001554	2.476783	NA	level4	HMDB	NA	HMDB026	NA
C25 H45 B	-0.00107	-2.34951	NA	level4	NA	NA	NA	NA
C26 H45 N	-0.00077	-1.48702	NA	level4	HMDB	NA	HMDB025	NA
C20 H32 C	17.02636	50639.02	30.33117	level3	mzCloud	NA	NA	NA
C12 H15 N	-0.00035	-1.12705	NA	level4	NA	C16629	NA	NA
C20 H32 C	-18.0107	-56242	92.03756	level2	mzCloud	NA	NA	NA
C43 H80 N	-0.00045	-0.58141	NA	level4	NA	NA	NA	NA
C21 H40 C	7.61E-05	0.213559	NA	level4	HMDB	NA	HMDB001	Lipids and
C22 H21 N	0.001429	3.509654	NA	level4	HMDB	NA	HMDB025	NA
C21 H24 C	-0.00206	-4.89215	2.206614	level4	Public	NA	NA	NA
C12 H14 N	0.000868	2.629811	NA	level4	HMDB	NA	HMDB025	NA
C17 H13 C	0.001484	4.510068	7.540995	level4	Public	NA	HMDB024	NA
C20 H32 C	-18.0107	-56242	93.22376	level2	mzCloud	NA	NA	NA
C15 H18 C	18.01047	64758.98	9.751715	level4	Public	NA	NA	NA
C19 H18 C	-1.86E-05	-0.05071	NA	level4	NA	C11562	NA	NA
C16 H20 C	-0.00014	-0.40783	NA	level4	HMDB	NA	HMDB006	Organic ox
C19 H18 C	-0.00079	-1.94229	NA	level4	HMDB	NA	HMDB025	NA
C14 H16	8.76E-05	0.475799	NA	level4	KEGG	C09633	NA	Terpenoid
C11 H10 C	0.000278	1.252738	11.83824	level4	Public	NA	NA	NA
C27 H54 N	0.000764	1.346077	NA	level4	HMDB	NA	HMDB024	NA
C25 H30 C	17.02716	35907.96	15.06563	level4	Public	NA	NA	NA
C15 H18 C	21.98061	70881.13	3.335669	level4	Public	NA	NA	NA
C4 H7 Cl C	-5.81E-05	-0.47602	NA	level4	HMDB	NA	HMDB025	NA
C4 H7 Cl2	-0.00073	-4.26118	NA	level4	HMDB	NA	HMDB024	NA
C30 H27 N	0.000101	0.176835	NA	level4	HMDB	NA	HMDB025	NA
C20 H36 C	21.98451	64610.65	45.50733	level3	mzCloud	NA	NA	NA
C18 H20 C	-0.0001	-0.38416	NA	level4	HMDB	NA	HMDB001	Phenylpro
C30 H42 C	-0.00301	-4.26391	NA	level4	HMDB	NA	HMDB025	NA
C18 H21 N	17.02668	54027.71	6.422152	level4	Public	NA	NA	NA
C25 H40 N	0.002646	4.861424	NA	level4	HMDB	NA	HMDB006	Organohe
C25 H25 N	0.001429	2.958583	NA	level4	HMDB	NA	HMDB024	NA
C15 H10 C	6.97E-05	0.24366	NA	level4	HMDB	NA	HMDB025	NA
C20 H38 C	-4.71E-05	-0.13773	NA	level4	HMDB	NA	HMDB025	NA
C22 H23 N	-0.00149	-4.25622	NA	level4	HMDB	NA	HMDB025	NA
C17 H14 C	-4.15E-05	-0.13199	NA	level4	HMDB	NA	HMDB024	NA
C22 H22 C	-0.00024	-0.66504	NA	level4	NA	NA	NA	NA
C23 H18 C	-2.47E-05	-0.05251	NA	level4	NA	NA	NA	NA
C18 H22 N	-0.00132	-3.42127	NA	level4	HMDB	NA	HMDB024	NA
C14 H17 N	-4.91E-06	-0.01502	NA	level4	HMDB	NA	HMDB001	Organic ox
C15 H14 C	0.003112	11.35353	50.50974	level3	mzCloud	C00774	HMDB000	Phenylpro
C6 H4 Cl2	0.000596	2.639863	NA	level4	NA	NA	NA	NA
C11 H17 N	-0.00014	-0.44098	NA	level4	HMDB	NA	HMDB024	NA
C33 H46 C	0.002526	4.557534	NA	level4	NA	C11961	NA	NA
C25 H40 C	-0.0014	-2.70985	NA	level4	NA	C17889	NA	NA
C27 H37 F	-0.00164	-3.55311	NA	level4	HMDB	NA	HMDB025	NA
C17 H14 N	-5.28E-05	-0.17965	12.46963	level4	Public	NA	NA	NA
C16 H23 N	37.95423	116727.5	7.150004	level4	Public	NA	NA	NA
C7 H14 N2	0.000305	1.750343	87.98782	level1	Decode	NA	HMDB002	Organic ac

C9 H17 N	0.000135	0.66661	96.20231	level1	BMDB	C02571	HMDB000	Lipids and
C14 H16 N	-0.00018	-0.73712	NA	level4	HMDB	NA	HMDB001	Organohe
C17 H23 N	-0.00103	-2.8394	NA	level4	HMDB	NA	HMDB025	NA
C33 H41 C	0.003503	4.437991	NA	level4	NA	C16314	NA	Flavonoids
C31 H39 F	-0.00131	-2.07595	NA	level4	HMDB	NA	HMDB025	NA
C23 H23 N	0.001649	3.806137	NA	level4	NA	C22168	NA	NA
C10 H12 C	-0.00082	-4.54424	NA	level4	HMDB	NA	HMDB024	NA
C19 H17 N	-0.00061	-2.08316	NA	level4	NA	C10954	NA	NA
C28 H44 C	0.000742	1.55845	NA	level4	NA	NA	NA	NA
C23 H40 N	-5.40E-05	-0.13215	NA	level4	NA	NA	NA	NA
C52 H97 N	0.00034	0.332366	NA	level4	HMDB	NA	HMDB000	Lipids and
C19 H27 N	-7.50E-06	-0.02631	40.9588	level3	Public	NA	HMDB003	Organohe
C6 H7 N5	0.000242	1.46392	91.31689	level2	mzCloud	NA	HMDB000	Organohe
C13 H10 C	0.000159	0.647577	NA	level4	HMDB	NA	HMDB025	NA
C8 H7 N C	0.000143	0.863764	55.97634	level3	Public	NA	HMDB025	NA
C14 H21 N	0.001413	4.26856	NA	level4	HMDB	NA	HMDB024	NA
C32 H44 C	0.002007	3.608611	NA	level4	HMDB	NA	HMDB025	NA
C21 H27 N	0.001387	3.033352	NA	level4	KEGG	C02864	NA	NA
C45 H52 N	0.001428	1.524847	NA	level4	HMDB	NA	HMDB000	Organohe
C37 H47 N	0.000393	0.654354	NA	level4	NA	C20533	NA	NA
C9 H10 N2	0.000177	1.089213	NA	level4	HMDB	NA	HMDB000	Organohe
C8 H10 O2	0.000187	1.353939	68.05823	level2	mzCloud	NA	HMDB003	Benzenoid
C37 H59 N	0.000621	0.939016	NA	level4	NA	C16998	NA	NA
C10 H20 N	-0.00109	-3.66641	NA	level4	HMDB	NA	HMDB001	Organic ni
C34 H63 C	-0.00102	-1.53801	NA	level4	NA	NA	NA	NA
C11 H17 N	0.000167	0.93478	40.7185	level3	mzCloud	D08206	NA	NA
C8 H10 O5	-0.0001	-0.45995	NA	level4	HMDB	NA	HMDB004	Organic ac
C21 H28 C	-7.45E-05	-0.25161	NA	level4	NA	NA	NA	NA
C19 H24 N	0.0007	2.133741	NA	level4	HMDB	NA	HMDB001	Organohe
C17 H15 N	0.001437	4.475281	NA	level4	HMDB	NA	HMDB024	NA
C15 H17 C	0.00144	4.614945	NA	level4	HMDB	NA	HMDB024	NA
C28 H46 C	-0.00104	-1.91839	NA	level4	NA	NA	NA	NA
C8 H16 N	0.000654	2.062565	NA	level4	KEGG	C04336	NA	NA
C12 H16 C	17.02677	75976.82	8.079085	level4	Public	NA	NA	NA
C9 H12 O6	-0.00059	-2.38077	NA	level4	HMDB	NA	HMDB024	Organic ac
C13 H15 N	-0.00153	-4.93696	NA	level4	HMDB	NA	HMDB001	Benzenoid
C13 H13 N	21.9805	79907.93	11.40094	level4	Public	NA	NA	NA
C18 H21 N	-0.00024	-0.69666	3.792809	level4	Public	NA	NA	NA
C25 H25 N	9.78E-05	0.275313	NA	level4	HMDB	NA	HMDB024	NA
C12 H13 N	5.42E-05	0.183704	11.85757	level4	Public	C13061	HMDB001	Benzenoid
C4 H7 N C	-18.0103	-153879	43.65373	level3	BMDB	NA	HMDB000	Organic ac
C20 H32 C	0.00023	0.65186	NA	level4	HMDB	NA	HMDB025	NA
C49 H76 C	21.98142	22327.65	2.78489	level4	Public	NA	HMDB024	NA
C5 H10 N6	0.000405	2.631108	NA	level4	HMDB	NA	HMDB024	NA
C16 H24 C	17.02489	49469.81	6.530654	level4	Public	NA	HMDB003	Organic ox
C10 H19 B	-0.00049	-1.95895	NA	level4	NA	NA	NA	NA
C42 H76 N	-0.00084	-1.11343	NA	level4	HMDB	NA	HMDB000	Lipids and
C6 H11 N2	0.000851	3.575287	NA	level4	KEGG	C04666	NA	Organic ac
C28 H52 N	-0.00268	-4.52128	NA	level4	NA	NA	NA	NA
C24 H40 C	-0.00031	-0.64304	NA	level4	NA	NA	NA	NA
C8 H15 N7	-0.00161	-4.77436	NA	level4	HMDB	NA	HMDB000	Organohe
C27 H27 N	-0.00233	-3.8419	NA	level4	HMDB	NA	HMDB025	NA
C16 H16 C	17.02643	59099.08	6.307464	level4	Public	NA	NA	NA
C19 H27 N	-0.00188	-3.32606	NA	level4	HMDB	NA	HMDB025	NA

C9 H8 O4	0.000246	1.36831	72.06773	level2	Public	C01405	HMDB000	Benzenoid
C11 H12 C	0.000197	1.229538	NA	level4	NA	NA	NA	NA
C30 H37 N	0.001202	2.196844	NA	level4	HMDB	NA	HMDB025	NA
C24 H35 N	37.95775	90970.92	14.91621	level4	Public	NA	NA	NA
C16 H22 C	0.000168	0.491919	29.82107	level4	Public	NA	NA	NA
C18 H20 N	3.22E-05	0.103285	NA	level4	HMDB	NA	HMDB001	Organic ac
C41 H55 N	-0.00405	-4.9737	NA	level4	HMDB	NA	HMDB026	NA
C32 H50 C	0.000187	0.32349	NA	level4	NA	NA	NA	NA
C18 H16 C	0.000235	0.851196	NA	level4	NA	C14549	NA	NA
C6 H11 N	-1.57E-05	-0.07517	NA	level4	HMDB	NA	HMDB025	NA
C10 H9 N7	0.000347	1.338919	NA	level4	HMDB	NA	HMDB025	NA
C15 H15 N	-0.00136	-4.18096	NA	level4	HMDB	NA	HMDB024	NA
C16 H13 C	-0.00155	-4.81286	NA	level4	NA	C18788	NA	NA
C9 H12 O6	7.26E-06	0.033619	NA	level4	NA	C20582	NA	NA
C6 H9 N C	0.000132	0.832694	NA	level4	HMDB	NA	HMDB024	NA
C32 H42 C	0.000747	1.094779	2.132479	level4	Public	NA	NA	NA
C28 H40 C	0.000429	1.010633	NA	level4	HMDB	NA	HMDB024	NA
C8 H14 O3	-0.00067	-3.54624	NA	level4	NA	C17220	NA	FA Fatty a
C15 H25 N	0.00181	4.318041	NA	level4	HMDB	NA	HMDB025	NA
C9 H18 N2	0.000165	0.704299	NA	level4	NA	C12025	NA	NA
C6 H6 N4	0.000863	4.033013	NA	level4	HMDB	NA	HMDB025	NA
C37 H40 C	21.99189	35004.04	43.94603	level3	mzCloud	NA	NA	NA
C28 H44 C	0.000126	0.318168	NA	level4	HMDB	NA	HMDB025	NA
C7 H8 O5	-18.0104	-104689	15.15378	level3	BMDB	C02637	NA	NA
C23 H29 F	-0.00178	-4.23801	NA	level4	HMDB	NA	HMDB025	NA
C19 H27 N	-0.00016	-0.51755	NA	level4	HMDB	NA	HMDB001	Organic ac
C10 H13 F	-0.00106	-3.84871	NA	level4	HMDB	NA	HMDB024	NA
C28 H30 C	0.003054	5.039018	12.09051	level4	Public	NA	NA	NA
C10 H18 N	-0.00018	-0.72567	NA	level4	NA	C20424	NA	NA
C9 H8 O7	-0.00085	-3.28554	NA	level4	HMDB	NA	HMDB004	Phenylpro
C15 H30 C	-0.00074	-2.19149	NA	level4	NA	NA	NA	NA
C21 H45 N	-0.00109	-2.77711	NA	level4	HMDB	NA	HMDB024	NA
C17 H34 C	1.41E-05	0.052095	NA	level4	HMDB	NA	HMDB024	NA
C20 H32 C	1.22E-05	0.03805	83.81098	level2	mzCloud	C14748	HMDB000	Lipids and
C27 H30 C	0.00035	0.572991	NA	level4	HMDB	NA	HMDB025	NA
C14 H19 N	-9.50E-05	-0.27843	NA	level4	HMDB	NA	HMDB024	NA
C7 H8 O5	2.48E-05	0.121738	90.79124	level1	Decode	NA	HMDB024	Organic ac
C5 H8 N2	9.50E-05	0.65958	NA	level4	NA	C19485	NA	Organic ac
C15 H12 C	17.02653	59106.87	11.50289	level4	Public	NA	NA	NA
C27 H49 N	-0.0024	-4.83938	NA	level4	HMDB	NA	HMDB024	NA
C9 H11 N	0.000271	1.376648	59.76856	level3	mzCloud	C00355	HMDB000	Organic ac
C13 H19 N	0.00113	3.392916	9.109959	level4	Public	NA	NA	NA
C6 H12 O4	0.00024	1.334508	50	level3	Masslist	C03089	HMDB000	Organic ox
C44 H84 N	-0.00079	-1.00966	NA	level4	HMDB	NA	HMDB000	Lipids and
C16 H24 N	-0.00013	-0.41579	NA	level4	HMDB	NA	HMDB025	NA
C8 H7 N C	0.000134	1.007231	81.65658	level2	mzCloud	C02040	NA	NA

C17 H28 N	-0.00025	-0.73582	11.96702	level4	Public	NA	HMDB006	Organic ac
C10 H17 N	0.000197	0.852182	NA	level4	HMDB	NA	HMDB025	NA
C6 H13 N	8.33E-05	0.56641	NA	level4	HMDB	NA	HMDB025	NA
C40 H76 N	0.000231	0.310329	NA	level4	NA	NA	NA	NA
C23 H38 N	17.02685	36516.43	23.76301	level4	Public	NA	NA	NA
C17 H17 N	-2.11E-05	-0.06786	41.81741	level3	Public	NA	NA	NA
C16 H22 C	-1.18E-05	-0.03843	5.585265	level4	Public	C18489	HMDB025	NA
C5 H9 N3	5.05E-05	0.455056	46.16708	level3	mzCloud	C00388	HMDB000	Organic ni
C23 H36 C	1.69E-05	0.043058	NA	level4	HMDB	NA	HMDB025	NA
C19 H23 C	21.98182	62782.15	38.57362	level4	Public	NA	NA	NA
C8 H7 N3	-0.00056	-2.50809	NA	level4	HMDB	NA	HMDB024	NA
C9 H16 N4	0.000172	0.703603	NA	level4	KEGG	C06657	NA	NA
C27 H42 C	-7.63E-05	-0.19948	NA	level4	HMDB	NA	HMDB025	NA
C15 H15 N	0.000702	3.355077	NA	level4	HMDB	NA	HMDB024	NA
C10 H7 N3	0.000311	1.546267	71.78604	level2	Public	C07131	HMDB001	Organohe
C24 H22 N	0.001973	4.956062	NA	level4	HMDB	NA	HMDB025	NA
C17 H14 C	-0.00244	-7.40553	60.22949	level2	mzCloud	C08716	HMDB013	Phenylpro
C16 H28 C	0.000269	0.894694	NA	level4	NA	NA	NA	NA
C10 H12 N	0.000291	1.234046	NA	level4	HMDB	NA	HMDB025	NA
C18 H19 N	0.00024	0.695008	NA	level4	KEGG	C08520	NA	NA
C8 H14	0.000139	1.264173	NA	level4	HMDB	NA	HMDB024	NA
C37 H70 N	-0.00015	-0.19914	NA	level4	HMDB	NA	HMDB025	NA
C15 H12 C	0.000212	0.743707	NA	level4	HMDB	NA	HMDB025	NA
C28 H44 N	-0.00098	-1.96405	NA	level4	NA	C10003	NA	Organic ac
C25 H24 F	-0.00169	-4.00621	NA	level4	HMDB	NA	HMDB024	NA
C20 H24 C	-0.00169	-3.84067	2.310924	level4	Public	NA	NA	NA
C8 H16 O3	-18.0104	-112488	44.36273	level3	Decode	NA	HMDB000	Lipids and
C34 H65 C	-0.00159	-2.52068	NA	level4	NA	NA	NA	NA
C18H13Cl4	0.000198	0.402183	NA	level4	KEGG	C08075	NA	Benzenoid
C3 H4 O4	2.67E-05	0.256249	88.45665	level1	BMDB	C00383	HMDB000	Organic ac
C8 H10 N2	17.02669	85965.55	1.105751	level4	Public	NA	NA	NA
C19 H22 C	-0.00399	-12.0706	45.54404	level3	mzCloud	C11867	NA	Terpenoid
C6 H8 O2	0.000216	1.92579	NA	level4	HMDB	NA	HMDB003	Organohe
C15 H16 C	-0.00021	-0.76673	21.87576	level4	Public	NA	NA	NA
C16 H25 N	0.000121	0.433412	17.11925	level4	Public	NA	NA	NA
C7 H6 Cl N	-0.00014	-0.48076	72.61609	level2	Public	C07461	HMDB001	Organohe
C16 H24 N	0.002307	4.486995	NA	level4	NA	C18032	NA	NA
C25 H33 C	-0.00247	-4.93016	NA	level4	HMDB	NA	HMDB025	NA
H Na2 O4	0.000228	1.605189	NA	level4	NA	C13558	NA	NA
C25 H30 N	0.000385	0.847549	NA	level4	HMDB	NA	HMDB024	NA
C18 H22 N	0.000679	2.278063	NA	level4	HMDB	NA	HMDB025	NA
C27 H37 N	1.75E-05	0.029658	NA	level4	HMDB	NA	HMDB025	NA
C19 H34 C	0.00056	1.327109	NA	level4	NA	C17614	NA	NA
C17 H18 C	-5.12E-05	-0.17903	NA	level4	HMDB	NA	HMDB025	NA
C19 H26 C	-0.00017	-0.58547	47.1439	level3	mzCloud	NA	NA	NA
C21 H33 N	-0.00015	-0.37352	NA	level4	NA	C11331	NA	NA
C20 H30 C	43.96449	138157.8	25.52811	level4	Public	NA	NA	NA
C30 H34 C	-0.00207	-4.09519	NA	level4	NA	NA	NA	NA
C45 H76 C	-0.00248	-2.84451	NA	level4	HMDB	NA	HMDB025	NA
C15 H10 C	17.02662	67018.67	6.128286	level4	Public	C10402	HMDB025	NA
C12 H12 N	-0.00016	-0.67213	56.27263	level3	Public	C07434	HMDB001	Organohe
C30 H38 C	0.000826	1.569458	NA	level4	HMDB	NA	HMDB025	NA
C11 H18 N	-0.00113	-4.73759	19.76449	level4	Public	C11079	HMDB025	NA
C4 H9 N C	0.000182	1.528355	94.89956	level1	BMDB	C00188	HMDB000	Organic ac
C9 H13 N	0.000285	1.433835	NA	level4	NA	C20941	NA	NA
C7 H11 N	0.00013	0.74922	NA	level4	HMDB	NA	HMDB024	NA

C30 H60 N	-0.00133	-2.29712	NA	level4	HMDB	NA	HMDB001	Lipids and
C5 H9 N3	0.000806	3.894233	NA	level4	HMDB	NA	HMDB025	NA
C33 H52 C	0.001473	2.359896	NA	level4	NA	C20007	NA	Marine bic
C11 H17 N	0.00118	4.555863	NA	level4	KEGG	C08338	NA	Amino aci
C4 H8 O S	6.98E-05	0.513343	NA	level4	NA	C19127	NA	NA
C28 H26 C	0.001046	2.066874	NA	level4	HMDB	NA	HMDB025	NA
C19 H25 N	-0.00016	-0.52605	NA	level4	HMDB	NA	HMDB024	NA
C24 H33 N	0.000854	1.998157	NA	level4	HMDB	NA	HMDB025	NA
C18 H11 N	-0.00076	-2.77883	NA	level4	HMDB	NA	HMDB024	NA
C20 H34 C	17.0266	48065.08	47.00158	level3	mzCloud	C06438	HMDB000	Lipids and
C23 H35 N	0.000137	0.301512	NA	level4	NA	NA	NA	NA
C21 H23 N	-0.001	-2.97027	NA	level4	HMDB	NA	HMDB025	NA
C17 H28 N	0.000163	0.489451	NA	level4	HMDB	NA	HMDB025	NA
C16 H15 F	6.84E-05	0.171394	NA	level4	HMDB	NA	HMDB025	NA
C38 H63 N	0.000607	0.801846	NA	level4	NA	C14060	NA	NA
C16 H20 C	9.92E-05	0.255653	NA	level4	NA	NA	NA	NA
C24 H38 C	4.61E-05	0.118135	NA	level4	HMDB	NA	HMDB024	NA
C13 H15 N	3.72E-05	0.140416	50	level3	Masslist	NA	HMDB005	Organic ac
C30 H48 C	17.02712	36047.27	46.12858	level3	mzCloud	C16939	HMDB000	Lipids and
C41 H79 C	0.00313	4.283761	NA	level4	HMDB	NA	HMDB025	NA
C8 H8 N4	-0.0004	-1.78018	NA	level4	NA	C19272	NA	NA
C15 H24 N	0.000595	1.724376	NA	level4	NA	C11002	NA	Benzenoid
C10 H11 N	-0.00166	-4.48847	NA	level4	HMDB	NA	HMDB024	NA
C6 H8 O3	8.61E-05	0.672356	NA	level4	HMDB	NA	HMDB004	Organohe
C21 H24 C	21.98095	52317.91	6.254992	level4	Public	NA	NA	NA
C16 H29 N	-6.64E-05	-0.22189	NA	level4	NA	NA	NA	NA
C11 H16	0.000274	1.852182	NA	level4	NA	C16993	NA	NA
C10 H12 C	0.000242	1.477025	NA	level4	HMDB	NA	HMDB024	NA
C15 H22 N	0.0016	4.518909	NA	level4	HMDB	NA	HMDB024	NA
C10 H16 N	0.000194	0.794657	NA	level4	NA	NA	NA	NA
C20 H24 N	0.001817	4.49601	NA	level4	HMDB	NA	HMDB025	NA
C29 H42 C	-0.00015	-0.31211	NA	level4	NA	NA	NA	NA
C27 H36 N	0.000165	0.28921	NA	level4	HMDB	NA	HMDB025	NA
C19 H37 N	-7.12E-05	-0.21746	NA	level4	HMDB	NA	HMDB025	NA
C59 H102	-0.00209	-2.30505	NA	level4	HMDB	NA	HMDB000	Lipids and
C19 H27 N	-9.21E-05	-0.25493	NA	level4	HMDB	NA	HMDB025	NA
C33 H40 N	0.000653	1.110637	NA	level4	KEGG	C05795	NA	NA
C9 H10 O3	8.29E-05	0.49919	89.39409	level2	mzCloud	C11380	NA	PK Polyke
C20 H17 C	0.000264	0.716633	NA	level4	HMDB	NA	HMDB001	Organohe
C31 H52 C	0.000587	1.162897	NA	level4	NA	NA	NA	NA
C13 H10 F	0.000618	2.246683	NA	level4	HMDB	NA	HMDB024	NA
C36 H64 C	0.000662	1.148258	NA	level4	NA	NA	NA	NA
C10 H19 N	0.000957	3.841604	NA	level4	HMDB	NA	HMDB024	NA
C18 H22 C	-0.00019	-0.71554	NA	level4	HMDB	NA	HMDB025	NA
C24 H21 N	0.000292	0.710281	NA	level4	HMDB	NA	HMDB025	NA
C11 H18 N	-7.04E-06	-0.02908	NA	level4	HMDB	NA	HMDB024	NA
C5 H10 N2	0.000118	0.807248	95.43123	level2	BMDB	C00064	HMDB000	Organic ac
C5 H10 O5	-0.00014	-0.91842	80.45697	level2	mzCloud	C00476	NA	Carbohydr
C13 H10 C	-9.62E-05	-0.39102	NA	level4	NA	NA	NA	NA
C35 H69 N	0.001233	2.236302	NA	level4	NA	NA	NA	NA
C16 H25 N	-0.00145	-4.77704	NA	level4	HMDB	NA	HMDB026	NA
C21 H39 N	-0.00038	-1.021	NA	level4	HMDB	NA	HMDB000	Lipids and

C33 H46 N	0.000444	0.747729	88.77962	level2	mzCloud	C05793	HMDB024	Organohe
C47 H93 N	0.000474	0.583218	NA	level4	HMDB	NA	HMDB001	Lipids and
C21 H16 F	-0.00134	-3.24657	NA	level4	HMDB	NA	HMDB025	NA
C12 H23 N	-0.00141	-3.48146	NA	level4	KEGG	C08406	NA	Amino aci
C29 H20 C	0.001436	3.739319	NA	level4	HMDB	NA	HMDB025	NA
C33 H58 C	0.00087	1.580851	NA	level4	NA	NA	NA	NA
C15 H13 C	-0.00205	-4.27102	NA	level4	HMDB	NA	HMDB025	NA
C29 H32 C	-0.00151	-2.36809	NA	level4	NA	NA	NA	NA
UnkownFc	363.1491	Inf	12.2141	level4	Public	NA	NA	NA
C15 H17 C	6.21E-05	0.212435	NA	level4	HMDB	NA	HMDB024	NA
C23 H19 C	0.000897	1.996913	NA	level4	HMDB	NA	HMDB025	NA
C38 H42 N	-0.00221	-3.552	NA	level4	KEGG	C09590	NA	Alkaloids
C18 H19 F	0.001831	3.742314	NA	level4	HMDB	NA	HMDB025	NA
C23 H31 N	-1.62E-05	-0.03747	NA	level4	HMDB	NA	HMDB025	NA
C26 H43 N	-0.0005	-0.97035	NA	level4	HMDB	NA	HMDB000	Lipids and
C36 H36 C	-0.00013	-0.17388	NA	level4	NA	NA	NA	NA
C10 H12 N	0.001274	4.029903	NA	level4	HMDB	NA	HMDB025	NA
C17 H30 C	-0.00013	-0.22542	NA	level4	HMDB	NA	HMDB006	Organic ac
C23 H32 C	37.9545	83932.14	11.71916	level4	Public	NA	NA	NA
C17 H17 C	-0.00021	-0.68952	NA	level4	HMDB	NA	HMDB025	NA
C5 H11 N	21.98214	187754.8	57.27272	level3	BMDB	C00719	HMDB000	Organic ac
C21 H20 F	0.00032	0.867764	NA	level4	HMDB	NA	HMDB001	Organohe
C25 H50 F	0.001193	1.930057	NA	level4	NA	NA	NA	NA
C6 H11 N	0.000164	1.454525	90.90136	level1	Decode	C06593	HMDB006	Organohe
C35 H44 C	8.20E-05	0.150574	NA	level4	NA	NA	NA	NA
C10 H15 N	-0.00025	-1.05371	NA	level4	HMDB	NA	HMDB024	NA
C28 H34 C	17.02724	34175.79	21.58558	level4	Public	NA	NA	NA
C21 H36 C	0.001958	4.368457	NA	level4	NA	C17858	NA	NA
C39 H44 N	-0.00018	-0.23124	NA	level4	HMDB	NA	HMDB001	Organohe
H2 O3 S2	5.87E-05	0.515225	NA	level4	HMDB	NA	HMDB000	Homogen
C15 H29 C	-0.00169	-4.59697	NA	level4	NA	NA	NA	NA
C16 H20 N	-0.00014	-0.40838	NA	level4	HMDB	NA	HMDB025	NA
C6 H5 N5	-0.00012	-0.65808	19.2179	level4	Public	NA	HMDB025	NA
C20 H19 N	-0.00078	-2.56966	NA	level4	HMDB	NA	HMDB024	NA
C19 H32 C	-5.28E-06	-0.01807	NA	level4	HMDB	NA	HMDB025	NA
C8 H16 N	-0.00022	-0.74292	NA	level4	HMDB	NA	HMDB025	NA
C59 H98 C	-0.0018	-1.98937	NA	level4	NA	NA	NA	NA
C11 H23 N	0.00083	3.824406	NA	level4	HMDB	NA	HMDB024	NA
C15 H23 N	-0.00017	-0.58927	NA	level4	HMDB	NA	HMDB006	Benzenoid
C39 H39 C	-0.00028	-0.32744	NA	level4	NA	C16299	NA	Flavonoids
C7 H8 N2	0.000117	0.860157	69.82231	level2	mzCloud	NA	HMDB000	Organohe
C24 H27 N	0.000955	2.26814	NA	level4	HMDB	NA	HMDB025	NA
C19 H22 C	17.02647	47016.71	21.51901	level4	Public	NA	NA	NA
C21 H29 F	-0.00117	-2.9419	NA	level4	NA	C14638	NA	NA
C19 H18 C	-0.00036	-1.00505	53.08713	level3	Public	NA	NA	NA
C25 H24 C	-0.00228	-4.87366	NA	level4	NA	C12403	NA	NA
C7 H9 N3	0.000219	1.451101	NA	level4	HMDB	NA	HMDB024	NA
C29 H54 N	-0.0027	-4.33451	NA	level4	NA	NA	NA	NA
C17 H19 N	-2.36E-05	-0.06462	NA	level4	HMDB	NA	HMDB025	NA
C4 H9 N C	0.000208	2.389144	NA	level4	HMDB	NA	HMDB025	NA
C28 H51 N	-36.0234	-64631.3	7.931955	level4	Public	NA	NA	NA
C30 H48 C	0.000791	1.475528	NA	level4	NA	NA	NA	NA
C7 H8 N4	-6.17E-05	-0.31477	94.2459	level2	mzCloud	NA	NA	NA
C22 H28 C	-0.00029	-0.86345	76.91572	level2	Public	NA	HMDB025	NA
C4 H4 N2	7.01E-05	0.626138	87.08949	level2	mzCloud	C00106	HMDB000	Organohe
C34 H65 N	0.000817	1.525925	NA	level4	NA	NA	NA	NA

C28 H49 N	5.25E-05	0.126466	NA	level4	HMDB	NA	HMDB024	NA
C10 H26 N	0.00013	0.494857	NA	level4	HMDB	NA	HMDB025	NA
C28 H51 N	-18.0085	-33264.4	13.26384	level4	Public	NA	NA	NA
C21 H39 N	0.000367	0.952714	NA	level4	HMDB	NA	HMDB001	NA
C23 H21 N	-0.00126	-2.63991	NA	level4	HMDB	NA	HMDB024	NA
C5 H8 O2	0.000251	1.904406	NA	level4	NA	C11074	NA	NA
C17 H26 N	0.00213	3.915719	NA	level4	NA	C11915	NA	NA
C14 H12	0.000179	0.991185	NA	level4	HMDB	NA	HMDB024	NA
C16 H19 N	-0.00033	-0.9011	NA	level4	HMDB	NA	HMDB001	Organohe
C27 H30 C	-0.00023	-0.38394	65.65217	level2	mzCloud	NA	NA	NA
C46 H87 C	-9.17E-05	-0.09562	NA	level4	NA	NA	NA	NA
C16 H14 N	-0.00132	-4.74032	NA	level4	HMDB	NA	HMDB024	NA
C15 H22 C	0.001035	2.857784	NA	level4	HMDB	NA	HMDB024	NA
C16 H21 N	-0.00014	-0.584	NA	level4	HMDB	NA	HMDB025	NA
C12 H13 N	-7.39E-05	-0.3374	NA	level4	HMDB	NA	HMDB024	NA
C14 H24 C	-7.76E-05	-0.32319	NA	level4	NA	NA	NA	NA
C25 H28 C	-0.00053	-1.36246	NA	level4	HMDB	NA	HMDB025	NA
C48 H67 B	0.001702	2.255686	NA	level4	NA	NA	NA	NA
C12 H10 C	17.02658	72746.74	10.37273	level4	Public	NA	NA	NA
C23 H46 N	-0.00099	-1.94149	NA	level4	NA	NA	NA	NA
C21 H31 C	-0.00207	-4.59422	NA	level4	HMDB	NA	HMDB025	NA
C18 H24 I	3.22E-05	0.040723	NA	level4	HMDB	NA	HMDB025	NA
C20 H39 N	-9.17E-05	-0.26866	49.19448	level3	mzCloud	NA	NA	NA
C25 H42 C	17.02681	41906.14	16.01689	level4	Public	NA	NA	NA
C21 H39 N	-0.00177	-2.95981	NA	level4	NA	C17571	NA	NA
C8 H11 N3	-0.00027	-1.10872	NA	level4	HMDB	NA	HMDB006	Nucleosid
C40 H74 N	0.000222	0.304696	NA	level4	HMDB	NA	HMDB000	Lipids and
C27 H42 C	0.000788	1.648023	NA	level4	NA	NA	NA	NA
C26 H34 C	-0.00062	-1.15005	28.15874	level4	Public	NA	NA	NA
C28 H41 N	0.000601	1.285374	NA	level4	HMDB	NA	HMDB025	NA
C29 H43 N	0.001822	3.634681	NA	level4	HMDB	NA	HMDB024	NA
C21 H24 F	-0.00188	-4.44852	NA	level4	HMDB	NA	HMDB024	NA
C8 H19 N	0.000187	1.446616	NA	level4	HMDB	NA	HMDB024	NA
C7 H13 N	0.000238	1.358203	NA	level4	HMDB	NA	HMDB024	NA
C22 H25 F	0.001411	3.256347	NA	level4	HMDB	NA	HMDB024	NA
C9 H15 N	0.000136	0.584252	NA	level4	HMDB	NA	HMDB025	NA
C16 H15 C	0.000574	1.973546	NA	level4	HMDB	NA	HMDB006	Benzenoid
C27 H46 C	-4.69E-05	-0.12135	56.42197	level4	Public	NA	HMDB025	NA
C15 H24 C	0.000306	1.387655	92.40085	level2	mzCloud	C16908	HMDB003	Lipids and
C20 H34 C	-18.0109	-50843.6	39.80473	level3	mzCloud	C00639	HMDB000	Lipids and
C36 H58 C	0.001121	1.767028	NA	level4	HMDB	NA	HMDB003	Lipids and
C30 H46 C	0.000171	0.375319	30.03582	level4	Public	NA	NA	NA
C21 H25 C	0.001499	3.822798	NA	level4	HMDB	NA	HMDB025	NA
C16 H21 N	1.91E-05	0.059015	NA	level4	HMDB	NA	HMDB025	NA
C13 H10 C	0.000251	1.268098	NA	level4	HMDB	NA	HMDB024	NA
C19 H38 C	-0.00017	-0.53032	NA	level4	NA	NA	NA	NA
C19 H18 C	21.98381	74742.95	24.26003	level4	Public	NA	NA	NA
C17 H24 N	-0.00173	-4.24731	NA	level4	HMDB	NA	HMDB025	NA
C34 H38 C	0.000488	0.900722	NA	level4	NA	NA	NA	NA
C31 H46 N	21.9838	38544.96	53.25296	level4	Public	NA	NA	NA

C18 H20 N	-0.00012	-0.32687	0.437341	level4	Public	C07713	HMDB001	Organohe
C29 H50 C	0.000564	1.310604	90.60582	level2	mzCloud	C02477	HMDB000	Lipids and
C21 H36 C	34.04217	92441.51	6.522917	level4	Public	NA	NA	NA
C23 H29 I	-0.00185	-3.62101	NA	level4	HMDB	NA	HMDB025	NA
C23 H21 F	0.000945	2.511885	NA	level4	HMDB	NA	HMDB024	NA
C10 H18 C	0.000176	0.872239	62.4499	level1	BMDB2	C08277	HMDB000	Lipids and
C29 H50 N	-0.00019	-0.28895	NA	level4	NA	NA	NA	NA
C17 H12 C	-0.00023	-0.63874	NA	level4	HMDB	NA	HMDB006	Organohe
C20 H30 C	17.02654	56337.36	40.12395	level4	Public	NA	NA	NA
C33 H60 C	-0.0008	-1.45639	NA	level4	NA	NA	NA	NA
C21 H27 N	2.55E-06	0.006905	NA	level4	HMDB	NA	HMDB024	NA
C14 H11 C	-0.00028	-0.94544	NA	level4	HMDB	NA	HMDB001	Benzenoid
C11 H13 F	-0.00102	-3.9896	NA	level4	NA	C15341	NA	NA
C10 H16 N	0.00028	1.225749	NA	level4	HMDB	NA	HMDB024	NA
C13 H16 N	-0.00018	-0.65963	96.10542	level2	mzCloud	NA	HMDB000	Organic ac
C16 H30 C	8.12E-05	0.319524	NA	level4	HMDB	NA	HMDB025	NA
C27 H24 F	0.000272	0.555388	NA	level4	HMDB	NA	HMDB025	NA
C30 H50 C	0.000192	0.433546	NA	level4	HMDB	NA	HMDB024	NA
C11 H12 N	-0.00088	-4.3896	NA	level4	HMDB	NA	HMDB006	Organohe
C30 H48 C	0.000929	1.7861	NA	level4	NA	NA	NA	NA
C38 H73 N	0.000522	0.882151	NA	level4	NA	NA	NA	NA
C23 H31 C	-0.0097	-23.8902	56.10806	level4	Public	NA	NA	NA
C16 H20 F	0.001354	3.835242	NA	level4	HMDB	NA	HMDB025	NA
C16 H10 C	-0.00021	-0.69984	NA	level4	HMDB	NA	HMDB003	Phenylpro
C9 H12 O	0.000238	1.751978	NA	level4	NA	NA	NA	NA
C12 H11 N	0.000308	1.320859	NA	level4	NA	C10654	NA	Alkaloids
C27 H27 N	-0.00141	-2.46108	NA	level4	HMDB	NA	HMDB024	NA
C11 H11 N	-8.69E-05	-0.37278	NA	level4	HMDB	NA	HMDB024	NA
C17 H22 C	0.000394	1.285895	20.28821	level4	Public	NA	NA	NA
C11 H14 C	-0.00067	-3.18067	NA	level4	HMDB	NA	HMDB006	Organohe
C25 H29 F	0.000758	1.619423	NA	level4	HMDB	NA	HMDB025	NA
C26 H43 N	-0.00028	-0.53654	NA	level4	HMDB	NA	HMDB000	Lipids and
C32 H50 C	0.001689	2.628755	NA	level4	NA	NA	NA	NA
C26 H29 N	0.005799	14.97723	38.54234	level3	mzCloud	C05011	HMDB006	Phenylpro
C11 H12 N	0.000118	0.499075	NA	level4	HMDB	NA	HMDB001	Organohe
C22 H27 N	0.001834	4.812588	NA	level4	HMDB	NA	HMDB026	NA
C21 H16 F	0.001516	3.653054	NA	level4	HMDB	NA	HMDB025	NA
C15 H17 N	-0.00012	-0.42731	38.50113	level4	Public	NA	NA	NA
C29 H32 C	-0.00061	-0.92866	NA	level4	NA	NA	NA	NA
C27 H32 C	2.26E-05	0.038922	NA	level4	NA	NA	NA	NA
C24 H50 N	0.0005	0.977192	NA	level4	NA	NA	NA	NA
C41 H81 C	0.001623	2.168458	NA	level4	NA	NA	NA	NA
C53 H89 C	0.001645	1.705439	NA	level4	NA	NA	NA	NA
C6 H10 O	-6.60E-05	-0.33998	98.03874	level2	mzCloud	C08350	NA	NA
C34 H54 C	0.000619	1.077361	NA	level4	HMDB	NA	HMDB025	NA
C42 H52 C	0.001163	1.588177	NA	level4	HMDB	NA	HMDB025	NA
C10 H11 F	-0.00094	-3.16308	NA	level4	HMDB	NA	HMDB001	Nucleoside
C13 H16 C	-0.00094	-3.087	NA	level4	HMDB	NA	HMDB025	NA
C16 H20 C	-0.00011	-0.36149	NA	level4	HMDB	NA	HMDB025	NA
C30 H47 N	0.002531	4.64174	33.47751	level4	Public	NA	HMDB024	NA
C25 H20 N	0.001768	3.877044	NA	level4	HMDB	NA	HMDB024	NA
C22 H41 N	0.000132	0.306131	10.89274	level4	Public	NA	NA	NA
C25 H24 C	-0.00238	-4.4731	NA	level4	NA	NA	NA	NA
C4 H8 N2	6.45E-05	0.488186	85.89409	level1	BMDB	C00152	HMDB000	Organic ac
C9 H13 N	0.000233	1.270993	NA	level4	HMDB	NA	HMDB001	Benzenoid
C27 H25 C	0.001455	2.283271	NA	level4	NA	C16290	NA	Flavonoids

C20 H24 (	5.18E-05	0.127016	NA	level4	HMDB	NA	HMDB025	NA
C20 H24 (	37.95749	110289.5	14.88275	level4	Public	NA	HMDB003	Lignans, n
C9 H11 N	4.33E-05	0.262554	97.65814	level1	BMDB	C02057	HMDB000	Organic ac
C23 H28 (	17.02806	31294.39	5.481752	level4	Public	NA	NA	NA
C18 H22 (	-0.00417	-14.5765	65.49822	level2	mzCloud	C05300	HMDB000	Lipids and
C8 H9 N	0.000223	1.872277	70.42499	level2	Public	NA	HMDB025	NA
C17 H10 (	-0.00039	-1.47093	25.0859	level4	Public	NA	NA	NA
C18 H32 (	0.001426	3.492165	NA	level4	NA	NA	NA	NA
C21 H36 (	17.02646	46235.35	26.59294	level4	Public	NA	NA	NA
C26 H45 N	0.000825	1.465344	87.61053	level2	mzCloud	C03642	HMDB000	Lipids and
C23 H48 N	-0.00029	-0.57309	NA	level4	NA	NA	NA	NA
C41 H82 N	-0.00056	-0.74669	NA	level4	HMDB	NA	HMDB025	NA
C19 H29 B	-0.00029	-0.79259	NA	level4	NA	NA	NA	NA
C5 H10 O3	-1.41E-05	-0.1197	90.1634	level2	mzCloud	NA	NA	NA
C5 H13 O1	0.000332	0.887099	NA	level4	NA	C12347	NA	NA
C13 H17 N	8.75E-05	0.372298	20.68815	level4	Public	NA	NA	NA
C17 H15 (	-0.0004	-1.01016	NA	level4	HMDB	NA	HMDB024	NA
C32 H48 (	-0.00046	-0.84707	NA	level4	HMDB	NA	HMDB025	NA
C19 H25 (	0.001708	4.399589	NA	level4	HMDB	NA	HMDB025	NA
C15 H18 F	-0.00169	-4.83886	NA	level4	HMDB	NA	HMDB024	NA
C24 H32 (	6.87E-05	0.165049	NA	level4	HMDB	NA	HMDB025	NA
C20 H40 (	-2.17E-05	-0.0694	41.6669	level3	mzCloud	C06425	HMDB000	Lipids and
C25 H46 N	0.000525	1.042653	NA	level4	NA	NA	NA	NA
C5 H10 N2	8.03E-05	0.54971	90.89219	level1	Decode	C00819	HMDB000	Organic ac
C15 H18	0.000269	1.357656	30.97768	level4	Public	C09675	HMDB003	Lipids and
C30 H50 (	-54.0315	-110186	51.82228	level3	Public	NA	NA	NA
C16 H14 (	0.001017	3.302106	NA	level4	NA	C14250	NA	NA
C15 H10 N	0.001218	4.869274	52.77093	level4	Public	NA	NA	NA
C4 H4 O3	0.000146	1.454767	NA	level4	NA	C19524	NA	Organic ac
C12 H19 N	-0.00016	-0.56043	NA	level4	NA	C19429	NA	NA
C21 H20 (	-0.00046	-0.8771	NA	level4	NA	NA	NA	NA
C25 H28 (	21.98042	53848.01	15.19818	level4	Public	NA	NA	NA
C9 H12	8.43E-05	0.70234	NA	level4	NA	C14518	NA	Benzenoid
C32 H52 (	0.000612	1.148724	NA	level4	HMDB	NA	HMDB026	NA
C14 H24 (	9.11E-05	0.406437	NA	level4	NA	NA	NA	NA
C11 H16 N	-134.059	-500002	3.120966	level4	Public	C02267	HMDB001	Organohe
C12 H15 N	0.001085	4.997773	NA	level4	HMDB	NA	HMDB001	Organohe
C14 H16 (	-1.28E-06	-0.00464	NA	level4	HMDB	NA	HMDB025	NA
C25 H39 N	0.00173	3.397686	NA	level4	HMDB	NA	HMDB025	NA
C52 H99 N	0.001296	1.370866	NA	level4	NA	NA	NA	NA
C14 H16 N	-0.00025	-0.86616	NA	level4	NA	C21473	NA	NA
C22 H22 I	-0.00189	-3.75268	NA	level4	HMDB	NA	HMDB024	NA
C24 H39 N	0.000197	0.55143	NA	level4	HMDB	NA	HMDB025	NA
C17 H25 N	-0.00172	-3.39667	NA	level4	KEGG	C06692	NA	Antibiotics
C45 H85 (	0.001917	2.34797	NA	level4	NA	NA	NA	NA
C5 H13 O8	-0.00074	-3.20754	NA	level4	HMDB	NA	HMDB024	NA
C24 H17 N	-0.00135	-2.86537	NA	level4	HMDB	NA	HMDB024	NA
C9 H18 N5	0.001172	3.301515	NA	level4	NA	C18910	NA	NA
C25 H35 (	-0.00129	-2.51827	NA	level4	NA	NA	NA	NA
C16 H14 (	9.93E-05	0.390995	NA	level4	HMDB	NA	HMDB025	NA
C30 H45 N	-0.00191	-4.09634	NA	level4	NA	NA	NA	NA
C12 H10	0.000127	0.821782	NA	level4	HMDB	NA	HMDB024	NA
C21 H25 N	-9.94E-05	-0.22042	NA	level4	HMDB	NA	HMDB025	NA
C9 H9 Cl2	5.02E-05	0.201457	NA	level4	HMDB	NA	HMDB024	NA
C13 H25 N	-0.00085	-2.91228	NA	level4	KEGG	C05118	NA	Lipids and

C9 H15 N	0.000372	2.01152	72.60212	level2	mzCloud	NA	NA	NA
C29 H39 N	-0.0024	-4.97953	NA	level4	HMDB	NA	HMDB025	NA
C20 H17 F	0.00203	4.9993	NA	level4	HMDB	NA	HMDB025	NA
C16 H24 N	-0.00025	-0.75035	NA	level4	HMDB	NA	HMDB024	NA
C6 H11 N	0.000112	0.693615	NA	level4	HMDB	NA	HMDB000	Organic ac
C31 H46 C	0.001912	3.500258	NA	level4	NA	NA	NA	NA
C21 H33 N	-8.90E-05	-0.2026	NA	level4	HMDB	NA	HMDB001	Organic ac
C9 H12 N6	0.000478	1.897482	NA	level4	HMDB	NA	HMDB024	NA
C24 H41 N	-5.02E-05	-0.12819	NA	level4	NA	NA	NA	NA
C12 H26 C	-0.00017	-0.66219	NA	level4	HMDB	NA	HMDB024	NA
C15 H23 N	18.01047	54721.51	0.17469	level4	Public	NA	NA	NA
C38 H48 N	-0.00329	-4.2367	NA	level4	NA	C15736	NA	NA
C25 H48 F	21.9874	45967.75	33.58345	level3	mzCloud	NA	NA	NA
C19 H14 C	-0.0007	-2.07287	26.02088	level4	Public	NA	NA	NA
C21 H23 N	0.001061	2.644995	NA	level4	HMDB	NA	HMDB025	NA
C9 H8 N2	0.000462	2.406427	NA	level4	HMDB	NA	HMDB024	NA
C26 H54 N	0.00073	1.313705	NA	level4	NA	NA	NA	NA
C42 H80 N	-5.79E-05	-0.07491	NA	level4	NA	NA	NA	NA
C8 H9 N5	-0.00017	-0.731	NA	level4	HMDB	NA	HMDB006	Organohe
C23 H37 N	-0.00019	-0.48345	73.43832	level2	mzCloud	NA	NA	NA
C25 H36 N	-0.00241	-4.12214	NA	level4	HMDB	NA	HMDB001	Benzenoid
C16 H14 C	17.0264	53529.68	15.93517	level4	Public	NA	NA	NA
C24 H18 C	-0.00066	-1.7103	NA	level4	NA	NA	NA	NA
C21 H16 N	-0.00096	-3.24287	NA	level4	HMDB	NA	HMDB024	NA
C8 H10 O7	-0.00021	-0.85557	NA	level4	HMDB	NA	HMDB000	Organic ac
C18 H31 N	0.001746	4.941677	NA	level4	HMDB	NA	HMDB025	NA
C24 H49 C	-0.00204	-3.98525	NA	level4	NA	NA	NA	NA
C24 H41 N	-0.00011	-0.25807	NA	level4	NA	NA	NA	NA
C13 H10 C	17.02655	69198.76	9.579974	level4	Public	NA	NA	NA
C13 H12 C	2.09E-05	0.090201	NA	level4	HMDB	NA	HMDB025	NA
C24 H34 C	-0.00148	-2.96338	NA	level4	NA	C11658	NA	PR Prenol
C28 H35 N	0.000803	1.528026	NA	level4	HMDB	NA	HMDB025	NA
C21 H25 C	-0.00098	-2.32866	NA	level4	HMDB	NA	HMDB025	NA
C13 H12 N	17.02682	74649.59	12.96088	level4	Public	NA	NA	NA
C21 H29 N	-0.0013	-3.40148	NA	level4	HMDB	NA	HMDB024	NA
C16 H15 F	0.000385	1.004005	75.99824	level2	Public	C11806	HMDB000	Organohe
C14 H23 N	-0.00017	-0.60307	NA	level4	NA	NA	NA	NA
C15 H31 N	3.64E-05	0.141624	50.34929	level4	Public	NA	NA	NA
C3 H6 O5	7.94E-05	0.51531	NA	level4	NA	C21181	NA	NA
C31 H40 C	0.001589	2.497724	NA	level4	HMDB	NA	HMDB025	NA
C25 H23 N	0.002262	4.624668	NA	level4	HMDB	NA	HMDB024	NA
C24 H20 C	0.001231	2.82255	NA	level4	NA	NA	NA	NA
C34 H34 N	-0.00051	-0.8425	NA	level4	NA	C21624	NA	NA
C16 H14 C	17.02635	63039.73	14.8406	level4	Public	C16976	HMDB025	NA
C18 H22 C	0.000797	2.05239	NA	level4	HMDB	NA	HMDB025	NA
C19 H28 N	0.001131	2.43652	NA	level4	KEGG	C02808	NA	NA
C15 H12 C	-0.00092	-3.01003	NA	level4	HMDB	NA	HMDB025	NA
C12 H12 N	-3.31E-05	-0.11817	NA	level4	HMDB	NA	HMDB025	NA
C19 H21 N	-3.52E-05	-0.12608	NA	level4	HMDB	NA	HMDB001	Benzenoid
C6 H10 O	-1.17E-06	-0.0119	NA	level4	HMDB	NA	HMDB025	NA
C14 H25 N	-1.62E-05	-0.05981	29.09887	level4	Public	NA	NA	NA
C17 H32 C	-3.58E-06	-0.0126	NA	level4	HMDB	NA	HMDB025	NA
C25 H46 C	-0.00178	-3.4067	NA	level4	NA	NA	NA	NA
C19 H16 C	0.000111	0.274028	NA	level4	HMDB	NA	HMDB002	Organic ox

C26 H38 C	2.04E-05	0.045734	NA	level4	NA	NA	NA	NA
C8 H14 O4	0.000364	2.093531	NA	level4	HMDB	NA	HMDB025	NA
C33 H40 C	0.007783	11.50908	40.86589	level3	mzCloud	C17555	NA	PK Polyke
C14 H8 N2	-0.00106	-3.18397	NA	level4	KEGG	C01359	NA	NA
C41 H78 N	0.003364	4.337079	NA	level4	NA	NA	NA	NA
C16 H18 N	3.91E-05	0.144763	NA	level4	HMDB	NA	HMDB001	Organohe
C18 H22 C	-5.51E-05	-0.15738	90.29202	level2	mzCloud	C02538	HMDB000	Lipids and
C26 H24 F	-0.00138	-3.09396	NA	level4	HMDB	NA	HMDB025	NA
C17 H22 N	-0.00114	-3.02021	NA	level4	KEGG	C01007	NA	NA
C14 H20 N	6.39E-06	0.022826	NA	level4	HMDB	NA	HMDB024	NA
C43 H82 N	-0.00047	-0.60747	NA	level4	HMDB	NA	HMDB025	NA
C25 H45 N	-0.00192	-3.81582	NA	level4	NA	C15760	NA	NA
C27 H41 N	-0.00168	-3.3025	NA	level4	HMDB	NA	HMDB025	NA
C11 H9 N	-0.00093	-3.27503	NA	level4	HMDB	NA	HMDB024	NA
C21 H30 N	0.000796	1.692692	NA	level4	HMDB	NA	HMDB025	NA
C32 H50 C	0.000808	1.570772	NA	level4	HMDB	NA	HMDB024	NA
C15 H16 N	-0.00016	-0.59781	NA	level4	HMDB	NA	HMDB025	NA
C10 H20 C	-0.0002	-0.64299	NA	level4	HMDB	NA	HMDB024	NA
C35 H68 N	0.000176	0.253832	NA	level4	NA	NA	NA	NA
C17 H20 C	37.95735	124805.9	16.98807	level4	Public	NA	NA	NA
C12 H18 C	3.63E-05	0.20361	NA	level4	HMDB	NA	HMDB001	Benzenoid
C7 H6 O3	0.000187	1.354473	62.3965	level2	mzCloud	C16700	HMDB005	Organic ox
C5 H12 O5	37.95614	249599	27.62147	level4	Public	C01904	HMDB000	Organic ox
C30 H24 N	-0.00035	-0.66917	NA	level4	HMDB	NA	HMDB024	NA
C30 H50 C	0.000383	0.835326	NA	level4	HMDB	NA	HMDB025	NA
C10 H19 C	0.001071	3.408652	NA	level4	HMDB	NA	HMDB006	Lipids and
C6 H13 N	0.001592	8.892222	60.92595	level2	mzCloud	C12212	NA	NA
C16 H27 N	-0.00025	-0.84364	NA	level4	NA	NA	NA	NA
C30 H50 C	0.000685	1.311573	NA	level4	NA	NA	NA	NA
C16 H22 N	0.001505	3.633008	NA	level4	KEGG	C06667	NA	NA
C16 H24 N	6.34E-05	0.186432	NA	level4	HMDB	NA	HMDB025	NA
C24 H47 N	0.000225	0.486893	NA	level4	HMDB	NA	HMDB024	NA
C10 H12 N	-8.67E-05	-0.28887	NA	level4	KEGG	C05513	NA	Organohe
C18 H20 N	9.04E-05	0.262774	NA	level4	HMDB	NA	HMDB025	NA
C8 H9 N C	-0.0001	-0.41984	NA	level4	HMDB	NA	HMDB024	Organic ac
C38 H76 N	-4.54E-05	-0.0643	NA	level4	HMDB	NA	HMDB000	Lipids and
C21 H16 N	-0.00061	-1.50228	NA	level4	HMDB	NA	HMDB025	NA
C10 H17 N	0.000344	1.879175	NA	level4	HMDB	NA	HMDB001	Organohe
C21 H19 F	0.000971	2.680927	NA	level4	HMDB	NA	HMDB025	NA
C34 H50 N	-0.00314	-4.55124	NA	level4	HMDB	NA	HMDB001	Phenylpro
C25 H26 N	-0.00177	-3.6923	NA	level4	HMDB	NA	HMDB026	NA
C29 H46 C	-1.49E-05	-0.0364	NA	level4	NA	C22123	NA	NA
C3 H5 N S	0.000132	1.519249	NA	level4	HMDB	NA	HMDB025	NA
C24 H26 F	-0.00173	-4.03871	NA	level4	HMDB	NA	HMDB001	Organohe
C9 H11 N5	0.00013	0.587755	NA	level4	KEGG	C02587	NA	NA
C22 H29 N	-0.00036	-0.93667	NA	level4	HMDB	NA	HMDB025	NA
C19 H19 N	1.78E-05	0.054724	NA	level4	HMDB	NA	HMDB024	NA
C10 H13 N	-0.00403	-15.081	31.4028	level3	mzCloud	C00212	HMDB000	Nucleoside
C41 H44 N	-0.00158	-2.01759	NA	level4	HMDB	NA	HMDB025	NA

C13 H18 C	-0.00019	-0.6914	NA	level4	HMDB	NA	HMDB024	NA
C31 H48 C	0.002262	4.381742	NA	level4	HMDB	NA	HMDB001	Benzenoid
C20 H18 C	-0.00072	-2.47758	NA	level4	HMDB	NA	HMDB025	NA
C21 H40 C	7.94E-05	0.233353	NA	level4	NA	NA	NA	NA
C21 H36 C	-18.0106	-44998.7	8.609085	level4	Public	NA	NA	NA
C19 H24 N	6.28E-05	0.182459	NA	level4	HMDB	NA	HMDB001	Benzenoid
C7 H8 O8	-0.00088	-3.98809	NA	level4	KEGG	C04115	NA	NA
C39 H70 C	0.000241	0.388919	NA	level4	HMDB	NA	HMDB000	Lipids and
C18 H22 C	-0.00023	-0.63138	NA	level4	HMDB	NA	HMDB001	Lipids and
C33 H56 C	0.000607	1.017665	NA	level4	NA	NA	NA	NA
C31 H35 N	-0.00152	-2.70452	NA	level4	HMDB	NA	HMDB024	NA
C6 H9 N C	0.000213	1.676747	NA	level4	HMDB	NA	HMDB025	NA
C21 H14 C	-0.00075	-2.17842	NA	level4	HMDB	NA	HMDB024	NA
C6 H16 N2	0.000199	1.716616	NA	level4	HMDB	NA	HMDB024	NA
C22 H34 C	-18.0106	-47616.7	31.31936	level4	Public	NA	NA	NA
C7 H9 N C	0.000717	3.049142	NA	level4	NA	C21577	NA	NA
C15 H8 O	1.91E-05	0.063538	68.84134	level2	Public	NA	NA	NA
C18 H15 C	-2.16E-05	-0.04649	NA	level4	HMDB	NA	HMDB006	Organic o
C26 H32 F	7.04E-05	0.142414	NA	level4	HMDB	NA	HMDB025	NA
C8 H11 N	-0.00021	-0.98638	NA	level4	HMDB	NA	HMDB025	NA
C10 H12 N	0.000377	1.494974	NA	level4	HMDB	NA	HMDB000	Nucleoside
C26 H48 N	-0.00284	-4.14498	NA	level4	NA	C18005	NA	NA
C28 H39 N	17.02848	33970.5	22.43779	level4	Public	NA	NA	NA
C22 H33 N	-0.00011	-0.25919	NA	level4	HMDB	NA	HMDB006	NA
C9 H18 N2	0.000404	1.99753	NA	level4	HMDB	NA	HMDB025	NA
H Cl O3	-1.32E-05	-0.15741	NA	level4	KEGG	C01485	NA	NA
C35 H40 N	0.002784	4.933329	NA	level4	KEGG	C09050	NA	NA
C20 H28 C	-0.00032	-0.6894	NA	level4	NA	C10715	NA	NA
C31 H41 C	-0.00126	-2.03533	NA	level4	NA	C20138	NA	NA
C26 H14 C	0.001097	1.692563	NA	level4	HMDB	NA	HMDB024	NA
C19 H23 C	0.001624	4.079562	NA	level4	KEGG	C09288	NA	Terpenoid
C23 H36 C	-6.92E-05	-0.20103	NA	level4	HMDB	NA	HMDB024	NA
C15 H18 N	-0.0013	-4.35681	NA	level4	HMDB	NA	HMDB025	NA
C34 H46 C	-0.00199	-4.08181	NA	level4	NA	NA	NA	NA
C12 H22 C	0.000109	0.51125	NA	level4	NA	NA	NA	NA
C8 H5 N C	0.00015	1.144907	NA	level4	HMDB	NA	HMDB025	NA
C15 H11 C	-0.00023	-0.7087	NA	level4	HMDB	NA	HMDB024	NA
C15 H12 C	0.001314	3.628651	NA	level4	HMDB	NA	HMDB025	NA
C29 H34 C	0.001096	1.857317	NA	level4	NA	NA	NA	NA
C26 H42 C	-0.00108	-2.16174	NA	level4	NA	NA	NA	NA
C10 H7 Cl	0.00013	0.382389	NA	level4	HMDB	NA	HMDB024	NA
C20 H15 N	17.02577	39682.32	1.065565	level4	Public	NA	NA	NA
C12 H11 N	-0.00141	-5.58984	41.56988	level3	Public	D00386	HMDB000	Organohe
C19 H39 N	-1.49E-05	-0.04754	NA	level4	NA	NA	NA	NA
C11 H15 N	-0.0004	-1.3506	95.03606	level1	BMDB	C00170	HMDB000	Nucleoside
C16 H32 C	9.64E-05	0.37635	NA	level4	HMDB	NA	HMDB025	NA
C10 H17 N	-0.00229	-4.90944	NA	level4	HMDB	NA	HMDB025	NA
C15 H23 N	-18.0106	-60609.5	20.65173	level4	Public	NA	NA	NA
C3 H4 N2	-0.00053	-3.60554	NA	level4	NA	C22085	NA	NA
C33 H42 C	-0.00185	-3.56182	NA	level4	KEGG	C09941	NA	NA
C10 H10 C	3.20E-05	0.219194	NA	level4	HMDB	NA	HMDB024	NA
C34 H48 C	0.00036	0.615468	NA	level4	NA	C11964	NA	NA
C7 H10 N	-0.03606	-182.041	53.96587	level3	Decode	C16366	HMDB000	Organic ni
C21 H34 C	-6.74E-05	-0.18403	28.16529	level4	Public	NA	NA	NA

C7 F16	0.001574	4.057642	NA	level4	HMDB	NA	HMDB025	NA
C38 H38 N	0.001874	2.899488	NA	level4	HMDB	NA	HMDB025	NA
C21 H30 O	-0.00016	-0.41718	92.22637	level2	mzCloud	NA	NA	NA
C24 H26 O	1.56E-05	0.034033	NA	level4	NA	NA	NA	NA
C24 H47 N	0.000538	1.12781	NA	level4	HMDB	NA	HMDB025	NA
C26 H40 O	-9.57E-05	-0.23906	NA	level4	HMDB	NA	HMDB024	NA
C14 H20 O	17.02637	59925.43	27.31247	level4	Public	NA	NA	NA
C20 H28 O	17.02525	56711.35	19.45465	level4	Public	C12078	HMDB006	Lipids and
C15 H15 N	-0.00024	-0.84246	NA	level4	HMDB	NA	HMDB024	NA
C15 H24 N	-4.93E-05	-0.19844	NA	level4	HMDB	NA	HMDB024	NA
C11 H19 N	37.95562	129492.3	8.764367	level4	Public	NA	NA	NA
C20 H39 N	-0.0002	-0.56579	NA	level4	NA	NA	NA	NA
C5 H11 N	8.00E-05	0.441759	NA	level4	HMDB	NA	HMDB001	Organic ac
C10 H13 N	0.00016	0.818304	NA	level4	HMDB	NA	HMDB001	Phenylpro
C10 H10 O	0.00014	0.959202	63.47718	level2	Public	NA	NA	NA
C25 H35 N	37.95445	88420.08	7.63813	level4	Public	NA	NA	NA
C8 H11 N	0.000106	0.691723	79.36646	level2	mzCloud	C03758	HMDB000	Benzenoid
C14 H11 O	-1.90E-05	-0.06123	81.9962	level2	mzCloud	NA	HMDB001	Benzenoid
C20 H31 N	-0.0008	-2.30211	NA	level4	HMDB	NA	HMDB025	NA
C21 H22 O	6.28E-05	0.163887	NA	level4	NA	C18491	NA	NA
C16 H17 N	0.000754	3.154659	NA	level4	HMDB	NA	HMDB025	NA
C13 H20 O	-0.00073	-2.66756	NA	level4	HMDB	NA	HMDB024	NA
C18 H30 N	-0.00223	-4.51573	NA	level4	HMDB	NA	HMDB024	NA
C25 H22 O	0.000901	2.001021	NA	level4	NA	NA	NA	NA
C40 H74 O	0.000743	0.974805	NA	level4	NA	NA	NA	NA
C16 H14 N	0.000233	0.875064	NA	level4	HMDB	NA	HMDB025	NA
C24 H47 O	-0.00044	-0.78137	NA	level4	NA	NA	NA	NA
C19 H22 N	-6.14E-05	-0.14537	4.588921	level4	Public	NA	NA	NA
C14 H14 O	-8.85E-06	-0.02854	NA	level4	HMDB	NA	HMDB024	Phenylpro
C22 H27 F	-8.59E-05	-0.19332	NA	level4	HMDB	NA	HMDB025	NA
C20 H24 F	0.000134	0.344748	NA	level4	HMDB	NA	HMDB024	NA
C9 H12 N2	7.12E-05	0.395298	NA	level4	HMDB	NA	HMDB026	NA
C19 H18 N	-1.67E-05	-0.04518	NA	level4	HMDB	NA	HMDB025	NA
C5 H9 N O	0.000129	1.124103	97.75867	level2	mzCloud	C00763	HMDB000	Organic ac
C10 H13 N	0.000972	2.668734	8.488124	level4	Public	NA	HMDB006	Nucleoside
C30 H46 O	0.000176	0.402419	NA	level4	NA	C21084	NA	NA
C12 H18 N	-5.28E-05	-0.15244	26.53462	level4	Public	NA	NA	NA
C11 H15 N	0.000146	0.825471	NA	level4	HMDB	NA	HMDB024	NA
C30 H49 N	-0.00303	-4.70465	NA	level4	NA	C11367	NA	NA
C27 H27 N	-0.00063	-1.47198	NA	level4	HMDB	NA	HMDB025	NA
C22 H31 N	-0.00118	-2.75603	NA	level4	HMDB	NA	HMDB025	NA
C17 H24 O	-5.44E-07	-0.00209	NA	level4	HMDB	NA	HMDB024	NA
C13 H16 O	-0.00018	-0.63411	NA	level4	HMDB	NA	HMDB001	Organic ox
C24 H39 N	-0.00015	-0.38909	NA	level4	NA	NA	NA	NA
C44 H78 N	-0.00023	-0.29131	NA	level4	NA	NA	NA	NA
C10 H11 N	-0.00047	-1.83144	NA	level4	HMDB	NA	HMDB024	NA
C14 H14 N	-8.17E-06	-0.03375	NA	level4	HMDB	NA	HMDB024	NA
C24 H35 N	0.001257	2.288827	NA	level4	HMDB	NA	HMDB025	NA
C14 H19 N	-0.00063	-1.8732	NA	level4	HMDB	NA	HMDB025	NA
C28 H46 O	-0.00229	-4.79649	NA	level4	NA	NA	NA	NA
C14 H21 N	0.000848	2.442106	NA	level4	HMDB	NA	HMDB024	NA

C18 H28 N	-0.00015	-0.26532	NA	level4	NA	C21384	NA	NA
C15H15Cl2	0.001287	2.891933	NA	level4	NA	C13962	NA	NA
C44 H84 N	0.000587	0.717865	NA	level4	NA	NA	NA	NA
C34 H47 N	-0.00202	-3.20456	NA	level4	KEGG	C08683	NA	Alkaloids
C19 H32 C	-0.00016	-0.53429	44.50813	level3	Public	NA	NA	NA
C6 H9 N C	0.00024	1.25827	NA	level4	HMDB	NA	HMDB024	NA
C6 H10 O4	0.000109	0.612929	NA	level4	HMDB	NA	HMDB024	Lipids and
C11 H11 F	0.000309	1.056448	NA	level4	HMDB	NA	HMDB006	Benzenoid
C20 H28 C	37.95405	120030.3	11.56959	level4	Public	NA	NA	NA
C3 H7 O4	5.81E-05	0.420863	NA	level4	HMDB	NA	HMDB001	Organic ac
C14 H22 C	0.000186	0.781102	25.91467	level4	Public	NA	NA	NA
C6 H9 N C	0.000206	1.856733	NA	level4	HMDB	NA	HMDB025	NA
C21 H38 C	0.000122	0.345044	NA	level4	HMDB	NA	HMDB025	NA
C24 H34 C	37.95438	87406.07	13.40187	level4	Public	NA	NA	NA
C13 H19 N	0.00014	0.552531	NA	level4	HMDB	NA	HMDB024	NA
C33 H38 N	0.00244	4.708315	NA	level4	KEGG	C09211	NA	Alkaloids
C10 H20 N	-0.0002	-0.815	NA	level4	HMDB	NA	HMDB025	NA
C23 H46 N	0.000431	0.900021	NA	level4	NA	NA	NA	NA
C25 H40 C	17.02659	40511.75	28.56423	level4	Public	NA	NA	NA
C15 H23 N	-0.00012	-0.40101	NA	level4	HMDB	NA	HMDB025	NA
C19 H30 C	37.95405	107153	3.723862	level4	Public	NA	NA	NA
C30 H52 N	-0.0029	-4.45977	NA	level4	NA	NA	NA	NA
C25 H18 N	0.001929	4.570369	NA	level4	HMDB	NA	HMDB025	NA
C33 H35 N	0.002003	3.445083	NA	level4	HMDB	NA	HMDB001	Organohe
C27 H43 C	0.001825	4.052597	NA	level4	NA	NA	NA	NA
C27 H42 N	0.000596	1.348278	NA	level4	NA	NA	NA	NA
C21 H18 C	0.000205	0.443205	66.63217	level2	Public	NA	NA	NA
C33 H43 N	0.002636	4.598332	NA	level4	HMDB	NA	HMDB025	NA
C22 H24 N	0.00072	1.525443	NA	level4	HMDB	NA	HMDB025	NA
C5 H11 N	0.000233	1.562593	95.47221	level2	mzCloud	C00073	HMDB000	Organic ac
C29 H44 C	0.001805	3.08968	NA	level4	HMDB	NA	HMDB001	Benzenoid
C25 H41 N	7.39E-05	0.183339	NA	level4	NA	NA	NA	NA
C15 H22 N	0.002047	3.848314	NA	level4	NA	C21351	NA	NA
C24 H38 C	-0.00021	-0.53889	73.32935	level1	BMDB	C03690	NA	NA
C12 H19 N	-0.00015	-0.57782	NA	level4	NA	NA	NA	NA
C4 H10 N	-0.00023	-1.237	NA	level4	HMDB	NA	HMDB024	NA
C22 H30 C	0.000166	0.443141	6.456495	level4	Public	NA	NA	NA
C10 H14 N	-0.00017	-0.49075	88.87391	level1	BMDB	C00362	HMDB000	Nucleoside
C H5 O4 P	0.000147	1.315069	NA	level4	KEGG	C06455	NA	NA
C15 H25 N	-1.39E-05	-0.04481	NA	level4	HMDB	NA	HMDB025	NA
C8 H16 Cl	17.02623	96143.4	7.721351	level4	Public	NA	NA	NA
C9 H10 O6	0.000274	1.112832	NA	level4	HMDB	NA	HMDB024	NA
C18 H20 C	4.92E-05	0.163805	NA	level4	HMDB	NA	HMDB024	NA
C26 H34 N	-0.00043	-0.85562	NA	level4	NA	C15675	NA	NA
C21 H23 N	-0.00106	-2.43466	NA	level4	HMDB	NA	HMDB025	NA
C24 H29 N	-0.00188	-3.76103	NA	level4	HMDB	NA	HMDB025	NA
C15 H15 N	0.000811	3.600531	NA	level4	HMDB	NA	HMDB024	NA
C20 H16 N	-0.00139	-4.08165	NA	level4	HMDB	NA	HMDB024	NA
C18 H18 N	0.000111	0.356844	NA	level4	HMDB	NA	HMDB025	NA
C3H8NO5	0.000448	1.963992	NA	level4	NA	C18564	NA	NA
C10 H7 N	0.00011	0.698533	NA	level4	NA	C14788	NA	Benzenoid
C19 H22 N	-8.15E-05	-0.23833	NA	level4	NA	C19061	NA	NA
C17 H27 N	6.59E-07	0.002377	NA	level4	HMDB	NA	HMDB000	Benzenoid
C35 H62 C	0.000345	0.612846	NA	level4	HMDB	NA	HMDB000	Lipids and

C14 H19 N	-0.0014	-4.83652	44.59456	level4	Public	NA	HMDB024	NA
C13 H8 O4	0.000151	0.661144	57.45582	level3	Public	NA	HMDB001	Phenylpro
C20 H30 C	-18.0106	-56598.2	79.24168	level2	mzCloud	NA	NA	NA
C14 H13 N	-0.00118	-4.62822	NA	level4	HMDB	NA	HMDB024	NA
C9 H10 O3	-18.0103	-108455	91.95177	level2	BMDB	C01198	HMDB003	Phenylpro
C9 H12 N2	-1.61E-05	-0.08231	NA	level4	HMDB	NA	HMDB024	NA
C26 H26 C	0.00081	1.528052	NA	level4	NA	NA	NA	NA
C16 H24 C	-7.78E-05	-0.21601	13.14472	level4	Public	NA	NA	NA
C7 H11 N	0.000381	3.490988	NA	level4	NA	C11520	NA	NA
C27 H44 C	-0.00099	-1.71037	NA	level4	NA	NA	NA	NA
C26 H30 C	-0.00039	-0.75706	7.853531	level4	Public	NA	NA	NA
C9 H14 O	0.000143	1.035384	87.52666	level2	mzCloud	C14743	HMDB003	Organic o
C22 H27 N	-0.00025	-0.63883	NA	level4	NA	C16709	NA	Alkaloids
C15 H22 C	-0.0002	-0.70389	27.52744	level4	Public	NA	NA	NA
C16 H26 C	-18.0106	-60402.3	49.61383	level3	mzCloud	NA	NA	NA
C26 H34 C	0.001953	3.857291	NA	level4	KEGG	C01593	NA	NA
C21 H24	-0.00014	-0.51757	NA	level4	HMDB	NA	HMDB024	NA
C14 H22	0.000266	1.396938	NA	level4	NA	NA	NA	NA
C43 H78 N	-0.00025	-0.32955	NA	level4	NA	NA	NA	NA
C12 H14 C	-0.00012	-0.56632	45.15797	level3	Public	NA	NA	NA
C15 H22 N	-2.00E-05	-0.07617	NA	level4	HMDB	NA	HMDB025	NA
C29 H38 N	0.000783	1.299953	NA	level4	HMDB	NA	HMDB001	Phenylpro
C18 H20 N	-6.31E-05	-0.17528	NA	level4	HMDB	NA	HMDB025	NA
C20 H34 C	17.02643	48064.6	37.20153	level3	mzCloud	NA	NA	NA
C50 H80 N	0.001176	1.194641	NA	level4	NA	C16905	NA	NA
C30 H50 C	0.000148	0.346548	NA	level4	HMDB	NA	HMDB000	Lipids and
C10 H12 N	0.000554	2.474446	NA	level4	HMDB	NA	HMDB025	NA
C18 H24 N	-0.00021	-0.65992	NA	level4	HMDB	NA	HMDB024	NA
C21 H19 C	-7.49E-06	-0.01901	NA	level4	HMDB	NA	HMDB024	NA
C5 H12 N2	-0.09881	-748.068	34.78024	level3	BMDB	C00515	HMDB000	Organic ac
C29 H44 C	0.000389	0.853017	NA	level4	NA	NA	NA	NA
C19 H21 C	-0.00174	-4.15149	NA	level4	HMDB	NA	HMDB025	NA
C30 H52 N	0.000405	0.710541	NA	level4	HMDB	NA	HMDB001	Lipids and
C16 H20 N	-7.98E-05	-0.24914	NA	level4	HMDB	NA	HMDB025	NA
C41 H80 N	0.000409	0.548087	NA	level4	HMDB	NA	HMDB025	NA
C20 H24 C	37.95813	96795.64	1.671337	level4	Public	NA	NA	NA
C22 H23 N	-0.00094	-2.6019	NA	level4	HMDB	NA	HMDB025	NA
C12 H20 C	0.000684	2.996599	NA	level4	HMDB	NA	HMDB000	Lipids and
C7 H10 O6	0.000132	0.693118	NA	level4	NA	NA	NA	NA
C22 H24 C	21.98036	54930.01	55.10521	level4	Public	NA	NA	NA
C44 H84 C	0.001285	1.628917	NA	level4	NA	NA	NA	NA
C8 H7 N C	0.000425	2.346824	NA	level4	HMDB	NA	HMDB025	NA
C9 H16 O4	0.000654	2.593273	NA	level4	HMDB	NA	HMDB025	NA
C20 H28 C	17.02801	42980.65	13.12217	level4	Public	NA	NA	NA
C24 H31 C	-0.00166	-3.97282	NA	level4	HMDB	NA	HMDB024	NA
C24 H16 N	-0.00046	-1.27317	NA	level4	HMDB	NA	HMDB025	NA
C6 H13 N	-17.0264	-104414	93.79607	level3	Decode	NA	HMDB025	NA
C6 H16 O1	-0.00231	-4.94227	NA	level4	NA	C20422	NA	NA
C18 H24 C	4.04E-05	0.140059	NA	level4	HMDB	NA	HMDB024	NA
C17 H18 C	37.95524	149353.3	34.46968	level4	Public	NA	NA	NA
C24 H34 N	0.001608	3.882028	NA	level4	HMDB	NA	HMDB025	NA
C33 H30 C	0.001517	2.818747	NA	level4	NA	NA	NA	NA

C16 H11 F	0.00083	2.593875	NA	level4	HMDB	NA	HMDB024	NA
C18 H20 C	0.000236	0.677409	NA	level4	HMDB	NA	HMDB025	NA
C24 H43 E	-0.00176	-3.97746	NA	level4	NA	NA	NA	NA
C24 H29 N	0.000315	0.640419	NA	level4	NA	C11813	NA	Alkaloids
C11 H18 N	5.06E-05	0.187139	NA	level4	HMDB	NA	HMDB025	NA
C18 H27 N	-0.00135	-3.89934	NA	level4	HMDB	NA	HMDB025	NA
C50 H72 N	-0.00318	-3.20183	6.905011	level4	Public	NA	NA	NA
C3 H3 N S	-2.54E-05	-0.29896	NA	level4	HMDB	NA	HMDB025	NA
C9 H8 O3	0.000195	1.188176	92.80674	level2	mzCloud	C01772	HMDB000	Phenylpro
C20 H23 N	-0.0009	-2.23822	NA	level4	HMDB	NA	HMDB026	NA
C24 H44 C	-0.00131	-2.95606	NA	level4	HMDB	NA	HMDB026	NA
C3 H3 N3	5.49E-05	0.485792	NA	level4	HMDB	NA	HMDB024	NA
C5 H3 F N	0.000755	4.341365	NA	level4	HMDB	NA	HMDB024	NA
C19 H22 C	17.02986	49199.1	32.51955	level3	mzCloud	C01699	HMDB000	Lipids and
C27 H34 C	-0.00072	-1.34529	8.408504	level4	Public	NA	NA	NA
C22 H29 F	0.001356	3.605163	NA	level4	HMDB	NA	HMDB025	NA
C7 H9 O4	0.000264	1.402931	NA	level4	HMDB	NA	HMDB024	NA
C25H25N2	-0.00198	-4.11741	NA	level4	NA	C17880	NA	NA
C27 H33 N	-0.00016	-0.37	NA	level4	HMDB	NA	HMDB025	NA
C29 H47 N	0.000466	0.922144	NA	level4	NA	C15684	NA	NA
C6 H15 N	0.000127	1.254069	NA	level4	HMDB	NA	HMDB025	NA
C19 H22 C	21.98062	66578.35	19.61906	level4	Public	NA	NA	NA
C19 H18 N	-0.001	-2.95451	NA	level4	HMDB	NA	HMDB025	NA
C21 H27 N	-2.81E-05	-0.06934	NA	level4	HMDB	NA	HMDB025	NA
C10 H12 N	5.55E-05	0.168647	95.82816	level1	BMDB	C00575	HMDB000	Nucleosid
C19 H20 C	17.02722	51891.52	16.51756	level4	Public	NA	NA	NA
C15 H22 C	-0.0001	-0.44017	34.20176	level4	Public	NA	NA	NA
C15 H18 N	0.000286	0.739644	NA	level4	NA	C15666	NA	NA
C37 H39 C	-6.35E-05	-0.08235	NA	level4	NA	NA	NA	NA
C43 H87 N	-0.00031	-0.40249	NA	level4	HMDB	NA	HMDB001	Lipids and
C26 H31 N	-0.00174	-3.84266	NA	level4	NA	NA	NA	NA
C8 H4 N2	-0.00129	-4.73527	NA	level4	HMDB	NA	HMDB024	NA
C31 H42 C	0.001952	3.220325	NA	level4	KEGG	C09074	NA	Terpenoid
C42 H32 C	0.002996	4.404256	NA	level4	HMDB	NA	HMDB024	NA
C14 H9 Cl	0.000781	1.877845	NA	level4	KEGG	C06651	NA	NA
C14 H8 O2	-0.00066	-3.16353	NA	level4	HMDB	NA	HMDB024	NA
C34 H32 N	-0.00018	-0.30266	NA	level4	KEGG	C03516	NA	NA
C7 H8 O5	0.000179	1.040478	NA	level4	NA	C18306	NA	NA
C27 H44 C	0.000355	0.765449	NA	level4	HMDB	NA	HMDB025	NA
C30 H48 C	-0.00015	-0.28886	4.971699	level4	Public	NA	NA	NA
C12 H14 C	0.00134	4.685169	NA	level4	HMDB	NA	HMDB005	Organic o
C18 H16 C	-0.00095	-2.63454	57.01901	level3	Public	NA	HMDB003	Phenylpro
C11 H18 C	-2.74E-05	-0.12775	NA	level4	NA	NA	NA	NA
C56 H84 C	-0.00101	-1.1897	NA	level4	NA	NA	NA	NA
C18 H16 C	9.42E-05	0.279478	NA	level4	HMDB	NA	HMDB025	NA
C16 H19 N	-0.00012	-0.45247	NA	level4	HMDB	NA	HMDB025	NA
C23 H24 C	17.0281	39771.61	11.54643	level4	Public	NA	NA	NA
C22 H28 N	-0.00027	-0.64862	NA	level4	HMDB	NA	HMDB025	NA
C28 H48 C	0.000392	0.582786	NA	level4	NA	NA	NA	NA
C12 H20 C	-0.00019	-0.51015	NA	level4	NA	C20304	NA	NA
C9 H12 N2	0.000207	0.975082	NA	level4	HMDB	NA	HMDB024	NA
C20 H35 N	-8.60E-05	-0.25501	NA	level4	HMDB	NA	HMDB024	NA

C24 H23 N	0.0017	3.532335	NA	level4	HMDB	NA	HMDB025	NA
C15 H22 N	-0.00014	-0.48838	NA	level4	HMDB	NA	HMDB025	NA
C5 H12 O	-0.00357	-14.5184	36.97297	level3	mzCloud	C00129	HMDB000	Lipids and
C16 H25 N	7.45E-05	0.266728	NA	level4	HMDB	NA	HMDB025	NA
C19 H26 C	-2.69E-05	-0.09408	67.8157	level2	mzCloud	C20217	NA	Polyketide
C24 H28 N	-0.0012	-2.55638	NA	level4	HMDB	NA	HMDB024	NA
C13 H21 C	0.000746	2.247629	NA	level4	KEGG	C02087	NA	NA
C14 H16 N	-0.00017	-0.67199	NA	level4	NA	C10605	NA	NA
C19 H18 N	-0.00081	-2.5179	NA	level4	HMDB	NA	HMDB024	NA
C20 H13 N	-0.00056	-1.64472	NA	level4	HMDB	NA	HMDB025	NA
C24 H24 C	-0.00119	-3.45964	NA	level4	HMDB	NA	HMDB025	NA
C23 H21 C	0.000456	0.982351	NA	level4	HMDB	NA	HMDB024	NA
C21 H21 N	0.002101	4.74208	NA	level4	HMDB	NA	HMDB025	NA
C42 H74 N	-0.00293	-3.89566	NA	level4	NA	NA	NA	NA
C7 H6 N2	0.000178	1.185008	NA	level4	HMDB	NA	HMDB024	NA
C8 H6 O	-4.23E-05	-0.35794	NA	level4	NA	C14512	NA	Organohe
C20 H24 C	0.00016	0.48905	NA	level4	HMDB	NA	HMDB025	NA
C17 H21 N	-0.00021	-0.73843	57.25227	level3	mzCloud	C08526	HMDB001	Alkaloids a
C18 H14 F	0.000263	0.618598	NA	level4	HMDB	NA	HMDB024	NA
C27 H46 C	-0.0001	-0.26069	NA	level4	HMDB	NA	HMDB024	NA
C25 H22 C	0.001194	2.561572	NA	level4	NA	NA	NA	NA
C30 H48 C	0.00025	0.568289	39.60139	level4	Public	NA	NA	NA
C10 H10 C	-18.0103	-92809	89.28889	level1	BMDB	C01494	HMDB000	Phenylpro
C10 H8 S	-0.00046	-2.90466	NA	level4	HMDB	NA	HMDB024	NA
C12 H14 C	-6.88E-05	-0.30973	NA	level4	HMDB	NA	HMDB024	NA
C23 H26 N	-1.16E-05	-0.02838	NA	level4	HMDB	NA	HMDB024	NA
C25 H36 C	17.02578	36674.45	20.02891	level4	Public	NA	NA	NA
C15 H14 C	17.02641	65971.04	22.36959	level4	Public	NA	NA	NA
C30 H48 C	0.000453	0.797204	NA	level4	HMDB	NA	HMDB024	NA
C40 H52 C	-3.78E-05	-0.06332	NA	level4	HMDB	NA	HMDB026	NA
C15 H22 C	0.000146	0.625613	62.58322	level2	mzCloud	C09712	NA	Terpenoid
C5 H8 N6	0.000525	3.124413	NA	level4	HMDB	NA	HMDB025	NA
C23 H45 N	2.93E-05	0.073374	95.84438	level2	mzCloud	NA	NA	NA
C32 H39 N	0.000379	0.732089	NA	level4	NA	C20552	NA	NA
C11 H18 C	0.001042	3.748273	3.715958	level4	Public	NA	NA	NA
C30 H49 N	21.97693	38196.28	32.18595	level3	mzCloud	NA	NA	NA
C21 H24 N	-0.00145	-4.16836	NA	level4	HMDB	NA	HMDB025	NA
C11 H14 C	0.000197	1.214196	NA	level4	NA	NA	NA	NA
C27 H44	-8.90E-05	-0.24173	NA	level4	HMDB	NA	HMDB024	NA
C8 H16	0.00013	1.163343	NA	level4	HMDB	NA	HMDB025	NA
C23 H30 N	-0.00146	-3.19247	NA	level4	HMDB	NA	HMDB025	NA
C11 H18 N	-5.66E-05	-0.21915	NA	level4	NA	C20819	NA	NA
C13 H16 C	0.000246	1.205308	6.813796	level4	Public	C10702	HMDB003	Organic o
C16 H24 N	-0.00025	-0.79059	NA	level4	HMDB	NA	HMDB025	NA
C28 H39 N	-0.00042	-0.68273	NA	level4	HMDB	NA	HMDB025	NA
C23 H43 N	-0.0016	-2.64293	NA	level4	HMDB	NA	HMDB025	NA
C13 H14	0.000147	0.862217	NA	level4	KEGG	C08395	NA	NA
C15 H14 C	17.02642	62121.15	15.95097	level4	Public	NA	NA	NA
C12 H20 N	-7.71E-05	-0.28738	NA	level4	HMDB	NA	HMDB025	NA
C30 H34 C	-0.00244	-4.40607	NA	level4	NA	C17686	NA	NA
C18 H35 N	-4.49E-07	-0.00126	NA	level4	NA	C11328	NA	NA
C18 H26 N	21.9809	53072.96	12.08075	level4	Public	NA	NA	NA

C16 H24 N	-0.00061	-1.59972	NA	level4	HMDB	NA	HMDB025	NA
C26 H29 N	0.001751	3.879848	NA	level4	NA	NA	NA	NA
C25 H41 N	0.000134	0.345118	NA	level4	HMDB	NA	HMDB025	NA
C39 H69 C	0.003985	4.929551	NA	level4	NA	NA	NA	NA
C35 H64 N	-0.00028	-0.40966	NA	level4	NA	NA	NA	NA
C6 H7 N5	0.000312	1.723989	67.85551	level2	mzCloud	NA	NA	NA
C16 H18 C	17.02635	50358.88	25.40324	level4	Public	NA	HMDB002	Organic o
C18 H32 C	0.001603	4.26098	NA	level4	NA	NA	NA	NA
C41 H70 C	0.000363	0.564648	NA	level4	HMDB	NA	HMDB000	Lipids and
C25 H25 N	-0.00516	-11.9654	33.15496	level3	mzCloud	NA	NA	NA
C8 H16 O3	0.000102	0.639553	87.53904	level1	Decode	C16651	HMDB001	Lipids and
C17 H16 N	17.02646	60782.49	6.862401	level4	Public	NA	NA	NA
C24 H38 C	-0.00192	-3.8293	NA	level4	NA	C17888	NA	NA
C10 H16 N	-0.00022	-0.7563	NA	level4	HMDB	NA	HMDB001	Organic ac
C20 H24 N	-0.00404	-12.4717	44.12892	level3	mzCloud	NA	NA	NA
C13 H19 N	37.9574	149951.3	38.47419	level4	Public	NA	NA	NA
C28 H42 N	-0.00219	-3.18668	NA	level4	HMDB	NA	HMDB025	NA
C31 H57 C	0.002168	3.684408	NA	level4	NA	NA	NA	NA
C21 H32 C	-0.00096	-2.63919	74.02432	level2	mzCloud	C05470	HMDB000	Lipids and
C33 H50 C	0.000881	1.492783	NA	level4	NA	NA	NA	NA
C13 H15 N	-0.00017	-0.59179	NA	level4	NA	C16518	NA	NA
C16 H22 C	0.00147	4.140074	NA	level4	HMDB	NA	HMDB025	NA
C53 H87 C	0.002386	2.478467	NA	level4	NA	NA	NA	NA
C40 H78 N	-2.14E-05	-0.02927	NA	level4	HMDB	NA	HMDB000	Lipids and
C2 H6 O4	7.59E-05	0.60203	NA	level4	HMDB	NA	HMDB003	Organic ac
C16 H30 C	7.33E-05	0.307532	74.80762	level2	mzCloud	NA	HMDB003	Organic o
C31 H38 C	-0.00134	-2.00767	NA	level4	HMDB	NA	HMDB024	NA
C16 H18 C	-5.63E-06	-0.01585	NA	level4	HMDB	NA	HMDB025	NA
C34 H65 C	-0.00172	-2.79416	NA	level4	NA	NA	NA	NA
C12 H22 C	-0.00015	-0.6687	78.186	level2	mzCloud	C02678	HMDB000	Lipids and
C27 H38 C	0.00032	0.812058	NA	level4	NA	NA	NA	NA
C27 H44 N	0.000859	1.63494	NA	level4	HMDB	NA	HMDB001	Lipids and
C11 H22 N	-0.00167	-4.8712	NA	level4	KEGG	C04628	NA	Vitamins a
C31 H35 F	0.001535	2.459217	NA	level4	HMDB	NA	HMDB026	NA
C17 H12 C	-0.00069	-2.21286	NA	level4	HMDB	NA	HMDB024	NA
C12 H10 C	0.000199	0.850918	NA	level4	KEGG	C06742	NA	NA
C29 H42 C	0.002463	4.475432	NA	level4	KEGG	C08858	NA	Terpenoid
C14 H14 N	0.000452	1.579823	NA	level4	HMDB	NA	HMDB026	NA
C15 H23 N	-6.28E-05	-0.22352	NA	level4	HMDB	NA	HMDB025	NA
C22 H33 N	-0.0011	-2.47013	NA	level4	HMDB	NA	HMDB025	NA
C19 H24 N	-0.0012	-3.13271	NA	level4	HMDB	NA	HMDB025	NA
C36 H56 C	-0.00268	-4.34771	NA	level4	HMDB	NA	HMDB024	NA
C26 H54 N	0.001004	1.919052	NA	level4	HMDB	NA	HMDB025	NA
C22 H25 N	-7.95E-05	-0.19906	NA	level4	HMDB	NA	HMDB024	NA
C16 H12 N	-0.00126	-4.72318	NA	level4	NA	C12189	NA	NA
C20 H16 C	17.02631	50658.5	22.73828	level4	Public	NA	NA	NA
C25 H38 C	-0.00205	-4.25867	NA	level4	HMDB	NA	HMDB001	Organohe
C12 H22 N	-0.00073	-1.79874	NA	level4	NA	C16070	NA	NA
C10 H11 C	0.000179	0.793553	NA	level4	HMDB	NA	HMDB025	NA
C32 H57 C	-0.00236	-3.24169	NA	level4	NA	NA	NA	NA
C19 H18 C	-35.0379	-112982	22.34453	level4	Public	NA	NA	NA
C11 H10 C	0.000117	0.742286	NA	level4	NA	C14089	NA	Benzenoid
C19 H21 N	0.000796	2.850927	33.74151	level4	Public	C06971	HMDB001	Organohe

C28 H54 N	0.000996	1.819615	NA	level4	HMDB	NA	HMDB001	Lipids and
C50 H89 C	-0.00241	-2.73132	NA	level4	NA	NA	NA	NA
C19 H23 N	-0.00144	-4.08284	NA	level4	HMDB	NA	HMDB025	NA
C4 H6 N2	17.02681	149301.8	86.09531	level1	BMDB	C00429	HMDB000	Organohe
C24 H17 N	-0.00068	-1.78144	NA	level4	HMDB	NA	HMDB024	NA
C20 H14 N	-0.00072	-1.97678	NA	level4	HMDB	NA	HMDB025	NA
C26 H36 C	-0.00256	-4.08928	NA	level4	HMDB	NA	HMDB025	NA
C31 H42 C	0.000138	0.310169	NA	level4	HMDB	NA	HMDB025	NA
C20 H41 N	0.001054	2.275808	NA	level4	HMDB	NA	HMDB025	NA
C23 H32 N	-0.00037	-0.74367	NA	level4	HMDB	NA	HMDB025	NA
C3 H7 N C	0.000137	0.895445	NA	level4	HMDB	NA	HMDB025	NA
C8 H14 O3	7.96E-05	0.503358	NA	level4	HMDB	NA	HMDB000	Organic ox
C13 H14 N	-0.00068	-1.98032	NA	level4	HMDB	NA	HMDB024	NA
C23 H46 N	-0.00014	-0.2848	NA	level4	NA	NA	NA	NA
C34 H69 N	0.000877	1.62575	NA	level4	HMDB	NA	HMDB001	Lipids and
C12 H9 N5	0.000309	1.141031	NA	level4	HMDB	NA	HMDB025	NA
C27 H45 N	7.54E-05	0.168659	NA	level4	HMDB	NA	HMDB025	NA
C18 H24 C	37.95426	124780.8	19.75344	level4	Public	NA	NA	NA
C26 H24 C	-0.0008	-1.91413	NA	level4	NA	NA	NA	NA
C27 H36 C	-0.00102	-1.85469	NA	level4	HMDB	NA	HMDB024	NA
C8 H8 O4	-21.9841	-130825	62.03811	level3	mzCloud	NA	HMDB000	Benzenoid
C11 H20 N	9.20E-06	0.040303	98.02885	level2	mzCloud	NA	NA	NA
C8 H15 N3	-0.0002	-0.67856	NA	level4	HMDB	NA	HMDB000	Organic ac
C32 H41 N	-75.0203	-130400	7.399022	level4	Public	NA	NA	NA
C10 H16 N	-0.00017	-0.68627	73.88846	level2	Public	C06952	HMDB001	Organohe
C28 H31 N	0.000728	1.441481	NA	level4	HMDB	NA	HMDB024	NA
C26 H32 C	21.98083	48180.88	27.22717	level4	Public	NA	NA	NA
C6 H6 O5	0.000181	1.143777	NA	level4	NA	C20901	NA	NA
C2 H3 N S	0.000103	1.41615	NA	level4	NA	C18587	NA	Organosul
H4 O7 P2	8.65E-05	0.48592	NA	level4	HMDB	NA	HMDB000	Homogen
C29 H46 C	-0.00218	-4.92677	NA	level4	HMDB	NA	HMDB025	NA
C27 H28 C	-0.00015	-0.2366	NA	level4	HMDB	NA	HMDB003	Phenylpro
C20 H19 N	17.02642	48216.24	25.1962	level4	Public	NA	HMDB024	NA
C20 H30 C	-0.00265	-8.31716	68.986	level2	mzCloud	NA	NA	NA
C10 H13 N	0.000274	1.092938	NA	level4	HMDB	NA	HMDB025	NA
C13 H12	0.000148	0.882935	NA	level4	HMDB	NA	HMDB025	NA
C14 H17 C	8.43E-06	0.03191	NA	level4	HMDB	NA	HMDB025	NA
C15 H15 C	0.000463	1.117267	NA	level4	NA	C10943	NA	NA
C21 H28 C	0.003954	11.48765	49.86305	level3	mzCloud	NA	NA	NA
C22 H44 N	1.08E-05	0.021803	NA	level4	NA	NA	NA	NA
C15 H14 C	-0.00024	-0.77712	NA	level4	HMDB	NA	HMDB025	NA
C16 H16 N	-0.00131	-4.68217	NA	level4	HMDB	NA	HMDB025	NA
C24 H46 C	-0.00143	-3.20398	NA	level4	NA	NA	NA	NA
C8 H11 N	0.000865	3.710588	NA	level4	HMDB	NA	HMDB025	NA
C16 H19 N	0.001094	3.323801	NA	level4	NA	C18917	NA	NA
C19 H14 C	21.98322	65023.9	37.9568	level4	Public	NA	NA	NA
C56 H72 C	0.003295	3.776937	NA	level4	NA	NA	NA	NA
C34 H49 N	0.00026	0.416527	NA	level4	HMDB	NA	HMDB024	NA
C24 H30 C	18.97253	49638.44	12.86732	level4	Public	NA	NA	NA
C13 H21 N	0.00022	1.15072	12.79738	level4	Public	NA	NA	NA
C14 H22 C	0.000187	0.843092	NA	level4	HMDB	NA	HMDB024	NA
C14 H14 C	8.32E-05	0.361603	NA	level4	HMDB	NA	HMDB000	Benzenoid
C8 H15 N	0.000177	1.256512	NA	level4	HMDB	NA	HMDB024	NA
C39 H70 C	0.001412	2.225865	NA	level4	HMDB	NA	HMDB025	NA
C14 H14 N	0.001412	3.110336	7.579712	level4	Public	NA	HMDB024	NA
C26 H43 N	465.3114	1000005	95.71615	level2	BMDB	C01921	HMDB000	Lipids and

C27 H32 C	0.000496	0.854195	NA	level4	NA	NA	NA	NA
C26 H38 C	0.00017	0.428017	NA	level4	HMDB	NA	HMDB025	NA
C30 H32 C	-18.0089	-32613.1	7.621265	level4	Public	NA	NA	NA
C11 H19 N	-0.00259	-7.95221	32.82637	level3	mzCloud	NA	HMDB000	Organic o:
C11 H17 N	0.000137	0.649295	32.57625	level4	Public	C06546	HMDB025	NA
C8 H17 N	-8.45E-05	-0.40781	NA	level4	HMDB	NA	HMDB001	Organohe
C11 H12 C	-0.00174	-9.04912	30.43351	level3	mzCloud	C10480	HMDB003	Organohe
C20 H32 C	2.97E-05	0.08843	NA	level4	HMDB	NA	HMDB025	NA
C12 H11 N	0.000263	1.150079	NA	level4	HMDB	NA	HMDB025	NA
C16 H27 N	0.000132	0.468376	12.94224	level4	Public	NA	NA	NA
C14 H26 N	-0.00086	-2.58706	NA	level4	HMDB	NA	HMDB025	NA
C36 H56 C	0.001079	1.624547	42.67145	level3	Public	NA	HMDB003	Lipids and
C14 H24 C	7.02E-05	0.219272	NA	level4	HMDB	NA	HMDB000	Organic o:
C19 H20 C	-0.00035	-1.19183	NA	level4	NA	C10285	NA	Skimate /
C14 H22 N	-0.0002	-0.52315	NA	level4	HMDB	NA	HMDB025	NA
C16 H20 C	-0.00027	-0.88551	10.26264	level4	Public	NA	NA	NA
C22 H22 C	-0.00073	-2.08474	NA	level4	HMDB	NA	HMDB025	NA
C16 H17 B	5.04E-05	0.157431	NA	level4	NA	NA	NA	NA
C24 H42 C	-1.58E-05	-0.03229	NA	level4	HMDB	NA	HMDB006	Lipids and
C7 H8 O2	-18.0104	-145184	88.4108	level2	BMDB	C06730	HMDB000	Benzenoid
C14 H19 N	-1.94E-05	-0.05209	NA	level4	KEGG	C03112	NA	NA
C10 H6 O:	0.000179	1.134037	NA	level4	HMDB	NA	HMDB024	NA
C7 H10 O	0.000147	1.339227	91.49571	level2	mzCloud	NA	NA	NA
C18 H38 N	0.000159	0.418718	NA	level4	HMDB	NA	HMDB024	NA
C9 H15 N	0.000313	1.557873	NA	level4	NA	C20940	NA	NA
C5 H12 N:	0.000146	1.259446	NA	level4	KEGG	C00990	NA	Organic a
C26 H38 C	0.000196	0.47269	NA	level4	HMDB	NA	HMDB025	NA
C6 H8 O6	2.42E-05	0.137554	78.20688	level2	mzCloud	C02670	HMDB000	Organohe
C25 H49 N	0.000294	0.71419	NA	level4	HMDB	NA	HMDB025	NA
C30 H59 C	0.002936	4.570795	NA	level4	NA	NA	NA	NA
C19 H25 C	0.001624	4.228414	9.476174	level4	Public	NA	NA	NA
C24 H28 C	-0.00096	-2.07642	2.386554	level4	Public	NA	NA	NA
C29 H54 C	-0.00137	-2.08263	NA	level4	HMDB	NA	HMDB025	NA
C14 H14 C	8.74E-05	0.355261	NA	level4	HMDB	NA	HMDB025	NA
C24 H30 C	17.0267	36839.27	2.584934	level4	Public	NA	NA	NA
C22 H29 N	0.000877	2.277522	NA	level4	KEGG	C09152	NA	NA
C24 H27 N	-5.85E-05	-0.16196	2.200992	level4	Public	NA	HMDB025	NA
C20 H21 N	0.002892	8.526578	40.63883	level3	mzCloud	C06533	HMDB001	Organohe
C19 H20 N	-0.00015	-0.46985	NA	level4	HMDB	NA	HMDB024	NA
C18 H22 C	-0.00099	-2.95871	NA	level4	HMDB	NA	HMDB024	NA
C23 H24 C	-0.00017	-0.41542	NA	level4	HMDB	NA	HMDB025	NA
C20 H30 C	37.95394	113561.6	8.448384	level4	Public	NA	NA	NA
C22 H36 C	-0.00043	-1.12595	32.97876	level4	Public	NA	NA	NA
C27 H40 C	0.00014	0.293519	NA	level4	NA	NA	NA	NA
C23 H42 C	-0.00125	-3.01834	NA	level4	NA	NA	NA	NA
C9 H6 O2	0.000191	1.304686	NA	level4	HMDB	NA	HMDB025	NA
C17 H25 C	0.000692	2.034273	NA	level4	HMDB	NA	HMDB024	NA
C34 H34 N	0.002634	4.555924	NA	level4	NA	C18063	NA	NA
C47 H74 C	-0.00157	-2.3459	NA	level4	HMDB	NA	HMDB000	Lipids and
C20 H30 N	-0.00206	-4.11108	NA	level4	HMDB	NA	HMDB024	NA
C25 H42 N	-0.00015	-0.22692	NA	level4	HMDB	NA	HMDB024	NA
C26 H52 N	-0.00087	-1.56949	NA	level4	NA	NA	NA	NA
C36 H35 N	-0.00193	-3.36338	NA	level4	HMDB	NA	HMDB025	NA
C39 H75 N	0.000859	1.418089	NA	level4	NA	NA	NA	NA
C7 H10 N:	0.000226	1.330454	NA	level4	NA	C19482	NA	NA
C23 H35 B	-0.0017	-4.02056	NA	level4	NA	NA	NA	NA
C48 H94 N	-0.00467	-4.64857	NA	level4	NA	NA	NA	NA
C45 H54 F	-0.00395	-4.8339	NA	level4	HMDB	NA	HMDB025	NA

C21 H36 C	-3.06E-05	-0.09102	NA	level4	HMDB	NA	HMDB025	NA
C23 H32 C	-6.86E-05	-0.17659	NA	level4	HMDB	NA	HMDB024	NA
C12 H14 C	0.0001	0.451529	84.30141	level2	mzCloud	NA	HMDB001	Benzenoid
C13 H11 N	0.000137	0.609411	NA	level4	HMDB	NA	HMDB024	NA
C21 H26 N	6.13E-05	0.146682	NA	level4	HMDB	NA	HMDB001	Organohe
C22 H17 F	-0.00076	-1.77045	NA	level4	NA	C11742	NA	NA
C15 H21 N	0.000164	0.527695	20.84327	level4	Public	NA	NA	NA
C10 H14 N	-0.00149	-4.7323	NA	level4	HMDB	NA	HMDB025	NA
C10 H11 N	-9.50E-05	-0.42207	NA	level4	NA	C11355	NA	NA
C16 H22 C	-8.01E-05	-0.28806	NA	level4	HMDB	NA	HMDB025	NA
C11 H12 C	0.000317	1.649359	NA	level4	HMDB	NA	HMDB025	NA
C26 H29 N	0.000371	0.773561	NA	level4	HMDB	NA	HMDB001	Organohe
C42 H70 N	-0.00152	-1.94752	NA	level4	NA	NA	NA	NA
C13 H12 N	5.99E-05	0.262552	NA	level4	HMDB	NA	HMDB024	NA
C36 H66 C	0.000199	0.343533	NA	level4	NA	NA	NA	NA
C25 H36 C	-0.00024	-0.46758	NA	level4	NA	NA	NA	NA
C20 H32 C	-18.0108	-51134.3	43.3624	level3	mzCloud	NA	NA	NA
C22 H28 F	0.0008	1.662427	87.83261	level2	mzCloud	D01915	HMDB001	Organohe
C45 H60 C	-0.00026	-0.28141	NA	level4	NA	C19724	NA	NA
C18 H14 C	0.001378	4.016562	NA	level4	HMDB	NA	HMDB006	Organohe
C14 H26 C	2.70E-05	0.10443	NA	level4	HMDB	NA	HMDB025	NA
C25 H45 C	0.001664	3.778836	NA	level4	NA	NA	NA	NA
C23 H24 N	-0.00025	-0.638	NA	level4	HMDB	NA	HMDB001	Organohe
C30 H56 N	-0.0014	-1.79748	NA	level4	NA	C16563	NA	NA
C17 H14 C	17.02655	51584.05	4.467307	level4	Public	NA	NA	NA
C11 H7 N3	-0.00099	-4.0558	NA	level4	HMDB	NA	HMDB024	NA
C19 H20 C	0.00142	4.314633	NA	level4	HMDB	NA	HMDB025	NA
C22 H28 C	-0.00181	-4.42138	NA	level4	HMDB	NA	HMDB001	Lipids and
C20 H35 N	9.49E-05	0.281508	NA	level4	NA	NA	NA	NA
C10 H11 I2	0.000428	0.957046	NA	level4	HMDB	NA	HMDB025	NA
C6 H10 O7	21.98205	113284.6	49.66095	level3	Decode	C00191	HMDB000	Organic ox
C8 H13 N	0.000268	1.725602	NA	level4	HMDB	NA	HMDB025	NA
C43 H80 N	0.003245	4.048413	NA	level4	NA	NA	NA	NA
C18 H21 F	0.00097	2.578367	NA	level4	HMDB	NA	HMDB024	NA
C6 H11 N5	0.000226	1.474799	NA	level4	KEGG	C07454	NA	NA
C11 H18 N	0.00018	0.697928	NA	level4	HMDB	NA	HMDB025	NA
C13 H15 N	0.000656	2.675671	NA	level4	HMDB	NA	HMDB006	Benzenoid
C29 H24 C	0.000166	0.32229	NA	level4	NA	NA	NA	NA
C11 H8 F N	0.001031	4.502198	NA	level4	HMDB	NA	HMDB024	NA
C25 H52 N	0.000509	0.998815	NA	level4	HMDB	NA	HMDB001	Lipids and
C37 H61 N	0.001528	2.1012	NA	level4	NA	C15680	NA	NA
C12 H13 N	-0.00151	-4.85573	NA	level4	HMDB	NA	HMDB024	NA
C7 H13 N	0.000172	1.352132	NA	level4	HMDB	NA	HMDB025	NA
C26 H45 N	0.000841	1.631181	93.46602	level1	BMDB2	C05122	HMDB000	Lipids and
C13 H18 C	-4.68E-05	-0.21085	NA	level4	HMDB	NA	HMDB006	Phenylpro
C11 H13 N	0.001131	5.566867	1.24318	level3	BMDB	C00977	HMDB001	Organohe
C23 H28 C	-0.00019	-0.47474	NA	level4	HMDB	NA	HMDB025	NA
C9 H16 N2	0.000265	1.438511	95.01813	level1	Decode	NA	HMDB006	Organohe
C24 H38 C	0.000257	0.608668	NA	level4	HMDB	NA	HMDB025	NA
C4 H6 Cl F	0.000177	1.090402	NA	level4	NA	C14700	NA	NA
C19 H34 C	-0.00081	-2.0645	18.03836	level4	Public	NA	NA	NA
C3 H5 N3	0.000172	1.731579	NA	level4	HMDB	NA	HMDB024	NA
C7 H8 N2	5.69E-05	0.374513	NA	level4	HMDB	NA	HMDB025	NA
C21 H20 C	-8.66E-05	-0.19331	NA	level4	HMDB	NA	HMDB024	Phenylpro
C27 H30 C	-0.00042	-0.67774	NA	level4	HMDB	NA	HMDB003	Phenylpro
C7 H11 N3	0.000244	1.593727	NA	level4	NA	C16399	NA	NA

C7 H15 O:	-0.00014	-0.47886	26.68596	level4	Public	C05382	HMDB025	NA
C26 H35 N	0.000611	1.436238	NA	level4	HMDB	NA	HMDB025	NA
C25 H48 N	-0.0004	-0.74797	NA	level4	NA	NA	NA	NA
C19 H20 N	-0.00022	-0.60435	NA	level4	HMDB	NA	HMDB025	NA
C13 H25 N	-0.00012	-0.47544	98.53992	level2	mzCloud	NA	HMDB000	NA
C17 H26 C	0.001397	3.44045	9.63378	level4	Public	NA	NA	NA
C17 H19 N	-0.00021	-0.57604	93.33292	level2	mzCloud	NA	HMDB001	Organohe
C14 H15 N	-0.00065	-2.24125	NA	level4	HMDB	NA	HMDB025	NA
C28 H31 N	0.00209	3.861895	NA	level4	HMDB	NA	HMDB024	NA
C10 H11 C	-0.00094	-3.09425	NA	level4	HMDB	NA	HMDB001	Nucleosid
C45 H78 N	1.57E-05	0.019779	NA	level4	HMDB	NA	HMDB000	Lipids and
C23 H42 N	-0.00116	-2.27924	NA	level4	NA	NA	NA	NA
C23 H28 N	-0.00116	-2.72463	NA	level4	HMDB	NA	HMDB025	NA
C5 H9 N C	3.78E-05	0.231698	89.69268	level2	BMDB	NA	HMDB000	Organic ac
C28 H24 N	-0.00198	-4.42135	NA	level4	HMDB	NA	HMDB025	NA
C23 H42 C	18.97532	38390.39	5.813219	level4	Public	NA	NA	NA
C45 H82 N	0.002204	2.66287	NA	level4	NA	NA	NA	NA
C16 H20 N	5.75E-06	0.018894	NA	level4	HMDB	NA	HMDB024	NA
C3 H7 N C	0.000184	1.517713	88.64781	level2	mzCloud	C00793	HMDB000	Organic ac
C17 H17 C	0.000678	2.222674	NA	level4	HMDB	NA	HMDB025	NA
C30 H42 C	-0.00089	-1.7379	NA	level4	HMDB	NA	HMDB025	NA
C31 H41 N	-0.00174	-3.42824	NA	level4	NA	NA	NA	NA
C29 H45 N	0.000334	0.734514	NA	level4	NA	NA	NA	NA
C17 H16 C	-0.00082	-2.44831	NA	level4	HMDB	NA	HMDB001	Organic ox
C18 H21 C	0.00195	4.92219	NA	level4	HMDB	NA	HMDB025	NA
C23 H38 N	-7.15E-05	-0.14947	NA	level4	HMDB	NA	HMDB025	NA
C21 H32 N	0.000344	0.826355	NA	level4	HMDB	NA	HMDB001	Benzenoid
C19 H21 N	-0.00023	-0.67589	NA	level4	HMDB	NA	HMDB025	NA
C31 H41 C	0.000119	0.196506	NA	level4	NA	C20137	NA	NA
C10 H22 C	3.89E-05	0.163359	NA	level4	HMDB	NA	HMDB025	NA
C20 H30 C	-0.00051	-1.51429	33.39508	level4	Public	NA	NA	NA
C7 H6 N2	0.000298	2.225759	NA	level4	HMDB	NA	HMDB024	NA
C27 H45 N	0.000474	0.956415	NA	level4	HMDB	NA	HMDB025	NA
C9 H14 O:	0.000103	0.605357	NA	level4	NA	NA	NA	NA
C5 H10	8.72E-05	1.24497	NA	level4	HMDB	NA	HMDB024	NA
C25 H44 N	-0.00104	-1.95167	NA	level4	NA	NA	NA	NA
C24 H32 N	-0.00134	-3.29275	NA	level4	NA	C10767	NA	Alkaloids
C20 H17 F	-0.00185	-4.38952	NA	level4	HMDB	NA	HMDB025	NA
C23 H24 N	-0.00121	-2.7714	NA	level4	HMDB	NA	HMDB025	NA
C14 H21 N	0.00138	3.640954	NA	level4	KEGG	C04794	NA	Organic ox
C15 H19 N	0.000941	3.446575	NA	level4	HMDB	NA	HMDB006	Organohe
C22 H22 N	0.000946	1.732194	NA	level4	HMDB	NA	HMDB024	NA
C3 H5 N C	0.000146	2.050369	NA	level4	HMDB	NA	HMDB024	NA
C44 H30 N	0.002234	3.637176	NA	level4	HMDB	NA	HMDB024	NA
C24 H45 N	0.000264	0.640804	NA	level4	NA	NA	NA	NA
C45 H86 N	-0.00142	-1.60978	NA	level4	NA	NA	NA	NA
C29 H30 N	0.000844	1.544462	NA	level4	HMDB	NA	HMDB025	NA
C50 H87 C	0.000816	0.880307	NA	level4	NA	NA	NA	NA
C19 H12 C	-0.00081	-2.64873	NA	level4	HMDB	NA	HMDB025	NA
C10 H16 N	-0.00019	-0.69355	NA	level4	HMDB	NA	HMDB025	NA
C31 H57 N	-0.00236	-3.66937	NA	level4	HMDB	NA	HMDB024	NA
C5 H11 N	6.23E-05	0.34426	NA	level4	HMDB	NA	HMDB025	NA
C16 H18 N	-0.00077	-3.01241	NA	level4	HMDB	NA	HMDB025	NA
C21 H32 C	-0.00015	-0.46145	81.97883	level2	mzCloud	C03681	HMDB000	Lipids and
C10 H16 N	0.000346	1.763606	NA	level4	HMDB	NA	HMDB024	Organic ac

C5 H13 N	5.20E-05	0.504654	89.33635	level2	mzCloud	C00114	HMDB000	Organic ni
C24 H36 (	-0.00011	-0.31907	NA	level4	HMDB	NA	HMDB025	NA
C20 H32 (	37.95411	107755.3	11.40132	level4	Public	NA	NA	NA
C11 H12 (	4.04E-05	0.18037	NA	level4	HMDB	NA	HMDB024	NA
C18 H30 N	-0.00162	-4.19248	NA	level4	HMDB	NA	HMDB025	NA
C36 H54 (	0.000134	0.207767	NA	level4	HMDB	NA	HMDB003	Lipids and
C22 H36 N	0.000369	0.837756	NA	level4	HMDB	NA	HMDB001	Organic ac
C23 H28 (	17.02812	40914.94	8.926693	level4	Public	NA	NA	NA
C25 H34 (	0.000449	0.97098	38.48742	level4	Public	NA	NA	NA
C17 H12 F	-0.00055	-1.49842	NA	level4	HMDB	NA	HMDB025	NA
C8 H8	0.000104	1.003794	NA	level4	HMDB	NA	HMDB003	Benzenoid
C12 H12	0.000157	1.008963	NA	level4	HMDB	NA	HMDB024	NA
C7 H6 O4	6.19E-05	0.401681	93.39914	level2	mzCloud	C00628	HMDB000	Benzenoid
C44 H80 N	-0.00255	-2.91189	NA	level4	NA	C11985	NA	PK Polyke
C16 H21 N	-7.09E-05	-0.20904	NA	level4	HMDB	NA	HMDB001	Organic ox
C37 H44 (	-6.74E-05	-0.09674	NA	level4	NA	C12406	NA	NA
C20 H32 (	4.17E-05	0.130127	56.95588	level3	mzCloud	C14776	HMDB000	Lipids and
C9 H11 N5	-0.00047	-1.97051	31.6448	level3	mzCloud	C00835	HMDB000	Organohe
C6 H7 N (	5.92E-05	0.542798	NA	level4	HMDB	NA	HMDB000	Benzenoid
C10 H8 O5	1.42E-05	0.068456	NA	level4	NA	C20772	NA	NA
C35 H48 N	-0.00023	-0.33258	NA	level4	HMDB	NA	HMDB026	NA
C19 H26 (	-0.00118	-2.73418	2.847817	level4	Public	NA	NA	NA
C18 H19 N	-0.00159	-4.96473	NA	level4	HMDB	NA	HMDB025	NA
C16 H20 N	4.66E-05	0.182093	NA	level4	HMDB	NA	HMDB025	NA
C14 H24 N	-0.00011	-0.39849	NA	level4	HMDB	NA	HMDB024	NA
C22 H26 N	0.001996	4.818253	NA	level4	HMDB	NA	HMDB024	NA
C18 H32 (	-0.00012	-0.38971	NA	level4	HMDB	NA	HMDB000	Lipids and
C10 H20 (	0.000146	0.714966	NA	level4	HMDB	NA	HMDB024	NA
C15 H14 (	0.000276	1.221208	NA	level4	HMDB	NA	HMDB025	NA
C12 H17 N	0.000101	0.421792	NA	level4	HMDB	NA	HMDB025	NA
C11 H10	0.000155	1.08744	NA	level4	NA	C14098	NA	NA
C15 H12 (	8.35E-05	0.326049	NA	level4	HMDB	NA	HMDB025	NA
C10 H14 N	2.88E-05	0.111428	NA	level4	HMDB	NA	HMDB024	NA
C32 H58 N	-0.0029	-4.72741	NA	level4	HMDB	NA	HMDB024	NA
C15 H10 (	0.003393	11.23313	37.43398	level3	mzCloud	C00389	HMDB000	Phenylpro
C18 H19 N	-8.77E-05	-0.25716	NA	level4	HMDB	NA	HMDB026	NA
C11 H15 N	0.000203	1.052484	41.88069	level3	Public	NA	NA	NA
C20 H25 (	0.001634	4.003636	NA	level4	HMDB	NA	HMDB000	Organohe
C24 H40 (	46.00498	112677.9	78.77353	level2	mzCloud	C17726	HMDB000	Lipids and
C18 H32 (	-0.00093	-1.83801	NA	level4	HMDB	NA	HMDB025	NA
C13 H16 N	-0.00117	-4.51565	NA	level4	HMDB	NA	HMDB025	NA
C27 H43 N	0.000341	0.765208	NA	level4	HMDB	NA	HMDB026	NA
C19 H37 N	-7.50E-05	-0.2185	NA	level4	NA	NA	NA	NA
C27 H44 (	-0.00028	-0.58177	NA	level4	HMDB	NA	HMDB024	NA

C9 H19 N	0.000297	1.448433	25.45765	level4	Public	NA	NA	NA
C9 H13 N	0.000318	1.734841	25.02238	level3	BMDB	C05589	HMDB000	Benzenoid
C29 H31 N	0.00098	1.970235	NA	level4	HMDB	NA	HMDB024	NA
C11 H16 C	0.00027	1.376752	28.87528	level4	Public	NA	NA	NA
C22 H32 C	0.001529	3.239101	2.820652	level4	Public	NA	NA	NA
C25 H34 C	21.98409	51097.79	66.42137	level2	mzCloud	D09393	NA	NA
C21 H26 C	21.98025	58743.55	6.032598	level4	Public	NA	HMDB003	Lignans, n
C38 H47 N	0.000954	1.273864	NA	level4	HMDB	NA	HMDB025	NA
C10 H8 O2	7.46E-05	0.466372	NA	level4	HMDB	NA	HMDB024	NA
C30 H30 N	0.001916	4.581146	NA	level4	NA	C15021	NA	NA
C20 H30 C	0.000116	0.346567	NA	level4	HMDB	NA	HMDB025	NA
C28 H54 C	-0.00219	-4.35083	NA	level4	NA	NA	NA	NA
C15 H16 N	-0.00097	-3.419	NA	level4	HMDB	NA	HMDB025	NA
C35 H66 N	-0.00013	-0.181	NA	level4	NA	NA	NA	NA
C30 H34 C	-0.00058	-1.26263	NA	level4	HMDB	NA	HMDB024	NA
C29 H51 N	0.000461	0.827522	NA	level4	NA	NA	NA	NA
C10 H8 O3	-3.79E-05	-0.18215	67.85607	level2	Public	C16202	HMDB025	NA
C13 H20 N	5.64E-05	0.210305	NA	level4	HMDB	NA	HMDB024	NA
C12 H21 N	-7.93E-05	-0.27618	NA	level4	NA	C11329	NA	NA
C14 H19 N	0.000126	0.383573	NA	level4	HMDB	NA	HMDB024	NA
C23 H33 N	-0.00019	-0.48586	NA	level4	NA	NA	NA	NA
C38 H42 C	-0.00277	-3.83437	NA	level4	KEGG	C09122	NA	Terpenoid
C49 H74 C	0.003482	3.928304	NA	level4	HMDB	NA	HMDB024	NA
C20 H32 C	0.000105	0.363845	NA	level4	HMDB	NA	HMDB026	NA
C21 H24 C	5.94E-05	0.153069	NA	level4	HMDB	NA	HMDB025	NA
C19 H21 N	-0.0015	-3.79891	NA	level4	HMDB	NA	HMDB024	NA
C12 H8	0.000128	0.83912	NA	level4	HMDB	NA	HMDB024	NA
C7 H16 N2	8.17E-05	0.425066	NA	level4	NA	C21888	NA	NA
C5 H14 N	21.98215	120077.7	66.27135	level1	BMDB	C00588	HMDB000	Organic ni
C27 H32 C	0.001621	2.648526	NA	level4	NA	NA	NA	NA
C28 H44 C	-0.00021	-0.41043	NA	level4	NA	NA	NA	NA
C25 H40 C	-0.00029	-0.59113	NA	level4	NA	NA	NA	NA
C9 H14 O2	0.000178	1.156473	NA	level4	HMDB	NA	HMDB024	NA
C31 H52 N	0.003038	4.485937	NA	level4	NA	NA	NA	NA
C20 H20 C	-0.00054	-1.52722	NA	level4	HMDB	NA	HMDB025	NA
C15 H24 C	17.02611	63490.57	21.59562	level4	Public	NA	NA	NA
C5 H4 N4	0.000304	1.652843	NA	level4	HMDB	NA	HMDB024	NA
C11 H16 N	0.001281	4.430935	NA	level4	HMDB	NA	HMDB025	NA
C21 H24 F	0.0012	2.990228	NA	level4	HMDB	NA	HMDB025	NA
C7 H11 N	0.008801	56.02991	61.90061	level1	Decode	NA	HMDB000	Organic ac
C46 H82 N	-0.00152	-1.88745	NA	level4	HMDB	NA	HMDB000	Lipids and
C21 H25 C	0.000666	1.556148	NA	level4	HMDB	NA	HMDB024	NA
C16 H22 C	-0.00031	-0.94548	NA	level4	NA	NA	NA	NA
C21 H25 C	0.000212	0.523463	42.47197	level3	mzCloud	NA	NA	NA
C21 H36 C	-6.56E-05	-0.17804	54.18081	level3	Public	NA	NA	NA
C46 H71 C	0.001543	1.894989	NA	level4	NA	NA	NA	NA
C24 H31 N	0.00073	1.857025	NA	level4	NA	C18545	NA	NA
C19 H18 N	0.000299	0.636599	NA	level4	NA	C19480	NA	NA
C47 H80 C	-0.00145	-1.58599	NA	level4	HMDB	NA	HMDB025	NA
C15 H21 N	-0.0001	-0.30787	NA	level4	HMDB	NA	HMDB025	NA
C44 H54 N	-0.00022	-0.26842	NA	level4	HMDB	NA	HMDB025	NA
C15 H22 C	0.000188	0.803062	NA	level4	HMDB	NA	HMDB024	NA
C23 H43 N	-5.88E-05	-0.14809	NA	level4	HMDB	NA	HMDB000	Lipids and
C31 H37 N	-0.00049	-0.92669	NA	level4	HMDB	NA	HMDB025	NA
C9 H10 O5	-18.0104	-90937.1	50.51932	level3	Decode	C05584	HMDB000	Benzenoid

C12 H17 N	0.000284	1.131149	NA	level4	HMDB	NA	HMDB024	NA
C25 H20 C	0.000979	2.110288	NA	level4	HMDB	NA	HMDB025	NA
C29 H34 C	-0.00104	-1.54954	NA	level4	NA	NA	NA	NA
C27 H40 C	0.000831	1.688893	NA	level4	HMDB	NA	HMDB024	NA
C20 H25 F	-0.00232	-4.17086	NA	level4	HMDB	NA	HMDB024	NA
C25 H28 C	-0.00093	-2.37342	NA	level4	HMDB	NA	HMDB024	NA
C24 H32 C	-0.00026	-0.48528	NA	level4	NA	C11647	NA	NA
C19 H26 F	-0.00117	-2.92214	NA	level4	HMDB	NA	HMDB025	NA
C9 H8 O4	0.000222	1.231858	NA	level4	HMDB	NA	HMDB003	Phenylpro
C22 H24 C	-2.30E-05	-0.04784	NA	level4	HMDB	NA	HMDB025	NA
C9 H13 N3	0.001506	6.195997	46.66517	level3	mzCloud	C00475	HMDB000	Nucleosid
C20 H28 N	-0.00145	-3.7266	NA	level4	HMDB	NA	HMDB025	NA
C27 H34 C	21.98073	45206.89	9.933324	level4	Public	NA	NA	NA
C21 H39 N	-8.21E-05	-0.19687	NA	level4	NA	NA	NA	NA
C28 H40 C	-0.00027	-0.53713	NA	level4	HMDB	NA	HMDB024	NA
C13 H20 C	0.00029	1.50785	NA	level4	HMDB	NA	HMDB024	NA
C18 H17 N	0.001735	4.674208	NA	level4	HMDB	NA	HMDB024	NA
C16 H26 C	21.98027	82574.17	37.38795	level3	mzCloud	NA	HMDB006	Lipids and
C22 H32 C	-0.00027	-0.62064	NA	level4	NA	C11899	NA	NA
C27 H38 C	0.000332	0.699198	NA	level4	KEGG	C09086	NA	Terpenoid
C27 H42 C	0.000625	1.225138	NA	level4	NA	NA	NA	NA
C13 H12 C	0.000291	1.348436	43.95801	level4	Public	NA	NA	NA
C24 H39 N	-0.00015	-0.32534	NA	level4	HMDB	NA	HMDB025	NA
C26 H39 N	0.000383	0.802989	NA	level4	HMDB	NA	HMDB025	NA
C20 H31 N	-0.0012	-2.83893	NA	level4	HMDB	NA	HMDB025	NA
C29 H31 F	-9.37E-05	-0.19707	NA	level4	HMDB	NA	HMDB001	Benzenoid
C42 H68 C	-0.00037	-0.45156	NA	level4	NA	NA	NA	NA
C20 H38 C	-0.00015	-0.45767	NA	level4	NA	NA	NA	NA
C15 H21 N	7.24E-05	0.213519	NA	level4	HMDB	NA	HMDB001	Benzenoid
C19 H21 F	0.001397	4.206023	NA	level4	NA	C15387	NA	NA
C21 H27 N	0.000189	0.410855	NA	level4	HMDB	NA	HMDB006	Benzenoid
C16 H28	0.000322	1.463811	NA	level4	NA	NA	NA	NA
C15 H25 N	4.30E-05	0.160843	NA	level4	HMDB	NA	HMDB000	Benzenoid
C26 H26 C	-0.00085	-1.56101	NA	level4	NA	NA	NA	NA
C21 H36 C	17.02648	44310.81	29.8082	level4	Public	NA	NA	NA
C9 H9 N C	0.000261	1.457289	NA	level4	HMDB	NA	HMDB025	NA
C15 H15 F	0.000953	2.853846	NA	level4	HMDB	NA	HMDB025	NA
C15 H10 C	7.15E-05	0.195255	NA	level4	HMDB	NA	HMDB024	Phenylpro
C15 H18 N	6.16E-05	0.212232	NA	level4	HMDB	NA	HMDB025	NA
C22 H24 N	-0.0012	-2.676	NA	level4	HMDB	NA	HMDB025	NA
C36 H73 N	0.000399	0.703626	NA	level4	HMDB	NA	HMDB001	Lipids and
C6 H5 N C	7.85E-05	0.564428	66.59357	level2	mzCloud	C01020	HMDB000	Organohe
C20 H24 C	1.88E-05	0.046053	62.34767	level2	Public	NA	NA	NA
C17 H26 N	-0.00111	-3.66064	NA	level4	HMDB	NA	HMDB001	Organohe
C21 H19 N	-0.00157	-3.98443	NA	level4	HMDB	NA	HMDB001	Organohe
C18 H16 C	17.02855	49488.72	0.375704	level4	Public	NA	NA	NA
C32 H63 N	0.000723	1.418592	NA	level4	HMDB	NA	HMDB001	Lipids and
C30 H25 F	0.002277	3.573104	NA	level4	HMDB	NA	HMDB024	NA
C22 H21 F	-0.00178	-4.82334	NA	level4	HMDB	NA	HMDB024	NA
C13 H21 N	0.000154	0.406121	NA	level4	HMDB	NA	HMDB025	NA
C18 H8 O8	-0.00225	-6.40266	47.12766	level3	Public	NA	HMDB003	Organohe
C17 H17 C	-5.67E-05	-0.18161	38.07479	level4	Public	NA	NA	NA
C16 H22 N	-0.00117	-2.97829	NA	level4	HMDB	NA	HMDB025	NA
C17 H31 N	-0.00054	-1.57588	NA	level4	HMDB	NA	HMDB024	NA
C21 H21 F	-0.00157	-3.89132	NA	level4	HMDB	NA	HMDB025	NA
C36 H71 N	0.00049	0.866891	NA	level4	HMDB	NA	HMDB001	Lipids and
C5 H11 N	9.29E-05	1.092285	67.18785	level2	Public	C01746	HMDB003	Organohe

C15 H14 S	0.000408	1.805245	NA	level4	HMDB	NA	HMDB024	NA
C33 H63 N	-0.00049	-0.88549	NA	level4	NA	NA	NA	NA
C26 H44 C	0.002158	3.350033	NA	level4	NA	NA	NA	NA
C17 H28 N	-0.00112	-3.50843	NA	level4	HMDB	NA	HMDB025	NA
C22 H37 N	-0.00014	-0.35029	NA	level4	HMDB	NA	HMDB001	Lipids and
C12 H12 C	-3.75E-06	-0.01335	NA	level4	HMDB	NA	HMDB024	NA
C13 H23 N	-2.68E-05	-0.08903	NA	level4	KEGG	C02487	NA	NA
C19 H20 C	7.39E-05	0.225153	40.61843	level3	Public	NA	NA	NA
C7 H6 O3	0.000306	2.213354	NA	level4	HMDB	NA	HMDB000	Benzenoid
C20 H30 C	21.98204	72734.13	9.20235	level4	Public	NA	NA	NA
C14 H21 N	8.14E-05	0.291426	NA	level4	HMDB	NA	HMDB001	Organohe
C6 H5 N C	8.08E-05	0.580969	76.2349	level2	mzCloud	C18620	HMDB001	Organohe
C12 H18 C	-0.00112	-4.07961	NA	level4	HMDB	NA	HMDB025	NA
C11 H13 N	4.06E-05	0.196265	52.08071	level4	Public	NA	HMDB025	NA
C28 H48 N	0.002131	3.717485	NA	level4	NA	NA	NA	NA
C10 H14 C	3.94E-05	0.237466	88.11513	level1	Decode	C11924	HMDB000	Lipids and
C27 H48 N	21.98111	37616.13	14.06801	level4	Public	NA	NA	NA
C19 H18 F	-0.00103	-2.8842	NA	level4	HMDB	NA	HMDB024	NA
C35 H44 C	0.001459	2.279375	NA	level4	NA	NA	NA	NA
C43 H65 N	0.001001	1.186717	NA	level4	NA	C15702	NA	NA
C28 H30 N	-3.59E-05	-0.06933	NA	level4	HMDB	NA	HMDB006	Alkaloids a
C6 H8 O6	0.000164	0.932739	NA	level4	HMDB	NA	HMDB026	NA
C46 H80 N	-0.00039	-0.48666	NA	level4	HMDB	NA	HMDB000	Lipids and
C15 H10 C	-0.0013	-3.88638	NA	level4	NA	NA	NA	NA
C23 H40 C	-7.94E-05	-0.20876	NA	level4	NA	NA	NA	NA
C7 H6 O4	35.03738	227476.1	11.87679	level4	Public	C16748	HMDB003	Organohe
C11 H17 N	4.70E-05	0.173432	NA	level4	HMDB	NA	HMDB024	NA
C12 H18 C	-0.00162	-4.77906	NA	level4	HMDB	NA	HMDB025	NA
C17 H20 F	-1.83E-05	-0.05493	NA	level4	HMDB	NA	HMDB001	Organohe
C25 H36 C	0.000228	0.491772	NA	level4	HMDB	NA	HMDB025	NA
C16 H27 N	-0.00014	-0.48652	NA	level4	NA	NA	NA	NA
C17 H16 C	-0.00017	-0.51858	NA	level4	HMDB	NA	HMDB006	Organohe
C47 H71 N	0.004035	4.7951	NA	level4	NA	C16907	NA	Marine bic
C46 H84 N	-6.51E-05	-0.08042	NA	level4	HMDB	NA	HMDB000	Lipids and
C12 H16 C	0.000195	1.013366	NA	level4	HMDB	NA	HMDB025	NA
C18 H18 N	-0.0002	-0.61289	NA	level4	HMDB	NA	HMDB025	NA
C24 H32 C	0.000241	0.557053	14.17582	level4	Public	NA	NA	NA
C30 H34 C	0.001848	3.770088	NA	level4	NA	NA	NA	NA
C15 H26 C	0.00011	0.460857	27.41473	level4	Public	NA	NA	NA
C10 H14 B	-0.00029	-0.86143	NA	level4	NA	C17103	NA	NA
C22 H28 C	0.002052	4.383686	NA	level4	HMDB	NA	HMDB025	NA
C45 H80 N	4.15E-05	0.052286	NA	level4	NA	NA	NA	NA
C17 H17 N	0.000589	1.777408	NA	level4	NA	C10718	NA	Alkaloids
C20 H33 N	-0.00111	-3.30703	NA	level4	NA	NA	NA	NA
C14 H13 N	-6.21E-05	-0.21349	NA	level4	KEGG	C08531	NA	NA
C20 H22 C	8.78E-05	0.23463	47.63688	level4	Public	NA	NA	NA
C9 H9 N C	0.000142	0.965924	NA	level4	HMDB	NA	HMDB024	NA
C17 H16 C	0.001054	3.696716	NA	level4	HMDB	NA	HMDB024	NA
C3 H5 Cl C	7.80E-05	0.629182	NA	level4	HMDB	NA	HMDB024	NA
C11 H14 N	-45.0577	-258780	88.96692	level2	mzCloud	C08304	HMDB003	Organohe
C23 H40 N	-0.00126	-2.66333	NA	level4	NA	NA	NA	NA

C28 H23 N	-0.0016	-3.19739	NA	level4	HMDB	NA	HMDB025	NA
C10 H17 N	0.000432	2.048133	NA	level4	NA	C18432	NA	NA
C21 H24 C	0.000201	0.540232	6.029692	level4	Public	NA	NA	NA
C17 H21 N	-0.00078	-1.85284	NA	level4	HMDB	NA	HMDB024	NA
C24 H34 C	-0.00052	-1.05096	NA	level4	NA	NA	NA	NA
C21 H32 C	0.000147	0.371302	58.17516	level3	BMDB	NA	HMDB000	Lipids and
C8 H11 N	0.00017	0.781074	NA	level4	HMDB	NA	HMDB000	Organic ac
C14 H18 N	1.91E-05	0.072925	NA	level4	HMDB	NA	HMDB001	Organohe
C30 H54 N	-0.00371	-4.76697	NA	level4	NA	C16564	NA	NA
C27 H30 N	0.001419	3.097085	NA	level4	HMDB	NA	HMDB025	NA
C22 H40 C	-9.26E-05	-0.23128	NA	level4	NA	NA	NA	NA
C27 H24 N	0.001703	3.106656	NA	level4	HMDB	NA	HMDB025	NA
C23 H28 C	-0.00045	-1.04545	7.532961	level4	Public	NA	NA	NA
C35 H58 C	0.002118	3.159505	NA	level4	NA	C13299	NA	NA
C23 H25 N	0.001455	3.283791	NA	level4	HMDB	NA	HMDB025	NA
C23 H31 N	9.83E-05	0.255189	NA	level4	HMDB	NA	HMDB025	NA
C26 H37 N	-0.00028	-0.67163	43.44242	level4	Public	NA	NA	NA
C19 H37 C	0.000128	0.327377	NA	level4	NA	NA	NA	NA
C22 H22 N	-0.00015	-0.45303	NA	level4	HMDB	NA	HMDB024	NA
C12 H20 N	-0.00019	-0.72273	NA	level4	HMDB	NA	HMDB025	NA
C35 H58 C	-0.00178	-3.10261	NA	level4	NA	NA	NA	NA
C26 H43 N	9.91E-05	0.237567	NA	level4	HMDB	NA	HMDB025	NA
C23 H38 C	0.000207	0.50489	NA	level4	NA	NA	NA	NA
C8 H8 O3	8.92E-05	0.586966	94.55981	level1	Decode	C00642	nan	NA
C16 H19 F	0.00126	3.727396	NA	level4	HMDB	NA	HMDB025	NA

C19 H26 C	0.003923	12.32997	53.03057	level3	mzCloud	C00399	HMDB000	Lipids and
C20 H12 N	-0.00059	-1.70927	NA	level4	HMDB	NA	HMDB024	NA
C8 H15 N	0.000254	1.79674	65.07496	level2	mzCloud	C00729	NA	Alkaloids
C19 H38 C	-0.0002	-0.60023	NA	level4	HMDB	NA	HMDB001	Lipids and
C42 H68 N	-0.00246	-3.15906	NA	level4	NA	NA	NA	NA
C17 H13 C	0.00121	4.03296	NA	level4	HMDB	NA	HMDB025	NA
C32 H58 N	0.000325	0.501317	NA	level4	NA	C13902	NA	GP Glycer
C14 H22 C	0.000181	0.879388	41.43183	level4	Public	NA	NA	NA
C25 H31 C	0.001907	3.766727	NA	level4	HMDB	NA	HMDB006	Alkaloids a
C11 H15 N	-0.0004	-1.27199	NA	level4	HMDB	NA	HMDB024	NA
C18 H21 N	8.14E-05	0.258269	NA	level4	HMDB	NA	HMDB001	Benzenoid
C8 H10 N2	0.00043	2.007321	NA	level4	NA	C13673	NA	NA
C12 H21 N	-0.00087	-2.87361	NA	level4	NA	C14324	NA	Organic ac
C12 H8 O4	0.001174	5.435505	64.89101	level2	mzCloud	C01864	HMDB001	Phenylpro
C39 H62 C	0.003211	4.168381	NA	level4	NA	C17922	NA	NA
C4 H6 O	0.000177	2.52148	NA	level4	HMDB	NA	HMDB025	NA

C26 H42 N	0.000316	0.69481	83.98889	level1	BMDB2	NA	NA	NA
C21 H30 C	0.000121	0.295686	NA	level4	HMDB	NA	HMDB025	NA
C21 H38 C	-18.0106	-50837.7	92.47763	level2	mzCloud	NA	HMDB001	Lipids and
C43 H72 C	0.002499	3.737804	NA	level4	HMDB	NA	HMDB000	Lipids and
C5 H9 N3	0.000228	1.301035	NA	level4	HMDB	NA	HMDB000	Organic ac
C22 H44 C	0.000218	0.538343	NA	level4	HMDB	NA	HMDB024	NA
C9 H17 N3	0.000245	1.060835	NA	level4	HMDB	NA	HMDB024	NA
C7 H15 N3	0.00029	1.673093	NA	level4	HMDB	NA	HMDB024	NA
C25 H50 C	0.001655	3.579665	NA	level4	NA	NA	NA	NA
C15 H18 N	-0.03643	-125.571	8.851067	level4	Public	NA	NA	NA
C23 H26 F	1.92E-05	0.044344	NA	level4	HMDB	NA	HMDB025	NA
C9 H8 F N	0.000951	4.827697	NA	level4	HMDB	NA	HMDB024	NA
C10 H16 N	0.000694	2.064091	NA	level4	HMDB	NA	HMDB025	NA
C22 H34 C	0.00022	0.539993	NA	level4	HMDB	NA	HMDB024	NA

C15 H24	21.98056	107648.8	2.419069	level4	Public	NA	NA	NA
C17 H31 N	0.000478	1.337138	NA	level4	HMDB	NA	HMDB024	NA
C27 H36 N	-0.001	-2.237	NA	level4	HMDB	NA	HMDB025	NA
C12H9O. N	-0.00084	-4.38141	NA	level4	NA	C19298	NA	NA
C12 H21 N	-0.00029	-0.85472	3.196025	level4	Public	NA	NA	NA
C27 H37 F	-0.00132	-2.76538	NA	level4	HMDB	NA	HMDB024	NA
C25 H26 C	-0.00039	-0.97101	NA	level4	NA	NA	NA	NA
C28 H44 C	0.000136	0.31803	NA	level4	HMDB	NA	HMDB001	Lipids and
C11 H13 C	0.0005	2.405141	NA	level4	HMDB	NA	HMDB024	NA
C15 H10 C	-0.0004	-1.4149	81.47576	level2	mzCloud	C01514	HMDB000	Phenylpro
C17 H20 C	17.02629	50655.25	9.928958	level4	Public	NA	NA	NA
C16 H24 C	0.0002	0.53276	40.22406	level4	Public	NA	NA	NA
C7 H7 N5	-0.00018	-0.94333	NA	level4	HMDB	NA	HMDB024	NA
C18 H33 N	17.03046	52042.56	53.22958	level3	mzCloud	NA	NA	NA
C6 H13 N	0.000154	1.175709	97.61924	level1	BMDB	C00123	HMDB000	Organic ac
C23 H24 N	-0.00147	-3.27615	NA	level4	HMDB	NA	HMDB024	NA
C24 H45 N	0.002144	3.554631	NA	level4	HMDB	NA	HMDB025	NA
C16 H19 N	-0.00022	-0.62872	NA	level4	HMDB	NA	HMDB025	NA
C30 H27 N	0.000248	0.471688	NA	level4	HMDB	NA	HMDB026	NA
C7 H8 N4	0.000213	1.185172	90.6463	level1	BMDB	C07130	HMDB000	Organohe
C24 H42 C	-18.0104	-40720.4	44.27155	level4	Public	NA	NA	NA
C10 H9 N	-46.0054	-240792	77.34967	level3	NA	NA	NA	NA
C25 H36 C	-0.00027	-0.57434	80.13862	level2	mzCloud	NA	NA	NA
C19 H26 C	-0.00015	-0.43695	NA	level4	NA	NA	NA	NA
C19 H26 C	-0.00017	-0.53348	NA	level4	HMDB	NA	HMDB024	NA
C29 H50 C	0.000479	1.001913	NA	level4	HMDB	NA	HMDB025	NA
C18 H24 C	-0.00023	-0.61589	NA	level4	NA	NA	NA	NA
C19 H36 C	-5.23E-05	-0.15928	42.89488	level4	Public	NA	HMDB003	Lipids and
C13 H12 N	-0.00018	-0.72824	NA	level4	HMDB	NA	HMDB025	NA
C13 H16 C	0.000198	1.054707	NA	level4	NA	NA	NA	NA
C9 H10 O4	21.9822	120742.9	29.29312	level3	BMDB	C05582	HMDB000	Benzenoid
C22 H39 B	-0.00184	-4.43994	NA	level4	NA	NA	NA	NA
C9 H16 N2	0.000171	0.790226	NA	level4	HMDB	NA	HMDB025	NA
C9 H11 Cl	-0.00063	-2.39775	NA	level4	HMDB	NA	HMDB025	NA
C18 H26 C	-2.97E-05	-0.10251	1.656899	level4	Public	NA	HMDB006	Phenylpro
C8 H17 N	6.47E-05	0.338812	34.44533	level4	Public	NA	NA	NA
C7 H14 N2	-18.0103	-94743.4	68.87966	level2	BMDB	C00666	HMDB000	Organic ac
C15 H24 C	17.02638	59917.8	9.943897	level4	Public	NA	NA	NA
C22 H30 N	0.002918	6.15299	14.67641	level4	Public	C07259	HMDB000	Benzenoid
C27 H40 C	-0.00031	-0.66525	NA	level4	HMDB	NA	HMDB025	NA
C19 H26 C	17.02783	39586.04	2.480113	level4	Public	NA	NA	NA
C10 H14 C	-2.30E-05	-0.11616	45.52495	level3	Public	NA	NA	NA
C13 H16 N	8.46E-05	0.235037	NA	level4	HMDB	NA	HMDB025	NA
C12 H15 N	0.000314	1.238934	NA	level4	HMDB	NA	HMDB024	NA
C30 H48 C	0.000121	0.285523	61.96794	level2	Public	NA	NA	NA
C9 H8 N4	0.000526	2.228379	NA	level4	HMDB	NA	HMDB025	NA
C18 H30 C	-18.0097	-52628.4	9.370935	level4	Public	NA	NA	NA
C29 H43 N	-0.00043	-0.88077	NA	level4	HMDB	NA	HMDB025	NA
C29 H28 F	-0.00159	-2.48442	NA	level4	HMDB	NA	HMDB006	Phenylpro
C22 H26 F	-0.00056	-1.34224	NA	level4	HMDB	NA	HMDB025	NA
C12 H24 C	0.005328	15.48197	35.52441	level3	mzCloud	D04845	HMDB000	Lipids and
C26 H36 C	0.002313	4.412703	NA	level4	KEGG	C09132	NA	Terpenoid
C5 H6 N6	0.0005	3.328897	NA	level4	HMDB	NA	HMDB024	NA
C18 H23 N	0.000242	0.764058	NA	level4	HMDB	NA	HMDB025	NA

C28 H42 C	9.67E-05	0.226803	NA	level4	NA	NA	NA	NA
C6 H5 O2	4.76E-05	0.437014	NA	level4	HMDB	NA	HMDB025	NA
C30 H28 C	0.000521	0.924205	3.86393	level4	Public	NA	NA	NA
C35 H58 C	0.001818	2.921172	NA	level4	HMDB	NA	HMDB024	NA
C17 H21 N	-0.00027	-0.89996	34.23627	level3	mzCloud	C01851	HMDB000	Organic ac
C56 H74 C	-0.0003	-0.33907	NA	level4	NA	NA	NA	NA
C11 H8 O	0.000196	1.254106	NA	level4	NA	C14099	NA	Benzenoid
C6 H8	0.000138	1.722638	NA	level4	HMDB	NA	HMDB025	NA
C18 H37 N	-0.00217	-3.83742	NA	level4	KEGG	C03281	NA	NA
C32 H46 C	-3.35E-06	-0.00617	NA	level4	HMDB	NA	HMDB025	NA
C8 H15 N	-18.0102	-81461.1	55.65936	level3	BMDB	C01074	HMDB000	Organic ox
C33 H25 N	0.001252	2.449066	NA	level4	HMDB	NA	HMDB025	NA
C21 H37 E	-0.0019	-4.75789	NA	level4	NA	NA	NA	NA
C26 H43 N	-6.01E-06	-0.01439	63.7249	level2	mzCloud	C12272	NA	NA
C15 H14 N	0.000245	1.02958	NA	level4	HMDB	NA	HMDB025	NA
C4 H6 O2	0.000221	1.474648	NA	level4	KEGG	C01892	NA	Organohe
C22 H30 C	-7.64E-05	-0.2133	63.76041	level2	mzCloud	C10784	NA	PK Polyke
C21 H18 C	-0.00064	-1.26261	NA	level4	HMDB	NA	HMDB024	NA
C24 H28 C	-0.00203	-4.26538	NA	level4	NA	NA	NA	NA
C41 H78 N	0.000423	0.569021	NA	level4	HMDB	NA	HMDB024	NA
C10 H21 N	0.000135	0.789824	NA	level4	HMDB	NA	HMDB024	NA
C32 H61 N	0.000645	1.270132	NA	level4	NA	NA	NA	NA
C8 H11 N	9.12E-05	0.493042	NA	level4	HMDB	NA	HMDB024	NA

C17 H33 N	0.001522	3.279715	NA	level4	NA	C17960	NA	NA
C7 H6 O3	5.03E-05	0.364137	95.76828	level2	mzCloud	C00805	HMDB000	Benzenoid
C23 H32 C	-4.19E-05	-0.10376	NA	level4	HMDB	NA	HMDB024	NA
C20 H35 E	-0.00174	-4.50961	NA	level4	NA	NA	NA	NA
C10 H18 C	-18.0104	-116848	93.20982	level2	mzCloud	C09844	HMDB000	Organohe
C12 H14 C	0.000211	0.838392	NA	level4	HMDB	NA	HMDB006	Organohe
C28 H31 N	0.0003	0.629498	NA	level4	HMDB	NA	HMDB024	NA
C22 H43 N	6.54E-06	0.016972	NA	level4	HMDB	NA	HMDB024	NA
C29 H34 C	0.002325	4.418324	22.06061	level4	Public	NA	NA	NA
C16 H16	6.63E-05	0.318447	NA	level4	HMDB	NA	HMDB024	NA
C41 H66 C	-0.00307	-4.17683	NA	level4	HMDB	NA	HMDB024	NA
C45 H82 N	-0.00011	-0.1369	NA	level4	HMDB	NA	HMDB025	NA
C20 H23 N	21.98367	64437.5	14.97019	level4	Public	NA	NA	NA
C9 H16 O2	0.000186	1.189032	NA	level4	HMDB	NA	HMDB000	Lipids and
C17 H20 N	-9.44E-05	-0.24827	NA	level4	HMDB	NA	HMDB001	Organic ac
C19 H26 C	-0.00083	-2.49678	NA	level4	HMDB	NA	HMDB025	NA
C35 H47 F	-0.00252	-4.38675	NA	level4	HMDB	NA	HMDB025	NA
C33 H35 F	0.000723	1.29538	68.93308	level2	Public	C06834	HMDB000	Organohe
C2 H2 O3	-5.57E-05	-0.75311	91.63961	level2	mzCloud	C00048	HMDB000	Organic ac
C8 H9 N C	5.58E-05	0.304673	NA	level4	HMDB	NA	HMDB025	NA
C10 H20 C	17.02676	109040.1	14.45257	level4	Public	NA	HMDB003	Lipids and
C12 H10 C	-0.00102	-3.27692	NA	level4	HMDB	NA	HMDB025	NA
C19 H14 C	-0.00065	-1.90979	NA	level4	NA	C19979	NA	Fungal tox

C17 H33 N	-0.00037	-1.18786	94.45381	level2	mzCloud	NA	HMDB000	Lipids and
C15 H20 C	0.000119	0.404931	31.82245	level4	Public	NA	HMDB025	NA
C39 H60 C	0.000687	0.893621	NA	level4	NA	NA	NA	NA
C10 H13 F	-0.00125	-4.54118	NA	level4	HMDB	NA	HMDB025	NA
C15 H20 C	7.71E-06	0.03107	55.10387	level4	Public	NA	HMDB030	NA
C44 H78 N	-0.00048	-0.62023	NA	level4	HMDB	NA	HMDB000	Lipids and
C18 H39 C	0.000147	0.368463	NA	level4	HMDB	NA	HMDB025	NA
C6 H12	7.24E-05	0.860701	NA	level4	HMDB	NA	HMDB024	NA
C26 H28 N	0.002015	4.704409	NA	level4	HMDB	NA	HMDB025	NA
C26 H52 N	0.000996	1.854296	NA	level4	HMDB	NA	HMDB024	NA
C37 H45 N	-0.00221	-3.88956	NA	level4	HMDB	NA	HMDB025	NA
C22 H46 C	-0.00197	-3.92196	NA	level4	NA	NA	NA	NA
C14 H20 N	-0.00108	-2.98939	NA	level4	KEGG	C08324	NA	Amino aci
C26 H35 N	0.00025	0.532914	NA	level4	HMDB	NA	HMDB025	NA
C9 H10 Cl	-0.0008	-3.44811	NA	level4	HMDB	NA	HMDB025	NA
C19 H26 N	-0.00098	-2.11394	NA	level4	HMDB	NA	HMDB025	NA
C34 H35 N	-0.00045	-0.69118	NA	level4	HMDB	NA	HMDB026	NA
C12 H14 N	-0.00014	-0.49853	NA	level4	HMDB	NA	HMDB025	NA
C11 H18 C	0.000195	0.982465	NA	level4	HMDB	NA	HMDB025	NA
C26 H40 C	0.000302	0.726187	NA	level4	NA	C14634	NA	NA
C22 H43 N	-0.00032	-0.93949	NA	level4	HMDB	NA	HMDB024	NA
C23 H42 N	-0.00201	-4.22243	NA	level4	NA	NA	NA	NA
C44 H70 N	-0.00228	-2.83049	NA	level4	HMDB	NA	HMDB026	NA
C6 H11 N	0.000122	0.690584	21.98488	level3	BMDB	C03145	HMDB000	Organic ac
C21 H34 C	-18.013	-51429.7	64.96294	level2	mzCloud	NA	NA	NA
C29 H40 C	-0.00154	-2.80693	NA	level4	KEGG	C09296	NA	Terpenoid
C42 H82 N	-0.00114	-1.50218	NA	level4	HMDB	NA	HMDB000	Lipids and
C19 H35 N	-0.0003	-0.84133	NA	level4	NA	NA	NA	NA
C37 H69 C	-0.00203	-3.09225	NA	level4	NA	NA	NA	NA
C8 H10 O4	0.000208	1.223439	NA	level4	HMDB	NA	HMDB025	NA
C21 H22 C	0.000291	0.647404	NA	level4	HMDB	NA	HMDB004	Phenylpro
C33 H44 C	-0.00184	-3.65601	NA	level4	NA	C10562	NA	Phenylpro
C21 H38 C	0.001074	2.08779	NA	level4	NA	NA	NA	NA
C42 H73 C	0.003564	4.735763	NA	level4	NA	NA	NA	NA
C5 H13 O	0.000142	0.658557	NA	level4	NA	C11434	NA	NA
C18 H28 N	0.001593	3.720879	NA	level4	HMDB	NA	HMDB000	Organic ac
C28 H48 N	0.000904	1.670264	NA	level4	HMDB	NA	HMDB001	Lipids and
C21 H20 C	-0.00049	-1.44413	NA	level4	NA	NA	NA	NA
C16 H11 C	0.000706	2.248176	NA	level4	HMDB	NA	HMDB001	Organohe
C8 H4 O2	0.000159	1.200589	NA	level4	NA	NA	NA	NA
C25 H30 N	0.000245	0.548942	NA	level4	HMDB	NA	HMDB001	Benzenoid
C12 H10 C	0.00021	1.231921	NA	level4	NA	C14342	NA	NA
C24 H24 C	0.00053	1.051262	NA	level4	NA	NA	NA	NA
C24 H28 C	21.98199	49489.1	8.965679	level4	Public	NA	NA	NA
C16 H21 C	0.000622	2.099129	NA	level4	HMDB	NA	HMDB025	NA
C19 H17 C	0.001727	4.262557	NA	level4	HMDB	NA	HMDB025	NA
C12 H10 S	8.47E-05	0.455501	NA	level4	HMDB	NA	HMDB025	NA
C32 H30 C	0.001456	2.767036	NA	level4	NA	NA	NA	NA
C20 H42 N	0.000315	0.717907	NA	level4	HMDB	NA	HMDB024	NA
C27 H48 C	0.000559	1.154324	NA	level4	NA	NA	NA	NA
C5 H10 N2	7.10E-05	0.398774	87.72862	level2	mzCloud	NA	NA	NA

C21 H23 N	0.001985	5.377177	1.303807	level4	Public	C02134	HMDB030	NA	
C12 H18 N	-9.63E-05	-0.33667	NA	level4	HMDB	NA	HMDB000	Benzenoid	
C30 H39 N	-0.00188	-3.93236	NA	level4	HMDB	NA	HMDB025	NA	
C11 H19 N	0.000256	1.199126	NA	level4	HMDB	NA	HMDB025	NA	
C10 H12 N	-1.89E-05	-0.0907	NA	level4	HMDB	NA	HMDB024	NA	
C20 H34 C	-0.00011	-0.30462	35.84663	level3	mzCloud	NA	NA	NA	
C11 H11 N	0.000222	1.173687	85.18689	level1	BMDB	NA	HMDB002	Organohe	
C26 H38 N	0.000801	1.533602	NA	level4	HMDB	NA	HMDB025	NA	
C48 H89 C	-0.00278	-3.30212	NA	level4	NA	NA	NA	NA	
C6 H10 O	-0.00251	-12.9174	34.62191	level3	mzCloud	C08348	HMDB000	Organic o	
C28 H30 C	0.00039	0.596652	NA	level4	HMDB	NA	HMDB004	Phenylpro	
C13 H17 N	-8.71E-05	-0.3703	NA	level4	HMDB	NA	HMDB025	NA	
C10 H7 N	0.000458	2.234077	78.38976	level1	BMDB	C02470	HMDB000	Organohe	
C18 H31 N	-2.48E-05	-0.08036	NA	level4	NA	NA	NA	NA	
C18 H18 C	0.001168	3.216327	NA	level4	HMDB	NA	HMDB025	NA	
C25 H49 N	-3.34E-05	-0.07545	NA	level4	HMDB	NA	HMDB001	Lipids and	
C21 H38 C	-6.49E-05	-0.19194	NA	level4	NA	C19426	NA	NA	
C15 H18 C	0.000152	0.523634	22.367	level4	Public	NA	NA	NA	
C30 H37 N	0.001651	3.155688	NA	level4	NA	NA	NA	NA	
C21 H15 N	-0.00069	-1.91872	NA	level4	HMDB	NA	HMDB024	NA	
C9 H11 N	0.000227	1.525536	28.47554	level4	Public	NA	NA	NA	
C33 H50 C	0.00036	0.644	NA	level4	HMDB	NA	HMDB025	NA	
C15 H25 N	-3.06E-05	-0.10794	28.59316	level4	Public	NA	NA	NA	
C26 H24 N	0.002103	4.452835	NA	level4	HMDB	NA	HMDB025	NA	
C15 H22 C	0.000254	1.01471	24.02712	level4	Public	NA	NA	NA	
C17 H12 C	-0.00073	-2.59484	NA	level4	HMDB	NA	HMDB025	NA	
C8 H16 N2	-2.05E-05	-0.10889	9.361923	level4	Public	NA	NA	NA	
C30 H49 N	-0.00119	-2.06287	NA	level4	HMDB	NA	HMDB024	NA	
C15 H18 C	0.000121	0.490655	NA	level4	HMDB	NA	HMDB024	NA	
C20 H18 C	-0.00098	-3.04495	NA	level4	HMDB	NA	HMDB025	NA	
C18 H37 N	-0.00186	-3.93719	NA	level4	HMDB	NA	HMDB025	NA	
C19 H36 N	-0.00012	-0.38142	4.892652	level4	Public	NA	NA	NA	
C6 H7 N	0.000159	1.707422	61.53497	level2	BMDB	C00292	HMDB000	Benzenoid	
C21 H16 C	-0.00063	-1.80756	NA	level4	NA	NA	NA	NA	
C24 H30 N	0.001141	2.490624	NA	level4	HMDB	NA	HMDB006	Organohe	
C33 H46 C	0.002401	4.209116	NA	level4	NA	C11963	NA	NA	
C34 H44 N	-0.00088	-1.53927	NA	level4	HMDB	NA	HMDB025	NA	
C23 H36 C	-0.00262	-6.17706	1.897551	level4	Public	NA	NA	NA	
C15 H24 C	17.02646	63491.88	36.72416	level4	Public	NA	NA	NA	
C24 H47 C	-0.00092	-1.80663	NA	level4	NA	NA	NA	NA	
C21 H34 C	-0.00073	-1.69066	NA	level4	NA	NA	NA	NA	
C24 H34 C	0.000349	0.83401	NA	level4	HMDB	NA	HMDB025	NA	
C9 H10 O3	0.000149	0.89473	NA	level4	HMDB	NA	HMDB000	Benzenoid	
C25 H50 N	0.000608	1.199147	NA	level4	NA	NA	NA	NA	
C27 H42 C	-0.00173	-2.71745	NA	level4	NA	C17430	NA	NA	
C18 H16 C	0.001447	4.007095	NA	level4	HMDB	NA	HMDB025	NA	
C23 H22 C	0.00043	0.953242	NA	level4	HMDB	NA	HMDB024	NA	
C22 H20 N	-0.00192	-4.89651	NA	level4	HMDB	NA	HMDB006	Alkaloids a	
C19 H22 N	21.98043	74719.34	13.60421	level4	Public	NA	NA	NA	
C40 H74 C	-0.00045	-0.58719	NA	level4	NA	NA	NA	NA	
C14 H19 N	-0.00013	-0.47266	NA	level4	HMDB	NA	HMDB025	NA	
C30 H52 C	0.0011	2.163427	NA	level4	NA	NA	NA	NA	
C19 H24 C	-6.39E-05	-0.2128	47.37181	level3	mzCloud	C05299	HMDB000	Lipids and	
C28 H45 N	0.00023	0.453229	NA	level4	HMDB	NA	HMDB024	NA	
C35 H66 C	0.000852	1.503764	NA	level4	HMDB	NA	HMDB000	Lipids and	

C15 H20 C	-0.00018	-0.575	19.07692	level4	Public	NA	NA	NA	
C12 H12 C	0.00014	0.687007	33.40614	level4	Public	NA	NA	NA	
C27 H30 F	0.002167	4.102241	NA	level4	HMDB	NA	HMDB001	Lipids and	

C27 H46 C	-1.37E-05	-0.03397	34.52391	level3	mzCloud	C14151	HMDB000	Lipids and
C16 H11 N	-0.00075	-2.5611	NA	level4	HMDB	NA	HMDB025	NA
C5 H9 N C	-18.0104	-122475	74.29474	level2	BMDB	C00979	HMDB000	Organic ac
C22 H25 C	0.001899	4.236749	NA	level4	HMDB	NA	HMDB024	NA
C11 H16 C	0.000255	1.202075	NA	level4	NA	C20790	NA	NA
C11 H15 N	0.000201	0.961327	NA	level4	HMDB	NA	HMDB024	NA
C23 H24 F	-0.00131	-3.32822	NA	level4	HMDB	NA	HMDB025	NA
C38 H68 C	5.90E-06	0.010023	NA	level4	NA	NA	NA	NA
C7 H13 N	0.036622	230.1948	86.71589	level1	Decode	NA	HMDB001	Organic ac
C29 H40 C	-0.0016	-3.00983	NA	level4	KEGG	C09716	NA	NA
C21 H41 C	-0.00195	-4.15456	NA	level4	NA	NA	NA	NA
C4 H8 O2	0.055644	631.9464	82.53028	level1	Decode	C00246	HMDB000	Lipids and
C33 H48 C	0.002641	4.887548	NA	level4	HMDB	NA	HMDB025	NA
C10 H13 N	0.000204	1.139716	53.35313	level3	Public	NA	NA	NA
C17 H27 N	2.59E-05	0.079518	NA	level4	NA	C10913	NA	NA
C17 H23 N	37.9575	131264.6	12.83855	level4	Public	NA	NA	NA
C23 H39 N	-0.00014	-0.31259	NA	level4	NA	NA	NA	NA
C16 H20 C	-0.00088	-1.88177	NA	level4	HMDB	NA	HMDB025	NA
C16 H33 N	-0.00027	-0.9353	NA	level4	NA	NA	NA	NA
C33 H52 C	0.000506	0.903577	NA	level4	HMDB	NA	HMDB025	NA
C9 H7 N	0.00023	1.785797	93.1661	level2	mzCloud	C06413	HMDB003	Organohe
C25 H26 C	37.95532	97275.67	23.90624	level4	Public	NA	NA	NA
C5 H10 N2	0.000125	0.858587	93.33535	level2	mzCloud	C00303	HMDB001	Organic ac
C11 H12 C	17.02673	88644.6	18.3786	level4	Public	NA	NA	NA
C23 H27 N	0.000402	0.776702	NA	level4	HMDB	NA	HMDB001	Organic ac
C16 H10 C	2.45E-05	0.078081	50.72492	level3	Public	NA	NA	NA
C7 H13 N	0.000244	1.275956	95.60124	level1	BMDB	C02712	HMDB001	Organic ac
C8 H8 N2	-0.00221	-13.4906	63.7187	level2	mzCloud	C01526	HMDB004	Organohe
C19 H27 N	0.001907	3.687499	NA	level4	HMDB	NA	HMDB025	NA
C7 H7 N C	-3.95E-08	-0.00033	NA	level4	HMDB	NA	HMDB024	NA
C28 H26 N	-0.00184	-3.93731	NA	level4	HMDB	NA	HMDB024	NA
C9 H13 N2	-0.00545	-17.6812	32.39927	level3	mzCloud	C00365	HMDB000	Nucleoside

C19 H24 N	-0.00019	-0.54586	NA	level4	HMDB	NA	HMDB001	Benzenoid
C26 H30 N	0.000725	1.454779	12.98127	level4	Public	NA	NA	NA
C8 H14 O5	0.000361	1.626398	NA	level4	NA	C17214	NA	NA
C8 H12 O4	0.00028	1.628481	35.88885	level3	mzCloud	NA	HMDB003	Lipids and
C6 H10 N6	0.002864	17.24363	63.13801	level2	mzCloud	C14147	HMDB002	Organohe
C19 H23 N	0.000392	0.880521	NA	level4	HMDB	NA	HMDB024	NA
C33 H49 N	0.001187	2.200244	57.55863	level4	Public	NA	NA	NA
C20 H24 C	0.000152	0.357638	65.56693	level2	Public	NA	NA	NA
C8 H14 N2	0.000267	1.436677	84.60492	level2	mzCloud	NA	NA	NA
C12 H20 C	1.41E-05	0.057774	NA	level4	NA	NA	NA	NA
C30 H28 N	-0.00072	-1.38187	NA	level4	HMDB	NA	HMDB025	NA
C33 H60 N	0.0016	2.362343	NA	level4	NA	NA	NA	NA
C9 H20 N2	0.000276	1.600432	NA	level4	HMDB	NA	HMDB024	NA
C16 H12 C	-0.00064	-2.53658	NA	level4	HMDB	NA	HMDB024	NA
C25 H36 C	-3.77E-05	-0.08122	NA	level4	HMDB	NA	HMDB000	Lipids and
C28 H38 C	21.9796	45201.16	23.8489	level4	Public	NA	NA	NA
C6 H6 O5	0.000121	0.765367	NA	level4	NA	NA	NA	NA
C41 H68 C	-0.00206	-3.21009	NA	level4	HMDB	NA	HMDB000	Lipids and
C30 H44 C	-0.00078	-1.45616	14.79653	level4	Public	NA	NA	NA

C10 H12 C	3.61E-05	0.124918	NA	level4	HMDB	NA	HMDB001	Organohe
C9 H15 N	-0.00066	-2.84212	NA	level4	HMDB	NA	HMDB024	NA
C30 H38 C	-0.00056	-1.3557	NA	level4	NA	C21766	NA	NA
C8 H20 N	-2.77E-05	-0.10782	NA	level4	HMDB	NA	HMDB025	NA
C13 H14 N	-0.00041	-1.6653	94.18599	level2	mzCloud	NA	NA	NA
C21 H34 C	37.95769	99304.45	10.5589	level4	Public	NA	NA	NA
C7 H16 Cl	-0.00026	-1.29978	19.77482	level4	Public	NA	NA	NA
C5 H6 N2	0.000113	0.895093	14.20921	level3	BMDB	C02835	HMDB000	Organohe
C17 H31 N	-0.00019	-0.61809	NA	level4	HMDB	NA	HMDB025	NA
C29 H46 C	0.00097	1.802062	NA	level4	NA	NA	NA	NA
C24 H18 C	0.001027	2.455215	NA	level4	NA	NA	NA	NA
C21 H30 C	-0.0003	-0.95843	27.41901	level4	Public	NA	NA	NA
C7 H17 N	-1.37E-05	-0.07026	NA	level4	HMDB	NA	HMDB025	NA
C18 H20 N	-0.00138	-4.25813	NA	level4	HMDB	NA	HMDB025	NA
C33 H36 N	0.001442	2.688047	NA	level4	HMDB	NA	HMDB025	NA
C35 H46 C	0.000174	0.328094	NA	level4	NA	NA	NA	NA
C17 H21 N	0.000285	0.940044	NA	level4	HMDB	NA	HMDB006	Organohe
C24 H48 N	0.000208	0.420902	NA	level4	HMDB	NA	HMDB025	NA
C4 H6 N2	18.01076	157929.7	30.45659	level3	mzCloud	C02565	HMDB000	Organohe
C27 H46 C	9.79E-05	0.234008	NA	level4	HMDB	NA	HMDB024	NA
C28 H22 F	0.001586	2.996403	NA	level4	HMDB	NA	HMDB001	Benzenoid
C24 H22 F	-0.0016	-3.68375	NA	level4	HMDB	NA	HMDB025	NA
C25 H44 C	-0.00211	-4.97496	NA	level4	HMDB	NA	HMDB025	NA
C7 H10 N2	0.000677	3.351095	NA	level4	NA	C20961	NA	NA
C16 H16 C	0.001459	4.782583	NA	level4	HMDB	NA	HMDB001	Organohe
C28 H38 N	0.002412	4.415663	NA	level4	HMDB	NA	HMDB025	NA
C28 H46 C	0.000279	0.603926	NA	level4	NA	NA	NA	NA
C42 H62 C	17.02613	20702.89	60.15362	level2	Public	C02284	HMDB002	Lipids and
C32 H39 N	0.0011	2.094912	NA	level4	HMDB	NA	HMDB025	NA
C15 H20 C	-9.23E-06	-0.02956	9.185472	level4	Public	NA	NA	NA
C6 H10 O3	0.000133	1.025063	NA	level4	HMDB	NA	HMDB025	NA
C35 H52 C	0.000149	0.241888	NA	level4	HMDB	NA	HMDB025	NA
C32 H59 C	0.002109	3.500964	NA	level4	NA	NA	NA	NA
C9 H14 N2	0.000263	1.443697	NA	level4	HMDB	NA	HMDB024	NA
C16 H18 C	21.98059	80185.85	4.622372	level4	Public	NA	NA	NA
C18 H20 C	-0.00013	-0.42883	NA	level4	HMDB	NA	HMDB024	NA
C14 H15 N	-7.20E-05	-0.23296	NA	level4	HMDB	NA	HMDB001	Lipids and
C9 H17 N	-0.00017	-0.71752	NA	level4	HMDB	NA	HMDB024	NA
C20 H32 C	-18.0126	-51139.6	40.19529	level3	mzCloud	NA	NA	NA
C20 H24 N	-0.00011	-0.29969	NA	level4	HMDB	NA	HMDB025	NA
C22 H34 C	0.000946	2.610177	NA	level4	HMDB	NA	HMDB025	NA
C17 H14 C	0.003413	10.33884	49.42961	level3	mzCloud	C16754	HMDB003	Phenylpro
C20 H31 N	0.000123	0.36794	NA	level4	HMDB	NA	HMDB025	NA
C33 H48 C	-5.13E-05	-0.09215	NA	level4	HMDB	NA	HMDB025	NA
C7 H10 O3	2.33E-05	0.163954	NA	level4	HMDB	NA	HMDB024	NA
C24 H26 N	-6.42E-05	-0.14142	NA	level4	HMDB	NA	HMDB025	NA
C9 H6 N2	0.00106	3.927016	NA	level4	KEGG	C04286	NA	NA
C8 H11 N5	-0.00053	-2.07742	NA	level4	HMDB	NA	HMDB024	NA
C28 H29 F	2.30E-05	0.049774	NA	level4	HMDB	NA	HMDB001	Benzenoid
C6 H8 N2	0.000182	1.298283	81.86066	level2	mzCloud	C05828	HMDB000	Organohe
C12 H24 C	-9.81E-05	-0.45397	66.18413	level2	mzCloud	C08317	HMDB000	Organic ac

C21 H36 C	-0.00011	-0.35851	NA	level4	HMDB	NA	HMDB024	NA
C24 H26 N	0.002119	4.748306	NA	level4	HMDB	NA	HMDB001	Benzenoid
C26 H32 C	-0.0011	-2.04433	0	level4	Public	NA	NA	NA
C29 H48 C	0.000463	1.005322	NA	level4	HMDB	NA	HMDB025	NA
C25 H45 C	0.0013	2.49944	NA	level4	NA	NA	NA	NA
C14 H18 C	0.000147	0.589222	NA	level4	HMDB	NA	HMDB025	NA
C22 H30 C	-0.00023	-0.6131	NA	level4	HMDB	NA	HMDB001	Lipids and
C40 H54 C	0.002024	3.293871	NA	level4	HMDB	NA	HMDB024	NA
C24 H24 N	-0.00145	-3.94245	NA	level4	HMDB	NA	HMDB025	NA
C2 H2 Cl3	0.000493	3.762746	NA	level4	HMDB	NA	HMDB025	NA
C22 H34 C	21.98197	63485.7	26.78964	level4	Public	NA	NA	NA
C20 H33 N	0.000275	0.71849	NA	level4	HMDB	NA	HMDB025	NA
C20 H41 N	8.34E-06	0.025493	NA	level4	HMDB	NA	HMDB025	NA
C22 H27 N	0.001911	4.670422	NA	level4	HMDB	NA	HMDB024	NA
C12 H10 F	0.001055	3.934312	NA	level4	HMDB	NA	HMDB025	NA
C26 H37 N	0.000542	1.102423	NA	level4	NA	NA	NA	NA
C13 H15 N	0.000177	0.537981	NA	level4	HMDB	NA	HMDB024	NA
C38 H52 N	-0.00124	-1.59778	NA	level4	HMDB	NA	HMDB025	NA
C22 H29 F	0.000156	0.396662	71.02603	level2	Public	NA	HMDB024	NA
C15 H24 C	17.02653	59918.31	8.879554	level4	Public	NA	NA	NA
C9 H13 N	9.89E-05	0.731934	NA	level4	HMDB	NA	HMDB001	Benzenoid
C5 H4 N2	0.000519	3.707599	NA	level4	HMDB	NA	HMDB024	NA
C21 H26 N	3.00E-05	0.074699	NA	level4	HMDB	NA	HMDB025	NA
C27 H42 C	2.04E-05	0.053287	54.37152	level3	Public	NA	HMDB000	Lipids and
C23 H32 N	0.002064	4.95956	NA	level4	HMDB	NA	HMDB001	Organic ac
C16 H22 N	328.1399	1071799	58.58383	level3	Public	NA	NA	NA
C9 H18	0.000145	1.15322	NA	level4	HMDB	NA	HMDB024	NA
C5 H11 O8	1.54E-05	0.066734	14.66571	level4	BMDB	C00117	HMDB000	Organic ox
C25 H47 N	-0.00181	-3.59007	NA	level4	HMDB	NA	HMDB024	NA
C20 H41 N	-1.87E-05	-0.04779	NA	level4	NA	NA	NA	NA
C12 H12 N	-0.00061	-2.61804	NA	level4	HMDB	NA	HMDB001	Organohe
C26 H48 C	-0.00069	-1.36283	NA	level4	HMDB	NA	HMDB025	NA
C31 H39 N	0.000136	0.249381	NA	level4	KEGG	C09996	NA	Organic ac
C20 H25 N	-0.0041	-11.936	45.94704	level3	mzCloud	NA	NA	NA
C4 H10 O6	-0.00011	-0.51967	NA	level4	HMDB	NA	HMDB024	NA
C8 H8 O7	-0.00025	-1.00758	NA	level4	HMDB	NA	HMDB004	Benzenoid
C15 H24 C	1.89E-05	0.075099	NA	level4	NA	C17612	NA	NA
C12 H12 N	0.000315	1.57291	NA	level4	NA	C14759	NA	NA
C7 H6 O3	-18.0105	-130481	72.63562	level1	BMDB	C00156	HMDB000	Benzenoid
C27 H46 C	0.000753	1.465072	NA	level4	NA	NA	NA	NA
C4 H12 N	5.68E-05	0.336206	NA	level4	NA	C13482	NA	Organic ac
C21 H32 C	21.97979	49481.8	11.44305	level4	Public	NA	NA	NA
C27 H35 N	0.000372	0.720155	NA	level4	HMDB	NA	HMDB024	NA
C16 H8 O2	-0.00058	-2.49195	NA	level4	NA	C18251	NA	NA
C17 H35 N	-1.95E-05	-0.06824	NA	level4	NA	NA	NA	NA
C23 H40 C	17.02638	42964.7	24.35994	level4	Public	NA	NA	NA
C28 H38 C	-0.00208	-4.57803	NA	level4	NA	NA	NA	NA
C18 H38 N	0.001505	3.979742	NA	level4	HMDB	NA	HMDB025	NA
C16 H12 N	0.001265	4.790034	NA	level4	HMDB	NA	HMDB025	NA
C10 H18 N	5.90E-05	0.275573	97.27125	level2	mzCloud	NA	NA	NA

C44 H82 N	-0.00049	-0.63537	NA	level4	HMDB	NA	HMDB024	NA
C19 H28 C	-5.75E-05	-0.21121	NA	level4	HMDB	NA	HMDB024	NA
C27 H42 C	0.001872	3.445089	NA	level4	NA	NA	NA	NA
C14 H22 N	0.001271	3.36094	NA	level4	NA	C20333	NA	NA
C27 H46 C	0.001103	2.364529	NA	level4	HMDB	NA	HMDB000	Lipids and
C25 H52 N	-0.00083	-1.53275	NA	level4	NA	NA	NA	NA
C22 H36 C	-1.56E-05	-0.03928	NA	level4	NA	NA	NA	NA
C33 H30 N	0.000781	1.51944	78.59954	level2	Public	C07710	HMDB001	Benzenoid
C11 H8	0.000117	0.836863	NA	level4	NA	C17796	NA	NA
C21 H34 C	-0.00022	-0.68469	21.44809	level4	Public	NA	NA	NA
C25 H32 F	0.002308	4.661176	NA	level4	HMDB	NA	HMDB025	NA
C33 H56 C	0.001144	1.691637	NA	level4	NA	NA	NA	NA
C18 H35 N	-0.00014	-0.45053	NA	level4	NA	C10167	NA	Alkaloids
C18 H36 C	-2.90E-06	-0.0102	60.30893	level2	mzCloud	C01530	HMDB000	Lipids and
C27 H32 F	0.00179	3.564048	NA	level4	NA	NA	NA	NA
C29 H41 N	-0.001	-1.65148	NA	level4	HMDB	NA	HMDB025	NA
C27 H44 C	0.001003	2.237449	NA	level4	NA	NA	NA	NA
C5 H8 Cl2	0.00054	3.504193	NA	level4	HMDB	NA	HMDB024	NA
C21 H27 F	-0.00153	-3.88499	NA	level4	HMDB	NA	HMDB001	Lipids and
C32 H65 N	0.000705	1.377728	NA	level4	HMDB	NA	HMDB001	Lipids and
C41 H76 N	-0.0009	-1.07718	NA	level4	HMDB	NA	HMDB001	Organic ox
C12 H12 N	-0.00058	-2.20801	NA	level4	HMDB	NA	HMDB006	Benzenoid
C21 H26 C	-0.00034	-1.04791	NA	level4	HMDB	NA	HMDB001	Lipids and
C12 H24 N	0.001711	3.055202	NA	level4	NA	C19460	NA	NA
C14 H25 N	-5.90E-05	-0.21741	22.15775	level4	Public	NA	NA	NA
C6 H15 N	0.000166	1.249172	NA	level4	HMDB	NA	HMDB025	NA
C21 H27 N	0.000747	1.515195	NA	level4	HMDB	NA	HMDB025	NA
C11 H19 N	0.000112	0.487515	NA	level4	HMDB	NA	HMDB024	NA
C19 H22 C	37.95741	127306.8	6.546777	level4	Public	NA	NA	NA
H Br	-3.58E-05	-0.44734	NA	level4	NA	C13645	NA	NA
C19 H24 C	5.53E-05	0.145504	NA	level4	NA	NA	NA	NA
C16 H24 C	37.95734	115669.9	9.232367	level4	Public	NA	NA	NA
C25 H42 C	0.000156	0.399638	NA	level4	NA	NA	NA	NA
C26 H30 C	-0.00045	-1.06144	22.80091	level4	Public	NA	NA	NA
C20 H26 N	0.001497	4.178011	NA	level4	HMDB	NA	HMDB025	NA
C21 H43 C	0.001811	3.851212	NA	level4	NA	NA	NA	NA
C44 H82 C	0.000131	0.166894	NA	level4	NA	NA	NA	NA
C27 H39 N	-0.0017	-3.47504	NA	level4	NA	C15846	NA	NA
C18 H19 N	-0.00028	-1.0741	NA	level4	HMDB	NA	HMDB001	Benzenoid
C20 H28 C	37.95734	114260.9	16.36389	level4	Public	NA	NA	NA
C21 H22 F	-0.00153	-3.75148	9.063444	level4	Public	NA	NA	NA
C23 H28 C	0.000565	1.110616	NA	level4	HMDB	NA	HMDB006	Benzenoid
C17 H23 N	17.02651	48213.6	10.21102	level4	Public	NA	NA	NA
C27 H48 C	-5.90E-05	-0.13038	NA	level4	HMDB	NA	HMDB024	NA
C39 H65 N	-0.00179	-2.45336	NA	level4	HMDB	NA	HMDB025	NA
C13 H16 N	0.001488	4.591572	NA	level4	HMDB	NA	HMDB025	NA
C25 H50 N	-9.34E-05	-0.18415	NA	level4	NA	NA	NA	NA
C9 H12 N	0.000929	3.354609	NA	level4	NA	C14442	NA	Organic ac
C21 H43 C	5.35E-05	0.12199	NA	level4	NA	NA	NA	NA
C22 H30 C	0.000377	1.006757	NA	level4	HMDB	NA	HMDB024	NA
C26 H46 C	0.000215	0.472873	NA	level4	NA	NA	NA	NA
C20 H32 C	-3.37E-06	-0.00878	NA	level4	NA	NA	NA	NA

C4 H12 N2	3.68E-05	0.417691	85.88793	level1	BMDB	C00134	HMDB000	Organic ni
C17 H24 C	-0.00014	-0.47372	NA	level4	HMDB	NA	HMDB024	NA
C10 H14 C	0.000192	1.280241	NA	level4	HMDB	NA	HMDB025	NA
C48 H84 N	0.000468	0.487561	NA	level4	NA	C11987	NA	PK Polyke
C8 H8 F N	-0.00078	-4.24082	NA	level4	HMDB	NA	HMDB024	NA
C10 H12 N	0.000134	0.598506	62.86893	level2	BMDB2	C03227	HMDB001	Organic ox
C11 H14 N	0.000889	2.94316	NA	level4	HMDB	NA	HMDB025	NA
UnkownFo	283.1782	Inf	36.55566	level4	Public	NA	NA	NA
C27 H36 C	-0.00178	-3.31223	NA	level4	HMDB	NA	HMDB001	Lipids and
C33 H58 C	0.000822	1.538218	NA	level4	NA	NA	NA	NA
C24 H27 N	21.97972	46452.2	1.85183	level4	Public	NA	NA	NA
C41 H83 N	2.04E-05	0.031938	NA	level4	NA	NA	NA	NA
C26 H43 C	0.002024	4.494806	NA	level4	NA	NA	NA	NA
C8 H19 O2	-0.00084	-3.46213	25.37459	level4	Public	C18687	HMDB025	NA
C10 H14 C	0.000777	2.876472	NA	level4	NA	C18380	NA	NA
C20 H22 C	0.001677	4.720838	NA	level4	HMDB	NA	HMDB001	Organohe
C22 H34 C	-6.04E-05	-0.15318	NA	level4	NA	NA	NA	NA
C37 H68 C	0.000518	0.897907	NA	level4	NA	NA	NA	NA
C14 H21 N	-9.66E-06	-0.02373	NA	level4	KEGG	C03174	NA	NA
C23 H27 F	0.00084	1.971367	NA	level4	HMDB	NA	HMDB001	Organohe
C26 H47 C	-0.0009	-1.63992	NA	level4	NA	NA	NA	NA
C26 H23 N	-0.00128	-2.75035	NA	level4	HMDB	NA	HMDB024	NA
C25 H25 N	-0.00068	-1.21655	NA	level4	HMDB	NA	HMDB024	NA
C20 H20 C	0.002433	6.831473	11.15251	level4	Public	NA	NA	NA
C26 H43 N	-0.00023	-0.51426	94.28373	level1	BMDB2	NA	HMDB000	Lipids and
C25 H43 N	0.002711	4.201544	NA	level4	HMDB	NA	HMDB024	NA
C22 H23 N	0.001908	4.901694	3.94624	level4	Public	NA	NA	NA
C16 H17 N	-0.00014	-0.47867	NA	level4	HMDB	NA	HMDB025	NA
C17 H17 N	-1.90E-05	-0.05544	NA	level4	HMDB	NA	HMDB006	Benzenoid
C26 H44 C	0.002125	4.248041	NA	level4	HMDB	NA	HMDB001	Lipids and
C15 H14 N	-8.11E-05	-0.30008	NA	level4	HMDB	NA	HMDB006	Organohe
C8 H15 N	0.000124	0.716005	87.19965	level2	mzCloud	NA	NA	NA
C29 H48 C	-0.00031	-0.59658	NA	level4	NA	NA	NA	NA
C24 H24 C	-0.00042	-1.11478	NA	level4	HMDB	NA	HMDB025	NA
C35 H54 C	-0.00218	-3.43609	NA	level4	HMDB	NA	HMDB025	NA
C19 H20 C	21.98195	83217.22	11.67538	level4	Public	NA	HMDB003	Phenylpro
C25 H36 C	-5.39E-05	-0.14637	NA	level4	HMDB	NA	HMDB025	NA
C27 H44 C	0.001566	2.507888	NA	level4	NA	C17431	NA	NA
C27 H31 N	-0.0017	-3.78433	NA	level4	NA	C17485	NA	NA
C25 H21 N	-0.00187	-4.92565	NA	level4	HMDB	NA	HMDB025	NA
C18 H26 N	0.0002	0.511812	NA	level4	HMDB	NA	HMDB025	NA

C22 H23 N	0.000431	0.902846	NA	level4	NA	NA	NA	NA
C16 H17 N	-0.00021	-0.73979	NA	level4	HMDB	NA	HMDB024	NA
C18 H14 C	-0.00081	-2.8993	13.66667	level4	Public	NA	HMDB025	NA
C18 H28 C	0.001193	2.838872	NA	level4	NA	C11645	NA	Terpenoid
C21 H22 C	17.02666	40721.32	2.493645	level4	Public	NA	NA	NA
C9 H10 N2	0.000105	0.648847	NA	level4	HMDB	NA	HMDB025	NA
C18 H34 N	-0.00204	-4.23511	NA	level4	NA	C20509	NA	NA
C14 H18 N	-2.51E-05	-0.08097	NA	level4	HMDB	NA	HMDB025	NA
C22 H41 N	-0.00021	-0.53605	NA	level4	NA	NA	NA	NA
C19 H36 C	3.70E-05	0.112785	13.05297	level4	Public	NA	HMDB003	Lipids and
C27 H32 C	-0.00244	-4.9571	NA	level4	HMDB	NA	HMDB025	NA
C24 H26 C	-0.00065	-1.58367	NA	level4	NA	NA	NA	NA
C15 H14 C	9.16E-05	0.334384	NA	level4	HMDB	NA	HMDB025	NA
C51 H86 C	0.000148	0.182673	NA	level4	NA	NA	NA	NA
C20 H26 N	-0.00137	-3.28222	NA	level4	HMDB	NA	HMDB025	NA
C14 H20 C	0.000176	0.74521	NA	level4	NA	C14718	NA	Benzenoid
C6 H11 N	0.000346	1.952665	NA	level4	HMDB	NA	HMDB000	Organosul
C16 H20 C	-0.00159	-4.87203	NA	level4	HMDB	NA	HMDB024	NA
C26 H39 N	-0.00013	-0.26141	NA	level4	HMDB	NA	HMDB025	NA
C41 H81 N	0.001454	2.177997	NA	level4	NA	NA	NA	NA
C32 H47 F	0.000967	1.594328	NA	level4	HMDB	NA	HMDB025	NA
C12 H13 N	0.000222	1.092374	NA	level4	HMDB	NA	HMDB001	Organohe
C14 H13 N	0.000271	0.809917	NA	level4	HMDB	NA	HMDB025	NA
C26 H29 N	37.95818	76040.43	11.29483	level4	Public	NA	NA	NA
C25 H31 F	-0.00168	-3.5192	NA	level4	HMDB	NA	HMDB025	NA
C8 H10 O2	0.000236	1.708657	50	level3	Masslist	C06728	HMDB004	Benzenoid
C23 H40 C	-0.00117	-2.73705	NA	level4	NA	NA	NA	NA
C37 H42 F	0.000784	1.11928	NA	level4	HMDB	NA	HMDB001	Organohe
C21 H36 C	2.96E-05	0.080412	NA	level4	HMDB	NA	HMDB000	Organic ox
C16 H14 C	-0.00132	-3.92491	NA	level4	NA	C19090	NA	NA
C47 H70 C	0.003075	4.505275	NA	level4	KEGG	C05809	NA	NA
C16 H29 N	4.38E-05	0.139102	NA	level4	NA	C12946	NA	NA
C17 H32 C	-3.14E-05	-0.10452	25.57317	level4	Public	NA	NA	NA
C22 H30 C	-2.10E-05	-0.06767	NA	level4	HMDB	NA	HMDB001	Lipids and
C36 H66 C	0.00027	0.391237	NA	level4	NA	NA	NA	NA
C14 H19 N	-7.39E-05	-0.27871	35.24962	level3	mzCloud	NA	NA	NA
C7 H13 N	0.000241	1.51699	NA	level4	HMDB	NA	HMDB000	Organic ac
C17 H28 C	0.000107	0.405364	NA	level4	HMDB	NA	HMDB024	NA
C28 H50 N	0.000612	1.278429	NA	level4	NA	C10135	NA	Alkaloids
C19 H20 C	17.02623	45268.58	4.614604	level4	Public	NA	NA	NA
C13 H19 N	17.02652	67263.55	0.786056	level4	Public	NA	NA	NA
C4 H6 O2	86.03717	1000005	59.49043	level3	BMDB	C00741	HMDB000	Organic ox
C25 H24 F	-0.00104	-2.38117	NA	level4	HMDB	NA	HMDB024	NA
C15 H11 C	-0.00104	-3.43786	NA	level4	HMDB	NA	HMDB025	NA
C19 H20 C	-145.078	-464790	18.09548	level4	Public	NA	NA	NA
C10 H16 C	3.56E-05	0.211707	49.96541	level3	Public	NA	NA	NA
C19 H16 C	-0.00078	-2.65415	NA	level4	HMDB	NA	HMDB025	NA
C21 H41 N	-0.00029	-0.73763	NA	level4	HMDB	NA	HMDB001	Lipids and
C26 H28 C	0.000363	0.642741	19.66794	level4	Public	NA	HMDB003	Phenylpro
C32 H63 N	0.000832	1.583569	NA	level4	NA	NA	NA	NA
C27 H48 C	0.001367	2.492614	NA	level4	NA	NA	NA	NA
C29 H52 N	-0.00011	-0.18244	NA	level4	NA	NA	NA	NA
C10 H12 C	6.60E-05	0.366698	46.68482	level3	mzCloud	D01422	HMDB003	Benzenoid
C10 H12 N	37.9569	182410.7	14.07862	level4	Public	C01718	HMDB025	NA
C27 H25 C	-0.00036	-0.70018	NA	level4	HMDB	NA	HMDB025	NA

C12H6Cl2I	-0.00041	-1.41048	NA	level4	KEGG	C03822	NA	NA
C17 H14 C	4.96E-05	0.125873	NA	level4	NA	NA	NA	NA
C36 H43 N	-0.00029	-0.50339	NA	level4	HMDB	NA	HMDB024	NA
C15 H20 C	0.00154	4.69273	9.392844	level4	Public	NA	NA	NA
C22 H28 N	-0.00059	-1.52661	NA	level4	HMDB	NA	HMDB025	NA
C14 H11 I	-0.00139	-3.74573	NA	level4	HMDB	NA	HMDB024	NA
C19 H27 N	-0.00021	-0.54333	NA	level4	NA	C10359	NA	Alkaloids
C26 H32 N	-0.0017	-3.76855	NA	level4	HMDB	NA	HMDB025	NA
C25 H44 N	0.000793	1.581329	NA	level4	HMDB	NA	HMDB025	NA
C42 H56 C	0.001699	2.721344	NA	level4	NA	NA	NA	NA
C13 H12 C	0.001397	4.298619	NA	level4	HMDB	NA	HMDB025	NA
C35 H60 C	0.000467	0.832415	NA	level4	NA	NA	NA	NA
C5 H6 N2	8.81E-05	0.937148	NA	level4	HMDB	NA	HMDB025	NA
C13 H12 C	0.000185	1.003091	NA	level4	HMDB	NA	HMDB024	NA
C37 H66 C	0.000378	0.657405	NA	level4	NA	NA	NA	NA
C10 H9 N	0.000262	1.267573	NA	level4	HMDB	NA	HMDB024	NA
C11 H20 N	0.000148	0.650639	90.93988	level2	mzCloud	NA	NA	NA
C13 H15 N	-2.63E-05	-0.11283	NA	level4	HMDB	NA	HMDB024	NA
C21 H24 F	-0.0005	-1.25134	NA	level4	HMDB	NA	HMDB001	Organohe
C5 H14 N	0.000196	1.068452	64.84613	level2	Public	NA	NA	NA
C9 H9 F O	0.000847	4.232011	NA	level4	HMDB	NA	HMDB024	NA
C19 H20 C	18.9727	67724.24	13.39449	level4	Public	NA	NA	NA
C21 H45 C	0.000155	0.364788	NA	level4	NA	NA	NA	NA
C20 H36 C	2.81E-05	0.096123	NA	level4	NA	NA	NA	NA
C26 H52 N	0.000389	0.746339	20.1185	level4	Public	NA	HMDB000	Lipids and
C24 H40 C	-0.00207	-4.53805	NA	level4	HMDB	NA	HMDB000	Lipids and
C27 H47 C	-0.00029	-0.51945	NA	level4	NA	NA	NA	NA
C14 H18 C	17.0264	60353.71	14.70695	level4	Public	NA	NA	NA
C12 H18 N	-8.38E-05	-0.27733	NA	level4	NA	C15486	NA	NA
C14 H19 N	-3.51E-05	-0.13455	NA	level4	HMDB	NA	HMDB006	Organohe
C15 H18 C	-9.78E-05	-0.28586	NA	level4	HMDB	NA	HMDB012	Organic ox
C36 H34 N	-0.00173	-2.85477	NA	level4	KEGG	C09370	NA	NA
C29 H46 C	8.74E-05	0.184296	NA	level4	NA	NA	NA	NA
C32 H42 C	0.001829	3.120156	3.401003	level4	Public	NA	NA	NA
C16 H24 C	1.11E-05	0.037517	32.58928	level4	Public	NA	NA	NA
C14 H22 N	6.05E-05	0.227487	NA	level4	HMDB	NA	HMDB025	NA
C27 H46 C	-0.00014	-0.35879	10.12019	level4	Public	NA	NA	NA
C19 H24 N	-0.00011	-0.30089	NA	level4	HMDB	NA	HMDB025	NA
C18 H32 C	37.95606	89477.97	5.736174	level4	Public	NA	NA	NA
C48 H38 N	-0.00273	-3.72065	NA	level4	HMDB	NA	HMDB024	NA
C24 H40 C	0.000313	0.737517	NA	level4	NA	NA	NA	NA
C20 H39 N	7.81E-05	0.200597	NA	level4	NA	NA	NA	NA
C14 H17 N	0.000234	0.794307	NA	level4	HMDB	NA	HMDB025	NA
C28 H40 C	-0.00212	-4.34176	NA	level4	NA	NA	NA	NA
C34 H66 C	0.000922	1.490493	NA	level4	NA	NA	NA	NA
C24 H30 C	-0.00206	-4.02844	4.629858	level4	Public	NA	NA	NA
C31 H44 C	0.000268	0.576851	NA	level4	NA	NA	NA	NA
C3 H6 N4	0.000194	1.4945	NA	level4	HMDB	NA	HMDB024	NA
C18 H30 N	7.38E-06	0.021063	NA	level4	HMDB	NA	HMDB024	NA
C33 H54 C	0.000527	0.886524	NA	level4	NA	NA	NA	NA
C5 H4 O2	7.67E-05	0.599024	NA	level4	HMDB	NA	HMDB025	NA
C16 H13 N	0.001685	5.709749	0.316402	level4	Public	NA	HMDB001	Benzenoid

C11 H22 N	-0.00011	-0.40404	NA	level4	KEGG	C00831	NA	Organic ac
C27 H28 N	0.002207	4.880873	NA	level4	HMDB	NA	HMDB025	NA
C27 H40 C	-0.00021	-0.42678	71.77244	level2	mzCloud	NA	NA	NA
C18 H36 N	21.99168	45415.01	38.84838	level3	mzCloud	C00304	HMDB001	Organic ox
C16 H28 C	0.001451	4.366727	NA	level4	HMDB	NA	HMDB025	NA
C22 H28 C	0.002188	4.501383	NA	level4	HMDB	NA	HMDB025	NA
C34 H52 C	0.001225	1.924434	NA	level4	NA	NA	NA	NA
C27 H41 N	0.000317	0.666572	NA	level4	HMDB	NA	HMDB001	Lipids and
C23 H29 N	0.000266	0.617102	NA	level4	NA	C19243	NA	Phenylpro
C28 H39 N	0.000665	1.480298	NA	level4	HMDB	NA	HMDB025	NA
C5 H5 N C	0.000123	1.297642	54.83676	level3	Public	NA	NA	NA
C22 H23 C	0.000273	0.714565	NA	level4	HMDB	NA	HMDB000	Organohe
C33 H54 C	0.001656	2.64387	NA	level4	KEGG	C09078	NA	Terpenoid
C10 H16 N	0.00012	0.567943	NA	level4	HMDB	NA	HMDB001	Organohe
C28 H33 C	0.001132	2.619431	NA	level4	HMDB	NA	HMDB001	Benzenoid
C6 H12 N2	0.000266	2.076354	NA	level4	HMDB	NA	HMDB025	NA
C31 H44 C	21.98561	36143.7	18.43989	level4	Public	NA	NA	NA
C51 H88 N	0.003654	3.831997	NA	level4	NA	NA	NA	NA
C15 H22 C	-0.00013	-0.44048	28.92135	level4	Public	NA	NA	NA
C15 H20 C	-8.98E-05	-0.32052	27.60558	level4	Public	NA	NA	NA
C27 H21 C	-0.0018	-3.81414	NA	level4	HMDB	NA	HMDB025	NA
C40 H38 C	-0.00211	-2.72719	NA	level4	NA	NA	NA	NA
C17 H13 C	0.002023	4.933455	NA	level4	NA	C18468	NA	NA
C18 H26 N	0.0017	4.693475	NA	level4	HMDB	NA	HMDB025	NA
C24 H36 N	6.31E-05	0.116826	NA	level4	HMDB	NA	HMDB025	NA
C29 H40 C	0.002251	4.229194	NA	level4	KEGG	C08854	NA	Terpenoid
C17 H29 N	-0.0002	-0.59377	32.61484	level4	Public	NA	NA	NA
C23 H41 N	-0.00128	-2.9446	NA	level4	NA	C20052	NA	Venoms
C19 H36 N	-0.00033	-0.93346	NA	level4	NA	NA	NA	NA
C18 H17 F	-0.0018	-4.91717	NA	level4	HMDB	NA	HMDB001	Benzenoid
C21 H21 F	-0.00168	-3.98962	NA	level4	HMDB	NA	HMDB024	NA
C8 H9 N C	5.90E-05	0.352875	62.1672	level2	BMDB	C00250	HMDB000	Organohe
C12 H12 C	0.000177	0.804371	16.57188	level4	Public	NA	HMDB002	Organohe
C16 H14 C	-0.00012	-0.41418	64.44661	level2	Public	NA	HMDB025	NA
C29 H34 C	8.22E-05	0.128872	NA	level4	NA	NA	NA	NA
C24 H39 N	0.000149	0.339651	NA	level4	HMDB	NA	HMDB024	NA
C40 H80 N	0.000203	0.276101	88.5497	level2	mzCloud	NA	NA	NA
C23 H30 N	0.013957	33.69488	15.6061	level4	Public	NA	HMDB024	NA
C24 H24 C	17.02704	37328.37	14.37304	level4	Public	NA	NA	NA
C17 H25 N	-0.00012	-0.39573	26.73802	level4	Public	NA	NA	NA
C39 H62 C	0.003404	4.511596	NA	level4	HMDB	NA	HMDB025	NA
C16 H15 F	-0.00114	-3.58698	NA	level4	HMDB	NA	HMDB024	NA
C10 H17 N	-6.93E-05	-0.22576	NA	level4	HMDB	NA	HMDB000	Organic ac
C8 H9 N C	0.000196	1.294904	82.23065	level2	mzCloud	C20634	HMDB002	Benzenoid
C31 H43 N	-18.0182	-30784.2	30.32168	level3	mzCloud	D06650	NA	NA
C10 H8 O3	0.000202	1.145011	10.0011	level4	Public	C09268	HMDB002	Phenylpro
C10 H14 C	-2.30E-05	-0.11616	49.68417	level3	Public	NA	NA	NA
C14 H10 C	0.000752	2.9146	29.50599	level4	Public	NA	NA	NA
C22 H26 C	-9.73E-05	-0.19974	NA	level4	HMDB	NA	HMDB001	Benzenoid
C23 H24 C	-0.00018	-0.48111	NA	level4	HMDB	NA	HMDB024	NA
C17 H22 N	-0.00125	-4.09448	NA	level4	NA	C10648	NA	Alkaloids
C40 H46 N	-0.00285	-4.20783	NA	level4	HMDB	NA	HMDB025	NA
C19 H16 C	0.00153	4.284064	18.40317	level4	Public	C01926	HMDB001	Organohe
C34 H63 N	0.000856	1.605191	NA	level4	NA	NA	NA	NA

C9 H12 N3	18.00994	59041	38.80892	level3	BMDB	C02354	HMDB001	Organic ac
C27 H34 C	-0.00062	-1.51484	NA	level4	HMDB	NA	HMDB024	NA
C39 H67 C	0.003325	4.787339	NA	level4	NA	NA	NA	NA
C16 H16 C	0.000325	1.195931	8.749676	level4	Public	NA	NA	NA
C14 H22	0.000181	0.952157	14.0104	level4	Public	NA	NA	NA
C8 H8 O	0.000122	1.01942	NA	level4	KEGG	C07209	NA	Benzenoid
C13 H16 C	0.00011	0.462575	8.429374	level4	Public	C07525	HMDB001	Benzenoid
C25 H34 N	0.001255	2.560412	NA	level4	HMDB	NA	HMDB025	NA
C23 H41 B	-0.00172	-4.00529	NA	level4	NA	NA	NA	NA
C24 H50 N	0.000139	0.280434	16.44208	level4	Public	C04230	HMDB001	Lipids and
C18 H18 N	-0.00024	-0.72383	NA	level4	NA	C20516	NA	NA
C11 H13 N	0.00016	0.597273	NA	level4	HMDB	NA	HMDB025	NA
C31 H40 C	-0.00252	-4.39849	43.09171	level4	Public	NA	NA	NA
C27 H48 C	-0.00012	-0.23935	NA	level4	NA	NA	NA	NA
C9 H19 N	0.000224	1.186884	NA	level4	HMDB	NA	HMDB025	NA
C11 H17 N	0.000773	2.10555	NA	level4	HMDB	NA	HMDB025	NA
C13 H23 N	2.89E-05	0.082787	NA	level4	HMDB	NA	HMDB025	NA
C16 H17 N	3.03E-05	0.091371	NA	level4	HMDB	NA	HMDB001	Organohe
C23 H26 C	0.002214	4.480275	NA	level4	HMDB	NA	HMDB002	Phenylpro
C21 H30 C	37.95574	89058.48	7.961071	level4	Public	NA	NA	NA
C12 H24 N	-0.00018	-0.54837	NA	level4	HMDB	NA	HMDB024	NA
C17 H29 N	-0.00012	-0.37792	22.58297	level4	Public	NA	NA	NA
C37 H71 C	-0.00206	-3.05862	NA	level4	HMDB	NA	HMDB000	Lipids and
C24 H40 N	0.000988	1.876901	NA	level4	HMDB	NA	HMDB000	Organic ac
C14 H14	0.000208	1.141208	NA	level4	HMDB	NA	HMDB024	NA
C15 H22 N	-0.00015	-0.52269	NA	level4	HMDB	NA	HMDB001	Organic ac
C19 H29 N	-0.00021	-0.61622	NA	level4	KEGG	C09337	NA	Alkaloids
C30 H46 C	0.000297	0.63241	94.43534	level2	mzCloud	C02283	NA	Terpenoid
C14 H25 N	-0.00029	-0.95911	NA	level4	HMDB	NA	HMDB025	NA
C9 H15 N3	0.000942	2.336431	NA	level4	HMDB	NA	HMDB000	Nucleoside
C44 H80 N	-0.00084	-1.08111	NA	level4	HMDB	NA	HMDB000	Lipids and
C15 H24 C	17.02612	56724.01	12.96023	level4	Public	NA	NA	NA
C12 H18 N	-0.00025	-0.72716	NA	level4	KEGG	C01081	NA	Organohe
C10 H16 N	-0.00034	-1.06068	NA	level4	HMDB	NA	HMDB024	NA
C48 H76 C	-0.00434	-4.38655	NA	level4	KEGG	C08959	NA	Terpenoid
C30 H48 C	18.01088	42441.4	27.04941	level4	Public	NA	NA	NA
C10 H12 C	0.000218	1.471926	18.32551	level4	Public	NA	HMDB003	Benzenoid
C9 H13 N	9.95E-05	0.543583	75.52061	level2	mzCloud	C00788	HMDB000	Benzenoid
C14 H10 C	0.000251	1.195659	NA	level4	HMDB	NA	HMDB024	NA
C21 H34 C	-2.06E-05	-0.06168	13.7687	level4	Public	NA	NA	NA
C16 H19 N	0.00087	3.185367	NA	level4	HMDB	NA	HMDB006	Benzenoid
C21 H20 C	-0.00059	-1.66173	NA	level4	HMDB	NA	HMDB025	NA
C27 H45 C	0.000365	0.691671	NA	level4	NA	NA	NA	NA
C25 H48 C	0.001467	3.185961	NA	level4	NA	NA	NA	NA
C27 H34 C	-0.00226	-4.79811	NA	level4	HMDB	NA	HMDB025	NA
C16 H24 N	0.002491	4.699816	NA	level4	KEGG	C06622	NA	NA
C32 H48 C	-0.00142	-2.7643	NA	level4	HMDB	NA	HMDB024	NA
C16 H17 F	0.00097	2.920512	NA	level4	HMDB	NA	HMDB024	NA
C21 H38 C	408.2501	1056912	0.899446	level4	Public	NA	NA	NA
C3 H7 N C	2.29E-05	0.217777	91.03591	level1	BMDB	C00065	HMDB000	Organic ac
C26 H34 C	-0.00084	-2.12754	NA	level4	HMDB	NA	HMDB025	NA

C20 H26 N	-0.00185	-4.779	NA	level4	HMDB	NA	HMDB024	NA	
C8H4O12S	0.002872	4.300461	NA	level4	NA	C11340	NA	NA	
C15 H23 N	4.62E-06	0.016438	45.04379	level4	Public	NA	NA	NA	
C6 H5 N C	0.000185	1.50397	69.30484	level1	BMDB2	C00253	HMDB000	Organohe	
C13 H10	0.000162	0.975833	NA	level4	HMDB	NA	HMDB025	NA	
C29 H42 C	-0.00214	-4.711	NA	level4	NA	NA	NA	NA	
C14 H21 N	-0.00018	-0.70284	NA	level4	HMDB	NA	HMDB024	NA	
C16 H18 C	-4.42E-05	-0.16129	9.103877	level4	Public	NA	NA	NA	
C13 H14 C	0.000193	0.956273	NA	level4	HMDB	NA	HMDB025	NA	
C21 H26 N	0.001064	2.754481	NA	level4	HMDB	NA	HMDB025	NA	
C15 H26 N	-9.01E-05	-0.2867	NA	level4	HMDB	NA	HMDB024	NA	
C26 H29 N	37.95817	76040.4	1.040194	level4	Public	NA	NA	NA	
C19 H29 F	-0.00115	-3.73746	NA	level4	HMDB	NA	HMDB026	NA	
C24 H36 C	0.000219	0.56505	NA	level4	NA	NA	NA	NA	
C20 H34 C	-0.00252	-7.80548	38.59719	level3	mzCloud	NA	NA	NA	
C19 H22 C	21.98014	66576.89	44.42089	level4	Public	NA	NA	NA	
C16 H28 C	-0.0002	-0.60515	51.33102	level4	Public	NA	NA	NA	
C39 H78 N	0.00105	1.459491	NA	level4	NA	NA	NA	NA	
C21 H20 C	-0.00026	-0.84038	NA	level4	HMDB	NA	HMDB025	NA	
C4 H8 O3	6.42E-06	0.061706	94.29017	level2	mzCloud	NA	HMDB000	NA	
C22 H26 C	-0.0005	-1.28697	NA	level4	NA	C10561	NA	Phenylpro	
C8 H10 N2	0.000343	1.490715	NA	level4	HMDB	NA	HMDB024	NA	
C32 H52 C	-0.00152	-2.61667	NA	level4	KEGG	C08893	NA	Terpenoid	
C42 H70 C	-0.00184	-2.30251	NA	level4	KEGG	C08985	NA	Terpenoid	
C8 H15 N	-0.0009	-4.38045	NA	level4	NA	NA	NA	NA	
C29 H51 N	-0.00077	-1.41712	NA	level4	NA	NA	NA	NA	
C23 H36 C	0.000392	0.999853	NA	level4	HMDB	NA	HMDB006	Lipids and	
C30 H48 C	-1.98E-05	-0.03381	NA	level4	HMDB	NA	HMDB000	Lipids and	
C39 H38 N	0.002351	3.405594	NA	level4	HMDB	NA	HMDB025	NA	
C22 H24 N	-0.00115	-2.60612	NA	level4	HMDB	NA	HMDB024	NA	
C23 H39 N	-2.76E-05	-0.07648	NA	level4	HMDB	NA	HMDB024	NA	
C27 H38 N	-0.00159	-3.48966	NA	level4	HMDB	NA	HMDB000	Benzenoid	
C20 H34 C	-18.0106	-50842.8	41.81421	level3	mzCloud	C04741	HMDB000	Lipids and	
C21 H24 C	21.98199	56488.72	10.96431	level4	Public	NA	NA	NA	
C25 H47 N	0.000201	0.454311	NA	level4	HMDB	NA	HMDB001	Lipids and	
C28 H48 C	-0.0004	-0.75498	NA	level4	NA	NA	NA	NA	
C26 H37 N	0.000386	0.811903	NA	level4	NA	NA	NA	NA	
C8 H8 F N	0.000992	4.049626	NA	level4	HMDB	NA	HMDB024	NA	
C18 H15 C	-0.00043	-1.23585	NA	level4	NA	C10993	NA	NA	
C28 H46 C	0.000261	0.585832	NA	level4	HMDB	NA	HMDB025	NA	
C18 H31 N	0.00013	0.320921	NA	level4	NA	NA	NA	NA	
C29 H42 C	-0.00204	-4.64657	NA	level4	NA	NA	NA	NA	
C7 H12 O2	7.24E-05	0.565406	NA	level4	HMDB	NA	HMDB025	NA	
C23 H40 C	6.74E-05	0.17013	NA	level4	NA	NA	NA	NA	
C18 H24 N	-0.00153	-4.05776	NA	level4	HMDB	NA	HMDB025	NA	
C25 H37 N	0.000336	0.725866	NA	level4	NA	NA	NA	NA	
C9 H6 N2	-4.57E-05	-0.24032	39.54769	level4	Public	C03474	HMDB024	NA	
C15 H19 C	0.001433	4.806138	NA	level4	HMDB	NA	HMDB024	NA	
C22 H37 N	-0.00185	-3.08932	NA	level4	HMDB	NA	HMDB025	NA	
C27 H28 C	-0.00122	-2.125	NA	level4	NA	NA	NA	NA	
C20 H28 C	-0.00016	-0.53139	82.98011	level2	mzCloud	C07058	HMDB000	Lipids and	

C8 H9 N C	0.00019	1.259248	67.15938	level2	mzCloud	NA	HMDB000	Organic ac
C23 H32 C	-1.46E-05	-0.04509	NA	level4	NA	NA	NA	NA
C6 H8 N4	0.000186	0.860764	NA	level4	NA	C19468	NA	NA
C32 H38 C	0.00132	2.041909	NA	level4	HMDB	NA	HMDB025	NA
C19 H23 N	-0.00124	-3.09006	NA	level4	HMDB	NA	HMDB025	NA
C16 H24 C	9.10E-05	0.366707	NA	level4	HMDB	NA	HMDB025	NA
C20 H30 C	-0.0004	-1.08723	32.80403	level4	Public	NA	NA	NA
(C4H5O3)3	-0.00015	-0.44979	NA	level4	NA	C17688	NA	NA
C23 H38 C	0.000344	0.845539	NA	level4	HMDB	NA	HMDB025	NA
C33 H40 C	0.000322	0.416628	NA	level4	HMDB	NA	HMDB024	NA
C21 H25 N	-0.00022	-0.58442	NA	level4	HMDB	NA	HMDB026	NA
C8 H6 O3	0.000192	1.278986	NA	level4	HMDB	NA	HMDB024	NA
C5 H7 N3	0.000212	1.946846	NA	level4	HMDB	NA	HMDB024	NA
C6 H12 O5	2.72E-05	0.13899	NA	level4	HMDB	NA	HMDB024	NA
C18 H39 N	-0.00083	-2.62872	NA	level4	NA	NA	NA	NA
C16 H27 N	18.01035	57506.34	0.36321	level4	Public	NA	NA	NA
C23 H25 N	0.002012	4.989364	NA	level4	HMDB	NA	HMDB025	NA
C24 H33 C	-0.00252	-4.95047	NA	level4	HMDB	NA	HMDB025	NA
C17 H31 N	9.73E-06	0.03106	NA	level4	HMDB	NA	HMDB001	Lipids and
C24 H40 C	-20.0262	-51049.1	94.41164	level1	BMDB2	NA	HMDB000	Lipids and
C18 H22 N	-0.00015	-0.4616	NA	level4	HMDB	NA	HMDB025	NA
C29 H38 N	0.00249	3.211894	NA	level4	KEGG	C01080	NA	NA
C29 H50 C	0.000185	0.415233	NA	level4	NA	NA	NA	NA
C25 H46 C	0.00137	2.47114	NA	level4	NA	NA	NA	NA
C11 H16 C	0.000201	1.11461	NA	level4	HMDB	NA	HMDB026	NA
C25 H39 N	35.03538	80860.31	20.14039	level4	Public	NA	NA	NA
C12 H24 N	-5.02E-05	-0.15489	NA	level4	HMDB	NA	HMDB000	Organic ac
C25 H35 N	-0.0025	-4.95191	NA	level4	HMDB	NA	HMDB001	Benzenoid
C19 H14 C	-0.00067	-2.09546	0.823788	level4	Public	NA	NA	NA
C12 H22 C	-4.48E-05	-0.10611	NA	level4	NA	C20985	NA	NA
C31 H38 C	0.001657	2.826356	1.810684	level4	Public	NA	NA	NA
C19 H35 N	0.000119	0.348311	NA	level4	HMDB	NA	HMDB001	Lipids and
C17 H17 N	-9.46E-05	-0.26334	NA	level4	HMDB	NA	HMDB006	Benzenoid
C32 H58 N	-0.00261	-4.13512	NA	level4	HMDB	NA	HMDB024	NA
C20 H28 C	-0.00013	-0.31034	NA	level4	HMDB	NA	HMDB024	NA
C26 H53 N	-0.00105	-2.01518	NA	level4	KEGG	C03916	NA	GP Glycer
C H F3 O	8.74E-06	0.101627	NA	level4	HMDB	NA	HMDB025	NA
C12 H14 N	-5.89E-05	-0.20024	NA	level4	HMDB	NA	HMDB001	Benzenoid
C17 H30 C	41.02615	137572.7	19.39687	level4	Public	NA	NA	NA
C15 H13 C	0.000726	1.885873	NA	level4	HMDB	NA	HMDB001	Phenylpro
C21 H34 C	-18.0107	-49177.3	85.79415	level2	mzCloud	NA	NA	NA
C18 H12 C	0.001209	4.379477	NA	level4	HMDB	NA	HMDB025	NA
C12 H23 N	-0.00014	-0.50462	NA	level4	NA	C20279	NA	NA
C8 H14 O	0.00012	0.951668	NA	level4	HMDB	NA	HMDB003	Organic ox
C8 H18 N4	0.000367	1.816224	NA	level4	HMDB	NA	HMDB025	NA
C17 H29 N	0.002279	4.307977	NA	level4	NA	C21395	NA	NA
C10 H12 C	0.000116	0.704966	NA	level4	HMDB	NA	HMDB024	NA
C19 H32 C	0.00079	1.748253	NA	level4	HMDB	NA	HMDB024	Lipids and
C4 H5 N3	111.0436	1000003	55.73165	level4	BMDB	C00380	HMDB000	Organohe
C18 H30 C	-0.00117	-2.58423	NA	level4	HMDB	NA	HMDB025	NA
C9 H16 N2	-4.68E-05	-0.21646	NA	level4	HMDB	NA	HMDB024	Organic ac
C29 H53 C	-0.00022	-0.3583	NA	level4	NA	NA	NA	NA
C19 H26 N	0.000657	1.814121	NA	level4	NA	C11709	NA	NA
C20 H24 N	-3.06E-05	-0.07581	NA	level4	NA	C12220	NA	NA
C23 H45 N	0.000132	0.317727	NA	level4	HMDB	NA	HMDB001	Lipids and
C26 H37 N	0.000458	1.014363	NA	level4	HMDB	NA	HMDB001	Alkaloids a
C26 H41 N	4.20E-05	0.08756	NA	level4	KEGG	C08675	NA	Alkaloids

C20 H32 (	0.003544	9.624343	43.67299	level3	mzCloud	NA	NA	NA
C21 H28 (	-0.00011	-0.34111	58.58007	level3	Public	NA	HMDB001	Lipids and
C13 H21 N	-6.65E-05	-0.26074	NA	level4	NA	C10861	NA	Alkaloids
C25 H50 N	-0.00058	-1.07292	NA	level4	HMDB	NA	HMDB024	NA
C8 H11 N	0.000288	2.104317	NA	level4	HMDB	NA	HMDB025	NA
C28 H43 N	0.000577	1.179229	NA	level4	NA	C15687	NA	NA
C15 H24 (	-18.0104	-67161.2	40.99674	level4	Public	NA	NA	NA
C11 H20 N	-0.00015	-0.62872	NA	level4	HMDB	NA	HMDB024	NA
C30 H42 N	0.001341	2.568045	NA	level4	HMDB	NA	HMDB006	Benzenoid
C H4 O4 S	4.73E-05	0.422064	NA	level4	HMDB	NA	HMDB025	NA
C15 H20 (	6.61E-05	0.250303	19.22015	level4	Public	NA	NA	NA
C15 H22 (	4.65E-05	0.164709	28.60363	level4	Public	NA	NA	NA
C25 H25 N	0.00057	1.231099	NA	level4	HMDB	NA	HMDB025	NA
C15 H22 (	37.95725	142615	50.23446	level4	Public	NA	NA	NA
C6 H8 N2	-0.0008	-3.40266	NA	level4	HMDB	NA	HMDB001	Organohe
C17 H15 N	6.28E-05	0.20052	NA	level4	HMDB	NA	HMDB024	NA
C21 H40 (	17.02651	47787.99	93.64523	level1	BMDB	NA	HMDB001	Lipids and
C34 H59 N	37.9536	53804.86	4.149524	level4	Public	NA	NA	NA
C7 H5 N S	0.000234	1.398486	NA	level4	NA	C14437	NA	Organohe
C15 H17 B	0.001458	4.732542	NA	level4	NA	C17177	NA	NA
C46 H78 N	-0.00228	-2.8415	NA	level4	HMDB	NA	HMDB026	NA
C24 H26 N	0.001187	2.326375	NA	level4	HMDB	NA	HMDB025	NA
C26 H34 (	17.02691	38501.9	35.38516	level4	Public	NA	NA	NA
C28 H48 (	0.001232	2.331586	NA	level4	NA	NA	NA	NA
C20 H18 (	21.98194	62076.53	19.89261	level4	Public	C10882	HMDB003	Lignans, n
C28 H52 (	-0.00085	-1.28624	NA	level4	NA	C22176	NA	NA
C31 H34 (	0.001589	2.458475	NA	level4	NA	C20977	NA	NA
C23 H34 (	-0.00013	-0.36769	NA	level4	HMDB	NA	HMDB025	NA
C24 H32 (	-18.0106	-41670.5	17.17279	level4	Public	NA	NA	NA
C19 H30 (	-0.00141	-3.6601	NA	level4	NA	NA	NA	NA
C22 H27 (	-0.00018	-0.47887	6.876136	level4	Public	NA	HMDB025	NA
C11 H23 N	0.000326	1.622788	NA	level4	NA	NA	NA	NA
C10 H19 N	-0.00016	-0.7064	NA	level4	HMDB	NA	HMDB001	Lipids and
C12 H14 N	1.09E-05	0.039193	56.25027	level3	mzCloud	C19530	HMDB001	Benzenoid
C47 H86 (	-0.00151	-1.75767	NA	level4	NA	NA	NA	NA
C20 H24 N	-0.00127	-3.31439	NA	level4	HMDB	NA	HMDB025	NA
C24 H42 (	0.000146	0.330062	NA	level4	NA	NA	NA	NA
C13 H16 (	-8.22E-05	-0.32589	NA	level4	HMDB	NA	HMDB024	NA
C40 H48 N	-0.00031	-0.4962	NA	level4	KEGG	C09085	NA	NA
C31 H48 (	0.000609	1.257319	NA	level4	HMDB	NA	HMDB024	NA
C19 H28 (	-0.00066	-1.53206	15.59485	level4	Public	NA	NA	NA
C17 H22 (	0.000526	1.719665	NA	level4	KEGG	C09603	NA	Terpenoid
C25 H26 N	0.002168	4.857808	NA	level4	HMDB	NA	HMDB024	NA
C27 H42 (	0.002745	4.900151	NA	level4	HMDB	NA	HMDB025	NA
C14 H20 (	21.98199	87183.02	20.90318	level4	Public	NA	NA	NA
C20 H28 (	2.99E-05	0.094411	NA	level4	HMDB	NA	HMDB001	Lipids and
C15 H22 (	0.00129	3.727079	20.25512	level4	Public	NA	NA	NA
C24 H38 (	0.000137	0.312261	NA	level4	NA	NA	NA	NA
C13 H20 (	-5.66E-05	-0.25269	NA	level4	HMDB	NA	HMDB025	NA
C6 H8 O4	0.036573	253.9069	30.92042	level3	Decode	NA	HMDB000	Lipids and
C14 H27 N	-0.00016	-0.65399	NA	level4	NA	NA	NA	NA
C30 H38 (	0.002447	4.512398	4.349811	level4	Public	NA	NA	NA
C9 H15 F (	-0.00088	-4.28524	NA	level4	HMDB	NA	HMDB025	NA
C22 H20 (	-5.56E-05	-0.14627	NA	level4	NA	NA	NA	NA

C21 H34 C	0.00031	0.88466	23.16199	level4	Public	NA	NA	NA
C13 H24 C	-3.52E-05	-0.16613	NA	level4	NA	NA	NA	NA
C19 H38 C	-2.09E-05	-0.06652	NA	level4	NA	NA	NA	NA
C24 H31 F	-0.00169	-4.04897	NA	level4	NA	C14653	NA	NA
C17 H15 C	-0.00034	-1.15104	NA	level4	HMDB	NA	HMDB025	NA
C18 H32 C	-0.0001	-0.35231	82.22208	level2	mzCloud	NA	NA	NA
C20 H28 N	-0.00013	-0.38835	NA	level4	HMDB	NA	HMDB001	Organohe
C19 H32 N	3.52E-05	0.095722	48.143	level4	Public	C07706	HMDB001	Organic ac
C25 H23 F	-0.00025	-0.59363	NA	level4	NA	C13334	NA	Organohe
C16 H22 C	-0.00159	-4.84973	NA	level4	HMDB	NA	HMDB024	NA
C17 H22 C	-5.31E-05	-0.17346	2.960359	level4	Public	NA	NA	NA
C25 H31 C	0.001578	3.259121	NA	level4	HMDB	NA	HMDB001	Lipids and
C10 H12 N	-0.00029	-1.06285	NA	level4	HMDB	NA	HMDB006	Nucleoside
C8 H10 O6	0.000793	3.386797	NA	level4	HMDB	NA	HMDB024	NA
C29 H49 N	-2.49E-05	-0.0507	NA	level4	NA	NA	NA	NA
C22 H25 N	37.95555	95086.61	2.210114	level4	Public	NA	NA	NA
C16 H12 C	0.000616	1.697591	NA	level4	HMDB	NA	HMDB025	NA
C6 H15 N	0.000159	1.354396	57.3581	level3	mzCloud	NA	HMDB003	Organic ni
C21 H24 C	21.98023	61714.06	30.64532	level4	Public	C09308	HMDB003	Phenylpro
C27 H48 C	0.000298	0.659487	NA	level4	HMDB	NA	HMDB026	NA
C6 H10 O4	8.29E-05	0.567657	63.14512	level2	mzCloud	C05282	HMDB001	Organic ac
C12H12N6	-0.00016	-0.31253	NA	level4	NA	C13016	NA	NA
C46 H48 N	0.000717	0.948234	NA	level4	HMDB	NA	HMDB024	NA
C28 H31 F	0.000771	1.79168	NA	level4	HMDB	NA	HMDB025	NA
C25 H31 N	17.02568	38939.74	4.627104	level4	Public	NA	NA	NA
C23 H33 N	0.000395	0.823441	NA	level4	HMDB	NA	HMDB025	NA
C26 H44 N	-0.00157	-2.36881	NA	level4	HMDB	NA	HMDB025	NA
C21 H33 N	0.000542	1.317252	NA	level4	NA	C10341	NA	Alkaloids
C14 H26 C	-6.51E-05	-0.2877	NA	level4	HMDB	NA	HMDB000	Lipids and
C22 H24 F	0.000927	2.102009	NA	level4	HMDB	NA	HMDB024	NA
C24 H42 C	6.11E-05	0.148961	NA	level4	HMDB	NA	HMDB024	NA
C24 H49 C	-0.00196	-3.95276	NA	level4	NA	NA	NA	NA
C12 H22 C	37.95584	110944.3	41.59386	level4	Public	NA	NA	NA
C23 H28 C	-5.54E-05	-0.11244	NA	level4	HMDB	NA	HMDB001	Benzenoid
C32 H28 F	-0.00134	-2.22887	NA	level4	HMDB	NA	HMDB024	NA
C5 H11 N	0.00015	1.127062	NA	level4	HMDB	NA	HMDB024	NA
C4 H9 N	0.000146	2.047836	NA	level4	HMDB	NA	HMDB024	NA
C19 H34 C	-0.00015	-0.51771	NA	level4	HMDB	NA	HMDB024	NA
C25 H26 C	-0.00064	-1.77545	NA	level4	NA	C14959	NA	NA
C8 H10 F2	-0.00028	-1.07019	NA	level4	HMDB	NA	HMDB025	NA
C10 H14	5.23E-05	0.389718	NA	level4	HMDB	NA	HMDB025	NA

C12 H17 N	0.00034	1.136023	NA	level4	HMDB	NA	HMDB001	Homogen	
C21 H30 C	-2.56E-05	-0.06772	NA	level4	HMDB	NA	HMDB026	NA	
C8 H14 O2	0.000113	0.796637	NA	level4	HMDB	NA	HMDB024	NA	
C25 H29 I2	-0.00041	-0.62996	NA	level4	HMDB	NA	HMDB001	Organic ox	
C19 H32 C	17.02604	52512.21	10.69562	level4	Public	NA	NA	NA	
C8 H10 O	0.000117	0.960464	NA	level4	HMDB	NA	HMDB002	Benzenoid	
C21 H23 N	-0.00104	-2.29233	NA	level4	HMDB	NA	HMDB024	NA	
C3 H7 N C	2.27E-05	0.187883	30.68493	level3	BMDB	C00097	HMDB000	Organic ac	
C27 H44 C	0.000107	0.24824	NA	level4	HMDB	NA	HMDB001	Lipids and	
C25 H39 C	0.002075	4.30377	NA	level4	NA	NA	NA	NA	
C25 H32 C	-2.97E-05	-0.06457	NA	level4	HMDB	NA	HMDB024	NA	
C21 H24 F	0.001163	2.599662	NA	level4	HMDB	NA	HMDB025	NA	
C15 H18 C	-0.00017	-0.57434	8.761264	level4	Public	NA	NA	NA	
C14 H19 N	0.000356	1.63979	49.38885	level3	Public	C07475	HMDB003	Organohe	
C20 H22 N	-0.00144	-3.62659	NA	level4	HMDB	NA	HMDB024	NA	
C15 H22 N	-0.0013	-4.23358	5.926807	level4	Public	NA	HMDB025	NA	
C23 H25 N	0.001731	4.128207	NA	level4	NA	C22171	NA	NA	
C23 H20 C	0.001938	4.941205	NA	level4	NA	NA	NA	NA	
C23 H36 C	0.000195	0.477387	NA	level4	NA	NA	NA	NA	
C22 H37 N	0.000216	0.525986	NA	level4	NA	NA	NA	NA	
C20 H30 C	3.95E-05	0.124258	NA	level4	HMDB	NA	HMDB001	Lipids and	
C11 H14 C	0.000109	0.483948	NA	level4	HMDB	NA	HMDB006	Phenylpro	
C28 H47 N	0.000608	1.193585	NA	level4	NA	C11998	NA	NA	
C25 H32 C	21.98368	44664.16	2.54998	level4	Public	NA	NA	NA	
C10 H14 C	0.000182	0.919082	NA	level4	HMDB	NA	HMDB025	NA	
C27 H44 C	0.000202	0.434433	NA	level4	HMDB	NA	HMDB025	NA	
C23 H28 C	-0.00028	-0.69445	NA	level4	HMDB	NA	HMDB025	NA	
C18 H36 N	0.001594	4.235735	NA	level4	HMDB	NA	HMDB024	NA	
C25 H26 C	-0.00083	-1.51426	NA	level4	NA	NA	NA	NA	
C16 H20 C	0.000139	0.481362	9.243179	level4	Public	NA	NA	NA	
C27 H34 N	-0.00156	-3.16397	NA	level4	HMDB	NA	HMDB025	NA	
C12 H13 N	-0.00126	-4.34549	NA	level4	HMDB	NA	HMDB025	NA	
C8 H17 N	2.12E-05	0.11114	NA	level4	HMDB	NA	HMDB025	NA	
C21 H32 C	0.000132	0.31981	NA	level4	HMDB	NA	HMDB024	NA	
C23 H23 N	-0.00086	-2.50526	NA	level4	HMDB	NA	HMDB025	NA	
C15 H21 N	-0.00011	-0.34506	NA	level4	HMDB	NA	HMDB026	NA	
C14 H16 C	0.000344	1.590988	NA	level4	NA	NA	NA	NA	
C30 H41 F	-0.00092	-1.73564	NA	level4	HMDB	NA	HMDB024	NA	
C27 H37 F	-0.00201	-4.87407	NA	level4	NA	NA	NA	NA	
C32 H57 C	0.000659	0.992203	NA	level4	NA	NA	NA	NA	
C27 H38 C	0.00066	1.304235	NA	level4	HMDB	NA	HMDB001	Lipids and	
C25 H30 N	-0.00073	-1.97278	NA	level4	HMDB	NA	HMDB001	Benzenoid	
C30 H50 N	-0.00224	-3.9508	NA	level4	HMDB	NA	HMDB001	Lipids and	
C24 H42 N	0.002216	4.342462	NA	level4	HMDB	NA	HMDB025	NA	
C9 H11 N	0.000298	1.509721	NA	level4	HMDB	NA	HMDB000	Organic ac	
C60 H86 C	-0.00145	-1.60276	NA	level4	NA	NA	NA	NA	
C26 H29 N	0.000337	0.75439	1.362692	level4	Public	NA	NA	NA	
C30 H59 N	0.000476	0.98892	NA	level4	HMDB	NA	HMDB000	Lipids and	
C30 H33 N	-0.00135	-2.43997	NA	level4	HMDB	NA	HMDB024	NA	
C14 H26 C	-3.42E-05	-0.11794	NA	level4	NA	NA	NA	NA	
C37 H47 N	-0.00012	-0.20829	NA	level4	NA	C20600	NA	Fungal tox	
C30 H31 C	-0.002	-3.68295	NA	level4	HMDB	NA	HMDB024	NA	
C38 H77 N	0.000606	1.017262	NA	level4	HMDB	NA	HMDB001	Lipids and	
C33 H42 C	0.002333	3.143079	NA	level4	NA	NA	NA	NA	

C31 H43 N	0.002557	4.723531	NA	level4	KEGG	C08658	NA	Alkaloids
C3 H6 N6	0.000419	3.322003	15.63374	level4	Public	C08737	HMDB004	Organohe
C24 H29 N	0.001819	4.032235	NA	level4	HMDB	NA	HMDB001	Organic ac
C10 H8 O	0.000196	1.3583	47.12316	level3	mzCloud	C11714	HMDB001	Benzenoid
C25 H34 C	7.24E-05	0.189387	NA	level4	HMDB	NA	HMDB025	NA
C28 H32 C	0.000356	0.569951	65.39092	level2	Public	NA	NA	NA
C23 H22 C	0.001003	2.190222	NA	level4	NA	NA	NA	NA
C20 H30 C	-18.0106	-51428.1	63.92546	level2	mzCloud	C20214	NA	Terpenoid
C18 H22 N	-0.00057	-1.82701	NA	level4	HMDB	NA	HMDB025	NA
C28 H40 C	-0.00019	-0.43739	NA	level4	HMDB	NA	HMDB024	NA
C32 H52 C	0.000418	0.721029	10.0167	level4	Public	NA	NA	NA
C32 H48 C	0.000381	0.679145	NA	level4	NA	C20787	NA	Terpenoid
C16 H21 N	-0.00034	-1.11211	NA	level4	NA	C10855	NA	NA
C13 H14 N	-0.0002	-0.81342	96.20018	level1	BMDB	NA	NA	NA
C24 H32 C	-0.00016	-0.34342	NA	level4	HMDB	NA	HMDB025	NA
C21 H38 C	0.000664	1.332914	NA	level4	NA	NA	NA	NA
C29 H48 C	0.000102	0.248085	NA	level4	HMDB	NA	HMDB000	Lipids and
C43 H83 C	0.002314	2.927546	NA	level4	NA	NA	NA	NA
C23 H32 C	21.98251	52312.56	3.513881	level4	Public	NA	NA	NA
C27 H40 C	0.000172	0.386123	NA	level4	HMDB	NA	HMDB024	NA
C23 H34 C	17.02833	30949.93	5.587821	level4	Public	NA	HMDB025	NA
C23 H28 N	-0.00091	-1.89685	NA	level4	HMDB	NA	HMDB025	NA
C29 H28 N	0.001784	2.933343	NA	level4	HMDB	NA	HMDB025	NA
C19 H38 N	-0.00015	-0.29605	NA	level4	NA	C18001	NA	NA
C15 H21 N	-0.00159	-4.73345	NA	level4	HMDB	NA	HMDB025	NA

C12 H22 C	0.000475	2.606824	NA	level4	NA	NA	NA	NA
C20 H29 N	0.000245	0.674412	NA	level4	HMDB	NA	HMDB025	NA
C8 H15 N	3.73E-05	0.197465	NA	level4	HMDB	NA	HMDB024	Organic ac
C25 H28 N	-0.0009	-1.90581	NA	level4	HMDB	NA	HMDB025	NA
C16 H29 N	4.14E-05	0.155093	NA	level4	NA	C20034	NA	Venoms
C12 H12 C	0.000169	0.76649	NA	level4	HMDB	NA	HMDB024	NA
C9 H17 N	9.63E-05	0.40972	NA	level4	HMDB	NA	HMDB026	NA
C18 H36 C	0.000645	2.039121	NA	level4	NA	NA	NA	NA
C17 H37 A	0.001194	3.595108	NA	level4	NA	NA	NA	NA
C37 H68 C	5.65E-05	0.095286	NA	level4	HMDB	NA	HMDB000	Lipids and

C20 H33 N	0.000309	0.649788	NA	level4	NA	C17375	NA	NA	
C21 H36 N	-0.00234	-4.36282	NA	level4	HMDB	NA	HMDB025	NA	
C30 H46 C	0.000282	0.598629	NA	level4	HMDB	NA	HMDB001	Lipids and	
C11 H10 C	0.000224	1.288902	NA	level4	KEGG	C07126	NA	NA	
C17 H22 C	1.50E-05	0.054531	7.467459	level4	Public	NA	NA	NA	
C22 H28 F	-0.00088	-1.82098	NA	level4	HMDB	NA	HMDB025	NA	
C11 H14 N	0.000153	0.567243	50	level3	Masslist	NA	HMDB024	Organic ac	
C33 H36 N	0.001027	1.860379	NA	level4	NA	C10000	NA	NA	
C40 H77 N	0.003366	4.318399	NA	level4	NA	NA	NA	NA	
C27 H50 N	-0.00227	-3.93931	NA	level4	HMDB	NA	HMDB025	NA	
C24 H28 C	-21.9833	-43260.8	3.016043	level4	Public	NA	NA	NA	
C30 H46 C	-0.00045	-0.88795	NA	level4	HMDB	NA	HMDB025	NA	
C11 H14 N	-0.0007	-2.75889	NA	level4	HMDB	NA	HMDB025	NA	
C19 H20 C	0.001673	4.631805	4.962862	level4	Public	NA	HMDB001	Benzenoid	
C24 H27 N	0.001873	4.324233	NA	level4	HMDB	NA	HMDB024	NA	
C21 H27 N	0.001921	4.164422	NA	level4	HMDB	NA	HMDB006	Benzenoid	
C32 H52 C	0.000164	0.274734	NA	level4	NA	NA	NA	NA	
C21 H32 C	1.35E-05	0.044952	NA	level4	HMDB	NA	HMDB025	NA	
C28 H48 C	0.000106	0.266003	NA	level4	HMDB	NA	HMDB024	NA	
C34 H50 C	21.98511	33806.07	1.477555	level4	Public	NA	NA	NA	
C33 H30 N	0.001998	3.739818	NA	level4	HMDB	NA	HMDB024	NA	
C16 H18 N	-0.00013	-0.46097	NA	level4	HMDB	NA	HMDB025	NA	
C10 H13 F	-0.00012	-0.33724	NA	level4	HMDB	NA	HMDB001	Nucleoside	
C21 H29 B	0.002079	4.901489	NA	level4	NA	NA	NA	NA	
C20 H24 N	-0.00099	-2.76711	NA	level4	HMDB	NA	HMDB025	NA	
UnkownFc	313.1509	Inf	39.46644	level4	Public	NA	NA	NA	
C20 H38 C	-0.00013	-0.42417	NA	level4	HMDB	NA	HMDB025	NA	
C15 H17 N	-18.0107	-61868.8	65.96559	level2	mzCloud	NA	NA	NA	
C8 H8 O2	0.000108	0.796628	15.17365	level3	BMDB	C00548	HMDB004	Benzenoid	
C26 H40 C	17.02653	36673.18	21.0917	level4	Public	NA	NA	NA	
C22 H34 C	37.95762	92527.56	6.674184	level4	Public	NA	NA	NA	
C15 H29 N	-0.00013	-0.46457	NA	level4	HMDB	NA	HMDB025	NA	
C16 H16 N	-0.00037	-1.38756	NA	level4	HMDB	NA	HMDB024	NA	
C25 H27 N	0.000151	0.336678	NA	level4	HMDB	NA	HMDB025	NA	
C21 H20 C	0.000299	0.692988	17.26511	level4	Public	NA	HMDB030	NA	
C15 H29 N	0.001896	4.948572	NA	level4	NA	C21650	NA	NA	
C24 H36 C	0.000164	0.405514	NA	level4	HMDB	NA	HMDB001	Organohe	
C12 H9 Cl	0.000184	0.902074	NA	level4	NA	C14755	NA	NA	
C16 H28 C	-2.81E-05	-0.08454	43.15024	level3	Public	NA	NA	NA	
C20 H25 N	-0.0001	-0.27445	NA	level4	HMDB	NA	HMDB025	NA	
C12 H14 N	-0.001	-4.64783	NA	level4	HMDB	NA	HMDB024	NA	
C14 H18 N	-0.0001	-0.36693	NA	level4	HMDB	NA	HMDB024	NA	
C27 H36 C	17.027	36055.35	29.19387	level4	Public	NA	NA	NA	
C25 H40 C	-0.00018	-0.38292	NA	level4	HMDB	NA	HMDB001	Benzenoid	
C21 H12 C	-0.00136	-3.22274	NA	level4	HMDB	NA	HMDB024	NA	
C15 H24 N	0.003251	12.30493	42.32513	level3	mzCloud	C10749	NA	Alkaloids	
C28 H41 N	-0.0016	-3.18551	NA	level4	NA	NA	NA	NA	
C27 H46 N	-0.00052	-0.97943	NA	level4	NA	NA	NA	NA	
C24 H32 C	0.003386	7.555056	62.63637	level2	mzCloud	C11237	NA	ST Sterol	
C9 H14 N4	0.000278	1.231207	10.37205	level4	BMDB	C00386	HMDB000	Organic ac	
C25 H42 C	-0.00039	-0.89505	NA	level4	NA	NA	NA	NA	
C25 H38 C	7.65E-05	0.19011	36.49542	level4	Public	NA	NA	NA	

C18 H20 (	-0.00153	-4.00278	NA	level4	HMDB	NA	HMDB025	NA
C27 H42 (	-0.00049	-0.89594	NA	level4	HMDB	NA	HMDB001	Lipids and
C15 H20 (	0.000111	0.476471	39.64676	level4	Public	NA	NA	NA
C20 H19 N	37.95733	107489.4	5.864675	level4	Public	NA	NA	NA
C31 H41 F	-0.00166	-2.83	NA	level4	HMDB	NA	HMDB024	NA
C45 H91 N	7.18E-05	0.091249	NA	level4	HMDB	NA	HMDB001	Lipids and
C8 H13 N3	0.000221	0.954444	NA	level4	HMDB	NA	HMDB024	NA
C42 H80 N	-0.00134	-1.76241	NA	level4	HMDB	NA	HMDB025	NA
C22 H28 (	-0.00159	-3.74062	NA	level4	HMDB	NA	HMDB001	Lipids and
C15 H13 N	1.98E-05	0.077745	NA	level4	HMDB	NA	HMDB024	NA
C22 H27 N	-0.00131	-3.05121	NA	level4	HMDB	NA	HMDB025	NA
C27 H24 F	0.002092	4.225275	NA	level4	HMDB	NA	HMDB025	NA
C9 H9 N (	0.000227	1.076431	34.82835	level4	Public	NA	NA	NA
C25 H32 (	21.98346	49488.35	23.75682	level4	Public	NA	NA	NA
C19 H12 (	-0.00075	-2.24217	NA	level4	HMDB	NA	HMDB001	Phenylpro
C11 H17 N	-0.00028	-0.94985	NA	level4	HMDB	NA	HMDB006	Organic ac
C22 H23 N	0.003267	7.907788	51.00541	level3	mzCloud	NA	HMDB003	Alkaloids a
C20 H24 (	21.98016	63865.65	9.647917	level4	Public	NA	NA	NA
C17 H22 (	0.000114	0.356865	NA	level4	NA	C18476	NA	NA
C25 H41 N	-0.00053	-1.05486	NA	level4	HMDB	NA	HMDB024	NA
C19 H11 F	-0.0017	-4.3017	NA	level4	HMDB	NA	HMDB025	NA
C33 H45 N	-0.00274	-4.57783	NA	level4	KEGG	C08677	NA	Alkaloids
C9 H15 N3	0.000127	0.554954	NA	level4	HMDB	NA	HMDB024	NA
C14 H10	0.000153	0.860481	NA	level4	HMDB	NA	HMDB024	NA
C7 H5 N (	8.27E-05	0.494976	86.56149	level2	mzCloud	C03722	HMDB000	Organohe
C21 H32 (	0.000172	0.471473	NA	level4	HMDB	NA	HMDB025	NA
C32 H65 N	0.000693	1.31307	NA	level4	NA	NA	NA	NA
C16 H23 N	9.89E-05	0.403194	NA	level4	HMDB	NA	HMDB025	NA
C14 H16 (	-2.69E-05	-0.07806	NA	level4	HMDB	NA	HMDB024	NA
C30 H34 (	-0.00021	-0.45068	NA	level4	NA	NA	NA	NA
C19 H27 N	-5.24E-05	-0.1269	NA	level4	NA	NA	NA	NA
C7 H15 N	0.000319	2.197401	87.68453	level1	Decode	NA	HMDB009	Organic ac
C19 H29 N	-0.00143	-3.96854	NA	level4	HMDB	NA	HMDB025	NA
C22 H29 N	0.000289	0.62485	NA	level4	HMDB	NA	HMDB001	Organic ac
C32 H51 N	0.000957	1.421008	NA	level4	NA	NA	NA	NA
C11 H16 N	-2.76E-05	-0.10762	NA	level4	HMDB	NA	HMDB025	NA
C27 H42 (	-0.00023	-0.44278	NA	level4	NA	NA	NA	NA
C32 H28 (	-0.00163	-3.04354	NA	level4	HMDB	NA	HMDB025	NA
C9 H13 N	0.000264	1.745624	18.47454	level4	Public	NA	HMDB024	NA
C12 H14 (	0.000153	0.876838	NA	level4	NA	NA	NA	NA
C33 H42 N	0.000282	0.477163	NA	level4	HMDB	NA	HMDB000	Organohe
C18 H20 N	3.50E-05	0.112157	NA	level4	HMDB	NA	HMDB026	NA
C10 H12 F	0.00075	2.964846	NA	level4	HMDB	NA	HMDB024	NA
C28 H50 N	-0.00249	-4.57921	NA	level4	HMDB	NA	HMDB001	Lipids and
C21 H34 (	18.01042	56591.01	4.037314	level4	Public	NA	NA	NA
C20 H25 N	0.000223	0.465372	NA	level4	NA	C21620	NA	NA
C31 H45 N	0.002253	3.835952	NA	level4	HMDB	NA	HMDB025	NA
C23 H34 N	-0.00169	-3.8796	NA	level4	NA	C21542	NA	NA
C27 H36 F	-0.00231	-4.67681	NA	level4	HMDB	NA	HMDB025	NA
C18 H18 (	0.000359	1.04733	NA	level4	HMDB	NA	HMDB024	NA
C2 H4 N4	-6.56E-05	-0.56581	NA	level4	HMDB	NA	HMDB024	NA
C22 H40 (	2.02E-05	0.050385	NA	level4	NA	NA	NA	NA
C7 H8 Cl N	-1.06E-05	-0.03585	NA	level4	HMDB	NA	HMDB000	Organohe
C28 H50 N	0.000774	1.424683	NA	level4	HMDB	NA	HMDB001	Lipids and
C23 H41 N	9.79E-05	0.237939	NA	level4	HMDB	NA	HMDB001	Lipids and
C35 H54 (	-0.00238	-3.84632	11.13472	level4	Public	NA	NA	NA
C28 H46 (	-0.00031	-0.58809	19.07359	level4	Public	NA	NA	NA
H2 Sn	-0.00046	-3.76274	NA	level4	NA	C19692	NA	NA

C40 H50 C	0.001972	3.408913	NA	level4	NA	NA	NA	NA
C17 H12 C	-0.00069	-2.60325	NA	level4	HMDB	NA	HMDB025	NA
C30 H50 C	0.000607	1.198868	NA	level4	NA	NA	NA	NA
C28 H46 C	8.16E-05	0.196819	NA	level4	NA	NA	NA	NA
C6 H6 O3	-0.00088	-7.02007	57.86551	level3	mzCloud	C02752	NA	NA
C20 H19 N	0.000748	2.359195	NA	level4	HMDB	NA	HMDB024	NA
C24 H36 C	0.000133	0.329692	NA	level4	HMDB	NA	HMDB024	NA
C26 H35 F	-0.00047	-1.0518	NA	level4	HMDB	NA	HMDB025	NA
C14 H18 N	-0.00085	-2.92738	NA	level4	HMDB	NA	HMDB001	Benzenoid
C8 H14 N3	0.001011	2.155341	NA	level4	HMDB	NA	HMDB006	Nucleoside
C11 H13 N	-0.00131	-4.99251	NA	level4	HMDB	NA	HMDB025	NA
C11 H13 N	-0.00019	-0.71122	35.30958	level3	mzCloud	C07318	HMDB001	Benzenoid
C20 H24 C	-3.15E-05	-0.10625	67.89789	level2	mzCloud	C07534	HMDB000	Lipids and
C25 H24 F	-0.00126	-2.76442	NA	level4	HMDB	NA	HMDB025	NA
C29 H34 C	0.000418	0.878861	NA	level4	HMDB	NA	HMDB024	NA
C13 H20 N	0.000597	1.842601	NA	level4	HMDB	NA	HMDB001	Organic ac
C12 H14 N	-0.00019	-0.55102	NA	level4	HMDB	NA	HMDB025	NA
C30 H39 N	0.002573	4.752757	NA	level4	NA	NA	NA	NA
C31 H52 C	21.98062	46530.5	21.82768	level4	Public	NA	HMDB003	Lipids and
C16 H20 C	-3.40E-05	-0.11655	28.91011	level4	Public	NA	HMDB003	Phenylpro
C10 H19 C	0.001354	4.103783	NA	level4	HMDB	NA	HMDB001	Lipids and
C6 H5 N C	8.96E-05	0.728173	NA	level4	HMDB	NA	HMDB000	Organohe
C15 H22 C	-18.0106	-63834.1	75.13794	level2	mzCloud	C09538	NA	Terpenoid
C33 H56 C	9.20E-05	0.167716	NA	level4	HMDB	NA	HMDB025	NA
C22 H25 C	-0.00156	-3.61025	NA	level4	HMDB	NA	HMDB024	NA
C39 H63 N	0.003592	4.52733	NA	level4	NA	C15762	NA	NA
C17 H26 C	3.34E-05	0.127466	24.51283	level4	Public	NA	NA	NA
C37 H66 C	0.000661	1.119056	NA	level4	HMDB	NA	HMDB000	Lipids and
C18 H32 N	0.000154	0.384609	14.60425	level4	Public	NA	NA	NA
C25 H48 N	-0.00023	-0.46238	NA	level4	HMDB	NA	HMDB025	NA
C21 H28 C	-6.53E-05	-0.1749	NA	level4	HMDB	NA	HMDB025	NA
C8 H9 N C	0.000203	1.343448	NA	level4	HMDB	NA	HMDB024	NA
C12 H14 N	-3.68E-05	-0.13839	NA	level4	HMDB	NA	HMDB025	NA
C21 H38 C	-9.30E-05	-0.26264	50.52134	level4	Public	NA	HMDB024	NA
C15 H10 C	0.001259	4.560056	NA	level4	HMDB	NA	HMDB025	NA
C18 H21 F	0.001154	2.81337	NA	level4	HMDB	NA	HMDB024	NA
C18 H24 C	-0.00427	-15.7056	35.75221	level3	mzCloud	C00951	HMDB000	Lipids and
C27 H50 N	0.000778	1.380778	NA	level4	NA	NA	NA	NA
C10 H18 N	-0.00045	-1.63089	NA	level4	KEGG	C03296	NA	NA
C21 H43 N	-0.00012	-0.27951	NA	level4	HMDB	NA	HMDB024	NA
C26 H28 N	0.000435	0.970259	NA	level4	NA	C12183	NA	NA
C26 H33 N	0.000198	0.468987	NA	level4	NA	C12335	NA	NA
C26 H32 C	35.03727	85827.27	10.62971	level4	Public	NA	NA	NA
C17 H32 N	-0.00035	-0.90164	54.16519	level4	Public	NA	NA	NA
C18 H37 N	-0.00016	-0.50896	NA	level4	NA	NA	NA	NA
C10 H14 C	0.000264	1.450998	NA	level4	HMDB	NA	HMDB024	NA
C33 H52 C	0.000389	0.675264	NA	level4	NA	NA	NA	NA
C24 H40 C	46.00551	117273.5	89.63174	level2	mzCloud	NA	NA	NA
C9 H10 N4	-0.00033	-1.38537	NA	level4	HMDB	NA	HMDB025	NA
C20 H33 N	-9.37E-05	-0.2445	48.32223	level4	Public	NA	NA	NA
C8 H6 N2	0.000112	0.764326	NA	level4	HMDB	NA	HMDB024	NA
C10 H8 O4	1.81E-05	0.09422	40.3466	level3	Public	NA	NA	NA
C34 H67 N	0.000431	0.777948	NA	level4	NA	NA	NA	NA
C10 H11 F	0.000587	2.311863	NA	level4	HMDB	NA	HMDB024	NA

C32 H46 (	21.98497	37243.15	3.849635	level4	Public	NA	NA	NA
C38 H56 (	-0.00228	-3.55897	NA	level4	NA	NA	NA	NA
C17 H29 N	-1.60E-05	-0.05127	NA	level4	HMDB	NA	HMDB001	Lipids and
C51 H94 N	0.004682	4.725943	NA	level4	NA	NA	NA	NA
C25 H41 N	7.78E-05	0.185608	NA	level4	NA	C12157	NA	NA
C31 H61 (	0.000548	0.834905	NA	level4	NA	NA	NA	NA
C40 H56 N	-0.00295	-4.58497	NA	level4	HMDB	NA	HMDB024	NA
C8 H17 N3	-5.80E-05	-0.31007	NA	level4	HMDB	NA	HMDB025	NA
C17 H24 (	-0.00028	-0.90193	NA	level4	HMDB	NA	HMDB024	NA
C26 H25 (	0.002025	4.353121	NA	level4	HMDB	NA	HMDB025	NA
C24 H38 (	-7.96E-05	-0.2039	95.59721	level1	BMDB2	NA	HMDB000	Lipids and
C32 H36 N	21.98361	43250.66	20.14669	level4	Public	NA	NA	NA
C27 H48 (	-0.00195	-3.90617	NA	level4	NA	NA	NA	NA
C21 H33 N	-0.00049	-1.35061	NA	level4	HMDB	NA	HMDB025	NA
C16 H20 N	-0.00044	-1.38113	NA	level4	HMDB	NA	HMDB024	NA
C22 H28 (	-9.65E-05	-0.27085	NA	level4	HMDB	NA	HMDB025	NA
C37 H64 (	-0.00092	-1.60799	NA	level4	NA	NA	NA	NA
C14 H14 (	4.85E-05	0.226352	NA	level4	HMDB	NA	HMDB024	NA
C47 H66 (	8.42E-05	0.100455	NA	level4	NA	C12070	NA	NA
C17 H20 N	0.000206	0.687466	NA	level4	HMDB	NA	HMDB024	NA
C23 H43 N	6.15E-05	0.168469	NA	level4	NA	NA	NA	NA
C19 H18 (	0.001323	4.04556	NA	level4	HMDB	NA	HMDB025	NA
C7 H5 N (	0.00018	1.077505	NA	level4	HMDB	NA	HMDB024	NA
C25 H30 (	21.98355	38556.9	3.139902	level4	Public	NA	NA	NA
C27 H42 (	-0.00158	-3.40752	NA	level4	NA	NA	NA	NA
C21 H14 F	0.001011	2.454328	NA	level4	HMDB	NA	HMDB025	NA
C10 H10 (	0.000144	0.890726	NA	level4	HMDB	NA	HMDB025	NA
C21 H42 N	-0.003	-4.62931	NA	level4	KEGG	C04526	NA	NA
C16 H22 (	0.000265	0.819422	NA	level4	HMDB	NA	HMDB025	NA
C17 H28 (	17.02635	57482.85	19.20509	level4	Public	NA	NA	NA
C43 H82 N	0.003173	3.948945	NA	level4	NA	NA	NA	NA
C32 H56 (	-0.00189	-2.98105	NA	level4	NA	NA	NA	NA
C22 H32 (	5.98E-05	0.135743	NA	level4	HMDB	NA	HMDB025	NA
C15 H14 N	-2.68E-05	-0.1053	NA	level4	HMDB	NA	HMDB024	NA
C20 H32 (	-8.77E-05	-0.23822	65.73618	level2	mzCloud	C05964	HMDB000	Lipids and
C7 H10 O2	8.95E-05	0.70961	NA	level4	HMDB	NA	HMDB024	NA
C23 H38 (	-2.19E-05	-0.05783	63.45035	level2	mzCloud	C13856	HMDB000	Lipids and

C16 H24 N	-0.00027	-0.92302	NA	level4	HMDB	NA	HMDB024	NA
C30 H47 N	-0.00317	-4.9441	NA	level4	NA	NA	NA	NA
C24 H31 N	5.51E-05	0.128395	NA	level4	HMDB	NA	HMDB025	NA
C24 H40 C	46.00558	122258.5	79.6426	level2	mzCloud	C03990	HMDB000	Lipids and
C13 H14 N	-0.00121	-4.98942	NA	level4	HMDB	NA	HMDB024	NA
C20 H28 F	-0.00121	-3.59218	NA	level4	NA	C14910	NA	NA
C26 H26 N	0.002172	4.911048	NA	level4	HMDB	NA	HMDB025	NA
C6 H11 N	0.000183	1.139279	NA	level4	HMDB	NA	HMDB025	NA
C10 H16 C	0.000321	1.383984	NA	level4	HMDB	NA	HMDB024	NA
C22 H31 C	0.000722	1.509039	NA	level4	HMDB	NA	HMDB025	NA
C11 H22 N	0.005542	17.15083	31.52675	level3	mzCloud	C06457	NA	NA
C20 H24 C	9.57E-05	0.256471	NA	level4	HMDB	NA	HMDB025	NA
C16 H28 C	-0.00022	-0.81057	NA	level4	NA	NA	NA	NA
C26 H39 N	-0.00022	-0.44309	NA	level4	HMDB	NA	HMDB025	NA
C7 H14 O2	0.000121	0.931066	NA	level4	NA	C12290	NA	Lipids and
C8 H14 N2	0.000205	0.940508	NA	level4	NA	C20958	NA	Organic ac
C26 H27 C	-0.0005	-1.1986	NA	level4	HMDB	NA	HMDB025	NA
C16 H27 N	9.29E-05	0.312509	33.02893	level4	Public	NA	NA	NA
C29 H36 C	0.000498	1.037851	NA	level4	HMDB	NA	HMDB025	NA
C41 H76 N	0.000338	0.455744	NA	level4	NA	NA	NA	NA
C7 H12 N2	0.000262	1.522738	52.51489	level3	mzCloud	NA	HMDB000	Organic ac
C28 H42 C	0.000608	1.240358	NA	level4	HMDB	NA	HMDB024	NA
C21 H34 C	6.21E-06	0.013914	38.62172	level4	Public	NA	NA	NA
C24 H40 C	21.98207	56034.87	69.69759	level1	BMDB2	NA	HMDB000	Lipids and
C15 H20 C	0.000124	0.402071	NA	level4	HMDB	NA	HMDB025	NA
C44 H72 C	-0.00355	-4.48008	NA	level4	KEGG	C06764	NA	Antibiotics
C25 H40 C	6.88E-05	0.170135	NA	level4	HMDB	NA	HMDB024	NA
C20 H37 N	0.000119	0.366715	NA	level4	HMDB	NA	HMDB024	NA
C21 H27 N	-0.01543	-49.8974	18.95468	level4	Public	C07163	HMDB001	Benzenoid
C18 H34 N	0.000263	0.647123	74.09606	level2	Public	NA	HMDB025	NA
C28 H33 N	-0.00137	-2.73929	NA	level4	HMDB	NA	HMDB024	NA
C18 H19 C	0.0008	2.423201	NA	level4	HMDB	NA	HMDB025	NA
C9 H9 N C	-2.85E-05	-0.14607	69.66186	level2	mzCloud	NA	HMDB000	Organic ac
C35 H40 N	-0.00016	-0.25579	NA	level4	NA	C18098	NA	NA
C17 H31 N	-0.00015	-0.44685	NA	level4	NA	NA	NA	NA
C33 H59 C	-0.00179	-2.76185	NA	level4	NA	NA	NA	NA
C14 H13 C	0.001594	4.859834	10.0188	level4	Public	NA	HMDB025	NA
C25 H44 C	1.84E-05	0.035564	NA	level4	NA	C17435	NA	PR Prenol
C26 H38 C	3.13E-05	0.082	NA	level4	HMDB	NA	HMDB024	NA
C22 H26 F	-0.0013	-2.9671	NA	level4	HMDB	NA	HMDB001	Organohe
C28 H40 C	5.54E-05	0.135774	NA	level4	NA	NA	NA	NA
C32 H40 C	-0.00197	-2.90041	NA	level4	NA	NA	NA	NA
C16 H31 N	-0.00038	-0.83309	NA	level4	NA	C17940	NA	NA
C32 H54 C	0.001619	2.63578	NA	level4	NA	NA	NA	NA
C14 H18 C	-5.00E-05	-0.18798	NA	level4	NA	NA	NA	NA
C14 H16 C	9.29E-05	0.374372	NA	level4	HMDB	NA	HMDB025	NA
C21 H28 C	21.98327	51826.15	3.865417	level4	Public	NA	NA	NA
C18 H20 C	37.95498	141545.8	3.150793	level4	Public	NA	NA	NA
C28 H36 C	21.98423	45398.88	12.46398	level4	Public	NA	NA	NA
C27 H40 C	-0.00017	-0.35331	NA	level4	NA	NA	NA	NA
C9 H8 O	0.00015	1.135386	NA	level4	HMDB	NA	HMDB024	NA
C18 H30 C	-18.0106	-55211.9	61.85706	level2	mzCloud	NA	NA	NA

C26 H27 N	0.001926	3.874447	NA	level4	HMDB	NA	HMDB025	NA
C20 H40 O	17.02651	51863	2.025259	level4	Public	NA	NA	NA
C25 H24 O	0.001029	2.276831	NA	level4	NA	NA	NA	NA
C19 H22 N	-0.00131	-4.28026	NA	level4	HMDB	NA	HMDB025	NA
C12 H14 O	0.000189	0.992392	NA	level4	HMDB	NA	HMDB025	NA
C18 H23 B	0.001156	3.302441	NA	level4	NA	NA	NA	NA
C21 H30 O	0.000135	0.429735	NA	level4	HMDB	NA	HMDB024	NA
C12 H22 O	-1.20E-05	-0.06039	20.4646	level4	Public	C12298	HMDB003	Lipids and
C13 H11 N	6.17E-05	0.269269	NA	level4	HMDB	NA	HMDB024	NA
C20 H28 O	9.39E-05	0.249003	NA	level4	NA	C18603	NA	NA
C27 H44 O	-0.00028	-0.56122	NA	level4	HMDB	NA	HMDB001	Lipids and
C15 H18 N	-9.69E-05	-0.30088	NA	level4	NA	C19022	NA	NA
C17 H26 O	21.98163	74720.92	38.9246	level4	Public	NA	NA	NA
C25 H43 N	0.000164	0.388125	NA	level4	HMDB	NA	HMDB000	Lipids and
C12 H22 O	0.000158	0.798439	NA	level4	NA	NA	NA	NA
C5 H6 N2	0.000148	1.348296	NA	level4	HMDB	NA	HMDB006	Organohe
C10 H12	6.72E-05	0.508735	NA	level4	HMDB	NA	HMDB024	NA
C13 H23 N	8.19E-05	0.318417	NA	level4	HMDB	NA	HMDB001	Lipids and
C10 H12 N	0.000346	1.801763	91.07271	level1	Decode	NA	HMDB000	Organohe
C32 H46 O	0.002539	4.682541	38.36502	level3	mzCloud	NA	NA	NA
C27 H40 O	0.001365	2.68568	NA	level4	NA	NA	NA	NA
C14 H22 O	-0.0003	-1.0102	NA	level4	HMDB	NA	HMDB001	Benzenoid
C13 H22 O	0.000175	0.834603	NA	level4	HMDB	NA	HMDB025	NA
C26 H50 N	0.000307	0.590804	NA	level4	HMDB	NA	HMDB001	Lipids and
C46 H62 N	-0.00353	-4.17468	NA	level4	HMDB	NA	HMDB001	Phenylpro
C21 H34 O	2.62E-06	0.007824	NA	level4	HMDB	NA	HMDB000	Lipids and
C8 H24 N4	-0.00068	-2.36561	NA	level4	HMDB	NA	HMDB025	NA
C8 H10 O3	17.02678	110518.3	24.72704	level4	Public	NA	NA	NA
C23 H30 O	0.001939	3.771361	0.575213	level4	Public	NA	NA	NA
C14 H19 N	-0.00155	-4.59712	NA	level4	HMDB	NA	HMDB024	NA
C21 H21 O	6.82E-05	0.141848	NA	level4	NA	NA	NA	NA
C49 H63 N	-0.00099	-1.05978	NA	level4	HMDB	NA	HMDB024	NA
C30 H46 O	-0.00066	-1.23787	NA	level4	NA	NA	NA	NA
C22 H37 N	-0.00014	-0.31262	NA	level4	HMDB	NA	HMDB025	NA
C27 H44 O	4.88E-05	0.126848	96.40264	level2	mzCloud	C00599	HMDB000	Lipids and
C19 H37 N	0.001494	3.463884	NA	level4	NA	C17996	NA	NA
C20 H28 O	-0.00011	-0.36299	39.6689	level3	mzCloud	C09066	HMDB003	Organohe
C15 H22 O	-0.00122	-4.57731	14.95237	level4	Public	NA	NA	NA
C19 H28 O	-18.0106	-62491.4	36.81851	level3	mzCloud	C01227	HMDB000	Lipids and
C15 H20 O	0.001113	3.390611	6.447631	level4	Public	NA	NA	NA
C28 H26 N	0.002118	4.392477	NA	level4	HMDB	NA	HMDB024	NA
C25 H27 N	-8.65E-06	-0.02073	NA	level4	HMDB	NA	HMDB024	NA
C20 H34 O	17.02644	52836	38.56654	level4	Public	NA	NA	NA

C8 H14 O4	-18.0103	-103455	79.83271	level1	BMDB	C08278	HMDB000	Lipids and
C27 H38 F	0.000236	0.449959	NA	level4	HMDB	NA	HMDB025	NA
C21 H34 C	1.23E-05	0.040647	NA	level4	HMDB	NA	HMDB025	NA
C4 H5 F3 (	0.000153	1.213648	NA	level4	HMDB	NA	HMDB025	NA
C10 H12 C	0.000175	1.181645	78.97287	level2	mzCloud	C06577	HMDB000	Lipids and
C7 H13 O5	-5.12E-05	-0.21335	NA	level4	NA	C18745	NA	NA
C13 H16 N	-0.00143	-4.4756	NA	level4	HMDB	NA	HMDB025	NA
C8 H14 N4	-0.00046	-2.13823	NA	level4	HMDB	NA	HMDB025	NA
C26 H28 C	-0.00063	-1.14405	15.97728	level4	Public	NA	NA	NA
C17 H18 N	-0.00026	-0.75563	NA	level4	HMDB	NA	HMDB001	Organohe
C10 H19 N	0.000138	0.591805	NA	level4	HMDB	NA	HMDB024	NA
C25 H34 C	-6.40E-06	-0.01748	NA	level4	HMDB	NA	HMDB025	NA
C10 H18 C	9.83E-05	0.57806	82.75352	level1	Decode	NA	HMDB003	Lipids and
C23 H36 C	0.000251	0.666149	NA	level4	NA	C15346	NA	NA
C21 H30 C	-0.0001	-0.34495	NA	level4	HMDB	NA	HMDB025	NA
C33 H36 N	0.000637	1.090514	89.32436	level2	mzCloud	C00486	HMDB000	Organohe
C14 H28 C	-1.20E-05	-0.04918	NA	level4	NA	NA	NA	NA
C24 H40 C	-7.79E-05	-0.16502	NA	level4	HMDB	NA	HMDB000	Lipids and
C15 H11 N	-0.00131	-3.92826	NA	level4	HMDB	NA	HMDB024	NA
C48 H89 C	0.002258	2.635527	NA	level4	NA	NA	NA	NA
C18 H36 C	-0.00023	-0.7611	NA	level4	HMDB	NA	HMDB025	NA
C31 H53 C	-0.00055	-0.83236	NA	level4	NA	NA	NA	NA
C14 H18 C	0.000164	0.657537	38.23758	level3	mzCloud	NA	HMDB000	Lipids and
C49 H70 C	-0.0029	-3.34177	NA	level4	NA	C16839	NA	Marine bic
C16 H27 N	-0.00016	-0.32142	NA	level4	NA	C15976	NA	Organohe
C5 H10 O5	21.98221	146496.5	58.10255	level4	BMDB	C00121	HMDB000	Organic ox
C19 H14 C	-0.00072	-2.35826	NA	level4	HMDB	NA	HMDB006	Phenylpro
C23 H30 C	-1.24E-05	-0.03099	NA	level4	HMDB	NA	HMDB001	Organohe
C24 H45 C	-0.00201	-3.9525	NA	level4	NA	NA	NA	NA
C30 H48	4.23E-05	0.103669	NA	level4	NA	NA	NA	NA
C20 H26 C	3.31E-05	0.095491	NA	level4	HMDB	NA	HMDB025	NA
C11 H14 C	0.000615	2.928186	NA	level4	NA	C11165	NA	NA
C33 H50 C	0.001237	1.987564	NA	level4	NA	NA	NA	NA
C35 H54 C	-0.00234	-3.69623	4.349031	level4	Public	NA	NA	NA
C10 H19 F	-0.00089	-4.67722	NA	level4	NA	NA	NA	NA
C25 H38 C	-4.28E-05	-0.11076	NA	level4	HMDB	NA	HMDB025	NA
C35 H52 N	0.001844	2.437828	NA	level4	HMDB	NA	HMDB024	NA
C23 H30 C	21.98024	59371.66	27.63043	level4	Public	NA	NA	NA
C13 H15 C	0.000362	1.359617	NA	level4	HMDB	NA	HMDB024	NA
C21 H20 C	-0.00101	-1.85152	NA	level4	NA	NA	NA	NA
C12 H16 C	0.000144	0.819112	NA	level4	HMDB	NA	HMDB024	NA
C8 H10	8.76E-05	0.825825	NA	level4	HMDB	NA	HMDB005	Benzenoid
C27 H44 C	0.000221	0.493251	NA	level4	NA	NA	NA	NA
C25 H51 C	0.002446	4.647172	NA	level4	NA	NA	NA	NA
C12 H18 C	0.000318	1.512762	NA	level4	NA	NA	NA	NA
C30 H50	9.48E-06	0.023104	NA	level4	HMDB	NA	HMDB000	Lipids and
C53 H97 C	-0.00138	-1.41972	NA	level4	NA	NA	NA	NA
C21 H34 C	0.00017	0.465256	NA	level4	HMDB	NA	HMDB025	NA
C15 H14 N	-0.00128	-4.82306	NA	level4	HMDB	NA	HMDB001	Organic ni
C23 H50 N	2.09E-05	0.044857	NA	level4	KEGG	C03640	NA	SP Sphing
C12 H16 C	0.00025	1.203524	NA	level4	HMDB	NA	HMDB024	NA
C23 H24 C	-0.00064	-1.83684	NA	level4	NA	C17005	NA	NA
C25 H42 C	0.000221	0.522442	58.87944	level4	Public	NA	NA	NA
C18 H20 N	0.00076	2.875442	12.96047	level4	Public	NA	HMDB001	Organohe
C25 H36 C	6.35E-05	0.158522	NA	level4	HMDB	NA	HMDB025	NA
C31 H44 C	-0.00496	-9.38444	36.69936	level3	mzCloud	C18557	NA	NA
C24 H41 N	-0.00186	-3.68652	NA	level4	NA	C17008	NA	NA
C15 H15 N	-0.00034	-1.13841	NA	level4	HMDB	NA	HMDB025	NA

C3 H8 N4	0.000265	2.006775	NA	level4	KEGG	C06381	NA	NA
C14 H12 C	-0.00227	-9.2816	61.88953	level2	mzCloud	C05901	HMDB000	Phenylpro
C8 H9 N C	2.14E-05	0.116763	91.84904	level1	Decode	C00847	HMDB000	Organohe
C16 H20 N	-0.00042	-1.08326	NA	level4	HMDB	NA	HMDB024	NA
C20 H23 N	0.001127	3.692786	NA	level4	HMDB	NA	HMDB025	NA
C9 H11 N	0.000173	1.163497	57.13466	level3	mzCloud	NA	NA	NA
C20 H36 C	-18.0107	-50555.4	41.67322	level3	mzCloud	NA	NA	NA
C14 H25 N	-0.00014	-0.54041	NA	level4	NA	NA	NA	NA
C21 H20 F	-0.00176	-4.43795	NA	level4	HMDB	NA	HMDB025	NA
C29 H50 N	21.98081	41133.45	34.67436	level4	Public	NA	NA	NA
C10 H21 N	0.000168	0.764718	NA	level4	NA	C17229	NA	NA
C35 H36 N	0.000508	0.880902	NA	level4	NA	C21603	NA	NA
C50 H91 C	-0.00156	-1.76278	NA	level4	NA	NA	NA	NA
C9 H7 N C	0.000165	1.138467	59.6123	level3	BMDB2	C08493	HMDB002	Organohe
C21 H34 C	-7.79E-05	-0.24492	31.7701	level4	Public	NA	HMDB025	NA
C18 H20 F	0.000773	1.986968	NA	level4	HMDB	NA	HMDB026	NA
C18 H21 N	-0.00162	-4.36731	NA	level4	HMDB	NA	HMDB024	NA
C23 H47 N	3.89E-05	0.105399	NA	level4	NA	NA	NA	NA
C28 H44 C	0.000123	0.286637	43.5352	level3	Public	NA	NA	NA
C22 H29 N	0.001713	3.795595	NA	level4	HMDB	NA	HMDB024	NA
C12 H16 N	-0.00013	-0.42396	NA	level4	HMDB	NA	HMDB024	NA
C30 H48 C	0.000116	0.272437	NA	level4	HMDB	NA	HMDB025	NA
C26 H26 F	-0.00117	-2.59254	NA	level4	HMDB	NA	HMDB025	NA
C23 H38 N	0.00178	4.216414	NA	level4	HMDB	NA	HMDB025	NA
C16 H26 C	0.000378	1.267197	8.277854	level4	Public	NA	NA	NA
C11 H14 N	-0.00113	-4.80666	NA	level4	HMDB	NA	HMDB025	NA
C19 H37 N	-0.00026	-0.75139	95.08961	level1	BMDB	NA	HMDB000	Lipids and
C30 H48 C	0.000376	0.853858	NA	level4	NA	NA	NA	NA
C9 H16 N3	0.001185	4.816382	NA	level4	NA	C21731	NA	NA
C29 H44 C	4.10E-06	0.00966	NA	level4	HMDB	NA	HMDB000	Lipids and
C18 H32 C	1.30E-05	0.049012	NA	level4	HMDB	NA	HMDB024	NA
C12 H12 N	0.000934	3.765636	56.11341	level4	Public	C07666	HMDB001	Benzenoid
C25 H26 C	-0.00059	-1.39409	7.789256	level4	Public	NA	HMDB002	Phenylpro
C10 H13 N	0.00015	0.628114	NA	level4	HMDB	NA	HMDB025	NA
C7 H13 N	0.0001	0.630361	73.83162	level2	mzCloud	NA	HMDB000	Organic ac
C20 H41 N	0.002324	4.731408	NA	level4	NA	C16562	NA	NA
C29 H48 C	-0.00058	-1.06542	NA	level4	NA	NA	NA	NA
C24 H34 N	0.002055	4.776016	NA	level4	HMDB	NA	HMDB001	Organic ac
C16 H28 C	0.000361	1.140522	NA	level4	NA	NA	NA	NA
C12 H12 C	-0.0013	-3.64438	NA	level4	HMDB	NA	HMDB025	NA
C15 H21 N	-0.00172	-4.91153	NA	level4	HMDB	NA	HMDB024	NA
C24 H44 C	0.000149	0.323414	NA	level4	NA	NA	NA	NA
C21 H30 C	-0.00027	-0.82116	81.06893	level2	mzCloud	C03205	HMDB000	Lipids and
C11 H16 N	0.000935	3.650703	NA	level4	HMDB	NA	HMDB025	NA
C13 H14 C	6.64E-05	0.283479	7.752462	level4	Public	NA	NA	NA
C38 H48 N	-0.00122	-1.6067	NA	level4	HMDB	NA	HMDB026	NA
C14 H27 N	5.75E-05	0.255188	NA	level4	NA	NA	NA	NA
C22 H38 C	-0.00035	-0.85263	1.126807	level4	Public	NA	NA	NA
C24 H42 C	-54.0318	-122163	45.63875	level3	Public	NA	NA	NA

C28 H43 N	0.002245	3.810135	NA	level4	HMDB	NA	HMDB025	NA	
C33 H56 O	0.001929	3.150317	NA	level4	HMDB	NA	HMDB001	Lipids and	
C27 H30 O	0.001556	3.122517	NA	level4	HMDB	NA	HMDB025	NA	
C37 H66 N	-0.00171	-2.39301	NA	level4	NA	NA	NA	NA	
C25 H51 O	-0.00178	-3.59585	NA	level4	NA	NA	NA	NA	
C25 H34 O	-4.31E-05	-0.09323	NA	level4	HMDB	NA	HMDB025	NA	
C21 H38 O	4.97E-05	0.140217	NA	level4	HMDB	NA	HMDB001	Lipids and	
C44 H32 N	0.001666	2.393641	NA	level4	HMDB	NA	HMDB025	NA	
C35 H34 O	0.000785	1.035632	NA	level4	NA	NA	NA	NA	
C14 H20 N	0.000646	1.970051	NA	level4	HMDB	NA	HMDB024	NA	
C27 H42 O	7.23E-05	0.174558	NA	level4	HMDB	NA	HMDB024	NA	
C23 H24 N	0.000444	0.886906	NA	level4	HMDB	NA	HMDB025	NA	
C12 H19 N	-4.64E-05	-0.18335	NA	level4	NA	NA	NA	NA	
C28 H36 N	-0.00065	-1.24438	NA	level4	HMDB	NA	HMDB025	NA	
C20 H30 O	37.95728	113571.7	29.41823	level4	Public	NA	NA	NA	
C25 H42 O	0.000272	0.598484	NA	level4	NA	NA	NA	NA	
C45 H54 N	0.000895	0.970276	NA	level4	KEGG	C06416	NA	NA	
C14 H20 O	17.02654	56732.29	22.09067	level4	Public	NA	NA	NA	
C26 H47 N	-0.00241	-4.45441	NA	level4	HMDB	NA	HMDB025	NA	
C50 H85 O	-0.00113	-1.22014	NA	level4	NA	NA	NA	NA	
C40 H52 O	-0.00367	-6.15123	26.86537	level4	Public	NA	NA	NA	
C18 H28 O	21.98183	71323.55	43.39154	level4	Public	NA	NA	NA	
C17 H20 N	1.08E-05	0.038077	NA	level4	HMDB	NA	HMDB025	NA	
C31 H44 O	0.002283	4.194789	NA	level4	HMDB	NA	HMDB025	NA	
C14 H20 O	0.000257	1.16556	76.90115	level2	mzCloud	NA	HMDB001	Lipids and	
C23 H38 O	-18.0106	-45680.6	86.83769	level2	mzCloud	NA	HMDB001	Lipids and	
C17 H26 O	0.001447	3.428534	9.279405	level4	Public	NA	NA	NA	
C38 H58 N	0.000722	1.144645	NA	level4	HMDB	NA	HMDB025	NA	
C2 H4 N4	9.82E-05	1.168748	62.53558	level2	Public	C11261	HMDB024	NA	
C16 H18 N	0.000108	0.424875	17.1888	level4	Public	NA	NA	NA	
C37 H68 N	-0.00216	-3.01672	NA	level4	NA	NA	NA	NA	
C25 H22 N	0.002135	4.828229	NA	level4	HMDB	NA	HMDB025	NA	
C18 H26 N	-0.00099	-2.98512	NA	level4	HMDB	NA	HMDB024	NA	
C21 H37 O	0.001824	4.219233	NA	level4	NA	NA	NA	NA	
C26 H34 O	-0.002	-3.82065	NA	level4	KEGG	C08769	NA	Terpenoid	
C9 H13 N3	0.00017	0.870961	NA	level4	NA	C19581	NA	Organohe	
C10 H11 N	0.00024	1.355424	NA	level4	HMDB	NA	HMDB025	NA	
C20 H16 O	-0.0008	-2.37506	35.6695	level3	mzCloud	C10523	HMDB003	Phenylpro	
C31 H33 N	0.001362	2.525544	NA	level4	HMDB	NA	HMDB025	NA	
C28 H42 O	0.000159	0.386476	NA	level4	HMDB	NA	HMDB024	NA	
C26 H28 N	0.001583	3.296362	NA	level4	HMDB	NA	HMDB025	NA	
C8 H15 N	0.000213	0.963588	92.02706	level2	BMDB	C00645	HMDB000	Organic ox	
C21 H23 O	0.001247	3.099805	NA	level4	HMDB	NA	HMDB025	NA	
C35 H52 O	-0.00132	-2.39422	NA	level4	HMDB	NA	HMDB025	NA	
C27 H34 O	17.02767	31874.48	45.59063	level3	mzCloud	C16915	NA	Phenylpro	
C12 H8 O2	0.000261	1.417917	NA	level4	HMDB	NA	HMDB024	NA	
C20 H28 O	2.13E-05	0.07503	36.32215	level3	mzCloud	C16681	HMDB000	Lipids and	
C26 H24 N	0.001326	3.249541	NA	level4	HMDB	NA	HMDB025	NA	
C46 H87 N	0.000792	0.992628	NA	level4	NA	NA	NA	NA	
C26 H27 F	0.000782	1.869319	NA	level4	HMDB	NA	HMDB024	NA	
C11 H23 N	0.000127	0.54296	NA	level4	NA	C17233	NA	NA	
C2 H4 N4	7.29E-05	0.628265	NA	level4	HMDB	NA	HMDB026	NA	
C19 H26 O	2.43E-05	0.080326	39.2695	level3	mzCloud	C14256	NA	NA	
C21 H27 N	5.07E-05	0.148472	NA	level4	HMDB	NA	HMDB001	Phenylpro	
C26 H27 N	-4.93E-05	-0.13364	NA	level4	HMDB	NA	HMDB025	NA	

C21 H36 C	18.01052	48907.58	22.36475	level4	Public	NA	NA	NA
C21 H21 F	-0.00106	-2.67607	NA	level4	HMDB	NA	HMDB025	NA
C21 H34 C	-18.0107	-47119.5	10.67683	level4	Public	NA	NA	NA
C35 H40 N	0.001702	2.779532	NA	level4	HMDB	NA	HMDB026	NA
C16 H24 C	4.62E-06	0.016488	26.18121	level4	Public	NA	NA	NA
C4 H6 O2	0.000148	1.72098	87.80698	level2	mzCloud	C01771	HMDB001	Lipids and
C15 H14 C	-0.00259	-8.44775	31.66197	level3	mzCloud	C12136	HMDB003	Phenylpro
C17 H20 C	0.000172	0.541789	NA	level4	HMDB	NA	HMDB025	NA
C20 H24 F	0.000819	2.218663	NA	level4	HMDB	NA	HMDB024	NA
C27 H48 C	0.000219	0.501971	NA	level4	HMDB	NA	HMDB024	NA
C18 H19 N	0.002428	8.636397	41.5147	level3	mzCloud	NA	NA	NA
C14 H20 N	7.16E-05	0.24522	NA	level4	HMDB	NA	HMDB024	NA
C35 H52 C	-0.00159	-2.64668	NA	level4	KEGG	C09157	NA	Terpenoid
C19 H36 C	-8.99E-05	-0.30333	NA	level4	HMDB	NA	HMDB025	NA
C20 H22 C	21.98007	58748.8	5.603161	level4	Public	NA	NA	NA
C25 H19 N	-0.00076	-2.00618	NA	level4	HMDB	NA	HMDB025	NA
C22 H30 N	-0.0015	-3.25368	NA	level4	HMDB	NA	HMDB025	NA
C23 H32 N	0.001773	4.259236	NA	level4	HMDB	NA	HMDB025	NA
C31 H48 C	-0.00218	-3.76041	NA	level4	NA	NA	NA	NA
C21 H31 N	-0.00152	-3.95142	NA	level4	HMDB	NA	HMDB001	Organohe
C18 H25 N	-0.00015	-0.45091	NA	level4	HMDB	NA	HMDB006	Organic ac
C21 H30 C	-0.00073	-1.84764	NA	level4	HMDB	NA	HMDB024	NA
C24 H40 C	392.2926	999999.9	96.19349	level1	BMDB	C04483	HMDB000	Lipids and
C27 H42 N	0.000577	0.9206	NA	level4	HMDB	NA	HMDB025	NA
C21 H27 N	0.001453	3.263446	NA	level4	HMDB	NA	HMDB001	Benzenoid
C20 H35 N	0.000149	0.343944	NA	level4	NA	NA	NA	NA
C19 H25 N	-6.29E-05	-0.16082	NA	level4	HMDB	NA	HMDB025	NA
C9 H16 N2	0.000348	1.401827	NA	level4	HMDB	NA	HMDB002	Organic ac
C13 H19 N	-1.85E-05	-0.06477	NA	level4	HMDB	NA	HMDB001	Benzenoid
C25 H28 C	-0.00077	-1.89242	11.92749	level4	Public	NA	NA	NA
C20 H28 N	-5.38E-05	-0.12793	NA	level4	HMDB	NA	HMDB025	NA
C23 H36 C	-0.00028	-0.78833	NA	level4	NA	C15417	NA	NA
C14 H16 C	-0.0005	-1.5129	NA	level4	HMDB	NA	HMDB024	NA
C23 H22 C	-18.0112	-43915	17.49169	level4	Public	NA	NA	NA
C14 H16 N	0.000141	0.490234	NA	level4	HMDB	NA	HMDB025	NA
C12 H12 C	0.000261	1.278187	NA	level4	HMDB	NA	HMDB024	NA
C40 H52 C	0.001829	2.274294	NA	level4	HMDB	NA	HMDB025	NA
C8 H12 O3	0.00031	1.988831	24.46739	level4	Public	NA	NA	NA
C7 H15 N	0.00047	2.249736	NA	level4	HMDB	NA	HMDB025	NA
C9 H10 O4	-18.0103	-98926.5	79.37989	level2	mzCloud	C10447	HMDB000	Phenylpro
C27 H25 F	0.001701	3.361199	NA	level4	HMDB	NA	HMDB024	NA
C25 H33 C	0.002434	4.905086	NA	level4	NA	NA	NA	NA
C29 H30 N	-0.0011	-2.51107	NA	level4	HMDB	NA	HMDB025	NA
C13 H9 F (	-0.00062	-2.67522	NA	level4	HMDB	NA	HMDB024	NA
C23 H18 F	-0.00045	-1.1457	NA	level4	HMDB	NA	HMDB025	NA
C16 H17 N	9.88E-05	0.23127	NA	level4	HMDB	NA	HMDB001	Organohe
C13 H16 C	2.76E-05	0.125506	NA	level4	HMDB	NA	HMDB025	NA
C13 H15 N	0.000268	0.855728	NA	level4	HMDB	NA	HMDB025	NA
C16 H14 C	3.89E-05	0.144107	NA	level4	HMDB	NA	HMDB025	NA
C20 H30 C	0.001883	4.927427	NA	level4	HMDB	NA	HMDB025	NA
C25 H38 C	-0.00023	-0.62867	NA	level4	NA	C21704	NA	NA
C19 H27 N	0.000165	0.398935	NA	level4	NA	NA	NA	NA
C5 H5 N5	8.47E-05	0.626875	63.64228	level1	BMDB	C00147	HMDB000	Organohe

C28 H44 C	0.000108	0.261744	NA	level4	HMDB	NA	HMDB025	NA	
C9 H10 N2	0.000131	0.579505	51.80785	level4	BMDB	NA	NA	NA	
C7 H12	0.000169	1.757688	NA	level4	HMDB	NA	HMDB025	NA	
C21 H33 F	-0.00118	-3.68969	NA	level4	NA	C14981	NA	NA	
C21 H41 N	-3.56E-05	-0.09595	NA	level4	HMDB	NA	HMDB025	NA	
C28 H43 N	-0.00132	-2.21493	NA	level4	NA	NA	NA	NA	
C13 H20 C	-0.00033	-1.22695	NA	level4	NA	NA	NA	NA	
C27 H38 C	0.00012	0.282396	NA	level4	HMDB	NA	HMDB024	NA	
C13 H21 N	0.000131	0.411854	NA	level4	HMDB	NA	HMDB025	NA	
C3H8NO5	0.000818	4.393821	NA	level4	NA	C18565	NA	NA	
C23 H43 N	-0.00139	-3.36934	NA	level4	HMDB	NA	HMDB025	NA	
C17 H22 B	17.02739	46386.27	4.298645	level4	Public	NA	NA	NA	
C13 H17 N	0.001296	3.23859	NA	level4	HMDB	NA	HMDB025	NA	
C24 H20 F	0.000125	0.2363	NA	level4	HMDB	NA	HMDB025	NA	
C10 H13 N	9.87E-05	0.406031	NA	level4	HMDB	NA	HMDB025	NA	
C17 H23 N	0.003303	11.42261	30.01835	level3	mzCloud	NA	NA	NA	
C20 H30 C	-0.00013	-0.42058	58.23673	level3	Public	NA	NA	NA	
C16 H8 N2	-0.00124	-4.24557	NA	level4	HMDB	NA	HMDB025	NA	
C34 H38 N	0.000453	0.757426	NA	level4	HMDB	NA	HMDB025	NA	
C10 H15 N	0.000202	0.946439	3.652742	level4	Public	NA	NA	NA	
C9 H10 O4	1.97E-05	0.107971	NA	level4	HMDB	NA	HMDB000	Phenylpro	
C29 H42 C	-0.0018	-3.69342	NA	level4	KEGG	C08176	NA	ST	Sterol
C6 H4 N2	2.99E-05	0.248906	NA	level4	HMDB	NA	HMDB024	NA	
C23 H17 N	-0.00063	-1.62093	NA	level4	HMDB	NA	HMDB025	NA	
C27 H48 C	-0.00032	-0.69069	NA	level4	HMDB	NA	HMDB025	NA	
C27 H46 C	-0.00016	-0.3603	NA	level4	HMDB	NA	HMDB000	Lipids and	
C26 H22 C	-0.00142	-2.83123	NA	level4	HMDB	NA	HMDB025	NA	
C26 H41 N	-0.00028	-0.61306	NA	level4	HMDB	NA	HMDB025	NA	
C7 H13 N	0.000301	1.453653	NA	level4	NA	C14876	NA	NA	
C17 H27 N	-9.16E-05	-0.29631	NA	level4	HMDB	NA	HMDB024	NA	
C9 H10	0.000133	1.12695	NA	level4	HMDB	NA	HMDB024	NA	
C36 H44 N	0.000615	0.932064	NA	level4	HMDB	NA	HMDB000	Organohe	
C20 H16 C	0.001048	3.445008	30.60575	level4	Public	NA	NA	NA	
C25 H40 N	0.000935	1.884161	NA	level4	HMDB	NA	HMDB000	Lipids and	
C15 H24 C	0.000261	1.036768	17.92489	level4	Public	NA	HMDB003	Lipids and	
C16 H23 N	7.74E-07	0.002217	NA	level4	HMDB	NA	HMDB025	NA	
C48 H80 C	0.000922	0.975871	NA	level4	KEGG	C08792	NA	Terpenoid	
C45 H84 N	0.002741	3.304666	NA	level4	NA	NA	NA	NA	
C18 H13 N	-0.00035	-1.44451	NA	level4	HMDB	NA	HMDB024	NA	
C50 H82 C	0.00349	4.392112	NA	level4	NA	NA	NA	NA	
C32 H54 C	0.000122	0.203582	NA	level4	NA	NA	NA	NA	
C24 H38 C	-20.0264	-49293.1	74.94166	level1	BMDB2	NA	HMDB000	Lipids and	
C27 H39 N	3.44E-05	0.075319	NA	level4	NA	NA	NA	NA	
C32 H63 C	-0.0008	-1.34673	NA	level4	NA	NA	NA	NA	
C20 H15 N	-0.00039	-1.17037	NA	level4	HMDB	NA	HMDB024	NA	
C8 H12 O4	0.00025	1.451792	NA	level4	HMDB	NA	HMDB000	Lipids and	
C17 H17 N	0.000129	0.294457	NA	level4	HMDB	NA	HMDB006	Organic ac	
C39 H79 C	-0.00211	-2.91821	NA	level4	NA	NA	NA	NA	
C30 H52 C	0.000489	1.026051	NA	level4	HMDB	NA	HMDB024	NA	
C12 H24 N	0.000533	1.495652	NA	level4	HMDB	NA	HMDB025	NA	
C9 H14 N2	6.26E-05	0.272261	NA	level4	NA	C20921	NA	NA	

C38 H44 (	0.00014	0.223469	NA	level4	NA	C10062	NA	Organohe
C22 H37 N	-0.00022	-0.64561	NA	level4	HMDB	NA	HMDB024	NA
C20 H22 (	21.98026	70868.19	57.79236	level4	Public	NA	NA	NA
C24 H49 (	-0.00137	-2.85367	NA	level4	NA	NA	NA	NA
C17 H20 N	-0.00053	-1.96707	NA	level4	HMDB	NA	HMDB025	NA
C13 H24 (	0.000164	0.720731	NA	level4	NA	NA	NA	NA
C19 H25 N	-0.00131	-3.68805	NA	level4	HMDB	NA	HMDB025	NA
C43 H84 N	0.00306	3.798416	NA	level4	NA	NA	NA	NA
C45 H86 N	0.002864	3.443839	NA	level4	NA	NA	NA	NA
C10 H17 N	0.000931	2.40591	NA	level4	HMDB	NA	HMDB025	NA
C30 H37 N	0.00262	4.491408	NA	level4	HMDB	NA	HMDB026	NA
C21 H14 (	-0.00067	-1.84406	NA	level4	NA	NA	NA	NA
C28 H50 (	-0.00213	-3.15688	NA	level4	NA	NA	NA	NA
C21 H32 (	-0.00021	-0.59989	22.87726	level4	Public	NA	NA	NA
C8 H19 O:	-0.00077	-2.9765	NA	level4	NA	C18978	NA	NA
C15 H22 (	-0.00125	-4.40286	NA	level4	HMDB	NA	HMDB025	NA
C44 H77 (	0.002029	2.599925	NA	level4	NA	NA	NA	NA
C42 H85 (	-0.00244	-3.18556	NA	level4	NA	NA	NA	NA
C4 H6 O2	0.00021	2.446414	NA	level4	HMDB	NA	HMDB025	NA
C35 H38 N	0.002105	3.448847	NA	level4	HMDB	NA	HMDB024	NA
C14 H24 (	0.001459	4.557659	NA	level4	HMDB	NA	HMDB025	NA
C14 H19 N	0.000317	0.987407	NA	level4	HMDB	NA	HMDB001	Organohe

C10 H12 (	0.000273	1.393532	NA	level4	HMDB	NA	HMDB025	NA
C12 H23 N	0.000708	2.051688	NA	level4	HMDB	NA	HMDB025	NA
C14H14N:	0.000786	2.775464	NA	level4	NA	C15452	NA	NA
C4 H5 N (	0.000209	1.818525	NA	level4	HMDB	NA	HMDB024	NA
C4 H9 N (	0.000195	1.889171	53.67568	level4	Public	C03665	HMDB000	Organic ac
C8 H9 N (	6.48E-05	0.429151	40.97994	level3	Public	C03005	HMDB003	Benzenoid
C26 H34 N	-0.00155	-3.56531	NA	level4	HMDB	NA	HMDB024	NA
C26 H43 N	449.3135	999998.7	97.56593	level1	BMDB2	C05464	HMDB000	Lipids and
C32 H50 (	-0.00194	-3.88327	NA	level4	HMDB	NA	HMDB024	NA
C16 H27 N	-0.00022	-0.82248	NA	level4	HMDB	NA	HMDB025	NA
C17 H22 (	0.000106	0.331913	44.767	level4	Public	NA	NA	NA
C27 H44 (	0.000138	0.332157	NA	level4	HMDB	NA	HMDB000	Lipids and
C26 H28 (	0.001493	2.438666	NA	level4	NA	NA	NA	NA
C25 H42 (	5.36E-05	0.131806	NA	level4	NA	NA	NA	NA
C25 H40 (	-7.68E-05	-0.19782	NA	level4	NA	C15422	NA	NA
C15 H20 N	17.0266	69736.1	13.47528	level4	Public	NA	NA	NA
C9 H10 O	0.000277	2.069173	88.36271	level2	mzCloud	NA	HMDB003	Benzenoid
C33 H54 (	0.000666	1.151581	NA	level4	NA	C17074	NA	Terpenoid
C8 H8 O4	17.02684	101324.8	4.96213	level4	Public	C10331	HMDB002	Organic o:
C8 H15 N	0.000151	0.683772	NA	level4	HMDB	NA	HMDB025	NA
C34 H54 N	0.001856	2.541479	NA	level4	NA	NA	NA	NA
C8 H15 N:	-0.00163	-4.93883	NA	level4	NA	C18964	NA	NA
C14 H23 N	-0.00182	-4.63321	NA	level4	HMDB	NA	HMDB025	NA
C23 H28 (	0.003389	7.841165	41.98231	level3	mzCloud	NA	NA	NA
C14 H18 (	0.000207	1.022695	NA	level4	NA	C12288	NA	Phenylpro
C25 H24 N	-0.00137	-3.11792	NA	level4	HMDB	NA	HMDB025	NA
C28 H44 N	-0.00141	-2.47528	NA	level4	NA	NA	NA	NA
C8 H5 N (	-1.56E-05	-0.07371	NA	level4	HMDB	NA	HMDB025	NA
C22 H25 N	17.02659	38077.78	23.90001	level4	Public	NA	NA	NA
C22 H21 N	-0.00056	-1.48654	NA	level4	NA	C12224	NA	NA
C15 H28 (	5.33E-05	0.195779	NA	level4	NA	NA	NA	NA

C26 H36 C	-0.00148	-3.32421	NA	level4	HMDB	NA	HMDB024	NA
C22 H34 C	-0.00041	-1.08754	34.76355	level4	Public	NA	NA	NA
C17 H23 N	0.001388	4.548213	NA	level4	HMDB	NA	HMDB024	NA
C13 H24 C	0.000238	1.120313	NA	level4	NA	NA	NA	NA
C13 H26 C	0.000185	0.804732	NA	level4	NA	NA	NA	NA
C14 H16 N	3.79E-05	0.145636	21.40072	level4	Public	NA	NA	NA
C29 H42 C	-0.00139	-2.67264	NA	level4	NA	NA	NA	NA
C50 H83 C	-0.0013	-1.40378	NA	level4	NA	NA	NA	NA
C19 H17 C	-0.00073	-1.77044	NA	level4	HMDB	NA	HMDB001	Benzenoid
C24 H30 C	4.01E-05	0.100782	NA	level4	HMDB	NA	HMDB025	NA
C8 H8 O4	0.000224	1.330862	NA	level4	HMDB	NA	HMDB000	Benzenoid
C15 H26 N	37.95453	142579.3	12.41363	level4	Public	NA	NA	NA
C44 H84 N	0.002606	3.18731	NA	level4	NA	NA	NA	NA
C21 H20 C	0.004763	11.4473	55.47609	level3	mzCloud	C10524	HMDB024	Phenylpro
C23 H38 C	17.02645	49168.58	16.24964	level4	Public	NA	NA	NA
C26 H32 N	0.002033	3.603705	NA	level4	HMDB	NA	HMDB025	NA
C14 H18 C	0.00011	0.373527	NA	level4	HMDB	NA	HMDB025	NA
C30 H46 C	-0.00176	-3.62088	15.89561	level4	Public	NA	NA	NA
C29 H33 C	-0.00016	-0.3096	NA	level4	HMDB	NA	HMDB025	NA
C21 H27 N	5.93E-05	0.127485	NA	level4	HMDB	NA	HMDB001	Organohe
C24 H39 N	6.13E-05	0.151201	NA	level4	HMDB	NA	HMDB025	NA
C35 H61 N	5.07E-05	0.073758	NA	level4	HMDB	NA	HMDB025	NA
C24 H40 C	392.2923	999999	97.46848	level1	BMDB2	C02528	HMDB000	Lipids and
C26 H46 C	0.000228	0.519023	NA	level4	NA	NA	NA	NA
C26 H48 N	-0.0017	-3.28234	NA	level4	HMDB	NA	HMDB001	Lipids and
C29 H39 C	-0.00268	-4.59708	NA	level4	HMDB	NA	HMDB024	NA
C13 H26 N	0.001836	3.80111	NA	level4	KEGG	C04832	NA	NA
C22 H24 C	-0.00204	-4.54162	NA	level4	NA	NA	NA	NA
C25 H29 C	0.000344	0.753559	NA	level4	HMDB	NA	HMDB024	NA
C35 H47 N	0.001132	1.765297	NA	level4	HMDB	NA	HMDB025	NA
C51 H84 C	-0.00363	-4.58554	NA	level4	HMDB	NA	HMDB025	NA
C22 H19 N	-0.00081	-2.23087	NA	level4	HMDB	NA	HMDB024	NA
C22 H25 N	37.9575	99060.93	10.94351	level4	Public	NA	NA	NA
C19 H20 C	21.98361	66996.42	45.90893	level4	Public	NA	NA	NA
C24 H34 C	-0.00021	-0.6271	NA	level4	NA	C14915	NA	NA
C22 H39 N	-8.52E-05	-0.24383	42.11951	level3	mzCloud	C13828	HMDB001	Organic ni
C18 H22 N	0.000742	1.963569	NA	level4	HMDB	NA	HMDB024	NA
C12 H12 N	0.000141	0.567039	NA	level4	HMDB	NA	HMDB006	Organohe
C29 H28 N	-0.00131	-2.72864	NA	level4	HMDB	NA	HMDB026	NA
C13 H24 C	-0.0002	-0.8189	NA	level4	HMDB	NA	HMDB024	NA
C34 H51 N	-0.00018	-0.27383	NA	level4	HMDB	NA	HMDB026	NA
C26 H43 N	-36.0213	-80169.6	74.80788	level2	Public	NA	HMDB024	NA
C14 H25 N	-0.00019	-0.66334	4.194454	level4	Public	NA	NA	NA
C59 H90 C	-0.00342	-3.68707	NA	level4	NA	C15956	NA	PR Prenol
C8 H10 N4	0.000227	1.168845	75.85048	level1	BMDB	C07481	HMDB000	Organohe
C10 H15 N	0.000559	3.384197	NA	level4	HMDB	NA	HMDB025	NA
C12 H20 C	0.000222	1.234633	NA	level4	NA	NA	NA	NA
C6 H8 O2	-18.0105	-160733	79.65498	level2	BMDB	NA	NA	NA
C37 H73 N	0.002025	3.311459	NA	level4	NA	NA	NA	NA
C23 H25 I2	-0.00045	-0.73363	NA	level4	HMDB	NA	HMDB006	Organic ox
C25 H39 N	0.000251	0.578436	NA	level4	NA	NA	NA	NA
C28 H45 N	0.000376	0.818965	NA	level4	NA	NA	NA	NA
C19 H22 C	-6.24E-05	-0.19871	NA	level4	HMDB	NA	HMDB025	NA
C28 H34 C	0.002116	4.11544	NA	level4	HMDB	NA	HMDB025	NA
C25 H40 C	6.33E-05	0.145074	NA	level4	HMDB	NA	HMDB006	Organic ac

C26 H44 C	0.000113	0.269678	NA	level4	HMDB	NA	HMDB024	NA
C24 H34 C	-7.50E-05	-0.20251	NA	level4	HMDB	NA	HMDB001	Lipids and
C28 H43 N	0.000189	0.413614	NA	level4	NA	NA	NA	NA
C10 H16 C	2.82E-05	0.141044	NA	level4	NA	NA	NA	NA
C23 H38 C	21.98207	58111.04	28.69205	level3	BMDB2	NA	HMDB030	NA
C16 H32 C	0.000109	0.452103	NA	level4	NA	C14499	NA	NA
C24 H34 C	-0.00019	-0.54694	NA	level4	HMDB	NA	HMDB024	NA
C26 H41 N	0.000154	0.343202	NA	level4	NA	NA	NA	NA
C12 H15 N	0.00017	0.524437	NA	level4	HMDB	NA	HMDB025	NA
C35 H44 C	0.002565	4.854823	NA	level4	NA	NA	NA	NA
C23 H30 C	21.98386	54658.45	54.47386	level4	Public	NA	NA	NA
C6 H10 O2	0.000105	0.718269	NA	level4	HMDB	NA	HMDB025	NA
C28 H33 N	0.001529	3.137354	NA	level4	HMDB	NA	HMDB025	NA
C43 H50 N	-0.00034	-0.47467	NA	level4	KEGG	C09254	NA	NA
C27 H42 C	-0.00017	-0.3409	NA	level4	NA	NA	NA	NA
C45 H86 C	-358.344	-474860	10.03892	level4	Public	NA	NA	NA
C8 H9 N C	0.000139	0.920478	76.85163	level1	Decode	C06804	HMDB000	Benzenoid
C11 H20 N	0.000174	0.821119	NA	level4	HMDB	NA	HMDB024	NA
C24 H23 N	-0.00065	-1.61348	NA	level4	NA	C12200	NA	NA
C15 H10 C	-0.00015	-0.51582	NA	level4	HMDB	NA	HMDB000	Phenylpro
C10 H9 Cl	0.00049	2.549077	NA	level4	NA	C21110	NA	NA
C29 H44 C	-0.002	-4.24249	NA	level4	HMDB	NA	HMDB025	NA
C15 H12 C	21.98364	80801.85	25.85466	level4	Public	NA	NA	NA
C26 H47 C	0.000296	0.508685	NA	level4	NA	NA	NA	NA
C35 H52 C	-0.00241	-3.91402	NA	level4	NA	C11972	NA	NA
C27 H46 C	-18.0106	-44763.4	87.58398	level1	BMDB	C15519	HMDB000	Lipids and
C19 H14 C	-0.00068	-2.00955	28.583	level4	Public	NA	NA	NA
C24 H27 C	0.000298	0.699025	NA	level4	HMDB	NA	HMDB025	NA
C15 H12 N	6.38E-05	0.237904	NA	level4	HMDB	NA	HMDB025	NA
C36 H54 C	-0.00066	-0.93117	NA	level4	HMDB	NA	HMDB024	NA
C33 H34 C	0.003065	4.586558	NA	level4	HMDB	NA	HMDB024	NA
C22 H40 N	-0.00012	-0.31484	NA	level4	NA	NA	NA	NA
C12 H20 C	-0.00014	-0.39499	NA	level4	HMDB	NA	HMDB025	NA
C26 H28 C	-4.54E-05	-0.08054	14.79997	level4	Public	NA	NA	NA
C27 H44 C	4.64E-05	0.096573	13.43389	level4	Public	NA	HMDB003	Lipids and
C12 H18 I	0.000449	1.482353	NA	level4	HMDB	NA	HMDB024	NA
C14 H15 C	0.000204	0.828347	NA	level4	NA	C14938	NA	NA
C14 H18 C	0.000266	1.009621	NA	level4	HMDB	NA	HMDB024	NA
C13 H19 C	-0.00142	-4.86725	NA	level4	HMDB	NA	HMDB025	NA
C30 H46 C	0.000402	0.827528	NA	level4	NA	NA	NA	NA
C21 H25 N	-0.00101	-2.27884	NA	level4	HMDB	NA	HMDB025	NA
C16 H22 N	0.000601	1.88823	NA	level4	HMDB	NA	HMDB024	NA
C27 H30 C	0.001658	3.188315	NA	level4	HMDB	NA	HMDB025	NA
C8 H16 N2	0.000749	3.403755	NA	level4	NA	C18923	NA	NA
C19 H14 C	-0.00076	-2.36931	17.23085	level4	Public	NA	NA	NA
C16 H17 N	347.0919	999994	0.728509	level4	Public	C06895	HMDB001	Organohe
C10 H18	0.000127	0.920977	NA	level4	HMDB	NA	HMDB025	NA
C21 H27 N	0.000112	0.257957	NA	level4	HMDB	NA	HMDB025	NA
C16 H23 N	1.87E-05	0.055576	NA	level4	HMDB	NA	HMDB024	NA
C21 H34 C	-18.0107	-51423	73.58147	level2	mzCloud	NA	NA	NA
C42 H83 N	0.003138	4.60366	NA	level4	NA	NA	NA	NA
C21 H28 C	-0.00031	-0.88793	NA	level4	HMDB	NA	HMDB000	Lipids and
C14 H17 N	-2.11E-05	-0.07674	NA	level4	HMDB	NA	HMDB001	Organic ac
C11 H20 C	0.000224	1.117629	NA	level4	NA	NA	NA	NA

C14 H18 C	0.000361	1.540067	NA	level4	HMDB	NA	HMDB024	NA
C16 H15 C	7.87E-05	0.210271	NA	level4	HMDB	NA	HMDB006	Organohe
C12 H11 N	0.000172	1.016837	NA	level4	HMDB	NA	HMDB024	NA
C21 H30 C	-0.00011	-0.30108	NA	level4	HMDB	NA	HMDB000	Lipids and
C24 H40 C	17.02668	41702.67	94.0859	level1	BMDB2	C00695	HMDB000	Lipids and
C31 H49 N	0.000305	0.652221	NA	level4	HMDB	NA	HMDB025	NA
C3 H7 N C	0.000336	1.673242	67.93249	level2	mzCloud	C05824	HMDB000	Organic ac
C27 H42 N	0.000705	1.393102	NA	level4	HMDB	NA	HMDB001	Phenylpro
C19 H34 N	0.0008	1.592741	NA	level4	NA	C19785	NA	NA
C21 H32 C	-4.59E-05	-0.14517	88.49596	level2	mzCloud	C01953	HMDB000	Lipids and
C11 H12 N	0.000166	0.656782	NA	level4	HMDB	NA	HMDB001	Organohe
C24 H39 N	0.00194	3.99742	NA	level4	HMDB	NA	HMDB024	NA
C26 H42 C	-2.47E-05	-0.059	NA	level4	HMDB	NA	HMDB025	NA
C33 H54 C	0.000284	0.465731	NA	level4	NA	NA	NA	NA
C10 H20 N	-0.00098	-4.01884	NA	level4	HMDB	NA	HMDB025	NA
C49 H100	0.004364	4.47763	NA	level4	NA	C20642	NA	NA
C27 H40 C	3.82E-05	0.100436	NA	level4	NA	NA	NA	NA
C20 H32 C	0.000248	0.673992	NA	level4	HMDB	NA	HMDB000	Lipids and
C15 H20 C	17.02653	60780.6	11.51802	level4	Public	NA	NA	NA
C15 H29 N	0.000124	0.393467	NA	level4	HMDB	NA	HMDB025	NA
C9 H17 N3	6.97E-05	0.264917	NA	level4	HMDB	NA	HMDB026	NA
C14 H27 N	3.38E-05	0.131284	NA	level4	NA	NA	NA	NA
C24 H36 C	0.000166	0.410572	82.59596	level2	BMDB2	NA	NA	NA
C25 H39 N	-8.39E-05	-0.20097	46.69182	level4	Public	NA	NA	NA
C26 H34 C	0.000501	1.133786	NA	level4	HMDB	NA	HMDB025	NA
C8 H15 N	-18.0103	-81461.6	94.73293	level2	BMDB	C00140	HMDB000	Organic ox
C16 H17 C	-0.00055	-1.81212	NA	level4	HMDB	NA	HMDB006	Organohe
C13 H20 C	0.000263	1.26449	NA	level4	HMDB	NA	HMDB024	NA
C36 H61 C	0.002484	3.807811	NA	level4	NA	NA	NA	NA
C34 H45 N	-0.00143	-2.30253	NA	level4	HMDB	NA	HMDB025	NA
C15 H20 C	-0.00011	-0.40003	29.06783	level4	Public	NA	NA	NA
C10 H17 N	0.000262	1.313542	19.44609	level4	Public	NA	NA	NA
C14 H18 N	8.59E-05	0.280495	NA	level4	HMDB	NA	HMDB024	NA
C15 H18 N	21.98097	90777.21	17.1881	level4	Public	NA	HMDB024	NA
C18 H25 N	-0.00048	-1.31923	NA	level4	HMDB	NA	HMDB025	NA
C16 H20 C	-7.67E-05	-0.24878	57.22421	level4	Public	NA	NA	NA
C15 H22	0.000153	0.758752	NA	level4	NA	NA	NA	NA
C10 H17 N	0.000329	1.654213	NA	level4	HMDB	NA	HMDB000	Alkaloids a
C28 H42 C	0.000256	0.578366	NA	level4	NA	NA	NA	NA
C27 H39 C	0.001468	2.993517	NA	level4	NA	C18214	NA	NA
C16 H29 N	-6.60E-05	-0.23288	NA	level4	NA	NA	NA	NA
C33 H48 N	-0.00067	-1.12005	NA	level4	KEGG	C05789	NA	NA
C23 H39 N	0.000141	0.345642	NA	level4	NA	NA	NA	NA
C30 H52 N	-0.00297	-4.80526	NA	level4	NA	NA	NA	NA
C16 H28 C	0.000521	1.496148	NA	level4	NA	C17638	NA	NA
C10 H11 N	-7.32E-05	-0.35009	NA	level4	HMDB	NA	HMDB000	Organic ac
C36 H40 N	-0.00034	-0.55047	NA	level4	NA	C21434	NA	NA
C23 H34 C	18.0107	46152.81	33.0052	level4	Public	NA	NA	NA
C26 H41 N	-0.0002	-0.40059	NA	level4	NA	NA	NA	NA
C15 H14 C	21.98366	90806.21	31.93223	level3	mzCloud	NA	NA	NA
C33 H49 N	-1.31E-05	-0.02151	NA	level4	NA	C10015	NA	NA
C11 H15 N	8.05E-05	0.313173	NA	level4	HMDB	NA	HMDB024	NA
C25 H32 N	0.00335	7.343765	38.64494	level3	mzCloud	C01626	NA	Alkaloids

C13 H8	0.000192	1.172249	NA	level4	KEGG	C08456	NA	NA
C24 H39 N	0.000113	0.267093	NA	level4	HMDB	NA	HMDB025	NA
C13 H19 N	0.00012	0.507644	NA	level4	HMDB	NA	HMDB025	NA
C16 H18 C	-0.00015	-0.50931	NA	level4	NA	NA	NA	NA
C25 H20 C	-0.00053	-1.51166	NA	level4	HMDB	NA	HMDB024	NA
C21 H31 F	-0.0017	-4.85523	NA	level4	NA	C15166	NA	NA
C12 H21 N	-1.32E-05	-0.05192	NA	level4	NA	C16138	NA	Peptides
C14 H17 C	0.000582	1.692118	NA	level4	HMDB	NA	HMDB025	NA
C28 H39 N	-0.00212	-4.50935	NA	level4	HMDB	NA	HMDB024	NA
C8 H10 O3	0.000316	2.053888	6.116241	level4	Public	NA	NA	NA
C27 H42 C	4.20E-05	0.105367	NA	level4	NA	NA	NA	NA
C17 H17 N	0.000833	3.342157	NA	level4	NA	C18717	NA	NA
C8 H16 N2	0.000105	0.478832	9.973825	level3	BMDB	NA	NA	NA
C35 H54 C	-0.00213	-3.28053	NA	level4	HMDB	NA	HMDB006	Lipids and
C18 H26 C	6.55E-05	0.205171	NA	level4	HMDB	NA	HMDB001	Organohe
C8 H9 N C	0.000169	1.013939	NA	level4	HMDB	NA	HMDB024	NA
C27 H40 C	0.000852	1.576756	NA	level4	HMDB	NA	HMDB001	Lipids and
C21 H29 F	-0.00161	-4.83796	NA	level4	NA	C15127	NA	NA
C51 H89 C	0.002455	2.6557	NA	level4	NA	NA	NA	NA
C8 H14 N2	0.000356	1.759197	NA	level4	KEGG	C06658	NA	NA
C21 H32 C	21.98183	69509.9	15.2648	level4	Public	NA	HMDB003	Benzenoid
C16 H19 N	3.27E-05	0.108571	NA	level4	HMDB	NA	HMDB024	NA
C50 H74 C	0.002933	3.264475	NA	level4	HMDB	NA	HMDB024	NA
C16 H24 C	0.000965	2.565535	3.728942	level4	Public	NA	NA	NA
C15 H24 C	0.000154	0.653904	NA	level4	HMDB	NA	HMDB025	NA
C10 H12 N	3.91E-05	0.135799	NA	level4	HMDB	NA	HMDB000	Nucleoside
C7 H7 N C	0.000151	1.098942	72.34884	level2	mzCloud	C01004	HMDB000	Alkaloids a
C26 H40 C	-2.15E-05	-0.05589	NA	level4	NA	NA	NA	NA
C26 H35 N	-1.90E-05	-0.04654	NA	level4	HMDB	NA	HMDB025	NA
C33 H52 C	-0.00145	-2.66182	NA	level4	NA	NA	NA	NA
C17 H24 C	7.26E-05	0.278947	16.88167	level4	Public	C08449	HMDB003	Lipids and
C51 H91 C	-0.00164	-1.73925	NA	level4	NA	NA	NA	NA
C37 H65 C	0.002583	3.864359	NA	level4	NA	NA	NA	NA
C12 H18 C	3.76E-05	0.155284	NA	level4	HMDB	NA	HMDB025	NA
C27 H40 N	0.000876	1.683259	NA	level4	NA	NA	NA	NA
C22 H32 C	-0.00014	-0.44069	NA	level4	HMDB	NA	HMDB025	NA
C30 H44 C	0.000348	0.769731	NA	level4	HMDB	NA	HMDB024	NA
C22 H30 N	-0.00209	-4.99021	NA	level4	HMDB	NA	HMDB025	NA
C35 H44 C	-0.00138	-2.60346	NA	level4	NA	NA	NA	NA
C27 H44 C	0.000285	0.63613	NA	level4	NA	NA	NA	NA
C30 H62 C	-0.00127	-1.66993	NA	level4	HMDB	NA	HMDB025	NA
C21 H20 C	-0.00061	-1.90471	NA	level4	NA	NA	NA	NA
C20 H27 B	3.97E-05	0.100844	NA	level4	NA	C15371	NA	NA
C27 H46 C	-0.00014	-0.30865	NA	level4	NA	NA	NA	NA
C26 H42 C	7.23E-05	0.187195	NA	level4	NA	C21964	NA	FA Fatty a
C15 H16 C	-1.07E-05	-0.04708	NA	level4	HMDB	NA	HMDB003	Benzenoid
C39 H72 N	-0.00304	-3.75739	NA	level4	HMDB	NA	HMDB006	Organic ox
C28 H35 C	0.00167	3.492564	NA	level4	HMDB	NA	HMDB025	NA
C26 H39 N	0.000147	0.330348	NA	level4	NA	NA	NA	NA
C17 H32 C	-3.13E-05	-0.10426	NA	level4	NA	NA	NA	NA
C20 H23 N	-0.00135	-3.70563	NA	level4	HMDB	NA	HMDB025	NA
C29 H44 C	-0.00138	-2.5702	NA	level4	HMDB	NA	HMDB024	NA
C21 H42 N	0.000766	1.58598	NA	level4	NA	NA	NA	NA
C29 H47 N	0.000368	0.705616	NA	level4	NA	C15683	NA	NA
C25 H35 N	0.000207	0.44842	NA	level4	NA	NA	NA	NA

C16 H19 C	7.76E-05	0.282994	NA	level4	KEGG	C06905	NA	NA
C22 H47 C	0.000363	0.772554	NA	level4	NA	NA	NA	NA
C23 H38 C	0.000113	0.285693	NA	level4	HMDB	NA	HMDB025	NA
C27 H42 C	0.000104	0.241861	43.71143	level4	Public	NA	NA	NA
C28 H41 N	0.000496	1.099104	NA	level4	HMDB	NA	HMDB025	NA
C48 H92 N	0.001043	1.112042	NA	level4	HMDB	NA	HMDB025	NA
C43 H58 N	-0.00139	-1.68874	NA	level4	HMDB	NA	HMDB025	NA
C27 H36 N	0.002447	4.631428	NA	level4	HMDB	NA	HMDB025	NA
C17 H25 N	-4.04E-05	-0.14668	NA	level4	HMDB	NA	HMDB025	NA
C12 H16 C	37.95739	139503.3	3.416108	level4	Public	C06186	HMDB002	Organic ox
C25 H34 C	17.0281	36839.38	2.940696	level4	Public	NA	NA	NA
C20 H30 C	21.98435	65779.17	33.93553	level3	mzCloud	NA	HMDB000	Lipids and
C31 H35 C	-0.00032	-0.57863	NA	level4	HMDB	NA	HMDB025	NA
C34 H34 N	0.000211	0.374404	NA	level4	HMDB	NA	HMDB000	Organohe
C30 H46 N	0.000168	0.319521	NA	level4	HMDB	NA	HMDB025	NA
C26 H44 C	0.000246	0.607835	NA	level4	HMDB	NA	HMDB024	NA
C13 H20 N	0.001412	4.302534	NA	level4	NA	C22024	NA	NA
C32 H34 N	0.000371	0.649801	NA	level4	HMDB	NA	HMDB025	NA
C26 H55 C	0.000802	1.523575	NA	level4	NA	NA	NA	NA
C22 H32 C	37.95731	105369.6	9.977402	level4	Public	NA	NA	NA
C27 H40 C	-0.00017	-0.43606	NA	level4	HMDB	NA	HMDB024	NA
C20 H34 C	37.95745	112218.6	29.76931	level4	Public	NA	NA	NA
C19 H20 C	-36.0219	-104677	34.86488	level4	Public	NA	NA	NA
C11 H12 C	0.000245	1.392309	NA	level4	NA	C14111	NA	NA
C30 H30 C	0.002286	3.926395	NA	level4	NA	NA	NA	NA
C28 H40 C	-0.00066	-1.43829	NA	level4	NA	NA	NA	NA
C4 H8 N2	-18.0104	-136387	21.33117	level3	BMDB	C02642	HMDB000	Organic ac
C6 H9 N C	0.000247	1.554205	NA	level4	HMDB	NA	HMDB025	NA
C26 H44 C	0.000296	0.677448	NA	level4	HMDB	NA	HMDB000	Lipids and
C39 H67 C	0.002118	2.62667	NA	level4	NA	NA	NA	NA
C29 H46 C	0.001058	2.156926	NA	level4	NA	NA	NA	NA
C25 H38 C	-4.29E-05	-0.12097	NA	level4	NA	NA	NA	NA
C27 H32 C	-0.00068	-1.68682	NA	level4	NA	C14645	NA	NA
C25 H36 C	0.000143	0.372752	NA	level4	HMDB	NA	HMDB025	NA
C24 H34 N	6.30E-05	0.128456	NA	level4	HMDB	NA	HMDB001	Benzenoid
C42 H50 N	-4.99E-06	-0.00687	NA	level4	KEGG	C09323	NA	Alkaloids
C17 H26 C	0.000118	0.449879	NA	level4	NA	NA	NA	NA
C46 H73 C	0.001976	2.419949	NA	level4	HMDB	NA	HMDB001	Lipids and
C25 H30 N	0.000493	1.104167	NA	level4	HMDB	NA	HMDB025	NA
C22 H42 N	-0.00893	-16.1055	40.73425	level3	mzCloud	C12661	HMDB000	Organic ac
C25 H27 N	9.64E-05	0.222452	NA	level4	HMDB	NA	HMDB024	NA
C44 H68 N	0.000943	1.176592	NA	level4	NA	NA	NA	NA
C27 H48 C	-0.00084	-1.68612	NA	level4	NA	NA	NA	NA
C23 H32 C	-0.00011	-0.33376	NA	level4	HMDB	NA	HMDB024	NA
C31 H40 C	-0.00024	-0.527	NA	level4	HMDB	NA	HMDB025	NA
C29 H44 C	-0.00191	-3.9114	NA	level4	HMDB	NA	HMDB024	NA
C26 H42 C	-1.94E-05	-0.04827	NA	level4	HMDB	NA	HMDB025	NA
C8 H15 N	0.000189	0.855314	NA	level4	HMDB	NA	HMDB025	NA
C51 H93 C	-0.00083	-0.88909	NA	level4	NA	NA	NA	NA
C23 H34 C	-0.00012	-0.3446	NA	level4	HMDB	NA	HMDB025	NA
C20 H43 N	-0.00034	-1.08949	NA	level4	NA	NA	NA	NA
C10 H8 Cl	4.71E-05	0.19852	NA	level4	NA	C18929	NA	NA
C30 H46 C	-5.11E-05	-0.10863	39.46511	level4	Public	NA	NA	NA
C45 H83 C	0.000966	1.185679	NA	level4	NA	NA	NA	NA
C8 H13 N	0.000278	1.366533	NA	level4	HMDB	NA	HMDB024	NA
C33 H57 C	-0.00229	-3.73527	NA	level4	NA	NA	NA	NA
C24 H28 C	9.61E-05	0.234903	NA	level4	HMDB	NA	HMDB025	NA

Sub Class	Class	Final Class	Super Path	Sub Pathw	Pathway											
						CC	CC	CCA	CC	CC	CC	CC	CC	CC	CC	CC	CC
						pC	pC	pCC/	pC	pC	iC(	pC	iC(	pC	pC	pC	pC
						p.R								p.Y			
						p.A											
							p.F										
														p.R			
							p.R										
						p.R											
																p.T27	
							p.R										
						p.C								p.C			
						p.P				p.S							
						p.G		p.R1			p.R17		p.V	p.E		p.R17	
						p.T		p.Q1								p.Q16	
									p.G						p.G		
						##	##	###	##	##	40	##	##	##	##	##	##
						##	4	6.4	14	4	2	4	##	25	2	2	48
						##	38	31	26	37	37	28	36	36	44	35	40
						##	62	277	##	##	8	##	##	##	##	##	##
						##	16	127	98	34	##	53	35	##	##	78	81
						##	19	251	##	39	37	87	43	73	##	##	95
						##	4	3.7	4	9	4	18	4	5	5	3	6
						##	1	1.8	2	4	1	3	7	2	5	2	2
						##	3	189	##	12	6	##	##	##	##	##	##
						Fe	Fe	Fem	Fe	Ma	Fe	Fe	Fe	Ma	Ma	Ma	Ma
						##	##	66	##	##	##	##	##	##	##	##	##
						CC	CC	CCA	CC	CC	CC	CC	CC	CC	CC	CC	CC
						T2	T2	T3N	T2	T3	T3	T3	T3	T3	T3	T3	T3I
						1	1	1	1	1	1	1	1	1	1	1	0
						##	##	###	##	##	##	##	##	##	##	##	##
						##	##	###	##	##	##	##	##	##	##	##	##
						##	##	###	##	##	##	##	##	##	##	##	##
						##	##	###	##	##	##	##	##	##	##	##	##
						##	##	###	##	##	##	##	##	##	##	##	##
						##	##	###	##	##	##	##	##	##	##	##	##
						5	1	2.2	3	7	10	6	2	10	10	13	3
						4	2	1.6	12	5	15	6	9	4	0	20	2
						7	8	15	25	15	20	28	56	4	10	34	8
						6	0	3.4	9	10	2	3	10	3	4	17	3
						21	1	11	6	13	4	9	20	1	2	36	10
						8	7	21	17	24	4	13	11	15	15	17	10
						15	9	24	10	38	24	22	17	16	22	27	22
						7	14	27	9	16	5	41	10	18	8	30	8
						##	##	###	##	##	##	##	##	##	##	##	##
						##	##	###	##	##	##	##	##	##	##	##	##
						##	##	###	##	##	##	##	##	##	##	##	##
						##	##	###	##	##	##	##	##	##	##	##	##
						32	49	51	##	##	##	##	##	##	##	53	##
						##	##	147	24	##	##	82	10	22	##	##	##
						56	##	96	##	##	##	##	##	##	##	##	##

Toluenes	Benzene a	Benzene a	NA	NA	NA	1	2	0.7	1	1	1	0	2	2	2	1	2
						37	56	79	59	##	##	##	##	##	##	##	##
Hydroxyci	Cinnamic a	Polyketide	NA	NA	NA	0	1	0.3	0	2	0	3	0	0	1	1	2
Arylsulfate	Organic su	Organic ac	NA	NA	NA	1	0	0.9	1	1	0	3	0	0	0	0	10
Arylsulfate	Organic su	Organic ac	NA	NA	NA	1	1	0.5	2	1	1	3	1	0	0	1	1
NA	NA	NA	NA	NA	NA	1	1	1.6	1	1	1	1	1	1	1	1	1
NA	NA	NA	NA	NA	NA	1	1	0.6	2	1	1	1	1	1	1	1	1
Fatty acids	Fatty Acyls	Fatty acyls	NA	NA	NA	1	1	1.3	1	1	1	1	1	1	1	1	1
NA	NA	NA	NA	NA	NA	1	1	0.6	2	1	1	1	0	1	1	1	2
Fatty acids	Fatty Acyls	Fatty acyls	NA	NA	NA	0	4	1.1	0	2	1	3	0	0	1	1	2
NA	NA	NA	NA	NA	NA	0	1	0.2	0	3	0	3	1	0	1	1	3
NA	NA	NA	NA	NA	NA	1	1	0.5	1	1	1	1	1	1	0	1	0
NA	NA	NA	NA	NA	NA	1	1	0.6	3	1	1	1	1	1	1	1	2
NA	NA	NA	NA	NA	NA	1	1	0.8	1	2	1	1	0	0	1	0	2
NA	NA	NA	NA	NA	NA	1	1	1.1	1	1	1	1	1	1	1	1	2
NA	NA	NA	NA	NA	NA	1	0	0.3	2	1	2	4	0	0	1	2	3
NA	NA	NA	NA	NA	NA	1	1	0.6	2	0	1	1	0	1	1	1	2
NA	NA	NA	NA	NA	NA	1	1	0.6	2	1	1	1	1	1	1	1	2
Benzoic ac	Benzene a	Benzene a	NA	NA	NA	0	0	0.9	1	5	1	6	1	1	0	1	1
						0	0	0	0	0	0	0	0	0	23	0	0
NA	NA	NA	NA	NA	NA	1	1	1.4	1	1	1	1	1	1	1	1	2
NA	NA	NA	NA	NA	NA	1	1	0.7	2	1	1	1	1	1	1	1	2
NA	NA	NA	NA	NA	NA	1	1	0.6	2	1	1	1	1	1	1	0	6
						0	0	0	6	0	0	0	0	0	0	0	0
Amino aci	Carboxylic	Amino aci	Amino aci	Lysine deg	map00310	0	1	0.4	3	0	1	2	3	0	1	1	3
NA	NA	NA	NA	NA	NA	1	1	0.6	2	1	1	1	1	1	1	1	2
NA	Phenanthr	Benzene a	NA	NA	NA	1	1	0.5	2	1	1	1	1	1	1	1	1
NA	NA	NA	NA	NA	NA	0	0	0.4	4	3	0	4	2	0	0	1	1
NA	NA	NA	NA	NA	NA	1	1	0.8	1	1	1	1	1	1	1	1	35
NA	NA	NA	NA	NA	NA	0	0	2.7	1	3	1	0	0	0	1	2	0
NA	NA	NA	Metabolis	Biosynthes	NA	1	2	0.6	2	1	1	1	0	1	1	0	2
NA	NA	NA	NA	NA	NA	1	1	1.3	1	2	2	4	1	1	1	1	0
NA	NA	NA	NA	NA	NA	1	2	0.6	2	1	1	1	1	2	1	1	18
NA	NA	NA	NA	NA	NA	1	1	1	1	1	1	1	1	1	1	1	1
NA	NA	NA	NA	NA	NA	0	1	1.4	2	2	1	2	2	1	1	1	1
NA	NA	NA	NA	NA	NA	0	1	0.2	1	1	2	1	1	0	1	0	5
Organosul	Organic su	Organic ac	Energy me	Sulfur met	map00920	1	0	0.2	1	1	1	1	1	0	1	1	2
NA	NA	NA	NA	NA	NA	1	3	0.4	0	2	##	14	1	0	0	0	4
NA	NA	NA	NA	NA	NA	1	1	0.8	1	1	55	1	1	1	1	1	1
Amino aci	Carboxylic	Amino aci	NA	NA	NA	1	1	0.8	1	1	1	3	1	1	2	1	1
NA	NA	NA	NA	NA	NA	1	1	1.6	0	1	1	3	1	1	1	1	0
NA	Phenylpro	Phenylpro	NA	NA	NA	0	5	0.3	0	2	0	2	1	0	1	0	0
NA	NA	NA	NA	NA	NA	1	1	1.3	1	1	1	1	1	1	1	1	1
NA	NA	NA	NA	NA	NA	1	1	0.7	1	1	14	1	1	1	1	1	2
NA	NA	NA	NA	NA	NA	1	1	0.9	1	0	1	2	1	0	0	0	1
Carbohydr	Organoox	Carbohydr	NA	NA	NA	1	1	1	1	1	1	1	1	1	1	1	1
Amino aci	Carboxylic	Amino aci	NA	NA	NA	1	1	1.1	1	1	0	3	1	1	1	1	2
						0	0	0	0	0	0	0	0	0	0	0	0
NA	NA	NA	NA	NA	NA	1	1	1.1	1	1	1	1	1	1	1	1	2
NA	NA	NA	NA	NA	NA	0	1	1.3	1	1	0	2	0	1	1	1	0
Isoflavono	Isoflavono	Flavonoids	NA	NA	NA	1	1	1.4	1	1	1	1	1	1	1	1	1
NA	NA	NA	NA	NA	NA	1	1	1	2	1	0	1	1	1	1	1	1
NA	NA	NA	NA	NA	NA	1	1	0.9	1	1	1	1	1	1	1	1	2
NA	NA	NA	NA	NA	NA	1	1	1	1	1	1	1	2	1	1	1	2
Lysergic ac	Ergoline al	Alkaloids a	NA	NA	NA	1	1	1.5	1	1	1	1	1	1	1	1	1
NA	NA	NA	NA	NA	NA	1	1	0.5	1	0	0	0	1	0	0	1	1
						0	0	0	0	0	0	0	0	0	0	0	0
NA	NA	NA	NA	NA	NA	1	1	1.6	1	1	1	1	1	1	5	1	1

NA	NA	NA	NA	NA	NA	1	1	1.8	1	2	2	1	1	1	1	1	0
NA	NA	NA	NA	NA	NA	1	1	1.5	1	1	1	1	2	1	1	1	1
Anthraqui	Anthracen	Quinone	NA	NA	NA	1	1	1	1	1	0	1	1	1	1	2	1
NA	NA	NA	NA	NA	NA	1	1	0.6	2	1	1	1	0	1	1	1	2
Carbohydr	Organoox	Carbohydr	NA	NA	NA	1	1	0.5	1	0	1	1	1	1	1	2	1
Deoxy sug	Monosacc	Carbohydr	NA	NA	NA	0	1	0.6	3	0	1	0	1	2	0	4	1
Carbohydr	Organoox	Carbohydr	NA	NA	NA	0	1	0.6	3	0	1	0	1	2	0	4	1
Indole alka	Alkaloids (	Alkaloids	Biosynthes	Indole alka	map0110(	1	1	0.8	1	1	1	1	1	1	1	1	1
NA	NA	NA	NA	NA	NA	1	1	0.5	1	1	2	2	3	0	1	1	1
						32	9	593	18	0	27	8	68	53	82	##	0
NA	NA	NA	NA	NA	NA	1	1	1.3	1	1	1	1	1	1	1	1	1
NA	NA	NA	NA	NA	NA	1	3	0.7	1	1	1	1	1	2	1	0	1
NA	NA	NA	NA	NA	NA	0	1	0.9	2	1	2	2	1	0	0	1	1
NA	NA	NA	NA	NA	NA	1	1	0.9	1	1	1	1	1	1	1	1	2
NA	NA	NA	NA	NA	NA	1	1	0.6	1	1	1	1	1	1	1	1	2
NA	NA	NA	NA	NA	NA	1	1	0.8	1	1	1	0	1	0	1	1	1
NA	NA	NA	NA	NA	NA	1	1	0.5	1	1	1	1	2	1	1	1	1
NA	NA	NA	NA	NA	NA	1	2	1.1	1	1	1	1	1	2	1	1	1
NA	NA	NA	NA	NA	NA	0	2	0.4	1	2	0	7	1	0	1	0	1
NA	Phenylpro	Phenylpro	NA	NA	NA	0	3	0.9	2	1	2	3	1	0	0	1	0
NA	NA	NA	NA	NA	NA	1	0	0.2	0	0	2	30	69	22	33	1	33
Fatty amid	Fatty Acyls	Fatty acyls	NA	NA	NA	1	1	1.4	1	1	1	0	1	0	2	0	0
						0	0	0	0	0	0	0	0	0	0	0	0
						0	0	0	8	11	0	10	7	30	0	0	0
NA	NA	NA	NA	NA	NA	0	1	0.7	0	0	0	0	22	1	42	0	9
NA	NA	NA	NA	NA	NA	0	2	0.4	1	2	0	7	1	0	1	0	1
NA	NA	NA	NA	NA	NA	1	1	0.8	1	1	1	1	1	1	1	1	2
NA	NA	NA	NA	NA	NA	1	1	1.1	1	1	1	1	1	1	1	1	2
NA	NA	NA	NA	NA	NA	1	1	0.9	1	1	1	1	1	1	1	1	1
FA0200 O	FA02 Octa	Fatty acyls	Lipid meta	alpha-Linc	map00592	7	1	1	0	10	6	15	0	0	0	1	0
NA	NA	NA	NA	NA	NA	1	1	1.6	1	1	1	1	1	1	1	1	1
Purines an	Imidazopy	Purines an	NA	NA	NA	1	1	1.1	4	1	1	1	1	1	1	1	1
NA	Tetralins	Benzene a	NA	NA	NA	2	0	0.9	1	3	1	3	0	0	1	2	2
NA	NA	NA	NA	NA	NA	2	1	0.6	1	2	2	1	1	1	1	1	1
NA	NA	NA	NA	NA	NA	1	1	1.1	1	1	1	1	2	1	1	1	2
NA	NA	NA	Metabolis	Biosynthes	NA	1	1	1.1	1	1	1	1	2	1	1	1	2
NA	NA	NA	NA	NA	NA	1	1	1.5	1	1	1	1	1	1	1	1	2
NA	NA	NA	NA	NA	NA	0	0	0.4	0	0	0	0	2	20	0	0	0
NA	NA	NA	NA	NA	NA	1	0	0.5	3	1	0	1	0	6	0	0	0
NA	NA	NA	NA	NA	NA	1	1	1.6	1	1	1	1	1	1	1	1	1
NA	NA	NA	NA	NA	NA	1	1	1.3	1	1	1	1	1	1	1	1	1
						0	0	0	0	0	0	0	0	0	0	0	0
NA	NA	NA	NA	NA	NA	1	3	0.5	1	1	1	1	1	3	1	0	2
						0	0	0	0	0	0	0	0	0	0	0	0
NA	NA	NA	NA	NA	NA	0	1	0.4	1	3	0	10	0	0	1	1	3
NA	NA	NA	NA	NA	NA	1	1	0.7	1	2	6	4	1	1	3	1	1
Amino aci	Carboxylic	Amino aci	Nucleotide	Purine me	map00230	1	1	1.7	1	0	2	3	1	1	1	3	2
FA0302 Le	FA03 Eicos	Fatty acyls	Lipid meta	Arachidon	map0059(	1	1	0.8	1	1	1	1	1	1	1	1	2
NA	NA	NA	NA	NA	NA	1	0	1.8	0	1	0	1	1	1	8	1	1
NA	NA	NA	NA	NA	NA	1	2	1.4	1	1	1	1	1	2	1	1	1
NA	NA	NA	NA	NA	NA	1	1	0.5	1	1	0	1	1	1	2	1	1
Hydroxypy	Pyridines a	Pyridine ar	NA	NA	NA	1	1	0.9	1	1	1	1	1	0	1	1	3
NA	NA	NA	NA	NA	NA	1	0	1	3	0	0	0	1	0	1	1	1
NA	NA	NA	NA	NA	NA	1	1	0.9	1	1	1	1	1	1	1	1	1
NA	NA	NA	NA	NA	NA	1	1	0.9	2	1	1	1	1	1	1	1	1
NA	NA	NA	NA	NA	NA	1	1	0.9	1	1	1	1	1	1	1	1	2
Purines an	Imidazopy	Purines an	NA	NA	NA	4	0	0.6	1	3	0	4	0	0	1	0	2
NA	NA	NA	NA	NA	NA	1	1	0.9	1	2	1	2	0	1	1	1	1

NA	NA	NA	NA	NA	NA	2	1	0.4	1	4	0	7	0	1	1	0	0
NA	NA	NA	NA	NA	NA	1	1	1.4	1	1	1	1	2	1	1	1	1
Carbonyl (	Organoox	Organic o:	NA	NA	NA	1	1	0.8	1	1	1	1	1	1	1	3	0
NA	NA	NA	NA	NA	NA	1	1	0.8	1	1	1	1	1	1	1	1	2
						0	0	0	0	0	0	0	0	0	0	0	0
NA	NA	NA	NA	NA	NA	1	1	1.2	1	1	1	0	1	1	1	2	1
						0	0	0	0	0	0	0	0	0	0	0	0
						0	0	0	0	0	0	0	0	0	0	0	0
NA	NA	NA	NA	NA	NA	1	1	0.8	1	1	1	1	1	1	1	1	2
Isoflavono	Isoflavono	Flavonoids	NA	NA	NA	1	1	0.6	1	1	2	1	0	2	1	1	1
NA	NA	NA	NA	NA	NA	1	1	1.4	1	1	1	1	1	1	1	1	1
NA	NA	NA	NA	NA	NA	1	1	0.7	0	1	1	0	1	1	0	1	2
NA	Nucleosid	Nucleic ac	Nucleotid	Pyrimidine	map00240	2	0	0.1	0	2	1	1	0	0	0	1	0
						0	0	0	0	0	0	0	0	0	0	0	0
NA	NA	NA	NA	NA	NA	1	1	2.4	1	1	1	2	1	1	1	1	1
NA	NA	NA	NA	NA	NA	1	1	1.4	1	##	1	2	1	1	1	5	##
NA	NA	NA	NA	NA	NA	1	1	0.7	1	1	1	1	2	1	2	1	1
						0	67	29	45	0	0	73	0	0	0	0	63
Carbohydr	Organoox	Carbohydr	NA	NA	NA	1	3	0.8	1	1	1	1	1	3	1	1	2
NA	NA	NA	NA	NA	NA	1	1	0.8	1	1	1	1	1	1	1	1	1
NA	NA	NA	NA	NA	NA	2	0	0.3	6	4	2	5	0	0	0	2	2
NA	NA	NA	NA	NA	NA	2	1	0.7	2	2	0	1	1	1	1	1	2
Hydroxycii	Cinnamic a	Polyketide	NA	NA	NA	1	1	1.3	2	0	2	0	1	0	1	1	0
Benzoic ac	Benzene a	Benzene a	Xenobiotic	Aminoben	NA	1	0	9.1	2	5	1	0	0	0	0	1	2
Isoflav-2-(	Isoflavono	Flavonoids	NA	NA	NA	0	0	2.7	15	1	1	0	1	1	22	5	1
NA	NA	NA	NA	NA	NA	1	2	1.5	1	1	1	1	1	1	1	1	1
NA	NA	NA	NA	NA	NA	0	0	1.7	0	5	0	1	1	1	1	1	1
Amino aci	Carboxylic	Amino aci	Amino aci	Cysteine a	map00270	2	1	0.3	1	1	1	1	1	0	1	0	1
NA	NA	NA	NA	NA	NA	1	1	0.7	3	1	0	0	1	0	1	1	0
NA	NA	NA	NA	NA	NA	1	1	0.7	1	1	2	1	1	1	1	1	2
NA	NA	NA	NA	NA	NA	1	0	1.4	1	2	1	1	1	0	1	2	0
NA	NA	NA	NA	NA	NA	0	2	0.4	1	1	0	8	0	0	1	0	1
						0	0	0	0	0	0	0	0	0	0	0	0
						0	0	0	0	0	0	0	47	0	0	0	0
Indolyl car	Indoles an	Indole anc	NA	NA	NA	0	1	0.3	2	0	1	26	1	0	2	1	0
Phenylpyr	Benzene a	Benzene a	Amino aci	Phenylalar	map0036C	1	3	1.8	1	2	1	2	1	1	1	1	1
NA	NA	NA	NA	NA	NA	0	0	0.4	4	1	0	1	0	0	5	3	26
Amino aci	Carboxylic	Amino aci	Metabolisı	Glutathion	map0048C	1	0	1.1	4	13	1	19	1	1	1	1	1
NA	NA	NA	NA	NA	NA	1	1	0.8	1	1	1	1	1	1	1	1	2
NA	NA	NA	NA	NA	NA	1	0	0.8	1	1	2	1	1	1	1	2	0
NA	NA	NA	NA	NA	NA	1	1	0.8	1	1	1	1	1	1	1	1	2
NA	NA	NA	NA	NA	NA	1	1	0.9	1	1	1	1	1	1	1	1	2
NA	NA	NA	NA	NA	NA	1	1	0.6	2	1	1	1	1	1	1	1	1
NA	NA	NA	NA	NA	NA	1	1	0.7	1	1	1	1	1	1	1	1	2
						0	0	0	0	0	0	0	0	0	0	0	0
NA	NA	NA	NA	NA	NA	0	1	0.4	1	2	1	8	1	0	0	1	2
NA	NA	NA	NA	NA	NA	1	1	1	7	1	1	1	22	1	3	1	1
NA	NA	NA	NA	NA	NA	1	1	0.7	1	1	2	1	1	1	1	1	2
NA	NA	NA	NA	NA	NA	1	1	1.4	1	1	1	1	1	1	1	1	1
NA	NA	NA	NA	NA	NA	1	1	0.8	1	1	1	1	1	1	1	1	2
NA	NA	NA	NA	NA	NA	1	1	0.8	1	1	1	1	1	1	1	1	2
						0	0	0	0	0	0	0	0	0	0	0	0
NA	NA	NA	NA	NA	NA	1	1	0.8	2	1	0	1	1	0	1	1	0
NA	NA	NA	NA	NA	NA	1	1	1.4	1	1	1	1	1	1	1	1	2
Piperazine	Diazinanes	Organohe	NA	NA	NA	1	1	1.3	1	1	1	1	1	1	1	1	2
NA	NA	NA	NA	NA	NA	1	1	0.7	1	1	14	2	2	1	1	1	2
Grayanoid	Diterpenoi	Terpenoid	NA	NA	NA	1	1	1.1	1	1	1	1	1	1	1	1	1
NA	NA	NA	NA	NA	NA	1	1	0.8	1	1	1	1	1	1	1	0	2

Phenylpip	Piperidine	Organohe	NA	NA	NA	1	0	0.8	1	1	0	1	0	##	1	0	13
NA	NA	NA	NA	NA	NA	1	1	1.7	1	1	1	1	1	1	1	1	1
						0	0	0	0	0	0	0	0	0	0	0	0
NA	NA	NA	NA	NA	NA	1	1	1.5	1	1	1	1	1	1	1	1	2
						0	0	0	0	0	0	0	0	0	0	0	0
Fatty alcoh	Fatty Acyls	Fatty acyls	NA	NA	NA	0	1	1.3	3	1	0	1	1	4	1	4	1
Fatty alcoh	Fatty Acyls	Fatty acyls	NA	NA	NA	2	1	0.8	1	2	3	3	0	0	0	1	1
NA	NA	NA	NA	NA	NA	1	1	0.7	1	1	5	2	2	1	1	1	2
Androstan	Steroids a	Steroids a	NA	NA	NA	1	1	0.7	1	3	1	1	1	1	1	1	1
NA	NA	NA	NA	NA	NA	1	1	0.8	1	1	1	1	1	1	1	1	2
NA	NA	NA	NA	NA	NA	1	1	1.3	1	2	1	2	11	1	1	2	1
NA	NA	NA	NA	NA	NA	1	2	0.8	2	2	1	1	1	0	1	2	1
						0	0	0	0	0	0	0	0	0	0	0	0
						0	0	0	0	0	0	0	0	0	0	0	0
NA	Purine nuc	Purines an	NA	NA	NA	1	0	5.7	0	7	1	1	2	1	0	6	##
NA	NA	NA	NA	NA	NA	1	1	1.6	1	1	1	1	1	1	1	1	1
NA	NA	NA	NA	NA	NA	1	1	0.8	1	1	1	1	1	1	1	1	2
NA	NA	NA	NA	NA	NA	1	1	0.6	2	1	1	1	1	1	1	1	1
NA	NA	NA	NA	NA	NA	1	1	1	1	1	1	1	1	1	1	1	2
						0	0	0	0	0	0	0	3	3	0	0	0
NA	NA	NA	NA	NA	NA	1	2	0.2	0	1	0	6	1	0	1	2	0
Naphthale	Naphthale	Benzene a	NA	NA	NA	0	1	0.5	2	11	1	1	3	1	4	0	3
						0	0	0	14	20	0	17	91	43	51	0	14
NA	NA	NA	NA	NA	NA	1	1	0.8	1	1	1	1	1	1	1	1	4
NA	NA	NA	NA	NA	NA	1	1	1.4	1	4	1	3	1	1	1	1	2
NA	NA	NA	NA	NA	NA	0	2	0.2	3	1	0	2	1	1	0	0	2
Anilides	Benzene a	Benzene a	NA	NA	NA	1	1	0.5	1	1	1	1	1	3	0	1	1
						0	0	0	0	0	0	0	0	0	0	0	0
						0	0	0	0	0	0	0	0	0	0	0	0
NA	NA	NA	NA	NA	NA	1	1	0.8	1	1	1	1	1	1	1	1	1
NA	NA	NA	NA	NA	NA	0	1	1.3	1	6	2	2	1	1	2	1	1
Amino aci	Carboxylic	Amino aci	Amino aci	Arginine b	map00220	1	1	0.8	2	1	3	2	1	1	1	3	5
NA	NA	NA	NA	NA	NA	1	1	1.5	1	1	1	1	1	1	1	1	8
						0	0	0	0	0	0	0	0	0	0	0	6
NA	NA	NA	NA	NA	NA	0	1	0.4	1	1	1	10	5	0	1	1	1
NA	NA	NA	NA	NA	NA	1	1	1.4	1	1	1	3	1	1	1	1	3
NA	NA	NA	NA	NA	NA	1	3	0.9	1	1	1	1	1	2	1	1	1
						0	0	0	0	0	0	0	0	0	0	0	0
						16	0	0	47	38	29	96	##	##	##	0	44
						0	0	0	0	0	0	0	0	0	0	0	0
						0	0	0	0	0	0	0	0	0	0	0	0
						0	0	0	0	0	0	0	0	0	0	0	0
						0	0	0	0	0	0	0	0	0	0	0	0
NA	NA	NA	NA	NA	NA	1	1	0.4	1	1	1	1	2	1	1	0	1
NA	NA	NA	NA	NA	NA	1	1	0.8	5	5	1	4	0	1	1	1	4
Lipoic acic	Dithiolane	Organohe	Metabolis	Lipoic acic	map00785	1	1	1.1	2	1	1	1	1	0	1	1	1
NA	NA	NA	NA	NA	NA	4	1	0.9	1	1	3	0	1	1	1	1	1
Fatty acids	Fatty Acyls	Fatty acyls	Metabolis	Biotin met	map00780	1	1	5.1	1	1	0	4	1	1	1	2	0
NA	NA	NA	NA	NA	NA	1	1	2.1	1	3	2	1	1	0	1	1	1
NA	NA	NA	NA	NA	NA	1	1	0.8	1	2	1	2	1	1	1	1	1
Glyceroph	Glyceroph	Glyceroph	NA	NA	NA	0	2	0.9	0	4	8	5	1	0	0	2	2
NA	NA	NA	NA	NA	NA	1	1	0.8	1	1	2	1	2	1	1	1	2
NA	NA	NA	NA	NA	NA	1	1	0.3	0	2	##	6	1	0	1	1	1
NA	NA	NA	NA	NA	NA	1	1	0.6	1	1	2	3	2	0	1	2	1
NA	NA	NA	NA	NA	NA	1	1	0.7	1	1	4	2	1	0	2	1	1
NA	NA	NA	NA	NA	NA	1	0	1	0	2	5	1	6	0	1	1	8
NA	NA	NA	NA	NA	NA	1	1	1.6	1	1	1	1	1	1	1	1	1
NA	NA	NA	NA	NA	NA	1	2	1.5	0	5	10	5	0	0	0	3	1

NA	NA	NA	NA	NA	NA	1	1	1.4	1	1	1	1	1	1	1	1	2
Phenylbut	Benzene a	Amines an	NA	NA	NA	2	1	1.3	2	16	##	22	1	1	1	1	2
NA	NA	NA	NA	NA	NA	1	1	5	1	4	1	8	1	1	1	3	1
Glucosinol	Glucosinol	Amino aci	NA	NA	NA	1	1	0.6	1	2	1	2	0	1	0	1	1
NA	NA	NA	NA	NA	NA	0	1	0.5	1	56	0	28	2	25	0	0	1
NA	NA	NA	NA	NA	NA	1	1	0.1	0	1	1	1	6	0	1	0	1
NA	NA	NA	NA	NA	NA	1	1	1	1	1	0	1	1	0	1	0	1
NA	NA	NA	NA	NA	NA	1	1	1.2	1	2	1	1	1	1	1	1	1
NA	NA	NA	NA	NA	NA	1	1	0.8	1	1	1	1	1	1	1	1	1
Fatty acids	Fatty Acyls	Fatty acyls	Lipid meta	Linoleic ac	map00591	1	0	0.5	1	4	49	4	1	1	1	1	1
NA	NA	NA	NA	NA	NA	1	3	0.7	1	1	1	1	1	2	1	0	1
NA	NA	NA	NA	NA	NA	1	2	1.4	1	1	1	1	1	2	1	1	1
NA	NA	NA	NA	NA	NA	1	1	0.7	2	1	1	2	1	1	1	1	1
NA	NA	NA	NA	NA	NA	1	1	0.6	1	1	2	1	1	1	1	1	2
NA	NA	NA	NA	NA	NA	0	0	0.6	1	1	0	1	1	10	0	0	1
Hydropyri	Pyridines a	Pyridine ar	NA	NA	NA	0	2	1.1	1	1	1	0	0	0	1	1	0
NA	NA	NA	NA	NA	NA	1	1	0.7	1	1	8	1	2	1	1	1	2
						0	0	0	0	0	0	0	51	28	47	0	45
						0	0	0	0	10	0	0	0	0	15	0	0
Pterocarpa	Isoflavono	Flavonoids	Biosynthes	Isoflavono	NA	1	1	1.6	1	1	1	2	1	1	1	1	1
NA	NA	NA	NA	NA	NA	1	2	0.6	2	1	1	1	0	2	1	4	2
NA	NA	NA	NA	NA	NA	1	1	1.4	1	1	1	1	1	1	1	1	1
Pseudogu	Sesquiterp	Terpenoid	NA	NA	NA	1	3	0.7	1	1	1	1	1	2	1	0	1
NA	NA	NA	NA	NA	NA	1	1	0.8	1	1	1	1	1	1	1	1	1
Carbohydr	Organoox	Carbohydr	Carbohydr	Glyoxylate	map0063C	1	0	0.7	1	1	1	1	1	0	34	10	3
NA	NA	NA	NA	NA	NA	1	1	1	7	1	1	2	3	13	2	1	1
NA	NA	NA	NA	NA	NA	1	1	2.1	1	1	1	2	2	1	1	1	1
NA	NA	NA	NA	NA	NA	1	1	0.8	1	1	1	1	1	1	1	1	2
NA	NA	NA	NA	NA	NA	1	2	2	2	1	0	6	1	1	1	2	1
Vitamin D	Steroids ar	Vitamins a	NA	NA	NA	1	2	1.4	1	1	1	1	1	2	1	1	1
NA	NA	NA	NA	NA	NA	1	1	0.9	2	2	2	2	1	1	0	1	1
NA	NA	NA	NA	NA	NA	1	3	1	1	1	1	1	1	2	1	1	1
NA	NA	NA	NA	NA	NA	1	1	1.6	1	1	1	1	1	1	1	1	1
NA	NA	NA	NA	NA	NA	1	3	1	1	1	1	1	1	2	1	1	1
						0	0	0	0	0	0	0	0	0	0	0	0
NA	NA	NA	NA	NA	NA	1	0	0.7	1	1	1	1	0	##	1	1	##
NA	NA	NA	Biosynthes	Biosynthes	NA	1	1	0.6	1	1	0	0	1	1	2	2	0
NA	NA	NA	NA	NA	NA	1	3	0.9	1	1	1	1	1	2	1	1	1
Indolizidin	Alkaloids (	Alkaloids	NA	NA	NA	1	1	2.2	1	7	1	11	1	2	1	9	1
NA	NA	NA	NA	NA	NA	1	1	0.6	1	5	1	4	1	0	1	1	5
NA	NA	NA	NA	NA	NA	1	1	1.5	1	1	1	1	1	1	1	1	2
NA	NA	NA	NA	NA	NA	0	0	1	2	0	0	0	2	2	1	1	5
NA	NA	NA	NA	NA	NA	3	2	0.3	1	2	1	2	0	0	0	1	5
NA	NA	NA	NA	NA	NA	9	1	1	1	3	0	6	1	0	0	1	2
NA	NA	NA	NA	NA	NA	1	1	1.6	1	1	1	1	1	1	1	1	1
NA	NA	NA	NA	NA	NA	1	3	0.5	1	1	1	1	1	3	1	0	2
						0	0	0	0	0	0	0	0	6	0	0	0
NA	NA	NA	NA	NA	NA	0	2	1.3	0	2	1	3	1	0	1	2	1
Pyrimidine	Diazines	Organohe	NA	NA	NA	1	1	1.6	1	2	1	2	1	1	1	1	1
NA	NA	NA	NA	NA	NA	1	2	1.5	1	1	1	1	1	1	1	1	1
NA	NA	NA	NA	NA	NA	1	1	0.8	1	1	1	1	1	1	1	1	3
NA	NA	NA	NA	NA	NA	1	1	0.8	1	3	1	0	1	1	1	1	1
NA	NA	NA	NA	NA	NA	1	0	2.8	1	4	1	1	2	1	2	1	1
NA	NA	NA	NA	NA	NA	1	1	0.5	3	1	1	3	3	1	1	1	1
NA	NA	NA	NA	NA	NA	1	1	0.6	1	1	1	1	0	3	1	2	1
NA	NA	NA	NA	NA	NA	0	1	1.7	1	1	1	2	2	1	2	1	1
Isoflavono	Isoflavono	Flavonoids	NA	NA	NA	1	1	4.8	1	1	1	0	1	1	1	39	7
NA	NA	NA	NA	NA	NA	1	0	0.4	3	0	1	0	1	0	1	1	1

Amino aci	Carboxylic	Amino aci	Amino aci	Lysine deg	map00310	2	1	1	1	1	0	8	1	2	1	1	1
FA0301 Pr	FA03 Eicos	Fatty acyls	Lipid meta	Arachidon	map00590	1	2	1.3	1	3	5	2	1	1	1	1	1
NA	Phenol eth	Benzene a	NA	NA	NA	1	1	1.5	1	1	3	1	1	1	1	1	1
NA	NA	NA	NA	NA	NA	1	1	1	1	1	1	1	1	1	1	1	2
NA	NA	NA	NA	NA	NA	1	1	0.1	0	1	1	1	6	0	1	0	1
Benzenedi	Phenols	Phenols ar	NA	NA	NA	1	2	1.5	1	1	1	1	1	1	1	1	1
						0	0	0	0	0	0	0	0	0	0	0	0
Fatty acids	Fatty Acyls	Fatty acyls	Carbohydr	C5-Branch	map0110(	1	1	0.9	1	1	1	2	1	0	1	1	2
NA	NA	NA	NA	NA	NA	1	0	0.8	0	1	0	4	2	2	0	0	0
NA	NA	NA	NA	NA	NA	1	1	0.9	1	1	1	1	1	1	1	1	1
						0	0	0	4	0	0	0	0	0	0	0	0
NA	NA	NA	NA	NA	NA	1	1	0.9	1	2	1	2	0	1	1	0	1
Purines an	Imidazopy	Purines an	Nucleotide	Purine me	map00230	1	2	0.3	0	1	2	1	1	1	0	1	2
NA	NA	NA	NA	NA	NA	1	1	0.5	1	1	1	2	2	1	0	1	76
NA	NA	NA	NA	NA	NA	0	0	0.4	2	0	19	1	1	0	0	0	2
NA	NA	NA	NA	NA	NA	1	1	1.6	1	1	1	1	1	1	1	1	2
NA	NA	NA	NA	NA	NA	0	0	1.4	1	7	1	2	0	0	1	1	13
NA	NA	NA	NA	NA	NA	1	0	4.6	1	42	1	0	2	1	1	13	##
NA	NA	NA	NA	NA	NA	0	3	0.7	1	1	1	1	1	3	1	1	2
NA	NA	NA	NA	NA	NA	1	1	1.3	1	1	1	1	1	1	1	1	1
Benzonitri	Benzene a	Benzene a	NA	NA	NA	0	4	0.5	1	7	0	12	1	1	0	1	0
Monorady	Glycerolipi	Glycerolipi	NA	NA	NA	0	1	0.4	1	3	1	10	1	0	1	0	3
NA	NA	NA	NA	NA	NA	1	1	1.1	1	1	2	1	1	1	1	2	1
Fatty acids	Fatty Acyls	Fatty acyls	Xenobiotic	Drug meta	map00982	1	1	0.3	1	0	1	10	0	1	1	0	1
Others	Others	Marine bio	NA	NA	NA	1	1	1.2	1	1	1	2	1	1	1	1	1
NA	NA	NA	NA	NA	NA	0	0	1.2	1	1	1	1	1	1	0	1	4
Pyrazoles	Azoles	Organohe	NA	NA	NA	1	1	1.5	1	1	1	1	1	1	1	1	1
Fatty acids	Fatty Acyls	Fatty acyls	NA	NA	NA	1	2	1.5	1	1	1	1	1	2	1	1	1
NA	NA	NA	NA	NA	NA	1	1	0.8	1	1	1	1	1	1	1	1	2
Fatty acids	Fatty Acyls	Fatty acyls	NA	NA	NA	1	1	2.7	0	1	1	4	2	2	1	1	2
Fatty acids	Fatty Acyls	Fatty acyls	NA	NA	NA	1	1	0.8	5	15	0	14	1	0	2	0	2
						0	0	0	18	17	0	20	##	33	86	0	0
Amino aci	Carboxylic	Amino aci	NA	NA	NA	1	2	1	1	1	1	8	1	1	1	2	1
Clerodane	Diterpenoi	Terpenoid	NA	NA	NA	1	1	0.9	1	1	1	1	1	1	1	1	2
Monorady	Glycerolipi	Glycerolipi	NA	NA	NA	1	1	1.5	1	1	1	1	1	1	1	1	1
NA	NA	NA	NA	NA	NA	1	1	1.2	1	2	1	1	1	1	0	1	1
Benzenesu	Benzene a	Benzene a	NA	NA	NA	1	1	0.8	1	0	4	2	0	0	1	0	0
NA	NA	NA	Metabolism	Biosynthes	NA	1	1	2	1	1	1	1	6	1	1	1	25
NA	NA	NA	NA	NA	NA	1	1	0.9	1	1	1	1	1	1	1	1	1
Oxosteroid	Steroids an	Steroids an	NA	NA	NA	1	0	0.1	13	11	0	14	5	0	0	0	13
NA	NA	NA	NA	NA	NA	1	1	0.5	1	0	1	0	1	1	1	1	0
NA	NA	NA	Lipid meta	Cutin, sub	NA	12	0	0.7	2	17	43	23	0	0	0	6	1
NA	NA	NA	NA	NA	NA	1	1	0.6	1	1	1	1	0	13	0	1	1
NA	NA	NA	NA	NA	NA	1	3	1.6	18	13	0	5	0	0	8	0	27
						0	0	0	0	0	0	0	0	0	0	0	0
						0	0	0	0	0	0	0	0	0	0	0	0
NA	NA	NA	NA	NA	NA	1	0	0.2	1	0	0	1	3	17	0	0	1
Diphenylm	Benzene a	Benzene a	NA	NA	NA	1	1	0.9	1	1	##	1	2	1	1	1	4
NA	NA	NA	NA	NA	NA	1	1	1	1	1	1	1	1	1	2	2	2
Medium-c	Hydroxy a	Organic ac	NA	NA	NA	1	2	0.7	6	26	0	25	1	0	3	0	4
						0	0	0	0	0	2	0	0	0	0	0	0
NA	NA	NA	NA	NA	NA	1	1	0.7	2	3	2	2	1	1	0	1	1
Amines	Organonit	Amines	NA	NA	NA	1	1	3.3	1	1	1	3	1	6	1	1	1
Terpene g	Prenol lipi	Terpenoid	NA	NA	NA	1	3	0.9	1	1	1	1	1	3	1	1	2
NA	NA	NA	NA	NA	NA	1	1	1	0	2	89	1	1	1	1	2	1
NA	Tetralins	Benzene a	NA	NA	NA	1	0	0.4	1	2	3	0	1	1	1	1	0
NA	NA	NA	NA	NA	NA	1	1	1.1	2	1	1	1	1	0	1	1	0
NA	NA	NA	NA	NA	NA	1	1	0.6	1	1	1	1	1	1	1	0	1

NA	NA	NA	NA	NA	NA	1	1	0.6	1	1	1	1	4	1	1	1	0
NA	NA	NA	NA	NA	NA	1	1	0.6	1	3	1	18	0	0	1	1	2
NA	NA	NA	NA	NA	NA	1	2	1	1	1	1	1	1	1	1	1	1
Amino aci	Carboxylic	Amino aci	NA	NA	NA	1	1	1.1	2	1	1	1	2	1	1	1	2
NA	NA	NA	NA	NA	NA	1	1	1	1	1	2	1	3	1	1	1	2
NA	NA	NA	NA	NA	NA	1	0	0.7	1	12	2	8	1	0	0	0	5
NA	NA	NA	NA	NA	NA	3	0	0.2	0	0	1	0	1	1	3	0	1
NA	NA	NA	NA	NA	NA	3	1	1.2	1	2	36	9	1	1	1	2	1
						0	0	0	0	0	0	0	0	0	0	0	0
NA	NA	NA	NA	NA	NA	1	1	0.8	2	2	2	2	1	1	0	1	1
NA	NA	NA	NA	NA	NA	1	2	0.7	1	1	39	1	2	1	1	1	2
						0	0	0	0	0	0	0	0	0	0	0	0
NA	NA	NA	NA	NA	NA	1	1	1.5	1	1	11	1	1	1	1	1	1
NA	NA	NA	NA	NA	NA	1	1	0.5	1	1	1	0	2	1	1	0	1
						0	0	0	0	##	0	30	0	0	68	0	0
NA	NA	NA	NA	NA	NA	2	0	0.3	7	2	2	4	0	0	0	1	4
NA	NA	NA	NA	NA	NA	2	1	0.7	1	16	1	16	0	1	1	1	1
						0	0	0	0	0	0	0	0	0	0	0	0
NA	NA	NA	NA	NA	NA	1	0	1.4	0	1	1	2	2	1	1	1	1
Fatty acids	Fatty Acyls	Fatty acyls	NA	NA	NA	1	1	1.3	1	1	2	5	5	2	1	1	4
NA	NA	NA	NA	NA	NA	1	1	2.3	1	2	1	2	1	3	1	2	1
NA	NA	NA	NA	NA	NA	1	1	1.5	1	1	1	2	1	1	1	1	3
NA	NA	NA	NA	NA	NA	1	1	0.8	1	1	1	1	1	1	1	1	2
NA	NA	NA	NA	NA	NA	1	1	0.9	1	0	0	1	1	0	1	1	0
NA	NA	NA	Metabolisı	Phosphon	map0044C	1	1	0.8	1	2	1	1	1	1	1	1	1
NA	NA	NA	NA	NA	NA	0	1	0.5	0	3	0	8	0	0	1	1	2
						0	0	0	0	0	0	0	0	0	0	0	0
						0	0	0	0	0	0	0	0	0	0	0	0
						0	0	0	0	0	0	0	0	0	0	0	0
						0	0	0	0	0	0	0	0	0	0	0	0
						0	0	0	0	0	0	0	0	0	0	0	0
						0	0	0	0	0	0	0	0	0	0	0	0
						0	0	0	0	0	0	0	0	0	0	0	0
						0	0	0	0	0	0	0	0	0	0	0	0
						0	0	0	0	0	0	0	0	0	0	0	0
						0	0	0	0	0	0	0	0	0	0	0	0
						0	0	0	0	0	0	0	0	0	0	0	0
						0	0	0	0	0	0	0	0	0	0	0	0
						0	0	0	0	0	0	0	0	0	0	0	0
Medium-c	Hydroxy a	Organic ac	NA	NA	NA	1	2	0.7	8	24	0	25	1	0	4	0	3
Carboxylic	Carboxylic	Organic ac	NA	NA	NA	1	1	0.8	1	1	1	1	1	2	1	1	1
NA	NA	NA	NA	NA	NA	1	2	0.4	1	2	1	12	0	0	1	0	0
NA	NA	NA	NA	NA	NA	1	1	1.6	1	1	1	1	1	1	1	1	1
NA	NA	NA	NA	NA	NA	1	1	0.8	5	0	1	0	1	1	1	1	0
NA	NA	NA	Metabolisı	Thiamine ı	map0073C	1	1	1.2	1	1	1	1	1	1	1	1	1
NA	NA	NA	NA	NA	NA	1	1	0.6	2	0	1	0	1	0	1	1	0
NA	NA	NA	NA	NA	NA	1	1	1.6	1	1	1	1	1	1	1	1	1
NA	NA	NA	NA	NA	NA	1	2	1	1	1	1	1	1	2	1	1	1
						0	0	0	0	0	0	0	0	0	0	0	0
						0	0	0	0	0	0	0	0	0	0	0	0
NA	Pyrenes	Benzene a	Xenobiotic	Metabolisı	map0098C	1	1	0.9	1	2	1	2	1	1	1	1	1
NA	NA	NA	NA	NA	NA	1	1	0.8	1	3	0	1	1	0	1	1	0
Benzylpipe	Piperidine:	Organohe	NA	NA	NA	1	3	1	1	1	1	1	1	2	1	1	1
NA	NA	NA	NA	NA	NA	1	1	1.1	1	0	1	0	1	47	1	1	2
NA	NA	NA	NA	NA	NA	1	1	1.8	1	2	1	2	1	1	1	1	1
Flavones	Flavonoids	Flavonoids	NA	NA	NA	1	1	0.8	1	1	1	1	2	1	1	1	9
NA	NA	NA	NA	NA	NA	##	0	2.3	4	1	35	1	4	0	4	1	0
NA	Aporphine	Alkaloids a	NA	NA	NA	0	1	0.2	3	0	1	0	1	0	0	1	0

NA	NA	NA	NA	NA	NA	0	1	0	3	0	0	5	1	0	2	1	1
NA	Organic di	Organosul	NA	NA	NA	1	1	1.3	1	1	1	1	1	1	1	2	1
NA	NA	NA	NA	NA	NA	1	1	1.5	1	1	1	4	1	1	1	1	1
NA	NA	NA	NA	NA	NA	1	1	0.5	1	1	1	1	2	0	1	0	1
Terpene g	Prenol lipi	Terpenoid	NA	NA	NA	1	3	0.9	1	1	1	1	1	3	1	1	1
NA	NA	NA	NA	NA	NA	0	1	0.1	1	0	0	0	2	0	1	4	6
NA	NA	NA	NA	NA	NA	1	1	0.8	2	2	2	2	1	1	0	1	1
NA	NA	NA	NA	NA	NA	1	0	0.5	6	##	0	##	1	0	5	0	53
Amino aci	Carboxylic	Amino aci	Metabolisı	Nicotinate	map00760	2	1	0.4	2	1	2	0	3	1	1	0	1
Pterins an	Pteridines	Pteridines	Metabolisı	Folate bio	map00790	1	1	0.6	2	0	0	1	1	3	1	1	1
NA	NA	NA	NA	NA	NA	0	1	0.1	1	2	0	14	1	0	0	0	2
NA	NA	NA	NA	NA	NA	1	1	1.2	1	1	1	1	1	1	1	1	1
NA	NA	NA	NA	NA	NA	2	1	2.2	2	2	1	1	2	0	0	1	0
NA	NA	NA	NA	NA	NA	3	1	2.1	2	1	1	1	0	1	1	1	4
NA	NA	NA	NA	NA	NA	1	0	0.5	1	0	0	1	3	1	2	1	6
NA	Stilbenes	Polyketide	NA	NA	NA	1	3	0.9	1	1	1	1	1	3	1	1	2
NA	NA	NA	NA	NA	NA	1	2	1.3	1	1	1	1	1	1	1	1	1
NA	NA	NA	NA	NA	NA	1	1	0.9	1	1	1	1	1	1	1	1	1
NA	NA	NA	NA	NA	NA	1	1	0.9	1	1	1	1	1	1	1	1	1
NA	NA	NA	NA	NA	NA	1	1	1.5	1	1	1	1	1	1	1	1	2
Terpene g	Prenol lipi	Terpenoid	NA	NA	NA	1	1	1.2	1	1	2	2	1	1	1	1	1
Amino aci	Carboxylic	Amino aci	NA	NA	NA	1	2	1.3	1	1	1	2	2	0	1	0	0
						0	0	0	0	0	0	0	0	0	0	0	0
						0	0	0	0	0	0	0	0	0	0	0	0
						0	0	0	0	0	0	0	0	0	0	0	0
						0	0	0	0	0	0	0	0	0	0	0	0
						0	0	0	0	0	0	0	0	0	0	0	0
						0	0	0	0	0	0	0	0	0	0	0	0
						0	0	0	0	0	0	0	0	0	0	0	0
						0	0	0	0	0	0	0	0	0	0	0	0
						0	0	0	0	0	0	0	0	0	0	0	0
						0	0	0	0	0	0	0	0	0	0	0	0
						0	0	0	0	0	0	0	0	0	0	0	0
						0	0	0	0	0	0	0	0	0	0	0	0
						0	0	0	0	0	0	0	0	0	0	0	0
						0	0	0	0	0	0	0	0	0	0	0	0
						0	0	0	0	0	0	0	0	0	0	0	0
						0	0	0	0	0	0	0	0	0	0	0	0
						0	0	0	0	0	0	0	0	0	0	0	0
						0	0	0	0	0	0	0	0	0	0	0	0
						0	0	0	0	0	0	0	0	0	0	0	0
NA	NA	NA	NA	NA	NA	0	5	2.9	2	1	1	7	3	0	1	4	0
NA	NA	NA	NA	NA	NA	1	1	0.6	2	2	1	3	1	1	1	1	2
NA	NA	NA	NA	NA	NA	2	2	0.3	2	6	1	1	1	1	4	2	1
NA	NA	NA	NA	NA	NA	2	1	0.7	2	1	2	1	1	5	1	1	1
NA	NA	NA	NA	NA	NA	1	1	0.5	1	0	1	1	1	12	1	1	1
NA	NA	NA	NA	NA	NA	0	0	1	1	1	1	1	0	2	1	1	1
						0	0	0	0	0	0	0	0	0	0	0	0
NA	NA	NA	NA	NA	NA	1	1	1.1	1	1	1	1	2	3	1	0	1
NA	NA	NA	NA	NA	NA	0	1	0.5	0	3	32	4	1	2	0	0	23
NA	NA	NA	NA	NA	NA	1	1	1.6	1	1	1	1	1	1	1	1	1
NA	NA	NA	NA	NA	NA	1	1	1.1	1	1	1	1	1	1	1	1	1
Amino aci	Carboxylic	Amino aci	NA	NA	NA	0	3	0.5	1	1	1	1	1	3	1	0	9
NA	NA	NA	NA	NA	NA	0	1	1.2	1	1	3	6	1	1	0	1	2
NA	NA	NA	NA	NA	NA	1	1	1.4	1	16	2	1	1	2	1	1	1
NA	NA	NA	NA	NA	NA	1	1	0.9	1	1	1	1	0	1	1	1	1
						0	0	0	0	0	0	0	0	0	0	0	0
NA	NA	NA	NA	NA	NA	1	3	0.5	1	1	1	1	1	3	1	0	2

Oleananes	Triterpeno	Terpenoid	NA	NA	NA	1	1	0.5	1	1	1	1	2	1	1	1	1
Biphenyls	Benzene a	Benzene a	NA	NA	NA	1	1	1.4	1	1	1	1	1	1	1	1	1
NA	NA	NA	NA	NA	NA	1	1	0.9	1	2	5	2	0	1	1	0	1
NA	NA	NA	NA	NA	NA	1	0	0.4	1	1	1	1	1	89	0	15	1
Anisoles	Phenol eth	Benzene a	NA	NA	NA	1	1	0.9	2	0	1	0	1	1	1	0	1
NA	NA	NA	NA	NA	NA	0	0	0.3	1	0	1	0	1	9	1	2	1
Steroid lac	Steroids ar	Steroids ar	NA	NA	NA	7	1	0.8	1	1	1	1	1	1	1	1	2
NA	NA	NA	NA	NA	NA	1	3	0.8	1	1	1	1	1	3	1	1	1
						0	0	0	0	0	0	0	0	16	0	0	0
						0	0	5	0	0	0	##	42	25	27	0	14
NA	Macrolide	Polyketide	NA	NA	NA	1	1	0.9	1	1	1	1	1	2	1	1	1
NA	NA	NA	NA	NA	NA	1	1	0.6	1	1	0	0	2	1	1	0	1
NA	NA	NA	NA	NA	NA	3	0	1.2	1	1	1	1	1	0	1	1	2
NA	NA	NA	NA	NA	NA	3	1	1.2	1	1	##	1	2	0	0	1	3
NA	NA	NA	NA	NA	NA	3	1	0.8	1	4	2	7	1	1	1	2	1
NA	NA	NA	NA	NA	NA	1	1	0.7	1	1	1	1	1	1	1	1	1
NA	NA	NA	NA	NA	NA	1	1	1.2	1	1	1	1	1	1	1	1	1
Benzoic ac	Benzene a	Benzene a	NA	NA	NA	1	1	1.3	0	1	1	2	2	1	2	1	1
NA	NA	NA	NA	NA	NA	2	0	0.3	6	2	2	5	0	0	1	1	12
						0	0	0	0	0	0	0	0	29	0	0	0
NA	Pyrimidine	Nucleic ac	Nucleotide	Pyrimidine	map00240	3	1	0	0	2	1	3	0	0	0	0	1
						11	0	3	0	19	0	12	25	0	0	0	0
NA	NA	NA	NA	NA	NA	1	1	0.4	1	0	0	1	3	0	0	0	2
NA	NA	NA	NA	NA	NA	1	1	0.5	1	1	1	1	2	1	1	0	1
Fatty acids	Fatty Acyls	Fatty acyls	NA	NA	NA	1	1	1.7	1	1	1	2	1	1	1	1	1
NA	NA	NA	NA	NA	NA	1	1	0.6	1	1	1	1	1	1	1	1	2
1,4-benzo	Benzodiaz	Organohe	NA	NA	NA	1	3	0.8	1	1	1	1	1	3	1	1	1
NA	NA	NA	NA	NA	NA	1	1	0.9	1	1	1	1	1	1	1	1	1
Eicosanoic	Fatty Acyls	Fatty acyls	Lipid meta	Arachidon	map00590	0	0	0.2	1	3	1	1	1	0	0	0	3
NA	NA	NA	NA	NA	NA	9	0	0.4	2	9	32	15	0	0	0	5	1
Carbohydr	Organoox	Carbohydr	NA	NA	NA	0	1	0.1	1	0	1	0	1	0	0	0	4
						0	0	0	0	0	0	0	0	0	31	0	0
						0	0	0	0	0	0	0	0	0	0	0	0
NA	NA	NA	NA	NA	NA	1	1	0.8	2	2	2	2	1	1	0	1	1
NA	NA	NA	NA	NA	NA	1	0	4.5	1	8	5	2	1	6	0	0	12
						0	0	0	0	0	0	0	0	0	0	0	0
						0	0	0	0	0	0	0	0	0	0	0	0
						0	0	0	0	0	0	0	0	0	0	0	0
						0	0	0	0	0	0	0	0	0	0	0	0
						0	0	0	0	0	0	0	0	0	0	0	0
						0	0	0	0	0	0	0	0	0	0	0	0
						0	0	0	0	0	0	0	0	0	0	0	0
						0	0	0	0	0	0	0	0	0	0	0	0
						0	0	0	0	0	0	0	0	0	0	0	0
						0	0	0	0	0	0	0	0	0	0	0	0
						0	0	0	0	0	0	0	0	0	0	0	0
						0	0	0	0	0	0	0	0	0	0	0	0
						0	0	0	0	0	0	0	0	0	0	0	0
						0	0	0	0	0	0	0	0	0	0	0	0
						0	0	0	0	0	0	0	0	0	0	0	0
						0	0	0	0	0	0	0	0	0	0	0	0
						0	0	0	0	0	0	0	0	0	0	0	0
						0	0	0	0	0	0	0	0	0	0	0	0
						0	0	0	0	0	0	0	0	0	0	0	0
						0	0	0	0	0	0	0	0	0	0	0	0
						0	0	0	0	0	0	0	0	0	0	0	0
						0	0	0	0	0	0	0	0	0	0	0	0
						0	0	0	0	0	0	0	0	0	0	0	0

						0	0	0	0	0	0	0	0	0	0	0	0
NA	NA	NA	NA	NA	NA	1	1	0.5	1	1	1	0	2	1	1	0	1
NA	NA	NA	NA	NA	NA	1	1	1	1	1	1	1	2	1	1	1	2
NA	NA	NA	NA	NA	NA	2	0	0.2	2	0	0	0	1	0	1	0	4
NA	NA	NA	NA	NA	NA	1	0	1.3	4	0	0	0	1	2	0	1	2
						0	0	0	0	0	0	0	0	0	0	0	0
Carbohydı	Organoox	Carbohydı	Carbohydı	Ascorbate	map00053	0	0	2	1	1	1	1	1	1	2	3	1
NA	NA	NA	NA	NA	NA	0	1	0.9	1	0	1	1	1	1	1	1	1
NA	NA	NA	NA	NA	NA	1	1	2.3	1	2	1	2	1	1	1	1	1
NA	NA	NA	NA	NA	NA	1	1	1.1	1	1	1	1	1	1	1	1	1
NA	NA	NA	NA	NA	NA	0	1	0.7	1	0	1	0	1	0	1	0	0
NA	NA	NA	NA	NA	NA	1	1	0.6	1	1	2	1	3	1	1	1	2
NA	NA	NA	NA	NA	NA	1	3	1.4	1	1	2	11	1	1	1	1	1
NA	NA	NA	NA	NA	NA	0	2	0.6	0	0	1	2	1	0	0	0	3
Indolyl car	Indoles an	Indole anc	NA	NA	NA	2	1	0.5	2	1	1	1	1	0	2	1	0
NA	NA	NA	NA	NA	NA	1	1	0.7	1	1	1	1	1	82	1	1	9
NA	NA	NA	NA	NA	NA	1	1	0.6	1	1	1	1	2	1	1	1	1
NA	NA	NA	NA	NA	NA	1	1	1.6	1	2	2	2	1	1	1	1	1
NA	NA	NA	NA	NA	NA	0	0	1	2	0	1	0	1	0	1	0	1
Benzoic ac	Benzene a	Benzene a	NA	NA	NA	1	1	1	1	1	22	1	1	1	1	1	1
NA	NA	NA	Metabolisı	Porphyrin	map0086C	1	1	0.6	2	0	3	0	3	0	1	1	0
NA	NA	NA	NA	NA	NA	1	1	1.1	1	1	1	1	0	1	1	1	1
Sulfated st	Steroids aı	Steroids aı	NA	NA	NA	0	1	0	0	0	1	1	1	3	3	0	6
NA	NA	NA	NA	NA	NA	1	0	0.5	1	9	2	7	1	0	1	0	4
						0	0	0	0	9	0	0	44	0	15	0	0
NA	NA	NA	NA	NA	NA	1	1	1.9	1	1	1	2	2	1	1	1	1
Glyceroph	Glyceroph	Glyceroph	NA	NA	NA	3	1	1	1	15	0	9	0	0	1	0	0
Steroid alk	Alkaloids (	Alkaloids	NA	NA	NA	1	1	1.3	1	1	1	1	1	1	1	1	22
NA	NA	NA	NA	NA	NA	1	3	0.8	1	1	1	1	1	3	1	1	1
						0	0	0	0	0	0	0	0	0	0	0	0
NA	NA	NA	NA	NA	NA	1	2	1	2	1	1	1	1	1	1	1	2
NA	NA	NA	NA	NA	NA	1	1	0.7	1	1	1	1	1	1	1	1	2
Beta hydrc	Hydroxy a	Organic ac	Carbohydı	Butanoate	map0065C	1	1	0.9	1	1	3	3	1	1	1	1	1
NA	NA	NA	NA	NA	NA	1	1	1.2	1	1	1	1	1	1	1	1	1
NA	NA	NA	NA	NA	NA	1	1	1.3	1	1	1	1	1	2	1	1	1
NA	NA	NA	NA	NA	NA	1	1	0.9	1	4	1	1	1	1	10	1	2
NA	NA	NA	NA	NA	NA	1	1	0.7	1	1	0	1	2	1	1	1	3
NA	NA	NA	NA	NA	NA	2	1	0.7	1	1	9	1	1	1	1	1	2
NA	NA	NA	NA	NA	NA	2	0	0.2	1	5	1	6	1	1	1	1	1
NA	NA	Alkaloids ε	Biosynthes	Tropane, ţ	NA	2	1	4	3	0	3	2	0	1	1	1	##
NA	NA	NA	NA	NA	NA	1	1	1.8	1	2	1	2	1	1	1	1	1
Amino aci	Carboxylic	Amino aci	NA	NA	NA	1	1	1.3	1	1	1	2	2	1	1	2	0
Retinoids	Prenol lipi	Prenol lipi	NA	NA	NA	0	1	1.9	1	0	0	1	1	0	1	1	0
						0	0	0	0	0	0	0	0	0	0	0	0
						0	0	0	0	0	0	0	0	0	0	0	0
NA	NA	NA	NA	NA	NA	1	1	1.3	1	1	1	2	1	1	1	1	1
						0	0	0	0	0	0	0	0	0	0	0	0
Pyrazoles	Azoles	Organohe	NA	NA	NA	1	1	0.6	1	1	1	1	1	3	1	1	1
NA	NA	NA	NA	NA	NA	1	1	1.7	2	2	1	2	1	1	1	0	0
						0	0	0	0	0	0	0	0	0	0	0	0
						0	0	0	0	0	0	0	0	0	0	0	0
NA	NA	NA	NA	NA	NA	1	1	0.9	1	1	1	1	1	2	1	1	1
Imidazoles	Azoles	Imidazoles	NA	NA	NA	2	0	0.2	1	10	2	7	1	0	0	0	4
NA	NA	NA	NA	NA	NA	0	1	0.7	1	0	0	1	1	1	1	1	1
NA	NA	NA	NA	NA	NA	1	1	0.9	1	1	1	1	1	1	1	1	1
						0	0	0	0	0	0	0	0	0	0	0	0
						0	0	0	0	0	0	0	0	0	0	0	0
						0	0	0	0	0	0	0	0	0	0	0	0

						0	0	0	0	0	0	0	0	0	0	0	0
						0	0	0	0	0	0	0	0	0	0	0	0
						0	0	0	0	0	0	0	0	0	0	0	0
						0	0	0	0	0	0	0	0	0	0	0	0
						0	0	0	0	0	0	0	0	0	0	0	0
NA	NA	NA	NA	NA	NA	6	1	0.8	1	17	13	22	1	0	1	9	1
NA	NA	NA	NA	NA	NA	0	3	0.8	1	0	0	1	1	2	1	1	1
NA	NA	NA	NA	NA	NA	0	3	0.7	1	1	1	1	1	3	1	1	2
NA	NA	NA	NA	NA	NA	2	1	1.9	1	2	1	2	1	2	2	2	0
NA	NA	NA	NA	NA	NA	1	1	0.9	1	2	1	2	1	1	1	1	1
NA	NA	NA	NA	NA	NA	1	1	0.7	1	1	1	1	1	1	1	1	1
Amino aci	Carboxylic	Amino aci	NA	NA	NA	1	1	0.9	1	1	1	1	1	1	1	1	1
Amino aci	Carboxylic	Amino aci	NA	NA	NA	2	0	0.8	2	1	6	2	1	2	0	0	12
NA	NA	NA	NA	NA	NA	1	1	0.4	2	1	0	0	1	0	1	0	2
						0	0	0	0	0	0	0	0	37	0	0	0
NA	NA	NA	NA	NA	NA	1	0	0.4	1	0	0	0	1	6	1	2	0
NA	NA	NA	NA	NA	NA	1	1	0.5	1	1	1	1	2	1	1	0	1
Amino aci	Carboxylic	Amino aci	NA	NA	NA	1	0	0.8	9	7	1	0	1	1	2	1	1
Fatty acids	Fatty Acyls	Fatty acyls	Lipid meta	Fatty acid	map00061	1	1	3.1	1	2	1	2	1	2	1	1	6
						0	0	0	0	0	0	0	7	0	0	0	0
						0	0	0	0	0	0	0	0	0	0	0	0
NA	NA	NA	NA	NA	NA	1	0	0.3	1	7	1	6	1	0	0	0	7
NA	NA	NA	NA	NA	NA	1	1	0.6	1	1	1	1	0	2	0	1	1
NA	NA	NA	NA	NA	NA	1	1	1.3	1	1	1	1	1	5	1	1	0
NA	NA	NA	NA	NA	NA	1	1	1.5	1	1	1	1	1	1	1	1	1
						0	11	0	0	12	0	37	0	0	44	0	##
NA	NA	NA	NA	NA	NA	1	1	0.8	1	1	1	2	2	2	1	1	1
Carbohydr	Organoox	Carbohydr	Carbohydr	Fructose a	map00051	0	2	0.4	1	1	3	4	1	1	2	1	4
Taxanes	Diterpenoi	Terpenoid	Metabolis	Diterpenoi	map0110	1	1	1.4	1	1	1	1	11	1	1	1	1
NA	NA	NA	NA	NA	NA	2	0	0.2	7	18	3	22	0	0	0	6	3
Amino aci	Carboxylic	Amino aci	NA	NA	NA	1	0	1	2	1	1	0	0	1	1	2	1
						0	0	0	0	0	0	0	0	0	0	0	0
Fatty acid	Fatty Acyls	Fatty acyls	NA	NA	NA	1	2	0.7	0	1	1	1	3	3	1	1	10
NA	NA	NA	NA	NA	NA	2	1	1.3	1	3	1	2	1	1	1	2	1
NA	NA	NA	NA	NA	NA	1	1	0.5	1	1	2	1	2	1	1	1	11
NA	NA	NA	NA	NA	NA	1	1	1.1	1	1	1	1	1	1	1	1	1
NA	NA	NA	NA	NA	NA	1	1	0.5	2	1	1	0	0	0	1	0	0
NA	NA	NA	NA	NA	NA	1	1	1.2	1	2	1	2	1	0	1	1	1
NA	NA	NA	NA	NA	NA	1	1	0.8	1	1	1	1	1	1	1	1	1
						0	0	0	0	0	0	0	0	0	0	0	0
Halobenze	Benzene a	Benzene a	NA	NA	NA	1	1	0.8	1	2	1	2	0	1	1	1	1
NA	NA	NA	NA	NA	NA	1	2	1	1	1	1	1	1	2	1	1	1
NA	NA	NA	NA	NA	NA	1	0	0.3	1	10	2	8	1	0	1	0	5
NA	NA	NA	NA	NA	NA	1	1	1	1	1	29	1	1	2	1	1	1
NA	NA	NA	NA	NA	NA	1	1	0.6	1	1	0	3	1	1	1	3	1
Sulfones	Sulfonyls	Organosul	Energy me	Sulfur met	map00920	2	1	1.3	1	1	2	2	0	1	1	3	0
						0	0	0	2	0	0	0	0	0	0	0	0
Fatty acid	Fatty Acyls	Fatty acyls	NA	NA	NA	0	1	0.5	0	1	1	1	2	2	1	3	1
Retinoids	Prenol lipi	Prenol lipi	NA	NA	NA	1	1	1.2	1	1	1	1	2	1	1	1	1
NA	NA	NA	NA	NA	NA	1	1	1.5	1	1	1	1	1	2	1	1	1
						0	0	0	0	0	0	0	0	0	0	0	0
						0	0	0	0	0	0	0	0	0	0	0	0
						0	0	0	0	0	0	0	0	0	0	0	0
						0	0	0	0	0	0	0	0	0	0	0	0
						0	0	0	0	0	0	0	0	0	0	0	0
						0	0	0	0	0	0	0	0	0	0	0	0
						0	0	0	0	0	0	0	0	0	0	0	0
						0	0	0	0	0	0	0	0	0	0	0	0

						0	0	0	0	0	0	0	0	0	0	0	0
						0	0	0	0	0	0	0	0	0	0	0	0
						0	0	0	0	0	0	0	0	0	0	0	0
						0	0	0	0	0	0	0	0	0	0	0	0
						0	0	0	0	0	0	0	0	0	0	0	0
						0	0	0	0	0	0	0	0	0	0	0	0
						0	0	0	0	0	0	0	0	0	0	0	0
						0	0	0	0	0	0	0	0	0	0	0	0
						0	0	0	0	0	0	0	0	0	0	0	0
						0	0	0	0	0	0	0	0	0	0	0	0
						0	0	0	0	0	0	0	0	0	0	0	0
NA	NA	NA	NA	NA	NA	1	1	0.9	1	1	1	1	1	1	1	1	2
NA	NA	NA	NA	NA	NA	1	1	1.6	1	1	2	2	1	1	1	1	1
NA	NA	NA	NA	NA	NA	1	1	1.3	1	2	1	2	1	1	1	1	1
Quinoline	Quinolines	Organohe	NA	NA	NA	0	1	1.4	2	1	3	1	1	1	1	1	0
NA	NA	NA	NA	NA	NA	3	1	0.6	0	4	1	5	0	0	1	0	1
NA	NA	NA	NA	NA	NA	1	1	1.1	1	1	1	1	2	1	1	1	1
						0	0	0	0	0	0	0	0	0	0	0	18
NA	NA	NA	NA	NA	NA	1	1	0.6	1	1	1	0	1	0	1	1	1
NA	Purine nuc	Purines an	NA	NA	NA	1	1	0.6	1	1	1	2	2	0	1	0	2
Amines	Organonit	Amines	Metabolis	Porphyrin	map00860	0	0	0.2	9	17	0	66	3	0	0	0	6
NA	NA	NA	NA	NA	NA	1	1	0.8	1	2	1	1	2	1	1	1	1
NA	NA	NA	NA	NA	NA	1	1	19	1	5	1	1	0	1	0	9	70
NA	NA	NA	NA	NA	NA	1	1	1.8	1	2	1	2	1	1	1	1	1
NA	NA	NA	NA	NA	NA	2	1	2.8	1	2	1	2	0	0	1	1	1
Fatty acids	Fatty Acyls	Fatty acyls	Amino aci	Lysine deg	map0031C	1	2	1	1	1	1	7	1	3	1	1	1
Others	Triterpeno	Terpenoid	NA	NA	NA	1	1	0.6	1	1	1	2	0	7	1	1	1
NA	NA	NA	NA	NA	NA	4	0	1	1	0	1	1	1	0	2	1	2
Aminoxide	Organonit	Organic ni	Energy me	Methane r	map0110(	0	1	0	6	1	4	3	2	1	1	3	1
NA	NA	NA	NA	NA	NA	1	1	0.6	1	6	2	12	1	0	1	4	1
NA	NA	NA	NA	NA	NA	1	1	0.9	1	2	1	2	1	1	1	1	1
NA	NA	NA	NA	NA	NA	1	0	0.4	1	0	1	1	3	1	1	0	9
NA	NA	NA	NA	NA	NA	1	1	0.7	1	1	1	1	1	1	1	1	2
NA	NA	NA	Global anc	Biosynthes	NA	1	1	0.9	1	1	1	1	1	1	1	1	1
NA	NA	NA	NA	NA	NA	2	1	0.2	1	0	1	0	1	1	0	0	2
Fatty acids	Fatty Acyls	Fatty acyls	NA	NA	NA	14	0	0.7	3	17	22	29	1	0	0	9	1
NA	NA	NA	NA	NA	NA	1	1	1.3	1	1	1	1	1	1	1	1	1
						0	0	0	0	0	0	0	0	0	0	0	0
NA	NA	NA	NA	NA	NA	1	1	0.6	1	1	1	6	1	0	1	0	1
NA	NA	NA	NA	NA	NA	1	1	1.2	2	1	1	3	1	1	1	1	1
						0	0	0	10	6	0	0	93	0	49	0	0
NA	NA	NA	Xenobiotic	Caprolacta	NA	1	1	1	1	5	1	13	1	0	2	2	1
NA	NA	NA	NA	NA	NA	0	1	6.4	0	1	1	12	1	26	0	2	0
NA	NA	NA	NA	NA	NA	1	0	0.6	1	2	0	1	1	2	2	0	8
Amino aci	Carboxylic	Amino aci	Amino aci	Glycine, se	map0026C	0	1	0.6	1	1	1	1	1	1	1	1	2
Amino aci	Carboxylic	Amino aci	Amino aci	Arginine a	map0033C	0	1	0.6	1	1	1	1	1	1	1	1	2
NA	NA	NA	NA	NA	NA	1	1	1.1	1	2	1	1	1	1	1	1	1
NA	NA	NA	NA	NA	NA	1	1	0.7	1	1	1	1	1	1	1	1	2
NA	NA	NA	NA	NA	NA	1	1	1.1	1	1	1	1	1	2	1	1	1
NA	NA	NA	NA	NA	NA	1	1	0.6	1	1	1	1	0	4	0	2	1
NA	NA	NA	NA	NA	NA	1	1	1.2	1	1	1	1	1	1	1	1	1
NA	NA	NA	NA	NA	NA	2	1	1.7	1	1	1	1	1	1	1	1	1
NA	NA	NA	NA	NA	NA	1	1	1	1	1	1	1	1	1	1	1	2
Biphenyls	Benzene a	Benzene a	NA	NA	NA	1	1	1.2	1	1	1	1	1	1	2	1	1
NA	NA	NA	NA	NA	NA	1	1	0.9	1	1	11	6	1	7	1	1	2
NA	NA	NA	NA	NA	NA	1	1	0.7	1	1	1	1	1	2	1	1	1
NA	NA	NA	NA	NA	NA	1	1	1.2	1	19	1	1	1	1	1	1	2
Amino aci	Carboxylic	Amino aci	Amino aci	Histidine r	map0034C	1	1	1	2	0	3	1	1	1	1	1	2

NA	NA	NA	NA	NA	NA	1	1	1.1	3	5	2	1	1	1	1	1	1
NA	NA	NA	NA	NA	NA	1	1	1.2	1	1	1	1	1	1	2	1	1
						0	0	0	0	0	0	0	0	0	0	0	0
						0	0	0	0	0	0	0	0	0	0	0	0
NA	NA	NA	NA	NA	NA	1	2	1	1	1	1	1	1	2	1	1	1
NA	NA	NA	NA	NA	NA	1	1	1	1	2	1	3	1	1	1	1	1
NA	Harmala a	Alkaloids a	Chemical s	Biosynthes	NA	1	1	2.1	0	3	1	1	1	0	0	0	1
NA	NA	NA	NA	NA	NA	0	1	1.2	3	1	0	0	1	0	1	1	0
						0	0	0	0	0	0	0	0	0	14	0	0
NA	NA	NA	NA	NA	NA	0	0	0.5	2	0	0	0	2	6	1	1	5
NA	NA	NA	NA	NA	NA	0	0	1.1	1	2	1	2	0	0	1	1	2
						0	0	0	0	0	0	0	0	0	0	0	0
NA	NA	NA	NA	NA	NA	0	0	1.6	0	0	0	0	1	9	3	0	1
NA	NA	NA	NA	NA	NA	1	1	0.6	1	1	1	1	0	4	1	2	1
NA	NA	NA	NA	NA	NA	2	0	0.7	3	0	0	0	1	2	1	1	3
NA	NA	NA	NA	NA	NA	8	0	0.5	2	##	0	8	5	0	3	0	93
NA	NA	NA	NA	NA	NA	1	1	1.8	1	2	1	2	1	1	1	1	1
NA	NA	NA	NA	NA	NA	1	3	0.8	0	1	1	1	18	0	4	1	55
NA	NA	NA	NA	NA	NA	1	1	0.7	1	1	1	1	1	1	1	1	1
Amino aci	Carboxylic	Amino aci	NA	NA	NA	0	2	1.6	1	1	2	0	0	1	1	6	0
NA	Phenylpro	Phenylpro	NA	NA	NA	0	3	0.8	1	1	1	1	1	3	1	1	1
NA	NA	NA	NA	NA	NA	1	1	2	1	3	1	4	1	1	1	2	1
NA	NA	NA	NA	NA	NA	1	1	1.3	1	1	4	1	4	1	1	1	1
Amino aci	Carboxylic	Amino aci	NA	NA	NA	1	3	0.9	1	1	1	1	1	3	1	1	2
						0	0	0	0	0	0	0	0	0	0	0	0
NA	NA	NA	NA	NA	NA	1	1	1.2	2	2	1	1	0	1	1	1	2
Bile acids,	Steroids a	Bile acids,	NA	NA	NA	1	0	0.3	4	0	0	0	4	4	2	2	11
NA	NA	NA	NA	NA	NA	1	3	0.6	1	1	1	1	1	3	1	0	2
NA	NA	NA	NA	NA	NA	0	3	0.9	1	1	1	1	1	3	1	1	2
NA	NA	NA	NA	NA	NA	1	1	2.5	0	0	0	1	2	1	2	1	0
NA	NA	NA	NA	NA	NA	1	1	0.9	1	1	1	1	1	7	1	1	1
NA	NA	NA	NA	NA	NA	1	1	1.2	1	1	1	1	4	1	2	4	1
NA	NA	NA	NA	NA	NA	1	2	1.2	1	1	1	1	2	2	1	1	1
NA	NA	NA	NA	NA	NA	4	0	1.1	1	5	##	6	0	0	1	3	1
NA	NA	NA	NA	NA	NA	0	0	0.2	1	0	0	0	1	5	1	6	0
NA	NA	NA	NA	NA	NA	1	1	0.7	2	1	3	2	1	1	1	1	2
NA	NA	NA	NA	NA	NA	1	1	1.3	1	1	1	1	1	8	1	1	1
NA	NA	NA	NA	NA	NA	0	1	1.2	2	3	0	3	1	2	1	0	1
NA	NA	NA	NA	NA	NA	1	1	1.1	1	1	1	1	3	1	1	2	3
NA	NA	NA	NA	NA	NA	0	0	1.8	1	1	0	0	8	1	3	1	1
NA	Phenanthr	Benzene a	NA	NA	NA	1	3	0.9	1	1	1	1	1	2	1	1	1
NA	NA	NA	NA	NA	NA	1	1	0.6	1	1	1	1	2	0	1	0	1
						0	0	0	0	0	0	0	9	0	0	0	0
						0	0	0	0	0	0	0	0	0	0	0	0
Amines	Organonit	Amines	NA	NA	NA	1	1	1	1	15	1	27	10	17	1	1	1
NA	NA	NA	NA	NA	NA	1	1	0.6	1	1	1	1	1	1	1	1	1
NA	NA	NA	NA	NA	NA	1	1	2.1	1	1	1	1	1	1	1	1	12
NA	NA	NA	NA	NA	NA	2	1	0.7	11	1	0	0	2	0	1	1	0
Furoic acic	Furans	Organohe	NA	NA	NA	1	2	1	2	2	5	6	1	0	1	1	0
						0	0	0	0	0	0	0	0	5	0	0	0
NA	NA	NA	NA	NA	NA	1	2	1.4	5	1	1	1	1	2	1	1	1
						0	0	0	0	0	0	0	0	0	0	0	0
NA	NA	NA	NA	NA	NA	0	1	0.3	0	1	3	3	2	5	1	0	4
NA	NA	NA	NA	NA	NA	0	3	0.8	1	1	1	1	1	3	1	1	2
NA	NA	NA	NA	NA	NA	6	1	0.5	1	7	4	4	1	0	1	4	1
						0	0	0	0	8	0	0	0	0	0	0	0
Isoflavono	Isoflavono	Flavonoids	Biosynthes	Isoflavono	NA	1	1	1	1	4	1	1	1	1	1	1	4
NA	NA	NA	NA	NA	NA	0	0	3	0	0	0	0	1	0	2	0	0

Pyrazolo[3	Pyrazolopy	Organohe	NA	NA	NA	6	1	0.5	0	4	0	3	0	1	1	0	1
Medium-c	Hydroxy a	Organic ac	NA	NA	NA	2	1	0.5	1	1	1	35	1	1	1	1	1
NA	NA	NA	NA	NA	NA	16	26	1	1	0	##	0	0	0	1	0	34
Purine ribo	Purine nuc	Purines an	NA	NA	NA	9	0	2.2	0	32	1	1	0	1	0	10	##
						0	0	0	0	0	0	0	0	0	0	0	0
NA	NA	NA	NA	NA	NA	0	0	0.5	1	0	0	0	1	10	1	1	1
NA	NA	NA	NA	NA	NA	1	0	121	0	1	0	6	0	4	11	28	0
NA	NA	NA	NA	NA	NA	1	1	0.6	1	1	0	0	2	1	1	0	1
Triterpeno	Prenol lipi	Terpenoid	NA	NA	NA	1	1	1.6	1	2	1	2	1	1	2	1	0
NA	NA	NA	NA	NA	NA	1	0	1	1	1	2	2	1	1	1	1	1
NA	NA	NA	NA	NA	NA	1	1	2	1	11	1	2	1	1	1	1	1
NA	NA	NA	NA	NA	NA	1	1	2.4	1	2	1	3	1	1	1	1	1
NA	NA	NA	NA	NA	NA	7	1	0.7	1	9	29	13	0	1	1	4	1
NA	NA	NA	NA	NA	NA	1	1	1.3	2	1	1	3	4	1	1	2	1
Amino aci	Carboxylic	Amino aci	NA	NA	NA	0	1	0.2	1	0	1	0	1	1	1	1	1
Fatty acids	Fatty Acyls	Fatty acyls	Lipid meta	Biosynthes	map01040	0	1	0.3	0	1	4	5	1	1	1	0	4
NA	NA	NA	NA	NA	NA	3	1	1	5	1	1	1	1	1	1	1	2
NA	NA	NA	NA	NA	NA	1	1	3	1	3	3	9	0	1	1	2	0
NA	Phenol est	Benzene a	NA	NA	NA	2	1	0.6	1	1	1	1	0	1	1	1	1
NA	NA	NA	NA	NA	NA	0	0	1.8	0	1	12	1	0	0	0	0	1
NA	NA	NA	NA	NA	NA	1	1	1.9	1	2	1	2	1	1	1	2	1
NA	NA	NA	NA	NA	NA	1	1	1	1	1	1	1	1	2	1	1	1
Bile acids,	Steroids ar	Bile acids,	NA	NA	NA	1	2	1.5	1	1	1	0	3	1	1	1	2
NA	NA	NA	NA	NA	NA	1	1	0.9	1	1	1	1	1	1	1	1	1
NA	NA	NA	NA	NA	NA	2	0	0.6	2	1	1	1	3	1	1	1	4
NA	NA	NA	NA	NA	NA	1	1	1	1	1	11	1	1	1	1	1	1
NA	NA	NA	NA	NA	NA	0	1	0.3	8	66	1	##	0	0	2	2	10
NA	NA	NA	NA	NA	NA	0	0	0.2	2	0	0	0	0	14	0	1	1
NA	NA	NA	NA	NA	NA	21	1	0.9	1	1	2	1	1	1	1	1	1
						0	0	0	0	0	0	0	0	0	0	0	0
NA	NA	NA	NA	NA	NA	1	1	1.4	1	1	1	1	1	1	1	1	1
NA	Tetracyclin	Polyketide	NA	NA	NA	1	0	0.5	1	0	1	0	2	0	1	1	0
NA	NA	NA	NA	NA	NA	0	1	0.5	1	1	1	1	0	1	1	1	0
NA	NA	NA	NA	NA	NA	1	1	0.8	1	0	0	0	1	0	2	2	0
NA	NA	NA	Amino aci	Tryptopha	map00380	##	1	0.7	1	2	1	2	0	1	1	1	2
NA	NA	NA	NA	NA	NA	1	1	1.1	2	1	1	0	7	1	1	1	1
NA	NA	NA	NA	NA	NA	1	1	0.6	2	1	1	1	1	1	1	2	1
Benzoic ac	Benzene a	Benzene a	NA	NA	NA	0	1	1.4	1	1	2	0	##	1	0	1	1
NA	NA	NA	NA	NA	NA	0	0	0.3	1	1	0	2	1	3	0	0	2
						0	0	0	0	7	0	0	0	0	0	0	0
Benzoic ac	Benzene a	Benzene a	NA	NA	NA	1	1	1	1	1	1	1	1	1	1	1	1
NA	NA	NA	NA	NA	NA	1	1	0.7	1	1	1	1	1	2	1	1	1
NA	NA	NA	NA	NA	NA	1	1	0.8	1	1	21	1	1	1	1	1	1
NA	NA	NA	NA	NA	NA	0	1	0.2	1	1	1	1	1	83	0	1	0
NA	NA	NA	NA	NA	NA	0	1	3.6	1	5	0	3	1	1	1	2	1
NA	NA	NA	NA	NA	NA	1	1	1.1	1	1	3	1	1	1	1	1	1
NA	NA	NA	NA	NA	NA	0	2	0.7	1	1	4	1	0	0	0	1	0
NA	NA	NA	NA	NA	NA	1	1	0.8	1	1	1	1	1	1	1	1	1
						0	0	0	0	0	0	55	0	0	0	0	0
NA	NA	NA	NA	NA	NA	1	1	1.3	2	1	1	1	1	1	1	1	3
NA	NA	NA	NA	NA	NA	2	0	0.3	2	0	0	0	1	2	1	0	2
NA	NA	NA	NA	NA	NA	1	3	1.2	1	10	1	1	0	1	1	1	1
Tricarboxy	Carboxylic	Organic ac	Carbohydr	Citrate cyc	map00020	1	2	1	2	2	5	6	1	0	1	1	0
NA	NA	NA	NA	NA	NA	1	1	1	1	1	10	1	1	1	1	1	1
NA	NA	NA	NA	NA	NA	0	0	3.6	0	0	0	0	2	0	4	0	0
NA	NA	NA	NA	NA	NA	1	2	1.3	2	0	0	1	0	2	1	1	1
NA	NA	NA	NA	NA	NA	1	2	0.6	1	1	1	1	0	3	1	1	1
Glyceroph	Glyceroph	Glyceroph	NA	NA	NA	2	2	0.7	0	6	0	3	1	0	0	0	0

NA	NA	NA	NA	NA	NA	0	1	3.1	5	0	0	0	5	0	5	1	0
NA	NA	NA	Biosynthes	Glucosinol	map0121(	0	2	0.4	1	1	1	1	2	0	0	0	4
NA	NA	NA	NA	NA	NA	1	1	0.7	2	1	31	1	2	1	1	2	1
Pyrazoles	Azoles	Organohe	NA	NA	NA	1	1	1.3	1	1	1	1	1	1	2	1	1
						0	0	0	0	5	0	0	0	0	0	0	0
						2	0	0	##	0	0	0	0	0	62	0	0
NA	NA	NA	NA	NA	NA	1	1	0.7	2	1	2	2	0	1	0	1	1
NA	NA	NA	NA	NA	NA	1	1	1	1	1	1	8	39	10	12	1	12
NA	NA	NA	NA	NA	NA	0	1	0.4	1	1	0	0	1	1	2	6	0
NA	NA	NA	NA	NA	NA	2	7	0.2	1	0	##	2	1	1	0	0	2
NA	NA	NA	NA	NA	NA	1	1	0.8	1	1	1	1	1	2	1	1	2
NA	NA	NA	NA	NA	NA	1	1	0.6	1	1	2	1	1	1	1	1	2
						0	0	0	0	0	0	0	0	0	0	0	0
NA	NA	NA	NA	NA	NA	29	1	0.6	1	1	1	1	0	1	1	1	47
NA	NA	NA	NA	NA	NA	1	1	0.6	1	2	1	2	0	1	1	1	1
NA	NA	NA	NA	NA	NA	1	1	0.5	1	0	0	1	4	4	1	1	8
NA	NA	NA	NA	NA	NA	1	1	0.7	1	2	2	2	0	1	0	1	1
Amino aci	Carboxylic	Amino aci	NA	NA	NA	2	1	0.6	1	1	1	1	1	1	1	1	1
						0	0	0	0	0	0	0	0	0	0	0	0
Flavonoid	Flavonoids	Flavonoids	NA	NA	NA	26	1	1.2	1	1	1	2	1	1	1	1	1
Fatty acid	Fatty Acyls	Fatty acyls	NA	NA	NA	0	1	0.2	0	1	0	0	0	0	1	2	1
NA	NA	NA	NA	NA	NA	1	1	0.5	1	23	1	16	1	1	1	1	2
NA	NA	NA	NA	NA	NA	1	0	2	1	1	1	1	1	1	1	2	1
Cardanolic	Steroids	Terpenoid	NA	NA	NA	1	1	0.8	1	1	1	1	1	12	1	1	1
1-hydroxy	Phenols	Phenols ar	Amino aci	Tyrosine m	map0035(	1	1	0.5	2	1	0	3	20	0	2	1	0
NA	NA	NA	NA	NA	NA	1	1	0.6	1	1	1	1	1	1	1	2	1
						0	0	0	0	2	0	0	0	16	0	0	40
NA	Purine nuc	Purines an	NA	NA	NA	1	1	0.7	2	0	1	0	1	0	0	0	0
NA	NA	NA	NA	NA	NA	1	1	1.6	1	1	1	1	1	1	1	1	1
NA	NA	NA	NA	NA	NA	5	1	0.4	1	2	4	3	1	1	0	1	2
Amines	Organonit	Amines	NA	NA	NA	0	1	1.1	1	2	1	1	1	1	1	1	0
NA	NA	NA	NA	NA	NA	0	1	0.5	5	1	0	1	0	0	0	0	0
NA	NA	NA	Lipid meta	Glyceroph	map00564	0	5	2.6	3	1	1	11	8	0	1	8	0
						0	0	0	0	0	0	0	0	0	0	0	0
NA	NA	NA	NA	NA	NA	2	1	0.7	1	3	1	1	1	33	0	0	2
NA	NA	NA	NA	NA	NA	1	0	1.3	2	1	1	2	0	0	1	2	0
NA	NA	NA	NA	NA	NA	1	1	0.9	1	1	1	3	1	1	1	9	1
NA	NA	NA	NA	NA	NA	1	1	0.6	1	15	1	1	1	0	1	1	3
NA	NA	NA	NA	NA	NA	1	0	0.5	1	0	1	1	0	0	1	1	15
NA	NA	NA	NA	NA	NA	1	1	0.9	1	1	1	1	1	3	1	1	1
Amino aci	Carboxylic	Amino aci	NA	NA	NA	0	1	0.7	1	1	1	2	1	2	1	1	2
NA	NA	NA	NA	NA	NA	1	1	0.5	1	0	1	2	2	0	0	0	4
						0	0	0	0	0	0	0	0	0	0	0	0
NA	NA	NA	NA	NA	NA	1	1	1.1	1	1	1	1	1	1	3	1	1
NA	NA	NA	NA	NA	NA	1	1	0.7	1	1	1	1	1	1	1	1	2
Cardanolic	23-Carbor	Sterol lipic	NA	NA	NA	1	0	0.8	5	14	0	3	1	2	2	1	26
NA	NA	NA	NA	NA	NA	0	5	1	0	1	5	1	4	1	1	1	2
						0	0	0	0	0	0	0	0	0	0	0	0
						0	0	0	0	0	0	0	0	0	0	0	0
NA	NA	NA	NA	NA	NA	1	0	0.4	1	1	0	1	1	##	0	1	0
Glyceroph	Glyceroph	Glyceroph	NA	NA	NA	1	1	1.5	1	1	2	2	1	1	1	1	1
NA	NA	NA	NA	NA	NA	1	1	0.7	1	1	1	1	1	1	2	1	1
NA	NA	NA	NA	NA	NA	2	1	0.2	0	0	0	0	1	0	2	0	10
NA	NA	NA	NA	NA	NA	1	0	6.4	2	58	0	16	0	0	0	1	1
						0	0	0	0	0	0	0	14	6	0	0	0
NA	NA	NA	NA	NA	NA	1	1	2.1	1	2	1	2	1	1	1	2	1
NA	NA	NA	NA	NA	NA	1	1	1.5	1	34	1	2	1	2	1	1	7
Amino aci	Carboxylic	Amino aci	Amino aci	Alanine, as	map0025(	1	3	7.3	1	2	4	3	1	1	1	4	1

NA	NA	NA	NA	NA	NA	1	1	1.9	1	2	1	3	1	1	1	1	2
NA	NA	NA	NA	NA	NA	1	2	0.7	1	1	2	1	1	1	1	2	2
Gamma-k	Keto acids	Organic a	NA	NA	NA	0	2	1.9	2	2	1	3	1	0	0	1	2
NA	NA	NA	NA	NA	NA	1	1	1.8	1	1	1	3	2	1	2	1	1
NA	NA	NA	NA	NA	NA	0	1	1	2	0	2	0	1	0	1	2	0
NA	NA	NA	NA	NA	NA	0	0	0.3	0	0	0	0	7	0	5	0	22
						0	0	0	0	0	0	0	0	0	0	0	0
NA	NA	NA	NA	NA	NA	6	1	0.8	1	1	1	1	0	##	1	1	2
NA	Indenes ar	Benzene a	NA	NA	NA	1	1	1.2	1	1	1	1	1	1	4	1	1
NA	NA	NA	NA	NA	NA	1	1	1.1	1	1	1	2	1	1	1	1	1
NA	NA	NA	NA	NA	NA	1	1	1.1	1	1	1	1	1	2	1	1	1
NA	NA	NA	NA	NA	NA	1	2	1	1	1	1	1	1	2	1	1	1
NA	NA	NA	NA	NA	NA	1	1	0.8	1	1	1	2	1	1	1	1	1
						0	0	0	0	0	0	0	0	0	0	0	0
NA	NA	NA	NA	NA	NA	1	1	0.6	1	1	1	1	1	2	1	1	1
NA	NA	NA	NA	NA	NA	1	1	1.1	1	1	1	1	1	1	1	1	1
NA	NA	NA	NA	NA	NA	1	1	1.2	1	1	1	1	1	1	1	2	1
NA	NA	NA	NA	NA	NA	1	1	0.8	1	0	1	0	1	1	1	2	1
NA	NA	NA	NA	NA	NA	0	1	0.3	0	0	0	2	1	3	0	1	4
NA	Purine nuc	Purines an	NA	NA	NA	0	2	0.7	1	1	1	1	2	0	0	0	3
NA	NA	NA	NA	NA	NA	1	1	0.5	2	1	3	5	1	2	1	1	10
NA	NA	NA	NA	NA	NA	1	1	1.9	1	2	1	2	1	1	1	1	1
NA	NA	NA	NA	NA	NA	1	1	1.4	1	1	1	1	1	1	1	1	1
NA	NA	NA	NA	NA	NA	1	1	0.9	2	1	1	1	1	1	1	0	1
NA	NA	NA	NA	NA	NA	1	1	0.8	1	1	1	1	1	2	1	1	1
NA	NA	NA	NA	NA	NA	1	7	1.6	1	1	##	2	1	1	1	1	6
NA	NA	NA	NA	NA	NA	1	1	1	1	1	1	1	1	1	2	1	1
NA	NA	NA	NA	NA	NA	1	5	1.1	1	13	1	1	0	1	1	1	1
						0	0	0	24	14	17	61	##	83	0	0	##
Quinazolir	Alkaloids (	Alkaloids	NA	NA	NA	1	0	0.5	1	1	0	1	1	7	1	2	0
NA	NA	NA	NA	NA	NA	1	1	0.6	1	1	0	0	2	1	1	0	1
Glyceroph	Glyceroph	Glyceroph	NA	NA	NA	6	0	1.6	0	14	10	14	0	5	0	4	1
NA	NA	NA	NA	NA	NA	0	3	0.8	1	1	1	1	1	3	1	1	1
NA	NA	NA	NA	NA	NA	1	1	0.6	1	1	1	1	0	2	1	1	1
NA	NA	NA	NA	NA	NA	2	1	2.3	1	4	43	7	1	1	0	3	1
Isoflavans	Isoflavono	Flavonoids	NA	NA	NA	1	1	0.7	1	1	1	1	0	3	0	1	1
NA	NA	NA	NA	NA	NA	1	1	0.8	1	1	0	1	1	5	4	3	1
NA	NA	NA	NA	NA	NA	1	1	0.7	2	2	1	4	1	1	1	2	1
NA	NA	NA	NA	NA	NA	1	1	0.9	1	2	1	2	1	1	1	1	1
NA	NA	NA	NA	NA	NA	1	1	1.7	1	2	1	2	1	1	1	1	1
NA	NA	NA	NA	NA	NA	0	2	2.2	1	3	2	1	0	1	1	1	0
NA	NA	NA	NA	NA	NA	0	1	0.8	2	0	1	0	1	0	2	0	0
NA	NA	NA	NA	NA	NA	0	1	0.5	1	0	1	0	4	20	2	0	0
NA	NA	NA	NA	NA	NA	2	1	0.3	1	6	1	7	1	0	0	1	0
Benzoic ac	Benzene a	Benzene a	NA	NA	NA	3	1	0.4	1	1	1	2	##	1	1	2	1
NA	NA	NA	NA	NA	NA	1	1	0.9	1	1	1	1	1	3	1	1	2
NA	NA	NA	NA	NA	NA	1	1	1.6	1	2	1	2	1	1	1	1	1
NA	NA	NA	NA	NA	NA	1	1	2	2	3	1	4	1	0	0	1	1
O-methyla	Flavonoids	Flavonoids	NA	NA	NA	1	1	0.6	1	1	1	1	1	2	1	1	2
NA	NA	NA	NA	NA	NA	1	1	0.6	3	1	1	1	1	1	1	1	0
NA	NA	NA	NA	NA	NA	1	0	0.4	1	0	1	0	1	##	1	1	9
NA	NA	NA	NA	NA	NA	0	6	1	3	43	1	55	0	3	1	1	5
NA	NA	NA	NA	NA	NA	0	1	0.4	1	11	1	11	1	1	1	0	1
Alpha hyd	Hydroxy a	Organic ac	Amino aci	Cysteine a	map00270	7	0	0.6	0	1	1	1	1	0	1	1	5
						0	0	0	0	0	0	0	0	0	0	0	0
Amino aci	Carboxylic	Amino aci	Amino aci	Arginine b	map00220	3	0	1.7	2	3	5	4	1	2	0	0	7
NA	NA	NA	NA	NA	NA	1	1	1.8	1	1	1	2	1	2	1	1	0
NA	NA	NA	NA	NA	NA	1	1	0.9	1	1	1	1	0	1	1	1	1

NA	NA	NA	NA	NA	NA	1	1	1.8	1	2	1	2	2	1	1	2	4
						0	0	0	0	0	0	0	0	0	0	0	5
Benzyl alc	Benzene a	Alcohols	Xenobiotic	Toluene d	map0110	0	4	0.3	0	3	0	0	2	5	0	0	0
NA	Phenanthr	Benzene a	NA	NA	NA	1	1	0.6	1	1	1	1	1	1	1	1	1
						0	0	0	0	0	0	0	0	0	0	0	0
Nitrophen	Phenols	Phenols ar	NA	NA	NA	1	0	1.6	1	1	1	1	1	1	1	1	0
NA	NA	NA	NA	NA	NA	1	2	6.5	4	1	1	1	20	32	13	1	1
						0	0	0	0	0	0	0	0	0	0	0	0
						0	0	0	0	0	0	0	0	0	0	0	0
						0	0	0	0	0	0	0	0	0	0	0	0
						0	0	0	0	0	0	0	0	0	0	0	0
						0	0	0	0	0	0	0	0	0	0	0	0
						0	0	0	0	0	0	0	0	0	0	0	0
						0	0	0	0	0	0	0	0	0	0	0	0
						0	0	0	0	0	0	0	0	0	0	0	0
						0	0	0	0	0	0	0	0	0	0	0	0
NA	NA	NA	NA	NA	NA	1	1	1.3	1	1	3	2	1	2	1	1	6
NA	NA	NA	NA	NA	NA	0	0	2.4	1	1	1	1	1	0	1	1	0
Phenethyl	Benzene a	Amines an	Amino aci	Tyrosine m	map00350	1	1	1.7	1	6	0	2	0	5	1	1	0
NA	NA	NA	NA	NA	NA	0	0	6.8	0	0	0	0	2	0	3	0	0
NA	NA	NA	NA	NA	NA	1	2	0.9	1	1	1	1	1	24	1	1	1
Stilbene g	Stilbenes	Polyketide	NA	NA	NA	1	1	0.8	1	24	2	7	1	23	1	1	1
NA	NA	NA	NA	NA	NA	1	1	1.5	1	1	1	3	2	2	2	2	1
NA	NA	NA	NA	NA	NA	0	1	0.2	2	0	0	1	1	0	4	5	3
NA	NA	NA	NA	NA	NA	1	1	1.6	1	2	1	2	2	1	1	1	1
Furoic acic	Furans	Organohe	NA	NA	NA	1	1	1	2	2	5	5	2	0	1	1	0
Fatty acids	Fatty Acyls	Fatty acyls	NA	NA	NA	1	1	1.6	1	2	1	7	1	6	1	1	1
						0	0	0	0	0	0	0	7	0	0	0	0
NA	NA	NA	NA	NA	NA	1	1	0.5	1	0	0	0	1	0	2	1	3
						0	0	0	7	0	2	0	0	0	0	0	0
NA	NA	NA	Biosynthes	Betalain bi	NA	2	1	0.7	1	1	1	1	1	1	1	1	1
NA	Morphinar	Alkaloids a	NA	NA	NA	1	1	1.8	1	2	1	1	10	1	1	1	1
NA	NA	NA	NA	NA	NA	5	0	0.4	3	25	20	17	1	0	1	12	7
NA	NA	NA	NA	NA	NA	1	1	1.1	1	1	1	1	1	1	1	1	1
NA	NA	NA	NA	NA	NA	1	1	1.1	1	1	1	1	1	1	1	2	1
NA	NA	NA	NA	NA	NA	1	1	2.2	1	2	1	2	1	1	1	2	0
NA	NA	NA	NA	NA	NA	1	1	1.7	1	1	1	1	1	1	1	1	1
NA	NA	NA	NA	NA	NA	1	1	1.7	1	1	1	1	1	1	1	1	8
NA	NA	NA	NA	NA	NA	2	1	0.7	1	2	12	3	1	1	1	2	2
Rotenoids	Isoflavono	Flavonoids	Biosynthes	Isoflavono	map0501	1	1	1	1	1	1	0	1	1	1	2	1
						0	0	0	0	0	0	0	0	0	0	0	0
						0	0	0	0	0	0	0	0	0	0	0	0
NA	NA	NA	NA	NA	NA	1	1	1.1	1	1	1	1	1	2	1	1	1
NA	NA	NA	NA	NA	NA	1	1	1.4	1	1	6	1	1	1	1	1	1
NA	NA	NA	NA	NA	NA	1	1	1.4	1	2	1	2	7	1	1	1	48
NA	NA	NA	NA	NA	NA	1	0	0.2	9	11	1	11	6	1	0	0	8
NA	Macrolides	Polyketide	NA	NA	NA	1	2	0.8	0	6	1	32	1	1	1	1	2
NA	NA	NA	NA	NA	NA	1	1	0.6	1	1	1	1	1	2	1	2	1
NA	NA	NA	NA	NA	NA	1	0	0.4	3	0	0	0	11	10	5	2	17
NA	Benzimida	Imidazole	NA	NA	NA	0	0	1.3	1	1	1	2	0	0	0	1	1
NA	NA	NA	NA	NA	NA	0	0	0.2	3	0	0	0	1	1	1	0	1
NA	NA	NA	NA	NA	NA	0	6	0.3	2	3	10	5	4	1	2	0	0
NA	NA	NA	NA	NA	NA	2	0	0.3	1	0	0	0	6	1	1	1	12
NA	NA	NA	NA	NA	NA	1	1	0.3	2	1	1	1	1	1	0	1	2
NA	NA	NA	NA	NA	NA	1	1	0.7	1	1	1	1	1	1	1	2	1
NA	Thienoimi	Organohe	NA	NA	NA	1	1	0.8	1	2	1	2	1	1	1	1	1
NA	NA	NA	NA	NA	NA	0	2	1	0	1	1	24	1	0	1	2	1
Amino aci	Carboxylic	Amino aci	Amino aci	Arginine b	map0022	1	2	1	1	4	4	10	1	1	1	2	1

NA	NA	NA	NA	NA	NA	1	1	1	1	1	1	1	1	1	1	1	2
NA	NA	NA	NA	NA	NA	1	1	0.6	1	1	1	1	1	1	1	1	1
NA	NA	NA	NA	NA	NA	3	1	0.5	1	1	6	0	3	1	1	1	2
NA	Benzene a	Benzene a	NA	NA	NA	1	1	1.3	1	1	1	1	1	11	1	1	2
NA	NA	NA	NA	NA	NA	1	1	1.1	1	3	1	1	1	1	2	2	1
Dibenzaze	Benzazepi	Organohe	NA	NA	NA	1	1	0.3	2	0	1	0	1	0	1	2	0
NA	NA	NA	NA	NA	NA	4	1	1.8	1	4	##	9	1	1	1	2	1
NA	NA	NA	NA	NA	NA	1	1	0.9	1	1	1	1	1	1	1	1	1
Bile acids,	Steroids a	Bile acids,	NA	NA	NA	1	0	0.2	1	0	0	0	8	4	1	1	18
Tyrosols a	Phenols	Phenols ar	NA	NA	NA	1	1	1.2	2	5	1	1	1	1	1	1	2
NA	NA	NA	NA	NA	NA	1	1	0.6	1	1	1	1	0	2	1	1	1
NA	NA	NA	Metabolisi	Glutathion	map0048C	1	2	0.7	1	1	1	1	1	2	1	0	1
NA	NA	NA	NA	NA	NA	0	1	0.3	2	1	0	32	1	0	0	1	1
NA	NA	NA	NA	NA	NA	0	0	0.2	2	0	0	0	0	4	1	0	5
NA	NA	NA	NA	NA	NA	12	1	1.3	1	1	1	1	1	1	1	1	5
NA	NA	NA	NA	NA	NA	3	0	1.2	1	7	7	6	0	0	2	5	2
						0	0	0	0	0	0	0	0	0	0	0	0
						0	0	0	0	0	0	0	0	0	0	0	0
NA	NA	NA	NA	NA	NA	1	1	0.7	1	1	1	1	1	1	1	1	1
Halobenze	Benzene a	Benzene a	NA	NA	NA	1	1	1.1	1	1	1	1	1	1	1	1	1
NA	NA	NA	NA	NA	NA	0	0	1.6	0	0	0	0	1	7	8	0	0
						0	0	0	0	0	0	0	0	28	0	0	0
NA	Coumarins	Coumarins	NA	NA	NA	2	1	1.2	1	2	1	1	0	1	2	2	2
NA	NA	NA	NA	NA	NA	1	1	1	1	0	0	1	1	1	1	1	1
FA0105 Hy	FA01 Fatty	Fatty acyls	Lipid meta	Cutin, sub	NA	1	0	0.6	1	36	1	15	1	0	1	2	1
NA	NA	NA	NA	NA	NA	1	1	0.9	1	1	1	1	1	1	1	1	1
NA	NA	NA	NA	NA	NA	1	1	1.4	1	1	1	1	1	1	1	1	1
NA	NA	NA	NA	NA	NA	1	1	2.3	1	1	1	2	1	0	0	1	1
Tryptamin	Indoles an	Amines an	NA	NA	NA	1	0	2.3	0	0	0	0	3	0	1	0	0
NA	NA	NA	NA	NA	NA	1	1	1.6	4	24	2	17	0	0	0	3	7
NA	NA	NA	NA	NA	NA	1	1	0.8	1	1	1	2	1	1	1	1	1
NA	NA	NA	NA	NA	NA	1	1	0.9	2	1	1	0	1	1	1	1	1
NA	NA	NA	NA	NA	NA	1	1	1	1	1	4	1	1	1	1	2	6
NA	Phenanthr	Benzene a	NA	NA	NA	1	1	0.9	1	1	1	1	1	1	1	1	1
						0	0	0	0	0	0	0	0	0	0	0	0
						0	0	0	0	0	0	0	0	0	0	0	0
						0	0	0	0	0	0	0	0	0	0	0	0
						0	0	0	0	0	0	0	0	0	0	0	0
						0	0	0	0	0	0	0	0	0	0	0	0
						0	0	0	0	0	0	0	0	0	0	0	0
						0	0	0	0	0	0	0	0	0	0	0	0
						0	0	0	0	0	0	0	0	0	0	0	0
						0	0	0	0	0	0	0	0	0	0	0	0
						0	0	0	0	0	0	0	0	0	0	0	0
						0	0	0	0	0	0	0	0	0	0	0	0
						0	0	0	0	0	0	0	0	0	0	0	0
						0	0	0	0	0	0	0	0	0	0	0	0
						0	0	0	0	0	0	0	0	0	0	0	0
						0	0	0	0	0	0	0	0	0	0	0	0
						0	0	0	0	0	0	0	0	0	0	0	0
						0	0	0	0	0	0	0	0	0	0	0	0
						0	0	0	0	0	0	0	0	0	0	0	0
NA	NA	NA	NA	NA	NA	1	1	1	2	2	1	1	1	1	1	1	0
NA	NA	NA	NA	NA	NA	1	1	1.5	1	1	1	1	1	1	1	1	1
NA	NA	NA	NA	NA	NA	1	1	1.5	1	1	1	2	1	1	1	1	0
NA	NA	NA	NA	NA	NA	1	1	0.8	2	1	1	0	2	1	2	1	1
NA	NA	NA	NA	NA	NA	1	0	0.4	1	5	0	0	0	5	1	0	1
NA	NA	NA	NA	NA	NA	0	0	0.3	2	0	0	0	0	71	0	1	2

NA	NA	NA	NA	NA	NA	1	1	1	1	1	1	1	1	1	1	1	2
NA	NA	NA	NA	NA	NA	1	1	1.1	1	1	1	0	1	1	1	2	1
						0	0	0	0	0	0	0	0	0	0	0	0
						0	0	0	0	0	0	0	0	0	0	0	0
NA	NA	NA	NA	NA	NA	1	1	1.1	1	1	1	1	2	1	1	1	1
Ketoses [F	Monosacc	Carbohydr	NA	NA	NA	2	0	0.8	2	9	0	11	1	0	2	1	1
NA	NA	NA	NA	NA	NA	1	1	0.8	1	1	12	1	1	1	1	1	1
NA	NA	NA	NA	NA	NA	1	1	1.5	1	1	1	1	1	2	1	1	2
NA	Pyrenes	Benzene a	Xenobiotic	Polycyclic	NA	1	1	2.5	1	2	1	1	1	1	1	1	0
Furanocou	Coumarins	Coumarins	NA	NA	NA	2	0	1.2	2	1	1	2	1	1	1	1	2
NA	NA	NA	NA	NA	NA	1	1	2.2	1	2	1	2	1	1	1	2	0
NA	NA	NA	NA	NA	NA	1	1	0.8	1	1	1	0	1	1	1	1	1
						0	0	0	0	0	0	0	0	0	0	0	0
GP2001 O	GP20 Oxic	Glyceroph	NA	NA	NA	1	2	0.8	1	1	1	1	1	3	1	0	1
NA	NA	NA	NA	NA	NA	1	0	0.1	9	9	0	10	4	0	0	0	10
NA	NA	NA	NA	NA	NA	1	1	0.9	2	1	1	4	1	1	1	1	1
1-benzopy	Benzopyra	Organohe	Biosynthes	Biosynthes	NA	1	1	1.4	1	1	3	2	1	1	1	1	1
NA	NA	NA	NA	NA	NA	1	1	0.7	1	2	1	1	1	2	1	1	1
NA	NA	NA	NA	NA	NA	2	1	1.3	1	6	1	6	1	1	1	1	0
NA	NA	NA	NA	NA	NA	1	1	0.7	1	1	2	0	1	1	1	1	1
Fatty acids	Fatty Acyls	Fatty acyls	Lipid meta	Fatty acid	map00061	1	1	0.3	1	9	1	9	0	1	1	0	2
NA	NA	NA	NA	NA	NA	1	1	0.7	1	1	1	1	1	1	1	1	1
NA	NA	NA	NA	NA	NA	1	0	1	1	1	0	1	1	1	1	2	1
NA	NA	NA	NA	NA	NA	1	2	0.9	1	1	1	1	1	1	1	1	1
Glyceroph	Glyceroph	Glyceroph	NA	NA	NA	1	1	1.9	1	2	1	2	3	1	1	1	1
Quinolizid	Alkaloids (	Alkaloids	Biosynthes	Tropane, p	NA	1	1	0.7	1	1	2	1	3	1	1	1	2
NA	NA	NA	NA	NA	NA	1	1	1.7	1	1	1	2	2	1	1	1	1
NA	NA	NA	NA	NA	NA	1	2	1.9	1	1	31	2	2	1	1	1	2
Amino aci	Carboxylic	Amino aci	Amino aci	Arginine a	map00330	1	1	0.5	2	3	4	1	1	1	1	4	1
NA	NA	NA	NA	NA	NA	2	1	0.2	0	0	0	3	1	0	0	0	3
NA	NA	NA	NA	NA	NA	1	0	1.5	0	1	1	0	1	10	1	0	1
NA	NA	NA	NA	NA	NA	1	1	1.3	1	1	1	1	4	2	1	2	1
Amino aci	Carboxylic	Amino aci	Amino aci	Lysine deg	map00310	2	1	1.7	1	1	2	3	1	1	1	1	3
Purines an	Imidazopy	Purines an	Biosynthes	Caffeine m	map00232	1	1	0.9	1	2	1	1	1	1	2	1	3
NA	NA	NA	NA	NA	NA	1	1	1	2	1	1	1	1	2	1	1	1
NA	NA	NA	NA	NA	NA	0	1	0.5	1	0	16	1	0	1	1	1	2
NA	NA	NA	NA	NA	NA	1	2	1.3	1	1	1	1	1	1	1	1	1
NA	NA	NA	NA	NA	NA	1	1	0.6	1	1	1	1	0	22	0	1	1
NA	NA	NA	NA	NA	NA	1	1	0.9	1	2	1	2	8	1	1	1	3
NA	NA	NA	NA	NA	NA	1	1	1.1	1	1	1	1	1	1	5	1	1
NA	NA	NA	NA	NA	NA	1	1	2.9	1	1	1	1	1	1	1	2	0
NA	NA	NA	NA	NA	NA	1	1	2.1	1	2	1	3	0	0	1	2	0
NA	NA	NA	NA	NA	NA	1	1	0.7	1	1	1	1	1	1	1	1	2
NA	NA	NA	NA	NA	NA	1	1	1	1	3	1	1	1	0	1	2	5
NA	NA	NA	NA	NA	NA	1	1	1	2	1	1	1	1	1	1	1	1
NA	NA	NA	NA	NA	NA	0	1	0.7	1	1	1	1	0	3	0	1	1
NA	NA	NA	NA	NA	NA	0	0	0.5	1	4	0	0	3	1	1	0	53
Bile acids,	Steroids a	Bile acids,	NA	NA	NA	0	1	0.5	1	1	0	0	6	2	13	1	7
NA	NA	NA	NA	NA	NA	1	1	2	1	2	1	2	1	1	1	1	1
NA	NA	NA	NA	NA	NA	1	1	1.3	2	1	1	1	1	1	1	2	1
NA	NA	NA	NA	NA	NA	1	1	1.8	1	2	1	2	7	1	1	1	1
NA	NA	NA	NA	NA	NA	1	1	0.9	1	1	1	1	1	1	1	1	1
						0	0	0	0	0	0	0	0	0	0	0	0
NA	NA	NA	NA	NA	NA	1	1	1	1	1	1	2	4	1	1	1	1
Carbohydr	Organoox	Carbohydr	NA	NA	NA	1	1	1.7	1	1	1	2	1	1	1	2	1
NA	NA	NA	NA	NA	NA	1	1	0.7	2	1	1	0	1	0	1	1	0
NA	NA	NA	NA	NA	NA	1	1	1.2	2	1	1	1	0	2	2	1	0
NA	NA	NA	NA	NA	NA	1	1	1.4	1	1	11	2	1	2	1	1	3

NA	NA	NA	NA	NA	NA	1	1	1	1	0	1	0	1	1	2	1	1
Carbohydr	Organoox	Carbohydr	NA	NA	NA	1	0	0.7	1	0	5	0	40	0	0	2	0
NA	NA	NA	NA	NA	NA	1	0	0.2	1	1	2	0	1	1	1	2	1
NA	NA	NA	NA	NA	NA	1	1	1.1	1	1	18	2	1	1	1	1	6
NA	NA	NA	NA	NA	NA	1	1	0.6	0	1	1	1	0	21	2	1	2
NA	NA	NA	NA	NA	NA	1	1	3.4	1	2	1	3	1	1	1	1	4
NA	NA	NA	NA	NA	NA	8	0	0.4	2	0	19	0	1	0	1	2	1
NA	NA	NA	NA	NA	NA	##	1	0.8	1	2	1	2	1	1	1	1	1
Bile acids,	Steroids ar	Bile acids,	NA	NA	NA	2	1	0.1	15	2	1	1	1	1	0	2	3
NA	Benzimida	Imidazole	Metabolisr	Riboflavin	map0074C	1	1	1.6	1	2	1	1	1	1	1	0	1
						0	0	0	0	0	0	0	0	0	0	0	0
						0	0	0	0	0	0	0	0	0	0	0	0
NA	NA	NA	NA	NA	NA	0	0	0.1	1	0	1	0	0	31	1	1	1
Organosul	Organic su	Organic ac	Lipid meta	Primary bi	map0012C	3	1	0.5	1	1	1	1	1	2	1	1	4
						0	0	0	0	0	0	0	##	34	0	0	0
NA	NA	NA	NA	NA	NA	1	1	0.5	1	1	1	1	3	0	0	0	2
Cinnamylp	Linear 1,3-	Phenols ar	NA	NA	NA	2	1	1.3	1	2	1	2	1	1	1	1	1
NA	NA	NA	NA	NA	NA	2	1	0.9	5	1	1	1	1	2	1	1	2
NA	NA	NA	NA	NA	NA	1	1	3.6	1	5	0	3	1	1	1	2	1
NA	NA	NA	NA	NA	NA	1	1	0.5	3	1	1	0	0	0	2	0	0
NA	NA	NA	NA	NA	NA	1	0	0.6	3	0	1	0	1	0	5	8	1
NA	NA	NA	NA	NA	NA	0	0	0.6	2	1	2	1	1	4	1	1	0
Fatty acids	Fatty Acyls	Fatty acyls	NA	NA	NA	1	1	1.2	1	2	0	2	1	1	1	2	0
Purines an	Imidazopy	Purines an	Nucleotide	Purine me	map0023C	6	0	0.2	0	3	0	3	1	0	0	0	1
Amines	Organonit	Amines	NA	NA	NA	0	2	1	2	3	0	0	0	0	1	0	0
Diradylgly	Glycerolipi	Glycerolipi	NA	NA	NA	1	1	0.8	1	1	1	1	1	1	1	1	2
NA	NA	NA	NA	NA	NA	2	0	0.5	1	2	3	3	0	0	0	0	3
NA	NA	NA	NA	NA	NA	2	1	0.4	2	0	1	0	0	0	1	0	1
NA	NA	NA	NA	NA	NA	1	0	0.9	3	0	0	0	0	0	5	1	0
Amino aci	Carboxylic	Amino aci	NA	NA	NA	1	0	0.2	1	0	1	0	0	1	1	0	2
NA	NA	NA	NA	NA	NA	2	1	0.1	3	11	0	40	1	0	1	0	16
NA	NA	NA	NA	NA	NA	2	1	0.6	1	13	1	1	2	1	0	1	5
Fatty acids	Fatty Acyls	Fatty acyls	NA	NA	NA	0	1	1	1	1	1	2	1	1	3	4	0
NA	NA	NA	NA	NA	NA	1	1	1.2	1	1	1	1	1	1	1	1	1
						0	0	0	2	2	0	0	11	0	0	0	0
NA	NA	NA	NA	NA	NA	2	2	0.6	1	1	1	1	1	1	2	1	1
NA	NA	NA	NA	NA	NA	7	1	0.4	1	0	64	0	1	1	3	0	5
Carbohydr	Organoox	Carbohydr	Carbohydr	Fructose a	map00051	1	2	1.1	1	4	5	12	0	2	1	2	2
Gamma bu	Lactones	Organohe	NA	NA	NA	1	1	1.2	1	1	1	1	1	1	1	2	1
NA	NA	NA	NA	NA	NA	1	1	0.6	1	1	1	1	1	1	1	1	8
NA	NA	NA	NA	NA	NA	1	1	1.8	1	2	3	2	1	1	1	2	1
NA	NA	NA	NA	NA	NA	1	1	2.1	23	2	1	18	4	1	5	1	1
NA	NA	NA	NA	NA	NA	1	1	1.4	1	1	2	2	4	1	1	1	##
NA	NA	NA	NA	NA	NA	1	1	0.8	1	1	1	1	1	1	1	1	1
Bile acids,	Steroids ar	Bile acids,	Lipid meta	Primary bi	map0012C	1	0	0.3	3	0	0	0	3	3	1	2	8
NA	NA	NA	NA	NA	NA	1	1	0.8	1	1	1	1	1	1	1	1	1
NA	NA	NA	NA	NA	NA	0	1	2.5	1	2	##	1	1	0	1	2	2
NA	NA	NA	NA	NA	NA	1	1	0.6	1	1	0	0	2	1	1	0	1
						0	0	0	0	0	0	0	0	0	0	0	0
						0	0	0	0	0	0	0	0	0	0	0	0
NA	NA	NA	NA	NA	NA	1	0	0.4	1	0	1	0	1	1	5	2	2
NA	NA	NA	NA	NA	NA	1	1	1	2	8	1	4	0	1	0	1	1
NA	NA	NA	NA	NA	NA	1	1	0.7	1	1	1	1	0	1	1	1	1
NA	NA	NA	NA	NA	NA	0	1	1.2	8	1	1	1	0	1	1	1	3
NA	NA	NA	NA	NA	NA	1	1	1.7	1	2	1	2	4	2	1	2	2
NA	NA	NA	NA	NA	NA	5	0	1.4	1	1	1	2	1	0	0	1	1
NA	NA	NA	NA	NA	NA	1	1	0.9	3	0	1	0	1	0	1	0	1
NA	NA	NA	NA	NA	NA	1	1	0.7	1	1	1	0	1	0	1	1	9

Pterins an	Pteridines	Pteridines	Metabolisı	Folate bio:	map0079C	1	1	0.6	2	1	1	1	0	0	0	0	7
NA	NA	NA	NA	NA	NA	1	1	1.8	1	1	1	1	0	1	2	0	0
NA	NA	NA	NA	NA	NA	0	6	0.2	0	1	4	1	1	2	0	0	3
						0	0	0	0	0	0	0	0	0	55	0	0
NA	NA	NA	NA	NA	NA	1	1	1.6	1	1	1	1	5	1	1	1	8
NA	NA	NA	NA	NA	NA	1	1	0.8	1	1	1	1	1	63	1	1	6
NA	NA	NA	NA	NA	NA	1	0	1.4	1	1	2	1	1	2	1	1	2
Fatty acid	Fatty Acyls	Fatty acyls	NA	NA	NA	2	0	1.9	3	1	0	1	1	0	2	0	5
NA	Morphinar	Alkaloids a	NA	NA	NA	1	1	0.6	1	1	1	1	1	3	1	1	1
NA	NA	NA	NA	NA	NA	7	0	0.3	0	3	1	7	1	0	1	0	0
NA	NA	NA	NA	NA	NA	0	1	1.1	1	0	0	0	7	1	1	0	1
NA	NA	NA	NA	NA	NA	1	1	1	1	1	1	1	1	1	1	1	1
NA	NA	NA	NA	NA	NA	1	1	1	1	1	1	2	1	1	1	1	84
NA	NA	NA	NA	NA	NA	3	1	0.6	1	11	1	9	1	1	1	1	2
NA	NA	NA	NA	NA	NA	0	0	1.2	1	1	1	3	0	2	0	2	1
NA	NA	NA	NA	NA	NA	4	2	2.1	##	4	3	2	2	2	0	0	##
NA	NA	NA	NA	NA	NA	0	1	0.6	1	0	0	1	2	1	1	2	1
NA	NA	NA	NA	NA	NA	1	1	0.7	1	1	1	1	1	1	1	1	1
Vitamin D	Steroids ar	Vitamins a	Lipid meta	Steroid bic	map0010C	1	1	1.5	1	1	2	2	1	2	1	1	1
NA	NA	NA	NA	NA	NA	1	1	1.2	1	1	1	1	1	2	1	1	1
Glyceroph	Glyceroph	Glyceroph	NA	NA	NA	1	1	1.6	1	2	1	2	15	3	1	1	5
Bile acids,	Steroids ar	Bile acids,	NA	NA	NA	3	2	1.8	##	3	3	1	2	2	0	0	98
Anthraqui	Anthraqui	Quinone	NA	NA	NA	1	0	2.7	1	2	2	1	1	1	1	2	1
NA	NA	NA	NA	NA	NA	1	0	0.1	2	0	0	0	3	2	1	1	4
NA	NA	NA	NA	NA	NA	3	1	0.7	2	4	3	3	1	1	0	1	1
NA	NA	NA	NA	NA	NA	9	1	2	1	1	1	2	2	1	1	1	1
						0	0	0	0	0	0	0	0	3	0	0	0
NA	NA	NA	NA	NA	NA	1	1	1.6	1	1	1	2	24	1	1	2	1
NA	Stilbenes	Polyketide	NA	NA	NA	2	1	0.8	1	1	1	1	1	0	1	1	1
NA	NA	NA	NA	NA	NA	1	0	2.4	0	0	0	0	1	2	1	0	0
Pyrimidine	Diazines	Organohe	NA	NA	NA	0	1	0.5	1	2	0	1	0	39	0	1	1
NA	NA	NA	Biosynthes	Acarbose a	map0110(	1	1	0.6	1	1	1	1	0	4	1	1	1
NA	NA	NA	NA	NA	NA	1	1	0.6	1	1	1	1	1	1	1	2	1
NA	NA	NA	NA	NA	NA	1	1	0.7	1	1	0	1	2	1	7	0	7
NA	NA	NA	NA	NA	NA	2	1	1.3	1	3	4	2	1	2	1	1	1
						0	0	0	20	0	0	0	9	0	0	0	0
NA	NA	NA	NA	NA	NA	1	1	0.7	1	1	1	1	1	1	1	1	1
NA	NA	NA	NA	NA	NA	1	0	0.2	4	0	0	0	1	0	3	0	0
Sulfated st	Steroids ar	Steroids ar	NA	NA	NA	1	4	1	1	1	3	7	1	1	1	1	##
NA	NA	NA	NA	NA	NA	1	1	1.1	1	1	1	1	1	2	1	1	1
NA	NA	NA	NA	NA	NA	1	1	0.6	2	1	1	1	1	1	1	1	2
NA	NA	NA	NA	NA	NA	1	1	0.8	1	1	1	0	1	1	1	1	1
NA	NA	NA	NA	NA	NA	1	1	1	1	1	1	1	0	1	1	1	1
NA	NA	NA	NA	NA	NA	1	1	0.7	1	1	1	1	1	1	1	1	1
Fatty acids	Fatty Acyls	Fatty acyls	NA	NA	NA	1	1	1.7	1	2	1	8	1	0	1	2	0
NA	NA	NA	NA	NA	NA	76	0	1	7	1	2	0	6	0	5	1	1
						0	0	0	0	0	0	4	0	18	0	0	12
NA	NA	NA	NA	NA	NA	1	1	0.7	1	2	1	2	0	2	0	1	1
NA	NA	NA	NA	NA	NA	1	1	0.8	1	1	1	0	1	1	1	1	0
NA	NA	NA	NA	NA	NA	2	1	0.4	2	0	0	0	16	1	1	15	3
NA	NA	NA	NA	NA	NA	1	1	0.6	1	1	0	1	1	1	1	1	2
NA	NA	NA	NA	NA	NA	1	1	1.4	1	1	1	1	49	0	1	1	0
						0	0	0	0	0	0	0	0	0	0	0	0
NA	NA	NA	NA	NA	NA	1	0	0.4	4	0	0	0	1	0	1	0	1
NA	NA	NA	NA	NA	NA	1	3	0.7	1	5	4	9	1	2	1	4	1
NA	NA	NA	NA	NA	NA	1	1	0.4	4	1	1	1	2	1	8	7	8
NA	NA	NA	NA	NA	NA	1	4	0.8	1	1	1	1	1	2	1	1	1
NA	NA	NA	NA	NA	NA	1	1	0.9	2	1	1	1	1	1	1	2	1

Flavones	Flavonoids	Flavonoids	NA	NA	NA	1	1	1	1	1	1	1	1	1	1	2	20
Beta lactar	Lactams	Organohe	NA	NA	NA	18	0	0.7	1	0	1	0	12	0	1	2	2
NA	NA	NA	NA	NA	NA	1	1	0.7	1	0	1	0	2	2	1	3	0
NA	NA	NA	NA	NA	NA	1	1	1.2	1	1	1	2	1	1	1	1	1
NA	NA	NA	NA	NA	NA	1	1	0.9	1	1	1	0	1	5	1	1	1
NA	NA	NA	NA	NA	NA	1	1	0.7	2	2	1	2	0	1	0	1	2
Beta lactar	Lactams	Organohe	NA	NA	NA	8	0	13	0	1	1	1	19	2	3	2	5
NA	NA	NA	NA	NA	NA	1	2	0.6	3	1	0	0	0	0	1	0	0
NA	NA	NA	NA	NA	NA	1	1	0.8	1	1	1	10	1	1	1	1	2
Terpene g	Prenol lipi	Terpenoid	Metabolisi	Monoterp	NA	1	1	1.2	1	2	1	1	1	1	1	1	1
NA	NA	NA	NA	NA	NA	0	0	0.2	6	23	1	2	0	0	1	1	0
Hydroxycii	Cinnamic a	Polyketide	NA	NA	NA	0	1	2.5	3	4	0	0	1	2	1	0	0
Carboxylic	Carboxylic	Organic ac	Amino aci	Histidine r	map0034C	2	1	1.6	1	8	1	1	4	1	1	1	1
						0	0	0	0	0	0	0	0	0	8	0	0
NA	NA	NA	NA	NA	NA	1	0	26	1	67	1	17	1	2	1	49	1
NA	NA	NA	NA	NA	NA	1	1	1.4	1	2	1	2	1	1	1	1	0
NA	NA	NA	NA	NA	NA	1	1	1.7	1	1	1	2	1	1	1	2	1
Amino aci	Carboxylic	Amino aci	NA	NA	NA	0	1	1.9	1	3	2	7	1	4	1	4	3
NA	NA	NA	NA	NA	NA	1	1	1.3	1	1	1	1	1	1	1	2	1
NA	NA	NA	NA	NA	NA	1	0	1.3	1	0	1	2	0	1	1	1	2
						0	0	0	0	0	0	0	0	0	0	0	0
						0	0	0	0	0	0	0	0	0	0	0	0
						0	0	0	0	0	0	0	0	0	0	0	0
						0	0	0	0	0	0	0	0	0	0	0	0
						0	0	0	0	0	0	0	0	0	0	0	0
						0	0	0	0	0	0	0	0	0	0	0	0
						0	0	0	0	0	0	0	0	0	0	0	0
						0	0	0	0	0	0	0	0	0	0	0	0
						0	0	0	0	0	0	0	0	0	0	0	0
						0	0	0	0	0	0	0	0	0	0	0	0
						0	0	0	0	0	0	0	0	0	0	0	0
						0	0	0	0	0	0	0	0	0	0	0	0
						0	0	0	0	0	0	0	0	0	0	0	0
						0	0	0	0	0	0	0	0	0	0	0	0
						0	0	0	0	0	0	0	0	0	0	0	0
Isoquinolir	Alkaloids (	Alkaloids	NA	NA	NA	3	1	0.7	1	1	0	1	1	6	1	0	2
NA	NA	NA	NA	NA	NA	1	1	0.7	1	0	1	0	1	1	1	2	2
NA	NA	NA	NA	NA	NA	1	1	0.8	2	1	1	2	1	1	1	1	6
Fatty acids	Fatty Acyls	Fatty acyls	NA	NA	NA	1	1	1.8	1	1	1	1	1	0	6	2	0
						0	0	0	0	0	0	0	0	0	0	0	0
NA	NA	NA	NA	NA	NA	0	1	0.8	1	0	7	0	4	0	0	4	1
Tricarboxy	Carboxylic	Organic ac	Carbohydi	C5-Branch	map0110(	1	2	1.5	1	2	3	6	1	1	1	1	1
NA	NA	NA	NA	NA	NA	0	3	1.6	1	3	1	0	0	0	1	0	0
Pyrrolidiny	Pyridines a	Pyridine ai	Biosynthes	Tropane, p	map0098(	6	1	0.7	2	1	1	0	5	0	1	2	1
NA	NA	NA	NA	NA	NA	1	1	1.8	1	1	1	2	1	1	1	1	1
NA	NA	NA	NA	NA	NA	1	1	1.7	1	2	1	2	1	2	1	1	1
NA	NA	NA	NA	NA	NA	0	1	6.5	0	1	1	12	1	26	0	2	0
NA	NA	NA	NA	NA	NA	1	2	2.3	1	1	1	4	1	1	5	2	1
Carbohydi	Organoox	Carbohydi	Carbohydi	Ascorbate	map00053	1	0	0.6	1	0	0	0	1	2	1	2	2
NA	5'-deoxyri	Nucleic ac	NA	NA	NA	1	1	0.6	2	0	2	0	1	0	1	1	0
NA	NA	NA	NA	NA	NA	0	1	0.8	3	0	2	0	1	0	1	1	1
Purines an	Imidazopy	Purines an	Nucleotid	Purine me	map0023C	1	1	1.8	1	1	1	1	1	1	1	1	1
NA	NA	NA	NA	NA	NA	1	1	1.1	2	1	1	2	1	1	1	1	1
NA	NA	NA	NA	NA	NA	1	1	0.9	1	1	1	1	1	1	1	1	3
NA	NA	NA	NA	NA	NA	0	1	0.5	1	0	0	1	0	1	1	0	1
						0	0	0	0	0	0	8	0	0	0	0	0
Dibenzaze	Benzazepi	Organohe	NA	NA	NA	1	1	0.5	1	0	1	0	1	1	1	0	3
NA	NA	NA	NA	NA	NA	1	1	0.3	1	0	3	0	1	1	1	5	0

Cyclamate	Sulfamic a	Organic ac	Xenobiotic	Caprolacta	NA	1	0	2.1	1	10	0	10	0	0	13	0	9
Imidazolid	Azolidines	Organohe	NA	NA	NA	2	1	0.2	1	0	2	0	1	0	2	1	0
Germacrer	Sesquiterp	Terpenoid	NA	NA	NA	2	0	0.5	7	0	2	0	1	0	5	3	0
NA	NA	NA	NA	NA	NA	1	1	1.2	0	3	5	5	0	2	1	2	0
NA	NA	NA	NA	NA	NA	0	8	0.2	0	1	0	1	1	0	1	0	12
Phosphos	Sphingolip	Sphingolip	NA	NA	NA	1	1	1.8	1	30	1	29	0	1	1	2	1
NA	Benzene a	Benzene a	Xenobiotic	Aminoben	NA	1	1	1.3	2	1	1	1	1	1	1	2	0
NA	NA	NA	NA	NA	NA	1	1	1.1	1	1	1	1	1	1	4	1	1
NA	NA	NA	NA	NA	NA	1	2	0.8	1	1	1	1	1	1	1	1	2
NA	NA	NA	NA	NA	NA	1	1	1.7	1	2	1	2	1	1	1	1	1
Steroid alk	Alkaloids (	Alkaloids	NA	NA	NA	1	1	1.3	1	11	1	3	2	63	1	1	1
Carbonyl (	Organoox	Organic o:	NA	NA	NA	1	1	0.3	3	0	1	0	1	0	1	2	0
NA	Pteridines	Pteridines	Metabolis	Riboflavin	map00740	1	0	0.4	1	1	0	0	1	44	2	1	8
NA	NA	NA	NA	NA	NA	4	1	1	1	5	##	7	1	0	1	3	1
Others	Diterpenoi	Terpenoid	NA	NA	NA	2	1	0.4	11	1	1	1	1	1	0	3	1
NA	NA	NA	NA	NA	NA	5	1	1.5	1	8	29	13	1	1	1	4	2
NA	NA	NA	NA	NA	NA	1	1	1.9	1	15	1	1	1	1	1	1	2
Hydroxyin	Indoles an	Indole anc	Amino aci	Tryptopha	map00380	4	0	1	1	0	1	10	34	2	3	1	9
NA	NA	NA	NA	NA	NA	1	1	0.5	2	1	0	0	0	0	1	0	0
Linear diar	Diarylhept	Polyketide	Biosynthes	Stilbenoid,	NA	1	0	1.3	4	0	0	0	1	1	1	1	2
NA	NA	NA	NA	NA	NA	1	29	1.5	1	1	1	1	1	1	1	1	1
NA	NA	NA	NA	NA	NA	2	1	0.7	1	1	1	1	1	1	1	1	1
NA	NA	NA	NA	NA	NA	1	1	0.8	1	1	49	1	1	1	1	1	1
NA	NA	NA	NA	NA	NA	1	1	2	1	2	1	2	1	1	1	2	1
NA	NA	NA	NA	NA	NA	9	1	0.8	1	1	1	1	1	1	1	1	1
NA	NA	NA	NA	NA	NA	2	1	0.2	1	0	2	0	5	0	2	1	7
NA	NA	NA	NA	NA	NA	1	1	0.9	1	8	3	5	3	1	1	1	5
NA	NA	NA	NA	NA	NA	0	1	0.3	1	1	2	1	1	2	0	0	23
NA	NA	NA	NA	NA	NA	1	1	0.7	1	0	1	1	1	1	1	1	1
NA	NA	NA	NA	NA	NA	1	1	1.5	1	1	1	2	1	1	1	1	1
NA	NA	NA	NA	NA	NA	1	1	0.9	2	1	1	1	1	2	1	1	1
NA	NA	NA	NA	NA	NA	0	1	2.4	1	1	1	1	1	1	1	2	1
NA	NA	NA	NA	NA	NA	1	1	2.2	1	2	1	2	1	1	1	1	0
NA	NA	NA	NA	NA	NA	2	1	0.4	1	0	1	0	3	0	3	1	2
NA	NA	NA	NA	NA	NA	2	1	0.9	1	4	5	1	2	1	0	1	1
NA	NA	NA	NA	NA	NA	1	1	0.3	2	1	1	0	1	1	1	0	0
Furanocou	Coumarins	Coumarins	NA	NA	NA	1	1	0.9	1	1	11	1	0	1	1	1	1
NA	NA	NA	NA	NA	NA	1	1	0.9	1	3	3	2	0	2	0	1	2
						0	0	0	0	##	0	0	0	0	0	0	0
						0	0	0	0	0	0	##	86	6	0	0	0
NA	NA	NA	NA	NA	NA	1	1	1.9	1	2	1	2	1	1	1	1	1
NA	Benzodiox	Organohe	NA	NA	NA	1	1	1.2	1	2	2	2	1	1	1	1	1
NA	NA	NA	NA	NA	NA	0	2	0.8	1	0	0	0	4	0	4	1	2
NA	NA	NA	NA	NA	NA	1	3	2.1	1	1	4	1	1	1	1	1	1
NA	NA	NA	NA	NA	NA	3	1	0.4	1	12	6	9	1	0	1	3	1
NA	NA	NA	NA	NA	NA	1	1	0.5	0	1	6	0	2	1	1	1	0
Amino aci	Carboxylic	Amino aci	NA	NA	NA	23	1	0.8	1	1	1	0	1	1	0	0	2
NA	NA	NA	NA	NA	NA	1	0	0.9	1	0	1	0	1	4	3	1	0
Amino aci	Carboxylic	Amino aci	Xenobiotic	Drug meta	map00982	0	0	33	4	##	0	##	1	1	70	2	##
NA	NA	NA	NA	NA	NA	1	5	1.8	1	0	1	1	3	0	1	1	1
NA	NA	NA	NA	NA	NA	1	1	0.8	1	1	1	0	1	1	1	1	1
						0	0	0	0	0	0	0	47	60	##	0	0
NA	NA	NA	NA	NA	NA	1	1	0.9	1	1	1	1	2	1	1	1	1
NA	NA	NA	NA	NA	NA	1	1	1.5	1	2	1	2	1	1	1	2	9
NA	NA	NA	NA	NA	NA	0	1	0.2	3	0	0	1	1	1	5	5	3
NA	NA	NA	NA	NA	NA	18	51	0.3	1	0	##	0	0	0	0	1	35
NA	NA	NA	NA	NA	NA	2	1	1.6	2	3	0	2	2	1	1	3	1
NA	NA	NA	NA	NA	NA	1	1	3.1	1	1	4	1	0	1	1	1	1

NA	NA	NA	NA	NA	NA	1	1	0.9	1	1	1	0	1	1	1	1	1
NA	NA	NA	NA	NA	NA	1	2	0.7	2	6	1	7	2	3	0	2	##
						0	0	0	0	3	0	0	0	28	0	0	0
Benzoic ac	Benzene a	Benzene a	NA	NA	NA	1	1	1.1	1	5	1	4	1	1	1	1	0
NA	NA	NA	NA	NA	NA	1	1	0.6	1	1	##	1	1	1	1	1	1
Fatty acids	Fatty Acyls	Fatty acyls	NA	NA	NA	1	1	0.7	1	1	5	4	1	0	1	1	1
						0	0	0	0	0	0	0	9	0	0	0	0
NA	NA	NA	NA	NA	NA	1	1	1.6	1	2	1	2	1	1	1	2	46
Null	PK04 Mac	Polyketide	Metabolis	Biosynthes	map0110	0	0	1.3	2	2	0	2	2	1	5	4	24
NA	NA	NA	NA	NA	NA	1	1	0.3	0	1	2	2	2	0	0	1	1
Androstan	Steroids a	Steroids a	NA	NA	NA	1	1	1	1	1	1	1	1	1	1	1	1
						0	0	0	0	3	0	5	0	0	13	0	0
Null	PK04 Mac	Polyketide	Metabolis	Biosynthes	map0110	1	1	1.6	1	2	1	2	1	1	1	2	##
NA	NA	NA	NA	NA	NA	1	1	1.4	1	1	1	1	2	1	1	1	1
NA	NA	NA	NA	NA	NA	1	1	1.6	1	2	1	2	1	1	1	2	##
Ursanes	Triterpeno	Terpenoid	NA	NA	NA	3	1	0.5	0	0	1	0	2	0	6	1	7
NA	NA	NA	NA	NA	NA	1	1	1	1	4	33	7	2	1	1	5	2
NA	NA	NA	NA	NA	NA	2	0	0.9	2	1	1	0	1	0	1	1	0
NA	NA	NA	NA	NA	NA	1	1	1.7	1	2	1	2	1	1	1	2	10
						0	0	0	0	0	0	14	7	19	8	0	0
NA	NA	NA	NA	NA	NA	1	1	1.7	1	2	1	2	1	1	1	2	0
NA	NA	NA	NA	NA	NA	1	1	1	1	2	1	1	1	0	1	1	0
NA	NA	NA	NA	NA	NA	4	1	0.6	1	5	82	11	1	1	1	4	1
						0	0	0	43	0	0	50	##	17	38	0	36
NA	NA	NA	NA	NA	NA	1	1	1.5	1	1	1	1	1	1	1	1	0
Phenothia	Benzothia	Organohe	NA	NA	NA	1	1	0.6	1	1	1	1	1	1	1	1	1
NA	NA	NA	NA	NA	NA	1	1	0.7	1	1	1	1	1	0	1	2	1
NA	NA	NA	NA	NA	NA	1	1	1	1	1	1	1	1	44	1	1	1
NA	NA	NA	NA	NA	NA	1	1	0.9	1	1	1	1	1	1	1	1	1
NA	NA	NA	NA	NA	NA	1	1	1.3	2	1	1	1	1	1	1	1	0
NA	NA	NA	NA	NA	NA	14	0	2.7	0	2	64	2	0	0	0	1	0
NA	NA	NA	NA	NA	NA	0	1	1.2	0	2	0	1	3	1	1	0	1
NA	NA	NA	NA	NA	NA	7	1	1	1	1	1	1	23	1	1	1	1
NA	NA	NA	NA	NA	NA	1	0	0.4	2	0	0	0	0	40	2	0	3
NA	NA	NA	NA	NA	NA	1	1	1.3	1	1	1	2	4	2	1	1	3
Amino aci	Carboxylic	Amino aci	Amino aci	Glycine, se	map00260	3	1	2	2	3	1	1	1	1	1	1	1
Lineolic ac	Fatty Acyls	Fatty acyls	NA	NA	NA	1	2	1	1	1	1	2	1	2	1	1	1
Trifluorom	Benzene a	Benzene a	NA	NA	NA	1	1	0.6	1	1	1	1	1	1	1	1	1
NA	NA	NA	NA	NA	NA	2	1	1	1	1	7	1	1	1	2	1	1
NA	NA	NA	NA	NA	NA	1	3	6.1	1	0	1	0	1	0	0	7	0
NA	NA	NA	NA	NA	NA	0	6	0.3	0	0	0	0	1	43	19	0	##
NA	NA	NA	NA	NA	NA	1	1	1	3	0	1	0	1	0	6	4	0
NA	NA	NA	NA	NA	NA	1	1	1.8	1	2	1	2	1	1	1	1	1
Terpene g	Prenol lipi	Terpenoid	NA	NA	NA	1	1	1.6	1	2	1	2	1	1	1	2	16
NA	NA	NA	NA	NA	NA	1	0	0.2	1	0	0	0	0	##	0	1	10
NA	NA	NA	NA	NA	NA	2	1	0.7	1	1	##	1	0	0	1	2	1
NA	NA	NA	Xenobiotic	Aminoben	NA	1	1	1	1	1	1	1	1	1	1	1	1
NA	NA	NA	NA	NA	NA	1	1	0.8	1	1	1	1	0	1	1	1	1
NA	NA	NA	NA	NA	NA	1	1	1.2	1	7	1	54	1	1	1	1	1
NA	NA	NA	NA	NA	NA	0	1	1.2	1	2	1	2	0	0	1	1	2
NA	NA	NA	NA	NA	NA	0	5	0.9	2	1	2	0	1	0	0	0	0
NA	NA	NA	NA	NA	NA	2	1	1.8	1	2	1	2	0	0	1	1	1
NA	NA	NA	NA	NA	NA	1	1	1.2	1	1	1	1	1	1	2	1	1
NA	NA	NA	NA	NA	NA	1	1	1.2	1	2	1	1	1	1	1	1	1
						0	0	0	0	5	0	0	0	0	8	0	0
NA	NA	NA	NA	NA	NA	1	2	0.5	1	1	2	0	2	0	1	1	3
						0	0	0	6	71	0	0	68	26	0	0	0
NA	NA	NA	NA	NA	NA	0	0	0.1	0	0	1	1	2	5	1	0	6

Sulfated st	Steroids ar	Steroids ar	NA	NA	NA	0	0	0.1	0	0	1	1	2	5	1	0	6
NA	NA	NA	Metabolisı	Biosynthes	map0110(	1	0	1.1	1	0	0	1	1	1	1	1	4
NA	NA	NA	NA	NA	NA	1	1	0.5	3	2	1	1	1	1	0	3	1
NA	NA	NA	NA	NA	NA	2	1	1	1	0	1	1	1	1	1	2	2
NA	NA	NA	NA	NA	NA	3	1	1.2	1	3	3	4	1	1	1	1	2
NA	NA	NA	NA	NA	NA	1	2	1.5	1	2	1	3	2	0	2	1	2
NA	NA	NA	NA	NA	NA	1	1	1.7	1	2	2	2	1	1	1	1	1
NA	NA	NA	Lipid meta	Biosynthes	map0104(	5	0	0.2	3	5	24	6	1	1	0	7	1
NA	NA	NA	NA	NA	NA	3	1	1.4	1	1	1	1	1	1	0	1	3
						0	0	0	0	0	0	0	0	0	12	0	0
						0	0	0	0	0	0	0	0	0	0	0	0
						0	0	0	0	0	0	0	0	0	0	0	0
						0	0	0	0	0	0	0	0	0	0	0	0
NA	NA	NA	NA	NA	NA	1	1	4.3	1	25	1	1	1	1	1	14	##
NA	NA	NA	NA	NA	NA	1	2	0.8	1	1	13	1	0	2	1	0	1
NA	NA	NA	NA	NA	NA	1	1	11	1	8	1	7	11	13	1	0	8
						0	0	0	0	0	0	0	0	0	0	0	0
						0	0	0	40	##	0	##	83	56	##	0	0
Fatty acid	Fatty Acyls	Fatty acyls	NA	NA	NA	0	1	0.3	0	1	3	1	2	0	1	0	80
NA	NA	NA	NA	NA	NA	1	1	0.9	2	2	1	1	1	1	1	2	2
Methoxyp	Phenols	Phenols ar	NA	NA	NA	1	1	1.5	1	4	1	5	0	2	1	1	0
NA	NA	NA	NA	NA	NA	1	1	1.8	1	2	1	2	2	1	0	2	1
NA	NA	NA	NA	NA	NA	1	1	2	1	1	1	2	1	3	2	1	1
NA	NA	NA	NA	NA	NA	1	0	0.6	1	1	0	0	1	0	3	0	0
NA	NA	NA	NA	NA	NA	1	1	1.1	1	2	2	1	1	1	1	1	1
NA	NA	NA	NA	NA	NA	1	1	1.7	1	1	1	1	1	1	1	1	1
						0	0	0	0	0	0	0	0	0	0	0	0
Arylsulfate	Organic su	Organic ac	NA	NA	NA	0	0	0.1	2	23	3	3	13	0	1	8	0
Indolyl car	Indoles an	Indole anc	NA	NA	NA	2	1	0.4	1	1	0	1	2	0	1	1	3
NA	NA	NA	NA	NA	NA	1	1	1.2	1	2	2	2	0	0	1	1	2
NA	NA	NA	NA	NA	NA	3	1	0.2	2	0	0	0	0	1	1	0	1
NA	NA	NA	NA	NA	NA	1	1	0.3	1	1	0	2	2	1	6	1	13
NA	NA	NA	NA	NA	NA	16	34	0.8	0	0	##	0	0	0	0	0	36
NA	NA	NA	NA	NA	NA	1	1	1.1	1	1	1	1	1	1	1	1	1
Pyrimidine	Pyrimidine	Nucleic ac	NA	NA	NA	1	1	0.5	2	0	1	1	1	0	0	0	0
NA	NA	NA	NA	NA	NA	1	4	0	2	0	11	0	2	0	1	1	13
NA	NA	NA	NA	NA	NA	1	1	1.2	1	1	1	1	1	1	1	2	2
NA	NA	NA	NA	NA	NA	1	0	1.1	2	1	0	1	0	27	1	1	0
NA	NA	NA	NA	NA	NA	2	1	1.3	1	2	1	2	1	1	1	1	1
NA	NA	NA	NA	NA	NA	1	1	0.6	4	9	1	13	1	1	1	1	2
NA	NA	NA	NA	NA	NA	0	1	3.2	8	4	1	0	1	0	11	16	1
						0	0	0	22	##	3	0	47	##	57	2	0
NA	NA	NA	NA	NA	NA	0	1	1.5	1	2	1	2	3	0	0	3	0
NA	NA	NA	NA	NA	NA	1	1	0.6	1	1	1	1	1	1	1	1	1
NA	NA	NA	NA	NA	NA	1	1	1.9	1	2	2	2	1	1	1	1	1
NA	NA	NA	NA	NA	NA	28	1	1.7	1	2	1	1	1	1	1	1	1
NA	NA	NA	NA	NA	NA	1	1	1.5	1	2	1	2	1	1	1	2	3
						0	0	0	0	0	0	0	0	12	0	0	0
NA	NA	NA	NA	NA	NA	3	1	0.8	1	1	1	1	3	14	0	1	2
						0	0	0	0	2	0	0	0	12	2	0	0
NA	NA	NA	NA	NA	NA	1	1	1.7	1	2	1	2	1	1	1	2	0
						0	0	0	0	0	0	0	3	0	0	0	0
						0	0	0	0	0	0	0	0	3	0	0	0
Carbohydı	Organoox	Carbohydı	NA	NA	NA	1	1	1.4	4	0	0	0	2	1	5	1	0
Amines	Organonit	Amines	NA	NA	NA	1	1	1.8	1	1	1	1	1	1	1	1	20
Carbohydı	Organoox	Carbohydı	NA	NA	NA	1	1	0.3	1	3	1	2	1	3	3	1	4
NA	NA	NA	NA	NA	NA	1	1	0.9	2	1	1	1	1	1	3	2	16
Benzoic ac	Benzene a	Benzene a	NA	NA	NA	8	1	1.4	1	1	1	1	3	1	1	1	1

Pyranones	Pyrans	Organohe	Metabolis	Polyketide	NA	1	0	3.9	0	3	2	1	0	0	2	1	0
NA	NA	NA	NA	NA	NA	2	7	0.4	0	2	0	0	1	0	2	0	1
Amino aci	Carboxylic	Amino aci	NA	NA	NA	1	1	0.6	1	1	6	1	1	1	2	1	1
NA	NA	NA	Biosynthes	Acarbose	NA	5	1	0.7	1	1	1	0	1	0	1	1	1
						0	0	0	0	0	0	0	5	0	12	0	0
NA	NA	NA	NA	NA	NA	1	1	1.8	0	2	1	2	1	1	1	1	0
Amino aci	Carboxylic	Amino aci	NA	NA	NA	1	1	0.9	2	2	1	1	1	1	1	2	1
NA	NA	NA	NA	NA	NA	0	0	2	0	1	0	0	1	4	8	0	0
NA	NA	NA	NA	NA	NA	0	1	0.4	1	11	1	8	7	2	0	0	0
NA	NA	NA	NA	NA	NA	1	2	0.6	1	3	1	9	1	0	1	1	9
Carbohyd	Organoox	Carbohyd	NA	NA	NA	1	1	0.7	1	3	##	6	1	3	1	1	3
Ceramides	Sphingolip	Sphingolip	NA	NA	NA	3	1	0.1	3	2	6	1	0	1	1	1	1
NA	Benzazepi	Organohe	NA	NA	NA	1	1	0.7	1	1	1	1	1	21	1	1	0
NA	NA	NA	NA	NA	NA	1	1	1	1	1	1	1	1	1	1	1	1
NA	NA	NA	NA	NA	NA	1	1	0.9	1	1	4	5	1	1	1	1	1
						0	0	0	0	##	0	0	0	0	0	0	0
NA	NA	NA	NA	NA	NA	1	1	0.7	2	1	1	1	1	0	2	5	0
NA	NA	NA	NA	NA	NA	0	0	0.3	0	4	34	0	1	4	0	2	2
NA	NA	NA	NA	NA	NA	7	1	0.8	1	4	1	16	1	1	1	3	3
NA	NA	NA	NA	NA	NA	1	1	1.1	1	1	1	1	1	1	1	1	1
NA	NA	NA	NA	NA	NA	1	1	2.3	1	2	1	2	1	1	1	2	0
NA	NA	NA	NA	NA	NA	41	0	0.5	0	1	0	1	0	0	1	1	1
NA	NA	NA	NA	NA	NA	4	0	0.4	1	3	11	4	1	0	0	3	2
NA	NA	NA	NA	NA	NA	0	4	1.9	1	1	1	4	1	4	1	1	0
NA	NA	NA	NA	NA	NA	1	1	1.2	1	2	1	1	1	1	1	1	1
NA	NA	NA	NA	NA	NA	1	0	0.2	2	0	0	0	0	14	1	1	1
NA	NA	NA	NA	NA	NA	0	3	2.7	1	9	1	1	0	1	1	0	0
NA	NA	NA	NA	NA	NA	1	1	0.5	0	2	2	2	1	1	1	2	1
NA	NA	NA	NA	NA	NA	1	0	0.6	3	0	1	1	7	0	1	1	1
Fatty acids	Fatty Acyls	Fatty acyls	NA	NA	NA	1	1	0.3	0	2	39	5	1	2	0	0	12
						0	0	0	0	0	0	0	0	0	0	0	0
						0	0	0	0	0	0	0	0	0	0	0	0
						0	0	0	0	0	0	0	0	0	13	0	0
NA	NA	NA	NA	NA	NA	8	0	0.7	1	7	##	9	0	1	0	6	0
NA	NA	NA	NA	NA	NA	1	1	1.1	1	1	1	2	1	1	2	1	4
NA	NA	NA	NA	NA	NA	1	1	1.4	1	1	1	1	18	1	1	1	2
NA	NA	NA	NA	NA	NA	80	1	0.6	13	40	0	39	0	1	0	46	10
						0	0	0	0	20	0	0	13	80	29	0	23
NA	NA	NA	NA	NA	NA	0	1	0.7	2	0	1	1	1	1	1	2	7
NA	NA	NA	NA	NA	NA	2	0	4.1	1	30	1	0	0	2	0	13	##
NA	NA	NA	NA	NA	NA	1	0	1.8	1	1	0	0	1	0	2	0	0
NA	NA	NA	NA	NA	NA	5	2	1.1	1	1	1	1	1	1	0	2	1
NA	NA	NA	NA	NA	NA	1	1	0.7	0	6	0	1	0	9	1	1	1
NA	NA	NA	NA	NA	NA	1	1	1	1	2	1	1	1	1	1	1	1
NA	NA	NA	Amino aci	Phenylalar	map0040C	1	1	1	1	0	2	0	1	0	1	1	0
NA	NA	NA	NA	NA	NA	1	1	1.9	1	2	1	2	1	1	1	1	1
NA	NA	NA	NA	NA	NA	1	1	0.7	1	1	1	1	1	1	1	2	1
NA	NA	NA	NA	NA	NA	1	1	1.1	1	1	2	1	1	1	1	1	1
NA	NA	NA	NA	NA	NA	1	1	1.3	1	1	1	1	1	1	1	4	1
						0	0	0	0	4	0	60	0	0	0	0	0
Benzyl alc	Benzene a	Alcohols	NA	NA	NA	1	1	1.3	1	2	1	2	0	1	1	1	2
						0	0	0	0	0	0	0	0	0	0	0	0
						0	0	0	0	0	0	0	0	0	0	0	0
						0	0	0	0	0	0	0	0	0	0	0	0
NA	NA	NA	NA	NA	NA	1	1	0.8	1	1	1	0	1	1	1	1	5
NA	NA	NA	NA	NA	NA	0	1	0.3	2	2	0	2	1	2	0	1	0
NA	NA	NA	NA	NA	NA	0	0	0.2	1	0	0	1	1	27	1	3	0
NA	NA	NA	NA	NA	NA	1	1	1.2	1	2	1	2	1	2	1	2	1

Ceramides	Sphingolip	Sphingolip	NA	NA	NA	1	1	1.9	1	2	5	2	1	1	1	2	1
NA	NA	NA	NA	NA	NA	5	0	0.1	20	5	5	1	0	1	0	1	0
Benzenedi	Phenols	Phenols ar	NA	NA	NA	1	1	0.6	1	0	1	1	1	1	1	1	1
NA	NA	NA	NA	NA	NA	0	0	1.3	2	0	1	0	3	0	4	3	0
NA	NA	NA	NA	NA	NA	1	1	1.5	1	2	1	1	2	1	1	2	1
NA	NA	NA	NA	NA	NA	0	3	0.1	2	0	4	1	3	0	1	1	1
NA	NA	NA	NA	NA	NA	1	1	0.7	1	1	1	1	1	0	1	2	1
NA	NA	NA	NA	NA	NA	1	1	1.7	1	2	4	4	1	2	1	1	1
NA	NA	NA	NA	NA	NA	1	4	0.6	1	1	1	0	1	0	1	1	1
NA	NA	NA	NA	NA	NA	1	1	0.9	1	1	0	2	2	1	1	1	1
Glyceroph	Glyceroph	Glyceroph	NA	NA	NA	1	1	0.5	0	3	10	3	1	0	0	1	1
NA	NA	NA	NA	NA	NA	1	1	0.8	1	60	1	16	5	2	1	1	1
NA	NA	NA	NA	NA	NA	1	1	0.8	1	0	1	0	2	1	1	2	1
NA	NA	NA	NA	NA	NA	1	1	0.8	2	2	1	1	1	1	1	2	2
Diphenyle	Benzene a	Benzene a	NA	NA	NA	2	1	0.7	9	1	1	1	1	1	1	1	1
NA	NA	NA	NA	NA	NA	1	1	0.6	11	1	3	1	1	0	1	2	1
NA	NA	NA	NA	NA	NA	1	1	0.9	1	1	3	1	1	1	1	1	1
						0	0	0	0	0	0	0	0	6	8	0	0
						0	0	0	0	0	0	0	0	0	0	0	0
NA	NA	NA	NA	NA	NA	2	1	1.2	1	0	1	1	1	0	1	3	2
Cyanogen	Cyanogen	Amino aci	NA	NA	NA	1	1	0.7	1	1	1	1	1	1	1	1	1
						0	0	0	20	##	0	56	##	34	##	0	0
						0	0	0	0	0	0	0	41	0	0	0	0
NA	NA	NA	NA	NA	NA	0	1	1.7	1	1	2	1	1	0	1	2	7
NA	NA	NA	NA	NA	NA	1	1	0.6	1	1	1	1	0	1	1	1	1
NA	NA	NA	NA	NA	NA	2	1	1.1	3	18	##	40	0	1	1	1	1
NA	NA	NA	NA	NA	NA	0	1	0.5	1	0	0	1	3	1	1	1	1
NA	NA	NA	NA	NA	NA	1	1	1.8	1	1	1	1	1	1	1	1	1
NA	NA	NA	NA	NA	NA	1	2	1.5	3	4	2	0	0	0	1	1	0
NA	NA	NA	NA	NA	NA	1	0	2.8	0	0	7	0	7	0	2	1	0
Arylsulfate	Organic su	Organic ac	NA	NA	NA	1	0	2.6	0	0	7	0	7	0	1	1	0
NA	NA	NA	NA	NA	NA	1	1	0.8	1	1	1	1	1	5	1	1	1
						0	0	10	28	42	12	70	##	21	##	0	0
NA	NA	NA	NA	NA	NA	1	0	0.3	1	0	1	0	1	1	1	1	1
FA0802 N-	FA08 Fatty	Fatty acyls	NA	NA	map04080	2	1	0.8	0	2	17	2	0	1	0	4	2
NA	NA	NA	NA	NA	NA	2	0	0.5	4	0	0	0	1	2	1	1	2
Glyceroph	Glyceroph	Glyceroph	NA	NA	NA	2	0	0.1	##	0	4	2	0	1	0	20	0
NA	NA	NA	NA	NA	NA	1	1	0.9	1	2	1	5	2	2	4	2	1
NA	Tetrapyrro	Organohe	Metabolis	Porphyrin	map00860	0	1	1.2	0	1	0	2	2	1	1	2	1
						0	0	0	13	14	0	65	39	18	0	0	0
NA	NA	NA	NA	NA	NA	1	3	0.2	3	0	4	0	3	0	0	0	3
NA	NA	NA	NA	NA	NA	1	2	0.2	3	23	1	22	0	1	0	0	1
Fatty acids	Fatty Acyls	Fatty acyls	NA	NA	NA	1	1	1.1	1	3	2	3	1	1	1	1	1
NA	NA	NA	NA	NA	NA	3	0	0.4	0	1	1	2	9	5	54	1	8
						0	0	0	0	0	0	0	24	0	0	0	0
NA	NA	NA	NA	NA	NA	1	0	1.2	1	0	1	1	1	0	1	1	1
						0	0	0	0	20	0	0	0	0	0	0	0
NA	NA	NA	NA	NA	NA	1	1	0.7	1	1	1	1	1	1	1	1	1
NA	NA	NA	NA	NA	NA	1	1	0.3	1	1	1	5	2	2	1	0	1
Fatty acid	Fatty Acyls	Fatty acyls	NA	NA	NA	1	1	1.9	1	2	1	2	1	1	1	2	1
Tetraterpe	Prenol lipi	Terpenoid	Metabolis	Carotenoi	NA	1	1	1.9	1	2	1	2	1	1	1	2	1
NA	NA	NA	NA	NA	NA	1	1	1.5	1	2	2	2	1	2	1	1	1
						0	0	0	0	0	0	0	0	5	0	0	0
						0	0	0	0	0	0	0	0	18	0	0	0
						0	0	0	0	0	0	0	0	0	0	0	0
						0	0	0	0	0	0	0	0	0	0	0	0
						0	0	0	0	0	0	0	0	0	0	0	0
NA	NA	NA	NA	NA	NA	2	0	0.7	6	0	1	0	2	1	1	1	6

Diphenylm	Benzene a	Benzene a	NA	NA	NA	0	0	5	0	2	1	3	3	1	1	0	1
Hydroxycii	Cinnamic a	Polyketide	NA	NA	NA	20	0	0.3	1	0	5	0	5	0	1	2	1
Amino aci	Carboxylic	Amino aci	NA	NA	NA	1	1	1.5	1	2	1	1	1	1	1	1	1
NA	NA	NA	NA	NA	NA	1	1	0.8	1	2	2	1	1	0	0	0	0
Arylsulfate	Organic su	Organic ac	NA	NA	NA	0	0	0.2	27	2	1	12	0	0	7	57	0
NA	NA	NA	NA	NA	NA	1	1	0.6	0	1	6	1	3	##	1	4	1
NA	NA	NA	NA	NA	NA	0	1	0.9	1	1	0	1	0	5	4	7	0
NA	NA	NA	NA	NA	NA	0	1	1.3	1	1	1	2	1	1	1	1	1
NA	NA	NA	NA	NA	NA	0	1	1.2	1	0	17	0	0	1	0	1	0
Amino aci	Carboxylic	Amino aci	Amino aci	Arginine b	map00220	1	1	2	1	1	1	2	1	1	0	2	1
NA	NA	NA	NA	NA	NA	0	1	0.1	0	1	1	1	0	1	1	0	6
NA	NA	NA	NA	NA	NA	6	0	0.7	2	5	##	11	1	1	0	5	1
Fatty acids	Fatty Acyls	Fatty acyls	NA	NA	NA	2	1	6	1	3	6	6	0	0	1	3	0
NA	NA	NA	NA	NA	NA	1	1	1.4	2	1	1	0	1	0	2	1	1
Androstan	Steroids a	Steroids a	NA	NA	NA	1	1	0.4	3	2	1	4	4	4	1	1	6
NA	NA	NA	NA	NA	NA	1	1	1.9	1	2	1	2	1	1	1	2	1
Arylsulfate	Organic su	Organic ac	NA	NA	NA	2	0	0.5	1	0	5	0	4	0	0	2	1
NA	NA	NA	NA	NA	NA	1	2	1.6	2	23	1	20	1	1	1	1	1
NA	NA	NA	NA	NA	NA	1	1	0.8	0	3	9	8	3	1	1	2	0
NA	NA	NA	NA	NA	NA	0	1	0.1	5	2	1	6	11	3	1	0	8
NA	NA	NA	NA	NA	NA	0	3	0.4	0	1	1	7	4	3	0	0	6
Amino aci	Carboxylic	Amino aci	NA	NA	NA	1	1	0.8	1	1	1	1	1	9	1	1	3
NA	NA	NA	NA	NA	NA	1	1	2	1	2	1	2	1	1	1	2	1
NA	NA	NA	NA	NA	NA	1	1	0.6	1	0	0	0	1	1	1	1	17
NA	NA	NA	NA	NA	NA	1	1	35	0	39	3	39	1	2	1	4	5
NA	NA	NA	NA	NA	NA	1	1	1.4	1	2	1	2	1	1	1	1	1
						0	0	2	0	6	0	0	73	0	0	0	8
						0	0	0	89	##	2	##	##	##	##	0	41
NA	NA	NA	NA	NA	NA	1	1	0.9	1	1	1	1	1	##	1	1	11
NA	NA	NA	NA	NA	NA	1	1	1.7	1	2	1	2	1	1	1	1	1
Hydroxyst	Steroids a	Steroids a	Lipid meta	Steroid ho	map00140	1	1	0.7	0	4	19	13	2	5	0	3	0
Carbonyl c	Organoox	Organic o	Metabolis	Cyanoami	map0110	1	1	0.3	0	4	1	20	0	1	1	1	1
						0	0	0	0	0	0	0	0	0	0	0	0
						0	0	0	0	0	0	0	0	0	0	0	0
						0	0	0	0	0	0	0	0	0	0	0	0
						0	0	0	0	0	0	0	0	0	0	0	0
						0	0	0	0	0	0	0	0	0	0	0	0
						0	0	0	0	0	0	0	0	0	0	0	0
						0	0	0	0	0	0	0	0	0	0	0	0
						0	0	0	0	0	0	0	0	0	0	0	0
						0	0	0	0	0	0	0	0	0	0	0	0
						0	0	0	0	0	0	0	0	0	0	0	0
						0	0	0	0	0	0	0	0	0	0	0	0
						0	0	0	0	0	0	0	0	0	0	0	0
						0	0	0	0	0	0	0	0	0	0	0	0
						0	0	0	0	0	0	0	0	0	0	0	0
						0	0	0	0	0	0	0	0	0	0	0	0
						0	0	0	0	0	0	0	0	0	0	0	0
NA	NA	NA	NA	NA	NA	1	1	2.7	5	5	1	3	0	1	1	2	0
NA	NA	NA	NA	NA	NA	0	1	0.9	0	2	1	2	1	1	1	1	1
NA	NA	NA	NA	NA	NA	0	5	1.8	1	7	1	0	0	0	0	0	0
Glyceroph	Glyceroph	Amines an	NA	NA	NA	##	2	3.7	1	5	1	##	1	11	10	8	1
						0	0	0	0	0	0	0	7	0	61	0	8
NA	NA	NA	NA	NA	NA	5	1	0.7	2	12	1	25	1	1	0	2	3
NA	NA	NA	NA	NA	NA	1	1	0.6	2	1	1	1	1	2	1	2	1
Pyrimidine	Diazines	Organohe	NA	NA	NA	1	1	0.5	1	1	0	1	1	1	1	1	4
NA	NA	NA	NA	NA	NA	0	1	1.8	2	1	1	1	2	1	1	1	0
NA	NA	NA	Biosynthes	Tropane, p	NA	1	1	4	1	1	1	4	0	1	1	3	1

Isoflav-2-(	Isoflavono	Flavonoids	Biosynthes	Isoflavono	NA	1	0	1.2	0	1	0	2	15	0	0	1	18
NA	NA	NA	NA	NA	NA	1	1	0.8	1	0	0	0	1	1	1	2	0
Amino aci	Carboxylic	Amino aci	NA	NA	NA	1	1	0.9	2	2	1	1	1	1	1	2	1
NA	NA	NA	NA	NA	NA	1	1	1.4	1	2	2	2	1	1	1	1	24
NA	NA	NA	NA	NA	NA	1	1	0.6	1	1	1	1	1	1	1	1	1
Cucurbitar	Triterpeno	Terpenoid	NA	NA	NA	1	2	1.8	1	2	1	2	1	1	1	1	1
						0	0	0	4	90	0	0	62	0	13	0	0
NA	NA	NA	Chemical s	Biosynthes	NA	0	1	1.1	1	2	8	2	0	0	1	1	2
						0	0	0	0	0	0	0	0	0	23	0	0
						0	0	0	0	0	0	0	0	0	0	0	0
						0	0	0	0	0	0	0	0	0	0	0	0
						0	2	0	51	##	4	##	##	##	##	##	##
NA	NA	NA	NA	NA	NA	1	1	1.5	2	0	1	1	2	0	0	2	1
NA	NA	NA	NA	NA	NA	1	1	1.3	1	2	1	2	1	1	1	1	1
NA	NA	NA	NA	NA	NA	1	1	1.4	1	2	1	2	2	2	1	1	1
NA	NA	NA	NA	NA	NA	1	0	0.2	1	1	1	3	1	0	1	1	3
NA	NA	NA	NA	NA	NA	1	1	1.1	1	1	1	1	1	1	1	1	1
NA	NA	NA	NA	NA	NA	1	2	1.4	1	1	1	3	10	2	1	1	1
						0	0	0	0	0	0	0	17	13	6	0	15
NA	NA	NA	NA	NA	NA	1	1	0.8	1	1	1	1	0	12	1	1	2
NA	NA	NA	NA	NA	NA	1	1	1.1	1	1	1	1	1	1	1	2	1
Amino aci	Carboxylic	Amino aci	Metabolisı	Biotin met	map0078C	1	1	0.8	1	1	1	1	1	1	1	1	1
Benzodiaz	Diazanaph	Organohe	NA	NA	NA	1	1	0.4	6	0	0	0	2	0	5	0	0
Glyceroph	Glyceroph	Glyceroph	NA	NA	NA	1	1	1.8	1	2	1	2	1	1	1	1	1
NA	NA	NA	NA	NA	NA	1	1	0.2	3	1	0	0	0	0	2	1	0
NA	NA	NA	NA	NA	NA	1	1	1.3	1	2	1	2	1	1	1	1	1
NA	NA	NA	NA	NA	NA	1	1	1.4	1	4	1	2	1	1	1	1	16
NA	Morphinar	Alkaloids a	NA	NA	NA	5	6	0.9	1	0	10	0	0	0	2	0	56
NA	NA	NA	NA	NA	NA	2	1	1.5	1	2	1	1	0	0	1	1	0
Pseudogu	Sesquiterp	Terpenoid	NA	NA	NA	1	1	1.2	1	1	1	2	1	1	1	1	13
NA	NA	NA	NA	NA	NA	0	1	0.3	5	0	0	0	2	0	0	0	0
Glucosinol	Glucosinol	Amino aci	Biosynthes	Glucosinol	map0121(	2	1	1.2	1	3	3	2	1	1	1	1	1
NA	NA	NA	NA	NA	NA	0	0	0.4	1	0	0	0	1	29	1	1	11
NA	NA	NA	NA	NA	NA	1	1	1.2	1	1	39	1	1	1	1	1	1
NA	NA	NA	NA	NA	NA	1	1	6.4	3	1	1	7	1	1	3	9	2
						0	0	0	0	0	0	0	0	0	0	0	0
Decarbam	Paralytic sl	Marine bic	NA	NA	NA	1	1	2.3	1	2	1	2	1	1	1	2	1
NA	NA	NA	NA	NA	NA	1	1	0.9	1	1	1	0	1	7	1	1	1
NA	NA	NA	NA	NA	NA	1	0	1.4	1	1	1	1	1	1	1	1	1
NA	NA	NA	NA	NA	NA	1	1	2.3	0	1	1	1	7	12	3	1	14
NA	NA	NA	NA	NA	NA	1	1	3.3	2	7	0	13	0	0	2	1	0
						0	0	0	0	0	0	0	0	0	0	0	21
						0	6	0	73	##	10	##	##	##	##	0	32
NA	NA	NA	NA	NA	NA	1	1	0.7	1	1	1	0	1	3	2	1	0
NA	NA	NA	NA	NA	NA	1	2	1.8	1	1	1	2	1	0	1	1	0
Carbohydr	Organoox	Carbohydr	NA	NA	NA	1	1	1.9	1	1	1	3	1	1	1	1	1
						0	0	0	0	0	0	0	0	0	0	0	0
NA	NA	NA	NA	NA	NA	1	0	0.6	0	3	1	2	1	1	6	1	16
NA	NA	NA	NA	NA	NA	0	1	5.7	3	3	1	16	0	1	6	5	8
Glyceroph	Glyceroph	Glyceroph	NA	NA	NA	0	1	1	1	2	1	1	1	1	1	1	2
NA	NA	NA	NA	NA	NA	1	1	0.6	1	1	1	1	1	1	1	1	2
Carbohydr	Organoox	Carbohydr	NA	NA	NA	1	1	0.7	1	1	1	1	1	1	1	1	2
						0	0	0	0	0	0	0	0	0	0	0	0
Amino aci	Carboxylic	Amino aci	NA	NA	NA	23	0	1.1	0	2	3	0	2	1	4	3	0
NA	NA	NA	NA	NA	NA	1	0	0.2	2	0	1	0	0	0	3	1	0
Substitute	Pyrroles	Organohe	NA	NA	NA	1	1	1.2	1	1	1	1	1	1	1	1	1
NA	NA	NA	NA	NA	NA	1	1	0.9	1	1	1	0	1	1	1	1	1
NA	NA	NA	NA	NA	NA	1	1	1.7	1	2	1	2	1	2	1	2	1

NA	NA	NA	NA	NA	NA	1	1	2.6	1	2	2	2	1	1	1	3	1
NA	NA	NA	NA	NA	NA	7	2	0.2	1	6	1	1	1	0	1	40	0
Pyrrolizidi	Alkaloids (	Alkaloids	NA	NA	NA	0	1	1.1	1	1	1	1	0	87	1	2	3
						0	0	0	0	0	0	36	0	0	0	0	0
						0	0	0	0	0	0	0	0	0	0	0	0
NA	NA	NA	NA	NA	NA	0	0	0.5	2	1	1	2	1	0	0	4	0
NA	NA	NA	NA	NA	NA	0	0	0.4	1	0	0	1	1	4	1	0	1
Androstan	Steroids ar	Steroids ar	Lipid meta	Steroid ho	map0014C	1	1	0.8	1	7	1	1	1	1	1	1	1
NA	NA	NA	NA	NA	NA	2	1	1.4	3	4	6	6	1	2	1	1	4
						0	0	0	0	0	0	0	17	0	38	0	0
Quinazolir	Alkaloids (	Alkaloids	NA	NA	NA	7	0	4.9	0	0	0	0	2	0	3	0	0
NA	NA	NA	NA	NA	NA	1	1	1.7	1	2	1	2	1	1	1	1	1
NA	NA	NA	NA	NA	NA	1	1	0.7	2	1	2	1	1	1	1	2	1
Amines	Organonit	Amines	NA	NA	NA	1	1	1.9	1	30	1	7	1	5	1	2	2
Null	FA10 Fatty	Fatty acyls	NA	NA	NA	1	1	0.8	0	1	3	1	6	2	1	1	6
NA	NA	NA	NA	NA	NA	1	0	1	3	0	1	0	2	0	2	2	0
NA	NA	NA	NA	NA	NA	1	1	46	5	10	1	5	1	1	1	2	7
NA	NA	NA	NA	NA	NA	1	1	1.5	1	1	1	1	1	1	1	2	1
NA	NA	NA	NA	NA	NA	1	1	1.3	1	1	1	1	1	1	1	1	1
NA	NA	NA	NA	NA	NA	1	1	2.1	1	2	1	2	1	1	1	2	3
NA	NA	NA	NA	NA	NA	1	1	0.8	1	0	1	0	2	2	1	2	0
NA	NA	NA	NA	NA	NA	1	1	0.2	0	0	1	0	3	1	2	1	4
						0	0	0	0	0	0	0	0	0	44	0	0
NA	NA	NA	Metabolisı	Biosynthes	NA	1	1	0.9	1	1	1	1	1	1	1	1	2
NA	NA	NA	NA	NA	NA	1	3	0.3	1	0	0	0	1	1	1	1	1
						0	0	0	0	0	0	0	0	4	0	0	0
NA	NA	NA	NA	NA	NA	1	1	2.2	1	2	1	2	1	1	1	2	1
NA	NA	NA	NA	NA	NA	1	1	1.4	1	1	1	1	2	1	2	1	3
Flavonoid	Flavonoids	Flavonoids	NA	NA	NA	2	1	1.2	1	2	1	2	1	1	1	1	1
NA	NA	NA	NA	NA	NA	1	1	1.9	1	2	1	2	1	1	1	1	0
NA	NA	NA	NA	NA	NA	0	2	1.3	2	2	0	0	0	0	1	0	0
NA	NA	NA	NA	NA	NA	0	1	0.5	0	4	8	2	0	1	0	2	2
NA	NA	NA	NA	NA	NA	1	1	0.9	0	2	8	3	2	0	0	2	1
NA	NA	NA	NA	NA	NA	3	2	0.5	1	1	1	0	2	1	1	2	1
NA	NA	NA	NA	NA	NA	1	1	1.7	1	2	1	2	1	1	1	1	1
Amino aci	Carboxylic	Amino aci	NA	NA	NA	4	0	0.7	1	1	3	1	1	4	2	1	1
NA	NA	NA	NA	NA	NA	1	1	0.5	2	0	1	0	2	0	1	1	1
NA	NA	NA	NA	NA	NA	1	1	1.1	1	1	1	1	1	1	1	1	1
NA	NA	NA	NA	NA	NA	4	1	1.2	3	0	3	0	1	0	1	2	1
NA	NA	NA	NA	NA	NA	15	1	0.4	3	10	1	24	1	0	1	7	3
Fatty acid	Fatty Acyls	Fatty acyls	NA	NA	NA	1	7	0.8	1	1	1	1	7	2	1	1	32
NA	NA	NA	NA	NA	NA	1	0	0.2	1	7	0	1	2	1	1	0	40
NA	NA	NA	NA	NA	NA	1	1	1.6	1	0	1	0	1	0	1	1	0
NA	NA	NA	NA	NA	NA	1	1	0.4	1	0	1	0	0	0	1	0	4
NA	NA	NA	NA	NA	NA	0	0	1.4	1	2	1	2	0	0	0	1	1
NA	NA	NA	NA	NA	NA	1	1	0.2	1	0	1	0	1	0	2	0	0
NA	NA	NA	NA	NA	NA	1	1	1.4	1	1	1	1	5	1	1	1	1
NA	NA	NA	NA	NA	NA	1	0	2.4	1	19	0	1	0	1	1	2	1
NA	NA	NA	NA	NA	NA	1	0	0.2	1	0	0	0	0	##	0	1	12
Amino aci	Carboxylic	Amino aci	Carbohydr	Pyruvate n	map00620	3	4	0.3	3	0	11	0	0	0	0	1	24
NA	NA	NA	NA	NA	NA	1	1	1	1	2	55	1	1	1	1	1	1
NA	NA	NA	NA	NA	NA	1	0	2.4	1	1	1	1	2	1	1	1	1
NA	NA	NA	NA	NA	NA	1	1	0.8	1	2	1	2	1	1	1	1	1
NA	NA	NA	NA	NA	NA	1	1	1.5	1	3	1	1	1	1	1	1	1
						0	0	0	0	0	0	0	0	0	0	0	0
						0	0	0	0	0	0	0	0	0	0	0	0
						0	0	0	0	0	0	0	0	0	0	0	0
						0	0	0	0	0	0	0	0	0	0	0	0

						0	0	0	0	0	0	0	0	0	0	0	0
						0	0	0	0	0	0	0	0	0	0	0	0
NA	NA	NA	NA	NA	NA	1	1	1	1	1	1	0	1	1	1	1	1
NA	NA	NA	NA	NA	NA	9	1	0.7	9	41	4	0	4	2	0	17	3
NA	NA	NA	NA	NA	NA	1	1	0.8	1	4	1	5	1	1	1	1	1
NA	NA	NA	NA	NA	NA	2	1	0.3	3	2	0	0	0	1	1	0	4
NA	NA	NA	NA	NA	NA	1	1	0.3	2	1	0	0	1	0	2	0	0
NA	NA	NA	NA	NA	NA	1	1	0.8	1	1	1	1	2	1	1	1	1
NA	NA	NA	NA	NA	NA	1	1	1.1	1	16	1	1	1	2	1	1	1
NA	NA	NA	NA	NA	NA	1	1	1.2	1	3	1	1	1	1	1	1	1
NA	NA	NA	NA	NA	NA	2	1	1.8	3	1	0	3	1	1	1	2	5
NA	NA	NA	NA	NA	NA	1	1	0.7	2	0	1	0	1	1	1	2	1
NA	NA	NA	NA	NA	NA	1	1	0.6	1	1	1	1	2	2	2	1	1
Tiglianes	Diterpenoi	Terpenoid	NA	NA	NA	1	1	0.7	1	0	1	0	1	1	1	2	1
NA	NA	NA	NA	NA	NA	0	0	1.5	1	1	36	1	1	1	1	1	0
NA	NA	NA	NA	NA	NA	1	1	0.6	2	1	1	1	1	1	0	1	2
Benzoic ac	Benzene a	Benzene a	NA	NA	NA	1	3	0.2	3	0	4	0	2	0	1	1	3
NA	NA	NA	NA	NA	NA	2	1	1.5	1	2	1	1	3	1	1	2	1
NA	NA	NA	NA	NA	NA	1	0	2.5	0	8	1	1	##	1	1	25	17
Benzylethe	Benzene a	Benzene a	NA	NA	NA	1	0	2.5	0	8	1	1	##	1	1	25	17
NA	NA	NA	NA	NA	NA	1	1	4	1	1	1	1	1	1	1	1	1
NA	NA	NA	NA	NA	NA	0	0	0.1	1	0	0	1	0	35	2	5	1
Pyridine al	Alkaloids (	Alkaloids	Metabolisı	Nicotinate	map0076C	1	2	2.8	1	5	3	7	0	1	4	4	2
NA	NA	NA	NA	NA	NA	1	1	0.6	3	1	1	1	1	1	1	1	1
NA	NA	NA	NA	NA	NA	1	1	1.4	1	1	1	1	1	1	2	2	1
NA	NA	NA	NA	NA	NA	1	1	1.5	1	2	1	5	1	1	1	1	1
NA	NA	NA	NA	NA	NA	1	1	1.4	1	1	1	2	1	1	2	1	0
						0	0	0	0	4	0	0	2	0	3	0	0
NA	NA	NA	NA	NA	NA	1	1	0.4	3	1	0	0	0	0	1	0	0
NA	NA	NA	Biosynthes	Staurospo	map0110(	1	1	6.2	0	1	1	3	1	1	1	1	6
Carbohydı	Organoox	Carbohydı	NA	NA	NA	1	1	1.8	1	1	17	2	1	1	1	2	1
NA	NA	NA	NA	NA	NA	1	0	0.5	1	1	23	1	1	0	1	1	8
						0	0	0	0	0	0	0	0	0	0	0	0
						0	0	0	0	0	0	0	0	0	0	0	0
NA	NA	NA	NA	NA	NA	1	1	1.9	1	2	1	2	1	1	1	2	1
NA	NA	NA	NA	NA	NA	1	1	1.7	1	1	1	1	1	1	1	1	2
Carbohydı	Organoox	Carbohydı	Carbohydı	Pentose pl	map0003C	1	0	1.4	2	1	1	1	1	0	5	3	1
NA	NA	NA	NA	NA	NA	1	1	0.6	1	0	2	0	3	1	3	3	3
NA	NA	NA	NA	NA	NA	0	1	1.2	2	1	1	1	1	0	1	1	2
Benzoic ac	Benzene a	Benzene a	NA	NA	NA	2	1	3.4	0	1	0	1	7	0	1	1	32
Amino aci	Carboxylic	Amino aci	Amino aci	Phenylalar	map0036C	1	1	0.6	3	2	1	1	1	2	1	0	1
NA	NA	NA	NA	NA	NA	1	1	0.8	1	2	1	1	0	1	1	1	1
NA	NA	NA	NA	NA	NA	1	1	2.7	1	1	2	3	1	1	1	1	0
Carboxylic	Carboxylic	Organic ac	NA	NA	NA	1	1	1.2	1	1	1	1	1	1	1	5	1
NA	NA	NA	NA	NA	NA	1	1	0.9	1	1	1	0	1	1	1	1	1
NA	NA	NA	NA	NA	NA	1	1	0.7	1	1	1	1	1	1	1	1	2
Benzoic ac	Benzene a	Benzene a	NA	NA	NA	3	1	1.5	1	3	2	14	1	1	1	1	1
NA	NA	NA	NA	NA	NA	18	0	5.3	1	2	4	1	0	0	0	2	1
NA	NA	NA	NA	NA	NA	1	1	2	1	2	1	2	5	1	1	1	1
						0	0	0	0	0	0	0	0	16	6	0	0
NA	NA	NA	NA	NA	NA	1	1	0.2	5	0	0	0	1	0	2	0	0
NA	NA	NA	NA	NA	NA	1	1	0.7	3	0	1	0	1	0	1	1	0
NA	NA	NA	Metabolisı	Biosynthes	NA	0	5	8.4	1	1	5	0	1	0	1	4	0
NA	NA	NA	NA	NA	NA	23	0	0.7	1	0	0	1	0	1	0	0	2
NA	NA	NA	NA	NA	NA	1	1	1.6	1	2	1	1	1	1	1	2	1
NA	NA	NA	NA	NA	NA	1	1	0.7	1	0	1	0	2	1	1	2	1
						0	0	0	0	0	0	0	0	0	0	0	0
						0	##	12	##	47	15	##	93	52	72	35	42

NA	NA	NA	NA	NA	NA	2	0	0.1	2	40	0	57	2	1	3	0	1
NA	NA	NA	NA	NA	NA	1	1	1.3	1	2	2	2	1	1	1	2	1
NA	NA	NA	NA	NA	NA	1	1	0.9	1	2	1	2	1	1	1	1	1
NA	NA	NA	NA	NA	NA	1	1	0.2	0	1	1	1	1	##	1	1	4
NA	NA	NA	NA	NA	NA	4	1	1.3	1	3	7	2	1	1	1	1	2
NA	NA	NA	NA	NA	NA	1	1	1.7	1	2	1	5	1	1	1	1	1
NA	NA	NA	NA	NA	NA	1	1	2.1	1	1	1	1	1	1	2	2	0
NA	NA	NA	NA	NA	NA	1	1	0.9	1	1	1	1	1	2	1	1	1
Hydroxyst	Steroids a	Steroids a	NA	NA	NA	1	1	0.9	1	1	1	0	1	1	1	1	1
						0	0	0	0	0	0	0	3	0	0	0	0
						0	0	0	0	0	0	0	0	11	0	0	0
NA	NA	NA	NA	NA	NA	0	1	0.2	1	0	0	0	0	23	1	0	0
NA	NA	NA	NA	NA	NA	1	0	0.5	2	0	0	0	0	1	2	0	0
Pyridoind	Indoles an	Indole an	NA	NA	NA	1	1	1.1	1	1	1	1	1	1	1	1	1
Amines	Organonit	Amines	NA	NA	NA	0	0	1	1	1	1	1	0	8	1	2	2
Phenylace	Benzene a	Benzene a	Metabolis	Ubiquinon	map00130	2	1	1	3	2	1	2	1	1	1	4	0
NA	NA	NA	NA	NA	NA	1	1	0.8	1	1	1	1	1	1	1	1	1
NA	NA	NA	NA	NA	NA	1	1	1.2	1	2	1	1	1	1	1	1	1
NA	NA	NA	NA	NA	NA	1	1	1	1	1	12	4	1	##	1	1	2
						0	0	0	0	0	0	0	12	26	0	0	0
NA	NA	NA	NA	NA	NA	1	4	1.2	1	1	1	1	1	1	1	22	1
NA	NA	NA	NA	NA	NA	1	1	0.9	1	2	1	2	1	1	1	1	1
NA	NA	NA	NA	NA	NA	1	1	2	2	1	1	1	1	1	1	1	2
NA	NA	NA	NA	NA	NA	1	1	0.6	4	0	1	0	2	1	1	1	1
NA	NA	NA	NA	NA	NA	1	1	2	1	1	20	0	1	1	1	9	1
NA	NA	NA	NA	NA	NA	0	2	4.5	1	2	11	2	1	1	1	9	0
Alcohols a	Organoox	Alcohols	Carbohyd	Galactose	map00052	1	0	0.4	2	9	0	11	2	1	2	0	1
NA	NA	NA	NA	NA	NA	1	1	1.3	1	1	1	2	1	1	1	1	1
NA	NA	NA	Xenobiotic	Metabolis	map00980	8	1	0.3	1	1	0	0	1	1	1	0	0
						0	0	0	0	##	0	8	0	0	0	0	0
Fatty acids	Fatty Acyls	Fatty acyls	Lipid meta	Fatty acid	map00061	1	1	1.6	1	1	9	2	1	1	1	1	0
Purines an	Imidazopy	Purines an	NA	NA	NA	1	1	0.6	1	1	1	2	2	1	2	0	1
NA	NA	NA	NA	NA	NA	4	0	0.9	0	0	1	18	##	2	6	0	20
Flavans	Flavonoids	Flavonoids	NA	NA	NA	32	1	1	1	1	1	1	1	1	1	1	3
NA	NA	NA	NA	NA	NA	1	1	1.8	1	2	1	2	1	1	1	2	2
NA	NA	NA	NA	NA	NA	1	1	0.9	2	10	1	9	0	1	1	1	1
NA	NA	NA	NA	NA	NA	1	0	0.7	0	7	2	0	1	2	2	1	0
NA	NA	NA	NA	NA	NA	0	0	0.1	2	0	0	0	1	20	1	1	16
NA	NA	NA	NA	NA	NA	1	2	0.3	1	1	1	2	2	0	0	0	2
NA	NA	NA	NA	NA	NA	1	1	0.6	1	1	1	1	1	1	1	1	2
NA	NA	NA	NA	NA	NA	1	1	0.9	1	3	1	1	1	1	2	2	46
NA	NA	NA	NA	NA	NA	1	1	5.7	0	4	0	9	0	2	2	1	3
NA	NA	NA	NA	NA	NA	1	1	1.8	1	2	1	2	3	1	1	1	0
						0	0	0	21	14	0	15	80	19	77	0	0
NA	NA	NA	NA	NA	NA	1	1	1.4	1	1	1	1	2	1	1	1	1
						0	0	0	0	21	0	0	0	0	0	0	0
NA	NA	NA	NA	NA	NA	1	1	1.3	1	1	1	1	1	1	1	1	1
NA	NA	NA	NA	NA	NA	1	0	0.8	4	1	1	0	1	0	2	1	0
NA	NA	NA	NA	NA	NA	1	1	1.4	1	2	1	1	1	1	1	1	1
NA	NA	NA	NA	NA	NA	1	1	1.7	1	2	1	2	1	1	1	1	1
NA	NA	NA	NA	NA	NA	1	1	0.3	1	0	0	0	3	0	2	0	0
NA	NA	NA	NA	NA	NA	1	1	1.7	1	2	1	5	2	1	1	1	1
NA	NA	NA	NA	NA	NA	1	1	0.1	2	0	1	0	1	0	1	1	0
						0	0	0	4	8	0	54	19	20	0	0	0
NA	NA	NA	NA	NA	NA	1	1	0.5	1	1	1	1	1	1	29	1	2
Fatty acids	Fatty Acyls	Fatty acyls	Lipid meta	Fatty acid	map00061	3	0	5.8	0	1	14	2	1	1	2	1	1
NA	NA	NA	NA	NA	NA	1	1	2.4	1	2	4	2	1	1	1	2	1
NA	NA	NA	NA	NA	NA	1	1	1	1	1	1	0	1	1	1	1	1

Others	Monolign	Phenylpro	NA	NA	NA	1	2	2.2	1	1	1	2	1	1	1	1	1
NA	NA	NA	NA	NA	NA	0	1	2.1	0	3	1	18	0	1	4	1	0
Benzoic ac	Benzene a	Benzene a	NA	NA	NA	1	1	0.6	1	0	0	1	2	0	1	1	1
Tryptamin	Indoles an	Amines an	Amino aci	Tryptopha	map0038C	1	2	0.9	1	1	2	2	3	0	1	1	1
						0	0	0	0	0	0	0	0	0	0	0	0
NA	NA	NA	NA	NA	NA	1	1	1	1	1	1	1	1	1	1	1	1
NA	NA	NA	NA	NA	NA	1	1	1.3	1	2	1	2	0	1	1	1	1
NA	NA	NA	NA	NA	NA	2	1	0.2	1	0	0	0	0	0	1	0	1
NA	NA	NA	NA	NA	NA	1	1	0.2	21	##	0	57	0	1	1	0	11
NA	NA	NA	NA	NA	NA	8	1	1	3	28	46	45	0	1	0	18	0
NA	NA	NA	NA	NA	NA	1	1	1.4	1	1	1	1	1	1	1	1	1
NA	NA	NA	NA	NA	NA	0	0	9.6	1	10	##	1	0	0	0	1	1
NA	NA	NA	NA	NA	NA	1	1	1.8	1	2	1	2	1	1	1	1	0
NA	NA	NA	Biosynthes	Indole dite	NA	1	1	0.2	1	2	0	1	0	27	1	0	2
Amino aci	Carboxylic	Amino aci	NA	NA	NA	1	1	0.9	2	2	1	1	1	1	1	2	2
NA	NA	NA	NA	NA	NA	1	1	0.8	1	1	1	0	1	1	1	1	7
NA	NA	NA	NA	NA	NA	1	1	1.2	1	1	1	1	1	1	1	1	1
NA	NA	NA	NA	NA	NA	1	0	0.4	1	0	1	1	5	0	1	2	8
NA	NA	NA	NA	NA	NA	2	1	0.5	1	0	1	0	1	0	2	1	5
						0	0	0	0	0	0	0	0	0	0	0	0
NA	NA	NA	NA	NA	NA	1	1	5.5	1	4	2	4	0	3	2	4	0
Diphenylm	Benzene a	Benzene a	NA	NA	NA	1	0	5.4	2	2	1	1	1	3	1	5	2
NA	NA	NA	NA	NA	NA	1	1	1.3	1	2	1	1	1	1	1	2	1
Amino aci	Carboxylic	Amino aci	NA	NA	NA	1	1	1.1	1	1	1	1	1	##	1	1	59
NA	NA	NA	NA	NA	NA	0	1	0.7	1	1	4	1	3	1	1	1	1
NA	NA	NA	NA	NA	NA	1	1	0.8	1	0	1	0	1	1	1	2	5
1,4-benzo	Benzodiaz	Organohe	NA	NA	NA	1	1	1	1	1	1	2	1	1	1	1	1
NA	NA	NA	NA	NA	NA	1	1	0.8	1	1	1	0	1	1	1	1	1
NA	NA	NA	NA	NA	NA	3	1	1.3	3	0	2	0	9	0	1	2	0
NA	NA	NA	NA	NA	NA	1	1	1.2	1	1	1	2	1	1	1	1	1
						0	0	0	4	0	0	0	24	27	35	0	45
						0	0	0	##	82	14	##	##	##	##	0	##
Benzoic ac	Benzene a	Benzene a	NA	NA	NA	0	12	15	0	10	2	2	0	0	1	1	0
NA	NA	NA	NA	NA	NA	1	1	0.9	1	1	1	0	1	1	1	1	1
NA	NA	NA	NA	NA	NA	1	1	0.8	1	1	1	1	1	1	1	1	2
Pyrimidine	Diazines	Organohe	NA	NA	NA	1	1	1	1	1	1	2	2	1	1	1	3
NA	NA	NA	NA	NA	NA	1	1	0.9	1	1	1	1	1	26	1	1	1
Halobenze	Benzene a	Benzene a	NA	NA	NA	1	1	0.6	3	5	1	11	1	0	1	3	1
Morpholin	Oxazinane	Organohe	NA	NA		1	1	2	1	2	2	2	1	1	1	1	0
						0	0	0	0	0	0	0	0	3	8	0	0
						0	0	0	0	4	0	0	0	0	42	0	0
NA	NA	NA	NA	NA	NA	1	3	1.6	1	7	5	23	1	1	1	2	0
NA	NA	NA	NA	NA	NA	2	0	2.1	1	2	3	3	1	1	0	1	3
Tricarboxy	Carboxylic	Organic ac	NA	NA	NA	1	1	0.6	3	1	0	2	3	0	1	0	4
NA	NA	NA	NA	NA	NA	1	1	1.1	1	1	1	0	1	1	1	1	1
						0	0	0	44	##	4	7	##	18	##	0	0
NA	NA	NA	NA	NA	NA	1	1	1	1	1	1	2	2	1	1	1	1
NA	NA	NA	NA	NA	NA	1	1	1.1	1	2	2	2	2	0	0	6	0
NA	NA	NA	Global anc	Biosynthes	NA	1	1	1.2	1	2	1	1	1	1	1	1	1
NA	NA	NA	NA	NA	NA	6	1	0.9	1	1	1	1	1	2	1	1	1
Fatty alcol	Fatty Acyls	Fatty acyls	NA	NA	NA	1	1	2.6	1	4	20	16	0	1	1	1	5
NA	NA	NA	NA	NA	NA	1	1	1.6	1	2	1	2	2	1	1	1	2
NA	NA	NA	NA	NA	NA	1	1	1.1	1	1	1	1	1	1	1	1	1
NA	NA	NA	NA	NA	NA	1	2	0.5	1	1	2	1	1	1	1	0	2
						0	0	0	0	0	0	0	0	0	0	0	0
NA	NA	NA	NA	NA	NA	1	1	0.9	1	1	1	1	1	1	1	1	1
NA	NA	NA	NA	NA	NA	##	1	1.5	1	1	1	1	1	1	1	1	1
NA	NA	NA	NA	NA	NA	1	1	2.2	2	4	0	3	1	2	4	4	2

Hybrid pe	Peptidomi	Organic ac	Amino aci	Histidine r	map00340	2	0	0.2	5	0	2	0	1	0	1	2	0
NA	NA	NA	NA	NA	NA	0	0	0.7	3	0	0	0	0	0	1	1	9
Terpene g	Prenol lipi	Terpenoid	NA	NA	NA	1	1	0.6	1	1	0	1	1	1	1	0	1
NA	NA	NA	NA	NA	NA	0	3	0.7	1	1	1	4	3	2	1	0	1
Beta hydro	Hydroxy a	Organic ac	Carbohydr	Citrate cyc	map00020	1	2	1.1	1	2	3	6	0	1	1	1	2
						0	0	0	0	0	0	0	0	5	0	0	0
						0	0	0	0	0	0	0	0	0	0	0	0
NA	NA	NA	NA	NA	NA	1	0	0.4	1	0	1	1	5	0	1	2	8
NA	NA	NA	NA	NA	NA	1	1	1.1	1	1	1	0	1	1	1	1	2
NA	NA	NA	NA	NA	NA	1	1	2.1	2	2	2	2	1	1	1	2	1
NA	NA	NA	NA	NA	NA	1	1	1.9	1	2	2	2	1	1	1	1	1
Arylsulfate	Organic su	Organic ac	NA	NA	NA	1	1	0.9	1	1	1	1	1	1	1	1	1
NA	NA	NA	NA	NA	NA	0	0	0.9	1	0	1	1	2	0	1	1	0
NA	NA	NA	NA	NA	map04614	1	1	1.5	1	2	2	1	1	3	1	1	1
NA	NA	NA	NA	NA	NA	2	0	3.7	3	2	0	1	0	1	3	3	37
NA	NA	NA	NA	NA	NA	1	1	1.3	1	1	1	2	1	1	1	1	16
NA	NA	NA	NA	NA	NA	2	5	0.6	4	8	1	14	0	0	1	1	1
NA	NA	NA	NA	NA	NA	1	1	1.5	2	2	1	2	1	2	1	1	9
NA	NA	NA	NA	NA	NA	1	1	0.1	1	2	0	0	0	1	1	0	1
NA	NA	NA	NA	NA	NA	1	1	1	1	1	1	0	1	1	1	1	1
NA	NA	NA	NA	NA	NA	0	1	0.4	3	3	1	13	1	0	3	0	1
NA	NA	NA	NA	NA	NA	1	1	1.8	1	2	1	2	1	1	1	1	26
NA	NA	NA	NA	NA	NA	2	1	0.6	3	0	1	0	5	0	5	1	0
NA	NA	NA	NA	NA	NA	2	0	0.3	3	1	1	0	0	0	3	0	0
NA	NA	NA	NA	NA	NA	1	1	1	1	9	3	9	1	1	1	1	1
NA	NA	NA	NA	NA	NA	6	1	1.5	1	1	1	2	1	1	1	1	1
NA	Benzene a	Benzene a	NA	NA	NA	2	1	0.6	1	0	1	0	1	1	3	1	6
NA	NA	NA	NA	NA	NA	1	1	1.4	1	2	1	1	1	1	1	2	1
NA	NA	NA	NA	NA	NA	1	1	1.1	1	2	1	2	1	1	1	2	1
NA	NA	NA	NA	NA	NA	1	1	1.9	1	2	1	2	1	1	1	2	1
NA	NA	NA	NA	NA	NA	1	1	0.9	1	1	1	1	1	2	1	1	1
NA	NA	NA	NA	NA	NA	1	1	2	1	2	2	1	1	1	0	1	1
NA	NA	NA	NA	NA	NA	1	1	1.1	1	1	1	0	1	1	1	1	1
Medium-c	Hydroxy a	Organic ac	NA	NA	NA	1	2	0.8	0	1	0	1	1	0	0	1	2
Pterins an	Pteridines	Pteridines	NA	NA	NA	1	1	0.7	2	1	1	1	1	1	7	1	1
NA	NA	NA	NA	NA	NA	2	1	0.6	1	1	1	0	1	0	1	14	1
NA	NA	NA	NA	NA	NA	2	1	0.6	0	2	1	2	1	2	1	1	1
NA	NA	NA	NA	NA	NA	1	1	0.7	1	1	1	0	1	0	1	1	1
NA	NA	NA	NA	NA	NA	81	1	1.2	0	0	4	0	1	1	0	0	3
NA	NA	NA	NA	NA	NA	1	1	0.9	1	2	2	1	1	1	2	1	2
NA	NA	NA	NA	NA	NA	1	1	0.6	1	0	1	1	1	1	1	1	1
NA	NA	NA	Amino aci	Arginine a	map00330	1	1	1.3	1	3	2	8	1	1	1	2	1
Benzoic ac	Benzene a	Benzene a	Amino aci	Tryptopha	map00380	1	1	1.5	2	1	1	4	1	1	2	1	1
NA	NA	NA	NA	NA	NA	27	0	1.5	8	3	2	0	2	1	1	3	1
NA	NA	NA	NA	NA	NA	0	1	0.7	3	1	##	1	1	1	1	2	4
NA	NA	NA	NA	NA	NA	1	1	1.7	0	1	1	2	2	1	1	1	0
						0	0	0	23	##	0	4	84	31	##	0	2
NA	NA	NA	NA	NA	NA	3	1	1.1	1	1	1	1	1	1	2	1	1
NA	NA	NA	NA	NA	NA	1	1	0.9	1	1	1	0	1	1	1	1	1
Indoles	Indoles an	Indoles	Amino aci	Tryptopha	map00380	1	0	0.5	1	0	0	3	0	25	1	1	1
NA	NA	NA	NA	NA	NA	1	1	0.8	1	1	1	1	1	1	1	1	1
NA	NA	NA	NA	NA	NA	1	1	1.1	1	1	1	0	1	1	1	1	1
NA	NA	NA	NA	NA	NA	1	1	2.5	1	2	2	3	0	1	1	2	0
						0	0	0	3	0	5	37	38	0	0	0	9
NA	NA	NA	NA	NA	NA	1	1	1	1	1	1	0	1	1	1	1	1
NA	NA	NA	NA	NA	NA	1	1	0.6	1	1	1	2	1	1	1	2	17
NA	NA	NA	NA	NA	NA	1	1	0.8	1	0	1	0	2	2	1	2	1
						0	0	0	##	##	15	##	##	##	##	0	75

NA	NA	NA	NA	NA	NA	1	1	1.2	1	1	1	0	1	1	1	2	1
NA	NA	NA	NA	NA	NA	1	1	0.7	5	0	1	0	1	0	2	0	0
NA	NA	NA	NA	NA	NA	1	1	0.8	1	1	1	0	1	1	1	1	1
NA	NA	NA	NA	NA	NA	1	1	1.6	1	1	4	2	1	1	1	1	7
Others	Sesquiterp	Terpenoid	NA	NA	NA	0	1	0	0	0	8	0	1	4	1	0	7
NA	NA	NA	NA	NA	NA	0	1	1.1	2	0	1	0	2	1	1	1	5
NA	Benzene a	Benzene a	NA	NA	NA	2	1	0.3	3	0	0	0	1	1	2	0	2
Amino aci	Carboxylic	Amino aci	NA	NA	NA	1	1	1.2	1	2	1	1	1	1	1	1	1
						0	0	0	0	2	0	0	0	0	0	0	0
Carbonyl c	Organoox	Organic o	Amino aci	Tryptopha	map0038C	1	2	0.6	4	2	1	7	5	1	0	2	2
NA	NA	NA	NA	NA	NA	1	3	0.5	1	1	1	1	2	3	2	0	1
						0	0	0	0	0	4	0	0	0	0	0	0
						0	0	0	0	0	0	0	0	0	0	0	0
NA	NA	NA	NA	NA	NA	1	1	1.1	1	1	1	1	1	1	1	1	1
NA	NA	NA	NA	NA	NA	3	1	1.2	1	1	2	1	2	1	1	2	1
NA	NA	NA	NA	NA	NA	1	1	1.1	1	2	1	1	0	1	1	1	1
NA	NA	NA	NA	NA	NA	1	1	1.7	1	2	1	2	1	2	1	1	1
NA	NA	NA	NA	NA	NA	1	1	1.7	1	2	1	2	1	1	1	2	2
NA	NA	NA	NA	NA	NA	2	0	0.5	0	1	0	0	0	8	1	1	1
Amino aci	Carboxylic	Amino aci	NA	NA	NA	1	1	0.3	1	1	1	1	1	1	0	0	1
NA	NA	NA	NA	NA	NA	1	1	0.5	1	1	1	1	1	1	33	1	3
NA	NA	NA	NA	NA	NA	1	1	0.6	3	1	0	1	2	1	2	19	0
NA	NA	NA	NA	NA	NA	1	1	1.4	0	2	1	2	1	1	1	1	1
						0	0	0	2	0	0	0	0	0	0	0	0
						0	0	0	0	0	0	0	0	0	0	0	0
NA	NA	NA	NA	NA	NA	1	0	0.3	2	3	0	2	0	0	1	0	3
NA	NA	NA	NA	NA	NA	1	2	0.7	1	1	1	1	1	5	1	1	0
NA	NA	NA	NA	NA	NA	1	1	0.7	2	1	0	0	1	1	2	1	0
Amino aci	Carboxylic	Amino aci	NA	NA	NA	1	1	0.8	1	1	1	1	1	1	1	1	1
						0	0	0	0	0	0	0	0	0	0	0	0
						0	0	0	0	0	0	0	0	0	0	0	0
						0	0	0	0	0	0	0	0	0	0	0	0
						0	0	0	0	0	0	0	0	0	0	0	0
						0	0	0	0	0	0	0	0	0	0	0	0
						0	0	0	0	0	0	0	0	0	0	0	0
						0	0	0	0	0	0	0	0	0	0	0	0
Flavonoid	Flavonoids	Flavonoids	NA	NA	NA	1	1	1.5	1	2	1	1	1	1	1	2	1
NA	NA	NA	NA	NA	NA	1	1	1.4	1	1	1	2	1	1	1	1	2
NA	NA	NA	NA	NA	NA	1	1	2	1	2	1	2	1	1	1	1	1
NA	NA	NA	NA	NA	NA	2	2	4.2	1	1	2	1	0	0	0	2	0
NA	NA	NA	Biosynthes	Staurospo	map0110(	2	1	1.3	1	2	1	2	1	0	1	1	0
Steroid est	Steroids a	Steroids a	NA	NA	NA	2	1	0.9	2	1	1	1	1	1	0	1	1
NA	NA	NA	NA	NA	NA	1	1	0.7	1	0	0	9	1	1	1	1	1
NA	NA	NA	NA	NA	NA	1	1	1	1	1	1	2	1	30	1	1	1
NA	NA	NA	NA	NA	NA	1	1	1.9	1	21	1	1	1	1	1	2	1
NA	NA	NA	NA	NA	NA	1	1	1	1	11	1	1	1	3	1	1	1
						0	0	0	0	##	0	0	0	0	4	0	0
NA	NA	NA	NA	NA	NA	1	1	1.1	1	1	1	1	1	1	1	2	1
Sulfated st	Steroids a	Steroids a	NA	NA	NA	1	1	1.5	1	2	1	2	1	1	1	1	1
NA	NA	NA	NA	NA	NA	1	1	0.2	1	0	1	0	1	1	2	0	1
Pyridoxam	Pyridines a	Amines an	Metabolis	Vitamin B6	map00750	2	0	1.9	1	1	3	2	1	1	1	1	3
NA	NA	NA	NA	NA	NA	1	1	0.8	3	1	1	1	1	1	0	1	1
NA	Tetracyclir	Polyketide	NA	NA	NA	1	0	0.4	1	1	0	0	1	3	1	0	0
NA	NA	NA	NA	NA	NA	1	1	1.2	1	1	1	2	1	1	1	1	1
NA	NA	NA	NA	NA	NA	1	1	1.2	1	2	1	2	1	1	1	1	1
NA	NA	NA	NA	NA	NA	0	1	0.6	1	1	1	1	1	0	0	0	0
NA	NA	NA	NA	NA	NA	1	1	0.3	2	1	0	2	0	##	1	1	1
NA	NA	NA	NA	NA	NA	15	0	0.5	6	6	69	28	1	0	1	9	2

NA	NA	NA	NA	NA	NA	1	1	0.7	1	1	1	1	1	1	1	1	1
NA	NA	NA	NA	NA	NA	1	1	1.4	1	1	1	2	1	1	1	1	4
						0	0	0	2	0	0	0	0	0	0	0	0
Fatty acids	Fatty Acyls	Fatty acyls	NA	NA	NA	1	1	2.6	1	3	2	2	0	3	1	2	0
Lineolic ac	Fatty Acyls	Fatty acyls	Lipid meta	alpha-Linc	map00592	4	1	2	1	4	69	9	1	1	0	4	0
NA	NA	NA	NA	NA	NA	1	1	1.1	1	1	1	1	1	1	1	2	1
NA	Morphinar	Alkaloids a	NA	NA	NA	2	3	0.7	3	0	22	0	0	0	0	1	3
NA	NA	NA	NA	NA	NA	1	1	1.2	1	1	1	1	1	1	1	1	3
NA	NA	NA	NA	NA	NA	1	7	2.3	0	0	4	0	0	0	4	0	0
NA	NA	NA	NA	NA	NA	1	0	0.6	3	1	0	1	2	0	1	1	0
NA	NA	NA	NA	NA	NA	1	1	0.9	1	7	1	1	2	1	2	1	1
						0	0	0	5	4	0	0	87	0	11	0	0
NA	NA	NA	NA	NA	NA	0	1	1.2	1	0	3	1	0	3	0	1	1
NA	NA	NA	NA	NA	NA	1	1	1.4	1	1	1	1	1	1	1	1	1
NA	NA	NA	NA	NA	NA	0	1	1.1	1	2	1	2	1	2	0	1	0
						0	7	3	19	46	0	25	##	19	86	0	26
NA	NA	NA	NA	NA	NA	1	1	0.6	1	1	0	1	2	16	1	0	1
Arylsulfate	Organic su	Organic ac	NA	NA	NA	1	1	0.1	1	0	7	0	3	0	0	2	3
NA	NA	NA	NA	NA	NA	0	1	0.5	0	1	1	0	11	15	3	0	8
NA	NA	NA	NA	NA	NA	1	1	0.2	4	0	0	0	1	1	1	0	3
NA	NA	NA	NA	NA	NA	1	1	0.9	2	1	1	1	1	1	1	1	1
NA	NA	NA	NA	NA	NA	1	1	0.8	1	0	1	0	1	0	2	2	0
Xylenes	Benzene a	Benzene a	Xenobiotic	Drug meta	map00982	0	1	1	3	54	1	28	1	2	4	4	14
NA	NA	NA	NA	NA	NA	1	1	1.1	1	1	1	1	1	1	1	1	88
NA	NA	NA	NA	NA	NA	1	1	0.8	1	0	1	0	1	6	1	2	1
NA	NA	NA	NA	NA	NA	1	1	2.8	1	2	1	2	1	1	1	2	1
Dammaren	Triterpeno	Terpenoid	NA	NA	NA	1	0	0.5	3	1	0	0	1	0	1	0	1
NA	NA	NA	NA	NA	NA	1	1	0.7	1	1	1	1	6	1	3	1	1
NA	NA	NA	NA	NA	NA	1	1	1.2	1	2	1	1	1	1	1	1	1
NA	NA	NA	NA	NA	NA	2	1	1.1	2	1	1	5	1	1	1	1	1
						0	0	0	0	0	0	0	0	0	0	0	0
						0	0	0	0	0	0	0	0	0	0	0	0
NA	NA	NA	NA	NA	NA	2	1	2	1	1	1	1	1	1	1	2	1
Xylenes	Benzene a	Benzene a	NA	NA	NA	1	1	0.2	2	0	2	0	0	0	1	2	0
NA	NA	NA	NA	NA	NA	1	1	1.6	1	1	1	2	1	1	1	2	1
Indolyl car	Indoles an	Indole anc	Amino aci	Tryptopha	map00380	2	1	1.8	2	6	1	1	1	0	1	1	1
NA	NA	NA	NA	NA	NA	1	1	1.2	1	1	1	1	0	1	1	1	1
NA	NA	NA	NA	NA	NA	1	1	1.8	1	2	1	2	1	1	1	2	0
NA	NA	NA	NA	NA	NA	2	1	0.6	8	1	4	1	9	0	0	1	1
Amino aci	Carboxylic	Amino aci	NA	NA	NA	1	1	2.3	1	2	3	1	2	1	1	1	1
NA	NA	NA	NA	NA	NA	1	1	1.1	1	1	1	1	11	1	1	2	1
NA	NA	NA	NA	NA	NA	1	2	1.4	1	1	1	1	1	2	1	1	1
NA	NA	NA	NA	NA	NA	2	1	2.5	1	2	7	2	0	1	1	2	0
NA	NA	NA	NA	NA	NA	1	1	0.8	1	1	1	1	0	2	1	1	1
						0	0	0	0	64	0	97	82	0	10	0	77
Amino aci	Carboxylic	Amino aci	NA	NA	NA	1	0	0.4	1	0	1	1	5	0	1	1	6
NA	NA	NA	NA	NA	NA	4	1	0.5	2	3	92	8	1	0	0	5	1
Carbohydr	Organoox	Carbohydr	NA	NA	NA	3	2	1	3	0	25	0	0	0	0	1	3
NA	NA	NA	NA	NA	NA	1	2	1.3	1	1	1	1	4	1	2	1	4
Isoquinolir	Alkaloids c	Alkaloids	Biosynthes	Isoquinolir	NA	1	1	1.2	1	2	1	1	1	1	1	1	1
Amines	Organonit	Amines	Amino aci	Arginine a	map00330	3	1	1.6	1	0	10	3	3	0	0	6	0
Quinoline	Quinolines	Organohe	Amino aci	Tryptopha	map00380	1	1	0.6	3	0	1	0	1	0	2	2	0
NA	NA	NA	NA	NA	NA	1	1	2	1	2	1	2	1	1	1	2	1
Benzoic ac	Benzene a	Benzene a	NA	NA	NA	0	4	1.7	2	1	2	1	0	0	1	4	0
NA	NA	NA	NA	NA	NA	1	1	1	1	1	1	1	1	1	1	1	1
NA	NA	NA	NA	NA	NA	3	1	0.5	1	0	0	0	1	1	2	0	6
NA	NA	NA	NA	NA	NA	0	1	0.3	1	3	1	0	2	0	2	1	24
NA	NA	NA	NA	NA	NA	1	1	1.1	2	1	1	0	1	0	2	1	0

NA	NA	NA	NA	NA	NA	1	1	0.7	1	1	2	1	2	2	1	1	7
NA	NA	NA	NA	NA	NA	1	0	0.4	2	1	0	0	1	5	1	1	1
NA	NA	NA	NA	NA	NA	4	1	0.3	1	0	0	1	3	0	1	0	5
NA	NA	NA	NA	NA	NA	1	1	1	1	1	1	1	1	1	1	1	1
Arylsulfate	Organic su	Organic ac	NA	NA	NA	26	0	0.6	0	0	0	1	3	0	2	0	4
NA	NA	NA	NA	NA	NA	1	1	1.5	1	1	1	2	1	1	1	1	1
NA	NA	NA	NA	NA	NA	1	1	2.3	1	2	1	2	1	1	1	2	1
NA	NA	NA	NA	NA	NA	1	1	1.7	1	2	1	2	1	1	1	1	2
NA	NA	NA	NA	NA	NA	1	1	0.6	1	1	1	1	1	2	1	1	1
NA	NA	NA	NA	NA	NA	1	1	1.3	1	7	1	1	0	2	1	1	1
NA	NA	NA	NA	NA	NA	1	1	0.7	1	0	1	0	1	9	1	1	0
NA	NA	NA	NA	NA	NA	1	1	0.7	1	0	1	0	1	1	1	1	0
NA	NA	NA	NA	NA	NA	1	1	1.2	1	2	1	2	0	1	0	1	2
NA	Cinnamyl a	Cinnamyl a	Chemical s	Biosynthes	NA	1	1	1.4	1	2	5	1	1	1	1	1	0
NA	NA	NA	NA	NA	NA	0	0	0.1	0	12	0	19	1	2	0	0	1
NA	NA	NA	NA	NA	NA	1	1	0.7	1	1	3	1	6	1	1	1	1
NA	NA	NA	NA	NA	NA	2	1	17	8	1	2	1	1	1	1	1	2
NA	NA	NA	NA	NA	NA	0	11	7.4	1	43	0	4	0	1	2	0	0
NA	NA	NA	NA	NA	NA	1	1	0.8	1	1	1	0	1	1	1	1	1
NA	NA	NA	NA	NA	NA	1	1	1.9	1	2	1	2	1	1	1	1	1
NA	NA	NA	NA	NA	NA	1	1	2.9	1	2	1	2	0	1	1	1	1
NA	NA	NA	NA	NA	NA	1	1	1	1	1	6	1	1	1	1	1	1
NA	NA	NA	NA	NA	NA	1	1	2	1	2	1	2	1	1	1	2	1
NA	NA	NA	NA	NA	NA	1	1	1.9	1	2	1	2	1	1	1	1	1
NA	NA	NA	NA	NA	NA	1	1	1.8	1	2	1	2	1	1	1	2	0
NA	NA	NA	NA	NA	NA	1	1	0.8	1	1	1	1	1	3	1	1	1
NA	NA	NA	NA	NA	NA	1	1	1.8	1	1	1	2	1	1	1	1	1
NA	NA	NA	NA	NA	NA	1	2	1.2	1	1	1	1	2	1	1	1	1
NA	NA	NA	NA	NA	NA	1	1	1.7	1	2	1	2	1	1	1	2	1
NA	NA	NA	NA	NA	NA	1	1	1.6	1	2	1	2	1	2	1	1	1
						0	0	0	0	0	0	0	0	0	0	0	0
NA	NA	NA	NA	NA	NA	1	1	1.7	1	2	1	2	1	1	1	2	0
Hydropyri	Pyridines a	Pyridine ar	NA	NA	NA	0	1	1.1	1	1	54	1	1	1	1	1	5
Pyrimidine	Diazines	Organohe	NA	NA	NA	1	1	0.7	1	1	1	2	1	1	1	1	1
NA	NA	NA	NA	NA	NA	1	1	0.7	2	1	2	1	1	1	1	2	1
						0	0	0	0	0	0	0	0	0	0	0	0
NA	NA	NA	NA	NA	NA	1	1	1.1	1	6	3	1	1	1	0	1	0
NA	NA	NA	NA	NA	NA	1	1	1.7	1	3	0	2	1	1	3	3	1
Purines an	Imidazopy	Purines an	Nucleotide	Purine me	map00230	1	1	0.9	2	0	1	1	1	1	1	1	3
NA	NA	NA	NA	NA	NA	1	1	1.2	1	1	37	1	1	2	1	1	2
NA	NA	NA	NA	NA	NA	3	1	1	1	3	0	1	0	1	1	1	68
NA	Yohimbine	Alkaloids a	NA	NA	NA	1	1	1.4	1	1	1	2	1	2	1	1	1
NA	NA	NA	NA	NA	NA	1	1	0.7	1	0	2	1	1	1	1	1	0
NA	NA	NA	NA	NA	NA	1	0	0.2	4	0	0	0	0	0	3	0	0
Alcohols a	Organoox	Alcohols	NA	NA	NA	1	1	2.8	1	3	1	3	0	1	1	2	0
NA	NA	NA	NA	NA	NA	1	1	1.1	1	2	1	2	0	1	0	1	2
NA	NA	NA	NA	NA	NA	1	1	1.1	1	1	1	2	1	2	1	1	1
NA	NA	NA	NA	NA	NA	1	3	0.5	1	1	1	1	1	1	1	0	2
Piperazine	Diazinanes	Organohe	NA	NA	NA	1	1	0.7	1	1	1	0	1	0	1	1	1
NA	NA	NA	NA	NA	NA	2	1	0.7	1	1	1	0	1	1	1	1	1
Pyrimidine	Diazines	Organohe	NA	NA	NA	25	1	1	1	1	1	1	1	1	1	1	1
NA	Phenol est	Benzene a	NA	NA	NA	1	1	0.4	1	1	2	1	1	2	1	2	1
NA	NA	NA	NA	NA	NA	2	1	0.9	2	1	2	1	2	1	3	1	7
Halobenze	Benzene a	Benzene a	NA	NA	NA	1	0	0.3	0	1	0	1	0	7	1	0	1
NA	NA	NA	Metabolism	Riboflavin	map00740	1	1	1.3	1	2	1	1	1	1	1	1	1
Amino aci	Carboxylic	Amino aci	NA	NA	NA	2	1	0.7	1	2	2	6	1	2	1	2	1
NA	NA	NA	NA	NA	NA	1	1	0.6	1	1	1	1	1	1	1	1	2
Phenothia	Benzothia	Organohe	NA	NA	NA	1	1	0.8	4	0	1	0	2	1	1	3	0

NA	NA	NA	NA	NA	NA	1	1	1.4	1	2	1	1	2	1	1	1	1
Benzenesu	Benzene a	Benzene a	NA	NA	NA	1	1	1.3	1	2	1	2	1	1	1	1	1
NA	NA	NA	NA	NA	NA	2	1	1	1	1	1	1	1	1	1	1	1
Amino aci	Carboxylic	Amino aci	NA	NA	NA	0	0	1.4	1	2	1	2	0	0	0	2	1
						0	0	0	0	0	0	63	0	0	0	0	0
Amino aci	Carboxylic	Amino aci	NA	NA	NA	10	0	0.1	1	2	1	1	1	0	0	0	14
NA	NA	NA	NA	NA	NA	1	2	1.3	1	1	1	1	1	2	1	1	2
NA	NA	NA	NA	NA	NA	1	1	1.4	3	1	1	0	0	0	2	0	0
NA	NA	NA	Metabolis	Biosynthes	map0110	1	1	1.9	1	2	1	2	1	1	1	2	1
NA	NA	NA	NA	NA	NA	1	1	1.9	1	2	1	2	1	1	1	1	1
						0	0	0	5	18	0	0	92	7	0	0	0
						0	0	0	27	##	0	##	89	27	38	0	0
NA	NA	NA	NA	NA	NA	1	0	0.1	2	1	0	0	0	1	2	0	1
						0	0	0	0	0	0	0	0	0	0	0	0
Carbohydr	Organoox	Carbohydr	NA	NA	NA	1	1	1.4	1	1	1	0	1	1	1	2	1
						0	0	0	0	0	0	0	0	0	0	0	0
NA	NA	NA	NA	NA	NA	1	1	0.7	1	1	1	1	0	3	1	1	1
NA	NA	NA	NA	NA	NA	2	1	1.1	1	1	1	1	1	1	1	1	1
NA	NA	NA	NA	NA	NA	1	2	0.9	2	1	1	0	1	0	2	0	0
						0	0	0	8	##	5	0	11	47	12	0	17
NA	NA	NA	NA	NA	NA	1	1	1.1	1	1	1	0	1	1	1	2	1
NA	NA	NA	NA	NA	NA	1	1	1.5	1	1	2	2	1	1	1	1	1
NA	NA	NA	NA	NA	NA	1	1	1	1	1	1	1	1	1	1	1	1
NA	NA	NA	NA	NA	NA	1	1	2.3	1	2	1	2	1	1	1	2	1
NA	NA	NA	NA	NA	NA	1	1	0.6	2	1	1	1	1	2	1	2	1
						0	24	0	0	0	0	0	92	17	19	0	##
NA	NA	NA	NA	NA	NA	1	1	0.3	2	0	0	0	1	1	2	2	5
NA	NA	NA	NA	NA	NA	1	3	2.3	1	2	9	2	2	1	2	2	0
NA	NA	NA	NA	NA	NA	1	1	1.8	1	2	1	2	1	1	1	2	1
NA	NA	NA	NA	NA	NA	1	1	2.7	3	3	1	12	1	1	1	1	0
NA	NA	NA	NA	NA	NA	1	1	1.2	2	1	1	0	1	0	2	1	0
NA	NA	NA	NA	NA	NA	1	0	2	1	3	1	2	1	1	1	1	0
NA	NA	NA	NA	NA	NA	1	1	1.5	1	1	1	2	1	1	1	1	1
NA	Purine nuc	Purines an	Nucleotide	Purine me	map0023	2	1	1	1	1	1	1	1	0	0	0	0
						0	0	0	0	0	0	0	0	0	8	0	0
Carbohydr	Organoox	Carbohydr	NA	NA	NA	1	2	0.8	3	1	1	0	0	0	1	2	1
NA	NA	NA	NA	NA	NA	1	1	3.4	1	3	1	3	1	1	2	3	0
Amino aci	Carboxylic	Amino aci	NA	NA	NA	1	1	1.9	1	2	1	2	1	1	1	2	1
NA	NA	NA	NA	NA	NA	1	1	1.2	2	1	1	1	1	1	1	0	2
NA	NA	NA	NA	NA	NA	1	1	1.2	1	1	1	1	1	1	1	1	1
NA	NA	NA	NA	NA	NA	1	1	1.8	1	2	1	2	1	1	1	2	0
NA	NA	NA	NA	NA	NA	1	1	0.4	3	0	1	0	1	0	2	1	0
NA	NA	NA	NA	NA	NA	1	0	1.1	0	2	1	1	1	0	1	0	1
NA	NA	NA	NA	NA	NA	0	0	40	0	0	0	0	1	0	2	0	0
NA	NA	NA	NA	NA	NA	1	1	1.3	1	2	1	1	1	1	1	1	1
NA	NA	NA	NA	NA	NA	2	1	1.4	1	2	1	2	1	1	1	2	1
NA	NA	NA	NA	NA	NA	0	1	2.1	1	0	11	1	2	1	7	1	4
Bile acids,	Steroids ar	Bile acids,	NA	NA	NA	1	1	0.8	1	1	1	0	2	2	1	1	4
NA	Phenol eth	Benzene a	NA	NA	NA	1	1	0.8	1	1	1	0	1	1	1	1	1
Phenylpyr	Benzene a	Benzene a	Metabolis	Ubiquinon	map0013	1	1	1.2	3	4	5	1	1	1	1	3	2
						0	0	0	0	0	0	0	0	0	0	0	0
						0	0	0	0	0	0	0	0	0	0	0	0
NA	NA	NA	NA	NA	NA	1	1	0.5	1	1	3	0	1	6	1	0	1
Glyceroph	Glyceroph	Glyceroph	NA	NA	NA	1	1	1.3	1	1	1	1	2	1	1	1	5
						0	0	0	0	0	0	0	0	0	0	0	0
NA	NA	NA	NA	NA	NA	2	1	1.8	1	1	1	2	1	1	1	1	0
Dibenzaze	Benzazepi	Organohe	NA	NA	NA	2	1	0.4	2	1	1	1	1	1	1	0	1
NA	NA	NA	NA	NA	NA	1	1	1.7	1	1	1	1	1	3	1	1	1

NA	NA	NA	NA	NA	NA	1	1	0.7	1	1	1	1	1	1	1	1	6
NA	NA	NA	NA	NA	NA	1	1	0.8	1	1	9	1	3	2	1	1	1
NA	NA	NA	NA	NA	NA	1	1	0.8	1	0	1	0	2	2	4	2	0
NA	NA	NA	NA	NA	NA	2	2	0.7	1	0	0	0	1	0	1	0	0
Purines an	Imidazopy	Purines an	NA	NA	NA	1	2	0.9	2	1	1	1	1	2	1	2	1
NA	NA	NA	NA	NA	NA	1	1	1	5	10	1	3	1	1	1	1	1
Glyceroph	Glyceroph	Glyceroph	NA	NA	NA	2	2	0.2	6	1	1	1	1	1	0	2	1
						0	0	0	4	6	0	0	0	4	87	0	3
NA	NA	NA	NA	NA	NA	1	1	1.6	1	4	1	2	2	1	1	1	1
NA	NA	NA	NA	NA	NA	2	0	0.1	1	0	0	0	0	0	1	0	1
NA	NA	NA	NA	NA	NA	2	1	0.6	1	1	1	1	0	3	1	1	1
NA	NA	NA	NA	NA	NA	1	1	2.7	1	3	1	3	1	2	2	1	1
Amino aci	Carboxylic	Amino aci	NA	NA	NA	1	1	1.2	1	2	3	4	1	1	1	1	1
Lineolic ac	Fatty Acyls	Fatty acyls	NA	NA	NA	2	1	1.2	0	1	2	1	1	1	1	1	1
NA	NA	NA	NA	NA	NA	52	2	1.3	3	0	0	0	0	1	0	1	2
NA	NA	NA	NA	NA	NA	1	1	1.6	0	1	1	1	1	1	2	1	1
NA	NA	NA	NA	NA	NA	1	0	3	0	1	##	1	2	0	0	0	3
NA	NA	NA	NA	NA	NA	0	1	1.6	1	1	1	1	14	1	4	1	0
NA	NA	NA	NA	NA	NA	1	1	0.9	1	0	0	1	1	1	1	1	1
NA	NA	NA	NA	NA	NA	1	1	0.7	1	1	1	1	1	1	1	2	1
NA	NA	NA	NA	NA	NA	1	1	1.7	1	2	1	2	1	1	1	1	1
NA	NA	NA	NA	NA	NA	1	1	0.8	1	1	1	0	1	0	1	2	1
NA	NA	NA	NA	NA	NA	1	1	1.1	1	23	1	1	1	1	1	1	1
NA	NA	NA	NA	NA	NA	1	1	1.3	1	2	1	1	1	1	1	1	1
NA	NA	NA	NA	NA	NA	1	1	1.2	1	1	1	1	1	1	2	1	1
NA	NA	NA	NA	NA	NA	1	2	0.9	2	1	1	2	1	1	1	3	0
NA	NA	NA	NA	NA	NA	1	1	1.7	1	2	1	2	1	1	1	1	1
NA	NA	NA	NA	NA	NA	1	1	0.8	2	1	0	0	1	1	1	1	0
NA	NA	NA	NA	NA	NA	1	1	1	1	1	1	2	1	1	1	1	1
NA	NA	NA	NA	NA	NA	1	1	1.2	1	1	1	1	1	20	1	1	1
NA	NA	NA	NA	NA	NA	1	1	0.8	1	1	1	1	1	1	1	1	5
Dibenzoxe	Benzoxepi	Organohe	NA	NA	NA	1	1	1	2	0	1	0	1	0	2	1	0
NA	NA	NA	NA	NA	NA	1	1	4.5	1	1	0	1	2	1	5	0	0
NA	NA	NA	NA	NA	NA	1	1	1.2	1	1	1	1	1	1	1	1	1
NA	NA	NA	NA	NA	NA	1	1	1	2	0	1	0	1	0	1	1	0
NA	NA	NA	NA	NA	NA	1	1	2.1	1	2	1	2	1	1	1	2	1
NA	NA	NA	NA	NA	NA	1	1	2.1	1	1	1	2	1	1	1	1	1
NA	NA	NA	NA	NA	NA	1	1	0.6	3	1	1	1	2	1	1	1	1
NA	NA	NA	NA	NA	NA	2	1	1.6	2	2	0	3	2	0	1	1	0
NA	NA	NA	NA	NA	NA	1	1	1.4	1	1	0	1	1	0	1	1	1
NA	NA	NA	NA	NA	NA	1	1	0.4	1	0	2	0	2	0	0	1	1
NA	NA	NA	NA	NA	NA	1	1	0.7	1	1	1	1	1	2	2	1	1
NA	NA	NA	NA	NA	NA	2	1	0.6	1	1	1	1	1	0	1	2	1
NA	NA	NA	NA	NA	NA	1	1	0.9	1	1	1	2	0	2	0	0	1
NA	NA	NA	NA	NA	NA	1	1	1.1	0	3	6	6	1	2	1	2	0
Phenylpro	Benzene a	Benzene a	NA	NA	NA	2	1	0.6	1	1	1	1	0	3	1	1	1
NA	NA	NA	NA	NA	NA	5	0	0	##	30	6	1	0	2	0	4	0
NA	NA	NA	NA	NA	NA	1	1	0.8	1	0	1	0	1	1	1	2	1
Tetrodoto:	Others	Marine bic	NA	NA	NA	1	1	0.9	1	1	1	2	1	1	1	1	15
NA	NA	NA	NA	NA	NA	1	1	0.7	1	1	1	1	1	2	1	1	1
Carbohydr	Organoox	Carbohydr	NA	NA	NA	1	1	1.1	1	1	1	1	0	1	1	1	1
NA	NA	NA	NA	NA	NA	1	1	0.7	2	1	1	1	1	2	1	2	1
Tryptamin	Indoles an	Amines an	NA	NA	NA	8	1	0.8	1	1	1	1	0	1	1	0	3
NA	NA	NA	NA	NA	NA	9	1	0.8	1	1	1	1	1	2	1	1	1
NA	NA	NA	NA	NA	NA	0	0	0.1	1	0	0	0	1	14	2	0	0
						0	0	0	0	0	0	##	16	0	0	0	12
NA	NA	NA	NA	NA	NA	0	1	1.2	1	2	1	9	1	1	1	0	1
NA	NA	NA	NA	NA	NA	1	4	7.6	1	2	1	1	0	1	1	1	0

NA	NA	NA	NA	NA	NA	1	1	1.7	1	2	1	2	1	1	1	1	1
NA	NA	NA	NA	NA	NA	0	0	1.8	4	0	1	0	1	0	6	6	1
NA	NA	NA	NA	NA	NA	1	1	2	1	2	1	2	1	1	1	1	1
NA	NA	NA	Biosynthes	Biosynthes	NA	11	0	3.2	1	1	1	0	0	0	5	17	22
NA	NA	NA	NA	NA	NA	2	1	1.8	1	1	9	2	0	1	1	1	2
NA	NA	NA	NA	NA	NA	1	1	0.8	1	1	8	1	0	1	1	1	1
NA	NA	NA	NA	NA	NA	1	1	0.7	1	0	1	1	1	7	1	2	1
Quaternar	Organonit	Organic ni	Lipid meta	Glyceroph	map00564	1	5	1	1	1	0	1	1	1	1	2	0
NA	NA	NA	NA	NA	NA	1	1	0.9	1	1	1	1	1	1	1	1	1
NA	NA	NA	Metabolisı	Terpenoid	map0090C	1	1	2.4	1	2	2	2	2	1	1	2	1
NA	NA	NA	NA	NA	NA	1	1	1.6	1	2	1	2	1	2	1	1	3
Pyrimidine	Diazines	Organohe	NA	NA	NA	1	0	0.4	2	0	0	0	2	2	1	1	4
3'-thia pyı	Nucleosid	Nucleic ac	NA	NA	NA	1	1	1.8	1	2	1	2	1	1	1	1	1
						0	0	0	7	##	0	17	23	2	78	0	0
NA	NA	NA	NA	NA	NA	1	1	1.7	1	2	1	2	1	1	1	2	0
NA	NA	NA	NA	NA	NA	1	1	1.3	2	1	4	5	1	0	1	1	1
NA	NA	NA	NA	NA	NA	1	1	1	1	1	1	1	1	1	1	1	1
NA	NA	NA	NA	NA	NA	1	1	1.1	1	1	1	1	1	2	1	1	1
Anilides	Benzene a	Benzene a	NA	NA	NA	1	1	0.9	2	1	1	0	1	0	1	1	0
NA	NA	NA	NA	NA	NA	2	1	0.7	1	1	1	0	1	3	1	1	1
NA	NA	NA	NA	NA	NA	1	2	0.9	1	1	1	1	2	3	2	1	2
NA	NA	NA	NA	NA	NA	2	2	1.1	1	11	1	1	1	1	1	4	1
NA	NA	NA	NA	NA	NA	1	1	0.8	1	1	1	1	2	1	1	2	1
NA	NA	NA	NA	NA	NA	1	1	0.8	1	0	1	0	1	1	1	2	1
Phosphos	Sphingolip	Sphingolip	NA	NA	NA	1	1	1.9	1	5	1	2	1	1	1	1	1
Benzoic ac	Benzene a	Benzene a	NA	NA	NA	2	1	0.9	1	1	1	1	1	0	1	1	1
NA	NA	NA	NA	NA	NA	1	1	0.8	2	1	1	1	1	1	2	1	0
NA	NA	NA	Xenobiotic	Polycyclic	NA	1	1	1	1	1	1	2	1	1	1	1	1
Benzoic ac	Benzene a	Benzene a	NA	NA	NA	1	3	0.7	2	1	1	1	2	0	1	1	1
NA	NA	NA	NA	NA	NA	0	0	0.2	3	0	0	0	1	0	1	0	0
						0	0	0	4	##	0	96	##	83	##	0	70
Fatty acid	Fatty Acyls	Fatty acyls	NA	NA	NA	7	3	0	0	1	0	0	0	1	3	0	0
NA	NA	NA	NA	NA	NA	2	2	0.9	1	4	1	5	1	1	1	2	1
NA	NA	NA	NA	NA	NA	1	1	1	1	1	1	2	1	1	1	1	1
Indolyl car	Indoles an	Indole anc	Amino aci	Tryptopha	map0038C	1	0	0.9	2	0	1	0	0	1	1	1	0
NA	NA	NA	NA	NA	NA	2	1	0.6	1	1	1	0	1	1	1	1	1
NA	NA	NA	NA	NA	NA	1	1	1.7	1	1	1	1	1	1	1	1	1
						0	0	0	0	0	0	0	0	0	0	0	0
NA	NA	NA	NA	NA	NA	1	1	1.6	1	1	1	1	1	1	2	1	1
NA	NA	NA	NA	NA	NA	1	1	0.8	0	2	1	1	1	29	1	1	2
NA	NA	NA	NA	NA	NA	1	2	0.6	1	1	1	1	1	1	1	1	6
NA	NA	NA	NA	NA	NA	0	1	0.4	3	3	1	12	1	0	1	0	1
NA	NA	NA	NA	NA	NA	1	1	0.7	1	0	1	0	1	5	1	1	0
NA	NA	NA	NA	NA	NA	2	1	0.6	3	2	0	0	1	1	2	0	0
NA	NA	NA	NA	NA	NA	2	0	0.3	3	2	0	0	1	5	3	0	1
						0	0	0	0	60	0	0	32	0	36	0	0
NA	NA	NA	NA	NA	NA	1	1	2.4	1	2	1	2	1	1	1	2	0
Carbonyl c	Organoox	Organic o	Amino aci	Tryptopha	map0038C	3	0	0.1	12	4	1	0	1	3	1	5	1
Tryptamin	Indoles an	Amines an	NA	NA	NA	1	1	1.2	1	3	2	5	0	1	1	1	2
NA	NA	NA	NA	NA	NA	1	1	1.2	1	1	1	1	1	1	1	1	1
NA	NA	NA	NA	NA	NA	0	0	5.9	0	1	33	1	0	0	0	0	6
NA	NA	NA	Metabolisı	Terpenoid	map0090C	1	1	0.6	1	1	1	1	1	25	5	1	2
NA	NA	NA	NA	NA	NA	1	1	0.9	1	1	1	0	1	1	1	1	1
Carbohydı	Organoox	Carbohydı	NA	NA	NA	2	0	0.3	3	0	0	0	1	1	5	0	0
NA	NA	NA	NA	NA	map0201C	1	1	0.9	1	1	1	0	1	1	1	1	1
NA	NA	NA	NA	NA	NA	1	1	1	1	2	1	1	1	1	1	1	1
						0	0	0	31	16	3	##	##	##	35	2	##
NA	NA	NA	NA	NA	NA	1	4	0.6	1	20	0	1	3	2	0	0	0

NA	NA	NA	NA	NA	NA	1	1	0.8	1	0	1	1	1	1	1	1	1
Amino aci	Carboxylic	Amino aci	Amino aci	Phenylalar	map00360	0	1	0.9	1	0	0	1	1	8	2	1	1
NA	NA	NA	NA	NA	NA	1	1	1.3	1	2	1	1	1	1	1	1	1
Monoterp	Prenol lipi	Terpenoid	NA	NA	NA	1	1	1.6	1	1	1	2	1	1	1	1	0
NA	NA	NA	NA	NA	NA	0	0	1.1	1	0	2	0	1	1	5	8	3
NA	NA	NA	NA	NA	NA	1	1	2	1	2	1	2	1	1	1	2	1
NA	NA	NA	NA	NA	NA	1	1	1.7	1	2	79	2	1	1	1	1	1
NA	NA	NA	NA	NA	NA	1	1	2.4	1	2	1	2	1	1	1	2	0
NA	NA	NA	Biosynthes	Penicillin a	map0110(	1	1	1	2	1	1	0	1	1	3	0	1
Sulfanilide	Benzene a	Benzene a	NA	NA	NA	1	1	1.1	1	1	1	1	1	2	1	1	2
						0	0	0	0	0	0	0	0	0	0	0	0
						0	0	0	0	0	0	0	0	0	0	0	0
						0	0	0	0	0	0	0	0	0	0	0	0
						0	0	0	0	0	0	0	0	0	0	0	0
						0	0	0	0	0	0	0	0	0	0	0	0
						0	0	0	0	0	0	0	0	0	0	0	0
						0	0	0	0	0	0	0	0	0	0	0	0
						0	0	0	0	0	0	0	0	0	0	0	0
						0	0	0	0	0	0	0	0	0	0	0	0
Fatty acids	Fatty Acyls	Fatty acyls	Lipid meta	Biosynthes	map0104(	1	1	0.5	0	2	15	5	2	3	0	1	11
NA	NA	NA	NA	NA	NA	1	1	0.7	1	1	1	0	1	0	1	1	1
NA	NA	NA	NA	NA	NA	1	1	0.9	1	1	1	0	1	1	1	1	0
NA	NA	NA	NA	NA	NA	1	1	1.4	1	1	1	1	1	1	1	1	1
NA	NA	NA	NA	NA	NA	0	1	1.1	1	2	1	2	0	0	1	1	2
NA	NA	NA	NA	NA	NA	1	1	0.8	1	2	1	2	1	1	1	1	5
Amines	Organonit	Amines	NA	NA	NA	1	1	2.1	1	2	1	2	1	2	1	1	1
NA	NA	NA	NA	NA	NA	1	0	0.3	3	0	0	0	1	0	1	0	0
NA	NA	NA	NA	NA	NA	1	2	2.7	1	3	2	5	1	1	2	5	0
NA	NA	NA	NA	NA	NA	2	3	0.5	1	2	33	4	0	1	0	3	1
						0	0	0	0	0	0	0	0	0	0	0	0
NA	NA	NA	NA	NA	NA	0	3	1.1	1	1	1	1	1	3	1	2	2
NA	NA	NA	NA	NA	NA	1	1	0.2	4	1	0	0	1	0	1	0	1
Carbohydr	Organoox	Carbohydr	NA	NA	NA	1	1	1.9	1	2	1	2	1	1	1	2	1
NA	NA	NA	NA	NA	NA	1	1	1	1	2	1	2	1	1	1	1	0
NA	NA	NA	NA	NA	NA	1	1	1.1	2	0	1	0	1	13	0	0	1
NA	NA	NA	NA	NA	NA	1	1	1.9	1	2	3	2	1	1	1	1	9
NA	NA	NA	NA	NA	NA	13	1	1.8	0	1	6	0	1	0	1	1	1
NA	NA	NA	NA	NA	NA	2	1	1.1	4	0	1	0	1	0	3	1	2
NA	NA	NA	NA	NA	NA	0	2	3.1	0	6	3	4	0	4	2	1	0
Cardanolic	Steroids	Terpenoid	NA	NA	NA	0	1	0.4	1	2	0	0	1	2	1	0	3
NA	NA	NA	NA	NA	NA	1	1	0.8	2	2	1	1	1	1	1	2	1
NA	NA	NA	NA	NA	NA	1	1	0.9	2	1	1	0	1	1	1	1	1
NA	NA	NA	NA	NA	NA	2	1	0.6	2	1	1	1	1	1	0	6	2
NA	NA	NA	NA	NA	NA	1	1	0.6	1	1	8	0	1	2	1	1	1
Lineolic ac	Fatty Acyls	Fatty acyls	Lipid meta	Linoleic ac	map00591	3	2	1.7	2	10	10	3	0	1	0	3	1
Carbonyl (	Organoox	Organic o:	NA	NA	NA	1	1	0.9	1	1	1	0	1	1	1	1	1
NA	NA	NA	NA	NA	NA	1	2	1.8	2	1	1	2	1	1	1	2	0
Methoxyp	Phenols	Phenols ar	NA	NA	NA	1	1	0.6	1	1	1	1	1	0	1	2	1
Phosphos	Sphingolip	Sphingolip	NA	NA	NA	0	3	0.7	2	13	0	13	1	4	0	1	2
NA	NA	NA	NA	NA	NA	1	2	0.6	1	1	2	1	1	3	1	1	1
NA	NA	NA	NA	NA	NA	1	1	0.6	1	1	1	1	2	1	1	2	1
NA	NA	NA	NA	NA	NA	0	2	0.6	1	0	1	1	1	2	1	0	1
						0	0	0	0	7	0	0	0	0	0	0	0
NA	NA	NA	NA	NA	NA	0	7	0.3	0	5	0	1	2	2	1	0	0
NA	NA	NA	NA	NA	NA	0	0	2	1	2	0	2	1	1	0	2	1
NA	NA	NA	NA	NA	NA	1	1	1	1	1	1	2	1	1	1	1	1
NA	NA	NA	NA	NA	NA	2	1	1.2	1	1	13	1	2	1	1	1	1
NA	NA	NA	NA	NA	NA	1	1	1.3	1	1	1	1	1	1	2	1	1

NA	NA	NA	NA	NA	NA	1	1	0.8	2	1	1	0	4	1	1	1	0
NA	NA	NA	NA	NA	NA	1	1	1.2	2	1	1	1	1	1	1	1	4
NA	NA	NA	NA	NA	NA	0	1	0.8	1	5	1	24	6	14	2	1	1
NA	NA	NA	NA	NA	NA	1	1	0.6	1	1	1	1	1	1	1	1	2
Carboxylic	Carboxylic	Organic ac	NA	NA	NA	1	1	1.2	1	7	0	5	4	8	1	1	1
NA	NA	NA	NA	NA	NA	1	1	0.6	1	1	1	1	1	1	1	1	2
						0	0	0	6	0	0	81	0	24	10	0	0
						0	0	0	0	10	0	0	94	19	0	0	7
NA	NA	NA	NA	NA	NA	2	0	0.1	0	5	8	0	20	0	4	0	0
NA	NA	NA	NA	NA	NA	0	1	0.3	2	1	1	0	1	13	1	1	1
NA	NA	NA	NA	NA	NA	0	1	0.2	1	0	0	0	1	1	1	1	4
NA	NA	NA	NA	NA	NA	1	1	1.1	1	2	1	2	0	1	0	1	1
NA	NA	NA	NA	NA	NA	0	3	0.6	1	3	1	2	1	7	1	1	1
NA	NA	NA	NA	NA	NA	0	1	0.1	2	0	0	0	1	0	3	2	5
NA	NA	NA	NA	NA	NA	0	1	0.8	1	1	1	3	53	5	7	1	25
NA	NA	NA	NA	NA	NA	1	1	0.8	1	1	1	1	1	1	2	1	1
NA	NA	NA	NA	NA	NA	1	1	1.7	1	2	1	2	1	1	1	2	0
NA	NA	NA	NA	NA	NA	6	0	0.3	2	8	38	9	1	1	0	9	2
Short-chai	Keto acids	Organic ac	Amino aci	Alanine, as	map0025C	1	3	0.5	1	5	1	30	1	1	0	1	0
Amino aci	Carboxylic	Amino aci	Amino aci	Arginine a	map0033C	1	1	1.3	1	1	1	2	1	1	1	1	1
Carbohydr	Organoox	Carbohydr	NA	NA	NA	1	1	1	1	1	1	1	0	1	1	1	1
NA	NA	NA	NA	NA	NA	2	1	0.8	1	1	1	1	0	##	1	1	2
NA	NA	NA	NA	NA	NA	1	1	0.8	2	1	2	1	2	1	1	2	1
Indolyl car	Indoles an	Indole anc	Amino aci	Tryptopha	map0038C	0	1	0.4	2	0	1	0	1	0	1	1	0
NA	NA	NA	NA	NA	NA	1	1	1.4	1	2	1	2	1	2	1	1	1
NA	NA	NA	NA	NA	NA	1	2	1.7	1	2	1	2	1	2	1	1	1
NA	NA	NA	NA	NA	NA	1	1	0.8	2	1	2	1	2	1	1	2	1
NA	NA	NA	Biosynthes	Streptomy	map0110(	1	1	1.2	1	2	1	1	1	1	1	1	1
NA	NA	NA	NA	NA	NA	1	1	1.9	1	2	1	2	1	1	1	2	1
NA	NA	NA	NA	NA	NA	1	1	0.9	1	1	1	0	1	1	1	1	1
NA	NA	NA	NA	NA	NA	1	1	1.5	1	2	1	1	1	1	1	2	1
Amino aci	Carboxylic	Amino aci	Amino aci	Alanine, as	map0025C	1	1	1.7	1	1	1	2	1	1	1	1	1
NA	NA	NA	NA	NA	NA	1	1	1.1	1	1	9	1	1	1	1	1	1
NA	NA	NA	NA	NA	NA	1	1	7.2	1	1	1	1	1	1	1	1	1
NA	NA	NA	NA	NA	NA	1	1	0.9	1	1	1	2	1	1	1	1	1
NA	NA	NA	NA	NA	NA	0	2	0.5	3	1	0	1	0	0	1	0	1
Amino aci	Carboxylic	Amino aci	NA	NA	NA	1	1	0.9	2	0	1	0	1	0	2	1	0
NA	NA	NA	NA	NA	NA	1	1	1.1	1	2	1	1	1	1	1	1	1
Diterpenoi	Prenol lipi	Terpenoid	NA	NA	NA	1	0	0.8	0	1	14	1	6	1	2	1	2
NA	NA	NA	NA	NA	NA	1	1	0.9	2	1	1	1	1	2	1	1	1
NA	NA	NA	NA	NA	NA	1	2	1.1	1	1	1	6	1	1	0	0	2
NA	NA	NA	NA	NA	NA	1	1	0.6	4	0	0	0	1	0	1	0	1
NA	NA	NA	NA	NA	NA	1	1	1.3	1	1	1	1	1	1	1	1	1
NA	NA	NA	Metabolis	Porphyrin	map0086C	1	1	1.1	1	1	1	0	1	1	1	1	1
NA	NA	NA	NA	NA	NA	1	1	0.5	3	0	0	0	0	0	4	1	0
NA	NA	NA	NA	NA	NA	13	1	1.8	0	1	6	0	1	0	1	1	1
NA	NA	NA	NA	NA	NA	##	1	1.3	0	0	1	0	1	0	14	1	1
NA	NA	NA	NA	NA	NA	0	0	0.2	1	0	0	0	8	4	4	0	0
Pyrimidine	Diazines	Organohe	NA	NA	NA	3	1	0.5	2	0	0	0	0	0	1	1	0
Oxirane ca	Epoxides	Organohe	NA	NA	NA	1	0	0.8	3	0	1	1	1	0	2	1	1
NA	NA	NA	NA	NA	NA	1	1	0.9	1	1	1	0	1	1	1	1	1
NA	NA	NA	NA	NA	NA	1	1	0.9	1	1	1	0	1	1	1	1	1
NA	NA	NA	NA	NA	NA	1	1	1.7	1	2	1	2	1	1	1	1	0
NA	NA	NA	NA	NA	NA	1	1	2	1	2	1	2	1	1	1	2	1
NA	NA	NA	NA	NA	NA	1	1	1.8	1	1	2	2	1	2	1	2	1
Amino aci	Carboxylic	Amino aci	NA	NA	NA	1	1	1.6	1	1	1	5	1	1	1	1	0
NA	Tetralins	Benzene a	NA	NA	NA	6	0	0.1	5	3	3	0	0	7	0	9	3
Carbohydr	Organoox	Carbohydr	NA	NA	NA	1	1	0.8	3	0	0	0	0	0	2	0	0

Null	PK04 Mac	Polyketide	Metabolis	Biosynthes	map0110	1	3	0.7	1	0	2	1	1	1	0	0	3
Indoles	Indoles an	Indoles	Amino aci	Tryptopha	map00380	0	1	0.9	1	0	3	0	0	0	4	16	0
NA	NA	NA	NA	NA	NA	0	4	0.5	0	1	1	1	6	1	4	0	4
NA	NA	NA	NA	NA	NA	1	1	1.1	1	1	1	2	1	1	1	1	4
NA	NA	NA	NA	NA	NA	1	0	1	2	3	0	0	2	3	2	1	14
NA	NA	NA	NA	NA	NA	0	1	0.7	0	2	34	2	0	1	0	1	1
NA	NA	NA	NA	NA	NA	1	1	1.9	1	2	1	2	1	1	1	2	1
Pyridoxine	Pyridines a	Pyridine ar	Metabolis	Vitamin B6	map00750	1	1	1.3	2	1	4	2	13	1	2	1	0
						0	0	0	0	7	0	0	0	0	37	0	0
						0	0	0	9	19	0	0	47	45	0	0	18
NA	NA	NA	NA	NA	NA	1	1	1.5	1	1	1	1	1	1	1	2	1
Phenylhyd	Benzene a	Benzene a	NA	NA	NA	0	0	0.9	2	0	1	1	1	0	4	2	1
Beta lactar	Lactams	Organohe	NA	NA	NA	1	1	1.2	1	1	1	1	1	0	0	0	1
NA	Indanes	Benzene a	NA	NA	NA	1	1	1.5	3	1	2	2	5	1	1	1	1
						0	0	0	42	##	7	56	##	71	##	0	0
NA	NA	NA	NA	NA	NA	1	0	0.7	1	0	1	1	1	0	1	2	2
NA	NA	NA	NA	NA	NA	1	1	0.4	1	1	1	1	1	0	1	0	2
NA	NA	NA	NA	NA	NA	1	1	1	2	1	1	0	1	1	1	1	1
NA	NA	NA	NA	NA	NA	1	0	0.4	1	1	0	1	1	3	0	0	1
Estrane ste	Steroids ar	Steroids ar	NA	NA	NA	10	9	1	0	0	86	0	0	1	0	1	29
						0	0	0	5	26	0	0	0	0	0	0	0
NA	NA	NA	NA	NA	NA	1	1	1	1	1	5	1	0	6	1	1	9
Lineolic ac	Fatty Acyls	Fatty acyls	Lipid meta	Linoleic ac	map00591	6	0	0.8	2	6	##	12	0	1	0	5	0
Cyanogen	Cyanogen	Amino aci	NA	NA	NA	2	1	1.3	1	2	1	2	4	1	1	1	1
NA	NA	NA	NA	NA	NA	1	2	0.2	2	1	0	0	0	1	1	0	0
						0	0	0	0	0	0	0	0	0	0	0	16
NA	NA	NA	NA	NA	NA	1	1	1.3	1	2	1	2	0	1	1	1	1
NA	NA	NA	Metabolis	Polyketide	NA	1	1	1.2	1	1	1	2	1	1	2	1	1
Biphenyls	Benzene a	Benzene a	NA	NA	NA	1	1	8.1	1	5	1	5	1	1	1	3	1
NA	NA	NA	NA	NA	NA	1	1	0.7	1	0	1	1	1	1	1	1	1
						0	0	0	0	0	0	72	46	13	21	0	0
NA	NA	NA	NA	NA	NA	1	1	1.2	1	1	1	1	1	1	1	1	1
NA	NA	NA	NA	NA	NA	1	1	1.7	1	1	2	2	1	1	1	2	1
NA	NA	NA	NA	NA	NA	5	1	0.9	1	0	1	1	1	1	1	1	1
NA	NA	NA	NA	NA	NA	1	1	1.1	1	1	1	2	1	1	1	1	1
NA	NA	NA	NA	NA	NA	1	1	0.5	2	1	2	2	1	1	0	3	14
NA	NA	NA	NA	NA	NA	5	1	0.9	1	1	1	4	29	1	1	0	3
NA	NA	NA	NA	NA	NA	0	0	2	0	1	1	1	0	0	3	1	1
NA	NA	NA	NA	NA	NA	1	1	0.4	2	1	0	1	1	0	3	0	0
NA	NA	NA	NA	NA	NA	0	0	0	1	0	0	0	7	2	2	0	9
NA	NA	NA	NA	NA	NA	2	1	1.7	1	1	1	2	1	1	1	1	1
NA	NA	NA	NA	NA	NA	1	1	0.6	1	1	1	1	2	2	2	1	11
NA	NA	NA	NA	NA	NA	2	0	0.6	1	0	0	0	1	1	2	1	14
NA	NA	NA	NA	NA	NA	0	1	2.2	4	4	1	2	1	1	1	2	0
NA	NA	NA	NA	NA	NA	0	1	0.3	1	0	1	0	2	1	0	0	2
NA	NA	NA	NA	NA	NA	1	1	1.1	1	1	1	1	1	1	1	1	1
NA	NA	NA	NA	NA	NA	1	1	0.9	2	1	1	0	1	1	2	1	0
Indolines	Indoles an	Indole anc	Biosynthes	Benzoxazi	NA	1	1	1	2	0	1	0	1	0	2	1	0
O-methyl	Flavonoids	Flavonoids	NA	NA	NA	1	1	1.6	1	1	1	1	1	3	1	1	1
NA	Triazolopy	Organohe	NA	NA	NA	1	1	1.3	1	1	1	2	1	1	1	1	1
NA	NA	NA	NA	NA	NA	1	1	0.6	1	0	1	0	1	2	1	2	1
Amino aci	Carboxylic	Amino aci	NA	NA	NA	1	1	0.2	1	0	1	0	2	1	3	4	5
NA	NA	NA	NA	NA	NA	1	1	2.3	1	2	1	2	1	1	1	1	0
NA	NA	NA	Biosynthes	Stilbenoid	NA	1	1	1.3	1	1	2	1	0	2	0	1	2
NA	NA	NA	Biosynthes	Biosynthes	NA	1	6	1	1	2	2	2	1	1	1	1	2
NA	NA	NA	NA	NA	NA	1	1	0.8	2	1	1	0	1	1	1	1	1
NA	NA	NA	NA	NA	NA	5	0	1	6	0	1	1	1	1	0	1	10
NA	NA	NA	NA	NA	NA	0	0	0.1	1	0	0	0	8	3	3	0	0

NA	NA	NA	NA	NA	NA	0	3	4.1	11	2	7	3	1	1	1	7	0
NA	NA	NA	NA	NA	NA	1	1	0.8	1	1	1	0	1	1	1	1	1
NA	NA	NA	NA	NA	NA	2	1	0.7	1	1	1	1	1	0	1	2	1
NA	NA	NA	NA	NA	NA	1	1	0.7	1	1	1	1	1	0	1	0	1
NA	NA	NA	NA	NA	NA	1	1	1.4	1	1	8	1	1	1	1	0	1
Isoflavono	Isoflavono	Flavonoids	NA	NA	NA	1	1	0.8	2	3	1	0	1	0	1	1	12
						0	0	0	8	##	0	18	47	6	0	0	0
NA	NA	NA	NA	NA	NA	1	1	0.8	1	0	2	0	1	0	1	2	0
						0	0	0	0	0	0	0	0	0	0	0	0
						0	0	0	0	0	0	0	0	0	0	0	0
						0	0	0	0	0	0	0	0	0	0	0	0
						0	0	0	0	0	0	0	0	0	0	0	0
						0	0	0	0	0	0	0	0	0	0	0	0
						0	0	0	0	0	0	0	0	0	0	0	0
						0	0	0	0	0	0	0	0	0	0	0	0
						0	0	0	0	0	0	0	0	0	0	0	0
NA	NA	NA	NA	NA	NA	1	1	1.3	1	1	1	1	1	5	1	2	1
NA	NA	NA	NA	NA	NA	1	1	2.1	1	2	1	2	1	3	2	2	0
NA	NA	NA	NA	NA	NA	1	1	1.4	1	2	1	1	1	1	1	2	1
Isoprenoic	Prenol lipi	Prenol lipi	Metabolis	Ubiquinon	map00130	1	1	1.8	1	1	1	2	1	1	1	2	1
NA	NA	NA	NA	NA	NA	1	1	2.1	1	2	1	2	1	1	1	2	0
NA	NA	NA	NA	NA	NA	1	1	1.1	1	1	1	1	0	28	1	2	1
NA	NA	NA	NA	NA	NA	0	2	0.4	1	0	1	0	0	0	1	0	0
NA	NA	NA	NA	NA	NA	1	1	1.1	1	2	1	1	1	1	1	1	1
NA	NA	NA	NA	NA	NA	1	0	1.1	1	1	1	0	1	1	1	2	1
NA	NA	NA	NA	NA	NA	0	0	21	0	8	21	2	1	0	0	0	8
Carbonyl c	Organoox	Organic o	NA	NA	NA	1	1	3.4	1	3	1	9	1	1	1	1	1
NA	NA	NA	NA	NA	NA	1	1	0.7	2	1	1	1	1	1	1	1	1
NA	NA	NA	NA	NA	NA	1	1	1.8	1	2	1	2	1	1	1	2	1
						0	0	0	43	##	13	70	##	##	##	8	##
NA	NA	NA	NA	NA	NA	1	1	1.1	1	1	1	4	2	1	1	3	1
NA	NA	NA	Metabolis	Insect hor	NA	1	4	0.4	1	1	1	1	2	0	1	1	1
NA	NA	NA	NA	NA	NA	1	2	1	1	1	26	3	1	1	1	5	2
NA	NA	NA	NA	NA	NA	0	0	0.1	1	9	1	9	14	3	1	0	1
Morpholin	Oxazinane	Organohe	NA	NA	NA	1	1	2.1	1	2	1	2	1	1	3	1	1
NA	NA	NA	NA	NA	NA	1	1	2.9	0	1	1	0	1	1	12	1	1
NA	NA	NA	NA	NA	NA	1	1	0.8	1	1	2	1	1	1	1	1	1
NA	NA	NA	NA	NA	NA	1	1	1.2	1	1	1	1	1	1	1	1	1
NA	NA	NA	NA	NA	NA	0	1	0.8	1	0	1	0	1	4	2	7	0
NA	NA	NA	NA	NA	NA	1	1	1.5	1	1	1	2	1	4	1	1	1
NA	NA	NA	NA	NA	NA	1	1	0.4	3	1	0	0	0	0	1	0	0
						0	0	0	3	##	2	0	23	22	44	0	0
NA	NA	NA	NA	NA	NA	1	4	0.3	0	0	11	1	3	0	0	1	2
NA	NA	NA	NA	NA	NA	1	1	1	1	1	1	2	1	1	1	1	1
Amino aci	Carboxylic	Amino aci	NA	NA	NA	1	3	1.8	1	3	1	1	1	1	1	1	1
NA	NA	NA	NA	NA	NA	1	0	2.3	2	1	1	1	0	3	2	1	0
NA	NA	NA	NA	NA	NA	0	0	1.5	1	2	1	2	0	0	1	1	1
						0	0	0	22	53	4	68	##	68	21	0	56
NA	NA	NA	NA	NA	NA	1	1	0.7	1	1	1	1	1	1	1	1	2
NA	NA	NA	NA	NA	NA	1	1	1.9	1	2	1	2	1	1	1	2	1
NA	NA	NA	NA	NA	NA	1	1	1.2	1	4	1	3	2	2	1	1	1
NA	NA	NA	NA	NA	NA	0	2	1.5	1	1	0	5	2	0	1	4	0
						0	0	0	9	##	0	0	20	7	92	0	0
NA	NA	NA	NA	NA	NA	2	1	2.2	6	2	1	2	1	1	0	1	9
NA	NA	NA	NA	NA	NA	1	1	0.7	1	1	1	0	1	0	1	1	1
Quinone a	Prenol lipi	Quinone	NA	NA	NA	1	1	1.2	0	1	1	1	1	1	1	1	3
NA	NA	NA	NA	NA	NA	1	1	1.2	1	2	1	1	0	1	1	1	1
NA	NA	NA	NA	NA	NA	2	1	0.7	1	1	1	1	1	0	1	1	1

NA	NA	NA	NA	NA	NA	1	1	2.1	1	1	1	1	1	1	1	1	1
NA	NA	NA	NA	NA	NA	1	1	0.8	1	1	1	0	1	1	1	1	1
NA	NA	NA	NA	NA	NA	2	1	0.9	7	1	1	0	1	3	1	1	1
Stigmastar	Steroids ar	Steroids ar	Lipid meta	Steroid bic	map00100	1	2	1.4	1	2	1	4	1	2	1	1	1
NA	NA	NA	NA	NA	NA	1	1	0.2	5	0	0	0	0	0	1	0	1
Tetrahydrc	Furanoid li	Lignans ar	NA	NA	NA	1	1	1.5	2	1	1	0	0	0	2	1	0
Pyrimidine	Diazines	Organohe	NA	NA	NA	1	1	0.7	1	1	1	1	1	0	2	4	1
NA	NA	NA	NA	NA	NA	1	1	1.4	1	2	1	2	1	1	1	1	1
Benzoyl de	Benzene a	Benzene a	NA	NA	NA	2	1	2	2	2	1	1	1	1	1	1	0
NA	NA	NA	NA	NA	NA	1	0	0.2	3	0	0	0	0	0	2	0	0
NA	NA	NA	NA	NA	NA	0	1	0.8	3	1	1	0	1	0	3	0	0
NA	NA	NA	NA	NA	NA	1	1	13	1	3	##	2	1	1	1	1	11
NA	NA	NA	NA	NA	NA	1	1	0.3	0	0	1	2	1	0	1	2	1
Benzenesu	Benzene a	Benzene a	NA	NA	NA	1	1	0.6	1	1	1	2	1	3	1	1	1
Cyclopyrrc	Pyrrolopyr	Organohe	NA	NA	NA	1	1	0.8	2	1	1	1	1	1	1	2	1
						0	0	0	0	0	0	0	0	0	0	0	0
Amino aci	Carboxylic	Amino aci	Nucleotide	Pyrimidine	map00240	1	0	0.7	1	1	1	1	1	0	1	2	##
NA	NA	NA	NA	NA	NA	2	1	1.5	1	1	1	1	1	1	1	2	2
NA	NA	NA	NA	NA	NA	3	1	0.5	0	31	0	6	0	31	0	0	0
Carbohydr	Organooxy	Carbohydr	NA	NA	NA	17	3	0.2	0	1	1	1	1	3	1	0	8
NA	NA	NA	NA	NA	NA	1	1	1.7	1	2	4	2	1	1	1	1	0
NA	NA	NA	NA	NA	NA	1	1	0.7	9	12	1	12	7	1	1	1	8
Indoles	Indoles an	Indoles	Amino aci	Tryptopha	map00380	1	1	1.6	2	1	3	3	2	1	1	1	2
NA	NA	NA	NA	NA	NA	1	1	0.9	1	1	1	2	1	2	1	1	1
NA	NA	NA	NA	NA	NA	1	1	0.6	1	1	2	1	2	7	1	1	2
NA	NA	NA	NA	NA	NA	1	0	0.4	4	0	2	0	1	0	1	1	1
Amino aci	Carboxylic	Amino aci	Amino aci	Lysine bios	map00310	2	0	1.7	1	1	3	3	1	1	1	1	4
NA	NA	NA	NA	NA	NA	1	1	0.7	2	2	2	1	1	1	1	2	1
Fatty acids	Fatty Acyls	Fatty acyls	Lipid meta	Arachidon	map00590	1	1	0.8	1	1	3	1	1	1	1	1	1
NA	NA	NA	NA	NA	NA	1	1	2.5	1	3	1	3	1	1	1	2	0
NA	NA	NA	NA	NA	NA	0	1	0.8	2	1	2	1	2	0	1	1	2
NA	NA	NA	NA	NA	NA	1	1	0.7	0	2	1	2	1	1	1	1	1
NA	NA	NA	NA	NA	NA	1	1	0.5	0	8	1	10	0	0	2	1	0
NA	NA	NA	NA	NA	NA	1	1	1.3	1	1	1	2	1	1	1	1	1
NA	NA	NA	NA	NA	NA	1	1	1	1	1	1	1	1	2	1	1	1
NA	NA	NA	NA	NA	NA	1	3	0.3	1	0	3	3	1	0	0	1	1
NA	NA	NA	NA	NA	NA	1	1	0.2	1	1	1	0	0	1	1	1	0
NA	NA	NA	NA	NA	NA	1	1	2.3	1	3	1	3	1	1	1	2	0
NA	NA	NA	Xenobiotic	Naphthale	NA	4	2	0.8	9	1	2	1	3	1	1	1	4
NA	NA	NA	NA	NA	NA	1	1	0.8	2	1	1	1	1	1	1	1	1
NA	NA	NA	NA	NA	NA	1	1	1.2	1	1	1	1	1	1	1	1	1
NA	NA	NA	NA	NA	NA	1	1	1.8	1	2	1	2	1	1	1	1	1
Fatty acids	Fatty Acyls	Fatty acyls	Lipid meta	Biosynthes	map01040	1	1	0.6	2	1	1	1	5	10	0	1	##
NA	NA	NA	NA	NA	NA	1	0	1.8	4	13	1	30	0	1	0	1	1
Bile acids,	Steroids ar	Bile acids,	NA	NA	NA	1	0	0.3	1	2	1	2	1	1	1	1	0
NA	NA	NA	NA	NA	NA	1	1	0.7	2	2	1	1	1	1	1	2	1
NA	NA	NA	NA	NA	NA	1	0	0.5	1	0	1	1	1	##	2	1	0
NA	NA	NA	NA	NA	NA	1	1	0.8	1	1	1	0	1	1	1	1	1
						0	0	0	0	0	0	0	0	0	0	0	0
						0	0	0	0	0	0	0	0	0	0	0	0
						0	0	0	0	0	0	0	0	0	0	0	0
Quinolizid	Alkaloids c	Alkaloids	Biosynthes	Tropane, p	NA	1	1	2.8	1	3	2	3	1	1	1	2	0
NA	NA	NA	NA	NA	NA	1	1	1.8	2	2	3	2	2	1	1	1	1
NA	NA	NA	NA	NA	NA	1	1	1.2	1	1	4	1	1	1	1	1	1
NA	NA	NA	NA	NA	NA	1	1	1.4	1	1	1	1	1	2	1	1	1
NA	NA	NA	NA	NA	NA	1	1	1.6	1	1	0	2	1	1	1	1	1
NA	NA	NA	NA	NA	NA	30	1	4.1	2	1	19	1	1	1	1	1	1
NA	NA	NA	NA	NA	NA	1	0	0.5	2	0	1	0	1	0	2	2	5

Dibenzoxa	Benzoxaze	Organohe	NA	NA	NA	0	0	0.5	4	0	1	0	2	0	1	1	4
NA	NA	NA	NA	NA	NA	1	1	0.7	1	1	1	6	1	0	1	1	1
NA	NA	NA	NA	NA	NA	1	1	0.5	1	1	1	1	2	0	9	1	2
NA	NA	NA	NA	NA	NA	1	0	18	2	1	1	0	0	0	1	7	1
NA	NA	NA	NA	NA	NA	1	1	1.1	1	1	1	1	1	1	1	1	3
NA	5'-deoxyri	Nucleic ac	NA	NA	NA	1	1	1.5	1	1	1	1	1	1	1	1	1
NA	NA	NA	NA	NA	NA	1	1	1.8	1	2	1	2	1	9	1	1	28
Benzoic ac	Benzene a	Benzene a	NA	NA	NA	1	7	0.7	1	4	##	6	0	0	2	1	0
NA	NA	NA	NA	NA	NA	1	1	0.9	1	1	1	1	14	2	2	1	1
Flavonoid	Flavonoids	Flavonoids	Biosynthes	Flavone ar	NA	1	1	1.3	19	1	1	2	1	1	1	5	0
						0	0	0	0	0	0	12	0	0	45	0	8
NA	NA	NA	NA	NA	NA	1	1	1.5	1	2	5	2	2	1	1	1	2
Carbohydr	Organoox	Carbohydr	Carbohydr	Galactose	map00052	1	1	2.4	1	1	3	2	1	1	1	1	1
NA	NA	NA	NA	NA	NA	1	1	0.6	1	1	1	1	1	1	1	1	2
NA	NA	NA	NA	NA	NA	1	1	1.3	1	1	2	1	0	1	1	1	1
NA	NA	NA	NA	NA	NA	6	1	1.9	1	2	1	5	1	1	1	1	1
Glucosinol	Glucosinol	Amino aci	Biosynthes	Glucosinol	map0121(	1	1	2	1	2	1	2	1	1	1	2	1
						0	0	0	0	0	0	0	0	3	44	0	0
Cinnamic a	Cinnamic a	Polyketide	NA	NA	NA	1	1	1	0	2	1	1	1	1	1	2	0
NA	NA	NA	NA	NA	NA	1	2	1.4	1	2	1	1	2	1	1	1	3
NA	NA	NA	NA	NA	NA	1	1	0.8	1	1	1	1	1	3	1	1	1
NA	NA	NA	NA	NA	NA	1	1	2.1	1	1	1	1	1	1	1	1	2
NA	NA	NA	NA	NA	NA	1	1	1	1	1	1	0	1	1	1	1	1
NA	Yohimbine	Alkaloids a	NA	NA	NA	1	1	1	1	1	1	1	1	2	1	1	1
NA	NA	NA	NA	NA	NA	1	1	0.7	1	1	1	1	1	2	2	2	0
NA	NA	NA	NA	NA	NA	1	1	1.2	1	1	1	1	1	1	1	1	1
						0	0	0	0	0	0	0	0	0	0	0	0
NA	NA	NA	NA	NA	NA	1	1	1.3	1	1	1	2	1	1	1	1	10
NA	NA	NA	NA	NA	NA	1	1	0.6	1	1	1	1	1	1	1	1	2
NA	NA	NA	NA	NA	NA	1	1	0.4	3	0	1	0	1	0	1	2	0
NA	NA	NA	NA	NA	NA	1	1	2.3	1	3	1	3	1	1	1	2	0
NA	NA	NA	NA	NA	NA	1	1	0.7	1	0	1	0	1	6	2	1	0
Morpholin	Oxazinane	Organohe	NA	NA	NA	1	1	0.8	1	1	1	1	0	2	1	1	1
Benzylisoc	Isoquinolir	Organohe	NA	NA	NA	16	0	2.1	0	2	0	1	2	2	1	0	4
NA	NA	NA	NA	NA	NA	3	2	0.9	1	1	1	1	2	1	1	1	1
NA	NA	NA	NA	NA	NA	1	1	0.8	2	0	1	0	1	1	2	1	0
NA	NA	NA	NA	NA	NA	1	1	1	2	0	1	0	1	0	2	2	0
Arylsulfate	Organic su	Organic ac	NA	NA	NA	1	1	1.6	1	1	1	1	0	1	1	1	1
NA	Biotin and	Organohe	Metabolisı	Biotin met	map0078(	1	1	2.9	1	2	1	2	1	1	1	1	1
Amino aci	Carboxylic	Amino aci	NA	NA	NA	0	4	6.3	1	1	2	2	0	1	1	12	1
NA	NA	NA	NA	NA	NA	0	0	1.4	1	2	1	2	0	0	0	1	1
						0	0	0	0	0	0	0	0	0	0	0	0
NA	NA	NA	NA	NA	NA	1	1	1.3	1	1	2	8	1	1	1	1	1
Androstan	Steroids ar	Steroids ar	NA	NA	NA	0	1	0.3	0	2	6	1	0	0	0	0	1
						0	0	0	0	0	0	0	17	9	0	0	7
NA	NA	NA	NA	NA	NA	1	2	1.6	1	2	1	2	1	2	1	1	1
NA	NA	NA	NA	NA	NA	1	1	1.5	1	1	1	2	0	1	1	1	1
NA	NA	NA	NA	NA	NA	1	1	1.8	1	2	2	2	1	1	1	2	0
NA	NA	NA	NA	NA	NA	1	1	0.9	1	1	1	0	1	1	1	1	1
Zingiber d	Others	Polyketide	Biosynthes	Stilbenoid	NA	1	3	1	1	2	1	7	1	1	1	1	1
Piperidine	Alkaloids c	Alkaloids	NA	NA	NA	1	1	2.6	1	1	1	1	0	1	4	1	1
NA	NA	NA	NA	NA	NA	1	1	0.8	1	1	1	1	1	1	1	1	5
Terpenoid	Alkaloids c	Alkaloids	NA	NA	NA	2	1	0.7	2	1	1	0	1	1	1	1	1
NA	NA	NA	NA	NA	NA	1	1	0.5	1	1	1	1	2	1	1	0	2
NA	NA	NA	NA	NA	NA	1	1	2.4	2	4	0	3	1	2	4	4	2
NA	NA	NA	NA	NA	NA	1	1	1.2	1	1	1	2	1	1	1	1	2
NA	NA	NA	NA	NA	NA	1	1	1	3	0	0	0	1	0	2	0	0
NA	NA	NA	NA	NA	NA	1	1	1	1	1	1	1	1	1	1	2	1

NA	NA	NA	NA	NA	NA	0	1	5.4	1	1	6	2	1	1	1	7	0
NA	NA	NA	NA	NA	NA	1	1	1.1	1	1	1	0	1	1	1	2	1
NA	NA	NA	NA	NA	NA	1	1	0.7	1	1	1	1	3	0	0	1	1
NA	NA	NA	NA	NA	NA	1	1	0.5	2	1	1	0	0	10	1	2	1
NA	NA	NA	NA	NA	NA	1	1	1	1	1	1	2	2	1	1	1	1
NA	NA	NA	NA	NA	NA	0	3	0.6	1	1	1	1	1	2	1	1	8
NA	NA	NA	NA	NA	NA	1	1	1.1	1	1	1	2	1	2	1	1	4
alpha-Nap	Naphthoq	Quinone	NA	NA	NA	1	1	15	0	4	96	3	1	1	2	1	8
						0	0	0	0	16	0	0	0	4	0	0	0
NA	NA	NA	NA	NA	NA	1	1	0.9	2	2	1	1	1	1	1	2	1
Pyrrolidiny	Pyridines a	Pyridine ar	NA	NA	NA	1	1	1.1	3	0	0	0	0	0	3	1	0
NA	NA	NA	NA	NA	NA	1	1	0.9	1	1	1	2	1	1	1	1	1
NA	NA	NA	NA	NA	NA	1	1	0.7	1	1	1	1	1	1	1	1	2
NA	NA	NA	NA	NA	NA	1	4	3	1	0	2	0	1	0	1	3	0
NA	NA	NA	NA	NA	NA	1	1	12	6	42	1	49	0	2	57	0	48
NA	NA	NA	NA	NA	NA	0	1	1.2	1	2	1	2	0	0	0	0	2
NA	NA	NA	NA	NA	NA	1	1	3	1	2	1	3	1	1	1	1	1
NA	NA	NA	NA	NA	NA	1	1	1	1	1	1	1	1	1	1	1	1
						0	0	0	29	20	7	##	##	##	##	0	##
NA	NA	NA	NA	NA	NA	1	1	1.3	1	2	1	1	1	1	1	1	1
NA	NA	NA	NA	NA	NA	1	1	0.3	3	0	0	0	11	0	4	6	3
NA	NA	NA	NA	NA	NA	1	1	1	1	1	1	0	1	1	1	1	1
NA	NA	NA	NA	NA	NA	1	1	0.8	1	1	4	1	8	1	1	1	3
NA	NA	NA	NA	NA	NA	1	1	2	1	2	1	2	1	1	1	2	0
NA	NA	NA	NA	NA	NA	1	1	1.2	1	1	32	1	2	1	1	1	1
						3	0	0	##	##	12	##	##	##	##	0	58
Triterpeno	Prenol lipi	Terpenoid	NA	NA	NA	1	2	0.5	0	1	2	1	1	4	8	0	8
						0	0	0	0	0	0	0	0	0	0	0	0
						0	0	0	0	0	0	0	0	0	0	0	0
O-methyla	Isoflavono	Flavonoids	Biosynthes	Isoflavono	NA	1	1	0.6	1	1	1	1	2	2	1	2	12
NA	Phenylpro	Phenylpro	NA	NA	NA	3	0	1.2	2	1	0	1	2	0	1	1	2
NA	NA	NA	NA	NA	NA	1	1	2	1	2	2	2	1	1	1	2	1
						0	0	0	0	0	0	0	0	0	0	0	0
						0	0	0	0	0	0	0	0	4	0	0	0
NA	NA	NA	NA	NA	NA	1	1	1	1	1	1	0	1	1	1	1	1
NA	NA	NA	NA	NA	NA	1	1	1	1	1	1	0	1	1	26	1	1
NA	NA	NA	NA	NA	NA	1	1	1.7	1	2	1	2	1	1	1	1	1
NA	NA	NA	NA	NA	NA	2	1	0.7	1	1	1	1	1	1	1	2	1
NA	NA	NA	NA	NA	NA	1	1	2.7	1	2	1	2	1	6	1	2	0
NA	NA	NA	NA	NA	NA	1	1	0.9	1	1	1	0	1	1	1	1	1
NA	NA	NA	Biosynthes	Stilbenoid	NA	2	1	2.3	1	1	1	0	1	3	1	1	1
NA	NA	NA	NA	NA	NA	1	1	1.2	1	1	1	2	1	1	1	1	1
NA	NA	NA	NA	NA	NA	0	0	0.7	1	4	##	2	1	0	1	0	2
Lipoamide	Dithiolane	Organohe	NA	NA	NA	1	1	0.8	1	0	1	1	2	1	1	2	1
NA	NA	NA	NA	NA	NA	1	1	4.8	1	10	1	18	0	1	1	1	0
NA	NA	NA	NA	NA	NA	3	2	0.2	10	0	1	1	0	0	1	1	3
NA	NA	NA	NA	NA	NA	1	1	0.8	1	0	1	0	1	1	1	2	0
NA	NA	NA	NA	NA	NA	0	1	1.9	0	2	1	2	0	2	1	2	0
NA	NA	NA	NA	NA	NA	1	1	1	1	1	1	1	1	1	1	1	1
NA	NA	NA	NA	NA	NA	1	1	0.9	2	1	1	0	1	1	1	1	1
Bufanolide	Steroids	Terpenoid	NA	NA	NA	1	1	0.7	1	1	1	0	1	2	1	1	1
NA	NA	NA	NA	NA	NA	1	1	2	1	2	1	2	1	1	1	2	1
NA	NA	NA	NA	NA	NA	1	2	0.2	0	1	2	1	1	0	0	1	12
NA	NA	NA	NA	NA	NA	66	3	1.4	1	1	0	1	0	0	0	1	1
Medium-c	Keto acids	Organic ac	NA	NA	NA	1	1	2.2	1	2	1	2	1	1	1	2	1
Terpene g	Prenol lipi	Terpenoid	NA	NA	NA	1	1	0.9	1	1	1	0	1	1	1	1	1
Imidazoles	Azoles	Imidazoles	Amino aci	Histidine r	map00340	3	0	4.2	2	0	1	1	0	2	2	1	1
NA	NA	NA	NA	NA	NA	4	2	2.1	1	0	0	1	1	0	0	1	1

Amino aci	Carboxylic	Amino aci	NA	NA	NA	1	1	1.9	1	2	1	2	1	1	1	2	1
NA	NA	NA	NA	NA	NA	1	1	1	1	1	3	1	1	1	1	1	4
NA	NA	NA	NA	NA	NA	1	0	0.5	4	0	1	0	3	0	2	1	1
NA	NA	NA	NA	NA	NA	1	1	1.7	1	2	6	2	1	1	1	1	1
						0	0	0	0	0	0	0	0	0	0	0	0
NA	NA	NA	NA	NA	NA	19	1	1.1	1	1	1	1	1	1	1	1	1
NA	NA	NA	NA	NA	NA	10	1	1.7	1	1	1	2	1	1	1	1	1
						0	0	0	5	##	0	10	51	20	18	0	0
NA	NA	NA	NA	NA	NA	1	1	1.1	1	1	1	1	1	1	1	1	1
NA	NA	NA	NA	NA	NA	2	1	1.9	1	3	1	2	1	1	1	1	1
NA	Fluorenes	Benzene a	NA	NA	NA	1	1	1.5	1	1	1	2	1	1	1	3	1
Benzenetr	Phenols	Phenols ar	Xenobiotic	Aminoben	NA	2	1	0.6	1	2	0	1	1	1	1	0	0
NA	NA	NA	NA	NA	NA	1	1	0.9	1	1	1	1	1	1	1	1	1
NA	NA	NA	NA	NA	NA	1	2	1.7	0	1	1	3	1	1	0	0	1
NA	NA	NA	NA	NA	NA	1	1	0.7	2	1	2	1	1	1	1	2	1
NA	NA	NA	NA	NA	NA	1	1	0.9	1	1	1	0	1	1	1	1	1
NA	NA	NA	NA	NA	NA	1	1	1.2	1	1	1	1	4	1	1	1	1
Fatty acids	Fatty Acyls	Fatty acyls	NA	NA	NA	1	1	4.1	1	2	2	2	1	1	1	4	1
NA	NA	NA	NA	NA	NA	1	2	0.4	1	1	1	4	2	0	1	1	1
NA	NA	NA	NA	NA	NA	2	1	0.6	1	1	1	1	0	3	1	2	1
NA	NA	NA	NA	NA	NA	1	1	0.1	1	0	1	0	1	1	0	0	1
NA	NA	NA	NA	NA	NA	1	1	25	1	1	##	1	1	1	1	1	1
NA	NA	NA	NA	NA	NA	1	1	0.9	2	0	1	0	1	0	2	1	0
						0	0	0	0	0	0	0	0	0	11	0	0
NA	NA	NA	NA	NA	NA	1	1	1	1	1	1	2	1	1	1	1	1
NA	NA	NA	NA	NA	NA	1	1	0.2	1	0	0	0	0	1	3	0	0
NA	NA	NA	NA	NA	NA	1	1	1.9	1	2	1	2	1	1	1	2	1
NA	NA	NA	NA	NA	NA	1	1	1.7	1	2	1	2	1	1	1	1	0
NA	NA	NA	NA	NA	NA	2	1	1.9	1	2	1	2	1	1	1	1	0
NA	NA	NA	NA	NA	NA	1	2	1.5	2	1	1	0	0	0	2	1	0
Ethers	Organoox	Organic o	NA	NA	NA	1	1	1.5	1	2	1	1	1	1	1	2	1
NA	NA	NA	NA	NA	NA	2	1	0.4	3	3	2	6	5	1	1	3	7
NA	NA	NA	NA	NA	NA	4	1	2.2	12	2	1	1	3	1	1	1	1
NA	NA	NA	NA	NA	NA	0	8	0.2	2	0	0	0	1	0	1	0	8
NA	NA	NA	NA	NA	NA	1	1	1.9	1	2	1	2	1	1	1	1	2
NA	NA	NA	Biosynthes	Streptomy	NA	2	1	0.5	1	0	6	0	1	1	1	1	1
NA	NA	NA	NA	NA	NA	1	1	1.1	1	1	9	1	5	1	1	1	2
Pterins an	Pteridines	Pteridines	Metabolis	Folate bio	map0079C	1	1	1.2	1	1	1	1	1	1	1	1	1
NA	NA	NA	NA	NA	NA	3	1	1.2	1	0	0	1	1	0	1	1	0
NA	NA	NA	NA	NA	NA	1	1	0.9	2	1	1	0	1	1	1	1	1
Benzoic ac	Benzene a	Benzene a	NA	NA	NA	1	1	1	1	1	1	0	1	1	1	1	1
NA	NA	NA	NA	NA	NA	1	1	1	1	1	1	0	1	1	1	1	1
NA	NA	NA	NA	NA	NA	10	0	3.7	0	3	12	1	##	0	1	3	1
Sulfinylber	Benzimida	Imidazole	NA	NA	NA	0	##	1.3	1	1	1	##	0	0	0	0	1
NA	NA	NA	NA	NA	NA	1	1	0.8	1	1	1	1	0	2	1	1	1
NA	NA	NA	NA	NA	NA	1	1	1.1	1	1	1	2	1	1	1	1	1
NA	NA	NA	NA	NA	NA	2	0	0.5	0	2	1	1	0	1	1	0	4
NA	NA	NA	NA	NA	NA	1	1	1.2	1	2	1	1	1	1	1	1	1
NA	NA	NA	NA	NA	NA	1	1	1.1	1	1	1	1	1	1	1	1	1
NA	NA	NA	NA	NA	NA	1	1	1.9	1	2	1	2	1	1	1	2	1
NA	NA	NA	NA	NA	NA	1	4	0.7	2	1	2	1	0	1	0	1	1
						0	0	0	0	0	0	0	0	0	0	0	0
NA	NA	NA	NA	NA	NA	1	1	1.4	1	1	1	2	1	1	1	1	1
Carbonyl c	Organoox	Organic o	NA	NA	NA	2	1	0.7	1	1	1	0	1	6	1	1	1
NA	NA	NA	NA	NA	NA	0	1	0.2	1	1	0	0	2	21	3	1	9
NA	NA	NA	NA	NA	NA	1	1	1.7	1	2	1	2	1	1	1	2	1
NA	NA	NA	NA	NA	NA	0	3	0.3	0	0	0	4	3	1	1	0	3
NA	NA	NA	NA	NA	NA	2	1	0.3	3	0	1	0	1	0	2	1	1

NA	NA	NA	NA	NA	NA	1	0	0.7	3	1	0	1	1	0	0	1	1
Eicosanoic	Fatty Acyls	Fatty acyls	NA	NA	NA	0	0	0.7	0	0	0	0	2	0	2	1	0
NA	NA	NA	NA	NA	NA	1	1	2	1	2	1	2	1	1	1	2	1
Purine ribo	Purine nuc	Purines an	NA	NA	NA	1	1	1.3	1	1	1	1	1	1	1	1	1
NA	NA	NA	NA	NA	NA	1	1	1	12	1	0	1	1	0	2	1	0
NA	NA	NA	NA	NA	NA	1	1	3	3	4	0	3	1	1	5	5	2
NA	NA	NA	Global and	Biosynthes	NA	1	1	1.5	1	1	1	1	1	1	1	1	1
NA	NA	NA	NA	NA	NA	3	1	2	1	2	1	2	1	1	1	1	1
NA	NA	NA	NA	NA	NA	1	2	1.8	1	2	1	2	1	2	1	1	1
NA	NA	NA	NA	NA	NA	1	1	0.6	1	1	1	1	1	1	1	1	2
NA	NA	NA	NA	NA	NA	1	1	1.4	1	1	1	1	1	1	1	1	1
NA	NA	NA	NA	NA	NA	1	1	0.6	3	0	1	0	1	0	1	0	1
NA	NA	NA	NA	NA	NA	1	1	0.9	1	30	1	2	1	1	1	1	2
NA	NA	NA	NA	NA	NA	1	1	1.8	1	2	1	2	1	1	1	2	1
NA	NA	NA	NA	NA	NA	0	0	15	0	18	##	3	1	1	0	0	4
NA	NA	NA	NA	NA	NA	1	7	1	0	1	1	1	0	0	4	1	0
NA	Phenol eth	Benzene a	NA	NA	NA	1	1	0.7	1	0	0	1	9	0	1	1	0
NA	NA	NA	NA	NA	NA	1	2	0.2	2	0	0	0	0	0	1	0	0
NA	NA	NA	NA	NA	NA	1	1	0.9	1	1	1	0	1	1	1	1	1
NA	NA	NA	NA	NA	NA	2	1	0.7	1	1	18	1	1	1	1	1	1
NA	NA	NA	NA	NA	NA	1	1	0.4	1	1	1	4	0	2	2	1	0
Nitrobenz	Benzene a	Benzene a	NA	NA	NA	1	1	0.9	1	1	1	0	1	1	2	1	1
NA	Linear 1,3-	Polyketide	NA	NA	NA	2	2	0.3	1	0	1	0	2	3	3	2	1
NA	NA	NA	NA	NA	NA	1	2	0.9	1	1	2	0	1	1	1	1	0
NA	NA	NA	NA	NA	NA	1	0	0.7	1	2	1	1	1	0	1	1	1
NA	NA	NA	NA	NA	NA	1	2	0.8	1	1	1	1	6	2	1	1	1
NA	NA	NA	NA	NA	NA	1	1	1.3	1	1	1	1	1	1	1	1	1
NA	NA	NA	NA	NA	NA	1	1	1	2	1	1	0	1	1	1	1	1
						0	0	0	0	0	0	0	0	15	0	0	0
NA	NA	NA	NA	NA	NA	2	1	0.5	1	0	1	0	2	0	7	1	7
NA	NA	NA	NA	NA	NA	1	1	1.5	1	2	39	2	1	2	1	1	1
Carbohydr	Organooxy	Carbohydr	NA	NA	NA	1	1	0.7	1	0	1	0	1	1	1	1	0
NA	NA	NA	NA	NA	NA	2	1	0.8	1	1	1	0	1	0	1	1	1
NA	NA	NA	NA	NA	NA	1	1	1.1	1	1	1	2	1	1	1	1	1
NA	NA	NA	NA	NA	NA	1	1	0.7	6	0	1	0	1	2	1	2	1
Guaianolid	Sesquiterp	Terpenoid	NA	NA	NA	1	1	0.7	1	0	1	0	2	2	1	2	1
Purine alka	Others	Alkaloids	Biosynthes	Caffeine m	map00232	1	3	0.9	2	2	0	0	0	0	2	0	0
Daphnane	Diterpenoi	Terpenoid	NA	NA	NA	1	8	1.8	1	2	1	2	1	1	1	1	1
Bilirubins	Tetrapyrro	Organohe	Metabolis	Porphyrin	map00860	0	0	0.1	1	0	0	0	1	14	2	0	2
NA	NA	NA	NA	NA	NA	1	1	1.5	1	1	1	1	2	1	1	1	1
NA	NA	NA	NA	NA	NA	1	1	1.5	1	1	2	2	1	1	1	2	1
Triterpeno	Prenol lipi	Terpenoid	NA	NA	NA	1	1	1.6	1	2	1	2	1	1	1	2	0
NA	NA	NA	NA	NA	NA	1	1	2	1	2	1	2	1	1	1	1	1
NA	Thienopyr	Pyridine ar	NA	NA	NA	0	0	0.9	6	0	0	0	1	0	5	0	0
NA	NA	NA	NA	NA	NA	1	1	0.7	1	1	1	1	1	1	1	1	2
NA	NA	NA	NA	NA	NA	0	1	0.7	0	0	0	0	2	1	9	0	4
Fatty acid	Fatty Acyls	Fatty acyls	NA	NA	NA	1	1	1.1	1	1	1	1	1	1	1	2	1
NA	NA	NA	NA	NA	NA	1	1	1.2	2	3	1	2	1	1	1	1	1
NA	NA	NA	NA	NA	NA	0	14	0.3	0	1	1	3	0	10	1	0	0
						0	0	0	69	##	12	##	##	68	97	3	31
NA	NA	NA	NA	NA	NA	1	1	0.9	1	1	3	2	1	1	1	1	1
NA	NA	NA	NA	NA	NA	1	1	0.7	1	1	1	0	1	0	1	1	1
NA	NA	NA	NA	NA	NA	14	1	0.5	2	41	19	25	0	0	0	10	4
NA	NA	NA	NA	NA	NA	2	3	2.3	2	12	12	3	0	2	0	2	0
NA	NA	NA	NA	NA	NA	1	1	1.4	1	1	1	1	1	1	1	1	0
Amino aci	Carboxylic	Amino aci	NA	NA	NA	1	1	2	1	2	1	2	1	1	1	1	1
NA	NA	NA	NA	NA	NA	1	1	1.7	1	2	1	2	1	1	1	1	0
Fatty acids	Fatty Acyls	Fatty acyls	NA	NA	NA	1	1	1.3	1	1	0	2	2	1	1	3	0

NA	NA	NA	NA	NA	NA	1	1	1.3	1	1	1	1	1	1	1	1	1
						0	0	0	0	0	0	##	##	85	55	0	83
NA	NA	NA	NA	NA	NA	1	1	2.3	1	2	1	1	1	1	1	2	1
NA	NA	NA	NA	NA	NA	1	1	1.3	1	1	1	1	1	1	1	1	1
Amino aci	Carboxylic	Amino aci	NA	NA	NA	1	1	0.9	1	1	1	1	2	1	1	1	92
NA	NA	NA	NA	NA	NA	16	1	1	2	7	1	1	1	1	1	4	##
NA	NA	NA	NA	NA	NA	48	1	1	1	1	1	1	1	1	1	1	1
NA	NA	NA	NA	NA	NA	1	0	2.2	1	1	3	1	1	1	1	0	0
NA	NA	NA	NA	NA	NA	1	1	1.3	0	1	22	1	5	1	1	1	10
NA	NA	NA	NA	NA	NA	1	1	0.5	1	0	1	0	2	0	2	0	0
NA	NA	NA	NA	NA	NA	1	1	1.1	1	2	1	1	1	1	1	1	1
NA	NA	NA	NA	NA	NA	1	1	1.4	1	1	1	1	0	1	1	1	1
NA	NA	NA	NA	NA	NA	1	1	0.6	1	1	34	1	4	0	1	2	2
						0	0	0	0	0	0	4	0	0	0	0	0
Fatty amid	Fatty Acyls	Fatty acyls	NA	NA	NA	1	1	1.5	1	2	1	2	1	10	1	2	3
NA	NA	NA	NA	NA	NA	2	1	0.4	4	1	0	0	0	1	6	0	0
NA	NA	NA	NA	NA	NA	25	1	3.6	0	4	10	1	10	1	1	1	5
NA	NA	NA	NA	NA	NA	1	1	0.7	3	0	1	0	1	0	2	1	0
NA	Benzothia:	Organohe	NA	NA	NA	1	0	0.8	3	2	1	2	1	1	0	2	1
NA	NA	NA	Biosynthes	Acarbose a	map0110(	1	1	0.9	1	1	5	1	3	1	1	1	7
NA	NA	NA	NA	NA	NA	1	1	0.9	1	1	1	0	1	1	1	1	1
NA	NA	NA	NA	NA	NA	1	0	0.2	4	0	0	0	0	0	3	0	0
NA	NA	NA	NA	NA	NA	1	1	1.6	1	2	3	2	1	1	1	1	1
NA	NA	NA	NA	NA	NA	1	1	1.2	2	1	2	0	3	0	3	1	0
Morpholin	Oxazinane	Organohe	NA	NA	NA	1	1	0.8	0	3	1	2	3	7	1	1	1
NA	NA	NA	NA	NA	NA	1	1	3.4	1	4	3	4	1	1	2	3	1
NA	NA	NA	NA	NA	NA	1	1	1.8	1	2	1	2	1	1	1	1	1
NA	NA	NA	NA	NA	NA	1	1	1.7	1	2	2	2	1	1	1	2	4
NA	NA	NA	NA	NA	NA	1	1	0.8	1	1	1	0	1	2	1	1	1
NA	NA	NA	NA	NA	NA	1	1	0.8	1	1	1	1	0	2	1	1	1
NA	NA	NA	NA	NA	NA	3	1	0.5	3	0	1	0	1	1	3	3	0
NA	NA	NA	NA	NA	NA	1	1	2.3	1	2	1	2	1	1	1	2	1
NA	NA	NA	NA	NA	NA	1	1	0.9	1	2	4	1	1	1	1	1	1
NA	NA	NA	NA	NA	NA	1	1	1.5	1	2	1	2	1	1	1	1	1
NA	NA	NA	NA	NA	NA	1	1	1.8	1	2	1	2	1	1	1	2	0
Diphenylm	Benzene a	Benzene a	NA	NA	NA	1	1	1.3	1	1	1	2	1	1	1	1	6
NA	NA	NA	Amino aci	Tyrosine m	map00350	3	1	0.8	4	1	1	1	1	1	0	1	1
						7	0	0	0	0	0	0	27	0	19	0	0
Lineolic ac	Fatty Acyls	Fatty acyls	NA	NA	NA	15	0	0.1	1	15	4	2	2	0	1	20	0
NA	NA	NA	NA	NA	NA	1	1	1	1	1	1	0	1	1	1	1	1
Fatty acid	Fatty Acyls	Fatty acyls	NA	NA	NA	2	0	0.1	2	3	0	1	0	1	1	0	6
NA	NA	NA	NA	NA	NA	1	2	3.3	1	1	11	1	2	1	1	2	2
NA	NA	NA	NA	NA	NA	1	1	1.8	1	1	1	0	1	1	2	1	1
NA	NA	NA	NA	NA	NA	1	1	1	8	1	1	1	8	1	6	1	5
NA	NA	NA	NA	NA	NA	1	3	7.1	5	1	1	3	1	1	0	2	1
NA	NA	NA	NA	NA	NA	1	1	0.9	1	1	1	0	1	1	1	1	1
NA	NA	NA	NA	NA	NA	1	1	2	1	2	1	2	1	1	1	2	1
NA	NA	NA	NA	NA	NA	2	1	0.5	3	0	0	0	1	0	2	0	1
NA	NA	NA	NA	NA	NA	1	1	0.8	1	0	1	0	1	1	1	2	18
NA	NA	NA	NA	NA	NA	1	4	0.9	0	1	1	6	0	1	1	0	0
Amino aci	Carboxylic	Amino aci	NA	NA	NA	2	1	0.7	1	1	1	1	1	0	1	2	1
NA	NA	NA	NA	NA	NA	1	1	0.8	1	1	1	1	1	1	2	2	0
Others	Alkaloids	Cyanotoxi	NA	NA	NA	1	0	0.7	3	1	1	1	1	0	3	2	1
NA	NA	NA	NA	NA	NA	1	1	0.9	1	1	1	0	1	10	1	1	1
NA	NA	NA	NA	NA	NA	1	1	0.7	2	1	1	0	2	1	1	2	1
NA	NA	NA	Biosynthes	Isoquinolir	map0110(	1	1	0.7	1	1	1	1	1	2	1	1	1
						0	0	7	4	15	0	97	37	0	##	0	0
NA	NA	NA	NA	NA	NA	1	1	0.6	1	1	1	1	1	1	1	1	2

NA	NA	NA	NA	NA	NA	1	1	1	1	1	10	2	1	1	1	1	1
NA	NA	NA	NA	NA	NA	1	1	1.2	1	1	1	1	1	1	1	1	1
NA	NA	NA	NA	NA	NA	1	1	1.2	1	2	1	2	0	1	0	1	2
NA	NA	NA	NA	NA	NA	1	1	0.9	1	0	1	0	1	1	1	1	0
NA	NA	NA	NA	NA	NA	1	2	1.4	1	1	26	2	1	1	1	1	5
NA	NA	NA	NA	NA	NA	1	1	1.8	1	1	1	1	1	1	1	0	1
NA	NA	NA	NA	NA	NA	1	1	0.9	1	1	1	0	1	1	1	1	1
NA	Pyrimidine	Nucleic ac	NA	NA	NA	1	1	1.1	1	1	1	2	2	1	1	1	0
NA	Isobenzof	Organohe	NA	NA	NA	1	1	0.8	2	2	0	5	3	4	1	1	0
NA	NA	NA	NA	NA	NA	0	1	0.7	1	5	0	2	1	1	0	1	4
NA	NA	NA	NA	NA	NA	1	1	1.2	1	1	1	1	1	1	1	1	1
						0	0	0	0	0	0	0	0	0	0	0	0
						0	0	0	0	0	0	0	0	0	0	0	0
						0	0	0	0	0	0	0	0	0	0	0	0
						0	0	0	0	0	0	0	0	0	0	0	0
						0	0	0	0	0	0	0	0	0	0	0	0
						0	0	0	0	0	0	0	0	0	0	0	0
						0	0	0	0	0	0	0	0	0	0	0	0
						0	0	0	0	0	0	0	0	0	0	0	0
						0	0	0	0	0	0	0	0	0	0	0	0
Cycloalkar	Others [Fi	Antibiotics	NA	NA	NA	3	1	1.1	1	2	1	2	1	3	1	1	5
						3	0	0	7	76	0	11	28	0	0	0	10
NA	NA	NA	NA	NA	NA	1	0	0.3	1	0	14	2	1	##	1	0	1
						2	0	0	25	##	0	##	53	36	91	0	9
						0	0	0	0	8	0	0	0	0	0	0	0
NA	NA	NA	NA	NA	NA	1	1	1.3	1	8	1	2	1	2	1	1	1
Pyrrolizidir	Alkaloids (	Alkaloids	NA	NA	NA	1	1	0.8	1	0	1	0	2	1	1	2	1
						0	0	0	0	0	0	0	0	0	0	0	0
NA	NA	NA	NA	NA	NA	1	1	0.8	1	1	1	1	1	1	1	1	1
NA	NA	NA	NA	NA	NA	1	1	1.7	1	2	1	2	1	1	1	3	4
NA	NA	NA	NA	NA	NA	1	1	0.9	1	1	1	0	1	1	1	0	1
NA	NA	NA	NA	NA	NA	3	0	0.4	1	2	1	1	1	0	1	1	1
Isoflavono	Isoflavono	Flavonoids	NA	NA	NA	12	0	0.3	10	0	0	4	1	0	1	8	0
NA	NA	NA	NA	NA	NA	1	1	1.4	1	2	1	1	1	1	1	2	1
Indoles	Indoles an	Indoles	NA	NA	NA	0	1	2.4	5	0	1	0	2	0	7	6	0
NA	NA	NA	NA	NA	NA	1	1	0.9	1	1	1	1	1	1	1	2	1
NA	NA	NA	NA	NA	NA	1	1	0.9	1	1	1	2	1	2	1	1	1
NA	NA	NA	NA	NA	NA	1	1	3.1	1	1	1	1	1	1	1	2	0
						0	0	0	0	0	0	0	82	0	0	0	0
NA	NA	NA	NA	NA	NA	2	1	1.6	1	2	1	2	1	1	1	1	0
NA	NA	NA	NA	NA	NA	1	1	1.3	1	1	##	2	1	1	1	2	1
Carbohydı	Organoox	Carbohydı	Carbohydı	Amino suç	map0052C	1	2	1	1	3	2	7	1	2	2	2	3
NA	NA	NA	NA	NA	NA	1	4	3.1	0	##	1	1	1	1	1	1	1
NA	NA	NA	Biosynthes	Isoquinolir	NA	1	1	0.9	1	1	1	1	0	2	1	1	1
Carbohydı	Organoox	Carbohydı	Carbohydı	Galactose	map00052	1	1	2.6	1	1	3	2	1	1	1	1	1
NA	NA	NA	NA	NA	NA	2	1	0.7	2	1	1	0	1	1	1	1	1
NA	Bi- and oli	Organohe	NA	NA	NA	1	4	0.9	1	8	6	25	1	1	1	2	1
NA	NA	NA	NA	NA	NA	1	1	0.6	1	1	1	1	0	40	1	1	7
Phenylbut	Benzene a	Amines an	NA	NA	NA	1	1	1.1	1	1	1	1	1	1	2	1	0
Benzothia	Thiadiazin	Organohe	NA	NA	NA	4	1	1.5	1	1	12	7	1	1	1	3	3
NA	NA	NA	NA	NA	NA	1	1	1.5	1	2	2	2	1	1	2	1	1
NA	NA	NA	NA	NA	NA	1	1	2.2	1	2	1	2	1	1	1	1	1
Pregnane	Steroids aı	Steroids aı	NA	NA	NA	0	1	0.3	0	1	11	1	1	1	2	4	87
NA	NA	NA	NA	NA	NA	2	1	0.5	1	1	1	1	1	3	0	25	1
NA	NA	NA	NA	NA	NA	1	1	0.4	3	0	1	0	1	0	2	1	1
Flavonoid	Flavonoids	Flavonoids	NA	NA	NA	1	1	1.3	1	1	2	1	5	1	1	1	2
NA	NA	NA	NA	NA	NA	1	1	0.8	1	1	1	1	4	0	3	1	0
NA	NA	NA	NA	NA	NA	1	1	1	2	0	1	0	1	0	2	1	0

NA	NA	NA	NA	NA	NA	1	1	1.8	1	4	2	2	1	1	1	1	1
NA	NA	NA	NA	NA	NA	5	3	0.6	6	0	40	0	0	0	0	6	7
NA	NA	NA	NA	NA	NA	1	1	0.9	2	1	3	0	1	2	2	1	2
NA	NA	NA	NA	NA	NA	1	2	1.8	1	2	1	2	1	2	1	1	1
NA	NA	NA	NA	NA	NA	1	1	1.8	1	0	1	1	1	1	1	1	1
NA	NA	NA	NA	NA	NA	1	1	1.7	1	2	1	2	1	1	1	2	1
NA	NA	NA	NA	NA	NA	2	2	0.4	1	0	0	1	1	4	1	0	43
NA	NA	NA	NA	NA	NA	2	1	0.8	1	1	1	1	1	1	1	1	1
NA	NA	NA	NA	NA	NA	0	0	0.3	1	0	0	0	9	5	3	0	0
NA	NA	NA	NA	NA	NA	1	1	1.4	1	2	1	1	1	1	1	2	1
NA	NA	NA	NA	NA	NA	0	1	0.5	1	1	1	1	2	1	1	1	1
NA	NA	NA	NA	NA	NA	1	2	2.3	1	2	1	2	1	1	1	2	0
Estrane ste	Steroids a	Steroids a	NA	NA	NA	1	1	0.9	2	1	1	0	1	1	1	1	1
NA	NA	NA	NA	NA	NA	1	1	1.9	1	2	1	2	1	1	1	2	1
NA	NA	NA	NA	NA	NA	1	1	3.3	0	4	8	6	1	2	2	2	0
Phenothia	Benzothia	Organohe	NA	NA	NA	1	1	0.9	1	1	1	0	1	1	1	1	1
Lineolic ac	Fatty Acyls	Fatty acyls	Lipid meta	Linoleic ac	map00591	1	1	1.8	6	4	1	3	1	1	1	2	0
Amines	Organonit	Amines	Lipid meta	Sphingolip	map0060	1	1	7.1	1	1	1	2	1	1	1	1	1
NA	Thienothia	Organohe	NA	NA	NA	1	1	1.7	1	2	1	2	1	1	1	2	1
NA	NA	NA	NA	NA	NA	1	2	1.7	1	2	1	1	1	2	1	1	1
NA	NA	NA	NA	NA	NA	1	1	1.1	1	2	1	1	1	1	1	1	1
						0	0	0	0	0	0	0	0	0	4	0	0
						0	0	0	0	0	0	0	0	0	0	0	0
NA	NA	NA	NA	NA	NA	2	0	1.2	1	1	1	1	2	1	0	0	1
NA	NA	NA	NA	NA	NA	0	2	0	2	7	0	8	2	0	2	3	0
Phenylace	Benzene a	Benzene a	NA	NA	NA	1	1	1.1	3	0	1	0	1	0	4	3	0
NA	NA	NA	NA	NA	NA	1	1	1	1	1	1	2	2	1	1	1	1
						0	0	0	0	0	0	0	0	0	0	0	0
						0	0	0	0	0	0	0	29	0	0	0	0
NA	NA	NA	NA	NA	NA	1	1	1.3	1	1	1	2	1	1	3	2	1
NA	NA	NA	NA	NA	NA	2	1	1.8	1	1	1	2	1	1	2	4	1
Isoflavono	Isoflavono	Flavonoids	NA	NA	NA	1	1	1.1	1	0	0	0	1	0	2	1	0
NA	NA	NA	NA	NA	NA	2	1	1.1	1	2	1	2	1	1	1	1	1
NA	NA	NA	NA	NA	NA	1	2	1.8	1	2	1	2	1	2	1	1	1
Isoflav-2-(	Isoflavono	Flavonoids	Biosynthes	Isoflavono	map0110	48	1	5.9	4	0	0	5	0	1	1	1	2
NA	NA	NA	NA	NA	NA	1	1	0.9	1	1	1	0	1	1	1	1	1
NA	NA	NA	NA	NA	NA	1	1	0.8	2	3	47	1	5	1	1	1	20
NA	NA	NA	NA	NA	NA	1	8	0.3	1	5	2	0	1	1	0	5	1
NA	NA	NA	NA	NA	NA	1	1	1.4	1	2	1	1	1	1	1	2	1
Alcohols a	Organoox	Alcohols	Metabolis	beta-Alan	map00410	1	2	0.5	1	0	1	1	2	0	1	1	1
NA	NA	NA	NA	NA	NA	1	1	1.2	1	1	1	1	1	1	2	1	1
Eicosanoic	Fatty Acyls	Fatty acyls	NA	NA	NA	1	2	1.8	1	2	1	2	1	2	1	1	1
Fatty acid	Fatty Acyls	Fatty acyls	NA	NA	NA	0	1	0.9	1	1	0	1	1	1	0	1	2
NA	NA	NA	NA	NA	NA	1	1	1.7	1	2	1	2	1	1	1	1	2
						##	##	354	##	85	##	##	##	##	##	##	##
NA	NA	NA	NA	NA	NA	2	1	1.7	1	2	9	2	1	1	1	2	11
NA	NA	NA	NA	NA	NA	1	1	1.1	1	##	1	2	1	1	1	1	19
FA0106 O:	FA01 Fatty	Fatty acyls	Biosynthes	Glucosinol	map0121	1	1	0.7	1	1	1	0	1	1	1	1	1
NA	NA	NA	NA	NA	NA	1	1	1.2	1	1	1	1	1	2	1	1	1
NA	NA	NA	NA	NA	NA	0	4	0.1	0	2	0	0	2	1	1	0	23
NA	NA	NA	NA	NA	NA	1	2	1.7	1	2	1	1	1	2	1	1	1
5'-deoxy-	5'-deoxyri	Nucleic ac	NA	NA	NA	1	27	0.6	1	1	1	1	1	1	1	1	1
NA	NA	NA	NA	NA	NA	1	1	1.8	1	2	1	2	1	2	1	1	23
NA	NA	NA	NA	NA	NA	1	0	0.2	5	1	1	0	1	0	2	1	5
NA	NA	NA	NA	NA	NA	1	1	2	1	2	1	2	1	1	1	2	1
Amino aci	Carboxylic	Amino aci	NA	NA	NA	1	0	1.6	8	0	1	0	2	0	1	2	1
NA	NA	NA	NA	NA	NA	1	1	0.8	1	0	1	0	2	1	1	2	1
NA	NA	NA	NA	NA	NA	1	1	1.3	1	2	1	1	3	3	1	1	9

NA	NA	NA	NA	NA	NA	1	1	1.1	13	55	1	36	1	1	1	1	3
NA	NA	NA	NA	NA	NA	1	1	0.8	1	3	4	5	1	1	1	1	3
NA	NA	NA	NA	NA	NA	2	1	1.1	1	1	3	2	1	1	1	2	2
NA	NA	NA	NA	NA	NA	1	1	0.6	1	0	1	1	1	7	1	1	1
Benzoic ac	Benzene a	Benzene a	NA	NA	NA	1	1	1.7	1	2	1	2	1	1	1	1	1
NA	NA	NA	NA	NA	NA	1	1	1.8	1	2	1	2	1	1	1	2	0
NA	NA	NA	NA	NA	NA	0	0	1.5	2	3	1	1	1	0	0	1	0
NA	NA	NA	NA	NA	NA	1	1	1.4	1	1	1	1	1	1	1	2	1
NA	NA	NA	Nucleotid	Purine me	map0023	1	1	1	2	0	1	1	1	2	1	1	2
NA	NA	NA	NA	NA	NA	1	1	2	1	2	1	2	1	1	1	1	1
Organosul	Organic su	Organic ac	NA	NA	NA	1	1	0.8	1	1	1	1	1	3	2	1	1
Purines an	Imidazopy	Purines an	NA	NA	NA	9	1	1.3	1	1	1	2	6	1	1	1	1
NA	NA	NA	NA	NA	NA	1	1	1.3	1	0	0	1	0	##	1	0	5
NA	NA	NA	NA	NA	NA	1	1	1.4	1	2	1	1	1	1	1	2	1
NA	NA	NA	NA	NA	NA	2	1	2.3	1	2	3	3	1	1	1	1	2
						0	0	0	0	0	2	0	4	0	0	0	0
NA	NA	NA	NA	NA	NA	1	1	0.5	2	1	1	0	1	0	1	0	2
NA	NA	NA	NA	NA	NA	1	1	1.8	1	2	2	2	1	1	1	1	1
Halogen h	Halogen o	Homogen	NA	NA	NA	1	2	0.7	1	18	1	15	1	4	1	4	0
NA	NA	NA	NA	NA	NA	1	1	2	1	1	1	2	1	1	1	1	1
NA	NA	NA	NA	NA	NA	1	1	0.9	1	1	1	1	1	2	2	1	1
						0	0	0	0	5	0	0	19	0	49	0	27
NA	NA	NA	NA	NA	NA	0	3	1.2	2	1	6	1	1	0	1	1	1
NA	NA	NA	NA	NA	NA	1	1	1.6	14	2	1	1	1	1	2	2	1
NA	NA	NA	NA	NA	NA	2	1	0.7	1	1	1	1	1	1	1	1	1
NA	NA	NA	NA	NA	NA	2	1	0.8	1	1	1	0	1	1	1	1	1
Amino sug	Monosacc	Carbohydr	Carbohydr	Amino sug	map0052	1	1	0.8	1	1	1	1	1	1	1	1	1
NA	NA	NA	NA	NA	NA	1	1	1.1	1	2	1	1	1	1	1	1	1
						0	0	0	0	##	0	2	0	0	11	0	0
NA	NA	NA	Biosynthes	Glucosinol	map0121	1	0	0.6	2	8	2	10	0	0	1	1	1
Amines	Organonit	Amines	NA	NA	NA	1	1	1.1	1	1	1	1	1	1	1	1	2
NA	NA	NA	NA	NA	NA	1	1	0.8	2	1	2	1	2	1	1	2	1
Medium-c	Hydroxy a	Organic ac	NA	NA	NA	0	2	3	1	20	1	12	1	2	1	2	1
NA	NA	NA	NA	NA	NA	0	1	1.2	1	0	3	0	1	8	1	0	1
						0	0	0	0	0	0	0	0	0	0	0	0
NA	NA	NA	NA	NA	NA	1	25	3.4	0	1	1	1	1	1	1	1	1
NA	NA	NA	NA	NA	NA	0	1	0.1	2	0	0	0	9	7	1	1	22
NA	NA	NA	NA	NA	NA	1	1	0.8	1	1	1	1	2	2	1	1	1
Diphenylm	Benzene a	Benzene a	NA	NA	NA	2	2	1.4	1	0	1	0	1	0	2	1	0
NA	NA	NA	NA	NA	NA	1	0	0.2	0	0	0	0	1	8	0	0	0
NA	NA	NA	NA	NA	NA	1	1	0.1	1	2	0	0	0	2	1	0	1
Quinoline	Alkaloids (	Alkaloids	NA	NA	NA	2	1	8.5	3	1	15	1	0	1	1	1	2
NA	NA	NA	NA	NA	NA	1	2	1	2	2	0	0	1	0	2	0	0
Carbohydr	Organoox	Carbohydr	NA	NA	NA	1	1	1.6	1	1	1	2	1	1	1	1	1
NA	NA	NA	NA	NA	NA	57	1	1.3	1	1	1	1	1	1	1	1	1
NA	NA	NA	NA	NA	NA	2	1	0.7	1	1	1	0	1	0	1	1	11
NA	NA	NA	NA	NA	NA	1	2	3.3	0	1	11	1	2	1	1	2	2
Amino aci	Carboxylic	Amino aci	NA	NA	NA	1	1	1.2	1	1	1	1	1	1	1	1	1
NA	NA	NA	NA	NA	NA	1	1	0.3	3	1	0	1	2	1	1	0	2
NA	NA	NA	NA	NA	NA	1	1	0.9	1	1	1	0	1	1	1	1	22
NA	NA	NA	NA	NA	NA	0	1	7.3	0	5	1	6	1	1	2	4	1
NA	NA	NA	Metabolis	Biosynthes	NA	3	2	0.7	1	1	1	1	1	1	1	1	1
NA	NA	NA	NA	NA	NA	1	1	0.3	1	1	1	1	1	1	2	2	1
NA	NA	NA	NA	NA	NA	1	1	1.4	1	2	1	1	1	1	1	2	1
NA	NA	NA	NA	NA	NA	1	1	1.7	1	2	1	2	1	1	1	2	1
NA	NA	NA	NA	NA	NA	1	1	1.7	2	2	0	2	1	1	1	14	0
NA	NA	NA	NA	NA	NA	1	1	0.4	1	0	0	0	1	1	2	1	1
NA	NA	NA	NA	NA	NA	1	1	1.4	1	2	1	1	1	1	1	2	1

Isoflavono	Isoflavono	Flavonoids	NA	NA	NA	3	1	1.3	1	2	1	1	1	1	1	2	1
NA	NA	NA	NA	NA	NA	1	2	1	1	1	1	6	1	0	1	2	2
NA	NA	NA	NA	NA	NA	1	2	1.7	1	2	10	2	1	2	1	2	0
NA	NA	NA	NA	NA	NA	1	2	1.5	1	0	3	1	1	1	2	5	0
NA	NA	NA	NA	NA	NA	1	1	0.8	1	1	1	0	1	1	1	1	0
NA	NA	NA	NA	NA	NA	1	1	1	1	1	1	2	1	1	1	1	1
NA	Phenylpro	Phenylpro	NA	NA	NA	0	4	3.1	1	2	4	3	0	1	1	2	2
Quinoline	Quinolines	Organohe	Amino aci	Tryptopha	map0038C	1	4	0.1	4	0	3	0	4	1	1	1	1
NA	NA	NA	NA	NA	NA	1	1	0.9	1	1	1	0	1	1	1	1	1
NA	NA	NA	NA	NA	NA	0	0	0.3	1	0	1	2	1	2	0	0	2
NA	NA	NA	NA	NA	NA	1	1	0.8	2	1	1	0	1	1	1	1	1
NA	NA	NA	NA	NA	NA	1	1	0.9	1	1	1	0	1	1	1	1	1
NA	NA	NA	NA	NA	NA	2	1	0.3	1	1	13	1	1	1	2	1	1
NA	NA	NA	NA	NA	NA	1	1	1.4	1	2	1	1	1	1	1	2	14
NA	NA	NA	NA	NA	NA	1	1	1.7	1	2	1	2	1	1	1	2	1
						0	0	0	0	0	0	0	0	0	0	0	0
NA	NA	NA	NA	NA	NA	2	1	1	1	1	2	2	1	3	0	1	1
NA	NA	NA	NA	NA	NA	1	1	0.7	1	1	1	1	1	0	1	2	1
NA	NA	NA	NA	NA	NA	1	1	0.7	1	1	1	0	1	1	1	1	1
NA	NA	NA	NA	NA	NA	1	1	1	1	2	1	2	2	1	1	1	1
Glyceroph	Glyceroph	Glyceroph	NA	NA	NA	0	1	0.1	0	2	0	3	3	1	1	0	8
NA	NA	NA	NA	NA	NA	1	1	1	1	2	1	2	1	1	1	1	1
NA	NA	NA	NA	NA	NA	1	1	1.3	1	4	1	6	1	1	1	1	1
PR0102 C1	PR01 Isopr	Prenol lipi	NA	NA	NA	1	1	1.1	1	2	1	2	0	1	0	1	2
NA	NA	NA	NA	NA	NA	1	0	1.2	5	14	0	30	0	2	38	1	29
						0	0	0	0	0	0	0	0	0	0	0	0
						0	0	0	0	0	0	0	0	0	0	0	0
						0	0	0	0	0	0	0	0	0	0	0	0
						0	0	0	0	0	0	0	0	0	0	0	0
						0	0	0	0	0	0	0	0	0	0	0	0
						0	0	0	0	0	0	0	0	0	0	0	0
						0	0	0	0	0	0	0	0	0	0	0	0
						0	0	0	0	0	0	0	0	0	0	0	0
						0	0	0	0	0	0	0	0	0	0	0	0
						0	0	0	0	0	0	0	0	0	0	0	0
						0	0	0	0	0	0	0	0	0	0	0	0
						0	0	0	0	0	0	0	0	0	0	0	0
						0	0	0	0	0	0	0	0	0	0	0	0
						0	0	0	0	0	0	0	0	0	0	0	0
						0	0	0	0	0	0	0	0	0	0	0	0
						0	0	0	0	0	0	0	0	0	0	0	0
						0	0	0	0	0	0	0	0	0	0	0	0
						0	0	0	0	0	0	0	0	0	0	0	0
						0	0	0	0	0	0	0	0	0	0	0	0
						0	0	0	0	0	0	0	0	0	0	0	0
						0	0	0	0	0	0	0	0	0	0	0	0
						0	0	0	0	0	0	0	0	0	0	0	0
						0	0	0	0	0	0	0	0	0	0	0	0
						0	0	0	0	0	0	0	0	0	0	0	0
						0	0	0	0	0	0	0	0	0	0	0	0
						0	0	0	0	0	0	0	0	0	0	0	0
						0	0	0	0	0	0	0	0	0	0	0	0
						0	0	0	0	0	0	0	0	0	0	0	0
						0	0	0	0	0	0	0	0	0	0	0	0
						0	0	0	0	0	0	0	0	0	0	0	0
						0	0	0	0	0	0	0	0	0	0	0	0
						0	0	0	0	0	0	0	0	0	0	0	0
						0	0	0	0	0	0	0	0	0	0	0	0

						0	0	0	0	0	0	0	0	0	0	0	0
						0	0	0	0	0	0	0	0	0	0	0	0
						0	0	0	0	0	0	0	0	0	0	0	0
						0	0	0	0	0	0	0	0	0	0	0	0
						0	0	0	0	0	0	0	0	0	0	0	0
						0	0	0	0	0	0	0	0	0	0	0	0
						0	0	0	0	0	0	0	0	0	0	0	0
						0	0	0	0	0	0	0	0	0	0	0	0
						0	0	0	0	0	0	0	0	0	0	0	0
						0	0	0	0	0	0	0	0	0	0	0	0
						0	0	0	0	0	0	0	0	0	0	0	0
						0	0	0	0	0	0	0	0	0	0	0	0
						0	0	0	0	0	0	0	0	0	0	0	0
						0	0	0	0	0	0	0	0	0	0	0	0
						0	0	0	0	0	0	0	0	0	0	0	0
						0	0	0	0	0	0	0	0	0	0	0	0
						0	0	0	0	0	0	0	0	0	0	0	0
						0	0	0	0	0	0	0	0	0	0	0	0
						0	0	0	0	0	0	0	0	0	0	0	0
						0	0	0	0	0	0	0	0	0	0	0	0
						0	0	0	0	0	0	0	0	0	0	0	0
						0	0	0	0	0	0	0	0	0	0	0	0
						0	0	0	0	0	0	0	0	0	0	0	0
						0	0	0	0	0	0	0	0	0	0	0	0
						0	0	0	0	0	0	0	0	0	0	0	0
						0	0	0	0	0	0	0	0	0	0	0	0
						0	0	0	0	0	0	0	0	0	0	0	0
Amino aci	Carboxylic	Amino aci	NA	NA	NA	0	1	1	2	0	3	0	1	0	0	1	0
NA	NA	NA	NA	NA	NA	1	1	0.9	1	2	1	1	1	1	1	1	1
NA	NA	NA	NA	NA	NA	1	1	1.2	0	3	4	5	1	2	0	2	0
NA	NA	NA	NA	NA	NA	1	1	0.8	1	0	1	0	2	1	1	2	1
NA	NA	NA	NA	NA	NA	1	1	1.6	1	1	1	2	3	1	1	1	1
NA	NA	NA	NA	NA	NA	1	1	6.9	1	2	15	6	1	1	0	1	0
NA	NA	NA	NA	NA	NA	1	6	1.2	0	1	1	1	1	1	1	1	0
NA	NA	NA	NA	NA	NA	1	1	1	1	1	1	3	1	1	1	1	1
NA	NA	NA	NA	NA	NA	1	1	1.4	1	1	1	9	1	1	1	2	1
NA	NA	NA	NA	NA	NA	1	2	0.8	1	1	4	1	1	1	1	1	3
NA	NA	NA	NA	NA	NA	1	0	0.3	1	0	0	0	1	17	2	2	12
NA	NA	NA	NA	NA	NA	1	0	2	1	2	0	2	1	1	1	2	1
NA	Purine nuc	Purines an	Nucleotid	Purine me	map00230	3	1	1	1	1	2	5	1	0	0	1	0
						0	0	0	0	0	0	##	80	3	0	0	0
NA	NA	NA	NA	NA	NA	1	1	1.3	1	1	1	1	1	1	1	1	1
NA	NA	NA	NA	NA	NA	1	1	0.9	1	1	1	2	1	1	1	1	1
NA	NA	NA	NA	NA	NA	1	1	0.9	1	0	1	0	1	1	1	2	0
NA	NA	NA	NA	NA	NA	1	1	0.9	1	1	1	1	1	1	2	1	1
NA	NA	NA	NA	NA	NA	1	1	1.3	1	1	1	1	1	1	1	1	1
NA	NA	NA	NA	NA	NA	1	2	1.5	1	1	1	1	1	2	1	1	1
NA	NA	NA	NA	NA	NA	1	1	0.9	1	1	1	0	1	1	1	1	1
						0	0	0	0	0	0	0	0	0	0	0	0
NA	NA	NA	NA	NA	NA	1	1	0.6	1	1	1	1	0	1	1	1	16
NA	NA	NA	NA	NA	NA	1	1	1	1	1	1	0	1	1	1	1	1
NA	NA	NA	NA	NA	NA	0	0	0.1	1	26	0	11	5	7	0	0	0
NA	NA	NA	NA	NA	NA	1	1	0.8	3	0	0	0	1	0	2	0	0
NA	NA	NA	NA	NA	NA	1	1	2.1	1	2	1	2	1	1	1	2	1
NA	NA	NA	NA	NA	NA	1	1	0.9	1	0	1	0	1	1	1	2	1
NA	NA	NA	NA	NA	NA	1	1	0.3	3	1	0	0	1	0	1	0	1
Benzenedi	Phenols	Phenols ar	NA	NA	NA	1	1	0.7	2	1	1	0	1	1	1	1	0
Amino aci	Carboxylic	Amino aci	NA	NA	NA	2	1	0.6	2	0	0	1	1	1	1	1	4

NA	NA	NA	NA	NA	NA	2	1	0.6	0	3	2	1	1	1	0	0	1
NA	NA	NA	NA	NA	NA	1	10	0.4	0	0	4	0	2	0	1	0	2
						0	0	0	0	0	0	0	0	0	0	0	0
NA	NA	NA	NA	NA	NA	1	1	1.3	2	1	0	1	3	0	1	0	5
NA	NA	NA	NA	NA	NA	1	1	1.9	1	1	1	1	2	1	1	1	1
NA	NA	NA	NA	NA	NA	1	1	0.9	1	1	1	1	1	1	1	2	1
NA	NA	NA	NA	NA	NA	1	1	1.3	1	2	1	1	1	1	1	2	1
NA	NA	NA	NA	NA	NA	1	1	2.4	2	1	1	1	1	1	0	1	1
NA	NA	NA	NA	NA	NA	1	2	0.7	10	1	0	0	0	0	2	0	0
Substitute	Pyrroles	Organohe	NA	NA	NA	1	2	1.5	1	1	1	1	1	2	1	2	1
NA	NA	NA	NA	NA	NA	1	1	0.3	1	0	1	0	1	1	1	0	0
NA	NA	NA	NA	NA	NA	1	1	2.2	1	1	3	1	2	1	1	3	0
NA	NA	NA	NA	NA	NA	1	3	0.1	1	##	1	68	0	0	1	0	1
NA	NA	NA	NA	NA	NA	1	1	1.6	1	2	1	1	1	1	1	1	1
Isoquinoli	Alkaloids a	Alkaloids	NA	NA	NA	0	1	0.2	2	0	0	0	3	2	1	2	6
NA	NA	NA	NA	NA	NA	1	1	10	5	6	1	6	1	1	1	3	1
Anthraqui	Anthraqui	Quinone	NA	NA	NA	1	1	0.8	1	0	1	0	2	1	1	2	1
NA	NA	NA	NA	NA	NA	1	1	1.3	1	2	1	1	1	1	1	2	1
NA	NA	NA	NA	NA	NA	1	1	1.3	1	2	1	1	1	1	1	2	1
NA	NA	NA	NA	NA	NA	1	1	0.9	2	1	0	1	3	1	1	1	0
NA	NA	NA	NA	NA	NA	1	1	0.6	1	1	1	1	1	4	0	1	1
						0	0	0	0	0	0	0	13	0	0	0	0
NA	NA	NA	NA	NA	NA	1	1	0.7	1	1	0	1	1	0	3	0	2
NA	NA	NA	NA	NA	NA	1	1	0.9	1	1	1	1	1	1	1	1	1
NA	NA	NA	NA	NA	NA	1	1	0.6	1	4	1	1	0	4	1	2	1
NA	NA	NA	NA	NA	NA	3	4	1.6	0	4	1	0	0	1	1	0	1
NA	NA	NA	NA	NA	NA	1	1	1.1	1	1	1	1	1	1	1	1	1
NA	NA	NA	NA	NA	NA	1	1	2	1	2	1	2	1	1	1	2	1
NA	NA	NA	NA	NA	NA	1	25	1.3	1	2	1	3	1	1	1	1	1
NA	NA	NA	NA	NA	NA	1	1	0.9	1	1	1	0	1	1	1	1	1
NA	NA	NA	NA	NA	NA	1	7	0.5	1	1	1	1	4	1	1	1	1
NA	NA	NA	NA	NA	NA	1	0	2.3	1	2	0	1	1	1	1	2	1
NA	NA	NA	NA	NA	NA	0	0	23	0	31	##	3	1	0	0	0	17
Morpholin	Oxazinane	Organohe	NA	NA	NA	1	1	1.5	1	2	1	2	1	1	1	1	1
NA	NA	NA	NA	NA	NA	1	1	0.6	5	0	2	1	2	0	0	2	0
NA	NA	NA	NA	NA	NA	1	1	1.7	1	1	1	1	1	1	2	1	1
NA	NA	NA	NA	NA	NA	0	2	2.7	0	2	7	2	1	1	1	4	2
NA	NA	NA	NA	NA	NA	1	1	1.3	1	2	2	2	1	2	1	1	1
NA	NA	NA	NA	NA	NA	1	0	1.6	5	0	1	0	1	0	11	24	0
NA	NA	NA	NA	NA	NA	6	3	0.2	22	4	9	1	0	1	0	1	2
NA	NA	NA	NA	NA	NA	3	1	1.1	1	2	1	1	1	2	1	1	1
NA	NA	NA	NA	NA	NA	1	1	0.9	1	3	1	6	1	1	1	1	1
NA	NA	NA	NA	NA	NA	1	1	2.2	1	2	1	2	1	1	1	1	1
						0	19	0	19	26	21	0	0	36	##	22	0
Hydroxyst	Steroids a	Steroids a	NA	NA	NA	0	0	39	1	5	11	1	1	0	1	0	2
NA	NA	NA	NA	NA	NA	1	1	1.8	1	2	1	2	1	1	1	1	1
Carboxylic	Carboxylic	Organic ac	NA	NA	NA	1	1	0.9	2	1	1	1	2	1	1	2	1
NA	NA	NA	NA	NA	NA	1	1	0.9	2	0	0	0	1	1	2	1	0
Isoquinoli	Alkaloids a	Alkaloids	NA	NA	NA	1	1	0.9	2	1	1	1	2	1	1	2	1
NA	NA	NA	NA	NA	NA	1	1	2.5	2	2	1	2	1	1	2	2	0
NA	NA	NA	NA	NA	NA	1	1	0.7	1	1	1	1	1	1	1	1	2
NA	NA	NA	NA	NA	NA	0	1	0.6	1	1	7	2	1	2	1	1	3
NA	NA	NA	NA	NA	NA	1	1	1.3	1	2	1	2	0	1	1	1	2
NA	NA	NA	NA	NA	NA	1	0	1.2	2	0	0	0	1	1	2	0	0
NA	NA	NA	NA	NA	NA	3	7	0.8	0	1	##	1	1	1	0	1	8
Amino aci	Carboxylic	Amino aci	NA	NA	NA	25	1	3.4	0	1	4	1	1	1	1	1	3
NA	NA	NA	NA	NA	NA	2	1	0.6	1	1	1	1	1	3	1	2	1
Pyrroloind	Alkaloids a	Alkaloids	NA	NA	NA	3	1	0.8	3	1	10	1	0	1	1	0	3

NA	NA	NA	NA	NA	NA	1	1	0.8	1	0	1	0	1	5	2	1	0
Vitamin D	Steroids ar	Vitamins a	NA	NA	NA	0	1	0.4	1	0	0	0	4	0	6	2	3
NA	NA	NA	NA	NA	NA	1	1	0.9	2	1	1	0	1	1	1	1	1
NA	NA	NA	NA	NA	NA	1	1	0.8	1	1	1	2	3	1	1	1	11
NA	NA	NA	NA	NA	NA	1	1	1.9	1	2	1	2	1	1	1	2	1
NA	NA	NA	NA	NA	NA	1	1	0.9	1	1	1	2	1	1	1	1	1
NA	NA	NA	NA	NA	NA	2	1	0.8	4	2	0	0	0	0	2	0	0
Lineolic ac	Fatty Acyls	Fatty acyls	NA	NA	NA	0	0	0.3	1	17	0	11	19	9	2	0	1
NA	NA	NA	NA	NA	NA	1	1	1.3	1	1	1	1	1	2	1	2	1
NA	NA	NA	NA	NA	NA	1	1	1.9	1	2	1	2	1	1	1	2	1
NA	NA	NA	NA	NA	NA	1	1	2	1	2	1	2	1	1	1	2	1
NA	Phenanthr	Benzene a	Xenobiotic	Metabolism	map0098C	1	1	1.7	1	2	1	2	1	1	1	1	15
NA	Phenylpro	Phenylpro	NA	NA	NA	1	2	1.5	1	2	3	6	1	1	0	2	0
O-methyla	Flavonoids	Flavonoids	NA	NA	NA	1	1	1.3	1	1	1	1	1	1	1	4	1
Pyrimidine	Diazines	Organohe	NA	NA	NA	1	1	1.2	3	1	1	0	1	0	2	0	0
NA	NA	NA	NA	NA	NA	2	2	0.5	1	1	1	1	1	1	1	1	1
NA	NA	NA	NA	NA	NA	1	1	1.1	1	9	1	2	1	1	1	1	1
						0	0	0	0	31	0	0	17	4	0	16	0
NA	NA	NA	NA	NA	NA	1	1	0.7	2	1	0	0	1	1	2	1	0
1-hydroxy	Phenols	Phenols ar	NA	NA	NA	1	1	1.4	2	1	1	1	1	1	1	2	0
Arylsulfate	Organic su	Organic ac	NA	NA	NA	1	1	1.1	1	1	1	2	1	1	1	1	1
NA	NA	NA	NA	NA	NA	1	1	13	1	1	##	2	1	1	1	1	3
NA	NA	NA	NA	NA	NA	1	1	3.9	1	2	1	2	1	2	7	2	0
NA	NA	NA	NA	NA	NA	1	1	1.9	1	2	1	2	1	1	1	2	1
NA	NA	NA	NA	NA	NA	1	1	1.9	1	2	1	2	1	1	1	2	1
NA	NA	NA	NA	NA	NA	1	1	1.9	1	2	1	2	1	1	1	2	1
NA	NA	NA	NA	NA	NA	1	1	1.9	1	2	1	2	1	1	1	2	1
NA	NA	NA	NA	NA	NA	1	1	1.9	1	2	1	2	1	1	1	2	1
Phenothia	Benzothia:	Organohe	NA	NA	NA	1	1	2	1	2	1	2	1	1	1	2	1
NA	NA	NA	NA	NA	NA	1	0	10	1	5	1	9	0	1	1	3	0
NA	NA	NA	NA	NA	NA	1	1	0.9	1	1	1	1	1	1	1	1	1
Halobenze	Benzene a	Benzene a	NA	NA	NA	1	0	0.5	6	0	1	0	3	1	4	1	1
NA	NA	NA	NA	NA	NA	1	1	1.3	1	1	1	1	1	1	1	1	1
NA	NA	NA	NA	NA	NA	3	3	0.2	0	0	2	0	2	0	2	1	0
Isoquinolin	Alkaloids (	Alkaloids	NA	NA	NA	1	1	0.8	1	1	1	2	1	1	1	1	1
NA	NA	NA	NA	NA	NA	1	2	2.3	2	2	0	0	0	1	2	0	0
NA	NA	NA	NA	NA	NA	1	1	0.9	1	2	1	1	1	1	1	1	1
Quinoline	Alkaloids (	Alkaloids	Chemical s	Biosynthes	NA	0	3	0.4	1	0	1	4	1	2	1	7	5
NA	NA	NA	NA	NA	NA	0	0	1.1	1	0	1	0	1	13	2	3	0
Phenothia	Benzothia:	Organohe	NA	NA	NA	1	1	0.9	0	2	23	2	3	1	1	1	0
NA	NA	NA	NA	NA	NA	1	1	1.6	1	2	1	2	1	1	1	1	1
NA	NA	NA	NA	NA	NA	1	1	1.9	4	1	1	1	0	2	1	1	3
Cholestan	Steroids ar	Steroids ar	NA	NA	NA	1	1	1.8	1	2	13	2	1	1	1	2	0
NA	Purine nuc	Purines an	NA	NA	NA	1	3	0.4	2	0	1	1	2	0	1	1	7
NA	NA	NA	NA	NA	NA	1	1	1	1	1	1	1	2	2	1	1	1
Pyrrolizidin	Alkaloids (	Alkaloids	NA	NA	NA	1	1	1.2	3	0	1	0	1	0	2	1	0
NA	Oxepanes	Organohe	NA	NA	NA	1	4	1.7	1	1	2	1	1	1	1	1	1
NA	NA	NA	NA	NA	NA	1	1	1.5	1	2	2	2	1	1	1	2	1
NA	NA	NA	NA	NA	NA	1	1	0.7	1	1	1	1	2	1	1	1	2
NA	NA	NA	NA	NA	NA	1	1	1.1	1	1	1	1	1	1	1	1	1
NA	NA	NA	NA	NA	NA	2	1	0.9	1	1	1	1	1	7	1	1	1
NA	NA	NA	NA	NA	NA	1	1	0.6	3	0	2	0	2	0	2	1	0
NA	NA	NA	NA	NA	NA	1	1	0.6	1	1	1	1	1	2	1	2	1
Benzoic ac	Benzene a	Benzene a	NA	NA	NA	1	1	0.9	1	1	1	0	1	1	1	1	1
NA	NA	NA	NA	NA	NA	1	2	1.4	1	1	1	1	1	2	1	2	2
NA	Morphinan	Alkaloids a	NA	NA	NA	1	1	1.4	2	1	1	0	1	1	2	2	1
NA	NA	NA	NA	NA	NA	2	1	0.6	1	1	1	1	0	3	1	2	1
NA	NA	NA	NA	NA	NA	1	1	2.6	1	0	1	0	2	0	2	9	0

NA	NA	NA	NA	NA	NA	0	3	0.8	1	1	0	1	19	13	3	1	18
NA	NA	NA	Carbohydr	Amino sug	map0052C	1	1	1.8	1	2	1	2	1	1	1	1	1
NA	NA	NA	NA	NA	NA	2	1	1	2	1	1	1	0	2	0	1	1
Benzenesu	Benzene a	Benzene a	NA	NA	NA	1	1	0.9	1	0	1	0	1	1	1	2	0
NA	NA	NA	NA	NA	NA	1	1	0.9	2	1	1	1	2	1	1	2	1
NA	NA	NA	NA	NA	NA	1	1	1	1	1	1	1	1	1	1	1	1
NA	NA	NA	NA	NA	NA	1	1	1.4	1	2	1	1	1	1	1	2	1
NA	NA	NA	NA	NA	NA	1	1	1.2	1	1	1	1	1	1	1	1	1
NA	NA	NA	Biosynthes	Biosynthes	NA	1	1	0.8	1	1	1	0	1	1	1	1	1
Gamma-k	Keto acids	Organic ac	Metabolisr	Nicotinate	map00760	2	1	0.6	5	0	1	0	1	0	5	1	0
Benzoic ac	Benzene a	Benzene a	NA	NA	NA	2	1	1.6	2	1	1	0	1	0	1	3	0
NA	Tetrahydro	Organohe	NA	NA	NA	1	1	1.5	2	1	1	1	1	2	1	6	1
NA	NA	NA	NA	NA	NA	1	1	1.2	1	1	1	0	1	1	1	2	1
NA	NA	NA	NA	NA	NA	1	1	0.8	2	1	1	0	1	1	1	1	1
Purines an	Imidazopy	Purines an	Metabolisr	Biosynthes	map0110(	1	1	0.9	2	1	1	0	1	1	1	1	1
NA	NA	NA	NA	NA	NA	1	1	2	1	2	1	2	1	1	1	2	1
NA	NA	NA	NA	NA	NA	1	1	1.2	1	1	5	1	2	1	1	1	1
NA	NA	NA	NA	NA	NA	1	1	1.4	1	2	1	1	1	1	1	2	1
NA	NA	NA	Xenobiotic	Dioxin deg	map0110(	1	1	1.9	1	2	1	2	1	1	1	2	1
NA	NA	NA	NA	NA	NA	1	1	0.9	1	1	2	1	1	1	2	1	1
NA	NA	NA	NA	NA	NA	1	1	1.2	2	1	1	0	1	0	2	1	0
NA	NA	NA	NA	NA	NA	1	1	2.3	1	1	1	2	1	1	1	2	0
NA	NA	NA	NA	NA	NA	15	1	0.9	1	1	1	1	1	2	1	1	1
NA	NA	NA	NA	NA	NA	1	1	1.4	1	1	1	1	1	1	1	2	1
Hydroxyin	Indoles an	Indole anc	Amino aci	Tryptopha	map00380	2	1	0.4	3	0	0	0	0	0	2	0	0
NA	NA	NA	NA	NA	NA	1	1	1	1	1	1	1	1	1	1	1	##
NA	NA	NA	NA	NA	NA	1	1	1.9	1	2	1	2	1	1	1	2	1
Aflatoxins	Mycotoxin	Fungal tox	Xenobiotic	Metabolisr	map0098C	1	1	2	1	2	1	2	1	1	1	2	1
NA	NA	NA	NA	NA	NA	1	1	1.1	12	1	1	27	1	1	1	1	21
NA	Stilbenes	Polyketide	NA	NA	NA	1	1	1.4	1	2	1	1	1	1	1	1	1
NA	NA	NA	NA	NA	NA	1	1	0.9	1	1	1	1	1	1	1	1	1
NA	NA	NA	NA	NA	NA	1	1	0.4	8	2	2	6	7	1	1	1	7
NA	NA	NA	NA	NA	NA	1	1	2.4	1	2	3	2	1	1	1	1	1
						0	0	0	0	6	0	0	0	0	8	0	0
Amino aci	Carboxylic	Amino aci	NA	NA	NA	1	1	1.1	1	1	8	1	1	1	2	3	1
1-benzopy	Benzopyra	Organohe	NA	NA	NA	1	1	0.8	2	1	1	0	1	1	1	1	1
NA	NA	NA	NA	NA	NA	1	1	1.9	1	2	1	2	1	1	1	2	1
						0	0	0	23	78	6	46	##	18	0	0	44
NA	NA	NA	NA	NA	NA	1	1	1.8	1	2	1	2	1	1	1	1	1
NA	NA	NA	NA	NA	NA	0	3	3.6	2	14	1	15	4	1	3	1	1
NA	NA	NA	NA	NA	NA	1	1	1.2	1	1	1	1	1	1	1	1	4
Tryptamin	Indoles an	Amines an	NA	NA	NA	1	1	0.7	1	1	1	1	1	2	1	1	1
NA	NA	NA	NA	NA	NA	2	0	0.6	1	1	1	0	2	1	2	1	13
NA	Stilbenes	Polyketide	NA	NA	NA	1	1	1	2	1	1	1	2	1	1	1	12
Pyrimidine	Diazines	Organohe	NA	NA	NA	2	0	1.6	2	1	3	1	1	0	1	4	0
NA	NA	NA	NA	NA	NA	1	1	0.9	1	1	1	0	1	1	2	1	1
NA	NA	NA	NA	NA	NA	4	0	0.8	1	0	1	1	1	16	1	1	0
NA	NA	NA	NA	NA	NA	0	1	0.2	2	0	0	1	10	7	1	1	21
NA	NA	NA	NA	NA	NA	1	1	0.7	1	0	1	1	1	2	1	1	1
NA	NA	NA	NA	NA	NA	1	1	0.7	1	1	1	1	2	1	1	1	2
NA	NA	NA	NA	NA	NA	2	1	0.7	1	1	1	1	1	0	1	1	1
Stilbene gl	Stilbenes	Polyketide	NA	NA	NA	1	1	0.9	1	1	1	0	1	1	1	1	1
NA	NA	NA	NA	NA	NA	2	1	1.4	1	1	3	2	1	1	1	2	6
NA	NA	NA	NA	NA	NA	1	1	1	1	1	1	1	1	1	1	1	1
						0	0	0	0	0	0	0	0	0	0	0	0
						0	0	0	0	0	0	0	0	0	0	0	0
						0	0	0	0	0	0	0	0	0	0	0	0
						0	0	0	0	0	0	0	0	0	0	0	0

						0	0	0	0	0	0	0	0	0	0	0	0
NA	NA	NA	NA	NA	NA	1	1	1.5	1	1	1	2	1	1	1	1	1
NA	NA	NA	NA	NA	NA	1	1	1.1	1	2	1	1	19	1	1	2	1
NA	NA	NA	NA	NA	NA	2	2	0.8	1	1	1	1	2	1	1	1	9
NA	NA	NA	NA	NA	NA	0	0	1	1	1	1	2	1	7	1	1	0
NA	NA	NA	NA	NA	NA	1	1	1.5	1	2	1	1	1	1	1	1	1
NA	NA	NA	NA	NA	NA	0	0	0.3	0	3	21	1	1	9	0	1	12
NA	NA	NA	NA	NA	NA	2	1	1.4	1	2	1	2	3	1	1	1	1
NA	NA	NA	NA	NA	NA	2	1	1.1	3	0	0	0	2	0	2	1	0
NA	NA	NA	NA	NA	NA	1	1	1	1	1	1	2	1	1	1	1	2
NA	NA	NA	NA	NA	NA	1	1	0.2	2	2	0	0	0	1	1	0	1
NA	NA	NA	NA	NA	NA	1	1	1.7	1	2	2	2	1	2	1	1	0
Isobenzof	Isocoumar	Organohe	NA	NA	NA	2	2	0.6	2	1	1	1	1	1	0	2	1
NA	NA	NA	NA	NA	NA	1	1	0.9	1	3	1	3	0	1	1	1	1
NA	NA	NA	NA	NA	NA	1	1	0.7	3	0	1	0	1	2	3	1	1
						0	0	0	0	0	0	0	0	0	0	0	0
Dicarboxyl	Carboxylic	Organic ac	Lipid meta	Fatty acid	map00071	1	1	0.8	2	0	1	0	1	1	2	1	0
NA	NA	NA	NA	NA	NA	1	1	0.8	2	0	1	0	1	1	2	1	0
NA	NA	NA	NA	NA	NA	1	1	1.7	1	2	1	2	1	1	1	2	1
NA	NA	NA	NA	NA	NA	1	1	0.9	2	1	1	1	2	1	1	2	1
NA	NA	NA	NA	NA	NA	1	1	1.3	1	1	2	2	1	1	1	1	1
NA	NA	NA	NA	NA	NA	2	1	0.8	1	1	1	0	1	1	1	1	1
NA	NA	NA	NA	NA	NA	1	1	0.7	1	2	0	3	1	2	7	2	5
NA	NA	NA	NA	NA	NA	0	3	0.2	0	3	0	0	2	2	1	0	3
NA	NA	NA	NA	NA	NA	2	1	1	2	3	1	3	1	8	4	1	1
NA	NA	NA	NA	NA	NA	1	1	0.7	1	0	1	0	1	7	1	1	1
NA	NA	NA	NA	NA	NA	1	1	1.1	1	1	1	1	2	1	1	1	1
NA	NA	NA	NA	NA	NA	5	1	1	2	6	##	13	0	1	0	6	0
Estrane ste	Steroids a	Steroids a	NA	NA	NA	5	1	1	2	6	##	13	0	1	0	6	0
						0	0	42	0	0	0	0	0	0	0	0	38
NA	NA	NA	NA	NA	NA	1	1	0.5	0	3	17	6	2	2	1	0	4
Indoles	Indoles an	Indoles	NA	NA	NA	1	1	0.6	2	0	0	0	0	1	5	1	0
NA	NA	NA	NA	NA	NA	1	1	0.9	1	1	1	1	1	1	1	1	1
						0	0	0	0	##	0	0	11	15	80	0	0
Cresols	Phenols	Phenols ar	Xenobiotic	Toluene d	map0110	0	1	0.1	2	6	0	6	2	0	2	2	0
NA	NA	NA	NA	NA	NA	1	1	0.9	1	1	1	1	1	1	1	1	1
NA	NA	NA	NA	NA	NA	1	1	1.5	1	2	1	2	1	1	1	1	2
NA	NA	NA	NA	NA	NA	0	1	1	2	0	1	0	1	0	1	0	1
NA	NA	NA	NA	NA	NA	2	1	0.7	1	1	1	0	2	0	1	1	1
NA	Cinnamic a	Polyketide	NA	NA	NA	1	0	0.4	2	0	0	0	0	18	1	2	1
Glyceroph	Glyceroph	Glyceroph	NA	NA	NA	1	1	0.4	2	7	0	7	1	0	0	0	5
NA	NA	NA	Metabolis	Biosynthes	NA	1	1	0.5	1	0	2	0	2	0	2	1	1
NA	NA	NA	NA	NA	NA	1	1	0.9	2	1	1	0	2	1	1	2	1
						0	0	0	0	0	0	0	0	0	0	0	0
Diphenylm	Benzene a	Benzene a	NA	NA	NA	1	1	0.9	1	0	1	0	1	1	1	2	0
NA	NA	NA	NA	NA	NA	1	1	1.1	1	1	1	1	1	1	1	1	1
						0	0	0	0	0	0	0	22	0	0	0	0
NA	NA	NA	NA	NA	NA	1	1	1.8	1	2	2	1	1	1	1	1	1
NA	Benzothia	Organohe	NA	NA	NA	1	1	1	2	1	1	1	1	0	2	3	1
NA	NA	NA	NA	NA	NA	1	1	1	1	1	1	1	1	1	2	1	1
NA	NA	NA	NA	NA	NA	1	1	0.8	1	0	1	0	2	2	1	2	1
NA	NA	NA	Metabolis	Biosynthes	NA	1	1	0.2	5	0	2	0	1	0	2	2	1
Amino aci	Carboxylic	Amino aci	NA	NA	NA	1	1	0.8	2	1	1	0	1	1	1	1	1
						0	0	0	0	0	0	##	49	13	0	0	0
NA	NA	NA	NA	NA	NA	1	1	1.3	1	2	1	2	1	1	1	1	1
Ureas	Organic ca	Organic ac	NA	NA	NA	1	1	1	1	1	1	1	1	1	1	2	2
NA	NA	NA	Xenobiotic	Benzoate (	NA	1	1	1.9	1	1	1	1	1	1	1	1	2
NA	NA	NA	NA	NA	NA	2	1	0.8	1	1	2	0	1	0	1	1	3

						0	0	0	0	19	0	26	0	0	0	0	0
NA	NA	NA	NA	NA	NA	1	1	2.3	1	2	1	2	1	1	1	2	1
NA	NA	NA	NA	NA	NA	2	1	1	1	1	1	1	1	1	1	1	1
NA	NA	NA	NA	NA	NA	1	1	0.8	1	1	1	1	0	17	1	1	1
Benzenedi	Phenols	Phenols ar	Amino aci	Tyrosine m	map00350	1	1	2.2	1	2	1	3	0	1	1	3	0
Tyrosols a	Phenols	Phenols ar	Amino aci	Tyrosine m	map00350	1	1	1.4	1	1	1	2	1	1	1	5	1
NA	NA	NA	NA	NA	NA	1	0	0	##	17	33	2	0	2	0	3	0
Phosphate	Organic pl	Organic ac	NA	NA	NA	1	1	2.4	1	2	1	2	1	1	2	2	1
NA	NA	NA	NA	NA	NA	1	1	1.3	1	1	3	1	1	1	1	1	1
NA	NA	NA	NA	NA	NA	2	1	1.2	1	0	0	1	2	2	1	0	9
NA	NA	NA	NA	NA	NA	1	2	0.2	2	0	2	0	1	1	1	0	1
NA	NA	NA	NA	NA	NA	1	1	1.9	1	2	1	2	1	1	1	2	1
NA	NA	NA	NA	NA	NA	1	1	2	1	2	1	2	1	1	1	2	1
NA	NA	NA	NA	NA	NA	1	1	2	1	2	1	2	1	1	1	2	1
NA	NA	NA	NA	NA	NA	1	1	0.9	1	1	1	1	2	3	1	1	1
NA	NA	NA	NA	NA	NA	1	2	1.1	1	1	49	1	1	1	1	2	1
NA	NA	NA	NA	NA	NA	2	1	0.9	2	1	1	1	0	2	1	1	3
NA	NA	NA	NA	NA	NA	1	1	1.1	1	1	2	1	1	1	1	1	1
NA	NA	NA	NA	NA	NA	1	1	1.9	1	2	1	2	1	1	1	2	1
						0	0	0	6	0	0	0	0	0	0	0	0
NA	NA	NA	NA	NA	NA	1	1	0.9	1	1	1	0	1	1	1	1	1
NA	NA	NA	NA	NA	NA	1	1	1.4	1	1	1	1	1	1	1	2	1
NA	NA	NA	NA	NA	NA	1	1	2.3	1	3	14	4	1	2	2	2	0
NA	NA	NA	NA	NA	NA	1	1	1.4	1	1	1	2	1	1	1	2	1
NA	NA	NA	NA	NA	NA	6	1	0.6	1	0	3	0	9	1	3	5	1
NA	NA	NA	NA	NA	NA	1	1	1	1	1	1	2	1	2	1	1	1
NA	NA	NA	NA	NA	NA	1	1	1.3	1	2	1	1	1	1	1	2	1
NA	NA	NA	NA	NA	NA	1	1	0.6	1	1	1	1	0	1	1	1	2
Glyceroph	Glyceroph	Glyceroph	NA	NA	NA	1	1	1.7	1	2	1	2	2	2	1	1	1
NA	NA	NA	NA	NA	NA	1	1	0.9	2	1	1	0	1	1	1	1	1
NA	NA	NA	NA	NA	NA	1	1	0.6	1	1	1	1	2	1	2	1	3
NA	NA	NA	NA	NA	NA	1	1	1.9	1	1	1	2	1	1	1	2	1
NA	NA	NA	NA	NA	NA	1	5	1.4	1	1	2	1	2	1	1	2	2
						0	0	0	0	0	0	0	0	0	0	0	0
NA	NA	NA	NA	NA	NA	1	1	1.5	1	1	1	1	1	1	5	72	1
						0	0	0	0	0	0	0	0	0	37	0	0
Others	Mycotoxin	Fungal tox	Biosynthes	Staurospo	map0110	1	1	0.3	2	1	0	0	1	1	1	0	1
NA	NA	NA	NA	NA	NA	0	2	3.3	1	3	1	3	0	1	1	2	0
NA	NA	NA	NA	NA	NA	1	10	2	1	2	1	2	1	1	1	20	1
NA	NA	NA	NA	NA	NA	1	1	0.9	1	0	1	0	1	1	1	1	1
NA	NA	NA	NA	NA	NA	0	1	0.7	0	1	32	3	1	2	1	1	2
NA	NA	NA	NA	NA	NA	1	2	0.7	1	1	1	1	1	11	1	1	1
						0	0	0	13	44	0	##	28	26	0	0	26
NA	NA	NA	NA	NA	NA	2	2	0.9	0	0	2	0	3	0	2	0	23
NA	NA	NA	Energy me	Methane r	map0110	##	1	0.9	2	3	0	1	0	1	0	1	4
NA	NA	NA	NA	NA	NA	1	1	1.9	1	2	1	2	1	1	1	2	1
						4	0	8	86	##	21	##	##	##	##	0	##
NA	NA	NA	NA	NA	NA	0	16	3	1	0	6	1	2	1	0	0	1
Beta-keto	Keto acids	Organic ac	NA	NA	NA	1	1	3.9	0	12	1	2	0	1	6	9	1
Ginkgolid	Diterpenoi	Terpenoid	NA	NA	NA	1	1	1.2	1	1	1	2	1	1	1	1	1
						0	0	0	0	2	0	0	55	0	0	0	0
NA	NA	NA	NA	NA	NA	1	1	0.8	1	1	83	1	0	1	1	1	3
NA	NA	NA	NA	NA	NA	1	1	0.5	1	0	1	0	2	0	0	1	1
NA	NA	NA	NA	NA	NA	1	2	2.4	1	2	1	2	0	1	5	2	1
NA	NA	NA	NA	NA	NA	1	3	1.1	2	0	2	0	2	0	1	3	0
Purine dec	Purine nuc	Purines an	Nucleotid	Purine me	map00230	1	1	1.3	1	1	16	1	1	1	1	1	88
NA	NA	NA	NA	NA	NA	1	2	0.6	1	2	4	7	0	11	4	2	1
NA	NA	NA	NA	NA	NA	1	1	1.6	2	2	0	1	1	1	1	11	1

NA	NA	NA	NA	NA	NA	1	1	0.9	1	2	1	2	2	1	1	1	2
NA	NA	NA	NA	NA	NA	1	1	1.9	1	2	1	2	1	1	1	2	1
Flavones	Flavonoids	Flavonoids	Biosynthes	Flavonoid	NA	1	1	1.4	1	32	1	1	1	1	1	2	1
Tricarboxy	Carboxylic	Organic ac	NA	NA	NA	3	1	0.3	3	1	1	0	1	2	0	1	1
NA	NA	NA	NA	NA	NA	1	1	0.9	2	1	1	0	1	1	1	1	1
NA	NA	NA	NA	NA	NA	3	4	1.8	1	0	23	0	9	1	2	0	4
NA	NA	NA	Metabolisı	Biosynthes	map0110(	1	0	0.8	1	1	2	1	3	1	0	0	1
NA	NA	NA	NA	NA	NA	1	3	0.5	0	2	3	4	2	1	1	44	3
NA	NA	NA	NA	NA	NA	1	1	2.4	1	1	1	2	1	1	1	1	2
NA	NA	NA	NA	NA	NA	2	1	0.8	1	1	1	0	1	0	1	1	1
Pyrimidine	Pyrimidine	Nucleic ac	NA	NA	NA	1	1	1.9	1	2	1	2	1	1	1	2	1
NA	NA	NA	NA	NA	NA	1	2	1	2	0	1	0	0	0	2	0	0
NA	NA	NA	NA	NA	NA	1	1	0.9	1	1	2	1	2	2	1	1	3
Amino aci	Carboxylic	Amino aci	NA	NA	NA	1	1	2.2	1	2	1	2	1	1	1	2	1
NA	NA	NA	NA	NA	NA	0	0	0.9	1	11	0	10	5	6	1	1	0
NA	NA	NA	NA	NA	NA	2	2	0.7	0	0	1	1	1	4	0	0	58
NA	NA	NA	NA	NA	NA	1	1	1.1	1	1	1	1	2	2	2	1	1
NA	NA	NA	NA	NA	NA	1	4	0.6	1	1	1	0	3	2	1	2	1
NA	NA	NA	NA	NA	NA	2	1	0.8	2	5	1	5	0	1	0	1	1
NA	NA	NA	NA	NA	NA	1	1	1.2	1	2	6	2	0	1	2	8	0
NA	NA	NA	NA	NA	NA	1	1	1.9	1	2	16	2	1	1	1	2	1
NA	NA	NA	NA	NA	NA	1	1	1.3	1	2	1	1	1	1	1	2	1
NA	NA	NA	NA	NA	NA	0	1	0.5	2	0	1	1	0	1	3	1	1
NA	NA	NA	NA	NA	NA	1	1	0.9	1	1	11	1	1	1	1	1	1
						0	0	0	0	0	0	29	0	0	0	0	0
Carbohydı	Organoox	Carbohydı	NA	NA	NA	1	1	1.2	1	1	1	1	1	1	2	1	1
Acridone a	Alkaloids c	Alkaloids	Biosynthes	Acridone a	NA	1	0	1	6	0	0	0	1	0	6	0	0
Amines	Organonit	Amines	NA	NA	map04024	0	1	1.1	1	2	1	3	2	1	1	1	20
NA	NA	NA	NA	NA	NA	1	1	0.8	1	1	1	1	0	2	1	1	1
1-benzoth	Benzothio	Organohe	NA	NA	NA	1	1	1.2	1	1	1	2	1	1	1	2	0
NA	NA	NA	NA	NA	NA	1	1	2	1	2	1	2	1	1	1	1	1
NA	NA	NA	NA	NA	NA	16	1	0.5	7	8	1	47	1	1	1	12	9
NA	NA	NA	Xenobiotic	Xylene deg	map0110(	1	1	0.6	2	1	0	0	0	1	4	0	0
NA	NA	NA	NA	NA	NA	1	1	0.8	1	1	1	1	1	4	1	1	1
Carbohydı	Organoox	Carbohydı	NA	NA	NA	1	1	2.1	1	2	1	2	1	1	1	2	1
NA	NA	NA	NA	NA	NA	1	1	1.2	1	1	1	2	1	1	1	1	1
						0	8	12	##	##	29	##	##	##	##	0	##
NA	NA	NA	NA	NA	NA	0	0	0.2	1	5	0	9	16	26	1	0	2
Piperazine	Diazinanes	Organohe	NA	NA	NA	1	1	0.9	1	0	1	0	1	1	1	2	0
NA	NA	NA	NA	NA	NA	1	1	1	1	1	1	1	1	1	1	1	1
NA	NA	NA	NA	NA	NA	1	1	1	1	2	1	2	4	2	1	1	1
NA	NA	NA	NA	NA	NA	1	1	1.4	1	1	1	2	5	1	1	1	1
NA	NA	NA	NA	NA	NA	1	1	0.6	1	1	1	1	1	1	1	1	2
NA	NA	NA	NA	NA	NA	0	1	0.4	1	0	1	1	1	6	1	1	2
NA	NA	NA	NA	NA	NA	1	1	0.8	1	1	1	1	5	1	3	1	1
Fatty acid	Fatty Acyls	Fatty acyls	NA	NA	NA	1	0	2.3	1	1	1	0	1	1	1	0	1
NA	NA	NA	NA	NA	NA	1	1	1.1	1	1	1	1	1	1	1	1	1
NA	NA	NA	NA	NA	NA	1	1	1.2	1	1	1	1	1	1	1	2	1
NA	NA	NA	NA	NA	NA	1	1	0.9	2	1	1	1	2	1	1	2	1
NA	NA	NA	NA	NA	NA	1	2	1.7	1	2	1	2	1	2	1	1	1
NA	NA	NA	NA	NA	NA	0	1	0.6	1	1	5	2	1	2	1	1	3
Carbohydı	Organoox	Carbohydı	NA	NA	NA	1	1	1.8	1	2	1	1	1	1	1	1	1
NA	NA	NA	NA	NA	NA	2	1	0.8	1	1	1	1	1	48	6	1	1
NA	NA	NA	NA	NA	NA	1	1	0.6	1	0	1	0	1	45	1	1	3
Androstan	Steroids aı	Steroids aı	NA	NA	NA	1	1	1.8	1	2	1	2	1	1	1	2	0
NA	NA	NA	NA	NA	NA	1	1	1.8	1	2	1	2	1	1	1	2	0
NA	NA	NA	Metabolisı	Porphyrin	map0086C	1	1	1.8	1	2	1	2	1	1	1	2	0
						6	7	0	12	##	0	7	0	23	34	0	59

Germacrer	Sesquiterp	Terpenoid	NA	NA	NA	3	0	0.2	8	0	5	0	3	0	10	0	0
NA	NA	NA	NA	NA	NA	1	1	1.2	1	1	1	1	1	1	1	1	1
NA	NA	NA	NA	NA	NA	4	1	1.2	1	1	1	1	1	7	1	1	1
NA	NA	NA	NA	NA	NA	9	0	0.9	1	3	1	0	4	0	1	0	0
NA	NA	NA	NA	NA	NA	1	1	0.9	2	1	1	0	1	1	1	1	1
Beta lactar	Lactams	Organohe	NA	NA	NA	1	1	1.7	1	1	1	0	1	1	2	1	1
NA	NA	NA	NA	NA	NA	1	1	1.3	1	1	2	1	1	1	1	1	2
NA	NA	NA	NA	NA	NA	1	1	0.8	1	1	3	1	1	23	1	1	1
Sugar acid	Monosacc	Carbohydr	Carbohydr	Pentose pl	map0003C	1	1	0.9	1	1	1	1	1	1	1	1	1
NA	NA	NA	NA	NA	NA	1	1	0.8	1	1	1	0	1	1	1	1	1
Piperidino	Piperidine:	Organohe	NA	NA	NA	1	1	1.9	1	2	1	2	1	1	1	1	1
Isoquinolir	Alkaloids (	Alkaloids	NA	NA	NA	1	2	0.5	8	1	1	1	1	3	0	1	2
NA	NA	NA	NA	NA	NA	1	1	2	1	2	1	3	1	2	2	1	1
NA	NA	NA	NA	NA	NA	1	1	2.4	1	0	3	0	3	0	3	4	0
NA	NA	NA	NA	NA	NA	1	1	1.8	1	2	1	2	1	1	1	2	0
NA	NA	NA	NA	NA	NA	1	3	0.9	0	1	1	1	1	1	1	1	0
NA	NA	NA	NA	NA	NA	1	1	1.3	1	1	1	1	1	1	1	1	1
NA	NA	NA	NA	NA	NA	1	1	2	1	2	1	2	1	1	1	2	1
NA	NA	NA	NA	NA	NA	1	1	1.3	1	1	1	1	1	1	1	1	##
Benzoic ac	Benzene a	Benzene a	NA	NA	NA	1	2	2	1	1	5	2	1	1	1	1	1
NA	NA	NA	NA	NA	NA	1	1	1	1	1	1	1	2	2	1	1	2
NA	NA	NA	NA	NA	NA	1	1	0.7	1	0	1	0	1	7	1	1	1
Hydroxyst	Steroids ar	Steroids ar	NA	NA	NA	1	0	0.1	1	0	0	1	0	6	1	0	62
NA	NA	NA	NA	NA	NA	1	1	2	1	2	1	2	1	1	1	2	1
NA	NA	NA	NA	NA	NA	1	1	1.9	1	1	7	1	1	1	1	2	1
1,4-benzo	Benzodiaz	Organohe	NA	NA	NA	1	1	2.1	1	1	1	3	1	1	1	2	1
NA	NA	NA	NA	NA	NA	1	1	1	1	2	25	4	0	2	2	4	1
NA	NA	NA	NA	NA	NA	1	1	1.2	1	1	1	1	1	1	1	1	2
NA	Tetralins	Benzene a	NA	NA	NA	1	1	1.3	1	2	1	1	1	1	1	2	1
NA	NA	NA	NA	NA	NA	2	1	0.6	3	2	0	0	2	5	2	0	2
NA	NA	NA	NA	NA	NA	0	1	0.1	1	0	1	0	1	1	2	0	23
NA	NA	NA	NA	NA	NA	1	1	1.9	1	2	1	2	1	1	1	2	1
NA	NA	NA	NA	NA	NA	0	0	2.3	0	2	0	4	1	1	0	1	2
NA	NA	NA	NA	NA	NA	0	0	2.3	0	2	0	4	1	1	0	1	2
NA	NA	NA	NA	NA	NA	1	1	1.8	1	2	1	2	1	1	1	2	0
NA	NA	NA	NA	NA	NA	1	1	1.8	1	2	1	2	1	1	1	2	0
NA	NA	NA	NA	NA	NA	1	1	1.8	1	2	1	2	1	1	1	2	0
NA	NA	NA	NA	NA	NA	1	1	1.4	1	1	1	1	1	1	1	1	1
NA	NA	NA	NA	NA	NA	1	0	0.3	2	0	1	1	5	0	0	1	5
NA	NA	NA	NA	NA	NA	1	1	1.3	3	1	1	1	1	0	1	1	2
NA	NA	NA	NA	NA	NA	1	2	1.7	1	2	1	2	1	2	1	1	1
Flavonols	Flavonoids	Flavonoids	NA	NA	NA	1	1	0.7	1	1	1	1	15	2	1	1	1
NA	NA	NA	NA	NA	NA	1	1	0.6	0	1	1	5	1	1	1	3	1
NA	NA	NA	NA	NA	NA	3	1	0.8	2	1	1	1	1	1	0	1	1
						0	0	0	0	54	##	64	31	48	18	8	22
NA	NA	NA	NA	NA	NA	1	1	1.6	1	2	13	2	1	1	1	3	1
						0	0	43	0	12	0	33	##	0	42	##	##
						0	0	0	0	0	0	0	15	12	0	0	4
NA	NA	NA	NA	NA	NA	1	2	0.5	2	0	0	0	0	0	1	0	0
NA	NA	NA	NA	NA	NA	0	0	2.1	5	1	1	1	0	1	15	1	1
NA	NA	NA	NA	NA	NA	1	1	1.8	1	2	1	2	1	1	1	2	0
NA	NA	NA	NA	NA	NA	7	2	0.6	2	1	0	1	3	0	50	1	0
NA	NA	NA	NA	NA	NA	1	1	2.2	1	1	4	1	1	1	1	1	1
NA	NA	NA	NA	NA	NA	0	1	0.6	0	2	1	2	4	2	1	0	6
						0	0	0	0	0	0	0	0	0	0	0	0
NA	NA	NA	NA	NA	NA	1	1	1.1	1	1	1	1	1	1	1	1	1
NA	NA	NA	NA	NA	NA	1	1	1.6	1	1	1	2	1	1	1	1	1
NA	NA	NA	NA	NA	NA	1	1	1.4	1	2	1	1	1	1	1	2	1

NA	NA	NA	NA	NA	NA	1	1	1.5	1	2	2	2	1	1	1	1	1
NA	NA	NA	NA	NA	NA	1	2	1.7	1	2	1	2	1	1	1	1	0
Indolines	Indoles an	Indole anc	NA	NA	NA	1	1	1.6	2	1	1	2	1	1	1	12	0
NA	NA	NA	NA	NA	NA	2	1	2.5	2	1	0	0	0	0	3	4	0
NA	NA	NA	NA	NA	NA	1	1	6.6	1	8	1	9	1	1	1	2	1
NA	NA	NA	NA	NA	NA	0	0	0.2	0	1	0	1	5	5	4	0	7
NA	NA	NA	NA	NA	NA	1	1	1.5	1	1	1	2	1	1	1	1	1
NA	NA	NA	NA	NA	NA	1	1	1.4	1	1	1	1	1	1	1	2	1
NA	NA	NA	NA	NA	NA	1	1	1.7	1	2	1	2	1	1	1	1	1
NA	NA	NA	NA	NA	NA	0	1	0.1	2	0	0	0	9	7	1	1	21
NA	NA	NA	NA	NA	NA	1	1	1.9	1	2	1	2	1	1	1	1	1
NA	NA	NA	NA	NA	NA	1	1	1.9	1	2	1	2	1	1	1	2	1
NA	NA	NA	NA	NA	NA	1	1	2.1	1	2	1	2	1	1	1	2	1
NA	NA	NA	NA	NA	NA	0	0	0.2	1	0	2	0	2	6	0	1	2
NA	NA	NA	NA	NA	NA	3	1	0.4	5	3	9	7	1	1	2	59	3
Amino aci	Carboxylic	Amino aci	Lipid meta	Primary bi	map00120	0	2	0.6	1	1	2	2	1	2	1	3	0
NA	NA	NA	NA	NA	NA	1	1	1.5	1	3	1	1	5	1	1	1	1
NA	NA	NA	NA	NA	NA	1	1	1.3	1	2	1	1	1	1	1	1	1
NA	NA	NA	NA	NA	NA	4	2	0.4	7	2	6	0	0	3	0	2	2
NA	NA	NA	NA	NA	NA	1	1	0.5	1	0	0	1	1	8	1	0	2
NA	NA	NA	NA	NA	NA	1	1	0.7	1	1	0	1	4	3	1	1	6
NA	NA	NA	NA	NA	NA	1	1	1.9	1	2	1	2	1	1	1	2	1
NA	NA	NA	NA	NA	NA	1	1	1.9	1	2	1	2	1	1	1	2	1
NA	NA	NA	NA	NA	NA	1	1	1.8	1	1	14	1	1	1	1	1	1
NA	NA	NA	NA	NA	NA	1	1	1.6	1	1	1	2	1	1	1	1	1
NA	NA	NA	NA	NA	NA	1	2	0.5	3	1	0	0	1	0	1	1	0
NA	NA	NA	NA	NA	NA	2	1	0.8	1	1	1	0	1	2	1	1	1
NA	NA	NA	NA	NA	NA	1	1	0.9	2	1	1	0	1	1	1	1	1
NA	NA	NA	NA	NA	NA	0	5	0.9	1	0	2	0	1	1	3	1	1
NA	NA	NA	NA	NA	NA	1	1	2.8	1	3	1	2	1	2	2	1	1
Phenylace	Benzene a	Benzene a	Amino aci	Phenylalar	map00360	1	1	1.4	1	3	1	7	1	1	1	1	0
NA	NA	NA	NA	NA	NA	1	1	1.3	1	2	1	1	0	1	1	1	1
NA	NA	NA	NA	NA	NA	1	1	0.7	2	0	1	0	1	0	2	1	0
NA	NA	NA	NA	NA	NA	1	1	0.7	1	1	1	1	1	99	4	1	1
NA	NA	NA	NA	NA	NA	1	1	0.7	1	1	2	1	1	1	1	1	1
NA	NA	NA	NA	NA	NA	1	1	1.4	1	2	1	2	1	1	1	1	23
Isoquinolir	Alkaloids (	Alkaloids	NA	NA	NA	1	1	0.9	1	1	1	1	1	5	1	1	1
NA	NA	NA	NA	NA	NA	1	1	0.4	2	0	1	0	1	2	1	1	9
Carbonyl (	Organoox	Organic o:	NA	NA	NA	1	1	0.8	1	1	1	1	0	2	1	1	1
NA	NA	NA	NA	NA	NA	10	0	1.7	5	0	0	1	16	0	2	7	4
NA	NA	NA	NA	NA	NA	1	1	2.2	1	2	11	2	1	1	1	2	0
NA	NA	NA	NA	NA	NA	1	1	1.5	1	2	1	3	0	1	1	1	0
Androstan	Steroids ar	Steroids ar	Lipid meta	Steroid ho	map00140	0	0	0.2	0	2	0	6	4	3	1	0	0
Dibenzaze	Benzazepi	Organohe	NA	NA	NA	1	1	2.7	1	3	6	4	1	1	2	2	0
NA	NA	NA	NA	NA	NA	1	1	1.9	1	2	1	2	1	1	1	2	1
NA	NA	NA	NA	NA	NA	1	1	1.9	1	2	1	2	1	1	1	2	1
NA	NA	NA	NA	NA	NA	1	2	1.5	1	1	1	1	1	15	1	1	2
NA	NA	NA	Biosynthes	Phenazine	map0110(	1	2	1	3	0	1	0	0	0	2	0	0
NA	NA	NA	NA	NA	NA	1	1	1.6	1	2	2	2	1	1	1	2	1
NA	NA	NA	NA	NA	NA	1	1	0.8	1	1	1	1	1	1	1	1	1
NA	NA	NA	NA	NA	NA	1	1	0.8	1	1	1	1	1	1	1	1	1
NA	NA	NA	NA	NA	NA	1	1	1.2	2	0	1	0	1	0	2	1	0
NA	NA	NA	NA	NA	NA	1	1	1.2	1	13	10	1	7	1	6	1	1
Carbohydr	Organoox	Carbohydr	NA	NA	NA	1	1	1.1	1	1	1	1	1	1	1	1	1
NA	NA	NA	NA	NA	NA	1	1	0.4	1	4	30	1	1	1	0	2	1
NA	NA	NA	NA	NA	NA	1	1	1.1	1	0	14	0	1	1	1	1	1
NA	NA	NA	NA	NA	NA	1	1	4.8	1	4	2	1	1	1	3	2	1
Neolignan	Lignans	Lignans	NA	NA	NA	1	1	0.6	8	0	1	0	1	0	3	0	0

Amino aci	Carboxylic	Amino aci	Metabolis	Glutathion	map0048	0	1	1.6	1	2	1	2	1	1	1	2	1
						##	##	122	##	##	16	##	##	##	##	59	##
NA	NA	NA	NA	NA	NA	1	0	0.7	1	1	0	1	1	0	1	1	1
NA	NA	NA	NA	NA	NA	1	10	2.1	7	30	0	49	0	0	0	3	2
Hydroxyci	Cinnamic a	Polyketide	NA	NA	NA	1	2	0.6	1	0	15	1	4	0	1	1	21
NA	NA	NA	NA	NA	NA	1	0	14	0	2	2	1	6	2	1	10	0
NA	NA	NA	NA	NA	NA	2	0	0.6	13	0	1	0	4	3	1	1	2
NA	NA	NA	NA	NA	NA	1	1	0.9	1	1	1	1	4	2	1	1	1
NA	NA	NA	Metabolis	Type I poly	NA	1	1	0.9	1	0	1	0	1	1	1	2	0
NA	NA	NA	NA	NA	NA	2	0	1.4	8	1	1	1	0	0	1	0	1
NA	NA	NA	NA	NA	NA	1	1	0.8	3	0	1	0	1	0	3	2	0
Pregnane	Steroids a	Steroids a	NA	NA	NA	1	1	1.3	1	2	1	1	1	1	1	1	1
						0	0	0	0	14	0	0	0	0	0	0	0
NA	NA	NA	NA	NA	NA	1	1	2	1	2	1	2	1	1	1	2	1
NA	NA	NA	NA	NA	NA	1	2	0.8	1	1	1	1	4	1	1	1	1
NA	NA	NA	NA	NA	NA	1	1	1.8	1	2	1	2	1	1	1	2	2
NA	NA	NA	NA	NA	NA	1	1	1	3	1	0	1	13	4	2	1	12
NA	NA	NA	NA	NA	NA	1	1	0.7	1	3	1	1	1	1	1	2	3
NA	NA	NA	NA	NA	NA	1	1	0.8	1	0	1	1	1	7	1	1	1
NA	NA	NA	NA	NA	NA	##	1	1.2	1	3	1	1	2	1	1	1	##
NA	NA	NA	NA	NA	NA	1	1	1.4	1	2	1	1	1	1	1	2	1
NA	NA	NA	NA	NA	NA	1	1	0.9	1	1	1	0	1	1	1	1	1
NA	NA	NA	NA	NA	NA	1	1	1	1	1	1	0	1	1	1	1	1
NA	NA	NA	NA	NA	NA	1	1	1.4	2	0	0	0	1	1	5	1	0
						11	0	0	##	##	5	##	##	##	##	0	##
NA	NA	NA	NA	NA	NA	1	1	1.6	1	2	1	2	1	2	1	1	1
NA	NA	NA	NA	NA	NA	1	0	0.4	1	0	1	1	2	1	1	1	3
NA	NA	NA	NA	NA	NA	1	1	1.3	1	2	1	1	1	1	1	1	1
NA	NA	NA	NA	NA	NA	1	1	1.9	1	2	1	0	1	1	2	2	4
NA	NA	NA	NA	NA	NA	1	1	1.7	1	2	1	2	1	1	1	2	0
NA	NA	NA	NA	NA	NA	1	1	1.1	1	1	1	1	1	1	1	1	1
NA	NA	NA	NA	NA	NA	2	1	1.3	1	1	1	1	1	1	1	1	1
NA	NA	NA	NA	NA	NA	1	1	0.9	1	1	1	1	1	1	1	1	1
Amino aci	Carboxylic	Amino aci	NA	NA	NA	1	1	1.3	1	2	1	2	1	1	1	1	1
NA	NA	NA	NA	NA	NA	1	1	0.8	1	1	1	1	1	1	2	1	2
NA	NA	NA	NA	NA	NA	1	1	0.4	2	1	0	0	0	1	2	0	0
NA	NA	NA	NA	NA	NA	1	1	1	1	2	3	1	1	2	1	1	1
NA	NA	NA	NA	NA	NA	1	1	1.1	1	1	1	2	1	1	1	1	1
						0	0	0	0	0	0	0	3	0	0	0	0
NA	NA	NA	NA	NA	NA	1	0	1.1	1	3	0	1	0	0	1	1	2
NA	NA	NA	NA	NA	NA	1	0	1	1	1	1	1	0	0	1	1	1
NA	NA	NA	NA	NA	NA	1	1	2.7	1	2	2	3	1	1	1	2	1
NA	NA	NA	Global an	Biosynthes	NA	0	0	49	0	8	15	3	2	0	1	0	5
NA	NA	NA	NA	NA	NA	1	1	1	1	1	1	0	1	1	1	1	1
NA	NA	NA	NA	NA	NA	1	1	2	1	1	1	0	2	1	2	1	0
NA	NA	NA	NA	NA	NA	2	1	0.7	3	2	0	0	0	0	1	0	0
NA	NA	NA	NA	NA	NA	1	1	1.9	1	2	1	2	1	1	1	2	1
NA	NA	NA	NA	NA	NA	1	1	1.3	1	1	1	1	1	11	1	1	1
NA	NA	NA	NA	NA	NA	1	1	0.8	3	0	1	0	1	0	3	3	0
Amino aci	Carboxylic	Amino aci	NA	NA	NA	1	0	0.5	1	0	1	1	1	0	1	1	1
NA	NA	NA	NA	NA	NA	1	1	0.9	2	1	1	0	2	1	1	2	1
NA	NA	NA	NA	NA	NA	0	1	0.1	2	0	0	1	10	10	0	1	30
Furanones	Dihydrofu	Organohe	Xenobiotic	Fluoroben	NA	5	1	90	2	3	2	1	0	0	0	3	1
Carbazole	Indoles an	Indole an	NA	NA	NA	1	1	1.4	1	2	1	1	1	1	1	2	1
Short-chai	Hydroxy a	Organic ac	NA	NA	NA	1	2	2.1	1	2	1	2	0	0	1	2	0
NA	NA	NA	NA	NA	NA	1	1	1.4	1	2	1	1	1	1	1	2	1
NA	NA	NA	NA	NA	NA	1	1	1.7	1	2	1	2	1	1	1	2	0
NA	NA	NA	NA	NA	NA	1	1	1.2	1	1	1	2	1	1	1	1	1

NA	NA	NA	NA	NA	NA	2	1	0.9	1	1	1	1	1	1	0	0	2
NA	NA	NA	NA	NA	NA	0	1	0.8	1	1	1	1	2	3	2	1	13
NA	NA	NA	NA	NA	NA	1	1	0.9	1	0	1	1	1	1	1	1	2
NA	NA	NA	NA	NA	NA	1	1	0.8	1	1	0	1	1	0	1	1	1
NA	NA	NA	NA	NA	NA	1	1	3.1	1	2	2	3	0	1	1	2	0
NA	NA	NA	NA	NA	NA	7	1	1.1	14	0	0	0	3	1	5	0	5
Fatty acids	Fatty Acyls	Fatty acyls	Lipid meta	Fatty acid	map00061	0	1	0.5	1	1	1	1	1	1	1	19	2
Fatty acids	Fatty Acyls	Fatty acyls	Lipid meta	Biosynthes	map0104C	0	1	0.2	0	1	14	1	1	7	0	1	4
NA	NA	NA	NA	NA	NA	0	1	0.4	2	1	1	1	4	3	2	2	1
NA	NA	NA	NA	NA	NA	1	1	0.8	1	1	1	1	2	1	1	1	1
Indanones	Indanes	Benzene a	NA	NA	NA	1	1	1.5	1	2	1	1	1	1	1	2	1
NA	NA	NA	NA	NA	NA	1	1	2	1	2	1	2	1	2	1	2	1
NA	NA	NA	NA	NA	NA	1	1	1.1	1	1	1	1	1	1	1	2	1
NA	NA	NA	NA	NA	NA	1	2	1.3	1	1	2	1	1	2	1	2	29
Benzenesu	Benzene a	Benzene a	NA	NA	NA	1	1	1.5	1	2	1	1	1	1	1	2	1
NA	NA	NA	NA	NA	NA	1	1	0.8	1	1	1	1	1	7	1	1	1
NA	NA	NA	Metabolisi	Porphyrin	map0086C	1	1	0.3	4	0	2	0	2	0	2	2	0
						0	0	0	8	40	0	0	14	0	##	0	0
NA	NA	NA	NA	NA	NA	2	1	1.4	6	8	0	1	1	1	1	1	0
NA	NA	NA	NA	NA	NA	1	2	1.1	1	3	1	2	1	1	1	3	1
Amino aci	Carboxylic	Amino aci	NA	NA	NA	0	0	30	0	5	9	2	2	1	1	0	2
NA	NA	NA	NA	NA	NA	1	0	0.2	1	3	0	0	0	2	1	0	1
Amino aci	Carboxylic	Amino aci	NA	NA	NA	29	2	0.9	1	1	1	2	1	1	1	12	1
NA	NA	NA	NA	NA	NA	0	7	1.3	10	1	11	0	0	1	0	1	2
NA	NA	NA	NA	NA	NA	1	1	2.2	1	2	1	2	1	1	1	2	1
NA	NA	NA	NA	NA	NA	1	1	0.8	1	1	1	0	1	1	1	1	1
NA	NA	NA	NA	NA	NA	1	1	1	1	1	1	0	1	1	1	1	1
NA	NA	NA	NA	NA	NA	1	2	1.5	1	1	1	3	7	3	1	1	1
NA	NA	NA	NA	NA	NA	1	1	1	1	1	1	2	1	1	1	1	1
NA	NA	NA	NA	NA	NA	1	5	0.4	1	1	2	1	2	1	1	1	1
Glyceroph	Glyceroph	Glyceroph	NA	NA	NA	4	2	1.2	1	7	1	5	1	1	2	1	2
NA	NA	NA	NA	NA	NA	1	3	0.5	2	1	0	0	0	0	1	0	0
NA	NA	NA	NA	NA	NA	6	1	0.1	85	7	35	2	0	1	0	2	0
NA	NA	NA	NA	NA	NA	1	1	0.8	1	1	1	2	1	1	1	1	1
NA	NA	NA	NA	NA	NA	3	0	1.5	2	1	1	1	1	0	5	2	1
NA	NA	NA	NA	NA	NA	1	1	0.7	1	64	1	14	1	1	1	2	7
NA	NA	NA	NA	NA	NA	1	1	0.3	3	0	0	0	2	0	2	1	1
NA	NA	NA	NA	NA	NA	1	2	6.7	1	14	0	45	0	1	1	1	0
NA	NA	NA	NA	NA	NA	2	1	0.9	1	0	0	1	1	0	2	1	1
						0	0	0	34	5	0	11	18	29	77	0	0
NA	NA	NA	NA	NA	NA	1	1	0.7	1	0	1	0	2	2	2	2	0
NA	NA	NA	NA	NA	NA	1	1	1.9	1	2	1	2	1	1	1	2	1
NA	NA	NA	NA	NA	NA	1	0	0.5	1	0	1	0	1	1	1	2	3
NA	NA	NA	NA	NA	NA	1	1	0.5	2	1	1	1	1	14	2	1	2
NA	NA	NA	NA	NA	NA	4	0	0.4	4	0	1	0	3	0	4	0	1
NA	NA	NA	NA	NA	NA	1	1	0.8	1	1	1	2	1	1	1	1	1
NA	NA	NA	NA	NA	NA	4	0	0.3	10	1	5	9	4	0	1	2	27
NA	NA	NA	NA	NA	NA	0	5	0.3	1	0	1	3	1	0	0	60	2
NA	NA	NA	NA	NA	NA	1	1	1.5	1	2	1	2	1	1	1	1	1
NA	NA	NA	NA	NA	NA	1	1	1.8	1	2	1	2	1	1	1	2	1
						0	0	0	9	6	12	69	##	##	##	0	##
Purines an	Imidazopy	Purines an	Biosynthes	Caffeine m	map00232	##	1	0.9	83	1	1	1	1	1	10	7	1
NA	NA	NA	NA	NA	NA	2	1	2.6	1	2	1	2	1	1	1	1	1
Dammarer	Triterpeno	Terpenoid	NA	NA	NA	2	1	0.5	1	0	1	0	2	0	3	1	6
NA	NA	NA	NA	NA	NA	1	0	1.5	0	2	1	2	0	1	1	2	1
NA	NA	NA	NA	NA	NA	1	2	0.8	1	1	1	1	1	0	1	1	0
						0	0	0	0	0	0	0	0	0	0	0	0
Amino aci	Carboxylic	Amino aci	Amino aci	Alanine, as	map0025C	2	1	1.6	2	2	4	2	0	1	1	1	2

NA	NA	NA	NA	NA	NA	1	1	1.4	1	1	1	1	1	1	1	1	1
NA	NA	NA	NA	NA	NA	2	3	0.4	1	0	0	2	1	2	1	1	2
NA	NA	NA	NA	NA	NA	1	1	1.4	1	1	1	1	1	1	1	2	1
1,4-benzo	Benzodiaz	Organohe	NA	NA	NA	1	1	1.2	1	1	1	2	1	1	1	1	1
NA	NA	NA	NA	NA	NA	1	0	0.6	4	0	1	0	1	0	9	3	0
NA	NA	NA	NA	NA	NA	1	1	0.7	2	1	1	0	2	1	1	2	1
NA	NA	NA	NA	NA	NA	1	1	1.6	1	2	1	1	1	1	1	2	1
Alcohols a	Organoox	Alcohols	NA	NA	NA	1	1	2	1	2	1	2	1	1	1	2	1
NA	NA	NA	NA	NA	NA	1	20	1.2	1	2	1	2	1	1	1	1	1
Fatty acids	Fatty Acyls	Fatty acyls	Metabolisı	Terpenoid	map0090C	1	1	1.6	1	3	1	5	4	0	1	3	1
NA	NA	NA	NA	NA	NA	1	1	1.5	1	2	1	4	1	1	1	2	1
Quinolizid	Alkaloids (	Alkaloids	Biosynthes	Tropane, p	NA	16	1	2.8	1	1	3	1	1	1	1	1	1
1-hydroxy	Phenols	Phenols ar	NA	NA	map0408C	2	1	1.1	1	1	3	2	1	1	1	2	2
NA	NA	NA	NA	NA	NA	1	3	2.9	0	2	59	3	1	1	1	1	1
Glyceroph	Glyceroph	Glyceroph	NA	NA	NA	0	1	0	1	1	0	1	7	3	2	0	4
NA	NA	NA	NA	NA	NA	1	1	0.3	1	0	1	1	2	1	0	1	2
NA	NA	NA	NA	NA	NA	0	2	0.2	2	0	0	0	17	11	1	2	39
NA	NA	NA	NA	NA	NA	1	2	1.1	1	1	1	1	2	2	2	1	1
Beta lactar	Lactams	Organohe	NA	NA	NA	1	16	1	1	1	1	1	1	1	1	1	1
NA	NA	NA	NA	NA	NA	0	0	0.2	1	0	0	0	8	3	4	0	0
NA	NA	NA	NA	NA	NA	1	1	1	1	2	1	1	1	1	1	1	1
NA	NA	NA	NA	NA	NA	1	1	1.4	1	1	1	1	1	1	1	1	1
NA	NA	NA	NA	NA	NA	2	1	0.7	1	1	1	1	1	1	1	1	4
Benzenesu	Benzene a	Benzene a	NA	NA	NA	1	0	0.8	2	0	0	0	1	1	2	0	0
NA	NA	NA	NA	NA	NA	1	20	1.2	1	1	1	1	1	1	1	1	1
NA	NA	NA	NA	NA	NA	0	1	1.6	3	0	3	0	2	0	1	3	0
NA	NA	NA	NA	NA	NA	1	1	2.2	1	2	1	2	1	1	1	2	1
NA	NA	NA	NA	NA	NA	1	1	2.3	1	1	1	1	0	1	11	1	1
						0	0	0	16	##	12	60	85	##	##		0	##
Amino aci	Carboxylic	Amino aci	Amino aci	Glycine, se	map0026C	1	1	1.6	1	1	1	2	1	1	1	1	0
NA	NA	NA	NA	NA	NA	1	1	1.2	1	2	1	1	1	1	1	1	1
Bisphosph	Organic pl	Organic ac	NA	NA	NA	1	1	1.1	1	1	1	2	1	1	1	1	1
NA	NA	NA	NA	NA	NA	0	1	3.4	1	1	20	2	0	1	1	1	1
NA	NA	NA	NA	NA	NA	1	1	0.1	1	3	0	2	1	2	1	0	2
Benzoic ac	Benzene a	Benzene a	NA	NA	NA	1	1	1.4	1	1	1	2	1	1	1	1	1
NA	NA	NA	NA	NA	NA	0	3	0.3	0	1	0	0	1	1	1	0	2
Dammarer	Triterpeno	Terpenoid	NA	NA	NA	0	1	0.7	1	1	0	1	3	1	1	1	1
Amino aci	Carboxylic	Amino aci	NA	NA	NA	53	0	1.9	13	0	0	2	0	0	2	4	1
NA	Phenylpro	Phenylpro	NA	NA	NA	3	1	2.9	4	1	2	2	1	0	2	1	1
NA	NA	NA	NA	NA	NA	1	1	1.1	0	1	1	1	7	1	1	1	1
NA	NA	NA	NA	NA	NA	1	1	1.5	1	55	1	2	1	1	1	1	1
NA	NA	NA	Biosynthes	Staurospo	map0110(	1	1	1.7	1	1	3	1	1	1	1	1	1
Carbonyl c	Organoox	Organic o:	NA	NA	NA	1	1	1.4	1	1	1	1	1	1	1	2	1
Purine ribo	Purine nuc	Purines an	NA	NA	NA	1	1	1.9	1	2	1	2	1	1	1	2	1
NA	NA	NA	NA	NA	NA	1	1	1.9	1	2	1	2	1	1	1	2	1
Pyrroloind	Indoles an	Indole anc	NA	NA	NA	1	1	1.2	1	1	1	1	1	1	1	1	1
Pyrimidine	Diazines	Organohe	NA	NA	NA	1	1	0.5	2	1	1	0	1	0	1	1	0
NA	NA	NA	NA	NA	NA	1	1	0.7	1	1	1	1	2	1	1	1	1
NA	NA	NA	NA	NA	NA	1	1	0.8	1	0	1	0	2	1	1	2	1
NA	NA	NA	NA	NA	NA	1	1	1.6	1	2	3	9	1	3	1	1	1
Phenylpro	Benzene a	Benzene a	Amino aci	Phenylalar	map0036C	1	1	2.1	1	1	1	2	1	1	1	2	1
NA	NA	NA	NA	NA	NA	1	0	0.2	6	0	1	0	6	0	2	0	0
NA	NA	NA	NA	NA	NA	1	1	1.5	1	2	2	2	1	1	2	1	1
O-methyla	Flavonoids	Flavonoids	NA	NA	NA	1	1	0.6	1	1	1	1	1	1	2	1	1
GP0105 M	GP01 Glyc	Glyceroph	NA	NA	NA	8	1	1.3	0	0	0	0	1	0	0	3	2
NA	NA	NA	NA	NA	NA	1	1	1.9	1	2	2	2	1	1	1	2	2
NA	NA	NA	NA	NA	NA	1	1	0.7	2	0	1	0	1	1	2	1	0
NA	NA	NA	NA	NA	NA	1	1	1	16	8	1	2	2	1	6	1	1

NA	NA	NA	NA	NA	NA	1	1	2	1	2	1	2	1	1	1	2	1
Carbohydr	Organoox	Carbohydr	NA	NA	NA	1	2	1.9	1	1	1	1	1	1	1	1	1
NA	NA	NA	Global anc	Biosynthes	NA	1	1	2.1	1	2	1	2	1	1	1	2	1
O-methyla	Isoflavono	Flavonoids	NA	NA	NA	1	1	1.5	1	1	1	1	1	1	1	1	6
NA	Morphinar	Alkaloids a	NA	NA	NA	1	1	0.9	2	1	1	0	1	1	1	1	1
NA	NA	NA	NA	NA	NA	11	1	1.5	1	1	50	2	1	1	1	1	1
NA	NA	NA	NA	NA	NA	1	1	1.5	1	2	1	2	1	2	1	1	1
Cumenes	Benzene a	Benzene a	NA	NA	NA	1	2	1.2	1	1	3	2	5	0	3	18	1
NA	NA	NA	NA	NA	NA	1	2	1	5	0	2	0	0	1	1	1	0
NA	Benzodiox	Organohe	NA	NA	NA	1	0	1.7	2	3	1	1	0	1	1	3	1
Amino aci	Carboxylic	Amino aci	Amino aci	Histidine r	map00340	3	1	2	3	1	1	2	1	2	1	1	0
NA	NA	NA	NA	NA	NA	6	0	3.3	1	1	59	1	0	0	1	1	0
NA	NA	NA	NA	NA	NA	1	30	1.2	1	2	1	18	1	1	1	1	1
NA	NA	NA	NA	NA	NA	1	1	1.6	1	2	1	2	1	1	1	1	24
NA	NA	NA	NA	NA	NA	1	1	1.2	1	2	2	2	1	1	1	2	1
NA	NA	NA	NA	NA	NA	1	1	1.9	1	2	1	2	1	1	1	2	1
NA	NA	NA	NA	NA	NA	1	1	0.9	1	1	7	0	1	1	1	1	1
NA	NA	NA	NA	NA	NA	1	2	2.1	1	3	1	2	2	1	1	1	1
NA	NA	NA	NA	NA	NA	1	1	0.7	1	1	0	2	1	1	1	1	1
Eicosanoic	Fatty Acyls	Fatty acyls	NA	NA	NA	1	2	1.3	1	1	1	1	1	1	0	1	1
NA	NA	NA	NA	NA	NA	4	0	0.4	7	0	0	0	1	0	14	10	1
NA	NA	NA	NA	NA	NA	9	1	1.8	1	2	1	13	1	1	1	2	1
NA	NA	NA	NA	NA	NA	1	1	1	4	1	1	1	2	1	8	5	2
NA	NA	NA	NA	NA	NA	1	1	1.3	1	1	1	1	1	1	2	1	1
Benzenedi	Phenols	Phenols ar	Amino aci	Tyrosine m	map00350	1	2	0.9	1	2	2	1	2	1	1	2	0
						0	0	0	0	11	0	0	0	0	0	0	0
						0	0	0	0	0	0	0	15	0	27	0	0
Flavans	Flavonoids	Flavonoids	NA	NA	map04152	1	1	0.8	1	1	1	0	2	1	1	2	1
Indolyl car	Indoles an	Indole anc	NA	NA	NA	1	1	1.8	1	2	1	3	1	1	1	2	1
NA	NA	NA	NA	NA	NA	1	1	1.1	1	1	2	1	1	1	1	1	3
NA	NA	NA	NA	NA	NA	1	1	0.8	1	1	1	1	7	1	1	2	0
						17	0	0	5	12	4	3	95	38	0	0	16
Glyceroph	Glyceroph	Glyceroph	NA	NA	NA	1	1	1.2	1	1	1	1	2	1	1	1	1
NA	NA	NA	NA	NA	NA	1	1	1.8	1	2	1	2	1	1	1	1	1
Methoxyp	Phenols	Phenols ar	Amino aci	Tyrosine m	map00350	1	7	1.2	5	1	3	1	1	1	1	1	1
NA	NA	NA	NA	NA	NA	3	0	3.9	1	2	1	2	0	1	0	0	1
NA	NA	NA	NA	NA	NA	1	1	0.7	1	0	1	0	1	11	1	2	1
NA	NA	NA	NA	NA	NA	1	1	1.4	1	1	1	1	1	1	1	1	1
NA	NA	NA	NA	NA	NA	5	0	0.6	1	1	0	1	3	1	5	1	0
NA	NA	NA	NA	NA	NA	1	1	2.1	1	1	1	2	1	1	1	16	1
NA	NA	NA	NA	NA	NA	1	1	0.7	1	1	1	0	1	1	1	1	1
NA	NA	NA	NA	NA	NA	0	1	0.3	3	1	0	0	0	1	1	0	1
NA	NA	NA	NA	NA	NA	3	1	0.8	1	3	2	2	1	3	1	1	14
NA	Tetrahydr	Organohe	NA	NA	NA	2	1	1.2	1	1	1	1	1	1	1	1	1
NA	NA	NA	NA	NA	NA	1	8	0.8	1	0	1	0	2	1	1	2	1
						0	0	0	0	35	0	0	0	0	0	0	0
NA	NA	NA	NA	NA	NA	3	1	0.8	3	5	2	9	5	1	2	3	5
NA	NA	NA	NA	NA	NA	2	1	0.6	2	1	1	1	1	2	0	1	1
NA	NA	NA	NA	NA	NA	1	1	1	1	0	1	0	1	1	1	1	1
NA	NA	NA	NA	NA	NA	1	1	1	1	1	1	0	1	1	1	1	1
NA	NA	NA	NA	NA	NA	1	1	1.6	1	2	1	2	1	1	1	1	0
NA	NA	NA	NA	NA	NA	1	1	0.4	0	0	19	1	3	0	1	0	3
Fatty acids	Fatty Acyls	Fatty acyls	Lipid meta	Cutin, sub	map0110	1	1	1.9	1	3	1	9	1	1	1	2	1
NA	NA	NA	NA	NA	NA	1	2	1.5	1	2	1	1	2	0	2	1	0
Amino aci	Carboxylic	Amino aci	NA	NA	NA	1	5	1.7	3	7	1	1	1	1	1	1	2
Fatty acids	Fatty Acyls	Fatty acyls	NA	NA	NA	2	1	1.4	5	1	11	1	21	1	1	1	0
NA	NA	NA	NA	NA	NA	1	1	1.3	1	1	1	1	1	0	1	1	0
NA	NA	NA	NA	NA	NA	1	1	1.2	1	1	1	2	1	1	3	1	1

NA	NA	NA	NA	NA	NA	1	1	2	1	1	1	2	1	1	1	1	1
Cyanogen	Cyanogen	Amino aci	NA	NA	NA	1	1	0.8	1	1	1	1	0	0	1	2	1
NA	NA	NA	Metabolisı	One carbc	map0067C	1	1	2.9	1	2	1	2	1	1	1	1	0
NA	NA	NA	NA	NA	NA	1	1	0.9	1	2	1	1	1	1	0	1	2
Fatty acids	Fatty Acyls	Fatty acyls	Lipid meta	Linoleic ac	map00591	1	0	0.3	0	2	23	1	3	9	0	1	8
NA	NA	NA	NA	NA	NA	1	0	1.7	1	1	1	1	1	1	0	1	1
						0	0	0	0	0	0	0	65	0	0	0	0
NA	NA	NA	NA	NA	NA	1	1	1.9	1	2	1	2	1	1	1	2	0
Substitute	Pyrroles	Organohe	NA	NA	NA	1	1	0.7	1	1	1	1	1	1	2	2	0
Carbohydı	Organoox	Carbohydı	NA	NA	NA	1	1	2	1	2	1	2	1	1	1	2	1
NA	NA	NA	NA	NA	NA	1	1	1	2	0	1	0	1	0	3	1	0
NA	NA	NA	NA	NA	NA	1	1	0.9	1	1	1	0	2	1	1	2	1
NA	NA	NA	Metabolisı	Nicotinate	map0076C	1	1	2	1	2	1	2	1	1	1	2	1
Terpene g	Prenol lipi	Terpenoid	NA	NA	NA	1	1	0.7	1	1	1	1	1	1	1	1	1
NA	NA	NA	NA	NA	NA	3	0	0.1	4	0	0	0	2	1	1	0	2
NA	NA	NA	Biosynthes	Monobact	map0110(	1	1	1.7	1	1	1	2	1	1	1	1	1
NA	NA	NA	NA	NA	NA	1	1	1.4	1	2	1	1	1	1	1	2	1
NA	NA	NA	Metabolisı	Porphyrin	map0086C	1	1	1.7	1	1	1	1	2	1	1	1	1
						0	0	0	0	0	0	0	0	0	0	0	0
						0	0	0	0	0	0	0	0	0	0	0	0
NA	NA	NA	Nucleotid	Purine me	map0023C	1	1	1.2	1	1	1	2	1	1	1	1	1
Glyceroph	Glyceroph	Glyceroph	NA	NA	NA	0	1	0.1	1	3	0	2	1	1	2	0	13
NA	NA	NA	Metabolisı	Nicotinate	map0076C	1	1	1.3	1	1	1	1	1	1	1	1	1
NA	Tetrahydr	Organohe	NA	NA	NA	1	1	0.9	1	1	1	0	1	1	1	1	1
NA	NA	NA	NA	NA	NA	1	1	0.9	1	1	1	1	1	1	1	1	1
NA	NA	NA	NA	NA	NA	0	4	0.3	0	1	4	2	1	1	1	1	25
NA	NA	NA	NA	NA	NA	1	1	1.4	1	2	2	2	##	1	1	2	1
						0	0	0	6	16	0	47	##	18	38	0	0
NA	NA	NA	NA	NA	NA	1	1	2.1	1	2	1	2	1	1	1	2	1
NA	NA	NA	NA	NA	NA	1	1	1.6	1	2	1	2	2	1	1	1	1
Carbonyl c	Organoox	Organic o	NA	NA	NA	1	2	0.6	1	0	4	1	0	1	0	0	7
						0	0	0	0	5	0	0	0	17	0	0	0
NA	NA	NA	NA	NA	NA	1	3	0.9	2	2	2	1	2	1	0	1	1
NA	NA	NA	NA	NA	NA	2	0	0.2	3	0	0	0	1	0	4	0	0
Paracoum	Monolign	Phenylpro	Biosynthes	Phenylpro	NA	1	2	1.1	1	1	1	1	2	1	1	1	3
NA	NA	NA	NA	NA	NA	2	1	0.7	3	2	1	2	1	1	0	2	1
NA	NA	NA	NA	NA	NA	1	1	0.8	3	0	1	0	1	0	4	3	0
NA	NA	NA	NA	NA	NA	1	2	1.2	1	2	3	0	1	1	1	1	1
Quaternar	Organonit	Organic ni	NA	NA	map04714	1	1	0.7	1	1	1	1	1	1	1	1	1
NA	NA	NA	NA	NA	NA	1	1	1.3	1	2	1	1	1	1	1	1	1
NA	NA	NA	NA	NA	NA	1	1	0.7	1	1	1	1	2	1	1	1	75
Bufanolide	Steroids	Terpenoid	NA	NA	NA	1	1	0.8	1	1	1	1	1	1	1	1	1
Chlorins	Tetrapyrro	Organohe	Metabolisı	Porphyrin	map0086C	1	1	1.7	1	2	2	2	1	2	1	1	1
NA	NA	NA	NA	NA	NA	2	1	1	3	2	1	1	0	1	1	1	3
NA	NA	NA	Amino aci	Arginine a	map0033C	1	1	0.8	1	0	1	0	1	6	2	1	0
NA	NA	NA	NA	NA	NA	1	1	1.8	1	2	3	2	1	1	1	1	6
NA	NA	NA	NA	NA	NA	2	0	0.3	0	3	6	2	1	1	2	0	0
NA	NA	NA	NA	NA	NA	1	1	1.8	1	2	1	2	1	1	1	1	1
NA	NA	NA	NA	NA	NA	61	1	0.8	1	1	1	1	1	1	1	1	1
NA	NA	NA	NA	NA	NA	1	1	0.7	1	1	1	1	2	2	1	1	3
NA	Coumarins	Coumarins	NA	NA	NA	1	1	23	1	1	74	0	0	3	0	1	2
NA	NA	NA	NA	NA	NA	2	2	0.6	2	1	1	2	1	1	0	2	1
NA	NA	NA	NA	NA	NA	1	1	2.1	1	2	1	2	1	1	1	1	1
Glyceroph	Glyceroph	Glyceroph	NA	NA	NA	1	1	1.2	1	2	1	1	4	1	1	1	3
NA	NA	NA	NA	NA	NA	1	1	1.9	1	2	1	2	1	1	1	2	1
Glyceroph	Glyceroph	Glyceroph	NA	NA	NA	0	2	5.3	1	1	2	2	5	0	0	5	1
Terpenoid	Alkaloids c	Alkaloids	NA	NA	NA	1	1	1.2	1	1	1	1	1	1	1	2	1
Benzoic ac	Benzene a	Benzene a	Amino aci	Tryptopha	map0038C	1	1	0.6	1	1	0	2	1	1	1	1	1

NA	NA	NA	NA	NA	NA	1	1	0.8	2	1	1	0	1	0	2	1	0
NA	NA	NA	NA	NA	NA	1	1	0.6	0	1	1	2	1	1	1	0	1
NA	NA	NA	NA	NA	NA	1	1	1.7	1	1	1	2	1	1	1	1	1
NA	NA	NA	NA	NA	NA	1	1	0.4	24	1	0	0	3	1	7	1	2
						0	0	0	0	0	0	0	0	0	12	0	0
PR0103 C1	PR01 Isopı	Prenol lipi	NA	NA	NA	1	1	2.5	1	2	2	2	1	1	2	2	0
NA	NA	NA	NA	NA	NA	1	1	2.5	1	2	1	2	1	1	1	2	0
NA	NA	NA	NA	NA	NA	3	3	1.2	3	4	25	2	0	0	0	1	11
NA	NA	NA	NA	NA	NA	1	1	1	1	1	1	0	1	1	2	1	1
						0	0	0	0	0	0	0	0	0	0	0	0
NA	NA	NA	NA	NA	NA	17	1	1	1	1	1	2	1	1	1	1	1
Anilides	Benzene a	Benzene a	NA	NA	NA	1	1	1.1	1	1	1	2	1	1	1	1	1
1,4-benzo	Benzodiaz	Organohe	NA	NA	NA	1	1	0.8	1	1	1	1	1	1	1	1	1
NA	NA	NA	NA	NA	NA	1	1	1.6	1	2	1	2	1	1	1	2	1
						0	0	0	0	5	0	0	0	11	24	0	2
NA	NA	NA	NA	NA	NA	1	1	0.8	1	0	1	0	2	1	1	2	1
NA	NA	NA	NA	NA	NA	1	1	1.5	1	2	3	2	1	2	1	1	1
NA	NA	NA	NA	NA	NA	2	1	0.4	2	1	0	1	0	1	2	0	1
NA	NA	NA	NA	NA	NA	1	1	1.4	1	2	1	1	1	1	1	2	1
NA	NA	NA	NA	NA	NA	1	1	1.9	1	2	1	2	1	1	1	2	1
NA	NA	NA	NA	NA	NA	1	1	0.9	7	1	1	1	1	5	1	1	1
Cinnamic a	Cinnamic a	Polyketide	NA	NA	NA	2	3	9.5	0	1	3	4	1	1	1	1	1
NA	NA	NA	NA	NA	NA	1	1	1.4	1	1	1	1	1	1	1	2	1
Carboximi	Carboximi	Organic ac	NA	NA	NA	4	1	1	1	4	1	1	0	1	1	2	0
NA	NA	NA	NA	NA	NA	1	1	0.9	1	1	1	1	1	1	1	1	1
NA	NA	NA	NA	NA	NA	2	1	1.4	1	1	2	2	1	1	1	1	9
NA	NA	NA	NA	NA	NA	1	1	0.9	1	2	1	1	1	31	1	1	2
NA	NA	NA	NA	NA	NA	1	1	1.7	1	2	1	2	2	2	1	2	1
NA	NA	NA	NA	NA	NA	1	1	0.8	2	1	2	1	2	1	1	2	1
NA	NA	NA	NA	NA	NA	0	1	3.4	2	1	2	1	4	0	0	4	1
NA	NA	NA	NA	NA	NA	1	1	0.9	1	0	1	0	1	1	1	2	0
Benzoic ac	Benzene a	Benzene a	Amino aci	Phenylalar	map0036C	2	1	1	2	1	1	0	1	0	1	1	0
NA	NA	NA	NA	NA	NA	1	1	0.8	1	0	1	1	2	1	1	2	0
NA	NA	NA	NA	NA	NA	1	4	0.2	5	0	2	0	40	1	1	1	5
						0	0	0	0	3	2	0	0	16	0	0	0
Amino aci	Carboxylic	Amino aci	NA	NA	NA	1	1	1.6	1	2	1	2	1	1	1	1	2
NA	NA	NA	NA	NA	NA	1	1	1.9	1	2	1	2	1	1	1	2	0
NA	NA	NA	NA	NA	NA	2	1	1.1	3	0	1	0	1	0	1	0	0
NA	NA	NA	NA	NA	NA	1	1	2	1	2	1	2	1	1	1	2	1
NA	NA	NA	NA	NA	NA	1	1	1.9	1	2	1	2	1	1	1	2	0
NA	NA	NA	NA	NA	NA	1	1	1.4	3	0	0	0	0	0	3	0	0
Halophen	Phenols	Phenols ar	Xenobiotic	Metabolisı	map0098C	1	1	1.7	1	2	1	2	1	1	1	2	1
Amino aci	Carboxylic	Amino aci	NA	NA	NA	1	2	1.8	2	2	1	2	1	1	1	2	1
NA	NA	NA	NA	NA	NA	0	0	12	0	3	3	2	1	0	1	0	1
NA	NA	NA	NA	NA	NA	2	0	0.5	3	0	1	0	1	1	6	3	3
NA	NA	NA	NA	NA	NA	0	1	0.6	1	0	1	0	6	21	3	0	0
NA	NA	NA	NA	NA	NA	0	1	0.7	1	1	1	8	4	68	1	1	3
NA	NA	NA	NA	NA	NA	1	1	1.7	2	1	1	1	1	1	1	2	1
NA	NA	NA	NA	NA	NA	1	1	1.2	1	1	1	0	1	1	1	1	1
						0	0	0	0	0	0	0	0	0	0	0	13
NA	NA	NA	NA	NA	NA	2	0	0.5	1	5	1	1	1	##	1	1	4
NA	NA	NA	NA	NA	NA	1	1	1.6	1	1	1	2	1	1	1	1	1
NA	NA	NA	NA	NA	NA	1	1	1.6	1	1	1	2	1	1	1	1	3
NA	NA	NA	NA	NA	NA	1	1	0.6	1	1	1	1	2	0	1	0	4
NA	NA	NA	NA	NA	NA	1	1	1.2	1	2	1	1	1	1	1	1	1
NA	NA	NA	NA	NA	NA	1	1	1.7	1	1	22	2	1	1	1	1	1
NA	NA	NA	NA	NA	NA	2	1	0.9	3	1	1	0	1	0	3	1	0
Fatty acid	Fatty Acyls	Fatty acyls	NA	NA	NA	1	1	0.9	0	0	0	0	1	0	3	1	1

NA	NA	NA	NA	NA	NA	1	1	2	1	2	1	2	1	1	1	2	1
NA	NA	NA	NA	NA	NA	1	1	0.8	1	6	33	1	2	1	1	4	1
NA	NA	NA	NA	NA	NA	1	1	1.5	1	1	1	2	1	1	2	1	1
NA	NA	NA	NA	NA	NA	1	1	1.2	1	1	1	1	1	4	1	1	1
NA	Phthalide i	Alkaloids a	Biosynthes	Isoquinolir	NA	1	1	1.2	4	0	1	0	1	0	1	1	0
NA	NA	NA	NA	NA	NA	1	1	0.3	2	0	0	0	2	0	1	0	0
NA	NA	NA	NA	NA	NA	1	1	2.1	1	2	1	2	1	1	1	2	1
Estrane ste	Steroids ar	Steroids ar	NA	NA	NA	1	1	2.7	1	2	3	1	1	1	2	1	1
NA	NA	NA	NA	NA	NA	1	1	2.4	0	8	1	7	1	1	2	2	5
						0	0	0	0	0	0	0	0	0	0	0	0
NA	NA	NA	NA	NA	NA	1	1	0.8	1	0	1	0	2	1	1	2	1
NA	NA	NA	NA	NA	NA	1	2	1.7	1	1	3	1	1	1	1	1	4
NA	NA	NA	NA	NA	NA	1	1	1.4	2	1	1	0	1	1	2	1	0
NA	NA	NA	NA	NA	NA	1	1	0.7	1	1	1	0	1	17	1	1	1
NA	NA	NA	NA	NA	NA	1	1	0.8	1	1	1	1	1	1	1	4	1
NA	NA	NA	NA	NA	NA	1	8	1.1	1	1	1	1	1	1	1	1	1
NA	NA	NA	NA	NA	NA	1	1	1.9	1	1	1	2	1	1	1	2	1
Imidazoles	Azoles	Imidazoles	NA	NA	NA	63	1	1.3	1	17	1	2	1	1	1	1	1
NA	NA	NA	NA	NA	NA	1	1	1	1	1	1	3	1	2	1	1	3
NA	NA	NA	NA	NA	NA	1	1	1.3	1	1	1	1	1	1	1	1	1
NA	NA	NA	NA	NA	NA	5	1	0.9	2	1	1	1	0	9	0	0	1
NA	NA	NA	NA	NA	NA	1	3	1	##	1	1	10	1	1	11	16	2
NA	NA	NA	NA	NA	NA	0	1	0.7	0	0	0	1	2	1	0	0	4
NA	NA	NA	NA	NA	NA	1	1	1.4	1	2	2	2	1	1	1	1	1
NA	NA	NA	NA	NA	NA	1	1	1.8	1	2	1	3	1	1	1	2	0
Benzenesu	Benzene a	Benzene a	NA	NA	NA	1	1	2.4	1	2	1	1	1	0	1	2	0
Tetraterpe	Prenol lipi	Terpenoid	NA	NA	NA	1	1	0.8	1	1	1	1	3	2	1	1	2
NA	NA	NA	NA	NA	NA	0	1	0.4	1	1	1	1	3	5	7	2	3
NA	NA	NA	NA	NA	NA	1	2	1	1	1	1	0	1	1	1	1	1
NA	NA	NA	NA	NA	NA	1	1	1.6	1	1	1	2	2	2	1	1	4
Betaxanthi	Betalains	Amino aci	NA	NA	NA	1	2	0.6	3	1	0	0	1	0	2	1	1
NA	Morphinar	Alkaloids a	NA	NA	NA	1	1	0.8	1	1	1	1	1	7	1	1	1
NA	NA	NA	NA	NA	NA	1	1	1.9	1	2	1	2	1	1	1	2	1
NA	NA	NA	NA	NA	NA	1	1	1.8	1	2	2	2	1	1	1	1	1
						0	0	0	0	0	0	22	0	0	0	0	0
NA	NA	NA	NA	NA	NA	1	1	1.1	1	0	13	1	1	1	1	1	1
NA	NA	NA	NA	NA	NA	1	1	1.7	1	2	1	2	1	1	1	2	1
Sesquiterp	Prenol lipi	Terpenoid	NA	NA	NA	1	1	0.9	1	1	1	1	1	1	1	1	1
Carbohydr	Organoox	Carbohydr	NA	NA	NA	1	1	1.9	1	2	1	2	1	1	1	2	1
NA	NA	NA	NA	NA	NA	2	1	0.4	4	0	1	1	2	0	1	0	2
NA	NA	NA	NA	NA	NA	1	1	1.8	1	2	1	2	1	1	1	2	1
NA	NA	NA	NA	NA	NA	2	0	0.6	2	1	1	1	1	0	2	1	1
						0	0	0	0	0	0	0	0	16	0	0	0
NA	NA	NA	NA	NA	NA	1	1	1.4	1	1	1	1	1	14	1	1	1
NA	NA	NA	NA	NA	NA	2	1	0.5	1	0	1	0	1	1	1	1	1
NA	NA	NA	NA	NA	NA	0	2	0.1	2	4	1	3	1	0	1	0	0
NA	NA	NA	NA	NA	NA	2	1	0.7	1	1	1	0	1	2	1	1	1
NA	NA	NA	NA	NA	NA	1	1	0.5	2	0	1	1	0	1	1	1	0
NA	NA	NA	NA	NA	NA	1	6	0.2	0	2	13	0	1	0	2	0	0
NA	NA	NA	NA	NA	NA	1	1	0.5	1	1	1	1	1	2	2	1	1
NA	NA	NA	NA	NA	NA	1	1	1.2	1	2	1	2	1	1	1	1	1
NA	NA	NA	NA	NA	NA	1	1	1.1	2	1	1	1	2	2	1	62	0
FA0201 12	FA02 Octa	Fatty acyls	Lipid meta	alpha-Linc	map00592	1	1	0.5	1	1	1	5	1	1	1	1	1
NA	NA	NA	NA	NA	NA	1	1	1	1	1	1	2	2	1	1	1	1
Glyceroph	Glyceroph	Glyceroph	NA	NA	NA	1	1	1.4	1	4	1	4	2	1	1	1	1
NA	NA	NA	NA	NA	NA	1	2	1	1	1	1	1	1	1	1	1	1
NA	NA	NA	NA	NA	NA	8	1	1.1	1	1	3	1	1	1	1	1	1
NA	NA	NA	NA	NA	NA	1	1	1.7	1	2	1	2	1	1	1	3	0

NA	NA	NA	NA	NA	NA	10	2	1.5	1	1	1	1	1	1	1	1	2
NA	NA	NA	NA	NA	NA	1	1	1.1	1	1	1	1	1	1	1	1	0
NA	NA	NA	Global anc	Biosynthes	NA	1	1	0.8	1	1	1	1	0	1	1	1	1
NA	NA	NA	NA	NA	NA	1	1	1.1	1	1	1	1	1	1	1	1	2
NA	NA	NA	NA	NA	NA	1	2	0.7	1	1	0	0	0	0	2	0	0
Fatty acid	Fatty Acyls	Fatty acyls	NA	NA	NA	0	0	0.4	0	1	0	0	0	3	5	0	21
NA	NA	NA	NA	NA	NA	1	1	1	1	1	2	1	1	7	1	1	1
NA	NA	NA	NA	NA	NA	1	1	0.9	1	1	1	2	2	0	1	1	1
NA	NA	NA	NA	NA	NA	2	1	0.7	5	1	1	1	1	1	1	1	1
NA	NA	NA	NA	NA	NA	1	2	1.5	2	1	1	2	20	1	1	1	1
NA	NA	NA	NA	NA	NA	1	1	1.7	2	2	3	2	1	1	1	2	1
NA	NA	NA	NA	NA	NA	1	1	0.8	1	1	1	1	1	1	1	1	1
NA	NA	NA	NA	NA	NA	1	1	1.4	1	1	1	2	1	1	1	1	0
NA	NA	NA	NA	NA	NA	1	1	0.6	3	0	0	0	0	0	4	1	0
Thiadiazol	Azoles	Organohe	NA	NA	NA	1	1	0.7	1	1	1	1	2	2	1	1	1
Glyceroph	Glyceroph	Glyceroph	NA	NA	NA	3	1	0.2	1	4	0	3	1	0	1	0	2
NA	NA	NA	NA	NA	NA	1	28	0.8	1	1	1	1	1	1	1	1	2
NA	NA	NA	NA	NA	NA	1	1	1.6	1	2	1	2	1	1	1	1	1
NA	Tetrahydr	Organohe	NA	NA	NA	1	1	2.3	1	2	1	2	1	1	1	2	1
NA	NA	NA	NA	NA	NA	1	1	0.7	1	1	3	1	1	2	1	1	1
NA	NA	NA	NA	NA	NA	1	1	2	1	2	1	2	1	1	1	2	1
NA	NA	NA	NA	NA	NA	1	1	1.5	1	2	1	2	1	1	1	1	1
NA	NA	NA	NA	NA	NA	2	1	1.4	6	1	1	1	0	1	4	1	1
NA	NA	NA	NA	NA	NA	1	1	0.9	2	1	1	0	2	1	1	2	1
NA	NA	NA	NA	NA	NA	2	1	0.7	1	1	1	1	1	1	1	1	1
						0	0	0	0	##	0	0	0	4	0	0	0
NA	Benzofura	Organohe	Global anc	Biosynthes	NA	1	1	1.2	1	4	1	2	1	1	1	1	1
NA	Benzoxadi	Organohe	NA	NA	NA	2	2	1.9	2	1	1	0	6	0	2	0	1
NA	NA	NA	NA	NA	NA	1	1	0.9	1	1	1	0	1	1	1	1	1
Flavonoid	Flavonoids	Flavonoids	NA	NA	NA	1	1	1.3	1	2	1	1	1	1	1	2	1
Quinoline	Quinolines	Organohe	NA	NA	NA	1	1	1.6	1	1	1	1	1	1	2	1	1
Amino aci	Carboxylic	Amino aci	Biosynthes	Monobact	map0110(	1	1	0.6	0	0	0	1	0	2	5	1	0
Benzoic ac	Benzene a	Benzene a	NA	NA	NA	1	1	1.2	1	3	1	5	1	1	1	1	0
NA	Coumarins	Coumarins	Biosynthes	Phenylpro	NA	3	0	1.4	2	1	4	2	1	1	0	2	2
Triradylcgl	Glycerolipi	Glycerolipi	NA	NA	NA	1	1	39	1	2	1	2	1	1	1	##	1
NA	NA	NA	NA	NA	NA	1	0	0.4	1	0	2	2	3	1	0	1	2
Fatty acid	Fatty Acyls	Fatty acyls	NA	NA	NA	0	0	0.3	1	5	0	1	1	7	0	1	33
NA	NA	NA	NA	NA	NA	1	0	2	2	1	1	1	0	1	8	1	2
Isoindoline	Isoindoles	Indole anc	NA	NA	NA	1	1	0.9	2	1	1	1	1	1	1	1	1
Amines	Organonit	Amines	Amino aci	Arginine a	map00330	2	1	1.9	2	3	3	2	2	1	0	2	0
Glyceroph	Glyceroph	Glyceroph	NA	NA	NA	1	1	1.7	1	2	1	2	3	1	1	1	4
NA	NA	NA	NA	NA	NA	1	0	4.5	3	5	95	1	0	0	0	0	2
NA	NA	NA	NA	NA	NA	1	1	1.1	1	1	1	1	1	2	1	1	1
NA	NA	NA	NA	NA	NA	1	1	1.1	1	1	1	1	1	1	1	1	1
NA	NA	NA	NA	NA	NA	1	2	0.4	0	2	40	2	1	4	0	1	7
NA	NA	NA	NA	NA	NA	2	1	1.3	3	0	1	0	1	0	2	0	0
NA	NA	NA	NA	NA	NA	1	1	0.9	2	1	1	1	1	1	2	3	1
NA	NA	NA	NA	NA	NA	2	1	0.8	1	1	24	2	2	1	2	1	3
NA	NA	NA	NA	NA	NA	1	1	0.7	1	1	1	1	2	1	1	1	1
NA	NA	NA	NA	NA	NA	3	1	0.5	2	1	1	1	2	1	0	0	1
NA	NA	NA	NA	NA	NA	1	1	0.9	2	1	1	0	1	1	1	1	1
NA	NA	NA	NA	NA	NA	1	1	1.2	1	1	1	1	1	1	1	1	1
NA	Nucleosid	Nucleic ac	NA	NA	NA	5	3	7.3	1	0	1	1	1	1	1	1	1
NA	NA	NA	NA	NA	NA	1	1	0.7	2	1	1	0	1	0	2	1	0
NA	NA	NA	NA	NA	NA	1	1	0.8	2	1	1	1	1	1	2	2	1
NA	NA	NA	NA	NA	NA	2	1	1.1	1	1	1	1	1	1	1	1	1
NA	NA	NA	NA	NA	NA	1	0	0.9	1	0	1	0	1	3	1	1	0
Triradylcgl	Glycerolipi	Glycerolipi	NA	NA	NA	1	1	18	1	2	1	2	1	1	1	57	1

NA	NA	NA	NA	NA	NA	1	1	2.2	1	2	1	2	1	1	1	1	0
NA	NA	NA	NA	NA	NA	1	1	1	1	1	1	2	1	1	1	1	1
NA	NA	NA	NA	NA	NA	1	1	0.2	2	0	1	0	2	0	1	1	0
Indolines	Indoles an	Indole anc	NA	NA	NA	0	4	3.5	1	13	0	15	1	1	1	2	0
NA	NA	NA	NA	NA	NA	1	1	1.7	1	2	1	2	1	1	1	2	1
NA	NA	NA	NA	NA	NA	0	2	1.5	1	0	5	0	1	0	1	1	1
NA	NA	NA	NA	NA	NA	1	0	1.3	1	0	1	1	3	0	4	1	13
NA	NA	NA	NA	NA	NA	1	7	1.4	1	1	2	1	1	1	4	1	3
NA	NA	NA	NA	NA	NA	7	1	1.1	1	1	1	1	1	1	1	1	3
Fatty acids	Fatty Acyls	Fatty acyls	NA	NA	NA	2	1	1.1	1	2	1	1	1	2	1	2	1
NA	NA	NA	NA	NA	NA	1	1	1.9	1	2	0	1	1	1	1	2	1
NA	NA	NA	NA	NA	NA	2	1	1.8	3	3	4	6	1	1	1	3	1
NA	NA	NA	NA	NA	NA	1	0	0.4	5	0	2	0	3	0	2	1	1
NA	NA	NA	NA	NA	NA	1	1	1.2	1	2	5	1	1	1	1	2	0
						0	0	0	0	34	0	0	44	0	0	0	0
NA	NA	NA	NA	NA	NA	1	1	0.9	1	1	1	1	1	2	1	1	1
NA	NA	NA	NA	NA	NA	1	1	1.4	1	1	1	1	1	1	1	1	1
NA	NA	NA	NA	NA	NA	13	1	0.5	2	0	0	0	3	0	3	1	4
NA	NA	NA	NA	NA	NA	3	1	0.7	5	1	0	0	1	0	3	0	0
NA	NA	NA	NA	NA	NA	1	1	2.3	1	2	1	2	1	1	1	2	0
NA	NA	NA	NA	NA	NA	1	1	1.1	1	1	1	1	1	1	1	1	1
Diphenylm	Benzene a	Benzene a	NA	NA	NA	1	2	0.7	1	1	18	1	2	1	1	1	1
NA	NA	NA	NA	NA	NA	1	1	1	1	2	4	1	5	1	1	1	10
						0	0	0	0	40	0	0	9	28	6	0	0
NA	NA	NA	NA	NA	NA	1	1	1	1	1	1	2	4	2	1	1	1
NA	NA	NA	NA	NA	NA	1	1	1.1	1	1	4	1	1	1	1	1	1
Steroidal g	Steroids ar	Steroids ar	NA	NA	NA	1	1	1.6	1	2	1	2	1	1	1	2	1
NA	NA	NA	NA	NA	NA	2	2	1.8	2	2	0	3	2	0	1	2	0
NA	NA	NA	NA	NA	NA	1	1	1.1	1	1	5	1	1	1	1	1	1
NA	NA	NA	NA	NA	NA	2	1	0.7	2	1	1	0	1	0	2	1	0
NA	NA	NA	NA	NA	NA	1	1	0.8	2	0	2	0	1	0	2	2	0
NA	NA	NA	NA	NA	NA	1	1	1.3	1	2	1	1	1	1	1	1	1
Carbohydr	Organoox	Carbohydr	NA	NA	NA	1	1	1	1	4	1	12	1	1	8	1	9
Fatty acids	Fatty Acyls	Fatty acyls	NA	NA	NA	1	2	1.4	1	2	2	2	1	1	1	1	1
NA	NA	NA	NA	NA	NA	1	1	1.1	2	0	0	0	1	0	4	0	0
NA	NA	NA	NA	NA	NA	1	1	2.3	1	2	1	2	1	1	1	3	0
Naphthyri	Diazanaph	Organohe	NA	NA	NA	1	1	0.8	2	1	1	1	1	0	2	3	1
NA	NA	NA	NA	NA	NA	2	1	0.5	1	0	3	1	1	1	1	1	1
NA	NA	NA	NA	NA	NA	0	1	0.9	3	0	5	0	2	0	1	0	1
NA	NA	NA	NA	NA	NA	1	1	0.4	0	3	18	6	2	2	1	0	5
NA	NA	NA	NA	NA	NA	1	1	1	1	1	1	2	1	1	1	1	1
NA	NA	NA	NA	NA	NA	1	1	1	1	1	1	2	1	1	1	1	1
NA	NA	NA	NA	NA	NA	69	1	0.8	1	1	1	1	1	1	1	1	1
NA	NA	NA	NA	NA	NA	1	1	0.8	1	1	1	1	1	4	1	1	0
NA	NA	NA	NA	NA	NA	2	50	0.7	1	1	1	0	1	3	1	1	1
Eicosanoic	Fatty Acyls	Fatty acyls	NA	NA	NA	0	1	0.5	1	2	6	4	2	0	1	1	0
NA	NA	NA	NA	NA	NA	1	1	0.1	1	1	0	1	7	1	1	0	2
NA	NA	NA	NA	NA	NA	1	2	2.2	1	3	1	2	1	1	2	2	0
FA0110 Ar	FA01 Fatty	Fatty acyls	Metabolisn	Phosphon	map00440	1	1	1.3	1	2	1	2	1	1	1	1	0
Chalcones	Linear 1,3-	Polyketide	Biosynthes	Flavonoid	NA	8	1	0.8	1	1	1	1	1	2	1	1	1
Methoxyp	Phenols	Phenols ar	NA	NA	NA	1	1	1.4	1	5	1	1	1	1	1	2	1
NA	NA	NA	NA	NA	NA	1	1	2	1	2	1	2	1	1	1	2	1
NA	NA	NA	NA	NA	NA	1	1	2.1	1	2	2	1	69	1	17	1	1
NA	NA	NA	NA	NA	NA	1	1	1.7	1	2	17	2	1	1	2	2	0
NA	NA	NA	NA	NA	NA	1	1	4.4	1	1	26	1	1	1	1	1	1
NA	NA	NA	NA	NA	NA	2	1	0.2	4	1	0	0	1	1	2	0	1
NA	NA	NA	NA	NA	NA	1	1	2	1	2	1	2	1	1	1	2	1
NA	NA	NA	NA	NA	NA	1	1	2	1	2	1	2	1	1	1	2	1

NA	NA	NA	NA	NA	NA	1	1	0.8	2	1	0	0	0	1	2	0	0
Benzenesι	Benzene a	Benzene a	NA	NA	NA	1	11	2	1	2	1	2	1	1	1	2	0
Isoquinolir	Alkaloids (	Alkaloids	Biosynthes	Isoquinolir	NA	1	1	1.4	1	1	1	1	0	2	1	1	1
NA	Stilbenes	Polyketide	NA	NA	NA	1	1	1.9	1	2	1	2	1	1	1	2	1
NA	NA	NA	NA	NA	NA	1	4	0.2	5	0	2	0	40	1	1	1	5
NA	NA	NA	NA	NA	NA	1	1	1.8	1	2	1	2	1	1	1	2	0
NA	NA	NA	NA	NA	NA	1	0	1	0	3	3	2	1	2	3	0	0
NA	NA	NA	NA	NA	NA	1	1	1.2	1	2	1	1	1	1	1	2	1
NA	NA	NA	NA	NA	NA	1	1	1.4	1	1	0	1	1	1	1	1	1
NA	NA	NA	NA	NA	NA	1	5	7.2	1	1	2	1	0	1	5	3	1
NA	NA	NA	NA	NA	NA	2	1	1.2	3	1	1	1	0	2	1	0	2
NA	NA	NA	NA	NA	NA	1	1	1.2	1	1	1	1	1	1	1	1	1
NA	NA	NA	NA	NA	NA	1	1	0.8	2	1	1	0	1	0	2	1	0
NA	NA	NA	NA	NA	NA	1	1	1.9	1	2	1	2	1	1	1	2	1
NA	NA	NA	NA	NA	NA	1	1	1.4	1	2	1	1	1	1	1	1	5
NA	NA	NA	NA	NA	NA	0	0	0.2	2	0	0	0	5	0	2	2	14
NA	NA	NA	NA	NA	NA	1	1	0.7	1	1	1	1	2	1	1	2	1
Phenylpro	Benzene a	Benzene a	NA	NA	NA	0	0	0.1	0	3	2	6	8	6	1	0	1
NA	NA	NA	NA	NA	NA	2	1	1.7	1	2	1	2	5	1	1	2	0
NA	NA	NA	NA	NA	NA	5	1	1.8	1	1	2	1	1	1	1	2	1
NA	NA	NA	NA	NA	NA	1	1	2.8	1	2	0	2	2	1	1	1	0
NA	NA	NA	NA	NA	NA	1	2	0.4	1	2	0	1	1	8	1	1	11
1,4-benzo	Benzodiaz	Organohe	NA	NA	NA	1	1	0.9	1	1	1	1	1	2	1	1	1
Imidazolid	Azolidines	Organohe	Amino aci	Histidine r	map0034(	1	2	2.3	2	4	7	9	1	2	1	1	0
NA	NA	NA	Biosynthes	Penicillin a	map0110(	1	1	1.8	1	2	1	2	1	1	1	2	1
NA	NA	NA	NA	NA	NA	1	1	1.9	1	2	1	2	1	3	1	2	0
NA	NA	NA	NA	NA	NA	1	1	0.1	0	3	2	2	1	2	0	0	2
NA	NA	NA	NA	NA	NA	3	1	0.8	1	1	28	0	3	2	1	1	2
NA	NA	NA	NA	NA	NA	1	1	0.8	3	0	1	0	1	0	3	3	0
NA	NA	NA	NA	NA	NA	1	1	0.7	2	0	1	0	1	1	2	2	0
NA	NA	NA	NA	NA	NA	1	1	1.4	1	1	1	1	1	1	1	1	1
NA	NA	NA	NA	NA	NA	2	1	1.2	0	3	28	4	0	1	0	3	2
NA	NA	NA	NA	NA	NA	1	1	1.1	1	1	1	2	1	1	1	1	1
NA	NA	NA	NA	NA	NA	1	2	0.9	1	1	1	1	2	1	1	2	6
Amino aci	Carboxylic	Amino aci	NA	NA	NA	0	7	0.5	1	1	1	1	1	3	1	0	9
Arylsulfate	Organic sι	Organic a(	NA	NA	NA	1	1	1.5	1	2	1	2	1	1	1	1	1
Glyceroph	Glyceroph	Glyceroph	Lipid meta	Glycerolipi	map00561	0	1	4.6	0	2	50	3	1	1	1	1	1
NA	NA	NA	NA	NA	NA	1	1	1.8	1	2	1	2	1	1	1	1	1
2-Arylben:	Isoflavono	Flavonoids	NA	NA	NA	1	1	0.6	1	1	1	1	2	1	1	1	13
NA	NA	NA	NA	NA	NA	1	1	0.8	1	1	1	0	1	3	1	1	0
Ceramides	Sphingolip	Sphingolip	NA	NA	NA	2	1	0.7	3	2	1	3	1	1	0	1	1
NA	NA	NA	NA	NA	NA	1	1	15	1	9	3	16	1	1	1	6	0
Fatty acid	Fatty Acyls	Fatty acyls	NA	NA	NA	1	2	1.5	2	1	1	1	2	1	3	7	0
NA	NA	NA	NA	NA	NA	1	1	1	1	1	1	0	1	1	1	1	1
NA	NA	NA	NA	NA	NA	1	1	1.9	1	2	1	3	1	1	1	2	1
NA	NA	NA	NA	NA	NA	1	1	1.2	3	1	0	0	1	0	3	0	0
Pyrimidine	Pyrimidine	Nucleic ac	Lipid meta	Glyceroph	map00564	1	1	1.5	1	2	5	2	1	1	1	1	17
NA	NA	NA	NA	NA	NA	0	1	0.3	1	0	0	0	7	10	3	0	0
NA	NA	NA	NA	NA	NA	1	1	0.9	2	1	1	0	1	1	1	1	1
Amino aci	Carboxylic	Amino aci	NA	NA	NA	2	1	0.8	1	1	67	1	1	1	1	1	1
						0	0	0	0	0	0	0	0	0	0	0	0
NA	NA	NA	NA	NA	NA	1	1	1.4	1	2	2	1	1	1	1	2	1
NA	NA	NA	Biosynthes	Monobact	NA	1	1	0.7	1	2	1	1	1	1	1	1	0
NA	NA	NA	NA	NA	NA	1	1	0.9	1	0	1	0	1	1	1	2	0
NA	NA	NA	NA	NA	NA	34	1	0.6	1	0	1	0	1	1	1	1	1
NA	NA	NA	NA	NA	NA	1	1	0.7	4	0	1	0	1	6	1	1	4
NA	NA	NA	NA	NA	NA	1	1	2.2	1	2	1	2	1	1	1	2	0
NA	NA	NA	NA	NA	NA	1	1	0.8	1	1	8	1	1	1	1	1	1

NA	NA	NA	NA	NA	NA	2	2	0.5	1	0	1	1	3	1	1	0	1
Phenoxy c	Benzene a	Benzene a	NA	NA	NA	1	1	1	1	1	1	1	1	1	1	1	1
Carbonyl (	Organoox	Organic o	Amino aci	Cysteine a	map0027	1	1	2.8	2	1	4	1	##	0	38	1	0
NA	NA	NA	NA	NA	NA	1	1	1.8	1	2	1	2	1	1	1	1	1
Diphenylm	Benzene a	Benzene a	NA	NA	NA	0	2	1.1	4	11	2	2	5	4	1	0	1
NA	NA	NA	NA	NA	NA	1	1	1.1	1	1	1	1	1	1	1	1	1
Benzoic ac	Benzene a	Benzene a	Xenobiotic	Aminoben	map0079	1	1	1.8	2	2	1	2	2	1	2	6	0
NA	NA	NA	NA	NA	NA	1	1	0.7	1	0	1	0	1	6	1	1	1
Isoprenoic	Prenol lipi	Prenol lipi	Metabolisı	Monoterp	NA	0	1	1.6	1	0	1	1	0	1	2	11	1
Fatty acids	Fatty Acyls	Fatty acyls	NA	NA	NA	1	1	1.6	2	1	1	2	1	0	2	1	0
Amino aci	Carboxylic	Amino aci	Amino aci	Arginine b	map0022	2	1	2.8	1	0	1	13	0	1	0	1	0
NA	Pyrenes	Benzene a	Xenobiotic	Polycyclic	NA	3	1	1.3	1	1	35	1	1	1	1	1	1
NA	NA	NA	Metabolisı	Riboflavin	map0074	1	1	2	1	2	1	2	1	1	1	2	1
NA	NA	NA	NA	NA	NA	1	1	1.9	1	2	1	2	1	1	1	2	0
NA	NA	NA	NA	NA	NA	1	1	1.3	1	2	2	2	1	1	1	1	1
NA	NA	NA	NA	NA	NA	1	1	1.8	1	2	1	2	1	1	1	2	0
NA	NA	NA	NA	NA	NA	1	1	1.7	1	1	3	2	1	1	1	2	1
Arylsulfate	Organic sı	Organic a	NA	NA	NA	0	1	1.3	4	2	0	8	2	0	1	1	0
NA	Glycerolipi	Glycerolipi	NA	NA	NA	1	1	1.9	1	2	1	2	1	1	1	2	1
NA	NA	NA	NA	NA	NA	1	1	0.6	1	1	0	1	4	1	1	1	11
NA	NA	NA	NA	NA	NA	1	1	1.2	1	1	1	2	67	1	1	1	1
NA	NA	NA	NA	NA	NA	1	5	1.3	1	1	1	1	9	2	2	1	1
NA	NA	NA	NA	NA	NA	1	0	0.3	1	14	0	22	1	2	4	0	1
NA	NA	NA	NA	NA	NA	1	1	1.2	1	1	2	3	1	1	1	1	1
NA	NA	NA	NA	NA	NA	1	1	0.9	1	0	1	0	1	1	1	2	0
NA	NA	NA	NA	NA	NA	2	1	0.4	2	1	1	1	1	4	1	2	1
NA	NA	NA	Lipid meta	Glyceroph	map00564	1	1	7.9	1	1	1	0	3	1	14	0	0
NA	NA	NA	NA	NA	NA	0	14	0.4	1	1	1	1	1	11	1	0	85
NA	NA	NA	NA	NA	NA	1	1	0.8	1	1	1	1	1	1	1	1	1
						0	0	0	4	##	0	0	18	0	0	0	0
NA	NA	NA	NA	NA	NA	1	1	0.9	2	0	1	0	1	0	4	1	0
NA	NA	NA	NA	NA	NA	1	1	1.6	1	1	1	2	1	1	1	1	1
NA	NA	NA	NA	NA	NA	1	1	1.3	1	2	1	2	5	1	1	1	1
NA	NA	NA	NA	NA	NA	1	1	1.4	1	2	1	1	1	1	1	2	1
NA	NA	NA	NA	NA	NA	1	1	1.3	1	1	2	1	1	1	1	1	1
NA	NA	NA	NA	NA	NA	1	1	1.2	1	2	1	1	7	1	2	1	0
NA	NA	NA	NA	NA	NA	1	1	0.7	1	0	1	0	2	0	2	1	0
NA	NA	NA	NA	NA	NA	0	1	0.9	0	1	1	2	2	10	1	1	1
Homoisofl	Homoisofl	Flavonoids	NA	NA	NA	1	1	1	1	1	28	0	1	1	2	1	1
						0	0	0	0	0	0	0	18	0	0	0	0
						0	0	0	2	##	0	69	8	7	13	0	0
NA	NA	NA	NA	NA	NA	25	1	2.5	1	2	2	2	1	1	1	2	1
NA	NA	NA	NA	NA	NA	1	3	0.9	1	0	4	0	3	2	1	0	0
						0	0	0	0	0	0	0	0	0	0	0	0
						0	0	0	0	0	0	0	0	0	0	0	0
						0	0	0	0	0	0	0	0	0	0	0	0
						0	0	0	0	0	0	0	0	0	0	0	0
						0	0	0	0	0	0	0	0	0	0	0	0
						0	0	0	0	0	0	0	0	0	0	0	0
						0	0	0	0	0	0	0	0	0	0	0	0
						0	0	0	0	0	0	0	0	0	0	0	0
						0	0	0	0	0	0	0	0	0	0	0	0
						0	0	0	0	0	0	0	0	0	0	0	0
NA	NA	NA	NA	NA	NA	1	1	0.7	2	1	0	0	1	0	2	0	0
NA	NA	NA	NA	NA	NA	1	1	1.3	1	2	1	1	0	6	1	1	1
NA	NA	NA	NA	NA	NA	1	1	1.5	1	2	1	1	1	1	1	2	1
NA	NA	NA	NA	NA	NA	1	2	1.7	2	2	1	1	1	2	1	1	1
NA	NA	NA	NA	NA	NA	1	1	1.7	1	2	1	2	1	1	1	1	1

NA	NA	NA	NA	NA	NA	1	1	1	1	1	1	0	1	1	1	1	1
Null	PK07 Linea	Polyketide	Metabolisı	Tetracyclir	map0110(	1	1	2.1	1	2	1	2	1	1	1	2	1
Bile acids,	Steroids ar	Bile acids,	NA	NA	NA	1	1	0.8	1	1	1	1	4	2	1	1	1
NA	NA	NA	NA	NA	NA	1	1	0.9	1	1	1	1	1	1	1	1	1
NA	NA	NA	NA	NA	NA	1	1	0.7	2	1	1	1	1	1	2	2	9
NA	NA	NA	NA	NA	NA	1	1	2.1	1	2	1	2	1	1	1	2	30
NA	NA	NA	NA	NA	NA	5	1	1	1	2	1	1	1	1	1	1	1
NA	NA	NA	NA	NA	NA	1	1	1.8	1	2	1	2	1	1	1	2	0
NA	NA	NA	NA	NA	NA	1	1	1.8	1	2	1	2	1	1	1	1	52
NA	NA	NA	NA	NA	NA	1	1	0.5	6	0	1	0	3	0	2	2	0
NA	NA	NA	Biosynthes	Phenazine	NA	1	1	0.7	3	0	1	0	1	0	1	0	0
NA	NA	NA	NA	NA	NA	1	1	1	1	1	1	1	1	1	1	1	1
NA	NA	NA	NA	NA	NA	1	1	1.2	1	1	1	2	1	1	1	1	2
						0	0	0	0	0	0	##	##	22	0	0	0
NA	NA	NA	NA	NA	NA	1	1	1.1	1	1	29	1	1	1	1	1	1
NA	NA	NA	NA	NA	NA	1	1	2	1	2	1	2	1	1	1	2	8
NA	NA	NA	NA	NA	NA	1	1	1.9	1	2	1	2	1	1	1	2	1
Tropane a	Alkaloids (	Alkaloids	NA	NA	NA	3	2	1.4	1	1	3	1	1	1	1	2	1
Fatty acids	Fatty Acyls	Fatty acyls	Lipid meta	Fatty acid	map00061	2	3	2.2	1	1	11	1	4	1	1	1	2
Fatty acids	Fatty Acyls	Fatty acyls	Lipid meta	Cutin, sub	map0110(	1	1	1.6	1	1	2	2	1	2	1	2	0
NA	NA	NA	NA	NA	NA	1	1	1.7	1	2	1	2	1	1	1	2	1
NA	NA	NA	NA	NA	NA	1	1	1.1	1	1	1	1	1	1	1	1	2
NA	NA	NA	NA	NA	NA	10	0	0.5	23	6	0	1	0	2	1	2	8
NA	NA	NA	NA	NA	NA	0	4	0.5	1	0	1	1	0	##	1	1	5
						0	0	0	0	0	0	0	0	0	0	0	0
						0	0	0	0	0	0	0	0	0	0	0	0
						0	0	0	0	0	0	0	0	0	0	0	0
Amino aci	Carboxylic	Amino aci	Amino aci	Arginine a	map00330	2	1	1	2	6	1	9	1	3	1	1	1
NA	NA	NA	NA	NA	NA	1	1	1.1	1	1	1	1	1	1	1	1	1
NA	NA	NA	NA	NA	NA	2	1	0.7	2	5	3	9	1	1	1	2	1
NA	NA	NA	NA	NA	NA	1	1	1	1	2	1	2	4	2	1	1	1
NA	NA	NA	NA	NA	NA	1	1	2	4	4	1	3	1	1	1	1	0
NA	NA	NA	NA	NA	NA	1	1	1.6	0	2	0	2	1	1	2	1	1
NA	NA	NA	NA	NA	NA	1	1	1.6	1	1	1	2	1	1	1	2	1
NA	NA	NA	NA	NA	NA	1	1	1.5	1	1	1	2	2	1	1	1	1
NA	NA	NA	NA	NA	NA	1	1	0.6	3	1	0	0	1	1	3	0	1
NA	NA	NA	NA	NA	NA	1	1	2	1	2	1	2	1	1	1	2	1
NA	NA	NA	NA	NA	NA	1	1	0.9	1	1	1	0	1	1	1	1	1
NA	NA	NA	NA	NA	NA	2	0	0.3	6	0	1	0	2	0	2	1	0
Monoterp	Prenol lipi	Terpenoid	Metabolisı	Geraniol d	NA	1	1	1	3	1	3	2	1	2	0	2	2
NA	NA	NA	NA	NA	NA	1	1	3.5	1	4	13	2	1	0	0	2	0
NA	NA	NA	NA	NA	NA	1	1	1	1	1	1	1	1	3	2	1	2
NA	NA	NA	NA	NA	NA	1	1	1.1	1	2	1	2	1	1	1	3	3
						0	0	0	0	0	0	0	0	0	0	0	0
NA	NA	NA	NA	NA	NA	1	1	1.4	1	2	1	1	1	1	1	1	1
NA	NA	NA	NA	NA	NA	1	1	1.1	1	1	1	1	0	1	1	1	1
NA	NA	NA	NA	NA	NA	1	1	1.4	1	2	1	1	1	1	1	2	1
NA	NA	NA	NA	NA	NA	2	1	1.5	1	1	1	2	1	1	1	1	1
NA	NA	NA	NA	NA	NA	1	1	1.8	1	2	2	2	1	1	1	1	1
Tetraterpe	Prenol lipi	Terpenoid	NA	NA	NA	1	1	0.8	1	1	1	1	4	2	1	1	1
NA	NA	NA	NA	NA	NA	1	1	2.1	1	2	1	2	1	1	0	1	1
Fatty acid	Fatty Acyls	Fatty acyls	NA	NA	NA	1	1	3.5	1	2	1	7	1	1	3	2	1
Guanidine	Organonit	Organic ni	NA	NA	NA	1	1	2.1	1	2	1	3	1	1	2	1	1
Glyceroph	Glyceroph	Glyceroph	NA	NA	NA	1	2	1.1	1	2	23	5	1	2	1	0	4
Terpenoid	Alkaloids (	Alkaloids	NA	NA	NA	1	2	0.9	1	1	1	1	1	1	1	1	2
NA	NA	NA	NA	NA	NA	1	1	2	1	2	1	2	1	1	1	2	11
						0	0	0	0	0	0	0	40	0	0	0	0
						0	0	0	0	0	0	0	60	0	0	0	0

NA	NA	NA	NA	NA	NA	3	1	0.4	4	1	0	0	0	1	3	0	1
NA	NA	NA	NA	NA	NA	1	1	1.3	1	2	1	1	1	1	1	2	1
NA	NA	NA	NA	NA	NA	4	1	0.6	7	0	0	0	1	0	3	0	2
NA	NA	NA	NA	NA	NA	1	1	0.9	1	1	1	1	1	1	1	1	1
Benzenedi	Phenols	Phenols ar	NA	NA	NA	1	1	0.9	1	1	1	1	1	1	1	1	1
NA	NA	NA	NA	NA	NA	1	1	2	1	2	1	2	1	1	1	1	1
NA	NA	NA	NA	NA	NA	5	2	1.9	0	0	24	0	2	1	1	1	4
NA	NA	NA	NA	NA	NA	0	2	0.4	2	1	0	2	1	1	1	11	1
NA	NA	NA	NA	NA	NA	2	1	1.2	1	1	1	1	1	1	1	1	1
Fatty acids	Fatty Acyls	Fatty acyls	Lipid meta	Cutin, sub	map0104	1	1	1.7	1	4	1	2	1	4	0	2	7
NA	NA	NA	NA	NA	NA	1	2	1.5	1	1	8	1	4	1	1	1	3
NA	NA	NA	NA	NA	NA	0	0	6.1	0	3	10	1	1	0	0	0	3
NA	NA	NA	NA	NA	NA	1	1	1.9	1	2	1	2	1	1	1	2	0
NA	NA	NA	NA	NA	NA	1	1	0.7	1	0	1	0	1	1	1	1	1
NA	NA	NA	NA	NA	NA	2	1	0.7	4	0	0	1	6	1	2	1	1
NA	NA	NA	NA	NA	NA	1	1	2.7	1	1	1	2	1	1	1	1	1
Diterpenoi	Prenol lipi	Terpenoid	NA	NA	NA	0	9	0.4	0	1	0	1	18	1	2	1	2
NA	NA	NA	NA	NA	NA	##	1	2.1	1	0	0	0	0	1	0	2	4
NA	NA	NA	NA	NA	NA	1	1	0.9	1	1	1	1	1	1	1	1	1
NA	NA	NA	NA	NA	NA	1	1	1.1	2	1	1	1	1	1	1	1	1
NA	NA	NA	NA	NA	NA	1	1	1.4	0	5	2	9	1	1	1	2	1
NA	NA	NA	NA	NA	NA	12	0	3.2	22	5	0	0	0	4	1	0	5
NA	NA	NA	NA	NA	NA	1	1	3.5	1	2	1	7	1	1	3	2	1
NA	NA	NA	NA	NA	NA	1	1	1.2	1	1	1	2	1	1	1	1	1
NA	NA	NA	NA	NA	NA	1	1	0.4	4	0	0	0	1	0	2	0	0
NA	NA	NA	NA	NA	NA	2	1	2.6	1	3	1	2	1	1	1	1	1
NA	NA	NA	NA	NA	NA	1	0	0.1	1	0	2	2	2	##	2	1	2
						0	0	0	0	##	##	0	0	0	0	##	0
Phenoxy c	Benzene a	Benzene a	NA	NA	NA	1	1	1.5	3	2	2	2	1	1	1	1	31
NA	NA	NA	NA	NA	NA	0	14	0.4	1	1	1	1	1	12	1	0	37
NA	NA	NA	NA	NA	NA	1	1	1.3	1	2	1	1	1	1	1	1	16
NA	NA	NA	NA	NA	NA	8	1	1.7	1	2	1	2	1	1	1	1	1
Ceramides	Sphingolip	Sphingolip	NA	NA	NA	2	1	0.5	3	3	1	2	1	1	0	2	1
NA	NA	NA	NA	NA	NA	1	1	1.7	1	2	1	2	2	2	1	1	1
NA	NA	NA	NA	NA	NA	0	0	0.4	1	0	0	1	5	1	1	28	1
NA	NA	NA	NA	NA	NA	1	4	1.5	1	1	1	0	4	1	1	1	1
NA	NA	NA	Chemical s	Biosynthes	NA	1	2	0.4	1	1	1	3	3	1	1	1	2
NA	NA	NA	NA	NA	NA	1	1	1.6	1	5	1	10	1	0	0	1	1
Dammarer	Triterpeno	Terpenoid	NA	NA	NA	1	1	1.5	1	1	1	1	4	2	1	1	7
NA	NA	NA	NA	NA	NA	2	1	1	2	3	1	3	1	1	0	2	1
NA	NA	NA	NA	NA	NA	1	1	1.8	1	2	1	2	1	1	1	2	1
Methoxyp	Phenols	Phenols ar	Xenobiotic	Aminoben	map0110	1	1	1.9	2	0	4	0	1	0	1	3	0
NA	NA	NA	NA	NA	NA	1	1	0.7	1	0	1	1	1	7	1	1	1
NA	Naphthale	Benzene a	NA	NA	NA	1	1	20	7	0	1	0	0	0	14	4	1
NA	NA	NA	NA	NA	NA	1	1	0.8	1	1	1	1	0	2	1	1	1
NA	NA	NA	NA	NA	NA	2	1	1	2	1	1	1	1	1	0	1	1
						0	0	2	0	0	0	13	8	0	0	0	41
NA	NA	NA	Biosynthes	Phenazine	map0110	2	1	0.7	2	1	1	0	1	0	2	1	0
NA	NA	NA	NA	NA	NA	1	1	0.9	1	1	##	1	1	1	1	1	1
NA	NA	NA	NA	NA	NA	1	1	2.5	1	2	1	3	1	2	1	5	1
NA	NA	NA	NA	NA	NA	1	0	0.3	2	0	1	0	2	1	6	1	0
NA	NA	NA	NA	NA	NA	1	1	0.8	2	0	1	1	1	2	1	2	1
NA	NA	NA	NA	NA	NA	2	1	1.1	2	2	5	2	1	1	1	1	2
NA	NA	NA	NA	NA	NA	1	1	1.8	1	2	1	3	1	1	1	3	1
NA	NA	NA	NA	NA	NA	1	4	1	1	1	1	1	14	1	1	1	1
NA	NA	NA	NA	NA	NA	6	0	4.3	0	1	36	1	47	0	2	11	2
NA	NA	NA	NA	NA	NA	2	1	0.6	1	1	1	0	1	1	1	1	1
NA	NA	NA	NA	NA	NA	1	5	1.2	0	1	1	1	39	1	4	1	2

Arylsulfate	Organic su	Organic ac	NA	NA	NA	10	1	5.7	3	6	0	5	0	0	1	3	0
NA	NA	NA	NA	NA	NA	2	1	2.2	1	1	11	1	3	0	1	2	2
						0	0	0	0	0	0	0	3	7	0	0	0
NA	NA	NA	NA	NA	NA	1	1	1.5	1	1	1	3	5	1	1	1	1
						0	0	0	0	0	0	0	0	10	0	0	7
NA	NA	NA	NA	NA	NA	1	1	0.8	1	1	1	1	1	1	2	2	9
NA	NA	NA	NA	NA	NA	0	0	0.3	1	5	0	9	16	26	1	0	2
NA	NA	NA	NA	NA	NA	1	1	1.4	1	2	1	2	1	1	1	2	1
NA	NA	NA	NA	NA	NA	1	1	1	1	1	1	1	1	1	1	1	1
NA	NA	NA	NA	NA	NA	1	2	2.4	2	0	1	0	2	0	2	2	0
Indolyl car	Indoles an	Indole anc	NA	NA	NA	1	2	2.4	2	0	1	0	2	0	2	2	0
NA	NA	NA	NA	NA	NA	2	1	0.3	1	1	0	1	1	0	0	0	1
NA	NA	NA	NA	NA	NA	1	1	0.8	1	0	8	1	1	1	1	1	1
						0	0	0	0	0	0	0	0	0	0	0	0
						0	0	0	0	0	0	0	0	0	0	0	8
NA	NA	NA	NA	NA	NA	1	5	0.5	0	1	0	0	0	1	0	1	0
NA	NA	NA	NA	NA	NA	2	1	0.6	3	1	1	0	1	1	1	0	1
NA	NA	NA	NA	NA	NA	1	1	1.3	7	1	1	0	3	0	13	2	0
Cholestan	Steroids ar	Steroids ar	NA	NA	NA	1	1	1.5	1	1	1	1	1	1	2	3	0
NA	NA	NA	NA	NA	NA	1	2	0.4	1	3	0	2	1	3	2	0	1
NA	NA	NA	NA	NA	NA	3	1	2	1	6	8	3	1	3	1	1	1
NA	NA	NA	NA	NA	NA	1	1	0.5	0	1	1	9	1	1	1	1	1
						0	0	0	0	43	23	13	##	51	##	2	15
						0	0	0	0	0	0	0	##	0	28	0	0
NA	NA	NA	NA	NA	NA	1	5	1.3	1	4	1	1	0	2	2	1	0
NA	NA	NA	NA	NA	NA	0	5	1.8	2	1	1	1	1	2	0	2	2
NA	NA	NA	NA	NA	NA	2	1	0.6	1	4	0	1	0	1	2	1	0
NA	NA	NA	NA	NA	NA	1	1	1.7	1	2	1	2	1	1	1	1	0
Carbohydr	Organoox	Carbohydr	NA	NA	NA	1	1	0.8	1	0	1	0	1	1	1	2	1
NA	NA	NA	NA	NA	NA	1	1	0.5	3	1	1	0	0	4	1	0	2
NA	NA	NA	NA	NA	NA	1	2	0.5	1	1	1	0	2	2	2	1	2
Steroidal	Steroids ar	Steroids ar	NA	NA	NA	1	1	0.2	1	0	2	0	2	2	0	1	3
NA	NA	NA	NA	NA	NA	1	1	2	1	2	1	2	1	1	1	2	1
NA	NA	NA	NA	NA	NA	1	1	1.3	1	23	1	2	5	2	1	1	1
NA	NA	NA	NA	NA	NA	0	0	1.2	1	1	1	1	0	48	1	1	2
NA	NA	NA	NA	NA	NA	1	1	0.6	1	1	1	1	1	5	2	1	1
NA	NA	NA	NA	NA	NA	1	1	1	1	1	1	1	1	1	1	1	1
NA	NA	NA	NA	NA	NA	18	1	1.9	1	3	1	3	1	1	1	2	1
NA	NA	NA	NA	NA	NA	1	1	2.7	1	1	1	2	1	1	1	2	2
NA	NA	NA	NA	NA	NA	1	1	1.7	1	2	1	2	2	1	1	1	1
NA	NA	NA	NA	NA	NA	1	1	0.5	2	1	1	1	1	0	2	0	2
NA	NA	NA	NA	NA	NA	1	1	1.5	1	2	1	2	1	1	1	2	1
NA	NA	NA	Xenobiotic	Toluene d	NA	1	0	1	7	0	12	0	13	0	1	10	0
NA	NA	NA	NA	NA	NA	1	1	5.2	1	2	2	2	1	1	1	1	1
Betacyanir	Betalains	Amino aci	Biosynthes	Betalain bi	NA	2	1	1.8	1	2	1	2	1	1	1	1	1
Arylsulfate	Organic su	Organic ac	NA	NA	NA	5	1	1.8	1	2	1	2	37	1	1	1	1
NA	NA	NA	NA	NA	NA	1	0	0.9	1	2	5	1	2	1	1	1	32
Amines	Organonit	Amines	Lipid meta	Glyceroph	map00564	2	1	3.7	2	3	2	4	0	1	1	3	0
Anisoles	Phenol eth	Benzene a	NA	NA	NA	1	1	0.5	1	1	1	1	1	##	1	18	3
NA	NA	NA	NA	NA	NA	2	1	0.4	1	2	2	1	1	2	0	1	1
NA	NA	NA	NA	NA	NA	1	1	2.5	1	2	4	3	1	2	1	2	0
NA	NA	NA	NA	NA	NA	1	1	1.8	1	2	2	2	1	1	1	1	1
NA	NA	NA	NA	NA	NA	0	1	1.4	2	1	0	0	1	1	1	1	0
NA	NA	NA	NA	NA	NA	1	1	1.5	3	1	2	2	0	1	1	1	66
NA	NA	NA	NA	NA	NA	2	0	0.3	3	0	0	0	6	0	2	0	1
NA	NA	NA	NA	NA	NA	0	0	35	0	7	27	2	1	0	0	0	8
NA	NA	NA	NA	NA	NA	0	10	0.1	9	0	0	1	3	4	0	0	3
NA	NA	NA	NA	NA	NA	2	1	1.6	1	2	1	2	1	1	1	1	1

NA	NA	NA	NA	NA	NA	6	1	1.7	1	6	1	5	1	1	1	2	1
NA	NA	NA	NA	NA	NA	1	1	1.9	1	2	1	2	1	1	1	2	1
NA	NA	NA	NA	NA	NA	0	2	0.6	1	1	1	0	3	0	1	1	2
NA	NA	NA	NA	NA	NA	0	14	0.2	1	0	0	0	1	1	0	2	17
NA	NA	NA	NA	NA	NA	1	1	0.7	3	0	1	0	1	0	3	3	0
NA	NA	NA	NA	NA	NA	1	1	2	1	2	0	1	1	1	1	2	1
NA	NA	NA	NA	NA	NA	1	1	1.2	1	1	2	3	1	1	2	1	1
NA	NA	NA	NA	NA	NA	1	1	3.7	1	1	30	1	1	1	1	2	1
NA	Purine nuc	Purines an	NA	NA	NA	1	1	1.1	1	1	1	1	1	1	1	1	1
NA	NA	NA	NA	NA	NA	1	1	0.2	1	6	0	2	1	2	3	1	1
Carbohydr	Organoox	Carbohydr	NA	NA	NA	4	1	1.7	6	2	2	2	1	1	1	1	2
NA	NA	NA	NA	NA	NA	1	1	1	1	1	1	0	1	1	1	1	1
NA	Quinolines	Organohe	NA	NA	NA	2	1	0.8	3	1	5	4	2	1	0	2	2
NA	NA	NA	NA	NA	NA	1	1	0.8	3	1	4	3	3	1	0	2	1
Dibenzoxe	Benzoxepi	Organohe	NA	NA	NA	1	1	1	4	1	0	0	0	2	10	1	0
NA	NA	NA	NA	NA	NA	1	1	2.1	0	4	1	8	1	1	3	2	0
NA	NA	NA	NA	NA	NA	1	0	0.4	1	0	1	1	1	##	1	2	1
NA	NA	NA	NA	NA	NA	1	1	1	1	1	1	1	1	1	1	1	1
NA	NA	NA	NA	NA	NA	1	1	2	1	2	1	2	1	1	1	2	1
NA	NA	NA	NA	NA	NA	1	1	1.4	1	1	2	2	1	1	1	1	1
NA	NA	NA	NA	NA	NA	1	1	1.4	1	1	1	1	1	1	1	2	1
NA	Purine nuc	Purines an	NA	NA	NA	1	5	0.1	1	0	2	1	5	0	1	1	2
Amino aci	Carboxylic	Amino aci	NA	NA	NA	1	2	1.3	0	2	1	2	2	2	0	1	1
NA	NA	NA	NA	NA	NA	1	2	1.2	1	2	56	2	1	1	2	2	1
NA	NA	NA	NA	NA	NA	1	1	2	1	2	1	2	1	1	1	2	1
NA	NA	NA	NA	NA	NA	2	1	0.2	0	0	1	4	1	2	0	1	16
NA	NA	NA	NA	NA	NA	2	1	1.9	1	2	1	2	1	1	1	2	1
NA	NA	NA	NA	NA	NA	2	2	0.1	1	0	5	0	3	0	4	1	4
NA	NA	NA	NA	NA	NA	1	1	1.8	1	2	1	2	1	1	1	1	1
NA	NA	NA	NA	NA	NA	2	2	1	0	1	41	1	2	0	3	0	5
NA	NA	NA	NA	NA	NA	1	1	0.8	1	1	1	1	1	2	2	1	1
Glyceroph	Glyceroph	Glyceroph	NA	NA	NA	0	0	0.4	1	3	0	3	2	2	1	1	9
NA	NA	NA	NA	NA	NA	6	2	1	1	0	0	1	3	1	1	1	2
NA	NA	NA	NA	NA	NA	1	1	0.9	1	1	2	1	1	1	1	1	23
Dammarer	Triterpeno	Terpenoid	NA	NA	NA	1	1	0.6	3	1	0	0	0	0	2	0	1
NA	NA	NA	NA	NA	NA	2	1	1.8	1	2	1	2	1	1	1	1	1
NA	NA	NA	NA	NA	NA	1	6	0.5	1	1	1	1	11	4	1	0	4
NA	NA	NA	NA	NA	NA	1	1	1	1	1	1	1	1	1	8	1	1
NA	NA	NA	NA	NA	NA	1	1	1.5	1	1	1	2	1	2	1	1	1
NA	NA	NA	NA	NA	NA	1	1	1.8	1	2	2	2	1	1	1	1	1
Fatty acid	Fatty Acyls	Fatty acyls	NA	NA	NA	1	1	2.5	1	2	1	2	1	1	1	2	0
						0	0	0	0	0	0	0	0	0	0	0	0
NA	NA	NA	NA	NA	NA	2	11	1.2	1	2	1	2	1	1	1	1	1
NA	NA	NA	NA	NA	NA	57	1	1.6	5	25	2	##	1	1	3	49	1
NA	NA	NA	NA	NA	NA	1	1	0.9	1	1	1	2	2	0	1	1	1
NA	NA	NA	NA	NA	NA	0	3	0.5	1	0	1	0	5	0	4	0	2
NA	NA	NA	NA	NA	NA	1	1	1.8	1	4	1	39	3	6	2	1	1
						0	0	0	0	6	0	0	0	0	0	0	0
NA	NA	NA	NA	NA	NA	1	1	0.9	1	1	1	0	1	2	1	1	1
NA	NA	NA	NA	NA	NA	1	1	0.7	1	2	1	1	1	6	1	1	2
NA	NA	NA	NA	NA	NA	0	2	3	1	3	2	2	0	2	1	4	0
NA	NA	NA	NA	NA	NA	2	0	2	2	0	0	1	0	0	2	2	2
Anthocyar	Flavonoids	Flavonoids	Biosynthes	Anthocyar	NA	1	1	1.9	1	2	1	2	1	1	1	2	1
NA	NA	NA	NA	NA	NA	1	2	1	1	2	1	2	2	1	1	1	0
NA	NA	NA	NA	NA	NA	2	1	0.7	1	0	1	1	1	1	1	1	12
NA	NA	NA	NA	NA	NA	0	1	0.1	0	0	0	1	2	0	2	1	2
NA	NA	NA	NA	NA	NA	1	1	0.9	1	1	1	1	1	1	1	1	1
						0	0	0	0	7	0	0	0	0	20	0	0

NA	NA	NA	NA	NA	NA	1	1	1.6	1	2	1	2	1	1	1	2	1
NA	NA	NA	NA	NA	NA	1	1	3.3	1	1	1	1	0	7	2	1	1
NA	NA	NA	NA	NA	NA	1	1	1.1	1	1	1	1	1	1	1	1	1
Amino aci	Carboxylic	Amino aci	NA	NA	NA	1	2	0.8	2	1	1	1	1	1	1	2	1
NA	NA	NA	NA	NA	NA	1	1	0.5	1	1	1	2	1	1	1	1	1
NA	NA	NA	NA	NA	NA	1	1	1.2	1	1	1	2	1	1	1	1	1
NA	NA	NA	NA	NA	NA	6	1	0.8	2	3	15	5	1	0	0	2	1
NA	NA	NA	NA	NA	NA	1	1	0.8	1	2	1	2	1	1	0	1	2
NA	NA	NA	NA	NA	NA	3	0	1.6	1	6	##	21	0	1	1	7	1
NA	NA	NA	NA	NA	NA	1	0	0.6	1	0	0	0	2	2	1	0	15
NA	NA	NA	NA	NA	NA	0	0	27	0	5	8	2	1	1	1	0	2
NA	NA	NA	NA	NA	NA	0	0	50	0	10	19	2	1	1	1	0	3
NA	NA	NA	NA	NA	NA	1	1	2.4	1	2	1	1	1	1	1	2	1
NA	NA	NA	NA	NA	NA	1	1	1.1	1	1	1	1	1	1	1	1	67
NA	NA	NA	NA	NA	NA	2	1	0.7	1	1	1	0	1	2	1	1	1
						0	0	0	0	7	0	0	8	11	0	0	0
NA	NA	NA	NA	NA	NA	1	2	0.6	4	2	1	0	0	0	2	4	0
NA	NA	NA	NA	NA	NA	2	1	1.1	1	1	1	1	1	1	1	1	1
NA	NA	NA	NA	NA	NA	1	2	0.8	1	1	1	1	2	2	1	1	3
NA	NA	NA	NA	NA	NA	0	1	1.8	2	0	1	1	0	2	0	1	2
Ceramides	Sphingolip	Sphingolip	NA	NA	NA	2	1	0.6	3	3	1	2	1	1	0	1	1
NA	NA	NA	NA	NA	NA	0	6	0.3	1	0	1	0	0	0	1	0	0
NA	NA	NA	NA	NA	NA	1	1	1.5	1	1	1	##	3	1	1	1	##
Pyrroloind	Indoles an	Indole anc	NA	NA	NA	3	1	1.3	1	2	1	2	1	1	1	1	1
NA	NA	NA	NA	NA	NA	2	1	0.8	1	0	1	0	3	1	3	1	0
NA	NA	NA	NA	NA	NA	1	1	1.7	1	2	1	1	1	1	1	1	1
NA	NA	NA	NA	NA	NA	1	2	6.1	1	2	12	4	2	1	1	19	0
NA	NA	NA	NA	NA	NA	1	1	0.8	3	1	1	1	3	1	1	1	2
NA	NA	NA	Metabolisı	Biosynthes	map0110(	1	1	0.9	1	1	2	1	1	1	1	1	1
NA	NA	NA	NA	NA	NA	1	1	5.7	1	2	2	2	1	1	1	1	1
NA	NA	NA	NA	NA	NA	1	1	1.9	1	2	1	2	1	1	1	2	1
NA	NA	NA	NA	NA	NA	1	1	0.9	2	0	2	0	2	0	2	2	0
NA	NA	NA	NA	NA	NA	16	1	0.8	2	1	2	2	1	2	1	1	0
NA	NA	NA	NA	NA	NA	1	1	1	1	2	1	1	1	1	1	1	2
NA	Aporphine	Alkaloids a	NA	NA	NA	1	1	3.8	1	1	1	1	1	0	1	5	2
Chalcones	Linear 1,3-	Polyketide	Biosynthes	Flavonoid	NA	1	1	1.4	1	1	1	2	1	37	1	1	11
NA	NA	NA	NA	NA	NA	1	1	1.6	1	2	1	2	1	1	1	1	1
Alpha hyd	Hydroxy a	Organic ac	NA	NA	NA	1	17	1.1	0	1	1	10	1	1	1	1	0
NA	NA	NA	NA	NA	NA	1	1	1.3	1	1	1	1	1	1	1	2	2
NA	NA	NA	NA	NA	NA	1	0	0.9	1	0	1	0	1	3	1	1	0
NA	NA	NA	NA	NA	NA	1	1	1.5	1	1	1	1	1	1	1	2	1
NA	NA	NA	NA	NA	NA	1	9	5.7	1	1	3	1	3	3	1	13	2
Fatty acid	Fatty Acyls	Fatty acyls	NA	NA	NA	1	1	0.9	3	1	0	0	1	1	2	0	0
NA	NA	NA	NA	NA	NA	2	1	1	1	1	1	1	1	1	1	1	1
						0	89	2	2	##	6	51	##	47	##	0	63
NA	NA	NA	NA	NA	NA	1	1	0.9	1	0	1	0	1	1	1	2	0
NA	NA	NA	NA	NA	NA	1	1	1.8	1	2	1	2	1	1	1	1	1
NA	NA	NA	NA	NA	NA	1	1	2	1	2	1	2	1	1	1	1	1
NA	NA	NA	NA	NA	NA	1	0	0.2	2	0	1	0	1	0	1	0	0
NA	NA	NA	NA	NA	NA	1	1	0.7	1	1	6	0	1	2	1	1	2
Methoxyb	Benzene a	Benzene a	NA	NA	NA	3	1	0.6	6	1	0	0	2	2	2	1	1
NA	NA	NA	NA	NA	NA	3	1	0.6	6	1	0	0	2	2	2	1	1
NA	NA	NA	NA	NA	NA	1	1	2.1	1	2	4	3	1	1	1	2	0
NA	NA	NA	NA	NA	NA	0	2	1.1	1	0	0	0	8	0	1	0	0
Amino aci	Carboxylic	Amino aci	Amino aci	Glycine, se	map0026(	1	1	2.3	1	2	1	2	1	1	1	2	1
NA	NA	NA	NA	NA	NA	1	1	0.6	1	0	2	1	3	1	0	1	0
NA	NA	NA	NA	NA	NA	1	1	0.3	1	0	2	0	2	0	0	0	0
NA	NA	NA	NA	NA	NA	9	1	1	1	1	2	1	1	1	1	1	1

						0	0	0	0	0	0	0	0	0	3	0	0
NA	NA	NA	NA	NA	NA	1	1	1.6	1	2	1	2	1	1	1	1	1
Non-meta	Non-meta	Homogen	NA	NA	NA	1	1	1.3	2	1	1	1	1	1	1	1	1
Oximes	Organonit	Organic ni	NA	NA	NA	1	1	1.4	1	1	1	1	1	1	1	2	22
Estrane ste	Steroids ai	Steroids ai	NA	NA	NA	1	1	0.6	4	1	2	1	0	1	1	2	2
NA	NA	NA	NA	NA	NA	1	0	1.1	1	1	0	14	1	1	4	1	1
NA	Cinnamalc	Polyketide	NA	NA	NA	1	1	2.3	1	2	0	3	1	1	2	2	1
NA	NA	NA	NA	NA	NA	2	1	0.1	1	0	1	0	8	0	0	0	2
Benzenedi	Phenols	Phenols ar	NA	NA	NA	1	1	1.3	2	1	1	1	7	1	1	1	1
Glyceroph	Glyceroph	Glyceroph	NA	NA	NA	3	1	1.9	1	2	1	2	1	1	1	1	1
Amino aci	Carboxylic	Amino aci	NA	NA	NA	2	1	0.2	2	1	2	0	3	1	1	1	1
Amino aci	Carboxylic	Amino aci	NA	NA	NA	1	1	1.3	1	1	6	1	1	1	2	1	1
NA	NA	NA	NA	NA	NA	1	1	1.1	1	1	1	1	1	1	1	1	1
NA	NA	NA	NA	NA	NA	1	5	0.4	1	0	7	0	1	0	0	0	35
NA	NA	NA	NA	NA	NA	1	1	0.9	1	1	1	1	1	1	1	1	1
Piperazine	Diazinanes	Organohe	NA	NA	NA	1	1	1.4	1	2	1	1	1	1	1	2	1
NA	Piperidine	Organohe	NA	NA	NA	1	1	1.6	1	2	1	2	1	1	1	2	0
NA	NA	NA	NA	NA	NA	1	1	1.3	1	1	1	2	1	1	1	1	1
						0	0	0	0	0	0	0	12	0	0	0	0
NA	NA	NA	NA	NA	NA	1	2	2.2	2	1	2	2	1	0	1	2	0
NA	NA	NA	NA	NA	NA	1	1	0.9	1	1	1	1	1	1	1	1	1
NA	NA	NA	NA	NA	NA	1	1	1	1	1	1	2	1	1	1	1	2
Apocarote	Carotenoi	Terpenoid	Biosynthes	Biosynthes	NA	1	1	1.1	1	2	2	2	0	1	0	1	2
NA	NA	NA	NA	NA	NA	2	0	0.6	3	1	0	0	1	0	7	3	0
						0	0	0	0	0	0	0	0	0	0	0	0
NA	NA	NA	NA	NA	NA	0	2	0.8	1	11	1	1	2	2	0	1	0
NA	NA	NA	NA	NA	NA	1	5	1.1	1	1	1	1	1	1	1	1	1
NA	NA	NA	NA	NA	NA	5	1	0.5	1	1	0	0	1	1	1	0	1
NA	NA	NA	NA	NA	NA	1	1	1.5	1	2	1	2	1	1	1	1	2
NA	NA	NA	NA	NA	NA	1	1	0.3	2	2	1	0	4	6	4	0	6
Pyridineca	Pyridines a	Pyridine ai	Metabolis	Nicotinate	map00760	0	1	1.8	33	2	0	1	3	0	0	1	0
NA	NA	NA	NA	NA	NA	1	1	1.4	1	1	1	1	1	1	1	1	1
NA	NA	NA	NA	NA	NA	1	1	0.7	1	1	1	1	1	0	1	1	2
NA	NA	NA	NA	NA	NA	0	2	6.1	0	4	14	4	1	1	0	3	1
NA	NA	NA	NA	NA	NA	1	1	1.9	1	2	1	2	1	1	1	2	1
Amino aci	Carboxylic	Amino aci	NA	NA	NA	1	1	1.8	1	2	1	2	1	1	1	1	1
NA	NA	NA	NA	NA	NA	1	1	0.9	1	2	2	1	1	1	1	1	1
NA	NA	NA	NA	NA	NA	38	1	0.9	1	1	14	1	3	1	1	1	1
NA	NA	NA	NA	NA	NA	1	1	0.6	1	##	1	83	5	71	1	1	0
Methoxyp	Phenols	Phenols ar	Amino aci	Tyrosine n	map00350	1	2	1.9	2	1	2	2	2	1	1	3	0
NA	NA	NA	NA	NA	NA	2	1	1.7	1	2	27	2	3	1	1	1	1
NA	NA	NA	NA	NA	NA	1	19	1.3	1	1	1	1	1	1	1	1	1
NA	NA	NA	NA	NA	NA	1	1	2.2	2	2	1	24	1	4	1	1	3
NA	NA	NA	NA	NA	NA	1	1	1.5	1	2	1	2	1	1	1	15	1
NA	NA	NA	NA	NA	NA	5	2	0.4	3	5	0	0	0	1	3	1	0
Ethers	Organoox	Organic o	NA	NA	NA	2	1	0.5	3	2	0	0	1	4	2	0	2
NA	NA	NA	NA	NA	NA	1	1	1.5	1	2	4	2	1	1	1	1	1
NA	NA	NA	NA	NA	NA	1	1	1.7	1	1	1	2	1	1	5	1	0
NA	NA	NA	NA	NA	NA	1	1	1.3	1	2	1	2	5	1	1	1	2
						0	0	0	0	0	0	0	0	0	0	0	0
						0	0	0	0	0	0	0	0	0	0	0	0
						0	0	0	0	0	0	0	0	0	0	0	0
NA	NA	NA	NA	NA	NA	1	1	1.2	1	1	1	18	1	1	1	2	1
NA	NA	NA	NA	NA	NA	2	1	0.9	2	1	4	3	2	1	0	2	2
Fatty alcoh	Fatty Acyls	Fatty acyls	NA	NA	NA	1	3	3.8	0	3	6	4	1	0	0	4	1
NA	NA	NA	NA	NA	NA	1	1	1.5	1	1	3	2	13	1	1	1	1
NA	NA	NA	NA	NA	NA	1	1	0.8	1	1	1	0	1	1	1	1	1
Coumarin	Coumarins	Coumarins	NA	NA	NA	1	1	0.9	1	1	1	1	1	1	1	1	3

NA	NA	NA	NA	NA	NA	1	1	1.8	1	2	1	2	1	1	1	1	1
NA	NA	NA	NA	NA	NA	1	1	1.5	1	1	2	2	1	1	1	1	1
Benzoic ac	Benzene a	Benzene a	Xenobiotic	Xylene deg	map0110(	1	1	1.2	1	2	1	2	1	2	1	1	1
						0	0	0	0	6	0	0	0	0	0	0	0
NA	NA	NA	NA	NA	NA	1	0	0.9	1	1	1	1	1	11	1	1	1
NA	NA	NA	NA	NA	NA	1	1	0.7	1	1	1	1	2	1	1	1	1
NA	NA	NA	NA	NA	NA	7	1	3.3	1	1	##	1	1	1	1	1	1
NA	NA	NA	NA	NA	NA	3	0	0.2	3	1	0	0	1	1	3	0	1
NA	NA	NA	NA	NA	NA	3	2	1.1	1	1	1	1	1	1	1	1	0
Indoles	Indoles an	Indoles	NA	NA	NA	2	1	0.8	3	1	4	3	2	1	0	2	2
NA	NA	NA	NA	NA	NA	1	1	0.9	1	1	3	1	7	1	1	1	1
NA	NA	NA	NA	NA	NA	1	1	0.1	1	0	1	0	2	2	3	0	1
Hydroxyst	Steroids ar	Steroids ar	Lipid meta	Steroid ho	map0014C	1	1	1.5	1	3	1	8	1	1	0	1	2
NA	NA	NA	NA	NA	NA	2	1	0.5	3	0	0	0	1	1	2	0	0
NA	NA	NA	NA	NA	NA	1	2	0.1	0	0	0	0	1	1	0	0	4
NA	NA	NA	NA	NA	NA	1	1	0.8	1	0	2	0	0	6	1	1	1
						0	0	0	11	##	0	0	0	7	21	0	0
Glyceroph	Glyceroph	Glyceroph	NA	NA	NA	1	3	0.7	2	13	0	11	1	0	1	1	5
NA	NA	NA	NA	NA	NA	1	2	2.8	1	1	1	2	1	1	1	0	0
NA	NA	NA	NA	NA	NA	2	1	1.3	4	0	1	0	1	0	4	1	0
NA	NA	NA	NA	NA	NA	1	1	0.8	2	1	1	0	1	3	1	1	1
NA	NA	NA	NA	NA	NA	1	1	1.6	2	3	1	1	1	1	2	2	2
NA	NA	NA	NA	NA	NA	1	1	1.4	1	1	1	2	1	1	1	1	1
NA	NA	NA	NA	NA	NA	1	1	4.6	0	2	1	1	1	3	7	5	1
NA	NA	NA	NA	NA	NA	1	1	1.8	1	2	1	2	1	1	2	2	1
NA	NA	NA	NA	NA	NA	1	1	0.9	1	1	3	1	1	1	1	1	1
NA	NA	NA	NA	NA	NA	1	1	1.8	1	2	1	2	1	1	1	2	1
NA	NA	NA	NA	NA	NA	1	1	1.1	0	3	15	7	2	3	1	3	0
Aniline an	Benzene a	Benzene a	NA	NA	NA	1	1	1.3	1	1	1	2	1	1	1	1	8
NA	NA	NA	NA	NA	NA	1	1	1.3	2	0	1	0	1	0	2	1	0
Quinone a	Prenol lipi	Quinone	NA	NA	NA	1	1	1.9	1	2	1	2	1	1	1	2	1
NA	NA	NA	NA	NA	NA	3	1	5.2	3	1	0	4	1	0	3	3	0
NA	NA	NA	NA	NA	NA	1	1	0.7	1	0	1	0	1	6	1	1	1
NA	NA	NA	NA	NA	NA	1	1	2	1	2	2	3	1	2	1	1	0
NA	NA	NA	NA	NA	NA	2	1	2.8	1	0	0	0	1	1	0	0	1
						0	0	0	0	0	0	0	0	0	0	0	0
						0	0	6	0	0	0	0	0	0	0	0	0
NA	Flavin nucl	Nucleic ac	Energy me	Oxidative	map0019C	1	1	1.1	1	1	87	1	1	1	1	1	3
NA	NA	NA	NA	NA	NA	1	1	1.3	1	2	1	2	1	1	1	2	1
Benzenesu	Benzene a	Benzene a	Xenobiotic	Toluene d	map0110(	1	1	2	1	1	1	1	2	1	1	1	0
NA	NA	NA	NA	NA	NA	1	1	2.1	1	2	1	2	1	1	1	1	1
						0	0	0	0	0	0	0	0	0	0	2	18
Carbohydr	Organoox	Carbohydr	NA	NA	NA	2	1	1.3	1	2	1	2	5	1	1	1	1
Sulfated st	Steroids ar	Steroids ar	NA	NA	NA	1	0	0.8	1	0	1	1	7	1	0	1	2
NA	NA	NA	NA	NA	NA	1	1	1.3	1	2	1	1	1	1	1	2	1
NA	NA	NA	NA	NA	NA	1	1	1.1	1	1	1	1	15	5	3	1	28
NA	Benzene a	Benzene a	NA	NA	NA	1	1	2.3	1	2	1	2	1	1	1	2	0
NA	NA	NA	NA	NA	NA	1	1	0.9	1	1	1	1	1	4	1	1	1
NA	NA	NA	NA	NA	NA	1	1	0.8	1	0	1	1	1	7	1	1	1
						0	0	0	3	86	0	0	29	0	0	0	0
NA	NA	NA	NA	NA	NA	1	0	2.5	1	1	8	1	1	0	5	1	0
Carbohydr	Organoox	Carbohydr	NA	NA	NA	1	1	1.9	1	2	1	2	1	1	1	2	1
NA	NA	NA	NA	NA	NA	1	1	1.9	1	2	1	2	1	1	1	2	1
Phenylmet	Benzene a	Amines an	NA	NA	NA	0	0	2.1	1	1	0	2	0	5	1	2	0
NA	NA	NA	NA	NA	NA	1	1	0.9	1	2	1	2	3	1	1	1	13
NA	NA	NA	NA	NA	NA	1	1	1.4	1	2	1	2	1	1	0	1	3
NA	NA	NA	NA	NA	NA	1	1	1.5	1	1	1	2	1	1	1	1	1
NA	NA	NA	NA	NA	NA	1	4	0.3	0	1	0	0	1	1	0	0	4

NA	NA	NA	NA	NA	NA	1	1	1.8	1	2	2	2	1	1	1	1	1
NA	NA	NA	NA	NA	NA	1	1	2	1	3	1	0	1	1	2	1	1
NA	NA	NA	NA	NA	NA	1	1	2.7	1	2	1	2	1	1	1	2	0
NA	NA	NA	NA	NA	NA	17	1	1.4	1	5	1	11	0	1	1	7	3
Carbohydr	Organoox	Carbohydr	Carbohydr	Pentose ar	map0040C	1	1	1.1	67	45	0	4	1	1	11	1	2
Carbohydr	Organoox	Carbohydr	Carbohydr	Pentose ar	map0040C	1	1	1.1	67	45	0	4	1	1	11	1	2
NA	NA	NA	NA	NA	NA	0	3	0.5	0	1	0	9	2	1	2	0	1
NA	NA	NA	NA	NA	NA	1	1	1.2	1	1	1	2	1	1	1	1	1
NA	NA	NA	NA	NA	NA	1	1	1.9	1	2	1	2	1	1	1	2	1
NA	NA	NA	NA	NA	NA	1	1	1.1	1	1	7	1	1	1	1	1	1
NA	NA	NA	NA	NA	NA	1	1	0.9	1	2	1	1	0	1	1	1	1
NA	NA	NA	NA	NA	NA	2	1	1.2	3	1	0	0	1	0	2	0	0
NA	NA	NA	NA	NA	NA	0	0	1.1	1	1	1	1	1	0	0	2	1
						0	0	0	0	11	0	0	0	0	9	0	0
NA	NA	NA	NA	NA	NA	1	1	2	7	5	1	3	1	2	1	1	0
NA	NA	NA	NA	NA	NA	1	1	0.9	3	2	0	0	0	0	1	0	0
NA	NA	NA	NA	NA	NA	0	1	1.7	1	0	1	0	1	1	3	7	0
Methoxyp	Phenols	Phenols ar	NA	NA	NA	1	3	2.3	1	2	1	1	5	2	1	0	0
NA	NA	NA	NA	NA	NA	0	0	0.5	0	18	1	15	13	22	1	0	1
FA0301 Pr	FA03 Eicos	Fatty acyls	Lipid meta	Arachidon	map0059C	8	0	0.6	2	12	51	17	1	0	1	39	2
NA	NA	NA	NA	NA	NA	1	1	1.2	1	1	2	2	1	1	1	1	2
Stilbene gl	Stilbenes	Polyketide	NA	NA	NA	1	1	0.9	1	1	1	1	1	1	1	1	1
NA	NA	NA	NA	NA	NA	1	1	1.5	1	1	1	1	1	1	1	1	1
NA	NA	NA	NA	NA	NA	1	1	1.4	1	2	1	2	1	1	1	1	1
Fatty acids	Fatty Acyls	Fatty acyls	Lipid meta	Arachidon	map0059C	1	1	0.4	0	3	15	1	1	6	0	1	4
NA	NA	NA	NA	NA	NA	0	7	0.5	1	1	1	1	1	3	1	8	9
NA	NA	NA	NA	NA	NA	2	0	0.5	4	1	1	1	2	1	2	13	0
NA	NA	NA	NA	NA	NA	1	1	2.4	1	2	1	2	1	1	1	2	1
						0	0	0	0	0	0	0	0	0	0	0	6
Amino aci	Carboxylic	Amino aci	NA	NA	NA	3	1	1.3	1	1	1	1	2	1	1	2	1
NA	NA	NA	NA	NA	NA	1	1	0.8	1	18	2	5	1	18	1	1	90
NA	NA	NA	NA	NA	NA	1	1	0.9	1	1	1	1	1	1	1	1	1
Amino aci	Carboxylic	Amino aci	Nucleotide	Pyrimidine	map0024C	1	1	0.7	1	1	2	1	1	1	1	1	1
Betaxanthi	Betalains	Amino aci	NA	NA	NA	1	1	1.8	1	2	1	2	1	1	1	2	1
NA	NA	NA	NA	NA	NA	1	1	1.9	1	2	1	2	1	1	1	2	1
NA	NA	NA	NA	NA	NA	1	1	1	1	2	1	1	4	2	1	1	1
NA	NA	NA	NA	NA	NA	1	1	1.8	1	1	1	2	1	1	1	0	1
NA	NA	NA	NA	NA	NA	1	1	1.6	4	1	1	1	1	1	1	1	5
Flavones	Flavonoids	Flavonoids	NA	NA	NA	2	1	2.2	3	0	6	1	3	14	2	3	1
NA	NA	NA	NA	NA	NA	##	1	1.4	1	1	1	2	1	1	1	1	1
NA	NA	NA	NA	NA	NA	1	1	1	1	2	1	14	4	7	1	1	1
NA	NA	NA	NA	NA	NA	1	1	1.8	1	2	1	2	1	1	1	1	1
Purines an	Imidazopy	Purines an	NA	NA	NA	1	1	1.1	1	1	1	1	1	1	1	1	4
1,4-benzo	Benzodiaz	Organohe	NA	NA	NA	1	1	1.9	1	2	1	2	1	1	1	2	1
NA	NA	NA	NA	NA	NA	2	1	0.9	0	0	3	0	13	0	3	6	1
NA	NA	NA	NA	NA	NA	3	1	0.5	3	0	1	1	5	1	2	4	1
						0	0	0	0	0	0	5	0	0	16	0	0
Indoles	Indoles an	Indoles	Amino aci	Tryptopha	map0038C	1	1	0.8	3	1	0	0	1	1	2	0	0
NA	NA	NA	NA	NA	NA	2	1	0.8	1	1	1	0	1	1	1	1	1
Others	Sesquiterp	Terpenoid	NA	NA	NA	1	1	1.5	1	2	1	1	1	1	1	2	1
Amino aci	Carboxylic	Amino aci	NA	NA	NA	27	1	1.1	2	1	3	1	0	1	0	0	7
NA	NA	NA	NA	NA	NA	1	1	1.4	1	2	1	1	1	1	1	1	1
NA	NA	NA	NA	NA	NA	1	2	4.1	1	4	1	2	1	2	1	3	1
NA	NA	NA	NA	NA	NA	1	1	1.4	1	2	1	1	1	1	1	2	1
Amino aci	Carboxylic	Amino aci	NA	NA	NA	1	1	1.7	1	2	1	2	10	1	1	1	1
						0	0	0	0	0	0	0	7	0	0	0	0
NA	NA	NA	NA	NA	NA	1	2	2.1	1	2	1	2	1	1	2	1	1
NA	NA	NA	NA	NA	NA	1	1	1.5	1	2	2	2	1	1	1	1	1

NA	NA	NA	NA	NA	NA	0	0	22	0	4	5	2	1	1	1	0	2
Indolyl car	Indoles an	Indole anc	Amino aci	Tryptopha	map00380	3	0	0.7	3	1	7	2	2	1	0	3	4
NA	NA	NA	NA	NA	NA	1	1	0.8	2	1	1	1	1	3	2	2	1
NA	Benzene a	Benzene a	NA	NA	NA	1	3	1.3	1	1	4	2	1	1	4	3	1
						0	0	0	0	0	0	0	0	0	0	0	0
NA	NA	NA	NA	NA	NA	1	1	1.9	3	0	3	0	2	0	2	2	0
NA	NA	NA	NA	NA	NA	1	1	1.5	1	1	1	2	3	1	1	1	1
NA	NA	NA	NA	NA	NA	5	1	2.6	1	1	1	2	1	1	1	1	0
NA	Phenylpro	Phenylpro	Biosynthes	Tropane, p	NA	5	1	2.6	1	1	1	2	1	1	1	1	0
NA	NA	NA	NA	NA	NA	1	1	1.8	1	2	1	2	1	1	1	1	1
NA	NA	NA	NA	NA	NA	1	1	0.8	3	1	3	0	1	0	1	1	1
NA	NA	NA	NA	NA	NA	1	1	1.2	1	7	1	1	2	2	1	1	2
NA	Naphthale	Benzene a	NA	NA	NA	2	1	1.2	1	2	1	2	1	1	1	1	1
Glyceroph	Glyceroph	Glyceroph	NA	NA	NA	1	1	0.1	0	4	1	1	1	25	1	0	3
NA	NA	NA	NA	NA	NA	0	0	1.6	2	1	1	1	3	0	1	5	70
NA	NA	NA	NA	NA	NA	2	1	0.5	5	1	0	0	1	0	2	0	1
NA	NA	NA	NA	NA	NA	3	1	0.5	1	0	1	0	2	0	2	0	4
NA	NA	NA	NA	NA	NA	2	1	1.9	3	1	0	0	1	0	2	0	0
NA	NA	NA	NA	NA	NA	1	1	1.6	1	2	53	2	1	1	1	1	1
Diphenylm	Benzene a	Benzene a	NA	NA	NA	1	1	0.9	2	1	1	0	1	1	1	1	1
						0	0	0	0	0	0	0	0	0	0	0	0
						0	0	0	0	0	0	0	0	0	0	0	0
						0	0	0	0	0	0	0	0	0	0	0	0
						0	0	0	0	0	0	0	0	0	0	0	0
Amino aci	Carboxylic	Amino aci	NA	NA	NA	1	1	1.9	1	2	1	2	1	1	1	1	1
NA	NA	NA	NA	NA	NA	1	1	29	1	1	##	2	1	1	1	1	7
NA	NA	NA	NA	NA	NA	2	1	0.8	3	1	1	1	0	26	0	1	1
NA	NA	NA	NA	NA	NA	1	1	1	1	2	1	2	4	2	1	1	1
NA	NA	NA	NA	NA	NA	0	0	0.2	1	6	0	11	15	9	2	0	1
NA	NA	NA	NA	NA	NA	1	2	0.2	1	0	0	0	3	0	2	2	3
NA	NA	NA	NA	NA	NA	1	1	0.7	1	1	1	0	1	1	1	1	1
NA	NA	NA	NA	NA	NA	1	1	1.1	1	9	1	1	2	2	1	1	2
Terpene la	Prenol lipi	Terpenoid	NA	NA	NA	1	1	0.7	1	1	1	3	1	2	1	2	1
NA	NA	NA	NA	NA	NA	3	1	0.5	2	0	1	0	1	0	1	1	2
NA	NA	NA	NA	NA	NA	2	1	0.8	1	1	1	1	1	1	1	1	1
NA	NA	NA	NA	NA	NA	1	1	1.3	1	1	31	1	1	1	1	2	0
						0	0	0	0	2	0	0	0	13	8	0	30
NA	NA	NA	Global anc	Biosynthes	NA	1	1	0.9	1	1	2	1	1	1	1	1	1
NA	NA	NA	NA	NA	NA	0	1	2.1	1	0	1	2	1	1	1	1	0
NA	NA	NA	NA	NA	NA	1	1	2.7	1	1	1	2	1	1	1	2	0
NA	NA	NA	NA	NA	NA	1	1	0.8	1	1	1	2	1	1	0	1	8
NA	NA	NA	NA	NA	NA	8	1	0.7	1	1	19	1	1	0	1	2	3
NA	NA	NA	NA	NA	NA	1	1	1.4	2	0	1	0	1	1	1	1	1
NA	NA	NA	NA	NA	NA	1	1	2.2	1	2	1	2	0	1	1	2	0
						0	0	0	0	0	0	0	0	0	18	0	0
Benzodiaz	Diazanaph	Organohe	NA	NA	NA	1	1	0.5	1	0	1	1	2	1	1	2	1
NA	NA	NA	NA	NA	NA	1	1	1.2	3	1	1	1	1	10	2	1	5
NA	NA	NA	NA	NA	NA	2	1	0.7	1	1	##	0	10	3	1	5	1
NA	NA	NA	NA	NA	NA	1	1	0.8	1	1	1	2	2	1	1	1	1
						0	0	0	0	0	6	55	##	43	31	0	11
						0	0	0	9	0	0	0	34	0	66	0	0
NA	NA	NA	NA	NA	NA	0	1	1.4	2	15	0	2	1	7	2	1	0
Pregnane	Steroids a	Steroids a	NA	NA	NA	0	3	0.9	2	1	15	0	3	0	1	1	0
NA	NA	NA	NA	NA	NA	1	2	2.5	2	0	1	0	2	0	1	1	0
NA	NA	NA	NA	NA	NA	1	2	0.5	##	7	4	1	1	6	31	1	1
NA	NA	NA	NA	NA	NA	1	1	94	1	1	##	2	1	1	1	1	1
NA	NA	NA	NA	NA	NA	0	1	0.8	1	2	1	0	1	42	10	1	1
NA	NA	NA	NA	NA	NA	1	3	1.2	4	1	1	1	5	1	1	1	2

Carbohydr	Organoox	Carbohydr	NA	NA	NA	1	2	0.4	1	6	0	1	3	1	2	2	1
NA	NA	NA	NA	NA	NA	1	1	1	1	1	1	2	2	1	1	1	2
NA	NA	NA	NA	NA	NA	1	1	1.6	1	1	1	2	1	1	1	1	1
NA	NA	NA	NA	NA	NA	1	1	1.9	1	2	1	2	1	1	1	2	3
NA	NA	NA	NA	NA	NA	1	1	2	1	2	1	2	1	1	1	2	1
NA	NA	NA	NA	NA	NA	1	1	0.9	1	3	0	2	2	1	2	1	0
Ceramides	Sphingolip	Sphingolip	NA	NA	NA	1	1	3.1	1	2	1	2	2	1	1	2	1
NA	NA	NA	NA	NA	NA	1	1	1.4	1	1	1	2	5	2	1	1	0
Fatty acids	Fatty Acyls	Fatty acyls	NA	NA	NA	1	2	1.5	1	3	1	4	2	0	1	2	2
NA	NA	NA	NA	NA	NA	2	1	0.7	1	1	1	0	1	2	1	1	1
NA	NA	NA	NA	NA	NA	2	1	1.2	1	1	1	1	1	1	1	1	1
NA	NA	NA	NA	NA	NA	1	1	2.8	1	2	1	1	1	1	1	1	0
						0	0	0	13	29	0	27	71	33	34	0	0
						0	0	0	0	0	0	0	48	0	0	0	0
Benzoxazi	Benzoxazi	Organohe	NA	NA	NA	20	0	0.9	10	8	22	0	0	3	33	1	25
						0	0	0	42	78	0	99	##	0	25	0	0
NA	NA	NA	NA	NA	NA	1	1	1.2	1	2	1	1	1	1	1	2	1
						0	0	0	0	0	0	0	0	0	0	0	0
						0	0	0	0	0	0	0	0	0	5	0	0
NA	NA	NA	NA	NA	NA	1	1	1.6	1	48	2	1	1	1	1	1	2
Bile acids,	Steroids ar	Bile acids,	NA	NA	NA	1	1	0.8	1	1	1	1	1	4	1	1	1
NA	NA	NA	NA	NA	NA	1	1	0.9	1	1	1	1	1	3	1	1	1
Benzenedi	Phenols	Phenols ar	Amino aci	Tyrosine m	map00350	1	1	2.2	2	2	0	2	1	1	1	2	1
NA	NA	NA	NA	NA	NA	1	1	1.3	1	1	1	1	1	1	1	1	1
NA	NA	NA	NA	NA	NA	1	1	1.3	1	1	1	1	1	1	1	1	1
Bile acids,	Steroids ar	Bile acids,	Lipid meta	Primary bi	map00120	2	1	0.8	1	2	1	0	1	1	1	0	2
Cyclohexy	Benzene a	Phenols ar	NA	NA	NA	1	1	0.6	1	1	1	1	1	1	1	1	1
NA	NA	NA	NA	NA	NA	1	1	1.7	1	2	1	2	1	1	4	2	0
NA	NA	NA	NA	NA	NA	1	1	1	1	1	1	1	4	2	1	1	1
NA	NA	NA	NA	NA	NA	0	5	0.1	0	0	4	1	4	2	0	0	##
NA	NA	NA	NA	NA	NA	1	2	0.8	2	2	1	1	1	1	1	2	1
NA	NA	NA	NA	NA	NA	1	1	1.9	1	2	6	2	1	2	2	1	1
NA	NA	NA	NA	NA	NA	1	1	1.7	1	1	2	0	1	1	1	1	64
NA	NA	NA	NA	NA	NA	1	0	1.6	1	3	1	0	0	1	2	2	0
NA	NA	NA	NA	NA	NA	0	0	52	0	9	21	3	2	0	1	0	3
NA	NA	NA	NA	NA	NA	0	1	1.2	0	1	2	0	1	1	0	2	0
NA	NA	NA	NA	NA	NA	1	3	0.6	5	0	0	0	1	0	3	1	0
NA	NA	NA	NA	NA	NA	1	0	3.3	0	4	2	1	0	1	2	3	0
NA	NA	NA	NA	NA	NA	1	1	0.8	1	2	1	2	1	1	0	1	2
NA	NA	NA	NA	NA	NA	1	1	1.3	1	2	1	2	1	3	1	1	0
Pyrrolizidi	Alkaloids (	Alkaloids	Biosynthes	Tropane, p	NA	1	1	5	1	1	9	1	2	0	1	0	1
NA	NA	NA	NA	NA	NA	1	0	0.5	1	1	1	0	1	1	2	1	0
NA	NA	NA	NA	NA	NA	1	1	1.1	1	1	1	1	1	1	1	1	1
Arylsulfate	Organic su	Organic ac	NA	NA	NA	1	1	2.2	1	2	1	2	1	1	1	2	1
NA	NA	NA	NA	NA	NA	1	3	1.8	1	1	7	1	0	1	0	4	1
NA	NA	NA	NA	NA	NA	1	2	0.6	2	1	1	1	1	1	0	3	1
NA	NA	NA	NA	NA	NA	1	1	1	4	1	4	1	5	9	2	1	3
NA	NA	NA	NA	NA	NA	1	1	1.4	1	1	1	2	1	1	1	1	1
NA	NA	NA	NA	NA	NA	2	31	0.6	2	1	1	1	4	0	1	0	1
NA	NA	NA	NA	NA	NA	1	1	0.9	3	0	1	0	1	0	3	3	0
NA	NA	NA	NA	NA	NA	6	1	1.6	1	1	1	2	1	1	1	1	1
NA	NA	NA	NA	NA	NA	1	1	1.8	1	2	1	2	1	1	1	1	0
NA	NA	NA	NA	NA	NA	1	2	1.7	1	1	1	4	2	1	3	2	1
Quinoline	Alkaloids (	Alkaloids	NA	NA	NA	2	0	0.1	4	1	0	0	0	1	2	0	1
NA	NA	NA	NA	NA	NA	21	0	0.9	1	0	1	0	0	0	1	1	1
Eicosanoic	Fatty Acyls	Fatty acyls	Lipid meta	Arachidon	map00590	1	1	1	1	1	1	1	2	1	1	1	2
NA	NA	NA	NA	NA	NA	0	1	1.2	0	3	14	2	1	1	1	4	1
Pregnane	Steroids ar	Steroids ar	NA	NA	NA	1	1	1.8	1	2	1	1	1	1	1	2	1

NA	NA	NA	NA	NA	NA	1	1	2.3	1	2	1	2	1	1	1	2	1
NA	NA	NA	NA	NA	NA	1	5	1.1	1	1	1	1	2	1	1	1	0
Estrane ste	Steroids ar	Steroids ar	NA	NA	NA	7	1	2	1	2	1	2	1	1	1	2	1
NA	NA	NA	NA	NA	NA	1	1	1.9	1	2	1	2	1	1	2	2	1
NA	NA	NA	NA	NA	NA	4	1	0.7	1	1	1	1	1	2	2	1	0
NA	NA	NA	NA	NA	NA	1	1	1.6	1	1	1	2	1	1	1	1	1
NA	NA	NA	NA	NA	NA	1	1	0.8	1	2	1	14	1	1	1	1	1
						0	0	0	8	##	0	28	53	31	27	0	0
NA	NA	NA	NA	NA	NA	1	1	1	2	1	1	0	1	1	1	1	1
						0	0	0	0	0	0	0	0	0	5	0	0
NA	NA	NA	NA	NA	NA	1	1	1	1	2	1	1	1	15	1	2	2
NA	NA	NA	NA	NA	NA	1	2	0.7	5	3	0	0	0	0	4	0	0
NA	NA	NA	NA	NA	NA	2	2	1.9	1	9	1	3	1	2	1	2	0
NA	NA	NA	NA	NA	NA	1	1	0.9	3	1	5	3	2	1	0	2	2
Indole dite	Mycotoxin	Fungal tox	NA	NA	NA	0	0	0.1	1	0	2	0	1	48	2	0	5
NA	NA	NA	Metabolis	Brassinost	NA	2	1	1.8	1	2	1	2	1	1	1	2	1
Amino aci	Carboxylic	Amino aci	NA	NA	NA	1	1	1.1	1	1	1	1	1	1	1	1	1
NA	NA	NA	NA	NA	NA	1	2	0.8	2	1	1	1	1	1	1	2	1
NA	NA	NA	NA	NA	NA	1	1	1.9	1	2	1	2	1	3	1	2	2
NA	NA	NA	NA	NA	NA	1	3	0.2	1	0	0	0	2	0	3	2	5
NA	NA	NA	NA	NA	NA	4	0	0.7	15	4	3	0	0	40	0	1	2
NA	NA	NA	NA	NA	NA	13	1	0.3	1	0	1	0	10	1	7	0	1
NA	NA	NA	NA	NA	NA	2	1	8.2	1	1	1	1	1	1	1	1	1
						0	0	0	4	0	0	0	0	0	0	0	0
NA	NA	NA	NA	NA	NA	1	1	0.3	0	6	2	12	1	1	6	1	0
NA	NA	NA	NA	NA	NA	1	2	1.6	1	1	1	2	0	1	1	1	1
Pregnane	Steroids ar	Steroids ar	Lipid meta	Steroid ho	map00140	1	7	0.8	2	1	1	1	2	1	1	1	1
NA	NA	NA	NA	NA	NA	1	1	0.9	1	1	1	1	1	1	1	1	1
NA	NA	NA	NA	NA	NA	1	1	1.7	1	2	1	2	1	1	1	1	1
						0	0	0	0	14	0	12	54	0	0	0	0
NA	NA	NA	NA	NA	NA	1	1	0.5	4	1	1	0	3	0	2	2	6
NA	NA	NA	NA	NA	NA	2	3	0.4	48	1	1	0	2	1	0	6	2
NA	NA	NA	NA	NA	NA	1	1	1.8	1	2	1	2	1	1	1	1	1
NA	NA	NA	NA	NA	NA	1	1	1.1	1	1	1	1	1	1	1	1	1
Short-chai	Keto acids	Organic ac	Lipid meta	Synthesis a	map00072	1	1	1.9	1	1	1	3	1	1	1	1	1
						7	0	0	52	##	14	##	##	##	##	0	50
NA	NA	NA	NA	NA	NA	11	5	2.5	0	1	1	0	1	1	4	1	4
NA	NA	NA	NA	NA	NA	1	3	0.4	1	1	1	1	1	1	1	1	2
Amino aci	Carboxylic	Amino aci	Amino aci	Lysine deg	map00310	4	1	0.7	1	1	1	1	1	3	2	1	1
NA	NA	NA	NA	NA	NA	2	1	1.1	0	2	1	4	0	1	1	0	0
NA	NA	NA	NA	NA	NA	1	1	0.5	1	1	1	1	2	1	1	0	3
Carbohyd	Organoox	Carbohyd	Carbohyd	Galactose	map00052	2	3	0.8	1	6	1	10	1	1	3	4	1
NA	NA	NA	NA	NA	NA	1	1	0.8	1	0	1	0	2	10	1	2	1
NA	NA	NA	NA	NA	NA	2	0	2.7	2	1	0	0	0	0	5	0	0
Fatty acids	Fatty Acyls	Fatty acyls	NA	NA	NA	0	6	0.2	0	0	9	0	1	4	1	2	2
NA	NA	NA	NA	NA	NA	1	1	1.7	1	2	1	2	1	48	1	2	1
						0	0	0	0	0	0	0	0	0	0	0	0
Amino aci	Carboxylic	Amino aci	NA	NA	NA	1	1	1.4	1	1	4	1	1	1	1	4	1
NA	NA	NA	NA	NA	NA	1	1	1.7	1	2	1	2	1	1	1	1	1
NA	NA	NA	NA	NA	NA	1	2	1.8	1	1	2	1	1	2	1	1	1
NA	Benzimida	Imidazole	NA	NA	NA	1	1	1.5	1	2	2	2	1	1	1	2	1
NA	NA	NA	Biosynthes	Isoquinoli	NA	1	0	0.5	3	1	1	1	1	##	1	1	1
NA	NA	NA	NA	NA	NA	1	1	2.3	1	2	1	2	1	1	1	2	1
NA	Macrolide	Polyketide	NA	NA	NA	1	1	2	1	2	1	2	2	1	1	2	8
Amino aci	Carboxylic	Amino aci	NA	NA	NA	1	1	1.8	1	2	1	2	1	1	1	2	1
Amines	Organonit	Amines	Lipid meta	Sphingolip	map00600	1	2	0.4	33	1	2	3	1	5	0	17	3
						0	0	0	0	22	0	0	20	0	0	0	0
NA	NA	NA	NA	NA	NA	1	1	1.1	1	1	1	1	1	1	1	1	5

NA	NA	NA	NA	NA	NA	2	1	1.2	1	2	1	2	3	1	1	1	1
NA	NA	NA	NA	NA	NA	2	1	0.7	1	1	1	0	1	2	1	1	1
NA	NA	NA	NA	NA	NA	3	2	0.9	2	1	1	1	3	1	1	1	13
NA	Tetracyclir	Polyketide	NA	NA	NA	1	3	1.5	1	1	11	5	1	1	1	2	3
NA	NA	NA	NA	NA	NA	1	##	1.5	1	2	1	2	1	1	1	1	1
NA	NA	NA	NA	NA	NA	1	1	0.8	1	1	1	1	1	1	1	1	2
						0	0	0	0	0	0	0	18	38	0	0	58
NA	NA	NA	NA	NA	NA	1	1	1.5	1	2	1	2	1	1	1	1	1
NA	NA	NA	NA	NA	NA	0	2	1.7	5	1	1	33	3	15	0	1	9
NA	NA	NA	NA	NA	NA	1	1	1.1	2	1	1	1	1	1	2	3	1
NA	NA	NA	NA	NA	NA	2	1	1	1	1	2	1	1	1	1	1	1
NA	NA	NA	NA	NA	NA	1	0	2.2	1	##	1	0	1	1	10	1	1
NA	NA	NA	NA	NA	NA	1	1	1.4	1	1	1	2	1	1	1	1	1
NA	NA	NA	NA	NA	NA	1	1	1.9	1	2	1	2	1	1	1	1	1
NA	NA	NA	NA	NA	NA	1	2	0.2	4	0	1	0	4	1	0	0	0
						0	0	0	0	0	0	96	78	0	30	0	10
NA	NA	NA	NA	NA	NA	1	1	1.4	1	1	1	2	1	7	1	1	##
NA	NA	NA	NA	NA	NA	1	1	2.1	1	2	2	1	1	1	1	2	1
Pyrimidine	Pyrimidine	Nucleic ac	Nucleotide	Pyrimidine	map00240	3	1	1	1	1	1	2	2	1	1	2	1
NA	NA	NA	NA	NA	NA	1	1	0.9	1	1	2	1	1	1	1	1	1
NA	NA	NA	NA	NA	NA	1	1	1.2	1	2	1	2	1	1	1	1	1
NA	NA	NA	NA	NA	NA	1	1	1.9	1	2	1	2	1	1	1	2	1
NA	NA	NA	Metabolisı	Riboflavin	map00740	0	##	0.7	1	1	1	1	18	1	44	1	3
NA	NA	NA	NA	NA	NA	1	13	1.1	1	2	5	1	1	1	1	1	1
NA	NA	NA	NA	NA	NA	1	1	1.6	2	10	1	2	1	2	3	1	1
NA	NA	NA	NA	NA	NA	0	1	0.9	1	3	2	4	2	6	4	1	1
NA	NA	NA	Metabolisı	Insect hor	NA	2	1	0.7	4	1	0	0	1	0	4	0	0
NA	NA	NA	NA	NA	NA	18	3	3.7	1	1	2	3	1	6	1	2	0
						0	38	###	7	0	0	0	0	0	0	0	4
NA	NA	NA	NA	NA	NA	74	1	1.3	1	1	1	1	0	2	1	1	1
NA	NA	NA	NA	NA	NA	1	1	1.1	1	1	1	1	1	1	1	2	1
NA	NA	NA	NA	NA	NA	1	1	2.3	1	2	1	2	1	2	1	2	1
NA	NA	NA	NA	NA	NA	3	1	1	16	1	73	1	1	1	9	1	1
Biflavonoi	Flavonoids	Flavonoids	NA	NA	NA	##	1	2.1	4	1	1	1	0	1	1	1	3
NA	NA	NA	NA	NA	NA	2	1	0.4	1	0	1	0	3	1	1	1	2
NA	NA	NA	NA	NA	NA	1	1	0.9	1	1	3	2	1	1	1	1	1
NA	NA	NA	NA	NA	NA	3	1	0.8	1	0	1	0	1	6	1	1	1
NA	NA	NA	NA	NA	NA	1	1	0.8	3	1	1	3	9	1	1	2	9
NA	NA	NA	NA	NA	NA	1	1	1.4	1	1	1	2	5	1	1	1	1
NA	NA	NA	NA	NA	NA	1	1	1.9	1	2	1	2	1	1	1	2	3
NA	NA	NA	NA	NA	NA	1	1	2.1	1	2	1	2	1	1	1	2	1
						0	0	0	0	59	0	39	50	0	19	0	0
NA	NA	NA	NA	NA	NA	3	1	15	1	1	1	0	1	1	1	1	0
NA	NA	NA	NA	NA	NA	1	2	0.5	2	0	2	0	4	2	1	0	11
NA	NA	NA	NA	NA	NA	1	1	0.9	1	10	2	2	2	1	1	1	1
NA	Morphinar	Alkaloids a	NA	NA	NA	1	1	0.8	1	1	9	0	1	1	1	1	1
NA	NA	NA	NA	NA	NA	1	11	0.9	3	1	4	1	4	2	1	1	2
NA	Stilbenes	Polyketide	NA	NA	NA	2	1	1.1	1	1	1	2	1	1	1	1	3
O-methyla	Flavonoids	Flavonoids	NA	NA	NA	2	1	0.7	1	1	2	1	1	1	2	1	1
NA	NA	NA	NA	NA	NA	1	1	0.6	3	1	0	0	1	1	2	0	1
NA	NA	NA	NA	NA	NA	0	0	2.8	2	1	1	1	1	8	2	0	1
NA	NA	NA	NA	NA	NA	1	1	1.4	1	1	1	1	1	1	1	1	2
						0	0	0	0	##	0	0	##	36	0	0	0
NA	NA	NA	NA	NA	NA	0	2	0.2	0	5	0	0	1	2	1	0	5
NA	NA	NA	NA	NA	NA	1	1	1.9	1	2	1	2	1	1	1	1	1
NA	NA	NA	NA	NA	NA	1	1	1.2	1	1	1	2	2	1	1	1	1
						0	0	0	0	0	0	0	0	0	0	0	0
NA	NA	NA	NA	NA	NA	1	31	1	1	1	1	1	1	1	1	2	1

2-benzimi	Benzimida	Imidazole	NA	NA	NA	1	1	0.8	1	1	1	0	1	1	1	1	1
NA	NA	NA	NA	NA	NA	1	0	0.6	1	0	0	0	4	0	13	1	0
NA	NA	NA	NA	NA	NA	1	0	0.7	1	0	1	0	1	3	1	1	0
NA	NA	NA	NA	NA	NA	1	1	0.7	1	0	1	0	1	15	1	1	59
NA	NA	NA	NA	NA	NA	1	1	0.9	1	1	1	1	1	2	1	1	1
NA	NA	NA	NA	NA	NA	1	3	1	8	0	1	0	0	1	2	0	0
NA	NA	NA	NA	NA	NA	1	1	1.4	1	1	5	2	1	1	1	1	1
Carbohydr	Organoox	Carbohydr	Carbohydr	Pentose ar	map0004(	4	1	1.8	4	0	2	0	2	1	3	1	19
Flavonoid	Flavonoids	Flavonoids	NA	NA	NA	1	1	1.2	0	1	1	2	2	1	1	1	40
NA	NA	NA	NA	NA	NA	0	1	0.3	0	0	1	0	2	1	7	1	5
NA	NA	NA	NA	NA	NA	1	1	2.3	1	2	1	2	1	1	1	2	0
						0	##	0	0	0	0	41	0	0	0	0	0
NA	NA	NA	NA	NA	NA	1	1	0.2	1	0	1	0	1	1	1	1	2
Carbonyl (	Organoox	Organic o:	NA	NA	NA	1	2	0.7	1	1	1	2	2	1	1	1	2
NA	NA	NA	NA	NA	NA	0	1	5	0	2	1	2	2	1	2	1	0
						0	0	0	0	0	0	6	0	65	0	0	0
Androstan	Steroids ar	Steroids ar	Lipid meta	Steroid ho	map0014(	2	1	0.3	5	1	1	5	2	1	1	1	13
NA	NA	NA	NA	NA	NA	1	0	5	4	2	1	1	0	0	1	3	1
NA	NA	NA	NA	NA	NA	1	1	1	1	1	1	2	4	2	1	1	1
NA	NA	NA	NA	NA	NA	59	0	6.2	2	1	3	1	0	1	0	0	1
NA	NA	NA	NA	NA	NA	1	2	0.7	1	1	22	2	2	1	1	1	1
NA	NA	NA	NA	NA	NA	1	1	2.7	1	1	1	1	1	1	16	1	1
NA	Phenol eth	Benzene a	NA	NA	NA	1	1	1.6	1	1	1	1	2	1	1	1	1
NA	NA	NA	NA	NA	NA	1	1	0.8	1	1	1	1	1	2	1	0	1
Fatty acids	Fatty Acyls	Fatty acyls	NA	NA	NA	1	2	0.8	1	1	1	10	5	2	1	1	1
Arylsulfate	Organic su	Organic ac	NA	NA	NA	1	1	1.7	1	2	1	2	1	1	1	1	1
NA	NA	NA	NA	NA	NA	2	1	1.8	1	2	1	2	1	1	1	1	1
Benzoic ac	Benzene a	Benzene a	NA	NA	NA	2	0	0.2	4	0	0	0	2	0	2	0	1
NA	NA	NA	NA	NA	NA	2	1	1.8	1	2	1	2	1	1	1	1	1
NA	NA	NA	NA	NA	NA	4	1	0.4	2	0	0	0	3	1	1	2	2
NA	NA	NA	NA	NA	NA	1	1	1.4	1	1	1	2	1	1	1	1	1
NA	NA	NA	NA	NA	NA	1	1	0.8	1	1	1	1	1	2	1	3	2
Pyrimidine	Pyrimidine	Nucleic ac	NA	NA	NA	1	1	0.3	2	0	0	0	2	2	1	1	1
NA	NA	NA	NA	NA	NA	1	1	0.5	##	1	1	7	0	2	2	1	1
NA	NA	NA	Energy me	Methane r	map0110(	1	1	0.8	1	1	1	0	1	1	1	1	1
Quinolone	Quinolines	Organohe	NA	NA	NA	2	1	0.4	2	0	0	0	1	2	1	##	0
NA	NA	NA	NA	NA	NA	8	1	0.8	1	1	1	1	1	1	1	1	1
NA	Corynanth	Alkaloids a	NA	NA	NA	2	1	0.9	1	1	1	1	1	1	1	1	12
NA	Phenol est	Benzene a	NA	NA	NA	1	3	3.2	3	0	2	0	2	0	1	2	0
NA	NA	NA	Metabolisı	Biosynthes	NA	1	1	1.7	1	2	1	2	1	1	1	1	1
Imidazoles	Azoles	Imidazoles	NA	NA	NA	##	1	296	1	2	1	1	0	1	1	4	1
1,4-benzo	Benzodiaz	Organohe	NA	NA	NA	1	1	1	1	1	2	1	5	1	1	1	1
Pyridineca	Pyridines a	Pyridine ar	Metabolisı	Nicotinate	map0076(	0	1	1	1	2	1	2	1	1	1	1	1
NA	NA	NA	NA	NA	NA	1	8	2.8	1	2	1	17	1	0	1	1	1
NA	NA	NA	NA	NA	NA	1	1	1.4	1	1	1	1	1	1	2	2	1
NA	NA	NA	NA	NA	NA	1	1	1.8	1	2	1	2	1	1	1	1	1
Hydroxycc	Coumarins	Coumarins	Biosynthes	Phenylpro	NA	1	1	1.8	2	0	4	0	3	0	1	3	0
1,3,5-triaz	Triazines	Organohe	Xenobiotic	Atrazine d	map0110(	2	0	0.7	3	1	0	0	1	0	3	0	1
NA	NA	NA	NA	NA	NA	0	0	0.1	0	0	3	0	8	2	0	0	6
NA	NA	NA	NA	NA	NA	1	1	1.4	1	2	1	2	1	1	2	2	1
NA	NA	NA	NA	NA	NA	1	1	1.8	2	2	0	2	2	0	1	2	0
NA	NA	NA	NA	NA	NA	1	1	2.2	2	2	1	2	1	1	1	1	0
NA	NA	NA	NA	NA	NA	1	1	1.3	1	1	7	2	1	1	1	1	1
NA	NA	NA	NA	NA	NA	0	2	0.2	0	0	2	2	1	0	8	1	1
Tetracarbc	Carboxylic	Organic ac	NA	NA	NA	1	2	2.1	1	1	2	1	1	1	1	1	1
NA	NA	NA	NA	NA	NA	1	1	1.6	1	2	1	1	1	1	1	2	1
NA	NA	NA	NA	NA	NA	1	2	2.1	1	1	2	1	1	1	1	1	1
NA	NA	NA	NA	NA	NA	1	1	1.3	1	2	1	1	1	1	1	1	1

NA	NA	NA	NA	NA	NA	2	1	0.8	2	1	1	0	1	1	1	2	1
NA	NA	NA	NA	NA	NA	1	1	1.9	1	2	2	2	2	1	2	2	0
NA	NA	NA	NA	NA	NA	1	1	1.8	1	2	4	2	1	1	1	1	1
NA	NA	NA	NA	NA	NA	1	1	0.9	1	1	1	1	1	2	1	1	1
Carbazole:	Indoles an	Indole anc	NA	NA	NA	4	1	1.2	1	1	1	2	5	1	1	1	1
						0	0	0	29	29	0	70	18	32	33	0	0
Fatty acids	Fatty Acyls	Fatty acyls	NA	NA	NA	1	1	0.2	0	3	3	4	1	1	1	28	3
NA	NA	NA	NA	NA	NA	1	1	1.8	1	##	1	##	1	80	1	1	1
Anilides	Benzene a	Benzene a	NA	NA	NA	1	1	1.9	1	2	1	2	1	1	1	2	13
NA	NA	NA	NA	NA	NA	1	2	0.7	1	8	1	1	1	1	1	1	0
NA	NA	NA	NA	NA	NA	1	1	0.2	1	3	0	1	1	3	2	1	0
NA	NA	NA	NA	NA	NA	1	1	1.2	1	2	1	1	1	2	1	2	1
NA	NA	NA	NA	NA	NA	1	1	0.9	1	1	1	2	1	1	1	1	1
NA	NA	NA	NA	NA	NA	1	1	1.1	1	1	1	1	1	1	1	1	1
NA	NA	NA	NA	NA	NA	1	1	1.2	1	1	1	2	1	1	1	1	1
NA	NA	NA	NA	NA	NA	1	2	2.3	1	2	2	2	1	1	1	2	1
NA	NA	NA	NA	NA	NA	1	1	1.8	1	2	2	2	1	1	1	1	1
NA	NA	NA	NA	NA	NA	1	1	2.2	1	2	2	2	1	1	1	1	1
Imidazolid	Azolidines	Organohe	NA	NA	NA	1	1	1	1	1	1	1	1	1	1	1	1
Steroidal g	Steroids ar	Steroids ar	Lipid meta	Steroid ho	map00140	1	2	0	0	0	19	0	1	2	1	0	3
Glucosinol	Glucosinol	Amino aci	NA	NA	NA	1	1	1.9	1	5	3	2	1	2	1	1	1
NA	NA	NA	NA	NA	NA	1	1	1	1	1	2	2	1	9	1	1	1
NA	NA	NA	NA	NA	NA	1	10	0.6	1	1	10	1	2	1	1	1	2
NA	NA	NA	NA	NA	NA	1	1	0.9	1	1	1	1	1	1	1	1	1
Furanocou	Coumarins	Coumarins	NA	NA	NA	1	1	1.3	1	1	1	1	1	2	1	1	1
NA	NA	NA	NA	NA	NA	1	1	1.2	1	1	1	1	1	1	1	2	1
NA	NA	NA	Biosynthes	Monobact	NA	3	0	1	3	0	0	0	1	0	9	0	0
NA	NA	NA	NA	NA	NA	1	1	2.7	1	1	1	3	1	1	1	2	1
Glyceroph	Glyceroph	Glyceroph	NA	NA	NA	1	1	1.3	1	2	1	2	1	3	1	1	1
						0	0	0	7	12	0	0	0	88	0	0	0
Amino aci	Carboxylic	Amino aci	Amino aci	Cysteine a	map00270	6	1	0.8	2	3	15	5	1	0	0	2	1
NA	NA	NA	NA	NA	NA	1	1	0.9	1	1	1	0	1	3	1	1	1
NA	NA	NA	NA	NA	NA	1	1	2.2	1	2	1	2	1	1	1	2	1
Diels-Alde	Complex f	Flavonoids	NA	NA	NA	2	0	0.3	1	0	0	0	20	0	1	1	0
NA	NA	NA	NA	NA	NA	1	1	1.9	1	2	1	2	1	1	1	2	1
NA	NA	NA	NA	NA	NA	1	1	1.1	1	3	1	1	1	4	1	1	0
NA	NA	NA	NA	NA	NA	1	3	1.2	1	4	1	4	2	1	1	1	1
NA	NA	NA	NA	NA	NA	1	1	1.5	1	1	2	1	1	1	1	1	2
NA	NA	NA	NA	NA	NA	1	1	0.8	1	1	3	1	1	1	1	1	1
NA	NA	NA	NA	NA	NA	1	1	1.6	1	2	1	1	1	1	1	2	1
NA	NA	NA	NA	NA	NA	1	1	1	1	1	1	1	3	1	5	5	9
Carbohydr	Organoox	Carbohydr	NA	NA	NA	1	1	1.3	1	1	1	1	0	2	1	0	1
Indolyl car	Indoles an	Amino aci	Amino aci	Glycine, se	map00260	2	1	0.7	2	1	5	3	2	1	0	2	3
NA	NA	NA	NA	NA	NA	1	1	0.6	1	1	1	2	3	1	1	1	2
NA	NA	NA	NA	NA	NA	1	6	18	1	1	12	1	1	1	1	12	1
NA	NA	NA	NA	NA	NA	1	2	0.3	1	3	0	2	1	3	2	0	1
NA	NA	NA	NA	NA	NA	1	1	0.9	1	1	1	1	1	1	1	1	1
NA	NA	NA	NA	NA	NA	1	1	1	1	2	1	0	2	2	2	2	1
NA	NA	NA	NA	NA	NA	0	0	4.6	0	2	0	3	1	##	3	0	1
Anilides	Benzene a	Benzene a	NA	NA	NA	0	0	3.7	1	0	1	0	1	8	1	1	0
NA	NA	NA	NA	NA	NA	1	1	2	1	2	1	2	1	1	1	2	1
NA	NA	NA	NA	NA	NA	1	1	1.7	1	2	1	2	2	1	1	1	2
NA	NA	NA	NA	NA	NA	1	1	1.4	1	2	1	1	1	1	1	1	1
NA	NA	NA	NA	NA	NA	0	2	8.1	1	0	6	0	0	0	24	7	0
Pyrimidine	Pyrimidine	Nucleic ac	Nucleotide	Pyrimidine	map00240	1	1	2	2	1	2	2	1	1	1	2	1
NA	NA	NA	Metabolisı	Porphyrin	map00860	1	1	1.6	1	2	1	2	1	1	1	1	1
NA	NA	NA	NA	NA	NA	1	1	0.9	1	0	1	1	2	1	1	1	2
NA	NA	NA	NA	NA	NA	1	1	0.8	1	1	1	1	1	1	2	1	1

NA	NA	NA	NA	NA	NA	1	1	1.5	1	2	1	2	2	1	1	1	1
NA	Benzimida	Imidazole	NA	NA	NA	1	1	2	1	2	1	2	1	1	1	2	1
Amino aci	Carboxylic	Amino aci	NA	NA	NA	0	3	0.6	3	1	0	0	1	0	2	1	0
Anilides	Benzene a	Benzene a	NA	NA	NA	1	1	0.8	1	0	0	1	1	3	1	1	2
NA	NA	NA	NA	NA	NA	2	1	0.7	9	0	0	0	2	0	2	1	1
NA	NA	NA	NA	NA	NA	2	1	1.3	1	1	1	2	11	1	1	1	1
Amino aci	Carboxylic	Amino aci	NA	NA	NA	1	1	1.1	1	1	1	1	1	1	1	1	1
NA	NA	NA	NA	NA	NA	1	1	1.3	1	1	1	2	1	1	1	1	1
NA	NA	NA	NA	NA	NA	1	1	0.1	1	3	0	0	1	4	1	0	0
NA	NA	NA	NA	NA	NA	2	1	0.7	1	1	1	0	1	2	1	1	1
						0	0	0	0	0	0	0	0	0	0	0	0
Pregnane	Steroids a	Steroids a	Lipid meta	Steroid ho	map00140	1	1	1.3	0	1	3	0	1	1	1	1	1
						0	0	0	0	0	0	0	0	5	0	0	0
Phosphos	Sphingolip	Sphingolip	NA	NA	NA	1	2	1.2	1	2	1	8	3	1	0	0	0
NA	Dioxolopy	Organohe	NA	NA	NA	1	1	0.7	2	1	1	1	1	1	2	2	1
NA	NA	NA	NA	NA	NA	3	3	0.5	1	2	1	0	3	1	3	0	10
NA	NA	NA	NA	NA	NA	1	1	1.6	1	2	1	2	1	1	1	1	1
Retinoids	Prenol lipi	Prenol lipi	NA	NA	NA	0	1	1.6	2	1	0	1	0	0	1	1	0
Benzenesu	Benzene a	Benzene a	NA	NA	NA	1	1	0.8	1	1	1	0	1	##	1	1	0
NA	NA	NA	NA	NA	NA	2	1	0.5	1	0	0	1	0	8	1	0	14
NA	NA	NA	NA	NA	NA	9	1	0.9	1	1	1	2	1	1	1	1	1
NA	NA	NA	NA	NA	NA	1	1	1.8	1	2	1	2	4	2	1	1	1
NA	NA	NA	NA	NA	NA	1	1	0.3	1	1	0	0	2	2	1	1	6
NA	NA	NA	NA	NA	NA	1	1	0.8	1	1	1	1	3	2	1	1	2
NA	NA	NA	NA	NA	NA	2	1	1.4	1	1	1	1	1	1	1	1	1
NA	NA	NA	NA	NA	NA	1	1	2.4	1	##	2	2	1	1	1	1	1
NA	NA	NA	NA	NA	NA	1	1	1.5	1	1	2	1	1	1	1	2	1
						0	0	0	0	24	0	0	12	0	15	0	0
NA	NA	NA	NA	NA	NA	1	1	1.7	1	2	1	2	1	1	1	1	1
NA	NA	NA	NA	NA	NA	2	1	1.2	1	12	2	1	1	1	3	1	1
NA	NA	NA	NA	NA	NA	1	1	1.9	1	2	1	2	1	1	1	2	1
NA	NA	NA	NA	NA	NA	1	1	0.8	2	1	1	1	1	1	1	2	1
NA	NA	NA	NA	NA	NA	1	1	0.8	1	0	3	0	6	1	1	2	1
NA	NA	NA	NA	NA	NA	1	1	0.8	1	0	1	0	1	4	1	2	0
NA	NA	NA	NA	NA	NA	1	1	0.8	1	1	1	1	1	1	1	1	1
NA	NA	NA	NA	NA	NA	7	1	1	1	1	1	3	1	2	1	1	1
NA	NA	NA	NA	NA	NA	0	2	0.9	0	1	1	2	1	1	1	2	0
NA	NA	NA	NA	NA	NA	2	1	0.7	2	0	0	0	1	0	2	0	2
NA	NA	NA	NA	NA	NA	1	1	1	3	1	0	0	0	0	2	0	0
NA	NA	NA	NA	NA	NA	1	1	1.2	1	2	1	2	1	1	1	1	1
NA	NA	NA	NA	NA	NA	1	1	1.8	1	2	2	2	1	1	1	1	1
Diradylgly	Glycerolipi	Glycerolipi	NA	NA	NA	1	1	2	1	2	1	2	2	1	1	1	1
NA	NA	NA	NA	NA	NA	2	1	1.9	1	1	1	1	0	1	1	1	1
NA	NA	NA	NA	NA	NA	1	1	1	0	1	2	1	1	1	1	1	1
NA	NA	NA	NA	NA	NA	1	5	0.9	1	1	1	1	4	6	1	1	32
NA	NA	NA	NA	NA	NA	1	1	0.9	1	0	1	1	1	3	1	2	0
NA	NA	NA	NA	NA	NA	0	1	0.1	0	2	0	1	2	3	3	0	7
Sesquiterp	Prenol lipi	Terpenoid	NA	NA	NA	2	1	1.9	1	1	1	1	1	1	1	2	1
NA	NA	NA	NA	NA	NA	1	1	1.5	1	2	1	1	1	1	1	2	1
NA	NA	NA	NA	NA	NA	2	1	1.9	2	1	1	1	2	4	1	5	1
NA	NA	NA	NA	NA	NA	2	1	1	1	1	1	1	1	1	1	1	1
Hydropyri	Pyridines a	Pyridine a	NA	NA	NA	1	1	1.1	2	1	2	0	1	0	2	3	0
Sulfated st	Steroids a	Steroids a	NA	NA	NA	1	1	1	2	1	1	1	3	1	1	0	2
Amines	Organonit	Amines	NA	NA	NA	1	1	1	1	3	1	2	0	3	14	1	1
NA	NA	NA	NA	NA	NA	1	1	1.2	1	1	1	1	1	1	1	28	1
Amino aci	Carboxylic	Amino aci	NA	NA	NA	4	1	0.8	2	7	1	31	0	1	0	0	1
						0	0	0	0	0	0	0	0	8	0	0	0
NA	NA	NA	NA	NA	NA	1	1	1.7	1	2	1	2	1	1	1	1	1

NA	NA	NA	NA	NA	NA	1	1	0.8	1	1	1	1	1	1	1	1	1
NA	NA	NA	NA	NA	NA	1	1	0.7	1	0	1	0	1	5	1	1	0
						0	0	0	59	46	4	74	##	##	##	0	56
Glucosinol	Glucosinol	Amino aci	NA	NA	NA	2	0	2.4	1	0	1	0	3	0	6	1	2
NA	NA	NA	NA	NA	NA	1	1	1.4	1	1	1	1	1	1	1	2	1
NA	NA	NA	NA	NA	NA	1	1	0.8	1	2	1	2	1	1	0	1	2
NA	NA	NA	NA	NA	NA	1	1	1.8	1	2	4	2	1	1	1	2	0
NA	NA	NA	NA	NA	NA	9	0	0.4	2	12	47	7	1	0	0	17	2
NA	NA	NA	NA	NA	NA	1	1	1.4	1	2	1	2	2	1	1	1	1
Terpenoid	Alkaloids (	Alkaloids	NA	NA	NA	1	1	1.6	1	2	1	1	1	1	1	2	1
						0	0	0	0	0	0	0	0	0	0	0	0
						0	0	0	0	0	0	0	0	0	0	9	0
						0	0	0	0	0	0	0	0	0	0	0	0
						0	0	0	0	0	0	0	0	0	0	0	0
Flavonoid	Flavonoids	Flavonoids	NA	NA	NA	1	1	2	1	2	0	1	1	1	8	2	1
NA	NA	NA	NA	NA	NA	1	1	0.7	1	1	1	0	1	4	1	2	1
NA	NA	NA	NA	NA	NA	12	1	1.2	1	1	1	2	1	1	1	2	##
NA	NA	NA	NA	NA	NA	0	2	0.3	0	4	0	1	1	4	2	0	1
NA	NA	NA	NA	NA	NA	1	1	1.4	2	1	0	1	1	1	1	4	0
NA	NA	NA	NA	NA	NA	1	1	2.5	1	2	1	2	1	1	1	2	1
NA	NA	NA	NA	NA	NA	1	1	2.2	1	2	1	2	1	1	1	2	1
NA	NA	NA	NA	NA	NA	1	1	1.3	1	1	2	2	5	1	1	1	1
NA	NA	NA	NA	NA	NA	1	68	1.5	1	1	1	##	1	1	1	1	1
						0	0	0	0	40	0	0	0	0	0	0	0
Phenylpro	Benzene a	Amines an	NA	NA	NA	1	1	2.1	1	2	1	2	1	1	1	2	1
Lineolic ac	Fatty Acyls	Fatty acyls	Lipid meta	Linoleic ac	map00591	2	1	0.6	2	1	1	2	1	1	0	1	2
NA	NA	NA	NA	NA	NA	1	4	0.7	2	1	2	1	4	1	1	1	2
NA	NA	NA	NA	NA	NA	0	1	0.4	0	0	3	0	2	2	2	1	3
NA	NA	NA	NA	NA	NA	0	1	0.1	0	3	0	1	1	23	1	0	1
Pyrrolizidi	Alkaloids (	Alkaloids	NA	NA	NA	1	1	0.4	2	1	1	0	0	8	1	2	0
Purines an	Imidazopy	Purines an	NA	NA	NA	1	6	3.1	1	2	0	2	1	0	1	2	1
NA	NA	NA	NA	NA	NA	1	1	0.9	1	1	1	1	4	2	1	1	1
NA	NA	NA	NA	NA	NA	4	1	2.8	1	1	1	1	3	1	1	2	1
NA	NA	NA	NA	NA	NA	1	1	0.9	1	0	1	0	1	4	1	2	0
NA	NA	NA	NA	NA	NA	1	1	0.9	1	0	1	0	1	4	1	2	0
NA	NA	NA	NA	NA	NA	1	0	0.5	1	0	3	0	2	1	0	3	0
Carbonyl (	Organooxy	Organic o	NA	NA	NA	1	1	2.2	1	2	1	2	1	3	2	1	0
NA	NA	NA	NA	NA	NA	1	1	0.8	0	1	1	1	1	9	1	1	2
NA	NA	NA	NA	NA	NA	1	6	7.4	1	4	65	1	1	1	1	4	0
NA	NA	NA	NA	NA	NA	1	1	1.3	1	2	1	1	1	1	1	3	1
NA	NA	NA	NA	NA	NA	1	1	4	2	1	5	2	3	0	1	4	0
NA	NA	NA	NA	NA	NA	1	1	1.3	1	2	1	1	1	1	1	1	1
NA	NA	NA	NA	NA	NA	1	1	1.6	1	1	1	2	0	1	1	1	1
NA	NA	NA	NA	NA	NA	1	1	1.5	1	2	1	2	1	1	1	1	1
Alcohols a	Organooxy	Alcohols	NA	NA	NA	1	1	2.4	1	2	1	2	1	1	1	2	0
NA	NA	NA	NA	NA	NA	1	1	1.3	1	1	1	1	1	13	1	2	13
NA	NA	NA	NA	NA	NA	7	2	3.5	1	1	0	1	1	1	1	1	0
NA	NA	NA	NA	NA	NA	1	1	1.8	1	2	1	2	3	1	1	2	1
NA	NA	NA	NA	NA	NA	1	4	1.7	1	2	6	2	1	1	2	1	1
NA	NA	NA	NA	NA	NA	1	2	0.4	3	0	0	0	1	0	1	1	0
Carbohydr	Organooxy	Carbohydr	Carbohydr	Pentose ar	map0004(	1	0	0.5	79	24	1	2	1	1	10	1	5
						0	0	0	6	##	0	0	28	29	99	0	0
NA	NA	NA	Metabolisr	Phosphon	map0044(	2	1	0.5	1	1	1	0	1	1	2	1	8
NA	NA	NA	NA	NA	NA	5	1	1.6	1	2	1	2	1	1	1	1	1
NA	NA	NA	NA	NA	NA	1	1	0.8	3	0	0	0	1	0	2	0	0
NA	NA	NA	NA	NA	NA	1	1	1.2	1	2	1	2	3	1	5	1	1
O-methyl	Isoflavono	Flavonoids	Biosynthes	Isoflavono	map0110(	1	1	1.9	3	1	1	2	0	1	0	0	2
Amines	Organonit	Amines	NA	NA	NA	2	1	8.9	0	3	0	2	1	1	2	2	1

						0	0	0	0	0	0	0	9	11	0	0	0
NA	NA	NA	NA	NA	NA	0	1	0.1	1	1	0	0	1	3	1	0	0
						0	0	0	24	52	0	##	##	60	##	3	10
Ceramides	Sphingolip	Sphingolip	NA	NA	NA	2	2	0	4	4	2	1	2	4	0	5	0
NA	NA	NA	NA	NA	NA	1	1	1.3	1	2	1	1	1	2	1	2	1
NA	NA	NA	NA	NA	NA	1	1	1.2	1	1	1	1	0	2	1	1	1
NA	NA	NA	NA	NA	NA	1	1	4.2	1	1	1	1	1	1	1	1	1
Medium-c	Hydroxy a	Organic ac	Xenobiotic	Caprolacta	NA	4	1	2.1	1	3	1	3	0	1	0	1	0
Flavones	Flavonoids	Flavonoids	Biosynthes	Flavonoid	NA	1	4	1.2	1	1	7	1	1	1	1	1	75
Flavonoid	Flavonoids	Flavonoids	NA	NA	NA	1	1	0.8	1	1	33	1	0	0	1	1	1
NA	NA	NA	NA	NA	NA	1	1	1	1	1	1	1	4	2	1	1	1
NA	NA	NA	NA	NA	NA	1	1	0.6	0	4	12	1	0	2	1	3	0
NA	NA	NA	NA	NA	NA	0	0	1.3	1	2	1	2	0	0	5	0	1
NA	Stilbenes	Polyketide	NA	NA	NA	1	1	1.7	1	2	1	2	1	1	1	1	1
NA	NA	NA	NA	NA	NA	1	1	1.2	1	2	1	2	2	2	1	1	0
NA	NA	NA	NA	NA	NA	1	0	0.9	1	1	0	1	1	1	1	2	4
NA	NA	NA	NA	NA	NA	1	1	1.9	2	2	1	1	2	1	1	2	1
FA0106 O:	FA01 Fatty	Fatty acyls	Biosynthes	Glucosinol	map0121(	1	1	1.3	1	2	1	1	1	1	1	2	1
Amino aci	Carboxylic	Amino aci	NA	NA	NA	1	1	1.8	1	2	1	2	1	1	1	2	1
Terpenoid	Alkaloids (	Alkaloids	NA	NA	NA	1	1	2	1	2	1	2	1	1	1	1	1
NA	NA	NA	NA	NA	NA	4	1	2.7	1	1	4	1	3	1	8	1	1
5'-deoxy-	5'-deoxyri	Nucleic ac	NA	NA	NA	1	1	0.9	4	1	5	0	3	0	7	2	1
NA	NA	NA	NA	NA	NA	1	1	1.4	1	1	2	1	1	1	4	1	##
NA	NA	NA	NA	NA	NA	0	1	0.3	0	2	24	1	2	8	0	1	7
Fatty amid	Fatty Acyls	Fatty acyls	NA	NA	NA	1	1	1	1	2	1	2	2	2	1	1	0
NA	NA	NA	NA	NA	NA	3	1	1.4	1	1	10	2	1	1	1	1	3
						0	0	0	11	##	0	14	62	8	31	0	0
Others	Diterpenoi	Terpenoid	NA	NA	NA	1	2	3	1	5	1	1	0	3	1	2	2
NA	NA	NA	NA	NA	NA	2	1	1.6	1	1	1	2	1	1	1	1	1
						0	0	0	0	94	0	0	0	0	0	0	0
NA	NA	NA	NA	NA	NA	1	1	1.3	1	6	1	1	1	1	1	2	1
NA	NA	NA	NA	NA	NA	1	1	0.7	2	1	2	1	1	1	1	2	1
NA	NA	NA	NA	NA	NA	1	1	2.2	1	2	1	2	1	1	1	2	1
1,4-benzo	Benzodiaz	Organohe	NA	NA	NA	1	1	2	1	2	1	2	1	1	1	2	1
NA	NA	NA	NA	NA	NA	1	2	0.6	2	1	1	1	2	2	1	1	1
NA	NA	NA	NA	NA	NA	0	2	0.2	1	6	1	4	2	6	2	0	3
Phenothia	Benzothia:	Organohe	NA	NA	NA	2	1	0.4	2	1	11	1	1	0	2	3	1
NA	NA	NA	NA	NA	NA	1	1	2.1	1	2	2	2	1	1	1	2	1
NA	NA	NA	NA	NA	NA	1	2	1	4	1	0	0	0	1	3	0	0
Retinoids	Prenol lipi	Prenol lipi	Metabolis	Retinol me	map0083(	1	1	1.6	1	1	7	1	1	1	1	2	0
NA	NA	NA	NA	NA	NA	34	1	1.7	1	2	2	2	1	1	1	1	2
NA	NA	NA	NA	NA	NA	1	0	0.5	1	2	0	1	1	4	1	0	6
NA	NA	NA	NA	NA	NA	1	2	2.7	1	3	2	3	1	2	1	2	0
NA	NA	NA	NA	NA	NA	0	2	0.5	1	3	1	3	1	10	1	0	1
Fatty acid	Fatty Acyls	Fatty acyls	NA	NA	NA	1	1	1.6	1	2	2	2	1	1	1	1	2
NA	NA	NA	NA	NA	NA	1	1	0.9	1	1	1	1	1	1	1	59	1
Phosphos	Sphingolip	Sphingolip	NA	NA	NA	0	2	0.6	3	13	1	13	0	2	0	1	0
NA	NA	NA	NA	NA	NA	1	1	1.6	1	3	1	2	2	25	1	1	1
NA	NA	NA	NA	NA	NA	2	0	0.2	1	0	1	0	4	0	1	1	1
Isoquinolir	Alkaloids (	Alkaloids	NA	NA	NA	1	1	0.5	1	1	1	1	2	1	1	0	2
Isoflav-2-(	Isoflavono	Flavonoids	Biosynthes	Isoflavono	NA	5	1	1.1	7	1	4	32	1	1	5	1	15
Bile acids,	Steroids a	Bile acids,	NA	NA	NA	0	25	0.2	0	1	0	3	0	26	1	0	26
NA	NA	NA	NA	NA	NA	0	5	1.9	1	2	1	2	1	1	1	1	1
NA	NA	NA	NA	NA	NA	4	1	0.7	0	2	0	3	0	2	2	1	1
NA	NA	NA	NA	NA	NA	1	1	1	1	1	2	2	2	1	1	1	1
NA	Imidazotet	Organohe	NA	NA	NA	1	1	1.3	1	2	2	2	1	1	1	2	1
NA	NA	NA	NA	NA	NA	1	1	0.5	1	0	1	1	1	29	1	2	2
NA	NA	NA	NA	NA	NA	1	1	2.2	1	2	1	2	1	1	1	2	0

NA	NA	NA	NA	NA	NA	1	1	0.9	1	1	1	1	1	1	1	1	1
NA	NA	NA	Energy me	Methane r	map0110(	0	0	0.7	2	1	0	5	1	0	7	0	0
NA	NA	NA	NA	NA	NA	2	1	0.7	2	1	1	0	1	1	1	1	1
NA	NA	NA	NA	NA	NA	1	1	1.1	1	1	3	1	1	1	1	1	1
Amino aci	Carboxylic	Amino aci	Amino aci	Arginine b	map00220	2	2	1.1	0	2	4	5	0	2	1	1	1
Quinolone	Quinolines	Organohe	NA	NA	NA	1	1	2.3	1	2	1	2	1	1	1	2	0
NA	NA	NA	NA	NA	NA	1	1	1.3	2	0	1	1	1	1	1	2	2
NA	NA	NA	NA	NA	NA	1	2	1.9	1	4	2	1	3	3	2	4	1
NA	NA	NA	NA	NA	NA	1	1	1.1	1	1	1	1	1	1	1	1	2
NA	NA	NA	NA	NA	NA	1	1	1.4	1	1	1	1	1	1	1	2	1
NA	NA	NA	NA	NA	NA	2	1	0.6	1	0	1	0	1	1	2	1	1
Pyrimidine	Diazines	Organohe	Nucleotide	Pyrimidine	map0024(	1	0	3.7	0	3	1	4	2	2	2	2	3
NA	NA	NA	NA	NA	NA	1	1	1.4	1	1	1	1	1	1	1	2	1
						0	0	0	0	0	0	0	0	0	0	0	14
						0	0	0	0	0	0	0	0	0	18	0	0
NA	NA	NA	NA	NA	NA	1	1	1.6	1	2	2	2	5	3	1	1	1
NA	NA	NA	NA	NA	NA	1	1	1.7	1	1	9	1	1	1	1	1	1
Amino aci	Carboxylic	Amino aci	NA	NA	NA	3	1	0.7	1	0	1	0	6	5	3	2	1
NA	NA	NA	NA	NA	NA	1	2	0.8	1	1	3	2	1	2	3	2	1
Diradylgly	Glycerolipi	Glycerolipi	NA	NA	NA	1	1	2.1	1	2	1	2	1	1	1	2	1
NA	NA	NA	NA	NA	NA	1	1	1.4	1	1	1	1	0	2	1	1	1
NA	NA	NA	NA	NA	NA	1	1	1	1	1	3	1	1	1	1	1	1
NA	NA	NA	NA	NA	NA	1	2	0.8	0	2	1	4	1	2	1	1	1
Cinnamic a	Cinnamic a	Polyketide	Metabolisr	Ubiquinon	map00130	2	1	1.1	1	1	4	3	1	1	1	1	1
1-benzopy	Benzopyra	Organohe	NA	NA	NA	1	2	1	2	0	1	0	4	0	2	4	1
NA	NA	NA	NA	NA	NA	2	1	0.7	1	1	1	0	1	3	1	1	1
NA	NA	NA	Biosynthes	Monobact	NA	3	1	1.4	1	1	13	2	1	1	1	2	1
NA	NA	NA	NA	NA	NA	1	7	0.6	1	0	3	0	3	1	1	1	5
NA	NA	NA	NA	NA	NA	1	1	1.9	1	2	1	2	1	1	1	1	1
NA	NA	NA	NA	NA	NA	1	1	1	1	1	1	1	1	2	1	2	1
NA	NA	NA	NA	NA	NA	0	2	0.2	3	1	1	1	1	1	2	2	1
Indolyl car	Indoles an	Indole and	NA	NA	NA	2	1	0.6	4	0	1	0	1	0	5	1	0
NA	NA	NA	NA	NA	NA	1	1	0.9	1	1	1	1	5	2	1	1	1
NA	NA	NA	NA	NA	NA	1	1	4.4	3	2	1	1	1	1	11	1	3
NA	NA	NA	NA	NA	NA	1	3	0.8	1	1	3	2	2	1	1	1	3
NA	NA	NA	NA	NA	NA	1	1	2.7	1	2	1	2	1	1	1	2	1
						0	0	0	0	0	0	0	0	0	14	2	0
NA	NA	NA	NA	NA	NA	2	1	1.5	1	2	1	1	1	1	1	1	1
NA	NA	NA	NA	NA	NA	0	0	1.2	2	0	0	0	3	1	9	0	1
NA	NA	NA	NA	NA	NA	1	1	1	1	1	1	1	1	1	2	2	0
NA	NA	NA	NA	NA	NA	1	0	2.4	1	2	0	1	2	1	1	2	1
NA	NA	NA	NA	NA	NA	0	1	0.7	0	1	0	1	1	1	##	0	7
NA	Phenanthr	Benzene a	NA	NA	NA	0	1	0.1	1	2	0	1	4	6	1	0	11
Cholestan	Steroids a	Steroids a	Lipid meta	Steroid bi	map0010(	1	1	6.9	1	7	1	1	1	2	4	7	1
NA	NA	NA	NA	NA	NA	1	1	1.5	0	2	1	0	1	2	1	1	21
NA	NA	NA	NA	NA	NA	1	2	1.2	1	0	20	2	2	0	4	1	2
NA	NA	NA	NA	NA	NA	1	1	1.2	1	1	1	1	1	1	1	1	0
NA	NA	NA	NA	NA	NA	1	1	1.1	1	1	6	1	1	1	1	1	2
NA	NA	NA	NA	NA	NA	1	3	0.6	1	1	2	1	5	1	0	1	1
NA	NA	NA	NA	NA	NA	1	1	1.3	1	1	8	2	1	1	1	1	1
Triradylcgl	Glycerolipi	Glycerolipi	NA	NA	NA	1	1	1.9	1	2	1	2	1	1	1	2	1
Carbohydr	Organoox	Carbohydr	NA	NA	map0201(	1	2	2.1	1	2	1	3	1	1	1	1	1
Alcohols a	Organoox	Alcohols	NA	NA	NA	1	1	0.8	1	9	1	10	4	6	1	1	1
						0	0	0	0	0	0	0	0	0	0	0	0
NA	NA	NA	NA	NA	NA	1	1	1	1	1	1	1	8	2	2	2	0
NA	NA	NA	NA	NA	NA	1	2	0.3	0	2	0	1	2	2	2	0	1
NA	NA	NA	NA	NA	NA	2	1	1.1	2	3	2	2	3	2	0	2	0
NA	NA	NA	NA	NA	NA	1	1	1	1	1	2	1	1	1	1	1	1

NA	NA	NA	NA	NA	NA	1	3	1.2	1	1	1	1	1	1	2	1	1
NA	NA	NA	NA	NA	NA	1	1	0.8	1	0	1	1	1	4	1	2	1
Benzoxazc	Benzoxazc	Organohe	NA	NA	NA	1	1	2	1	2	1	2	1	1	1	2	1
NA	NA	NA	NA	NA	NA	0	1	1.1	0	2	0	1	4	2	2	1	3
NA	NA	NA	NA	NA	NA	1	1	1.6	1	2	1	1	1	1	1	2	1
Furanones	Dihydrofu	Organohe	Xenobiotic	Chlorocycl	NA	3	1	0.6	2	0	1	0	1	3	3	1	2
NA	NA	NA	NA	NA	NA	1	1	1.5	1	2	1	1	1	1	1	1	1
Androstan	Steroids ar	Steroids ar	NA	NA	NA	1	1	1.6	1	1	1	2	3	1	1	2	1
NA	NA	NA	NA	NA	NA	1	2	0.2	2	0	2	0	5	1	1	1	0
NA	NA	NA	NA	NA	NA	1	1	0.9	1	1	1	4	4	2	1	1	1
NA	NA	NA	NA	NA	NA	1	1	0.7	1	1	2	1	2	1	1	1	1
NA	NA	NA	NA	NA	NA	2	0	2.3	3	1	1	1	3	1	1	1	3
NA	NA	NA	NA	NA	NA	1	1	0.9	2	1	1	1	1	0	2	3	2
NA	NA	NA	NA	NA	NA	0	4	0.6	1	4	2	0	0	1	1	0	1
NA	NA	NA	NA	NA	NA	1	1	1.8	1	1	2	2	2	2	1	1	4
NA	NA	NA	NA	NA	NA	1	1	1.1	1	1	1	1	1	1	1	1	1
NA	NA	NA	NA	NA	NA	1	1	0.3	2	0	1	0	2	1	1	1	5
NA	NA	NA	NA	NA	NA	1	1	2.7	1	2	1	2	1	1	2	2	1
Amino aci	Carboxylic	Amino aci	Amino aci	Arginine b	map00220	2	0	0.3	5	0	0	0	2	0	2	0	3
NA	NA	NA	NA	NA	NA	1	1	1.4	1	1	1	1	1	1	1	2	1
NA	NA	NA	NA	NA	NA	1	1	1.8	1	2	1	2	1	1	1	2	2
NA	NA	NA	NA	NA	NA	1	0	43	2	9	15	2	1	0	1	0	3
NA	NA	NA	NA	NA	NA	2	1	0.5	2	1	1	1	2	0	2	1	1
NA	NA	NA	NA	NA	NA	1	1	7.4	3	2	1	2	1	1	3	1	0
NA	NA	NA	NA	NA	NA	1	1	2.4	1	2	1	2	0	2	1	2	1
Glyceroph	Glyceroph	Glyceroph	NA	NA	NA	1	1	0.9	1	3	0	1	1	3	1	1	3
NA	NA	NA	NA	NA	NA	1	1	1.3	1	2	1	1	1	1	1	1	1
NA	NA	NA	NA	NA	NA	2	1	1	2	1	3	4	2	1	0	2	1
NA	NA	NA	NA	NA	NA	1	11	0.4	1	0	0	1	1	9	1	0	5
Aminoquir	Quinolines	Organohe	NA	NA	NA	1	1	2.1	1	2	3	2	1	1	1	2	0
Glyceroph	Glyceroph	Glyceroph	NA	NA	NA	0	2	0	1	1	0	1	2	4	2	0	5
Amino aci	Carboxylic	Amino aci	NA	NA	NA	1	1	1	1	1	1	1	1	65	1	1	1
NA	NA	NA	NA	NA	NA	2	1	1.3	1	1	1	1	1	6	1	1	1
NA	NA	NA	NA	NA	NA	1	1	0.9	1	1	1	1	1	1	1	2	1
NA	NA	NA	NA	NA	NA	1	1	0.6	1	3	1	1	1	3	1	1	1
NA	NA	NA	NA	NA	NA	1	1	0.4	1	0	1	0	1	1	1	1	2
Short-chai	Keto acids	Organic ac	NA	NA	NA	1	1	2	1	2	1	2	2	1	1	1	0
NA	NA	NA	Biosynthes	Carbapene	NA	1	1	1.5	1	2	1	1	1	1	1	2	1
NA	NA	NA	NA	NA	NA	1	1	2.2	1	2	1	2	1	1	1	2	0
Steroidal g	Steroids ar	Steroids ar	NA	NA	NA	0	0	0.7	15	0	0	0	2	1	25	13	1
NA	NA	NA	Biosynthes	Indole dite	NA	2	1	1.3	1	2	2	2	1	1	1	1	1
NA	NA	NA	NA	NA	NA	10	0	1	6	0	0	1	12	17	18	1	7
NA	NA	NA	NA	NA	NA	2	1	0.9	2	0	1	0	2	0	2	2	1
NA	NA	NA	NA	NA	NA	1	1	1.5	1	2	1	1	1	1	1	2	1
Amino aci	Carboxylic	Amino aci	NA	NA	NA	8	1	2	1	1	2	1	0	1	1	1	1
NA	NA	NA	NA	NA	NA	1	1	1.4	1	1	5	2	1	1	1	1	1
Carbonyl c	Organoox	Organic o:	NA	NA	NA	0	3	1.7	1	1	3	5	1	1	1	1	2
NA	NA	NA	NA	NA	NA	1	1	2.2	1	2	1	2	1	1	1	2	0
NA	NA	NA	NA	NA	NA	1	1	0.9	1	1	1	1	5	2	1	1	1
NA	NA	NA	NA	NA	NA	1	1	1.3	1	1	1	1	1	2	1	1	1
Glyceroph	Glyceroph	Glyceroph	NA	NA	NA	0	1	0.1	1	1	0	2	7	1	1	0	3
NA	NA	NA	NA	NA	NA	1	1	0.7	1	1	1	1	0	1	1	1	5
NA	Purine nuc	Purines an	NA	NA	NA	1	6	0.2	2	0	2	0	4	0	1	1	3
NA	NA	NA	NA	NA	NA	2	1	0.4	1	0	1	0	1	1	1	0	2
Pyrroloind	Indoles an	Indole anc	NA	NA	NA	1	2	1.3	3	0	1	0	1	0	1	0	0
NA	NA	NA	NA	NA	NA	1	1	1.3	1	1	1	1	1	1	1	1	2
NA	NA	NA	NA	NA	NA	5	2	2	3	1	11	1	2	1	1	2	1
Terpene la	Prenol lipi	Terpenoid	NA	NA	NA	1	1	0.6	3	0	0	0	1	0	2	0	0

NA	NA	NA	NA	NA	NA	3	2	1.2	0	0	2	0	1	0	5	1	4
						0	0	0	0	0	0	0	0	14	0	0	0
NA	NA	NA	NA	NA	NA	1	1	2	0	1	1	0	2	0	7	1	7
NA	NA	NA	NA	NA	NA	1	1	1.5	1	2	1	1	1	1	1	2	1
NA	NA	NA	NA	NA	NA	1	1	1.8	1	2	1	2	1	1	1	2	1
Glyceroph	Glyceroph	Glyceroph	NA	NA	NA	1	1	1.9	1	2	1	2	2	1	1	2	1
NA	NA	NA	NA	NA	NA	1	1	1.8	1	2	1	2	1	1	1	1	2
NA	NA	NA	NA	NA	NA	2	1	1	4	1	0	0	0	1	3	0	0
NA	NA	NA	NA	NA	NA	3	0	0.4	1	1	10	0	6	1	0	1	4
						0	0	0	0	0	0	0	0	0	0	0	0
						0	0	0	0	0	0	0	0	0	0	0	0
NA	NA	NA	NA	NA	NA	10	1	1.2	5	1	1	1	3	1	2	1	1
NA	NA	NA	NA	NA	NA	1	1	0.9	1	1	0	1	1	2	1	1	2
NA	NA	NA	NA	NA	NA	42	2	0.7	1	1	1	1	1	1	1	1	5
NA	NA	NA	NA	NA	NA	2	1	0.8	2	1	1	1	1	2	11	1	1
NA	NA	NA	NA	NA	NA	1	1	1.1	2	0	2	0	2	1	2	2	0
NA	Phenol eth	Benzene a	NA	NA	NA	1	1	1.3	1	1	1	2	17	1	6	1	49
NA	NA	NA	NA	NA	NA	1	1	1	1	1	9	1	1	1	1	1	1
NA	NA	NA	NA	NA	NA	0	2	5	1	0	11	1	1	0	1	6	0
NA	NA	NA	NA	NA	NA	1	1	1.9	1	2	1	2	1	1	1	2	1
NA	NA	NA	NA	NA	NA	1	1	0.3	1	1	2	1	3	3	3	1	1
NA	NA	NA	NA	NA	NA	1	1	2.5	1	1	1	1	2	1	1	1	1
NA	NA	NA	NA	NA	NA	1	1	1.3	1	36	1	2	1	1	1	1	1
Indoles	Indoles an	Indoles	Amino aci	Tryptopha	map00380	2	1	0.9	3	1	4	4	2	1	0	2	2
NA	NA	NA	NA	NA	NA	1	2	0.8	1	35	1	9	1	2	1	1	21
NA	NA	NA	NA	NA	NA	0	0	66	0	7	14	2	1	0	2	0	2
NA	NA	NA	NA	NA	NA	1	1	2.3	1	1	2	3	1	1	1	2	1
NA	NA	NA	NA	NA	NA	1	1	0.8	1	11	1	2	2	1	1	1	1
						0	0	0	0	0	0	0	0	0	25	0	0
Phosphate	Organic pl	Organic ac	Lipid meta	Glyceroph	map00564	3	1	7.8	2	3	1	9	0	10	0	1	0
NA	NA	NA	NA	NA	NA	4	12	1.4	1	1	##	2	1	4	1	1	33
NA	NA	NA	NA	NA	NA	1	1	1.1	2	0	3	0	1	0	2	2	0
Glyceroph	Glyceroph	Glyceroph	NA	NA	NA	0	2	0	1	0	0	0	4	3	4	0	3
NA	NA	NA	NA	NA	NA	1	1	2	1	2	1	3	1	2	1	1	0
NA	NA	NA	NA	NA	NA	0	3	0.3	0	1	0	1	3	3	2	0	34
NA	NA	NA	NA	NA	NA	5	1	0.9	0	1	1	1	1	1	1	1	5
NA	NA	NA	NA	NA	NA	4	3	0.8	4	1	45	0	0	1	1	6	1
NA	NA	NA	NA	NA	NA	2	1	0.6	1	1	1	1	1	1	2	1	1
Benzoic ac	Benzene a	Benzene a	NA	NA	NA	1	1	1.5	1	33	1	2	1	1	1	1	1
NA	NA	NA	NA	NA	NA	1	1	1.3	1	1	1	2	1	1	1	1	2
NA	NA	NA	NA	NA	NA	1	1	0.9	1	1	1	0	1	5	1	1	1
PK1205 Isc	PK12 Flavc	Polyketide	NA	NA	NA	2	1	0.4	3	0	0	0	1	0	1	0	0
NA	NA	NA	NA	NA	NA	1	1	0.9	3	0	2	0	2	1	2	2	0
NA	NA	NA	NA	NA	NA	1	1	0.7	1	1	1	4	2	1	1	2	1
NA	NA	NA	Biosynthes	Neomycin	map00524	1	1	1.4	1	1	2	1	1	1	2	1	1
NA	NA	NA	NA	NA	NA	1	4	0.4	2	2	1	0	0	1	0	1	1
NA	NA	NA	NA	NA	NA	1	1	1.1	1	2	1	2	2	1	1	1	1
NA	NA	NA	NA	NA	NA	3	1	0.8	1	1	1	1	1	1	1	1	3
NA	NA	NA	NA	NA	NA	1	1	1.4	0	2	32	8	1	3	1	3	0
NA	NA	NA	NA	NA	NA	1	##	1.1	1	1	1	1	1	1	1	1	1
						29	0	0	0	0	0	0	27	0	0	0	0
NA	NA	NA	NA	NA	NA	1	1	0.9	1	1	1	1	1	1	1	1	1
Fatty acid	Fatty Acyls	Fatty acyls	NA	NA	NA	1	0	1.3	1	2	1	1	0	1	1	2	1
NA	NA	NA	NA	NA	NA	1	1	1.2	8	1	1	2	0	1	1	17	1
NA	NA	NA	NA	NA	NA	1	2	0.3	1	1	0	1	1	1	0	0	1
NA	NA	NA	NA	NA	NA	1	0	0.8	1	1	26	1	1	1	0	2	1
NA	NA	NA	NA	NA	NA	1	1	0.9	1	0	1	1	1	1	1	1	1
NA	NA	NA	NA	NA	NA	1	1	0.9	1	2	1	1	4	2	1	1	1

NA	NA	NA	NA	NA	NA	1	1	2.4	1	2	1	2	1	1	1	2	0
						0	0	0	6	42	0	4	28	38	54	0	0
Amino aci	Carboxylic	Amino aci	NA	NA	NA	3	0	0.9	2	1	5	2	1	1	0	3	3
NA	NA	NA	NA	NA	NA	1	1	1.6	1	1	2	1	1	1	1	1	1
Benzoic ac	Benzene a	Benzene a	NA	NA	NA	2	0	1.5	3	2	1	1	0	2	1	3	0
NA	NA	NA	NA	NA	NA	2	1	0.6	7	0	1	0	3	0	4	1	0
NA	NA	NA	NA	NA	NA	1	1	0.8	1	1	1	1	1	0	3	1	0
NA	NA	NA	NA	NA	NA	1	1	0.8	1	0	1	1	1	1	1	1	1
Amino aci	Carboxylic	Amino aci	Amino aci	Valine, leu	map0028	1	2	2.1	0	2	2	2	1	1	1	1	0
NA	NA	NA	NA	NA	NA	2	1	4.1	6	2	0	3	1	0	11	1	1
NA	NA	NA	NA	NA	NA	1	1	2.4	0	2	1	2	2	2	2	2	1
NA	NA	NA	NA	NA	NA	1	1	1	1	1	0	1	1	1	1	1	2
O-methyla	Flavonoids	Flavonoids	Biosynthes	Flavonoid	NA	1	1	1.2	1	1	1	2	1	0	24	2	1
NA	NA	NA	NA	NA	NA	1	1	1	1	1	1	2	1	1	1	1	4
NA	NA	NA	NA	NA	NA	38	0	0.5	2	1	5	1	3	0	3	1	0
Pyrimidine	Diazines	Organohe	NA	NA	NA	2	4	1.8	6	1	1	3	2	1	0	5	1
Beta lactar	Lactams	Organohe	NA	NA	NA	4	1	1.1	1	1	40	1	0	1	1	1	3
Furanones	Dihydrofu	Organohe	Carbohydr	Ascorbate	map0053	2	0	0.1	0	3	0	0	1	4	0	1	3
NA	NA	NA	NA	NA	NA	1	1	2.4	1	2	1	2	1	1	1	1	1
NA	NA	NA	NA	NA	NA	1	1	0.6	1	1	1	1	1	2	0	2	1
Carbohydr	Organoox	Carbohydr	NA	NA	NA	1	1	0.2	0	1	6	4	16	48	0	1	2
NA	NA	NA	NA	NA	NA	1	1	1.1	2	1	1	0	1	0	2	1	0
Biphenyls	Benzene a	Benzene a	NA	NA	NA	1	1	1.6	1	2	1	2	1	1	1	1	1
						28	0	0	0	0	0	0	0	0	0	0	0
NA	NA	NA	NA	NA	NA	0	3	0.5	0	0	0	1	2	0	1	2	16
NA	NA	NA	NA	NA	NA	1	1	1.1	1	1	1	1	0	1	1	1	1
NA	NA	NA	NA	NA	NA	1	1	1.5	1	2	1	1	1	1	1	2	1
NA	NA	NA	NA	NA	NA	0	3	0.5	0	1	0	1	6	2	3	0	1
NA	NA	NA	NA	NA	NA	0	2	1.5	1	1	0	2	5	6	1	1	1
NA	NA	NA	NA	NA	NA	2	1	1.7	2	0	2	0	1	0	5	1	0
NA	NA	NA	NA	NA	NA	2	1	1.6	1	1	1	2	1	1	1	1	1
Androstan	Steroids ai	Steroids ai	Lipid meta	Steroid ho	map0014	1	1	1	1	1	15	1	2	2	1	1	1
NA	NA	NA	NA	NA	NA	3	1	0.3	0	0	9	0	2	0	0	0	0
Imidazolid	Azolidines	Organohe	NA	NA	NA	1	1	1.5	1	1	1	2	1	1	1	1	1
NA	NA	NA	NA	NA	NA	5	1	0.7	2	1	1	1	1	1	0	0	3
NA	NA	NA	NA	NA	NA	1	5	0.9	0	1	1	1	3	1	1	1	12
Sesquiterp	Prenol lipi	Terpenoid	NA	NA	NA	1	2	8.5	2	3	2	3	2	1	1	1	0
NA	NA	NA	NA	NA	NA	2	1	0.6	6	1	0	0	2	0	12	0	0
						0	0	0	0	22	0	0	0	37	0	0	0
1,4-benzo	Benzodiaz	Organohe	NA	NA	NA	1	1	1.7	1	2	1	2	1	1	1	1	1
NA	NA	NA	NA	NA	NA	1	1	0.8	1	0	1	0	1	6	1	1	1
NA	NA	NA	NA	NA	NA	2	2	0.6	1	0	2	5	1	1	1	2	0
NA	NA	NA	NA	NA	NA	1	1	1.2	1	2	1	1	1	2	1	2	1
						0	0	0	0	0	0	0	0	0	0	0	0
						0	0	0	0	0	0	0	0	0	0	0	0
NA	NA	NA	NA	NA	NA	1	1	1.4	1	1	1	2	1	1	1	1	1
NA	NA	NA	NA	NA	NA	2	1	0.5	1	0	1	0	0	2	1	1	17
NA	NA	NA	Biosynthes	Carbapene	NA	1	1	1.8	1	2	2	2	1	1	1	1	1
NA	NA	NA	NA	NA	NA	1	1	1.1	1	1	1	1	1	1	1	1	1
						0	0	0	0	0	0	0	0	0	0	0	0
NA	NA	NA	NA	NA	NA	1	4	1.5	1	2	1	4	4	1	1	1	1
Carbohydr	Organoox	Carbohydr	NA	NA	NA	4	1	1.1	2	2	1	1	1	4	1	1	1
Sulfated st	Steroids ai	Steroids ai	NA	NA	NA	1	1	1.8	1	2	1	2	2	1	1	1	3
NA	NA	NA	NA	NA	NA	1	1	1.1	1	2	1	1	1	2	1	2	1
NA	NA	NA	NA	NA	NA	1	1	0.9	1	1	1	1	5	2	1	2	3
Carbohydr	Organoox	Carbohydr	NA	NA	NA	1	1	0.8	1	0	1	1	1	3	1	2	1
NA	NA	NA	NA	NA	NA	1	1	1	1	1	1	1	1	1	2	2	0
NA	NA	NA	NA	NA	NA	1	1	5.7	1	2	54	2	1	1	1	2	1

NA	NA	NA	NA	NA	NA	2	1	1.6	1	2	1	2	2	1	1	1	1
Carboxylic	Carboxylic	Organic ac	NA	NA	NA	1	1	2.2	1	2	1	2	0	4	1	2	0
NA	NA	NA	NA	NA	NA	1	1	1.6	1	2	1	2	2	2	1	1	2
Eicosanoic	Fatty Acyls	Fatty acyls	Lipid meta	Arachidon	map00590	##	1	186	1	1	1	1	1	1	1	1	1
NA	NA	NA	NA	NA	NA	1	1	0.9	1	0	1	1	1	1	1	1	1
						0	0	0	0	5	0	0	0	0	0	0	0
NA	NA	NA	NA	NA	NA	2	1	0.9	3	1	1	1	0	2	1	0	3
NA	NA	NA	NA	NA	NA	1	1	2.5	1	2	1	2	1	1	1	2	0
NA	NA	NA	NA	NA	NA	1	1	1	1	1	3	1	1	1	1	1	1
NA	NA	NA	NA	NA	NA	1	2	0.1	2	0	0	0	3	4	3	0	10
NA	NA	NA	NA	NA	NA	1	1	1.9	3	0	1	0	2	0	1	1	0
Amino aci	Carboxylic	Amino aci	NA	NA	NA	1	1	0.8	1	1	1	1	1	1	1	1	1
NA	NA	NA	NA	NA	NA	4	1	1.2	1	1	7	2	1	1	1	2	1
NA	NA	NA	NA	NA	NA	1	1	1.9	1	2	33	7	1	3	1	3	0
NA	NA	NA	NA	NA	NA	2	0	0.5	1	0	1	0	5	2	1	0	43
NA	NA	NA	NA	NA	NA	1	1	0.7	1	1	1	1	1	2	1	1	1
NA	NA	NA	NA	NA	NA	1	1	0.9	1	1	1	1	2	1	1	1	2
NA	NA	NA	NA	NA	NA	8	1	1.1	1	1	4	1	1	1	1	1	1
NA	NA	NA	NA	NA	NA	1	2	0.4	2	0	1	0	5	0	3	4	1
NA	NA	NA	NA	NA	NA	1	1	1.4	1	2	1	2	5	1	1	1	1
NA	NA	NA	NA	NA	NA	1	1	2.2	1	2	1	2	1	1	3	1	0
NA	NA	NA	NA	NA	NA	2	1	1.3	1	2	1	2	5	1	1	1	1
NA	NA	NA	NA	NA	NA	2	1	2.4	2	1	3	1	1	1	1	1	10
1-benzopy	Benzopyra	Organohe	NA	NA	NA	1	1	1.5	1	2	2	3	1	1	1	1	1
NA	NA	NA	NA	NA	NA	1	1	2.5	1	2	1	2	1	1	1	2	0
NA	NA	NA	NA	NA	NA	1	6	0.3	1	1	1	0	7	0	0	2	2
NA	NA	NA	NA	NA	NA	0	2	1.5	1	1	1	3	6	1	0	0	0
NA	NA	NA	NA	NA	NA	1	1	1.6	1	2	1	2	1	2	1	1	1
NA	NA	NA	NA	NA	NA	1	1	1.3	0	2	3	3	1	1	1	2	0
NA	NA	NA	NA	NA	NA	1	1	2	1	2	2	3	1	1	1	1	1
Fatty acids	Fatty Acyls	Fatty acyls	NA	NA	NA	1	1	0.4	0	0	0	0	4	1	2	0	35
NA	NA	NA	NA	NA	NA	1	1	0.8	1	1	1	1	1	1	1	1	1
NA	NA	NA	NA	NA	NA	48	8	1.1	1	1	2	1	1	1	1	1	1
NA	NA	NA	Xenobiotic	Dioxin deg	NA	1	1	1.2	19	8	1	2	1	1	2	1	1
						0	0	0	0	0	3	32	0	0	0	0	30
NA	NA	NA	NA	NA	NA	1	2	0.7	1	2	0	3	14	1	4	0	1
NA	Morphinar	Alkaloids a	NA	NA	NA	1	1	1.3	1	2	1	1	1	1	1	2	1
Terpenoid	Alkaloids c	Alkaloids	NA	NA	NA	0	4	0.8	0	1	1	0	1	11	2	0	4
NA	NA	NA	NA	NA	NA	0	1	0.8	1	0	0	1	0	##	1	1	1
Macrolides	Diarrhetic	Marine bic	NA	NA	NA	3	1	1.5	10	3	4	3	0	1	0	2	1
Carbohydr	Organoox	Carbohydr	NA	NA	NA	2	4	1.4	1	1	8	1	1	0	1	1	5
NA	NA	NA	NA	NA	NA	1	1	1.3	1	2	2	2	1	1	1	1	1
NA	NA	NA	NA	NA	NA	6	1	0.9	1	2	0	1	1	2	0	0	3
NA	NA	NA	NA	NA	NA	2	6	0.6	1	1	1	1	4	2	1	0	2
NA	NA	NA	NA	NA	NA	1	1	0.8	1	2	1	1	1	1	1	1	1
Isoquinolir	Alkaloids c	Alkaloids	NA	NA	NA	1	2	0.7	1	1	1	1	1	1	1	1	1
NA	NA	NA	NA	NA	NA	1	1	1.4	1	1	2	2	1	1	1	1	1
Morpholin	Oxazinane	Organohe	NA	NA	NA	1	1	2.7	1	1	1	1	1	1	1	2	0
NA	NA	NA	NA	NA	NA	1	1	1.1	1	1	1	1	1	1	1	1	1
NA	NA	NA	NA	NA	NA	1	1	0.8	1	1	1	1	1	1	1	1	1
NA	NA	NA	NA	NA	NA	2	1	0.7	1	1	1	0	1	2	1	1	1
NA	NA	NA	NA	NA	NA	1	1	1.8	1	2	1	2	1	1	1	2	1
NA	NA	NA	NA	NA	NA	1	1	2.2	1	2	1	2	1	1	1	2	1
Lignan gly	Lignans	Lignans	NA	NA	NA	1	1	0.9	0	2	1	3	2	1	1	1	1
Glyceroph	Glyceroph	Glyceroph	NA	NA	NA	1	2	0.9	1	1	1	1	13	5	4	1	1
Benzoic ac	Benzene a	Benzene a	Amino aci	Phenylalar	map00360	0	2	0.2	2	1	0	1	2	0	1	1	1
NA	NA	NA	NA	NA	NA	0	2	0.4	1	26	0	1	1	1	1	0	2
NA	NA	NA	NA	NA	NA	1	1	1	1	1	3	1	1	1	1	1	1

NA	Phenylpro	Phenylpro	NA	NA	NA	1	1	0.8	2	0	2	0	2	0	1	1	1
NA	NA	NA	NA	NA	NA	1	1	0.4	3	0	1	0	1	1	1	0	1
NA	NA	NA	NA	NA	NA	1	3	1.7	1	2	2	2	8	1	1	1	1
Amino aci	Carboxylic	Amino aci	NA	NA	NA	1	1	15	1	2	1	19	1	1	2	1	1
NA	NA	NA	NA	NA	NA	1	1	1.4	1	1	1	2	1	1	1	1	9
NA	NA	NA	NA	NA	NA	1	1	0.7	1	0	1	1	1	42	1	1	1
Dicarboxyl	Carboxylic	Organic ac	NA	NA	NA	6	1	0.9	1	0	1	1	1	1	1	1	0
NA	NA	NA	NA	NA	NA	1	1	1.6	1	2	1	2	1	1	1	2	1
NA	NA	NA	NA	NA	NA	1	1	1.9	2	2	1	0	0	1	1	2	1
NA	NA	NA	NA	NA	NA	2	1	1.2	1	2	1	2	4	1	1	1	1
NA	NA	NA	NA	NA	NA	1	2	1.4	1	1	2	1	19	1	1	1	1
Carbonyl c	Organoox	Organic o	NA	NA	NA	1	2	2.1	2	1	1	2	2	1	0	0	1
Amino aci	Carboxylic	Amino aci	NA	NA	NA	1	12	0.4	1	11	1	1	1	7	1	0	8
NA	NA	NA	NA	NA	NA	3	1	1	1	1	1	1	1	1	1	1	38
NA	NA	NA	NA	NA	NA	1	1	0.9	2	2	1	1	1	1	1	1	2
NA	NA	NA	NA	NA	NA	7	1	1.1	1	1	1	1	1	1	1	1	1
NA	NA	NA	NA	NA	NA	1	1	1.2	1	1	1	3	1	1	1	1	1
Amino aci	Carboxylic	Amino aci	NA	NA	NA	2	5	1.9	9	0	1	1	5	0	2	10	1
NA	NA	NA	NA	NA	NA	1	2	8.6	6	1	1	0	1	0	9	0	0
NA	NA	NA	NA	NA	NA	1	1	1.4	1	2	1	1	1	1	1	2	1
NA	NA	NA	NA	NA	NA	2	1	1.7	1	2	1	2	1	1	1	1	1
NA	NA	NA	NA	NA	NA	0	2	0.5	1	##	1	##	2	##	1	0	1
NA	NA	NA	NA	NA	NA	1	1	1.3	1	1	1	2	1	1	1	1	1
NA	NA	NA	NA	NA	NA	2	2	0.4	1	1	1	0	1	1	1	1	1
NA	NA	NA	NA	NA	NA	1	1	1	1	1	1	0	5	1	1	1	1
NA	NA	NA	NA	NA	NA	0	73	1.3	1	1	1	##	0	1	1	1	1
NA	NA	NA	NA	NA	NA	1	1	1.3	1	1	2	1	1	1	1	1	23
NA	NA	NA	NA	NA	NA	1	1	1.9	1	2	1	2	1	1	1	2	1
NA	NA	NA	NA	NA	NA	1	0	1.8	2	1	1	1	0	1	8	1	2
Indolequir	Indoles an	Indole anc	Global anc	Biosynthes	NA	1	1	1.6	1	2	1	2	1	1	1	1	1
NA	NA	NA	NA	NA	NA	1	1	0.1	1	2	1	2	0	0	0	1	4
NA	NA	NA	NA	NA	NA	2	1	0.7	1	1	1	1	1	3	1	1	1
Lysergic a	Ergoline a	Alkaloids a	NA	NA	NA	1	1	0.9	2	0	3	0	1	1	2	2	0
NA	NA	NA	NA	NA	NA	##	1	1.7	1	2	1	2	1	1	1	1	1
Terpenoid	Alkaloids c	Alkaloids	NA	NA	NA	1	1	0.9	1	0	1	0	1	0	2	1	0
NA	NA	NA	NA	NA	NA	1	1	0.8	1	1	1	1	1	1	1	1	1
NA	NA	NA	NA	NA	NA	1	1	0.3	1	1	2	1	3	3	3	1	1
NA	NA	NA	NA	NA	NA	2	1	1.3	1	1	1	1	1	1	1	1	1
NA	NA	NA	NA	NA	NA	1	2	1	1	3	1	5	1	1	2	7	0
NA	NA	NA	Energy me	Methane r	map0110(	1	1	1.6	1	1	1	2	2	1	1	1	1
NA	NA	NA	NA	NA	NA	1	1	0.6	1	2	2	3	0	13	3	1	1
NA	NA	NA	NA	NA	NA	1	1	0.7	1	1	88	1	0	1	0	0	1
NA	NA	NA	NA	NA	NA	1	3	0.4	1	1	0	1	1	2	0	0	7
Amino aci	Carboxylic	Amino aci	NA	NA	NA	1	1	1.3	1	2	1	1	1	2	1	2	1
NA	NA	NA	NA	NA	NA	1	1	3.2	1	15	1	3	1	4	1	1	1
Limonoids	Triterpeno	Terpenoid	NA	NA	NA	1	1	2.3	1	0	1	0	0	1	2	0	0
NA	NA	NA	Biosynthes	Staurospo	map0110(	2	1	1.6	1	1	2	2	1	1	1	1	1
NA	NA	NA	NA	NA	NA	2	1	1.7	1	2	1	2	1	1	1	1	1
Piperazine	Diazinanes	Organohe	NA	NA	NA	1	1	1.3	1	1	1	2	1	1	1	1	1
NA	NA	NA	Metabolisı	Porphyrin	map0086(	1	1	0.8	1	0	1	0	1	6	1	1	1
Estrane ste	Steroids a	Steroids a	Lipid meta	Steroid ho	map0014(	1	4	0.5	1	0	0	0	1	1	1	0	2
NA	NA	NA	NA	NA	NA	1	1	1	1	4	1	3	1	1	1	2	2
NA	NA	NA	NA	NA	NA	1	2	0.9	1	1	1	0	2	1	2	2	0
NA	NA	NA	NA	NA	NA	1	1	0.2	0	1	1	2	1	1	0	1	2
NA	NA	NA	NA	NA	NA	1	1	1.5	1	3	2	1	1	1	1	1	1
NA	NA	NA	NA	NA	NA	1	2	0.9	1	1	1	0	1	1	2	1	##
NA	NA	NA	Carbohydı	Amino suç	map0052(	36	1	1.1	1	1	1	3	1	1	1	1	1
NA	NA	NA	NA	NA	NA	1	1	1.6	1	2	1	2	2	1	1	1	1

NA	NA	NA	NA	NA	NA	2	1	1.9	4	0	1	0	1	0	2	1	0
Fatty acyl	Fatty Acyls	Fatty acyls	NA	NA	NA	1	2	0.3	1	1	1	1	3	1	0	0	1
						0	0	0	0	0	0	11	0	0	0	0	0
NA	NA	NA	NA	NA	NA	2	1	0.7	1	1	1	0	1	2	1	1	1
						0	0	0	0	0	0	0	0	26	0	0	0
						0	0	0	0	0	0	0	0	0	0	0	0
NA	NA	NA	NA	NA	NA	2	0	0.7	2	0	0	0	1	19	3	2	1
Amino aci	Carboxylic	Amino aci	Amino aci	Alanine, as	map0025	1	1	1.6	1	2	1	2	1	1	1	1	1
NA	NA	NA	NA	NA	NA	1	1	2.2	1	2	1	2	1	1	1	2	0
NA	NA	NA	NA	NA	NA	1	0	0.1	1	0	1	0	0	2	1	2	53
NA	NA	NA	NA	NA	NA	1	1	0.5	2	0	3	1	38	0	1	1	1
NA	NA	NA	NA	NA	NA	1	1	0.9	1	1	1	1	4	2	1	1	1
NA	NA	NA	NA	NA	NA	1	1	1.6	2	1	2	1	1	0	1	1	1
Steroidal g	Steroids ar	Steroids ar	NA	NA	NA	1	1	0.6	1	1	3	1	3	1	1	1	1
						0	0	0	0	3	0	0	9	11	31	0	0
NA	NA	NA	NA	NA	NA	1	2	0.9	1	1	1	1	2	1	1	1	3
NA	NA	NA	NA	NA	NA	16	1	0.7	2	1	1	0	1	2	1	1	1
Benzylami	Benzene a	Amines an	NA	NA	NA	1	1	0.7	1	1	1	3	1	1	1	1	1
NA	NA	NA	NA	NA	NA	4	3	23	6	1	3	0	0	1	##	23	0
NA	NA	NA	NA	NA	NA	1	1	2.2	1	2	1	2	1	1	1	2	2
NA	NA	NA	NA	NA	NA	1	1	1.4	1	2	1	1	1	1	1	2	1
NA	NA	NA	NA	NA	NA	11	1	1.9	1	2	1	2	1	1	1	2	1
Porphyrins	Tetrapyrro	Organohe	NA	NA	NA	4	1	0.9	10	0	0	0	1	0	6	0	0
NA	NA	NA	NA	NA	NA	2	1	0.7	1	1	1	0	1	2	1	1	1
NA	NA	NA	NA	NA	NA	5	1	1.2	1	1	1	1	3	1	1	1	1
Terpenoid	Alkaloids (	Alkaloids	NA	NA	NA	1	1	0.7	1	1	2	1	1	1	1	1	1
Pyrimidine	Diazines	Organohe	NA	NA	NA	1	1	0.8	1	1	6	1	1	1	1	1	1
NA	NA	NA	NA	NA	NA	1	1	0.2	5	0	1	0	1	2	2	0	1
NA	NA	NA	NA	NA	NA	1	2	1.3	1	1	1	1	1	1	1	1	0
NA	NA	NA	NA	NA	NA	1	1	0.9	1	0	1	0	1	5	1	1	0
NA	NA	NA	NA	NA	NA	1	1	0.3	1	1	1	0	2	2	1	1	0
NA	NA	NA	NA	NA	NA	1	1	2.3	5	2	1	2	1	1	1	1	1
NA	NA	NA	NA	NA	NA	1	0	3.2	4	0	3	0	1	0	3	0	0
NA	NA	NA	NA	NA	NA	1	3	2.6	0	3	1	3	2	1	1	1	0
						0	0	0	0	0	0	0	0	0	0	0	0
NA	NA	NA	NA	NA	NA	1	1	0.8	1	0	1	0	1	6	1	1	1
NA	NA	NA	NA	NA	NA	2	1	1.4	1	1	1	2	1	1	1	1	1
NA	NA	NA	NA	NA	NA	6	0	1.7	1	11	##	21	0	1	1	13	0
NA	NA	NA	NA	NA	NA	1	1	1.4	1	1	3	2	16	2	1	2	4
NA	NA	NA	NA	NA	NA	2	0	1	1	1	1	1	1	3	2	1	1
NA	NA	NA	NA	NA	NA	1	1	1.2	1	1	1	1	1	2	2	1	1
NA	NA	NA	NA	NA	NA	26	1	80	1	2	1	2	1	1	1	1	1
NA	NA	NA	NA	NA	NA	0	2	0.6	0	1	15	1	1	8	1	1	3
NA	NA	NA	NA	NA	NA	2	1	0.8	4	1	0	0	1	1	3	0	1
NA	NA	NA	NA	NA	NA	1	1	1.9	1	1	1	2	1	1	2	2	1
NA	NA	NA	NA	NA	NA	1	1	0.9	1	1	1	1	2	1	1	1	1
NA	NA	NA	NA	NA	NA	2	1	1.7	1	2	1	2	1	1	1	1	1
NA	NA	NA	NA	NA	NA	4	0	1.8	1	0	0	0	3	1	5	0	2
NA	NA	NA	NA	NA	NA	1	1	0.8	1	1	24	0	1	1	1	1	1
NA	NA	NA	NA	NA	NA	4	1	0.8	2	1	1	0	1	1	3	1	1
NA	NA	NA	NA	NA	NA	10	1	1.3	1	5	1	3	1	1	5	8	1
Carbohydr	Organoox	Carbohydr	NA	NA	NA	1	1	1	1	1	1	1	1	0	2	2	##
NA	NA	NA	NA	NA	NA	0	0	0.4	0	1	1	1	5	4	1	0	1
NA	NA	NA	NA	NA	NA	1	1	0.4	1	0	0	0	0	3	1	1	0
NA	NA	NA	NA	NA	NA	0	2	0.1	3	1	1	1	1	1	2	1	1
NA	NA	NA	NA	NA	NA	1	2	0.9	1	1	1	1	3	2	2	1	1
NA	NA	NA	NA	NA	NA	20	0	1.2	1	1	3	1	2	1	3	0	1
Amino aci	Carboxylic	Amino aci	NA	NA	NA	##	0	8.5	1	4	68	1	0	0	0	0	3

NA	NA	NA	NA	NA	NA	2	1	0.8	4	1	1	1	1	1	1	1	1
Ergostane	Steroids	Terpenoid	NA	NA	NA	1	2	0.3	2	0	1	0	3	3	1	1	17
NA	NA	NA	NA	NA	NA	1	1	1.9	1	2	1	2	1	1	1	2	1
Methoxyp	Phenols	Phenols ar	NA	NA	NA	1	1	1.9	1	2	1	2	1	1	1	2	1
NA	NA	NA	NA	NA	NA	2	1	0.6	1	0	1	0	1	1	2	1	1
NA	NA	NA	NA	NA	NA	2	1	1.7	1	32	1	2	1	1	1	1	1
Benzoic ac	Benzene a	Benzene a	Xenobiotic	Aminoben	NA	0	3	1.3	1	1	1	1	0	1	1	2	0
NA	NA	NA	NA	NA	NA	1	1	1.5	1	3	1	2	2	1	1	1	1
NA	NA	NA	NA	NA	NA	1	1	2.4	1	1	1	1	##	8	9	1	0
NA	NA	NA	NA	NA	NA	2	0	0.5	5	0	1	1	1	0	7	1	0
NA	NA	NA	NA	NA	NA	1	1	1.5	1	2	1	2	1	1	1	1	1
NA	NA	NA	NA	NA	NA	1	1	1.3	1	1	1	1	1	7	1	2	1
Purines an	Imidazopy	Purines an	Biosynthes	Caffeine m	map00232	1	8	0.2	1	0	3	1	3	0	0	0	3
Pyrimidine	Pyrimidine	Nucleic ac	NA	NA	NA	1	1	1.9	1	1	1	1	1	1	1	1	2
NA	NA	NA	NA	NA	NA	3	1	0.3	8	0	0	0	0	1	5	0	1
NA	NA	NA	NA	NA	NA	2	2	1.2	3	0	0	0	1	0	3	0	0
NA	NA	NA	NA	NA	NA	1	1	1.6	1	2	1	1	1	1	1	2	1
NA	NA	NA	NA	NA	NA	1	1	0.8	1	0	1	0	1	2	1	1	1
NA	NA	NA	NA	NA	NA	1	1	0.4	3	0	0	0	0	1	3	0	1
NA	NA	NA	NA	NA	NA	2	1	0.9	4	0	1	0	1	0	2	0	0
NA	NA	NA	NA	NA	NA	1	1	1.9	1	2	19	2	1	2	1	2	0
						0	0	0	0	14	##	0	0	0	0	0	0
NA	NA	NA	NA	NA	NA	1	1	0.8	9	2	1	3	1	1	1	1	6
NA	NA	NA	NA	NA	NA	1	1	1.2	1	1	1	1	1	2	1	1	2
NA	NA	NA	NA	NA	NA	15	1	3.2	2	0	32	0	1	1	0	1	3
NA	NA	NA	NA	NA	NA	1	1	0.9	1	1	1	1	1	2	1	1	1
Pyridinesu	Pyridines a	Pyridine ar	NA	NA	NA	1	2	1.9	1	2	40	5	1	1	1	1	1
Amino aci	Carboxylic	Amino aci	NA	NA	NA	1	1	0.4	6	0	1	0	1	0	3	0	0
NA	NA	NA	NA	NA	NA	1	1	1	1	1	6	1	0	2	1	1	1
Fatty acid	Fatty Acyls	Fatty acyls	NA	NA	NA	4	2	0.6	0	0	0	0	4	1	11	0	5
NA	NA	NA	NA	NA	NA	2	1	0.8	4	1	0	0	1	1	3	0	1
						0	0	0	0	0	0	0	0	0	0	0	0
Indoles	Indoles an	Indoles	Amino aci	Tryptopha	map0038C	1	1	2	1	2	1	2	1	2	1	2	1
Lignan gly	Lignans	Lignans	NA	NA	NA	1	1	0.7	1	1	3	1	2	1	1	1	1
NA	NA	NA	NA	NA	NA	2	1	1	3	1	4	3	2	1	0	2	2
						0	0	0	0	0	0	0	0	0	0	0	0
NA	NA	NA	NA	NA	NA	1	1	2.1	1	2	1	2	2	1	1	2	1
NA	NA	NA	NA	NA	NA	0	1	2.2	5	1	2	1	5	0	0	3	2
NA	NA	NA	NA	NA	NA	1	1	0.3	7	0	0	0	1	0	3	0	0
NA	NA	NA	NA	NA	NA	0	3	0.7	0	1	2	1	3	1	1	48	3
NA	NA	NA	NA	NA	NA	1	1	1.8	1	2	1	1	1	2	1	1	0
NA	NA	NA	NA	NA	NA	2	2	0.6	4	0	10	0	0	0	0	2	5
Methoxyp	Phenols	Phenols ar	NA	NA	NA	1	0	2.1	1	3	10	0	0	1	1	2	1
NA	NA	NA	NA	NA	NA	1	1	0.8	1	0	1	1	1	1	1	2	1
NA	NA	NA	NA	NA	NA	1	1	0.9	2	2	9	1	1	1	1	2	1
NA	NA	NA	NA	NA	NA	1	1	2	4	1	1	1	0	1	1	1	3
NA	NA	NA	NA	NA	NA	1	1	1.3	1	2	1	2	1	1	1	1	1
NA	NA	NA	NA	NA	NA	1	1	1.4	1	2	2	3	1	1	1	2	1
NA	NA	NA	NA	NA	NA	1	2	1.5	1	2	1	1	1	1	1	1	0
NA	NA	NA	NA	NA	NA	8	1	0.7	2	1	1	0	11	0	1	1	1
NA	NA	NA	NA	NA	NA	38	1	0	1	11	0	0	1	42	0	0	##
NA	NA	NA	NA	NA	NA	1	0	0.3	1	0	2	1	1	##	1	1	1
NA	NA	NA	NA	NA	NA	1	1	1.4	1	2	1	2	1	1	1	1	1
Hydroxycc	Coumarins	Coumarins	NA	NA	NA	1	1	1.3	1	2	3	1	1	1	1	2	1
Amino aci	Carboxylic	Amino aci	NA	NA	NA	1	1	1.9	1	2	1	2	1	2	1	1	1
NA	NA	NA	NA	NA	NA	2	2	0.6	3	2	1	1	1	1	0	2	1
Amino aci	Carboxylic	Amino aci	NA	NA	NA	40	1	1.2	1	1	2	2	1	1	1	1	13
NA	NA	NA	NA	NA	NA	1	1	0.4	1	1	6	2	1	1	1	1	1

NA	NA	NA	NA	NA	NA	1	1	1.9	1	2	1	2	1	1	2	2	1
NA	NA	NA	NA	NA	NA	1	1	1.2	1	1	1	1	2	2	2	1	1
NA	NA	NA	NA	NA	NA	1	1	0.8	1	1	1	1	1	1	1	1	1
NA	NA	NA	NA	NA	NA	0	2	0.1	3	1	1	1	2	1	2	1	1
NA	NA	NA	NA	NA	NA	9	4	1.3	23	3	6	0	0	0	0	5	0
NA	NA	NA	NA	NA	NA	1	1	2	1	2	1	2	1	2	1	1	1
NA	NA	NA	NA	NA	NA	2	##	0.7	1	1	1	8	1	1	1	1	1
NA	NA	NA	NA	NA	NA	1	1	2.2	1	2	1	2	1	1	1	2	0
NA	NA	NA	NA	NA	NA	2	1	0.7	1	1	1	0	1	3	1	1	1
						0	0	0	0	0	0	23	40	0	0	0	0
NA	NA	NA	NA	NA	NA	2	1	1	1	1	1	1	0	1	1	1	1
NA	NA	NA	NA	NA	NA	1	1	0.9	1	1	1	1	6	1	1	1	10
NA	NA	NA	NA	NA	NA	1	1	1	1	1	1	1	4	2	1	1	1
NA	NA	NA	NA	NA	NA	6	1	1.5	1	1	1	1	1	1	1	1	1
NA	NA	NA	NA	NA	NA	1	1	0.9	2	1	1	1	1	1	1	0	2
NA	NA	NA	NA	NA	NA	1	1	1.2	1	1	1	1	1	1	8	3	2
NA	NA	NA	NA	NA	NA	28	1	1	1	1	24	1	1	1	1	1	3
NA	NA	NA	NA	NA	NA	1	2	0.2	0	2	0	1	3	4	2	0	4
NA	NA	NA	NA	NA	NA	2	1	0.8	1	1	1	0	1	1	1	1	1
NA	NA	NA	NA	NA	NA	2	1	0.9	2	1	1	1	0	2	1	1	3
Stilbene gl	Stilbenes	Polyketide	NA	NA	NA	0	3	0.3	2	0	0	0	19	11	1	2	43
NA	NA	NA	NA	NA	NA	0	2	0.4	1	0	0	0	6	1	0	0	0
NA	NA	NA	NA	NA	NA	1	3	1	1	1	1	2	1	0	1	0	1
NA	NA	NA	NA	NA	NA	1	1	1.4	1	1	2	1	1	1	1	1	1
Benzoic ac	Benzene a	Benzene a	NA	NA	NA	1	1	1.3	1	1	1	1	1	1	1	2	1
NA	NA	NA	NA	NA	NA	1	2	5	4	2	1	3	1	2	1	1	1
NA	NA	NA	NA	NA	NA	0	1	0.3	1	0	1	0	1	6	4	1	3
NA	NA	NA	Energy me	Methane r	map0110(	1	1	29	1	1	1	0	1	1	2	1	2
NA	NA	NA	NA	NA	NA	1	1	0.7	1	0	2	0	1	1	1	1	1
NA	NA	NA	NA	NA	NA	3	0	0.3	1	3	0	1	0	37	0	0	12
NA	NA	NA	NA	NA	NA	1	1	0.9	1	1	1	1	3	1	1	1	60
Indole alka	Alkaloids (	Alkaloids	Biosynthes	Indole alka	map0110(	2	1	0.7	2	1	1	0	1	2	1	1	1
NA	NA	NA	NA	NA	NA	1	1	2.3	1	2	1	2	1	1	1	2	1
Hydroxycc	Coumarins	Coumarins	NA	NA	NA	1	1	1	1	1	3	3	2	0	3	1	0
Isoquinoli	Alkaloids (	Alkaloids	NA	NA	NA	23	0	6.2	0	0	22	0	0	0	0	0	2
Fatty acids	Fatty Acyls	Fatty acyls	NA	NA	NA	2	5	21	16	17	0	5	1	0	0	1	0
NA	NA	NA	NA	NA	NA	1	2	3.4	1	2	1	2	5	1	7	6	1
Purines an	Imidazopy	Purines an	NA	NA	NA	1	9	0.4	1	1	1	1	2	1	0	0	1
NA	NA	NA	NA	NA	NA	1	1	0.6	1	1	0	1	1	1	1	1	1
NA	NA	NA	NA	NA	NA	1	0	1	4	0	8	0	2	0	1	1	23
NA	NA	NA	NA	NA	NA	2	1	0.7	0	2	2	2	2	1	1	1	3
NA	NA	NA	NA	NA	NA	1	2	0.4	1	0	2	0	4	3	2	1	7
NA	NA	NA	NA	NA	NA	1	1	1.4	1	1	2	1	1	1	1	1	1
NA	NA	NA	NA	NA	NA	1	1	0.3	0	3	1	1	1	26	0	0	2
NA	NA	NA	NA	NA	NA	1	1	1	1	1	58	1	1	1	1	1	1
NA	NA	NA	NA	NA	NA	1	1	1.1	2	0	1	0	2	0	2	1	0
Benzodiaz	Diazanaph	Organohe	NA	NA	NA	1	1	1.7	1	2	11	2	1	6	1	3	0
Benzoyl de	Benzene a	Benzene a	NA	NA	NA	1	1	1.4	1	1	4	1	1	1	1	2	1
NA	NA	NA	NA	NA	NA	2	1	1.7	1	2	1	2	44	1	1	1	1
NA	NA	NA	NA	NA	NA	1	1	0.9	1	1	1	1	1	1	1	1	1
NA	NA	NA	NA	NA	NA	1	1	1	1	1	2	1	1	1	1	1	1
Glyceroph	Glyceroph	Glyceroph	NA	NA	NA	0	4	0	1	1	0	0	5	14	4	0	13
NA	NA	NA	NA	NA	NA	2	1	1.7	1	2	1	2	1	1	1	1	1
NA	NA	NA	NA	NA	NA	1	1	0.6	6	1	3	1	1	1	0	1	2
Flavanone	Flavonoids	Flavonoids	Biosynthes	Flavonoid	NA	8	1	1.2	1	1	2	1	1	1	1	1	1
NA	NA	NA	NA	NA	NA	1	1	1	2	0	1	0	1	##	1	1	1
NA	NA	NA	NA	NA	NA	1	1	1.4	1	1	1	1	1	1	1	1	1
						0	0	0	0	##	0	22	25	24	38	0	10

NA	NA	NA	NA	NA	NA	3	1	0.8	1	0	1	0	5	6	2	1	1
Fatty acid	Fatty Acyls	Fatty acyls	NA	NA	NA	2	1	1.2	1	1	1	1	1	1	1	1	1
Estrane ste	Steroids ar	Steroids ar	NA	NA	NA	1	1	0.9	1	1	1	1	1	1	1	1	1
NA	NA	NA	NA	NA	NA	1	1	1.8	1	2	2	2	1	1	1	2	1
NA	NA	NA	NA	NA	NA	6	1	1	1	2	1	2	0	1	1	1	2
NA	NA	NA	NA	NA	NA	1	1	1.9	1	1	0	1	1	1	1	1	3
NA	NA	NA	NA	NA	NA	2	1	1	1	1	1	1	1	1	1	1	1
NA	NA	NA	NA	NA	NA	6	0	0.4	1	0	2	2	3	0	3	1	7
Benzodiaz	Diazanaph	Organohe	NA	NA	NA	1	1	1.9	1	2	1	2	1	1	1	1	1
NA	NA	NA	NA	NA	NA	3	1	1.5	1	2	1	2	1	1	1	1	3
Cardanolic	Steroids	Terpenoid	NA	NA	NA	1	1	2.6	1	2	1	2	1	1	1	2	1
NA	NA	NA	NA	NA	NA	2	1	0.7	2	1	1	0	1	1	1	1	1
NA	NA	NA	NA	NA	NA	1	5	0.7	0	1	0	1	3	2	0	0	0
NA	NA	NA	NA	NA	NA	1	1	0.5	1	0	1	0	1	2	1	1	1
NA	NA	NA	NA	NA	NA	1	1	1.8	7	2	2	2	1	1	1	1	1
NA	NA	NA	NA	NA	NA	1	1	1.1	1	1	2	1	1	2	1	1	1
NA	NA	NA	NA	NA	NA	1	1	2.5	1	2	1	2	1	1	1	2	0
NA	NA	NA	NA	NA	NA	0	1	1.1	1	2	0	2	2	1	1	1	1
NA	NA	NA	NA	NA	NA	0	2	0.1	4	1	1	1	1	1	2	2	1
NA	NA	NA	NA	NA	NA	2	1	0	##	##	##	0	0	3	0	32	0
NA	NA	NA	NA	NA	NA	1	2	1	1	3	0	7	1	1	1	4	1
NA	NA	NA	NA	NA	NA	3	3	0.4	1	0	2	0	12	2	6	0	3
NA	NA	NA	NA	NA	NA	1	1	1.3	1	1	1	1	1	1	1	2	1
NA	NA	NA	NA	NA	NA	2	1	1.6	1	2	1	2	1	1	1	1	1
Benzoic ac	Benzene a	Benzene a	NA	NA	NA	1	1	1.2	1	1	3	1	71	0	1	1	1
Hydroxycii	Cinnamic a	Polyketide	Biosynthes	Phenylpro	map0110(	0	2	0.3	2	1	0	0	2	1	1	0	1
Phenethyl	Benzene a	Amines an	NA	NA	NA	1	2	0.5	1	1	1	1	0	4	0	1	2
Glyceroph	Glyceroph	Glyceroph	NA	NA	NA	1	2	0.8	1	1	1	1	4	2	1	0	5
Glyceroph	Glyceroph	Glyceroph	NA	NA	NA	0	1	0	0	1	0	1	5	2	1	0	3
Pyrimidine	Diazines	Organohe	NA	NA	NA	1	1	0.2	1	2	0	0	0	0	1	3	24
NA	NA	NA	NA	NA	NA	1	1	0.9	1	1	0	1	1	2	1	1	2
NA	NA	NA	NA	NA	NA	##	1	10	1	1	74	1	1	1	1	2	2
NA	NA	NA	NA	NA	NA	3	1	0.3	15	2	6	0	0	1	0	29	1
NA	NA	NA	NA	NA	NA	1	1	1.6	1	2	1	2	1	1	1	1	1
NA	NA	NA	NA	NA	NA	1	1	1.1	1	1	1	1	1	1	1	1	1
NA	NA	NA	NA	NA	NA	1	2	0.2	1	3	1	1	2	3	2	0	1
NA	NA	NA	NA	NA	NA	1	1	1	1	2	1	1	5	2	1	1	1
NA	Phenol eth	Benzene a	NA	NA	NA	1	1	1	1	2	1	1	1	1	1	2	2
NA	NA	NA	NA	NA	NA	1	1	2.2	1	0	4	0	1	6	1	1	1
NA	NA	NA	NA	NA	NA	3	1	1.4	1	1	1	2	1	1	1	1	1
NA	NA	NA	NA	NA	NA	1	1	1.2	1	1	1	1	15	2	1	1	1
NA	NA	NA	NA	NA	NA	1	1	2.3	1	2	1	2	1	1	1	2	0
NA	NA	NA	NA	NA	NA	1	1	0.4	1	0	0	0	14	9	1	2	33
NA	NA	NA	NA	NA	NA	1	1	1.9	1	2	1	2	1	1	1	2	1
NA	NA	NA	NA	NA	NA	1	1	1	1	1	1	1	1	1	1	3	1
NA	NA	NA	NA	NA	NA	1	1	1.2	1	1	1	1	0	1	1	1	1
NA	NA	NA	NA	NA	NA	1	1	0.4	0	2	2	1	1	1	1	2	1
Vitamin D	Steroids ar	Vitamins a	NA	NA	NA	33	0	90	50	6	2	87	0	##	##	##	1
Indolines	Indoles an	Indole anc	NA	NA	NA	0	0	0.2	0	0	1	1	3	2	1	0	5
NA	NA	NA	NA	NA	NA	1	1	0.7	1	0	1	0	1	1	1	2	1
Ceramides	Sphingolip	Sphingolip	NA	NA	NA	4	1	0	29	6	39	3	1	3	0	10	0
Pregnane	Steroids ar	Steroids ar	NA	NA	NA	0	4	0.4	1	0	6	0	1	0	1	4	1
Flavonoid	Flavonoids	Flavonoids	NA	NA	NA	1	1	0.7	1	3	1	0	1	2	1	1	1
						0	0	0	22	6	7	19	70	47	68	0	##
NA	NA	NA	NA	NA	NA	1	1	0.7	6	0	1	0	1	2	2	1	1
NA	NA	NA	NA	NA	NA	1	1	1.4	1	1	1	1	1	1	1	2	1
NA	NA	NA	NA	NA	NA	1	1	0.1	7	3	6	1	3	1	2	6	0
NA	NA	NA	NA	NA	NA	3	1	1.4	1	1	1	1	1	1	1	1	1

NA	NA	NA	NA	NA	NA	3	1	0.1	1	4	0	1	0	60	1	0	3
NA	NA	NA	NA	NA	NA	1	1	1.2	1	2	1	1	3	1	2	1	0
NA	NA	NA	NA	NA	NA	2	1	1.3	1	1	1	1	1	1	1	1	1
NA	NA	NA	NA	NA	NA	2	1	1.7	1	2	1	2	58	1	1	1	1
NA	NA	NA	NA	NA	NA	1	1	1.9	1	2	1	2	1	1	1	1	1
NA	NA	NA	NA	NA	NA	1	1	0.6	3	0	0	0	1	0	2	0	1
NA	NA	NA	NA	NA	NA	1	1	0.7	5	0	0	0	2	0	2	0	0
NA	NA	NA	NA	NA	NA	1	0	0.6	1	0	1	0	2	2	4	1	2
NA	NA	NA	NA	NA	NA	1	2	1.6	1	2	1	2	21	1	1	1	1
Benzylethe	Benzene a	Benzene a	NA	NA	NA	1	1	2	1	2	2	2	1	1	1	2	1
NA	Benzotriaz	Organohe	NA	NA	NA	1	1	1.9	1	1	1	1	1	10	1	1	2
NA	NA	NA	NA	NA	NA	1	1	28	0	16	2	25	2	1	2	11	0
NA	NA	NA	NA	NA	NA	1	1	1.1	1	1	2	2	1	1	8	13	1
NA	NA	NA	NA	NA	NA	1	3	0.4	0	3	0	16	1	1	0	1	6
NA	NA	NA	NA	NA	NA	2	1	0.4	5	0	1	0	2	0	2	0	1
Hydroxyst	Steroids ar	Steroids ar	Lipid meta	Steroid ho	map00140	1	2	1.8	2	2	1	2	2	1	1	1	1
NA	NA	NA	NA	NA	NA	1	2	0.4	2	0	1	0	4	1	1	1	1
NA	NA	NA	NA	NA	NA	2	1	0.3	1	0	0	0	2	1	1	1	9
NA	NA	NA	Metabolis	Biosynthes	NA	1	1	1	1	1	1	1	1	1	1	1	1
NA	NA	NA	NA	NA	NA	1	1	1.5	1	2	1	2	1	2	1	1	1
Others	Mushroom	Fungal tox	NA	NA	NA	1	1	1.7	1	2	1	3	1	1	1	1	1
NA	NA	NA	NA	NA	NA	2	1	0.7	1	1	1	1	1	2	14	1	1
Fatty acids	Fatty Acyls	Fatty acyls	Lipid meta	Fatty acid	map00061	1	1	3.1	1	2	1	2	1	1	2	2	0
NA	NA	NA	NA	NA	NA	1	1	0.8	2	1	0	2	1	1	1	0	2
NA	NA	NA	NA	NA	NA	1	1	2.2	1	2	1	2	1	1	1	2	0
NA	NA	NA	NA	NA	NA	1	1	2	1	2	2	2	1	1	1	2	0
Vitamin D	Steroids ar	Vitamins a	NA	NA	NA	2	3	0.7	1	0	0	0	2	0	3	2	7
NA	NA	NA	NA	NA	NA	1	1	2.4	1	2	1	2	1	1	1	2	0
NA	NA	NA	NA	NA	NA	1	2	1.8	3	0	2	0	1	1	2	3	0
Carbohydr	Organoox	Carbohydr	NA	NA	NA	2	0	0.2	10	0	1	0	1	0	23	1	0
NA	NA	NA	NA	NA	NA	0	3	5.8	0	##	0	2	1	2	1	0	4
Other non	Other non	Homogen	NA	NA	NA	1	1	0.3	1	1	3	1	2	0	0	2	2
NA	NA	NA	NA	NA	NA	1	1	1.9	35	4	1	1	1	1	2	1	4
NA	NA	NA	NA	NA	NA	0	1	0.1	3	0	0	0	5	6	3	0	10
NA	NA	NA	NA	NA	NA	1	1	0.8	1	1	4	1	1	1	1	1	1
NA	NA	NA	NA	NA	NA	1	1	0.3	1	5	0	2	1	3	2	0	1
NA	NA	NA	NA	NA	NA	2	1	0.7	2	1	1	0	1	1	1	1	1
Flavonoid	Flavonoids	Flavonoids	NA	NA	NA	2	1	0.7	1	1	1	0	1	3	1	1	1
NA	NA	NA	NA	NA	NA	1	1	1.8	1	2	1	2	1	1	1	1	1
Amines	Organonit	Amines	Lipid meta	Sphingolip	map00600	0	0	1.2	0	1	1	2	2	38	3	1	10
NA	NA	NA	NA	NA	NA	1	1	0.8	1	1	1	1	1	3	1	2	1
Others	Sesquiterp	Terpenoid	NA	NA	NA	0	3	0.2	1	0	0	0	23	4	4	0	1
NA	NA	NA	NA	NA	NA	1	1	1.1	1	1	1	1	1	1	1	1	1
NA	NA	NA	NA	NA	NA	1	1	1.2	1	2	1	1	1	1	1	1	1
NA	NA	NA	NA	NA	NA	38	2	0.3	1	0	0	0	0	0	0	0	0
NA	NA	NA	NA	NA	NA	3	1	1.3	1	1	1	1	1	1	1	1	1
NA	NA	NA	NA	NA	NA	1	1	2.5	1	2	1	2	1	1	1	2	0
NA	NA	NA	NA	NA	NA	1	1	2.5	1	2	1	2	1	1	1	2	0
NA	NA	NA	NA	NA	NA	1	1	2.5	1	2	1	2	1	1	1	2	0
NA	NA	NA	NA	NA	NA	2	1	1.4	1	1	1	1	1	1	1	1	1
NA	NA	NA	NA	NA	NA	1	1	1.3	1	1	1	2	1	1	1	1	2
NA	NA	NA	NA	NA	NA	1	1	1.2	1	2	1	2	1	2	1	1	17
NA	NA	NA	NA	NA	NA	98	1	0.8	1	1	2	1	1	1	1	1	1
NA	NA	NA	NA	NA	NA	10	1	0.9	2	4	8	1	0	2	1	0	0
NA	NA	NA	NA	NA	NA	1	1	1.9	1	2	2	2	1	1	1	1	1
NA	Thiochrom	Organohe	NA	NA	NA	1	1	1.6	1	2	1	2	1	1	1	1	1
NA	Benzene a	Benzene a	NA	NA	NA	1	1	2.1	1	2	1	2	1	1	1	2	1
NA	NA	NA	NA	NA	NA	1	1	2.5	1	2	1	2	1	1	1	2	0

NA	NA	NA	NA	NA	NA	1	1	1.7	1	2	1	2	1	1	1	1	1
						0	0	0	6	##	0	0	24	11	0	0	0
NA	NA	NA	NA	NA	NA	1	1	1.5	1	1	1	2	5	1	1	1	12
NA	NA	NA	NA	NA	NA	0	1	0.3	1	3	1	2	2	2	1	2	8
						0	0	0	0	0	0	21	0	0	0	0	0
NA	NA	NA	NA	NA	NA	1	1	1.2	1	1	1	1	1	2	1	1	1
NA	NA	NA	NA	NA	NA	1	1	1.4	1	1	1	2	1	1	1	1	1
Gamma-k	Keto acids	Organic a	Carbohydr	Citrate cyc	map0002C	1	5	1.5	1	2	4	3	1	0	1	1	1
Non-meta	Non-meta	Homogen	Energy me	Oxidative	map0019C	1	1	2.3	1	2	1	3	0	1	1	1	1
NA	NA	NA	NA	NA	NA	1	1	1	1	1	1	1	1	1	1	1	1
NA	NA	NA	NA	NA	NA	3	0	0.1	1	0	0	0	1	7	1	0	1
						0	0	0	4	##	2	4	38	0	##	0	0
NA	NA	NA	NA	NA	NA	1	1	0.6	1	1	1	1	1	1	1	1	2
Phenylpyri	Pyridines a	Pyridine ar	NA	NA	NA	2	0	0.5	1	0	1	0	1	1	2	1	0
						0	0	0	19	39	0	0	29	54	66	0	0
NA	NA	NA	NA	NA	NA	1	1	2.5	1	2	1	2	1	1	1	2	0
Diradylgly	Glycerolipi	Glycerolipi	NA	NA	NA	14	1	1.2	8	2	1	1	2	1	10	1	1
NA	NA	NA	NA	NA	NA	1	1	1.6	1	1	2	2	1	1	1	1	1
						0	0	0	0	0	0	0	0	0	0	0	0
						0	0	0	0	0	0	0	0	0	0	0	0
NA	NA	NA	NA	NA	NA	1	5	0.4	1	0	2	0	4	1	1	0	1
NA	NA	NA	NA	NA	NA	1	1	2.5	1	2	1	2	1	1	1	2	0
NA	NA	NA	NA	NA	NA	1	1	0.9	1	1	1	2	1	2	1	1	1
NA	NA	NA	NA	NA	NA	4	1	0.7	1	4	0	3	1	0	1	0	2
NA	NA	NA	NA	NA	NA	1	1	2.8	1	1	1	1	2	0	6	13	1
Pregnane	Steroids a	Steroids a	NA	NA	NA	1	1	0.6	2	1	0	1	1	1	1	5	2
NA	NA	NA	NA	NA	NA	1	1	1	1	1	1	1	1	1	2	2	4
NA	NA	NA	NA	NA	NA	1	1	1.3	1	1	1	2	1	1	1	1	1
NA	NA	NA	NA	NA	NA	1	1	2.5	1	2	1	2	1	1	1	2	0
NA	NA	NA	NA	NA	NA	1	1	2.5	1	2	1	2	1	1	1	2	0
NA	NA	NA	NA	NA	NA	1	1	2.5	1	2	1	2	1	1	1	2	0
NA	NA	NA	NA	NA	NA	1	1	2.5	1	2	1	2	1	1	1	2	0
NA	NA	NA	NA	NA	NA	1	1	2.5	1	2	1	2	1	1	1	2	0
NA	NA	NA	NA	NA	NA	1	2	0.3	4	0	1	0	5	2	3	2	2
NA	NA	NA	Metabolisı	Polyketide	map0110(	1	1	1.5	1	2	3	2	1	1	1	1	1
NA	NA	NA	NA	NA	NA	1	1	0.9	1	1	1	1	1	1	1	1	1
NA	NA	NA	NA	NA	NA	1	1	1.4	1	1	1	1	1	1	1	1	1
NA	NA	NA	NA	NA	NA	0	1	0.4	2	0	1	0	1	0	1	1	2
NA	NA	NA	NA	NA	NA	1	1	1.1	1	2	1	1	2	2	1	1	2
NA	NA	NA	NA	NA	NA	5	3	0.9	4	1	1	1	0	1	1	3	2
NA	NA	NA	NA	NA	NA	1	1	0.3	4	1	1	2	14	0	1	2	0
NA	NA	NA	NA	NA	NA	1	1	1.9	3	2	2	2	1	6	1	1	0
Phenoxazi	Benzoxazi	Organohe	NA	NA	NA	1	1	0.6	1	1	1	1	1	1	1	1	3
NA	NA	NA	NA	NA	NA	5	1	1.3	1	1	1	1	1	1	1	1	1
Bilirubins	Tetrapyrro	Organohe	Metabolisı	Porphyrin	map0086C	2	1	0.4	1	0	1	0	1	1	3	1	2
NA	NA	NA	NA	NA	NA	0	1	0.3	0	0	0	0	0	2	6	0	4
NA	NA	NA	NA	NA	NA	1	1	1	1	2	1	1	1	1	1	2	2
NA	NA	NA	NA	NA	NA	1	1	1.6	1	1	1	2	1	1	1	1	1
						0	0	0	0	0	0	0	0	0	0	0	0
Steroid lac	Steroids a	Steroids a	NA	NA	NA	1	1	1.2	1	1	1	1	1	1	1	1	2
NA	NA	NA	NA	NA	NA	2	1	1.2	1	1	1	1	1	1	1	1	1
NA	NA	NA	NA	NA	NA	1	1	2.5	1	2	1	2	1	1	1	2	0
NA	NA	NA	NA	NA	NA	1	1	0.9	1	1	1	1	1	1	1	2	1
NA	NA	NA	NA	NA	NA	1	1	1.7	1	2	1	2	1	1	1	1	1
NA	NA	NA	NA	NA	NA	1	1	0.6	1	1	1	2	1	2	1	1	1
NA	NA	NA	NA	NA	NA	0	10	0.6	1	1	1	1	2	1	2	0	3
NA	NA	NA	NA	NA	NA	0	1	0.5	1	1	1	1	2	1	1	1	13
NA	NA	NA	NA	NA	NA	1	2	1.2	1	1	1	1	1	1	1	1	1

Carbonyl c	Organoox	Organic o	Xenobiotic	Bisphenol	map0110	0	3	0.3	2	1	0	1	2	0	2	1	0
Isoquinolir	Alkaloids c	Alkaloids	Biosynthes	Isoquinolir	NA	1	1	1.4	1	1	1	1	1	2	1	1	1
NA	NA	NA	NA	NA	NA	0	1	0	0	0	0	0	3	1	3	0	2
NA	NA	NA	NA	NA	NA	1	1	1.8	1	1	3	2	1	1	2	1	0
NA	NA	NA	NA	NA	NA	1	1	1.4	1	1	2	2	1	1	1	1	1
NA	NA	NA	NA	NA	NA	1	1	1.1	1	2	1	1	1	1	1	2	2
						0	0	0	0	4	0	0	2	16	0	0	0
NA	NA	NA	NA	NA	NA	2	1	0.8	1	1	1	2	1	1	1	1	1
NA	NA	NA	NA	NA	NA	2	2	0.2	1	0	0	0	1	2	13	0	13
NA	NA	NA	NA	NA	NA	0	2	2.4	0	1	11	1	0	1	2	1	2
NA	NA	NA	NA	NA	NA	1	1	1.3	1	1	1	1	1	3	1	1	81
NA	NA	NA	NA	NA	NA	1	1	1.6	1	1	1	1	1	1	1	2	3
NA	NA	NA	NA	NA	NA	1	1	1.6	0	1	1	0	1	1	3	1	16
NA	NA	NA	NA	NA	NA	2	1	1.3	1	2	1	2	5	1	1	1	##
NA	NA	NA	NA	NA	NA	1	1	1.2	1	2	2	2	1	1	0	1	0
Benzoic ac	Benzene a	Benzene a	Xenobiotic	Aminoben	NA	1	1	3.5	2	1	1	1	1	1	1	2	5
Diphenylm	Benzene a	Benzene a	NA	NA	NA	16	0	0.3	0	0	3	0	0	0	0	0	26
NA	Morphinar	Alkaloids a	NA	NA	NA	1	1	0.9	1	1	1	1	1	1	1	1	1
Amino aci	Carboxylic	Amino aci	NA	NA	NA	2	1	0.7	2	0	1	0	1	0	1	1	1
NA	NA	NA	NA	NA	NA	1	3	1.3	1	1	1	2	1	1	1	1	1
NA	NA	NA	NA	NA	NA	1	1	1.7	1	1	9	1	1	1	1	1	1
Benzoic ac	Benzene a	Benzene a	NA	NA	NA	0	2	1.8	1	1	1	1	10	3	3	1	1
NA	NA	NA	NA	NA	NA	1	1	1	1	1	1	1	1	1	1	1	1
NA	NA	NA	NA	NA	NA	1	1	0.8	2	1	3	0	0	0	1	1	1
NA	NA	NA	NA	NA	NA	1	1	1.1	1	2	23	2	1	10	1	5	1
NA	NA	NA	NA	NA	NA	1	1	1.3	1	1	2	1	1	1	2	1	1
NA	NA	NA	NA	NA	NA	3	1	1.3	1	1	1	1	3	1	1	1	1
NA	NA	NA	NA	NA	NA	1	4	0.3	0	2	9	1	0	0	1	1	2
NA	NA	NA	NA	NA	NA	1	2	0.7	1	1	1	1	1	2	1	1	1
NA	NA	NA	NA	NA	NA	1	1	2.2	1	2	1	2	1	1	1	2	1
Amino aci	Carboxylic	Amino aci	NA	NA	NA	2	1	1.5	1	2	1	2	1	1	1	1	1
Hydroxycc	Coumarins	Coumarins	NA	NA	NA	1	1	1.4	3	1	1	1	1	1	3	1	0
NA	NA	NA	NA	NA	NA	1	1	1.5	1	1	1	2	1	1	2	2	1
Carbohydr	Organoox	Carbohydr	NA	NA	NA	1	1	0.8	1	0	1	0	1	6	1	1	1
NA	NA	NA	NA	NA	NA	1	1	1.7	1	2	2	2	1	1	1	2	1
						0	0	0	0	##	0	0	47	17	51	0	58
NA	Tropane a	Alkaloids a	NA	NA	NA	1	1	0.5	1	1	0	0	0	4	2	0	3
NA	NA	NA	NA	NA	NA	1	1	1	0	1	1	1	1	7	1	1	5
						0	0	0	0	0	0	0	0	7	0	0	0
NA	NA	NA	NA	NA	NA	1	3	1.3	3	0	1	0	1	0	2	0	0
NA	NA	NA	NA	NA	NA	1	1	1	1	0	1	0	1	5	1	2	1
NA	NA	NA	NA	NA	NA	1	1	1.9	1	2	2	2	1	1	1	1	1
NA	NA	NA	NA	NA	NA	1	1	1.3	60	10	4	23	1	1	8	##	1
NA	NA	NA	NA	NA	NA	1	1	2.7	1	2	5	2	1	1	1	1	1
NA	NA	NA	NA	NA	NA	1	3	0.6	1	1	1	1	0	2	1	1	3
NA	NA	NA	NA	NA	NA	1	2	0.8	1	1	3	1	2	1	1	1	1
NA	NA	NA	NA	NA	NA	3	1	0.7	1	1	1	1	1	4	1	2	1
NA	NA	NA	NA	NA	NA	1	4	0.1	1	0	0	0	3	3	0	0	3
Amino aci	Carboxylic	Amino aci	Metabolisr	One carbc	map0067	1	5	1	1	1	8	1	0	1	1	1	4
						0	0	0	31	##	8	79	42	27	89	0	5
Carbonyl c	Organoox	Organic o	NA	NA	NA	1	1	0.8	1	8	1	0	2	1	1	2	1
Arylsulfate	Organic su	Organic ac	NA	NA	NA	1	1	1	1	2	1	4	2	1	1	2	0
NA	NA	NA	NA	NA	NA	1	10	0.4	0	1	1	2	1	7	1	0	3
NA	NA	NA	NA	NA	NA	2	1	0.7	1	1	1	0	1	1	1	1	1
NA	NA	NA	NA	NA	NA	##	1	50	1	1	1	1	1	1	1	1	1
Medium-c	Hydroxy a	Organic ac	NA	NA	NA	1	1	0.9	1	1	1	1	1	1	1	1	1
Isoquinolir	Alkaloids c	Alkaloids	Biosynthes	Isoquinolir	NA	1	1	0.8	1	0	2	0	1	3	1	1	1
NA	NA	NA	Xenobiotic	Nitrotolue	NA	1	1	1.8	1	2	1	2	1	1	1	2	1

NA	NA	NA	NA	NA	NA	1	1	0.2	1	1	0	0	4	2	2	0	33
						0	0	0	0	0	0	0	0	0	0	0	0
						0	0	0	0	0	0	0	0	0	0	0	0
Amino aci	Carboxylic	Amino aci	Amino aci	Phenylalar	map00360	0	2	0.1	3	1	1	1	1	1	2	1	1
NA	NA	NA	NA	NA	NA	2	2	1.5	1	1	3	1	5	1	1	1	1
NA	NA	Alkaloids a	NA	NA	NA	1	1	2.5	1	3	1	4	2	1	1	5	0
NA	NA	NA	NA	NA	NA	2	1	0.4	3	0	2	1	2	0	0	1	1
NA	NA	NA	NA	NA	NA	1	3	0.1	0	0	0	0	7	1	0	1	4
NA	NA	NA	NA	NA	NA	0	11	0.8	1	1	1	2	0	10	0	1	0
Linear diar	Diarylhept	Polyketide	NA	NA	NA	1	1	0.4	1	1	44	1	1	1	1	0	2
Quinoline	Quinolines	Organohe	NA	NA	NA	1	1	1	1	1	1	1	1	1	1	1	1
NA	NA	NA	NA	NA	NA	1	1	0.8	0	2	3	1	1	3	1	0	3
NA	NA	NA	NA	NA	NA	0	3	0.1	1	4	1	0	1	4	1	0	1
NA	NA	NA	NA	NA	NA	1	1	0.7	1	1	1	1	3	1	1	1	7
NA	NA	NA	NA	NA	NA	19	0	0.8	22	0	4	4	0	0	1	4	1
Biphenyls	Benzene a	Benzene a	NA	NA	NA	1	2	3.6	3	0	4	0	2	0	3	5	0
NA	NA	NA	NA	NA	NA	1	2	1.1	1	1	1	1	1	1	1	1	1
NA	NA	NA	NA	NA	NA	1	1	1.3	1	1	1	1	1	1	1	1	6
NA	NA	NA	Metabolis	Brassinost	NA	8	2	1.1	1	1	1	5	1	0	1	1	2
NA	NA	NA	NA	NA	NA	0	2	0.3	1	1	4	3	2	0	1	2	1
NA	NA	NA	NA	NA	NA	1	1	1.9	1	2	1	2	4	1	3	1	0
NA	NA	NA	NA	NA	NA	1	1	1.9	1	2	1	2	1	1	1	2	1
NA	NA	NA	NA	NA	NA	1	1	1	1	1	1	1	1	1	1	1	1
NA	NA	NA	NA	NA	NA	1	2	1.1	1	1	0	3	1	0	1	1	2
NA	NA	NA	NA	NA	NA	1	1	2.3	1	3	6	18	1	1	1	8	1
NA	NA	NA	NA	NA	NA	2	1	0.6	0	1	7	1	15	1	1	0	0
NA	NA	NA	NA	NA	NA	2	2	2	1	1	1	0	1	0	3	1	0
NA	NA	NA	NA	NA	NA	1	1	2.2	1	2	1	2	1	1	1	2	1
NA	NA	NA	NA	NA	NA	0	1	0.5	1	2	6	1	1	3	1	1	3
NA	NA	NA	NA	NA	NA	1	1	0.5	1	2	1	0	1	10	1	3	2
NA	NA	NA	NA	NA	NA	1	2	0.5	1	1	1	0	2	2	1	1	2
NA	NA	NA	NA	NA	NA	2	1	0.6	1	0	0	1	2	1	1	1	0
						0	0	0	0	0	0	0	##	0	0	0	0
NA	NA	NA	NA	NA	NA	1	1	0.9	1	2	0	1	1	2	1	1	1
NA	NA	NA	NA	NA	NA	1	1	1	1	1	1	1	1	1	1	1	1
Hydroxycii	Cinnamic a	Polyketide	NA	NA	NA	9	1	0.9	1	1	2	1	1	1	1	1	1
NA	NA	NA	NA	NA	NA	1	1	1.3	1	2	1	1	1	1	1	2	1
NA	NA	NA	NA	NA	NA	1	1	0.8	1	0	1	0	1	4	1	2	0
NA	NA	NA	NA	NA	NA	1	1	0.7	1	0	1	1	1	5	1	2	1
NA	NA	NA	NA	NA	NA	1	1	1.3	1	1	1	1	2	1	1	2	1
NA	NA	NA	NA	NA	NA	1	1	1	1	1	1	1	1	1	1	1	1
NA	NA	NA	NA	NA	NA	0	1	0.1	1	2	0	1	5	7	1	0	14
NA	NA	NA	NA	NA	NA	1	1	1.8	3	0	1	0	2	0	2	1	0
NA	NA	NA	NA	NA	NA	1	3	0.3	1	0	0	1	7	1	1	0	12
NA	NA	NA	NA	NA	NA	2	1	0.3	1	0	0	0	2	1	9	0	0
NA	NA	NA	NA	NA	NA	1	3	1.6	1	1	1	2	1	1	1	1	9
NA	NA	NA	NA	NA	NA	1	6	6	1	1	13	1	1	1	1	3	1
NA	NA	NA	NA	NA	NA	1	2	1.1	1	2	1	2	5	1	1	1	0
NA	NA	NA	NA	NA	NA	1	3	0.5	0	1	0	2	1	3	4	1	1
NA	NA	NA	NA	NA	NA	1	3	0.1	1	0	3	0	2	2	0	0	1
NA	NA	NA	NA	NA	NA	1	2	0.8	1	1	1	1	1	1	1	1	2
Carbohydr	Organoox	Carbohydr	NA	NA	NA	1	1	0.4	1	1	3	1	1	1	1	1	5
NA	NA	NA	NA	NA	NA	1	3	0.6	1	1	1	0	3	1	2	1	13
NA	NA	NA	NA	NA	NA	2	1	2.1	1	3	2	2	1	3	1	1	1
NA	NA	NA	NA	NA	NA	1	1	2.1	1	3	1	3	1	1	2	3	0
NA	Benzopyra	Organohe	NA	NA	NA	1	1	1.4	1	1	1	2	1	1	1	1	1
Betaxanthi	Betalains	Amino aci	NA	NA	NA	1	1	0.9	1	2	1	0	1	1	1	1	1
NA	NA	NA	NA	NA	NA	1	3	0.9	2	0	1	0	1	0	1	1	1

NA	Sulfoxides	Organosul	NA	NA	NA	1	0	0.9	1	##	1	51	5	41	1	1	0
NA	NA	NA	NA	NA	NA	1	1	1.2	1	1	1	0	1	1	1	1	1
NA	NA	NA	NA	NA	NA	1	1	1.7	1	1	1	2	8	3	1	2	1
NA	Thienopyr	Pyridine ar	NA	NA	NA	1	1	1.4	1	1	1	2	1	1	1	1	2
NA	NA	NA	NA	NA	NA	6	1	0.7	1	1	5	1	1	1	1	1	3
NA	NA	NA	NA	NA	NA	1	1	1.1	1	2	1	2	1	1	1	1	1
NA	NA	NA	NA	NA	NA	1	1	1.9	1	2	1	2	1	1	1	2	1
Methoxyp	Phenols	Phenols ar	Biosynthes	Biosynthes	map0110(	1	1	1.2	1	2	1	1	1	1	1	2	1
NA	NA	NA	NA	NA	NA	3	3	0.2	1	##	0	##	1	1	0	0	1
NA	NA	NA	NA	NA	NA	1	1	1.4	1	2	1	1	1	1	1	2	1
NA	NA	NA	NA	NA	NA	2	1	2.7	0	1	1	1	1	0	4	1	1
						24	0	0	0	0	0	0	0	8	0	0	0
NA	NA	NA	Biosynthes	Aflatoxin b	map0110(	1	1	1	1	1	2	2	1	1	1	1	2
Flavones	Flavonoids	Flavonoids	NA	NA	NA	1	1	1.3	1	1	1	2	1	1	1	1	2
NA	NA	NA	NA	NA	NA	5	1	1.4	1	1	2	1	1	1	1	1	1
NA	NA	NA	NA	NA	NA	1	1	2.1	5	0	2	0	1	0	4	5	0
NA	NA	NA	NA	NA	NA	1	2	1.1	1	2	3	2	1	1	1	2	0
Carbohydr	Organoox	Carbohydr	NA	NA	NA	7	0	3.5	17	6	0	1	1	2	2	1	7
NA	NA	NA	NA	NA	NA	0	1	2.8	1	2	1	2	2	0	0	2	1
NA	NA	NA	NA	NA	NA	1	1	0.2	0	2	24	1	1	6	0	1	18
NA	NA	NA	NA	NA	NA	1	1	2	1	2	1	2	1	1	1	1	1
NA	NA	NA	NA	NA	NA	1	1	0.9	1	2	1	1	3	1	1	1	2
NA	NA	NA	NA	NA	NA	2	1	0.2	1	7	0	3	1	4	2	0	1
NA	NA	NA	NA	NA	NA	1	1	0.8	2	1	1	1	1	1	1	1	1
Pyrimidine	Pyrimidine	Nucleic ac	Nucleotide	Pyrimidine	map0024(	1	2	0.9	0	1	4	3	1	2	1	1	4
NA	NA	NA	NA	NA	NA	1	1	1.4	1	1	1	1	1	1	1	1	1
NA	NA	NA	NA	NA	NA	2	1	0.6	5	0	1	0	2	0	2	1	1
NA	NA	NA	NA	NA	NA	1	1	1.9	1	2	1	2	1	1	1	2	1
NA	NA	NA	NA	NA	NA	6	1	0.8	8	0	0	0	0	0	11	0	0
NA	NA	NA	NA	NA	NA	3	1	0	##	10	##	2	0	5	0	3	0
NA	NA	NA	NA	NA	NA	1	1	1	1	2	1	2	1	8	1	2	1
NA	NA	NA	NA	NA	NA	1	1	1.9	1	2	1	2	1	1	1	2	1
NA	NA	NA	NA	NA	NA	1	0	0.9	0	0	0	0	13	1	8	1	3
NA	NA	NA	NA	NA	NA	1	1	1	1	1	1	1	1	1	1	1	1
NA	NA	NA	NA	NA	NA	1	4	0.1	2	0	0	0	1	3	1	0	3
Diphenyle	Benzene a	Benzene a	NA	NA	NA	1	11	0.8	1	1	1	15	1	1	1	0	1
Fatty acids	Fatty Acyls	Fatty acyls	NA	NA	NA	1	1	0.5	1	4	0	0	2	4	3	0	2
						0	0	0	0	9	0	0	0	0	0	0	0
						0	0	0	0	0	0	0	0	0	0	0	0
NA	NA	NA	NA	NA	NA	1	1	0.9	3	5	1	2	0	1	2	2	4
NA	NA	NA	NA	NA	NA	2	1	0.9	1	1	1	1	2	1	1	1	6
Hydroxycc	Coumarins	Coumarins	Biosynthes	Phenylpro	NA	0	2	1.9	1	1	4	3	1	1	1	3	0
NA	NA	NA	NA	NA	NA	1	1	2	1	2	1	2	1	1	1	2	1
NA	NA	NA	NA	NA	NA	1	1	0.5	1	1	0	1	1	2	1	1	1
Fatty acids	Fatty Acyls	Fatty acyls	NA	NA	NA	1	1	2.2	1	8	2	2	1	1	1	2	1
NA	Morphinar	Alkaloids a	NA	NA	NA	2	1	0.6	5	1	2	1	2	2	0	2	1
NA	NA	NA	NA	NA	NA	1	1	2.5	1	2	1	2	1	1	1	2	1
Delta valer	Lactones	Organohe	NA	NA	NA	1	1	2.2	1	2	1	2	1	1	1	2	1
NA	NA	NA	NA	NA	NA	4	0	0.4	18	0	2	0	16	0	3	3	1
Diradylgly	Glycerolipi	Glycerolipi	NA	NA	NA	3	1	0.1	31	22	24	2	0	1	2	9	0
Flavonoid	Flavonoids	Flavonoids	NA	NA	NA	1	1	0.7	1	0	1	1	1	3	1	1	1
NA	NA	NA	NA	NA	NA	7	2	2	13	0	0	0	15	0	5	4	1
NA	NA	NA	NA	NA	NA	1	1	1.1	1	1	1	1	1	1	1	1	1
NA	NA	Alkaloids a	Chemical s	Biosynthes	NA	0	0	1.6	1	1	2	2	1	4	1	1	0
NA	NA	NA	NA	NA	NA	1	1	0.8	1	0	1	0	1	2	1	2	1
NA	NA	NA	NA	NA	NA	1	1	1.7	1	2	2	2	1	1	1	1	1
NA	NA	NA	NA	NA	NA	1	1	1.4	3	2	20	2	1	1	1	1	1
NA	NA	NA	NA	NA	NA	1	1	2.5	2	4	1	0	2	2	4	4	0

NA	NA	NA	NA	NA	NA	2	1	0.5	5	1	0	0	1	0	2	0	1
NA	NA	NA	NA	NA	NA	1	1	1.4	1	2	1	1	1	1	1	2	1
NA	NA	NA	NA	NA	NA	1	1	1.8	1	2	1	3	1	1	1	1	0
Imidazoles	Azoles	Imidazoles	NA	NA	NA	1	0	2.2	1	2	1	1	1	0	2	1	1
NA	NA	NA	NA	NA	NA	1	1	1.3	1	2	1	1	1	1	1	2	1
Halobenze	Benzene a	Benzene a	NA	NA	NA	2	1	2.1	1	2	1	2	6	1	3	7	1
NA	NA	NA	NA	NA	NA	1	1	1.9	1	2	1	2	1	1	1	2	1
NA	NA	NA	NA	NA	NA	0	0	4.3	1	0	0	0	3	0	36	0	0
NA	NA	NA	NA	NA	NA	1	1	1.8	1	1	1	2	1	1	1	2	1
Amino aci	Carboxylic	Amino aci	NA	NA	NA	1	1	1.6	1	2	1	1	0	1	2	1	1
NA	NA	NA	NA	NA	NA	0	2	18	1	4	37	4	1	1	0	15	1
NA	NA	NA	NA	NA	NA	1	1	1.3	1	2	2	1	1	1	1	1	1
NA	NA	NA	NA	NA	NA	1	1	0.8	1	2	2	2	1	2	1	2	2
NA	NA	NA	NA	NA	NA	1	1	2.3	2	1	6	1	1	1	1	1	1
NA	NA	NA	NA	NA	NA	69	1	12	3	1	1	1	1	1	1	1	2
NA	NA	NA	NA	NA	NA	0	1	1.7	1	2	5	2	1	1	2	3	0
NA	NA	NA	NA	NA	NA	1	1	1.2	7	2	1	3	1	1	1	1	1
						0	0	0	0	0	0	0	0	5	16	0	34
NA	NA	NA	NA	NA	NA	1	1	1.6	1	2	1	2	1	1	1	2	1
NA	NA	NA	NA	NA	NA	1	2	2.2	1	5	8	1	2	0	0	2	1
						0	0	0	0	0	0	0	0	0	0	0	0
						0	0	0	0	0	0	0	0	0	0	0	0
						0	0	0	0	0	0	0	0	0	0	0	0
						0	0	0	0	0	0	0	0	0	0	0	0
						0	0	0	0	0	0	0	0	0	0	0	0
						0	0	0	0	0	0	0	0	0	0	0	0
						0	0	0	0	0	0	0	0	0	0	0	0
NA	NA	NA	NA	NA	NA	1	1	0.7	1	1	0	1	1	1	1	1	1
NA	NA	NA	NA	NA	NA	2	0	0.5	4	0	2	0	3	0	3	3	0
NA	NA	NA	NA	NA	NA	1	0	0.6	1	0	1	0	1	1	0	1	1
NA	NA	NA	NA	NA	NA	2	1	0.8	1	1	1	0	1	3	1	1	1
NA	NA	NA	NA	NA	NA	1	1	1.2	1	1	1	1	4	1	2	1	12
NA	NA	NA	NA	NA	NA	1	1	0.8	1	0	1	0	1	3	1	1	76
NA	NA	NA	NA	NA	NA	1	3	0.1	1	0	0	0	1	1	1	0	40
NA	NA	NA	NA	NA	NA	2	1	0.5	1	1	4	1	1	1	1	1	1
NA	NA	NA	NA	NA	NA	2	1	0.3	4	0	0	0	1	1	3	0	1
NA	NA	NA	NA	NA	NA	1	2	1.1	1	1	1	1	1	1	1	2	1
NA	NA	NA	NA	NA	NA	2	1	0.5	1	1	2	1	1	1	1	1	6
Amino aci	Carboxylic	Amino aci	NA	NA	NA	1	1	0.9	1	1	1	1	1	1	1	1	1
NA	NA	NA	NA	NA	NA	1	1	0.5	4	0	0	0	0	1	3	1	1
NA	NA	NA	NA	NA	NA	1	1	1.2	1	2	1	1	1	1	1	2	1
Arylsulfate	Organic su	Organic ac	NA	NA	NA	2	2	6.6	5	6	0	4	1	0	1	2	1
Phenoxyac	Benzene a	Benzene a	NA	NA	NA	1	2	3.2	3	0	7	0	2	0	2	11	0
NA	NA	NA	NA	NA	NA	1	1	0.6	2	0	1	0	2	0	2	1	0
NA	NA	NA	NA	NA	NA	1	1	1.3	1	2	2	2	1	1	1	1	1
NA	NA	NA	NA	NA	NA	1	1	1.1	1	1	1	1	1	1	1	1	10
Spirostan (	27-Carbor	Sterol lipic	Chemical s	Biosynthes	NA	0	5	0.2	1	0	3	8	8	1	0	1	1
NA	NA	NA	NA	NA	NA	2	3	1.4	1	3	1	5	1	2	1	2	1
NA	NA	NA	NA	NA	NA	1	1	1.3	1	2	1	1	1	1	1	2	1
NA	NA	NA	NA	NA	NA	1	1	6.6	1	3	2	3	2	1	4	2	0
						0	0	0	0	11	0	0	0	0	0	0	0
NA	NA	NA	NA	NA	NA	1	2	0.2	1	0	2	2	3	1	1	1	1
NA	NA	NA	NA	NA	NA	1	0	1	2	0	0	0	4	4	3	0	49
NA	NA	NA	NA	NA	NA	1	1	1	1	2	16	1	4	2	1	1	1
NA	NA	NA	NA	NA	NA	46	3	1.9	0	1	1	1	0	1	4	1	1
NA	NA	NA	NA	NA	NA	0	1	0.2	6	0	0	1	71	0	6	5	4
Dibenzoxa	Benzoxaze	Organohe	NA	NA	NA	1	1	1.4	1	1	2	1	1	1	1	1	1
Alloxazine	Pteridines	Pteridines	Metabolis	Riboflavin	map00740	4	1	0.2	1	0	1	0	2	0	1	0	1

Ethers	Organoox	Organic o	NA	NA	NA	1	1	93	2	1	71	1	1	1	1	1	1
NA	NA	NA	NA	NA	NA	1	1	1.2	1	2	1	1	1	1	1	2	1
NA	NA	NA	NA	NA	NA	1	2	0.1	0	1	1	1	4	6	0	0	18
NA	NA	NA	NA	NA	NA	1	1	1.4	1	1	1	2	1	1	1	1	0
NA	NA	NA	NA	NA	NA	1	1	1.3	1	1	1	1	4	1	1	1	22
NA	NA	NA	NA	NA	NA	0	2	0.6	2	1	1	2	3	0	0	0	5
Ureas	Organic ca	Organic ac	NA	NA	NA	1	1	1.4	1	2	2	2	1	1	1	1	1
NA	NA	NA	NA	NA	NA	1	2	1.1	1	1	3	1	25	1	1	2	1
NA	NA	NA	NA	NA	NA	2	3	2.4	2	3	1	3	2	0	1	1	1
NA	NA	NA	NA	NA	NA	4	1	1.3	1	1	1	3	1	1	1	2	1
NA	NA	NA	NA	NA	NA	1	1	1.8	1	1	1	0	1	0	2	1	0
Amino aci	Carboxylic	Amino aci	NA	NA	NA	1	1	1.1	1	1	1	2	1	1	1	1	1
NA	NA	NA	NA	NA	NA	6	1	1.3	1	1	2	1	1	1	1	1	1
Sesquiterp	Prenol lipi	Terpenoid	Lipid meta	Steroid bi	map00100	1	1	1.5	1	2	1	2	1	1	1	2	1
NA	NA	NA	NA	NA	NA	0	3	0.4	1	2	0	2	1	0	1	1	0
NA	NA	NA	NA	NA	NA	3	1	1.9	3	1	1	0	1	1	4	0	1
Amino aci	Carboxylic	Amino aci	NA	NA	NA	15	0	2.6	1	1	1	1	1	1	0	1	7
Pyrroloind	Indoles an	Indole anc	NA	NA	NA	1	1	0.8	1	0	1	0	1	0	1	1	3
NA	NA	NA	NA	NA	NA	0	4	1.1	0	1	30	1	3	1	1	2	1
NA	NA	NA	NA	NA	NA	1	3	13	10	12	1	3	1	1	1	1	1
NA	NA	NA	NA	NA	NA	2	1	0.4	3	1	0	0	2	1	2	0	1
NA	NA	NA	NA	NA	NA	1	1	1	1	2	1	2	1	1	1	1	2
NA	NA	NA	NA	NA	NA	1	1	0.8	1	0	1	1	1	1	1	1	1
						0	0	0	0	0	0	0	0	0	24	0	0
NA	NA	NA	NA	NA	NA	3	3	1.2	2	1	1	0	2	1	1	2	1
NA	NA	NA	NA	NA	NA	1	1	0.9	1	1	1	1	1	1	1	1	1
Carbohydr	Organoox	Carbohydr	Global anc	Biosynthes	NA	1	1	1.8	1	1	1	2	1	1	1	2	1
NA	NA	NA	NA	NA	NA	1	1	1.1	3	8	1	1	1	13	8	2	1
NA	NA	NA	NA	NA	NA	0	4	0.1	0	1	0	1	2	4	1	0	19
NA	NA	NA	NA	NA	NA	1	1	1.3	1	1	1	2	1	1	1	1	1
NA	NA	NA	NA	NA	NA	1	3	0.7	1	1	1	1	1	1	1	1	1
						0	0	0	0	0	0	0	0	0	0	0	0
Androstan	Steroids ar	Steroids ar	NA	NA	NA	0	2	0.9	1	0	2	1	0	1	1	0	1
NA	NA	NA	NA	NA	NA	1	2	2.1	1	1	2	1	1	1	1	1	1
NA	NA	NA	NA	NA	NA	2	1	0.7	1	1	1	0	1	0	1	1	1
NA	NA	NA	NA	NA	NA	1	1	2.8	1	2	1	2	1	1	1	1	1
						0	0	0	0	2	0	0	0	0	0	0	0
Oxosteroid	Steroids ar	Steroids ar	NA	NA	NA	1	1	1	2	1	1	1	1	3	1	1	1
NA	NA	NA	NA	NA	NA	1	1	0.8	1	0	1	1	1	5	1	2	1
NA	NA	NA	NA	NA	NA	1	1	0.8	2	1	1	1	1	1	1	1	1
NA	NA	NA	NA	NA	NA	1	1	1.7	1	2	1	2	1	1	1	2	1
						0	0	0	0	0	0	0	0	0	0	0	0
NA	NA	NA	NA	NA	NA	1	1	0.6	2	1	1	0	1	0	3	1	0
Glyceroph	Glyceroph	Glyceroph	NA	NA	NA	0	1	0	1	1	0	0	3	3	3	0	5
NA	NA	NA	NA	NA	NA	1	0	1.3	1	6	0	2	8	1	11	6	9
NA	NA	NA	NA	NA	NA	2	1	0.4	2	0	1	0	1	1	3	1	1
NA	NA	NA	NA	NA	NA	1	1	0.9	1	0	1	0	1	2	1	2	1
NA	NA	NA	NA	NA	NA	1	1	1.3	1	2	1	1	1	1	1	2	2
NA	Protoberb	Alkaloids a	Biosynthes	Isoquinolir	map0415	1	1	17	23	2	0	1	1	1	2	1	2
Vitamin D	Steroids ar	Vitamins a	Lipid meta	Steroid bi	map00100	1	1	0.5	2	0	1	0	2	1	3	1	1
Amino aci	Carboxylic	Amino aci	Amino aci	Valine, leu	map00280	5	1	1	1	1	9	3	1	1	0	2	2
						0	0	0	0	0	0	13	58	0	41	0	##
NA	NA	NA	NA	NA	NA	##	1	2.1	1	1	0	1	1	2	0	1	5
NA	NA	NA	NA	NA	NA	1	1	2.2	1	2	1	2	1	1	1	2	0
NA	NA	NA	NA	NA	NA	1	1	0.8	1	1	1	1	1	1	28	1	2
						0	0	0	0	0	0	0	0	0	0	0	0
NA	NA	NA	NA	NA	NA	1	1	1.3	1	2	1	1	1	1	1	2	1
NA	NA	NA	Xenobiotic	Nitrotolue	NA	0	9	1.3	1	##	1	2	1	3	0	1	3

NA	NA	NA	Metabolis	Porphyrin	map0086	1	2	0.2	0	0	0	0	2	0	2	0	0
Triterpeno	Prenol lipi	Terpenoid	NA	NA	NA	8	1	2.8	1	1	14	1	1	1	1	1	1
NA	NA	NA	NA	NA	NA	1	1	1.2	1	2	1	1	1	1	1	1	1
NA	NA	NA	NA	NA	NA	6	1	1.4	1	1	1	1	1	1	1	1	1
NA	NA	NA	NA	NA	NA	3	1	0.6	3	1	1	0	1	1	2	0	0
NA	NA	NA	NA	NA	NA	1	2	1.4	1	1	1	1	27	2	1	2	1
						0	0	0	0	0	0	0	0	0	0	0	0
NA	NA	NA	NA	NA	NA	2	1	2.8	1	2	1	2	4	1	1	1	1
NA	NA	NA	NA	NA	NA	2	0	0.4	5	0	0	0	1	1	2	0	1
NA	NA	NA	NA	NA	NA	1	1	0.9	1	1	1	1	1	2	2	1	1
NA	NA	NA	NA	NA	NA	1	1	2.7	2	3	1	3	1	2	0	1	0
NA	NA	NA	NA	NA	NA	1	1	1	1	1	1	1	1	2	1	5	3
NA	NA	NA	NA	NA	NA	1	0	0.7	0	1	0	1	3	3	1	1	55
NA	NA	NA	NA	NA	NA	1	5	1.2	1	2	1	2	2	1	1	1	1
NA	NA	NA	NA	NA	NA	1	1	1.1	0	2	18	1	1	1	1	3	1
NA	NA	NA	NA	NA	NA	0	2	0.1	1	1	0	0	2	2	4	0	5
Monorady	Glycerolipi	Glycerolipi	NA	NA	NA	0	1	6.2	1	2	##	2	1	1	1	1	3
NA	NA	NA	NA	NA	NA	1	2	18	1	1	1	1	7	1	1	1	2
NA	NA	NA	NA	NA	NA	1	1	1	1	0	1	0	2	1	1	1	0
NA	NA	NA	NA	NA	NA	2	0	0.2	3	0	0	0	2	1	2	0	2
NA	NA	NA	NA	NA	NA	1	1	2	1	2	1	2	1	1	1	2	1
NA	NA	NA	NA	NA	NA	1	1	0.3	0	2	23	1	1	7	0	1	14
NA	NA	NA	NA	NA	NA	1	9	0.9	1	1	2	0	2	0	1	1	1
NA	NA	NA	NA	NA	NA	1	1	0.8	2	1	1	2	0	3	0	0	1
Carbohydr	Organoox	Carbohydr	NA	NA	NA	1	1	4.2	1	1	2	1	0	2	1	1	1
NA	NA	NA	NA	NA	NA	1	1	0.6	1	0	1	0	1	6	8	1	0
Others	Sesquiterp	Terpenoid	NA	NA	NA	1	2	0.4	1	1	1	0	1	2	1	1	3
NA	NA	NA	NA	NA	NA	1	1	1.8	1	1	16	1	1	1	1	1	1
NA	NA	NA	NA	NA	NA	0	2	0.1	0	2	0	1	2	1	2	0	4
NA	NA	NA	NA	NA	NA	7	2	1.1	1	1	12	1	1	1	1	2	1
NA	NA	NA	NA	NA	NA	0	2	0.1	3	1	1	1	1	1	2	2	1
NA	NA	NA	NA	NA	NA	1	1	1.4	1	1	2	2	1	1	1	1	1
NA	NA	NA	NA	NA	NA	1	1	1	1	2	1	2	1	4	1	1	2
NA	NA	NA	NA	NA	NA	1	1	1.1	1	1	1	1	1	1	1	1	1
NA	NA	NA	NA	NA	NA	1	1	0.8	1	1	2	1	1	1	1	1	1
NA	Stilbenes	Polyketide	NA	NA	NA	1	1	2	1	2	1	2	1	1	1	2	1
NA	NA	NA	NA	NA	NA	1	2	0.8	1	1	1	2	1	1	1	2	1
NA	NA	NA	NA	NA	NA	3	1	0.4	3	0	1	0	2	2	2	1	1
NA	Pyridines a	Pyridine ar	NA	NA	NA	1	1	1	1	1	1	1	1	1	1	1	1
NA	NA	NA	NA	NA	NA	1	1	1.3	1	2	1	1	1	1	1	2	1
NA	NA	NA	NA	NA	NA	1	1	0.8	1	1	1	1	1	4	1	2	1
NA	NA	NA	NA	NA	NA	1	2	3.7	1	2	35	2	1	1	0	11	0
NA	NA	NA	NA	NA	NA	2	1	1.3	1	2	1	2	5	1	1	1	1
NA	NA	NA	NA	NA	NA	1	1	2.4	2	1	1	1	0	1	13	1	1
NA	NA	NA	NA	NA	NA	1	6	0.7	1	1	2	1	6	1	1	1	1
NA	NA	NA	NA	NA	NA	1	1	1.5	1	2	2	2	1	1	1	1	1
						0	0	0	0	0	0	0	0	0	0	0	0
NA	NA	NA	NA	NA	NA	1	1	1.4	1	1	1	2	1	3	1	1	1
Carbohydr	Organoox	Carbohydr	NA	NA	NA	48	1	1.9	1	2	1	2	1	1	1	2	1
Chalcones	Linear 1,3-	Polyketide	Biosynthes	Flavonoid	NA	1	1	0.6	1	0	1	1	1	12	1	1	0
NA	NA	NA	NA	NA	NA	1	1	2.5	1	2	1	2	1	1	1	2	0
NA	NA	NA	NA	NA	NA	1	1	0.9	1	1	1	1	1	1	1	1	1
NA	NA	NA	Metabolis	Biosynthes	map0110	1	1	0.3	2	0	1	0	2	1	3	1	6
NA	NA	NA	NA	NA	NA	1	1	1.8	1	2	1	2	10	1	1	2	15
NA	NA	NA	NA	NA	NA	1	1	0.6	1	1	1	0	1	0	1	1	3
NA	NA	NA	NA	NA	NA	9	1	1.1	6	1	0	1	19	0	8	8	0
NA	NA	NA	NA	NA	NA	1	20	0.7	8	1	1	33	2	1	2	1	1
Amino aci	Carboxylic	Amino aci	NA	NA	NA	23	1	2.6	1	1	15	1	1	1	0	1	1

Fatty acid	Fatty Acyls	Fatty acyls	NA	NA	map04931	1	0	0.2	1	15	0	1	0	12	1	0	9
Imidazoles	Azoles	Imidazoles	NA	NA	NA	2	1	0.9	4	0	1	0	1	0	2	0	0
NA	NA	NA	NA	NA	NA	1	1	0.7	2	0	1	1	2	1	1	1	1
Anthocyar	Flavonoids	Flavonoids	Biosynthes	Anthocyar	NA	1	1	0.8	1	0	1	0	1	6	1	1	1
NA	NA	NA	NA	NA	NA	3	0	0.3	3	0	0	0	2	0	6	0	0
NA	NA	NA	NA	NA	NA	1	1	0.9	1	1	11	1	6	2	2	8	1
NA	NA	NA	NA	NA	NA	1	3	0.7	1	1	1	1	7	0	2	1	1
NA	NA	NA	NA	NA	NA	1	1	1.4	1	1	2	2	1	1	1	1	1
NA	NA	NA	NA	NA	NA	0	2	0.2	2	0	2	0	3	4	3	1	5
NA	NA	NA	NA	NA	NA	1	1	1	1	1	4	1	2	1	1	1	3
Glycosphir	Sphingolip	Sphingolip	NA	NA	NA	1	1	0.9	2	1	1	1	1	1	0	1	2
Quinolone	Quinolines	Organohe	NA	NA	NA	1	1	1.3	1	1	1	1	1	2	1	1	1
Purines an	Imidazopy	Purines an	NA	NA	NA	1	6	0.2	3	0	2	0	5	0	1	1	3
NA	NA	NA	NA	NA	NA	1	1	6.5	1	1	24	1	1	1	1	1	1
NA	NA	NA	NA	NA	NA	1	1	1.4	1	1	12	1	1	1	2	1	1
NA	NA	NA	NA	NA	NA	1	1	1.2	1	1	1	2	1	1	1	1	1
NA	NA	NA	NA	NA	NA	1	1	0.1	1	0	2	0	2	1	1	0	1
NA	NA	NA	NA	NA	NA	1	1	0.6	1	1	0	1	1	1	1	1	1
Bilirubins	Tetrapyrro	Organohe	NA	NA	NA	1	2	1	1	2	1	2	3	1	1	1	1
NA	NA	NA	NA	NA	NA	1	1	1.2	1	3	2	2	1	2	1	1	4
NA	Pyridines a	Pyridine ar	NA	NA	NA	0	0	0.4	2	0	0	1	1	27	1	1	0
Methoxyb	Benzene a	Benzene a	NA	NA	NA	2	1	1.3	1	2	1	5	2	2	3	1	0
NA	NA	NA	NA	NA	NA	1	1	0.9	1	2	4	1	3	1	1	1	1
Amines	Organonit	Amines	NA	NA	NA	1	1	1.7	1	2	1	2	1	1	1	1	1
NA	NA	NA	NA	NA	NA	2	1	1.4	1	1	1	2	1	1	1	1	1
NA	NA	NA	NA	NA	NA	1	1	1.3	1	1	37	1	8	1	1	2	1
Arylsulfate	Organic su	Organic ac	NA	NA	NA	1	1	0.6	6	11	2	8	##	0	3	6	0
NA	NA	NA	NA	NA	NA	1	1	1.3	1	1	1	2	1	1	1	1	1
Phenothia	Benzothia	Organohe	NA	NA	NA	1	1	0.3	1	0	1	1	8	1	2	1	9
NA	NA	NA	NA	NA	NA	1	1	0.8	3	1	1	1	0	51	2	1	3
NA	NA	NA	NA	NA	NA	31	0	0.5	1	1	1	1	0	0	0	0	27
NA	NA	NA	NA	NA	NA	1	1	2.6	1	1	1	1	1	1	2	7	0
NA	NA	NA	NA	NA	NA	1	1	1.3	1	2	1	1	1	1	1	1	1
						41	49	79	0	##	15	49	15	0	4	18	0
NA	NA	NA	NA	NA	NA	1	1	0.5	3	1	1	1	0	##	0	0	5
Arylsulfate	Organic su	Organic ac	NA	NA	NA	1	1	1.7	1	2	1	3	1	1	1	3	1
						0	0	0	0	0	0	0	3	0	83	0	0
Benzenesu	Benzene a	Benzene a	NA	NA	NA	1	1	0.8	1	0	1	1	1	1	1	2	1
NA	NA	NA	NA	NA	NA	1	1	1.9	1	2	2	2	1	1	1	2	1
NA	NA	NA	NA	NA	NA	2	2	1.3	3	0	1	0	1	0	1	1	0
NA	NA	NA	NA	NA	NA	2	1	1.7	1	2	1	2	1	1	1	1	1
Benzenesu	Benzene a	Benzene a	NA	NA	NA	1	10	1.3	1	1	1	2	1	1	1	1	1
Amino aci	Carboxylic	Amino aci	NA	NA	NA	1	1	4.6	1	2	1	2	1	1	1	1	0
NA	NA	NA	NA	NA	NA	1	1	1.1	1	1	1	1	1	1	1	1	1
NA	NA	NA	NA	NA	NA	1	1	0.9	1	3	2	4	0	1	1	1	3
NA	NA	NA	NA	NA	NA	1	1	2.8	1	2	1	1	1	2	1	1	1
Carbohydr	Organoox	Carbohydr	NA	NA	NA	1	2	1.4	2	13	1	0	1	0	2	1	0
NA	NA	NA	NA	NA	NA	1	5	5.3	0	1	1	1	1	1	1	1	1
Glyceroph	Glyceroph	Glyceroph	NA	NA	NA	0	3	0	1	0	0	0	5	6	4	0	9
						0	0	0	0	40	5	0	38	39	##	0	6
Phosphate	Organic pl	Organic ac	Amino aci	Histidine r	map0034C	1	1	1.2	1	2	1	2	6	1	1	1	1
NA	NA	NA	NA	NA	NA	3	1	0.6	2	1	0	3	2	1	0	10	1
NA	NA	NA	NA	NA	NA	1	3	0.3	1	0	1	0	3	3	1	0	19
Thiazoles	Azoles	Organohe	NA	NA	NA	0	4	0.9	0	1	34	2	7	0	1	10	1
NA	NA	NA	NA	NA	NA	1	1	1.4	1	1	1	2	1	1	1	1	1
NA	NA	NA	NA	NA	NA	1	1	1.4	2	0	1	0	1	0	2	1	0
NA	NA	NA	NA	NA	NA	1	1	0.7	1	3	34	0	1	2	1	1	2
						0	0	0	0	0	0	0	0	0	0	0	0

Benzoic ac	Benzene a	Benzene a	NA	NA	map04976	0	2	1.9	1	1	4	4	1	1	1	3	0
NA	NA	NA	NA	NA	NA	1	1	1	1	2	1	1	3	1	2	1	0
NA	NA	NA	NA	NA	NA	0	79	0.4	1	0	0	1	2	45	1	0	7
NA	NA	NA	NA	NA	NA	1	1	0.9	1	0	1	0	1	2	1	1	1
NA	NA	NA	NA	NA	NA	1	1	0.6	0	1	1	2	1	2	1	1	3
Amino aci	Carboxylic	Amino aci	NA	NA	NA	27	1	1.4	4	1	1	4	0	0	2	2	1
NA	NA	NA	NA	NA	NA	1	2	1	6	2	1	1	1	1	1	11	1
						0	0	0	3	0	0	57	0	0	0	0	0
NA	NA	NA	NA	NA	NA	1	1	0.7	1	1	1	1	2	1	1	1	2
NA	NA	NA	NA	NA	NA	1	1	1.2	1	1	1	1	1	1	1	1	1
NA	NA	NA	NA	NA	NA	1	1	1.4	1	1	3	2	1	1	1	1	0
NA	NA	NA	NA	NA	NA	3	0	2.9	2	1	1	0	4	1	11	1	0
NA	NA	NA	NA	NA	NA	1	1	1.5	1	1	1	2	1	1	1	1	4
NA	NA	NA	NA	NA	NA	1	1	1.5	1	1	1	2	1	1	1	1	1
NA	NA	NA	Energy me	Methane r	map0110(	2	2	0.2	1	10	0	0	0	2	0	0	0
NA	NA	NA	NA	NA	NA	1	1	2.7	1	1	1	2	1	1	1	1	2
NA	NA	NA	NA	NA	NA	1	1	7.3	2	0	10	0	2	0	2	2	0
NA	NA	NA	NA	NA	NA	1	1	1.5	1	1	1	2	3	1	1	1	1
FA0106 O:	FA01 Fatty	Fatty acyls	Biosynthes	Glucosinol	map0121(	1	1	2.2	1	2	1	2	1	1	1	2	1
						0	0	0	0	0	0	0	2	0	0	0	0
NA	NA	NA	NA	NA	NA	1	1	1.1	1	1	1	1	3	1	1	1	1
						0	0	0	0	6	0	0	0	0	0	0	0
NA	NA	NA	NA	NA	NA	1	1	1.4	1	1	1	1	1	1	1	1	1
NA	NA	NA	NA	NA	NA	1	1	0.9	3	1	1	3	1	1	1	2	1
NA	NA	NA	NA	NA	NA	1	3	5.5	2	0	3	0	2	1	4	1	1
NA	NA	NA	NA	NA	NA	1	2	0.5	3	0	1	1	4	1	2	1	3
NA	NA	NA	Amino aci	Phenylalar	map00400	4	1	0.4	2	0	1	0	1	2	3	1	3
NA	NA	NA	NA	NA	NA	1	1	1	1	1	1	1	1	1	1	1	1
Amino aci	Carboxylic	Amino aci	NA	NA	NA	2	1	0.6	4	1	2	1	2	4	0	2	1
						0	0	0	0	0	0	0	0	0	0	0	0
						0	0	0	0	0	0	0	0	0	0	0	0
						0	0	0	0	0	0	0	0	0	0	0	0
						0	0	0	0	0	0	0	0	0	0	0	0
						0	0	0	0	0	0	0	0	0	0	0	0
						0	0	0	0	0	0	0	0	0	0	0	0
						0	0	0	0	0	0	0	0	0	0	0	0
NA	NA	NA	NA	NA	NA	2	1	0.5	2	0	1	0	1	5	3	1	1
NA	NA	NA	NA	NA	NA	1	1	1	1	1	1	1	1	2	1	1	8
NA	NA	NA	Carbohydr	Amino sug	map00520	6	1	1.2	1	4	26	10	0	0	0	0	2
Hydroxyci	Cinnamic a	Polyketide	NA	NA	NA	1	1	0.4	1	1	0	1	1	1	2	1	2
NA	NA	NA	NA	NA	NA	1	6	1.5	1	4	1	4	1	8	6	1	1
NA	NA	NA	NA	NA	NA	0	8	0.6	0	3	0	4	1	13	5	1	4
NA	NA	NA	NA	NA	NA	0	5	0.3	1	0	1	1	27	1	2	1	2
Fatty acids	Fatty Acyls	Fatty acyls	Lipid meta	Arachidon	map00590	1	1	0.5	1	3	15	4	1	2	0	2	2
NA	NA	NA	NA	NA	NA	1	1	0.8	1	1	1	1	1	1	1	1	1
NA	NA	NA	NA	NA	NA	3	2	0.8	1	0	1	0	1	1	2	0	0
Arylsulfate	Organic su	Organic ac	NA	NA	NA	0	1	0	5	5	1	8	81	0	2	11	0
						0	0	0	0	79	0	0	22	22	24	0	0
Amino aci	Carboxylic	Amino aci	NA	NA	NA	1	1	1.6	1	2	1	2	1	1	1	2	1
NA	NA	NA	NA	NA	NA	3	0	0	2	0	0	0	1	3	2	0	1
NA	NA	NA	NA	NA	NA	1	1	1.2	1	1	1	1	1	2	1	1	1
Amino aci	Carboxylic	Amino aci	Amino aci	Tyrosine n	map00350	1	1	1.2	1	1	1	1	1	2	2	1	0
NA	NA	NA	NA	NA	NA	1	1	0.7	1	1	1	1	4	3	1	2	2
Carbohydr	Organoox	Carbohydr	Amino aci	Cysteine a	map00270	1	1	2.1	1	2	1	3	1	1	1	2	1
Glyceroph	Glyceroph	Glyceroph	NA	NA	NA	0	2	0	1	1	0	1	2	2	3	0	2
NA	NA	NA	NA	NA	NA	1	1	2.2	1	2	1	2	1	1	1	2	1
NA	NA	NA	NA	NA	NA	1	2	1.4	2	0	1	1	1	0	2	1	1
						0	0	0	0	0	0	0	16	0	0	0	0

Amino aci	Carboxylic	Amino aci	NA	NA	NA	1	1	2.3	1	3	1	1	1	1	1	2	1
NA	NA	NA	NA	NA	NA	1	1	1.8	1	2	1	2	1	1	1	2	1
NA	NA	NA	NA	NA	NA	1	1	0.7	1	0	1	1	1	2	3	1	1
NA	NA	NA	NA	NA	NA	0	2	0.2	1	0	0	0	11	6	3	0	13
NA	NA	NA	NA	NA	NA	2	1	1.2	1	1	3	2	1	1	1	1	1
NA	NA	NA	NA	NA	NA	1	1	1.3	1	1	1	1	1	1	1	1	1
NA	NA	NA	NA	NA	NA	1	1	1.9	1	2	1	2	1	1	1	1	1
Amines	Organonit	Amines	Amino aci	Histidine r	map0034(	1	1	2.6	1	3	1	1	1	2	1	1	1
NA	NA	NA	NA	NA	NA	1	1	2.4	1	2	1	2	1	1	1	2	1
NA	NA	NA	NA	NA	NA	1	1	1.5	1	58	1	2	1	1	1	1	1
NA	NA	NA	NA	NA	NA	3	1	1.4	2	2	8	5	2	0	0	5	2
NA	NA	NA	Biosynthes	Clavulanic	map0110(	2	1	1.7	1	1	2	2	1	1	1	2	1
NA	NA	NA	NA	NA	NA	1	2	0.2	1	1	6	1	2	13	0	1	3
NA	NA	NA	NA	NA	NA	1	1	2.2	1	2	1	2	1	1	1	2	1
NA	Benzimida	Imidazole	NA	NA	NA	2	1	0.9	2	13	3	1	1	1	1	1	5
NA	NA	NA	NA	NA	NA	1	1	0.6	1	1	1	1	3	0	2	2	0
O-methyla	Flavonoids	Flavonoids	NA	NA	NA	1	1	2.3	1	1	27	1	1	1	0	1	1
NA	NA	NA	NA	NA	NA	1	2	1	0	3	18	7	1	4	0	1	1
NA	NA	NA	NA	NA	NA	0	14	0.3	0	0	1	0	2	1	0	0	0
NA	NA	NA	NA	NA	NA	1	2	1.3	2	2	1	2	1	2	1	1	1
NA	NA	NA	NA	NA	NA	1	1	2.2	1	2	1	2	1	1	1	2	0
						0	0	0	0	0	0	0	0	0	0	0	0
NA	NA	NA	NA	NA	NA	2	0	16	3	3	0	0	1	1	64	1	3
NA	NA	NA	NA	NA	NA	1	1	1.9	1	1	6	1	1	1	1	0	0
Amino aci	Carboxylic	Amino aci	NA	NA	NA	1	1	0.9	1	1	1	1	1	1	1	1	1
NA	NA	NA	NA	NA	NA	0	0	1	1	0	1	0	1	2	2	1	0
NA	NA	NA	NA	NA	NA	1	1	1.4	1	2	1	2	1	1	1	1	20
Fatty acids	Fatty Acyls	Fatty acyls	NA	NA	NA	1	1	3	0	1	2	3	1	3	0	2	0
NA	NA	NA	NA	NA	NA	1	1	1.1	1	1	1	1	2	1	1	1	2
Halobenze	Benzene a	Benzene a	NA	NA	NA	1	1	0.7	1	0	1	1	1	5	1	2	1
Dicarboxyl	Carboxylic	Organic ac	Lipid meta	Fatty acid	map00061	2	1	0.7	1	0	1	0	1	1	2	1	1
NA	NA	NA	NA	NA	NA	2	1	1.2	1	##	1	1	1	1	1	1	1
Gibberellir	Diterpenoi	Terpenoid	Metabolis	Diterpenoi	NA	3	1	1.7	1	2	2	2	1	1	1	1	1
Furanones	Dihydrofui	Organohe	NA	NA	NA	1	1	2.1	1	1	1	2	1	2	2	2	1
NA	NA	NA	NA	NA	NA	1	1	1.6	1	2	1	2	1	1	1	1	1
NA	NA	NA	NA	NA	NA	1	1	1.2	1	1	1	1	1	2	1	1	1
Benzothia	Thiadiazin	Organohe	NA	NA	NA	1	1	4.3	1	1	1	2	1	1	1	1	0
NA	NA	NA	Metabolis	Polyketide	map0110(	5	0	0.6	6	1	4	1	1	0	0	3	1
						0	0	0	0	2	0	0	0	0	0	0	0
NA	NA	NA	NA	NA	NA	2	1	0.7	1	1	1	0	1	3	1	1	1
NA	NA	NA	NA	NA	NA	1	1	2.1	1	2	1	3	1	1	1	2	1
NA	NA	NA	NA	NA	NA	2	1	16	1	18	##	2	1	1	1	1	7
NA	NA	NA	NA	NA	NA	1	##	0.9	1	1	1	##	1	1	1	1	2
NA	NA	NA	NA	NA	NA	0	1	0.2	4	0	0	0	6	0	19	0	2
NA	NA	NA	NA	NA	NA	2	1	0.7	1	1	1	1	1	1	1	1	1
NA	NA	NA	NA	NA	NA	1	1	0.8	1	1	9	1	1	2	1	1	1
NA	NA	NA	NA	NA	NA	1	1	0.4	1	0	1	1	1	1	1	0	2
NA	NA	NA	NA	NA	NA	1	1	1	1	1	2	1	1	1	1	1	1
NA	NA	NA	NA	NA	NA	3	0	1.6	5	1	1	0	1	3	4	3	0
NA	NA	NA	NA	NA	NA	1	1	0.8	1	0	1	0	1	5	1	1	0
NA	NA	NA	NA	NA	NA	1	1	0.9	1	1	1	1	11	3	4	1	1
NA	NA	NA	NA	NA	NA	1	1	2	1	1	1	2	1	1	1	2	1
Pyrimidine	Diazines	Organohe	NA	NA	NA	2	1	0.5	3	0	1	0	1	1	1	0	0
NA	NA	NA	NA	NA	NA	1	1	1.9	4	2	5	0	1	1	1	3	4
NA	NA	NA	NA	NA	NA	1	1	8.1	1	3	1	2	0	1	1	3	1
Amino aci	Carboxylic	Amino aci	Amino aci	Glycine, se	map0026(	1	1	1.7	2	1	2	2	1	4	2	3	1
NA	NA	NA	Biosynthes	Biosynthes	map0110(	2	0	0.8	1	1	1	0	0	1	1	2	1
NA	NA	NA	NA	NA	NA	1	1	1.9	1	2	1	2	1	1	1	1	1

						0	0	0	0	0	0	0	0	0	0	0	0
Glyceroph	Glyceroph	Glyceroph	NA	NA	NA	0	0	0.3	1	0	2	0	2	1	7	1	0
NA	NA	NA	NA	NA	NA	2	3	6.4	4	6	0	4	1	0	1	2	1
Macrolide:	Diarrhetic	Marine bic	NA	NA	NA	2	1	0.2	2	1	0	0	2	5	2	0	1
Cyanogen	Cyanogen	Amino aci	NA	NA	NA	3	1	0.8	2	0	1	1	0	1	0	0	2
NA	NA	NA	NA	NA	NA	1	1	1.4	1	1	1	1	1	1	1	2	1
NA	NA	NA	NA	NA	NA	1	1	2.3	1	1	1	1	0	1	15	1	1
NA	NA	NA	NA	NA	NA	2	1	0.9	2	1	1	2	1	1	1	1	1
NA	NA	NA	NA	NA	NA	8	2	1.9	13	0	0	0	14	0	5	4	1
NA	NA	NA	NA	NA	NA	1	1	0.8	1	0	1	1	1	3	1	2	1
Eicosanoic	Fatty Acyls	Fatty acyls	NA	NA	NA	1	1	1.2	1	1	1	2	1	2	1	1	1
NA	NA	NA	NA	NA	NA	1	1	0.8	1	1	1	1	1	1	1	1	1
NA	NA	NA	NA	NA	NA	1	1	0.2	2	0	1	0	9	1	1	1	0
NA	NA	NA	NA	NA	NA	1	2	2.7	0	8	12	2	2	0	3	3	0
						0	0	47	0	0	0	0	0	0	0	0	0
NA	NA	NA	NA	NA	NA	1	1	0.9	1	7	1	0	1	1	12	2	0
NA	NA	NA	NA	NA	NA	1	1	1.5	1	1	2	1	1	1	1	1	1
NA	NA	NA	NA	NA	NA	1	1	1.7	1	2	4	2	1	1	1	1	1
NA	NA	NA	NA	NA	NA	0	2	1.3	1	1	1	1	5	3	4	1	2
Amino aci	Carboxylic	Amino aci	NA	NA	NA	2	1	0.7	5	0	0	0	1	1	7	0	0
Triterpeno	Prenol lipi	Terpenoid	NA	NA	NA	1	5	0.3	1	0	2	2	10	0	3	1	17
NA	NA	NA	NA	NA	NA	1	1	1.2	1	1	1	1	2	1	1	1	1
NA	NA	NA	NA	NA	NA	2	1	0.9	1	1	3	2	1	1	1	1	1
Benzoic ac	Benzene a	Benzene a	NA	NA	NA	2	1	1.5	1	1	2	1	1	1	1	1	2
NA	NA	NA	NA	NA	NA	0	18	3	1	4	1	1	26	2	##	1	3
Furanones	Dihydrofu	Organohe	NA	NA	NA	1	1	1.8	1	2	1	2	1	1	1	2	1
NA	NA	NA	NA	NA	NA	1	1	3.1	1	2	1	1	1	1	1	2	1
						0	0	0	0	35	0	9	7	0	28	0	0
NA	NA	NA	NA	NA	NA	10	2	0.3	2	0	1	0	5	1	1	0	2
NA	NA	NA	NA	NA	NA	1	2	1.6	3	1	2	2	1	1	1	1	0
						0	0	0	0	0	0	##	0	0	20	0	0
NA	NA	NA	NA	NA	NA	1	1	1.1	0	1	3	1	1	0	4	2	1
						0	0	0	0	0	0	0	0	0	28	0	0
NA	NA	NA	NA	NA	NA	1	1	1.2	1	2	2	5	1	1	1	2	1
						0	0	0	5	0	0	0	10	0	0	0	0
NA	NA	NA	NA	NA	NA	8	1	0.9	1	0	12	0	2	1	0	1	5
NA	NA	NA	NA	NA	NA	1	1	1.6	1	2	1	2	7	1	1	2	1
NA	NA	NA	NA	NA	NA	2	1	0.5	1	1	2	1	3	1	1	1	61
NA	NA	NA	NA	NA	NA	1	1	0.8	1	1	1	2	1	1	1	1	1
NA	NA	NA	NA	NA	NA	1	1	2.3	10	2	2	1	1	1	1	4	1
Triradylcgl	Glycerolipi	Glycerolipi	NA	NA	NA	1	1	4.6	1	2	1	2	1	1	1	26	0
NA	NA	NA	NA	NA	NA	22	1	0.6	0	0	6	1	0	1	2	1	0
NA	NA	NA	Metabolisı	Porphyrin	map0086C	1	1	2.5	1	2	1	2	1	2	1	2	0
PK1202 Fl	PK12 Flavc	Polyketide	NA	NA	NA	1	6	1.3	1	1	2	1	1	1	1	1	0
1,4-benzo	Benzodiaz	Organohe	NA	NA	NA	1	1	1.5	1	1	25	1	1	1	1	1	3
NA	NA	NA	NA	NA	NA	4	2	1.4	2	2	1	1	1	1	2	1	1
NA	NA	NA	NA	NA	NA	10	1	0.8	1	1	24	1	1	1	1	1	1
NA	NA	NA	NA	NA	NA	1	2	1.1	1	2	1	2	2	1	1	1	1
NA	NA	NA	NA	NA	NA	1	1	2.4	1	2	1	2	3	1	1	2	1
NA	NA	NA	NA	NA	NA	0	1	0.9	1	0	0	0	9	1	1	0	1
NA	NA	NA	NA	NA	NA	1	1	0.9	1	0	1	0	1	3	1	1	1
NA	NA	NA	NA	NA	NA	1	4	0.4	0	1	0	1	1	0	0	0	4
Amino aci	Carboxylic	Amino aci	Amino aci	Arginine b	map0022C	0	2	0	3	1	1	1	1	0	1	1	1
Aldoses [F	Monosacc	Carbohydı	Carbohydı	Pentose aı	map0004C	1	1	0.6	1	1	1	1	1	1	1	1	1
NA	NA	NA	NA	NA	NA	1	1	2	1	2	1	2	1	1	1	2	1
NA	NA	NA	NA	NA	NA	1	1	1.5	2	1	1	1	1	1	2	1	2
NA	NA	NA	NA	NA	NA	1	0	0.2	0	3	0	1	0	5	1	0	39
Fatty acid	Fatty Acyls	Fatty acyls	NA	NA	NA	1	1	0.4	0	1	0	1	1	3	1	0	13

Bilirubins	Tetrapyrro	Organohe	Metabolisı	Porphyrin	map0086C	1	4	0.1	1	1	0	2	10	0	2	##	0
Phosphos	Sphingolip	Sphingolip	NA	NA	NA	2	2	0.9	2	4	44	56	2	64	0	0	1
NA	NA	NA	NA	NA	NA	1	1	2.3	1	1	1	1	1	1	1	1	1
Glucosinol	Glucosinol	Amino aci	NA	NA	NA	1	1	1.2	1	1	1	1	1	21	1	1	4
NA	NA	NA	NA	NA	NA	1	1	0.8	1	1	3	1	0	17	1	1	2
NA	NA	NA	NA	NA	NA	7	1	1.4	2	1	1	2	1	1	1	1	3
NA	NA	NA	NA	NA	NA	1	1	0.7	1	1	3	1	2	1	1	1	1
NA	NA	NA	NA	NA	NA	7	1	1.5	1	1	2	1	1	1	1	1	1
NA	NA	NA	NA	NA	NA	1	0	5.3	2	3	31	0	1	0	1	1	1
NA	NA	NA	NA	NA	NA	0	4	3.2	2	3	1	3	0	2	2	3	1
NA	NA	NA	NA	NA	NA	1	0	2.4	1	0	1	0	2	1	10	3	0
Isoquinolir	Alkaloids (	Alkaloids	NA	NA	NA	1	1	0.5	1	1	1	1	2	27	1	0	3
NA	NA	NA	NA	NA	NA	1	1	1.1	1	1	1	1	1	1	1	1	1
NA	NA	NA	NA	NA	NA	12	1	1.6	16	4	1	2	1	3	1	1	2
Bile acids,	Steroids aı	Bile acids,	NA	NA	NA	1	1	0.3	0	0	1	0	1	1	2	61	0
NA	NA	NA	NA	NA	NA	1	1	1.1	1	1	1	1	1	1	1	1	1
NA	NA	NA	NA	NA	NA	1	1	1	1	1	1	1	1	1	1	9	1
Amino aci	Carboxylic	Amino aci	NA	NA	NA	1	6	0.7	0	1	19	1	2	0	1	1	6
NA	NA	NA	NA	NA	NA	2	1	0.5	0	1	0	2	1	6	0	0	4
NA	NA	NA	NA	NA	NA	1	1	0.9	1	0	1	0	1	2	1	2	1
Amino aci	Carboxylic	Amino aci	Amino aci	Glycine, se	map0026C	2	2	5.8	2	4	2	10	0	9	1	1	0
Substitute	Pyrroles	Organohe	NA	NA	NA	1	1	4.1	1	1	0	0	2	1	7	0	0
NA	NA	NA	NA	NA	NA	1	1	2.4	1	3	2	2	1	8	2	1	1
Caprolacta	Lactams	Organohe	Xenobiotic	Caprolacta	map0110(	3	2	1.2	3	1	1	1	3	1	1	2	1
NA	NA	NA	NA	NA	NA	1	1	1.3	1	2	1	2	2	2	1	1	16
NA	NA	NA	NA	NA	NA	1	1	2	1	1	10	2	1	1	1	2	1
NA	NA	NA	NA	NA	NA	1	1	0.8	1	1	1	1	1	3	1	1	1
NA	NA	NA	NA	NA	NA	4	1	1.4	1	1	1	1	1	1	2	1	4
Bilirubins	Tetrapyrro	Organohe	NA	NA	NA	5	1	1.4	1	1	0	1	1	1	3	1	1
						0	0	0	0	0	0	5	54	21	3	0	19
Non-meta	Non-meta	Homogen	NA	NA	NA	3	2	1	0	4	4	8	1	0	1	4	1
NA	NA	NA	NA	NA	NA	1	0	1	3	1	1	0	1	7	2	1	0
NA	NA	NA	NA	NA	NA	1	1	0.8	2	0	1	0	2	5	4	2	0
NA	NA	NA	NA	NA	NA	6	1	1.1	1	2	2	2	0	1	1	1	1
NA	NA	NA	NA	NA	NA	1	1	2	1	2	2	2	1	1	1	1	1
NA	NA	NA	NA	NA	NA	1	2	1.7	2	1	4	1	1	0	1	2	0
NA	NA	NA	NA	NA	NA	1	1	0.9	1	0	1	1	1	1	1	1	1
NA	NA	NA	NA	NA	NA	1	1	1.9	1	2	1	2	1	1	2	2	1
						0	0	0	0	0	0	0	0	8	0	0	0
NA	NA	NA	NA	NA	NA	##	1	1	1	1	1	1	1	1	1	1	8
Benzoic ac	Benzene a	Benzene a	NA	NA	NA	##	1	1.9	1	1	1	2	1	1	1	1	1
Anthocyar	Flavonoids	Flavonoids	Biosynthes	Anthocyar	NA	1	1	0.8	1	1	1	1	1	1	1	1	1
Pyridineca	Pyridines a	Pyridine aı	NA	NA	NA	1	1	1.1	4	0	0	0	1	1	0	0	0
NA	NA	NA	NA	NA	NA	1	14	1.9	1	2	1	2	1	1	1	2	1
NA	NA	NA	NA	NA	NA	2	2	1	4	0	1	0	1	0	2	1	0
NA	NA	NA	NA	NA	NA	1	1	1.1	1	1	1	1	1	1	1	2	1
NA	NA	NA	NA	NA	NA	1	1	1.6	1	2	1	1	1	1	1	2	1
NA	NA	NA	NA	NA	NA	2	1	1.2	1	1	1	1	1	1	1	1	92
NA	NA	NA	NA	NA	NA	0	1	0.4	2	1	2	2	1	0	1	2	0
NA	NA	NA	NA	NA	NA	1	1	3.4	1	3	1	2	1	1	0	2	1
NA	NA	NA	NA	NA	NA	1	1	0.9	1	1	1	1	1	1	1	1	1
NA	NA	NA	NA	NA	NA	2	1	2.2	1	3	1	6	1	2	1	1	1
NA	NA	NA	NA	NA	NA	3	1	0.9	1	0	0	0	1	1	1	1	4
NA	NA	NA	NA	NA	NA	4	4	1.4	20	3	8	0	0	1	0	6	1
NA	NA	NA	NA	NA	NA	37	1	1.6	1	2	1	2	1	1	1	1	43
NA	NA	NA	NA	NA	NA	##	1	524	1	1	1	1	1	1	1	1	2
Pyrimidine	Diazines	Organohe	Nucleotide	Pyrimidine	map0024C	1	1	1.7	1	2	1	2	1	1	1	1	0
NA	NA	NA	NA	NA	NA	1	3	0.1	2	2	1	0	1	0	1	0	5

NA	NA	NA	NA	NA	NA	0	2	0.6	0	0	0	0	7	2	4	0	1
NA	NA	NA	NA	NA	NA	2	4	2.4	1	1	22	1	7	1	2	1	3
NA	NA	NA	NA	NA	NA	0	2	0.1	0	2	0	1	4	4	1	0	6
						0	0	0	0	0	0	8	76	0	0	0	0
NA	NA	NA	NA	NA	NA	3	2	0.3	1	0	1	0	3	2	1	0	16
NA	NA	NA	NA	NA	NA	0	1	1.5	2	0	1	1	1	1	2	1	1
NA	NA	NA	NA	NA	NA	4	1	0.6	1	2	15	4	1	7	1	1	1
						0	0	0	0	4	0	0	0	0	0	0	0
						0	0	0	3	58	0	0	0	0	15	0	0
NA	NA	NA	Metabolisı	Polyketide	map0110	42	1	1.2	1	1	1	2	1	1	1	2	1
NA	NA	NA	NA	NA	NA	1	2	0.5	1	1	1	0	2	2	1	1	1
Beta lactar	Lactams	Organohe	NA	NA	NA	1	1	0.9	1	0	1	0	1	1	1	1	0
NA	NA	NA	NA	NA	NA	1	1	1.7	1	2	4	2	1	1	1	1	1
NA	NA	NA	NA	NA	NA	1	9	0.3	5	0	17	0	3	7	1	0	6
NA	NA	NA	NA	NA	NA	1	9	0.9	1	1	2	0	2	0	1	1	1
NA	NA	NA	NA	NA	NA	1	1	0.8	1	0	1	0	1	6	1	1	1
NA	NA	NA	NA	NA	NA	2	1	1.3	1	1	1	1	1	1	1	1	1
NA	NA	NA	NA	NA	NA	5	3	0.6	2	2	0	0	1	1	0	0	1
NA	NA	NA	NA	NA	NA	3	2	1.5	0	1	1	4	1	0	1	2	1
NA	NA	NA	NA	NA	NA	1	1	1.9	1	2	1	2	1	1	1	2	8
NA	NA	NA	NA	NA	NA	1	1	1.1	1	1	1	1	1	1	1	1	1
NA	NA	NA	NA	NA	NA	1	2	0.3	3	0	4	0	1	0	1	0	4
NA	NA	NA	NA	NA	NA	1	1	1	1	1	1	1	1	2	1	1	1
NA	NA	NA	NA	NA	NA	1	4	1.3	1	2	1	1	1	1	1	3	1
NA	NA	NA	NA	NA	NA	1	1	4.3	1	2	1	45	1	1	1	2	78
NA	NA	NA	NA	NA	NA	0	2	0.9	1	2	13	3	2	1	1	1	3
						0	0	0	0	0	0	0	0	0	0	0	0
NA	NA	NA	NA	NA	NA	0	4	0.1	0	1	0	1	2	4	1	0	32
NA	NA	NA	NA	NA	NA	2	1	0.8	1	1	1	0	7	1	1	1	4
NA	Nucleosid	Nucleic ac	NA	NA	NA	5	1	1.7	1	2	1	2	1	1	1	1	1
Glyceroph	Glyceroph	Glyceroph	NA	NA	NA	0	2	0.2	1	0	0	0	11	6	3	0	13
NA	NA	NA	NA	NA	NA	1	1	1.2	1	1	1	1	1	1	1	2	1
NA	NA	NA	NA	NA	NA	0	1	0.8	1	1	19	1	3	2	1	1	13
NA	NA	NA	NA	NA	NA	1	2	0.2	1	0	1	0	3	3	2	1	6
NA	NA	NA	NA	NA	NA	2	9	1	24	1	8	0	0	3	0	9	5
NA	NA	NA	NA	NA	NA	1	1	2	1	2	1	2	1	1	1	2	0
						0	0	0	0	0	0	0	0	0	0	0	0
NA	NA	NA	NA	NA	NA	1	1	2.1	1	2	1	2	1	1	1	1	1
NA	NA	NA	NA	NA	NA	1	1	2.2	1	2	1	3	1	1	1	1	1
NA	NA	NA	NA	NA	NA	1	1	1.2	1	1	1	1	1	2	1	1	1
NA	NA	NA	NA	NA	NA	1	1	2.8	1	1	1	1	1	1	1	1	1
NA	Tetralins	Benzene a	NA	NA	NA	1	1	0.9	1	1	1	1	1	1	1	1	1
NA	NA	NA	NA	NA	NA	0	4	0.7	0	1	0	1	34	4	3	0	5
Sesquiterp	Prenol lipi	Terpenoid	NA	NA	NA	23	1	1.1	1	1	2	1	1	1	1	2	6
						0	0	0	0	0	0	0	0	0	0	0	0
Eicosanoic	Fatty Acyls	Fatty acyls	Lipid meta	Arachidon	map0059	1	1	2.3	1	2	1	2	1	1	1	2	0
						0	0	0	0	0	0	0	41	0	0	0	15
Monoterp	Prenol lipi	Terpenoid	NA	NA	NA	2	2	0.2	1	0	1	0	1	1	1	1	2
NA	NA	NA	NA	NA	NA	1	1	1	2	1	1	1	4	1	1	2	1
NA	NA	NA	NA	NA	NA	4	1	0.8	1	1	1	1	1	2	4	1	1
						0	0	0	0	0	0	0	0	0	0	0	0
NA	NA	NA	NA	NA	NA	10	1	0	1	1	0	0	1	11	0	0	1
NA	NA	NA	NA	NA	NA	1	1	1.6	1	1	1	2	2	1	1	2	1
NA	NA	NA	NA	NA	NA	1	1	1.4	1	2	1	1	2	2	2	1	2
NA	NA	NA	NA	NA	NA	0	1	5.4	3	0	1	0	1	2	0	3	0
NA	NA	NA	NA	NA	NA	1	26	0.5	1	1	2	1	11	1	0	0	1
NA	NA	NA	NA	NA	NA	1	1	1.6	1	1	2	1	1	1	1	1	1
NA	NA	NA	NA	NA	NA	4	2	0.7	3	1	0	3	2	0	1	10	1

Hydropyri	Pyridines a	Pyridine ar	NA	NA	NA	3	1	0.8	2	0	1	0	2	1	2	1	1
Quinone a	Prenol lipi	Quinone	Metabolis	Ubiquinon	map0013C	1	2	0.2	1	1	0	4	1	2	4	0	3
NA	NA	NA	NA	NA	NA	1	0	23	1	5	70	1	1	1	1	1	11
						0	0	0	0	0	0	0	0	0	6	0	0
						0	0	0	0	0	0	0	0	0	12	0	0
NA	NA	NA	NA	NA	NA	1	1	1	1	1	1	1	1	2	1	1	1
NA	NA	NA	NA	NA	NA	85	1	1.4	3	63	1	2	1	1	1	1	42
Fatty acids	Fatty Acyls	Fatty acyls	NA	NA	NA	1	2	3.4	1	3	2	3	1	1	1	1	0
NA	NA	NA	NA	NA	NA	8	1	0.7	10	1	1	1	4	1	7	1	3
1,4-benzo	Benzodiaz	Organohe	NA	NA	NA	1	1	1.2	0	2	1	2	1	1	1	1	1
NA	NA	NA	NA	NA	NA	1	1	0.8	0	3	33	1	1	8	0	1	5
NA	NA	NA	NA	NA	NA	2	2	1.2	1	1	1	1	2	1	1	1	1
NA	NA	NA	NA	NA	NA	1	1	0.6	1	1	1	0	1	1	1	1	1
Halobenze	Benzene a	Benzene a	NA	NA	NA	1	1	1.8	1	1	2	2	1	1	1	2	1
NA	NA	NA	NA	NA	NA	6	1	0.9	7	1	41	1	32	2	1	4	3
NA	NA	NA	NA	NA	NA	2	2	2.5	2	5	1	3	1	0	0	0	1
Amino aci	Carboxylic	Amino aci	NA	NA	NA	4	2	1.8	2	7	32	10	0	0	0	0	3
NA	NA	NA	NA	NA	NA	0	1	3.7	1	2	3	2	0	1	1	1	0
NA	NA	NA	NA	NA	NA	1	1	0.9	1	1	1	1	1	1	1	1	1
NA	NA	NA	NA	NA	NA	2	1	1.5	1	1	2	1	1	1	2	1	3
Benzodiaz	Diazanaph	Organohe	NA	NA	NA	1	1	1.9	1	1	2	1	1	1	1	2	1
NA	NA	NA	NA	NA	NA	1	1	2.1	1	2	1	2	1	1	1	2	1
NA	NA	NA	NA	NA	NA	0	2	0.5	1	1	1	0	1	0	7	1	1
NA	NA	NA	NA	NA	NA	1	0	0.9	1	1	1	2	2	1	1	1	1
NA	NA	NA	NA	NA	NA	2	1	1.8	1	2	1	2	1	1	1	2	1
Isoflav-2-(	Isoflavono	Flavonoids	NA	NA	NA	1	1	0.7	1	1	1	1	2	1	1	1	4
NA	NA	NA	NA	NA	NA	2	1	1.4	1	1	1	2	1	1	1	1	1
Quinoline	Alkaloids (	Alkaloids	NA	NA	NA	1	1	0.4	2	0	1	0	1	0	1	0	1
NA	NA	NA	NA	NA	NA	1	1	1.6	1	2	1	2	11	1	1	1	10
NA	NA	NA	NA	NA	NA	1	2	0.7	0	3	2	3	1	2	1	1	1
NA	NA	NA	NA	NA	NA	2	1	0.1	2	0	2	0	2	0	5	0	2
Phenylpip	Piperidine	Organohe	NA	NA	NA	1	1	1	1	1	13	1	1	1	1	1	1
NA	NA	NA	NA	NA	NA	2	3	0.2	1	0	2	0	6	3	0	0	0
Bile acids,	Steroids a	Bile acids,	NA	NA	NA	0	2	0.1	1	0	1	0	6	3	0	1	17
NA	NA	NA	NA	NA	NA	1	1	1.6	2	1	0	1	1	1	2	2	1
NA	Stilbenes	Polyketide	Xenobiotic	Drug meta	map00982	1	2	0.1	1	0	0	0	1	1	0	0	10
1-benzoth	Benzothio	Organohe	NA	NA	NA	1	8	0.6	5	1	1	1	2	1	2	1	0
NA	NA	NA	NA	NA	NA	4	1	1.2	1	1	1	1	1	1	1	1	1
NA	NA	NA	NA	NA	NA	1	3	1.3	1	2	1	2	1	1	1	2	1
NA	NA	NA	NA	NA	NA	12	1	0	1	2	0	0	1	7	0	0	1
NA	NA	NA	NA	NA	NA	1	1	2.1	1	2	1	2	1	1	1	2	1
NA	NA	NA	NA	NA	NA	1	1	1.5	1	2	1	1	1	1	3	2	1
NA	NA	NA	NA	NA	NA	1	4	1	1	1	1	1	1	2	3	1	8
NA	NA	NA	NA	NA	NA	1	3	1.2	1	1	1	1	2	1	1	1	1
NA	NA	NA	NA	NA	NA	1	4	0.8	5	2	3	0	0	2	0	##	7
NA	NA	NA	NA	NA	NA	2	1	0.7	1	0	1	0	1	1	2	1	1
NA	NA	NA	NA	NA	NA	1	1	0.3	0	0	1	1	2	1	3	1	1
NA	NA	NA	NA	NA	NA	0	3	0.5	8	0	1	3	13	4	1	4	5
Pyrimidine	Pyrimidine	Nucleic ac	NA	NA	NA	1	2	0.7	1	1	43	6	2	1	1	11	1
NA	NA	NA	NA	NA	NA	1	1	1.1	1	1	1	2	8	4	1	2	1
NA	NA	NA	NA	NA	NA	3	1	4.4	2	3	2	1	1	0	1	2	3
NA	NA	NA	NA	NA	NA	0	1	0.1	1	1	0	1	8	1	1	0	2
NA	NA	NA	NA	NA	NA	1	1	0.6	1	0	11	1	2	0	2	1	8
NA	NA	NA	NA	NA	NA	3	2	0.3	1	0	1	0	3	2	1	0	16
NA	NA	NA	NA	NA	NA	1	1	1.7	1	2	1	2	1	1	1	2	1
Amino aci	Carboxylic	Amino aci	Amino aci	Alanine, as	map0025C	1	1	2	2	2	7	5	1	4	1	5	2
Phenylpro	Benzene a	Benzene a	NA	NA	NA	1	1	0.9	1	1	1	1	1	2	1	2	0
Anthocyar	Flavonoids	Flavonoids	Biosynthes	Anthocyar	NA	1	1	2	1	2	1	2	1	1	1	2	1

NA	NA	NA	NA	NA	NA	1	1	0.7	1	0	1	1	1	5	1	2	1
						0	0	0	0	91	0	0	0	28	0	0	0
Tetrahydr	Furanoid l	Lignans ar	NA	NA	NA	1	0	2.2	1	1	1	1	0	1	11	1	1
Amino aci	Carboxylic	Amino aci	NA	NA	NA	3	0	1	2	1	6	3	1	1	0	2	2
NA	NA	NA	NA	NA	NA	1	1	0.8	1	1	1	2	1	1	1	1	1
Estrane ste	Steroids ar	Steroids ar	Lipid meta	Steroid ho	map0014C	1	4	0.5	1	1	0	1	2	2	0	1	3
NA	NA	NA	NA	NA	NA	3	0	1	2	1	6	3	1	1	0	2	2
NA	NA	NA	NA	NA	NA	1	1	1.3	1	1	1	2	1	1	1	1	1
NA	NA	NA	NA	NA	NA	##	1	1.4	1	1	1	2	1	1	1	1	4
NA	NA	NA	NA	NA	NA	2	1	0.4	0	0	0	0	0	2	5	0	7
Bile acids,	Steroids ar	Bile acids,	NA	NA	map04976	1	0	0.7	2	0	0	0	3	1	2	1	1
NA	NA	NA	NA	NA	NA	0	1	0.1	1	3	0	1	3	9	1	0	17
NA	NA	NA	NA	NA	NA	1	1	1.3	1	1	1	1	2	3	4	1	2
NA	NA	NA	NA	NA	NA	1	1	4.1	1	1	0	0	2	1	6	0	0
NA	NA	NA	NA	NA	NA	2	2	5.1	4	6	0	4	1	0	1	2	1
NA	NA	NA	NA	NA	NA	1	1	2	1	2	1	2	1	2	1	2	1
NA	NA	NA	NA	NA	NA	1	0	2.7	1	1	1	3	1	1	1	0	3
NA	NA	NA	NA	NA	NA	1	1	0.8	1	0	1	0	1	6	1	1	1
NA	NA	NA	NA	NA	NA	1	1	0.7	0	1	2	1	2	1	1	0	1
NA	NA	NA	NA	NA	NA	0	4	0.3	1	0	3	0	1	0	0	0	2
NA	NA	NA	NA	NA	NA	1	1	0.9	1	1	1	1	1	1	1	1	1
NA	NA	NA	NA	NA	NA	1	1	1.6	1	1	2	1	1	1	1	1	1
Fatty acids	Fatty Acyls	Fatty acyls	Lipid meta	Biosynthes	map0104C	0	2	4.7	1	1	##	3	1	1	1	1	3
NA	NA	NA	NA	NA	NA	1	1	1	1	1	1	1	2	1	1	1	1
Amino aci	Carboxylic	Amino aci	Metabolis	D-Glutami	map00471	1	2	4.3	1	5	12	31	0	1	0	12	1
Sesquiterp	Prenol lipi	Terpenoid	NA	NA	NA	1	2	0.5	1	1	1	0	1	2	2	1	2
NA	NA	NA	NA	NA	NA	1	1	0.4	1	1	6	1	6	1	0	1	1
NA	NA	NA	NA	NA	NA	1	1	3	1	2	1	2	1	1	1	2	0
NA	NA	NA	NA	NA	NA	1	74	2.7	1	1	1	1	0	1	1	1	1
Dicarboxyl	Carboxylic	Organic ac	NA	NA	NA	6	1	0.9	1	0	1	1	1	1	1	1	0
NA	NA	NA	NA	NA	NA	12	0	0.8	1	1	1	1	2	0	1	1	7
NA	NA	NA	NA	NA	NA	8	1	0.7	1	1	20	1	12	1	0	1	21
						0	0	0	0	0	0	67	23	0	0	0	0
NA	NA	NA	NA	NA	NA	1	1	1.2	1	1	1	1	1	1	1	1	1
						0	0	0	7	55	0	13	48	22	93	0	0
NA	Benzene a	Benzene a	NA	NA	NA	1	2	0.6	1	0	1	0	3	2	3	2	1
NA	NA	NA	NA	NA	NA	1	1	1.8	1	2	1	2	1	1	1	1	1
NA	NA	NA	NA	NA	NA	1	2	0.9	1	2	2	1	3	0	1	2	0
NA	Imidazodi	Organohe	NA	NA	NA	1	6	0.6	3	3	0	5	2	0	1	0	0
Pyrazoles	Azoles	Organohe	NA	NA	NA	1	1	0.9	3	1	1	1	7	1	5	1	28
NA	NA	NA	NA	NA	NA	1	2	1.3	1	1	2	2	1	1	1	1	1
NA	NA	NA	NA	NA	NA	1	1	1	1	1	1	1	1	1	1	1	1
NA	NA	NA	NA	NA	NA	1	1	0.9	1	1	1	1	2	1	1	1	2
NA	NA	NA	Biosynthes	Phenazine	map0110(	2	1	1.9	4	0	2	0	2	0	4	2	0
NA	NA	NA	NA	NA	NA	1	1	0.9	1	1	1	1	1	1	1	1	1
NA	NA	NA	NA	NA	NA	1	1	1.5	1	2	1	2	5	1	1	1	1
Peptide nu	Others [Fi	Antibiotics	NA	NA	NA	1	1	1.3	1	66	1	0	1	2	7	1	1
NA	NA	NA	NA	NA	NA	1	1	0.9	1	1	1	1	2	1	1	1	1
NA	NA	NA	NA	NA	NA	1	2	1.5	1	2	1	3	1	1	1	1	1
NA	NA	NA	NA	NA	NA	1	1	1.4	1	1	2	2	1	1	1	1	1
NA	NA	NA	Metabolis	Riboflavin	map0074C	1	1	1.4	1	1	1	2	1	1	1	3	1
NA	NA	NA	NA	NA	NA	1	4	1.6	2	1	1	1	1	1	1	1	1
NA	NA	NA	NA	NA	NA	1	1	0.8	1	1	4	1	1	1	1	1	1
NA	NA	NA	NA	NA	NA	1	1	0.9	1	1	1	1	1	2	1	1	1
NA	NA	NA	NA	NA	NA	1	2	0.5	1	1	1	0	2	2	1	1	3
NA	NA	NA	NA	NA	NA	1	1	1	1	1	1	1	1	2	2	2	0
NA	NA	NA	NA	NA	NA	2	1	1.1	1	2	1	2	1	1	1	1	1
Fatty acyl ı	Fatty Acyls	Fatty acyls	NA	NA	NA	1	1	1.5	1	1	2	1	1	1	1	1	1

NA	NA	NA	NA	NA	NA	1	1	0.7	2	1	0	1	2	1	1	26	0
NA	NA	NA	NA	NA	NA	5	1	0.8	0	78	0	1	1	##	0	0	2
NA	NA	NA	NA	NA	NA	1	1	1.6	1	2	1	2	1	1	1	1	1
NA	NA	NA	NA	NA	NA	1	1	1.1	1	1	3	1	1	3	1	1	1
Amino aci	Carboxylic	Amino aci	NA	NA	NA	1	1	1.9	1	2	1	4	1	1	1	2	2
NA	NA	NA	NA	NA	NA	2	1	1.6	2	1	2	0	3	3	3	1	21
Amino aci	Carboxylic	Amino aci	NA	NA	NA	1	1	0.4	1	1	0	1	1	1	1	2	1
NA	NA	NA	NA	NA	NA	1	1	0.8	1	2	9	1	1	1	1	1	1
NA	NA	NA	NA	NA	NA	0	9	0.6	0	4	0	5	1	12	3	1	4
NA	NA	NA	NA	NA	NA	2	0	3.1	1	4	1	3	0	2	1	3	0
NA	NA	NA	NA	NA	NA	1	1	1.5	1	1	2	1	1	1	1	1	1
NA	NA	NA	NA	NA	NA	1	1	1.1	1	1	1	1	1	1	1	1	1
NA	NA	NA	NA	NA	NA	1	1	1.2	1	2	1	1	1	1	1	2	1
NA	NA	NA	NA	NA	NA	2	0	0.3	3	0	5	0	2	2	0	0	0
						0	0	0	0	16	0	0	0	0	0	0	0
NA	NA	NA	NA	NA	NA	1	1	1.2	1	1	1	1	1	1	1	1	1
NA	NA	NA	NA	NA	NA	##	1	1.5	1	1	1	2	1	1	3	2	1
NA	NA	NA	NA	NA	NA	0	2	0.2	0	2	0	1	3	4	1	0	5
NA	NA	NA	NA	NA	NA	0	3	0.3	0	1	0	1	3	1	1	0	4
Purines an	Imidazopy	Purines an	NA	NA	NA	8	1	1.4	1	1	10	2	1	1	1	1	1
NA	NA	NA	NA	NA	NA	2	0	0.2	0	0	0	0	3	3	3	0	15
Benzenesu	Benzene a	Benzene a	NA	NA	NA	1	1	1.2	1	1	4	1	1	1	1	1	1
NA	NA	NA	NA	NA	NA	3	1	0.2	2	0	0	0	1	1	3	0	0
NA	NA	NA	NA	NA	NA	1	1	0.9	1	1	1	1	3	1	1	1	1
NA	NA	NA	NA	NA	NA	2	1	1.7	1	2	1	2	1	1	1	1	1
Arylsulfate	Organic su	Organic ac	NA	NA	NA	1	3	0.5	1	6	1	7	1	2	3	1	0
NA	NA	NA	NA	NA	NA	1	1	1	1	1	2	2	3	1	1	1	1
NA	NA	NA	NA	NA	NA	1	12	0.5	1	2	0	6	0	11	2	1	2
NA	NA	NA	NA	NA	NA	1	1	1.2	1	1	1	1	2	1	1	1	1
NA	NA	NA	NA	NA	NA	2	1	1.9	3	0	2	0	1	0	2	1	0
NA	NA	NA	NA	NA	NA	1	1	1.4	1	2	8	1	1	1	1	2	1
PR0102 C1	PR01 Isop	Prenol lipi	NA	NA	NA	1	1	2	1	2	2	2	1	1	1	2	1
NA	NA	NA	NA	NA	NA	2	1	0.6	4	0	0	0	1	1	2	0	0
NA	NA	NA	NA	NA	NA	1	3	0.4	1	0	1	0	3	1	1	1	7
NA	NA	NA	NA	NA	NA	1	1	0.8	1	1	1	1	2	0	1	1	1
NA	NA	NA	NA	NA	NA	1	1	0.7	2	0	1	1	1	4	1	1	1
Sulfinylber	Benzimida	Imidazole	NA	NA	NA	1	1	0.9	1	0	1	0	1	2	##	2	1
NA	NA	NA	NA	NA	NA	1	0	1.2	1	0	1	1	0	0	1	1	1
NA	NA	NA	NA	NA	NA	0	1	2.1	1	2	1	2	1	1	1	1	0
NA	NA	NA	NA	NA	NA	1	1	0.7	1	1	1	1	1	1	1	1	1
NA	NA	NA	NA	NA	NA	7	1	1.4	1	1	2	1	1	1	1	1	29
NA	NA	NA	NA	NA	NA	1	1	1.4	1	1	1	2	1	1	1	2	1
NA	NA	NA	NA	NA	NA	1	1	0.8	0	2	1	3	1	1	1	1	1
						0	0	0	0	0	0	20	9	0	2	0	0
NA	NA	NA	NA	NA	NA	7	1	1.5	1	1	2	1	1	1	1	1	1
NA	NA	NA	NA	NA	NA	1	2	1.8	2	0	1	0	1	0	2	1	0
NA	NA	NA	NA	NA	NA	2	0	0.1	2	0	1	0	1	1	2	1	1
NA	NA	NA	NA	NA	NA	3	0	0.5	4	0	1	1	7	1	3	5	1
NA	NA	NA	NA	NA	NA	1	1	1	1	1	1	1	1	1	1	2	1
NA	NA	NA	NA	NA	NA	1	1	1.2	0	1	4	1	1	1	2	3	1
NA	Dibenzocy	Benzene a	NA	NA	NA	1	1	1.1	1	0	0	0	1	0	1	0	0
NA	NA	NA	NA	NA	NA	4	1	1.3	1	1	4	2	3	1	1	1	0
NA	NA	NA	NA	NA	NA	2	1	1.1	1	4	0	3	0	3	1	0	2
NA	NA	NA	NA	NA	NA	4	0	1	1	1	0	3	1	2	1	7	1
NA	NA	NA	NA	NA	NA	2	3	0.5	5	1	8	12	0	2	0	1	1
Carbohydr	Organoox	Carbohydr	NA	NA	NA	1	1	1.2	1	1	4	13	1	1	1	1	1
						0	0	0	0	0	0	0	0	0	0	0	0
						0	0	0	0	0	0	0	0	0	0	0	0

						0	0	0	0	0	0	0	0	0	0	0	0
						0	0	0	0	0	0	0	0	0	0	0	0
						0	0	0	0	0	0	0	0	0	0	0	0
						0	0	0	0	0	0	0	0	0	0	0	0
NA	NA	NA	NA	NA	NA	1	1	0.9	1	1	1	0	1	2	1	1	1
NA	NA	NA	NA	NA	NA	1	1	2.4	1	2	1	2	1	1	1	1	1
PK1211 Fla	PK12 Flavo	Polyketide	NA	NA	NA	1	1	1.1	1	2	1	1	1	2	1	2	1
NA	NA	NA	NA	NA	NA	2	1	0.7	2	0	3	0	1	3	1	1	0
NA	NA	NA	NA	NA	NA	2	0	0	##	##	14	0	0	3	0	50	0
Phenothia	Benzothia	Organohe	NA	NA	NA	0	2	0.1	3	1	1	1	1	1	2	2	1
Sulfated st	Steroids a	Steroids a	Lipid meta	Steroid ho	map0014C	1	2	0.1	0	0	1	1	2	2	1	0	16
NA	NA	NA	NA	NA	NA	1	1	1.3	1	2	1	1	1	1	1	2	1
NA	NA	NA	Metabolis	Riboflavin	map0074C	1	1	0.8	1	0	1	0	1	5	1	1	0
NA	NA	NA	NA	NA	NA	##	1	1.8	0	0	0	1	1	1	0	1	2
NA	NA	NA	NA	NA	NA	0	1	0	1	2	0	1	2	1	5	0	4
NA	NA	NA	NA	NA	NA	2	9	1.9	14	1	12	1	0	3	1	2	3
NA	NA	NA	NA	NA	NA	1	1	1.5	1	1	2	1	1	1	1	1	1
NA	NA	NA	NA	NA	NA	1	1	1.2	1	2	7	4	1	1	1	9	2
NA	NA	NA	NA	NA	NA	1	1	1.3	1	2	1	1	1	1	1	2	1
NA	NA	NA	NA	NA	NA	1	1	0.7	1	1	1	1	1	1	2	1	1
						0	0	0	0	0	0	0	0	0	59	0	0
NA	NA	NA	NA	NA	NA	3	1	1.9	1	2	15	2	1	1	1	2	1
						0	0	0	7	16	4	54	70	0	51	0	0
NA	NA	NA	NA	NA	NA	4	1	1.2	1	30	1	2	4	1	1	1	1
NA	NA	NA	NA	NA	NA	1	0	1.6	1	8	1	1	1	3	0	0	1
						0	0	0	0	0	0	17	0	0	70	0	42
NA	NA	NA	NA	NA	NA	1	1	1.8	1	2	1	2	3	1	1	1	1
Cumenes	Benzene a	Benzene a	NA	NA	NA	1	1	0.6	1	1	1	1	2	2	1	1	1
Carbonyl (	Organoox	Organic o:	Biosynthes	Isoquinolir	NA	1	1	2.2	3	0	5	1	5	0	1	4	0
Carbohydı	Organoox	Carbohydı	Carbohydı	Pentose ar	map0004C	1	1	1.7	42	32	1	2	1	1	10	1	2
NA	NA	NA	NA	NA	NA	2	0	0.3	2	1	0	0	1	0	3	0	1
						0	0	0	0	59	0	46	0	0	52	0	0
NA	NA	NA	NA	NA	NA	1	3	0.2	4	0	1	1	6	1	2	0	1
Fatty acid	Fatty Acyls	Fatty acyls	NA	NA	NA	2	1	0.5	1	0	1	0	1	1	2	1	1
NA	NA	NA	Biosynthes	Biosynthes	map0110	65	1	1.1	2	1	4	1	2	1	0	1	1
NA	NA	NA	NA	NA	NA	0	2	0.9	1	1	3	1	2	1	1	1	0
NA	NA	NA	NA	NA	NA	1	1	2.3	1	2	1	2	1	1	1	1	1
						0	0	0	0	0	0	##	##	19	0	0	0
NA	NA	NA	NA	NA	NA	2	1	0.3	7	0	1	0	4	0	2	1	0
NA	NA	NA	NA	NA	NA	1	1	1.8	1	2	1	4	1	1	1	2	0
NA	NA	NA	NA	NA	NA	1	1	1	1	1	1	1	1	1	1	2	1
Purines an	Imidazopy	Purines an	Nucleotide	Purine me	map0023C	1	1	1.9	1	2	1	2	1	1	1	2	1
NA	NA	NA	NA	NA	NA	1	2	0.7	1	0	0	0	2	0	3	3	0
Arylsulfate	Organic su	Organic ac	NA	NA	NA	0	13	0.2	3	0	7	0	7	0	11	14	0
Glyceroph	Glyceroph	Glyceroph	NA	NA	NA	1	1	0.1	3	2	1	1	3	6	3	0	7
NA	NA	NA	NA	NA	NA	2	1	1.6	1	1	1	2	1	1	1	1	1
Piperidino	Piperidine	Organohe	NA	NA	NA	1	2	4.8	1	1	0	6	0	2	2	4	1
NA	NA	NA	NA	NA	NA	0	0	1.5	0	2	1	2	2	1	1	1	1
NA	Macrolide	Polyketide	NA	NA	NA	1	1	0.7	1	1	1	1	1	1	2	1	1
NA	NA	NA	NA	NA	NA	1	1	1.5	1	1	4	2	4	1	1	1	1
NA	NA	NA	Lipid meta	Steroid bic	map0010C	1	1	0.4	1	0	4	0	5	0	6	2	4
NA	NA	NA	NA	NA	NA	1	1	1.9	1	20	1	10	1	8	1	2	0
Substitute	Pyrroles	Organohe	NA	NA	NA	1	1	0.2	0	1	1	1	1	2	1	1	0
NA	NA	NA	Metabolis	Folate bio:	map0079C	1	1	1.1	1	2	1	1	1	1	1	1	1
NA	NA	NA	NA	NA	NA	1	1	1.1	1	2	1	1	1	2	1	2	1
NA	NA	NA	NA	NA	NA	1	1	1.2	1	1	1	1	1	1	1	1	1
NA	Purine nuc	Purines an	Nucleotide	Purine me	map0023C	0	1	0.4	9	1	2	1	3	0	2	3	0
NA	NA	NA	NA	NA	NA	1	1	0.8	1	0	1	0	1	5	1	1	1

NA	NA	NA	NA	NA	NA	1	1	1	0	1	1	2	1	1	1	1	1
Phenylpro	Benzene a	Benzene a	NA	NA	NA	1	2	0.5	1	0	1	0	5	2	2	0	5
NA	NA	NA	NA	NA	NA	2	2	0.7	1	1	10	1	1	0	0	1	1
NA	NA	NA	NA	NA	NA	1	0	9	0	2	##	2	1	1	0	1	7
NA	NA	NA	NA	NA	NA	1	1	2.4	1	2	1	2	1	1	1	2	0
Phenethyl	Benzene a	Amines an	NA	NA	NA	1	1	2.2	2	1	1	0	1	0	4	3	0
NA	NA	NA	Xenobiotic	Benzoate (	map0110	1	1	1.3	1	1	1	2	1	3	1	1	1
Diradylgly	Glycerolipi	Glycerolipi	NA	NA	NA	1	4	1.1	1	1	0	1	17	1	13	1	1
Sulfated st	Steroids ar	Steroids ar	NA	NA	NA	1	1	0.8	1	1	2	1	1	13	1	1	4
						0	0	0	0	0	0	0	0	0	10	0	0
NA	NA	NA	NA	NA	NA	1	2	0.2	1	0	4	0	6	1	1	1	1
NA	NA	NA	NA	NA	NA	1	1	0.1	2	0	0	0	4	2	1	0	3
NA	NA	NA	NA	NA	NA	1	4	2.7	1	2	71	2	1	2	1	2	1
NA	NA	NA	NA	NA	NA	4	8	0.3	3	0	4	0	2	0	0	0	0
NA	NA	NA	NA	NA	NA	2	1	1.1	1	1	1	1	1	2	1	1	2
NA	NA	NA	NA	NA	NA	1	5	0.4	1	0	6	0	2	1	1	0	1
NA	NA	NA	NA	NA	NA	0	2	0.4	2	1	0	1	3	0	1	1	0
NA	NA	NA	NA	NA	NA	1	1	1.4	1	1	1	2	1	1	1	1	1
Carbohydr	Organoox	Carbohydr	NA	NA	NA	0	15	0.4	1	0	1	1	1	10	1	0	7
						0	0	0	0	0	0	0	0	0	0	0	0
NA	NA	NA	NA	NA	NA	4	1	0.8	3	1	0	0	0	1	4	0	0
NA	NA	NA	NA	NA	NA	1	1	0.7	1	0	1	1	1	1	1	1	1
Purine 2'-(	Purine nuc	Purines an	NA	NA	NA	1	1	1.1	1	1	3	1	1	1	1	1	4
NA	NA	NA	Biosynthes	Neomycin	map00524	2	1	1.1	3	1	1	1	2	1	8	1	1
NA	NA	NA	NA	NA	NA	1	1	0.6	1	1	2	1	1	1	1	1	17
NA	NA	NA	NA	NA	NA	1	1	0.3	1	0	1	0	3	1	1	1	2
NA	NA	NA	NA	NA	NA	3	2	2	3	1	0	2	1	1	1	1	1
NA	NA	NA	NA	NA	NA	1	1	1.5	1	1	2	3	1	1	1	2	0
NA	NA	NA	NA	NA	NA	1	7	1.1	1	1	2	1	10	0	3	14	0
NA	NA	NA	NA	NA	NA	1	1	1.4	1	1	5	2	2	1	1	1	4
NA	NA	NA	Metabolisr	Biosynthes	NA	6	1	1.5	1	1	2	1	1	1	1	1	26
NA	NA	NA	NA	NA	NA	1	2	1.5	1	2	1	3	1	1	1	1	5
						0	0	0	0	##	32	0	0	0	0	0	0
Guaianolic	Sesquiterp	Terpenoid	NA	NA	NA	3	1	1.5	1	2	2	2	1	1	1	1	1
NA	NA	NA	NA	NA	NA	2	1	0.8	19	1	9	1	2	1	0	0	2
NA	NA	NA	NA	NA	NA	1	2	0.8	1	0	2	0	2	0	1	1	0
NA	NA	NA	NA	NA	NA	1	1	1.1	1	1	1	1	1	1	1	1	1
NA	NA	NA	NA	NA	NA	1	1	2.6	1	2	1	2	1	1	1	2	0
NA	NA	NA	NA	NA	NA	1	1	1.2	3	3	1	15	2	1	1	1	0
NA	NA	NA	NA	NA	NA	1	1	1	1	2	1	1	1	1	1	2	2
NA	NA	NA	NA	NA	NA	1	1	1.4	1	1	1	2	2	1	1	1	1
NA	NA	NA	NA	NA	NA	1	1	1.4	1	2	3	2	1	1	1	1	1
NA	NA	NA	NA	NA	NA	1	1	0.7	1	1	1	0	1	2	1	1	1
NA	NA	NA	NA	NA	NA	1	1	1.2	1	1	0	1	1	1	1	1	1
						4	0	0	0	##	5	0	0	0	0	0	0
NA	NA	NA	NA	NA	NA	1	1	3.7	1	1	0	0	2	1	7	0	0
NA	Pteridines	Pteridines	NA	NA	NA	0	0	0.1	3	0	0	0	1	1	1	0	1
NA	NA	NA	NA	NA	NA	1	1	0.9	1	1	1	1	5	##	1	1	7
5'-deoxy-	5'-deoxyri	Nucleic ac	Amino aci	Cysteine a	map00270	0	1	4	2	0	1	0	43	1	17	0	0
NA	NA	NA	NA	NA	NA	1	1	3	1	1	20	3	1	3	1	1	1
NA	NA	NA	NA	NA	NA	2	0	0.4	2	0	1	0	3	1	2	0	2
NA	NA	NA	NA	NA	NA	0	1	0.4	0	13	0	1	9	10	0	0	2
NA	NA	NA	Xenobiotic	Atrazine d	map0110	0	2	0.5	1	1	1	2	1	0	0	0	9
NA	NA	NA	NA	NA	NA	1	2	1	1	2	1	2	2	1	1	1	8
NA	NA	NA	NA	NA	NA	1	1	2.3	1	2	1	2	1	1	1	2	1
NA	NA	NA	Metabolisr	Biosynthes	map0110	1	1	1.1	1	1	2	1	1	1	1	1	8
N-arylami	Organonit	Organic ni	Biosynthes	Caffeine m	map00232	1	1	1.3	1	0	1	1	1	3	2	1	1
NA	NA	NA	NA	NA	NA	1	2	1	1	3	3	2	1	1	1	0	1
[truncated: 686,668 more chars]
